# Insight into medicinal chemistry behind traditional Chinese medicines: polybenzyls in *Gastrodia elata*

**DOI:** 10.1007/s13659-026-00625-z

**Published:** 2026-09-11

**Authors:** Yanan Wang, Wenjing Wang, Min Zhang, Chenggen Zhu, Chengbo Xu, Xia Li, Xiaoqiang Lei, Qinglan Guo, Jiangong Shi

**Affiliations:** State Key Laboratory of Bioactive Substance and Function of Natural Medicines, Institute of Materia Medica, Chinese Academy of Medical Sciences and Peking Union Medical College, Beijing, 100050 China

**Keywords:** Orchidaceae, *Gastrodia elata*, polybenzyls, Gastrotetrabenzins A − F, Gastropentabenzins A − E, Gastrohexabenzins A and B, Medicinal chemistry behind TCM

## Abstract

Thirteen undescribed minor polybenzyl constituents included derivatives of six tetrabenzyls (gastrotetrabenzins A−F, **1**−**6**), five pentabenzyls (gastropentabenzins A−E, **7**−**11**), and two hexabenzyls (gastrohexabenzins A and B, **12** and **13**), along with a known pentabenzyl (**14**), were isolated from an aqueous extract of *Gastrodia elata* Blume rhizomes (tian ma). Meanwhile, **4**−**10** and **12**−**14** as well as fourteen undescribed polybenzyls (**15**−**28**) and one known analogue (**29**) were isolated and structurally characterized from a refluxed water solution of *p*-hydroxybenzyl alcohol. Subsequent UPLC-HRESIMS analysis of the extracts of commercial tian ma and fresh *G. elata* rhizomes proved that the polybenzyls from the aqueous extract are producible by the traditional application methods of processing and decocting. Bioassays showed that the polybenzyls were much more active than the main constituents* p*-hydroxybenzyl alcohol and gastrodin of tian ma. These results provide experimental evidences to explain why tian ma is processed and decocted in traditional Chinese medicine (TCM), unravelling structural modification and active optimization behind the TCM application methods.

**Graphical Abstract:**

**Figure:**
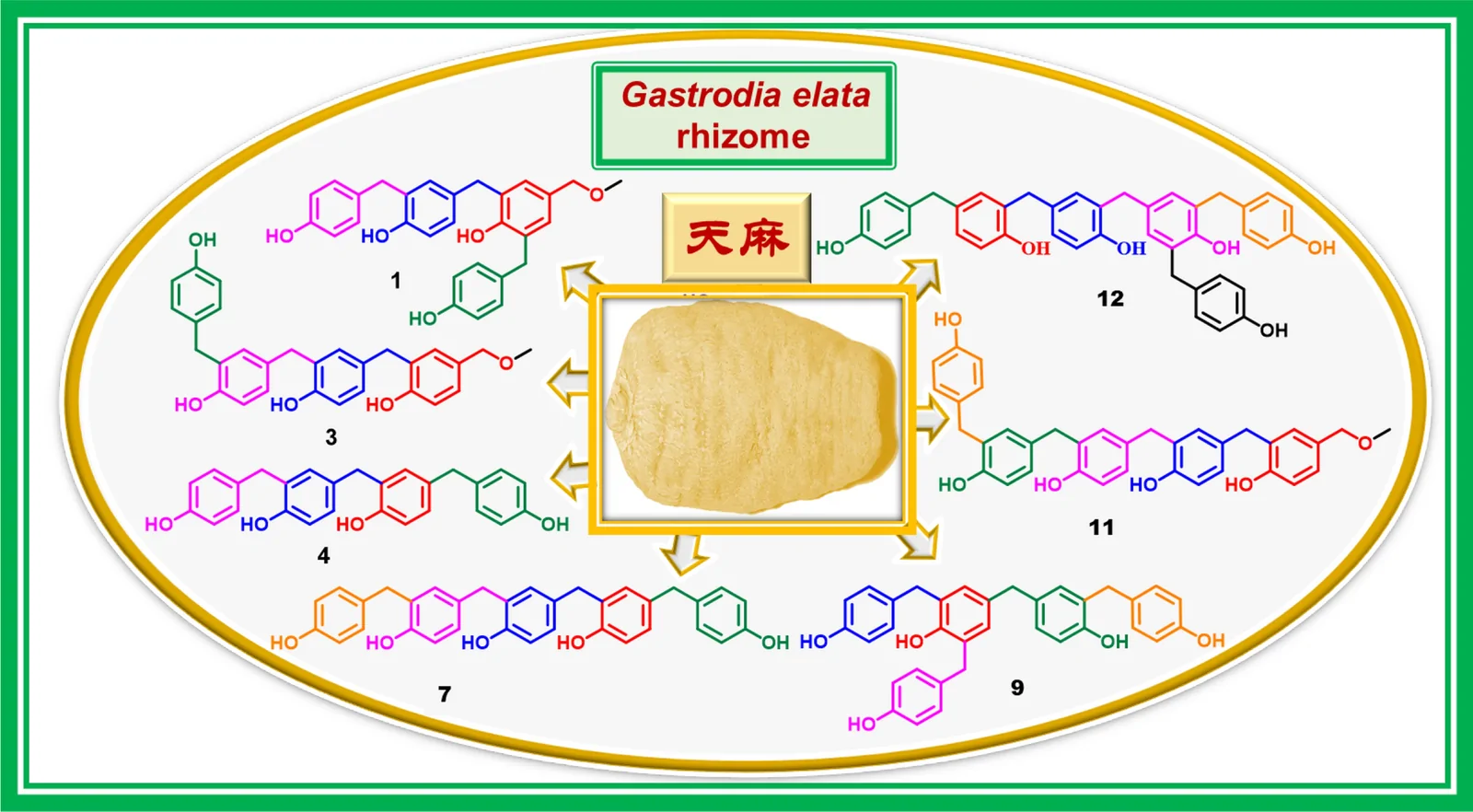

**Supplementary Information:**

The online version contains supplementary material available at https://doi.org/10.1007/s13659-026-00625-z.

## Introduction

**Fig. 1:**
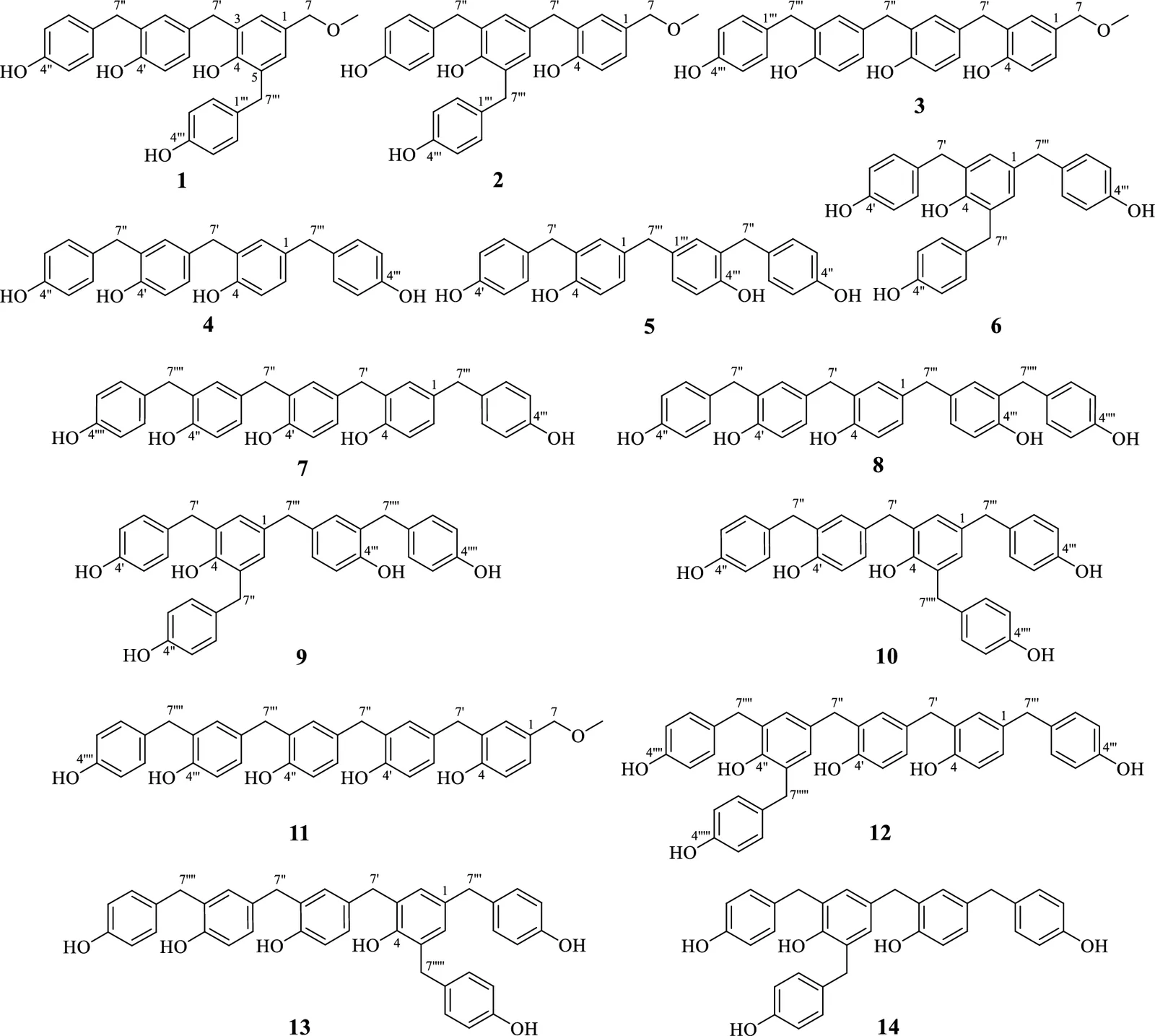
Structures of compounds **1**−**14**

**Fig. 2:**
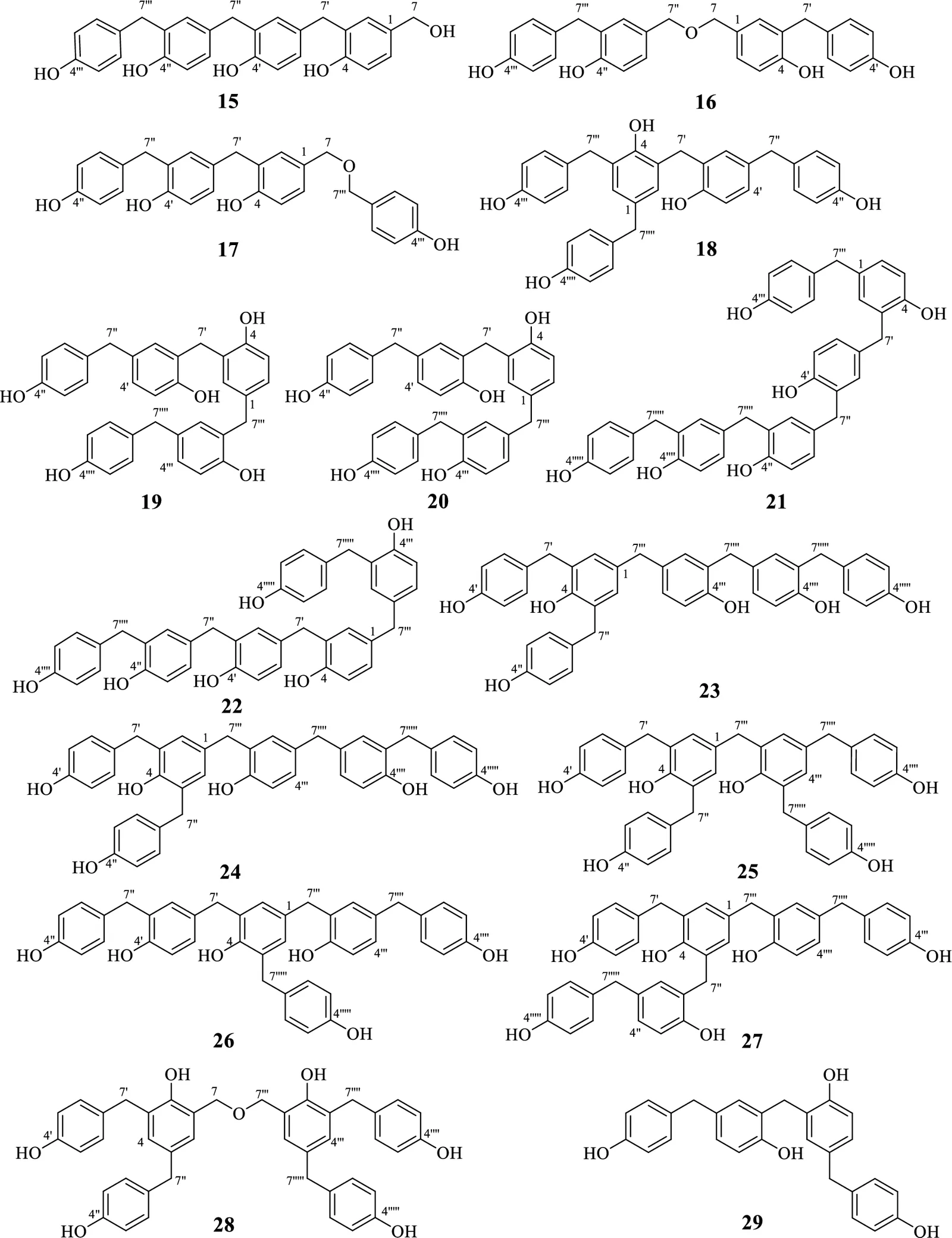
Structures of compounds **15**−**29**

Tian ma (the steamed and dried *Gastrodia elata* Blum rhizomes) is a precious plantable herbal medicine. In traditional Chinese medicine (TCM), it is mainly used for the treatment of various neuralgic and nervous disorders, and it also has health benefits enhancing strength and virility as well as improving memory and blood circulation [1]. From extracts of this remedy, more than 270 constituents with diverse chemical structures and biological activities have been isolated and identified by systemic studies [2]. Most the structures are associated to the main component *p*-hydroxybenzyl alcohol, such as gastrodin which is considered as the active constituent of tian ma [2–6]. Meanwhile, some studies indicated that an aqueous extract of tian ma after removal of gastrodin retained anti-hypoxia, sedative, hypnotic and anti-inflammatory effects [7, 8]. In addition, procession and decoction to detoxify and/or to enhance effects of drugs are claimed in TCM, but absent experimental evidence in most cases. We believe that some chemical reactions must take place to destroy toxic chemical structures and to produce more effective chemicals [4, 9–20] during processing and decocting. This is the logic of medicinal chemistry behind ancient TCM, but normally ignored in modern investigations. Therefore, as part of our project to unravel diverse chemical structures and biological activities of several popular herbal medicines [21–30], a water extract of tian ma was systematically investigated. In previous papers [31–42], 37 new and 50 known chemical constituents of the aqueous extract, along with their bioassays and pharmacological activities have been reported. Especially some *p*‑hydroxybenzyl alcohol‑derived dimers and trimers as well as *p*-hydroxybenzyl-modified gastrodins from the extract were proved to be generated by *p*-hydroxybenzyl alcohol and the co-occurring gastrodin during decocting the plant materials [36, 37]. An accumulating investigation of the water extract led to isolation and structural characterization of 13 undescribed minor polybenzyl derivatives, including six tetrameric gastrotetrabenzins A − F (**1**−**6**), five pentameric gastropentabenzins A − E (**7**−**11**), and two hexameric gastrohexabenzins A and B (**12** and **13**) as well as one known pentameric derivative (**14**) (Fig. 1). Except for methyl ethers (**1**−**3** and **11**), the polybenzyls (**4**−**10** and **12**−**14**), together with 15 analogues (**15**−**29**, Fig. 2), were also isolated and characterized from the refluxed water solution of *p*-hydroxybenzyl alcohol. Importantly, in various in vitro bioassays the polybenzyls were more active than the known active constituents of tian ma, *p*-hydroxybenzyl alcohol and gastrodin. Production of the more active polybenzyls during decocting was unambiguously evidenced by parallel UPLC/HRESIMS analysis of the extracts of commercially available processed tian ma and fresh *G. elata* rhizomes. Herein described are details.

## Results and discussion

Following the extraction and preliminary fractionation of the aqueous extract [31, 37], subsequent separation of fractions C2-1-1−C2-1-3 by column chromatography (CC) over normal phase silica gel, Sephadex LH-20, and/or HW-40C gel, in combination with purification by preparative silica gel TLC, reversed phase high-performance liquid chromatography (RP-HPLC), and chiral HPLC, obtained compounds **1**−**14** as white amorphous powders or brown gums (see Experimental Section, Supporting Information).

Compound **1** showed IR absorptions for hydroxyl (3329 cm^−1^) and aromatic ring (1612, 1598, and 1512 cm^−1^) functionalities. Its molecular formula C_29_H_28_O_5_ was determined by HRESIMS at *m/z* 479.1846 [M + Na]^+^ (calcd. for C_29_H_28_O_5_Na, 479.1829). The NMR spectrum of **1** in acetone-*d*_6_ showed resonances attributable to two *para*-substituted phenyls [*δ*_H_ 7.05 (2H, d, *J* = 8.5 Hz, H-2‴/6‴), 7.04 (2H, d, *J* = 8.5 Hz, H-2ʺ/6ʺ), 6.73 (2H, d, *J* = 8.5 Hz, H-3‴/5‴), and 6.70 (2H, d, *J* = 8.5 Hz, H-3ʺ/5ʺ)]; a *meta*-*para*-substituted phenyl [*δ*_H_ 6.97 (1H, d, *J* = 2.0 Hz, H-2ʹ), 6.86 (1H, dd, *J* = 8.0, 2.0 Hz, H-6ʹ), and 6.74 (1H, d, *J* = 8.0 Hz, H-5ʹ)]; a *meta*-*meta*-*para*-trisubstituted phenyl [*δ*_H_ 6.83 (1H, d, *J* = 2.0 Hz, H-2) and 6.87 (1H, d, *J* = 2.0 Hz, H-6)]; four methylenes [*δ*_H_ 4.20 (2H, s, H-7), 3.91 (2H, s, H-7‴), 3.87 (2H, s, H-7ʹ), and 3.82 (2H, s, H-7ʺ)]; four phenolic hydroxyl groups [*δ*_H_ 8.07 (1H, s, 4‴-O*H*), 8.04 (1H, s, 4ʹ-O*H*), 8.01 (1H, s, 4ʺ-O*H*), and 7.12 (1H, s, 4-O*H*), and a methoxy group [*δ*_H_ 3.20 (3H, s, OC*H*_*3*_)]. The ^13^C NMR and DEPT spectra of **1** exhibited carbon resonances assignable to the above units. As compared with those of the previously reported compounds from the same plant [2–5], the spectroscopic data indicated that **1** was an unprecedented methyl tetrameric benzyl ether. To unambiguously establish connections among the structural units, the 2D NMR spectra of **1** were acquired.

**Fig. 3:**
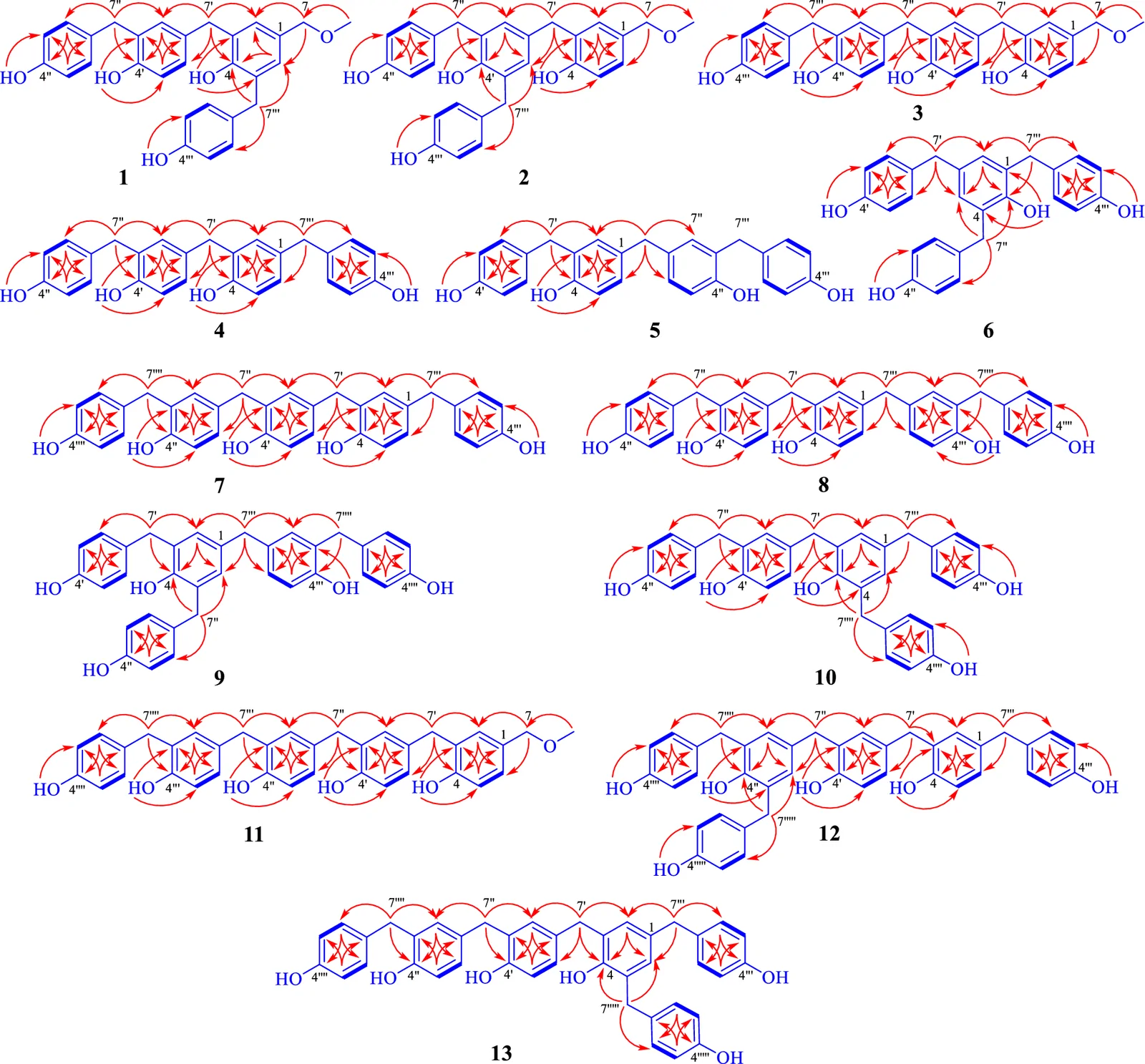
The ^1^H-^1^H COSY (thick lines) and key HMBC (arrows) correlations of **1**−**13**

Analysis of the ^1^H-^1^H COSY and HSQC spectra assigned unequivocally the proton and proton-bearing carbon resonances in the NMR spectra of **1** (Table S1, Supporting Information). Besides two- and three-bond heteronuclear coupling correlations confirming the above units (Fig. 3), in the HMBC spectrum of **1**, the correlations of H-2ʹ/C-7ʺ; H_2_-7″/C-2′, C-3′, and C-4′; 4ʹ-O*H*/C-3′, C-4′, and C-5′; and 4ʺ-O*H*/C-3ʺ (C-5ʺ), together with chemical shifts of these proton and carbon resonances, indicated the presence of a 4ʹ-hydroxy-3ʹ-(4ʺ-hydroxybenzyl)benzyl moiety. In addition, the correlations of H_2_-7′/C-2, C-3, and C-4; H_2_-7‴/C-4, C-5, and C-6; 4-O*H*/C-3, C-4, and C-5; and 4‴-O*H*/C-3‴ (C-5‴) and C-4‴, along with their chemical shifts, suggested that 4ʹ-hydroxy-3ʹ-(4ʺ-hydroxybenzyl)benzyl and 4‴-hydroxy benzyl substituted at C-3 and C-5 of the remaining *p*-hydroxybenzyl, respectively. Furthermore, the correlation of H_2_-7/O*C*H_3_ and OC*H*_3_/C-7 located the methoxy group at C-7. Accordingly, the structure of compound **1** was determined and named gastrotetrabenzin A.

The spectroscopic data indicated that compound **2** was an isomer of **1**. The NMR spectroscopic data (Table S1) revealed that **2** differed from **1** by the presence of two equivalent *p*-hydroxybenzyls. This difference suggested that *p*-hydroxybenzyl at C-5 in **1** was moved to C-5ʹ in **2**. The suggestion was verified by 2D NMR experimental data of **2** (Fig. 3), especially by the HMBC correlations from H_2_-7 to O*C*H_3_; from H_2_-7′ to C-2, C-3, and C-4, and from H_2_-7ʺ/7‴ to C-3′/5′ and C-4′, in combination with the chemical shifts, integrations, and intensities of these proton and carbon resonances. Thus, the structure of compound **2** was determined and named gastrotetrabenzin B.

The spectroscopic data showed that compound **3** was another isomer of **1** and **2**. The NMR spectroscopic data (Table S1) demonstrated the presence of only one terminal *p*-hydroxybenzyl in **3**, instead of two in **1** and **2**. The difference indicated that *p*-hydroxybenzyl at C-5 in **1** was moved to C-5ʺ in **3**, which was by the HMBC correlations of H_2_-7‴/C-2ʺ, C-3ʺ, and C-4ʺ (Fig. 3). Hence, the structure of compound **3** was determined and named gastrotetrabenzin C. The same structure was proposed by UHPLC/Q-TOF–MS/MS analysis of a 50% ethanol extract of *G. elata* [43], but absent other spectroscopic evidence.

Compound **4** has the molecular formula C_27_H_24_O_4_ as indicated by the HRESIMS at *m/z* 411.1604 [M − H]^−^ (calcd. for C_27_H_23_O_4_, 411.1602) and NMR spectroscopic data (Table S1). The NMR spectra of **4** in DMSO-*d*_6_ exhibited the characteristic resonances attributable to two *p*-hydroxyphenyls, two *meta*-substituted *p*-hydroxyphenyls, and three methylenes. The connections among these units were determined by analysis of 2D NMR experimental data of **4** (Fig. 3). In particular, the HMBC correlations of 4-O*H*/C-3, C-4 and C-5; 4′-O*H*/C-3′, C-4′ and C-5′; 4ʺ-O*H*/C-4ʺ and C-3ʺ (C-5ʺ); H_2_-7′/C-1′, C-2′, C-2, C-3, C-4, and C-6′; H_2_-7ʺ/C-1ʺ, C-2ʹ, C-3ʹ, C-4ʹ, and C-2ʺ (C-6ʺ), along with their chemical shifts, indicated the presence of a 3-[4′-hydroxy-3′-(4ʺ-hydroxybenzyl)benzyl]-4-hydroxyphenyl moiety in **4**. Meanwhile, the HMBC correlations from H_2_-7‴ to C-1, C-1‴, C-2, C-6, and C-2‴/6‴ demonstrated substitution of 4‴-hydroxybenzyl at C-1 of the above moiety. Hence, the structure of compound **4** was determined and named gastrotetrabenzin D.

Compound **5** is an isomer of **4** as exhibited by HRESIMS at *m/z* 435.1569 [M + Na]^+^ (calcd. for C_27_H_24_O_4_Na, 435.1567). However, the NMR spectra of **5** in DMSO-*d*_6_ displayed almost the half resonances expected from the molecular formula. Explanation of the NMR spectroscopic data indicated that there were two equivalent *p*-hydroxybenzyls, two equivalent *meta*-substituted *p*-hydroxyphenyls, and an isolated methylene. This suggested that 4-hydroxybenzyl at C-3′ in **4** was migrated to C-3‴ in **5** to form a symmetric structure. The deduction was confirmed by 2D NMR experimental data analysis (Fig. 3). In the HMBC spectrum of **5**, the correlations from H_2_-7‴ to C-1/C-1‴, C-2/C-2‴, and C-6/C-6‴; from H-2/2‴ and H-6/6‴ to C-7‴, and from 4/4‴-O*H* to C-3/C-3‴, C-4/C-4‴, and C-5/C-5‴, together with their chemical shifts and proton integrations, verified the isolated methylene-bridged connection between the two *meta*-substituted *p*-hydroxyphenyls to form a symmetric central moiety. Meanwhile, the correlations from H_2_-7′/7″ to C-1′/1″, C-2′/2″/6′/6″, C-2/2‴, C-3/3‴, and C-4/4‴ and from 4′/4″-O*H* to C-3′/3″, C-4′/4″, and C-5′/5″, along with their chemical shifts and proton integrations, evidenced a symmetrical substitution of the two *p*-hydroxybenzyls at C-3 and C-3‴ of the central moiety. Therefore, the structure of compound **5** was determined and named gastrotetrabenzin E.

Compound **6** is another isomer of **4** according to ( −)-HRESIMS at *m/z* 411.1582 [M − H]^−^ (calcd. for C_27_H_23_O_4_, 411.1602). The NMR spectra of **6** displayed resonances attributable to one *meta*-*meta*-disubstituted *p*-hydroxybenzyl and three *p*-hydroxybenzyls (two equivalent). This suggested that the *p*-hydroxybenzyl at C-3ʹ in **4** was moved to C-5 in **6**. The suggestion was proved by the HMBC correlations from O*H*-4 to C-3, C-4, and C-5; from H_2_-7‴ to C-1, C-1‴, C-2, C-2‴/6‴, and C-6; from H_2_-7′/7″ to C-1′/1″, C-2′/2″/6′/6″, C-3/5, and C-4 (Fig. 3), together with their chemical shifts. Thus, the structure of compound **6** was determined and named gastrotetrabenzin F.

It should be noted that compounds **3**−**5** were preliminarily presented in the 10th national conference on medicinal plants and phytomedicines [44] and patented by the authors [45, 46], meanwhile, a synthetic sample of compound **4** (coded 20C) was pharmacologically investigated by collaborators [41, 42]. In addition, **3** and **5** were also proposed by UPLC/Q-TOF MS analysis of ethanolic extracts of *G. elata* [43, 47], but absent other spectroscopic evidence, while compound **6** was synthesized as mixtures without purification and characterization [48]. Nevertheless, this is the first report for complete structural characterization of **3**−**6**, and trivially named for consistency and convenience.

The molecular formula of compound **7** was determined as C_34_H_30_O_5_ by HRESIMS at 541.1979 [M + Na]^+^ (calcd. for C_34_H_30_O_5_Na, 541.1985) combined with the NMR spectroscopic data (Table S2). Comparing with those of **4**, the NMR spectra of **7** in acetone-*d*_6_ showed the specific signals due to two* p*-hydroxyphenyls, three *meta*-substituted *p*-hydroxyphenyls, and four methylenes. These spectroscopic data indicated that **7** was a derivative of **4** with one more *p*-hydroxybenzyl attaching at C-3″. The deduction was proved by 2D NMR (Fig. 3) and NOE difference experimental data (Fig. S97). Especially, in the NOE difference spectrum of **7**, irradiation of H_2_-7‴ enhanced H-2, H-2‴/6‴, and H-6 without influence on any hydroxyl proton, confirming the methylene-bridged connection between C-1 of the headed *p*-hydroxyphenyl and C-1‴ of one *meta*-substituted *p*-hydroxyphenyl. In addition, the enhancements of H-2⁗/6⁗ and H-2″ as well as of 4″-O*H* by irradiation of H_2_-7⁗ evidenced the methylene-bridged connection between C-1⁗ of the tailed *p*-hydroxyphenyl and C-1″ of another *meta*-substituted *p*-hydroxyphenyl. Furthermore, the enhancements of H-2, H-2′/2″, and H-6′/6″ as well as of 4-O*H* and 4′-O*H* upon irradiation of H_2_-7′/7″ unambiguously verified the methylene-bridged connections between C-1′ and C-3 as well as between C-1″ and C-3′ in **7**. Therefore, the structure of compound **7** was determined and named gastropentabenzin A (Fig. 1).

The spectroscopic data indicated that compound **8** was an isomer of **7**. Comparison of the NMR spectroscopic data between the two compounds (Table S2) revealed that the structural units in **8** was assembled in a more symmetric pattern than that in **7**. The structure of **8** was finalized by analysis of 2D NMR (Fig. 3) and NOE difference experimental data (Fig. S110). In particular, the HMBC spectrum showed the correlations of H-2′/C-4′ and H-2‴/C-4‴ as well as from both H-2′ and H-2‴ to the overlapped C-7″/7⁗. These correlations, together with their chemical shifts, revealed that there were two *m*-(*p*-hydroxybenzyl)-*p*-hydroxybenzyls in **8**. The deduction was further confirmed by the enhancements of H-2′, H-2‴, and H-2ʺ/2⁗/6ʺ/6⁗ as well as of 4′-O*H* and 4‴-O*H* upon irradiation of H_2_-7″/7⁗ in the NOE difference spectrum of **8**. In addition, irradiation of H_2_-7′ gave the enhancements of H-2, H-2′, H-6′, and 4-O*H*; while H-2, H-2‴, H-6, and H-6‴ were enhanced upon irradiation of H_2_-7‴. These enhancements in combination with the coupling constants of the proton resonances located the two *m*-(*p*-hydroxybenzyl)-*p*-hydroxybenzyls at C-1 and C-3 of the remaining *p*-hydroxyphenyl unit in **8**. Therefore, the structure of compound **8** was determined and named gastropentabenzin B.

Compound **9** is another isomer of **7** and **8** as shown by spectroscopic data. The NMR spectroscopic data indicated that **9** differed from **8** by consisting of three *p*-hydroxybenzyls (two equivalent); one *meta*-substituted* p*-hydroxybenzyl; and one symmetrically *meta*-*meta*-disubstituted* p*-hydroxyphenyl. These differences indicated that *p*-hydroxybenzyl at C-3′ in **8** was moved to C-5 in **9**, which was verified by 2D NMR experimental data of **9**. Especially in the HMBC spectrum of **9**, the correlations from H_2_-7′/7″ to C-2/6, C-3/5, C-4, and C-2′/2″/6′/6″; from H-2/6 to C-7′/7″ and C-7‴; from H_2_-7⁗ to C-2‴, C-2⁗/6⁗, and C-4‴; and from H_2_-7‴ to C-2/6, C-2‴, and C-6‴ (Fig. 3), in combination with their shifts, integrations, and intensities, confirmed the methylene-bridged connections of *p*-hydroxyphenyls. This was further proved by the enhancements of H-2/6 and H-2′/2″/6′/6″ upon irradiation of H_2_-7′/7″; of H-2/6, H-2‴, and H-6‴ upon irradiation of H_2_-7‴; and of H-2‴ and H-2⁗/6⁗ upon irradiation of H_2_-7⁗ in the NOE difference spectrum of **9** (Fig. S123). Accordingly, the structure of compound **9** was determined and named gastropentabenzin C.

The spectroscopic data indicated that compound **10** is one more isomer of **7** − **9**. Comparing with those of **7**, the NMR spectroscopic data of **10** in acetone-*d*_6_ suggested that *p*-hydroxybenzyl at C-3″ in **7** was migrated to C-5 in **10**. The suggestion was also verified by 2D NMR (Fig. 3) and NOE difference experiments of **10**. Particularly in the NOE difference spectrum (Fig. S136) of **10**, irradiation of H_2_-7′ or H_2_-7‴ enhanced H-2, while H-6 was enhanced upon irradiation of H_2_-7‴ or H_2_-7⁗. Meanwhile, irradiation of H_2_-7‴ and H_2_-7⁗ enhanced H-2‴/6‴ and H-2⁗/6⁗, respectively, and H-2′ and H-6′ were enhanced upon irradiation of H_2_-7′. These enhancements, together with splitting patterns and coupling constants of H-2, H-2′, H-2‴/6‴, H-2⁗/6⁗, H-6, and H-6′, confirmed that two *p*-hydroxybenzyls were located at C-1 and C-5 of the central *p*-hydroxyphenyl in **10**, respectively, while one *meta*-substituted* p*-hydroxybenzyl was attached at C-2. Furthermore, the NOE enhancement of H-2″/6″ and H-2′ upon irradiation of H_2_-7″ proved the remaining *p*-hydroxybenzyl at C-3′. Therefore, the structure of compound **10** was determined and named gastropentabenzin D.

The molecular formula of compound **11** was determined as C_36_H_34_O_6_ by ( +)-HRESIMS at *m/z* 585.2241 [M + Na]^+^ (calcd. for C_36_H_34_O_6_Na, 585.2248). The NMR spectra of **11** in acetone-*d*_6_ indicated the presence of four *m*-substituted* p*-hydroxybenzyls, one *p*-hydroxybenzyl, and one methoxy group. The chemical shifts of the methoxy group (*δ*_H_ 3.20 and *δ*_C_ 57.5) and one of the five benzylic methylene (*δ*_H_ 4.22 and *δ*_C_ 74.9), along with the presence of five distinctive resonances for the phenolic hydroxyl protons, revealed that **11** was a *p*-hydroxybenzyl-derived pentameric analogue of **3**. Comparison of the NMR spectroscopic data between **11** and **3**, suggested that **11** had one more *p*-hydroxybenzyl at C-3‴ than **3**. The elucidation was evidenced by 2D NMR experimental data of **11** (Fig. 3). Especially the HMBC correlations from H_2_-7 to C-2, C-6, and O*C*H_3_; from H-2⁗/6⁗ to C-4⁗ and C-7⁗; from 4-O*H* to C-3, C-4, and C-5; and from 4′-O*H* to C-3′, C-4′, and C-5′, along with their chemical shifts, confirmed the presence of a methyl *meta*-substituted* p*-hydroxybenzyl ether head and a *p*-hydroxybenzyl tail in **11**. Meanwhile, the HMBC correlations from 4″-O*H* to C-3″, C-4″, and C-5″; from 4‴-O*H* to C-3‴, C-4‴, and C-5‴; and from 4⁗-O*H* to C-3⁗/5⁗ and C-4⁗, in combination with their shifts, proved the presence of three *meta*-substituted* p*-hydroxyphenyls. In addition, the HMBC correlations of H_2_-7′/C-4, H_2_-7″/C-4′, H_2_-7‴/C-4″, and H_2_-7⁗/C-4‴, together with their shifts, confirmed the methylene-bridged sequential connections of the remaining *meta*-substituted* p*-hydroxyphenyls in **11**. Hence, the structure of compound **11** was determined and named gastropentabenzin E.

Compound **12** has the molecular formula C_41_H_36_O_6_ as determined by ( +)-HRESIMS at *m/z* 647.2384 [M + Na]^+^ (calcd. for C_41_H_36_O_6_Na, 647.2404). The NMR spectra of **12** in acetone-*d*_6_ displayed the resonances assignable to three *p*-hydroxybenzyls (two equivalent), two *meta*-substituted* p*-hydroxybenzyls, and one *meta*-*meta*-disubstituted *p*-hydroxybenzyl. This indicated that **12** was a *p*-hydroxybenzyl-derived hexamer. Comparison of the NMR spectroscopic data between **12** and **7** suggested that **12** was the derivative of **7** possessing an additional* p*-hydroxybenzyl at C-5ʺ. This was proved by the correlations from H_2_-7ʹ to C-2, C-3, and C-4; from H_2_-7ʺ to C-2ʹ, C-3ʹ, and C-4ʹ; from H_2_-7‴ to C-1, C-2, and C-6; and from H_2_-7⁗/7⁗ʹ to C-2ʺ/6ʺ, C-3ʺ/5ʺ, and C-4ʺ in the HMBC spectrum of **12** (Fig. 3), along with the chemical shifts of these proton and carbon resonances. Therefore, the structure of compound **12** was determined and named gastrohexabenzin A.

The spectroscopic data of compound **13** indicated that it was an isomer of **12** with three inequivalent *p*-hydroxybenzyls. Comparison of the NMR spectroscopic data between the two compounds suggested that *p*-hydroxybenzyl at C-5ʺ in **12** was moved to C-5 in **13**. The suggestion was verified by 2D NMR experimental data of **12** (Fig. 3), especially by the HMBC correlations from H_2_-7ʹ to C-2, C-3, and C-4; H_2_-7ʺ to C-2ʹ, C-3ʹ, and C-4ʹ; H_2_-7‴ to C-1, C-2, and C-6; H_2_-7⁗ to C-2ʺ, C-3ʺ, and C-4ʺ; and H_2_-7⁗ʹ to C-4, C-5, and C-6. Therefore, the structure of compound **13** was determined and named gastrohexabenzin B.

Compound **14** was identified as gastropolybenzylol G by comparison of spectroscopic data with those reported in literature [49] as well as by analysis of 2D NMR experimental data.

**Fig. 4:**
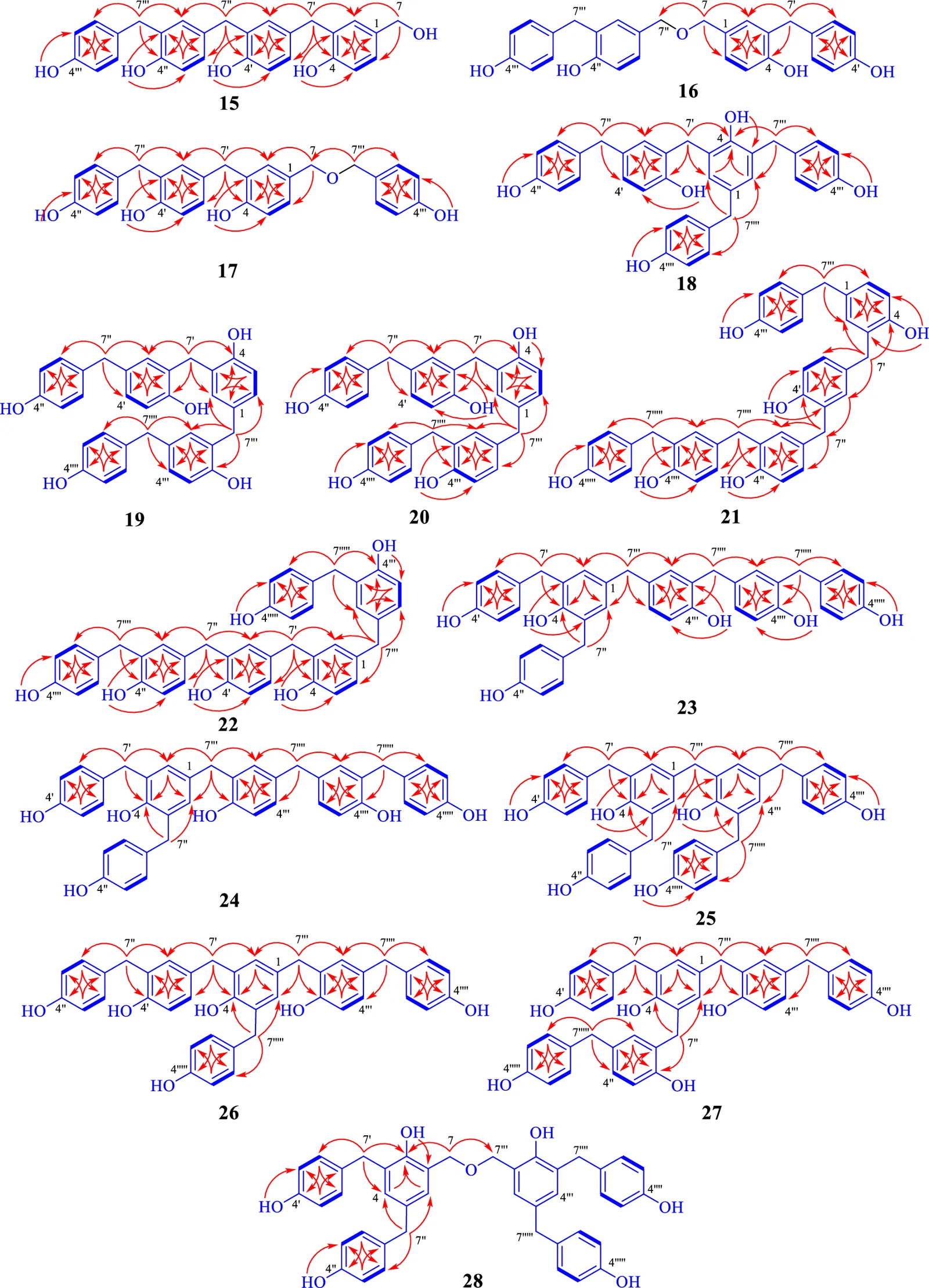
The ^1^H-^1^H COSY (thick lines) and key HMBC (arrows) correlations of **15**−**28**

As we speculated and partially proved before that most the constituents containing *p*-hydroxybenzyl unit(s) from the extracts of tian ma could be generable from chemical reactions with the co-occurring precursor *p*-hydroxybenzyl alcohol during processing and decocting application procedures [36, 37], the aforementioned 14 compounds from the water extract of tian ma should be producible from *p*-hydroxybenzyl alcohol. Therefore, products of a refluxed water solution of *p*-hydroxybenzyl alcohol were further isolated. As expected, except for the methyl ethers (**1**−**3** and **11**), compounds **4**−**10** and **12**−**14**, together with 14 undescribed (**15**−**28**) and one known (**29**) [50] polybenzyls, were obtained and structurally characterized from the refluxed aqueous solution of *p*-hydroxybenzyl alcohol. The structures of **15**−**29** were unambiguously determined by spectroscopic analysis including the 2D NMR experimental data (Fig. 4 and Supporting Information).

According to systematical nomenclature, compounds **15**−**29** are respectively named 4-hydroxy-3-{4-hydroxy- 3-[4-hydroxy-3-(4-hydroxylbenzl)benzyl]benzyl}benzyl alcohol (**15**), di-[4-hydroxy-3-(4-hydroxybenzyl)benzyl]ether (**16**), 4-hydroxy-3-[4-hydroxy-3-(4-hydroxybenzyl)benzyl]benzyl4-hydroxybenzyl ether (**17**), 1,5-di-(4-hydroxybenzyl)- 3-[2-hydroxy-5-(4-hydroxybenzyl)benzyl]phenol (**18**), 1,3-di-[2-hydroxy-5-(4-hydroxybenzyl)benzyl]-4-hydroxyphene (**19**), 1-[4-hydroxy-5-(4-hydroxybenzyl)benzyl]-3-[2-hydroxy-5-(4-hydroxybenzyl)benzyl]phenol (**20**), 1-(4-hydroxybenzyl)-4-{4-hydroxy-3-{4-hydroxy-3-[4-hydroxy-3-(4-hydroxybenzyl)benzyl]benzyl}benzylphenol (**21**), 1-[4-hydroxy-3-(4-hydroxybenzyl)benzyl]-3-{4-hydroxy-3-[4-hydroxy-3-(4-hydroxybenzyl)] benzyl}benzylphenol (**22**), 1-{4-hydroxy-3-[4-hydroxy-3-(4-hydroxybenzyl)]benzyl}benzyl-3,5-di-(4-hydroxybenzyl)]benzyl}phenol (**23**), 1-{2-hydroxy-3-[4-hydroxy-3-(4-hydroxybenzyl)benzyl]benzyl}-3,5-di-(4-hydroxybenzyl)phenol (**24**), 1-[2-hydroxy- 3,5-di-(4-hydroxybenzl)benzyl]-3,5-di-(4-hydroxybenzl)phenol (**25**), 1-[2-hydroxy-5-(4-hydroxybenzyl)benzyl]-3- [4-hydroxy-3-(4-hydroxybenzyl)benzyl]-5-(4-hydroxybenzyl)phenol (**26**), 1,5-di-[2-hydroxy-5-(4-hydroxybenzyl) benzyl]-3-(4-hydroxybenzyl)phenol (**27**), di-[2-hydroxy-3,5-di-(4-hydroxybenzyl)]benzyl ether (**28**), and 4,4ʹ-bis(4-hydroxybenzyl)-2,2ʹ-methylene diphenol (**29**). Although compounds **15**−**29** were not isolated and identified from the water extract of tian ma, their occurrences in trace amounts are highly expected because they come from *p*-hydroxybenzyl alcohol and because *p*-hydroxybenzyl alcohol exists in tian ma.

Following our protocols [37], samples of the treated* p*-hydroxybenzyl alcohol as well as of the commercial processed tian ma and the fresh *G. elata* rhizomes were analyzed by UPLC/HRESIMS (Figs. S323−S375, Supporting Information). The methyl ethers **1**, **2**, and **11** were detectable in a refluxed methanol solution of *p*-hydroxybenzyl alcohol by UPLC/HRESIMS analysis. However, only compounds **4** and **6** were detectable in the H_2_O, MeOH, and EtOH extracts of the processed commercial tian ma sample, while **5** was detectable in the H_2_O and MeOH extracts. More significantly, none of the polybenzyls was detectable in the H_2_O, MeOH, or EtOH extracts of fresh *G. elata* rhizomes. The detectable compounds **4**−**6** in the extracts of tian ma samples were exactly the major products in the refluxed water solution of *p*-hydroxybenzyl alcohol. These results strongly evidenced that the polybenzyls **1**−**14** in the water extract of tian ma were mainly generated during the processing and/or experimental procedures, though their productions by the plant itself could not be fully excluded since potential lower contents in the fresh *G. elata* rhizomes and method limitation may lead to them being undetectable.

In the in vitro bioassays, at a concentration of 10 *μ*M, as compared with the blank control, compounds **1**−**3** and **6** attenuated rotenone-induced PC12 cell damage by increasing the cell viability from 67.11 ± 5.61% to 73.74 ± 5.52%, 72.01 ± 2.78%, and 71.00 ± 4.27%, respectively (Table S8, Supporting Information). Compound **4** showed protective effect against *α*-syn- and rotenone-induced PC12 cell damages (Table S9) as well as anti-HIV-1 activity (Table S10). Compounds **4**−**6** were synthesized [46] for pharmacological evaluation, of which **4** (20C) has evident neuroprotective effects [41, 42, 46]. At the same concentration, compounds **1**−**3**, **6**−**8**, **10**, **14**, and the positive control dexamethasone inhibited LPS-induced NO production in mouse peritoneal macrophages with inhibition rates of 46.9−95.3% (Table S11), while **4**−**10**, **12**−**29**, and the positive control curcumin inhibited Fe^2+^-cystine-induced rat liver microsomal lipid peroxidation with inhibition rates of 67.82−100.49% (Table S13). In addition, **5**, **7**−**10**, **12**−**14**, and **19**−**29** inhibited *α*-glucosidase activity with inhibition rates of > 78% and IC_50_ of 0.45−4.60 μM (positive control acarbose, 200 *µ*M, with 75.2% inhibition; Table S15), while **12**−**14** and **23**−**27** inhibited PTP1B activity with IC_50_ values of 2.25−4.92 μM (positive control CC06240, 10 µM, with inhibition of 100.5%; Table S16). Furthermore, compounds **5**, **8**, **13**, and **16** showed activity against influenza virus A/Hanfang/359/95 (H3N2) with IC_50_ values of 3.70−11.11 μM and selective index (SI) of 1.7−4.3 (positive controls oseltamivir and ribavirin with IC_50_’s of 2.01 and 2.47 *µ*g/mL as well as SI’s of > 99.5 and > 81.0, respectively; Table S17), while **4**, **5**, **7**, **16**, and **17** inhibited Coxsackie virus B3 (CVB3) replication, with IC_50_ values of 6.42−19.25 and SI of 1.7−4.0, respectively (the positive controls, oseltamivir and ribavirin with IC_50_ value of 0.0021 and 517.38 µg/mL as well as SI’s of 7338.1 and 3.9, respectively; Table S18). However, in the parallel assays, the main components of tian ma extracts, gastrodin and *p*-hydroxybenzyl alcohol were less active or inactive as compared with the polybenzyls. Therefore, the producible polybenzyls during processing and decocting of the drug material would have some important contributions to enhance the clinic effects of tian ma decoction.

## Conclusions

Thirteen undescribed polybenzyls including six tetramers, five pentamers, and two hexamers, along with one known analogue, were isolated from the water extract of tian ma and the refluxed water solution of *p*-hydroxybenzyl alcohol. In addition, fourteen undescribed polybenzyls including three tetramers, three pentamers, and eight hexamers, together with one known tetramer, were isolated from the refluxed water solution of *p*-hydroxybenzyl alcohol. UPLC/HRESIMS analysis of the commercial available processed tian ma and the fresh *G. elata* rhizomes unequivocally proves that the diverse polybenzyls are generable from *p*-hydroxybenzyl alcohol during the processing and decocting. The in vitro bioactivity assays revealed that the polybenzyls were much more active than the main chemical components *p*-hydroxybenzyl alcohol and gastrodin. These results explain why the steamed and dried *G. elata* rhizomes are classically and clinically utilized instead of the unprocessed and fresh rhizomes and why patients need to take decoctions of tian ma instead of eating the rhizomes. This study provides experimental evidences of medicinal chemistry behind the processing and decocting protocols of TCM including tian ma.

## Experimental

See Supporting information.

## Supplementary Information

Additional file1 (PDF 54257 kb)

## Data Availability

All data generated or analyzed during this study are included in this published article and its supplementary information files.
