# Supplementary material for: Insight into medicinal chemistry behind traditional Chinese medicines: polybenzyls in Gastrodia elata
Source: Nat Prod Bioprospect. 2026 Sep 11;16(1):87. doi: 10.1007/s13659-026-00625-z (PMC13569811; doi:10.1007/s13659-026-00625-z)
Supplement: Supplementary file 1 [file 13659_2026_625_MOESM1_ESM.pdf]

Insight into medicinal chemistry behind traditional Chinese medicines: polybenzyls in *Gastrodia elata*

Yanan Wang[†], Wenjing Wang[†], Min Zhang, Chenggen Zhu, Chengbo Xu, Xia Li, Xiaoqiang Lei, Qinglan Guo[*], and Jiangong Shi*

State Key Laboratory of Bioactive Substance and Function of Natural Medicines, Institute of Materia Medica, Chinese Academy of Medical Sciences and Peking Union Medical College, Beijing 100050, people's Republic of China

Supporting Information

List of Contents

No.	Content	Page
1	**1. General experimental procedures**	S14
2	**2. Plant material**	S15
3	**3. Extraction and isolation**	S15
4	**Chemical-physical properties and spectroscopic data of compounds 1−13**	S16
5	**Table S1** NMR spectroscopic data of compounds **1−6**	S19
6	**Table S2** NMR spectroscopic data of compounds **7−11** in acetone-d_6	S21
7	**Table S3** NMR spectroscopic data for compounds **12** and **13** in acetone-d_6	S22
8	**4. Isolation of the refluxed aqueous solution of *p*-hydroxybenzyl alcohol**	S22
9	**Chemical-physical properties and spectroscopic data of compounds 15−28**	S24
10	**Table S4** NMR spectroscopic data of compounds **15–17** in acetone-d_6	S25
11	**Table S5** NMR Spectroscopic Data of Compounds **18–20** in acetone-d_6	S26
12	**Table S6** NMR Spectroscopic Data for Compounds **21–24** in acetone-d_6	S28
13	**Table S7** NMR Spectroscopic Data for Compounds **25–28** in acetone-d_6	S30
14	**5. Synthesis of 4, 5, and 13**	S30
15	**6. Preparation and UPLC/HRESIMS analysis of the *p*-hydroxybenzyl alcohol**	S31
16	**7. Preparation and UPLC/HRESIMS analysis of the fresh *G. elata* rhizomes and Tian ma extracts**	S31
17	**8. Bioassays**	S31
18	**8.1 Protective effect against cytotoxicity induced PC12 cell damage**	S31
19	**Table S8** and **Table S9** Survival rate of PC12 cells (10 μM, means ± SD, n = 3)	S32
20	**8.2 Anti-HIV-1 (VSVG/HIV-luc) activity assay**	S32
21	**Table S10** Inhibition activity against HIV-1 replication in 293T cells	S33
22	**8.3 Inhibitory activity against NO release in LPS-induced RAW 264.7 cells**	S33
23	**Table S11** and **Table S12** Inhibition rate against NO release in LPS-induced RAW 264.7 cells (10 *μ*M)	S33/ S34
24	**8.4 Inhibitory activity assay against Fe^{2+}-cysteine induced lipid peroxidation of liver microsomes**	S34
25	**Table S13** Inhibition rate against Fe^{2+}-cysteine induced lipid peroxidation of liver microsomes	S35
26	**8.5 Inhibition assay against TNF-α secretion in LPS-induced RAW264.7**	S35
27	**Table S14** Inhibitory activity against TNF-*α* secretion in LPS-induced RAW 264.7 cells (10 *μ*M)	S35
28	**8.6 Assay for α-glucosidase inhibitors**	S35
29	**Table S15** Inhibition rate against *α*-glucosidase activity	S36
30	**8.7 Assay for PTP1B (human *p*rotein tyrosine *p*hosphatase 1B) inhibitors**	S36
31	**Table S16** Inhibition rate against PTP1B actvity	S37
32	**8.8 Assay against replication of influenza virus A/Hanfang/359/95 (H3N3)**	S37
33	**Table S17** Anti-A/Hanfang/359/95 (H3N3) activity	S38
34	**8.9 Assay against replication of coxsackievirus B3 type (CVB3)**	S38
35	**Table S18** Activity against CVB3 replication	S39
36	**Figure S1.** The UV spectrum of compound **1**	S40
37	**Figure S2.** The IR spectrum of compound **1**	S41
38	**Figure S3.** The ESIMS of compound **1**	S42
39	**Figure S4.** The HR-ESIMS report of compound **1** (page 1)	S43
40	**Figure S5.** The HR-ESIMS report of compound **1** (page 2)	S44
41	**Figure S6.** The HR-ESIMS report of compound **1** (page 3)	S45

Figure S7. The ^{1}H NMR spectrum of compound **1** in acetone-d_6 (500 MHz) S46
Figure S8. The ^{13}C NMR spectrum of compound **1** in acetone-d_6 (125 MHz) S47
Figure S9. The DEPT spectrum of compound **1** in acetone-d_6 (125 MHz) S48
Figure S10. The ^{1}H-^{1}H COSY spectrum of compound **1** in acetone-d_6 (500 MHz) S49
Figure S11. The HSQC spectrum of compound **1** in acetone-d_6 (500 MHz for ^{1}H) S50
Figure S12. The HMBC spectrum of compound **1** in acetone-d_6 (500 MHz for ^{1}H) S51
Figure S13. The ^{1}H NMR spectrum of compound **1** in DMSO-d_6 (500 MHz) S52
Figure S14. The ^{13}C NMR spectrum of compound **1** in DMSO-d_6 (125 MHz) S53
Figure S15. The DEPT spectrum of compound **1** in DMSO-d_6 (125 MHz) S54
Figure S16. The ^{1}H-^{1}H COSY spectrum of compound **1** in DMSO-d_6 (500 MHz) S55
Figure S17. The HMQC spectrum of compound **1** in DMSO-d_6 (500 MHz for ^{1}H) S56
Figure S18. The HMBC spectrum of compound **1** in DMSO-d_6 (500 MHz for ^{1}H) S57
Figure S19. The UV spectrum of compound **2** S58
Figure S20. The IR spectrum of compound **2** S59
Figure S21. The ESIMS of compound **2** S60
Figure S22. The HR-ESIMS report of compound **2** (page 1) S61
Figure S23. The HR-ESIMS report of compound **2** (page 2) S62
Figure S24. The HR-ESIMS report of compound **2** (page 3) S63
Figure S25. The ^{1}H NMR spectrum of compound **2** in DMSO-d_6 (500 MHz) S64
Figure S26. The ^{13}C NMR spectrum of compound **2** in DMSO-d_6 (125 MHz) S65
Figure S27. The DEPT spectrum of compound **2** in DMSO-d_6 (125 MHz) S66
Figure S28. The ^{1}H-^{1}H COSY spectrum of compound **2** in DMSO-d_6 (500 MHz) S67
Figure S29. The HSQC spectrum of compound **2** in DMSO-d_6 (500 MHz for ^{1}H) S68
Figure S30. The HMBC spectrum of compound **2** in DMSO-d_6 (500 MHz for ^{1}H) S69
Figure S31. The UV spectrum of compound **3** S70
Figure S32. The IR spectrum of compound **3** S71
Figure S33. The ESIMS of compound **3** S72
Figure S34. The HR-ESIMS report of compound **3** (page 1) S73
Figure S35. The HR-ESIMS report of compound **3** (page 2) S74
Figure S36. The HR-ESIMS report of compound **3** (page 3) S75
Figure S37. The ^{1}H NMR spectrum of compound **3** in DMSO-d_6 (600 MHz) S76
Figure S38. The ^{13}C NMR spectrum of compound **3** in DMSO-d_6 (150 MHz) S77
Figure S39. The DEPT spectrum of compound **3** in DMSO-d_6 (150 MHz) S78
Figure S40. The ^{1}H-^{1}H COSY spectrum of compound **3** in DMSO-d_6 (600 MHz) S79
Figure S41. The HSQC spectrum of compound **3** in DMSO-d_6 (600 MHz for ^{1}H) S80
Figure S42. The HMBC spectrum of compound **3** in DMSO-d_6 (600 MHz for ^{1}H) S81
Figure S43. The UV spectrum of compound **4** S82
Figure S44. The IR spectrum of compound **4** S83
Figure S45. The ESIMS of compound **4** S84
Figure S46. The HR-ESIMS report of compound **4** (page 1) S85
Figure S47. The HR-ESIMS report of compound **4** (page 2) S86
Figure S48. The HR-ESIMS report of compound **4** (page 3) S87
Figure S49. The ^{1}H NMR spectrum of compound **4** in DMSO-d_6 (500 MHz) S88
Figure S50. The ^{13}C NMR spectrum of compound **4** in DMSO-d_6 (125 MHz) S89
Figure S51. The DEPT spectrum of compound **4** in DMSO-d_6 (125 MHz) S90
Figure S52. The ^{1}H-^{1}H COSY spectrum of compound **4** in DMSO-d_6 (500 MHz) S91

Figure S53. The HSQC spectrum of compound **4** in DMSO-d_6 (500 MHz for ^{1}H) S92
Figure S54. The HMBC spectrum of compound **4** in DMSO-d_6 (500 MHz for ^{1}H) S93
Figure S55. The ^{1}H NMR spectrum of compound **4** in acetone-d_6 (500 MHz) S94
Figure S56. The ^{13}C NMR spectrum of compound **4** in acetone-d_6 (125 MHz) S95
Figure S57. The UV spectrum of compound **5** S96
Figure S58. The IR spectrum of compound **5** S97
Figure S59. The ESIMS of compound **5** S98
Figure S60. The HR-ESIMS report of compound **5** (page 1) S99
Figure S61. The HR-ESIMS report of compound **5** (page 2) S100
Figure S62. The HR-ESIMS report of compound **5** (page 3) S101
Figure S63. The ^{1}H NMR spectrum of compound **5** in DMSO-d_6 (500 MHz) S102
Figure S64. The ^{13}C NMR spectrum of compound **5** in DMSO-d_6 (125 MHz) S103
Figure S65. The DEPT spectrum of compound **5** in DMSO-d_6 (125 MHz) S104
Figure S66. The ^{1}H-^{1}H COSY spectrum of compound **5** in DMSO-d_6 (500 MHz) S105
Figure S67. The HSQC spectrum of compound **5** in DMSO-d_6 (500 MHz for ^{1}H) S106
Figure S68. The HMBC spectrum of compound **5** in DMSO-d_6 (500 MHz for ^{1}H) S107
Figure S69. The ^{1}H NMR spectrum of compound **5** in acetone-d_6 (500 MHz) S108
Figure S70. The ^{13}C NMR spectrum of compound **5** in acetone-d_6 (125 MHz) S109
Figure S71. The UV spectrum of compound **6** S110
Figure S72. The IR spectrum of compound **6** S111
Figure S73. The ESIMS of compound **6** S112
Figure S74. The HR-ESIMS report of compound **6** (page 1) S113
Figure S75. The HR-ESIMS report of compound **6** (page 2) S114
Figure S76. The HR-ESIMS report of compound **6** (page 3) S115
Figure S77. The ^{1}H NMR spectrum of compound **6** in DMSO-d_6 (500 MHz) S116
Figure S78. The ^{13}C NMR spectrum of compound **6** in DMSO-d_6 (125 MHz) S117
Figure S79. The DEPT spectrum of compound **6** in DMSO-d_6 (125 MHz) S118
Figure S80. The ^{1}H-^{1}H COSY spectrum of compound **6** in DMSO-d_6 (500 MHz) S119
Figure S81. The HSQC spectrum of compound **6** in DMSO-d_6 (500 MHz for ^{1}H) S120
Figure S82. The HMBC spectrum of compound **6** in DMSO-d_6 (500 MHz for ^{1}H) S121
Figure S83. The ^{1}H NMR spectrum of compound **6** in acetone-d_6 (500 MHz) S122
Figure S84. The ^{13}C NMR spectrum of compound **6** in acetone-d_6 (125 MHz) S123
Figure S85. The UV spectrum of compound **7** S124
Figure S86. The IR spectrum of compound **7** S125
Figure S87. The ESIMS of compound **7** S126
Figure S88. The HR-ESIMS report of compound **7** (page 1) S127
Figure S89. The HR-ESIMS report of compound **7** (page 2) S128
Figure S90. The HR-ESIMS report of compound **7** (page 3) S129
Figure S91. The ^{1}H NMR spectrum of compound **7** in acetone-d_6 (600 MHz) S130
Figure S92. The ^{13}C NMR spectrum of compound **7** in acetone-d_6 (150 MHz) S131
Figure S93. The DEPT spectrum of compound **7** in acetone-d_6 (150 MHz) S132
Figure S94. The ^{1}H-^{1}H COSY spectrum of compound **7** in acetone-d_6 (600 MHz) S133
Figure S95. The HSQC spectrum of compound **7** in acetone-d_6 (600 MHz for ^{1}H) S134
Figure S96. The HMBC spectrum of compound **7** in acetone-d_6 (600 MHz for ^{1}H) S135
Figure S97. The NOESY1D spectrum of compound **7** in acetone-d_6 (500 MHz) S136
Figure S98. The UV spectrum of compound **8** S137

Figure S99. The IR spectrum of compound **8** S138
Figure S100. The ESIMS of compound **8** S139
Figure S101. The HR-ESIMS report of compound **8** (page 1) S140
Figure S102. The HR-ESIMS report of compound **8** (page 2) S141
Figure S103. The HR-ESIMS report of compound **8** (page 3) S142
Figure S104. The ^{1}H NMR spectrum of compound **8** in acetone-d_6 (600 MHz) S143
Figure S105. The ^{13}C NMR spectrum of compound **8** in acetone-d_6 (150 MHz) S144
Figure S106. The DEPT spectrum of compound **8** in acetone-d_6 (150 MHz) S145
Figure S107. The ^{1}H-^{1}H COSY spectrum of compound **8** in acetone-d_6 (600 MHz) S146
Figure S108. The HSQC spectrum of compound **8** in acetone-d_6 (600 MHz for ^{1}H) S147
Figure S109. The HMBC spectrum of compound **8** in acetone-d_6 (600 MHz for ^{1}H) S148
Figure S110. The NOESY1D spectrum of compound **8** in acetone-d_6 (500 MHz) S149
Figure S111. The UV spectrum of compound **9** S150
Figure S112. The IR spectrum of compound **9** S151
Figure S113. The ESIMS of compound **9** S152
Figure S114. The HR-ESIMS report of compound **9** (page 1) S153
Figure S115. The HR-ESIMS report of compound **9** (page 2) S154
Figure S116. The HR-ESIMS report of compound **9** (page 3) S155
Figure S117. The ^{1}H NMR spectrum of compound **9** in acetone-d_6 (600 MHz) S156
Figure S118. The ^{13}C NMR spectrum of compound **9** in acetone-d_6 (150 MHz) S157
Figure S119. The DEPT spectrum of compound **9** in acetone-d_6 (150 MHz) S158
Figure S120. The ^{1}H-^{1}H COSY spectrum of compound **9** in acetone-d_6 (600 MHz) S159
Figure S121. The HSQC spectrum of compound **9** in acetone-d_6 (600 MHz for ^{1}H) S160
Figure S122. The HMBC spectrum of compound **9** in acetone-d_6 (600 MHz for ^{1}H) S161
Figure S123. The NOESY1D spectrum of compound **9** in acetone-d_6 (500 MHz) S162
Figure S124. The UV spectrum of compound **10** S163
Figure S125. The IR spectrum of compound **10** S164
Figure S126. The ESIMS of compound **10** S165
Figure S127. The HR-ESIMS report of compound **10** (page 1) S166
Figure S128. The HR-ESIMS report of compound **10** (page 2) S167
Figure S129. The HR-ESIMS report of compound **10** (page 3) S168
Figure S130. The ^{1}H NMR spectrum of compound **10** in acetone-d_6 (600 MHz) S169
Figure S131. The ^{13}C NMR spectrum of compound **10** in acetone-d_6 (150 MHz) S170
Figure S132. The DEPT spectrum of compound **10** in acetone-d_6 (150 MHz) S171
Figure S133. The ^{1}H-^{1}H COSY spectrum of compound **10** in acetone-d_6 (600 MHz) S172
Figure S134. The HSQC spectrum of compound **10** in acetone-d_6 (600 MHz for ^{1}H) S173
Figure S135. The HMBC spectrum of compound **10** in acetone-d_6 (600 MHz for ^{1}H) S174
Figure S136. The NOESY1D spectrum of compound **10** in acetone-d_6 (500 MHz) S175
Figure S137. The UV spectrum of compound **11** S176
Figure S138. The IR spectrum of compound **11** S177
Figure S139. The ESIMS of compound **11** S178
Figure S140. The HR-ESIMS report of compound **11** (page 1) S179
Figure S141. The HR-ESIMS report of compound **11** (page 2) S180
Figure S142. The HR-ESIMS report of compound **11** (page 3) S181
Figure S143. The ^{1}H NMR spectrum of compound **11** in acetone-d_6 (800 MHz) S182
Figure S144. The ^{13}C NMR spectrum of compound **11** in acetone-d_6 (200 MHz) S183

180 **Figure S145.** The DEPT spectrum of compound **11** in acetone-d_6 (200 MHz) S184
181 **Figure S146.** The ^{1}H-^{1}H COSY spectrum of compound **11** in acetone-d_6 (800 MHz) S185
182 **Figure S147.** The HSQC spectrum of compound **11** in acetone-d_6 (800 MHz for ^{1}H) S186
183 **Figure S148.** The HMBC spectrum of compound **11** in acetone-d_6 (800 MHz for ^{1}H) S187
184 **Figure S149.** The NOESY spectrum of compound **11** in acetone-d_6 (800 MHz) S188
185 **Figure S150.** The UV spectrum of compound **12** S189
186 **Figure S151.** The IR spectrum of compound **12** S190
187 **Figure S152.** The ESIMS of compound **12** S191
188 **Figure S153.** The HR-ESIMS report of compound **12** (page 1) S192
189 **Figure S154.** The HR-ESIMS report of compound **12** (page 2) S193
190 **Figure S155.** The HR-ESIMS report of compound **12** (page 3) S194
191 **Figure S156.** The ^{1}H NMR spectrum of compound **12** in acetone-d_6 (600 MHz) S195
192 **Figure S157.** The ^{13}C NMR spectrum of compound **12** in acetone-d_6 (150 MHz) S196
193 **Figure S158.** The DEPT spectrum of compound **12** in acetone-d_6 (150 MHz) S197
194 **Figure S159.** The ^{1}H-^{1}H COSY spectrum of compound **12** in acetone-d_6 (600 MHz) S198
195 **Figure S160.** The HSQC spectrum of compound **12** in acetone-d_6 (600 MHz for ^{1}H) S199
196 **Figure S161.** The HMBC spectrum of compound **12** in acetone-d_6 (600 MHz for ^{1}H) S200
197 **Figure S162.** The UV spectrum of compound **13** S201
194 **Figure S163.** The IR spectrum of compound **13** S202
195 **Figure S164.** The ESIMS of compound **13** S203
196 **Figure S165.** The HR-ESIMS report of compound **13** (page 1) S204
197 **Figure S166.** The HR-ESIMS report of compound **13** (page 2) S205
198 **Figure S167.** The HR-ESIMS report of compound **13** (page 3) S206
199 **Figure S168.** The ^{1}H NMR spectrum of compound **13** in acetone-d_6 (600 MHz) S207
200 **Figure S169.** The ^{13}C NMR spectrum of compound **13** in acetone-d_6 (150 MHz) S208
201 **Figure S170.** The DEPT spectrum of compound **13** in acetone-d_6 (150 MHz) S209
202 **Figure S171.** The ^{1}H-^{1}H COSY spectrum of compound **13** in acetone-d_6 (600 MHz) S210
203 **Figure S172.** The HSQC spectrum of compound **13** in acetone-d_6 (600 MHz for ^{1}H) S211
204 **Figure S173.** The HMBC spectrum of compound **13** in acetone-d_6 (600 MHz for ^{1}H) S212
205 **Figure S174.** The UV spectrum of compound **14** S213
206 **Figure S175.** The IR spectrum of compound **14** S214
207 **Figure S176.** The ESIMS of compound **14** S215
208 **Figure S177.** The HR-ESIMS report of compound **14** (page 1) S216
209 **Figure S178.** The HR-ESIMS report of compound **14** (page 2) S217
210 **Figure S179.** The HR-ESIMS report of compound **14** (page 3) S218
211 **Figure S180.** The ^{1}H NMR spectrum of compound **14** in acetone-d_6 (600 MHz) S219
212 **Figure S181.** The ^{13}C NMR spectrum of compound **14** in acetone-d_6 (150 MHz) S220
213 **Figure S182.** The DEPT spectrum of compound **14** in acetone-d_6 (150 MHz) S221
214 **Figure S183.** The ^{1}H-^{1}H COSY spectrum of compound **14** in acetone-d_6 (600 MHz) S222
215 **Figure S184.** The HSQC spectrum of compound **14** in acetone-d_6 (600 MHz for ^{1}H) S223
216 **Figure S185.** The HMBC spectrum of compound **14** in acetone-d_6 (600 MHz for ^{1}H) S224
217 **Figure S186.** The NOESY1D spectrum of compound **14** in acetone-d_6 (500 MHz) S225
218 **Figure S187**. The IR spectrum of compound **15** S226
219 **Figure S188.** The UV spectrum of compound **15** in MeOH S227
220 **Figure S189**. The HR-ESIMS report of compound **15** S228
221 **Figure S190**. The ^{1}H NMR spectrum of compound **15** in acetone-d_6 (600 MHz) S229

Figure S191. The ^{13}C NMR spectrum of compound **15** in acetone-d_6 (150 MHz) S230
Figure S192. The DEPT spectrum of compound **15** in acetone-d_6 (150 MHz) S231
Figure S193. The ^{1}H-^{1}H COSY spectrum of compound **15** in acetone-d_6 (600 MHz) S232
Figure S194. The HSQC spectrum of compound **15** in acetone-d_6 (600 MHz for ^{1}H) S233
Figure S195. The HMBC spectrum of compound **15** in acetone-d_6 (600 MHz for ^{1}H) S234
Figure S196. The IR spectrum of compound **16** in MeOH S235
Figure S197. The UV spectrum of compound **16** in MeOH S236
Figure S198. The HR-ESIMS report of compound **16** S237
Figure S199. The ^{1}H NMR spectrum of compound **16** in acetone-d_6 (600 MHz) S238
Figure S200. The ^{13}C NMR spectrum of compound **16** in acetone-d_6 (150 MHz) S239
Figure S201. The DEPT spectrum of compound **16** in acetone-d_6 (150 MHz) S240
Figure S202. The ^{1}H-^{1}H COSY spectrum of compound **16** in acetone-d_6 (600 MHz) S241
Figure S203. The HSQC spectrum of compound **16** in acetone-d_6 (600 MHz for ^{1}H) S242
Figure S204. The HMBC spectrum of compound **16** in acetone-d_6 (600 MHz for ^{1}H) S243
Figure S205. The IR spectrum of compound **17** in MeOH S244
Figure S206. The UV spectrum of compound **17** in MeOH S245
Figure S207. The HR-ESIMS report of compound **17** S246
Figure S208. The ^{1}H NMR spectrum of compound **17** in acetone-d_6 (600 MHz) S247
Figure S209. The ^{13}C NMR spectrum of compound **17** in acetone-d_6 (150 MHz) S248
Figure S210. The DEPT spectrum of compound **17** in acetone-d_6 (150 MHz) S249
Figure S211. The ^{1}H-^{1}H COSY spectrum of compound **17** in acetone-d_6 (600 MHz) S250
Figure S212. The HSQC spectrum of compound **17** in acetone-d_6 (600 MHz for ^{1}H) S251
Figure S213. The HMBC spectrum of compound **17** in acetone-d_6 (600 MHz for ^{1}H) S252
Figure S214. The IR spectrum of compound **18** in MeOH S253
Figure S215. The UV spectrum of compound **18** in MeOH S254
Figure S216. The HR-ESIMS report of compound **18** S255
Figure S217. The ^{1}H NMR spectrum of compound **18** in acetone-d_6 (600 MHz) S256
Figure S218. The ^{13}C NMR spectrum of compound **18** in acetone-d_6 (150 MHz) S257
Figure S219. The DEPT spectrum of compound **18** in acetone-d_6 (150 MHz) S258
Figure S220. The ^{1}H-^{1}H COSY spectrum of compound **18** in acetone-d_6 (600 MHz) S259
Figure S221. The HSQC spectrum of compound **18** in acetone-d_6 (600 MHz for ^{1}H) S260
Figure S222. The HMBC spectrum of compound **18** in acetone-d_6 (600 MHz for ^{1}H) S261
Figure S223. The IR spectrum of compound **19** in MeOH S262
Figure S224. The UV spectrum of compound **19** in MeOH S263
Figure S225. The HR-ESIMS report of compound **19** S264
Figure S226. The ^{1}H NMR spectrum of compound **19** in acetone-d_6 (600 MHz) S265
Figure S227. The ^{13}C NMR spectrum of compound **19** in acetone-d_6 (150 MHz) S266
Figure S228. The DEPT spectrum of compound **19** in acetone-d_6 (150 MHz) S267
Figure S229. The ^{1}H-^{1}H COSY spectrum of compound **19** in acetone-d_6 (600 MHz) S268
Figure S230. The HSQC spectrum of compound **19** in acetone-d_6 (600 MHz for ^{1}H) S269
Figure S231. The HMBC spectrum of compound **19** in acetone-d_6 (600 MHz for ^{1}H) S270
Figure S232. The IR spectrum of compound **20** in MeOH S271
Figure S233. The UV spectrum of compound **20** in MeOH S272
Figure S234. The HR-ESIMS report of compound **20** S273
Figure S235. The ^{1}H NMR spectrum of compound **20** in acetone-d_6 (600 MHz) S274
Figure S236. The ^{13}C NMR spectrum of compound **20** in acetone-d_6 (150 MHz) S275

Figure S237. The DEPT spectrum of compound **20** in acetone-d_6 (150 MHz) S276
Figure S238. The ^{1}H-^{1}H COSY spectrum of compound **20** in acetone-d_6 (600 MHz) S277
Figure S239. The HSQC spectrum of compound **20** in acetone-d_6 (600 MHz for ^{1}H) S278
Figure S240. The HMBC spectrum of compound **20** in acetone-d_6 (600 MHz for ^{1}H) S279
Figure S241. The IR spectrum of compound **21** in MeOH S280
Figure S242. The UV spectrum of compound **21** in MeOH S281
Figure S243. The HR-ESIMS report of compound **21** S282
Figure S244. The ^{1}H NMR spectrum of compound **21** in acetone-d_6 (600 MHz) S283
Figure S245. The ^{13}C NMR spectrum of compound **21** in acetone-d_6 (150 MHz) S284
Figure S246. The DEPT spectrum of compound **21** in acetone-d_6 (150 MHz) S285
Figure S247. The ^{1}H-^{1}H COSY spectrum of compound **21** in acetone-d_6 (600 MHz) S286
Figure S248. The HSQC spectrum of compound **21** in acetone-d_6 (600 MHz for ^{1}H) S287
Figure S249. The HMBC spectrum of compound **21** in acetone-d_6 (600 MHz for ^{1}H) S288
Figure S250. The IR spectrum of compound **22** in MeOH S289
Figure S251. The UV spectrum of compound **22** in MeOH S290
Figure S252. The HR-ESIMS report of compound **22** S291
Figure S253. The ^{1}H NMR spectrum of compound **22** in acetone-d_6 (600 MHz) S292
Figure S254. The ^{13}C NMR spectrum of compound **22** in acetone-d_6 (150 MHz) S293
Figure S255. The DEPT spectrum of compound **22** in acetone-d_6 (150 MHz) S294
Figure S256. The ^{1}H-^{1}H COSY spectrum of compound **22** in acetone-d_6 (600 MHz) S295
Figure S257. The HSQC spectrum of compound **22** in acetone-d_6 (600 MHz for ^{1}H) S296
Figure S258. The HMBC spectrum of compound **22** in acetone-d_6 (600 MHz for ^{1}H) S297
Figure S259. The IR spectrum of compound **23** in MeOH S298
Figure S260. The UV spectrum of compound **23** in MeOH S299
Figure S261. The HR-ESIMS report of compound **23** S300
Figure S262. The ^{1}H NMR spectrum of compound **23** in acetone-d_6 (600 MHz) S301
Figure S263. The ^{13}C NMR spectrum of compound **23** in acetone-d_6 (150 MHz) S302
Figure S264. The DEPT spectrum of compound **23** in acetone-d_6 (150 MHz) S303
Figure S265. The ^{1}H-^{1}H COSY spectrum of compound **23** in acetone-d_6 (600 MHz) S304
Figure S266. The HSQC spectrum of compound **23** in acetone-d_6 (600 MHz for ^{1}H) S305
Figure S267. The HMBC spectrum of compound **23** in acetone-d_6 (600 MHz for ^{1}H) S306
Figure S268. The IR spectrum of compound **24** in MeOH S307
Figure S269. The UV spectrum of compound **24** in MeOH S308
Figure S270. The HR-ESIMS report of compound **24** S309
Figure S271. The ^{1}H NMR spectrum of compound **24** in acetone-d_6 (600 MHz) S310
Figure S272. The ^{13}C NMR spectrum of compound **24** in acetone-d_6 (150 MHz) S311
Figure S273. The DEPT spectrum of compound **24** in acetone-d_6 (150 MHz) S312
Figure S274. The ^{1}H-^{1}H COSY spectrum of compound **24** in acetone-d_6 (600 MHz) S313
Figure S275. The HSQC spectrum of compound **24** in acetone-d_6 (600 MHz for ^{1}H) S314
Figure S276. The HMBC spectrum of compound **24** in acetone-d_6 (600 MHz for ^{1}H) S315
Figure S277. The IR spectrum of compound **25** in MeOH S316
Figure S278. The UV spectrum of compound **25** in MeOH S317
Figure S279. The HR-ESIMS report of compound **25** S318
Figure S280. The ^{1}H NMR spectrum of compound **25** in acetone-d_6 (600 MHz) S319
Figure S281. The ^{13}C NMR spectrum of compound **25** in acetone-d_6 (150 MHz) S320
Figure S282. The DEPT spectrum of compound **25** in acetone-d_6 (150 MHz) S321

252 **Figure S283**. The ^{1}H-^{1}H COSY spectrum of compound **25** in acetone-d_6 (600 MHz) S322
253 **Figure S284.** The HSQC spectrum of compound **25** in acetone-d_6 (600 MHz for ^{1}H) S323
254 **Figure S285.** The HMBC spectrum of compound **25** in acetone-d_6 (600 MHz for ^{1}H) S324
255 **Figure S286**. The IR spectrum of compound **26** in MeOH S325
194 **Figure S287.** The UV spectrum of compound **26** in MeOH S326
195 **Figure S288**. The HR-ESIMS report of compound **26** S327
196 **Figure S289**. The ^{1}H NMR spectrum of compound **26** in acetone-d_6 (600 MHz) S328
197 **Figure S290**. The ^{13}C NMR spectrum of compound **26** in acetone-d_6 (150 MHz) S329
198 **Figure S291.** The DEPT spectrum of compound **26** in acetone-d_6 (150 MHz) S330
199 **Figure S292**. The ^{1}H-^{1}H COSY spectrum of compound **26** in acetone-d_6 (600 MHz) S331
200 **Figure S293.** The HSQC spectrum of compound **26** in acetone-d_6 (600 MHz for ^{1}H) S332
201 **Figure S294.** The HMBC spectrum of compound **26** in acetone-d_6 (600 MHz for ^{1}H) S333
202 **Figure S295**. The IR spectrum of compound **27** in MeOH S334
203 **Figure S296.** The UV spectrum of compound **27** in MeOH S335
204 **Figure S297**. The HR-ESIMS report of compound **27** S336
205 **Figure S298**. The ^{1}H NMR spectrum of compound **27** in acetone-d_6 (600 MHz) S337
206 **Figure S299**. The ^{13}C NMR spectrum of compound **27** in acetone-d_6 (150 MHz) S338
207 **Figure S300.** The DEPT spectrum of compound **27** in acetone-d_6 (150 MHz) S339
208 **Figure S301**. The ^{1}H-^{1}H COSY spectrum of compound **27** in acetone-d_6 (600 MHz) S340
209 **Figure S302.** The HSQC spectrum of compound **27** in acetone-d_6 (600 MHz for ^{1}H) S341
210 **Figure S303.** The HMBC spectrum of compound **27** in acetone-d_6 (600 MHz for ^{1}H) S342
211 **Figure S304**. The 2D TOCSY spectrum of compound **27** in acetone-d6 (600 MHz) S343
212 **Figure S305**. The HSQC-TOCSY spectrum of compound **27** in acetone-d6 (600 MHz for 1H) S344
213 **Figure S306**. The IR spectrum of compound **28** in MeOH S345
214 **Figure S307.** The UV spectrum of compound **28** in MeOH S346
215 **Figure S308**. The HR-ESIMS report of compound **28** S347
216 **Figure S309**. The ^{1}H NMR spectrum of compound **28** in acetone-d_6 (600 MHz) S348
217 **Figure S310**. The ^{13}C NMR spectrum of compound **28** in acetone-d_6 (150 MHz) S349
218 **Figure S311.** The DEPT spectrum of compound **28** in acetone-d_6 (150 MHz) S350
219 **Figure S312**. The ^{1}H-^{1}H COSY spectrum of compound **28** in acetone-d_6 (600 MHz) S351
220 **Figure S313.** The HSQC spectrum of compound **28** in acetone-d_6 (600 MHz for ^{1}H) S352
221 **Figure S314.** The HMBC spectrum of compound **28** in acetone-d_6 (600 MHz for ^{1}H) S353
222 **Figure S315**. The IR spectrum of compound **29** in MeOH S354
223 **Figure S316.** The UV spectrum of compound **29** in MeOH S355
224 **Figure S317**. The HR-ESIMS report of compound **29** S356
225 **Figure S318**. The ^{1}H NMR spectrum of compound **29** in acetone-d_6 (600 MHz) S357
226 **Figure S319**. The ^{13}C NMR spectrum of compound **29** in acetone-d_6 (150 MHz) S358
227 **Figure S320.** The DEPT spectrum of compound **29** in acetone-d_6 (150 MHz) S359
228 **Figure S321.** The HSQC spectrum of compound **29** in acetone-d_6 (600 MHz for ^{1}H) S360
229 **Figure S322.** The HMBC spectrum of compound **29** in acetone-d_6 (600 MHz for ^{1}H) S361
230 **Figure S323.** Overlaid chromatograms of the extracted negative ion at *m/z* 455.187 $[M–H]^–$: (a) and (b) compounds **1** and **2** in CH_3CN, respectively; (c)–(e) H_2O, MeOH, and EtOH solutions of *p*-hydroxybenzyl alcohol were sonicated for 0.5 h then refluxed for 1.0 h, respectively S362
231 **Figure S324.** Overlaid chromatograms of the extracted negative ion at *m/z* 411.161 $[M–H]^–$: (a)−(c) compounds **4**−**6** in CH_3CN, respectively; (d)–(f) H_2O, MeOH, and EtOH solutions of *p*-hydroxybenzyl alcohol were sonicated for 0.5 h then refluxed for 1.0 h, respectively S363

232 **Figure S325**. Overlaid chromatograms of the extracted negative ion at *m/z* 517.202 $[M–H]^-$: (a)−(e) compounds **7−10** and **14** in CH_3CN, respectively; (f)–(h) H_2O, MeOH, and EtOH solutions of *p*-hydroxybenzyl alcohol were sonicated for 0.5 h then refluxed for 1.0 h, respectively S364

233 **Figure S326**. Overlaid chromatograms of the extracted negative ion at *m/z* 561.229 $[M–H]^-$: (a) compound **11** in CH_3CN; (b)–(d) H_2O, MeOH, and EtOH solutions of **11** were sonicated for 0.5 h then refluxed for 1.0 h, respectively S365

234 **Figure S327**. Overlaid chromatograms of the extracted negative ion at *m/z* 623.245 $[M–H]^-$: (a) compound **12** in CH_3CN, respectively; (b)–(d) H_2O, MeOH, and EtOH solutions of *p*-hydroxybenzyl alcohol were sonicated for 0.5 h then refluxed for 1.0 h, respectively S366

235 **Figure S328**. Overlaid chromatograms of the extracted negative ion at *m/z* 455.187 $[M–H]^-$: (a) and (b) compounds **1** and **2** in CH_3CN, respectively; (c)–(g) H_2O solution of *p*-hydroxybenzyl alcohol was sonicated for 0.5 h then refluxed for 1.0 h, 2.0 h, 4.0 h, and 6.0 h, respectively S367

236 **Figure S329**. Overlaid chromatograms of the extracted negative ion at *m/z* 411.161 $[M–H]^-$: (a)−(c) compounds **4−6** in CH_3CN, respectively; (d)–(f) H_2O solution of *p*-hydroxybenzyl alcohol was sonicated for 0.5 h then refluxed for 1.0 h, 2.0 h, 4.0 h, and 6.0 h, respectively S368

237 **Figure S330**. Overlaid chromatograms of the extracted negative ion at *m/z* 517.202 $[M–H]^-$: (a)−(c) compounds **7−9** in CH_3CN, respectively; (d)–(h) H_2O solution of *p*-hydroxybenzyl alcohol was sonicated for 0.5 h then refluxed for 1.0 h, 2.0 h, 4.0 h, and 6.0 h, respectively S369

238 **Figure S331**. Overlaid chromatograms of the extracted negative ion at *m/z* 517.202 $[M–H]^-$: (a) and (b) compounds **10** and **14** in CH_3CN, respectively; (b)–(g) H_2O solution of *p*-hydroxybenzyl alcohol was sonicated for 0.5 h then refluxed for 1.0 h, 2.0 h, 4.0 h, and 6.0 h, respectively S370

239 **Figure S332**. Overlaid chromatograms of the extracted negative ion at *m/z* 561.229 $[M–H]^-$: (a) compound **11** in CH_3CN; (b)–(f) H_2O solution of *p*-hydroxybenzyl alcohol was sonicated for 0.5 h then refluxed for 1.0 h, 2.0 h, 4.0 h, and 6.0 h, respectively S371

240 **Figure S333**. Overlaid chromatograms of the extracted negative ion at *m/z* 623.245 $[M–H]^-$: (a) compound **12** in CH_3CN; (b)–(f) H_2O solution of *p*-hydroxybenzyl alcohol was sonicated for 0.5 h then refluxed for 1.0 h, 2.0 h, 4.0 h, and 6.0 h, respectively S372

241 **Figure S334**. Overlaid chromatograms of the extracted negative ion at *m/z* 455.187 $[M–H]^-$: (a) and (b) compounds **1** and **2** in CH_3CN, respectively; (c)–(g) MeOH solution of *p*-hydroxybenzyl alcohol was sonicated for 0.5 h then refluxed for 1.0 h, 2.0 h, 4.0 h, and 6.0 h, respectively S373

242 **Figure S335**. Overlaid chromatograms of the extracted negative ion at *m/z* 411.161 $[M–H]^-$: (a)−(c) compounds **4−6** in CH_3CN, respectively; (d)–(h) MeOH solution of *p*-hydroxybenzyl alcohol was sonicated for 0.5 h then refluxed for 1.0 h, 2.0 h, 4.0 h, and 6.0 h, respectively S374

243 **Figure S336**. Overlaid chromatograms of the extracted negative ion at *m/z* 517.202 $[M–H]^-$: (a)−(c) compounds **7−9** in CH_3CN, respectively; (d)–(h) MeOH solution of *p*-hydroxybenzyl alcohol was sonicated for 0.5 h then refluxed for 1.0 h, 2.0 h, 4.0 h, and 6.0 h, respectively S375

244 **Figure S337**. Overlaid chromatograms of the extracted negative ion at *m/z* 517.202 $[M–H]^-$: (a) and (b) compounds **9** and **14** in CH_3CN, respectively; (c)–(g) MeOH solution of *p*-hydroxybenzyl alcohol was sonicated for 0.5 h then refluxed for 1.0 h, 2.0 h, 4.0 h, and 6.0 h, respectively S376

245 **Figure S338**. Overlaid chromatograms of the extracted negative ion at *m/z* 561.229 $[M–H]^-$: (a) compound **11** in CH_3CN; (b)–(f) MeOH solution of *p*-hydroxybenzyl alcohol was sonicated for 0.5 h then refluxed for 1.0 h, 2.0 h, 4.0 h, and 6.0 h, respectively S377

246 **Figure S339**. Overlaid chromatograms of the extracted negative ion at *m/z* 623.245 $[M–H]^-$: (a) compound **12** in CH_3CN; (b)–(f) MeOH solution of *p*-hydroxybenzyl alcohol was sonicated for 0.5 h then refluxed for 1.0 h, 2.0 h, 4.0 h, and 6.0 h, respectively S378

247 **Figure S340**. Overlaid chromatograms of the extracted negative ion at *m/z* 455.187 $[M–H]^-$: (a) and (b) compounds **1** and **2** in CH_3CN, respectively; (c)–(g) EtOH solution of *p*-hydroxybenzyl alcohol was sonicated for 0.5 h then refluxed for 1.0 h, 2.0 h, 4.0 h, and 6.0 h, respectively S379

248 **Figure S341**. Overlaid chromatograms of the extracted negative ion at *m/z* 411.161 $[M–H]^-$: (a)−(c) compounds **4−6** in CH_3CN, respectively; (d)–(h) EtOH solution of *p*-hydroxybenzyl alcohol was sonicated for 0.5 h then refluxed for 1.0 h, 2.0 h, 4.0 h, and 6.0 h, respectively S380

249 **Figure S342**. Overlaid chromatograms of the extracted negative ion at *m/z* 517.202 $[M–H]^-$: (a)−(c) compounds **7−9** in CH_3CN, respectively; (d)–(h) EtOH solution of *p*-hydroxybenzyl alcohol was sonicated for 0.5 h then refluxed for 1.0 h, 2.0 h, 4.0 h, and 6.0 h, respectively S381

250 **Figure S343**. Overlaid chromatograms of the extracted negative ion at *m/z* 517.202 $[M–H]^-$: (a) and (b) compounds **10** and **14** in CH_3CN, respectively; (c)–(g) EtOH solution of *p*-hydroxybenzyl alcohol was sonicated for 0.5 h then refluxed for 1.0 h, 2.0 h, 4.0 h, and 6.0 h, respectively S382

251 **Figure S344**. Overlaid chromatograms of the extracted negative ion at *m/z* 561.229 $[M–H]^-$: (a) compound **11** in CH_3CN; (b)–(f) EtOH solution of *p*-hydroxybenzyl alcohol was sonicated for 0.5 h then refluxed for 1.0 h, 2.0 h, 4.0 h, and 6.0 h, respectively S383

252 **Figure S345**. Overlaid chromatograms of the extracted negative ion at *m/z* 623.245 $[M–H]^-$: (a) compound **12** in CH_3CN; (b)–(f) EtOH solution of *p*-hydroxybenzyl alcohol was sonicated for 0.5 h then refluxed for 1.0 h, 2.0 h, 4.0 h, and 6.0 h, respectively S384

253 **Figure S346**. Overlaid chromatograms of the extracted negative ion at *m/z* 455.187 $[M–H]^-$: (a) and (b) compounds **1** and **2** in CH_3CN, respectively; (c)–(g) H_2O solution of “tianma” was sonicated for 0.5 h then refluxed for 1.0 h, 2.0 h, 4.0 h, and 6.0 h, respectively S385

254 **Figure S347**. Overlaid chromatograms of the extracted negative ion at *m/z* 411.161 $[M–H]^-$: (a)−(c) compounds **4−6** in CH_3CN, respectively; (d)–(h) H_2O solution of “tianma” was sonicated for 0.5 h then refluxed for 1.0 h, 2.0 h, 4.0 h, and 6.0 h, respectively S386

255 **Figure S348**. Overlaid chromatograms of the extracted negative ion at *m/z* 517.202 $[M–H]^-$: (a)−(c) compounds **7−9** in CH_3CN, respectively; (d)–(h) H_2O solution of “tianma” was sonicated for 0.5 h then refluxed for 1.0 h, 2.0 h, 4.0 h, and 6.0 h, respectively S387

254 **Figure S349**. Overlaid chromatograms of the extracted negative ion at *m/z* 517.202 $[M–H]^-$: (a) and (b) compounds **10** and **14** in CH_3CN, respectively; (c)–(g) H_2O solution of “tianma” was sonicated for 0.5 h then refluxed for 1.0 h, 2.0 h, 4.0 h, and 6.0 h, respectively S388

255 **Figure S350**. Overlaid chromatograms of the extracted negative ion at *m/z* 561.229 $[M–H]^-$: (a) compound **11** in CH_3CN; (b)–(f) H_2O solution of “tianma” was sonicated for 0.5 h then refluxed for 1.0 h, 2.0 h, 4.0 h, and 6.0 h, respectively S389

256 **Figure S351**. Overlaid chromatograms of the extracted negative ion at *m/z* 623.245 $[M–H]^-$: (a) compound **12** in CH_3CN; (b)–(f) H_2O solution of “tianma” was sonicated for 0.5 h then refluxed for 1.0 h, 2.0 h, 4.0 h, and 6.0 h, respectively S390

257 **Figure S352.** Overlaid chromatograms of the extracted negative ion at *m/z* 455.187 $[M–H]^-$: (a) and (b) compounds **1** and **2** in CH_3CN, respectively; (c)–(g) MeOH solution of “tianma” was sonicated for 0.5 h then refluxed for 1.0 h, 2.0 h, 4.0 h, and 6.0 h, respectively S391

258 **Figure S353**. Overlaid chromatograms of the extracted negative ion at *m/z* 411.161 $[M–H]^-$: (a)−(c) compounds **4−6** in CH_3CN, respectively; (d)–(h) MeOH solution of “tianma” was sonicated for 0.5 h then refluxed for 1.0 h, 2.0 h, 4.0 h, and 6.0 h, respectively S392

259 **Figure S354**. Overlaid chromatograms of the extracted negative ion at *m/z* 517.202 $[M–H]^{-}$: (a)−(c) compounds **7−9** in CH_3CN, respectively; (d)–(h) MeOH solution of "tianma" was sonicated for 0.5 h then refluxed for 1.0 h, 2.0 h, 4.0 h, and 6.0 h, respectively S393

260 **Figure S355**. Overlaid chromatograms of the extracted negative ion at *m/z* 517.202 $[M–H]^{-}$: (a) and (b) compounds **10** and **14** in CH_3CN, respectively; (c)–(g) MeOH solution of "tianma" was sonicated for 0.5 h then refluxed for 1.0 h, 2.0 h, 4.0 h, and 6.0 h, respectively S394

261 **Figure S356**. Overlaid chromatograms of the extracted negative ion at *m/z* 561.229 $[M–H]^{-}$: (a) compound **11** in CH_3CN; (b)–(f) MeOH solution of "tianma" was sonicated for 0.5 h then refluxed for 1.0 h, 2.0 h, 4.0 h, and 6.0 h, respectively S395

262 **Figure S357**. Overlaid chromatograms of the extracted negative ion at *m/z* 623.245 $[M–H]^{-}$: (a) compound **12** in CH_3CN; (b)–(f) MeOH solution of "tianma" was sonicated for 0.5 h then refluxed for 1.0 h, 2.0 h, 4.0 h, and 6.0 h, respectively S396

263 **Figure S358**. Overlaid chromatograms of the extracted negative ion at *m/z* 455.187 $[M–H]^{-}$: (a) and (b) compounds **1** and **2** in CH_3CN, respectively; (c)–(g) EtOH solution of "tianma" was sonicated for 0.5 h then refluxed for 1.0 h, 2.0 h, 4.0 h, and 6.0 h, respectively S397

264 **Figure S359**. Overlaid chromatograms of the extracted negative ion at *m/z* 411.161 $[M–H]^{-}$: (a)−(c) compounds **4−6** in CH_3CN, respectively; (d)–(h) EtOH solution of "tianma" was sonicated for 0.5 h then refluxed for 1.0 h, 2.0 h, 4.0 h, and 6.0 h, respectively S398

265 **Figure S360**. Overlaid chromatograms of the extracted negative ion at *m/z* 517.202 $[M–H]^{-}$: (a)−(c) compounds **7−9** in CH_3CN, respectively; (d)–(h) EtOH solution of "tianma" was sonicated for 0.5 h then refluxed for 1.0 h, 2.0 h, 4.0 h, and 6.0 h, respectively S399

266 **Figure S361**. Overlaid chromatograms of the extracted negative ion at *m/z* 517.202 $[M–H]^{-}$: (a) and (b) compounds **10** and **14** in CH_3CN, respectively; (c)–(g) EtOH solution of "tianma" was sonicated for 0.5 h then refluxed for 1.0 h, 2.0 h, 4.0 h, and 6.0 h, respectively S400

267 **Figure S362**. Overlaid chromatograms of the extracted negative ion at *m/z* 561.229 $[M–H]^{-}$: (a) compound **11** in CH_3CN; (b)–(f) EtOH solution of "tianma" was sonicated for 0.5 h then refluxed for 1.0 h, 2.0 h, 4.0 h, and 6.0 h, respectively S401

268 **Figure S363**. Overlaid chromatograms of the extracted negative ion at *m/z* 623.245 $[M–H]^{-}$: (a) compound **12** in CH_3CN; (b)–(f) EtOH solution of "tianma" was sonicated for 0.5 h then refluxed for 1.0 h, 2.0 h, 4.0 h, and 6.0 h, respectively S402

269 **Figure S364**. Overlaid chromatograms of the extracted negative ion at *m/z* 455.187 $[M–H]^{-}$: (a) and (b) compounds **1** and **2** in CH_3CN, respectively; (c)–(g) H_2O solution of fresh *G. elata* rhizome was sonicated for 0.5 h then refluxed for 1.0 h, 2.0 h, 4.0 h, and 6.0 h, respectively S403

270 **Figure S365**. Overlaid chromatograms of the extracted negative ion at *m/z* 411.161 $[M–H]^{-}$: (a)−(c) compounds **4−6** in CH_3CN, respectively; (d)–(h) H_2O solution of fresh *G. elata* rhizome was sonicated for 0.5 h then refluxed for 1.0 h, 2.0 h, 4.0 h, and 6.0 h, respectively S404

271 **Figure S366**. Overlaid chromatograms of the extracted negative ion at *m/z* 517.202 $[M–H]^{-}$: (a)−(c) compounds **7−9** in CH_3CN, respectively; (d)–(h) H_2O solution of fresh *G. elata* rhizome was sonicated for 0.5 h then refluxed for 1.0 h, 2.0 h, 4.0 h, and 6.0 h, respectively S405

272 **Figure S367**. Overlaid chromatograms of the extracted negative ion at *m/z* 517.202 $[M–H]^{-}$: (a) and (b) compounds **10** and **14** in CH_3CN, respectively; (c)–(g) H_2O solution of fresh *G. elata* rhizome was sonicated for 0.5 h then refluxed for 1.0 h, 2.0 h, 4.0 h, and 6.0 h, respectively S406

273 **Figure S368**. Overlaid chromatograms of the extracted negative ion at *m/z* 561.229 $[M–H]^{-}$: (a) compound **11** in CH_3CN; (b)–(f) H_2O solution of fresh *G. elata* rhizome was sonicated for 0.5 h then refluxed for 1.0 h, 2.0 h, 4.0 h, and 6.0 h, respectively S407

274	**Figure S369**. Overlaid chromatograms of the extracted negative ion at *m/z* 623.245 [M–H]$^-$: (a) compound **12** in CH_3CN; (b)–(f) H_2O solution of fresh *G. elata* rhizome was sonicated for 0.5 h then refluxed for 1.0 h, 2.0 h, 4.0 h, and 6.0 h, respectively	S408
275	**Figure S370**. Overlaid chromatograms of the extracted negative ion at *m/z* 455.187 [M–H]$^-$: (a) and (b) compounds **1** and **2** in CH_3CN, respectively; (c)–(g) MeOH solution of fresh *G. elata* rhizome was sonicated for 0.5 h then refluxed for 1.0 h, 2.0 h, 4.0 h, and 6.0 h, respectively	S409
276	**Figure S371**. Overlaid chromatograms of the extracted negative ion at *m/z* 411.161 [M–H]$^-$: (a)−(c) compounds **4−6** in CH_3CN, respectively; (d)–(h) MeOH solution of fresh *G. elata* rhizome was sonicated for 0.5 h then refluxed for 1.0 h, 2.0 h, 4.0 h, and 6.0 h, respectively	S410
277	**Figure S372**. Overlaid chromatograms of the extracted negative ion at *m/z* 517.202 [M–H]$^-$: (a)−(c) compounds **7−9** in CH_3CN, respectively; (d)–(h) MeOH solution of fresh *G. elata* rhizome was sonicated for 0.5 h then refluxed for 1.0 h, 2.0 h, 4.0 h, and 6.0 h, respectively	S411
278	**Figure S373**. Overlaid chromatograms of the extracted negative ion at *m/z* 517.202 [M–H]$^-$: (a) and (b) compounds **10** and **14** in CH_3CN, respectively; (c)–(g) MeOH solution of fresh *G. elata* rhizome was sonicated for 0.5 h then refluxed for 1.0 h, 2.0 h, 4.0 h, and 6.0 h, respectively	S412
279	**Figure S374**. Overlaid chromatograms of the extracted negative ion at *m/z* 561.229 [M–H]$^-$: (a) compound **11** in CH_3CN; (b)–(f) MeOH solution of fresh *G. elata* rhizome was sonicated for 0.5 h then refluxed for 1.0 h, 2.0 h, 4.0 h, and 6.0 h, respectively	S413
280	**Figure S369**. Overlaid chromatograms of the extracted negative ion at *m/z* 623.245 [M–H]$^-$: (a) compound **12** in CH_3CN; (b)–(f) MeOH solution of fresh *G. elata* rhizome was sonicated for 0.5 h then refluxed for 1.0 h, 2.0 h, 4.0 h, and 6.0 h, respectively	S414
281	**Figure S370**. Overlaid chromatograms of the extracted negative ion at *m/z* 455.187 [M–H]$^-$: (a) and (b) compounds **1** and **2** in CH_3CN, respectively; (c)–(g) EtOH solution of fresh *G. elata* rhizome was sonicated for 0.5 h then refluxed for 1.0 h, 2.0 h, 4.0 h, and 6.0 h, respectively	S415
282	**Figure S371**. Overlaid chromatograms of the extracted negative ion at *m/z* 411.161 [M–H]$^-$: (a)−(c) compounds **4−6** in CH_3CN, respectively; (d)–(h) EtOH solution of fresh *G. elata* rhizome was sonicated for 0.5 h then refluxed for 1.0 h, 2.0 h, 4.0 h, and 6.0 h, respectively	S416
283	**Figure S372**. Overlaid chromatograms of the extracted negative ion at *m/z* 517.202 [M–H]$^-$: (a)−(c) compounds **7−9** in CH_3CN, respectively; (d)–(h) EtOH solution of fresh *G. elata* rhizome was sonicated for 0.5 h then refluxed for 1.0 h, 2.0 h, 4.0 h, and 6.0 h, respectively	S417
284	**Figure S373**. Overlaid chromatograms of the extracted negative ion at *m/z* 517.202 [M–H]$^-$: (a) and (b) compounds **10** and **14** in CH_3CN, respectively; (c)–(g) EtOH solution of fresh *G. elata* rhizome was sonicated for 0.5 h then refluxed for 1.0 h, 2.0 h, 4.0 h, and 6.0 h, respectively	S418
285	**Figure S374**. Overlaid chromatograms of the extracted negative ion at *m/z* 561.229 [M–H]$^-$: (a) compound **11** in CH_3CN; (b)–(f) EtOH solution of fresh *G. elata* rhizome was sonicated for 0.5 h then refluxed for 1.0 h, 2.0 h, 4.0 h, and 6.0 h, respectively	S419
286	**Figure S375**. Overlaid chromatograms of the extracted negative ion at *m/z* 623.245 [M–H]$^-$: (a) compound **12** in CH_3CN; (b)–(f) EtOH solution of fresh *G. elata* rhizome was sonicated for 0.5 h then refluxed for 1.0 h, 2.0 h, 4.0 h, and 6.0 h, respectively	S420

1. General experimental procedures

UV spectra were measured on a V-650 spectrometer (JASCO, Tokyo, Japan). IR spectra were recorded on a Nicolet 5700 FT-IR microscope spectrometer (FT-IR microscope transmission) (Thermo Electron Corporation, Madison, WI, USA) and a Thermo Scientific Nicolet Is50 FT-IR ATR (Thermo Fisher Scientific, Madison, WI, USA) by microscope transmission method. 1D- and 2D-NMR spectra were obtained at 500 MHz, 600 MHz or 800 MHz for ^{1}H and 125 MHz, 150 MHz, or 200 MHz for ^{13}C, respectively, on INOVA 500 MHz, SYS 600 MHz or BRUKER 800 MHz spectrometers (Varian Associates Inc., *p*alo Alto, CA, USA) (Bruker BioSpin AG, Fällanden, Switzerland), with solvent peaks serving as references. ESI-MS data were measured with a Q-Trap LC/MS/MS (Turbo Ionspray source) spectrometer. HR-ESI-MS data were measured using a Q Exactive Focus LC-MS/MS spectrometer (Thermo Fisher Scientific, WI, USA), or a Micromass Autospec−Ultima ETOF spectrometer (Waters Corporation, Milford, USA). Column chromatography (CC) was *p*erformed with silica gel (200–300 mesh, Qingdao Marine Chemical Inc. Qingdao, China) and Sephadex LH-20 (Pharmacia Biotech AB, Uppsala, Sweden), Toyopearl HW-40C (Tosoh Corporation, Tokyo, Japan), and MCI gel (CHP20P, 75–150 μm) (Mitsubishi Chemical Corporation, Tokyo, Japan). HPLC separation was *p*erformed on an instrument with a LabAlliance prep pump (Teledyne SSI, *p*A, USA) and a UV6000LP *p*DA Detector (Thermo Fisher Scientific, MA, USA) on a silica, *p*FP, MGII C18 or AQ C18 column (250 × 10 mm, 5μm) (Daiso Chemical CO., LTD., Tokyo, Japan), Grace C18 column (250 × 10 mm,5 μm) (Grace Inc., Columbia, USA), YMC ph column (250 × 10 mm, 5 μm) (YMC Corporation, Kyoto, Japan), or an AD-H chiral column (250 × 10 mm, 5 μm) [Daicel Chiral Technologies (China) CO., LTD, Shanghai, China].

TLC was carried out with glass *p*recoated silica gel GF_{254} *p*lates (Qingdao Marine Chemical Inc.). Spots were visualized under UV light or by spraying with 5% H_2SO_4 in EtOH followed by heating.

2. Plant material

The steamed and dried rhizomes of *Gastrodia elata* were *p*urchased at the *p*lantation field Xiao Cao Ba, Yunnan *p*rovince, China, in December 2009. A voucher specimen (no. ID-S-2384) was deposited at the herbarium of the Department of Chemistry of Natural *p*roducts, Institute of Materia Medica. And the fresh *G. elata* rhizomes were collected at the same field in October 2022. plant identification was verified by Mr. Lin Ma (Institute of Materia Medica, Beijing 100050, China).

3. Extraction and isolation

For extraction and preliminary fractionation of the extract see Ref. 31−37 of manuscript. Fraction C2 (302 g) was subjected CC over silica gel (ethyl acetate-EtOH-H_2O, 16:2:1−2:2:1) to give subfractions C2-1−C2-5. Subfraction C2-1 (52.5 g) was chromatographed over silica gel, eluting with a gradient of increasing MeOH (0−100%) in $CHCl_3$, to yield C2-1-1−C2-1-6. Fraction C2-1-1 (9.5 g) was separated by CC over Sephadex LH-20 (50% MeOH in H_2O) to yield C2-1-1-1−C2-1-1-8, of which C2-1-1-3 (1.2 g) was further fractionated by CC over silica gel ($CHCl_3$-MeOH, 10:1) to give C2-1-1-3-1−C2-1-1-3-3. Separation of C2-1-1-3-2 (120 mg) by preparative silica gel TLC ($CHCl_3$-MeOH, 10:1) yielded C2-1-1-3-2-1 and C2-1-1-3-2-2, of which C2-1-1-3-2-1 (16 mg) was isolated by chiral HPLC (hexane-isopropanol, 1.65:1, AD-H column, 1.5 mL min^{-1}) to obtain **7** (4.5 mg, t_R = 36.2 min), **8** (3.5 mg, t_R = 40.5 min), and **11** (1.2 mg, t_R = 28.3 min). Separation of C2-1-1-3-2-2 (35 mg) by RP-HPLC (50.5% CH_3CN in H_2O, Ph column, 2.0 mL

min^{-1}) yielded **14** (7.5 mg, t_R = 18.5 min) and **10** (8.2 mg, t_R = 20.3 min). Fraction C2-1-1-4 (1.2 g) was separated by CC over silica gel ($CHCl_3$-MeOH, 20:1) to yield C2-1-1-4-1 and C2-1-1-4-2, of which C2-1-1-4-2 (200 mg) was further isolated by preparative silica gel TLC ($CHCl_3$-MeOH, 10:1) to obtain C2-1-1-4-2-1 and C2-1-1-4-2-2. Separation of C2-1-1-4-2-2 (21 mg) by RP-HPLC (63% MeOH in H_2O, Ph column, 2.0 mL min^{-1}) afforded **4** (5.2 mg, t_R = 37.8 min) and **5** (10 mg, t_R = 38.8 min). Fraction C2-1-1-5 (1.3 g) was separated by CC over silica gel ($CHCl_3$-MeOH, 10:1) to give C2-1-1-5-1−C2-1-1-5-4. Isolation of C2-1-1-5-2 (270 mg) by CC over HW-40C gel (MeOH) yielded C2-1-1-5-2-1 and C2-1-1-5-2-2, of which C2-1-1-5-2-2 (46 mg) was separated by preparative silica gel TLC ($CHCl_3$-MeOH, 10:1), followed by purification with RP-HPLC (60% MeOH in H_2O, Ph column, 2.0 mL min^{-1}) to obtain **1** (2.3 mg, t_R = 57.5 min) and **6** (5.3 mg, t_R = 63.1 min). Fraction C2-1-1-5-3 (380 mg) was isolated by preparative silica gel TLC ($CHCl_3$-MeOH, 15:1) to give C2-1-1-5-3-1−C2-1-1-5-3-3, of which C2-1-1-5-3-2 (23 mg) was separated by chiral HPLC (hexane-isopropanol, 2:1, AD-H column, 1.5 mL min^{-1}) to afford **3** (6.8 mg, t_R = 26.5 min) and **2** (9.5 mg, t_R = 31.6 min). Fraction C2-1-2 (7.2 g) was chromatographed over silica gel, eluting with a gradient increasing MeOH (0−100%) in $CHCl_3$, to yield C2-1-2-1−C2-1-2-5, of which C2-1-2-5 (1.5 g) was further fractionated by CC over Sephadex LH-20 (MeOH) to give C2-1-2-5-1−C2-1-2-5-3. Isolation of C2-1-2-5-2 (180 mg) by CC over silica gel ($CHCl_3$-MeOH, 10:1) yielded C2-1-2-5-2-1−C2-1-2-5-2-3, of which C2-1-2-5-2-2 (76 mg) was isolated by RP-HPLC (60% MeOH in H_2O, C_{18} column， 2.0 mL min^{-1}) to obtain **9** (8.1 mg, t_R = 81.2 min). Separation of C2-1-2-5-2-3 (42 mg) by RP-HPLC (65% MeOH in H_2O, Ph column, 2.0 mL min^{-1}), followed by chiral HPLC (hexane-isopropanol, 1:2, AD-H column, 1.3 mL min^{-1}), obtained **13** (2.5 mg, t_R =

91.8 min). Fractionation of C2-1-3 (7.2 g) by CC over HW-40C (MeOH) yielded C2-1-3-1−C2-1-3-3, of which C2-1-3-1 (25 mg) was further isolated by preparative silica gel TLC ($CHCl_3$-MeOH, 5:1), followed by RP-HPLC purification (70% MeOH in H_2O, C_{18} column, 1.5 mL min^{-1}) to afford **12** (1.8 mg, t_R = 31.5 min).

Chemical-physical properties and spectroscopic data of compounds 1−13

Gastrotetrabenzin A (**1**): white amorphous powder; UV (MeOH) λ_{max} (log ε) 208 (4.39), 277 (3.50) nm; IR ν_{max} 3329, 3017, 2925, 2703, 2614, 1890, 1612, 1598, 1512, 1476, 1441, 1380, 1234, 1173, 1142, 1106, 1076, 1004, 947, 912, 873, 826, 782 cm^{-1}; ^1H NMR (acetone-d_6, 600 MHz) and ^{13}C NMR (acetone-d_6, 150 MHz), see Table S1; (+)-ESIMS *m/z* 479 $[M + Na]^+$; (–)-ESIMS *m/z* 455 $[M - H]^-$, 491 $[M + Cl]^-$; (+)-HRESIMS *m/z* 479.1846 $[M + Na]^+$ (calcd. for $C_{29}H_{28}O_5Na$, 479.1829), 495.1599 $[M + K]^+$ (calcd. for $C_{29}H_{28}O_5K$, 495.1568).

Gastrotetrabenzin B (**2**): white amorphous powder; UV (MeOH) λ_{max} (log ε) 203 (4.37), 226 (3.92), 280 (3.56) nm; IR ν_{max} 3335, 3020, 2926, 2858, 2705, 2608, 1651, 1612, 1597, 1513, 1476, 1444, 1365, 1234, 1174, 1101, 1074, 1004, 947, 909, 825, 784 cm^{-1}; ^1H NMR (DMSO-d_6, 500 MHz) and ^{13}C NMR (DMSO-d_6, 125 MHz), see Table S1; (+)-ESIMS *m/z* 479 $[M + Na]^+$; (–)-ESIMS *m/z* 455 $[M - H]^-$, 491 $[M + Cl]^-$; (–)-HRESIMS *m/z* 455.1847 $[M - H]^-$ (calcd. for $C_{29}H_{27}O_5$, 455.1864).

Gastrotetrabenzin C (**3**): white amorphous powder; UV(MeOH) λ_{max} (log ε) 204 (4.12), 226 (3.59), 280 (3.18) nm; IR ν_{max} 3289, 3018, 2929, 2827, 2725, 2607, 2256, 2127, 2045, 1889, 1611, 1510, 1440, 1376, 1263, 1106, 1076, 1044, 1022, 1000, 950, 911, 881, 824, 780 cm^{-1}; ^1H NMR (DMSO-d_6, 600 MHz) and ^{13}C NMR (DMSO-d_6, 150MHz), see Table S1; (+)-ESIMS *m/z* 479 [M

+ Na]$^+$, 495 [M + K]$^+$; (–)-ESIMS *m/z* 455 [M − H]$^-$, 491 [M + Cl]$^-$; (+)-HRESIMS *m/z* 479.1831 [M + Na]$^+$ (calcd. for $C_{29}H_{28}O_5Na$, 479.1829).

Gastrotetrabenzin D (**4**): white amorphous powder; UV(MeOH) λ_{max} (log ε) 205 (4.13), 223 (3.55), 280 (3.32) nm; IR ν_{max} 3277, 3016, 2917, 2849, 1890, 1651, 1612, 1511, 1437, 1365, 1245, 1172, 1107, 1006, 948, 910, 822, 773 cm^{-1}; ^{1}H NMR (DMSO-d_6, 500 MHz) and ^{13}C NMR (acetone-d_6, 125 MHz), see Table S1; (–)-ESIMS *m/z* 411 [M − H]$^-$, 447 [M + Cl]$^-$; (–)-HRESIMS *m/z* 411.1604 [M − H]$^-$ (calcd. for $C_{27}H_{23}O_4$, 411.1602).

Gastrotetrabenzin E (**5**): white amorphous powder; UV(MeOH) λ_{max} (log ε) 207 (4.31), 223 (3.42), 281 (3.35) nm; IR ν_{max} 3354, 3019, 2922, 2851, 1890, 1674, 1611, 1511, 1439, 1365, 1233, 1173, 1107, 1002, 946, 912, 822, 779 cm^{-1}; ^{1}H NMR (DMSO-d_6, 500MHz) and ^{13}C NMR (acetone-d_6, 125 MHz), see Table S1; (+)-ESIMS *m/z* 435 [M + Na]$^+$, 451 [M + K]$^+$; (–)-ESIMS *m/z* 411 [M − H]$^-$, 447 [M + Cl]$^-$; (+)-HRESIMS *m/z* 435.1569 [M + Na]$^+$ (calcd. for $C_{27}H_{24}O_4Na$, 435.1567).

Gastrotetrabenzin F (**6**): white amorphous powder; UV(MeOH) λ_{max} (log ε) 208 (4.33), 274 (3.53) nm; IR ν_{max} 3333, 3018, 2918, 2693, 2612, 2496, 1612, 1597, 1513, 1476, 1445, 1366, 1233, 1172, 1134, 1101, 1003, 948, 827, 772, 730 cm^{-1}; ^{1}H NMR (DMSO-d_6, 500 MHz) and ^{13}C NMR (DMSO-d_6, 125 MHz), see Table S1; (–)-ESIMS *m/z* 411 [M − H]$^-$, 447 [M + Cl]$^-$; (–)-HRESIMS *m/z* 411.1582 [M − H]$^-$ (calcd. for $C_{27}H_{23}O_4$, 411.1602).

Table S1 NMR spectroscopic data of compounds **1−6** a).

	**1**		**2**		**3**		**4**		**5**		**6**	
no	δ_H	δ_C	δ_H	δ_C	δ_H	δ_C	δ_H	δ_C	δ_H	δ_C	δ_H	δ_C
1		129.0		126.7		128.3		132.0		132.0		132.0
2	6.83 d (2.0)	129.0	6.80 brs	129.8	6.86 d (1.8)	129.9	6.78 d (2.0)	131.3	6.80 d (2.0)	130.3	6.66 s	128.8
3		129.2		127.7		127.9		127.5		127.7		128.3
4		152.6		154.3		154.3		152.9		152.9		150.2
5		129.2	6.72 d (8.0)	114.5	6.74 d (8.4)	114.6	6.64 d (8.0)	114.7	6.65 d (8.5)	114.8		128.3
6	6.87 d (2.0)	129.0	6.90 brd (8.0)	129.4	6.92 dd (8.4, 1.8)	126.7	6.75 dd (8.5, 2.0)	126.8	6.74 dd (8.5, 2.0)	126.8	6.66 s	128.8
7	4.20 s	75.0	4.16 s	73.8	4.18 s	73.7						
1′		132.3		131.5		131.0		132.1		131.3		131.2
2′	6.97 d (2.0)	131.9	6.70 s	128.6	6.84 d (2.4)	123.6	6.85 d (2.0)	130.5	6.96 d (8.5)	129.4	6.95 d (8.0)	129.5
3′		129.0		128.6		127.5		127.7	6.63 d (8.5)	114.9	6.63 d (8.0)	114.9
4′		154.0		150.2		152.8		152.8		155.2		155.2
5′	6.74 d (8.0)	115.9		128.6	6.64 d (8.4)	114.7	6.65 d (7.5)	114.8	6.63 d (8.5)	114.9	6.63 d (8.0)	114.9
6′	6.86 dd (8.0, 2.0)	128.2	6.70 s	128.6	6.78 dd (8.4, 2.4)	126.9	6.77 dd (7.5, 2.0)	127.0	6.96 d (8.5)	129.4	6.95 d (8.0)	129.5
7′	3.87 s	35.8	3.62 s	34.2	3.67s	34.4	3.62 s	34.4	3.67 s	34.4	3.75 s	34.7
1″		133.0		131.2		131.3		131.4		131.3		131.2
2″	7.04 d (8.5)	130.6	6.94 d (7.5)	129.4	6.87 d (2.4)	123.5	6.96 d (8.5)	129.4	6.96 d (8.5)	129.4	6.95 d (8.0)	129.5
3″	6.70 d (8.5)	115.8	6.62 d (7.5)	114.9		127.4	6.63 d (8.5)	114.9	6.63 d (8.5)	114.9	6.63 d (8.0)	114.9
4″		156.3		155.2		152.8		155.3		155.2		155.2
5″	6.70 d (8.5)	115.8	6.62 d (7.5)	114.9	6.65 d (8.4)	114.7	6.63 d (8.5)	114.9	6.63 d (8.5)	114.9	6.63 d (8.0)	114.9
6″	7.04 d (8.5)	130.6	6.94 d (7.5)	129.4	6.76 dd (8.4, 2.4)	127.0	6.96 d (8.5)	129.4	6.96 d (8.5)	129.4	6.95 d (8.0)	129.5
7″	3.82 s	35.5	3.74 s	34.7	3.63 s	34.3	3.66 s	34.5	3.67 s	34.4	3.75 s	34.7
1‴		132.4		131.2		131.4		131.3		131.3		132.5
2‴	7.05 d (8.5)	130.6	6.94 d (7.5)	129.4	6.96 d (8.4)	129.4	6.91 d (8.5)	129.3	6.80 d (2.0)	130.3	6.88 d (8.5)	129.3
3‴	6.73 d (8.5)	115.8	6.62 d (7.5)	114.9	6.62 d (8.4)	114.8	6.64 d (8.5)	114.9		127.7	6.61 d (8.5)	115.0
4‴		156.5		155.2		155.2		155.2		152.9		155.2
5‴	6.73 d (8.5)	115.8	6.62 d (7.5)	114.9	6.62 d (8.4)	114.8	6.64 d (8.5)	114.9	6.65 d (8.5)	114.8	6.61 d (8.5)	115.0
6‴	7.05 d (8.5)	130.6	6.94 d (7.5)	129.4	6.96 d (8.4)	129.4	6.91 d (8.5)	129.3	6.74 dd (8.5, 2.0)	126.8	6.88 d (8.5)	129.3
7‴	3.91 s	35.7	3.74 s	34.7	3.67s	34.5	3.61 s	39.7	3.58 s	39.6	3.57	39.6
O*Me*	3.20 s	57.6	3.15 s	56.9	3.17 s	56.9						
4-O*H*	7.12 s		9.30 s		9.31 s		9.08 s		9.07 s		8.14 s	
4′-O*H*	8.04 s		8.11 s		9.04 s		9.09 s		9.08 s		9.12 s	
4″-O*H*	8.01 s		9.10 s		9.04 s		9.16 s		9.08 s		9.12 s	
4‴-O*H*	8.07 s		9.10 s		9.08 s		9.12 s		9.07 s		9.13 s	

a) Data were measured in acetone-d_6 for **1** at 500 MHz for ^{1}H and at 125 MHz for ^{13}C, and in DMSO-d_6 for **2**, **4**−**6** at 500 MHz for ^{1}H and at 125 MHz for ^{13}C, and **3** at 600 MHz for ^{1}H and at 150 MHz for ^{13}C, respectively. Proton coupling constants (*J*) in Hz are given in parentheses. The assignments were based on ^{1}H-^{1}H COSY, HSQC, and HMBC experiments.

Gastropentabenzin A (**7**): brown gum; UV (MeOH) λ_{max} (log ε) 205 (4.58), 226 (4.26), 282 (3.69); IR (Nujol) ν_{max} 3360, 3019, 2918, 2848, 1890, 1697, 1611, 1510, 1439, 1365, 1248, 1173, 1103, 1042, 912, 879, 820, 777 cm^{-1}; ^{1}H NMR (acetone-d_6, 600 MHz) and ^{13}C NMR (acetone-d_6, 150 MHz), see Table S2; (+)-ESIMS *m/z* 541 $[M + Na]^+$, 557 $[M + K]^+$, (–)-ESIMS *m/z* 517 $[M - H]^-$, 553 $[M + Cl]^-$; (+)-HRESIMS *m/z* 541.1979 $[M + Na]^+$ (calcd. for $C_{34}H_{30}O_5Na$, 541.1985).

Gastropentabenzin B (**8**): brown gum; UV (MeOH) λ_{max} (log ε) 205 (4.58), 225 (4.28), 282 (3.49); IR (Nujol) ν_{max} 3382, 3018, 2923, 2851, 2700, 1889, 1696, 1651, 1611, 1510, 1439, 1357, 1247, 1174, 1107, 1016, 912, 824, 779 cm^{-1}; ^{1}H NMR (acetone-d_6, 600 MHz) and ^{13}C NMR (acetone-d_6, 150 MHz), see Table S2; (+)-ESIMS *m/z* 541 $[M + Na]^+$, (–)-ESIMS *m/z* 517 $[M - H]^-$, 553 $[M + Cl]^-$; (+)-HRESIMS *m/z* 541.1984 $[M + Na]^+$ (calcd. for $C_{34}H_{30}O_5Na$, 541.1985).

Gastropentabenzin C (**9**): brown gum; UV (MeOH) λ_{max} (log ε) 205 (4.54), 225 (4.31), 281 (3.65); IR (Nujol) ν_{max} 3383, 3018, 2921, 2850, 1700, 1647, 1612, 1512, 1474, 1443, 1368, 1236, 1173, 1134, 1104, 1044, 911, 878, 823, 782 cm^{-1}; ^{1}H NMR (acetone-d_6, 600 MHz) and ^{13}C NMR (acetone-d_6, 150 MHz), see Table S2; (+)-ESIMS *m/z* 541 $[M + Na]^+$, 557 $[M + K]^+$, (–)-ESIMS *m/z* 517 $[M - H]^-$, 553 $[M + Cl]^-$; (+)-HRESIMS *m/z* 541.1986 $[M + Na]^+$ (calcd. for $C_{34}H_{30}O_5Na$, 541.1985).

Gastropentabenzin D (**10**): brown gum; UV (MeOH) λ_{max} (log ε) 205 (4.57), 225 (4.33), 282 (3.68); IR (Nujol) ν_{max} 3382, 3020, 2923, 2851, 1888, 1674, 1648, 1612, 1512, 1474, 1442, 1368, 1229, 1173, 1104, 1044, 1015, 943, 913, 879, 828, 778 cm^{-1}; ^{1}H NMR (acetone-d_6, 600 MHz) and ^{13}C NMR (acetone-d_6, 150 MHz), see Table S2; (+)-ESIMS *m/z* 519 $[M + H]^+$, 541 $[M + Na]^+$, 557 $[M + K]^+$, (–)-ESIMS *m/z* 517 $[M - H]^-$, 553 $[M + Cl]^-$; (+)-HRESIMS *m/z* 541.1987 $[M + Na]^+$ (calcd. for $C_{34}H_{30}O_5Na$, 541.1985).

Gastropentabenzin E (**11**): brown gum; UV (MeOH) λ_{max} (log ε) 204 (4.46), 225 (4.14), 282 (3.66); IR (Nujol) ν_{max} 3368, 3021, 2922, 2852, 1700, 1611, 1510, 1439, 1360, 1260, 1203, 1107, 1076, 932, 824, 778 cm^{-1}; ^{1}H NMR (acetone-d_6, 800 MHz) and ^{13}C NMR (acetone-d_6, 200 MHz), see Table S2; (+)-ESIMS *m/z* 585 $[M + Na]^{+}$, (−)-ESIMS *m/z* 561 $[M - H]^{-}$, 597 $[M + Cl]^{-}$; (+)-HRESIMS *m/z* 585.2241 $[M + Na]^{+}$ (calcd. for $C_{36}H_{34}O_{6}Na$, 585.2248).

Table S2 NMR spectroscopic data of compounds **7−11** in acetone-d_6 [a]

	**7**		**8**		**9**		**10**		**11**[c)]	
no	δ_H	δ_C	δ_H	δ_C	δ_H	δ_C	δ_H	δ_C	δ_H	δ_C
1		133.0		133.1		134.1		129.2		130.2
2	6.88 d (2.4)	131.6	6.86 d (2.4)	131.6	6.76 s	129.7	6.76 d (2.4)	129.7	6.95 d (1.6)	131.1
3		129.0		128.9		129.2		133.7		129.0
4		153.8		153.8		151.4		151.4		155.3
5	6.73 d (8.4)	115.7	6.69 d (8.4)	115.7	6.76 s	129.7		124.0	6.80 d (8.0)	115.6
6	6.81 dd (8.4,2.4)	127.9	6.77 dd (7.8 2.4)	127.9		129.2	6.73 d (2.4)	129.7	6.96 dd (8.0, 1.6)	127.6
7									4.22 s	74.9
1′		133.1		133.1		132.5		132.6		133.0
2′	6.95 d (2.4)	131.8	6.98 d (2.4)	131.9	7.02 d (8.4)	130.6	6.93 d (2.4)	131.8	6.99 d (1.6)	131.1
3′		128.7		128.8	6.71 d (8.4)	115.9		128.9		128.8
4′		153.7		153.8		156.4		153.9		153.8
5′	6.69 d (8.4)	115.6	6.72 d (8.4)	115.8	6.71 d (8.4)	115.8	6.70 d (7.8)	115.7	6.68 d (8.0)	115.5
6′	6.86 dd (8.4, 2.4)	128.0	6.85 dd (8.0, 2.4)	128.2	7.02 d (8.4)	130.6	6.82 dd (7.8, 2.4)	128.1	6.85 dd (8.0, 1.6)	128.0
7′	3.77 s	35.6	3.77 s	35.6	3.86 s	35.9	3.82 s	35.9	3.76 s	35.4
1″		133.1		133.8		132.5		132.5		133.0
2″	7.00 d (2.4)	131.9	7.05 d (8.4)	130.6	7.02 d (8.4)	130.6	7.01 d (9.0)	130.5	6.95 d (1.6)	131.8
3″		128.7	6.69 d (7.5)	115.8	6.71 d (8.4)	115.9	6.70 d (8.4)	115.9		128.9
4″		153.7		156.2		156.4		156.3		153.8
5″	6.69 d (7.8)	115.6	6.69 d (7.5)	115.8	6.71 d (8.4)	115.8	6.70 d (8.4)	115.9	6.69 d (8.0)	115.5
6″	6.84 dd (7.8, 2.4)	128.1	7.05 d (8.4)	130.6	7.02 d (8.4)	130.6	7.01 d (9.0)	130.5	6.86 dd (8.0, 1.6)	128.1
7″	3.77 s	35.6	3.81 s	35.5	3.86 s	35.9	3.80 s	35.9	3.80 s	35.6
1‴		133.7		133.1		133.8		129.2		133.0
2‴	6.97 d (8.4)	130.4	6.90 d (2.4)	131.7	6.87 d (2.4)	131.6	6.94 (8.4)	130.4	6.95 d (1.6)	131.8
3‴	6.71 d (8.4)	115.9		128.9		128.9	6.68 d (8.4)	115.8		129.0
4‴		156.3		153.8		153.8		156.3		153.8
5‴	6.71 d (8.4)	115.9	6.71 d (8.0)	115.7	6.70 d (8.4)	115.7	6.68 d (8.4)	115.8	6.70 d (8.0)	115.5
6‴	6.97 d (8.4)	130.4	6.78 dd (8.4, 2.4)	128.0	6.78 dd (8.4, 2.4)	128.0	6.94 d (8.4)	130.4	6.83 dd (8.0, 1.6)	128.1
7‴	3.70 s	40.8	3.66 s	40.9	3.64 s	41.0	3.66 s	40.9	3.76 s	35.6
1⁗		133.7		133.8		133.0		133.0		133.0
2⁗	7.06 d (8.4)	130.5	7.05 d (8.4)	130.6	7.04 d (8.4)	130.6	7.03 d (8.4)	130.6	7.05 d (8.0)	130.5
3⁗	6.70 d (8.4)	115.7	6.69 d (7.5)	115.8	6.71 d (8.4)	115.8	6.71 d (7.8)	115.9	6.71 d (8.0)	115.8
4⁗		156.2		156.3		156.3		156.3		156.4
5⁗	6.70 d (8.4)	115.7	6.69 d (7.5)	115.8	6.71 d (8.4)	115.8	6.71 d (7.8)	115.9	6.71 d (8.0)	115.8
6⁗	7.06 d (8.4)	130.5	7.05 d (8.4)	130.6	7.04 d (8.4)	130.6	7.03 d (8.4)	130.6	7.05 d (8.0)	130.5
7⁗	3.82 s	35.5	3.81 s	35.5	3.80 s	35.6	3.85 s	35.6	3.79 s	35.6
4-O*H*	7.95 s		7.99 s		6.90 s		6.90 s		8.44 s	
4′-O*H*	7.98 s		8.03 s		8.01~8.06[b)]		8.05		8.13 s	
4″-O*H*	7.99 s		8.03 s		8.01~8.06[b)]		8.05		8.17 s	
4‴-O*H*	8.06 s		7.99 s		8.01~8.06[b)]		8.08		8.16 s	
4⁗-O*H*	8.01 s		8.02 s		8.01~8.06[b)]		8.08		8.21 s	

[a] Data were measured in acetone-d_6 at 600 MHz for ^{1}H and at 150 MHz for ^{13}C for **7−10**, and at 800 MHz for ^{1}H and at 200 MHz for ^{13}C for **11**, respectively. Proton coupling constants (J) in Hz are given in parentheses. The assignments were based on ^{1}H-^{1}H COSY, HSQC, and HMBC experiments. b) Unresolved broad signals. c) Data for hydroxymethyl (δ_H 3.20 s; δ_C 57.5) of **11**.

Gastrohexabenzin A (**12**): brown gum; UV (MeOH) λ_{max} (log ε) 205 (4.65), 225 (4.38), 282 (3.75); IR ν_{max} 3381, 3017, 2917, 2850, 1890, 1675, 1611, 1511, 1477, 1440, 1357, 1224, 1172, 1101, 1015, 911, 821, 777 cm^{-1}; ^{1}H NMR (acetone-d_6, 600 MHz) and ^{13}C NMR (acetone-d_6, 150

MHz), see Table S3; (+)-ESIMS *m/z* 647 $[M + Na]^+$, (−)-ESIMS *m/z* 623 $[M - H]^-$, 659 $[M + Cl]^-$; (+)-HRESIMS *m/z* 647.2384 $[M + Na]^+$ (calcd. for $C_{41}H_{36}O_6Na$, 647.2404).

Gastrohexabenzin B (**13**): brown gum; UV (MeOH) λ_{max} (log ε) 204 (4.37), 225 (4.12), 282 (3.48); IR ν_{max} 3362, 3029, 2925, 2854, 1886, 1678, 1612, 1597, 1511, 1476, 1441, 1361, 1207, 1143, 1107, 983, 833, 778, 726 cm^{-1}; ^1H NMR (acetone-d_6, 600 MHz) and ^{13}C NMR (acetone-d_6, 150 MHz), see Table S3; (+)-ESIMS *m/z* 647 $[M + Na]^+$, 663 $[M + K]^+$, (−)-ESIMS *m/z* 623 $[M - H]^-$, 659 $[M + Cl]^-$; (−)-HRESIMS *m/z* 623.2433 $[M - H]^-$ (calcd. for $C_{41}H_{35}O_6$, 623.2439).

Table S3 NMR spectroscopic data for compounds **12** and **13** in acetone-d_6 [a]

	**12**		**13**			**12**		**13**	
no.	δ_H	δ_C	δ_H	δ_C	no.	δ_H	δ_C	δ_H	δ_C
1		133.0		133.7	5‴	6.70 d (8.4)	115.7	6.74 d (8.4)	115.9
2	6.89 d (1.8)	131.7	6.77 d (2.4)	129.7	6‴	6.96 d (8.4)	130.4	6.95 d (8.4)	130.3
3		128.9		129.2	7‴	3.69 s	40.8	3.67 s	41.0
4		153.9		153.9	1⁗		132.5		132.4
5	6.73 d (7.8)	115.9		129.1	2⁗	7.02 d (8.4)	130.5	7.06 d (8.4)	130.5[b]
6	6.80 dd (7.8, 1.8)	128.0	6.74 d (2.4)	129.7	3⁗	6.70 d (8.4)	115.8	6.74 d (8.4)	115.9
1′		133.7		133.1	4⁗		156.3		156.2
2′	6.93 d (1.8)	131.7	7.00 d (2.4)	132.0	5⁗	6.70 d (8.4)	115.8	6.74 d (8.4)	115.9
3′		128.9		128.9	6⁗	7.02 d (8.4)	130.5	7.06 d (8.4)	130.5[b]
4′		153.7		153.8	7⁗	3.85 s	35.9	3.77 s	35.9
5′	6.71 d (7.8)	115.6	6.71 d (8.4)	115.7	1⁗′		132.5		133.1
6′	6.83 dd (7.8, 1.8)	128.0	6.86 dd (8.4, 2.4)	128.2	2⁗′	7.02 d (8.4)	130.5	7.02 d (8.4)	130.6[b]
7′	3.76 s	35.6	3.82 s	35.6	3⁗′	6.70 d (8.4)	115.8	6.72 d (8.4)	115.9
1″		133.3		133.1	4⁗′		156.3		156.4
2"	6.85 s	130.0	6.92 d (1.8)	132.6	5⁗′	6.70 d (8.4)	115.8	6.72 d (8.4)	115.9
3"		128.9		128.9	6⁗′	7.02 d (8.4)	130.5	7.02 d (8.4)	130.6[b]
4″		151.3		151.4	7⁗′	3.85 s	35.9	3.86 s	35.9
5"		128.9	6.71 d (8.4)	115.8	4-O*H*	8.04 s		8.02~8.07 s	
6"	6.85 s	130.0	6.81 dd (8.4, 1.8)	128.2	4′-O*H*	7.99 s		8.02~8.07 s	
7"	3.75 s	35.5	3.82 s	35.6	4″-O*H*	6.89 s		6.89 s	
1‴		133.7		134.0	4‴-O*H*	8.09 s		8.02~8.07 s	
2‴	6.96 d (8.4)	130.4	6.95 d (8.4)	130.3	4⁗-O*H*	8.10 s		8.02~8.07 s	
3‴	6.70 d (8.4)	115.7	6.74 d (8.4)	115.9	4⁗′-OH	8.09 s		8.02~8.07 s	
4‴		156.2		156.3					

[a] Data were measured at 600 MHz for ^1H and at 150 MHz for ^{13}C. Proton coupling constants (J) in Hz are given in parentheses. The assignments were based on DEPT, ^1H-^1H COSY, HSQC, and HMBC experiments.

4. Isolation of the refluxed aqueous solution of *p*-hydroxybenzyl alcohol

For preliminary fractionation of the refluxed aqueous solution see Ref. 37 of manuscript. Fr.1-6 (20 mg) was separated by PTLC (CH_2Cl_2-MeOH, 15:1) to yield **29** (7.0 mg, $R_f = 0.50$). Isolation

of Fr.1-9 (71 mg) by PTLC (hexane-EtOAc-MeOH, 6:2.5:1) afforded Fr.1-9-1−Fr.1-9-4, of which Fr.1-9-2 (29.5 mg) was further purified by HPLC (MGII C_{18} column, 59% MeOH in H_2O, 2.0 mL min^{-1}) to give **4** (9.0 mg, t_R = 86.0 min), **5** (8.0 mg, t_R = 97.0 min), and **6** (10.0 mg, t_R = 92.0 min). Fr.1-10 (99 mg) was isolated by PTLC (hexane-EtOAc-MeOH, 6:2.5:1) to obtain subfractions Fr.1-10-1−Fr.1-10-4. Separation of Fr.1-10-2 (50 mg) by HPLC (PFP column, 56% MeCN in H_2O, 2.0 mL min^{-1}) afforded Fr.1-10-2-1− Fr.1-10-2-3 and **9** (5.0 mg, t_R = 30.0 min), of which Fr.1-10-2-2 and Fr.1-10-2-3 were separately isolated by HPLC (Silica column, hexane-EtOH 85:15, 2.0 mL min^{-1}) to give **7** (14.0 mg, t_R = 36.8 min) and **14** (10.0 mg, t_R = 33.5 min) from the former, and **8** (10.0 mg, t_R = 30.4 min) and **10** (15.0 mg, t_R = 40.9 min) from the later. PTLC separation (hexane-EtOAc-MeOH, 6:2.5:1) of Fr.1-11 (163 mg) obtained Fr.1-11-1−Fr.1-11-5, and further isolation of Fr.1-11-4 (5.0 mg) by HPLC (MGII C_{18} column, 53% MeOH in H_2O, 2.0 mL min^{-1}) gave **15** (1.0 mg, t_R = 42 min). Separated isolation of Fr.1-12 (20 mg) and Fr.1-13 (201 mg) by PTLC (hexane-EtOAc-MeOH, 6:2.5:1) followed by HPLC (MGII C_{18} column, 2.0 mL min^{-1}) afforded **28** (1.0 mg, t_R = 28.9 min) from Fr.1-12, and **16** (2.0 mg, t_R = 58 min) and **17** (2.0 mg, t_R = 50 min) from Fr.1-13. Fr.1-14 (310 mg) was isolated by HPLC (Silica column, hexane-EtOH 9:1, 2.5 mL min^{-1}) to obtain **4** (109 mg, t_R = 9.2 min), **5** (50 mg, t_R = 17.5 min) and **6** (80 mg, t_R = 16.5 min) once again. Fr.1-15 (61 mg) was chromatographed by PTLC (petroleum ether-acetone 6:4) to give Fr.1-15-1−Fr.1-15-3. Fr.1-15-1 was further separated by HPLC (Silica column, hexane-EtOH 88:12, 3.0 mL min^{-1}) to obtain Fr.1-15-1-1−Fr.1-15-1-3, of which Fr.1-15-1-1 (5.0 mg) was chromatographed by HPLC (MGII C_{18} column, 70% MeOH in H_2O, 2.5 mL min^{-1}) to afford **19** (2.0 mg, t_R = 38.0 min). PTLC separation of Fr.1-16 (230 mg) with developing solvent systems of CH_2Cl_2-MeOH (10:1) and petroleum ether-acetone (6:4) in turn gave Fr.1-16-1−Fr.1-16-3. Fr.1-16-1 (20.0 mg)

was isolated by HPLC (Silica column, hexane-EtOH 88:12, 3.0 mL min^{-1}) to yield Fr.1-16-1-1 (6.0 mg) and Fr.1-16-1-2 (7.0 mg), which was separately purified by HPLC (MGII C_{18} column, 70% MeOH in H_2O, 2.5 mL min^{-1}) to afford **18** (4.0 mg, t_R = 48.0 min) and **20** (1.0 mg, t_R = 26.0 min), respectively. Fr.1-16-2 (120 mg) was chromatographed by CC over silica gel eluted with CH_2Cl_2-MeOH (10:1) to afford Fr.1-16-2-1 and Fr.1-16-2-2. Fr.1-16-2-1 (75 mg) was isolated by CC over HW-40C (CH_2Cl_2-MeOH 1:1) to yield Fr.1-16-2-1-1− Fr.1-16-2-1-5. Separation of Fr.1-16-2-1-2 (40 mg) by HPLC (Silica column, hexane-EtOH 88:12, 2.0 mL min^{-1}) yielded Fr.1-16-2-1-2-1−Fr.1-16-2-1-2-3, which were separately isolated by HPLC (MGII C_{18} column, 70% MeOH in H_2O, 2.5 mL min^{-1}) to obtain **23** (2.0 mg, t_R = 56.0 min), **24** (3.0 mg, t_R = 51.5 min) and **25** (3.0 mg, t_R = 32.0 min) from Fr.1-16-2-1-2-1; **12** (2.0 mg, t_R = 43.5 min), **13** (6.0 mg, t_R = 54.0 min), and **26** (5.0 mg, t_R = 48.5 min), from Fr.1-16-2-1-2-2; and **21** (1.0 mg, t_R = 20.5 min) and **22** (1.0 mg, t_R = 23.0 min) from Fr.1-16-2-1-2-3. Isolation of Fr.1-17 (235 mg) by CC over silica gel (petroleum ether-acetone, 7:3 and 6:4) afforded Fr.1-17-1−Fr.1-17-20. Fr.1-17-5 (20 mg) was isolated by HPLC (MGII C_{18} column, 70% MeOH in H_2O, 2.5 mL min^{-1}) to obtain Fr.1-17-5-1−Fr.1-17-5-3, then Fr.1-17-5-2 (5 mg) was further purified by HPLC (MGII C_{18} column, 70% MeOH in H_2O, 2.5 mL min^{-1}) to give **27** (3.0 mg, t_R = 42.4 min).

Chemical-physical properties and spectroscopic data of compounds 15–28

4-Hydroxy-3-{4-hydroxy-3-[4-hydroxy-3-(4-hydroxybenzl)benzyl]benzyl}benzyl alcohol (**15**): white amorphous powder; UV (MeOH) λ_{max} (log ε) 203 (4.52), 225 (4.16), 282 (3.54); IR ν_{max} 3374, 3019, 2920, 2707, 2609, 1884, 1695, 1611, 1510, 1439, 1364, 1255, 1106, 999, 913, 823, 778 cm^{-1}; ^{1}H NMR (acetone-d_6, 600 MHz) and ^{13}C NMR (acetone-d_6, 150 MHz) data, see Table S4; (+)-HRESIMS: m/z 465.1673 [M + Na]$^+$ (calcd. for $C_{28}H_{26}O_5Na$, 465.1673).

Table S4 NMR spectroscopic data of compounds **15–17** in acetone-d_6 [a]

	15		16		17	
no	δ_H	δ_C	δ_H	δ_C	δ_H	δ_C
1		134.3		130.6		130.6
2	6.99 d (1.8)	130.1	7.01 d (2.4)	131.2	7.00 d (2.5)	131.1
3		128.9		129.0		129.1
4		154.7		155.2		155.1
5	6.79 d (8.4)	115.6	6.81 d (7.8)	115.6	6.80 d (9.0)	115.7
6	7.00 dd (8.4, 1.8)	126.6	6.96 dd (7.8, 2.4)	127.8	7.00 dd (9.0, 2.5)	127.6
7	4.44 d (6.0)	64.7	4.30 s	72.0	4.33 s	72.1
1′		133.1		132.9		132.9
2′	6.97 d (1.8)	131.9	7.07 d (8.4)	130.6	7.01 d (2.5)	132.0
3′		128.8 [d)]	6.72 d (8.4)	115.9		128.8
4′		153.8		156.4		153.8
5′	6.71 d (8.4)	115.7	6.72 d (8.4)	115.9	6.74 d (8.0)	115.5
6′	6.86 dd (8.4, 1.8)	128.2 [e)]	7.07 d (8.4)	130.6	6.90 dd (8.0, 2.5)	128.3
7′	3.80 s [b)]	35.6	3.85 s	35.4	3.83 s	35.4
1″		133.1		130.6		133.1
2″	6.99 d (2.4)	131.9	7.01 d (2.4)	131.2	7.06 d (8.5)	130.5
3″		128.9 [d)]		129.0	6.69 d (8.5)	115.8
4″		153.8		155.2		156.2
5″	6.71 d (8.4)	115.7	6.81 d (7.8)	115.6	6.69 d (8.5)	115.8
6″	6.86 dd (8.4, 2.4)	128.1 [e)]	6.96 dd (7.8, 2.4)	127.8	7.06 d (8.5)	130.5
7″	3.81 s [b)]	35.6	4.30 s	72.0	3.83 s	35.5
1‴		133.0		132.9		130.6
2‴	7.05 d (8.4)	130.6	7.07 d (8.4)	130.6	7.14 d (8.5)	130.2
3‴	6.70 d (8.4)	115.8	6.72 d (8.4)	115.9	6.79 d (8.5)	115.7
4‴		156.3		156.4		157.7
5‴	6.70 d (8.4)	115.8	6.72 d (8.4)	115.9	6.79 d (8.5)	115.7
6‴	7.05 d (8.4)	130.6	7.07 d (8.4)	130.6	7.14 d (8.5)	130.2
7‴	3.78 s	35.5	3.85 s	35.4	4.35 s	71.9
7-OH	3.86 t (6.0)					
4-OH	8.12 s		8.16 s		8.03 s	
4′-OH	7.96 s [c)]		8.38 s		8.04 s	
4″-OH	7.97 s [c)]		8.16 s		8.25 s	
4‴-OH	8.02 s		8.38 s		8.29 s	

[a] Data were measured at 600 MHz for ^{1}H and at 150 MHz for ^{13}C for **15** and **16**, and at 500 MHz for ^{1}H and at 125 MHz for ^{13}C for **17**. Proton coupling constants (J) in Hz are given in parentheses. The assignments were based on ^{1}H-^{1}H COSY, HSQC, and HMBC experiments. [b], [c], [d], [e] The assignments with the same symbol in the same column may be exchangeable.

Di-[4-hydroxy-3-(4-hydroxybenzyl)benzyl]ether (**16**): white amorphous powder; UV (MeOH) λ_{max} (log ε) 203 (4.54), 226 (4.15), 280 (3.52); IR ν_{max} 3336, 3021, 2922, 2865, 2707, 2605, 1890, 1696, 1612, 1512, 1442, 1362, 1236, 1173, 1154, 1109, 1046, 945, 913, 894, 824, 780 cm^{-1}; ^{1}H NMR (acetone-d_6, 600 MHz) and ^{13}C NMR (acetone-d_6, 150 MHz) data, see Table S4; (+)-HRESIMS: m/z 465.1672 [M + Na]$^+$ (calcd. for $C_{28}H_{26}O_5Na$, 465.1673).

4-Hydroxy-3-[4-hydroxy-3-(4-hydroxybenzyl)benzyl]benzyl 4-hydroxybenzyl ether (**17**): white amorphous powder; UV (MeOH) λ_{max} (log ε) 203 (4.42), 226 (4.08), 281 (3.41); IR ν_{max} 3354, 3021, 2921, 2865, 2707, 2605, 1889, 1697, 1612, 1512, 1441, 1362, 1242, 1173, 1107, 1046, 913, 826, 780 cm^{-1}; ^{1}H NMR (acetone-d_6, 600 MHz) and ^{13}C NMR (acetone-d_6, 150 MHz) data, see Table S4; (+)-HRESIMS: m/z 441.1697 [M – H]$^-$ (calcd. for $C_{28}H_{25}O_5$, 441.1708).

Table S5 NMR Spectroscopic Data of Compounds **18–20** in acetone-d_6 [a]

	**18**		**19**		**20**	
no	δ_H	δ_C	δ_H	δ_C	δ_H	δ_C
1		134.0		133.5		134.1[g]
2	6.94 d (2.4)	129.7	7.11 d (2.4)	132.0	7.00 d (2.4)	131.7
3		128.1		127.8		127.9[e]
4		151.2		153.2		153.4[f]
5		127.9	6.71 d (8.4)	115.8	6.72 d (8.4)	115.7
6	6.76 d (2.4)	129.8	6.88 dd (8.4, 2.4)	128.4	6.69 dd (8.4,2.4)	128.2
1′		129.7		128.9		128.0[e]
2′		152.2		153.4		153.3[f]
3′	6.77 d (8.4)	115.8	6.73 d (8.4)	116.1[b)]	6.73 d (8.4)[d]	116.0[h]
4′	6.84 dd (8.4, 2.4)	128.4	6.82 dd (8.4, 2.4)	128.2	6.82 dd (8.4,2.4)	128.2
5′		135.0		134.2		133.7[i]
6′	7.08 d (2.4)	131.8	7.04 d (2.4)	131.8	7.02 d (2.4)	131.7
7′	3.85 s	31.3	3.86 s	30.8	3.84 s	30.6
1″		133.5		133.8[c)]		133.6[i]
2″	6.98 d (8.4)	130.5	6.97 d (8.4)	130.5	6.97 d (8.4)	130.4
3″	6.73 d (8.4)	115.9	6.72 d (8.4)	115.9	6.71 d (8.4)	115.9
4″		156.4		156.4		156.3
5″	6.73 d (8.4)	115.9	6.72 d (8.4)	115.9	6.71 d (8.4)	115.9
6″	6.98 d (8.4)	130.5	6.97 d (8.4)	130.5	6.97 d (8.4)	130.4
7″	3.72 s	40.8	3.71s	40.9	3.70 s	40.8
1‴		132.9		128.1		134.2[g]
2‴	7.02 d (9.0)	130.6		153.9	6.91 d (2.4)	131.7
3‴	6.68 d (8.4)	115.7	6.73 d (8.4)	115.9[b)]		128.9
4‴		156.2	6.81 dd (8.4, 2.4)	128.0		153.8
5‴	6.68 d (8.4)	115.7		133.7[c)]	6.72 d (8.4)[d]	115.9[h]
6‴	7.02 d (9.0)	130.6	6.90 d (8.4)	131.7	6.78 dd (8.4, 2.4)	128.0
7‴	3.81 s	35.9	3.79 s	35.5	3.66 s	40.9
1⁗		133.7		133.7[c)]		133.0
2⁗	6.97 d (9.0)	130.4	6.97 d (8.4)	130.5	7.05 d (8.4)	130.5
3⁗	6.72 d (8.4)	115.9	6.72 d (8.4)	115.9	6.70 d (8.4)	115.7
4⁗		156.3		156.4		156.2
5⁗	6.72 d (8.4)	115.9	6.72 d (8.4)	115.9	6.70 d (8.4)	115.7
6⁗	6.97 d (9.0)	130.4	6.97 d (8.4)	130.5	7.05 d (8.4)	130.5
7⁗	3.69 s	40.9	3.71 s	40.9	3.81 s	35.5
4-OH	7.72 s		8.01~8.14 s		8.36 s	
2′-OH	9.08 s		8.01~8.14 s		8.36 s	
4″-OH	8.11 s		8.01~8.14 s		8.08 s	
2‴-OH			8.01~8.14 s			
4‴-OH	8.08 s				8.05 s	
4⁗-OH	8.03 s		8.01~8.14 s		8.03 s	

[a] NMR data were measured at 600 MHz for ^{1}H and at 150 MHz at ^{13}C. Proton coupling constants (J) in Hz are given in parentheses. Assignments were based on DEPT, ^{1}H-^{1}H gCOSY, gHSQC, and gHMBC experiments. [b], [c], [d], [e], [f], [g], [h], [i] The assignments with the same symbol in the same column may be exchangeable.

1,5-Di-(4-hydroxybenzyl)-3-[2-hydroxy-5-(4-hydroxybenzyl)benzyl]phenol (**18**): yellowish gum; UV (MeOH) λ_{max} (log ε) 203 (4.68), 225 (4.41), 280 (3.76); IR ν_{max} 3349, 3021, 2921, 2844, 2704, 2607, 1888, 1694, 1611, 1599, 1511, 1477, 1443, 1365, 1224, 1173, 1139, 1101, 1015, 911, 890, 833, 773, 728 cm^{-1}; ^{1}H NMR (acetone-d_6, 600 MHz) and ^{13}C NMR (acetone-d_6, 150 MHz) data, see Table S5; (+)-HRESIMS: m/z 541.1983 [M + Na]$^+$ (calcd. for $C_{34}H_{30}O_5Na$, 541.1986).

1,3-Di-[2-hydroxy-5-(4-hydroxybenzyl)benzyl]phenol (**19**): yellowish gum; UV (MeOH) λ_{max} (log ε) 203 (4.92), 225 (4.54), 281 (4.00); IR ν_{max} 3328, 3019, 2917, 2842, 2706, 2607, 1889, 1672,

1611, 1509, 1439, 1366, 1234, 1173, 1110, 1015, 914, 818, 774, 725 cm^{-1}; ^{1}H NMR (acetone-d_6, 600 MHz) and ^{13}C NMR (acetone-d_6, 150 MHz) data, see Table S5; (+)-HRESIMS: *m*/*z* 541.1986 $[M + Na]^+$ (calcd. for $C_{34}H_{30}O_5Na$, 541.1986).

1-[4-Hydroxy-5-(4-hydroxybenzyl)benzyl]-3-[2-hydroxy-5-(4-hydroxybenzyl)benzyl]phenol (**20**): yellowish gum; UV (MeOH) λ_{max} (log ε) 203 (4.92), 224 (4.56), 282 (3.98); IR ν_{max} 3314, 3108, 2975, 2918, 2708, 2612, 1692, 1611, 1598, 1510, 1440, 1367, 1233, 1173, 1110, 1044, 913, 879, 818, 779, 725 cm^{-1}; ^{1}H NMR (acetone-d_6, 600 MHz) and ^{13}C NMR (acetone-d_6, 150 MHz) data, see Table S5; (+)-HRESIMS: *m*/*z* 541.1985 $[M + Na]^+$ (calcd. for $C_{34}H_{30}O_5Na$, 541.1986).

1-(4-Hydroxybenzyl)-4-{4-hydroxy-3-{4-hydroxy-3-[4-hydroxy-3-(4-hydroxybenzyl)benzyl] benzyl}benzylphenol (**21**): yellowish gum; UV (MeOH) λ_{max} (log ε) 204 (4.91), 224 (4.51), 282 (3.95); IR ν_{max} 3357, 3019, 2919, 2851, 1692, 1654, 1611, 1509, 1437, 1362, 1249, 1174, 1103, 1015, 913, 820, 776 cm^{-1}; ^{1}H NMR (acetone-d_6, 600 MHz) and ^{13}C NMR (acetone-d_6, 150 MHz) data, see Table S6; (+)-HRESIMS: *m*/*z* 647.2399 $[M + Na]^+$ (calcd. for $C_{41}H_{36}O_6Na$, 647.2404).

1-[4-Hydroxy-3-(4-hydroxybenzyl)benzyl]-3-{4-hydroxy-3-[4-hydroxy-3-(4-hydroxybenzyl)] benzyl}benzylphenol (**22**): yellowish gum; UV (MeOH) λ_{max} (log ε) 204 (4.94), 225 (4.57), 282 (3.98); IR ν_{max} 3366, 3019, 2918, 2849, 2706, 2605, 1889, 1697, 1611, 1510, 1438, 1366, 1239, 1174, 1107, 1043, 1015, 913, 880, 822, 779, 704 cm^{-1}; ^{1}H NMR (acetone-d_6, 600 MHz) and ^{13}C NMR (acetone-d_6, 150 MHz) data, see Table S6; (+)-HRESIMS: *m*/*z* 647.2403 $[M + Na]^+$ (calcd. for $C_{41}H_{36}O_6Na$, 647.2404).

1-{4-Hydroxy-3-[4-hydroxy-3-(4-hydroxybenzyl)]benzyl}benzyl-3,5-di-(4-hydroxybenzyl)] benzyl}phenol (**23**): yellowish gum; UV (MeOH) λ_{max} (log ε) 203 (5.01), 225 (4.71), 280 (4.05); IR ν_{max} 3352, 3020, 2974, 2617, 2845, 2708, 2610, 1890, 1696, 1650, 1612, 1600, 1511, 1476, 1441,

1367, 1237, 1174, 1104, 1044, 1015, 983, 942, 911, 879, 823, 780, 730 cm^{-1}; ^{1}H NMR (acetone-d_6, 600 MHz) and ^{13}C NMR (acetone-d_6, 150 MHz) data, see Table S6; (+)-HRESIMS: m/z 647.2401 $[M + Na]^+$ (calcd. for $C_{41}H_{36}O_6Na$, 647.2404).

Table S6 NMR Spectroscopic Data for Compounds **21–24** in acetone-d_6 [a]

	21		22		23		24	
no.	δ_H	δ_C	δ_H	δ_C	δ_H	δ_C	δ_H	δ_C
1		133.7		133.0		134.1		133.3
2	6.87 d (2.4)	131.6	6.85 d (2.4)	131.6	6.76 s	129.7	6.84 s	130.0
3		129.0		128.9		129.2		129.1
4		153.8		153.8		151.4		151.3
5	6.70 d (8.4)	115.6	6.69 d (8.4)	115.6		129.2		129.1
6	6.79 dd (8.4, 2.4)	127.9	6.76 dd	127.9	6.76 s	129.7	6.84 s	130.0
1′		133.0		133.7		132.5		132.5
2′	6.96 d (2.4)	131.8	6.99 d (1.8)	131.9	7.01 d (8.4)	130.6	7.01 d (8.4)	130.6
3′		128.7		128.7	6.71 d (8.4)	115.9	6.70 d (8.4)	115.9
4′		153.7		153.7		156.4		156.4
5′	6.69 d (8.4)	115.7	6.68 d (8.4)	115.7	6.71 d (8.4)	115.9	6.70 d (8.4)	115.9
6′	6.85 dd (8.4, 2.4)	128.1	6.85 dd	128.1	7.01 d (8.4)	130.6	7.01 d (8.4)	130.6
7′	3.77 s	35.6	3.75 s	35.6	3.85 s	35.9	3.85 s	35.9
1″		133.0		133.8		132.5		132.5
2″	6.99 d (2.4)	131.9	6.95 d (2.4)	131.8	7.01 d (8.4)	130.6	7.01 d (8.4)	130.6
3″		128.7		128.6	6.71 d (8.4)	115.9	6.70 d (8.4)	115.9
4″		153.7		153.7		156.4		156.4
5″	6.69 d (8.4)	115.6	6.71 d (8.4)	115.6	6.71 d (8.4)	115.9	6.70 d (8.4)	115.9
6″	6.85 dd (8.4, 2.4)	128.0	6.77 d (8.4,2.4)	128.0	7.01 d (8.4)	130.6	7.01 d (8.4)	130.6
7″	3.77 s	35.6	3.77 s	35.6	3.85 s	35.9	3.85 s	35.9
1‴		133.0		133.0		133.7		128.9
2‴	6.95 d (8.4)	130.4	6.89 d (2.4)	131.6	6.84 d (2.4)	131.5		153.9
3‴	6.71 d (8.4)	115.7		128.9		128.9	6.68 d (8.4)	115.8
4‴		156.2		153.8		153.8	6.75 dd (8.4,2.4)	127.8
5‴	6.71 d (8.4)	115.7	6.68 d (8.4)	115.6	6.71 d (8.4)	115.7[b]		133.7[c]
6‴	6.95 d (8.4)	130.4	6.83 dd	128.0	6.84 dd (8.4,2.4)	128.1	6.83 d (2.4)	131.7
7‴	3.68 s	40.8	3.65 s	40.9	3.63 s	41.0	3.74 s	35.5
1⁗		133.7		133.1		133.1		133.8[c]
2⁗	6.94 d (2.4)	130.5	7.04 d (8.4)	130.5	6.99 d (2.4)	132.0	6.90 d (2.4)	131.5
3⁗		115.9	6.69 d (8.4)	115.7		128.7		129.1
4⁗		156.3		156.2		153.8		153.8
5⁗	6.68 d (8.4)	115.9	6.69 d (8.4)	115.7	6.68 d (8.4)	115.8[b]	6.73 d (8.4)	115.7
6⁗	6.83 dd (8.4, 2.4)	130.5	7.04 d (8.4)	130.5	6.76 dd (8.4,2.4)	127.9	6.77 dd (8.4, 2.4)	128.0
7⁗	3.80 s	35.5	3.80 s	35.5	3.76 s	35.6	3.65s	41.0
1⁗′		133.0		133.1		133.1		133.1
2⁗′	6.94 d (8.4)	130.4	7.04 d (8.4)	130.5	7.05 d (8.4)	130.6	7.05 d (8.4)	130.6
3⁗′	6.71 d (8.4)	115.7	6.69 d (8.4)	115.7	6.71 d (8.4)	115.9	6.69 d (8.4)	115.8
4⁗′		156.2		156.2		156.2		156.3
5⁗′	6.71 d (8.4)	115.7	6.69 d (8.4)	115.7	6.71 d (8.4)	115.9	6.69 d (8.4)	115.8
6⁗′	6.94 d (8.4)	130.4	7.04 d (8.4)	130.5	7.05 d (8.4)	130.6	7.05 d (8.4)	130.6
7⁗′	3.75 s	35.5	3.80 s	35.5	3.81 s	35.6	3.82s	35.6
4-OH	8.00 s		7.93 s		6.91 s		6.87 s	
4′-OH	7.97 s		7.94 s		8.00 s		7.99~8.08 s	
4″-OH	7.95 s		7.94 s		8.02 s		7.99~8.08 s	
2‴-OH							7.99~8.08 s	
4‴-OH	8.01 s		7.99 s		8.03 s			
4⁗-OH	8.07 s		7.98 s		8.09 s		7.99~8.08 s	
4⁗′-OH	7.94 s		7.99 s		8.09 s		7.99~8.08 s	

[a] ^{1}H NMR data were measured at 600 MHz. ^{13}C NMR data were measured at 150 MHz at ^{13}C. Proton coupling constants (J) in Hz are given in parentheses. Assignments were based on DEPT, ^{1}H-^{1}H gCOSY, gHSQC, and gHMBC experiments. [b], [c] The assignments with the same symbol in the same column may be exchangeable.

1-{2-Hydroxy-3-[4-hydroxy-3-(4-hydroxybenzyl)benzyl]benzyl}-3,5-di-(4-hydroxybenzyl) phenol (**24**): yellowish gum; UV (MeOH) λ_{max} (log ε) 204 (4.88), 225 (4.59), 281 (3.98); IR ν_{max}

3364, 3018, 2917, 2849, 2704, 2605, 1890, 1697, 1612, 1511, 1477, 1441, 1365, 1232, 1174, 1103, 1015, 911, 824, 780 cm^{-1}; ^{1}H NMR (acetone-d_6, 600 MHz) and ^{13}C NMR (acetone-d_6, 150 MHz) data, see Table S6; (+)-HRESIMS: *m*/*z* 647.2398 $[M + Na]^+$ (calcd. for $C_{41}H_{36}O_6Na$, 647.2404).

1-[2-Hydroxy-3,5-di-(4-hydroxybenzl)benzyl]-3,5-di-(4-hydroxybenzl)phenol (**25**): yellowish gum; UV (MeOH) λ_{max} (log ε) 204 (4.93), 225 (4.72), 280 (4.00); IR ν_{max} 3360, 3020, 2911, 2846, 1889, 1770, 1646, 1612, 1598, 1512, 1475, 1442, 1340, 1223, 1173, 1135, 1101, 1015, 911, 827, 785 cm^{-1}; ^{1}H NMR (acetone-d_6, 600 MHz) and ^{13}C NMR (acetone-d_6, 150 MHz) data, see Table S7; (+)-HRESIMS: *m*/*z* 647.2400 $[M + Na]^+$ (calcd. for $C_{41}H_{36}O_6Na$, 647.2404).

1-[2-Hydroxy-5-(4-hydroxybenzyl)benzyl]-3-[4-hydroxy-3-(4-hydroxybenzyl)benzyl]-5-(4-hydroxybenzyl)phenol (**26**): yellowish gum; UV (MeOH) λ_{max} (log ε) 204 (4.94), 225 (4.65), 280 (4.08); IR ν_{max} 3381, 3019, 2913, 2841, 2703, 2606, 1889, 1789, 1611, 1511, 1476, 1440, 1357, 1228, 1174, 1103, 1015, 984, 913, 821, 777 cm^{-1}; ^{1}H NMR (acetone-d_6, 600 MHz) and ^{13}C NMR (acetone-d_6, 150 MHz) data, see Table S7; (+)-HRESIMS: *m*/*z* 647.2396 $[M + Na]^+$ (calcd. for $C_{41}H_{36}O_6Na$, 647.2404).

1,5-Di-[2-hydroxy-5-(4-hydroxybenzyl)benzyl]-3-(4-hydroxybenzyl)phenol (**27**): yellowish gum; UV (MeOH) λ_{max} (log ε) 204 (5.03), 224 (4.70), 282 (4.10); IR ν_{max} 3328, 3019, 2916, 2842, 2703, 2598, 1887, 1696, 1611, 1598, 1510, 1475, 1440, 1365, 1229, 1172, 1100, 1015, 910, 817, 772 cm^{-1}; ^{1}H NMR (acetone-d_6, 600 MHz) and ^{13}C NMR (acetone-d_6, 150 MHz) data, see Table S7; (+)-HRESIMS: *m*/*z* 647.2399 $[M + Na]^+$ (calcd. for $C_{41}H_{36}O_6Na$, 647.2404).

Di-[2-hydroxy-3,5-di-(4-hydroxybenzyl)]benzyl ether (**28**): yellowish gum; UV (MeOH) λ_{max} (log ε) 204 (4.78), 224 (4.50), 281 (3.86); IR ν_{max} 3365, 3020, 2914, 2848, 1697, 1612, 1598, 1513, 1481, 1445, 1363, 1231, 1172, 1143, 1101, 1055, 1014, 887, 833, 775 cm^{-1}; ^{1}H NMR (acetone-d_6,

600 MHz) and ^{13}C NMR (acetone-d_6, 150 MHz) data, see Table S7; (+)-HRESIMS: *m*/*z* 677.2507 $[M + Na]^+$ (calcd. for $C_{42}H_{38}O_7Na$, 677.2510).

Table S7 NMR Spectroscopic Data for Compounds **25–28** in acetone-d_6

no.	**25**		**26**		**27**		**28**	
	δ_H	δ_C	δ_H	δ_C	δ_H	δ_C	δ_H	δ_C
1		132.7		132.5		133.5		124.2
2	6.79 s	129.9	6.82 s	129.9	6.80 d (2.4)	130.0		152.7
3		129.2		129.0		127.9		129.7
4		151.4		151.3		151.1	6.92 d (2.4)	131.7
5		129.2		129.0		129.4		133.5
6	6.79 s	129.9	6.81 s	129.9	6.99 d (2.4)	129.8	6.85 d (2.4)	128.5
7							4.66 s	71.0
1′		132.5		133.1		132.9		132.6
2′	7.01 d (9.0)	130.6	6.97 d (1.8)	131.9	7.01 d (8.4)	130.5	7.04 d (9.0)	130.5
3′	6.71 d (9.0)	115.9		129.0	6.80 d (8.4)	115.7	6.71 d (9.0)	115.9
4′		156.4		153.9[d]		156.2		156.4
5′	6.71 d (9.0)	115.9	6.71 d (8.4)	115.7[e]	6.80 d (8.4)	115.7	6.71 d (9.0)	115.9
6′	7.01 d (9.0)	130.6	6.81 dd	128.1	7.01 d (8.4)	130.5	7.04 d (9.0)	130.5
7′	3.85 s	35.9	3.82 s	35.9	3.79 s	35.9	3.85 s	35.4
1″		132.5		133.2		128.0		133.9
2″	7.01 d (9.0)	130.6	7.05 d (8.4)	130.6		152.2	6.98 d (8.4)	130.6
3″	6.71 d (9.0)	115.9	6.71 d (8.4)	115.8[f]	6.76 d (8.4)	115.8	6.71 d (8.4)	116.0
4″		156.4		156.4	6.82 dd (8.4, 2.4)	128.3		156.4
5″	6.71 d (9.0)	115.9	6.71 d (8.4)	115.8[f]		134.9	6.71 d (8.4)	116.0
6″	7.01 d (9.0)	130.6	7.05 d (8.4)	130.6	7.07 d (2.4)	131.8	6.98 d (8.4)	130.6
7″	3.85 s	35.9	3.74 s	35.6	3.83 s	31.4	3.72 s	40.8
1‴		129.2[b]		128.9		127.9		124.2
2‴		151.4		153.8[d]		153.8		152.7
3‴		129.1[b]	6.70 d (8.4)	115.8[e]	6.71 d (8.4)	115.7		129.7
4‴	6.71 d (1.8)	129.6	6.79 dd	127.9	6.79 dd (8.4, 2.4)	127.9	6.92 d (2.4)	131.7
5‴		133.8[c]		133.8[g]		133.5		133.5
6‴	6.76 d (1.8)	129.6	6.84 d (2.4)	131.5	6.87 d (2.4)	131.6	6.85 d (2.4)	128.5
7‴	3.80 s	35.9	3.81 s	35.5	3.75 s	35.5	4.66 s	71.0
1⁗		133.9[c]		133.7[g]		133.7		132.6
2⁗	6.94 d (8.4)	130.4	6.96 d (8.4)	130.4	6.96 d (8.4)	130.4	7.04 d (9.0)	130.5
3⁗	6.70 d (8.4)	115.9	6.72 d (8.4)	115.9[f]	6.71 d (8.4)	115.9	6.71 d (9.0)	115.9
4⁗		156.3		156.4		156.3		156.4
5⁗	6.70 d (8.4)	115.9	6.72 d (8.4)	115.9[f]	6.71 d (8.4)	115.9	6.71 d (9.0)	115.9
6⁗	6.94 d (8.4)	130.4	6.96 d (8.4)	130.4	6.96 d (8.4)	130.4	7.04 d (9.0)	130.5
7⁗	3.66 s	41.0	3.69 s	40.9	3.69 s	40.8	3.85 s	35.4
1⁗′		132.6		132.6		133.8		133.9
2⁗′	7.01 d (8.4)	130.5	7.05 d (8.4)	130.5	6.97 d (8.4)	130.5	6.98 d (8.4)	130.6
3⁗′	6.71 d (8.4)	115.9	6.71 d (8.4)	115.8[f]	6.71 d (8.4)	115.9	6.71 d (8.4)	116.0
4⁗′		156.4		156.3		156.3		156.4
5⁗′	6.71 d (8.4)	115.9	6.71 d (8.4)	115.8[f]	6.71 d (8.4)	115.9	6.71 d (8.4)	116.0
6⁗′	7.01 d (8.4)	130.5	7.05 d (8.4)	130.5	6.97 d (8.4)	130.5	6.98 d (8.4)	130.6
7⁗′	3.85 s	35.9	3.85 s	35.9	3.70 s	40.8	3.72 s	40.8
4-OH	6.90 s		6.85 s		6.96 s			
2-OH							7.63 s	
2″-OH					7.99~8.06 s			
4′-OH	8.08 s		8.00~8.07 s		7.99~8.06 s		8.11 s	
4″-OH	8.08 s		8.00~8.07 s				8.10 s	
2‴-OH	6.92 s		8.00~8.07 s		7.99~8.06 s		7.63 s	
4⁗-OH	8.08 s		8.00~8.07 s		7.99~8.06 s		8.11 s	
4⁗′-OH	8.08 s		8.00~8.07 s		7.99~8.06 s		8.10 s	

a) ^{1}H NMR data were measured at 600 MHz. ^{13}C NMR data were measured at 150 MHz at ^{13}C. Proton coupling constants (*J*) in Hz are given in parentheses. Assignments were based on DEPT, ^{1}H-^{1}H gCOSY, gHSQC, and gHMBC experiments. b), c), d), e), f) The assignments with the same symbol in the same column may be exchangeable.

5. Synthesis of 4, 5, and 13

See ref. 46 of manuscript.

6. Preparation and UPLC/HRESIMS analysis of the *p*-hydroxybenzyl alcohol

See ref. 37 of manuscript. Difference from ref. 37 of manuscript: flow rate, 0.3 mL min^{-1}; gradient elution of increasing CH_3CN in H_2O from 30% to 45% in 15.0 min.

7. Preparation and UPLC/HRESIMS analysis of the fresh *G. elata* rhizomes and Tian ma extracts

See ref. 37 of manuscript. The diluted samples were individually filtrated and the filtrates were analyzed by UPLC/HRESIMS under the above-described conditions, respectively.

8. Bioassays

8.1 Protective effect against cytotoxicity induced PC12 cell damage

PC12 cells at a density of 5×10^3 cells per well in 96-well plates were cultured in DMEM media (Hyclone) supplemented with 5% fetal bovine serum (FBS, Hyclone) and 5% horse serum (Hyclone), and L-glutamine (2 μM). Cultures were maintained at 37 °C in 5% CO_2 in a humidified incubator. After incubation for 24 h, compounds at concentrations of 10 μM or 1 μM NGF ((Nerve Growth Factor) were added to the cells, the serum-free model group was replaced with DMEM medium containing 1% fetal bovine serum and 1% horse serum, while the glutamate model group was replaced with DMEM medium containing 20 μM glutamate. After incubation for another 24 h 10 μL of the 0.5 mg/mL MTT was added and maintained for 4 h. Absorbance was measured at 570 nm using BIORAD 550 microplate reader. Cell viability was evaluated as $(OD_{sample} - OD_{model})/(OD_{control} - OD_{model})\times100\%$.

Table S8 Survival rate of PC12 cells (10 μM, means ± SD, n = 3)

no	serum-free model	rotenone-induced model
control	100.00 ± 2.96	100.00 ± 3.76
model	61.84 ± 15.65$^{###}$	67.11 ± 5.61$^{###}$
**1**	23.76 ± 4.96^{+++}	73.74 ± 5.52**
**2**	49.11 ± 15.10	72.01 ± 2.78**
**3**	41.24 ± 22.76+	71.00 ± 4.27*
**6**	17.87 ± 2.33^{+++}	81.56 ± 2.40***
control	100 ± 4.9	100 ± 3.2
model	75.9 ± 4.3$^{###}$	74.0 ± 11.6$^{###}$
**5**	56.86 ± 0.128	88.26 ± 0.010
**7**	26.00 ± 0.016**	97.64 ± 0.104
**8**	26.53 ± 0.008**	102.58 ± 0.075
**10**	26.40 ± 0.005**	104.80 ± 0.084
**14**	25.64 ± 0.011**	111.94 ± 0.070
gastrodin	62.30 ± 0.076	60.03 ± 0.354

p<0.001; *** *p*<0.001; ***p*<0.01; +++ *p*<0.001; "+" with toxic effect, * with protective effect.

Table S9 Survival rate of PC12 cells (10 μM, means ± SD, n = 3)

no	serum-free model	α-syn induced model	rotenone-induced model
control	100.00 ± 5.47	100.00 ± 9.55	100.00 ± 4.69
model	78.93 ± 10.09$^{###}$	62.98 ± 4.59$^{###}$	56.08 ± 4.42$^{###}$
**4**	32.86 ± 11.35^{+++}	78.13 ± 15.32**	73.94 ± 9.88***
p-hydroxybenzyl alcohol		71.61 ± 12.62*	60.06 ± 7.75
gastrodin		68.80 ± 11.18	63.51 ± 8.94*

p<0.001; *** *p*<0.001; ***p*<0.01; +++ *p*<0.001; "+" with toxic effect,"*" with protective effect.

8.2 Anti-HIV-1 (VSVG/HIV-luc) activity assay

293T cells at a density of 2.2×10^6 cells per well in 100 mm plates were cultured. After 24 h, 3 μg VSV-G plasmid and 8 μg pNL4-3. luc.R^-E^- (HIV-1 core) using improved calcium phosphate were co-transfected. For another 16 h, wash the cells with PBS and continue to culture in fresh medium for 32 h. Collect the supernatant and filter it through a 0.45 *μ*M membrane, of which contains VSVG/HIV pseudovirus particles. On the day before infection, inoculate onto a 24 well plate at a density of 6×10^4 cells per well. Add tested compounds dissolved in DMSO to the cell culture medium at 15 min before infection, and use DMSO solvent as a blank control. Infect cells with VSVG/HIV pseudovirus particles solution of appropriate dilution, and after 48 h of infection, add 50 μL cell lysate (Promega) to each well. Mix 30 μL luciferase substrate (Promega) and 20 μL cell lysate and measure the activity of cell luciferin using an FB12 fluorescence detector (Sirius) instrument, of which the strength of the activity reflects the level of HIV-1 replication. The

inhibition rate is calculated using the following formula:

Inhibition (%) = (control group average luciferase activity − test group average luciferase activity) / control group average luciferase activity × 100%

Table S10 Inhibition activity against HIV-1 replication in 293T cells

group	C (μM)	inhibition (%)
efavirenz	0.01	99.60±0.12
control	0.001	51.27±3.61
**4**	10	70.31±1.06
**4**	3	33.36±3.13
**4**	1	4.62±3.69
p-hydroxybenzyl alcohol	10	9.71±12.11
gastrodin	10	25.82±5.04
efavirenz	0.01	99.11±0.07
control	0.001	41.35±4.15
**1**	10	54.03±1.76
**2**	10	50.19±1.99
**3**	10	51.97±10.59
**6**	10	24.25±6.35

8.3 Inhibitory activity against NO release in LPS-induced RAW 264.7 cells

Sodium thioglycolate induced RAW 264.7 cells 2×10^4 cells per well in 96-well plates were cultured in RPMI-1640 media (contained 10% fetal bovine serum, 100 U/mL penicillin, and 100 μg/mL streptomycin) and maintained at 37 °C in 5% CO_2 in a humidified incubator. The experiment was divided into blank control group, LPS-induced group, administration group, and positive drug group (DEX or curcumin). Among them, the blank control group only added culture medium, the LPS group added LPS (final concentration of 1 μg/mL), and the treatment group added both LPS and drugs simultaneously. After 24 h, take 100 μL supernatant, add Griess reagent and react for 10 min. Use ELISA reader to measure the OD (optical density) value at 540 nm, and calculate the NO production based on the NO standard curve.

Table S11 Inhibition rate against NO release in LPS-induced RAW 264.7 cells (10 μM)

compound	C (μM)	NO release inhibition (%)	Cell growth inhibition (%)
Dex	1	86.5	8.6
**1**	10	73.5	−31.3
**2**	10	53.3	−13.3
**3**	10	46.9	−20.3
**6**	10	66.8	−35.4
Dex	1	62.3	14.5
**4**	10	22.8	−44.8

DEX	1	83.4	6.9
**7**	10	90.0	−41.9
**8**	10	95.3	89.7
**10**	10	84.1	−48.3
**14**	10	88.9	−40.8
p-hydroxybenzyl alcohol	10	15.04	−18.30
gastrodin		−18.59	−16.96

Table S12 Inhibitory activity against NO release in LPS-induced RAW 264.7 cells (10 μM)

compound	NO release inhibition (%)	compound	NO release inhibition (%)
**15**	5.41	**24**	15.15
**16**	0.00	**25**	4.36
**17**	25.00	**26**	8.33
**18**	5.53	**27**	14.02
**19**	37.98	**28**	17.79
**20**	18.87	**29**	0.00
**21**	12.88	**30**	4.21
**22**	29.92	Curcumin	90.65
**23**	29.92		

8.4 Inhibitory activity assay against Fe^{2+}-cysteine induced lipid peroxidation of liver microsomes

Rat liver microsomes were extracted for use. A blank group, a model group, a positive drug group and an administration group were set up, with 3 replicate wells in each group. 155 μL PBS buffer, 30 μL liver microsomes, and 50 μL cysteine were add to each tube, a total of 235 μL/tube of the mixed system. 2.5 μL DMSO was added to each well of blank group and model group, 2.5 μL curcumin (10^{-3} M) (final concentration 10^{-5} M) was added to each tube of positive drug group, and 2.5 μL compound (10^{-3} M) was added to each tube of administration group (final concentration 10^{-5} M) for screening. Then, shaked in a water bath at 37°C for 15 min. Each tube of positive drug group, model group and administration group was added with 13 μL FeSO4 solution (10^{-2} M) (currently used and prepared, quick operation), and the blank group was added with 13 μL PBS buffer, and shaken in a water bath at 37°C for 15 min. 250 μL TCA solution and 500 μL TBA solution were added to each tube, and be placed in a water bath at 100 °C for 10 min. 600 μL of each tube was taken into a 1.5 mL EP tube and centrifuged at 8000 rpm for 10 min. The supernatant

200 μL was taken into a 96-well culture plate, and measure the absorbance (OD) at 532 nm (select MDA).

MDA concentration (nM/mL) = $(OD_{test} - 0.006)/0.07 \times 10$

MDA production inhibition rate (%) = $[1-(C_{compound} - C_{blank})/(C_{model} - C_{blank})] \times 100\%$

Table S13 Inhibition rate against Fe^{2+}-cysteine induced lipid peroxidation of liver microsomes

compound	MDA inhibition (%)	compound	MDA inhibition (%)
**4**	92.36	**19**	86.53
**5**	92.11	**20**	83.67
**6**	99.88	**21**	85.58
**7**	99.88	**22**	89.39
**8**	97.29	**23**	94.87
**9**	93.33	**24**	92.85
**10**	100.49	**25**	95.11
**12**	94.28	**26**	94.76
**13**	94.04	**27**	91.06
**14**	97.26	**28**	78.31
**15**	89.52	**30**	80.02
**16**	80.02	curcumin	97.66
**17**	84.83	*p*-hydroxybenzyl alcohol	6
**18**	78.55		

8.5 Inhibition assay against TNF-α secretion in LPS-induced RAW264.7

RAW264.7 cells at a density of 2×10^4 cells per well in 96-well plates were cultured in 1640 media (Invitrogen) supplemented with 10% fetal bovine serum (FBS, Gibco). Cultures were maintained at 37 °C in 5% CO_2 in a humidified incubator. After incubation for 12-18 h, compounds at concentrations of 10 μM or 10μL/well dexamethasone were added to the cells. After incubation for 1 h. 10 μL LPS (Sigma) was added and maintained for 24 h. Absorbance was measured at 450 and 570 nm using ELISA (ebioscience) reagent test kit by BioTek H1 microplate reader, then using the standard curve method to obtain the concentration of TNF-α. Inhibition rate was calculated as $(C_{model}-C_{sample})/C_{model} \times 100\%$ evaluated.

Table S14 Inhibitory activity against TNF-α secretion in LPS-induced RAW 264.7 cells (10 μM)

compound	NO release inhibition (%)	compound	NO release inhibition (%)
**4**	69.70#	**19**	−13.67
**5**	−278.92	**20**	2.45
**6**	−171.44	**21**	−57.17
**7**	33.74	**22**	−53.64
**8**	−169.31	**23**	−52.09
**9**	−142.89	**24**	−98.99

**10**	−136.39	**25**	−54.03
**12**	−47.87	**26**	−74.64
**13**	−97.19	**27**	−59.94
**14**	−154.1	**28**	−41.12
**15**	−145.92	**29**	16.76
**16**	0.07	**30**	−0.83
**17**	−31.33	DEX	66.85±0.64
**18**	−53.64		

8.6 Assay for α-glucosidase inhibitors

Set up blank control group (DMSO, 0.1M PBS, 0.005U/L *α*-glucosidase, 2 Mm PNPP), model group (DMSO, 0.1M PBS, 2 mM PNPP), positive drug group (positive drug 10 μg/ml, 0.1M PBS, 0.005U/L *α*-glucosidase, 2 mM PNPP), and sample group (test compound 10 μM, 0.1M PBS, 0.005U/L *α*-glucosidase, 2 mM PNPP) respectively. 150 *μ*L reactive system contained 10 *μ*M or 10 *μ*g/mL tested compound, 0.1M PBS, 0.005U/L *α*-glucosidase, and 2 mM PNPP shake at 30 °C and 90 rpm for 15 min, and add 0.4 M Na_2CO_3 to terminate reaction. Then absorbance was measured at 405 nm. Read the OD value at 405 nm to determine the concentration of PNPP in the sample and calculate the inhibition rate.

Inhibition (%) = $[C_{test} - C_{control}]/[C_{model} - C_{blank}] \times 100\%$

Table S15 Inhibition rate against *α*-glucosidase activity

compound	inhibition (%)	IC_{50} (μM)	compound	inhibition (%)	IC_{50} (μM)
**4**	63.5		**16**	20.4	
**5**	94.9	0.45	**17**	35.9	
**6**	38.6		**18**	61.2	
**7**	89.8	2.88	**19**	90.9	1.46
**8**	78.1	3.90	**23**	99.8	1.04
**9**	88.6	3.46	**24**	99.9	4.60
**10**	89.4	2.30	**25**	99.6	1.42
**12**	89.0	1.57	**26**	99.9	1.03
**13**	99.9	1.05	**27**	98.0	1.84
**14**	90.4	3.63	**29**	91.4	0.84
**15**	30.1		Acar (200 μM)	75.2	

8.7 Assay for PTP1B (human protein tyrosine phosphatase 1B) inhibitors

Set up blank control group (DMSO, 50 mM Hepes buffer, PTP1B protein, 2 mM PNPP),

model group (DMSO, 50 mM Hepes buffer, 2 mM PNPP), positive drug group (positive drug 10 μg/ml, 50 mM Hepes buffer, PTP1B protein, 2 mM PNPP), and sample group (test compound 10 μM, 50 mM Hepes buffer, PTP1B protein, 2 mM PNPP) respectively. 150 μL reactive system contained 10 μM or 10 μg/mL tested compound, 50 mM Hepes buffer (pH 7.0), PTP1B protein, and 2 mM PNPP shake at 30 °C and 90rpm for 10 min, and add 3 M NaOH to terminate reaction. Then absorbance was measured at 405 nm. Read the OD value at 405 nm to determine the concentration of PNPP in the sample and calculate the inhibition rate.

Inhibition (%) = $[C_{test} - C_{control}]/[C_{model} - C_{blank}]\times100\%$

Table S16 Inhibition rate against PTP1B actvity

compound	inhibition (%)	IC_{50} (μM)	compound	inhibition (%)	IC_{50} (μM)
**4**	10.0		**16**	−5.0	
**5**	20.9		**17**	−13.0	
**6**	0.0		**18**	66.2	
**7**	64.6		**19**	79.2	
**8**	36.5		**23**	96.7	2.63
**9**	68.1		**24**	96.1	4.92
**10**	77.2		**25**	96.5	2.25
**12**	93.5	4.41	**26**	96.3	2.85
**13**	95.5	4.55	**27**	97.3	3.47
**14**	84.6	4.74	**29**	7.9	
**15**	1.7		CC06240	100.5	

8.8 Assay against replication of influenza virus A/Hanfang/359/95 (H3N3)

MDCK cells were seeded in 96-well culture plates and cultured at 37°C with 5% CO_2. After 24 hours, the influenza virus was infected for 10^{-5}, adsorbed for 2 hours, the virus solution was discarded, and the maintenance solution containing different concentrations of samples and positive control drug (ribavirin) was added, and cell control wells and virus control were set at the same time. The wells were incubated at 37°C with 5% CO_2. When the cytopathic effect (CPE) of the virus control group reached 4+, the CPE of each group was observed, and the Reed-Muench method was used to calculate the half toxic concentration (TC_{50}) of the samples to cells and the half inhibitory concentration (IC_{50}) to virus, respectively.

Table S17 Anti-A/Hanfang/359/95 (H3N3) activity

Compound	Initial concentration (μM)	TC_{50} (μM)	IC_{50} (μM)	SI
**4**	100	19.25	>11.11	-
**5**	100	19.25	11.11	1.7
**6**	100	7.70	>3.70	
**7**	100	6.42	>3.70	-
**8**	100	6.42	3.70	1.7
**9**	100	7.70	>3.70	-
**10**	100	19.25	>11.11	-
**12**	100	6.42	>3.70	-
**13**	100	6.42	3.70	1.7
**14**	100	6.42	>3.70	
**15**	100	19.25	>11.11	-
**16**	100	48.07	11.11	4.3
**17**	100	23.11	>11.11	-
**18**	100	19.25	>11.11	-
**19**	100	6.42	>3.70	-
**20**	100	11.11	>3.70	-
**21**	100	19.25	>11.11	-
**22**	100	19.25	>11.11	-
**23**	100	19.25	>11.11	-
**24**	100	6.42	>3.70	-
**25**	100	19.25	>11.11	-
**26**	100	7.70	>3.70	-
**27**	100	19.25	>11.11	-
**28**	100	19.25	>11.11	-
**29**	100	19.25	>11.11	
Oseltamivir (μg/mL)	200	>200	2.01	>99.5
RBV (μg/mL)	200	>200	2.47	>81.0

8.9 Assay against replication of coxsackievirus B3 type (CVB3)

Vero cells were seeded in 96-well culture plates and cultured at 37°C with 5% CO_2. After 24 hours, the influenza virus was infected for 10^{-5}, adsorbed for 2 hours, the virus solution was discarded, and the maintenance solution containing different concentrations of samples and positive control drug (ribavirin) was added, and cell control wells and virus control were set at the same time. The wells were incubated at 37°C with 5% CO_2. When the cytopathic effect (CPE) of the virus control group reached 4+, the CPE of each group was observed, and the Reed-Muench method was used to calculate the half toxic concentration (TC_{50}) of the samples to cells and the half inhibitory concentration (IC_{50}) to virus, respectively.

Table S18 Activity against CVB3 replication

Compound	Initial concentration (μM)	TC_{50} (μM)	IC_{50} (μM)	SI
**4**	100	19.25	11.11	1.7
**5**	100	6.42	3.70	1.7
**6**	100	6.42	>3.70	-
**7**	100	7.70	3.70	2.1
**8**	100	19.25	>11.11	-
**9**	100	19.25	>11.11	-
**10**	100	6.42	>3.70	-
**12**	100	11.11	>3.70	-
**13**	100	19.25	>11.11	-
**14**	100	16.02	>3.70	-
**15**	100	57.74	>33.33	-
**16**	100	57.74	14.32	4.0
**17**	100	19.25	8.62	2.2
**18**	100	19.25	>11.11	-
**19**	100	16.02	>3.70	-
**20**	100	16.02	>3.70	-
**21**	100	19.25	>11.11	-
**22**	100	19.25	>11.11	-
**23**	100	6.42	>3.70	-
**24**	100	16.02	>3.70	-
**25**	100	19.25	>11.11	-
**26**	100	7.70	>3.70	-
**27**	100	19.25	>11.11	-
**28**	100	19.25	>11.11	-
**29**	100	11.11	>3.70	
Oseltamivir (μg/mL)	200	15.41	0.0021	7338.1
RBV (μg/mL)	200	2000	517.38	3.9

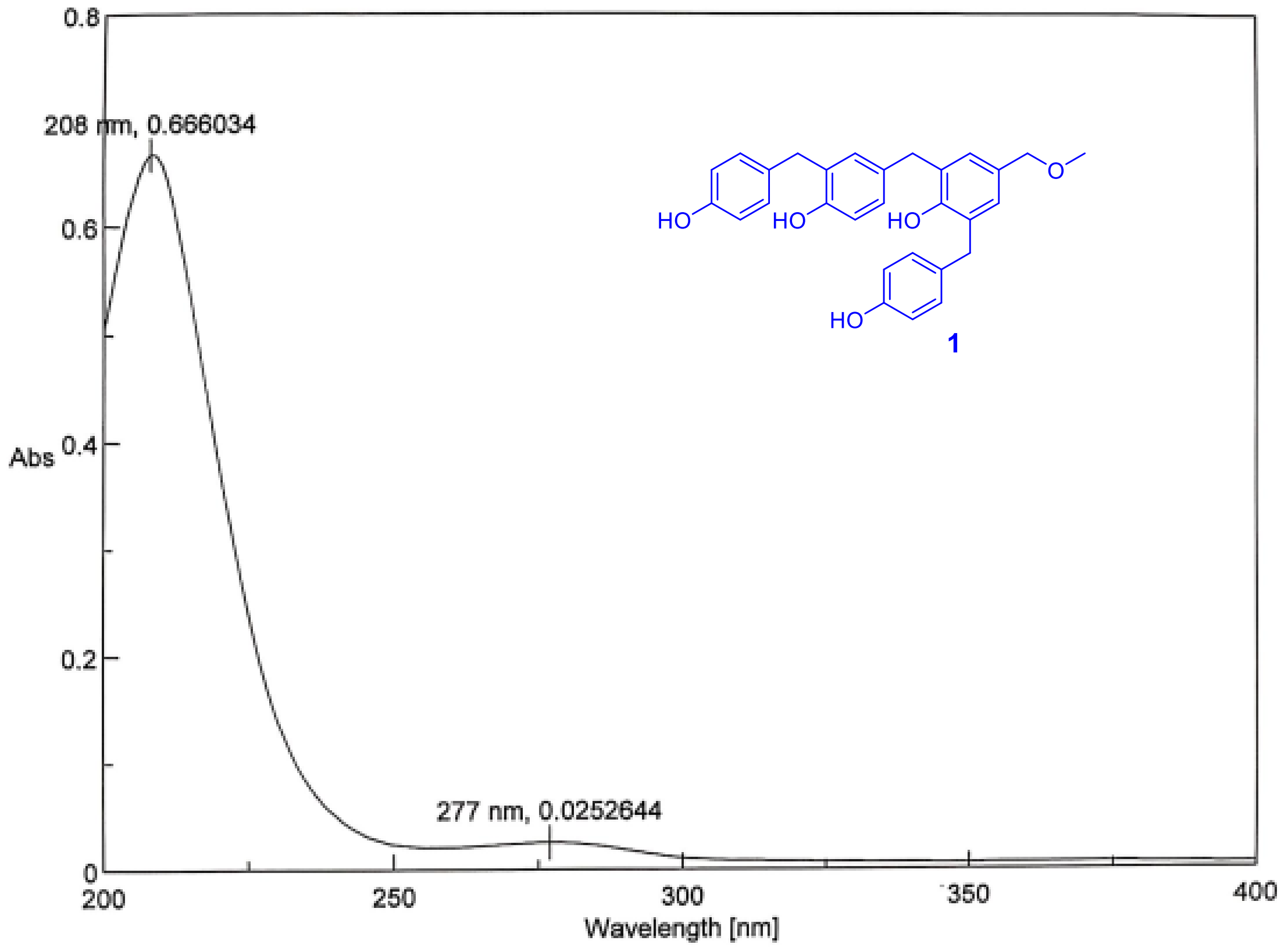

[Comment]
Sample Name wyn-33
Comment
User 王亚男
Division
Company 324
[Measurement Information]
Instrument Name V-650
Model Name V-650
Serial No. A034461150

Accessory PSC-718
Accessory S/N A001761114
Position 1
Cell Length 10 mm
Temperature 20.00 C
Control Sonsor Holder
Monitor Sensor Holder
Start Mode Start immediately

Photometric Mode Abs
Measurement range 400 - 190 nm
Data pitch 1 nm
Band width(UV/Vis) 1.0 nm
Response Medium
Scanning speed 200 nm/min
Source Change 340 nm
Light Source D2/WI
Filter Exchange Step
Correction Baseline

[Data Information]
Creation Date 2011-5-11 19:25

Data array type Linear data array
Horizontal Wavelength [nm]
Vertical Abs
Start 400 nm
End 190 nm
Data pitch 1 nm
Data points 211

Figure S1. The UV spectrum of compound **1**

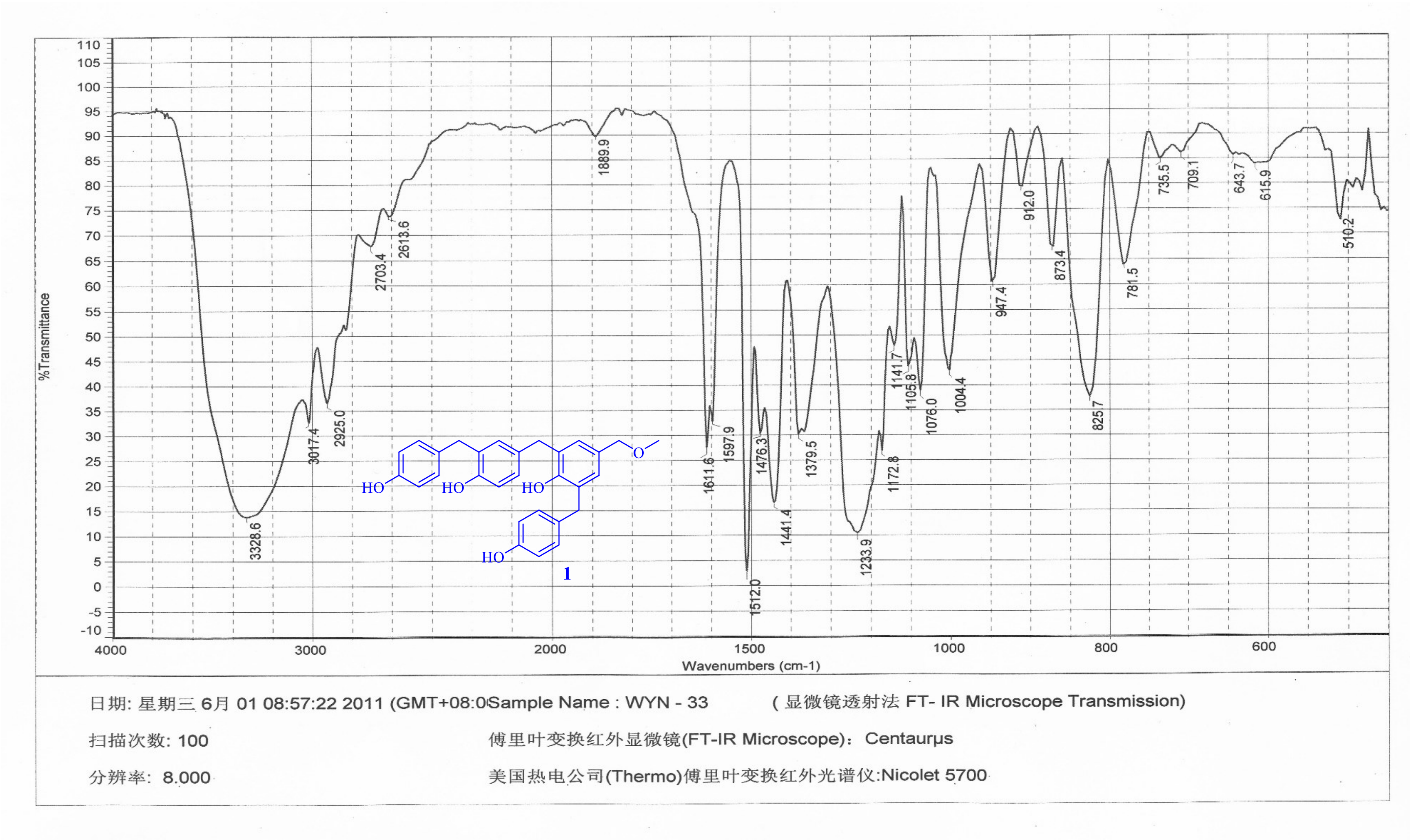

Figure S2. The IR spectrum of compound **1**

Single Mass Spectrum Deconvolution Report

Analysis Name: WANGY006.d **Instrument:** LC-MSD-Trap-SL **Print Date:** 7/9/2010 11:16:38 AM
Method: TEST.MS **Operator:** Operator **Acq. Date:** 7/9/2010 11:14:34 AM
Sample Name: WYN-33
Analysis Info:

Acquisition Parameter:

Mass Range Mode	Std/Normal	Trap Drive	53.0	Scan Begin	100 m/z
Ion Polarity	Positive	Octopole RF Amplitude	171.0 Vpp	Scan End	700 m/z
Ion Source Type	ESI	Capillary Exit	-106.0 Volt	Averages	5 Spectra
Dry Temp (Set)	330 °C	Skimmer	-40.0 Volt	Max. Accu Time	200000 µs
Nebulizer (Set)	15.00 psi	Oct 1 DC	-12.00 Volt	ICC Target	20000
Dry Gas (Set)	5.00 l/min	Oct 2 DC	-1.70 Volt	Charge Control	on

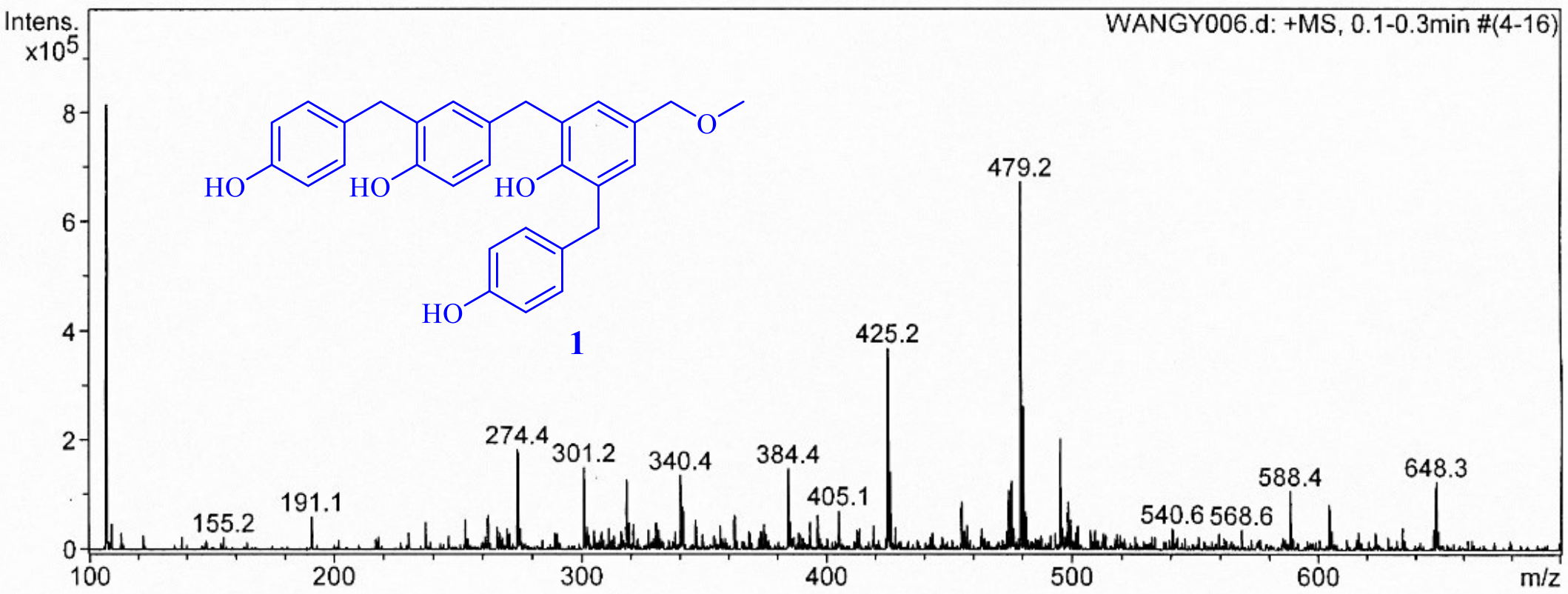

Component	Molecular Mass	Molecule	Absolute Abundance	Relative Abundance

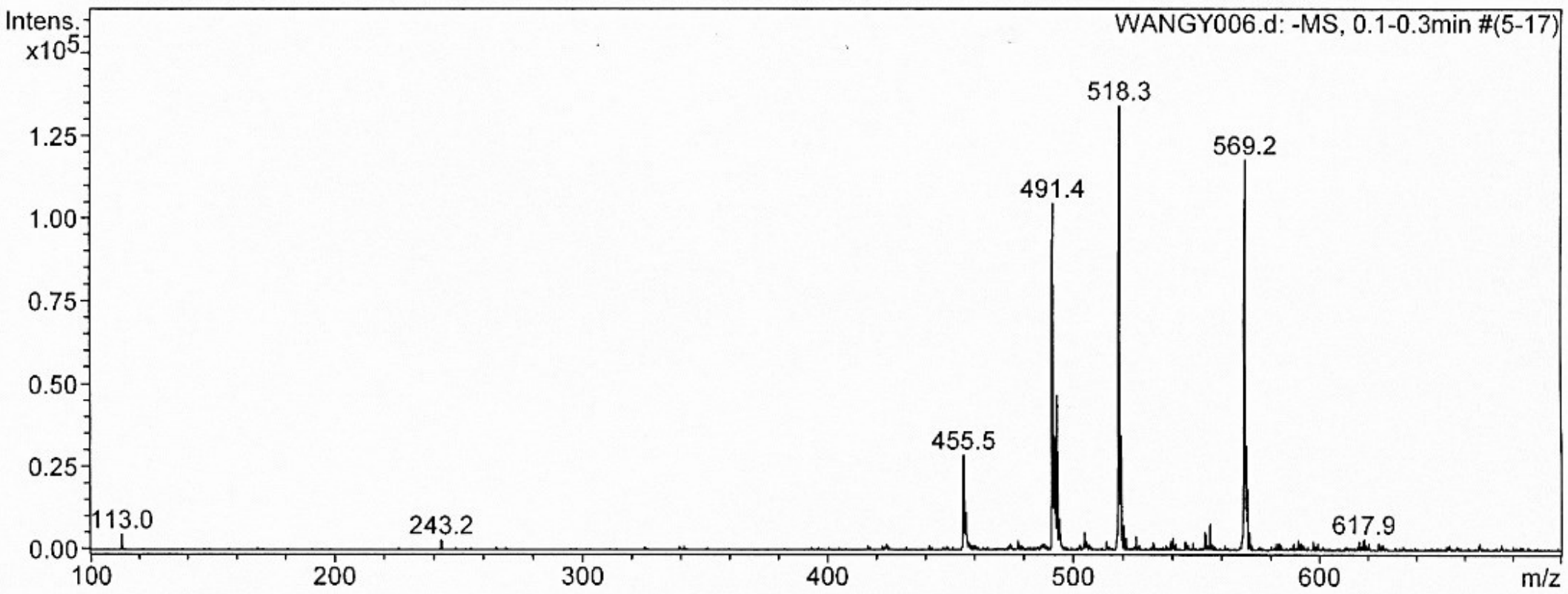

Component	Molecular Mass	Molecule	Absolute Abundance	Relative Abundance

MSD Trap Report v 4 (A4-Opt2) Page 1 of 1 Agilent Technologies

Figure S3. The ESIMS of compound **1**

Qualitative Analysis Report

**Data Filename**	201101176.d	**Sample Name**	WYN-33
**Sample Type**	Sample	**Position**	P1-C6
**Instrument Name**	Instrument 1	**User Name**	
**Acq Method**		**IRM Calibration Status**	Success
**DA Method**	TEST LCMS.m	**Comment**	

User Chromatograms

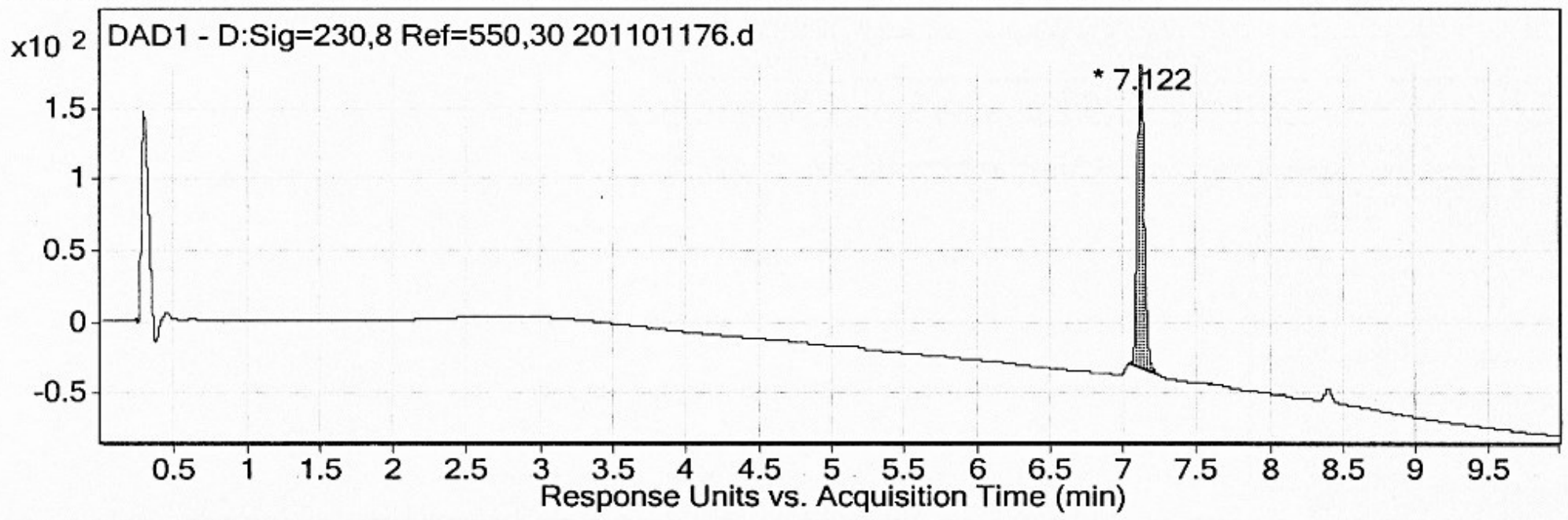

Integration Peak List

Peak	Start	RT	End	Height	Area	Area %
1	7.046	7.122	7.243	213.3	699.33	100

Fragmentor Voltage 135 **Collision Energy** 0 **Ionization Mode** ESI

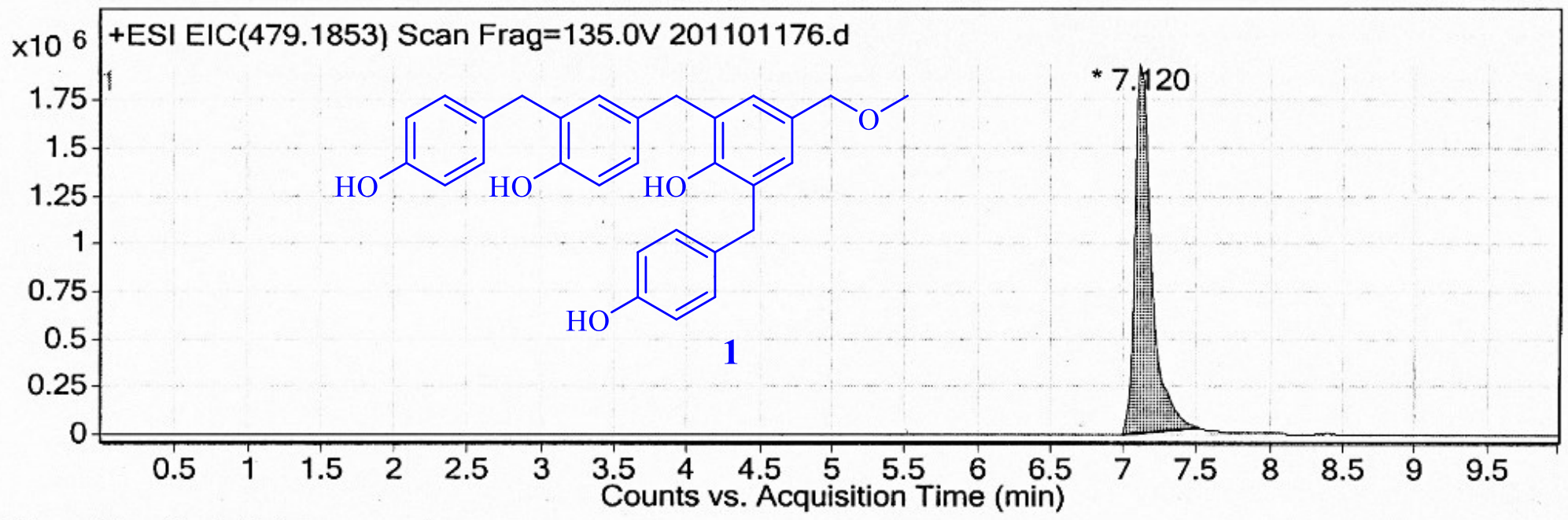

Integration Peak List

Peak	Start	RT	End	Height	Area	Area %
1	6.991	7.12	7.522	1925014	14960970	100

User Spectra

Fragmentor Voltage	Collision Energy	Ionization Mode
135	0	Esi

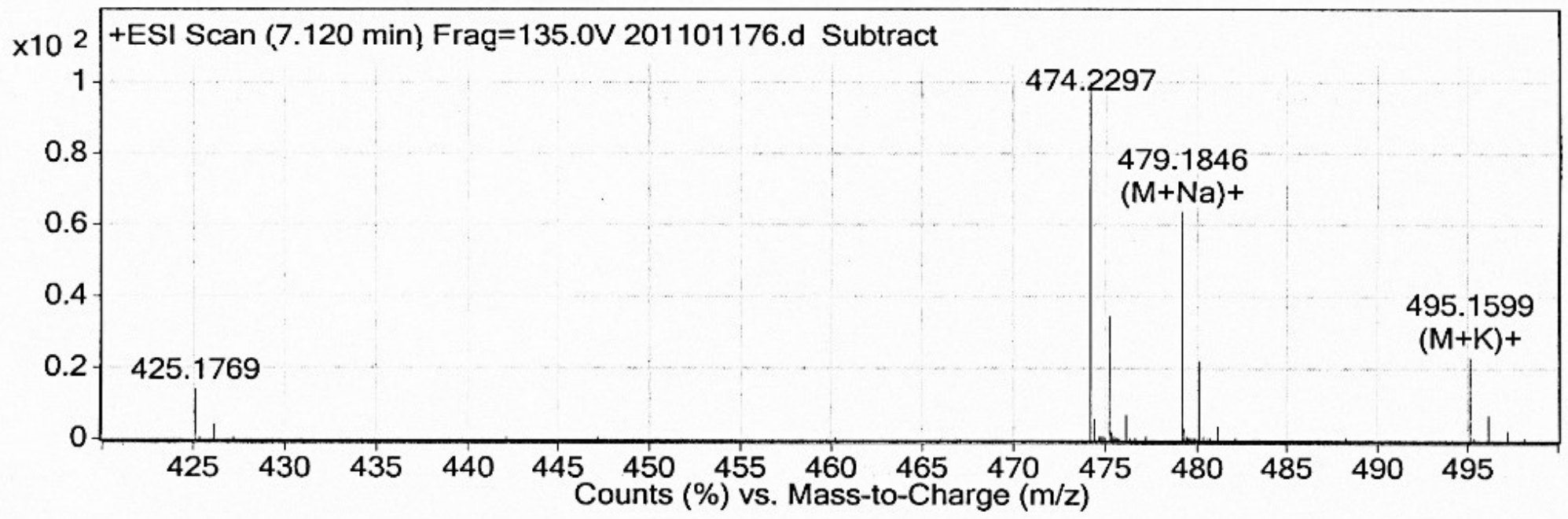

Figure S4. The HR-ESIMS report of compound **1** (page 1)

Qualitative Analysis Report

Peak List

m/z	z	Abund	Formula	Ion
425.1769		426343		
474.2297	1	3033089		
474.4159		176891		
475.2337	1	1055573		
476.236	1	213647		
479.1846	1	1930580	C29 H28 Na O5	(M+Na)+
480.1893	1	667039	C29 H28 Na O5	(M+Na)+
495.1599	1	709804	C29 H28 K O5	(M+K)+
496.1626	1	217431	C29 H28 K O5	(M+K)+
615.1604		160845		

Formula Calculator Element Limits

Element	Min	Max
C	3	100
H	0	120
O	0	30
N	0	3
S	0	0
Cl	0	0

Formula Calculator Results

Formula	Best	Mass	Tgt Mass	Diff (ppm)	Ion Species	Score
C29 H28 O5	TRUE	456.1954	456.1937	-3.8	C29 H28 Na O5	99.54
C22 H32 O10		456.1954	456.1995	9.07	C22 H32 Na O10	96.48
C17 H32 N2 O12		456.1954	456.1955	0.24	C17 H32 N2 Na O12	95.06
C29 H28 O5	TRUE	456.1968	456.1937	-6.75	C29 H28 K O5	99.32
C22 H32 O10		456.1968	456.1995	6.13	C22 H32 K O10	98.64
C17 H32 N2 O12		456.1968	456.1955	-2.71	C17 H32 K N2 O12	97.34

--- End Of Report ---

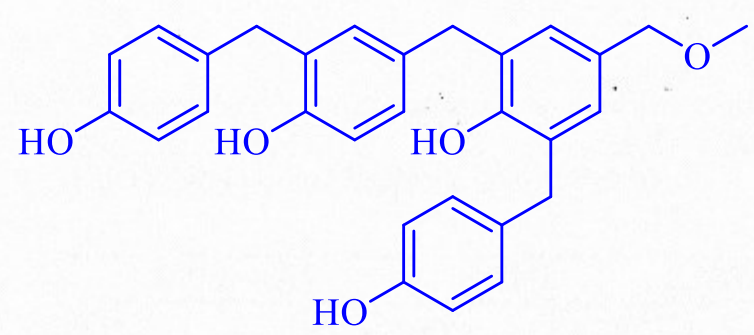

1

Agilent Technologies Page 2 of 2 Printed at: 10:54 AM on: 1/17/2011

Figure S5. The HR-ESIMS report of compound **1** (page 2)

MS Formula Results: + Scan (7.120 min) Sub (201101176.d)

m/z	Ion	Formula	Abundance
479.1846	(M+Na)+	C29 H28 Na O5	1930580.4

Best	Formula (M)	Ion Formula	Calc m/z	Score	Cross S	Mass	Calc Mass	Diff (ppm)	Abs Diff (ppm)	Abund Match	Spacing Mat	Mass Match	m/z	DBE
☑	C29 H28 O5	C29 H28 Na O5	479.1829	99.54		456.1954	456.1937	-3.8	3.8	99.3	99.78	99.55	479.1846	16
☐	C22 H32 O10	C22 H32 Na O10	479.1888	96.48		456.1954	456.1995	9.07	9.07	92.03	99.8	97.49	479.1846	7
☐	C17 H32 N2 O12	C17 H32 N2 Na O12	479.1847	95.06		456.1954	456.1955	0.24	0.24	82.94	99.73	100	479.1846	3

m/z	Ion	Formula	Abundance
495.1599	(M+K)+	C29 H28 K O5	709803.7

Best	Formula (M)	Ion Formula	Calc m/z	Score	Cross S	Mass	Calc Mass	Diff (ppm)	Abs Diff (ppm)	Abund Match	Spacing Mat	Mass Match	m/z	DBE
☑	C29 H28 O5	C29 H28 K O5	495.1568	99.32		456.1968	456.1937	-6.75	6.75	99.89	99.93	98.69	495.1599	16
☐	C22 H32 O10	C22 H32 K O10	495.1627	98.64		456.1968	456.1995	6.13	6.13	97.1	99.94	98.91	495.1599	7
☐	C17 H32 N2 O12	C17 H32 K N2 O12	495.1587	97.34		456.1968	456.1955	-2.71	2.71	91.07	99.96	99.79	495.1599	3

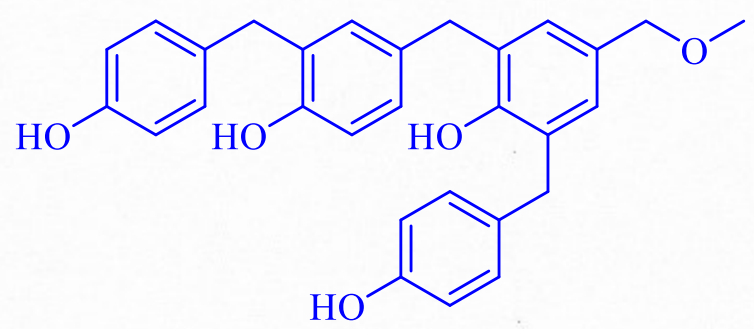

1

page 1

Figure S6. The HR-ESIMS report of compound **1** (page 3)

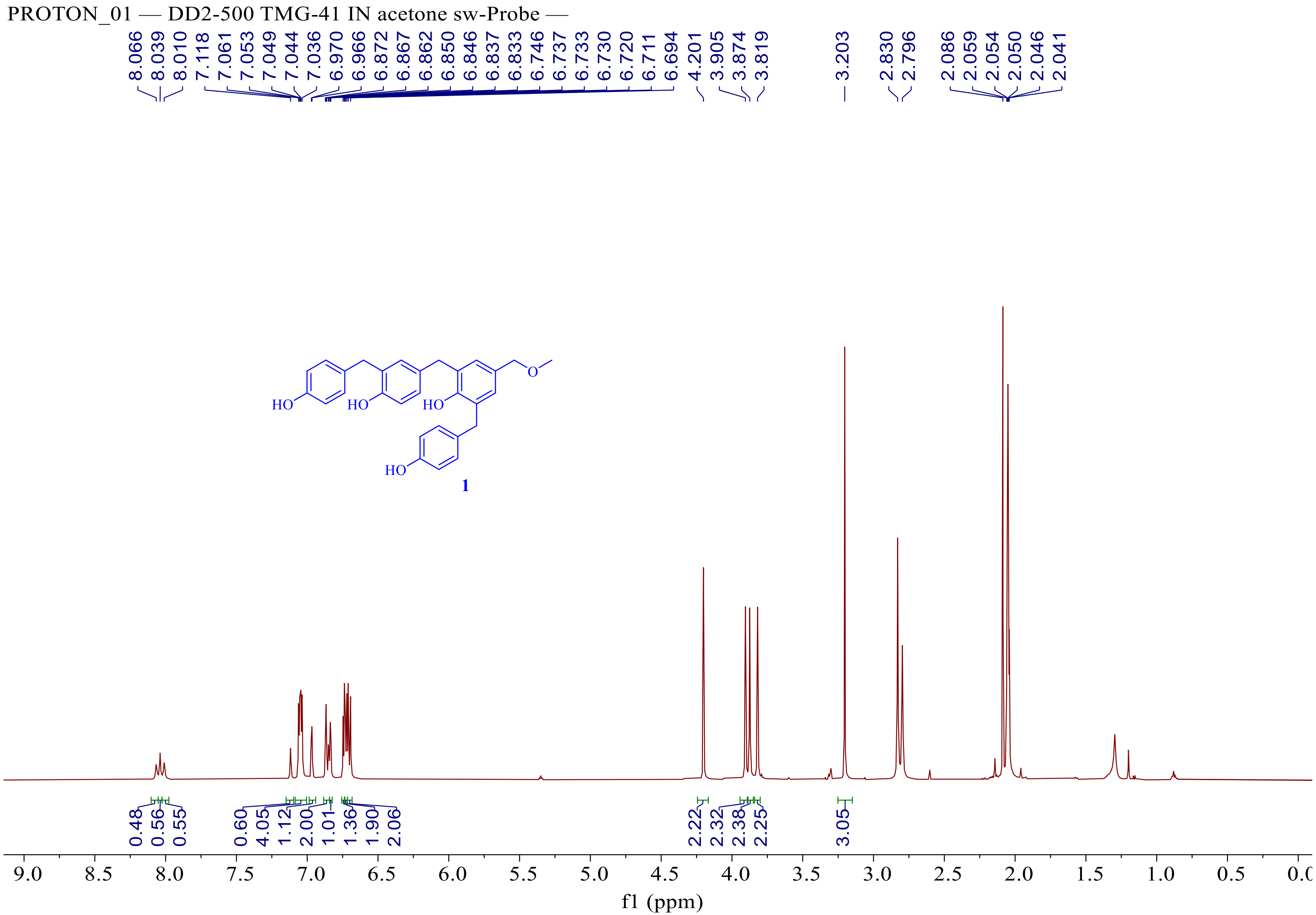

Figure S7. The ^{1}H NMR spectrum of compound **1** in acetone-d_6 (500 MHz)

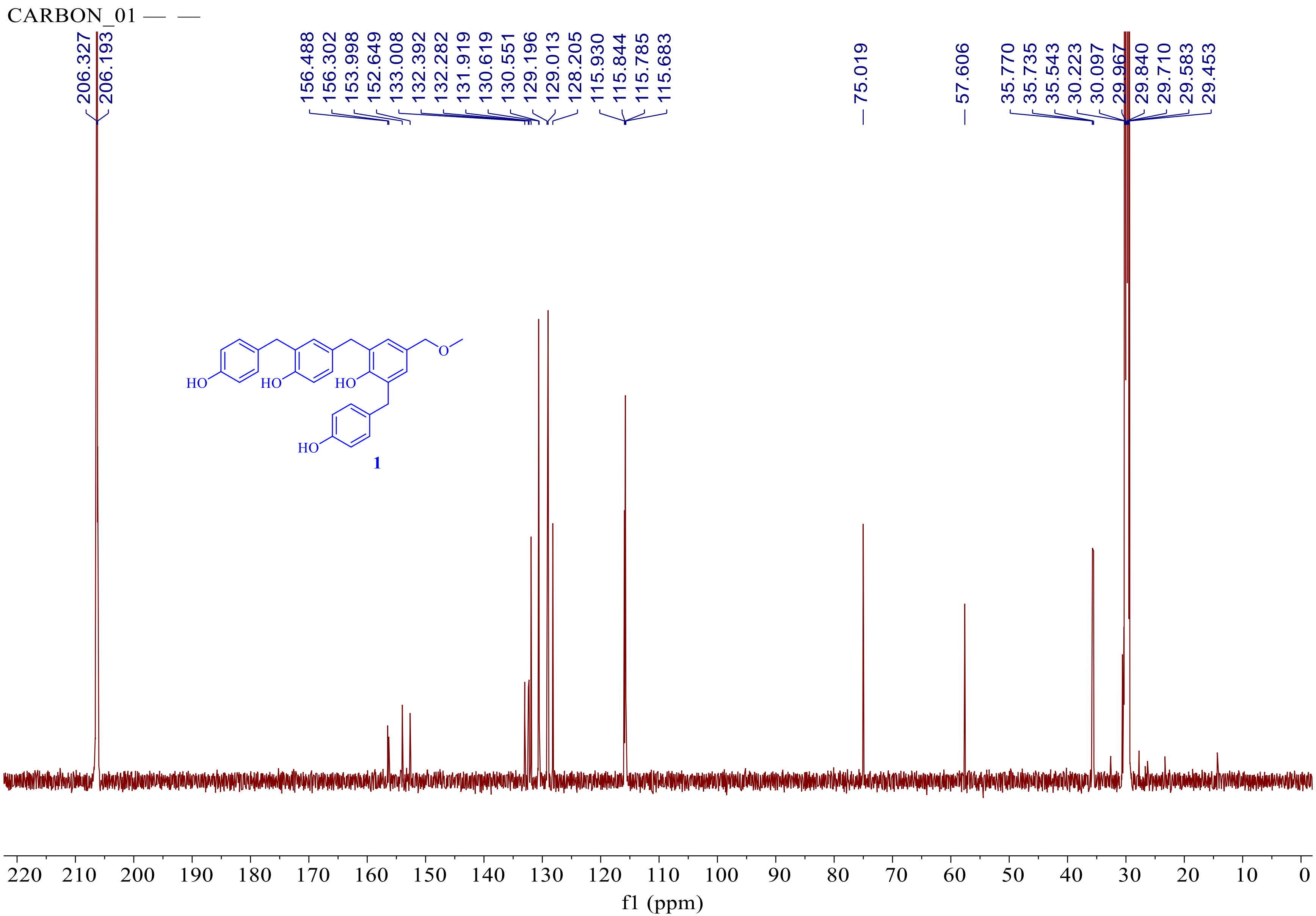

Figure S8. The ^{13}C NMR spectrum of compound **1** in acetone-d_6 (125 MHz)

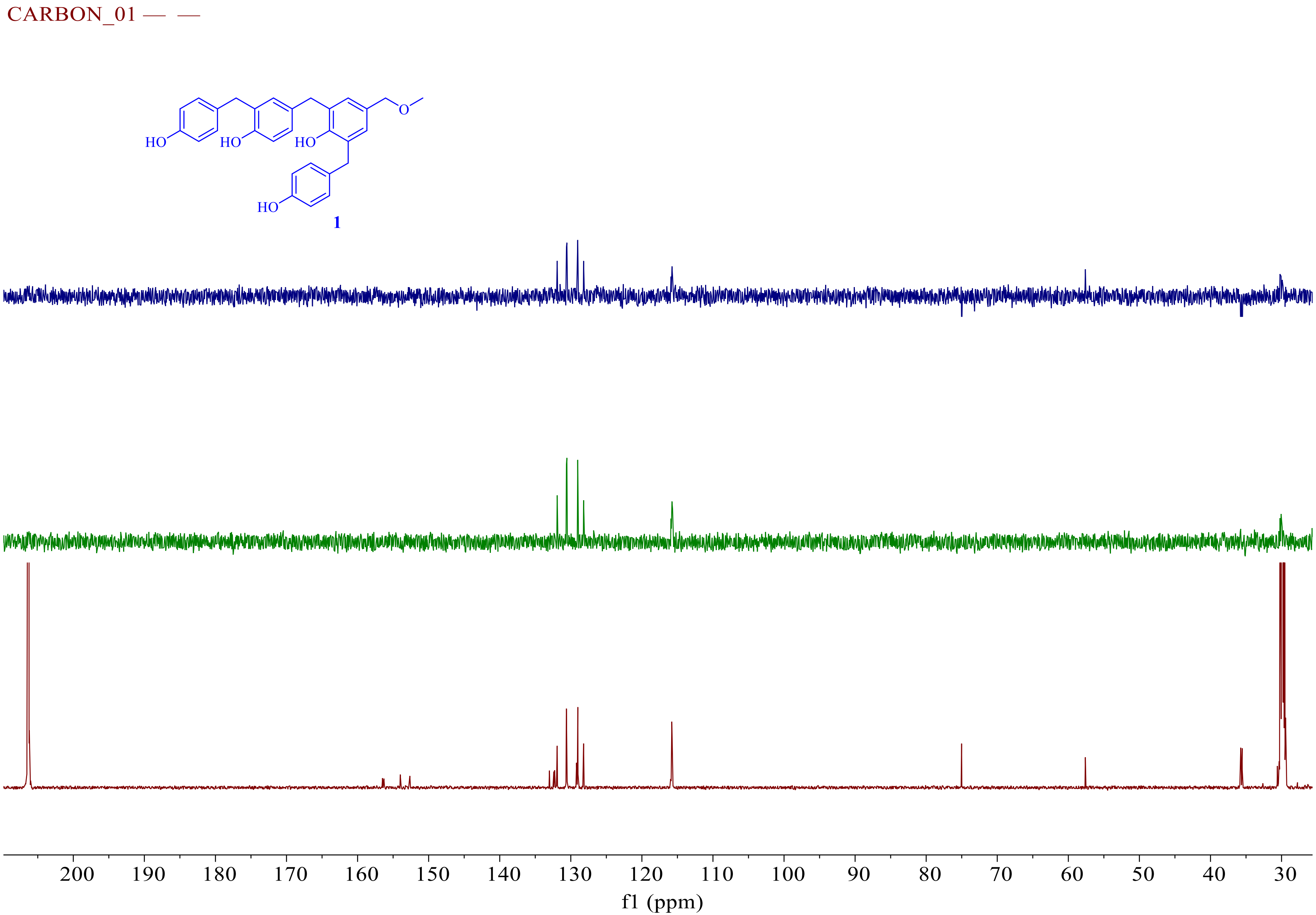

Figure S9. The DEPT spectrum of compound **1** in acetone-d_6 (125 MHz)

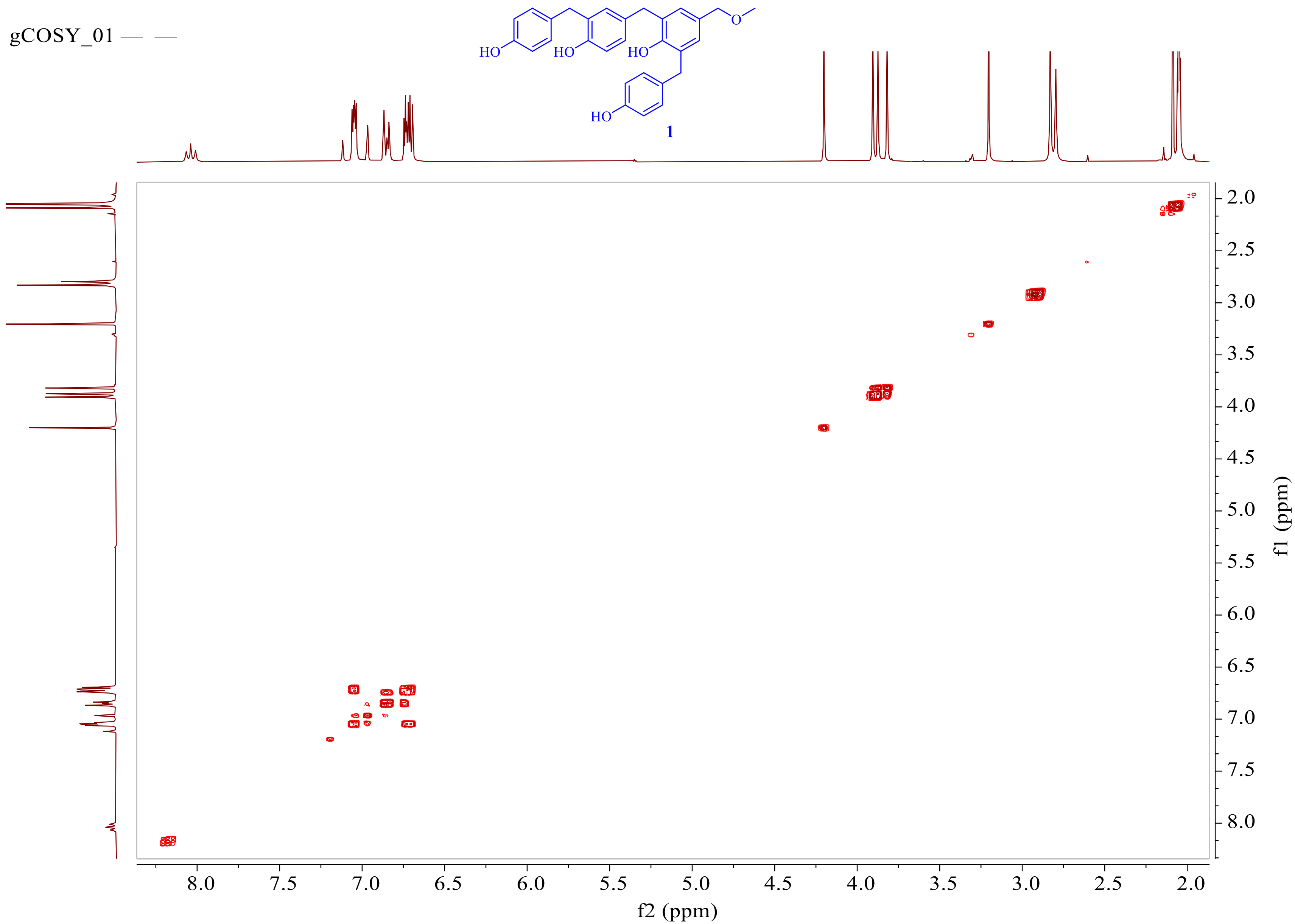

Figure S10. The ^{1}H-^{1}H COSY spectrum of compound **1** in acetone-d_6 (500 MHz)

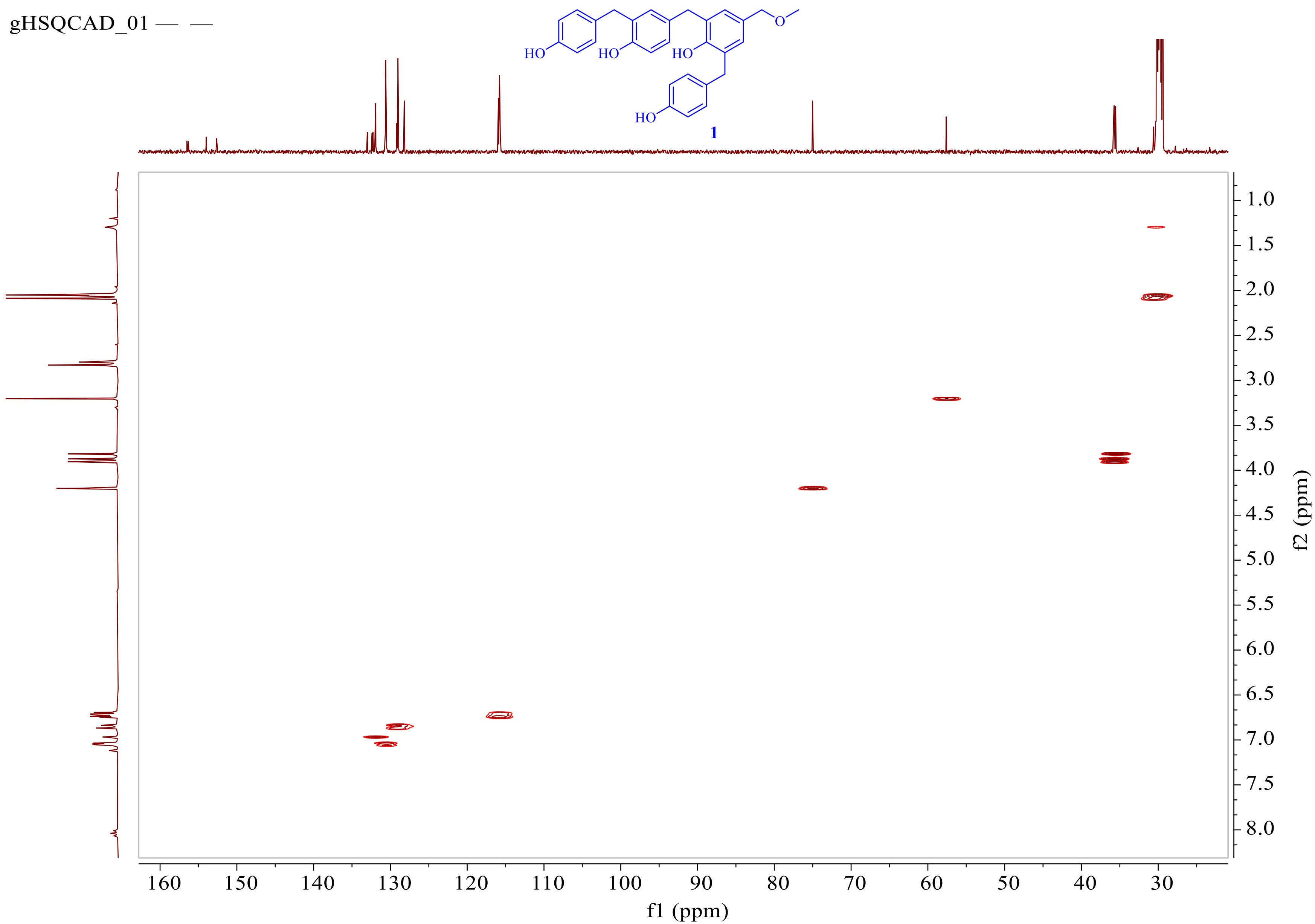

Figure S11. The HSQC spectrum of compound **1** in acetone-d_6 (500 MHz for ^1H)

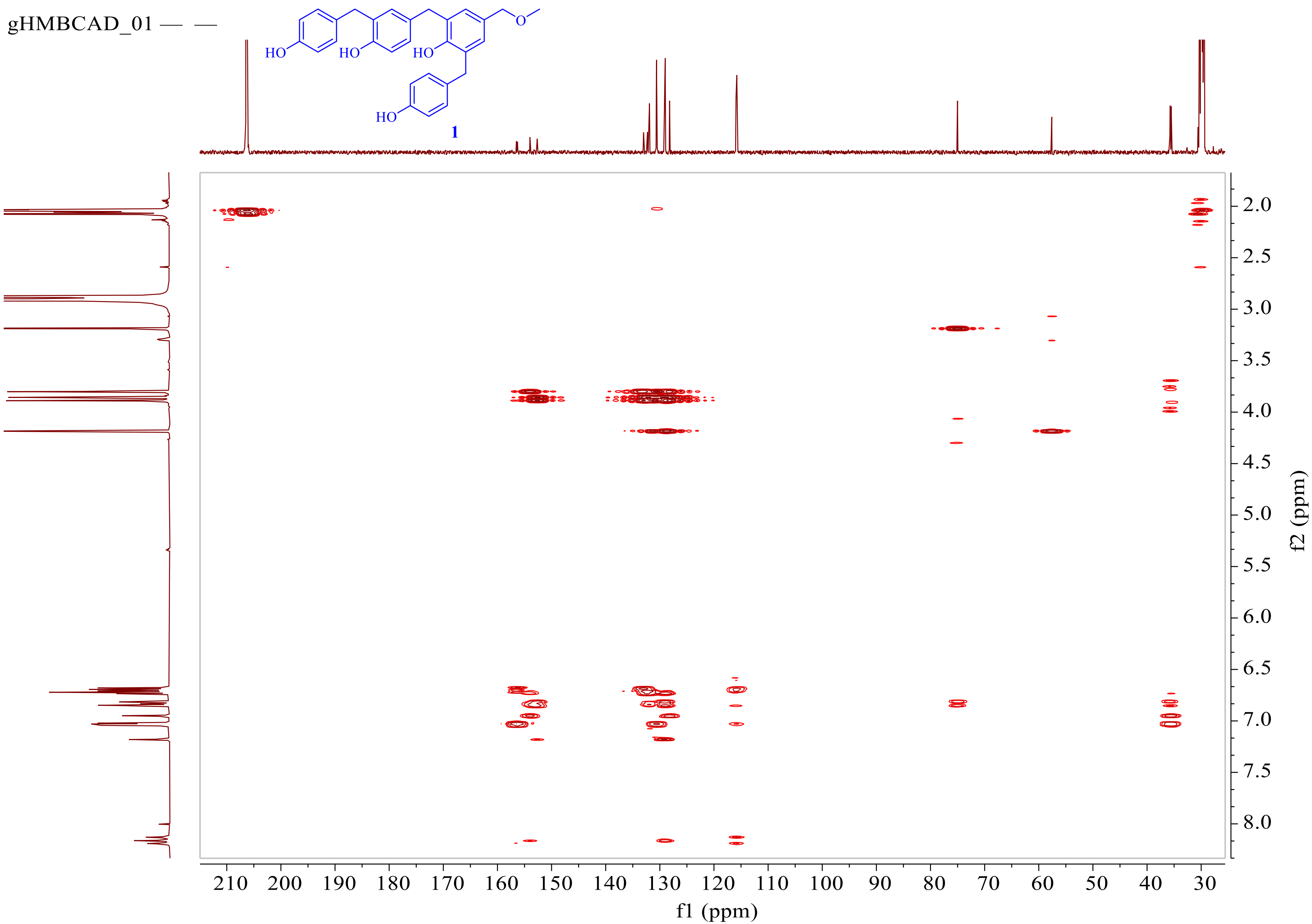

Figure S12. The HMBC spectrum of compound **1** in acetone-d_6 (500 MHz for ^{1}H)

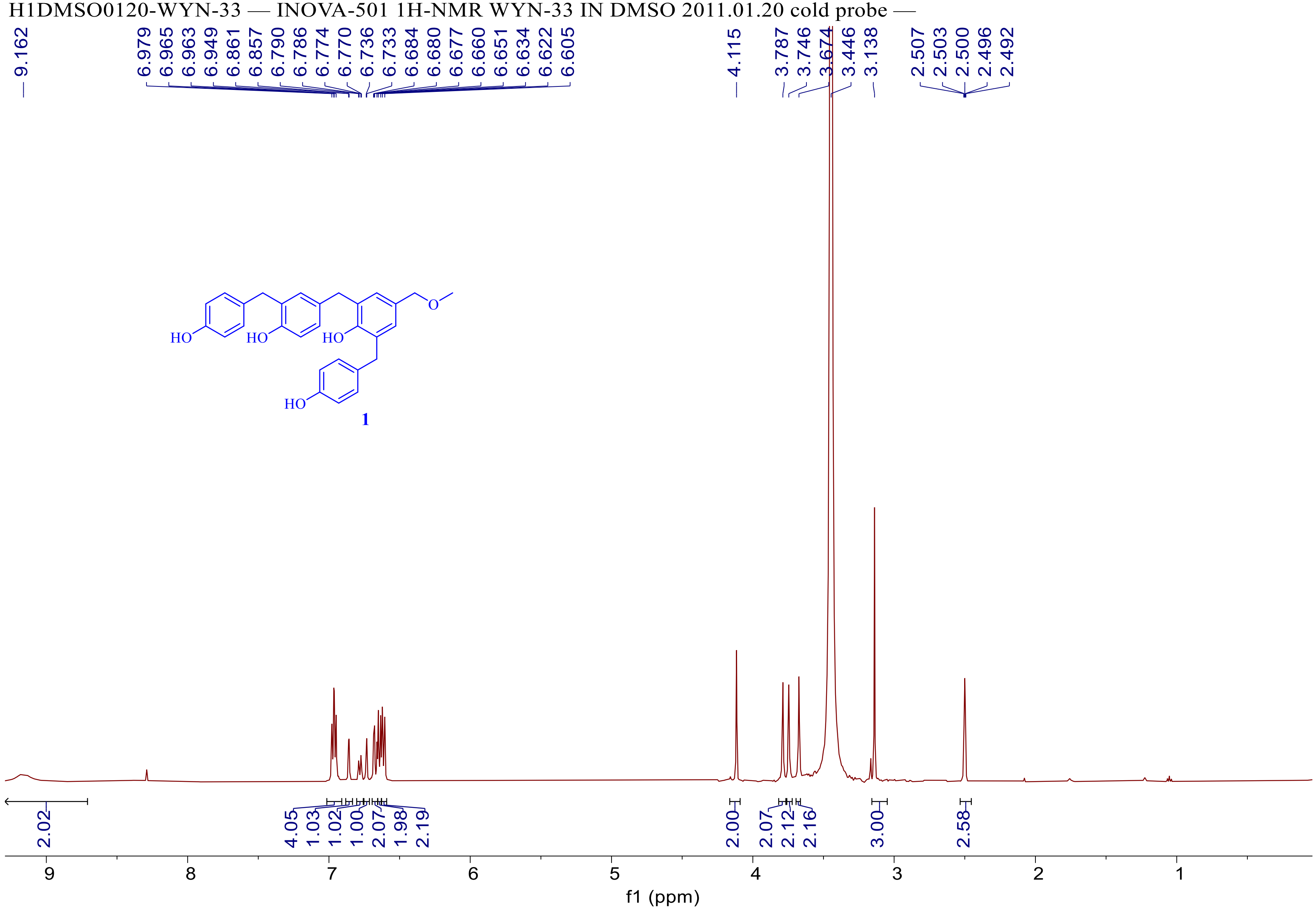

Figure S13. The ^{1}H NMR spectrum of compound **1** in DMSO-d_6 (500 MHz)

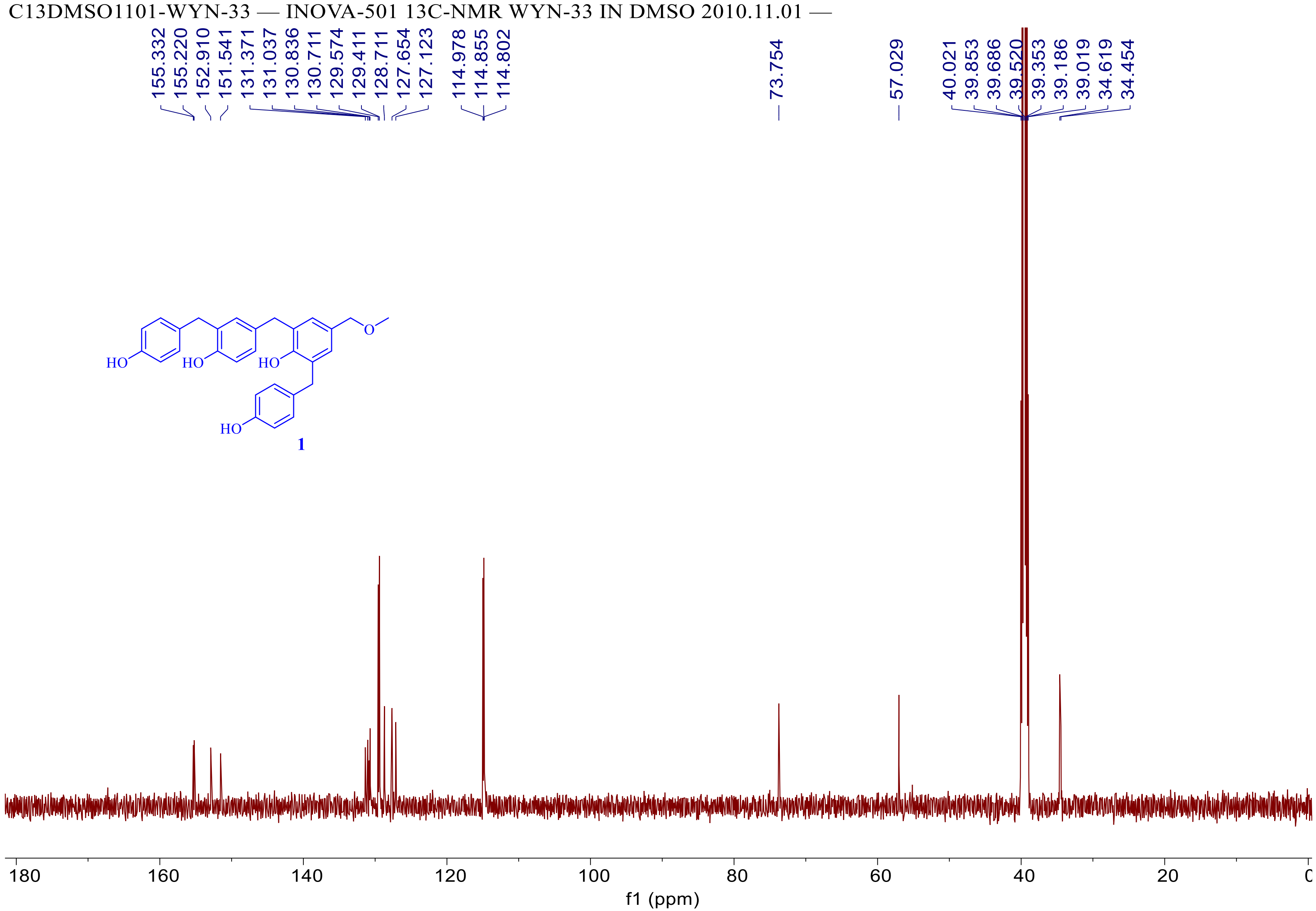

Figure S14. The ^{13}C NMR spectrum of compound **1** in DMSO-d_6 (125 MHz)

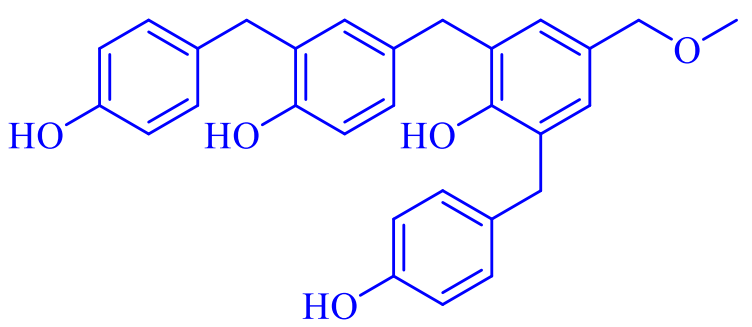

1

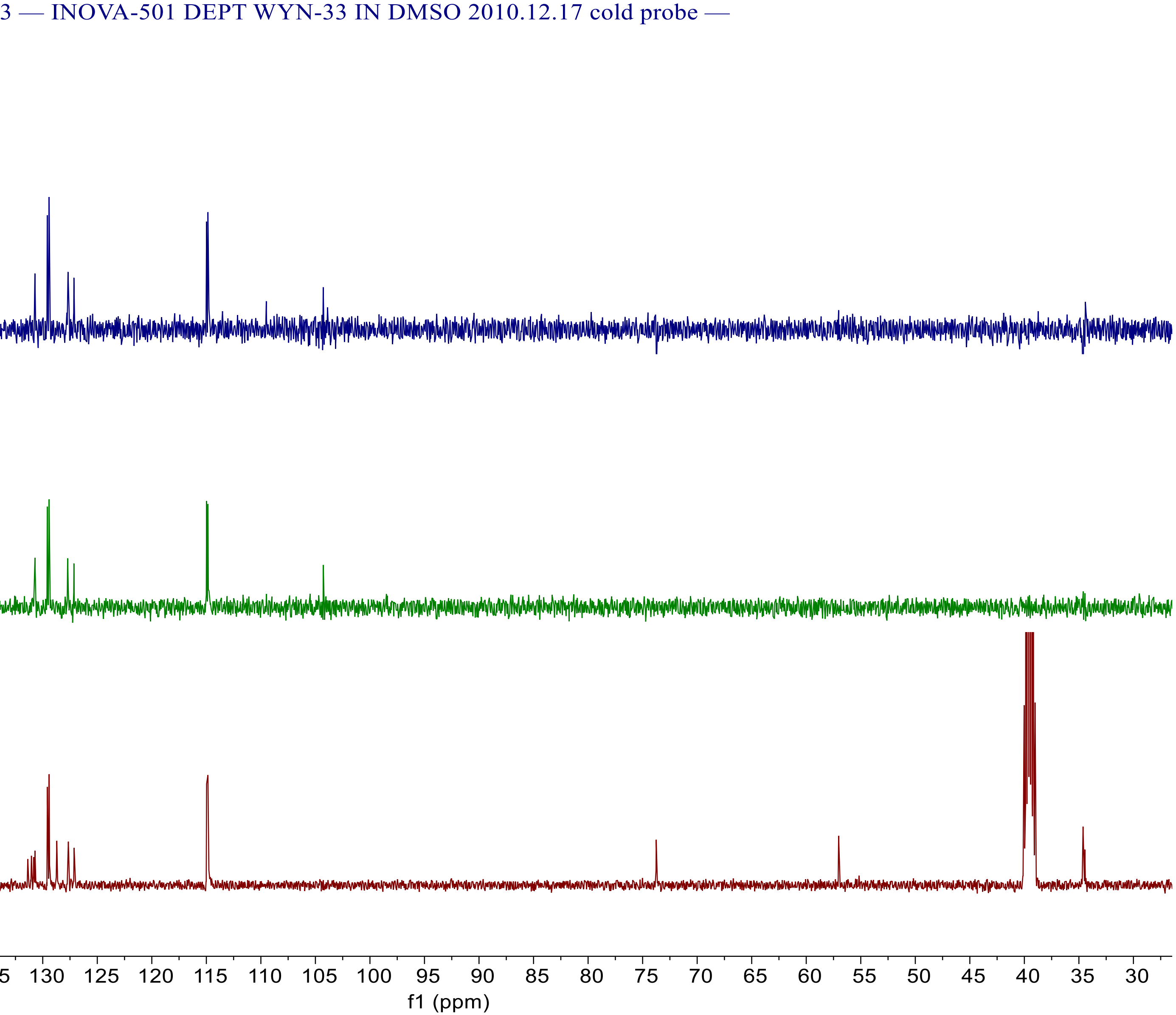

Figure S15. The DEPT spectrum of compound **1** in DMSO-d_6 (125 MHz)

COSYDMSO0120-WYN-33 — INOVA-501 gCOSY WYN-33 IN DMSO 2011.01.20 cold probe —

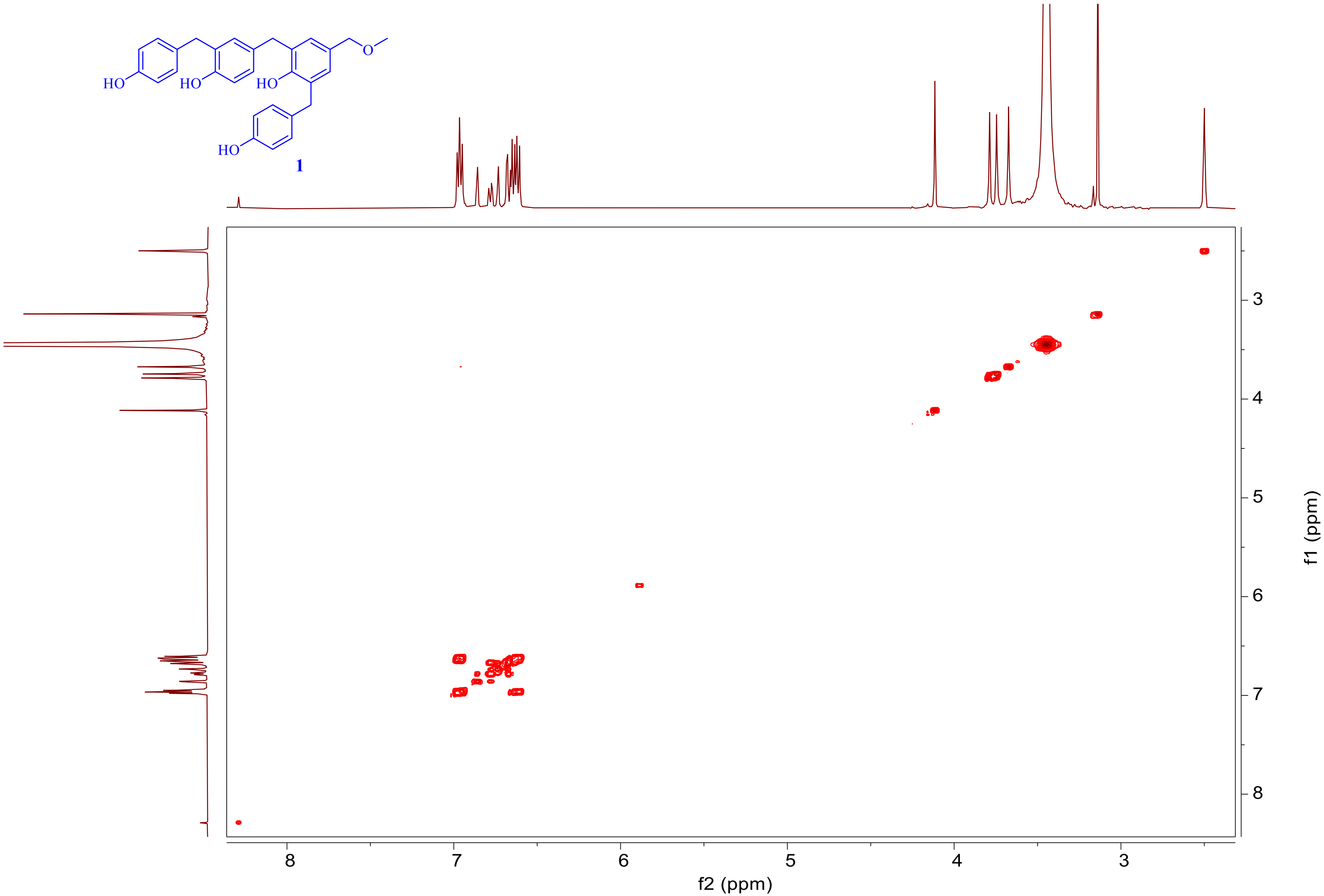

Figure S16. The ^{1}H-^{1}H COSY spectrum of compound **1** in DMSO-d_6 (500 MHz)

HMQCDMSO0120-WYN-33 — INOVA-501 gHMQC WYN-33 IN DMSO 2011.01.20 cold probe —

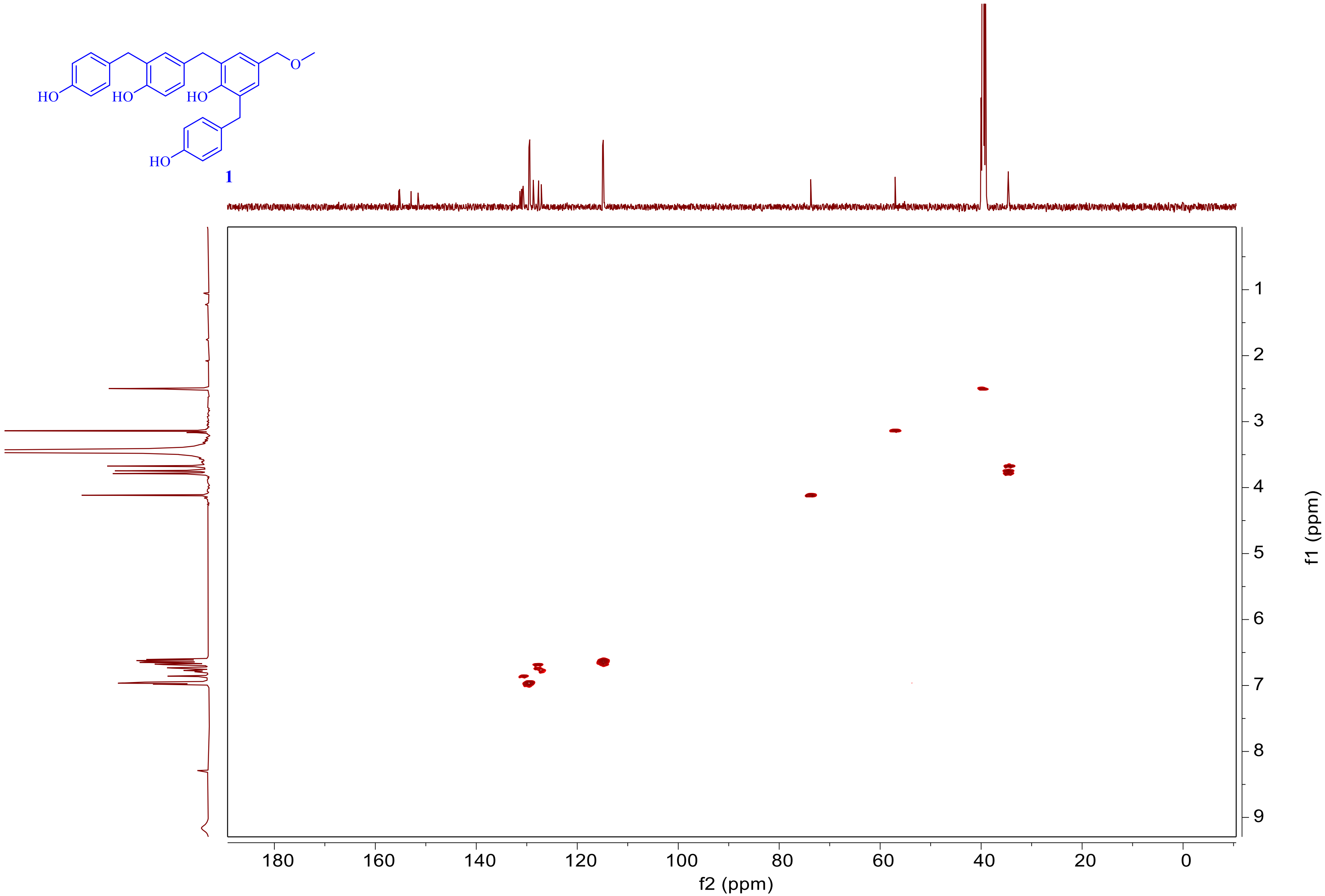

Figure S17. The HMQC spectrum of compound **1** in DMSO-d_6 (500 MHz for ^{1}H)

HMBCDMSO0120-WYN-33 — INOVA-501 gHMBC WYN-33 IN DMSO 2011.01.20 cold probe —

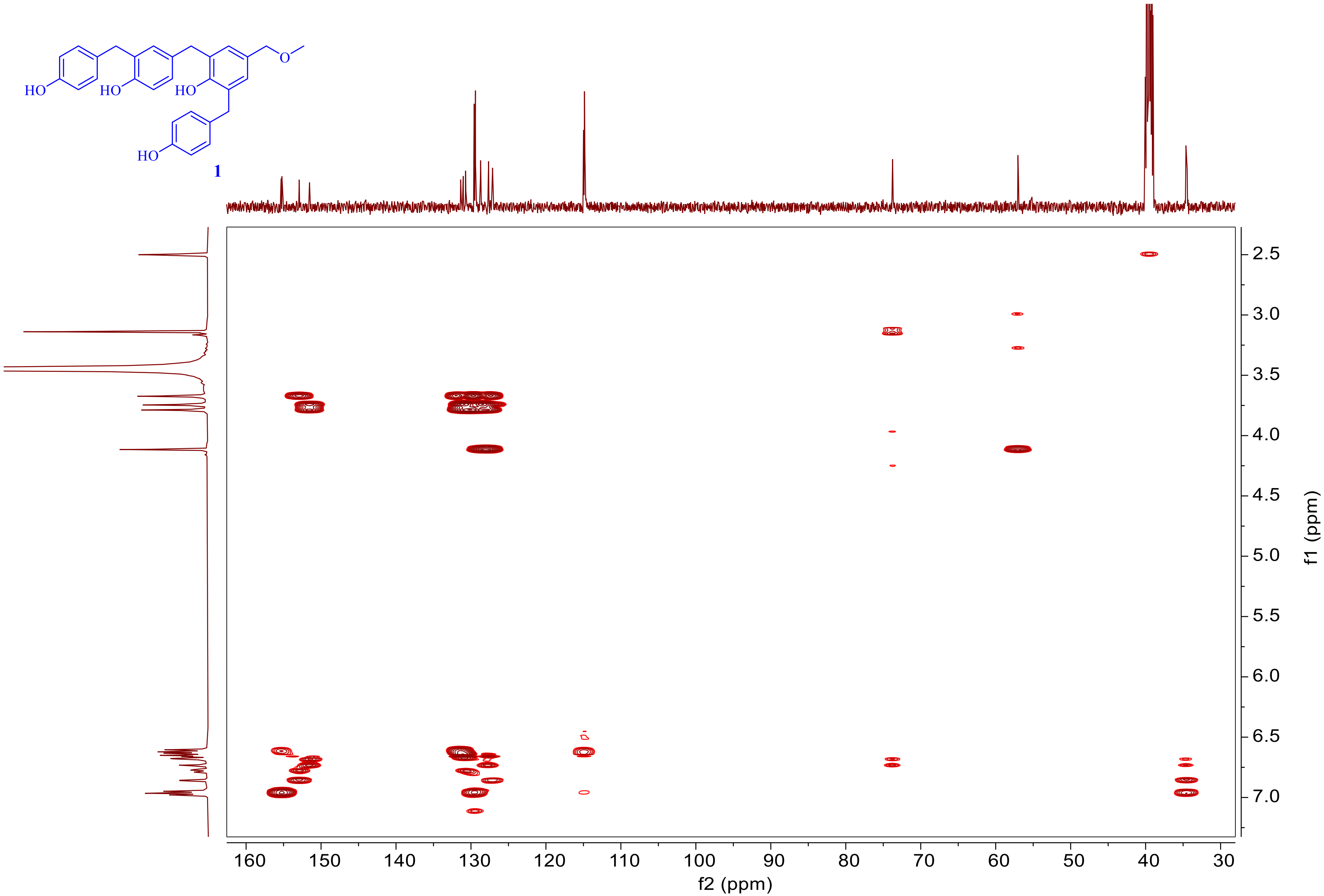

Figure S18. The HMBC spectrum of compound **1** in DMSO-d_6 (500 MHz for ^{1}H)

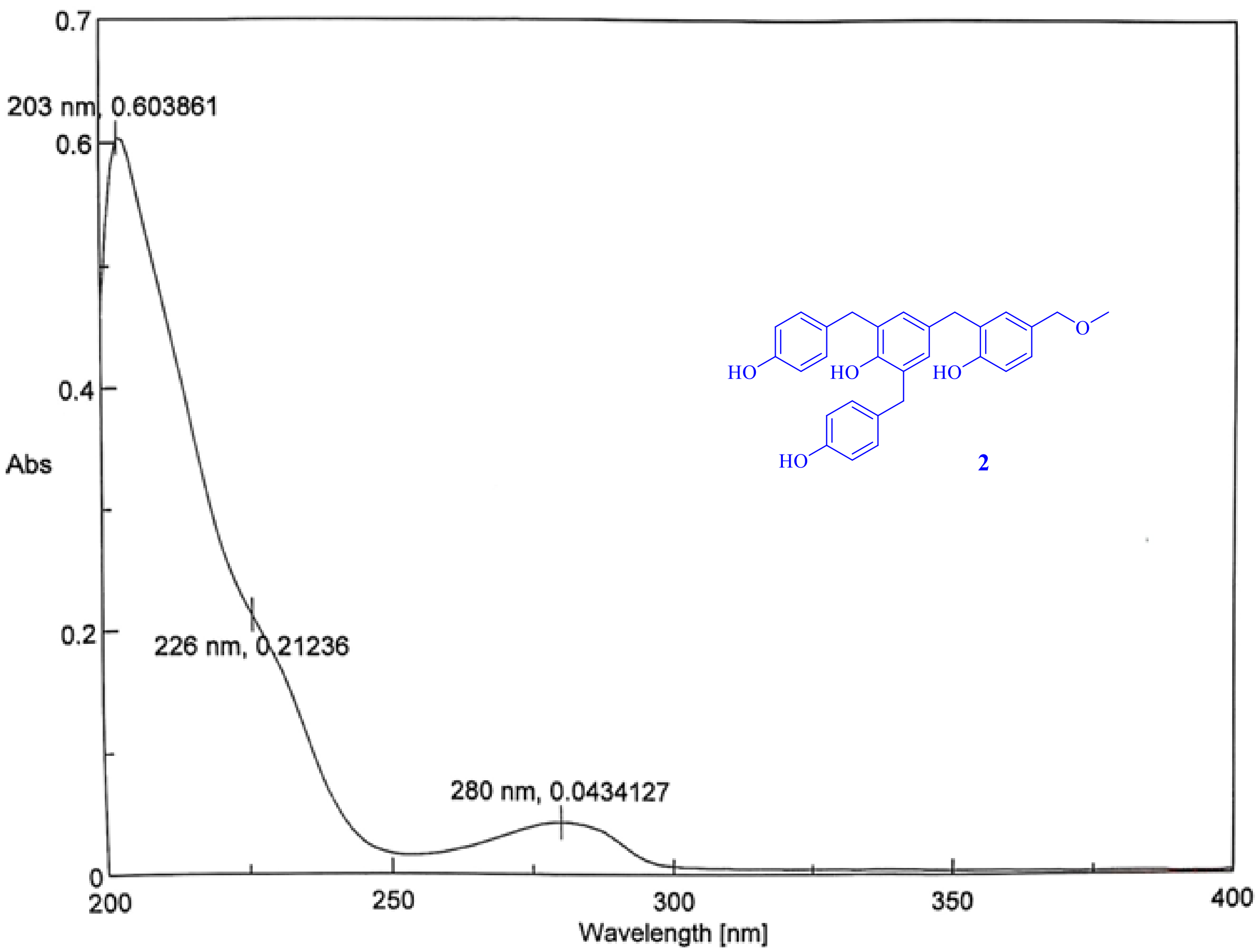

wyn-34-1-w

[Comment]
Sample Name wyn-34
Comment
User 王亚男
Division
Company 324
[Measurement Information]
Instrument Name V-650
Model Name V-650
Serial No. A034461150

Accessory PSC-718
Accessory S/N A001761114
Position 1
Cell Length 10 mm
Temperature 19.98 C
Control Sonsor Holder
Monitor Sensor Holder
Start Mode Start immediately

Photometric Mode Abs
Measurement range 400 - 190 nm
Data pitch 1 nm
Band width(UV/Vis) 1.0 nm
Response Medium
Scanning speed 200 nm/min
Source Change 340 nm
Light Source D2/WI
Filter Exchange Step
Correction Baseline

[Data Information]
Creation Date 2011-5-11 18:30

Data array type Linear data array
Horizontal Wavelength [nm]
Vertical Abs
Start 400 nm
End 190 nm
Data pitch 1 nm
Data points 211

Figure S19. The UV spectrum of compound **2**

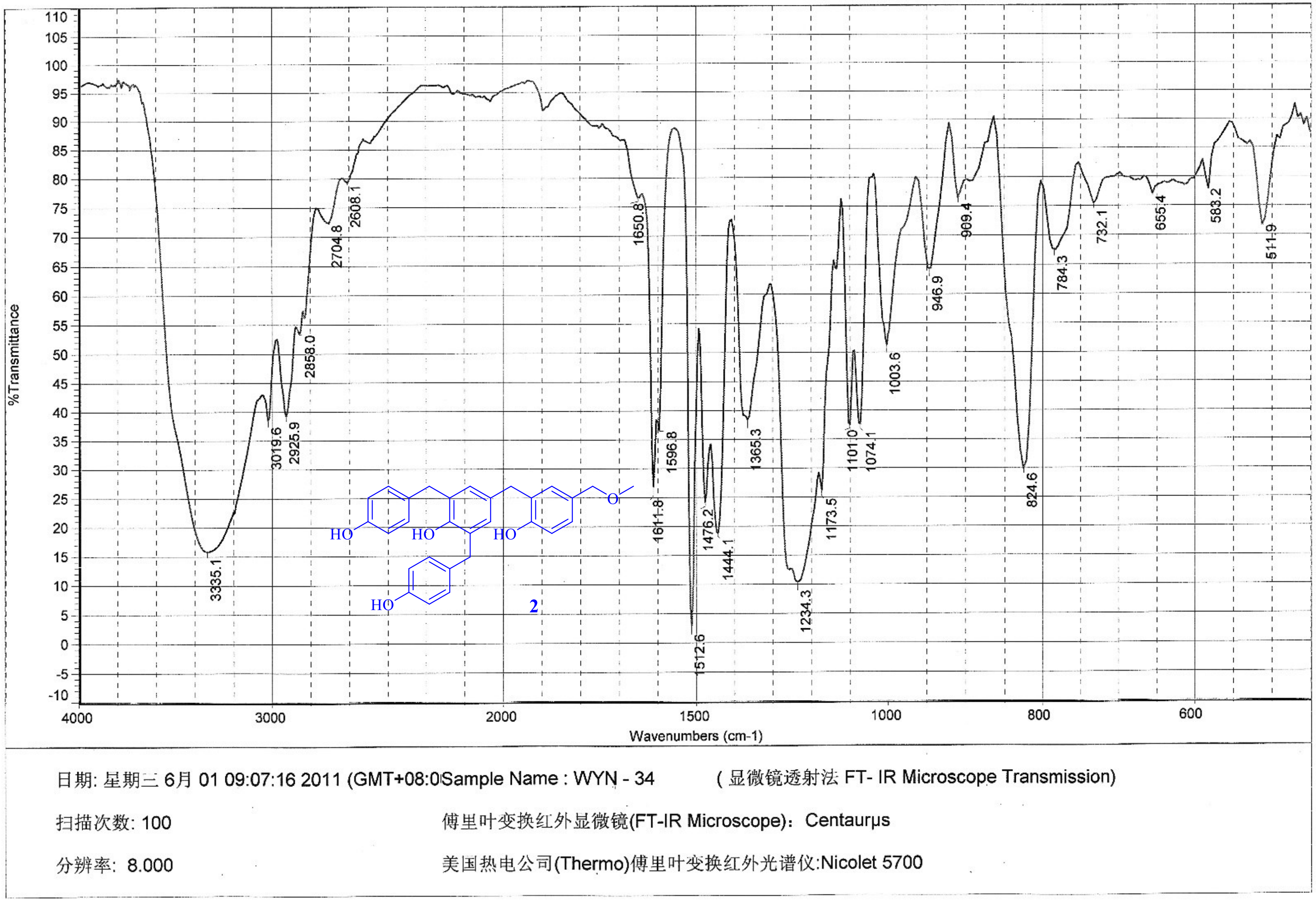

Figure S20. The IR spectrum of compound **2**

Single Mass Spectrum Deconvolution Report

Analysis Name: WANGY007.d **Instrument:** LC-MSD-Trap-SL **Print Date:** 7/9/2010 11:20:05 AM
Method: TEST.MS **Operator:** Operator **Acq. Date:** 7/9/2010 11:17:21 AM
Sample Name: WYN-34
Analysis Info:

Acquisition Parameter:

Mass Range Mode	Std/Normal	Trap Drive	53.0	Scan Begin	100 m/z
Ion Polarity	Positive	Octopole RF Amplitude	171.0 Vpp	Scan End	700 m/z
Ion Source Type	ESI	Capillary Exit	-106.0 Volt	Averages	5 Spectra
Dry Temp (Set)	330 °C	Skimmer	-40.0 Volt	Max. Accu Time	200000 µs
Nebulizer (Set)	15.00 psi	Oct 1 DC	-12.00 Volt	ICC Target	20000
Dry Gas (Set)	5.00 l/min	Oct 2 DC	-1.70 Volt	Charge Control	on

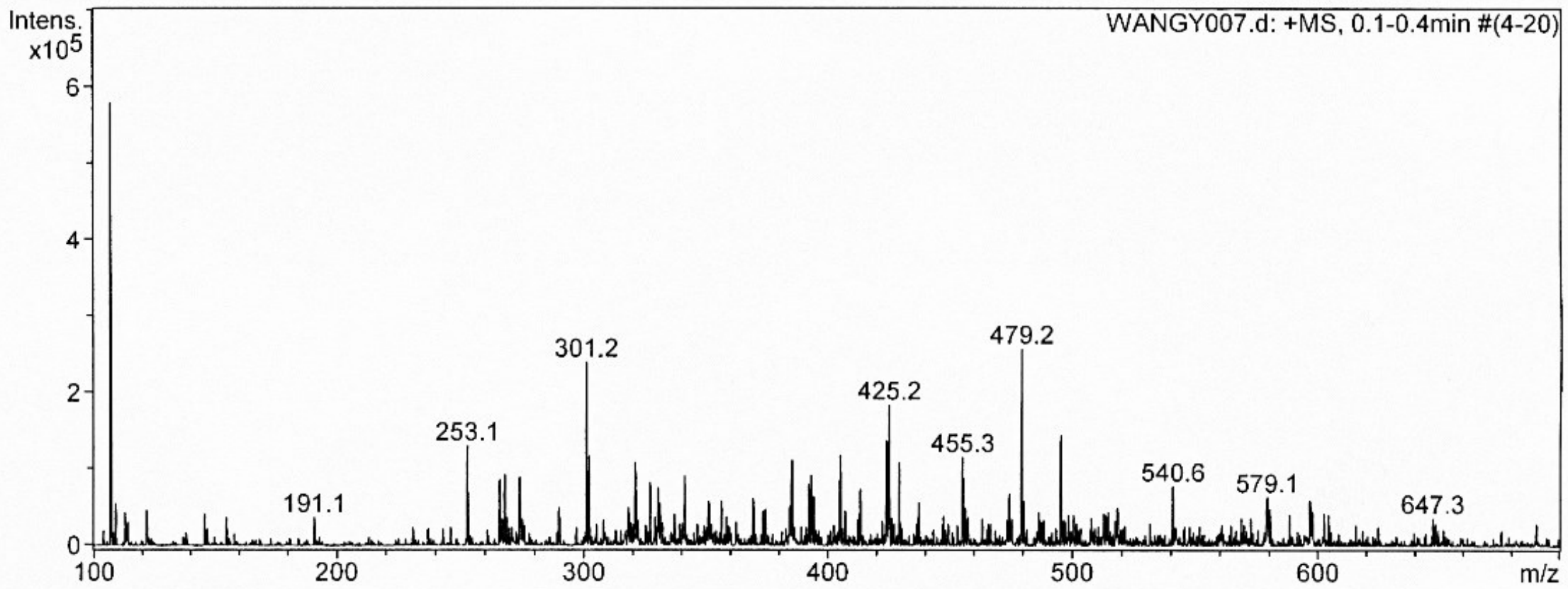

Component	Molecular Mass	Molecule	Absolute Abundance	Relative Abundance

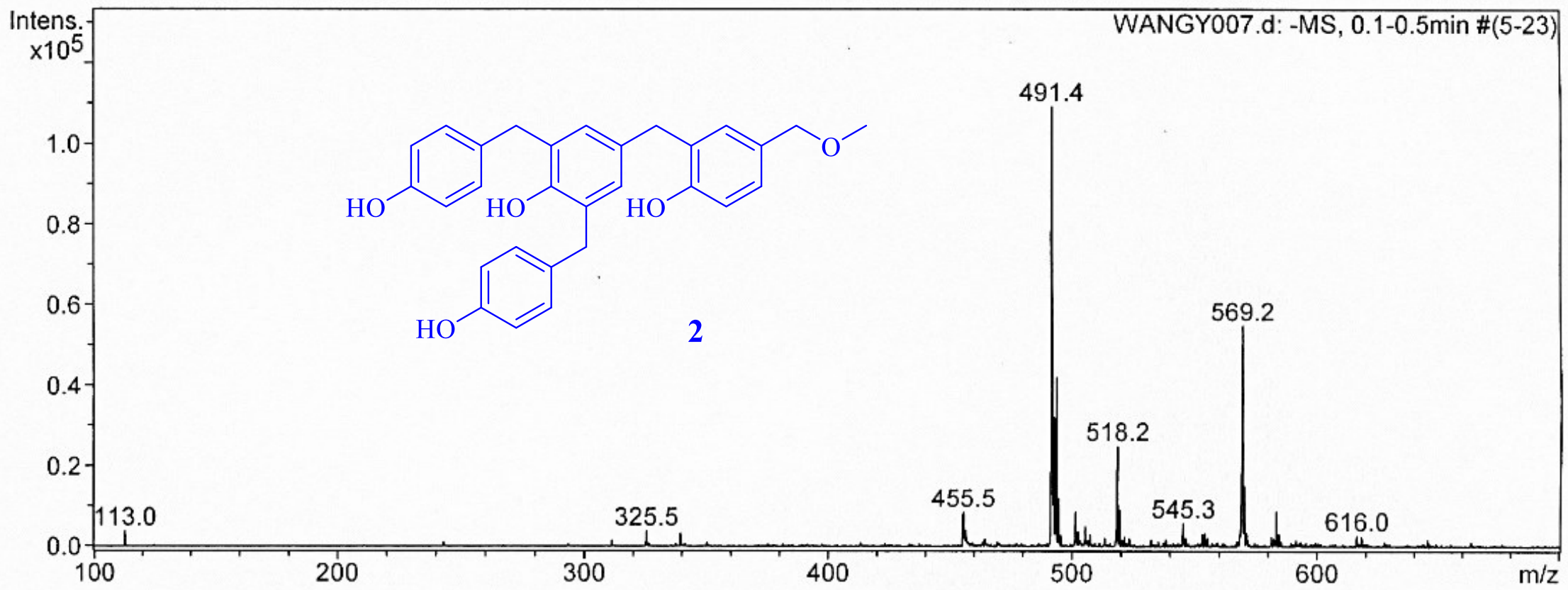

Component	Molecular Mass	Molecule	Absolute Abundance	Relative Abundance

MSD Trap Report v 4 (A4-Opt2) Page 1 of 1 Agilent Technologies

Figure S21. The ESIMS of compound **2**

Qualitative Analysis Report

**Data Filename**	2011011712.d	**Sample Name**	WYN-34
**Sample Type**	Sample	**Position**	P1-D5
**Instrument Name**	Instrument 1	**User Name**	
**Acq Method**		**IRM Calibration Status**	Success
**DA Method**	TEST LCMS.m	**Comment**	

User Chromatograms

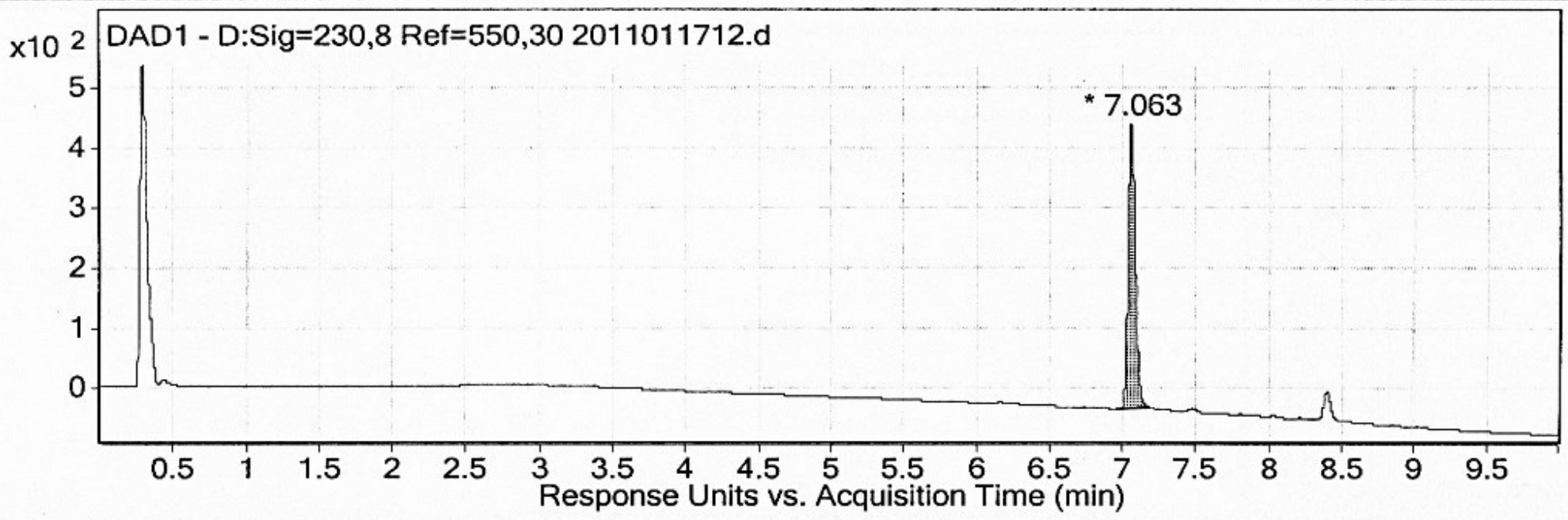

Integration Peak List

Peak	Start	RT	End	Height	Area	Area %
1	6.956	7.063	7.188	472.54	1573.52	100

Fragmentor Voltage 135 **Collision Energy** 0 **Ionization Mode** ESI

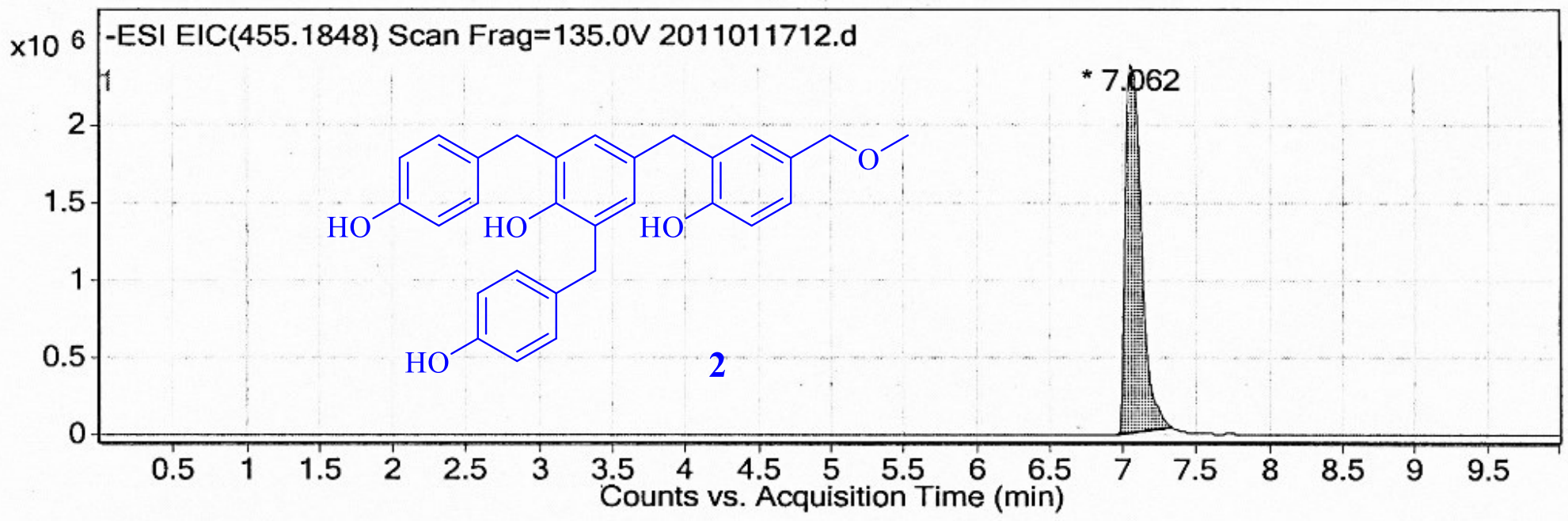

Integration Peak List

Peak	Start	RT	End	Height	Area	Area %
1	6.95	7.062	7.336	2369089	17349233	100

User Spectra

Fragmentor Voltage	Collision Energy	Ionization Mode
135	0	Esi

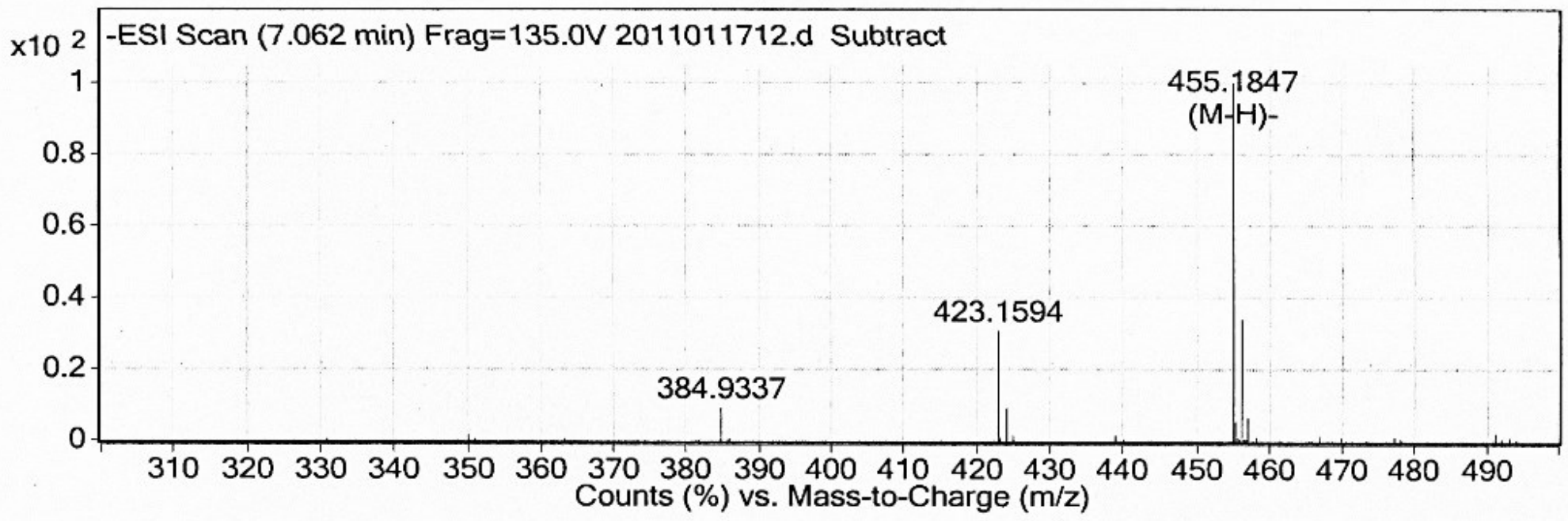

Figure S22. The HR-ESIMS report of compound **2** (page 1)

Qualitative Analysis Report

Peak List

m/z	z	Abund	Formula	Ion
423.1594	1	730337		
455.1847	1	2382884	C29 H27 O5	(M-H)-
456.1893	1	813377	C29 H27 O5	(M-H)-
520.9093		850716		
537.1885	1	473684		
656.8838		489472		
911.3778	1	1861240		
912.3817	1	1248523		
913.3855	1	461016		
933.3612	1	544234		

Formula Calculator Element Limits

Element	Min	Max
C	3	100
H	0	120
O	0	30
N	0	2
S	0	0
Cl	0	0

Formula Calculator Results

Formula	Best	Mass	Tgt Mass	Diff (ppm)	Ion Species	Score
C29 H28 O5	TRUE	456.1921	456.1937	3.55	C29 H27 O5	99.6
C24 H28 N2 O7		456.1921	456.1897	-5.28	C24 H27 N2 O7	98.43
C36 H24		456.1921	456.1878	-9.32	C36 H23	98.16
C17 H32 N2 O12		456.1921	456.1955	7.59	C17 H31 N2 O12	94.38

--- End Of Report ---

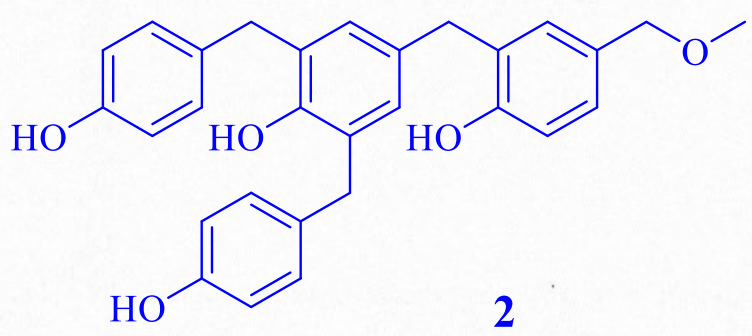

2

Figure S23. The HR-ESIMS report of compound **2** (page 2)

MS Formula Results: - Scan (7.062 min) Sub (2011011712.d)

m/z	Ion	Formula	Abundance
455.1847	(M-H)-	C29 H27 O5	2382883.5

Best	Formula (M)	Ion Formula	Calc m/z	Score	Cross S	Mass	Calc Mass	Diff (ppm)	Abs Diff (ppm)	Abund Match	Spacing Mat	Mass Match	m/z	DBE
☑	C29 H28 O5	C29 H27 O5	455.1864	99.6		456.1921	456.1937	3.55	3.55	99.48	99.82	99.57	455.1847	16
☐	C24 H28 N2 O7	C24 H27 N2 O7	455.1824	98.43		456.1921	456.1897	-5.28	5.28	96.28	99.75	99.06	455.1847	12
☐	C36 H24	C36 H23	455.1805	98.16		456.1921	456.1878	-9.32	9.32	98.55	99.82	97.09	455.1847	25
☐	C17 H32 N2 O12	C17 H31 N2 O12	455.1882	94.38		456.1921	456.1955	7.59	7.59	83.82	99.69	98.06	455.1847	3

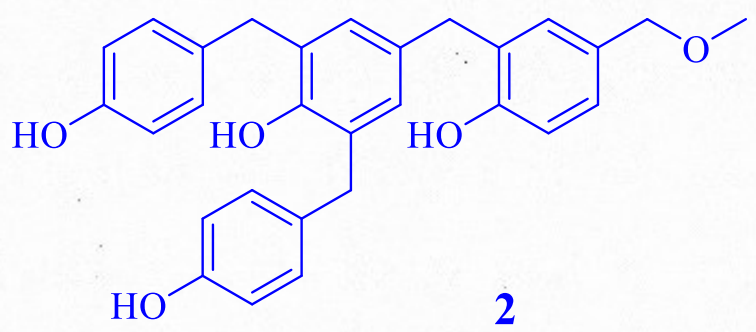

2

page 1

Figure S24. The HR-ESIMS report of compound **3** (page 3)

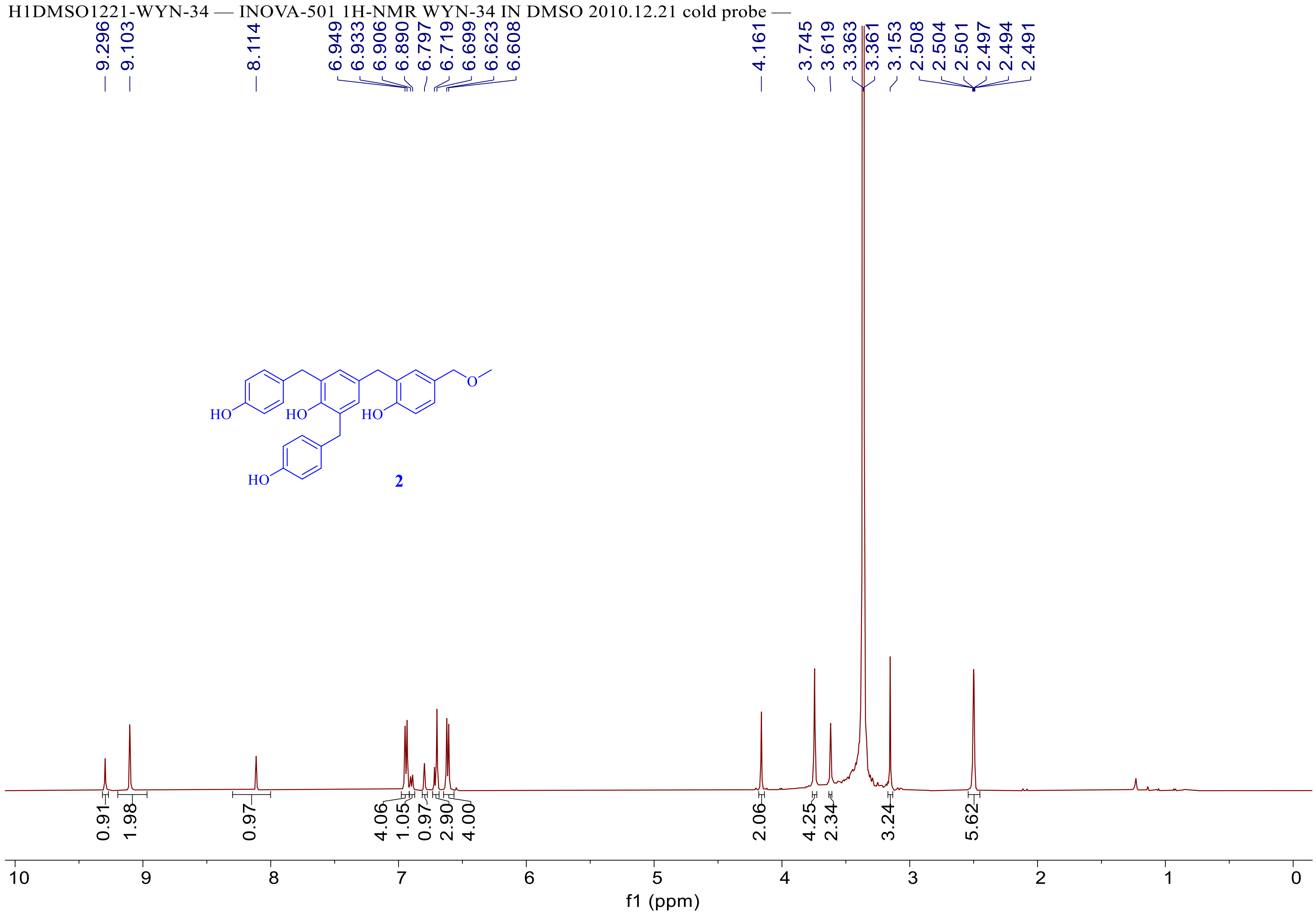

Figure S25. The ^{1}H NMR spectrum of compound **2** in DMSO-d_6 (500 MHz)

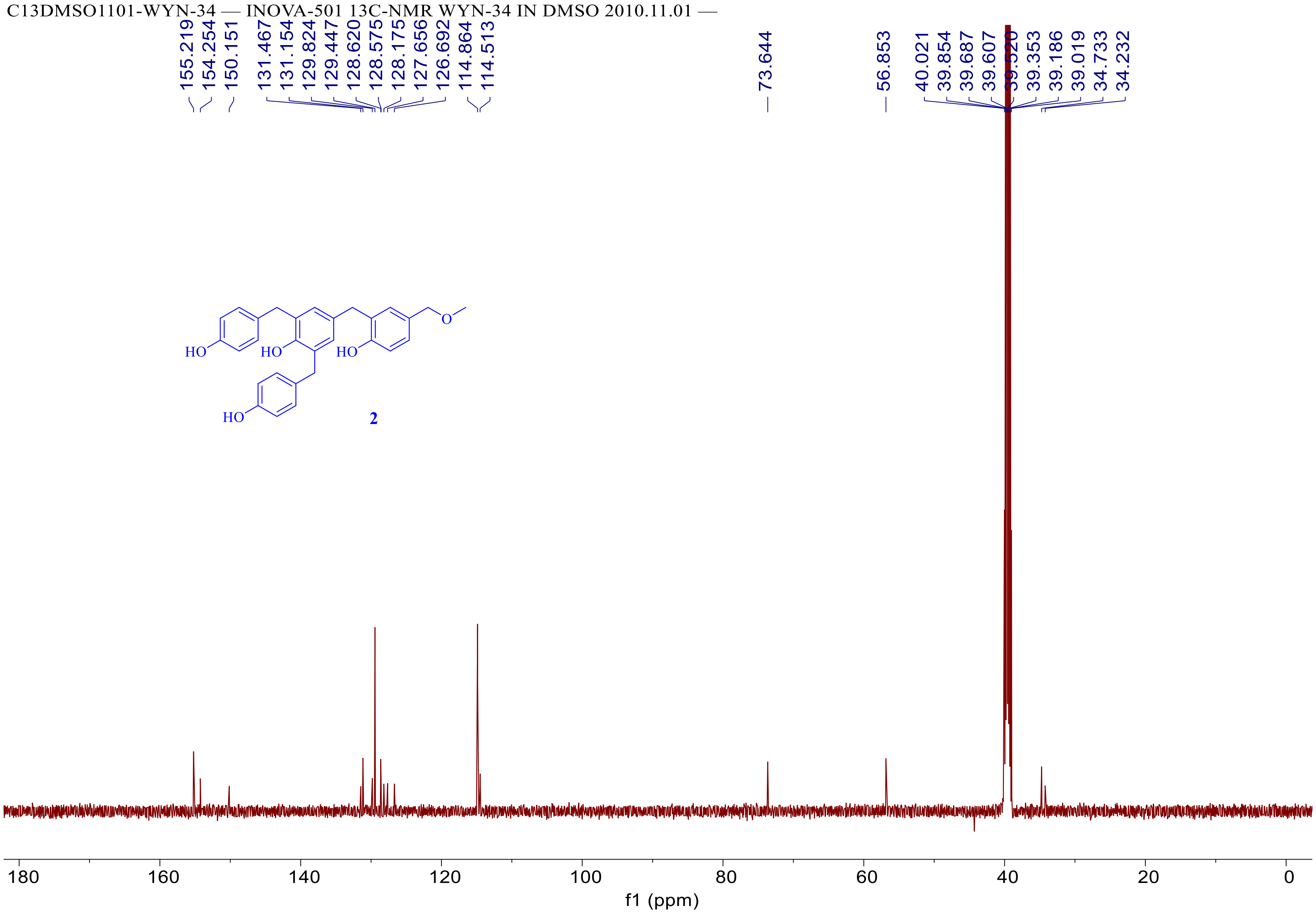

Figure S26. The ^{13}C NMR spectrum of compound **2** in DMSO-d_6 (125 MHz)

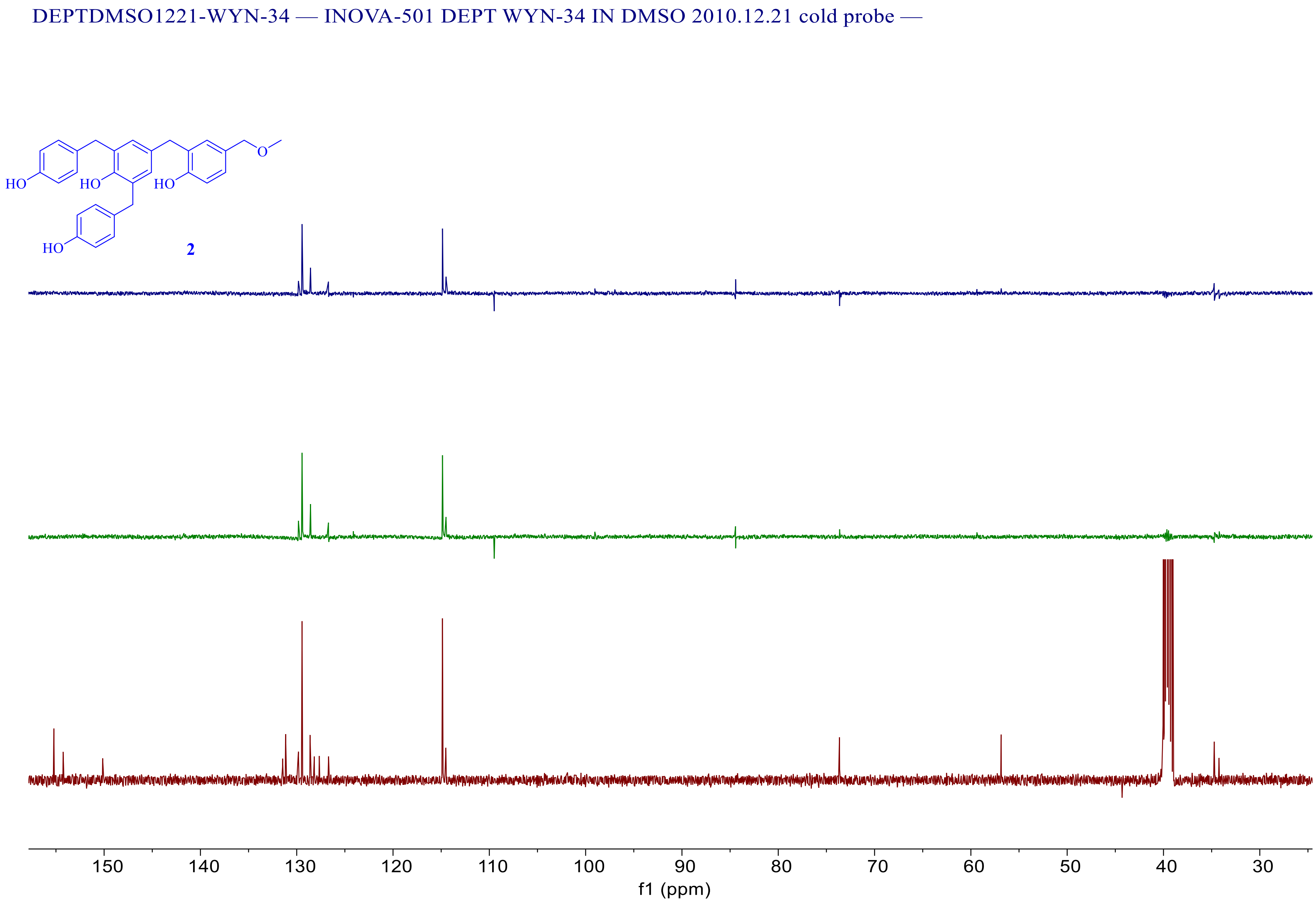

Figure S27. The DEPT spectrum of compound **2** in DMSO-d_6 (125 MHz)

COSYDMSO1221-WYN-34 — INOVA-501 gCOSY WYN-34 IN DMSO 2010.12.21 cold probe —

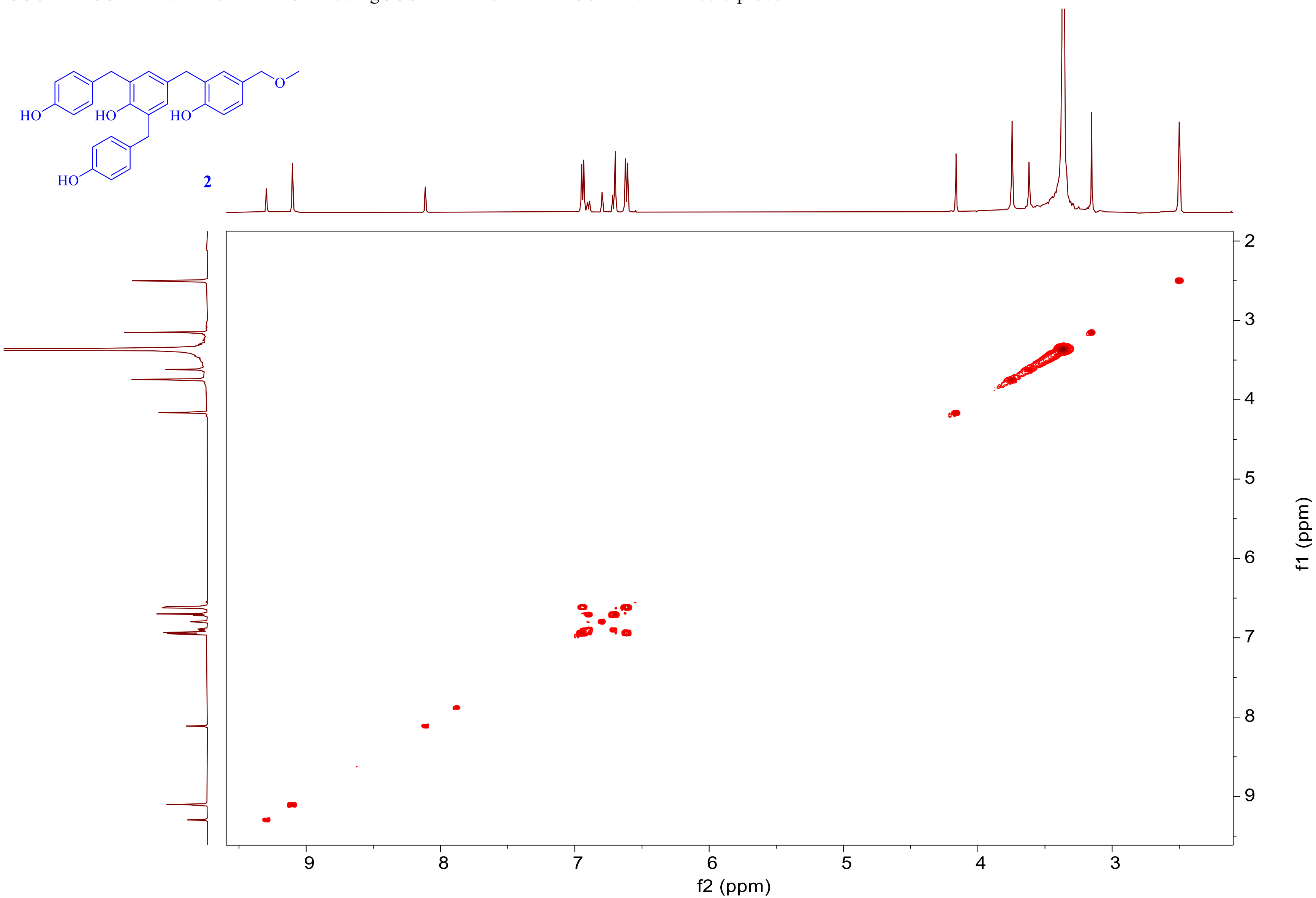

Figure S28. The ^{1}H-^{1}H COSY spectrum of compound **2** in DMSO-d_6 (500 MHz)

HSQCDMSO1122-WYN-34 — INOVA-501 gHSQC WYN-34 IN DMSO 2010.11.22 —

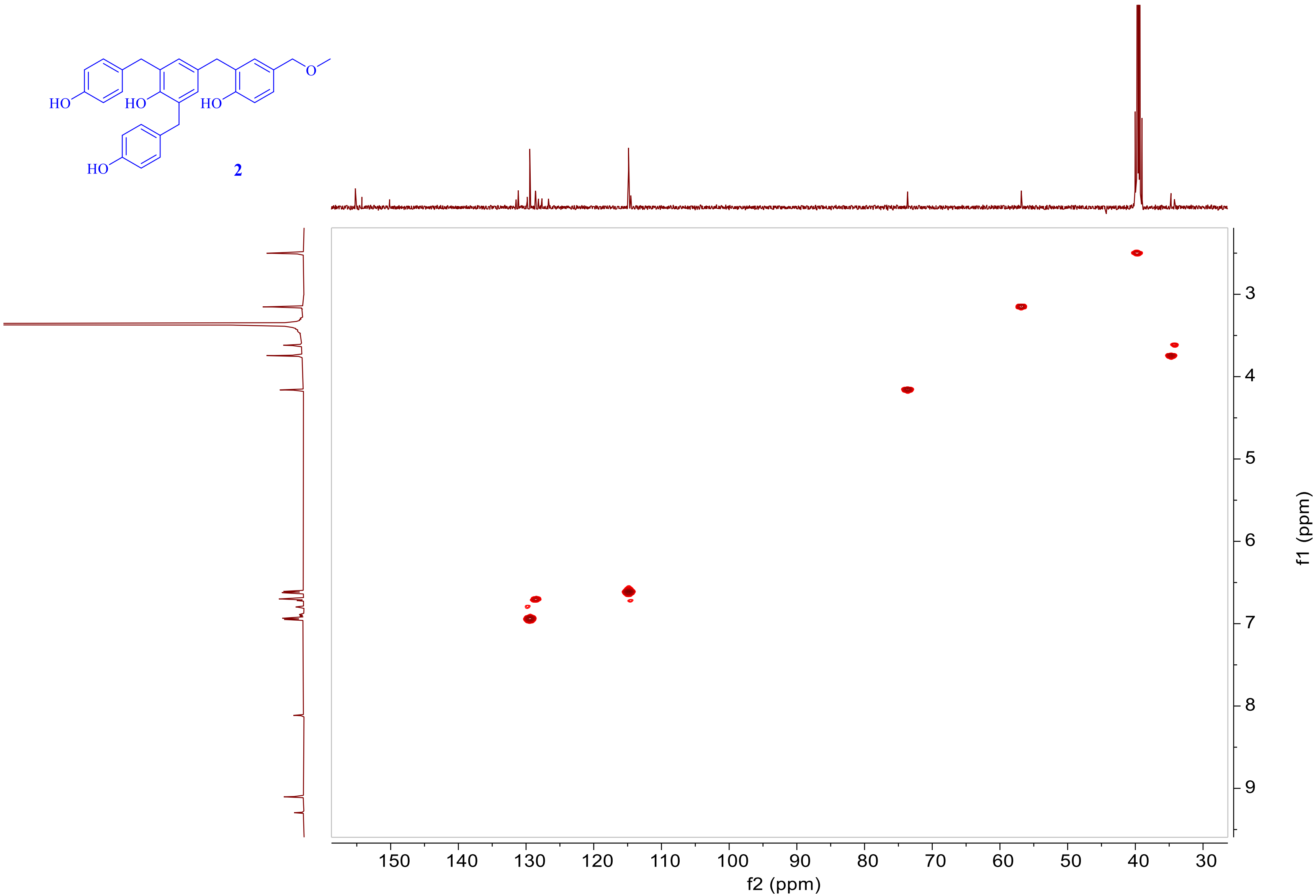

2

Figure S29. The HSQC spectrum of compound **2** in DMSO-d_6 (500 MHz for ^{1}H)

HMBCDMSO1122-WYN-34 — INOVA-501 gHMBC WYN-34 IN DMSO 2010.11.22 —

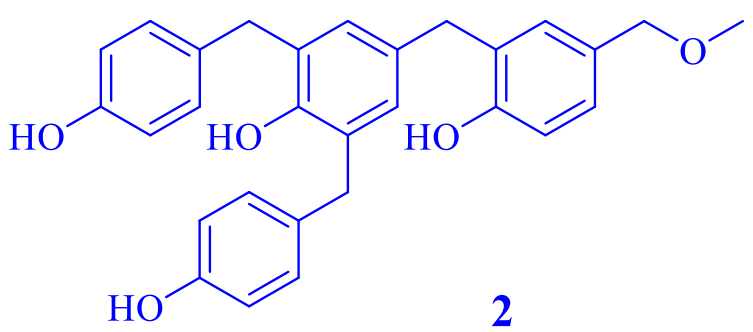

2

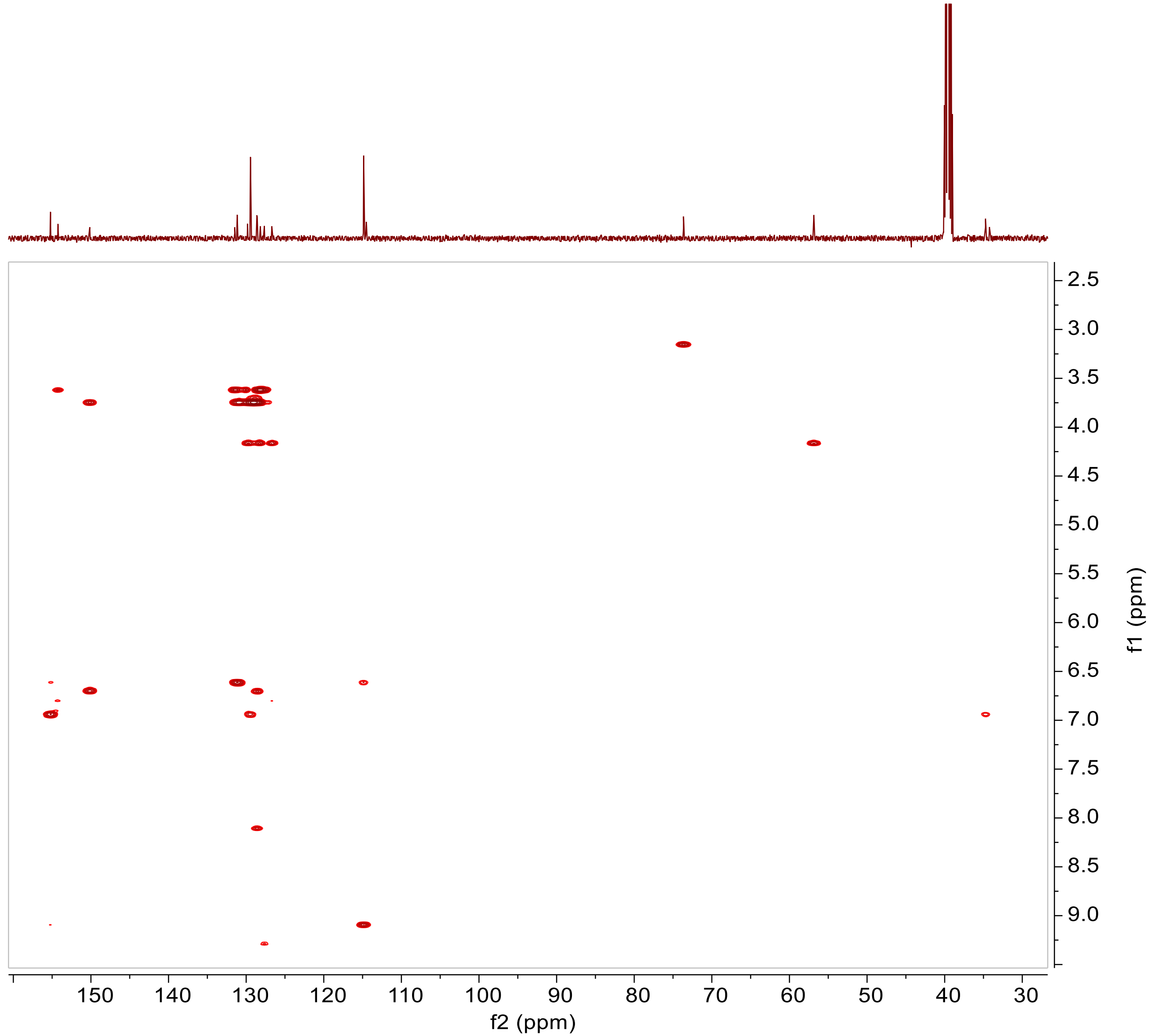

Figure S30. The HMBC spectrum of compound **2** in DMSO-d_6 (500 MHz for ^{1}H)

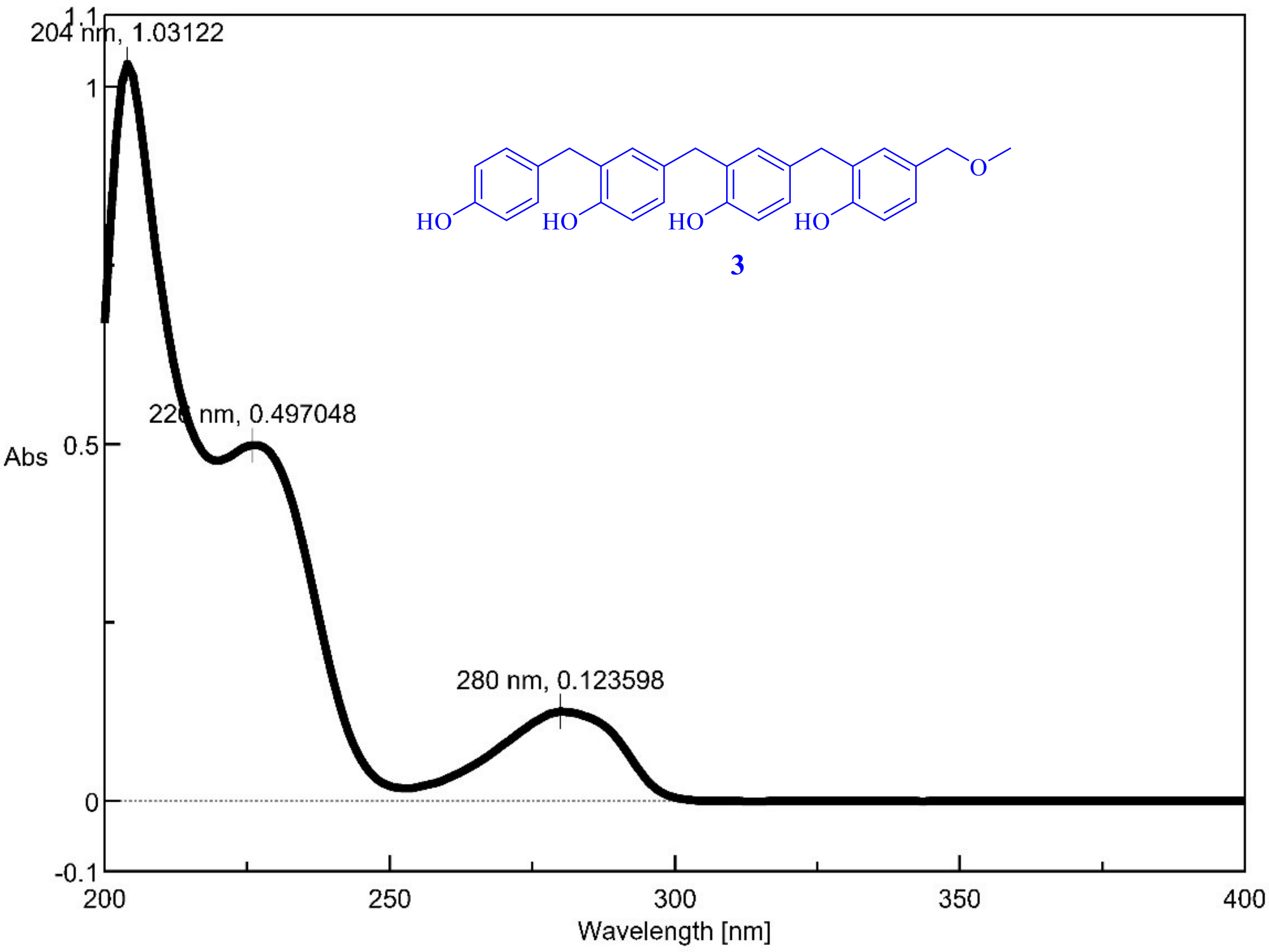

Memory-13

[Comment]
Sample Name wyn-35
Comment
User wyn
Division
Company 324

[Measurement Information]
Instrument Name V-650
Model Name V-650
Serial No. A034461150

Accessory PSC-718
Accessory S/N A001761114
Position 1
Cell Length 10 mm
Temperature 20.00 C
Control Sonsor Holder
Monitor Sensor Holder
Start Mode Start immediately

Photometric Mode Abs
Measurement range 400 - 195 nm
Data pitch 1 nm
Band width(UV/Vis) 1.0 nm
Response Medium
Scanning speed 200 nm/min
Source Change 340 nm
Light Source D2/WI
Filter Exchange Step
Correction Baseline

[Data Information]
Creation Date 2011-10-12 14:53

Data array type Linear data array
Horizontal Wavelength [nm]
Vertical Abs
Start 400 nm
End 195 nm
Data pitch 1 nm
Data points 206

Figure S31. The UV spectrum of compound **3**

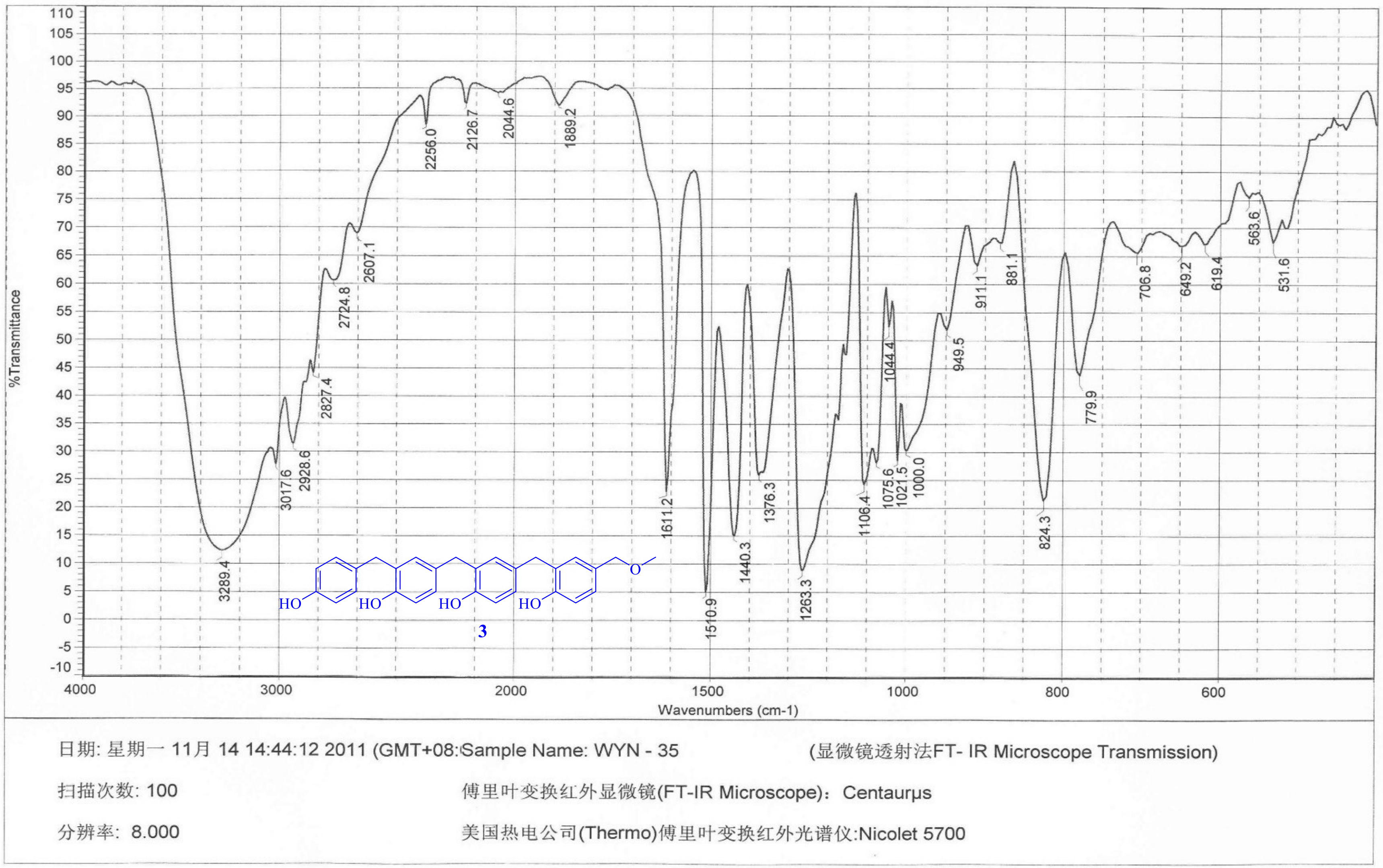

Figure S32. The IR spectrum of compound **3**

Single Mass Spectrum Deconvolution Report

Analysis Name: wangy021.d **Instrument:** LC-MSD-Trap-SL **Print Date:** 7/31/2010 2:48:37 PM
Method: LCMS.m **Operator:** Operator **Acq. Date:** 7/31/2010 2:32:09 PM
Sample Name: WYN-35
Analysis Info:

Acquisition Parameter:

Mass Range Mode	Std/Normal	Trap Drive	49.3	Scan Begin	100 m/z
Ion Polarity	Positive	Octopole RF Amplitude	162.2 Vpp	Scan End	800 m/z
Ion Source Type	ESI	Capillary Exit	-106.8 Volt	Averages	7 Spectra
Dry Temp (Set)	330 °C	Skimmer	-40.0 Volt	Max. Accu Time	200000 µs
Nebulizer (Set)	15.00 psi	Oct 1 DC	-12.00 Volt	ICC Target	30000
Dry Gas (Set)	5.00 l/min	Oct 2 DC	-1.70 Volt	Charge Control	on

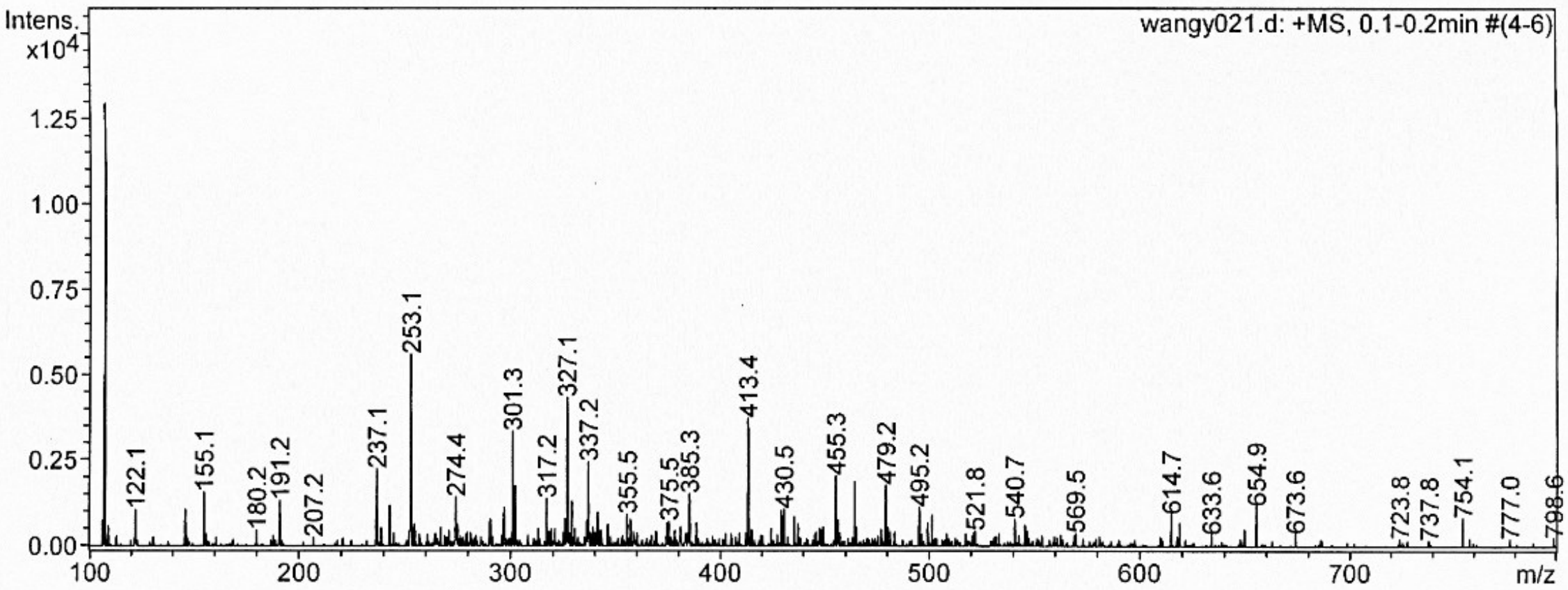

Component	Molecular Mass	Molecule	Absolute Abundance	Relative Abundance

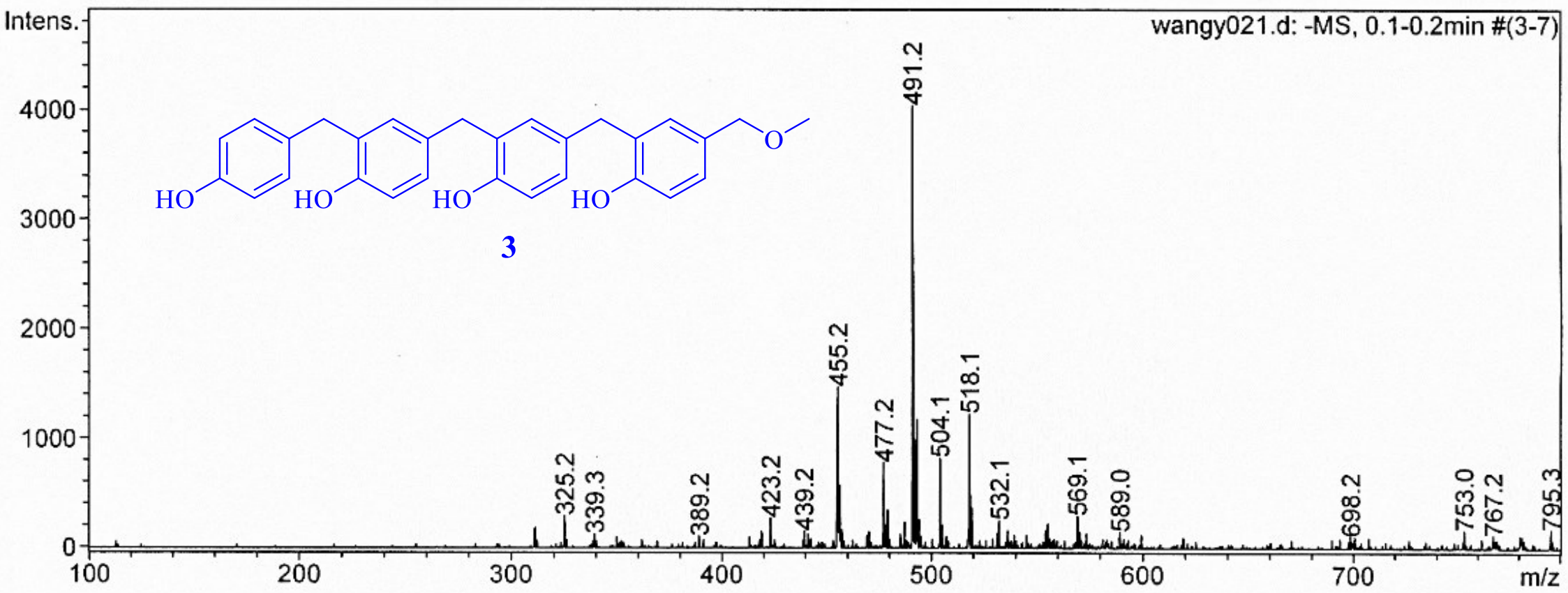

Component	Molecular Mass	Molecule	Absolute Abundance	Relative Abundance

MSD Trap Report v 4 (A4-Opt2) Page 1 of 1 Agilent Technologies

Figure S33. The ESIMS of compound **3**

Qualitative Analysis Report

**Data Filename**	201106131.d	**Sample Name**	WYN-35
**Sample Type**	Sample	**Position**	P1-B1
**Instrument Name**	Instrument 1	**User Name**	
**Acq Method**		**IRM Calibration Status**	Success
**DA Method**	TEST LCMS.m	**Comment**	

User Chromatograms

Fragmentor Voltage 135 **Collision Energy** 0 **Ionization Mode** ESI

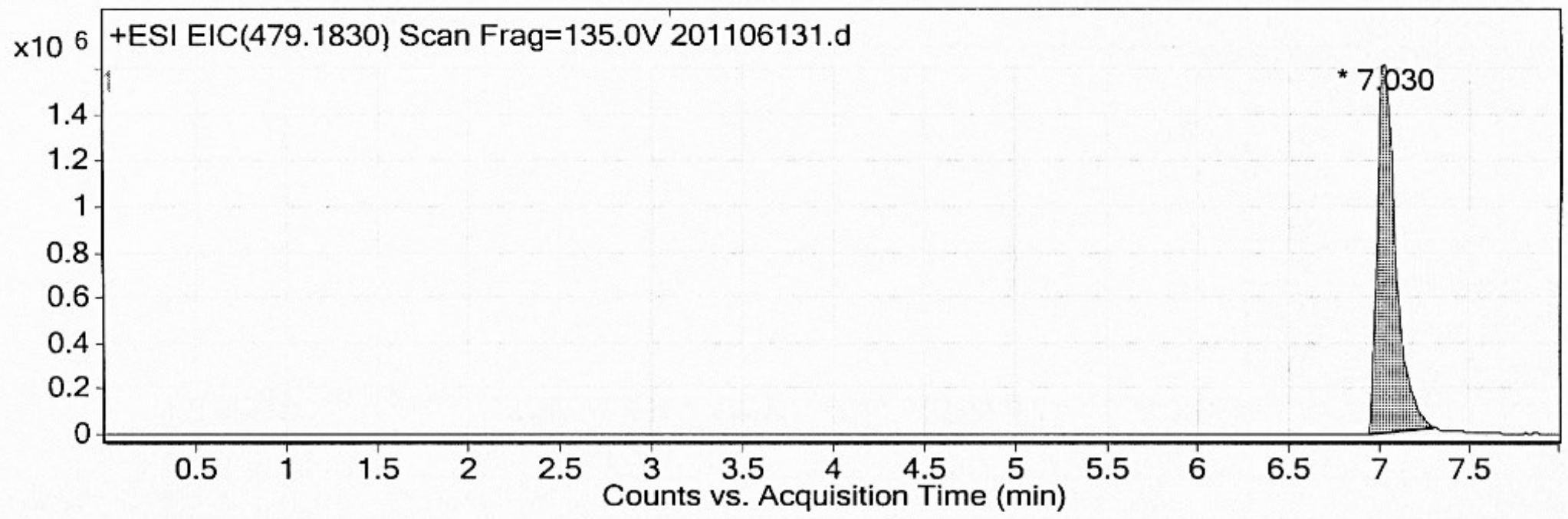

Integration Peak List

Peak	Start	RT	End	Height	Area	Area %
1	6.934	7.03	7.303	1610532	11583365	100

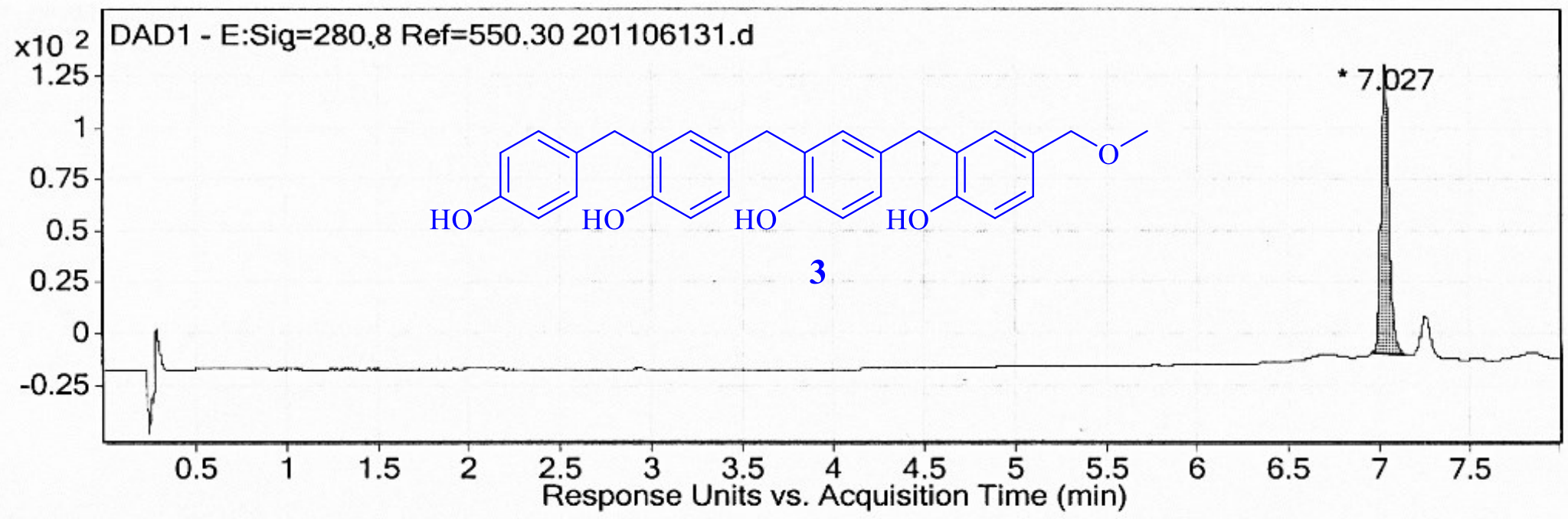

Integration Peak List

Peak	Start	RT	End	Height	Area	Area %
1	6.954	7.027	7.143	140.59	446.79	100

User Spectra

**Fragmentor Voltage**	**Collision Energy**	**Ionization Mode**
135	0	Esi

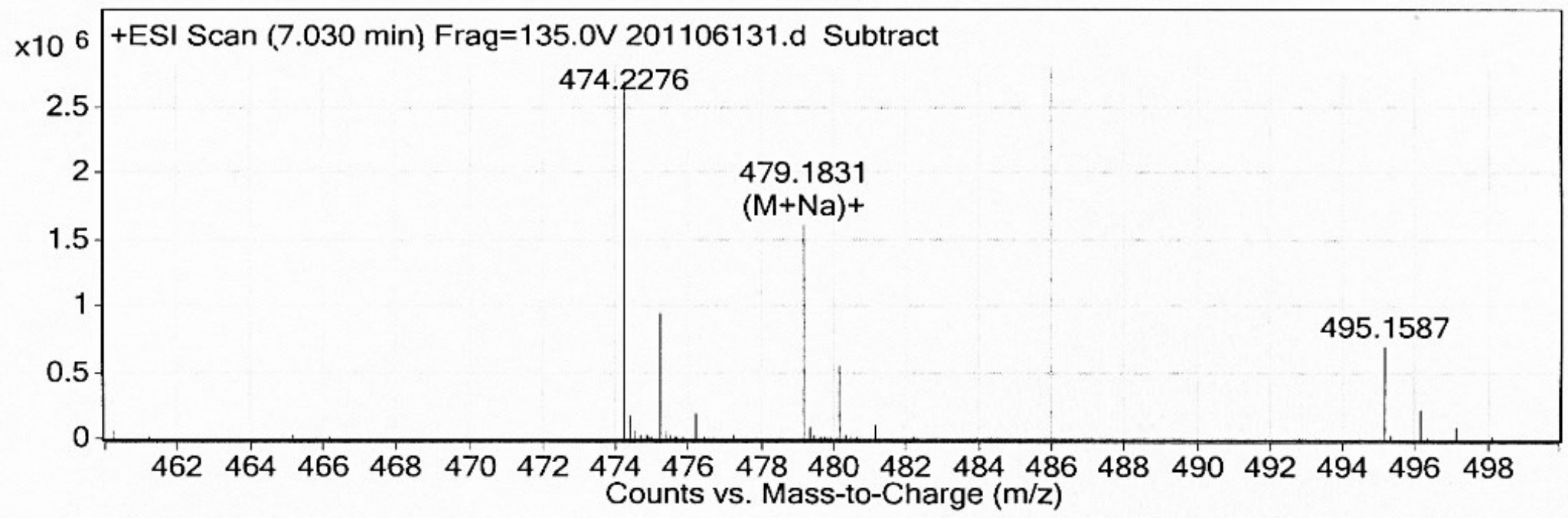

Agilent Technologies

Page 1 of 2

Printed at: 12:38 PM on: 6/13/2011

Figure S34. The HR-ESIMS report of compound **3** (page 1)

Qualitative Analysis Report

Peak List

m/z	z	Abund	Formula	Ion
425.175		257899		
474.2276	1	2684884		
474.4149		168240		
475.2316	1	937634		
476.2335	1	185416		
479.1831	1	1617543	C29 H28 Na O5	(M+Na)+
480.1872	1	547043	C29 H28 Na O5	(M+Na)+
495.1587	1	702899		
496.1612	1	221569		

Formula Calculator Element Limits

Element	Min	Max
C	3	100
H	0	120
O	0	30
N	0	2
S	0	0
Cl	0	0

Formula Calculator Results

Formula	Best	Mass	Tgt Mass	Diff (ppm)	Ion Species	Score
C29 H28 O5	TRUE	456.1939	456.1937	-0.58	C29 H28 Na O5	99.86
C24 H28 N2 O7		456.1939	456.1897	-9.41	C24 H28 N2 Na O7	97.72
C17 H32 N2 O12		456.1939	456.1955	3.46	C17 H32 N2 Na O12	95.34

--- End Of Report ---

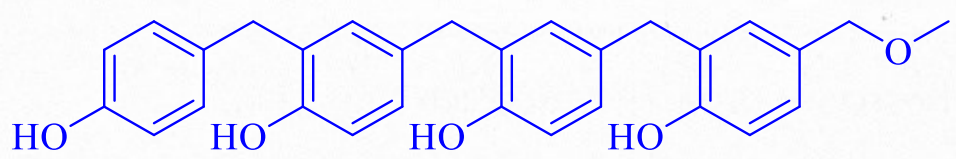

3

Agilent Technologies | Page 2 of 2 | Printed at: 12:38 PM on: 6/13/2011

Figure S35. The HR-ESIMS report of compound **3** (page 2)

MS Formula Results: + Scan (7.030 min) Sub (201106131.d)

m/z	Ion	Formula	Abundance
479.1831	(M+Na)+	C29 H28 Na O5	1617543.4

Best	Formula (M)	Ion Formula	Calc m/z	Score	Cross S	Mass	Calc Mass	Diff (ppm)	Abs Diff (ppm)	Abund Match	Spacing Mat	Mass Match	m/z	DBE
☑	C29 H28 O5	C29 H28 Na O5	479.1829	99.86		456.1939	456.1937	-0.58	0.58	99.58	99.95	99.99	479.1831	16
☐	C24 H28 N2 O7	C24 H28 N2 Na O7	479.1789	97.72		456.1939	456.1897	-9.41	9.41	96.58	99.92	97.3	479.1831	12
☐	C17 H32 N2 O12	C17 H32 N2 Na O12	479.1847	95.34		456.1939	456.1955	3.46	3.46	84.41	99.89	99.63	479.1831	3

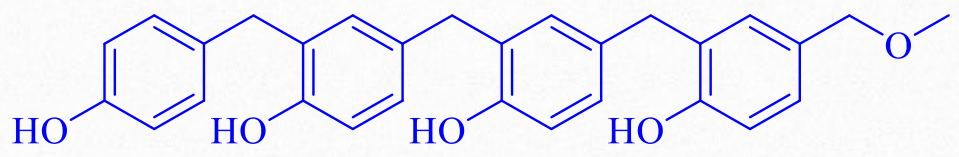

3

page 1

Figure S36. The HR-ESIMS report of compound **3** (page 3)

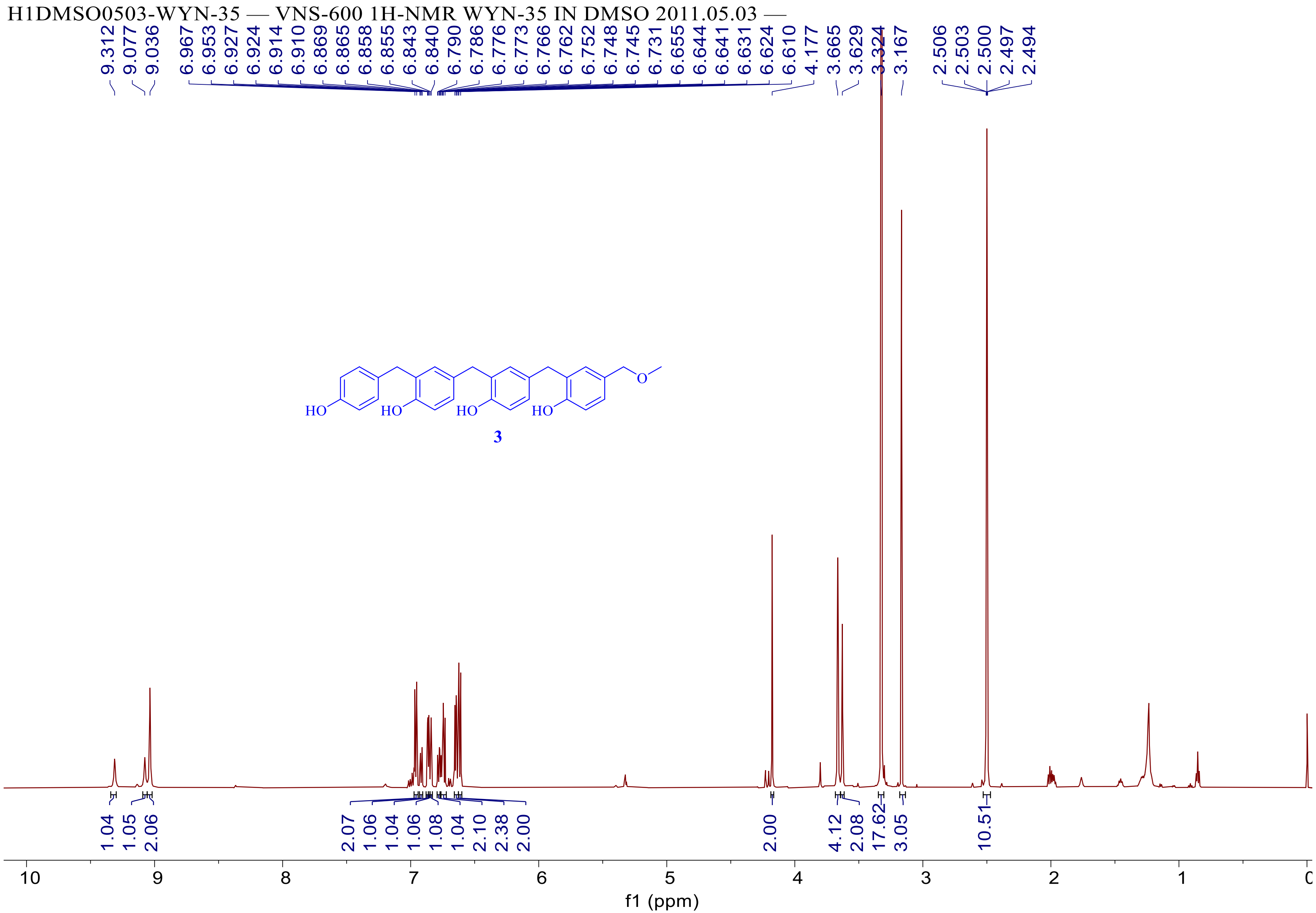

Figure S37. The ^{1}H NMR spectrum of compound **3** in DMSO-d_6 (600 MHz)

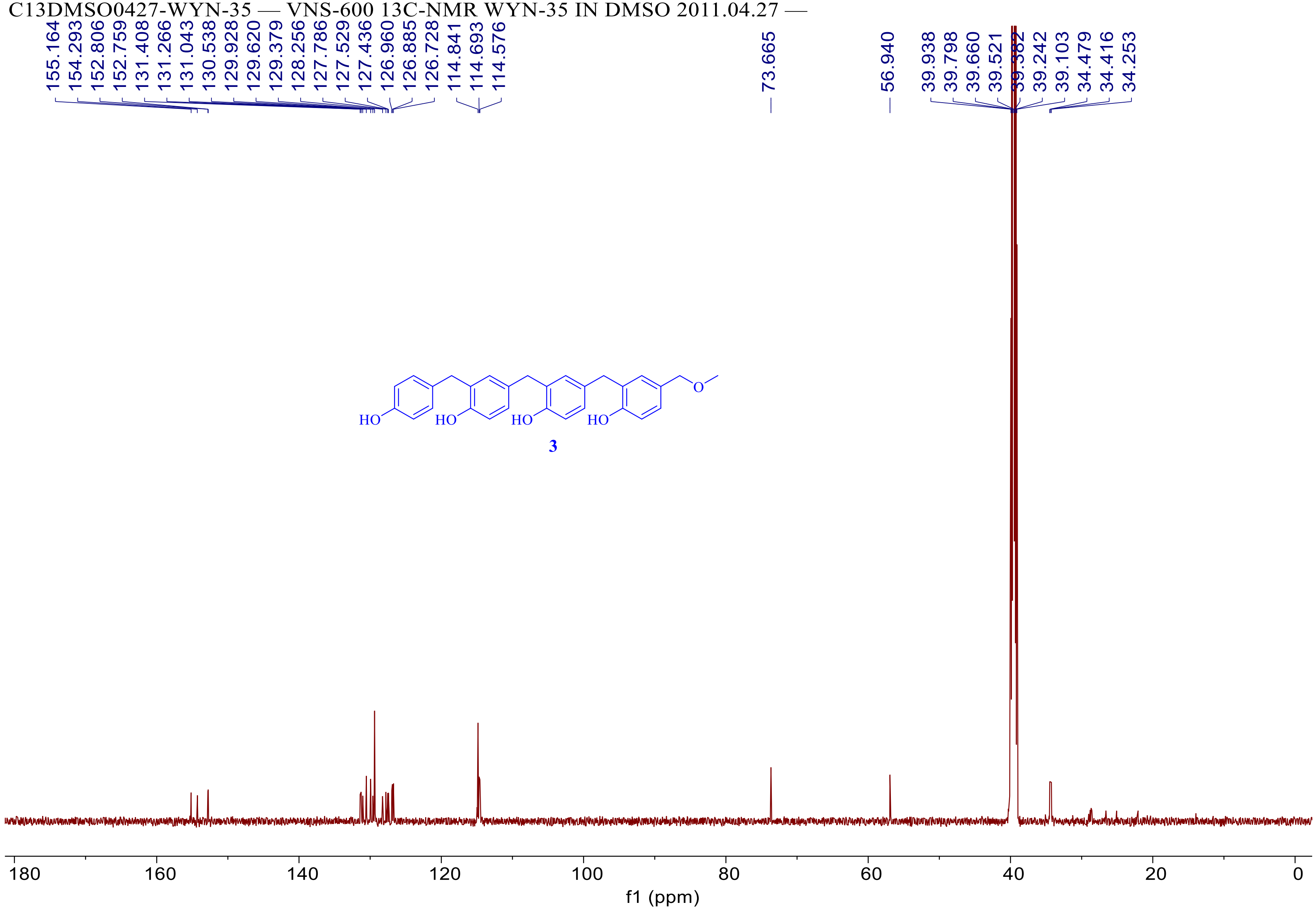

Figure S38. The ^{13}C NMR spectrum of compound **3** in DMSO-d_6 (150 MHz)

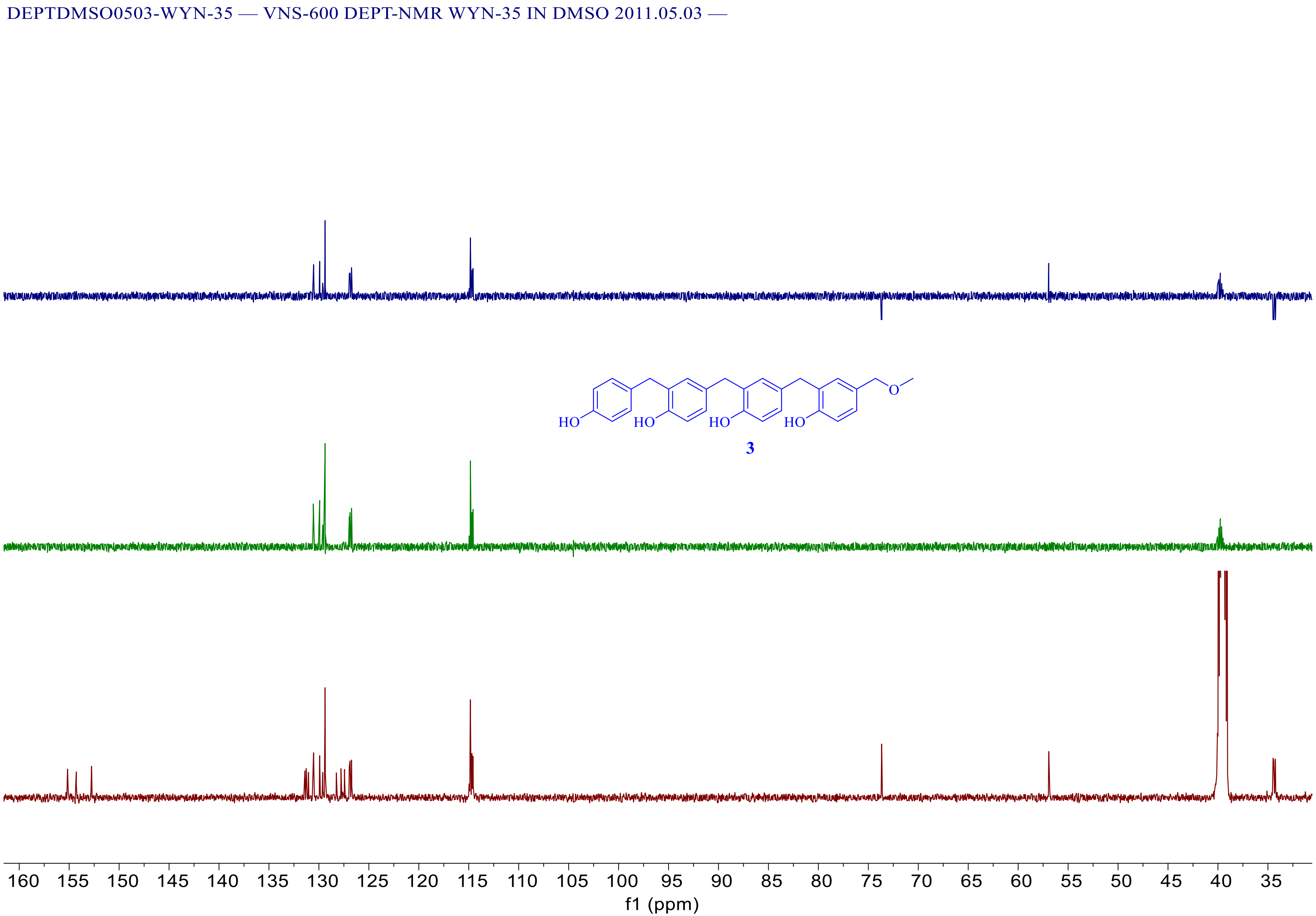

Figure S39. The DEPT spectrum of compound **3** in DMSO-d_6 (150 MHz)

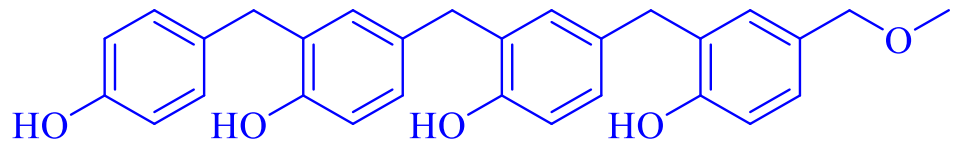

3

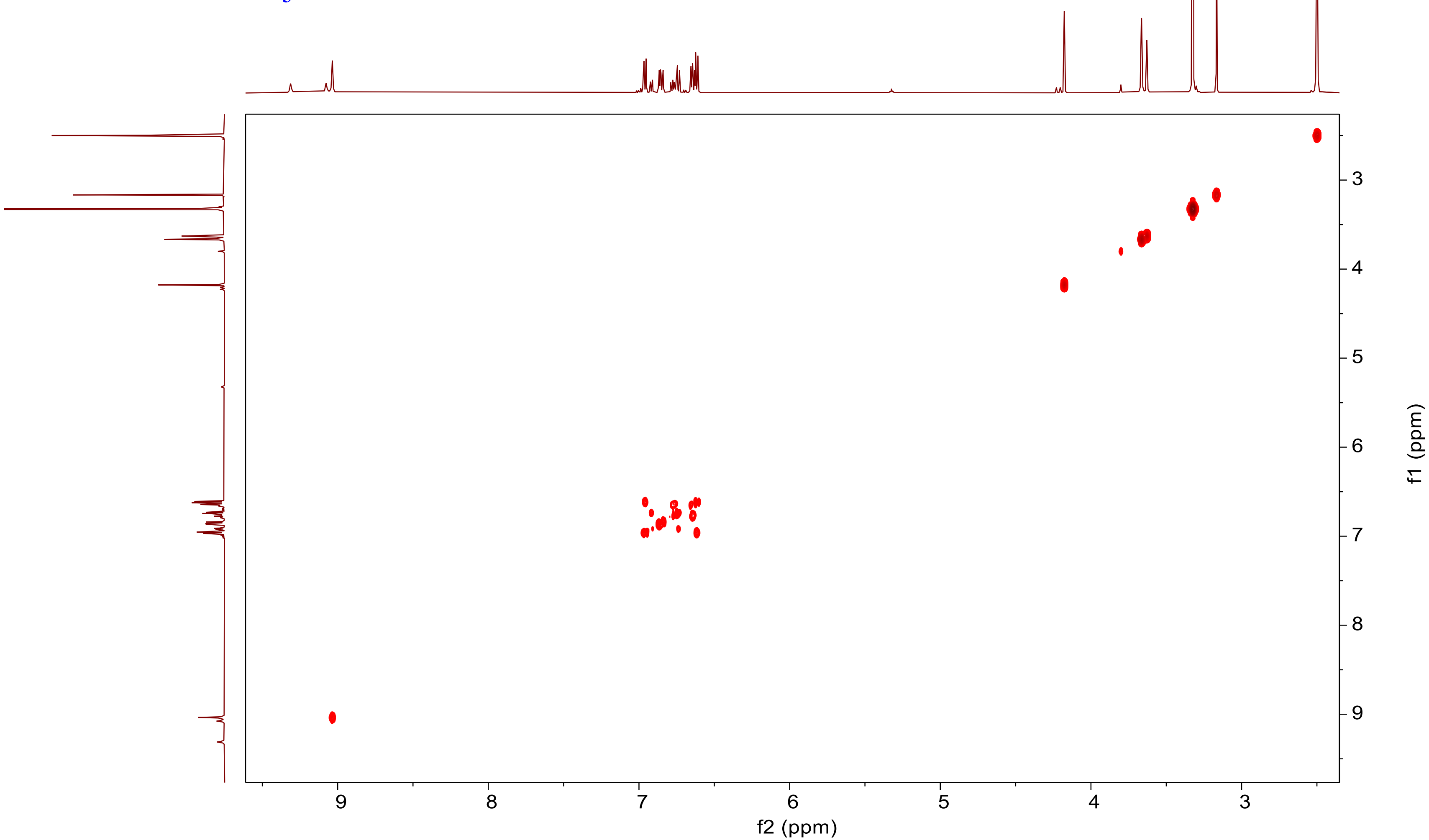

Figure S40. The ^{1}H-^{1}H COSY spectrum of compound **3** in DMSO-d_6 (600 MHz)

HSQCDMSO0503-WYN-35 — VNS-600 gHSQC WYN-35 IN DMSO 2011.05.03 —

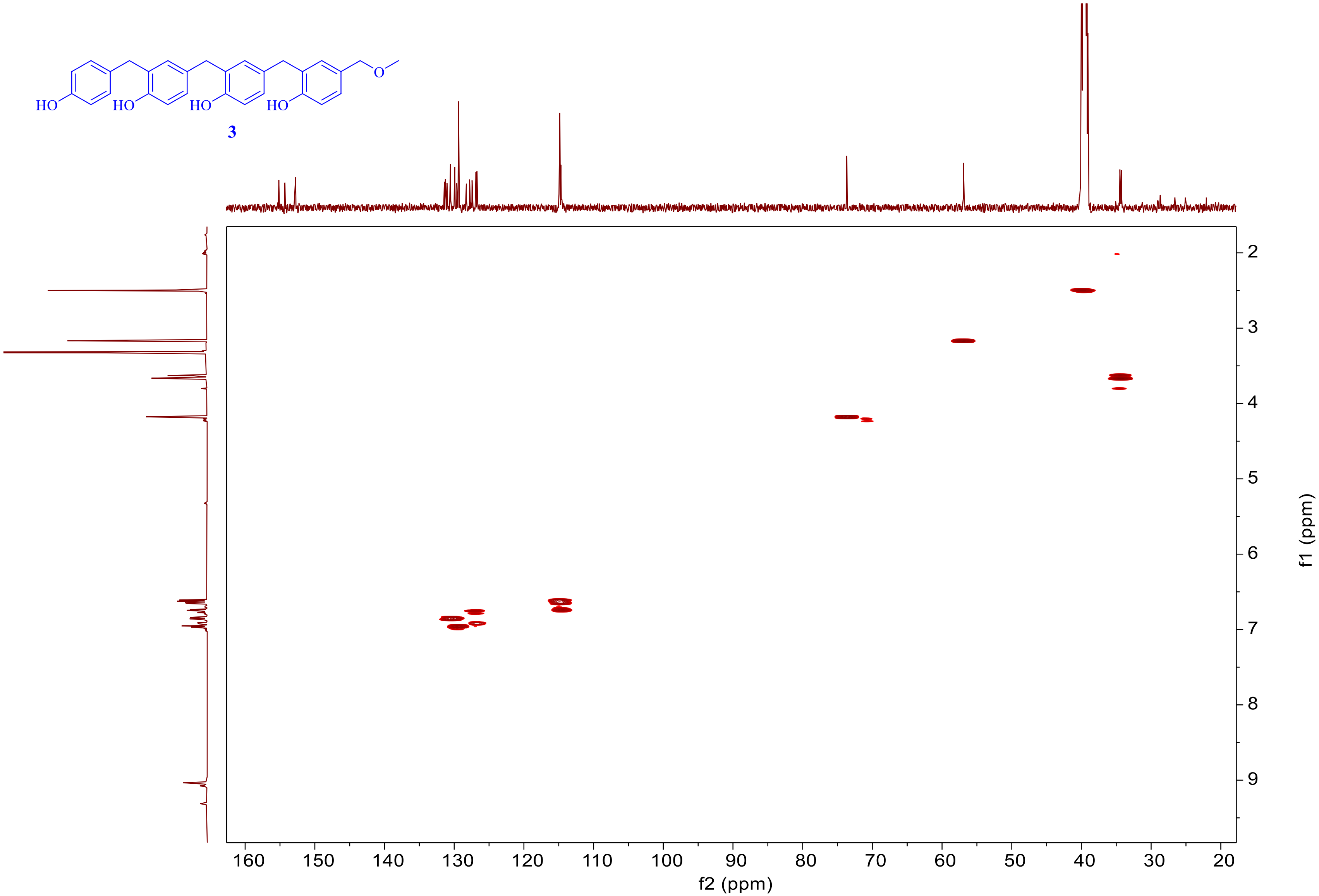

Figure S41. The HSQC spectrum of compound **3** in DMSO-d_6 (600 MHz for ^{1}H)

HMBCDMSO0503-WYN-35 — VNS-600 gHMBC WYN-35 IN DMSO 2011.05.03 —

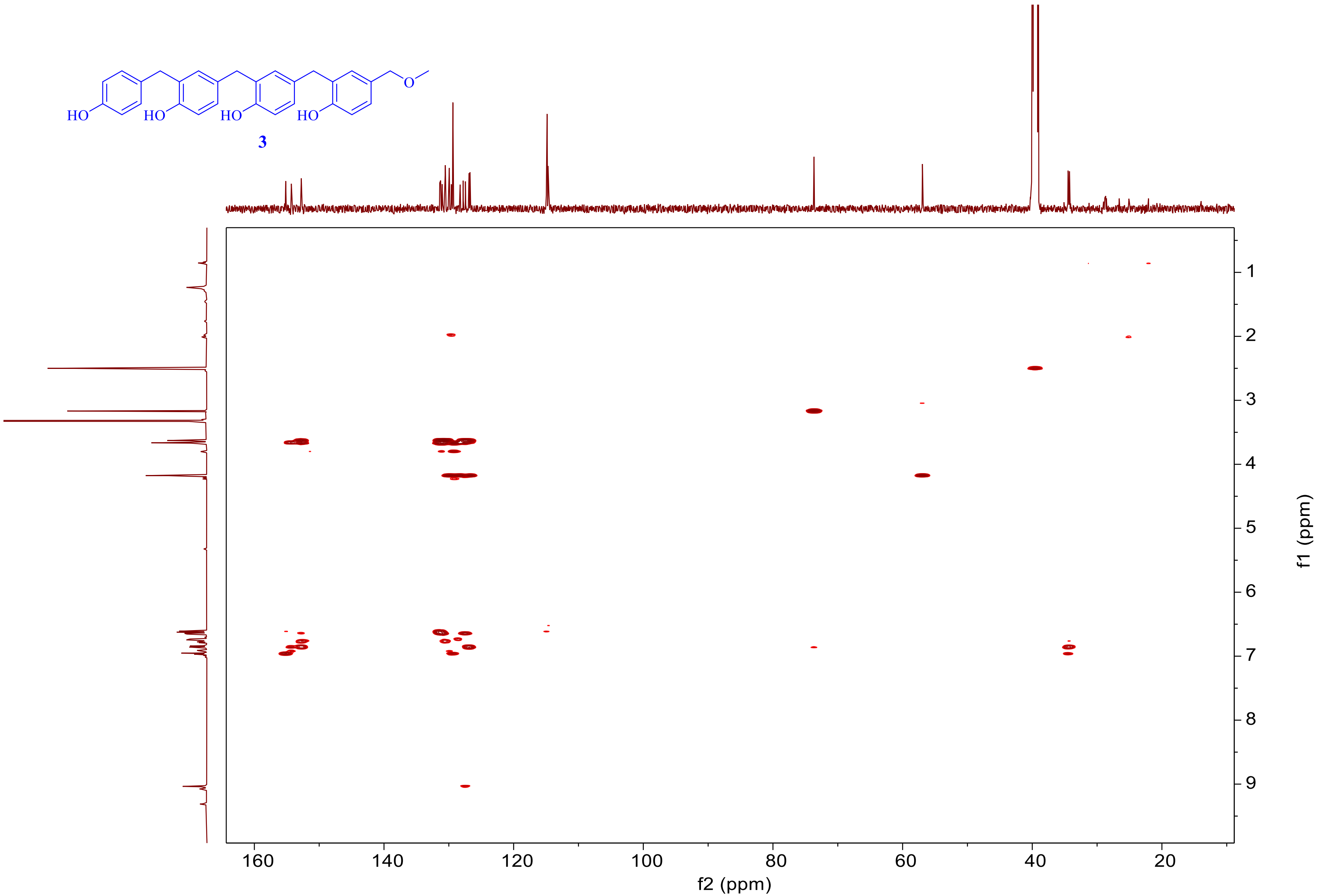
3

Figure S42. The HMBC spectrum of compound **3** in DMSO-d_6 (600 MHz for ^{1}H)

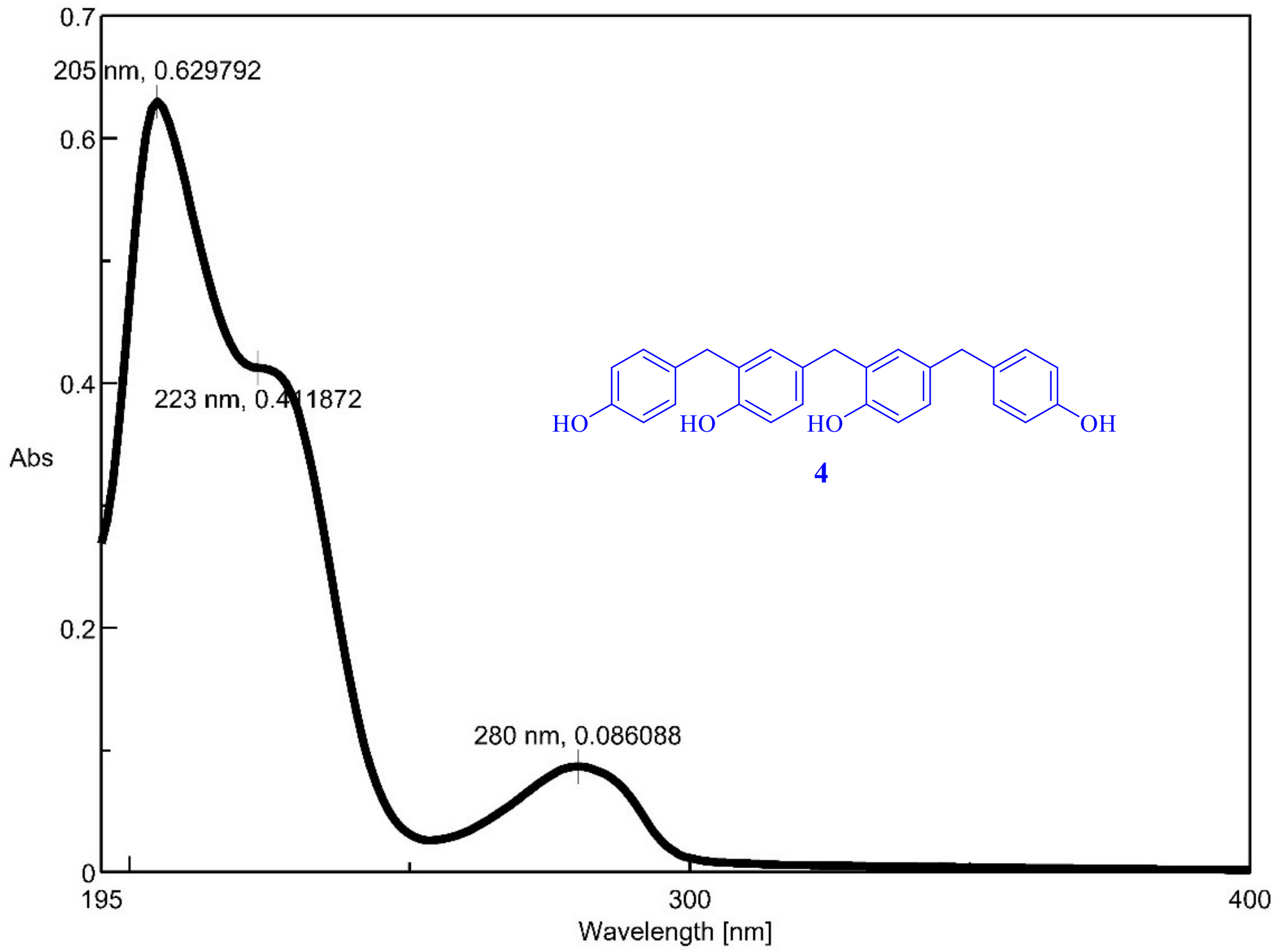

Memory-1

[Comment]	
Sample Name	wyn-20C
Comment	
User	wyn
Division	
Company	324
[Measurement Information]	
Instrument Name	V-650
Model Name	V-650
Serial No.	A034461150
Accessory	PSC-718
Accessory S/N	A001761114
Position	1
Cell Length	10 mm
Temperature	19.98 C
Control Sonsor	Holder
Monitor Sensor	Holder
Start Mode	Start immediately
Photometric Mode	Abs
Measurement range	400 - 195 nm
Data pitch	1 nm
Band width(UV/Vis)	1.0 nm
Response	Medium
Scanning speed	200 nm/min
Source Change	340 nm
Light Source	D2/WI
Filter Exchange	Step
Correction	Baseline

[Data Information]	
Creation Date	2011-10-12 14:21
Data array type	Linear data array
Horizontal	Wavelength [nm]
Vertical	Abs
Start	400 nm
End	195 nm
Data pitch	1 nm
Data points	206

Figure S43. The UV spectrum of compound **4**

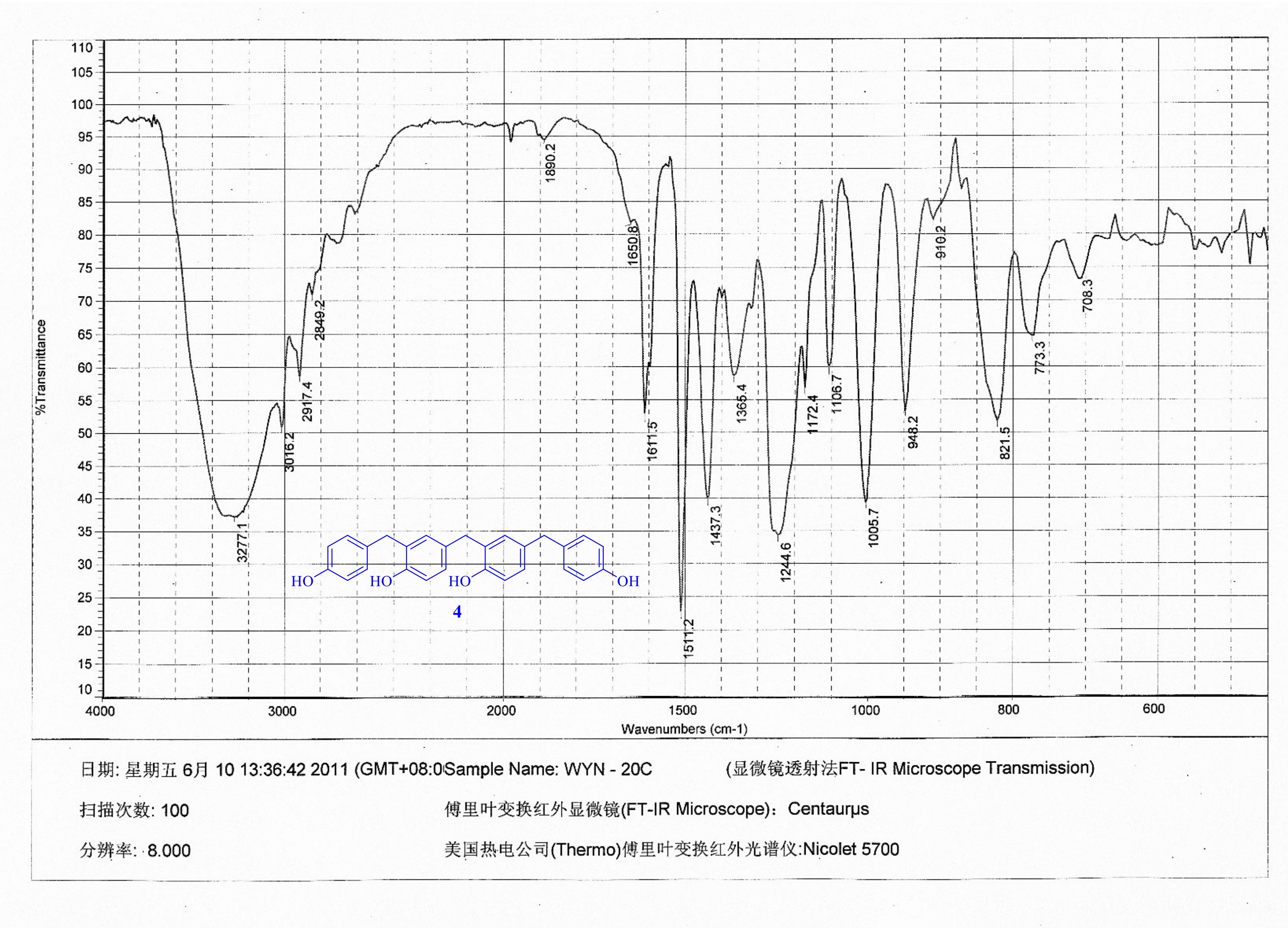

Figure S44. The IR spectrum of compound **4**

Single Mass Spectrum Deconvolution Report

Analysis Name: WANGY002.d **Instrument:** LC-MSD-Trap-SL **Print Date:** 7/9/2010 11:07:47 AM
Method: TEST.MS **Operator:** Operator **Acq. Date:** 7/9/2010 11:04:04 AM
Sample Name: WYN-20C
Analysis Info:

Acquisition Parameter:

Mass Range Mode	Std/Normal	Trap Drive	53.0	Scan Begin	100 m/z
Ion Polarity	Positive	Octopole RF Amplitude	171.0 Vpp	Scan End	600 m/z
Ion Source Type	ESI	Capillary Exit	-106.0 Volt	Averages	5 Spectra
Dry Temp (Set)	330 °C	Skimmer	-40.0 Volt	Max. Accu Time	200000 µs
Nebulizer (Set)	15.00 psi	Oct 1 DC	-12.00 Volt	ICC Target	20000
Dry Gas (Set)	5.00 l/min	Oct 2 DC	-1.70 Volt	Charge Control	on

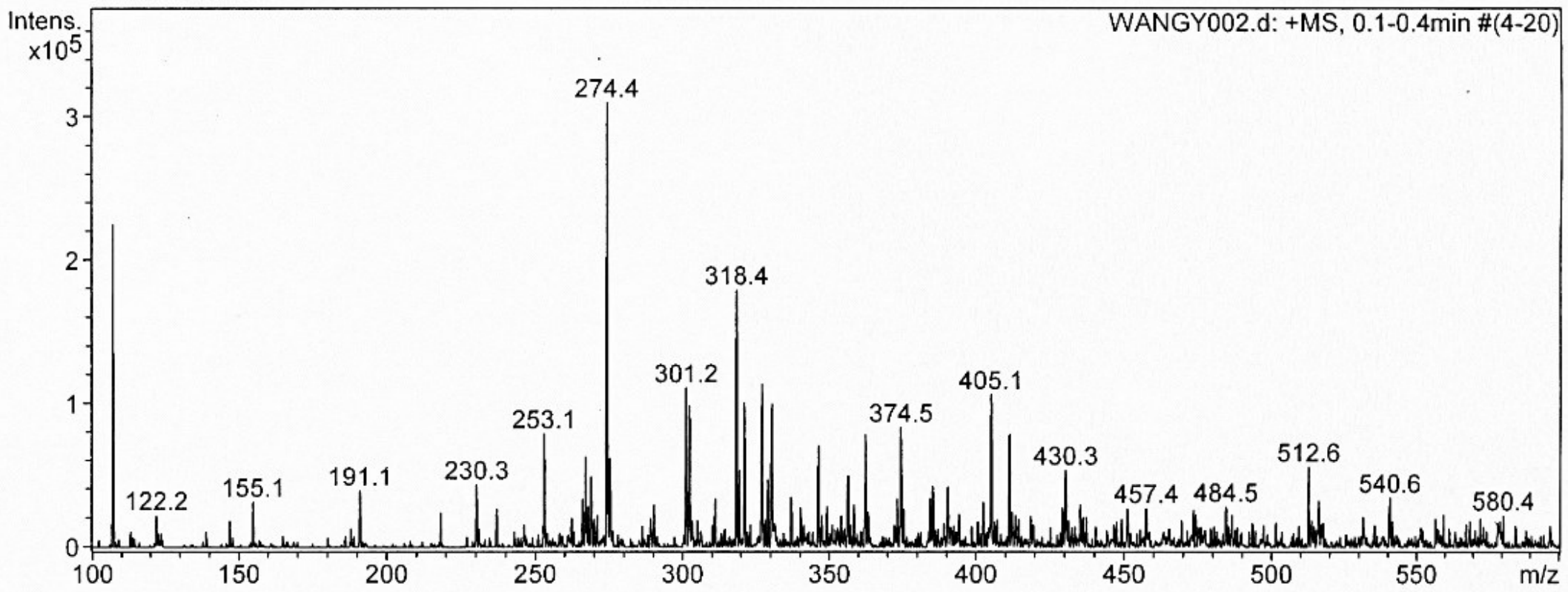

Component	Molecular Mass	Molecule	Absolute Abundance	Relative Abundance

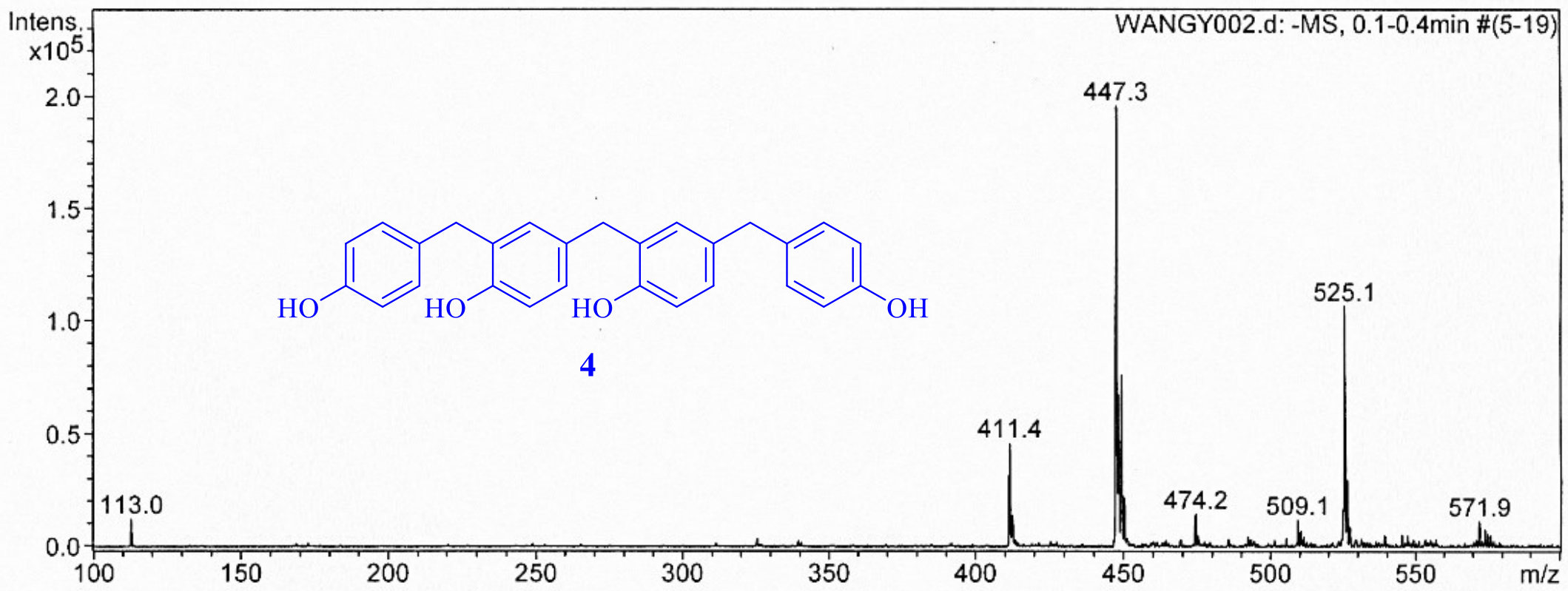

Component	Molecular Mass	Molecule	Absolute Abundance	Relative Abundance

MSD Trap Report v 4 (A4-Opt2) Page 1 of 1 Agilent Technologies

Figure S45. The ESIMS of compound **4**

Qualitative Analysis Report

**Data Filename**	2011011714.d	**Sample Name**	WYN-20C
**Sample Type**	Sample	**Position**	P1-D7
**Instrument Name**	Instrument 1	**User Name**	
**Acq Method**		**IRM Calibration Status**	Success
**DA Method**	TEST LCMS.m	**Comment**	

User Chromatograms

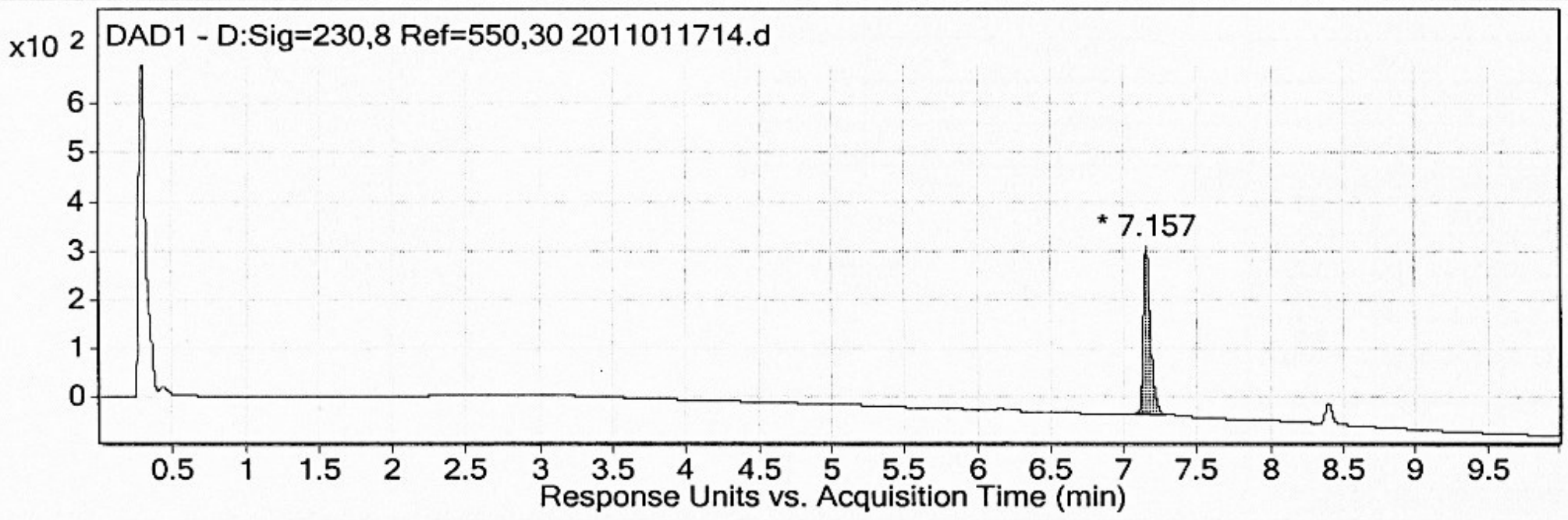

Integration Peak List

Peak	Start	RT	End	Height	Area	Area %
1	7.076	7.157	7.285	345.56	1182.46	100

Fragmentor Voltage 135 **Collision Energy** 0 **Ionization Mode** ESI

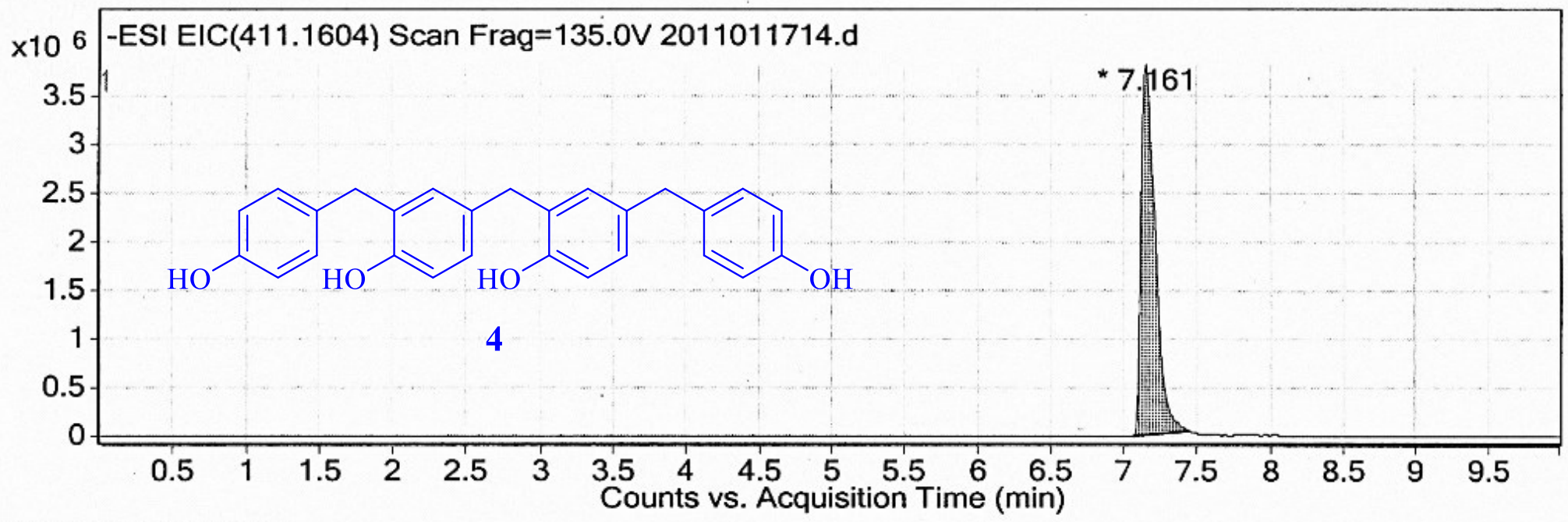

Integration Peak List

Peak	Start	RT	End	Height	Area	Area %
1	7.065	7.161	7.45	3799712	25832379	100

User Spectra

Fragmentor Voltage	Collision Energy	Ionization Mode
135	0	Esl

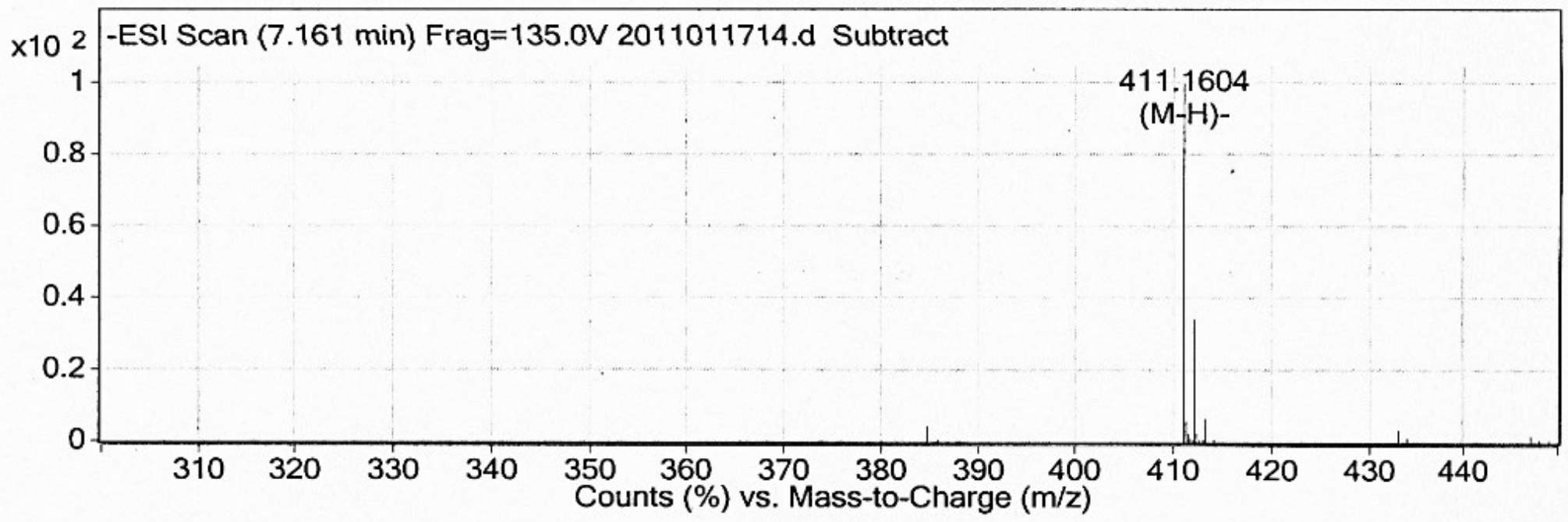

Figure S46. The HR-ESIMS report of compound **4** (page 1)

Qualitative Analysis Report

Peak List

m/z	z	Abund	Formula	Ion
248.961		329428		
411.1604	1	3813106	C27 H23 O4	(M-H)-
412.1637	1	1297346	C27 H23 O4	(M-H)-
520.9113		678378		
656.8854		416035		
792.8602		343023		
823.328	1	603031		
824.3315	1	354830		
845.3099	1	485183		
1033.9882		298899		

Formula Calculator Element Limits

Element	Min	Max
C	3	100
H	0	120
O	0	30
N	0	2
S	0	0
Cl	0	0

Formula Calculator Results

Formula	Best	Mass	Tgt Mass	Diff (ppm)	Ion Species	Score
C27 H24 O4	TRUE	412.1677	412.1675	-0.46	C27 H23 O4	99.55
C15 H28 N2 O11		412.1677	412.1693	4.01	C15 H27 N2 O11	93.57

--- End Of Report ---

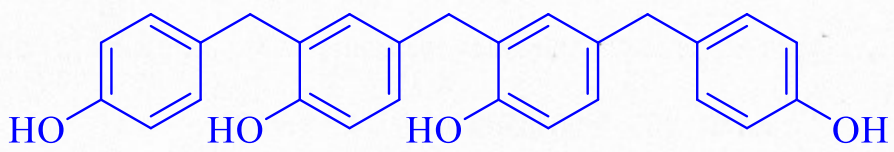

4

Figure S47. The HR-ESIMS report of compound **4** (page 2)

MS Formula Results: - Scan (7.161 min) Sub (2011011714.d)

m/z	Ion	Formula	Abundance
411.1604	(M-H)-	C27 H23 O4	3813105.5

Best	Formula (M)	Ion Formula	Calc m/z	Score	Cross S	Mass	Calc Mass	Diff (ppm)	Abs Diff (ppm)	Abund Match	Spacing Mat	Mass Match	m/z	DBE
☑	C27 H24 O4	C27 H23 O4	411.1602	99.55		412.1677	412.1675	-0.46	0.46	98.43	100	99.99	411.1604	16
☐	C15 H28 N2 O11	C15 H27 N2 O11	411.162	93.57		412.1677	412.1693	4.01	4.01	78.45	99.94	99.47	411.1604	3

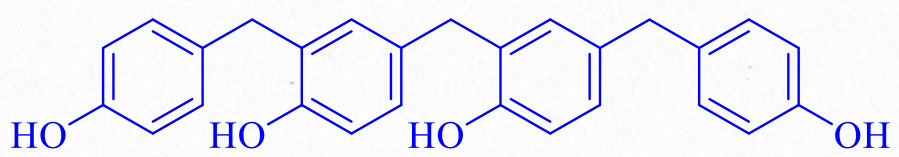

4

page 1

Figure S48. The HR-ESIMS report of compound **4** (page 3)

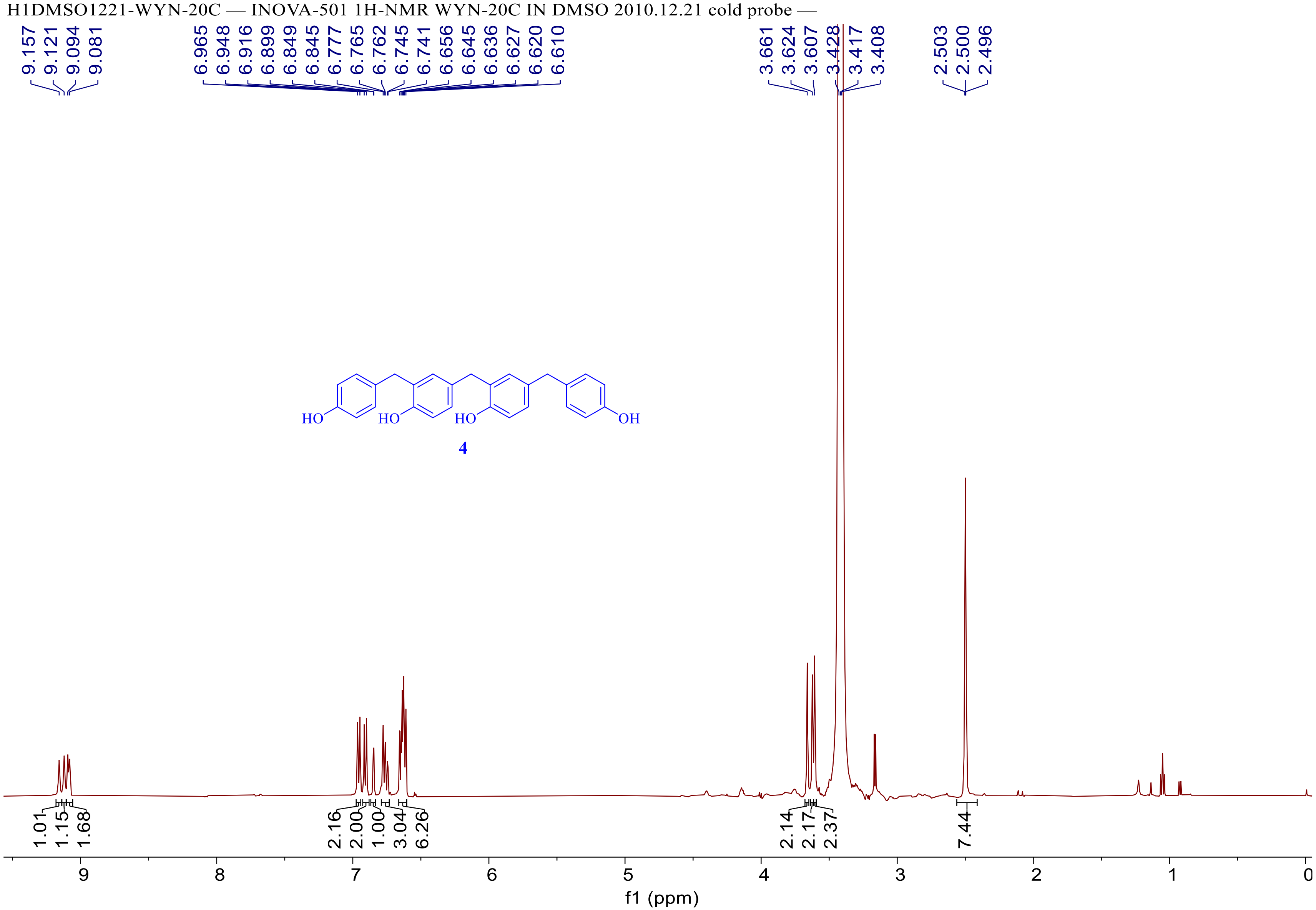

Figure S49. The ^{1}H NMR spectrum of compound **4** in DMSO-d_6 (500 MHz)

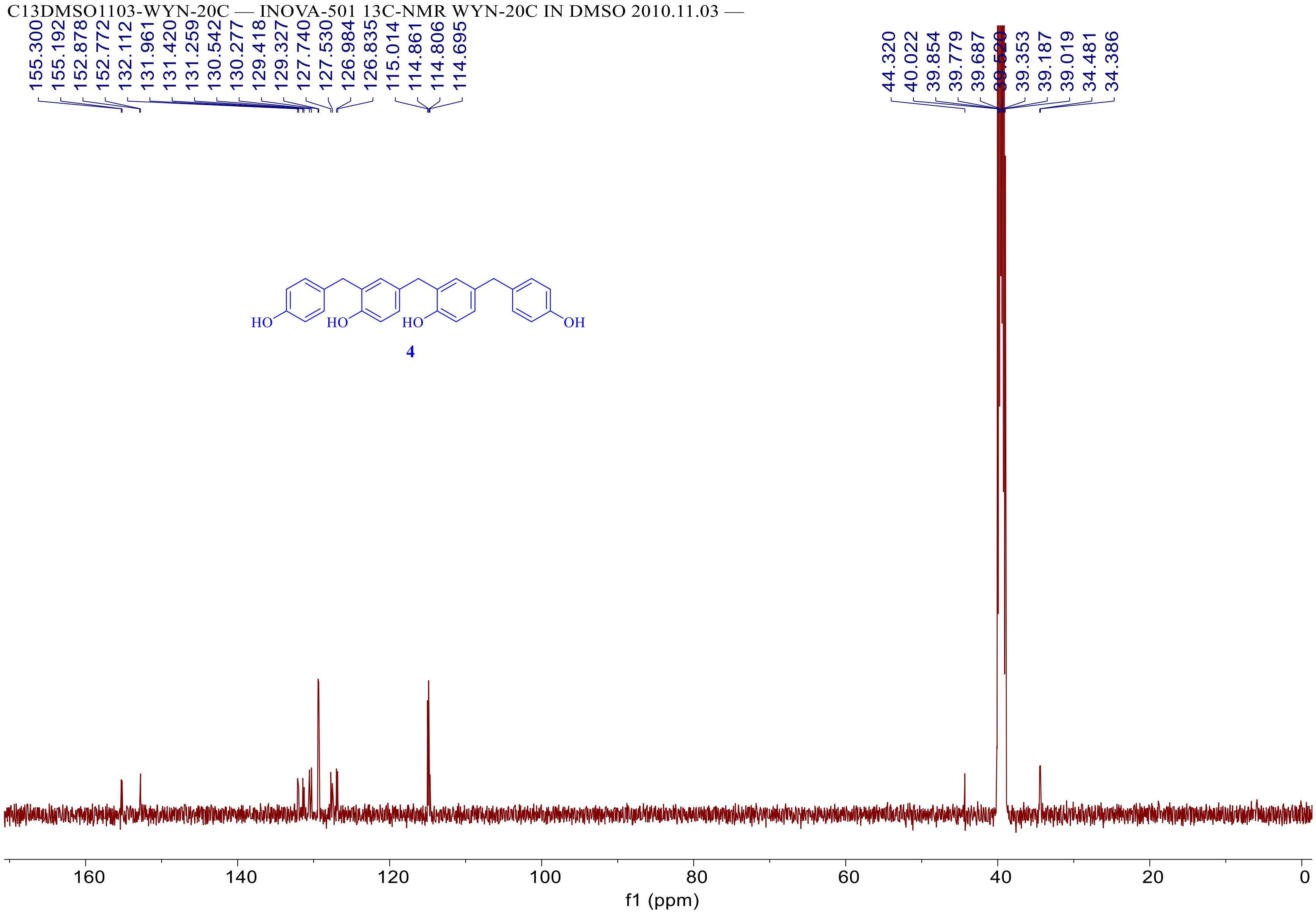

Figure S50. The ^{13}C NMR spectrum of compound **4** in DMSO-d_6 (125 MHz)

DEPTDMSO1221-WYN-20C — INOVA-501 DEPT WYN-20C IN DMSO 2010.12.21 cold probe —

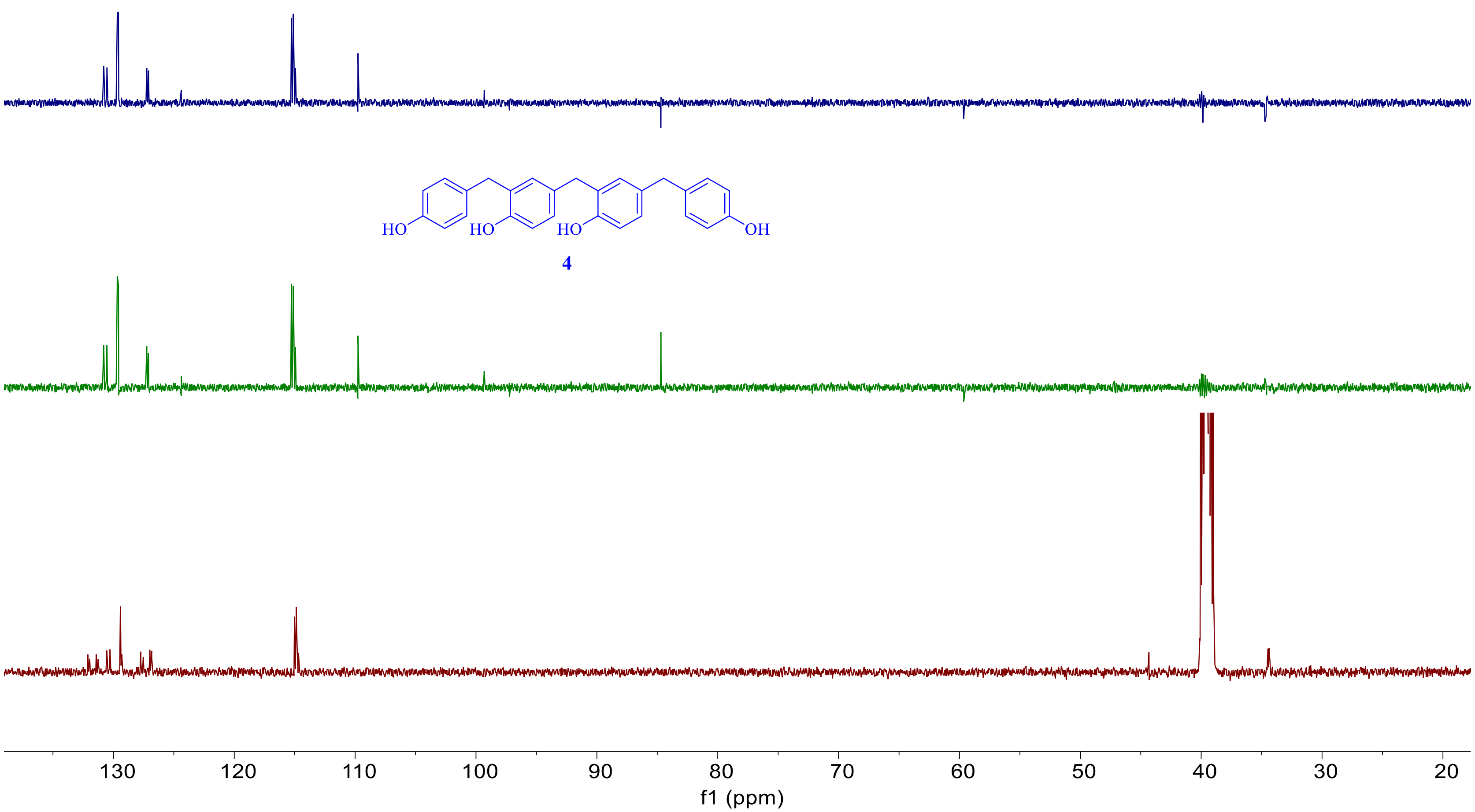

Figure S51. The DEPT spectrum of compound **4** in DMSO-d_6 (125 MHz)

COSYDMSO1221-WYN-20C — INOVA-501 gCOSY WYN-20C IN DMSO 2010.12.21 cold probe —

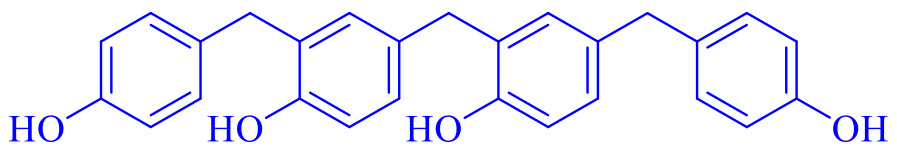

4

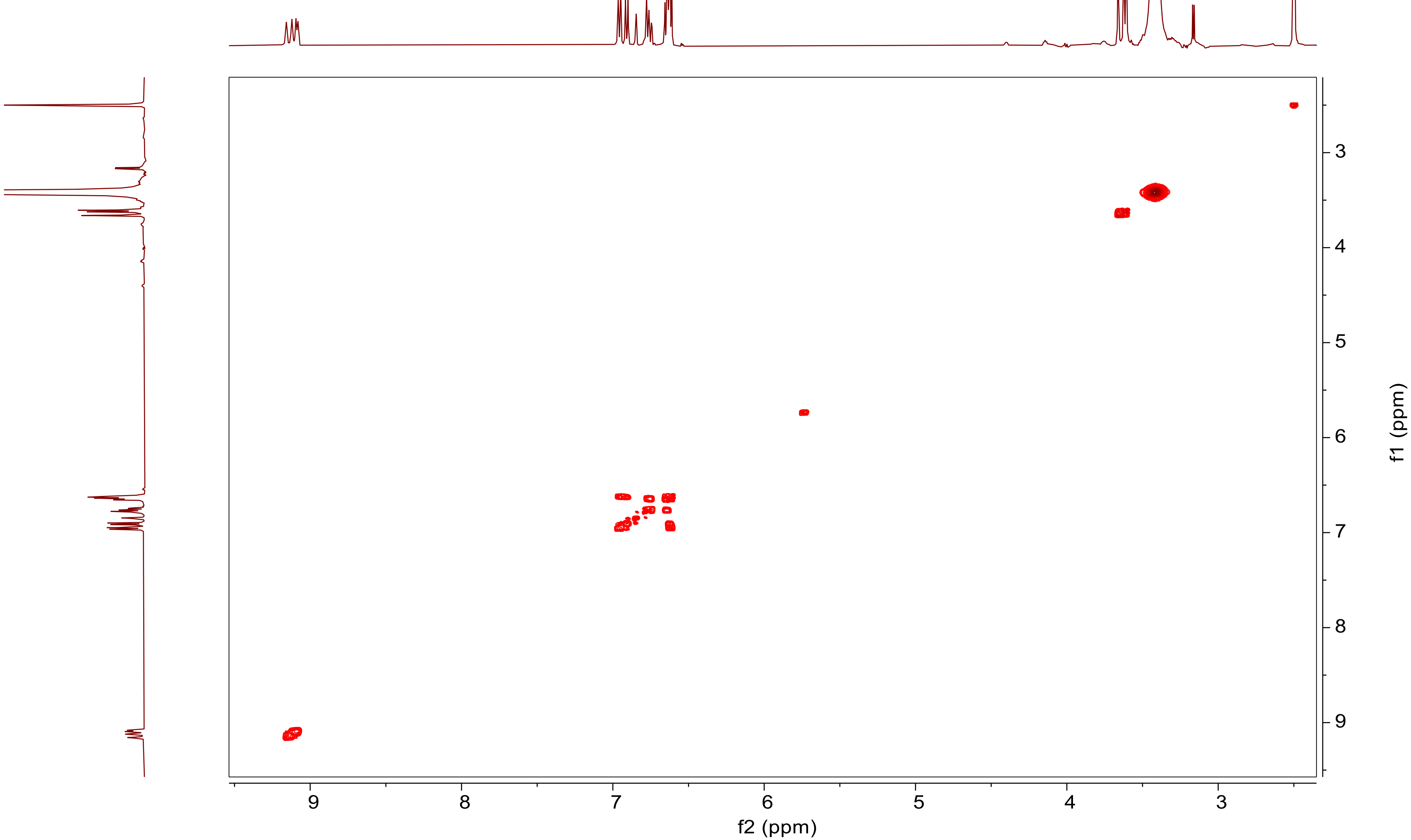

Figure S52. The ^{1}H-^{1}H COSY spectrum of compound **4** in DMSO-d_6 (500 MHz)

HSQCDMSO1221.WYN-20C — INOVA-501 gHSQC WYN-20C IN DMSO 2010.12.21 cold probe —

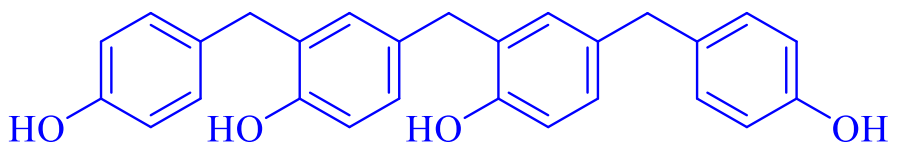

4

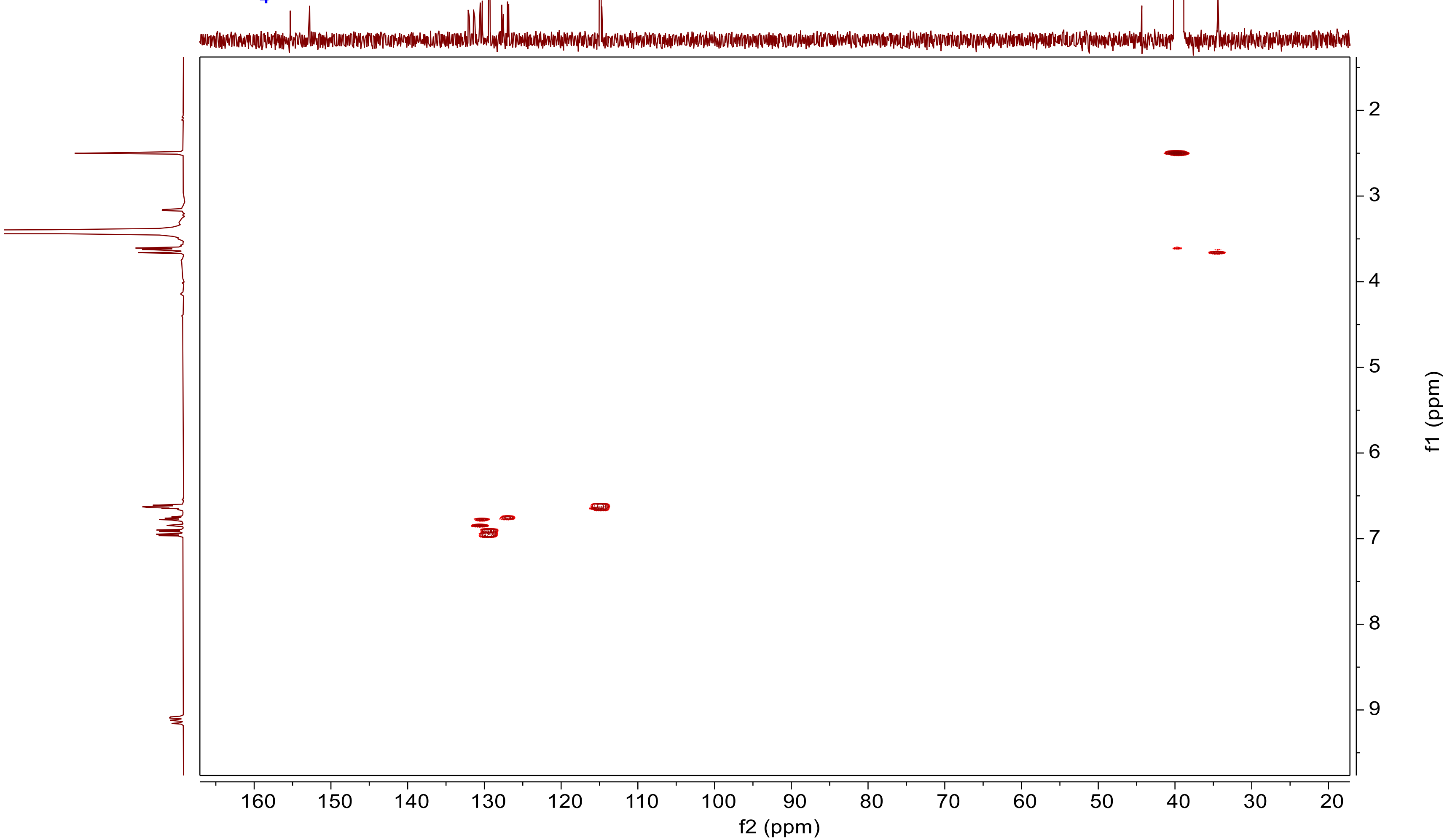

Figure S53. The HSQC spectrum of compound **4** in DMSO-d_6 (500 MHz for ^{1}H)

HMBCDMSO1221-WYN-20C — INOVA-501 gHMBC WYN-20C IN DMSO 2010.12.21 cold probe —

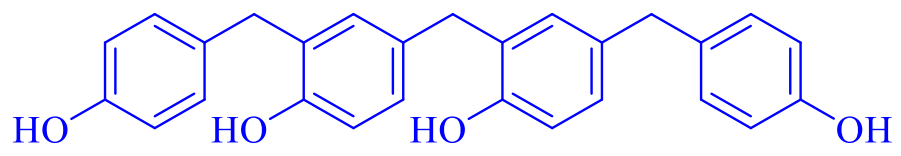

4

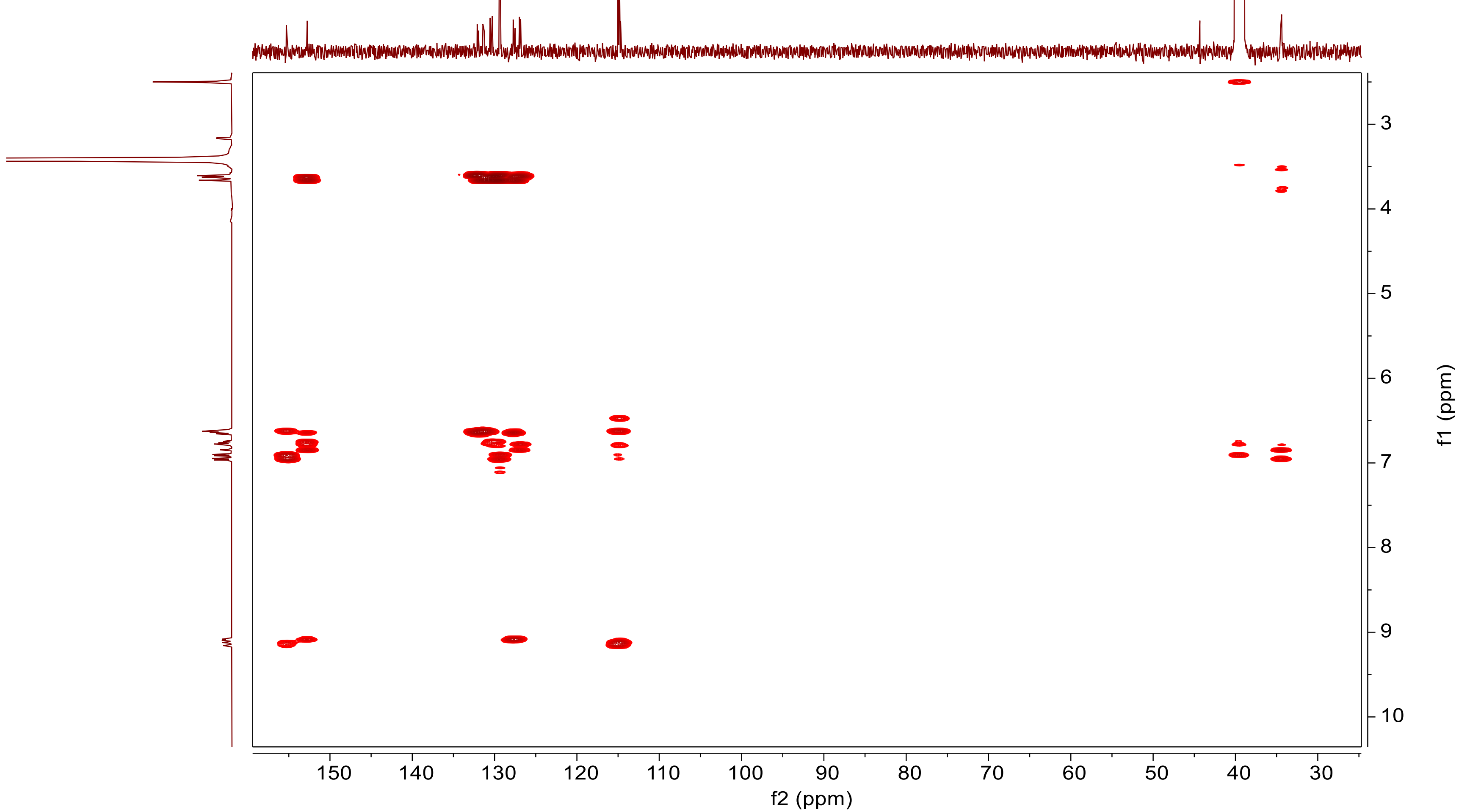

Figure S54. The HMBC spectrum of compound **4** in DMSO-d_6 (500 MHz for ^{1}H)

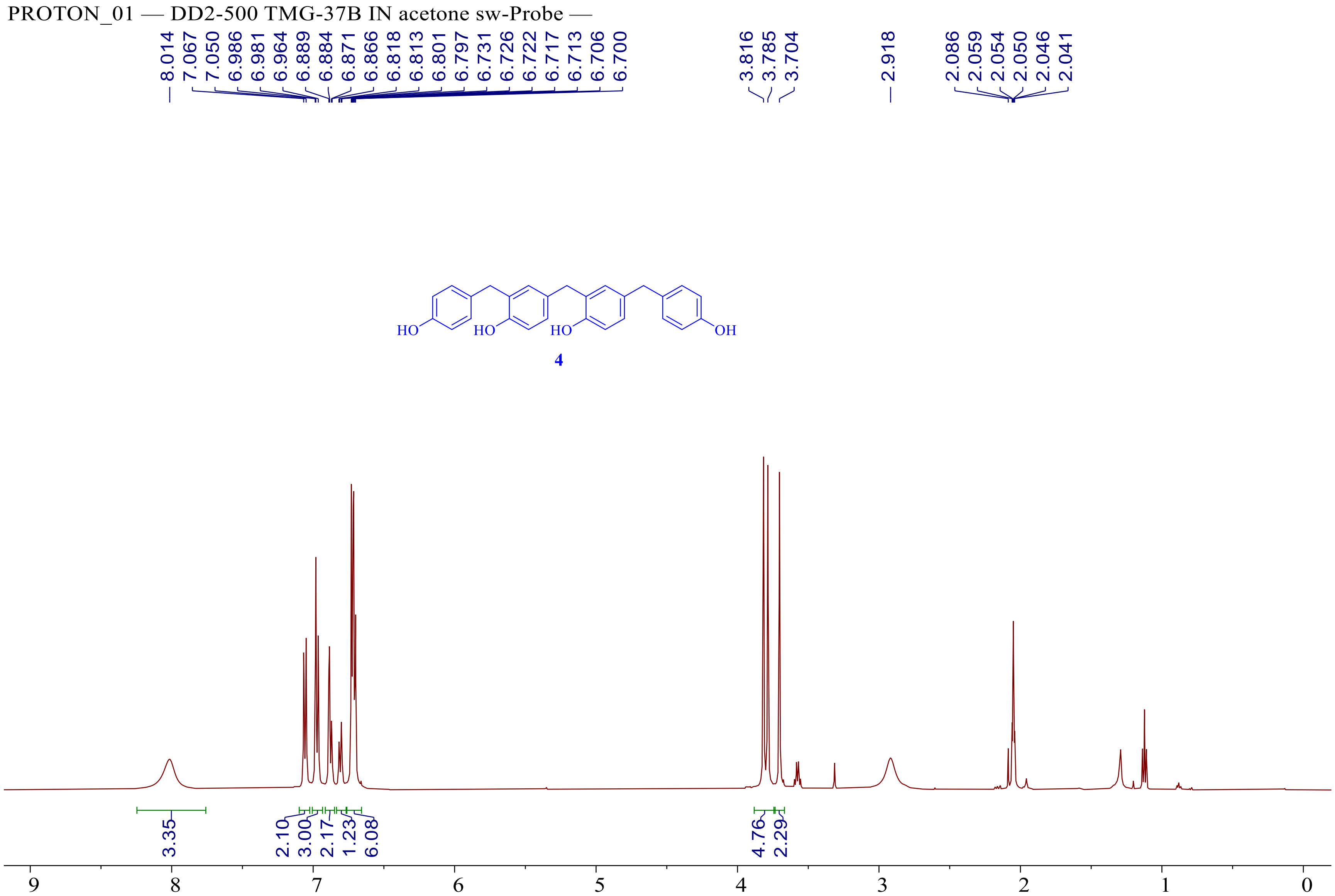

Figure S55. The ^{1}H NMR spectrum of compound **4** in acetone-d_6 (500 MHz)

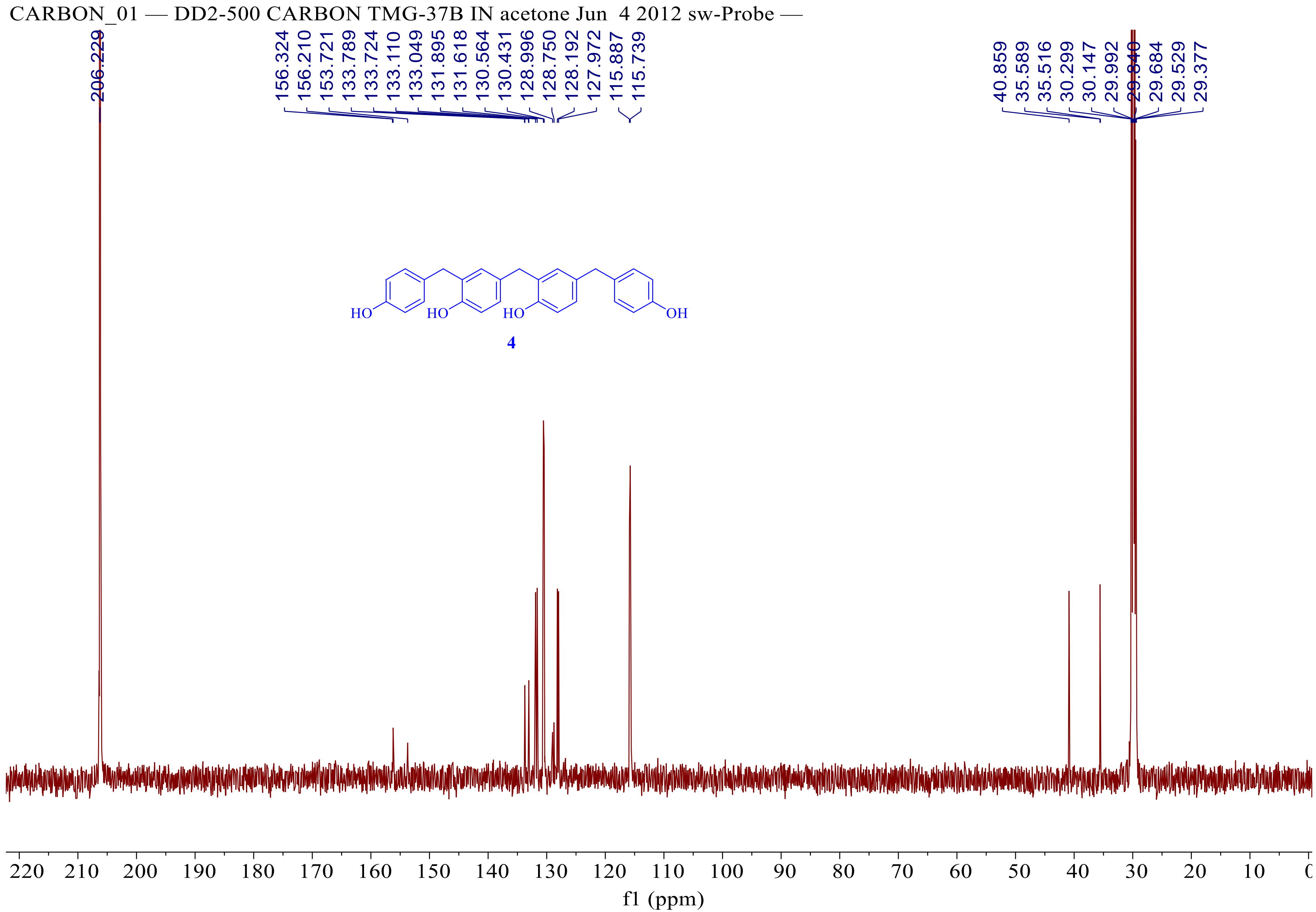

Figure S56. The ^{13}C NMR spectrum of compound **4** in acetone-d_6 (125 MHz)

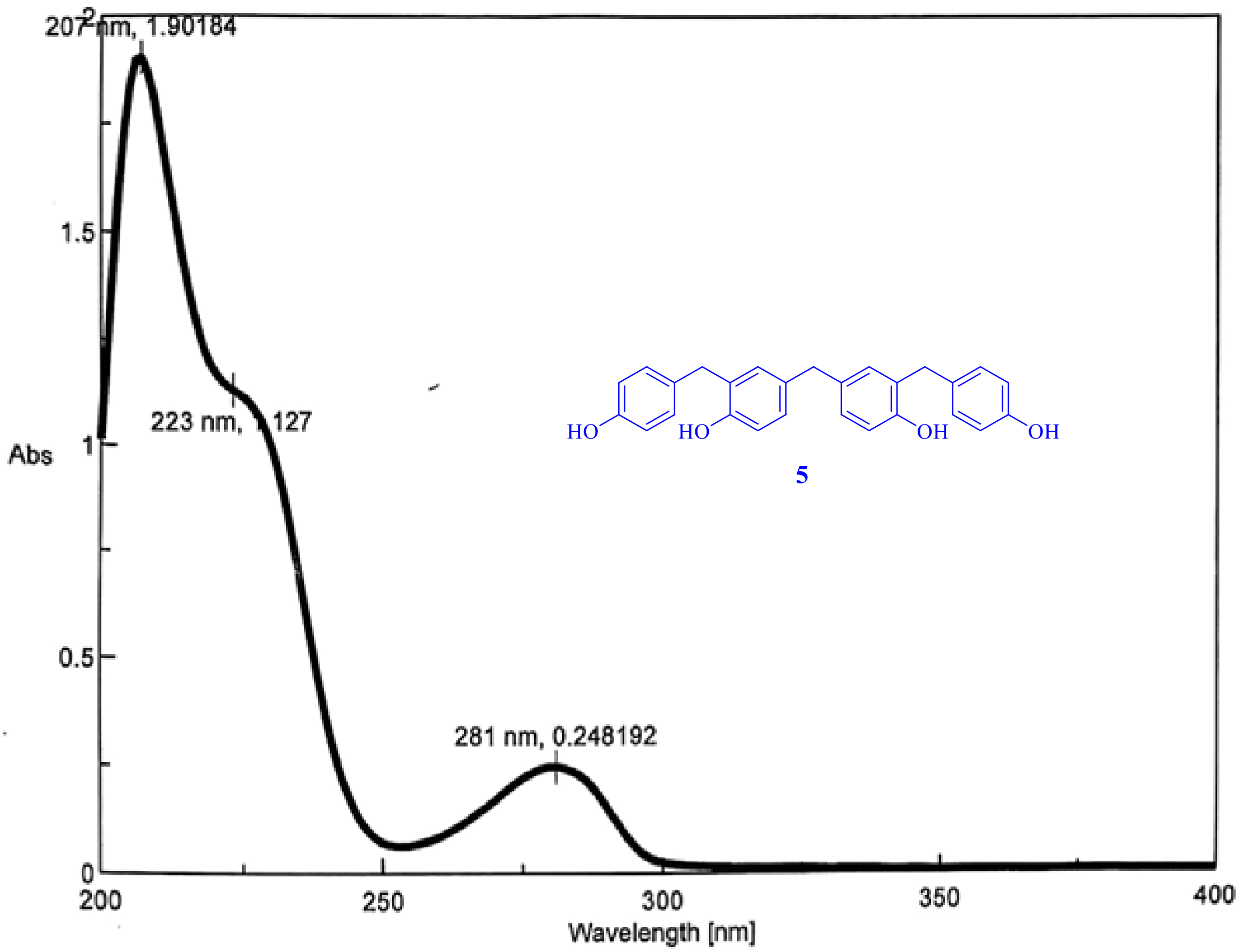

Memory-8

[Comment]	
Sample Name	wyn-27B
Comment	
User	wyn
Division	
Company	324
[Measurement Information]	
Instrument Name	V-650
Model Name	V-650
Serial No.	A034461150
Accessory	PSC-718
Accessory S/N	A001761114
Position	1
Cell Length	10 mm
Temperature	19.96 C
Control Sonsor	Holder
Monitor Sensor	Holder
Start Mode	Start immediately
Photometric Mode	Abs
Measurement range	400 - 195 nm
Data pitch	1 nm
Band width(UV/Vis)	1.0 nm
Response	Medium
Scanning speed	200 nm/min
Source Change	340 nm
Light Source	D2/WI
Filter Exchange	Step
Correction	Baseline

[Data Information]	
Creation Date	2011-10-12 14:48
Data array type	Linear data array
Horizontal	Wavelength [nm]
Vertical	Abs
Start	400 nm
End	195 nm
Data pitch	1 nm
Data points	206

Figure S57. The UV spectrum of compound **5**

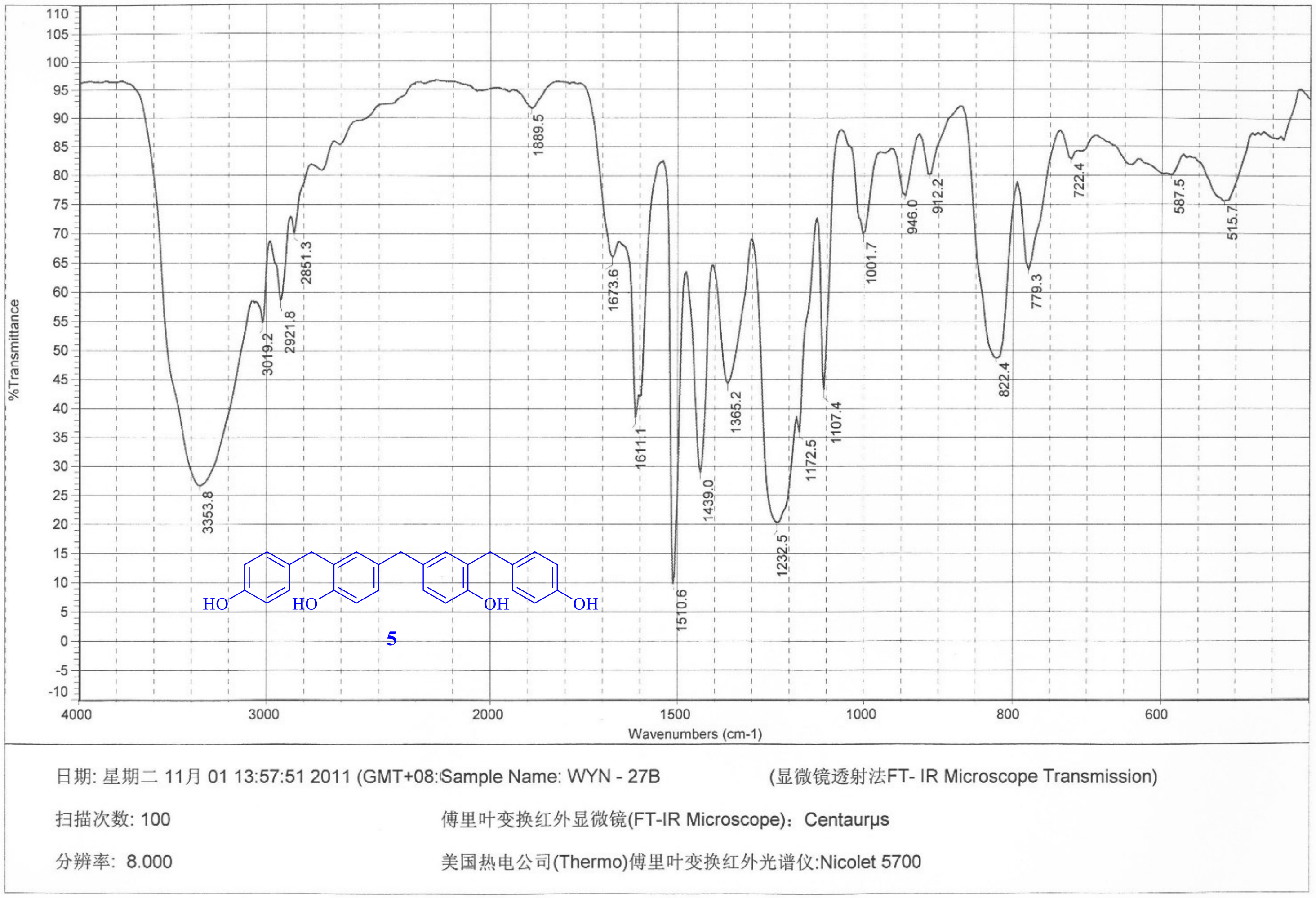

Figure S58. The IR spectrum of compound **5**

Single Mass Spectrum Deconvolution Report

Analysis Name: wangy030.d **Instrument:** LC-MSD-Trap-SL **Print Date:** 1/5/2011 9:44:11 AM
Method: def_lcms.m **Operator:** Operator **Acq. Date:** 1/5/2011 9:37:38 AM
Sample Name: WYN-27b
Analysis Info:

Acquisition Parameter:

Mass Range Mode	Std/Normal	Trap Drive	53.0	Scan Begin	100 m/z
Ion Polarity	Positive	Octopole RF Amplitude	171.0 Vpp	Scan End	1000 m/z
Ion Source Type	ESI	Capillary Exit	-106.0 Volt	Averages	5 Spectra
Dry Temp (Set)	330 °C	Skimmer	-40.0 Volt	Max. Accu Time	200000 µs
Nebulizer (Set)	15.00 psi	Oct 1 DC	-12.00 Volt	ICC Target	10000
Dry Gas (Set)	5.00 l/min	Oct 2 DC	-1.70 Volt	Charge Control	on

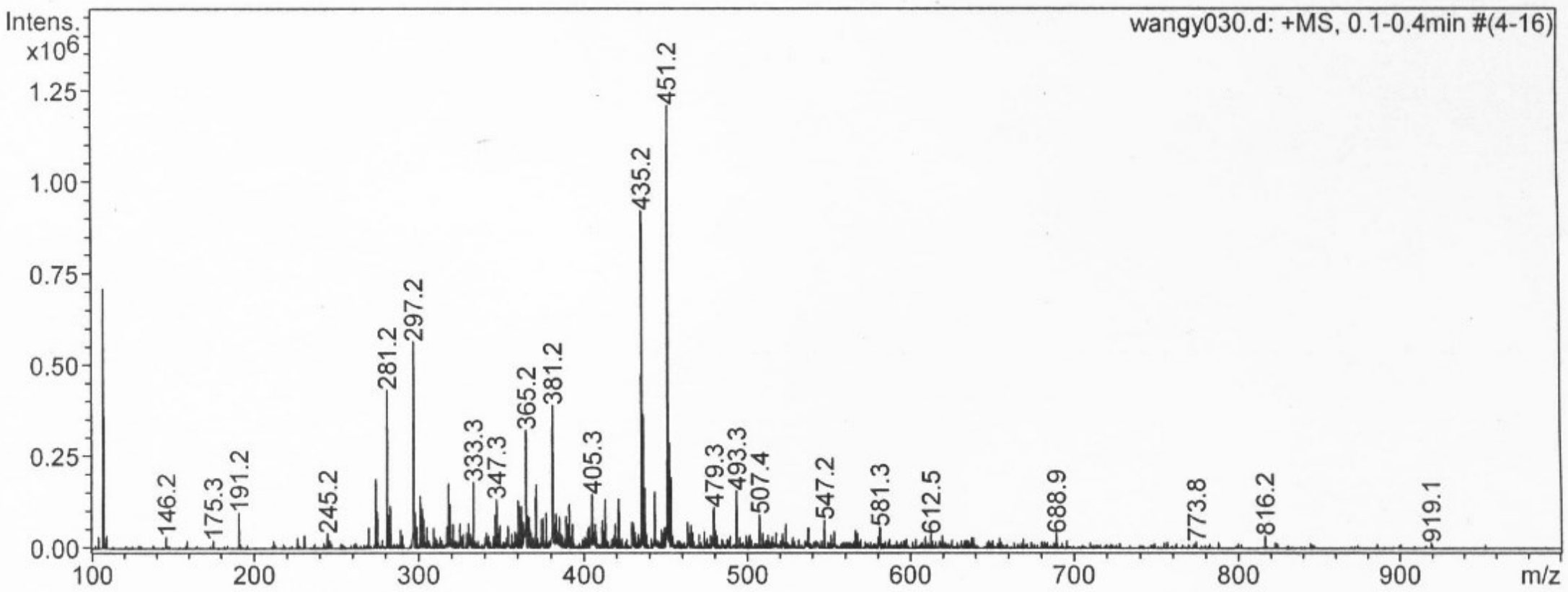

Component Molecular Mass Molecule Absolute Abundance Relative Abundance

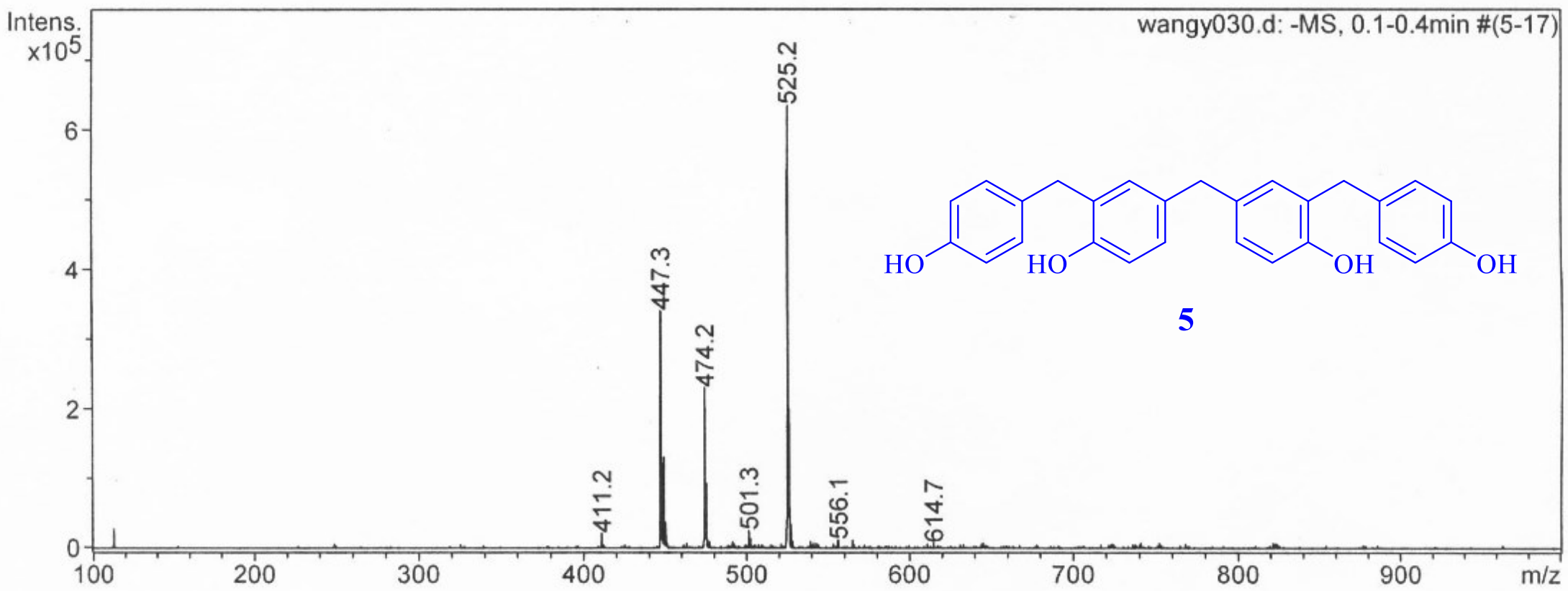

Component Molecular Mass Molecule Absolute Abundance Relative Abundance

MSD Trap Report v 4 (A4-Opt2) Page 1 of 1 Agilent Technologies

Figure S59. The ESIMS of compound **5**

Qualitative Analysis Report

**Data Filename**	201105051.d	**Sample Name**	WYN-27b
**Sample Type**	Sample	**Position**	P1-B1
**Instrument Name**	Instrument 1	**User Name**	
**Acq Method**		**IRM Calibration Status**	Success
**DA Method**	TEST LCMS.m	**Comment**	

User Chromatograms

Fragmentor Voltage 135 **Collision Energy** 0 **Ionization Mode** ESI

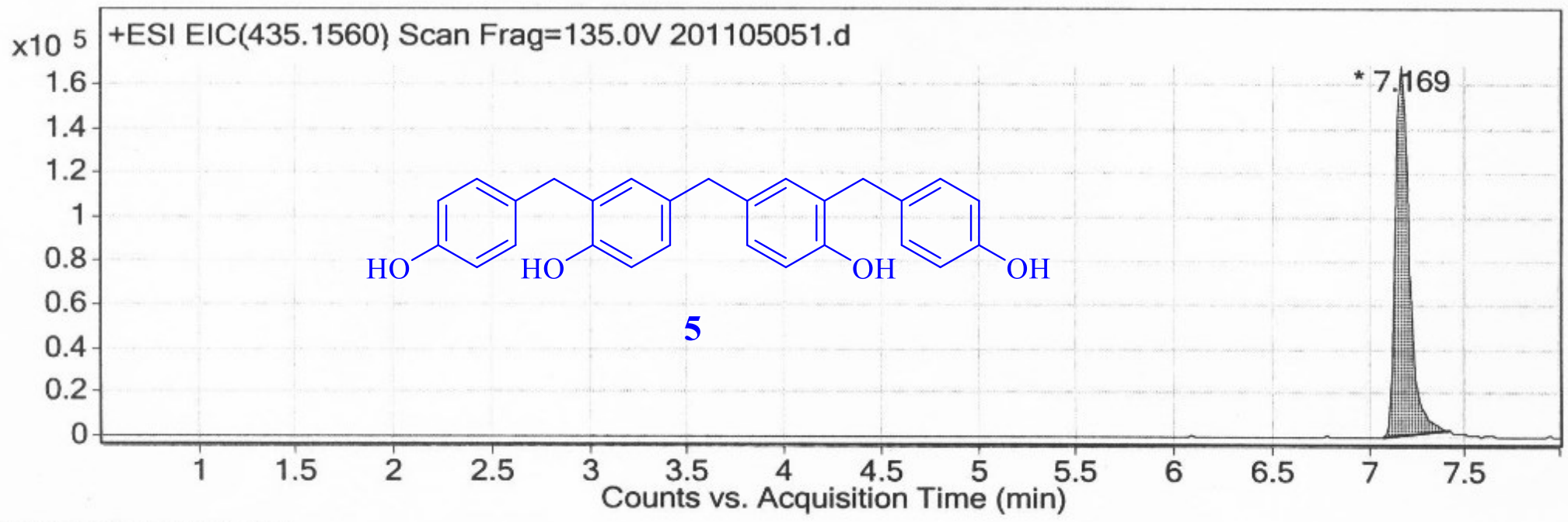

Integration Peak List

Peak	Start	RT	End	Height	Area	Area %
1	7.072	7.169	7.426	167803	877877	100

User Spectra

Fragmentor Voltage	Collision Energy	Ionization Mode
135	0	Esi

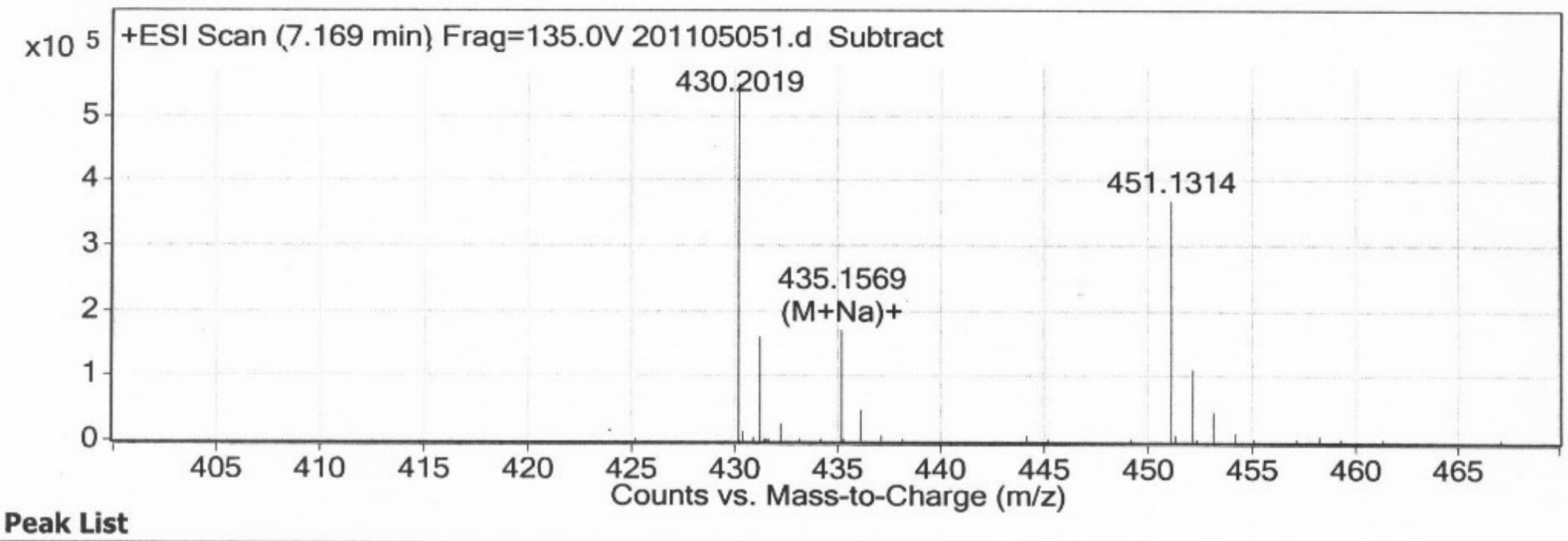

Peak List

m/z	z	Abund	Formula	Ion
107.0412		90414		
163.0388		51970		
237.1121		99951		
259.094		87430		
430.2019	1	548572		
431.205	1	161604		
435.1569	1	168630	C27 H24 Na O4	(M+Na)+
436.1603	1	46640	C27 H24 Na O4	(M+Na)+
451.1314	1	368779		
452.1341	1	109277		

Formula Calculator Element Limits

Element	Min	Max
C	3	100
H	0	120
O	0	30

Agilent Technologies Page 1 of 2 Printed at: 2:30 PM on: 5/5/2011

Figure S60. The HR-ESIMS report of compound **5** (page 1)

Qualitative Analysis Report

Element	Min	Max
N	0	2
S	0	0
Cl	0	0

Formula Calculator Results

Formula	Best	Mass	Tgt Mass	Diff (ppm)	Ion Species	Score
C27 H24 O4	TRUE	412.1677	412.1675	-0.6	C27 H24 Na O4	99.78
C15 H28 N2 O11		412.1677	412.1693	3.88	C15 H28 N2 Na O11	97.05

--- End Of Report ---

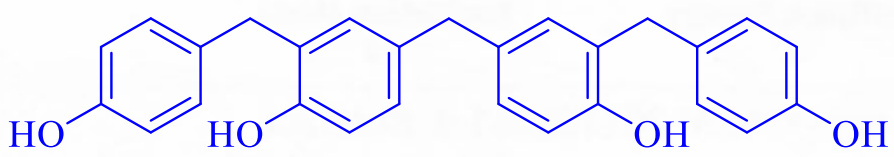

5

Figure S61. The HR-ESIMS report of compound **5** (page 2)

MS Formula Results: + Scan (7.169 min) Sub (201105051.d)

m/z	Ion	Formula	Abundance
435.1569	(M+Na)+	C27 H24 Na O4	168629.5

Best	Formula (M)	Ion Formula	Calc m/z	Score	Cross S	Mass	Calc Mass	Diff (ppm)	Abs Diff (ppm)	Abund Match	Spacing Mat	Mass Match	m/z	DBE
☑	C27 H24 O4	C27 H24 Na O4	435.1567	99.78		412.1677	412.1675	-0.6	0.6	99.51	99.68	99.99	435.1569	16
☐	C15 H28 N2 O11	C15 H28 N2 Na O11	435.1585	97.05		412.1677	412.1693	3.88	3.88	90.87	99.46	99.55	435.1569	3

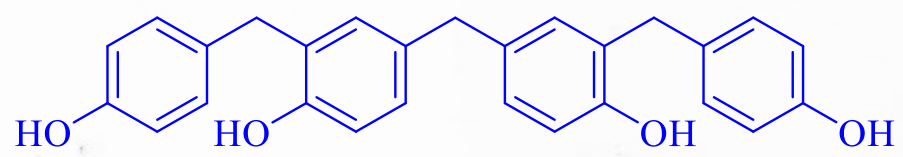

5

page 1

Figure S62. The HR-ESIMS report of compound **5** (page 3)

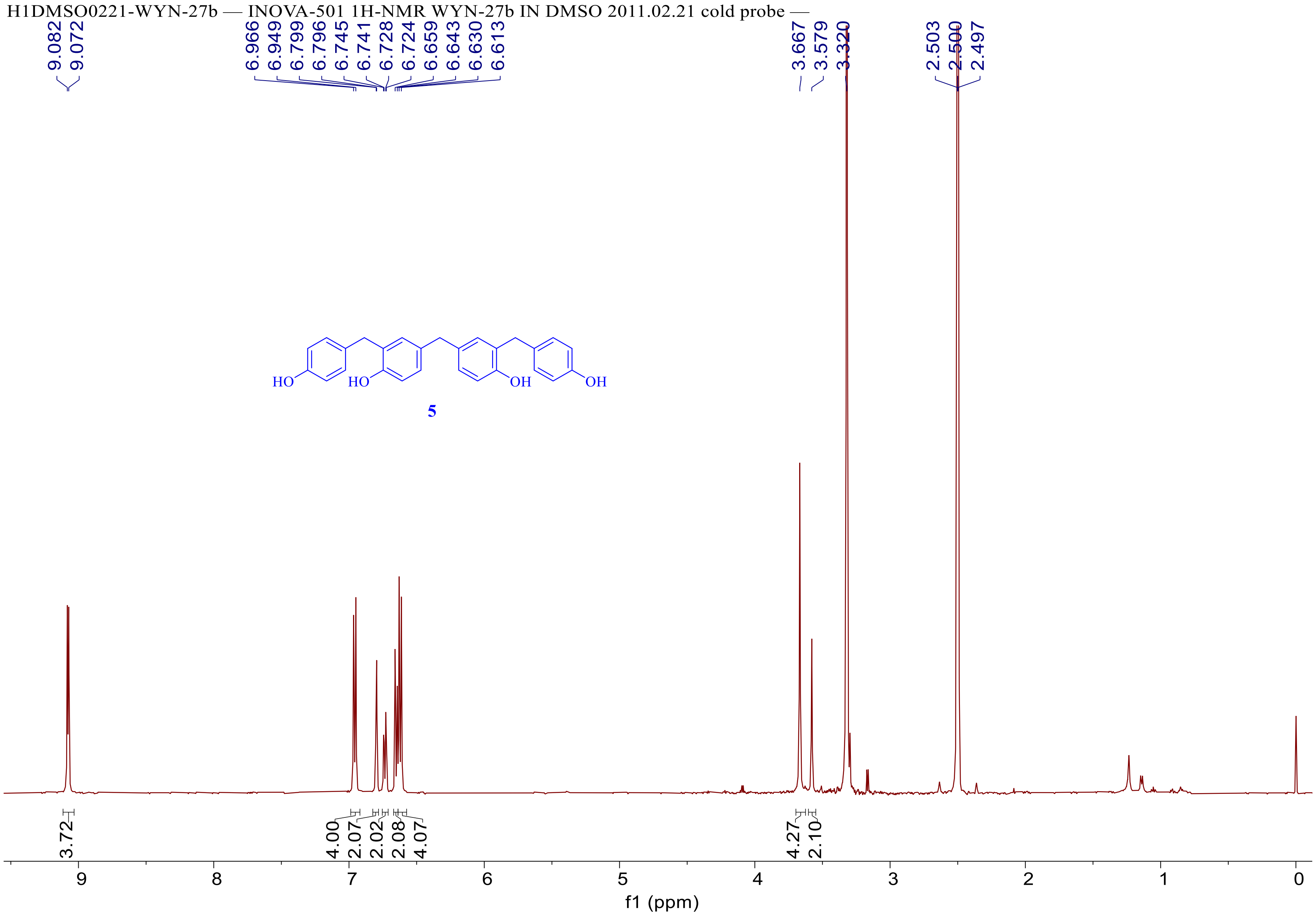

Figure S63. The ^{1}H NMR spectrum of compound **5** in DMSO-d_6 (500 MHz)

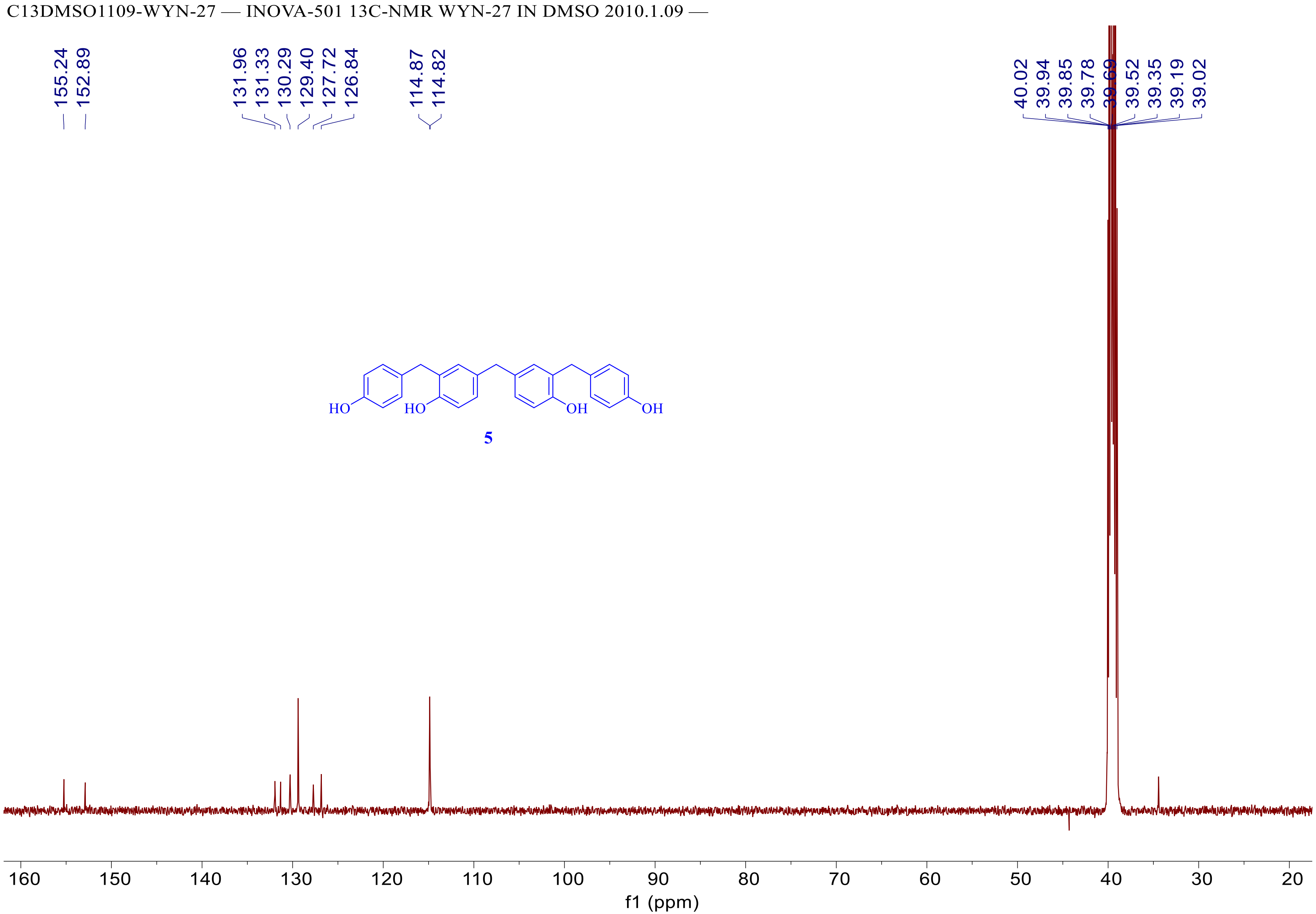

Figure S64. The ^{13}C NMR spectrum of compound **5** in DMSO-d_6 (125 MHz)

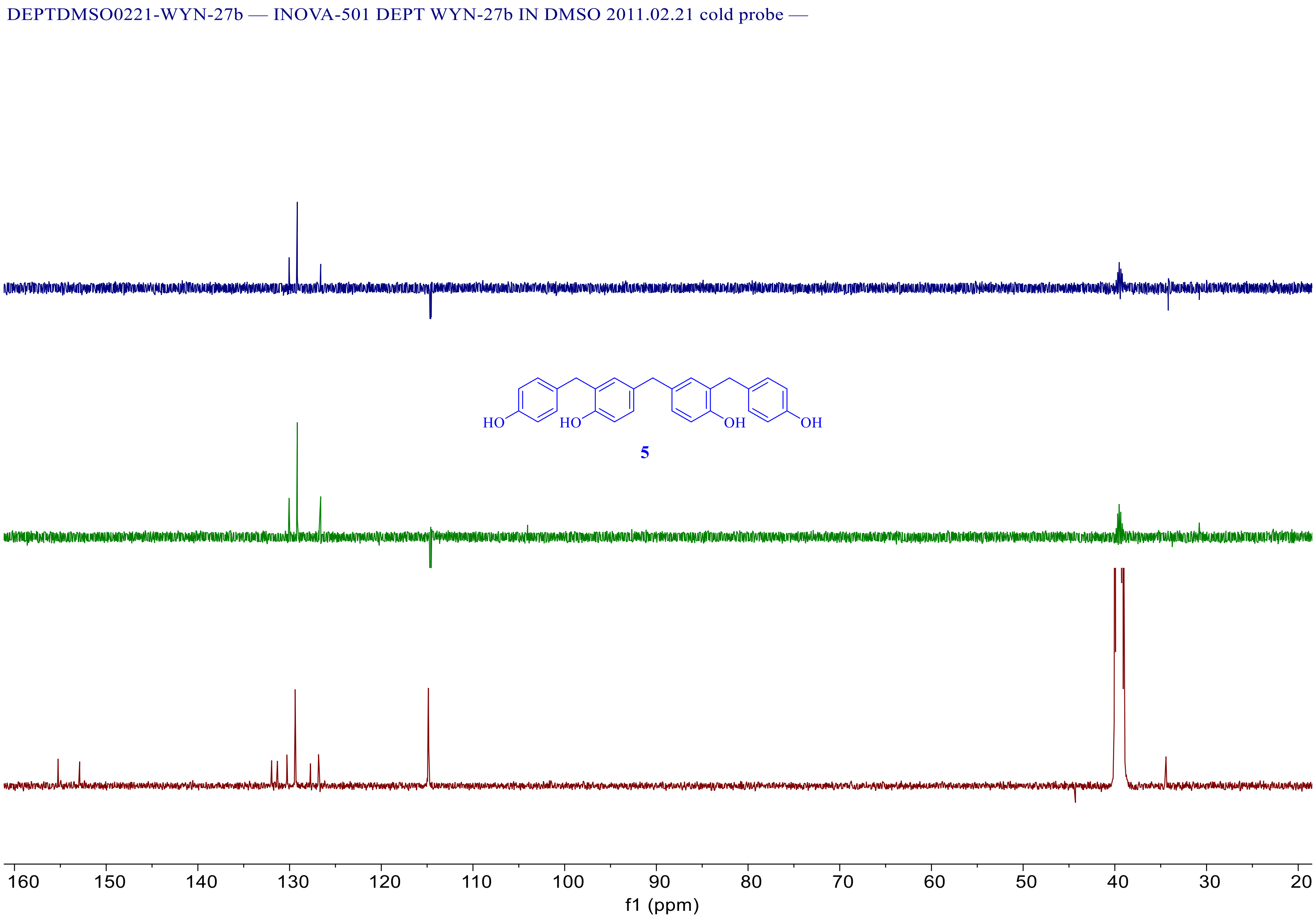

Figure S65. The DEPT spectrum of compound **5** in DMSO-d_6 (125 MHz)

COSYDMSO0124-WYN-27b — INOVA-501 gCOSY WYN-27b IN DMSO 2911.01.24 cold probe —

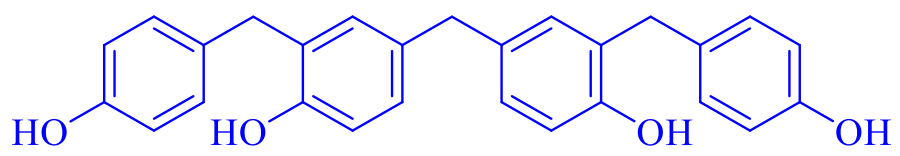

5

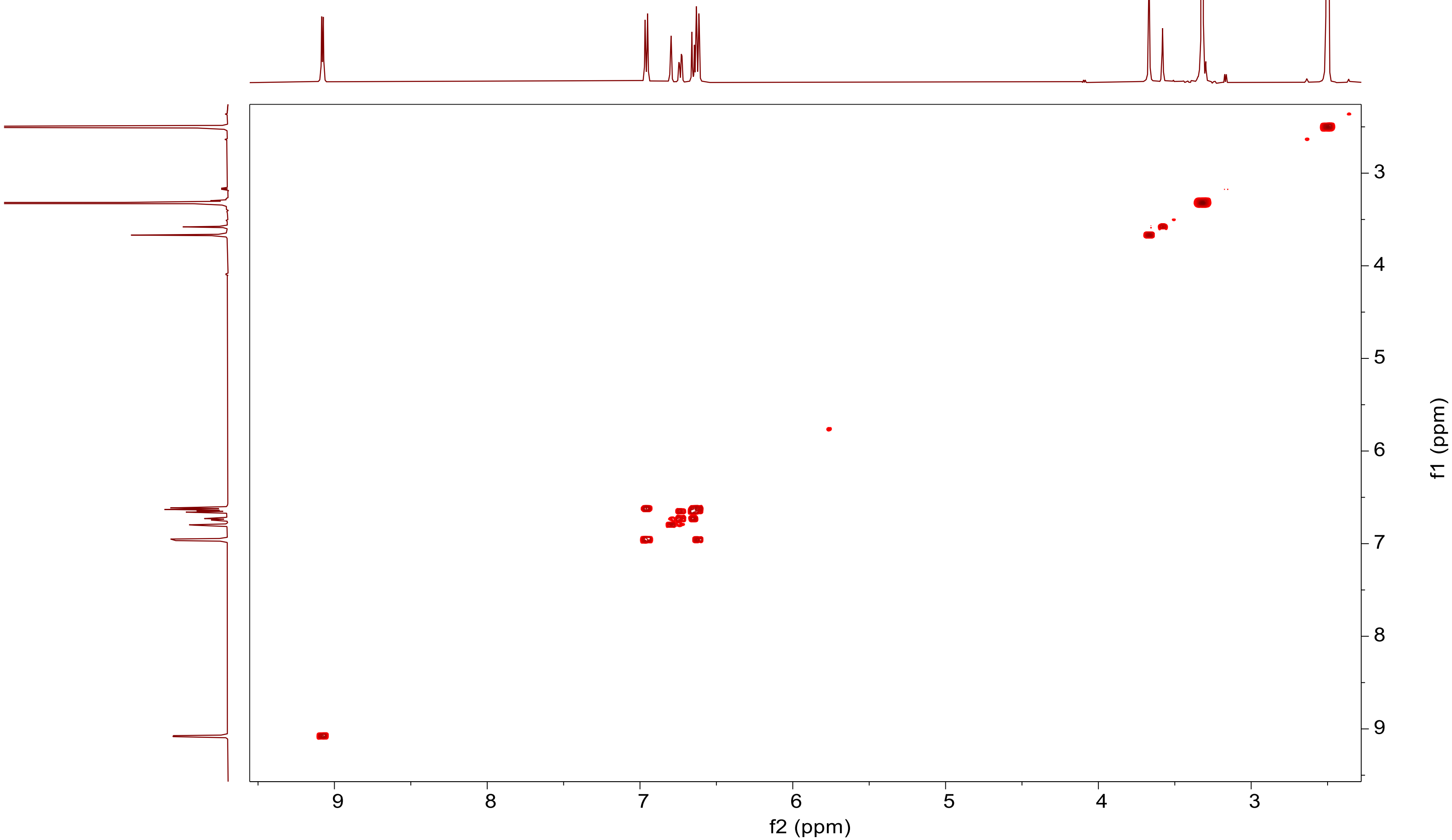

Figure S66. The ^{1}H-^{1}H COSY spectrum of compound **5** in DMSO-d_6 (500 MHz)

HSQCDMSO0221-WYN-27b — INOVA-501 gHSQC WYN-27b IN DMSO 2011.02.21 cold probe —

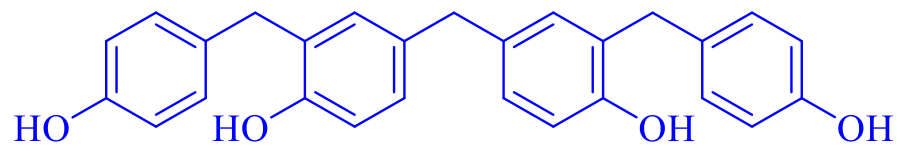

5

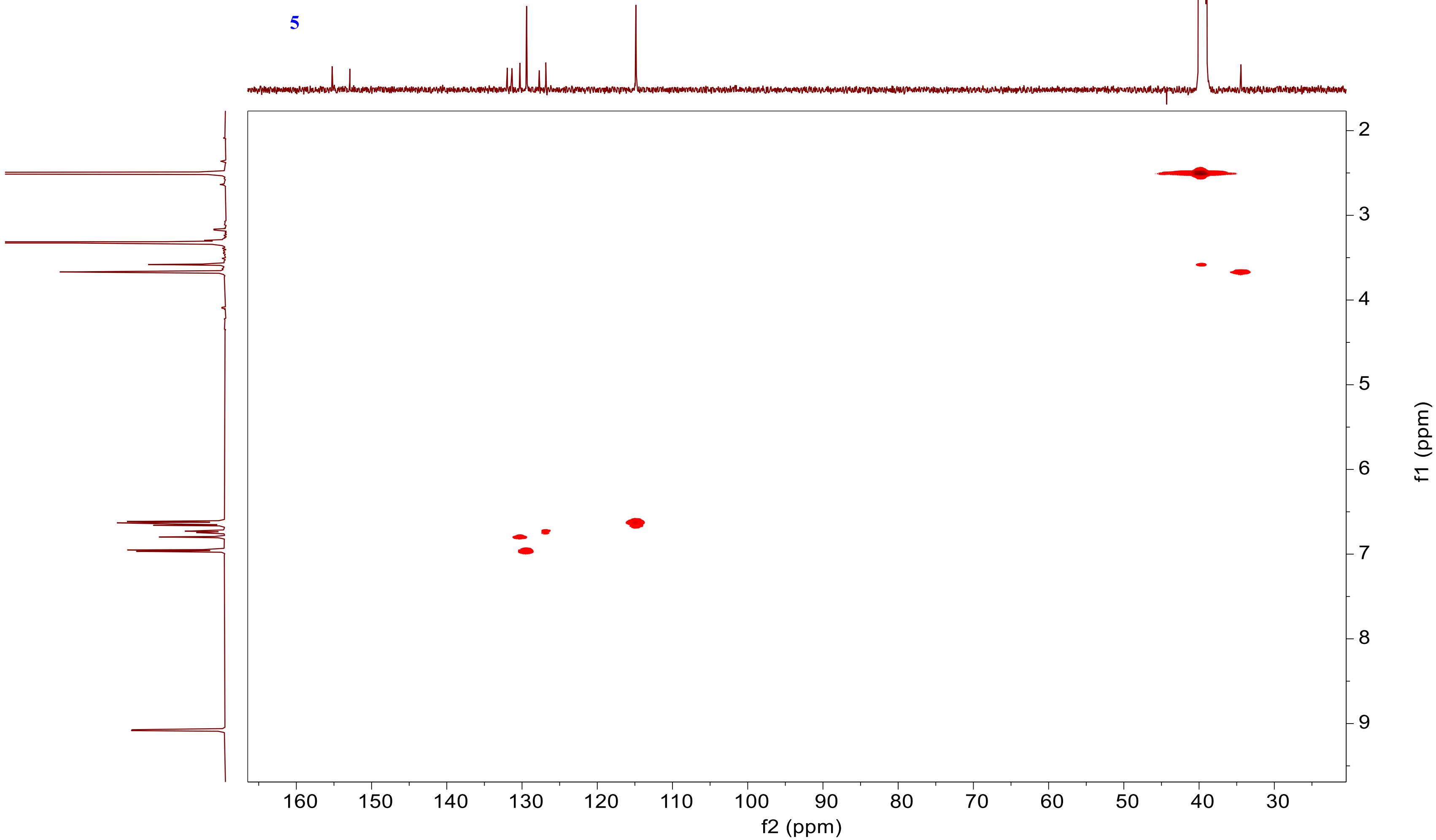

Figure S67. The HSQC spectrum of compound **5** in DMSO-d_6 (500 MHz for ^{1}H)

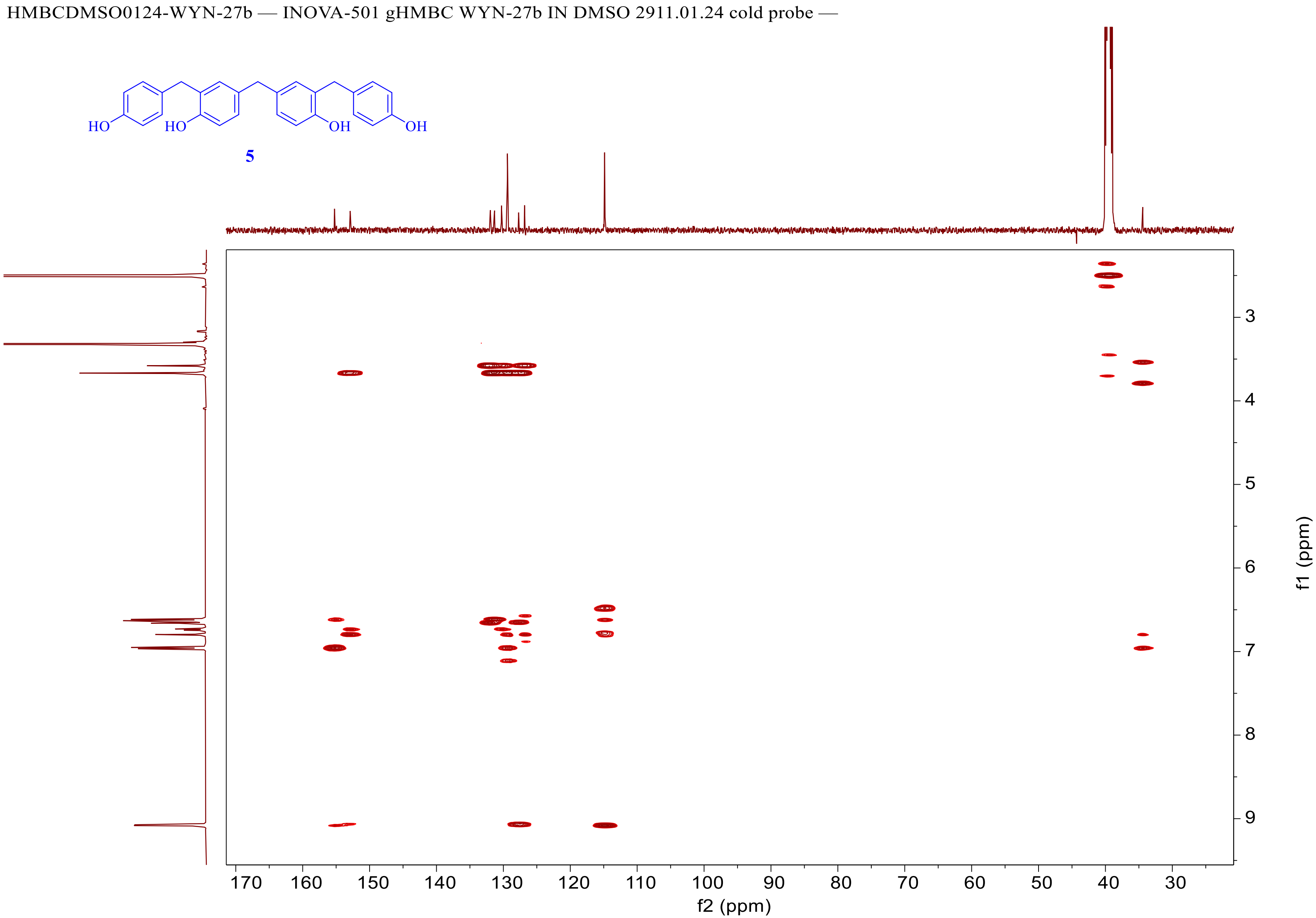

Figure S68. The HMBC spectrum of compound **5** in DMSO-d_6 (500 MHz for ^{1}H)

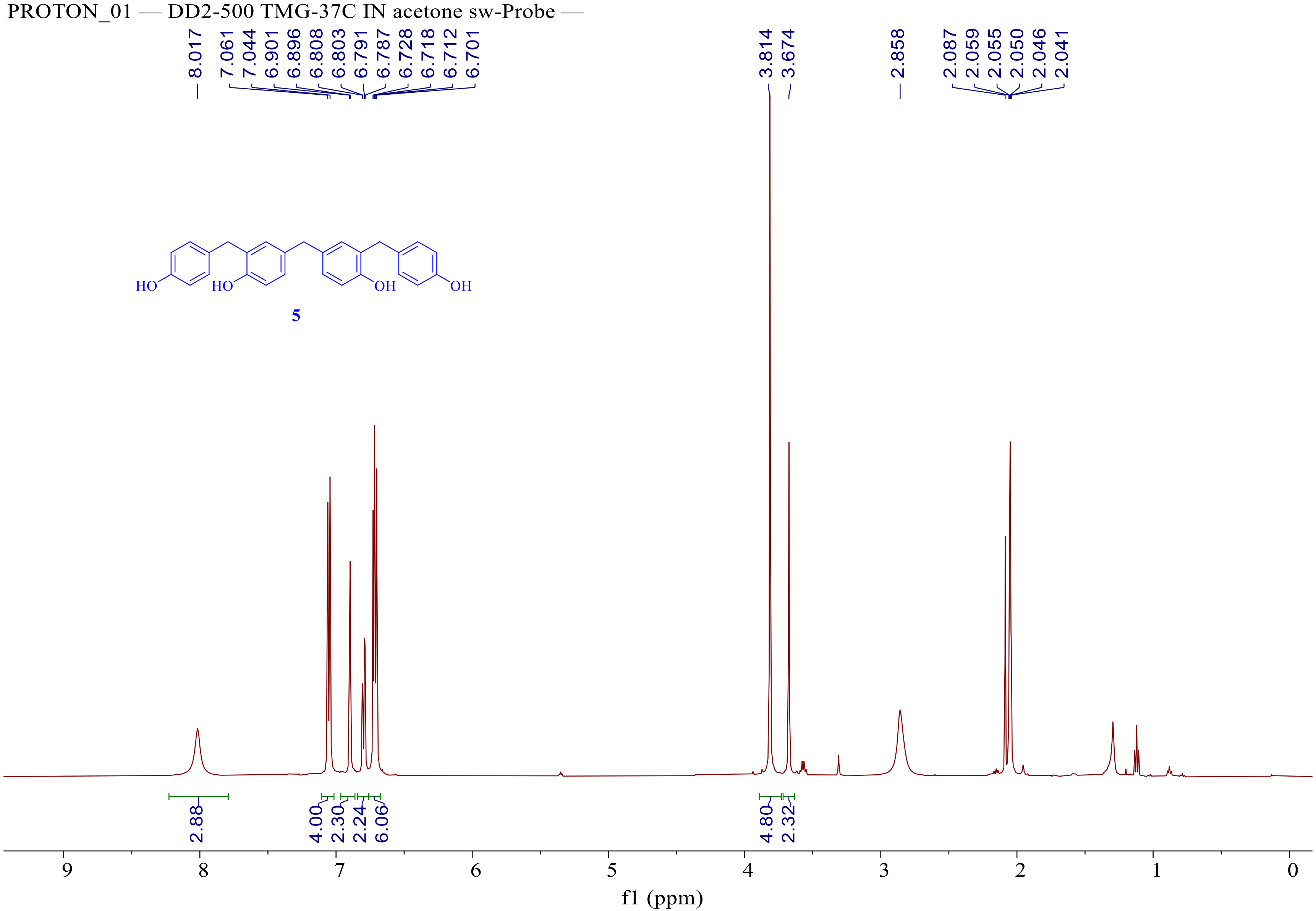

Figure S69. The ^{1}H NMR spectrum of compound **5** in acetone-d_6 (500 MHz)

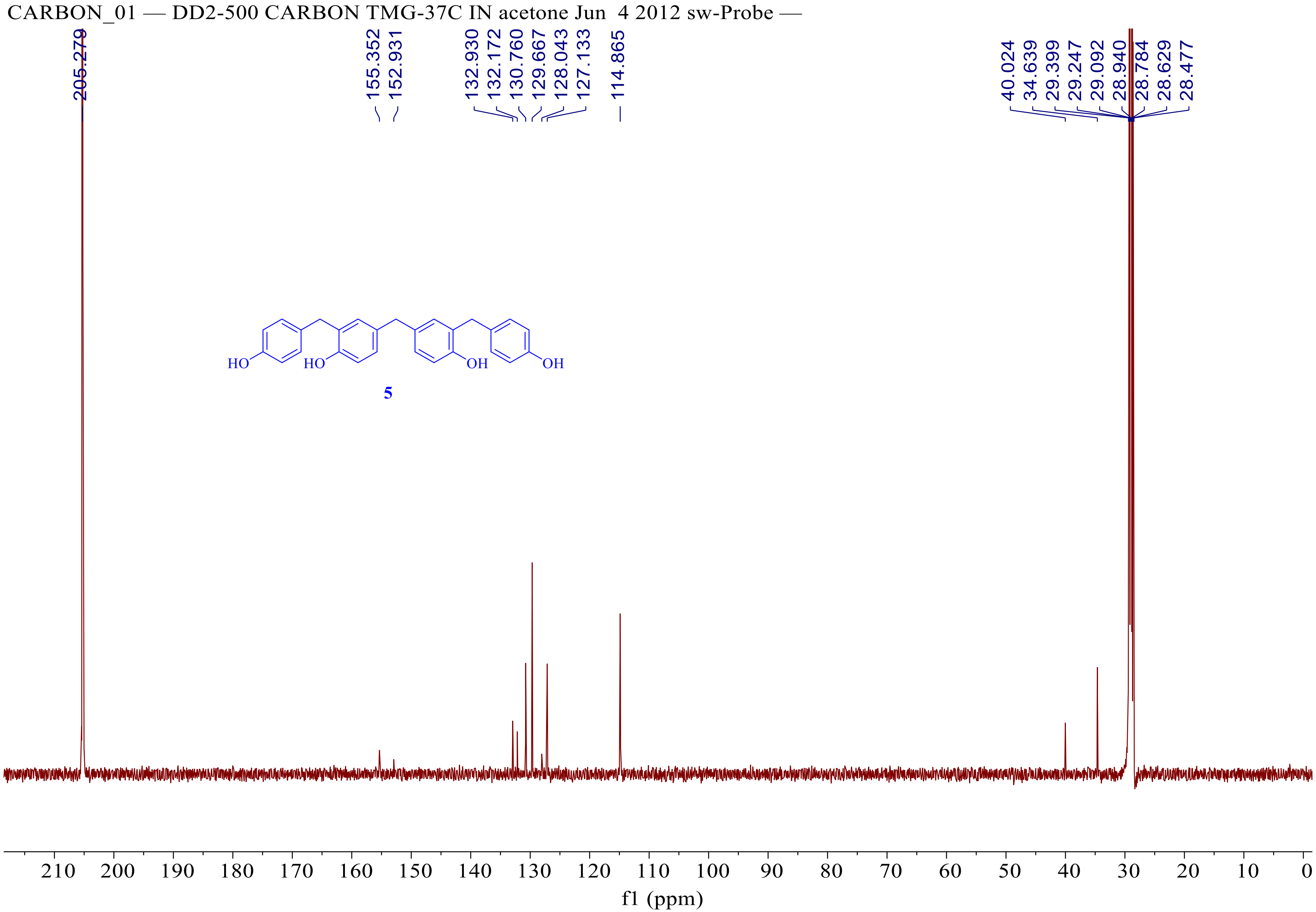

Figure S70. The ^{13}C NMR spectrum of compound **5** in acetone-d_6 (125 MHz)

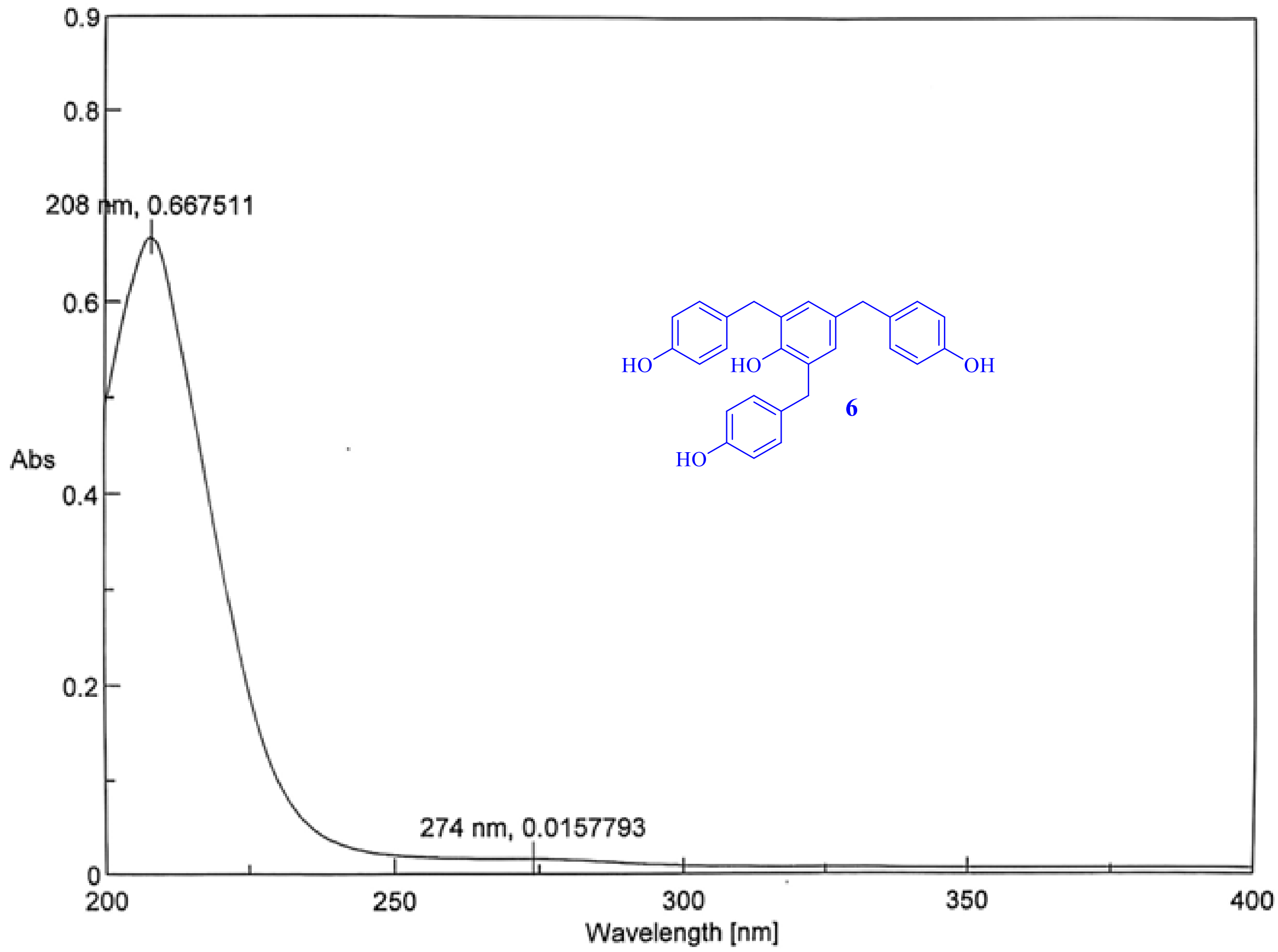

wyn-32-7-w

[Comment]

Sample Name	wyn-32
Comment	
User	王亚男
Division	
Company	324

[Measurement Information]

Instrument Name	V-650
Model Name	V-650
Serial No.	A034461150
Accessory	PSC-718
Accessory S/N	A001761114
Position	1
Cell Length	10 mm
Temperature	19.98 C
Control Sonsor	Holder
Monitor Sensor	Holder
Start Mode	Start immediately
Photometric Mode	Abs
Measurement range	400 - 190 nm
Data pitch	1 nm
Band width(UV/Vis)	1.0 nm
Response	Medium
Scanning speed	200 nm/min
Source Change	340 nm
Light Source	D2/WI
Filter Exchange	Step
Correction	Baseline

[Data Information]

Creation Date	2011-5-11 19:01
Data array type	Linear data array
Horizontal	Wavelength [nm]
Vertical	Abs
Start	400 nm
End	190 nm
Data pitch	1 nm
Data points	211

Figure S71. The UV spectrum of compound **6** in MeOH

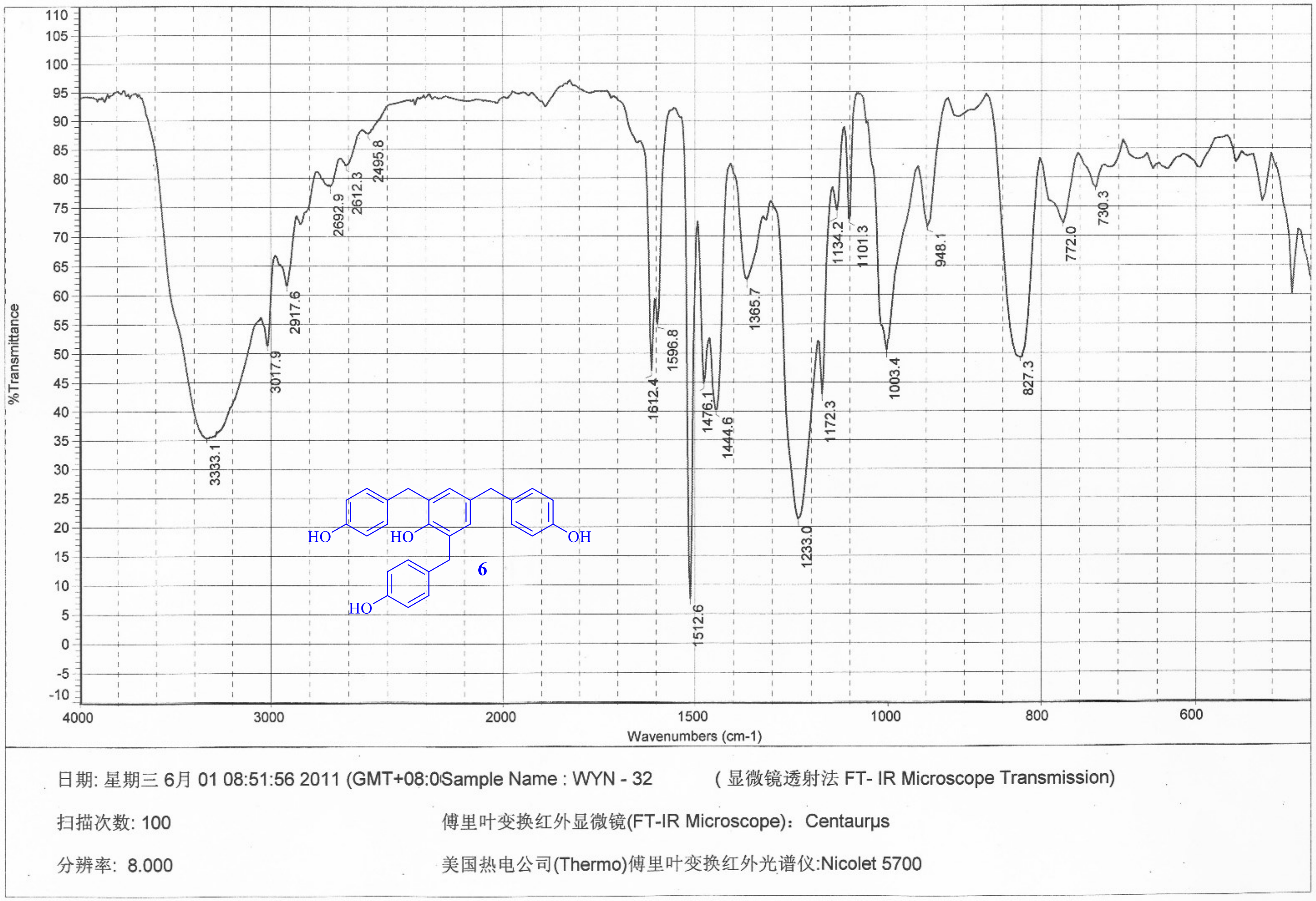

Figure S72. The IR spectrum of compound **6**

Single Mass Spectrum Deconvolution Report

Analysis Name: WANGY005.d **Instrument:** LC-MSD-Trap-SL **Print Date:** 7/9/2010 11:15:47 AM
Method: TEST.MS **Operator:** Operator **Acq. Date:** 7/9/2010 11:11:57 AM
Sample Name: WYN-32
Analysis Info:

Acquisition Parameter:

Mass Range Mode	Std/Normal	Trap Drive	53.0	Scan Begin	100 m/z
Ion Polarity	Positive	Octopole RF Amplitude	171.0 Vpp	Scan End	600 m/z
Ion Source Type	ESI	Capillary Exit	-106.0 Volt	Averages	5 Spectra
Dry Temp (Set)	330 °C	Skimmer	-40.0 Volt	Max. Accu Time	200000 µs
Nebulizer (Set)	15.00 psi	Oct 1 DC	-12.00 Volt	ICC Target	20000
Dry Gas (Set)	5.00 l/min	Oct 2 DC	-1.70 Volt	Charge Control	on

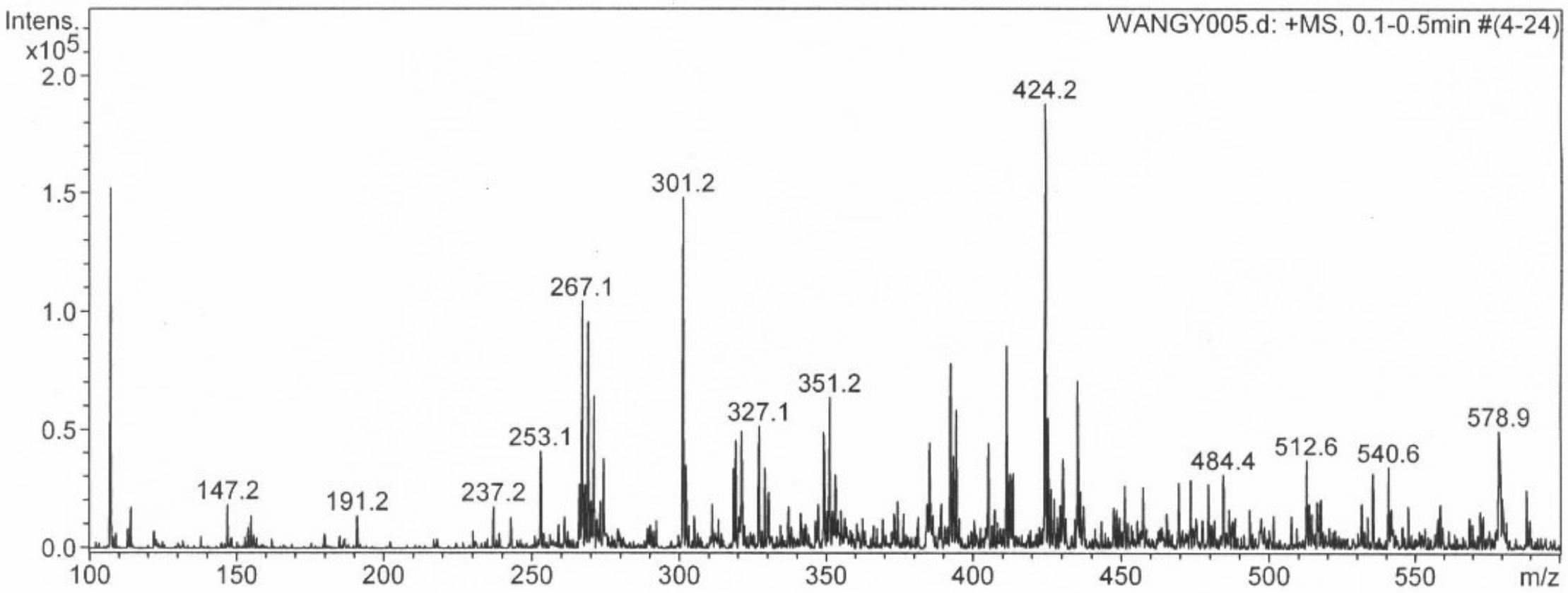

Component	Molecular Mass	Molecule	Absolute Abundance	Relative Abundance

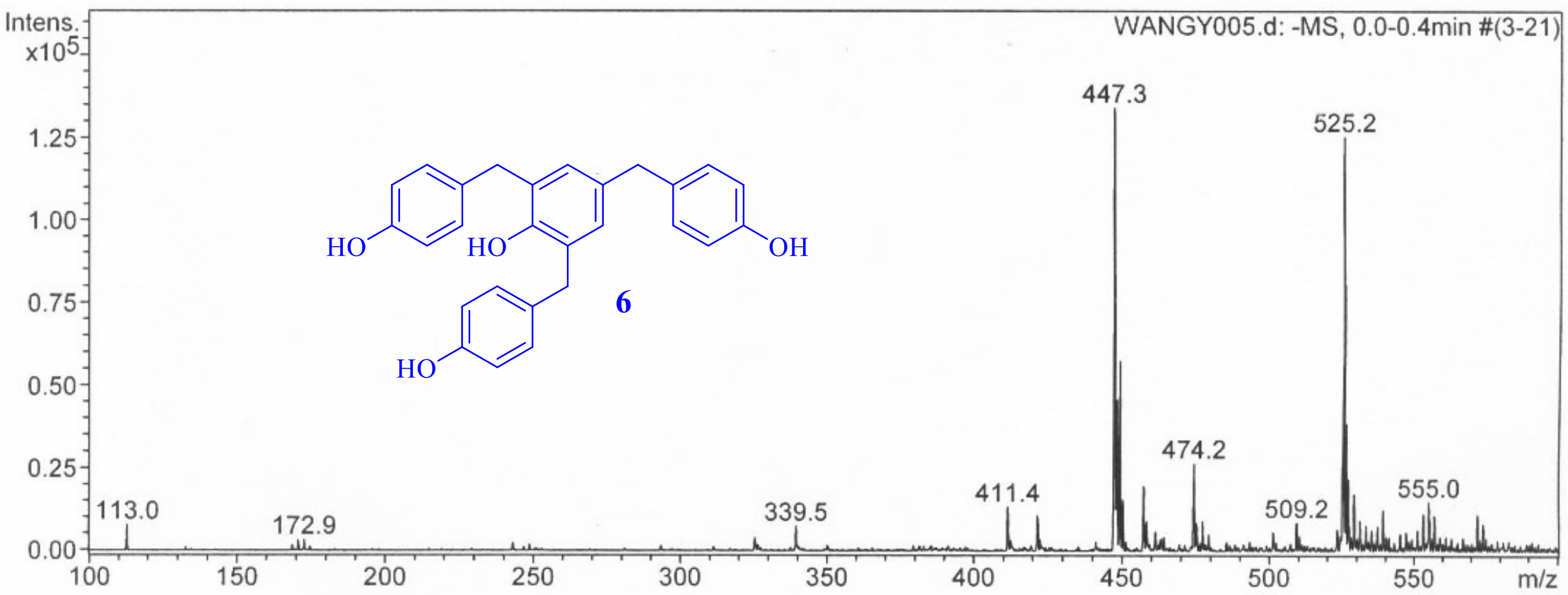

Component	Molecular Mass	Molecule	Absolute Abundance	Relative Abundance

Figure S73. The ESIMS of compound **6**

Qualitative Analysis Report

**Data Filename**	2011011713.d	**Sample Name**	WYN-32
**Sample Type**	Sample	**Position**	P1-D6
**Instrument Name**	Instrument 1	**User Name**	
**Acq Method**		**IRM Calibration Status**	Success
**DA Method**	TEST LCMS.m	**Comment**	

User Chromatograms

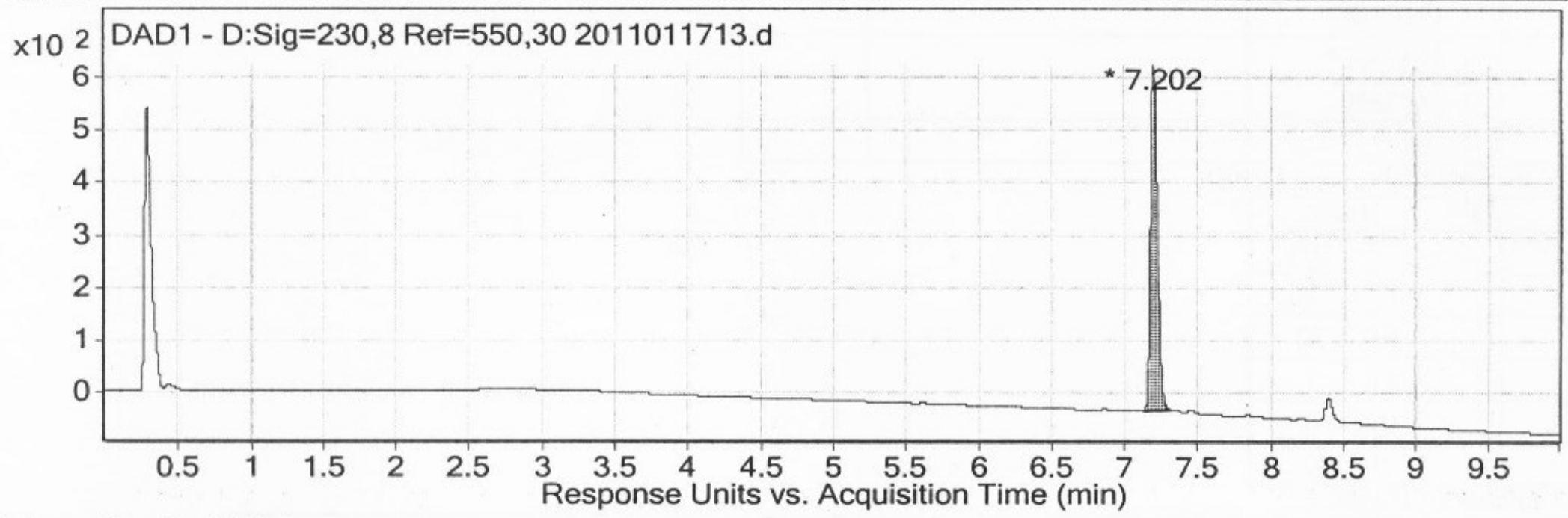

Integration Peak List

Peak	Start	RT	End	Height	Area	Area %
1	7.128	7.202	7.314	661.53	2161.75	100

Fragmentor Voltage 135 **Collision Energy** 0 **Ionization Mode** ESI

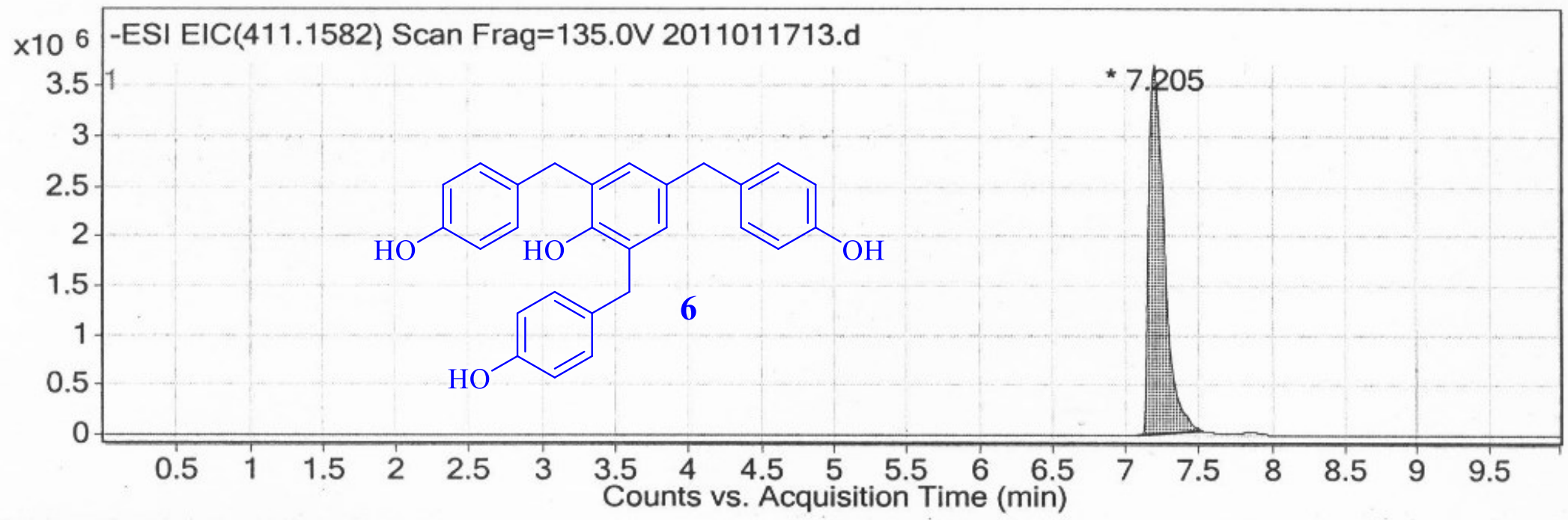

Integration Peak List

Peak	Start	RT	End	Height	Area	Area %
1	7.076	7.205	7.526	3709208	27937600	100

User Spectra

Fragmentor Voltage	Collision Energy	Ionization Mode
135	0	Esi

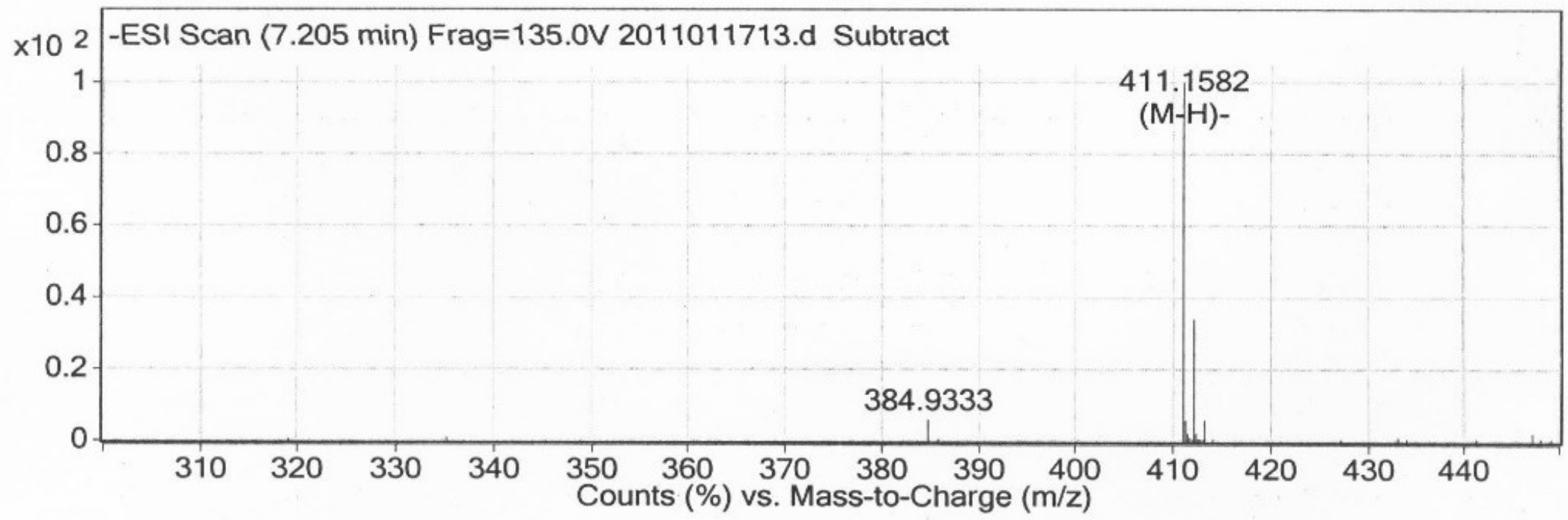

Figure S74. The HR-ESIMS report of compound **6** (page 1)

Qualitative Analysis Report

Peak List

m/z	z	Abund	Formula	Ion
411.1582	1	3719592	C27 H23 O4	(M-H)-
412.1619	1	1265809	C27 H23 O4	(M-H)-
493.1617		526379		
520.9081		904875		
656.8833		563683		
792.8578		473375		
823.3247	1	2261667		
824.3281	1	1420954		
825.3324	1	489486		
845.3075	1	489739		

Formula Calculator Element Limits

Element	Min	Max
C	3	100
H	0	120
O	0	30
N	0	2
S	0	0
Cl	0	0

Formula Calculator Results

Formula	Best	Mass	Tgt Mass	Diff (ppm)	Ion Species	Score
C27 H24 O4	TRUE	412.1655	412.1675	4.77	C27 H23 O4	99.19
C22 H24 N2 O6		412.1655	412.1634	-5.01	C22 H23 N2 O6	97.76
C15 H28 N2 O11		412.1655	412.1693	9.24	C15 H27 N2 O11	92.46

--- End Of Report ---

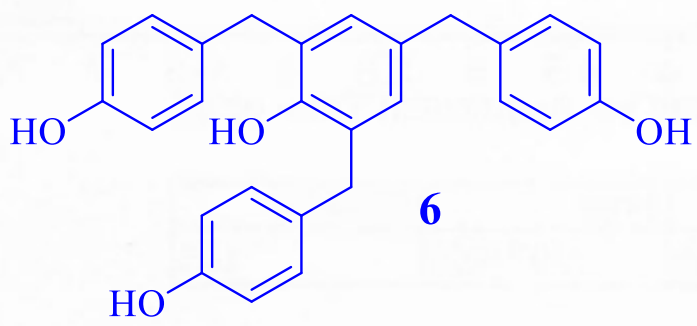

6

Figure S75. The HR-ESIMS report of compound **6** (page 2)

MS Formula Results: - Scan (7.205 min) Sub (2011011713.d)

m/z	Ion	Formula	Abundance
411.1582	(M-H)-	C27 H23 O4	3719592.3

Best	Formula (M)	Ion Formula	Calc m/z	Score	Cross S	Mass	Calc Mass	Diff (ppm)	Abs Diff (ppm)	Abund Match	Spacing Mat	Mass Match	m/z	DBE
☑	C27 H24 O4	C27 H23 O4	411.1602	99.19		412.1655	412.1675	4.77	4.77	98.43	99.98	99.24	411.1582	16
☐	C22 H24 N2 O6	C22 H23 N2 O6	411.1562	97.76		412.1655	412.1634	-5.01	5.01	93.6	99.94	99.17	411.1582	12
☐	C15 H28 N2 O11	C15 H27 N2 O11	411.162	92.46		412.1655	412.1693	9.24	9.24	78.39	99.88	97.19	411.1582	3

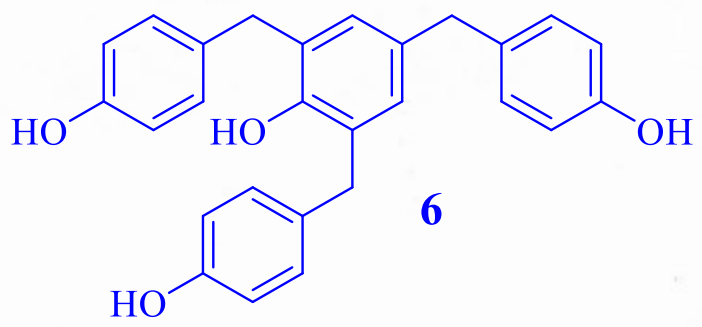

6

page 1

Figure S76. The HR-ESIMS report of compound **6** (page 3)

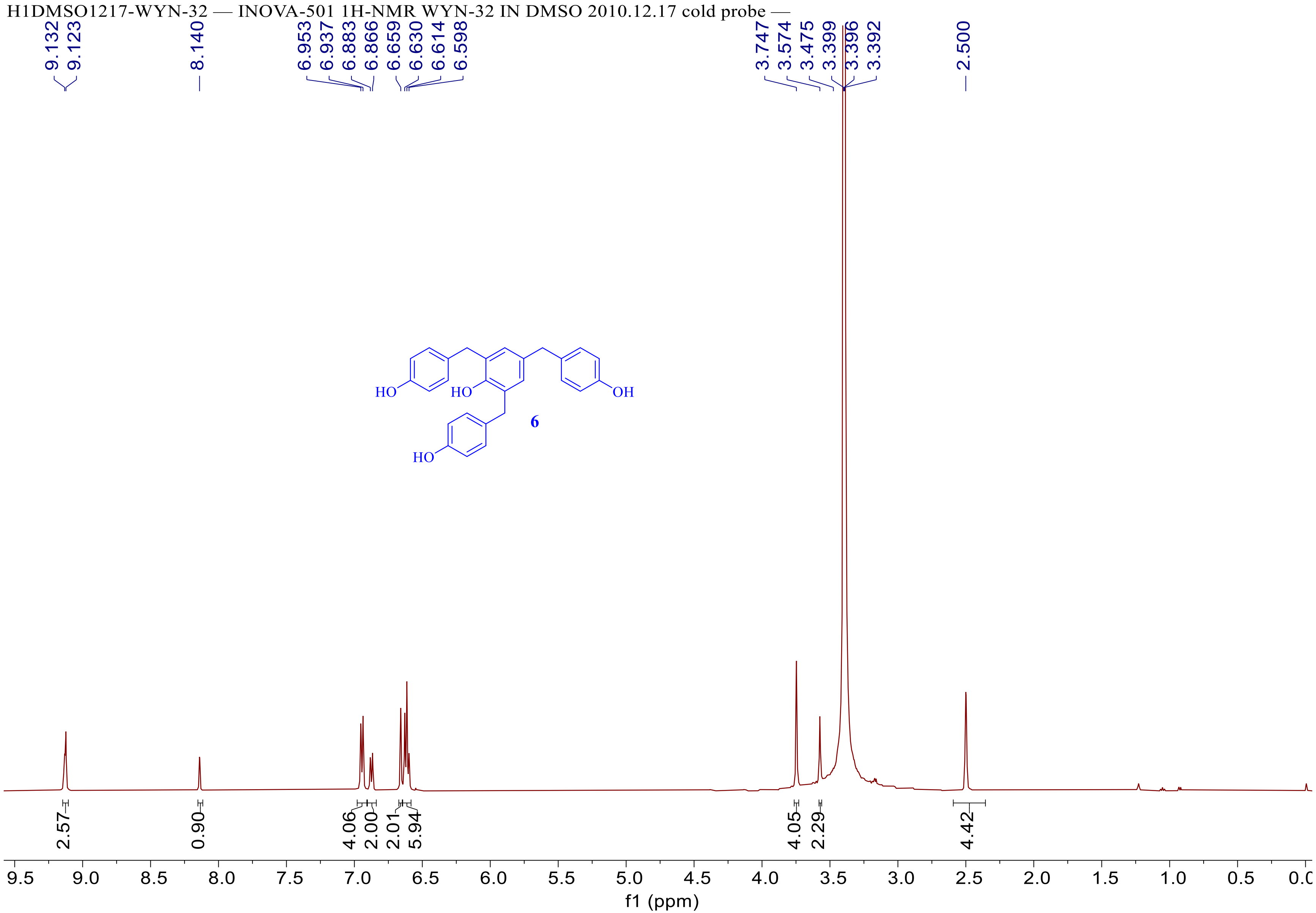

Figure S77. The ^{1}H NMR spectrum of compound **6** in DMSO-d_6 (500 MHz)

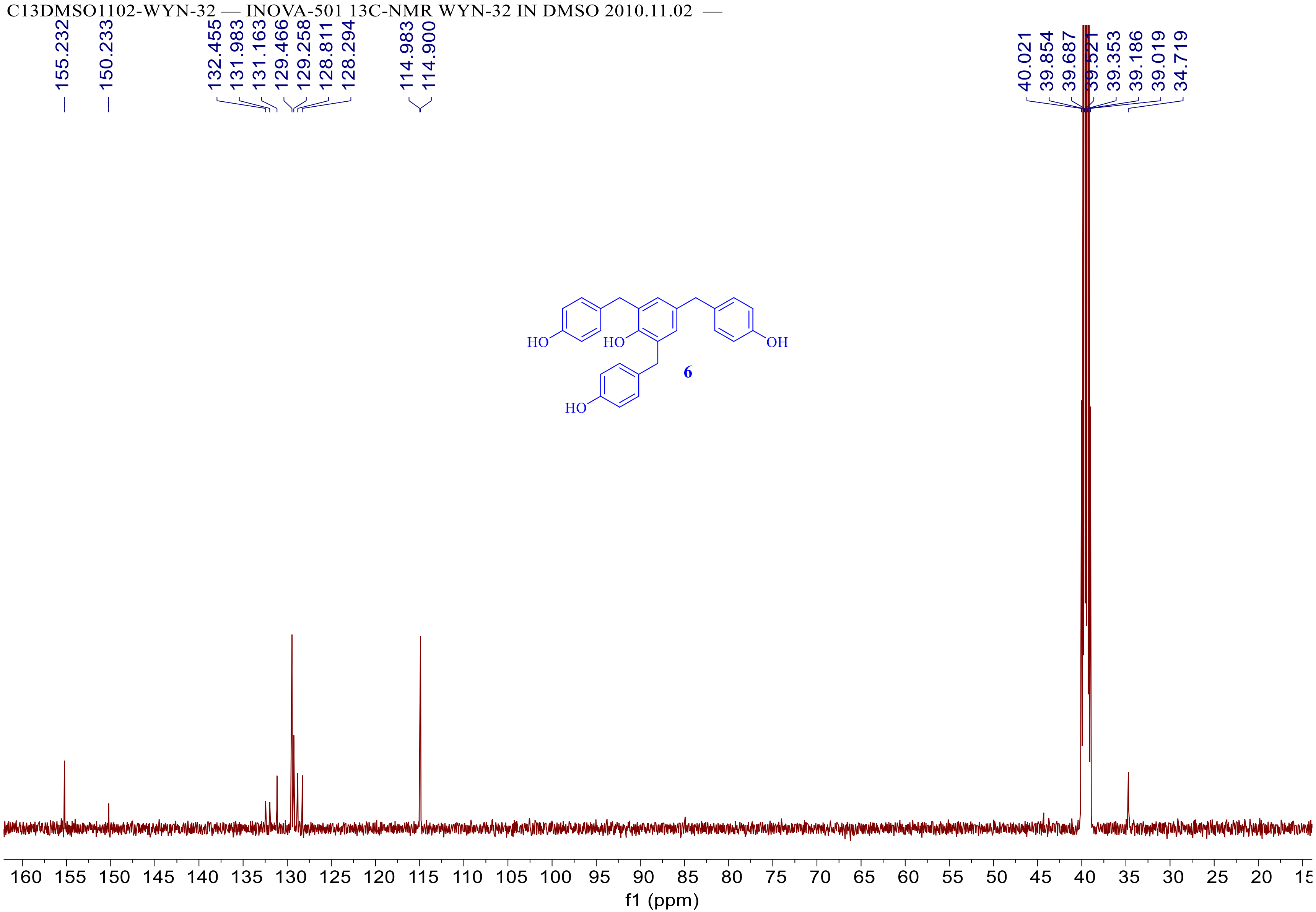

Figure S78. The ^{13}C NMR spectrum of compound **6** in DMSO-d_6 (125 MHz)

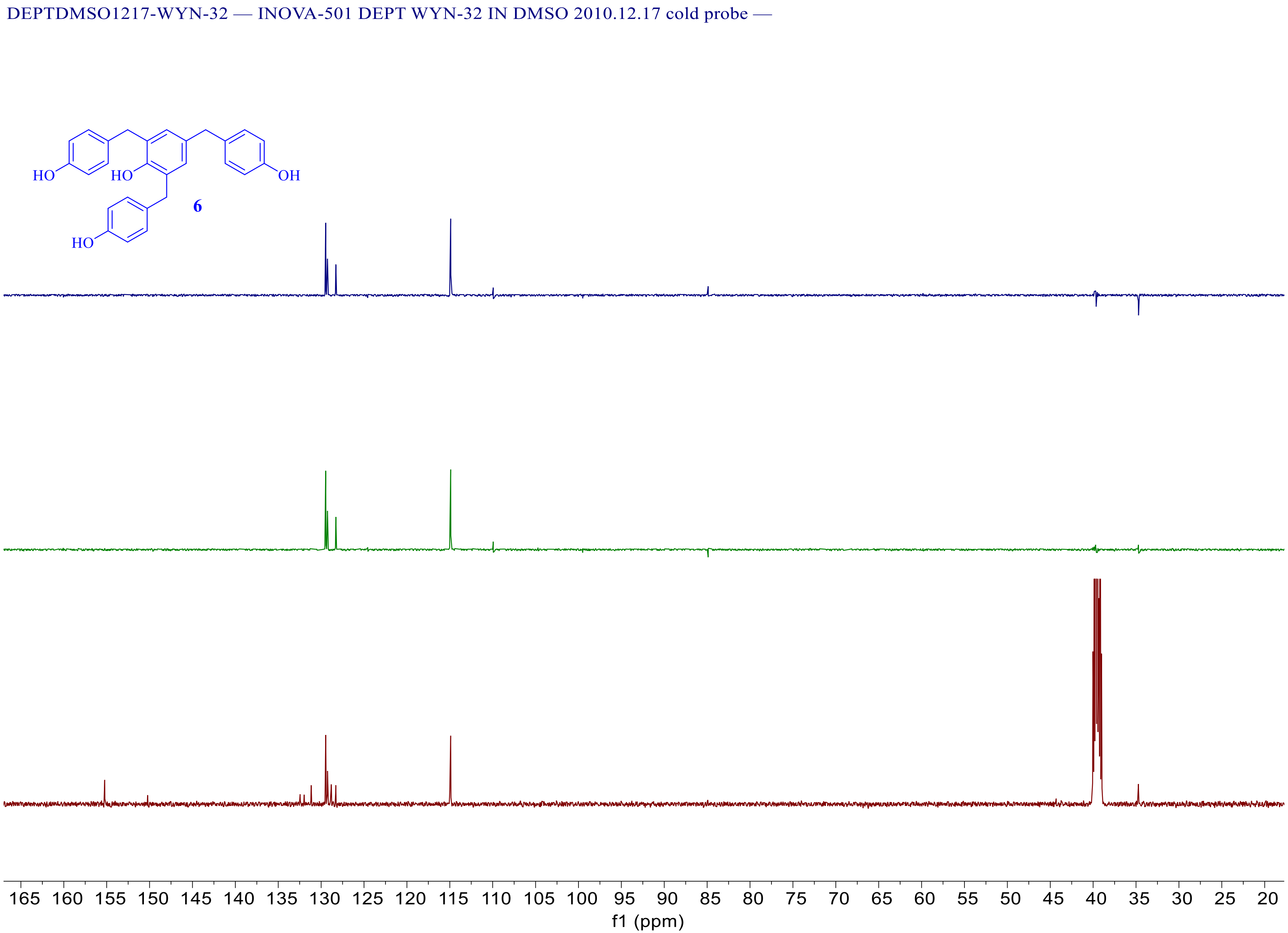

Figure S79. The DEPT spectrum of compound **6** in DMSO-d_6 (125 MHz)

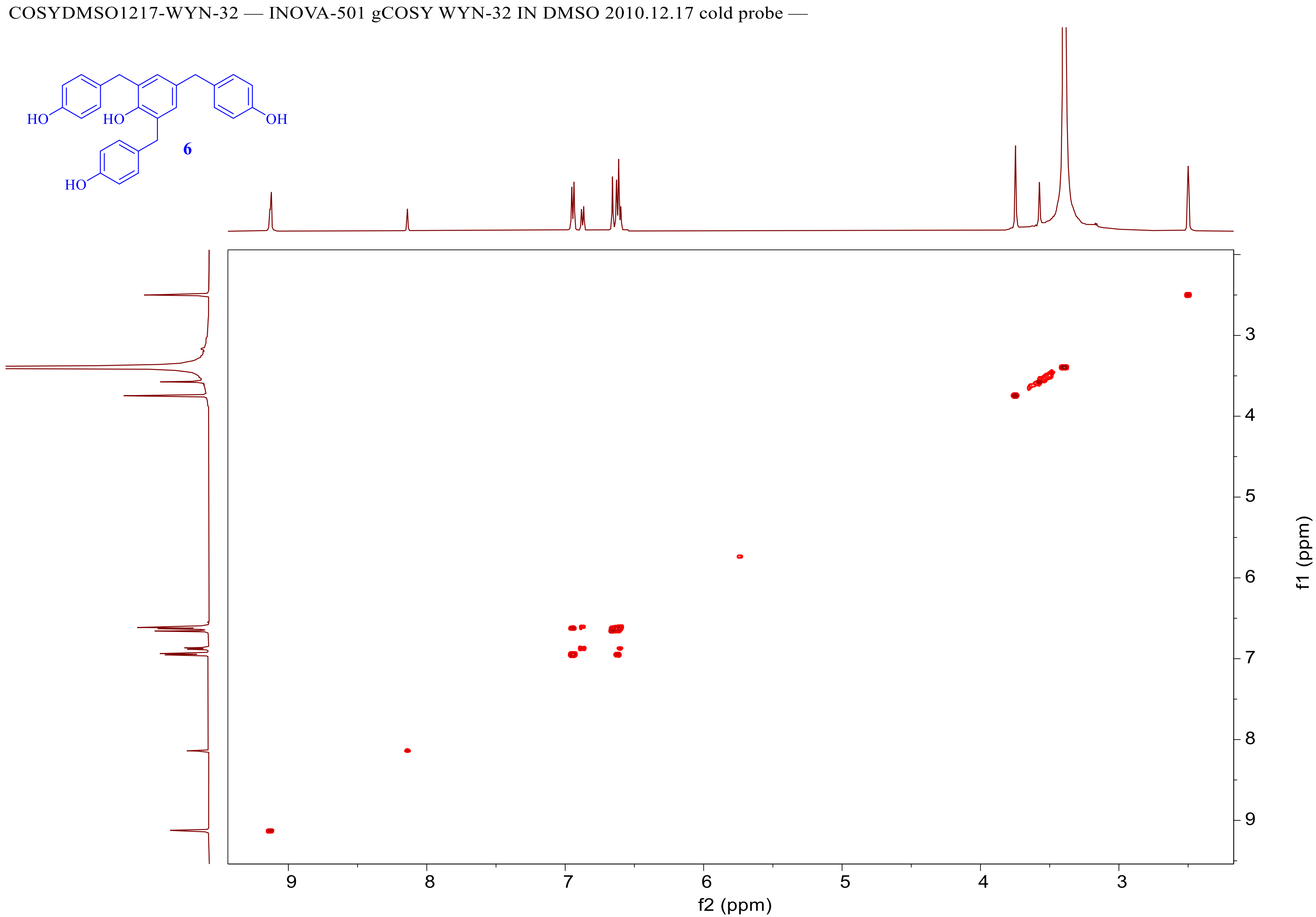

Figure S80. The ^{1}H-^{1}H COSY spectrum of compound **6** in DMSO-d_6 (500 MHz)

HSQCDMSO1217-WYN-32 — INOVA-501 gHSQC WYN-32 IN DMSO 2010.12.17 cold probe —

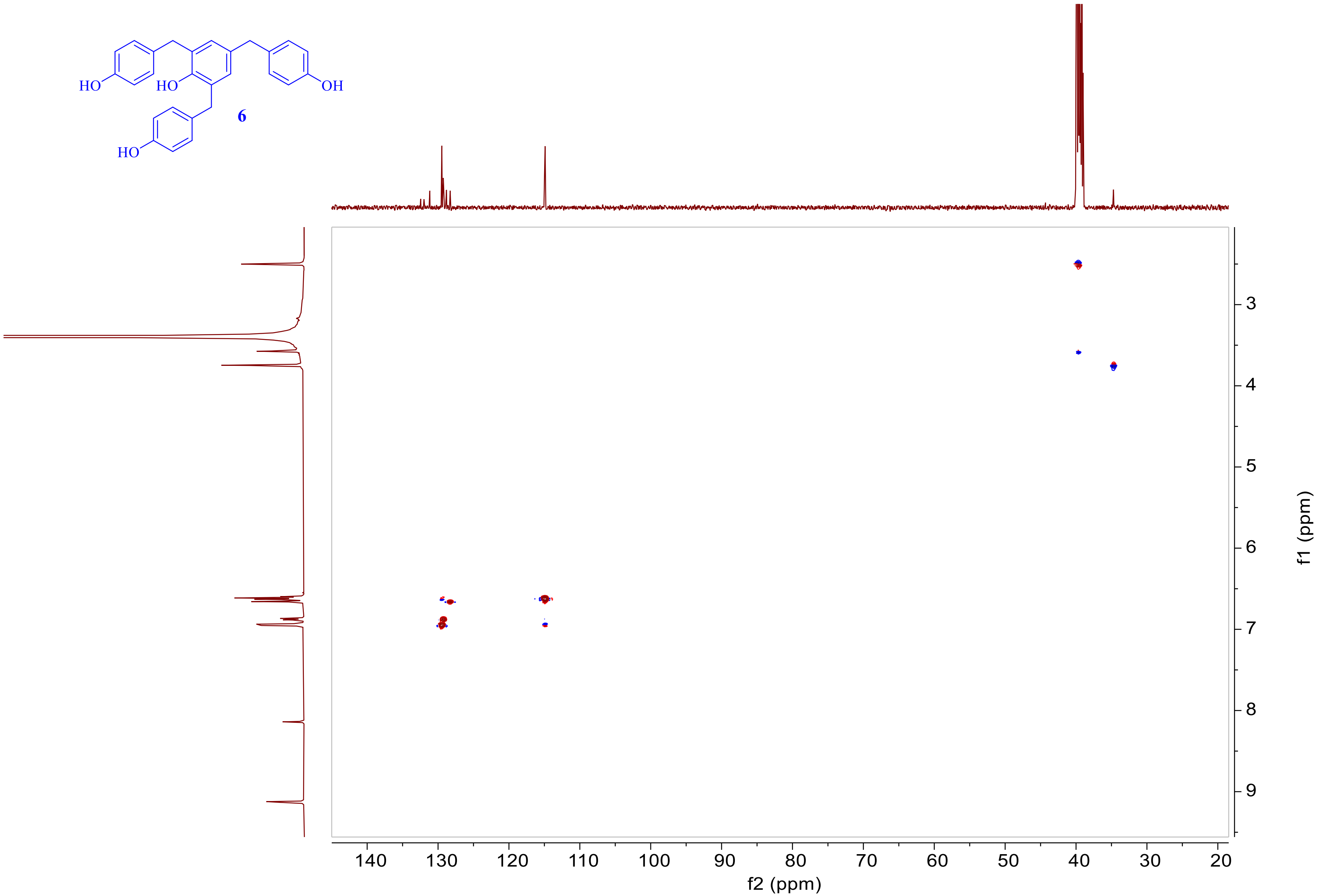

Figure S81. The HSQC spectrum of compound **6** in DMSO-d_6 (500 MHz for ^{1}H)

HMBCDMSO1217-WYN-32 — INOVA-501 gHMBC WYN-32 IN DMSO 2010.12.17 cold probe —

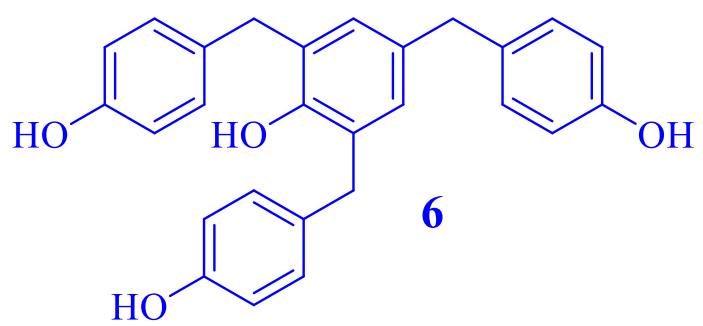

6

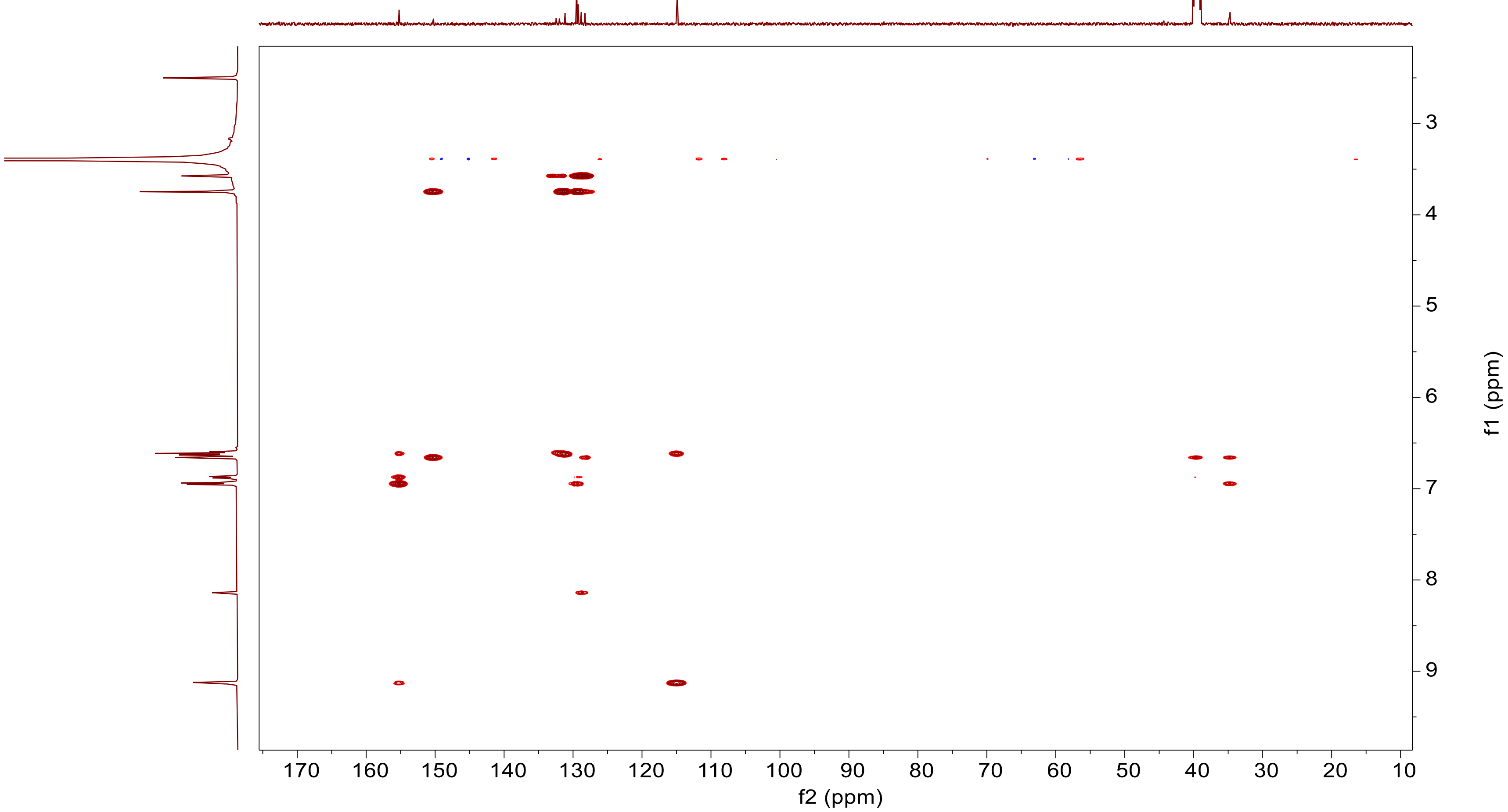

Figure S82. The HMBC spectrum of compound **6** in DMSO-d_6 (500 MHz for ^{1}H)

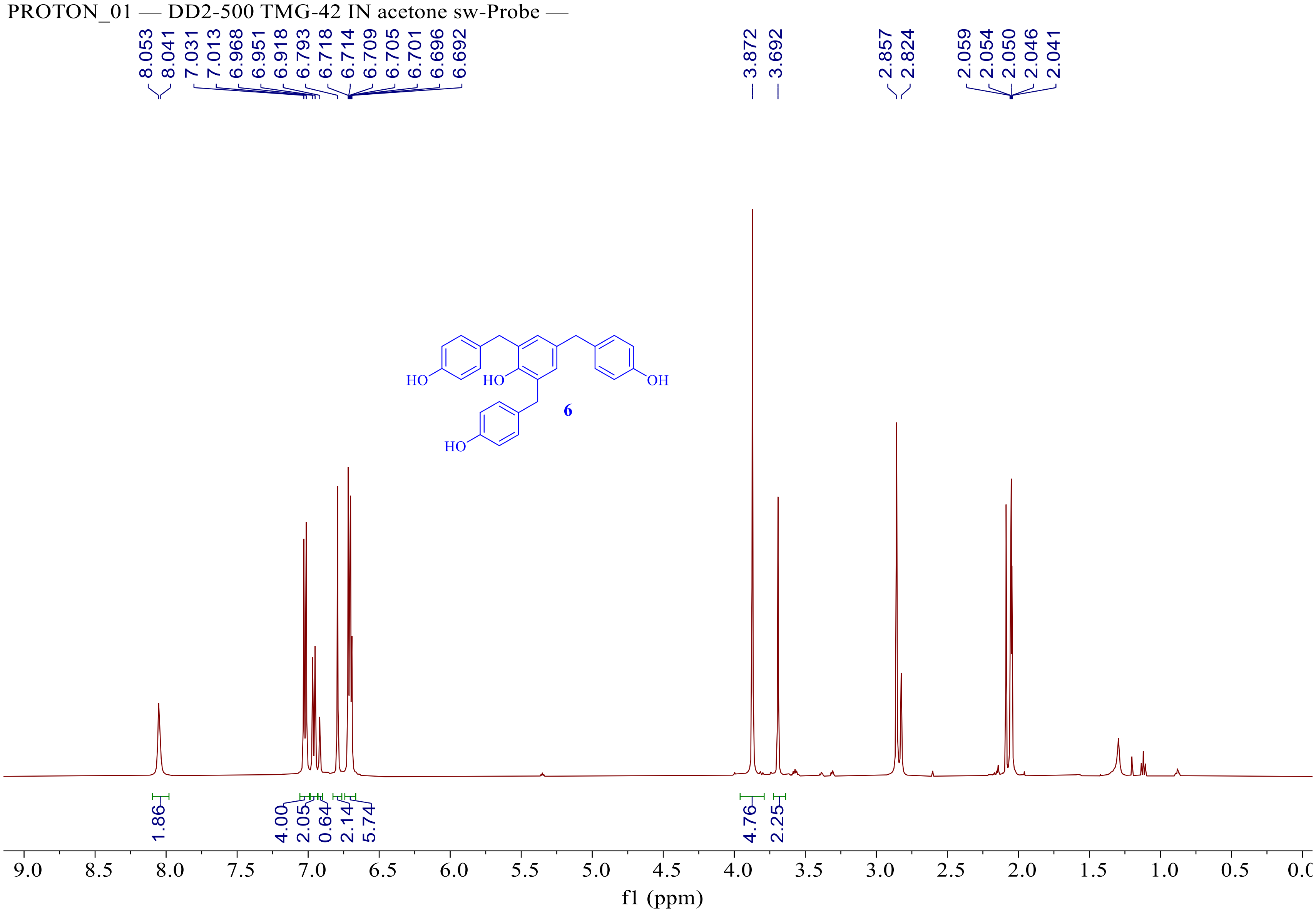

Figure S83. The ^{1}H NMR spectrum of compound **6** in acetone-d_6 (500 MHz)

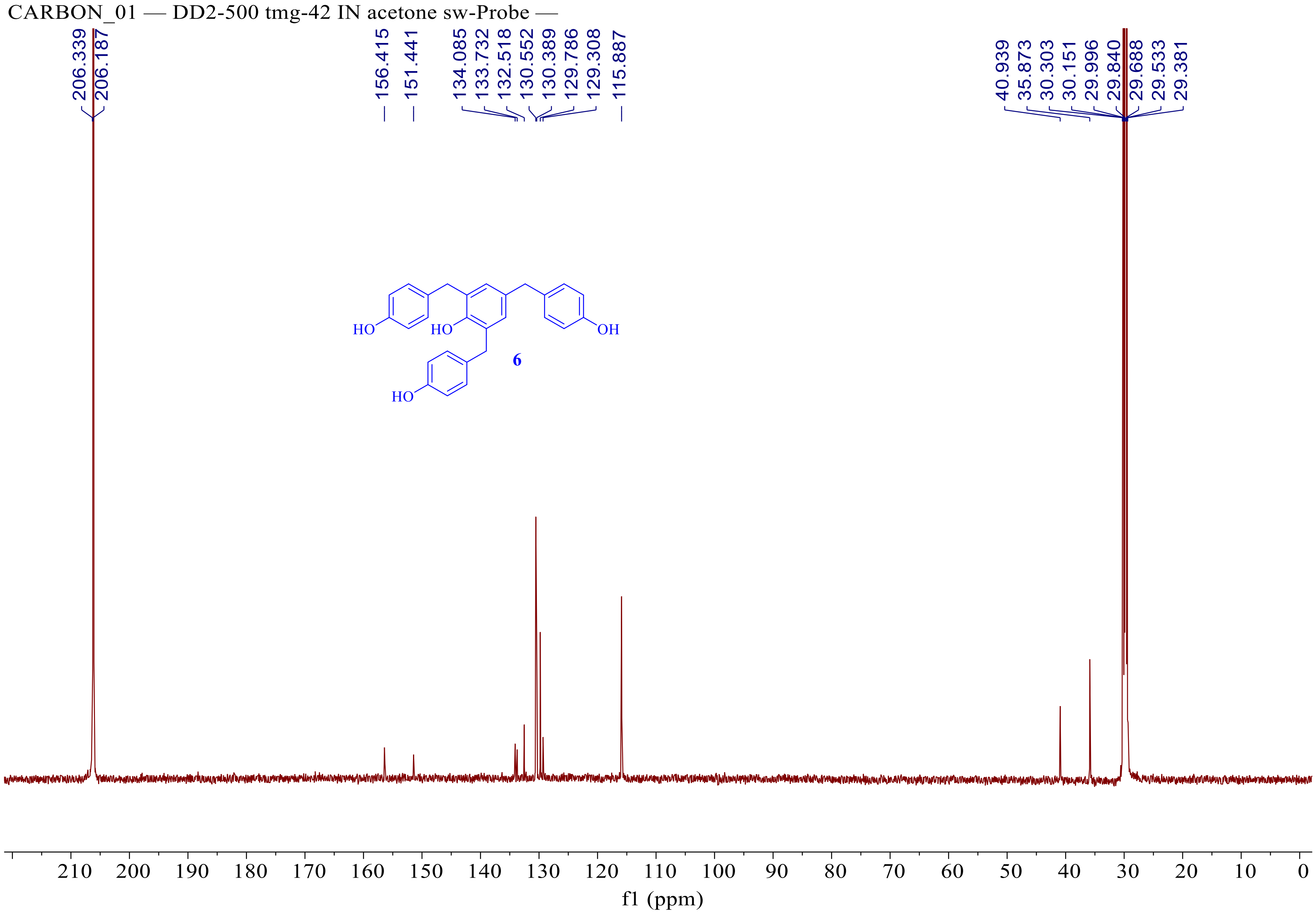

Figure S84. The ^{13}C NMR spectrum of compound **6** in acetone-d_6 (125 MHz)

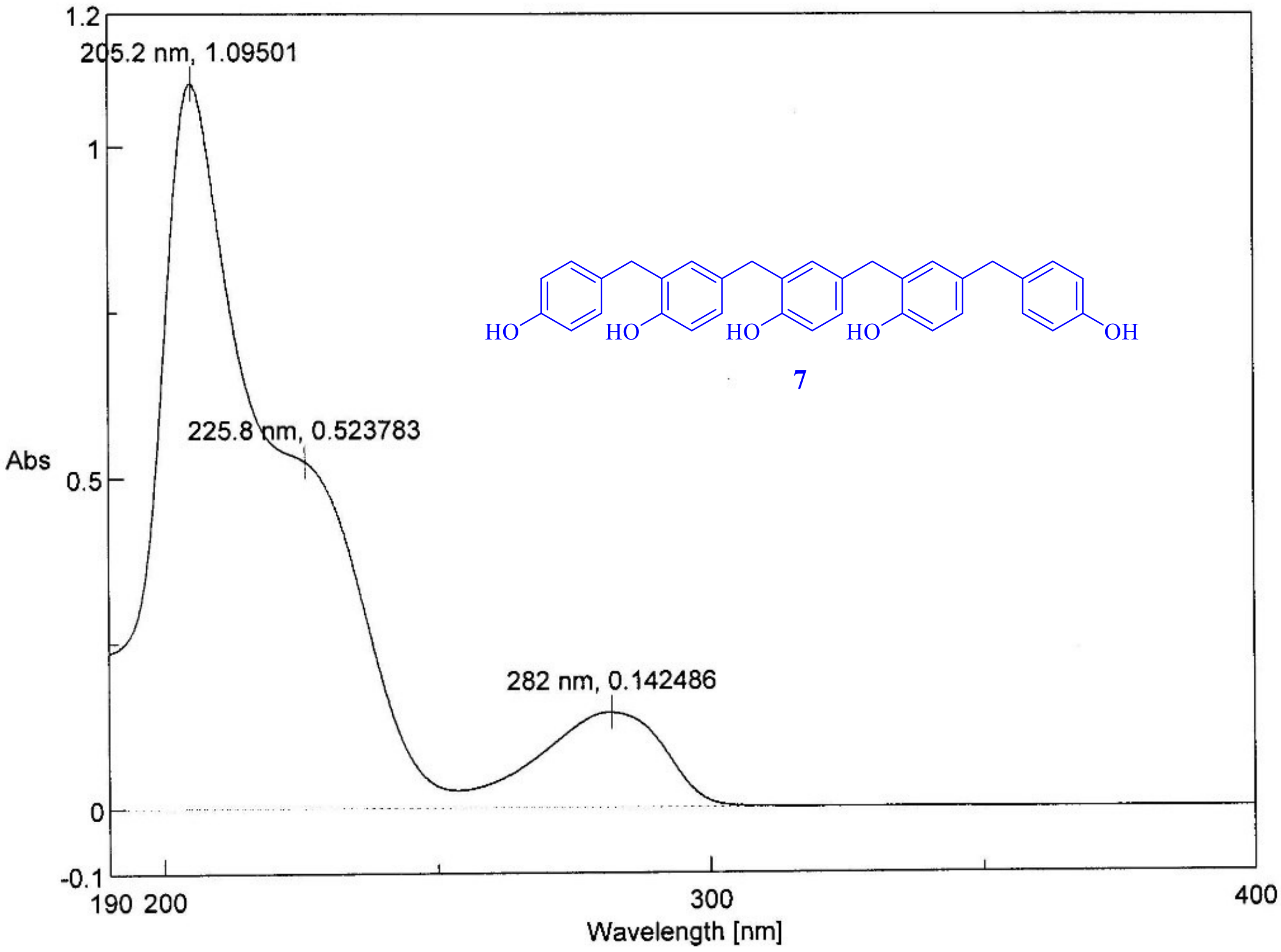

Memory-10

[Comment]

Sample Name	TMG-47
Comment	0.02
User	
Division	UV
Company	324

[Measurement Information]

Instrument Name	V-650
Model Name	V-650
Serial No.	A034461150
Accessory	PSC-718
Accessory S/N	A001761114
Position	1
Cell Length	10 mm
Temperature	19.97 C
Control Sonsor	Holder
Monitor Sensor	Holder
Start Mode	Start immediately
Photometric Mode	Abs
Measurement range	400 - 190 nm
Data pitch	0.2 nm
Band width(UV/Vis)	2.0 nm
Response	Medium
Scanning speed	200 nm/min
Source Change	340 nm
Light Source	D2/WI
Filter Exchange	Step
Correction	Baseline

[Data Information]

Creation Date	2014-2-26 9:29
Data array type	Linear data array
Horizontal	Wavelength [nm]
Vertical	Abs
Start	400 nm
End	190 nm
Data pitch	0.2 nm
Data points	1051

Figure S85. The UV spectrum of compound **7**

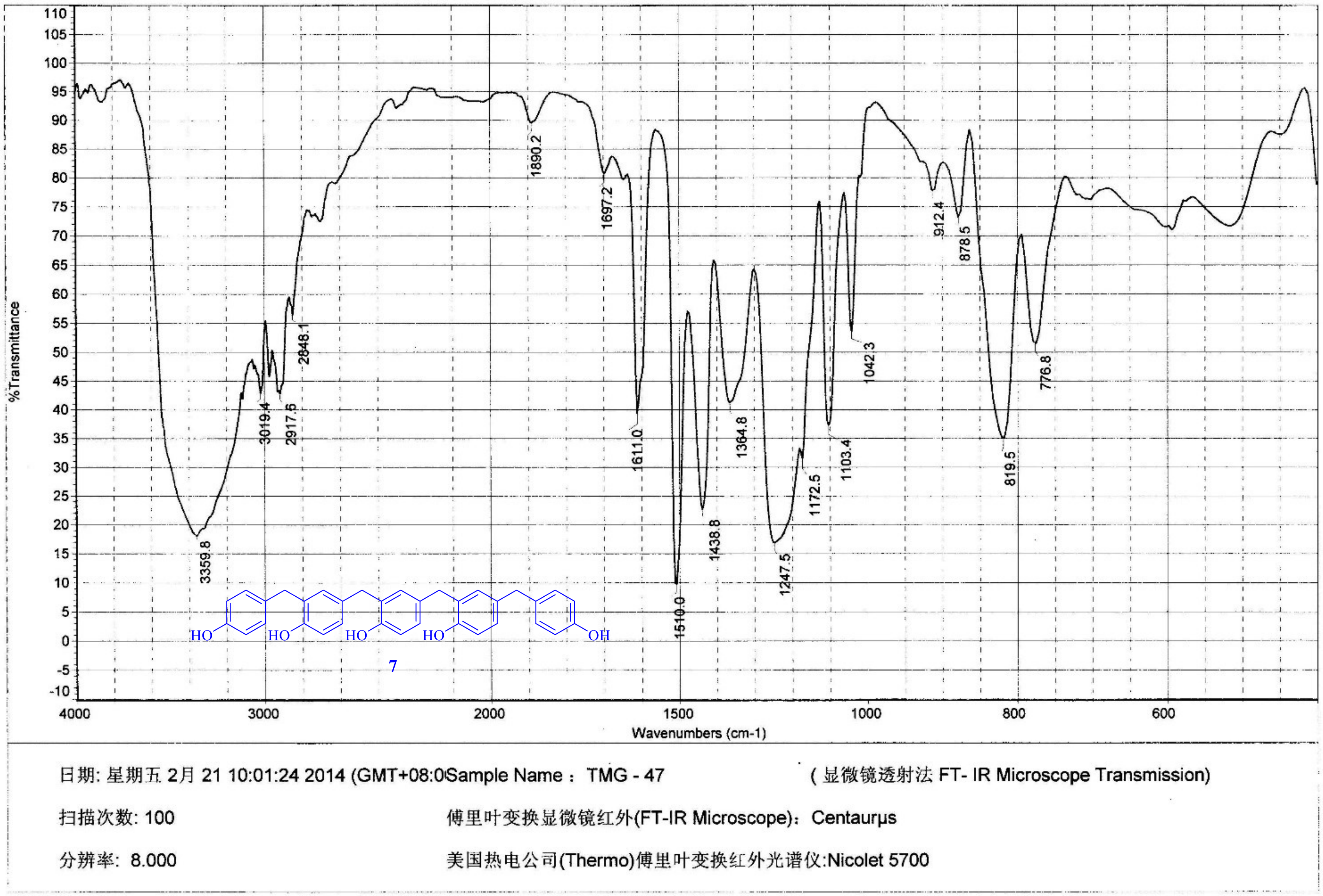

Figure S86. The IR spectrum of compound **7**

Single Mass Spectrum Deconvolution Report

Analysis Name: guoql060.d **Instrument:** LC-MSD-Trap-SL **Print Date:** 7/3/2012 11:38:09 AM
Method: TEST.MS **Operator:** Operator **Acq. Date:** 7/3/2012 11:28:45 AM
Sample Name: TMG-47
Analysis Info:

Acquisition Parameter:

Mass Range Mode	Std/Normal	Trap Drive	45.5	Scan Begin	100 m/z
Ion Polarity	Positive	Octopole RF Amplitude	152.8 Vpp	Scan End	800 m/z
Ion Source Type	ESI	Capillary Exit	-102.3 Volt	Averages	5 Spectra
Dry Temp (Set)	330 °C	Skimmer	-40.0 Volt	Max. Accu Time	200000 µs
Nebulizer (Set)	15.00 psi	Oct 1 DC	-12.00 Volt	ICC Target	20000
Dry Gas (Set)	5.00 l/min	Oct 2 DC	-1.70 Volt	Charge Control	on

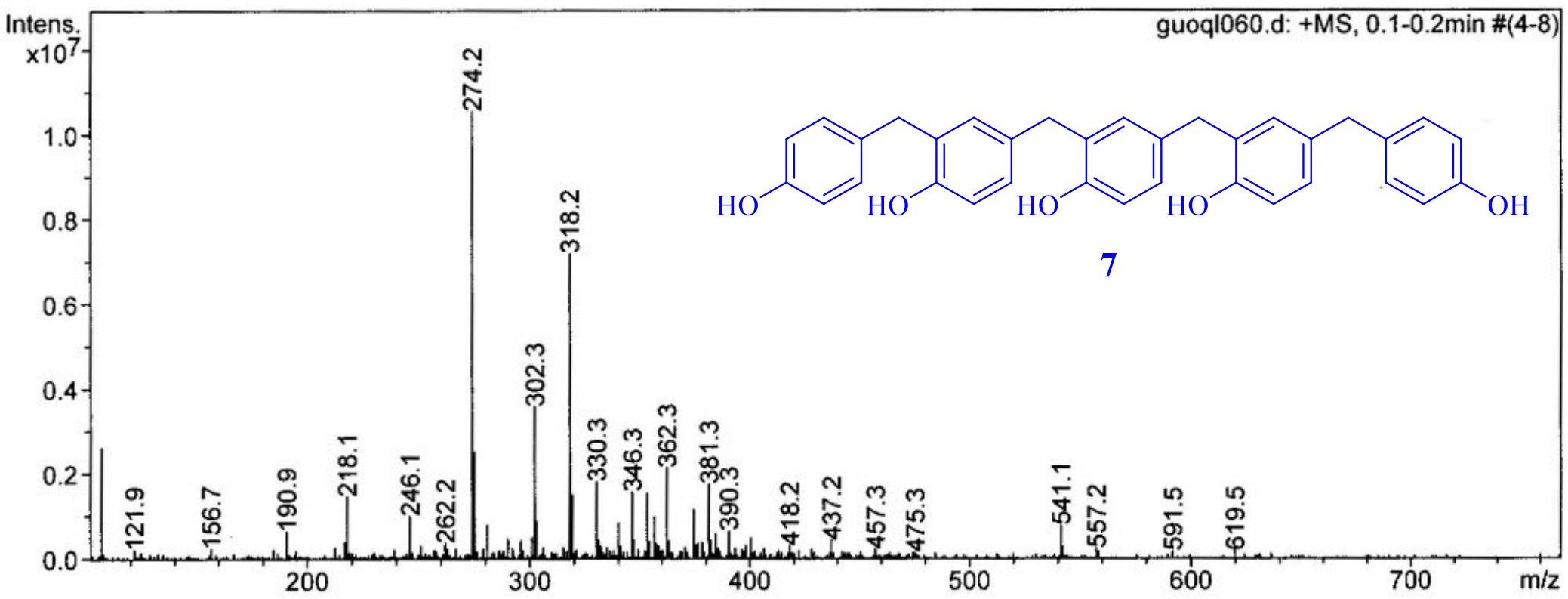

Component	Molecular Mass	Molecule	Absolute Abundance	Relative Abundance

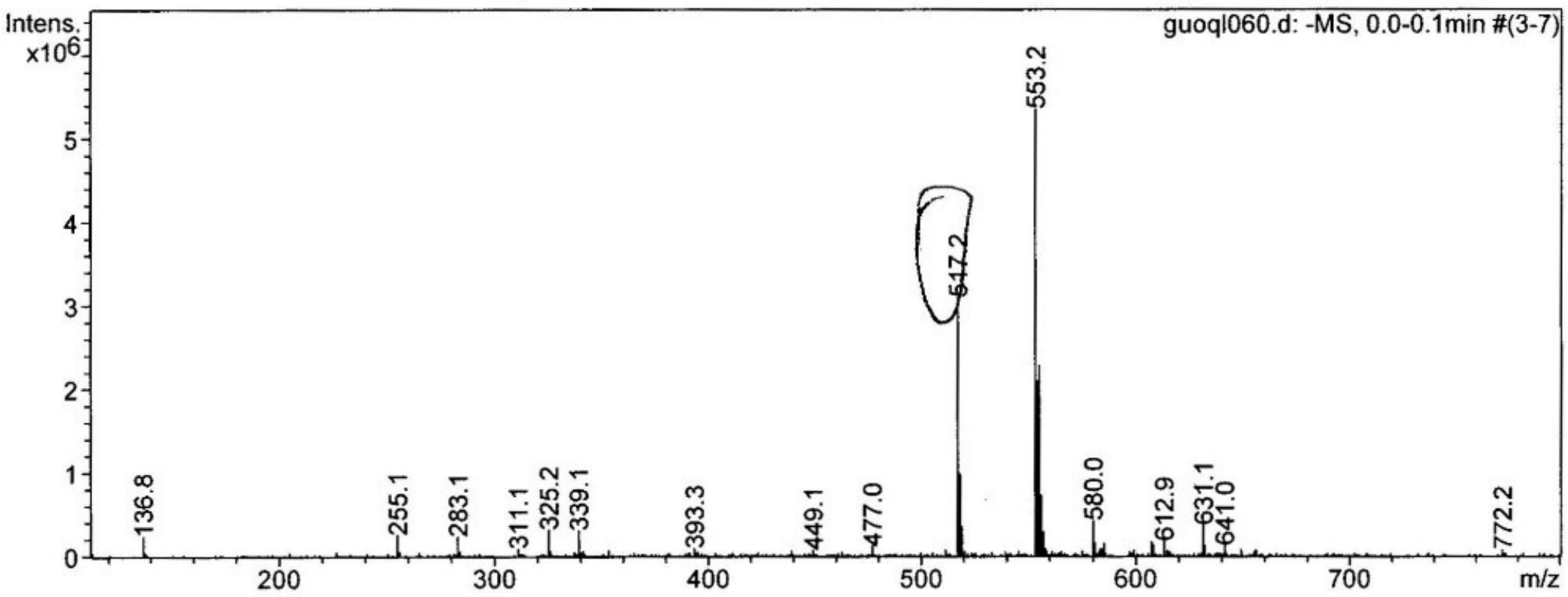

Component	Molecular Mass	Molecule	Absolute Abundance	Relative Abundance

Figure S87. The ESIMS of compound **7**

Qualitative Analysis Report

**Data Filename**	2014022606.d	**Sample Name**	TMG-47
**Sample Type**	Sample	**Position**	P1-C6
**Instrument Name**	Instrument 1	**User Name**	
**Acq Method**		**IRM Calibration Status**	Success
**DA Method**	TEST LCMS.m	**Comment**	

User Chromatograms

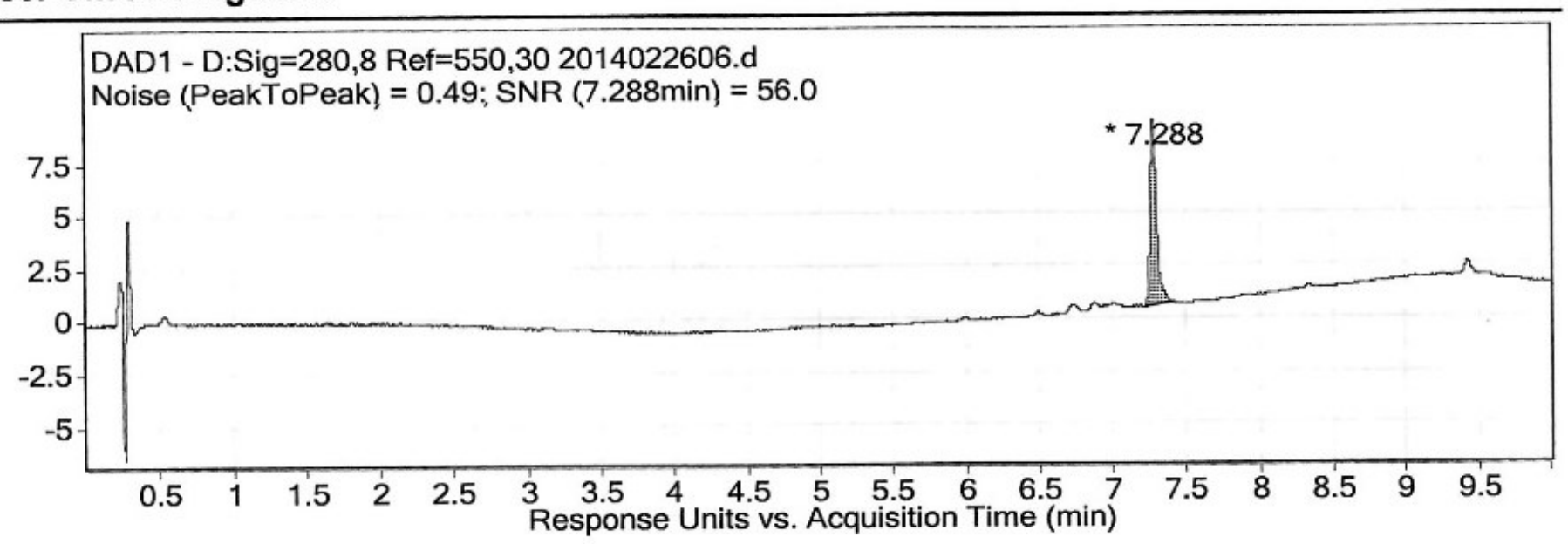

Integration Peak List

Peak	Start	RT	End	Height	Area	Area %	Signal To Noise
1	7.223	7.288	7.41	8.85	27.47	100	56

Noise Measurements

Noise Type	Signal Definition	Noise Multiplier	Noise Value
Peak-to-Peak	Area	1	0.490665436

Noise Regions

Start	End
0.5	1
5	5.3
9.99	11

Fragmentor Voltage 135 **Collision Energy** 0 **Ionization Mode** ESI

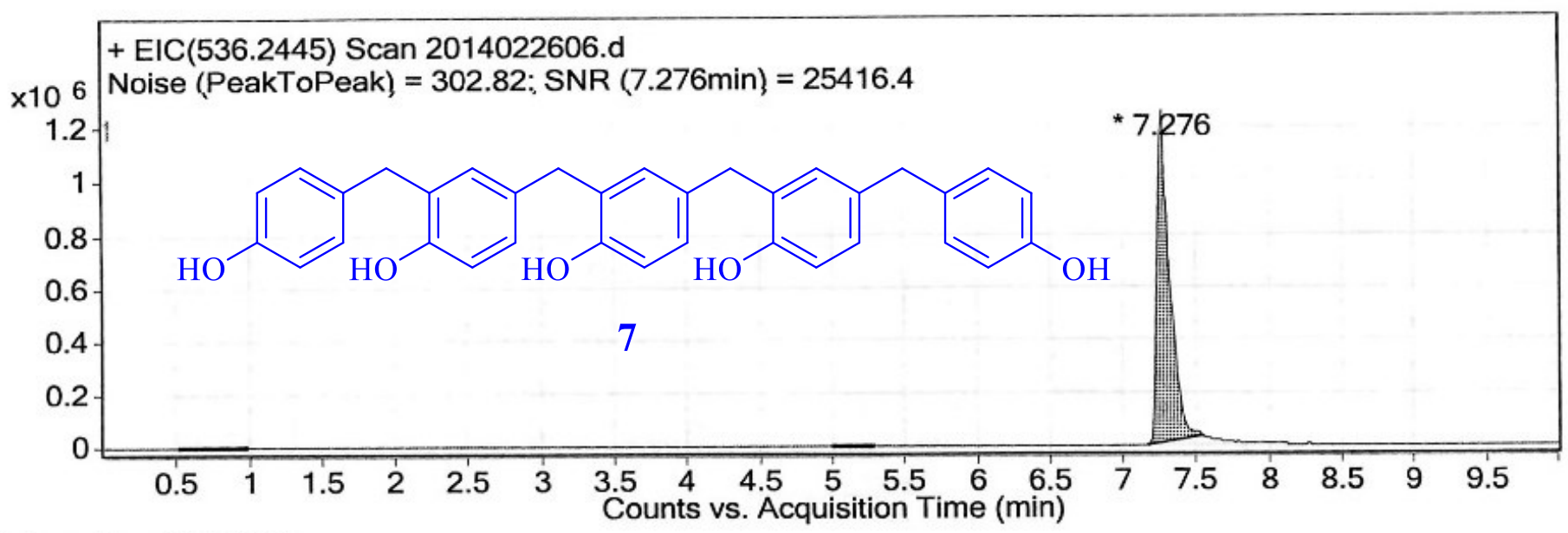

Integration Peak List

Peak	Start	RT	End	Height	Area	Area %	Signal To Noise
1	7.163	7.276	7.549	1247435	7696697	100	25416.4

Noise Measurements

Noise Type	Signal Definition	Noise Multiplier	Noise Value
Peak-to-Peak	Area	1	302.8244629

Noise Regions

Start	End
0.5	1
5	5.3
9.99	11

User Spectra

Figure S88. The HR-ESIMS report of compound **7** (page 1)

Qualitative Analysis Report

Fragmentor Voltage	Collision Energy	Ionization Mode
135	0	Esi

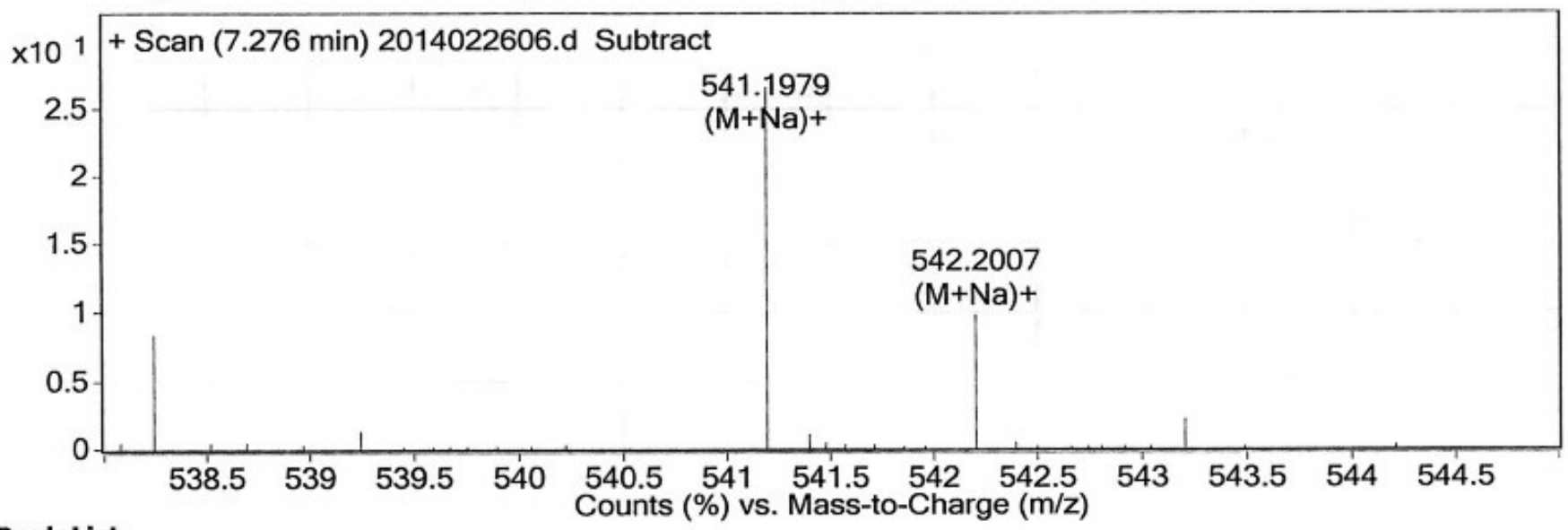

Peak List

m/z	z	Abund	Formula	Ion
425.1735		80392		
536.2423	1	1257610		
536.434		81014		
537.2461	1	494712		
538.2481	1	106051		
541.1979	1	332949	C34 H30 Na O5	(M+Na)+
542.2007	1	124193	C34 H30 Na O5	(M+Na)+
550.2577	1	183681		
557.1712		155139		
922.0097		185183		

Formula Calculator Element Limits

Element	Min	Max
C	3	100
H	0	500
O	0	90
N	0	5
S	0	5
Cl	0	2
Br	0	0
Si	0	0
F	0	0
P	0	0

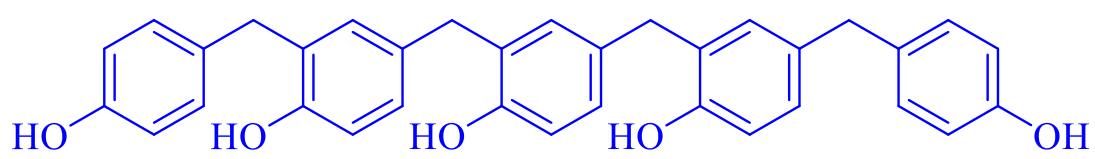

7

Formula Calculator Results

Formula	Best	Mass	Tgt Mass	Diff (ppm)	Ion Species	Score
C34 H30 O5	TRUE	518.2086	518.2093	1.31	C34 H30 Na O5	99.96
C35 H26 N4 O		518.2087	518.2107	3.87	C35 H26 N4 Na O	99.65
C38 H30 S		518.2086	518.2068	-3.53	C38 H30 Na S	98.96
C26 H34 N2 O7 S		518.2087	518.2087	0.03	C26 H34 N2 Na O7 S	98.29
C35 H34 S2		518.2087	518.2102	2.97	C35 H34 Na S2	97.68
C30 H34 N2 O2 S2		518.2087	518.2062	-4.81	C30 H34 N2 Na O2 S2	97.26

--- End Of Report ---

Agilent Technologies Page 2 of 2 Printed at: 9:53 AM on: 2/26/2014

Figure S89. The HR-ESIMS report of compound **7** (page 2)

MS Formula Results: + Scan (7.276 min) Sub (2014022606.d)

m/z	Ion	Formula	Abundance
541.1979	(M+Na)+	C34 H30 Na O5	332949

Best	Formula (M)	Ion Formula	Calc m/z	Score	Cross S	Mass	Calc Mass	Diff (ppm)	Abs Diff (ppm)	Abund Match	Spacing Mat	Mass Match	m/z	DBE
☑	C34 H30 O5	C34 H30 Na O5	541.1985	99.96		518.2086	518.2093	1.31	1.31	99.99	99.96	99.95	541.1979	20
☐	C35 H26 N4 O	C35 H26 N4 Na O	541.1999	99.65		518.2087	518.2107	3.87	3.87	99.58	99.97	99.52	541.1979	25
☐	C38 H30 S	C38 H30 Na S	541.196	98.96		518.2086	518.2068	-3.53	3.53	97.42	99.5	99.6	541.1979	24
☐	C26 H34 N2 O7 S	C26 H34 N2 Na O7 S	541.1979	98.29		518.2087	518.2087	0.03	0.03	94.74	99.13	100	541.1979	11
☐	C35 H34 S2	C35 H34 Na S2	541.1994	97.68		518.2087	518.2102	2.97	2.97	93.29	98.89	99.72	541.1979	19
☐	C30 H34 N2 O2 S2	C30 H34 N2 Na O2 S2	541.1954	97.26		518.2087	518.2062	-4.81	4.81	92.75	98.66	99.27	541.1979	15

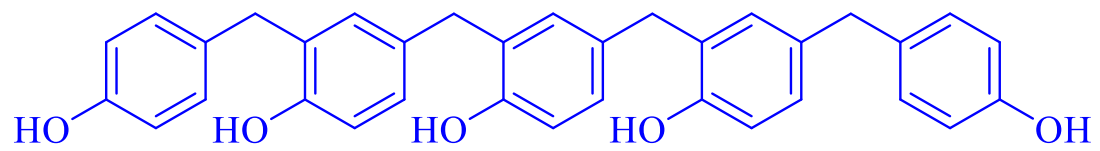

7

page 1

Figure S90. The HR-ESIMS report of compound **7** (page 3)

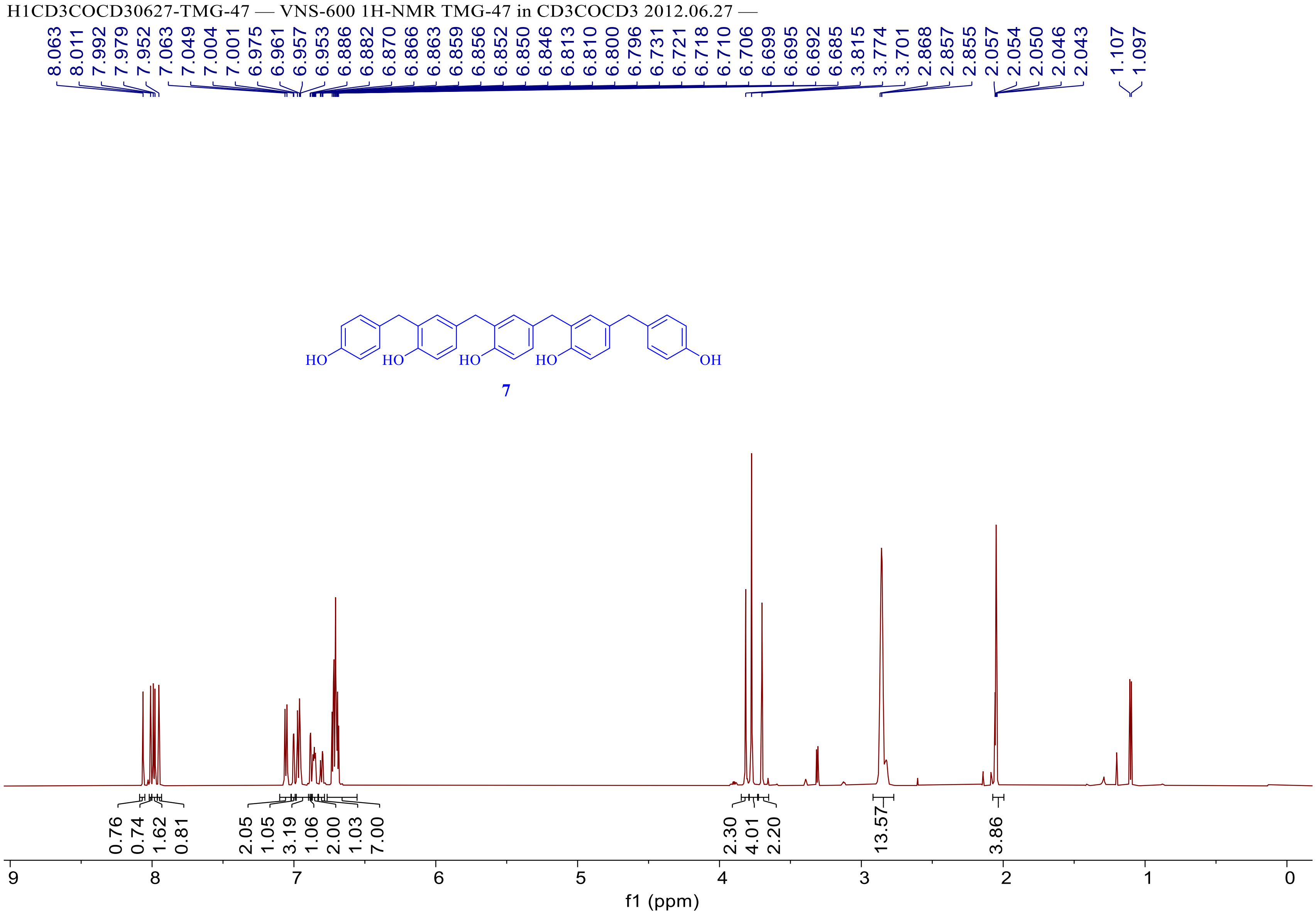

Figure S91. The ^{1}H NMR spectrum of compound **7** in acetone-d_6 (600 MHz)

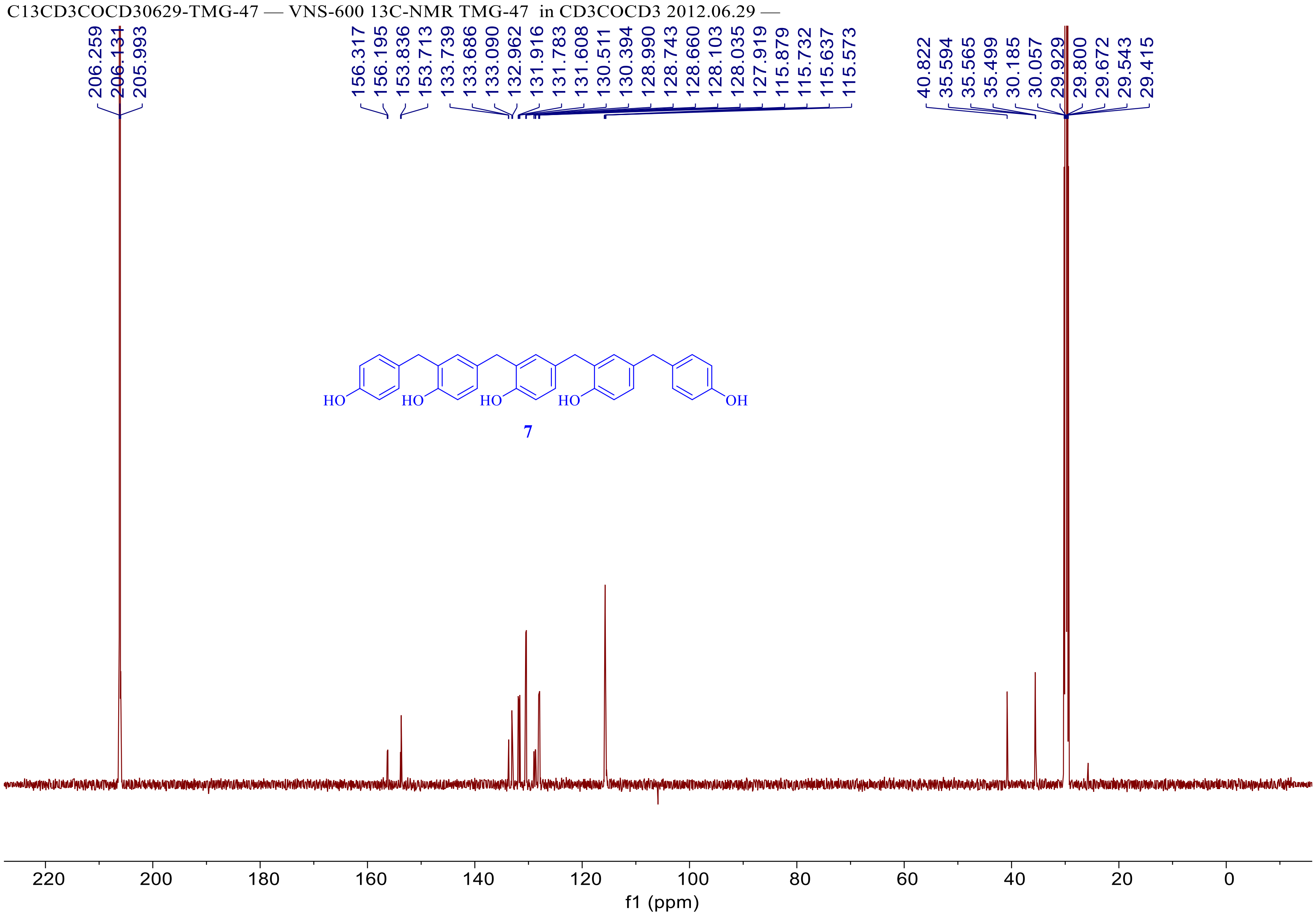

Figure S92. The ^{13}C NMR spectrum of compound **7** in acetone-d_6 (150 MHz)

DEPTCD3COCD30629-TMG-47 — VNS-600 DEPT-NMR TMG-47 in CD3COCD3 2012.06.29 —

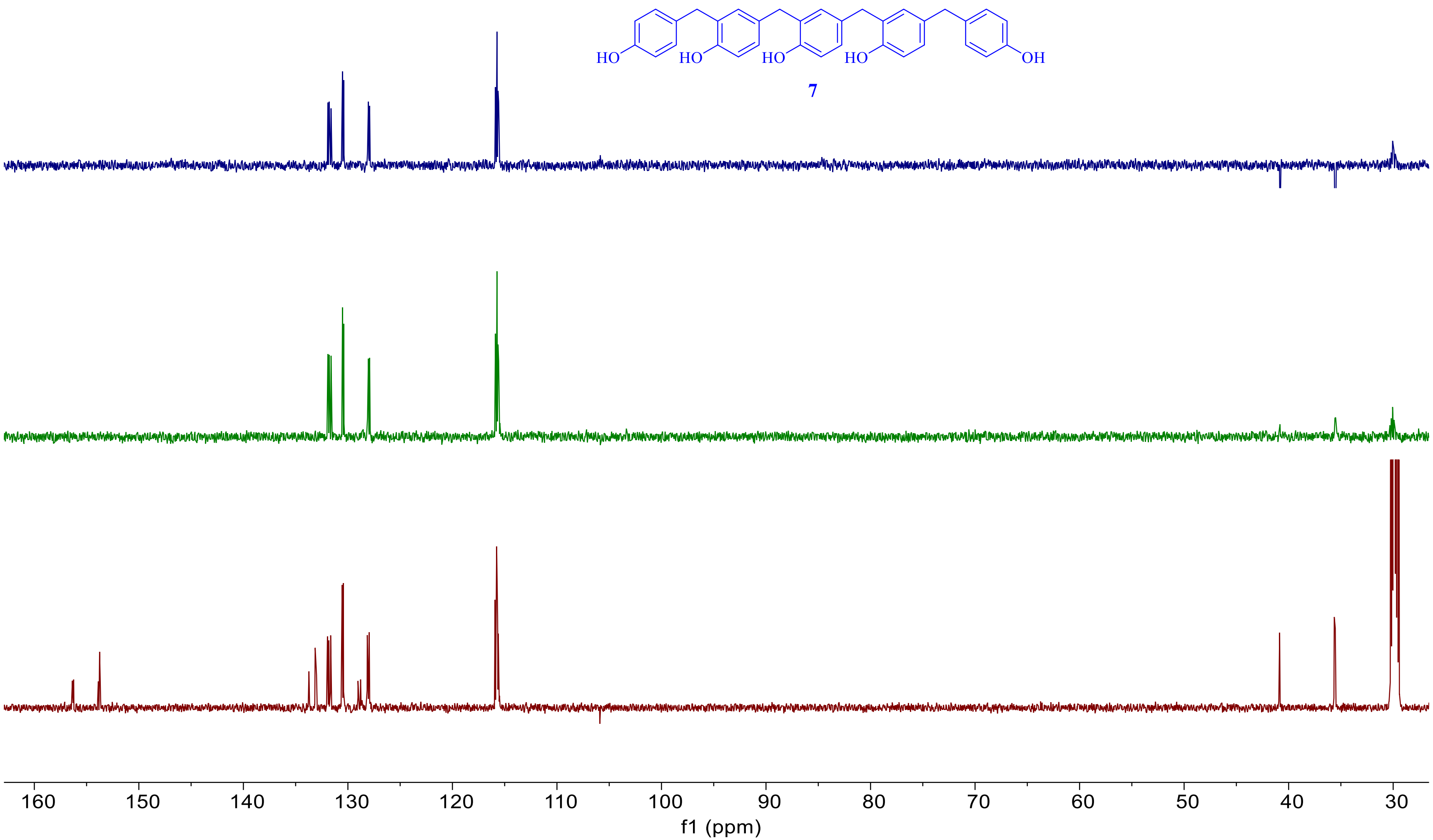

Figure S93. The DEPT spectrum of compound **7** in acetone-d_6 (150 MHz)

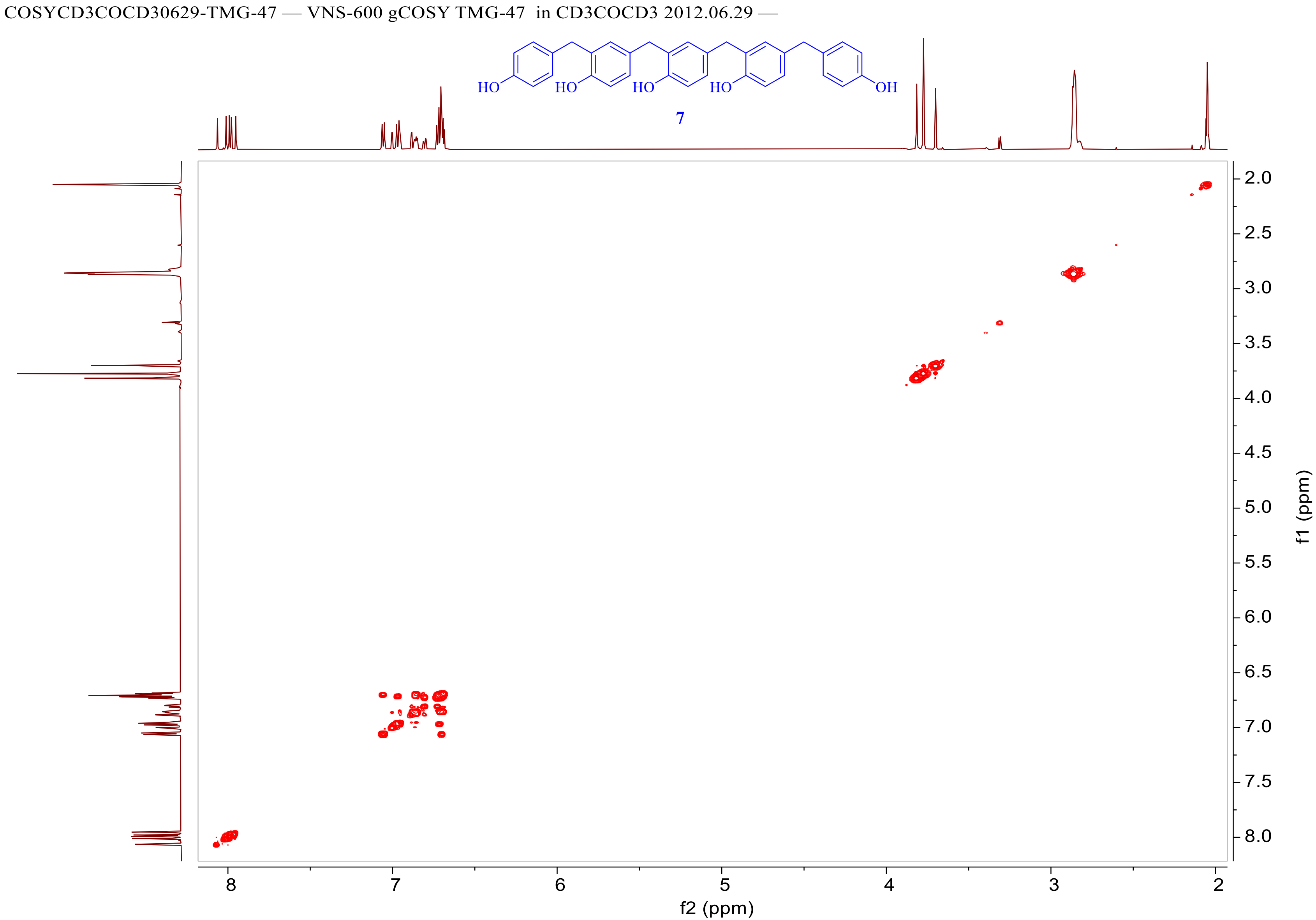

Figure S94. The ^{1}H-^{1}H COSY spectrum of compound **7** in acetone-d_6 (600 MHz)

HSQCCD3COCD30629-TMG-47 — VNS-600 gHSQC TMG-47 in CD3COCD3 2012.06.29 —

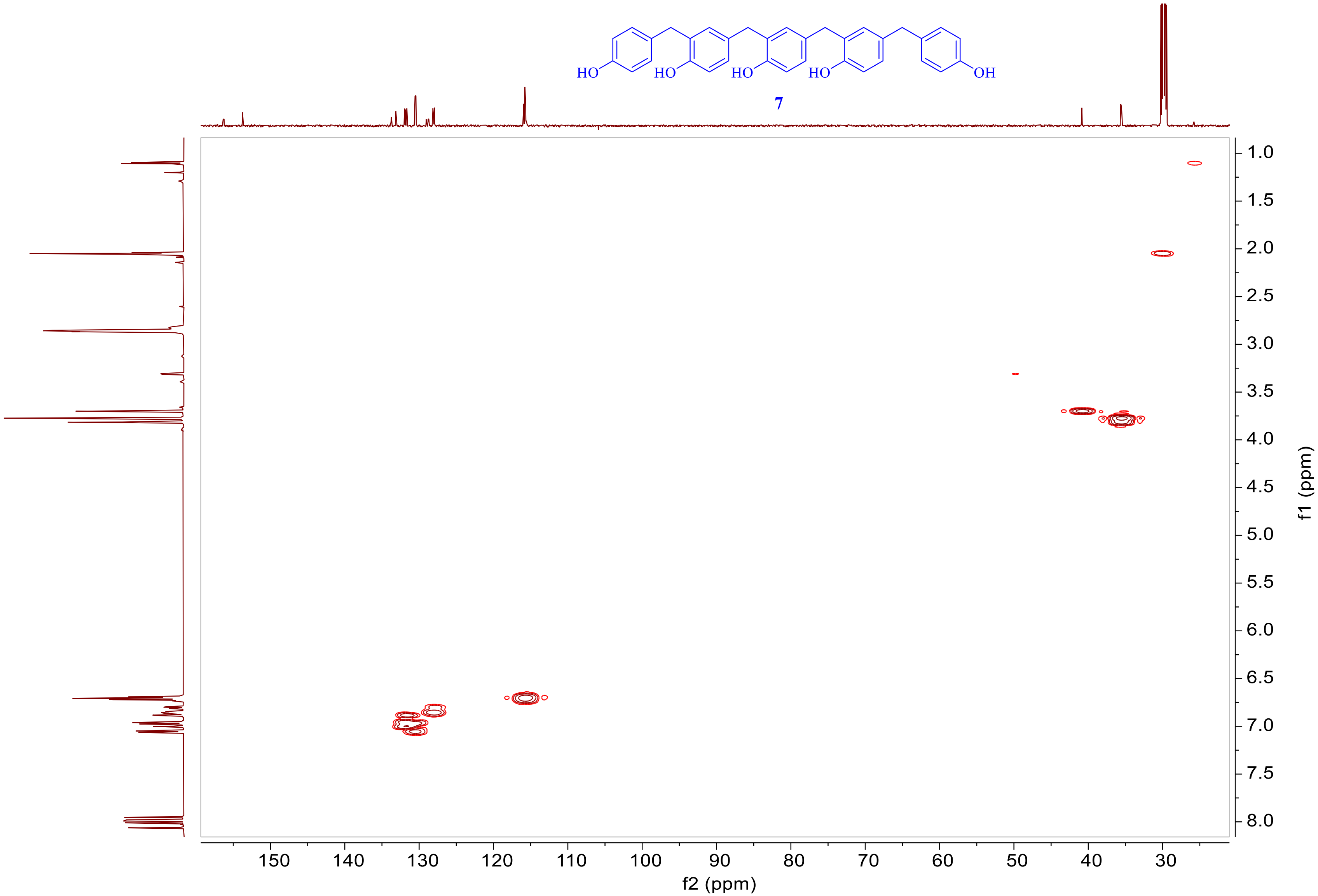

Figure S95. The HSQC spectrum of compound **7** in acetone-d_6 (600 MHz for ^{1}H)

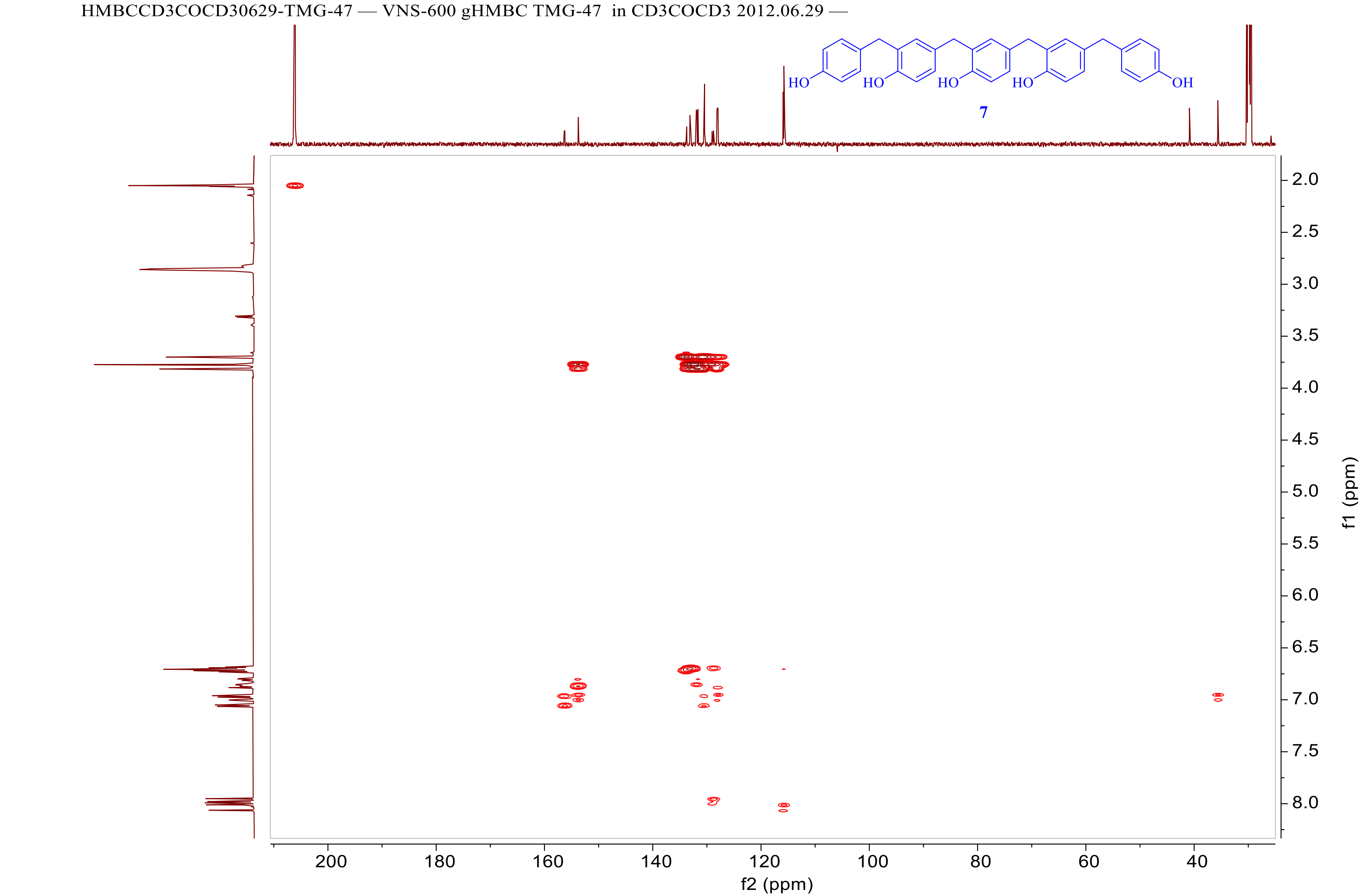

Figure S96. The HMBC spectrum of compound **7** in acetone-d_6 (600 MHz for ^1H)

TMG-47-NOESY1D — DD2-500 NOESY1D TMG-47 IN acetone coldprobe-Probe —

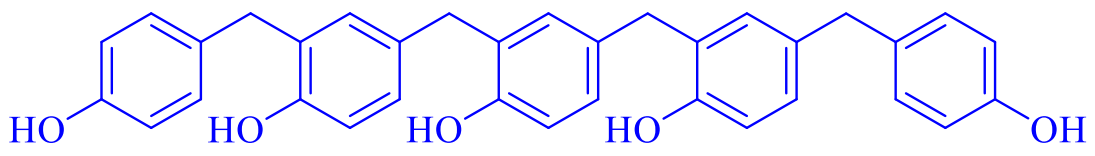
7

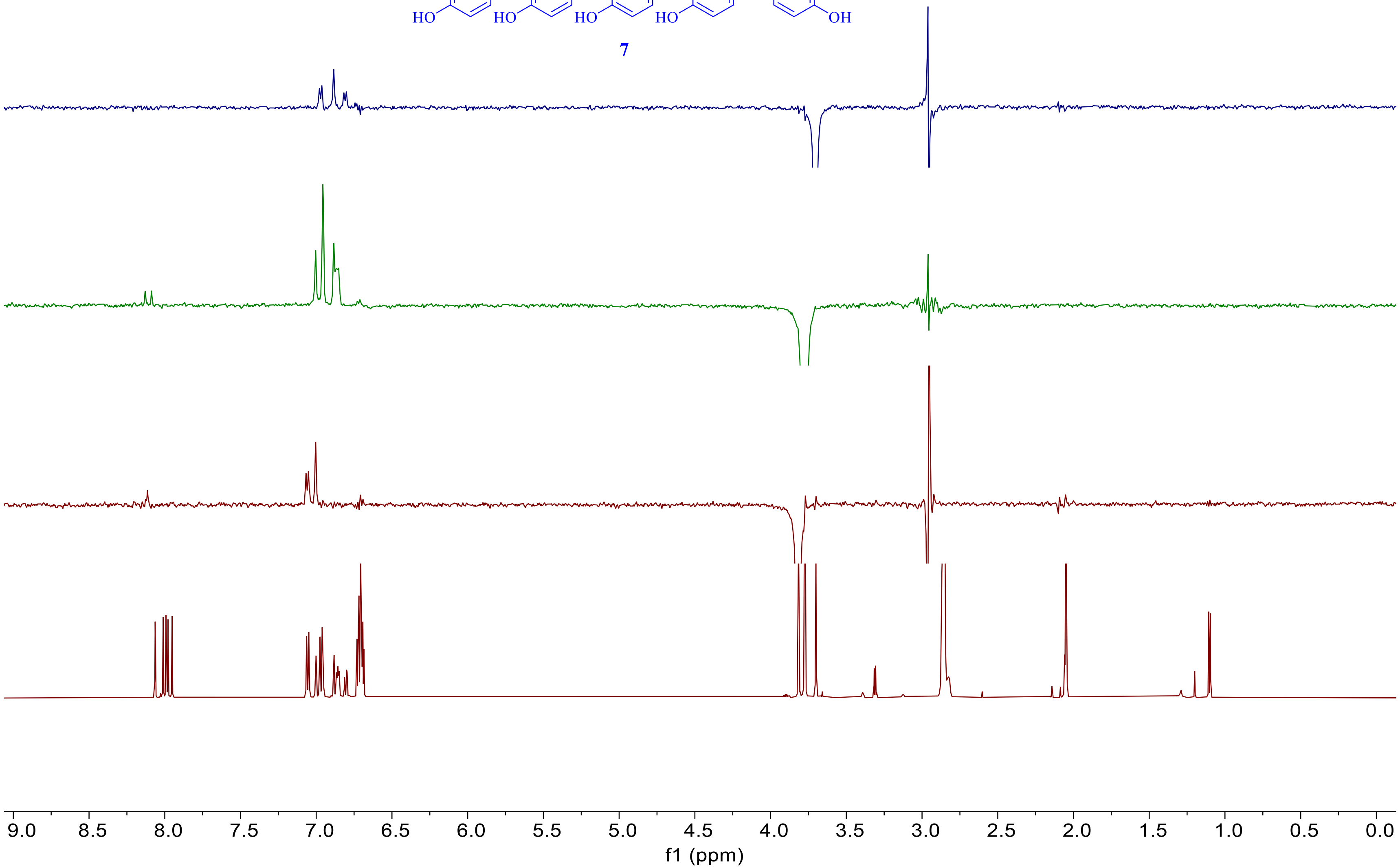

Figure S97. The NOESY1D spectrum of compound **7** in acetone-d_6 (500 MHz)

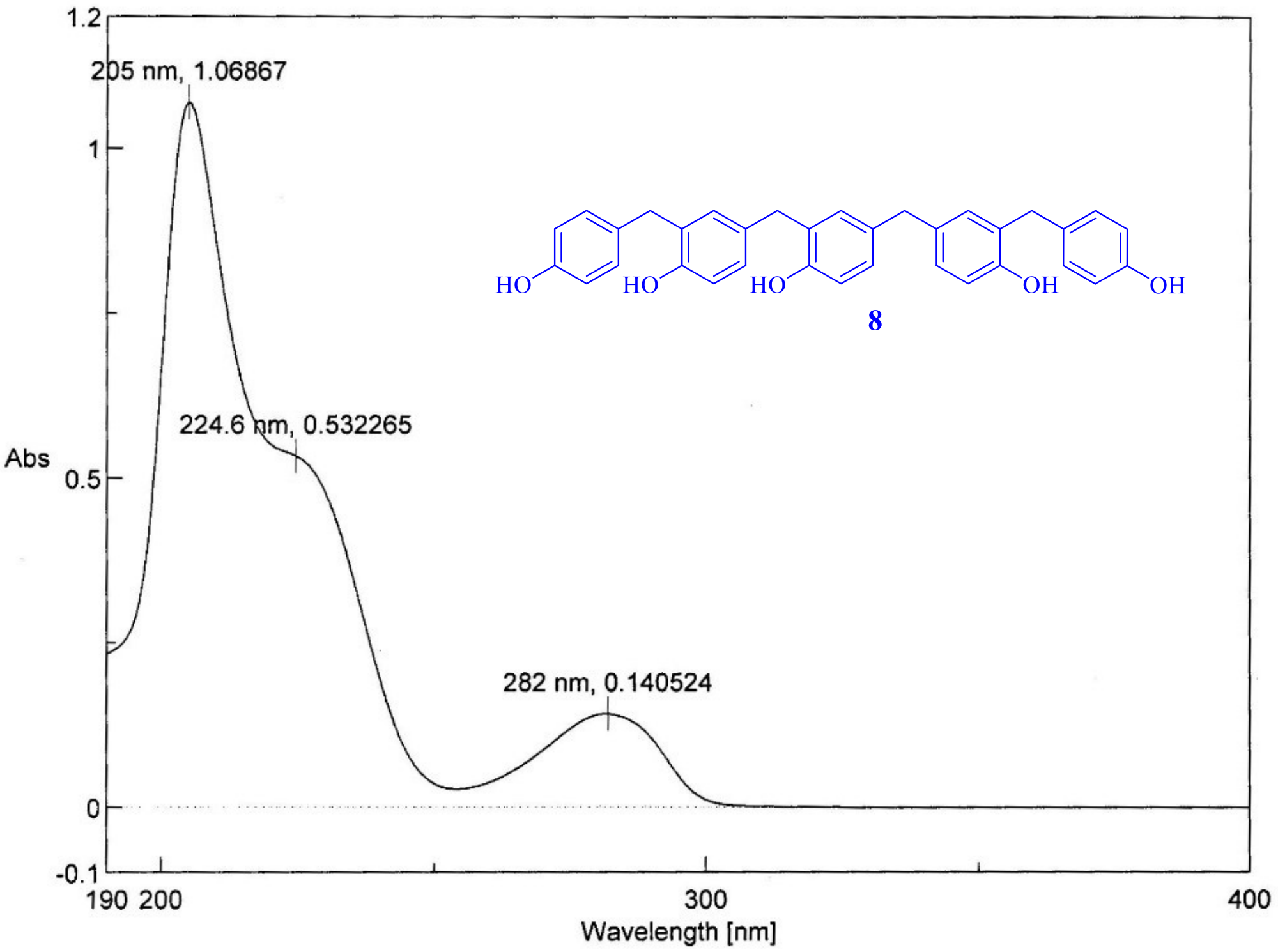

[Comment]
Sample Name TMG-48
Comment 0.02
User
Division UV
Company 324
[Measurement Information]
Instrument Name V-650
Model Name V-650
Serial No. A034461150

Accessory PSC-718
Accessory S/N A001761114
Position 1
Cell Length 10 mm
Temperature 19.96 C
Control Sonsor Holder
Monitor Sensor Holder
Start Mode Start immediately

Photometric Mode Abs
Measurement range 400 - 190 nm
Data pitch 0.2 nm
Band width(UV/Vis) 2.0 nm
Response Medium
Scanning speed 200 nm/min
Source Change 340 nm
Light Source D2/WI
Filter Exchange Step
Correction Baseline

[Data Information]
Creation Date 2014-2-26 9:33

Data array type Linear data array
Horizontal Wavelength [nm]
Vertical Abs
Start 400 nm
End 190 nm
Data pitch 0.2 nm
Data points 1051

Figure S98. The UV spectrum of compound **8**

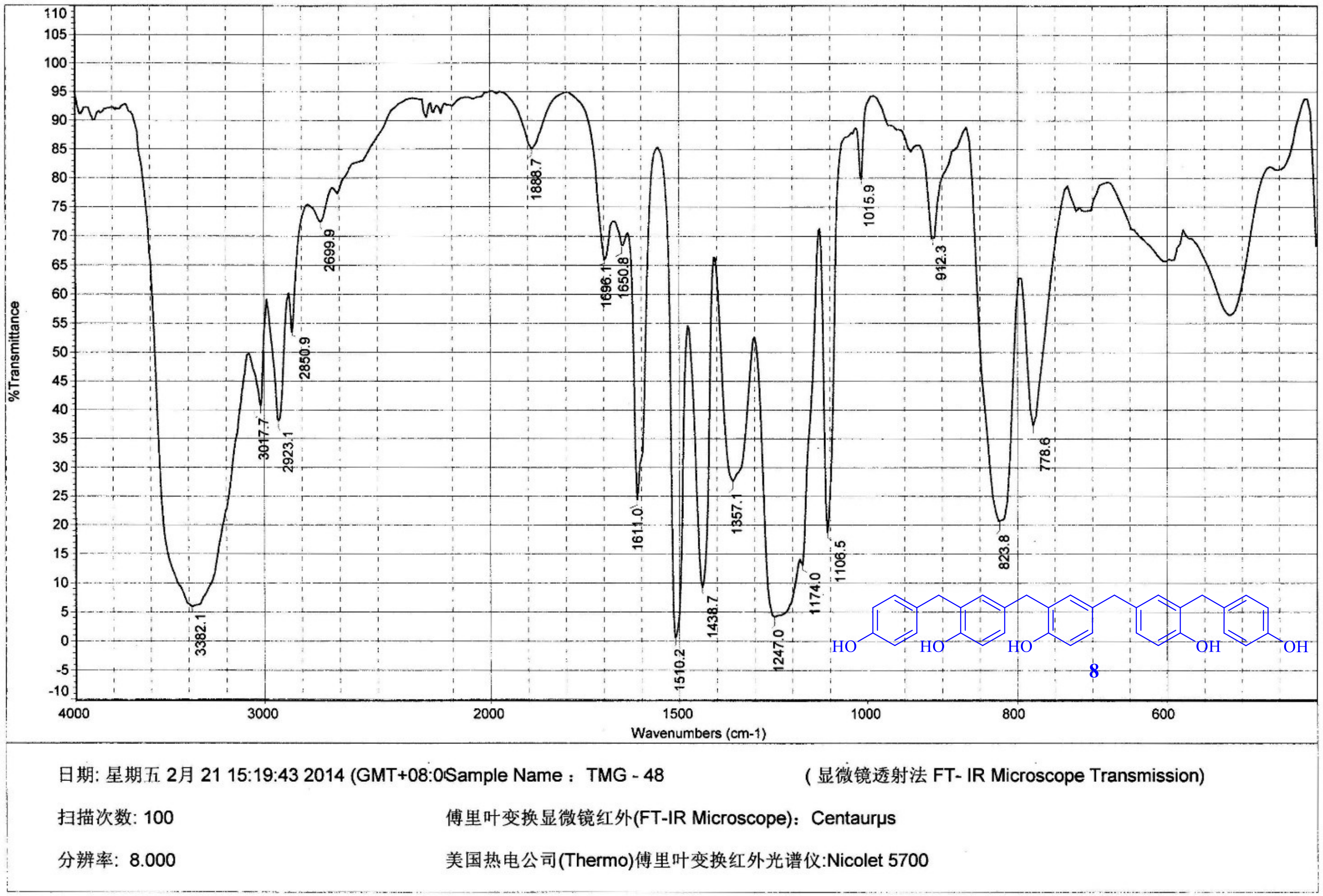

Figure S99. The IR spectrum of compound **8**

Single Mass Spectrum Deconvolution Report

**Analysis Name:**	guoql061.d	**Instrument:**	LC-MSD-Trap-SL	**Print Date:**	7/3/2012 11:38:26 AM
**Method:**	TEST.MS	**Operator:**	Operator	**Acq. Date:**	7/3/2012 11:31:01 AM
**Sample Name:**	TMG-48				
**Analysis Info:**					

Acquisition Parameter:

Mass Range Mode	Std/Normal	Trap Drive	29.0	Scan Begin	100 m/z
Ion Polarity	Positive	Octopole RF Amplitude	152.8 Vpp	Scan End	800 m/z
Ion Source Type	ESI	Capillary Exit	102.3 Volt	Averages	5 Spectra
Dry Temp (Set)	330 °C	Skimmer	40.0 Volt	Max. Accu Time	200000 µs
Nebulizer (Set)	15.00 psi	Oct 1 DC	12.00 Volt	ICC Target	20000
Dry Gas (Set)	5.00 l/min	Oct 2 DC	1.70 Volt	Charge Control	on

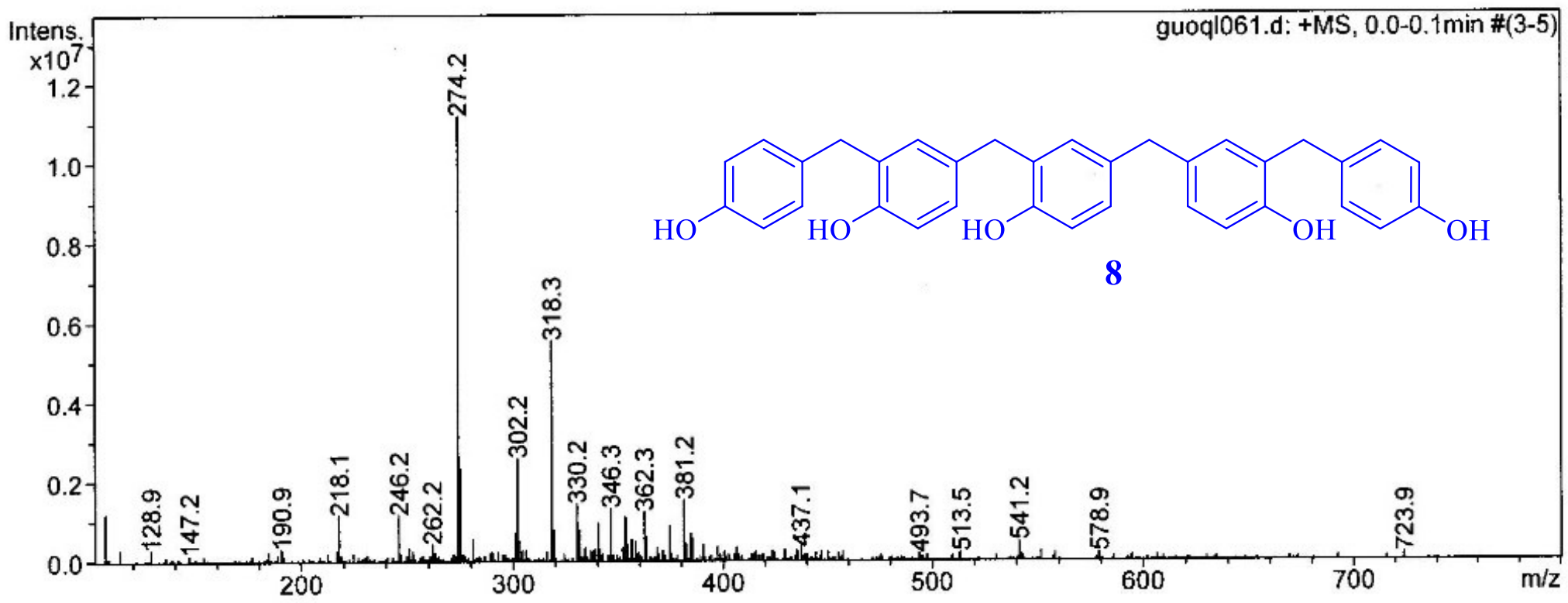

Component Molecular Mass Molecule Absolute Abundance Relative Abundance

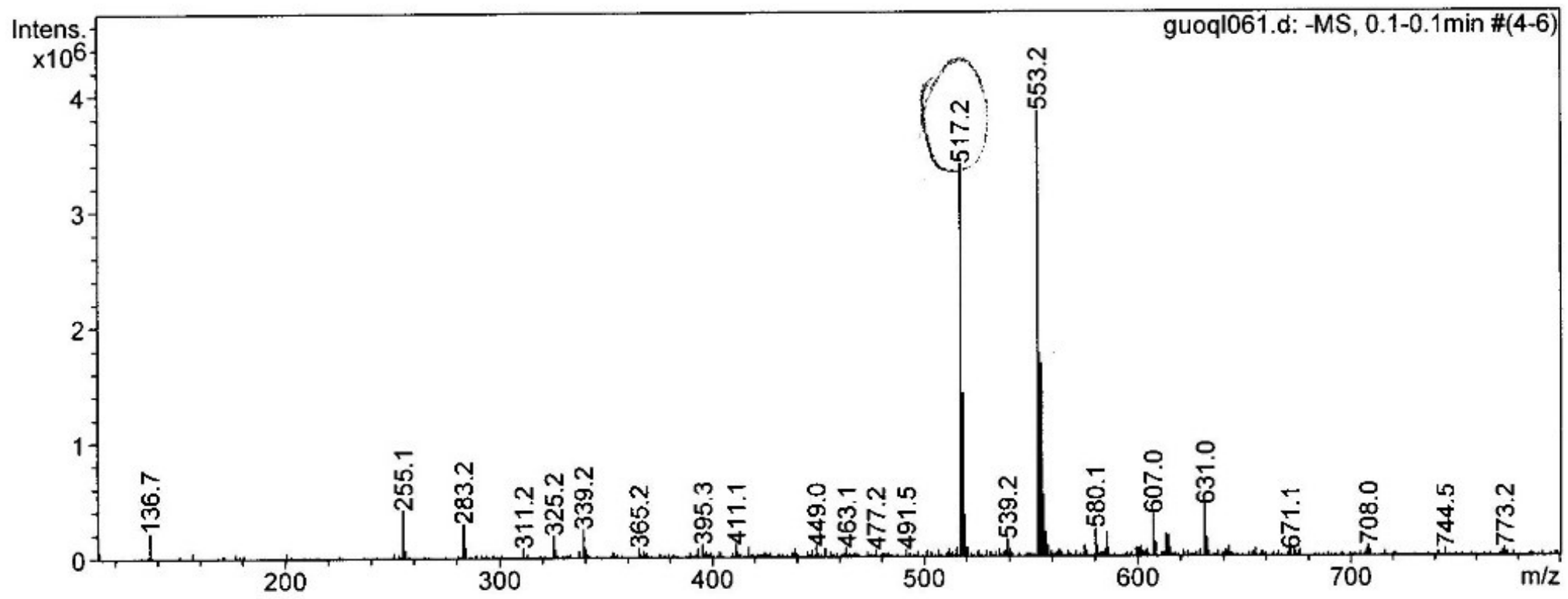

Component Molecular Mass Molecule Absolute Abundance Relative Abundance

MSD Trap Report v 4 (A4-Opt2) Page 1 of 1 Agilent Technologies

Figure S100. The ESIMS of compound **8**

Qualitative Analysis Report

**Data Filename**	2014022607.d	**Sample Name**	TMG-48
**Sample Type**	Sample	**Position**	P1-C7
**Instrument Name**	Instrument 1	**User Name**	
**Acq Method**		**IRM Calibration Status**	Success
**DA Method**	TEST LCMS.m	**Comment**	

User Chromatograms

Fragmentor Voltage 135 **Collision Energy** 0 **Ionization Mode** ESI

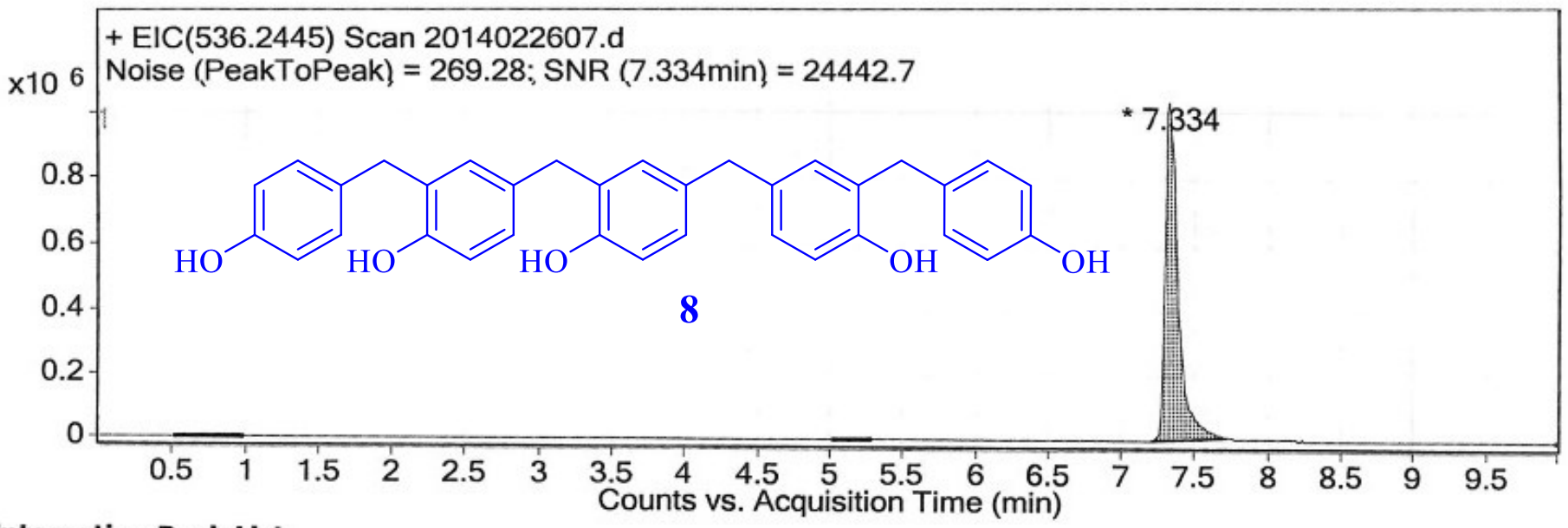

Integration Peak List

Peak	Start	RT	End	Height	Area	Area %	Signal To Noise
1	7.205	7.334	7.72	1026338	6581901	100	24442.7

Noise Measurements

Noise Type	Signal Definition	Noise Multiplier	Noise Value
Peak-to-Peak	Area	1	269.2791748

Noise Regions

Start	End
0.5	1
5	5.3
9.99	11

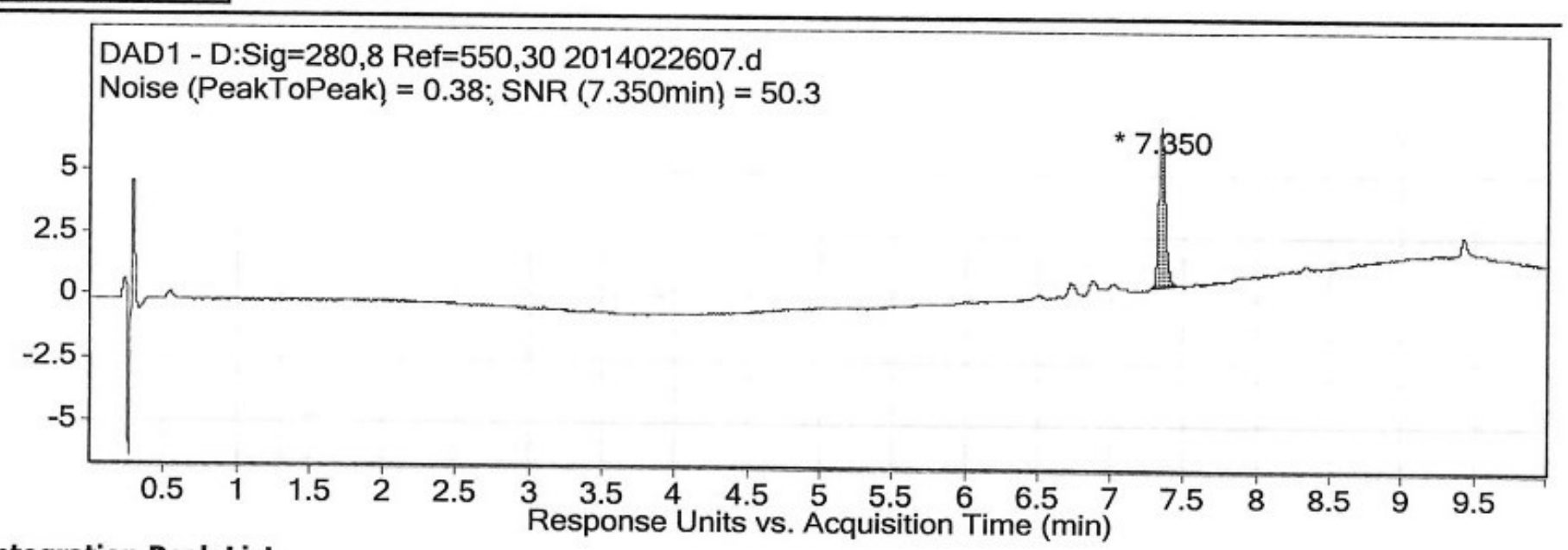

Integration Peak List

Peak	Start	RT	End	Height	Area	Area %	Signal To Noise
1	7.277	7.35	7.449	6.37	18.99	100	50.3

Noise Measurements

Noise Type	Signal Definition	Noise Multiplier	Noise Value
Peak-to-Peak	Area	1	0.377178192

Noise Regions

Start	End
0.5	1
5	5.3
9.99	11

User Spectra

Figure S101. The HR-ESIMS report of compound **8** (page 1)

Qualitative Analysis Report

Fragmentor Voltage	Collision Energy	Ionization Mode
135	0	Esi

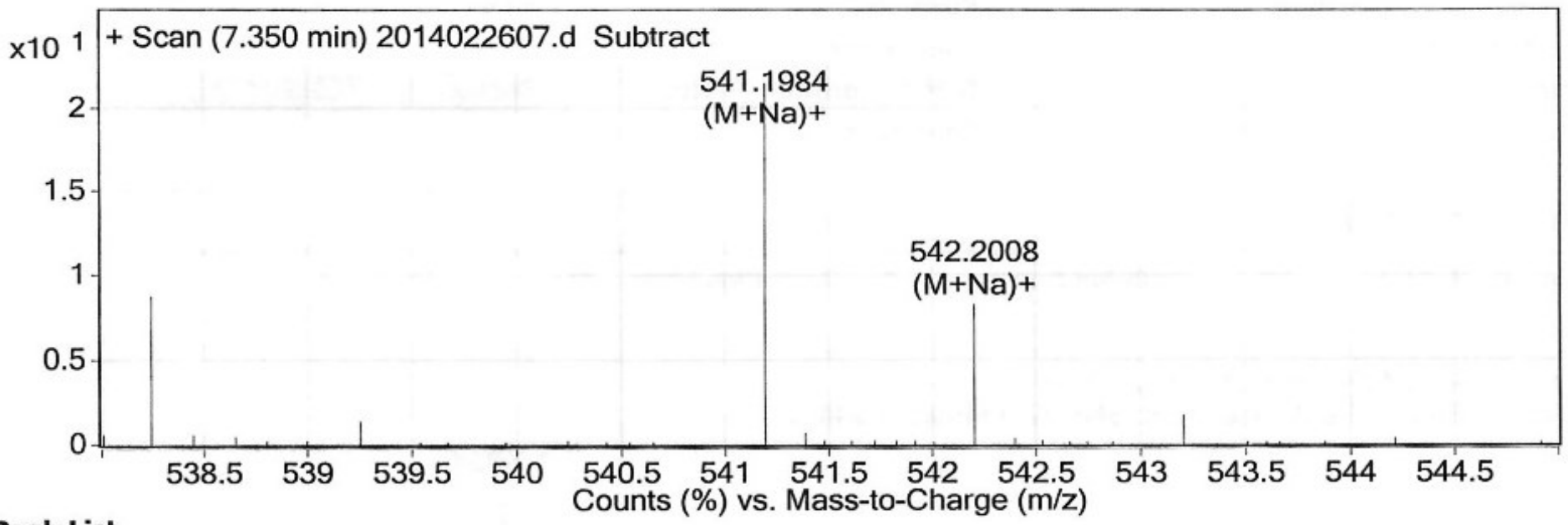

Peak List

m/z	z	Abund	Formula	Ion
227.1637	1	381554		
241.1733		103769		
244.1899		283474		
249.1457		94475		
536.2437	1	927620		
537.2465	1	378930		
541.1984	1	199472	C34 H30 Na O5	(M+Na)+
550.258	1	139739		
557.1719	1	155244		
922.0097		130242		

Formula Calculator Element Limits

Element	Min	Max
C	3	100
H	0	500
O	0	90
N	0	5
S	0	5
Cl	0	2
Br	0	0
Si	0	0
F	0	0
P	0	0

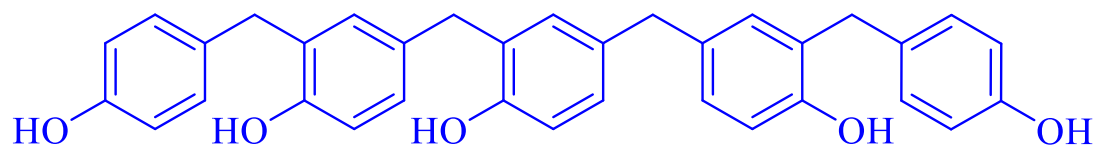

8

Formula Calculator Results

Formula	Best	Mass	Tgt Mass	Diff (ppm)	Ion Species	Score
C34 H30 O5	TRUE	518.2091	518.2093	0.36	C34 H30 Na O5	99.9
C35 H26 N4 O		518.2092	518.2107	2.92	C35 H26 N4 Na O	99.73
C38 H30 S		518.2091	518.2068	-4.48	C38 H30 Na S	98.74
C26 H34 N2 O7 S		518.2092	518.2087	-0.93	C26 H34 N2 Na O7 S	97.81
C35 H34 S2		518.2091	518.2102	2.02	C35 H34 Na S2	97.48

--- End Of Report ---

Agilent Technologies Page 2 of 2 Printed at: 10:05 AM on: 2/26/2014

Figure S102. The HR-ESIMS report of compound **8** (page 2)

MS Formula Results: + Scan (7.350 min) Sub (2014022607.d)

m/z	Ion	Formula	Abundance
541.1984	(M+Na)+	C34 H30 Na O5	199472.1

Best	Formula (M)	Ion Formula	Calc m/z	Score	Cross S	Mass	Calc Mass	Diff (ppm)	Abs Diff (ppm)	Abund Match	Spacing Mat	Mass Match	m/z	DBE
☑	C34 H30 O5	C34 H30 Na O5	541.1985	99.9		518.2091	518.2093	0.36	0.36	99.92	99.69	100	541.1984	20
☐	C35 H26 N4 O	C35 H26 N4 Na O	541.1999	99.73		518.2092	518.2107	2.92	2.92	99.76	99.69	99.73	541.1984	25
☐	C38 H30 S	C38 H30 Na S	541.196	98.74		518.2091	518.2068	-4.48	4.48	97.6	98.85	99.36	541.1984	24
☐	C26 H34 N2 O7 S	C26 H34 N2 Na O7 S	541.1979	97.81		518.2092	518.2087	-0.93	0.93	93.77	98.33	99.97	541.1984	11
☐	C35 H34 S2	C35 H34 Na S2	541.1994	97.48		518.2091	518.2102	2.02	2.02	93.06	98.01	99.87	541.1984	19

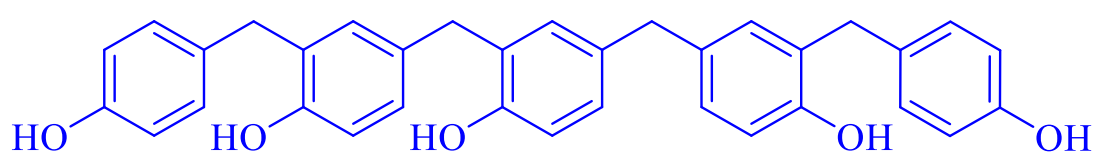

8

page 1

Figure S103. The HR-ESIMS report of compound **8** (page 3)

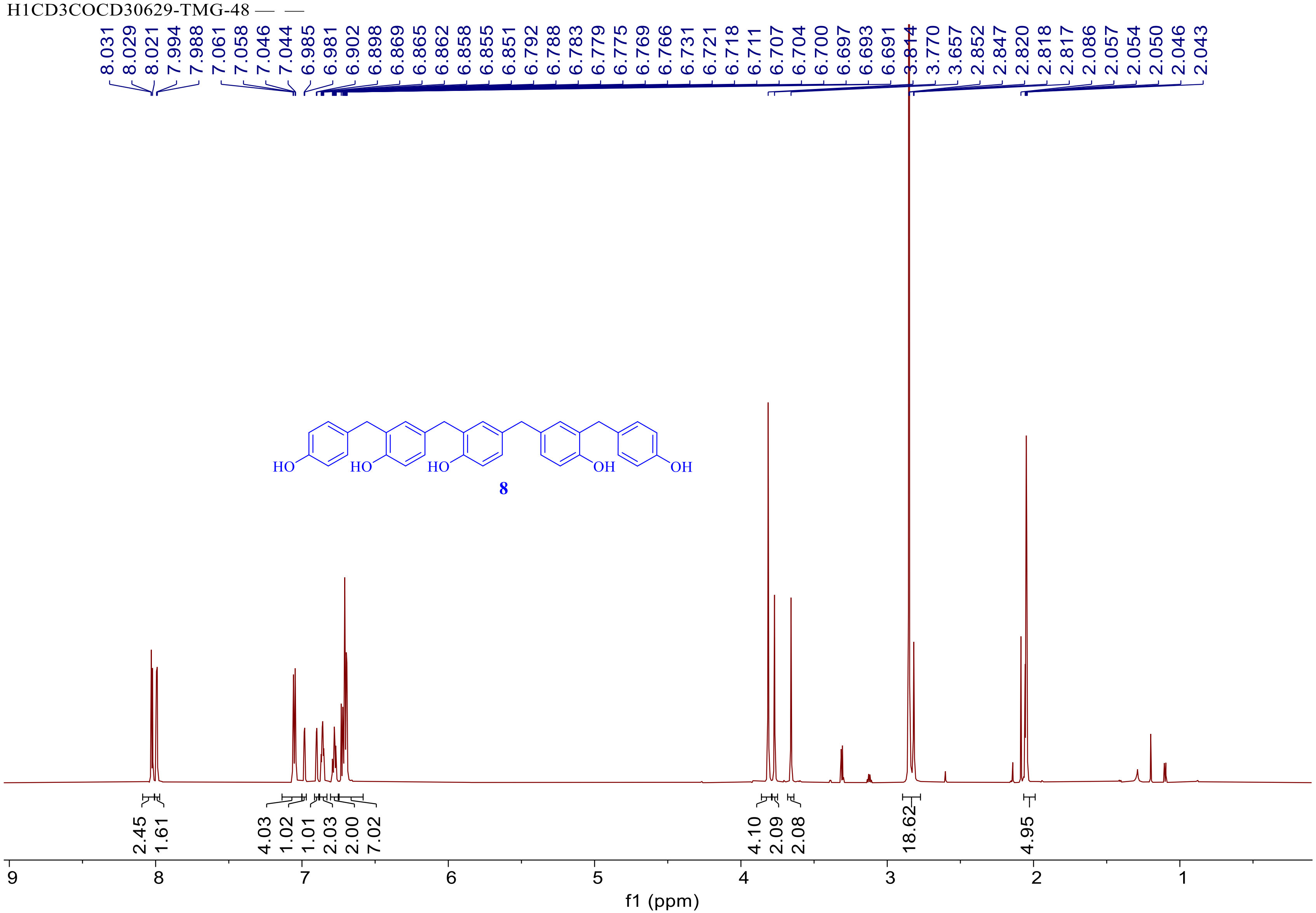

Figure S104. The ^{1}H NMR spectrum of compound **8** in acetone-d_6 (600 MHz)

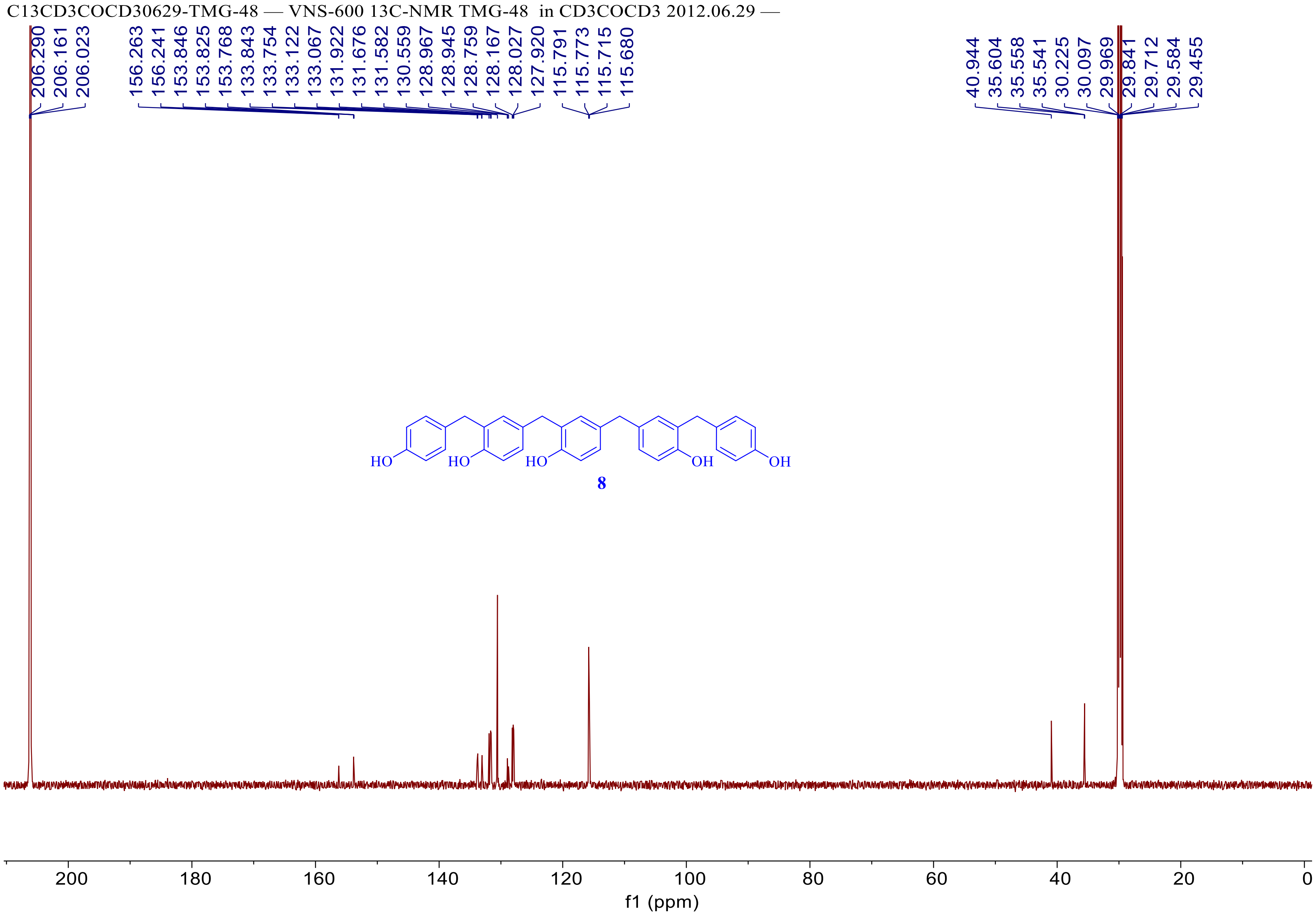

Figure S105. The ^{13}C NMR spectrum of compound **8** in acetone-d_6 (150 MHz)

DEPTCD3COCD30629-TMG-48 — VNS-600 DEPT-NMR TMG-48 in CD3COCD3 2012.06.29 —

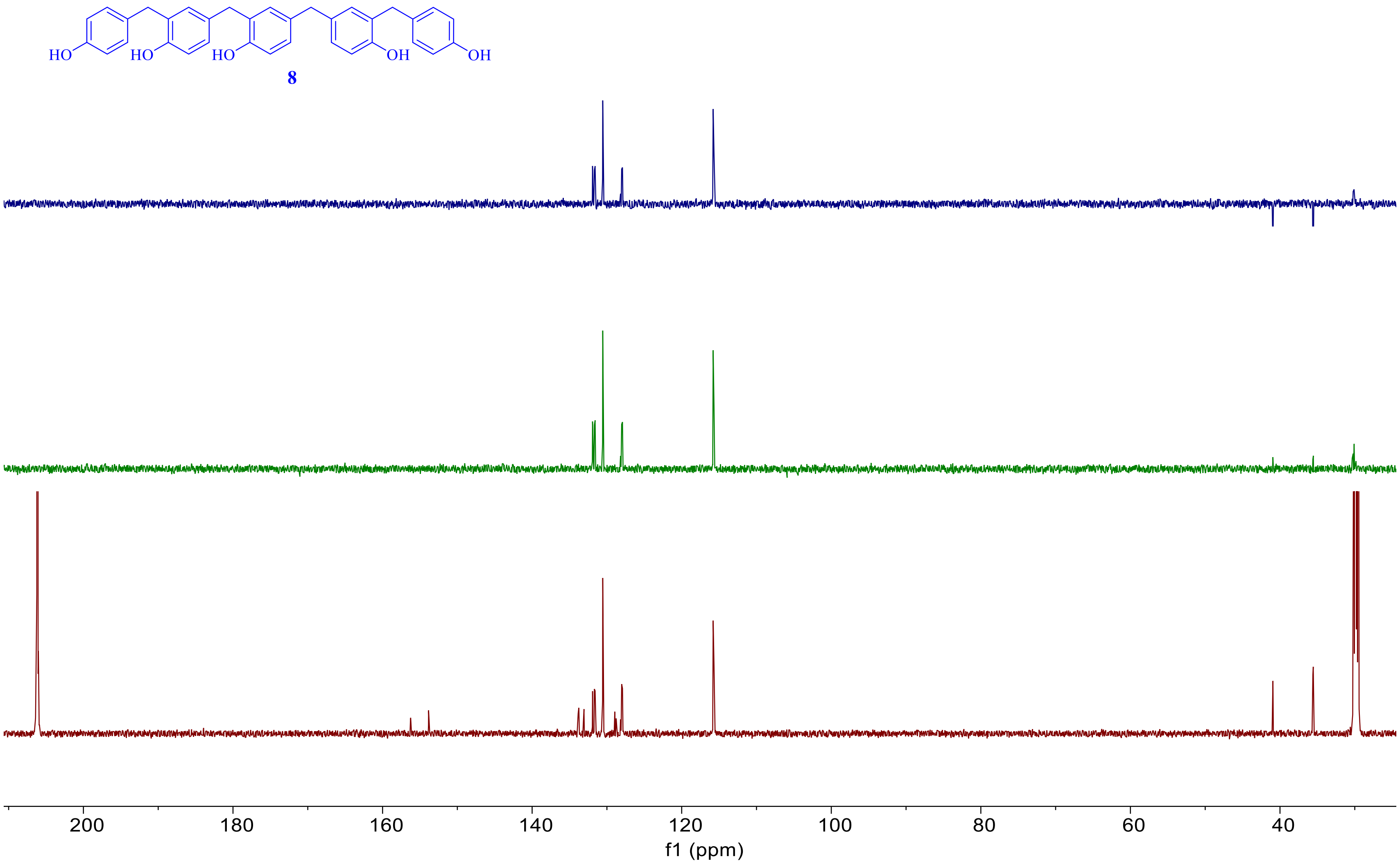

Figure S106. The DEPT spectrum of compound **8** in acetone-d_6 (150 MHz)

COSYCD3COCD30629-TMG-48 — VNS-600 gCOSY TMG-48 in CD3COCD3 2012.06.29 —

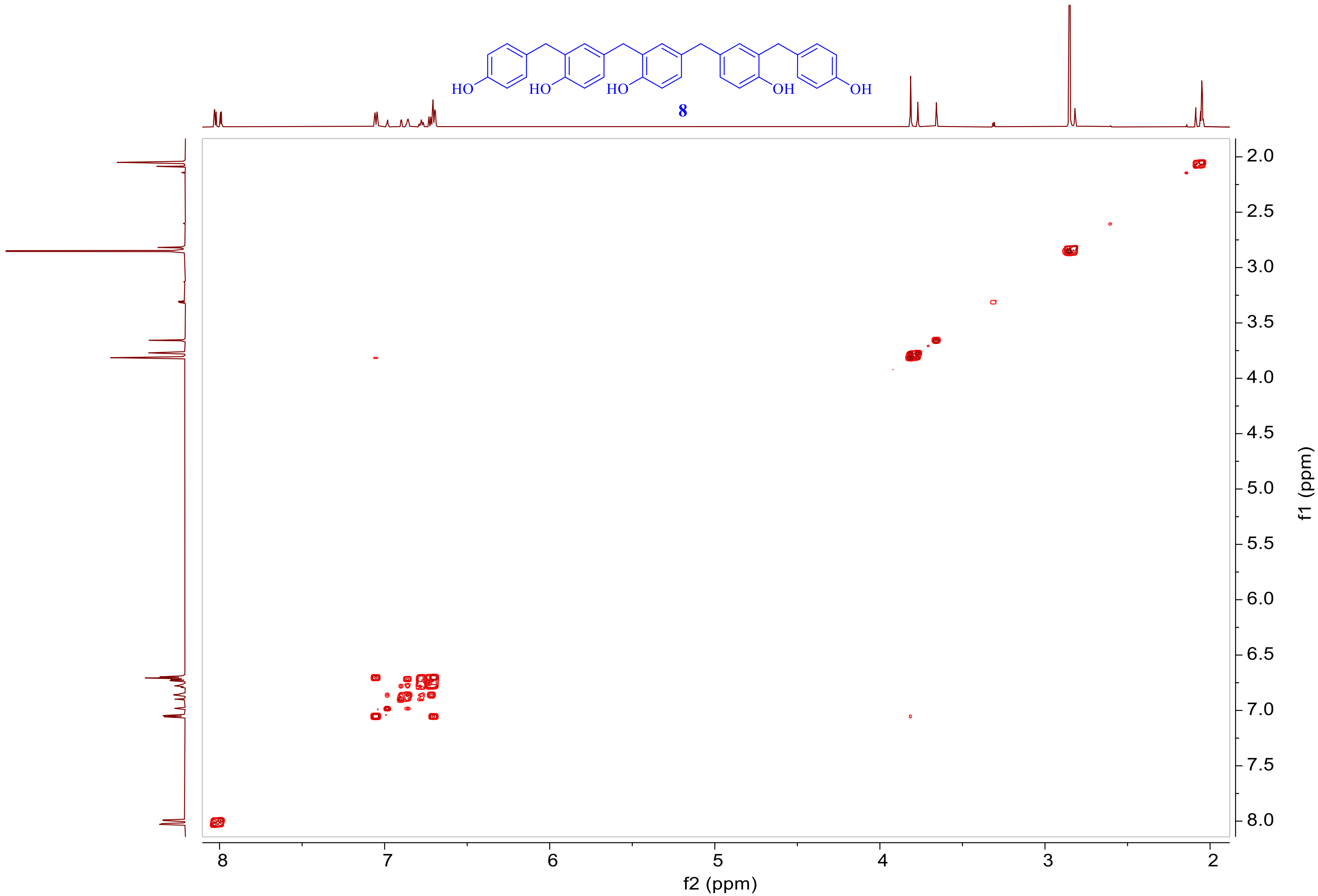

Figure S107. The ^{1}H-^{1}H COSY spectrum of compound **8** in acetone-d_6 (600 MHz)

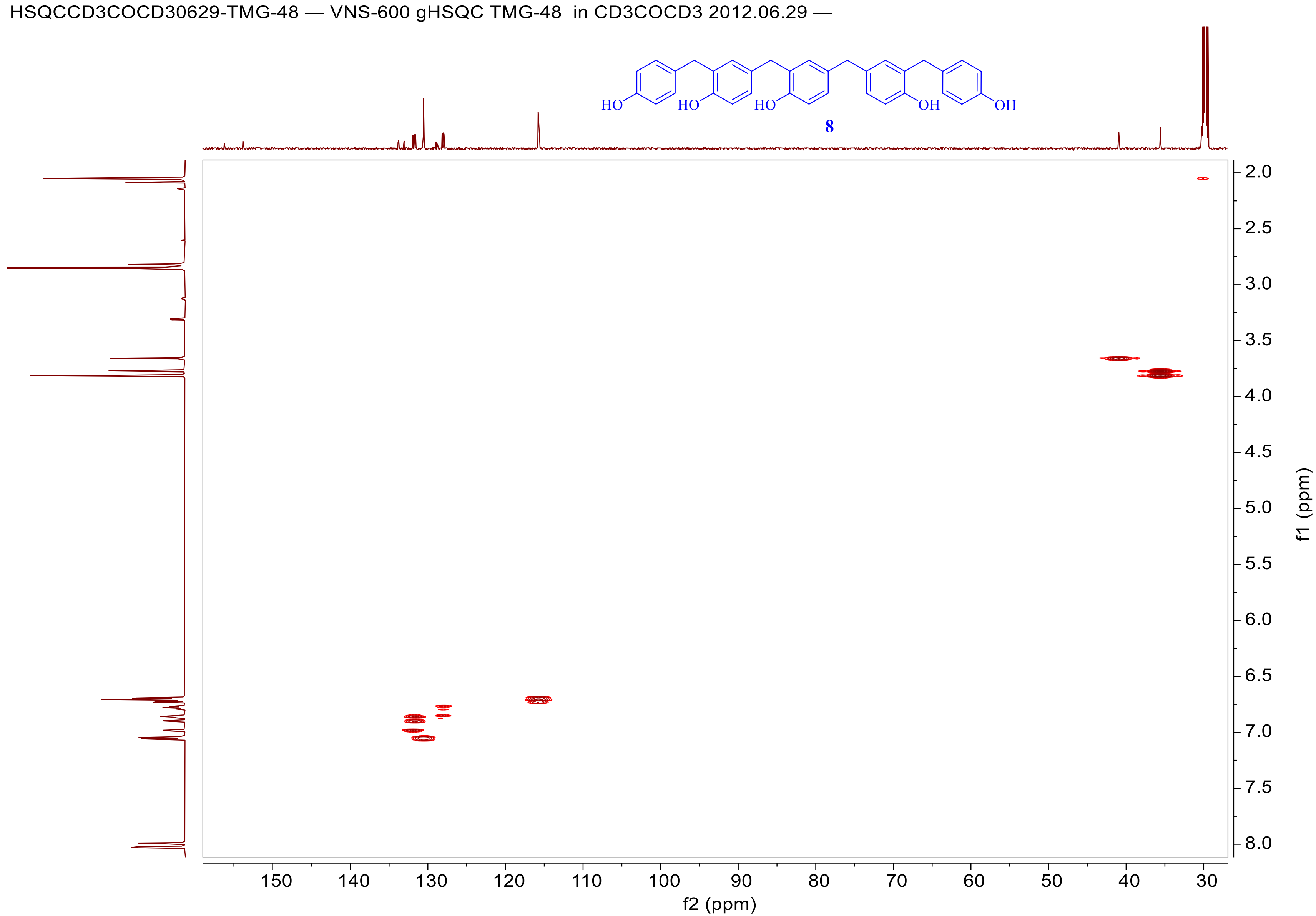

Figure S108. The HSQC spectrum of compound **8** in acetone-d_6 (600 MHz for ^{1}H)

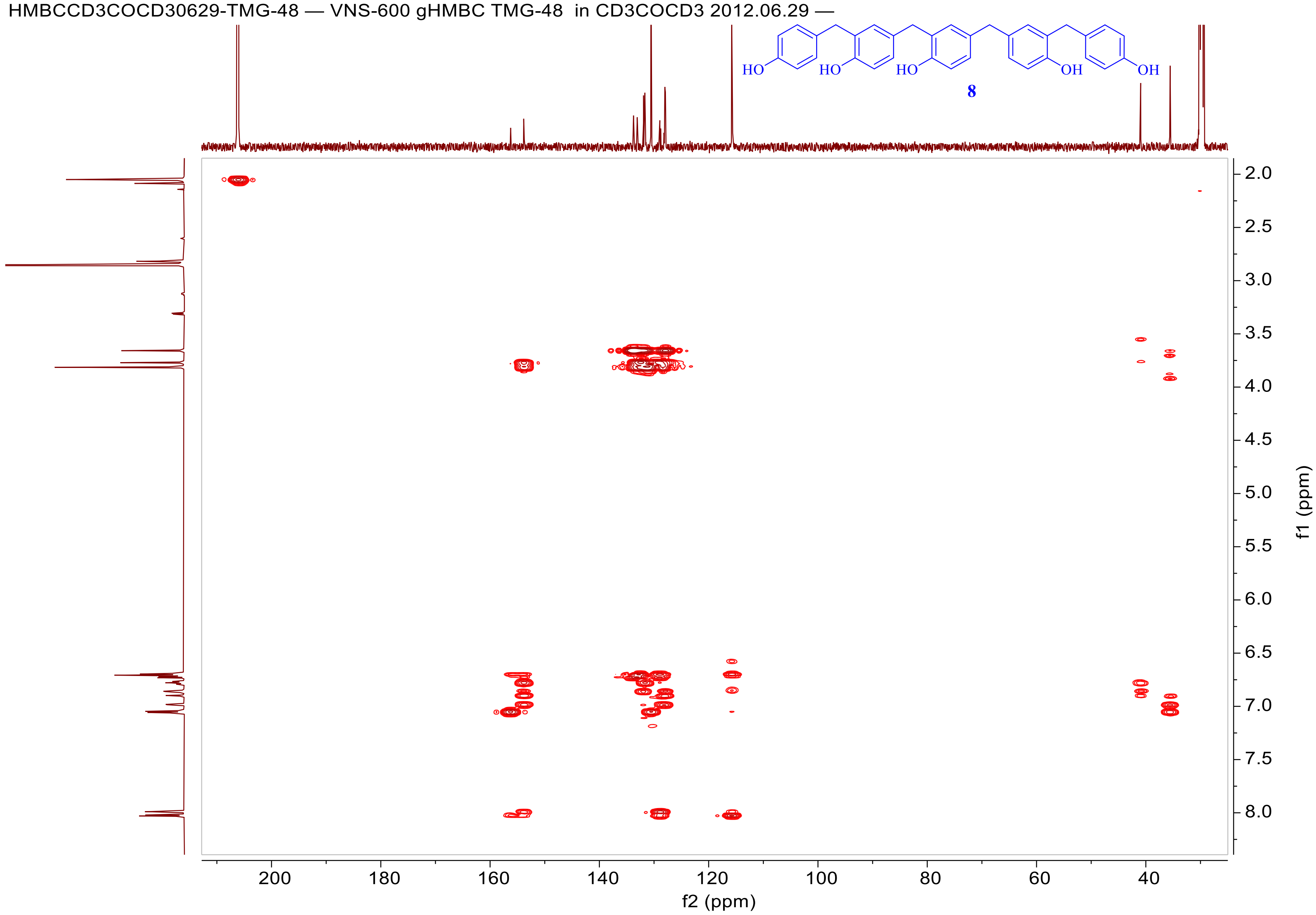

Figure S109. The HMBC spectrum of compound **8** in acetone-d_6 (600 MHz for ^1H)

DD2-500 NOESY1D TMG-48 IN acetone coldprobe-Probe

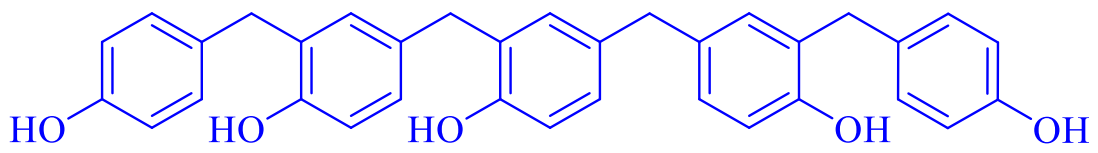

8

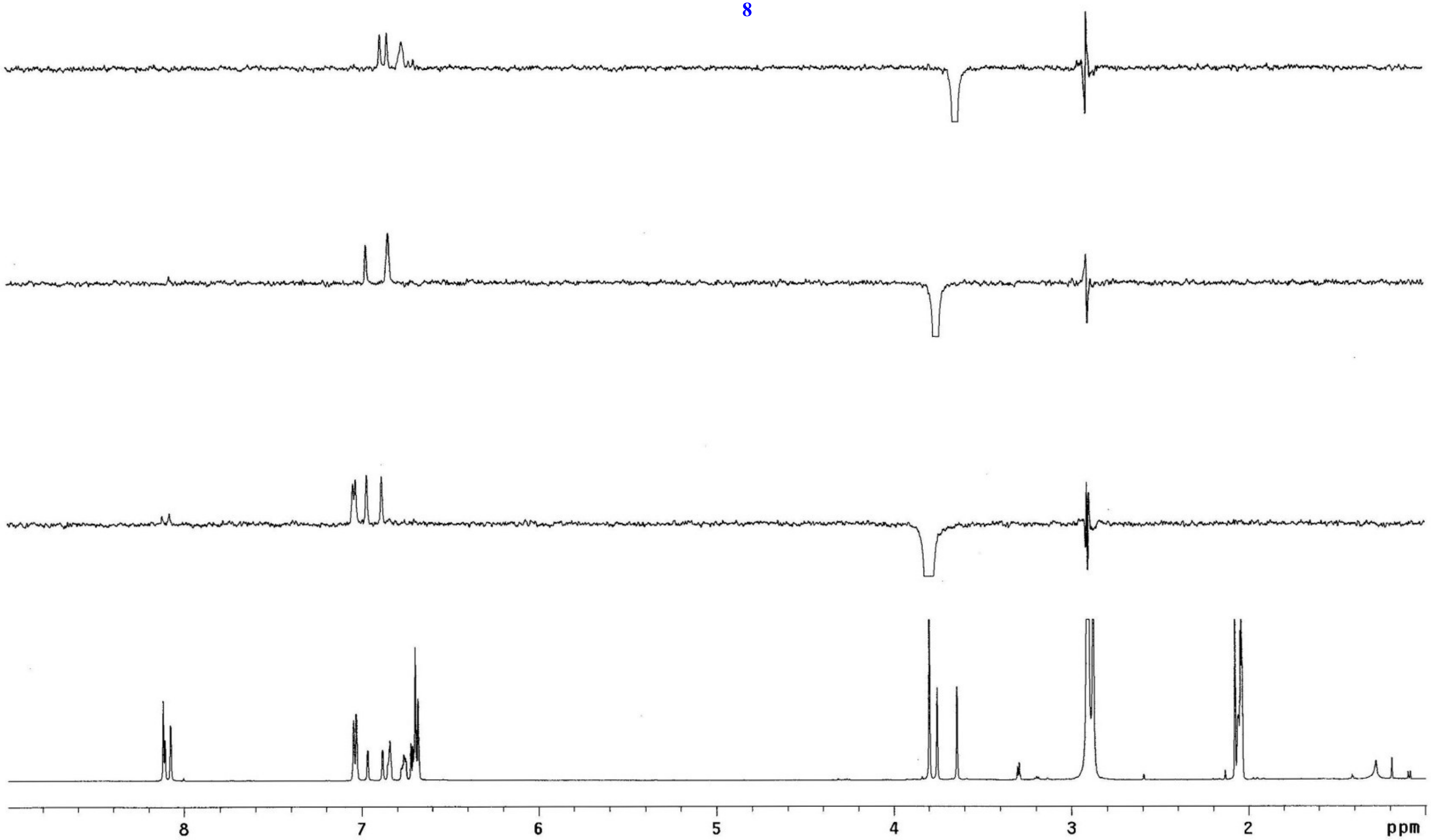

Figure S110. The NOESY1D spectrum of compound **8** in acetone-d_6 (500 MHz)

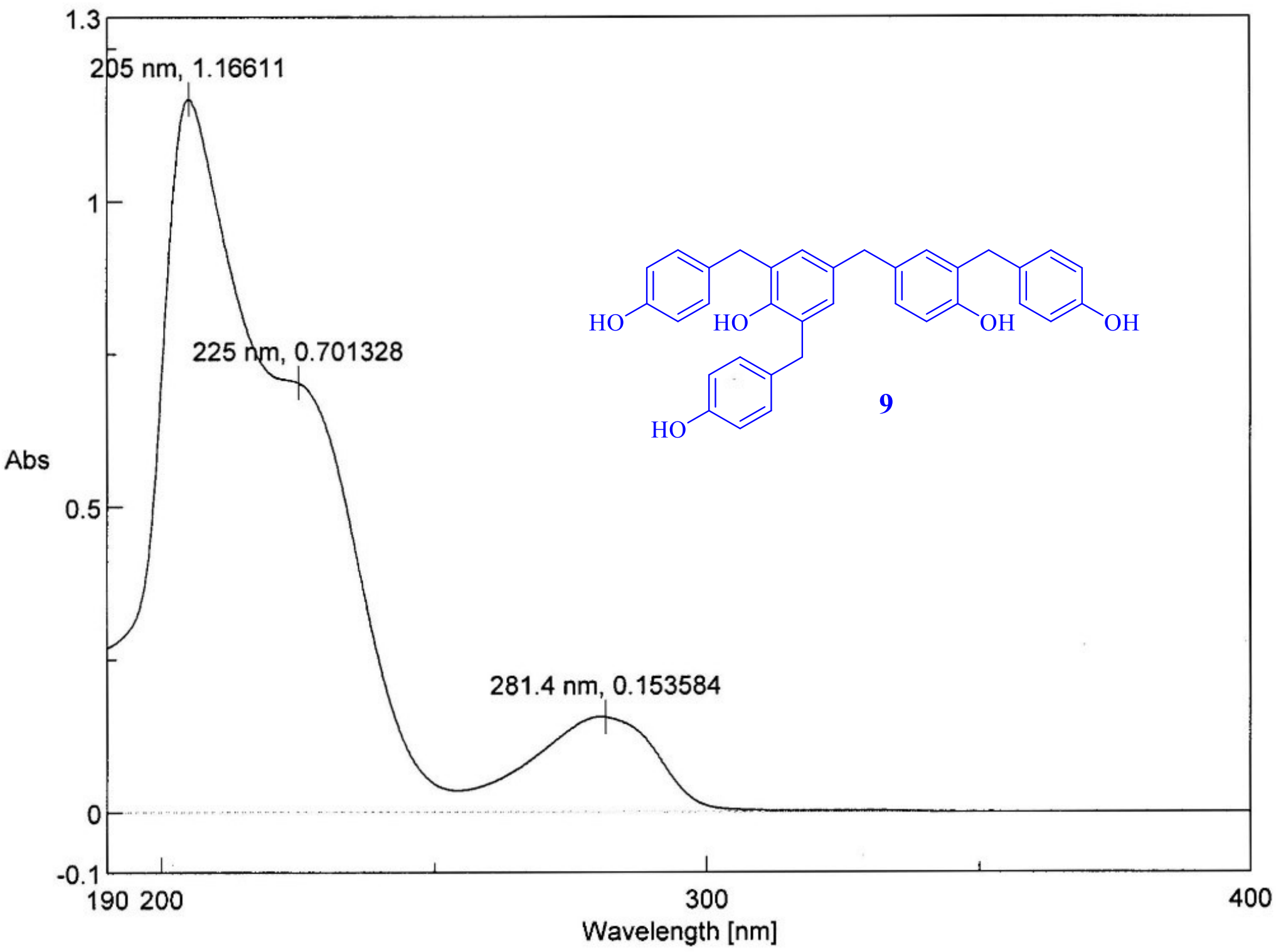

Memory-15

[Comment]

Sample Name	TMG-62
Comment	0.02
User	
Division	UV
Company	324

[Measurement Information]

Instrument Name	V-650
Model Name	V-650
Serial No.	A034461150
Accessory	PSC-718
Accessory S/N	A001761114
Position	1
Cell Length	10 mm
Temperature	19.95 C
Control Sonsor	Holder
Monitor Sensor	Holder
Start Mode	Start immediately
Photometric Mode	Abs
Measurement range	400 - 190 nm
Data pitch	0.2 nm
Band width(UV/Vis)	2.0 nm
Response	Medium
Scanning speed	200 nm/min
Source Change	340 nm
Light Source	D2/WI
Filter Exchange	Step
Correction	Baseline

[Data Information]

Creation Date	2014-2-26 9:38
Data array type	Linear data array
Horizontal	Wavelength [nm]
Vertical	Abs
Start	400 nm
End	190 nm
Data pitch	0.2 nm
Data points	1051

Figure S111. The UV spectrum of compound **9**

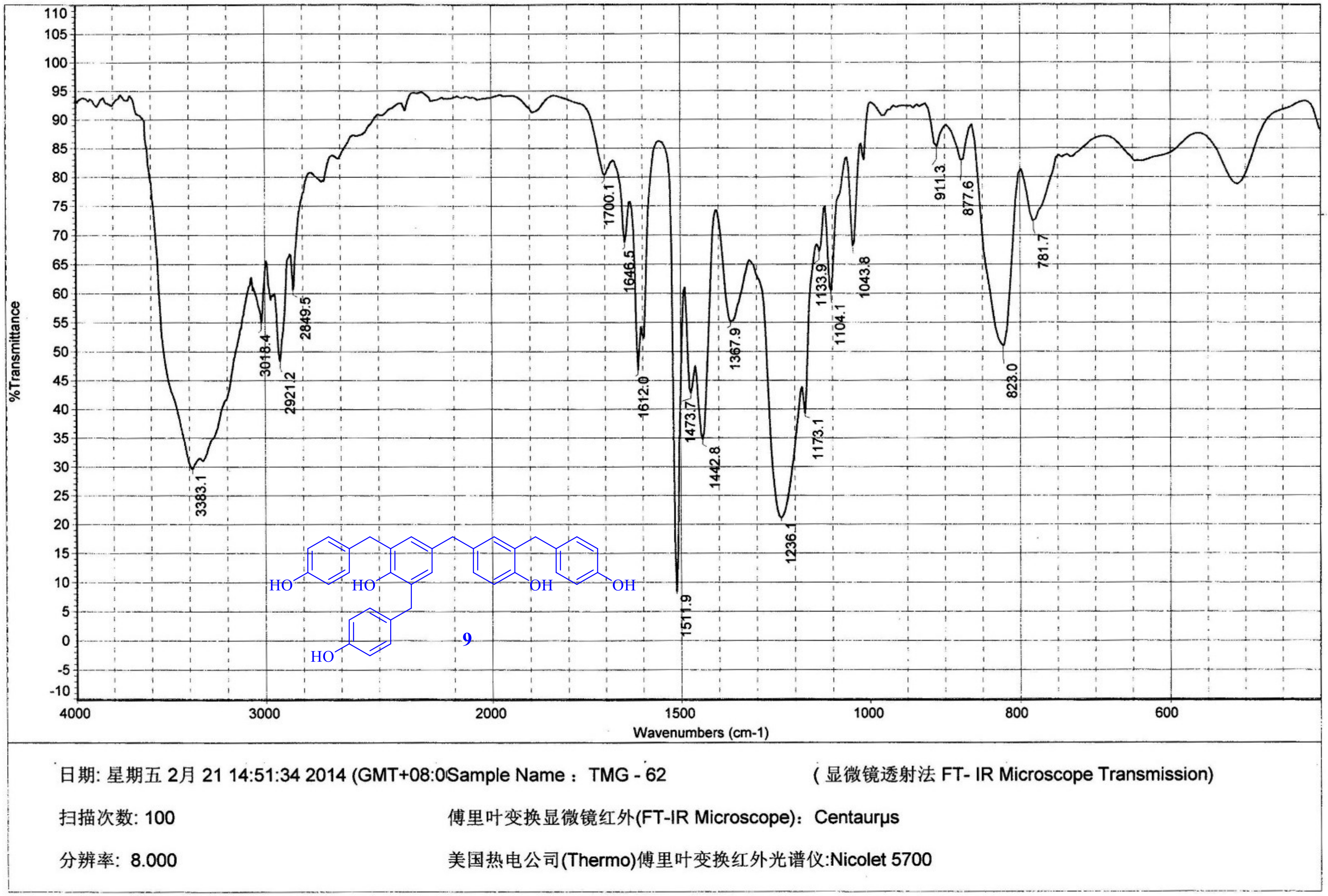

Figure S112. The IR spectrum of compound **9**

Single Mass Spectrum Deconvolution Report

Analysis Name: guoql076.d **Instrument:** LC-MSD-Trap-SL **Print Date:** 10/30/2012 1:53:33 PM
Method: TEST.MS **Operator:** Operator **Acq. Date:** 10/30/2012 1:08:46 PM
Sample Name: TMG-62
Analysis Info:

Acquisition Parameter:

Mass Range Mode	Std/Normal	Trap Drive	45.5	Scan Begin	100 m/z
Ion Polarity	Positive	Octopole RF Amplitude	152.8 Vpp	Scan End	700 m/z
Ion Source Type	ESI	Capillary Exit	-102.3 Volt	Averages	5 Spectra
Dry Temp (Set)	330 °C	Skimmer	-40.0 Volt	Max. Accu Time	200000 µs
Nebulizer (Set)	15.00 psi	Oct 1 DC	-12.00 Volt	ICC Target	20000
Dry Gas (Set)	6.00 l/min	Oct 2 DC	-1.70 Volt	Charge Control	on

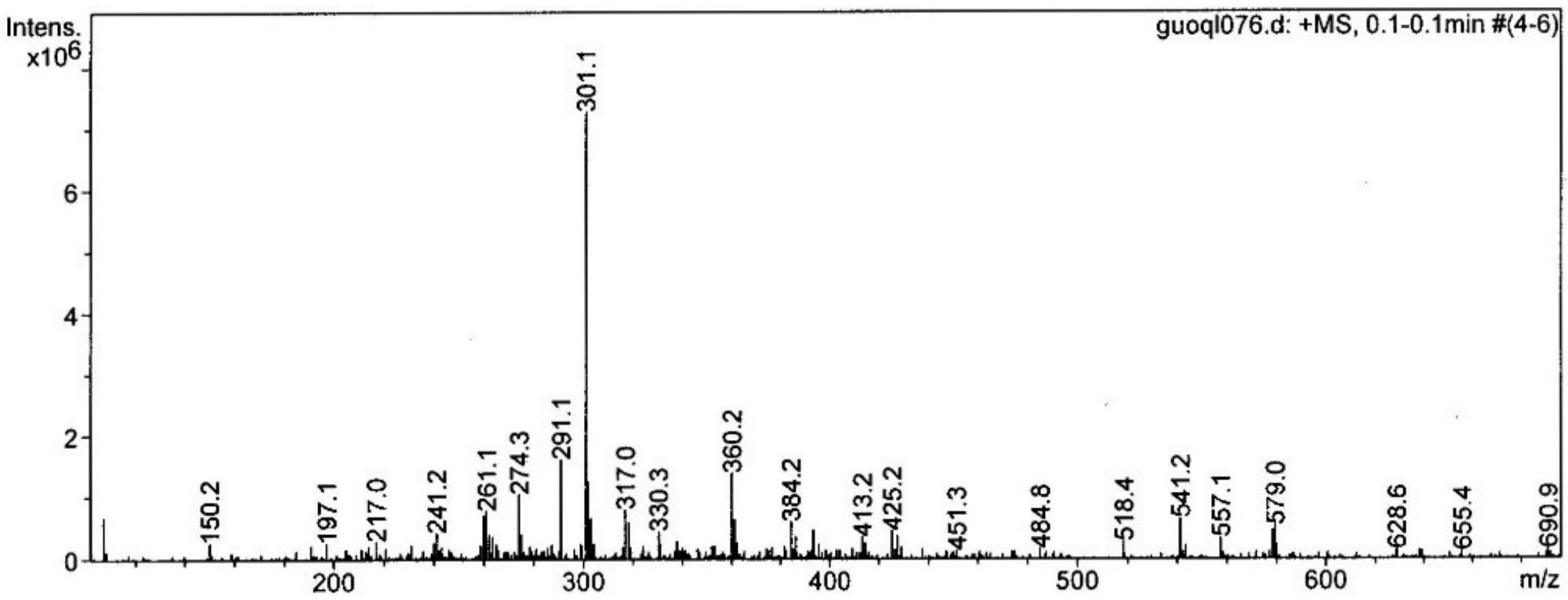

Component	Molecular Mass	Molecule	Absolute Abundance	Relative Abundance

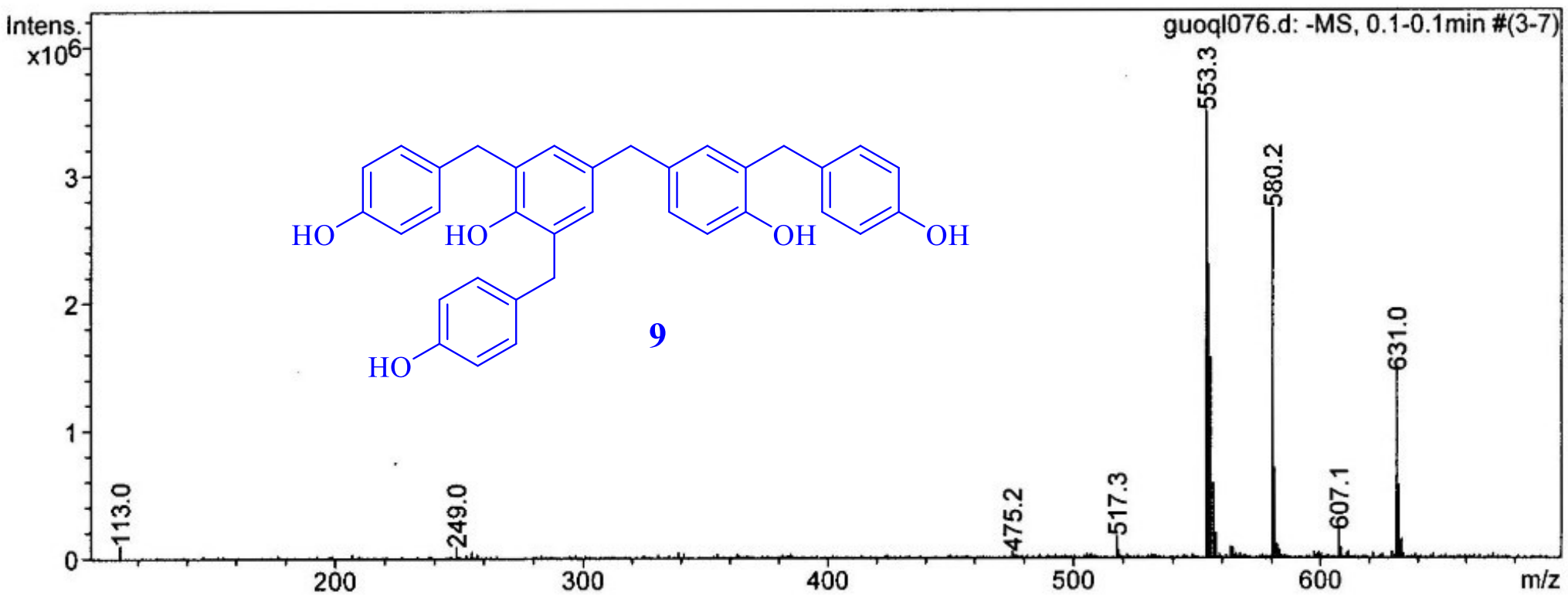

Component	Molecular Mass	Molecule	Absolute Abundance	Relative Abundance

Figure S113. The ESIMS of compound **9**

Qualitative Analysis Report

**Data Filename**	2014022609.d	**Sample Name**	TMG-62
**Sample Type**	Sample	**Position**	P1-C9
**Instrument Name**	Instrument 1	**User Name**	
**Acq Method**		**IRM Calibration Status**	Success
**DA Method**	TEST LCMS.m	**Comment**	

User Chromatograms

Fragmentor Voltage 135 **Collision Energy** 0 **Ionization Mode** ESI

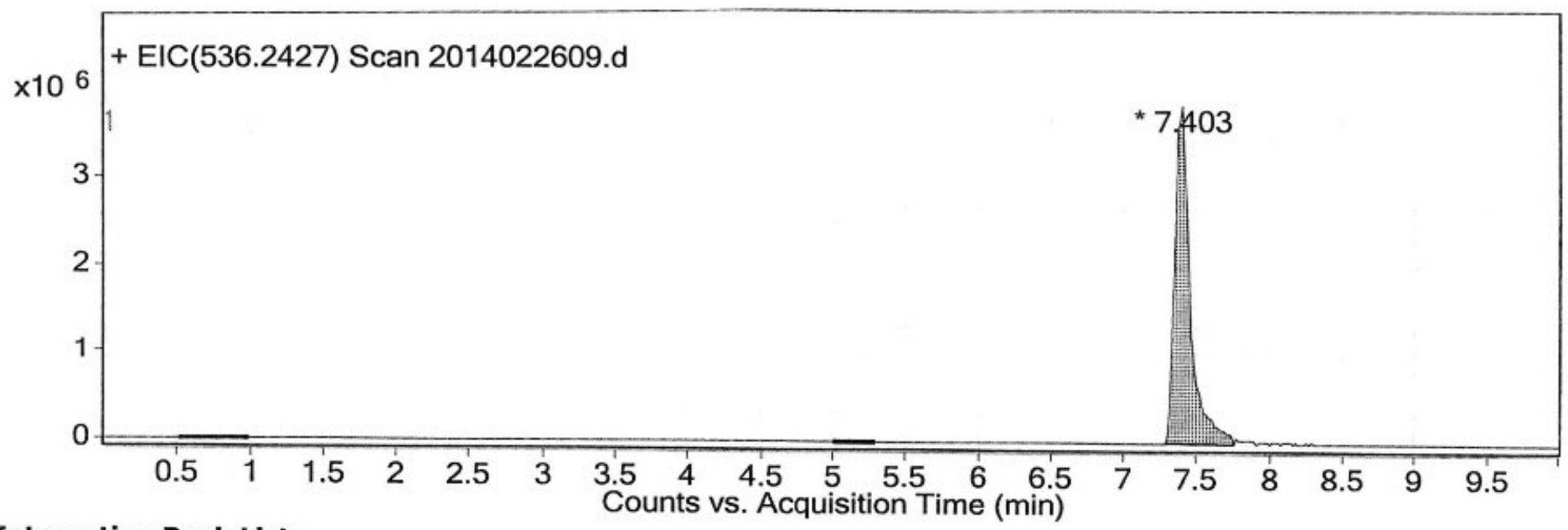

Integration Peak List

Peak	Start	RT	End	Height	Area	Area %	Signal To Noise
1	7.291	7.403	7.758	3802063	27918937	100	Infinity

Noise Measurements

Noise Type	Signal Definition	Noise Multiplier	Noise Value
Peak-to-Peak	Area	1	0

Noise Regions

Start	End
0.5	1
5	5.3
9.99	11

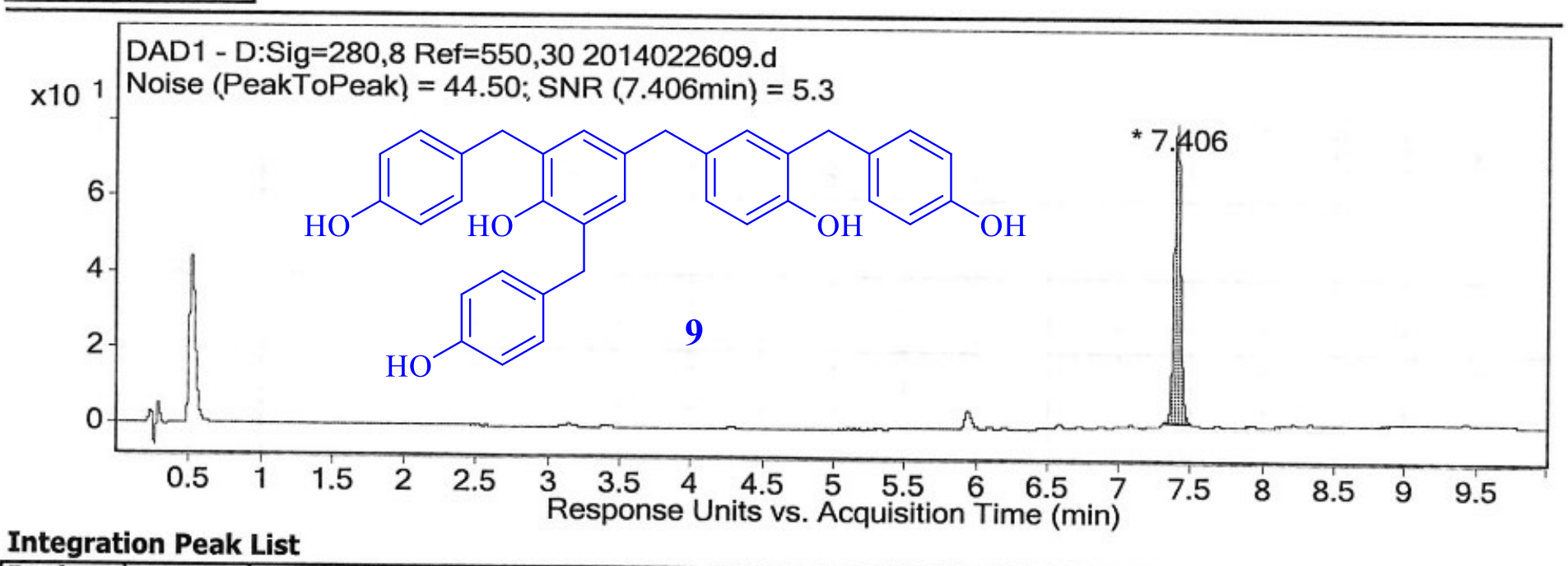

Integration Peak List

Peak	Start	RT	End	Height	Area	Area %	Signal To Noise
1	7.308	7.406	7.51	79.2	236.27	100	5.3

Noise Measurements

Noise Type	Signal Definition	Noise Multiplier	Noise Value
Peak-to-Peak	Area	1	44.50416565

Noise Regions

Start	End
0.5	1
5	5.3
9.99	11

User Spectra

Figure S114. The HR-ESIMS report of compound **9** (page 1)

Qualitative Analysis Report

Fragmentor Voltage	Collision Energy	Ionization Mode
135	0	ESI

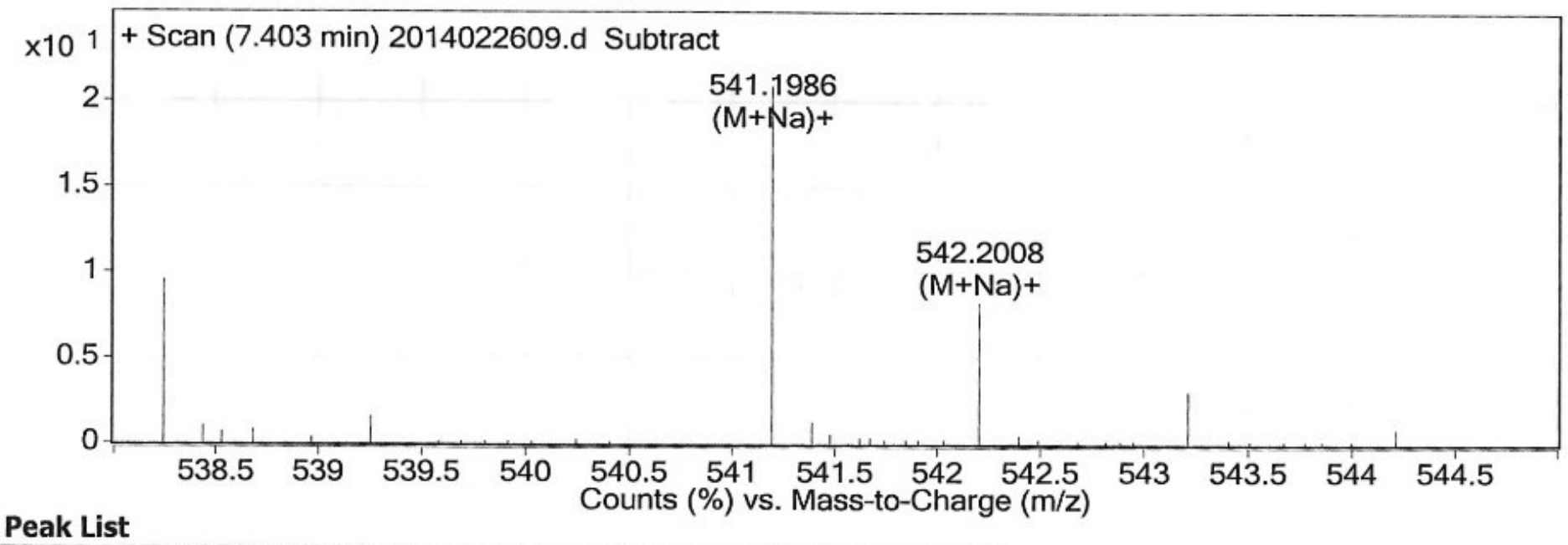

Peak List

m/z	z	Abund	Formula	Ion
274.2738		910187		
425.1739		409188		
536.2422	1	3809435		
536.4325		326846		
537.2453	1	1596717		
538.2479	1	365183		
541.1986	1	793545	C34 H30 Na O5	(M+Na)+
542.2008	1	312739	C34 H30 Na O5	(M+Na)+

Formula Calculator Element Limits

Element	Min	Max
C	3	100
H	0	500
O	0	90
N	0	5
S	0	5
Cl	0	2
Br	0	0
Si	0	0
F	0	0
P	0	0

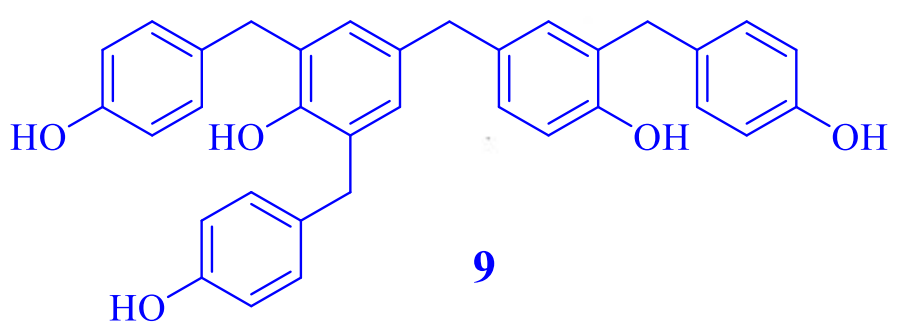

9

Formula Calculator Results

Formula	Best	Mass	Tgt Mass	Diff (ppm)	Ion Species	Score
C35 H34 S2		518.2093	518.2102	1.65	C35 H34 Na S2	98.67
C38 H30 S		518.2093	518.2068	-4.83	C38 H30 Na S	98.55
C34 H30 O5	TRUE	518.2093	518.2093	0.03	C34 H30 Na O5	98.35
C35 H26 N4 O		518.2093	518.2107	2.58	C35 H26 N4 Na O	97.98
C26 H34 N2 O7 S		518.2093	518.2087	-1.28	C26 H34 N2 Na O7 S	97.66

--- End Of Report ---

Agilent Technologies   Page 2 of 2   Printed at: 10:52 AM on: 2/26/2014

Figure S115. The HR-ESIMS report of compound **9** (page 2)

MS Formula Results: + Scan (7.403 min) Sub (2014022609.d)

m/z	Ion	Formula	Abundance
541.1986	(M+Na)+	C34 H30 Na O5	793544.8

Best	Formula (M)	Ion Formula	Calc m/z	Score	Cross S	Mass	Calc Mass	Diff (ppm)	Abs Diff (ppm)	Abund Match	Spacing Mat	Mass Match	m/z	DBE
☐	C35 H34 S2	C35 H34 Na S2	541.1994	98.67		518.2093	518.2102	1.65	1.65	99.53	95.15	99.91	541.1986	19
☐	C38 H30 S	C38 H30 Na S	541.196	98.55		518.2093	518.2068	-4.83	4.83	98.81	96.8	99.26	541.1986	24
☑	C34 H30 O5	C34 H30 Na O5	541.1985	98.35		518.2093	518.2093	0.03	0.03	95.26	98.76	100	541.1986	20
☐	C35 H26 N4 O	C35 H26 N4 Na O	541.1999	97.98		518.2093	518.2107	2.58	2.58	94.35	98.71	99.79	541.1986	25
☐	C26 H34 N2 O7 S	C26 H34 N2 Na O7 S	541.1979	97.66		518.2093	518.2087	-1.28	1.28	95.45	95.76	99.95	541.1986	11

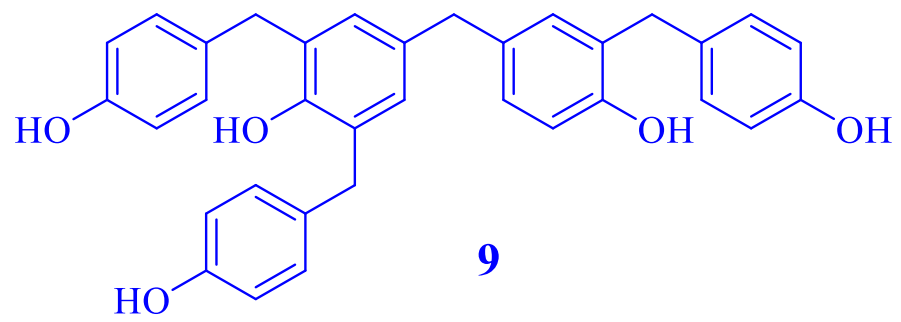

9

page 1

Figure S116. The HR-ESIMS report of compound **9** (page 3)

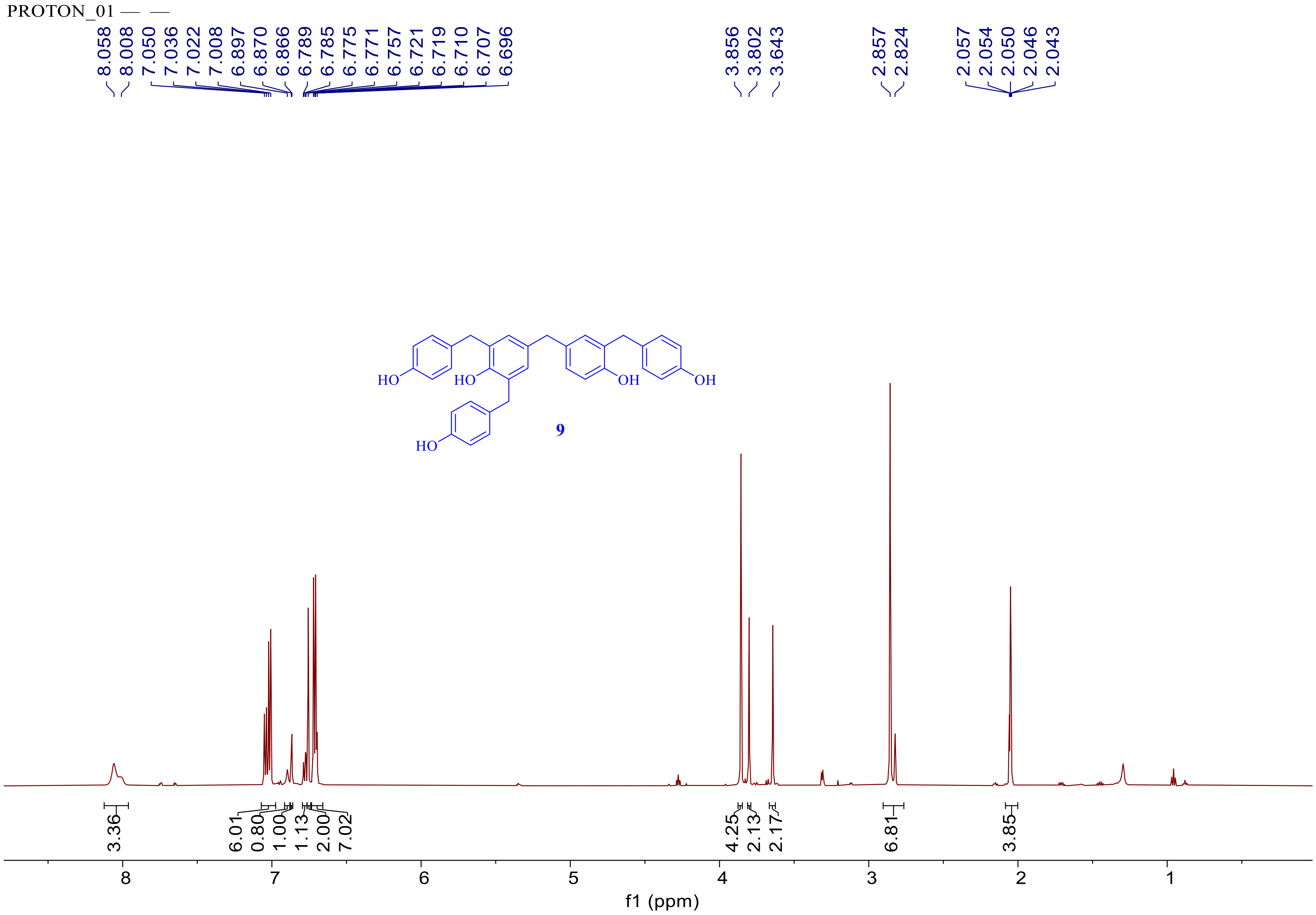

Figure S117. The ^{1}H NMR spectrum of compound **9** in acetone-d_6 (600 MHz)

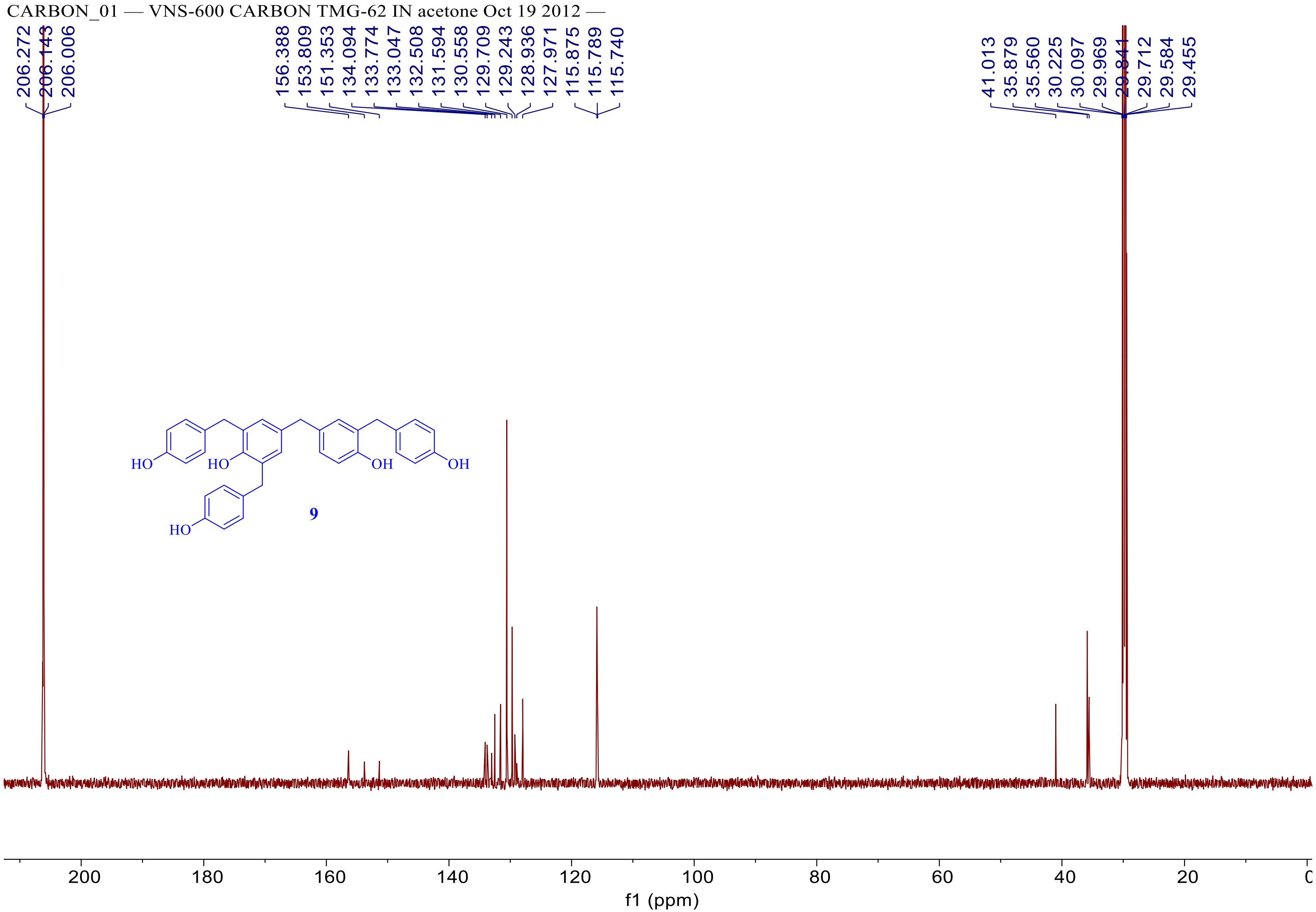

Figure S118. The ^{13}C NMR spectrum of compound **9** in acetone-d_6 (150 MHz)

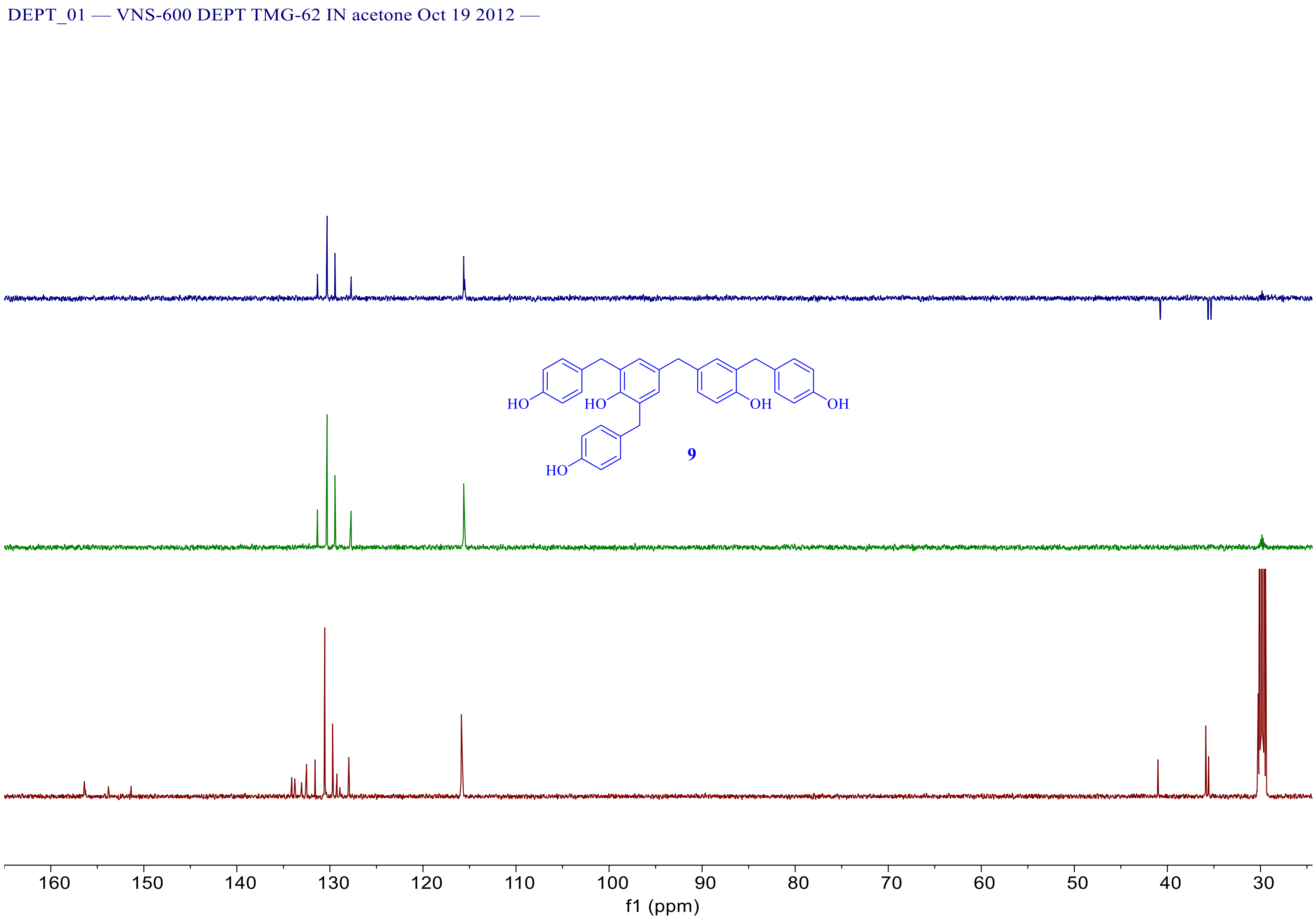

Figure S119. The DEPT spectrum of compound **9** in acetone-d_6 (150 MHz)

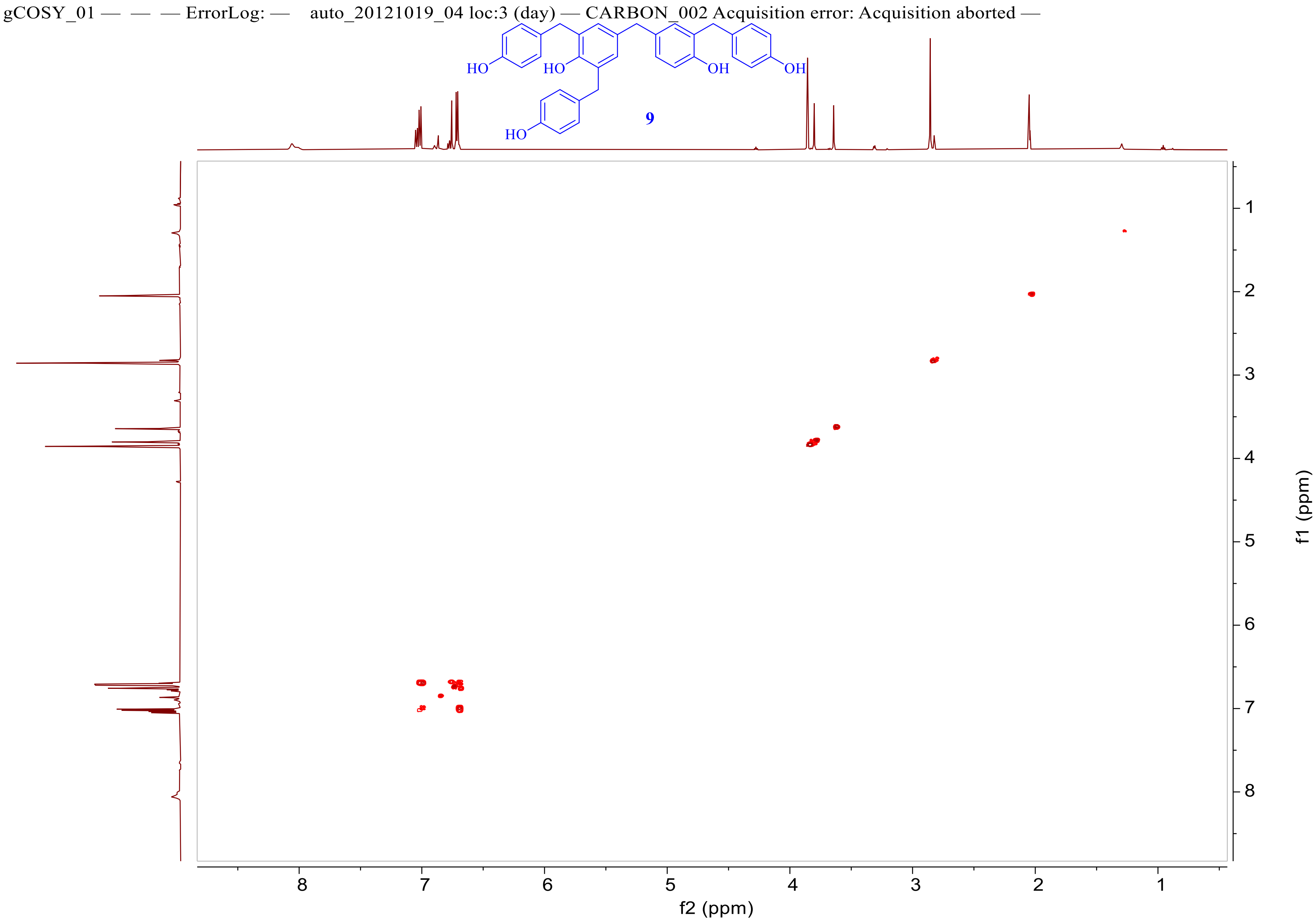

Figure S120. The ^{1}H-^{1}H COSY spectrum of compound **9** in acetone-d_6 (600 MHz)

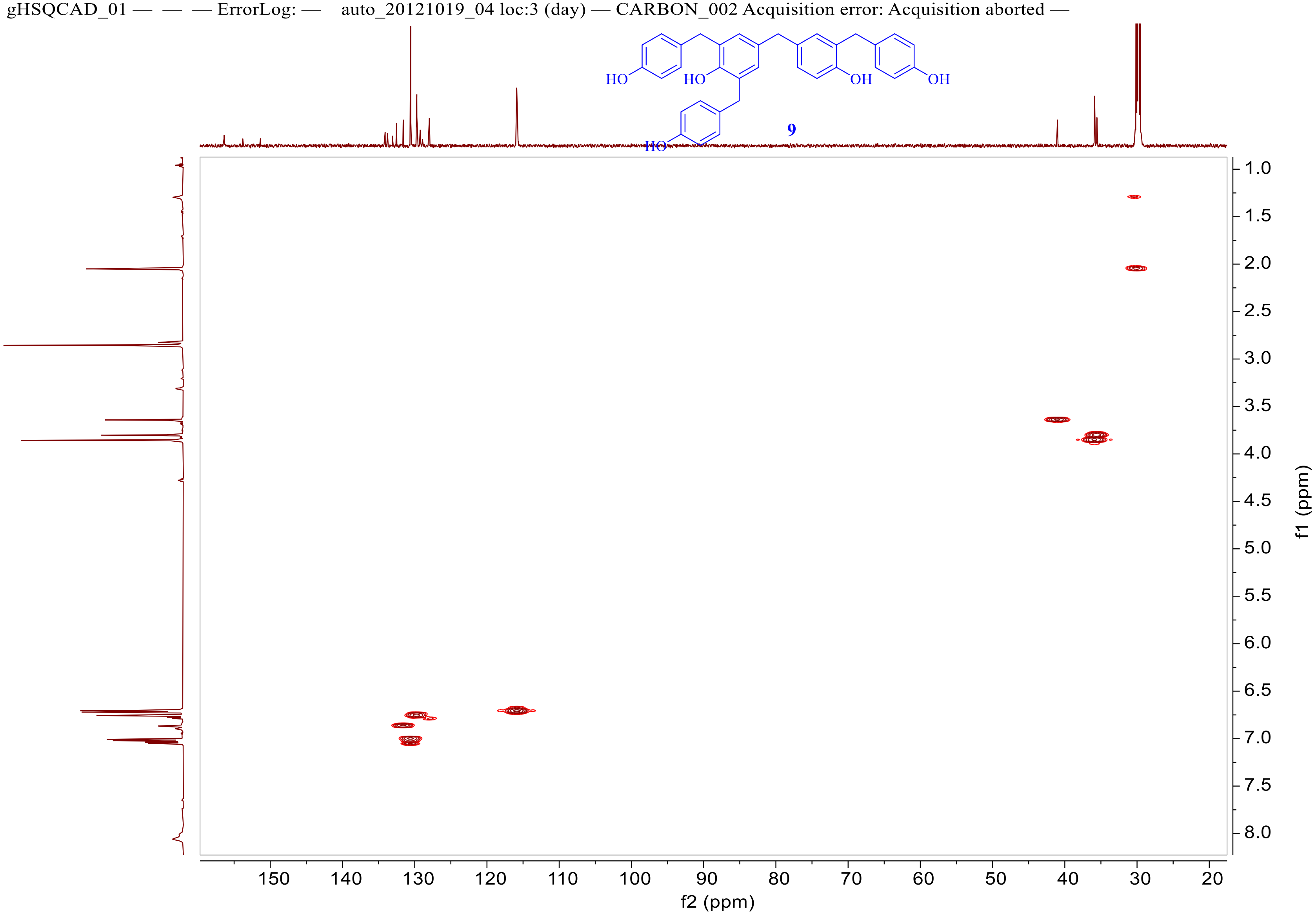

Figure S121. The HSQC spectrum of compound **9** in acetone-d_6 (600 MHz for ^{1}H)

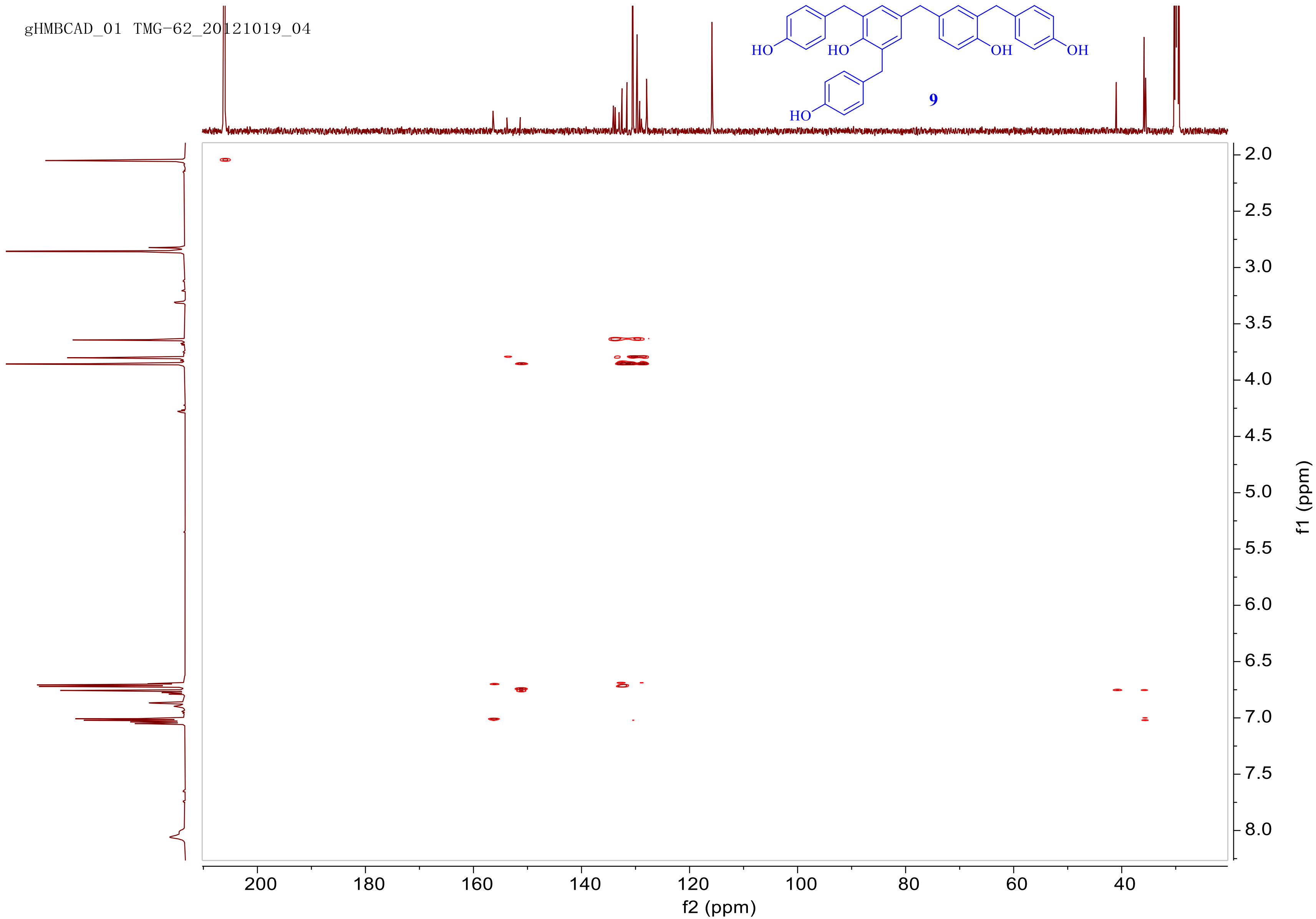

Figure S122. The HMBC spectrum of compound **9** in acetone-d_6 (600 MHz for ^1H)

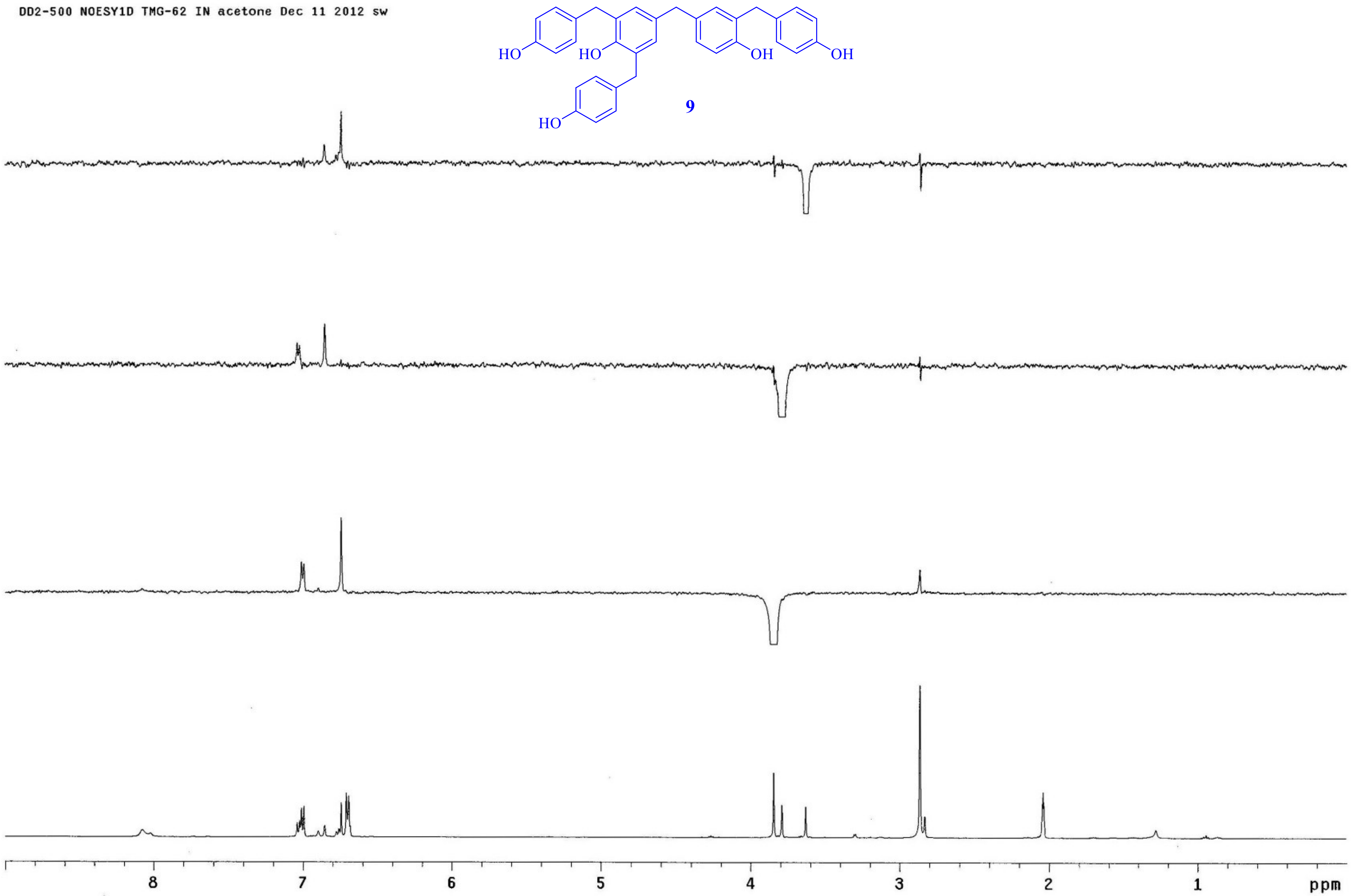

Figure S123. The NOESY1D spectrum of compound **9** in acetone-d_6 (500 MHz)

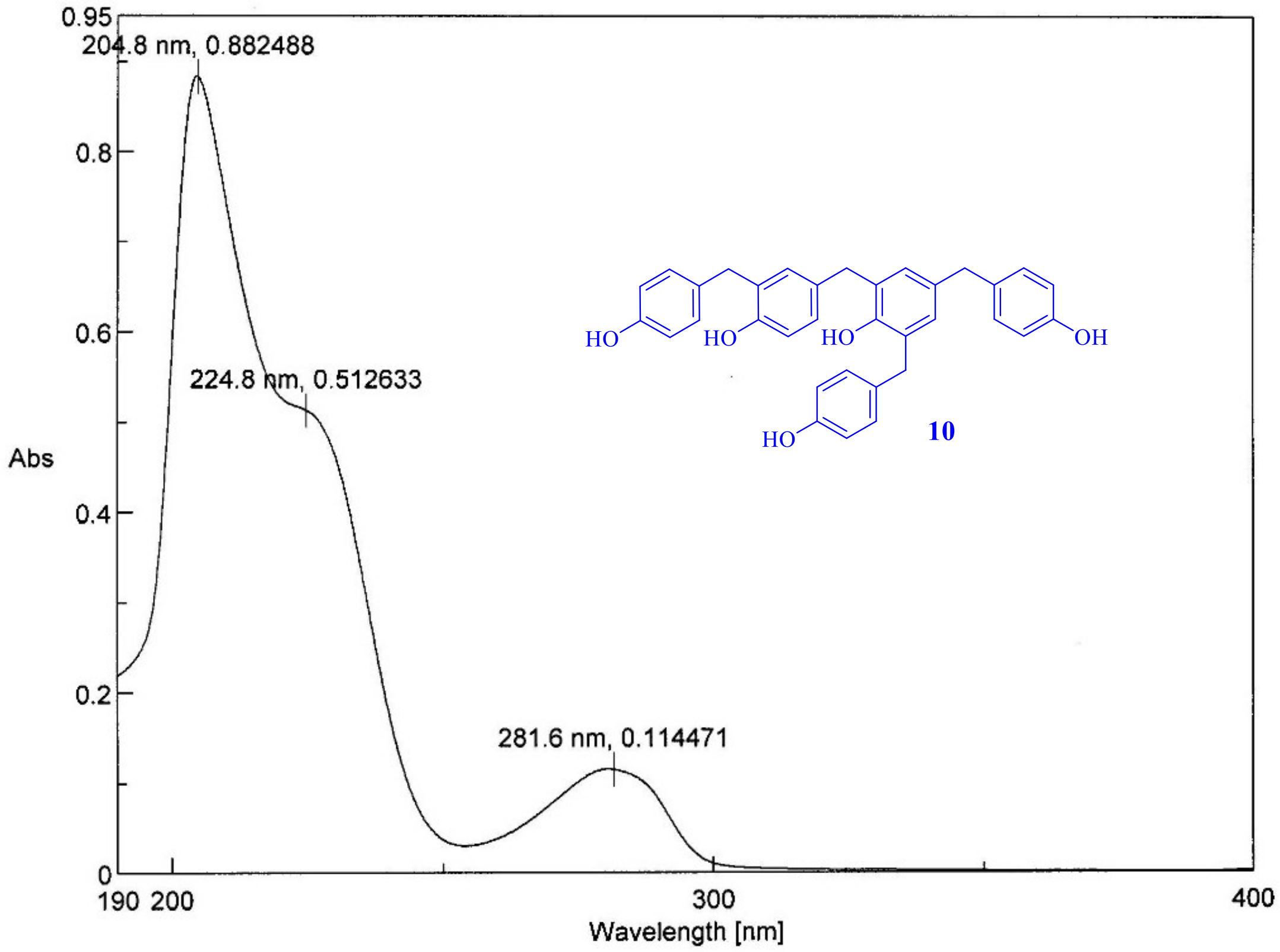

TMG-38

[Comment]
Sample Name TMG-38
Comment 0.02
User
Division UV
Company 324
[Measurement Information]
Instrument Name V-650
Model Name V-650
Serial No. A034461150

Accessory PSC-718
Accessory S/N A001761114
Position 1
Cell Length 10 mm
Temperature 20.01 C
Control Sonsor Holder
Monitor Sensor Holder
Start Mode Start immediately

Photometric Mode Abs
Measurement range 400 - 190 nm
Data pitch 0.2 nm
Band width(UV/Vis) 2.0 nm
Response Medium
Scanning speed 200 nm/min
Source Change 340 nm
Light Source D2/WI
Filter Exchange Step
Correction Baseline

[Data Information]
Creation Date 2014-2-26 9:48

Data array type Linear data array
Horizontal Wavelength [nm]
Vertical Abs
Start 400 nm
End 190 nm
Data pitch 0.2 nm
Data points 1051

Figure S124. The UV spectrum of compound **10**

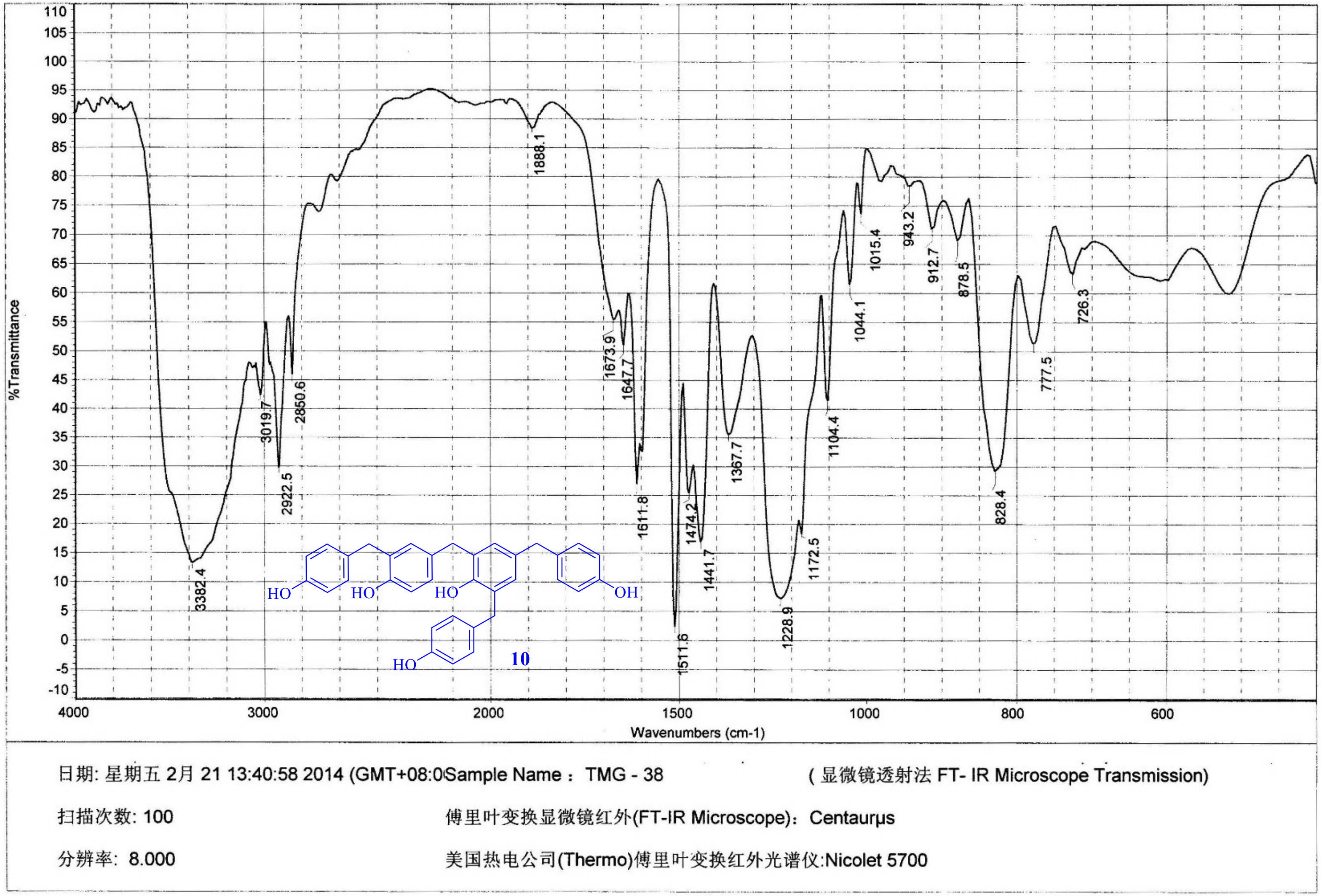

Figure S125. The IR spectrum of compound **10**

Single Mass Spectrum Deconvolution Report

**Analysis Name:**	guoql075.d	**Instrument:**	LC-MSD-Trap-SL	**Print Date:**	10/30/2012 1:53:18 PM
**Method:**	TEST.MS	**Operator:**	Operator	**Acq. Date:**	10/30/2012 1:07:14 PM
**Sample Name:**	TMG-61				
**Analysis Info:**					

Acquisition Parameter:

Mass Range Mode	Std/Normal	Trap Drive	29.0	Scan Begin	100 m/z
Ion Polarity	Positive	Octopole RF Amplitude	152.8 Vpp	Scan End	700 m/z
Ion Source Type	ESI	Capillary Exit	102.3 Volt	Averages	5 Spectra
Dry Temp (Set)	330 °C	Skimmer	40.0 Volt	Max. Accu Time	200000 µs
Nebulizer (Set)	15.00 psi	Oct 1 DC	12.00 Volt	ICC Target	20000
Dry Gas (Set)	6.00 l/min	Oct 2 DC	1.70 Volt	Charge Control	on

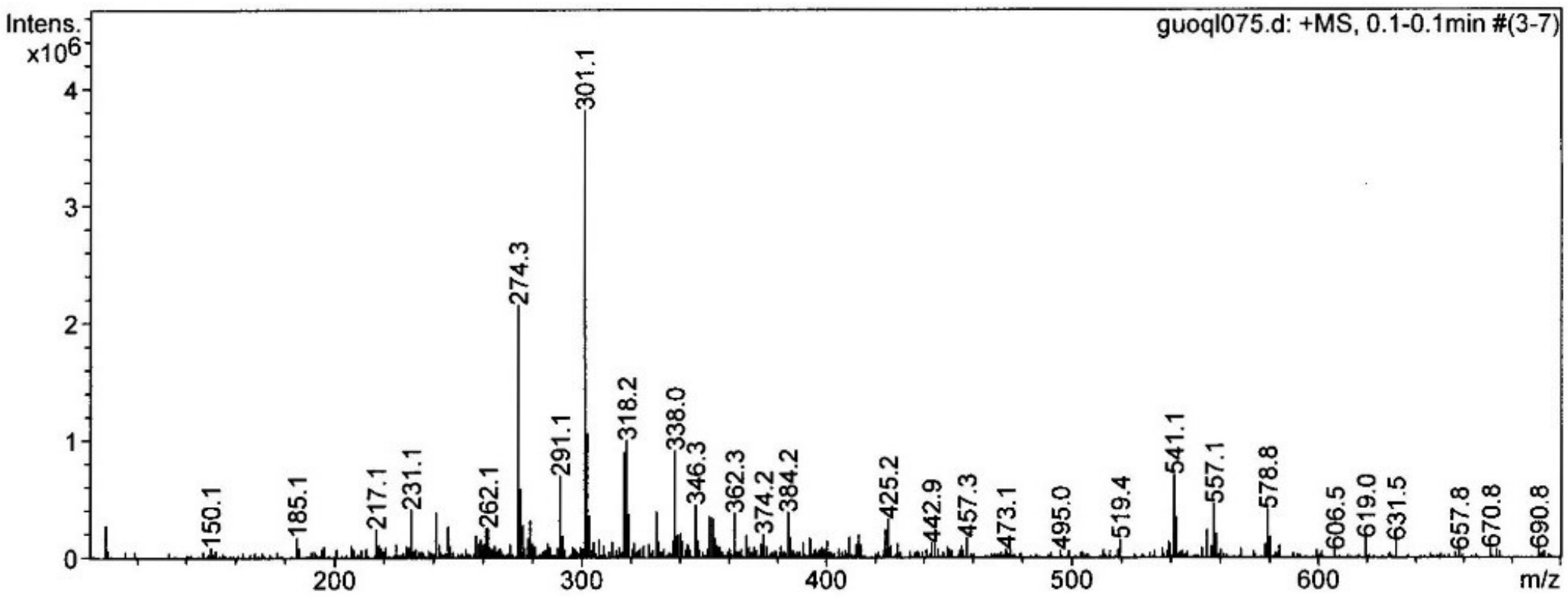

Component	Molecular Mass	Molecule	Absolute Abundance	Relative Abundance

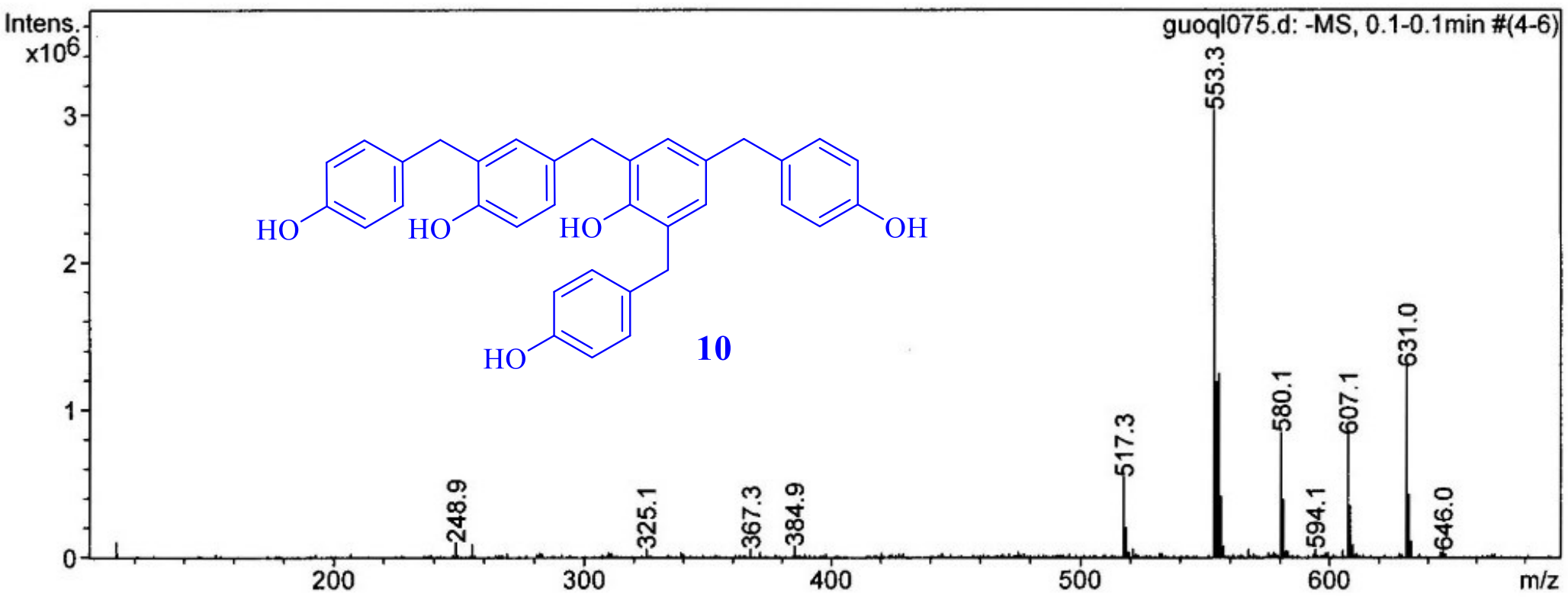

Component	Molecular Mass	Molecule	Absolute Abundance	Relative Abundance

MSD Trap Report v 4 (A4-Opt2) Page 1 of 1 Agilent Technologies

Figure S126. The ESIMS of compound **10**

Qualitative Analysis Report

**Data Filename**	2014022604.d	**Sample Name**	TMG-38
**Sample Type**	Sample	**Position**	P1-C4
**Instrument Name**	Instrument 1	**User Name**	
**Acq Method**		**IRM Calibration Status**	Success
**DA Method**	TEST LCMS.m	**Comment**	

User Chromatograms

Fragmentor Voltage 135 **Collision Energy** 0 **Ionization Mode** ESI

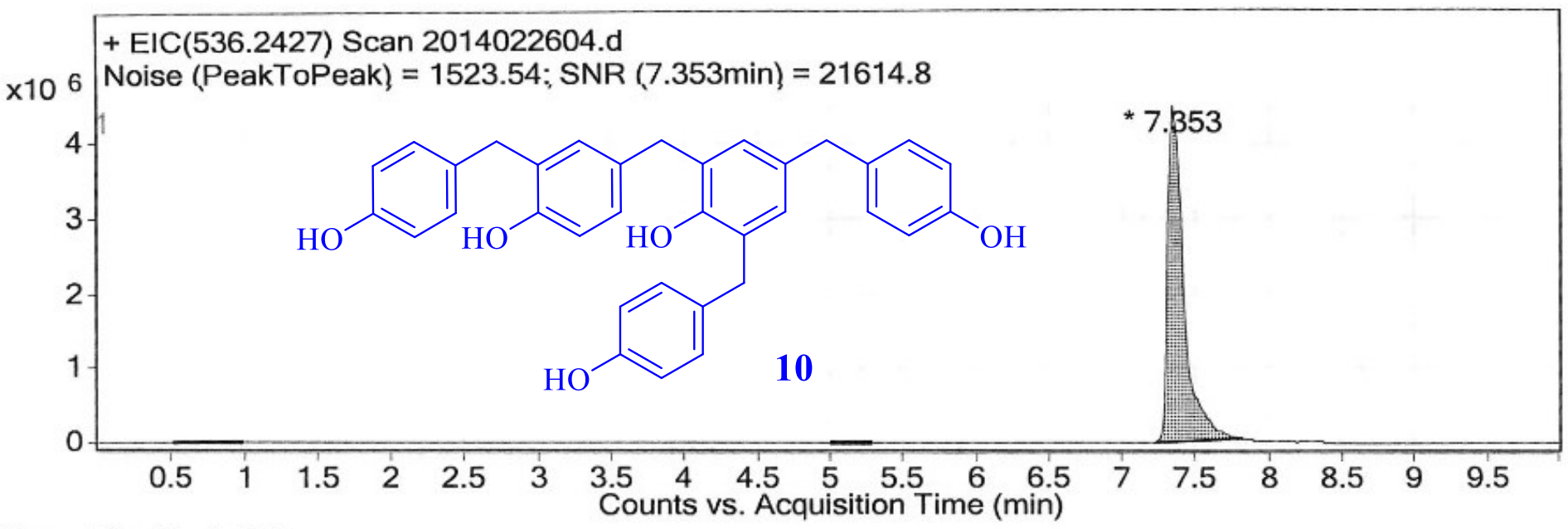

Integration Peak List

Peak	Start	RT	End	Height	Area	Area %	Signal To Noise
1	7.225	7.353	7.82	4491210	32931022	100	21614.8

Noise Measurements

Noise Type	Signal Definition	Noise Multiplier	Noise Value
Peak-to-Peak	Area	1	1523.542969

Noise Regions

Start	End
0.5	1
5	5.3
9.99	11

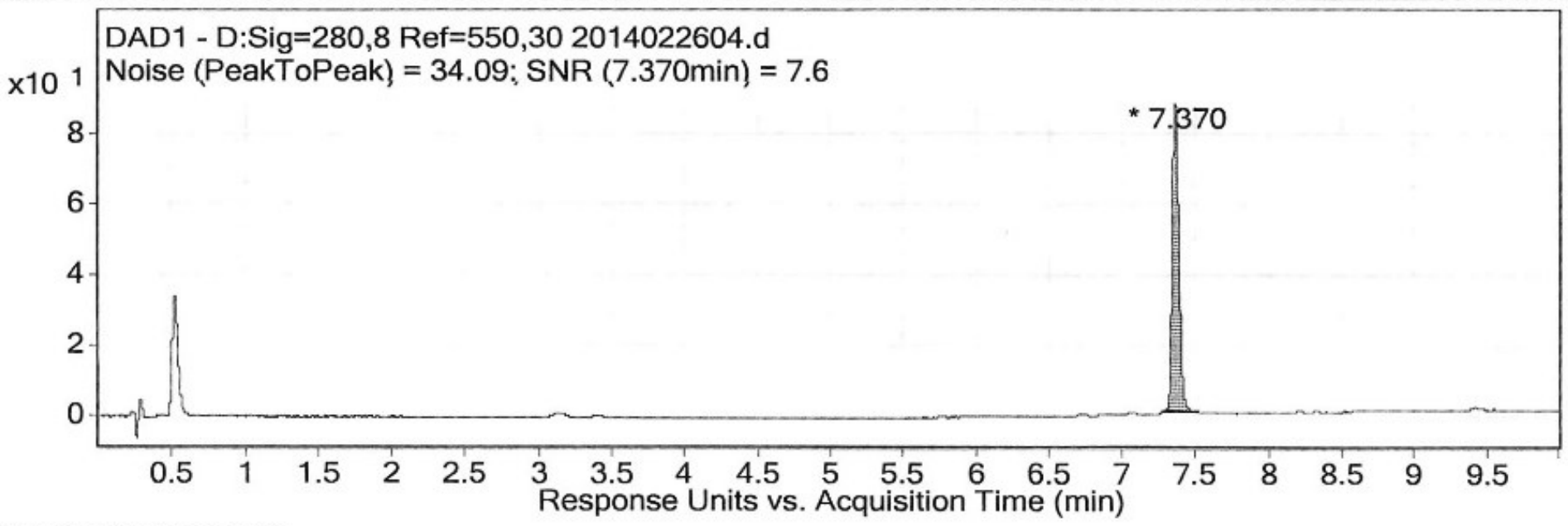

Integration Peak List

Peak	Start	RT	End	Height	Area	Area %	Signal To Noise
1	7.278	7.37	7.524	87.29	259.57	100	7.6

Noise Measurements

Noise Type	Signal Definition	Noise Multiplier	Noise Value
Peak-to-Peak	Area	1	34.08527374

Noise Regions

Start	End
0.5	1
5	5.3
9.99	11

User Spectra

Agilent Technologies Page 1 of 2 Printed at: 9:15 AM on: 2/26/20

Figure S127. The HR-ESIMS report of compound **10** (page 1)

Qualitative Analysis Report

Fragmentor Voltage	Collision Energy	Ionization Mode
135	0	ESI

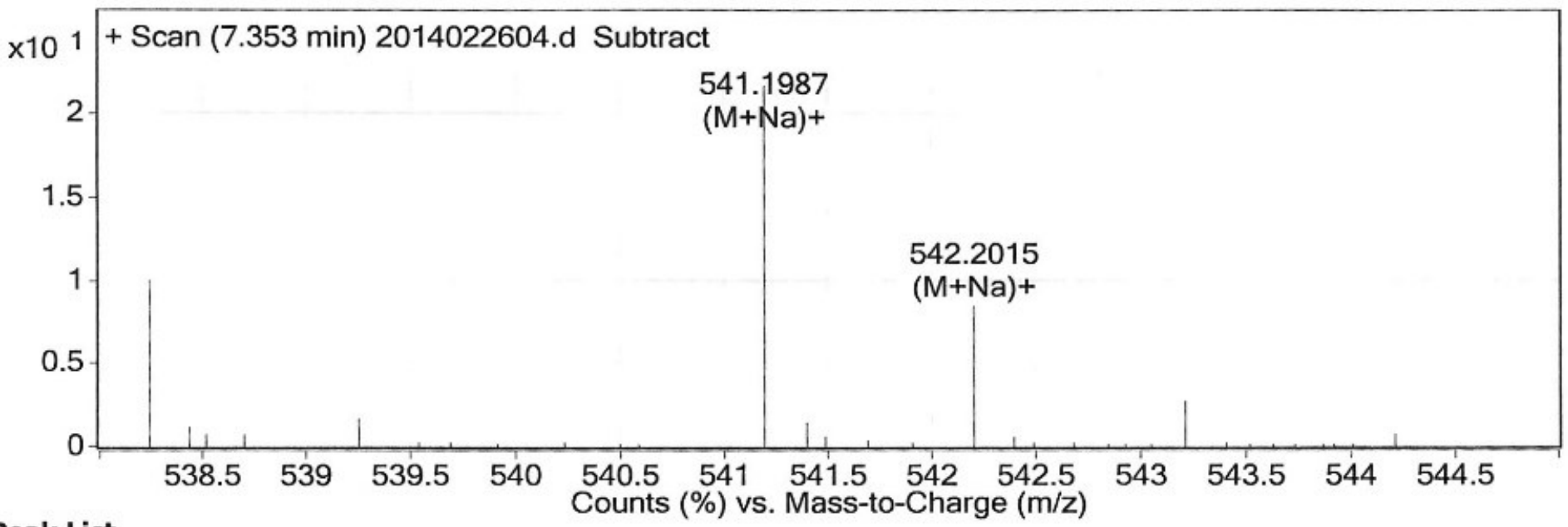

Peak List

m/z	z	Abund	Formula	Ion
425.1744		453831		
536.2427	1	4506314		
536.4322		405515		
537.2453	1	1937097		
538.2491	1	449715		
541.1987	1	976002	C34 H30 Na O5	(M+Na)+
542.2015	1	381896	C34 H30 Na O5	(M+Na)+

Formula Calculator Element Limits

Element	Min	Max
C	3	100
H	0	500
O	0	90
N	0	5
S	0	5
Cl	0	2
Br	0	0
Si	0	0
F	0	0
P	0	0

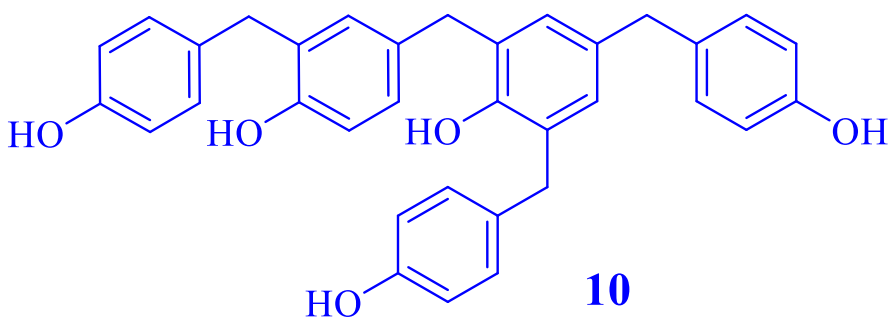

10

Formula Calculator Results

Formula	Best	Mass	Tgt Mass	Diff (ppm)	Ion Species	Score
C34 H30 O5	TRUE	518.2094	518.2093	-0.18	C34 H30 Na O5	99.14
C38 H30 S		518.2094	518.2068	-5.03	C38 H30 Na S	98.88
C35 H26 N4 O		518.2094	518.2107	2.38	C35 H26 N4 Na O	98.85
C35 H34 S2		518.2094	518.2102	1.46	C35 H34 Na S2	98.6
C26 H34 N2 O7 S		518.2094	518.2087	-1.48	C26 H34 N2 Na O7 S	97.94

--- End Of Report ---

Figure S128. The HR-ESIMS report of compound **10** (page 2)

MS Formula Results: + Scan (7.353 min) Sub (2014022604.d)

m/z	Ion	Formula	Abundance
541.1987	(M+Na)+	C34 H30 Na O5	976001.5

Best	Formula (M)	Ion Formula	Calc m/z	Score	Cross S	Mass	Calc Mass	Diff (ppm)	Abs Diff (ppm)	Abund Match	Spacing Mat	Mass Match	m/z	DBE
☑	C34 H30 O5	C34 H30 Na O5	541.1985	99.14		518.2094	518.2093	-0.18	0.18	97.61	99.25	100	541.1987	20
☐	C38 H30 S	C38 H30 Na S	541.196	98.88		518.2094	518.2068	-5.03	5.03	99.44	97.56	99.2	541.1987	24
☐	C35 H26 N4 O	C35 H26 N4 Na O	541.1999	98.85		518.2094	518.2107	2.38	2.38	96.96	99.19	99.82	541.1987	25
☐	C35 H34 S2	C35 H34 Na S2	541.1994	98.6		518.2094	518.2102	1.46	1.46	98.53	96.04	99.93	541.1987	19
☐	C26 H34 N2 O7 S	C26 H34 N2 Na O7 S	541.1979	97.94		518.2094	518.2087	-1.48	1.48	95.75	96.59	99.93	541.1987	11

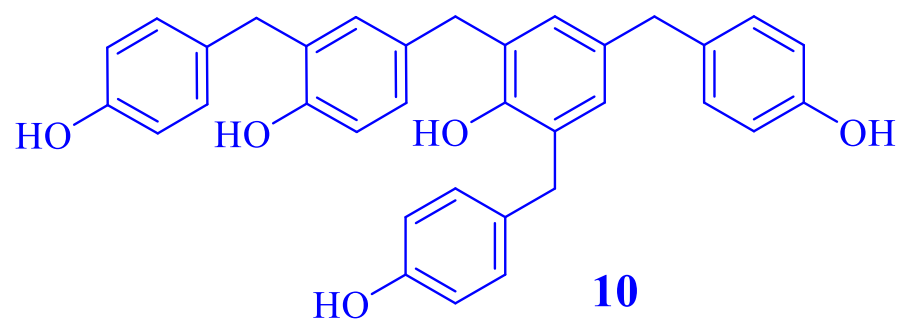

10

page 1

Figure S129. The HR-ESIMS report of compound **10** (page 3)

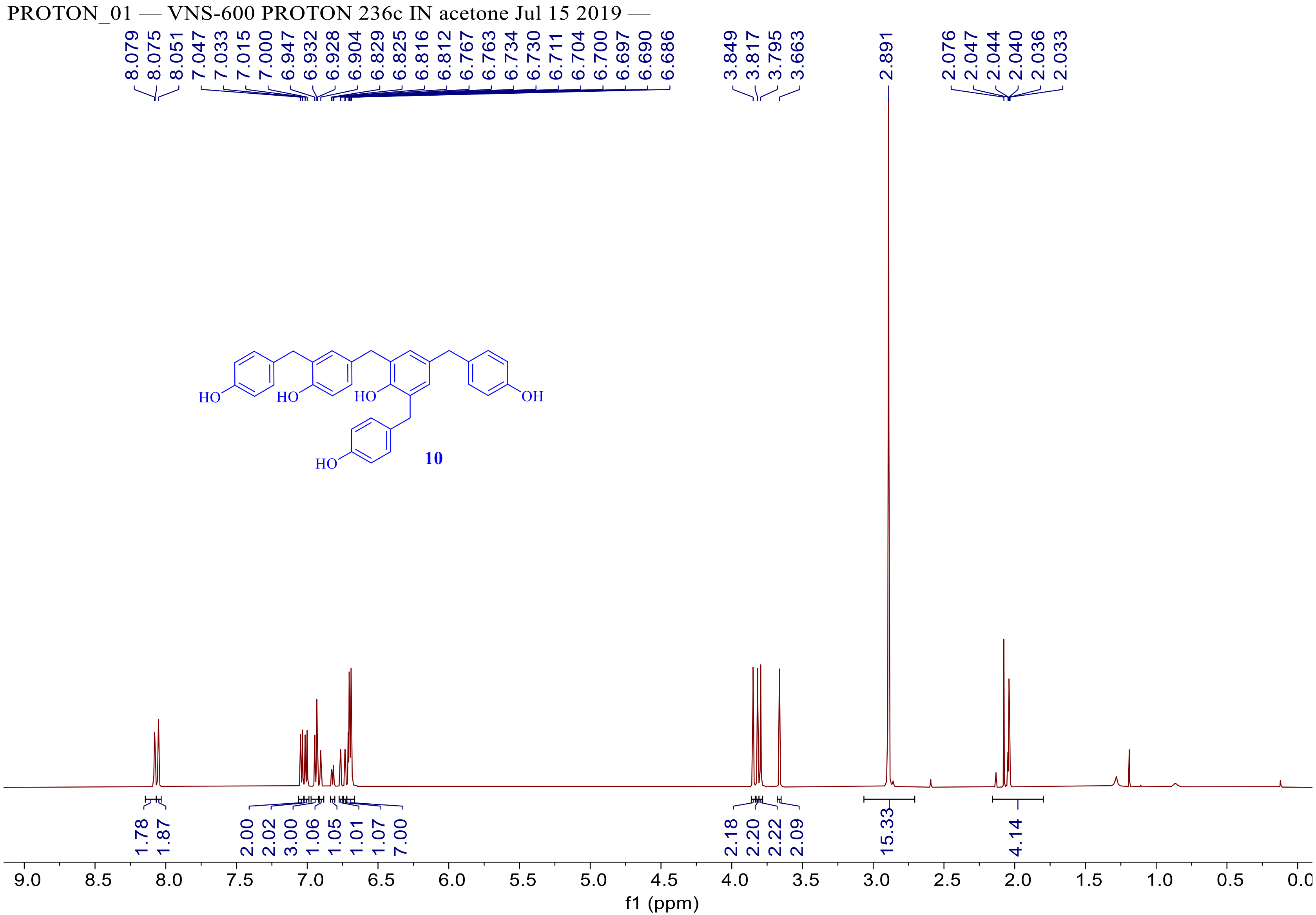

Figure S130. The ^{1}H NMR spectrum of compound **10** in acetone-d_6 (600 MHz)

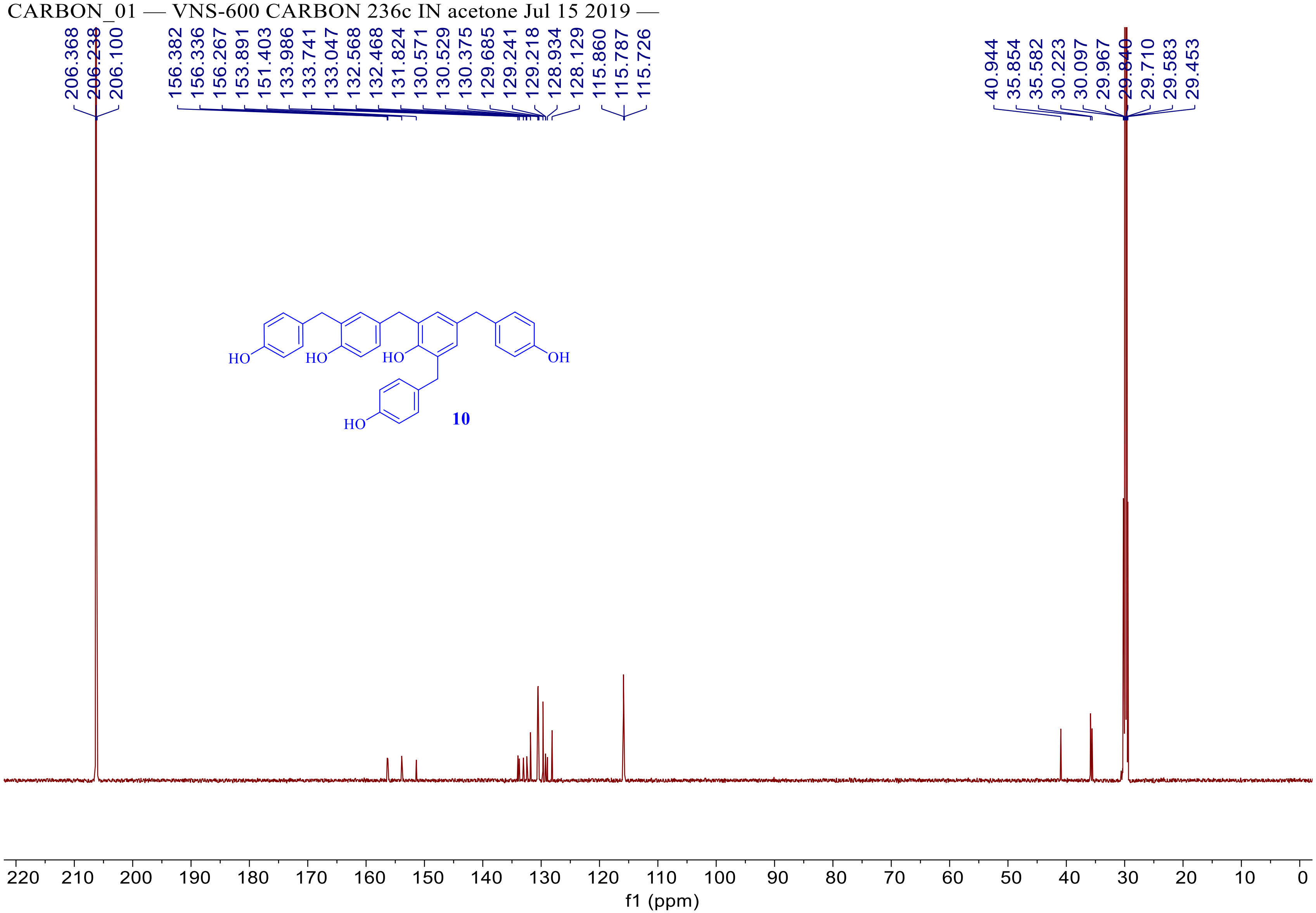

Figure S131. The ^{13}C NMR spectrum of compound **10** in acetone-d_6 (150 MHz)

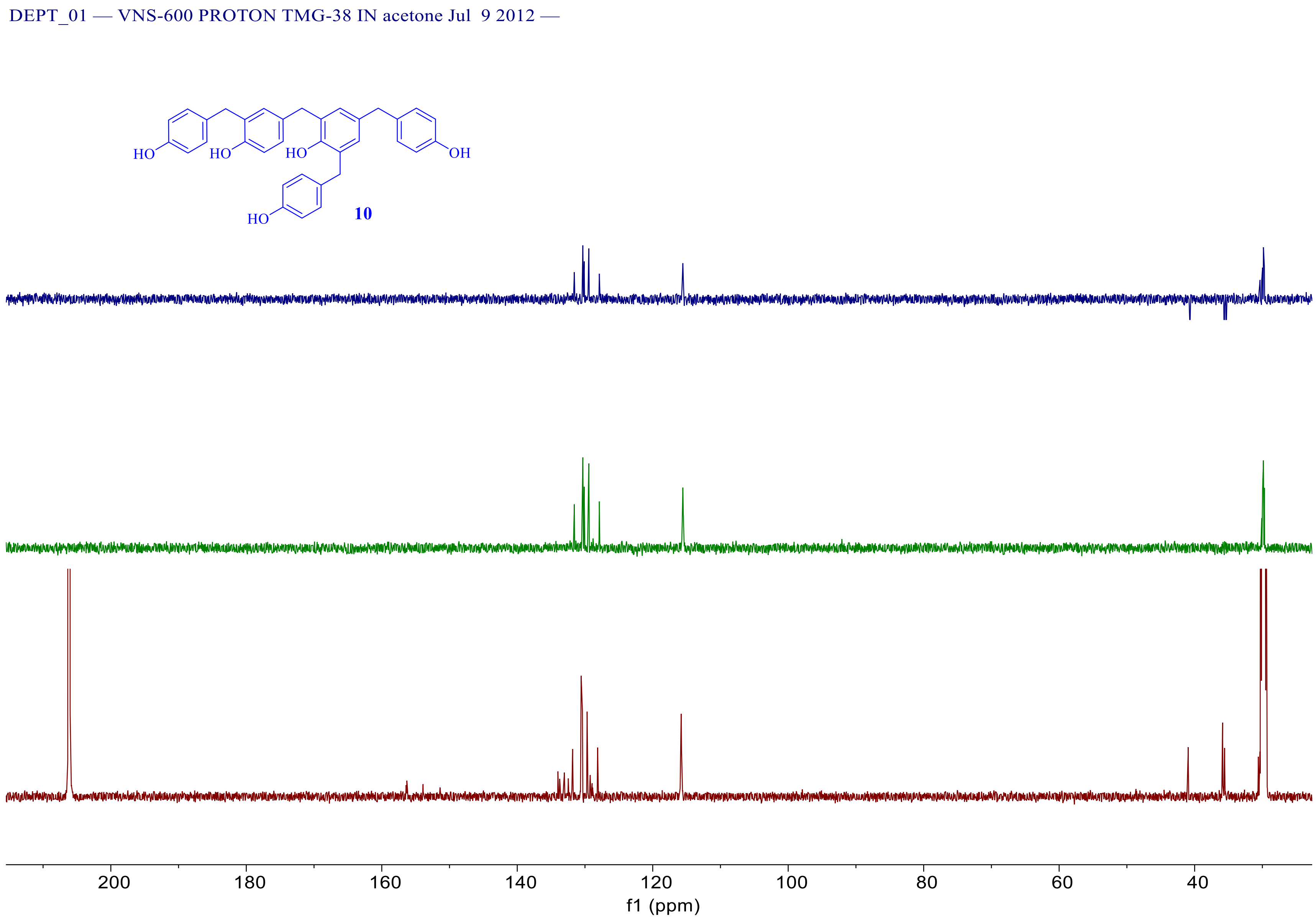

Figure S132. The DEPT spectrum of compound **10** in acetone-d_6 (150 MHz)

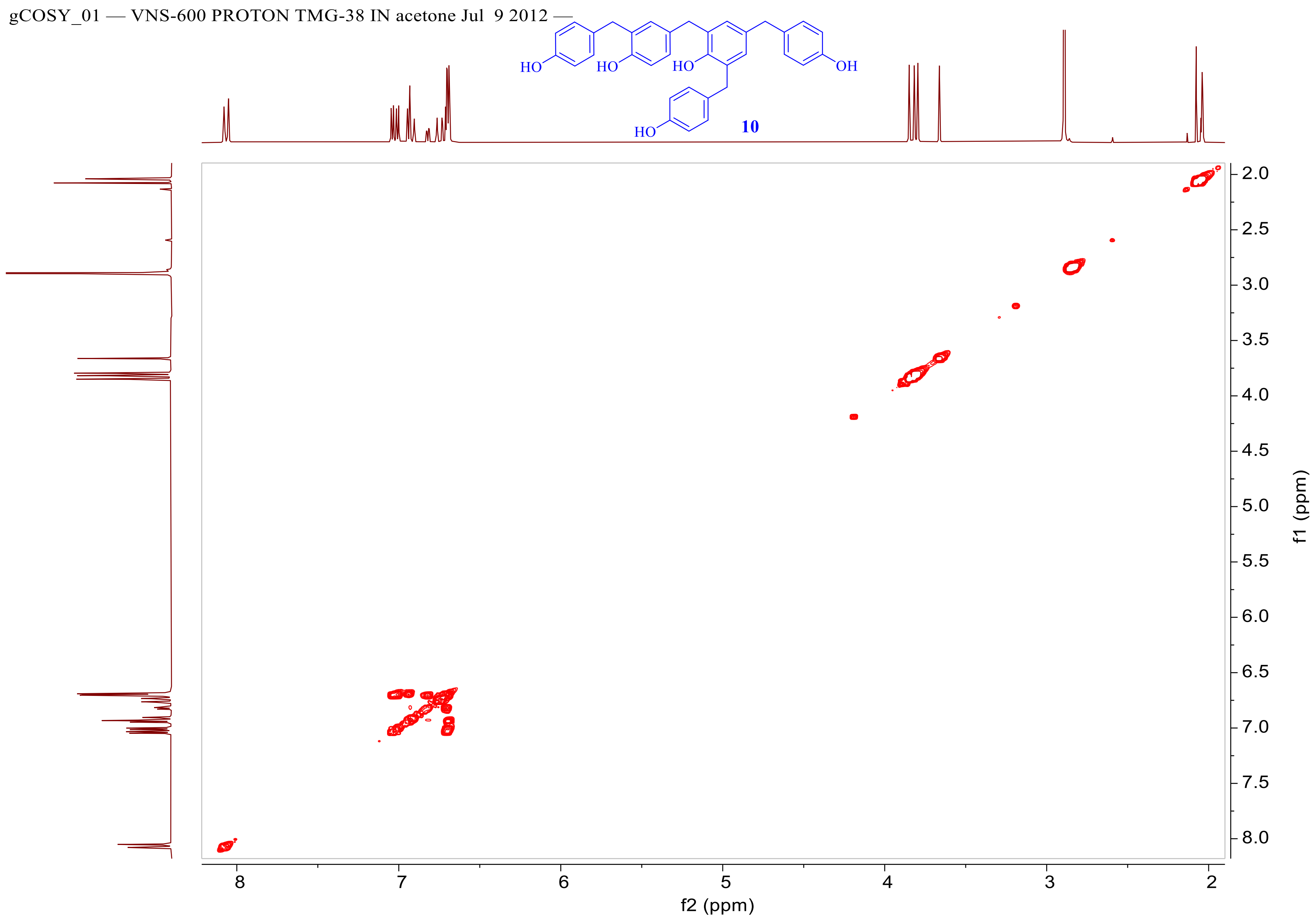

Figure S133. The ^{1}H-^{1}H COSY spectrum of compound **10** in acetone-d_6 (600 MHz)

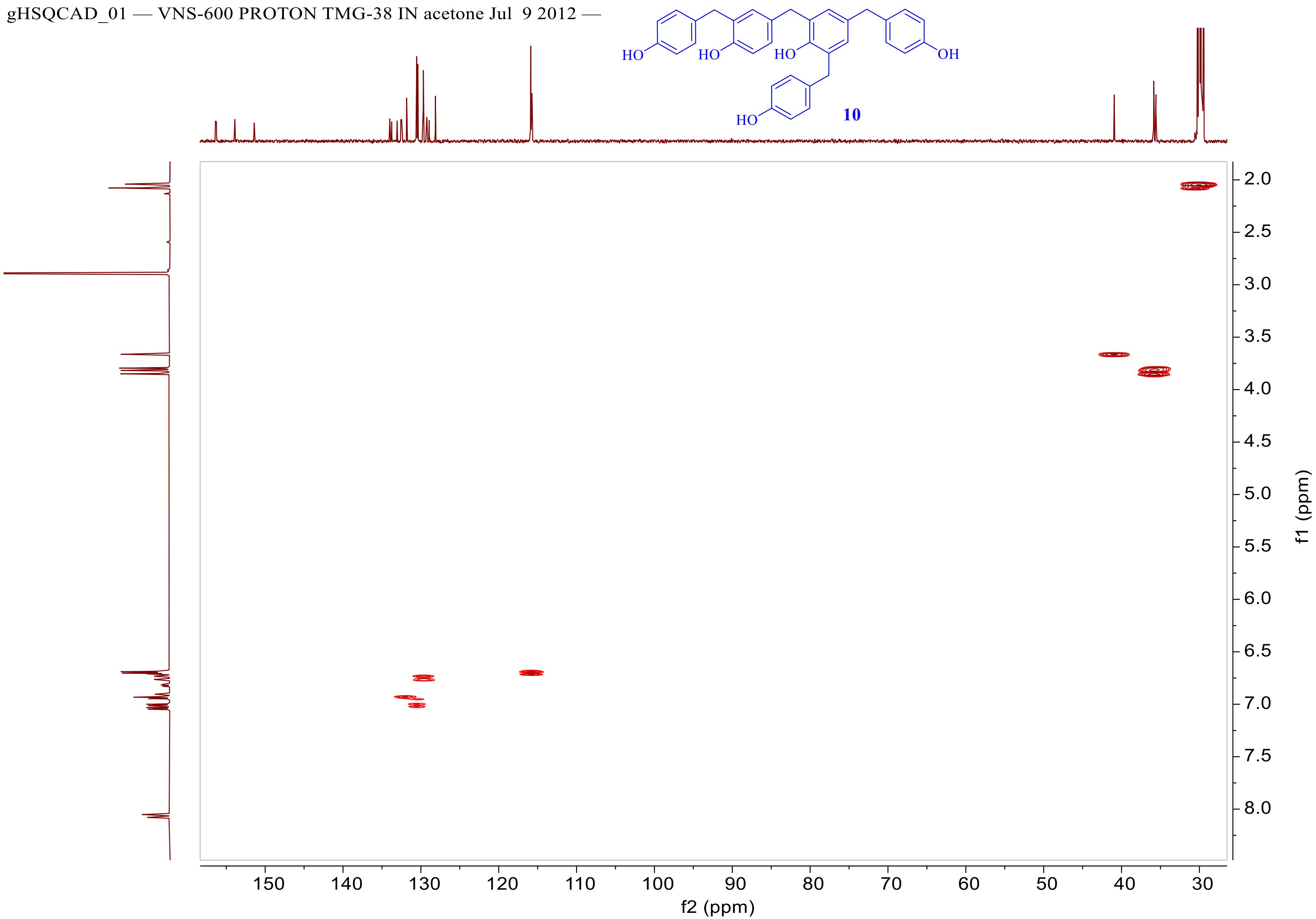

Figure S134. The HSQC spectrum of compound **10** in acetone-d_6 (600 MHz for ^{1}H)

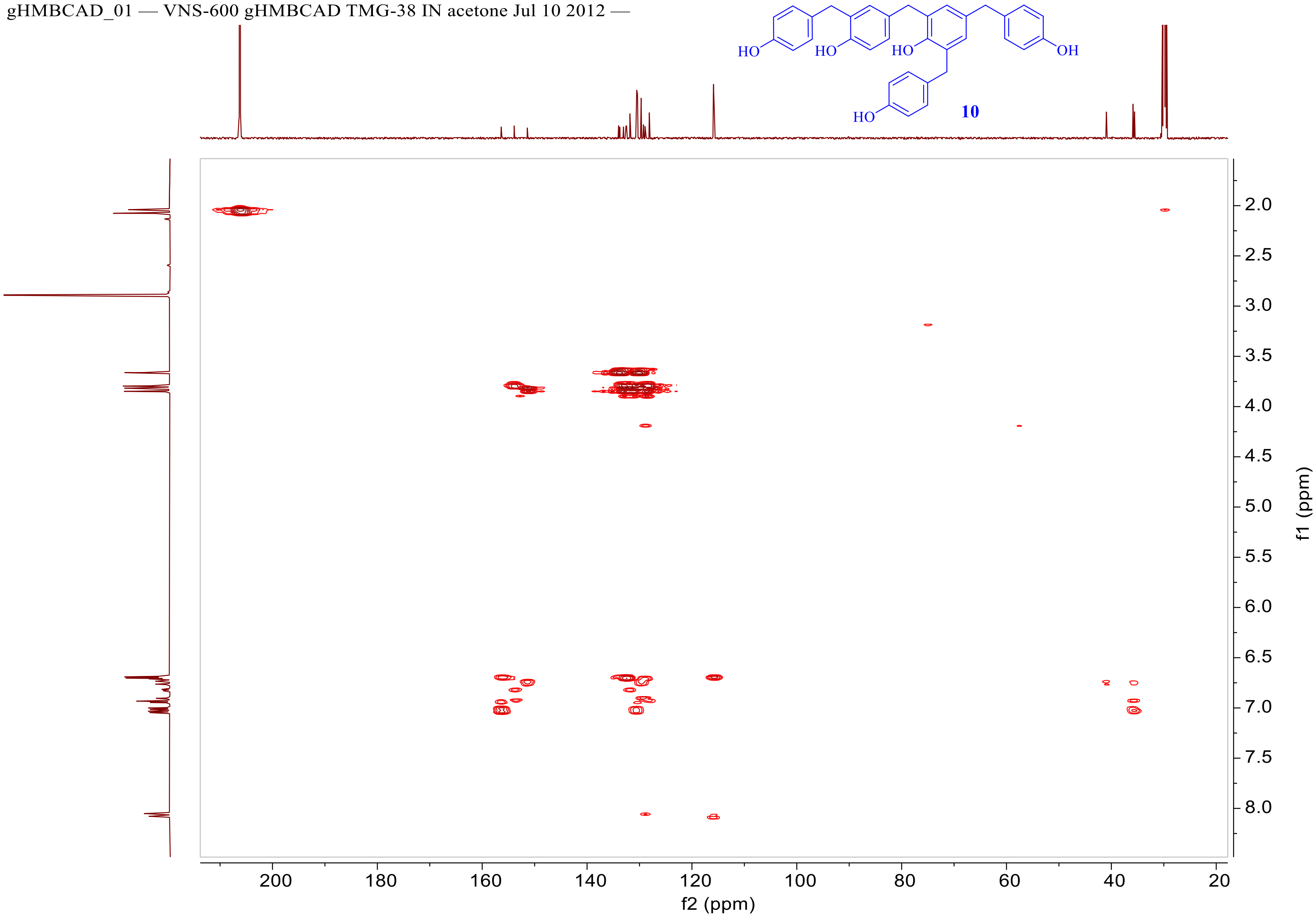

Figure S135. The HMBC spectrum of compound **10** in acetone-d_6 (600 MHz for ^1H)

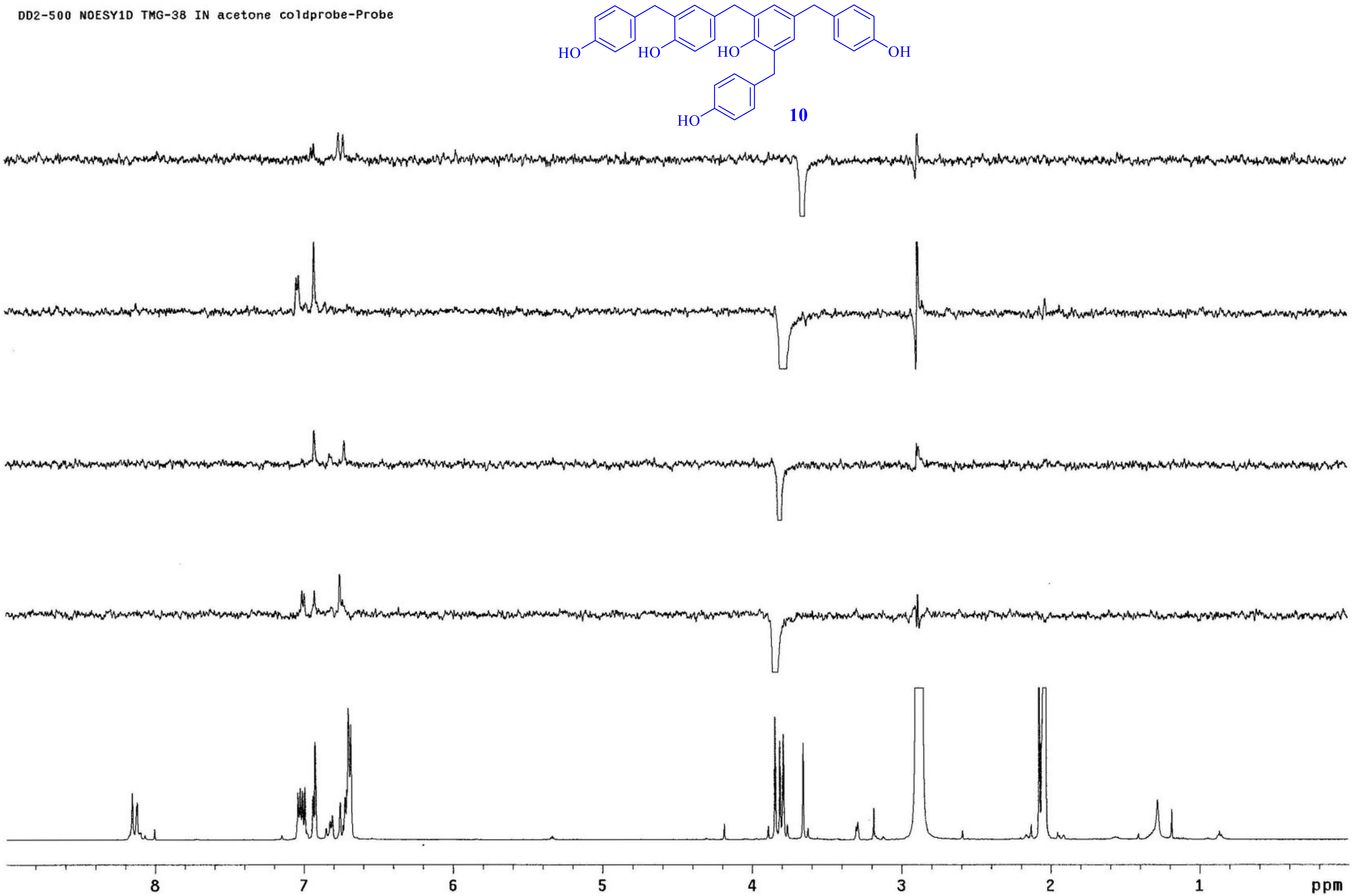

Figure S136. The NOESY 1D spectrum of compound **10** in acetone-d_6 (600 MHz)

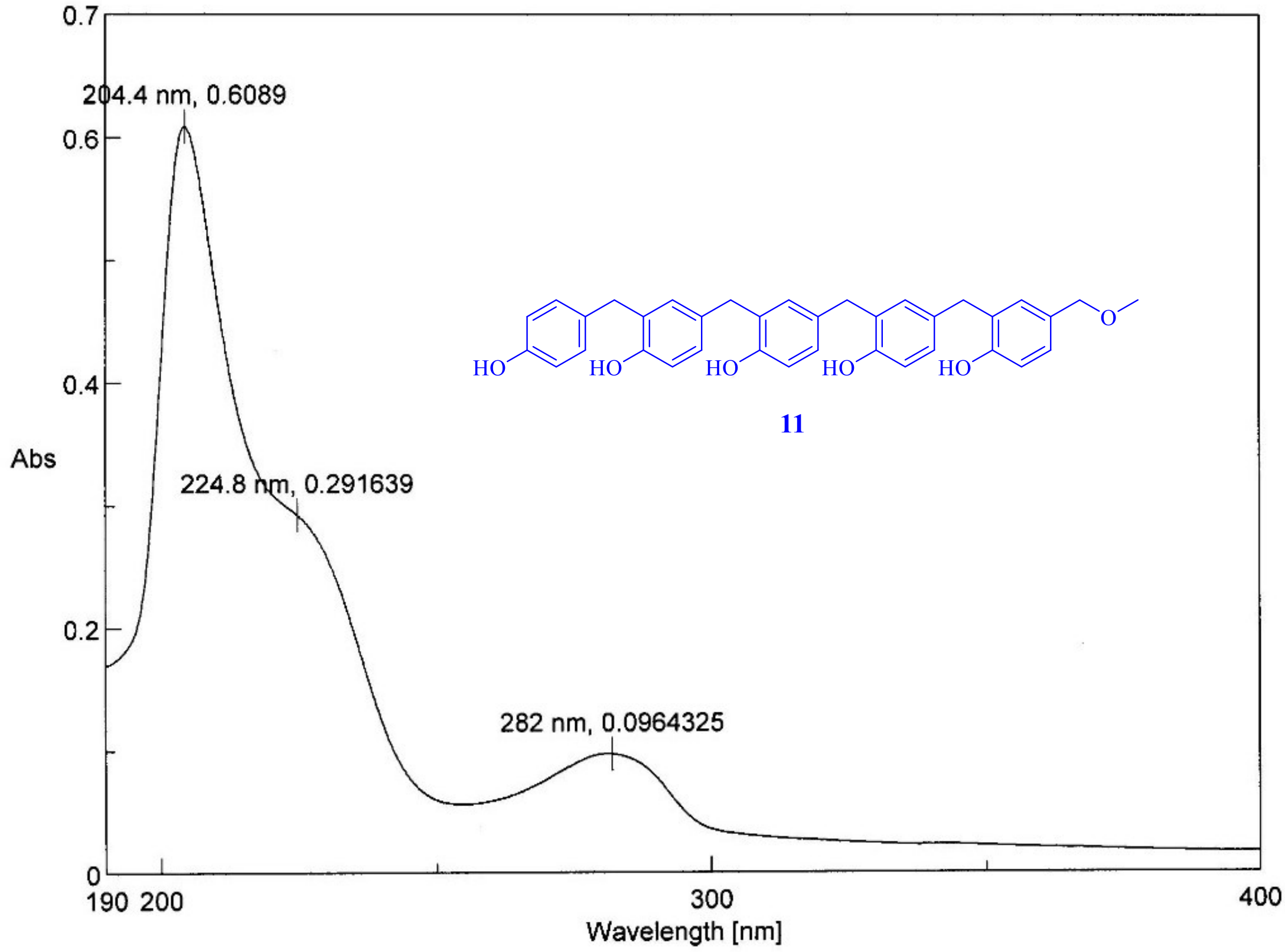

Memory-21

[Comment]
Sample Name TMG-46
Comment 0.02
User
Division UV
Company 324

[Measurement Information]
Instrument Name V-650
Model Name V-650
Serial No. A034461150

Accessory PSC-718
Accessory S/N A001761114
Position 1
Cell Length 10 mm
Temperature 19.97 C
Control Sonsor Holder
Monitor Sensor Holder
Start Mode Start immediately

Photometric Mode Abs
Measurement range 400 - 190 nm
Data pitch 0.2 nm
Band width(UV/Vis) 2.0 nm
Response Medium
Scanning speed 200 nm/min
Source Change 340 nm
Light Source D2/WI
Filter Exchange Step
Correction Baseline

[Data Information]
Creation Date 2014-2-26 9:51

Data array type Linear data array
Horizontal Wavelength [nm]
Vertical Abs
Start 400 nm
End 190 nm
Data pitch 0.2 nm
Data points 1051

Figure S137. The UV spectrum of compound **11** in MeOH

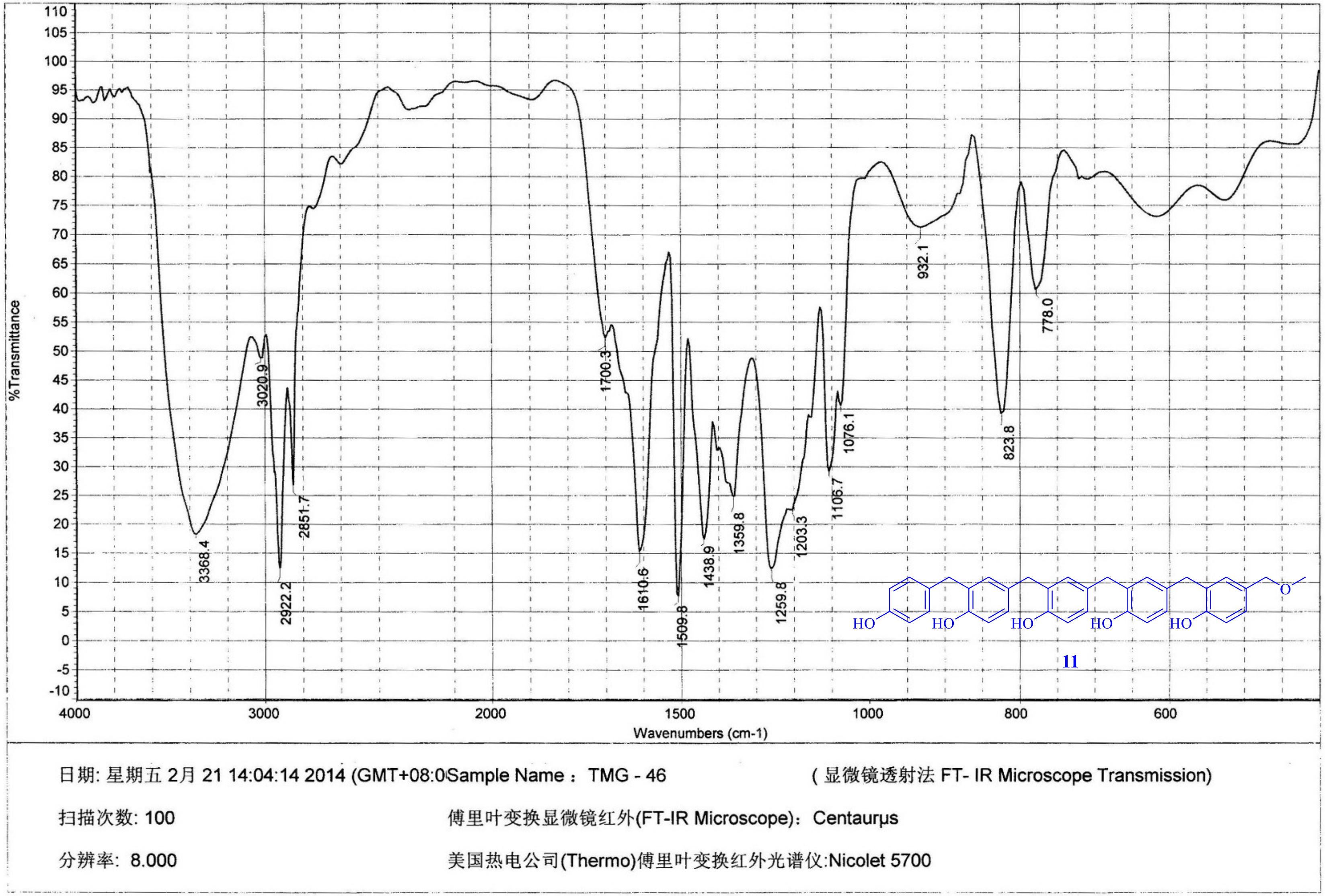

Figure S138. The IR spectrum of compound **11**

Single Mass Spectrum Deconvolution Report

Analysis Name: guoql059.d
Method: TEST.MS
Sample Name: TMG-46
Analysis Info:

Instrument: LC-MSD-Trap-SL
Operator: Operator

Print Date: 7/3/2012 11:37:52 AM
Acq. Date: 7/3/2012 11:26:30 AM

Acquisition Parameter:

Mass Range Mode	Std/Normal	Trap Drive	45.5	Scan Begin	100 m/z
Ion Polarity	Positive	Octopole RF Amplitude	152.8 Vpp	Scan End	800 m/z
Ion Source Type	ESI	Capillary Exit	-102.3 Volt	Averages	5 Spectra
Dry Temp (Set)	330 °C	Skimmer	-40.0 Volt	Max. Accu Time	200000 µs
Nebulizer (Set)	15.00 psi	Oct 1 DC	-12.00 Volt	ICC Target	20000
Dry Gas (Set)	5.00 l/min	Oct 2 DC	-1.70 Volt	Charge Control	on

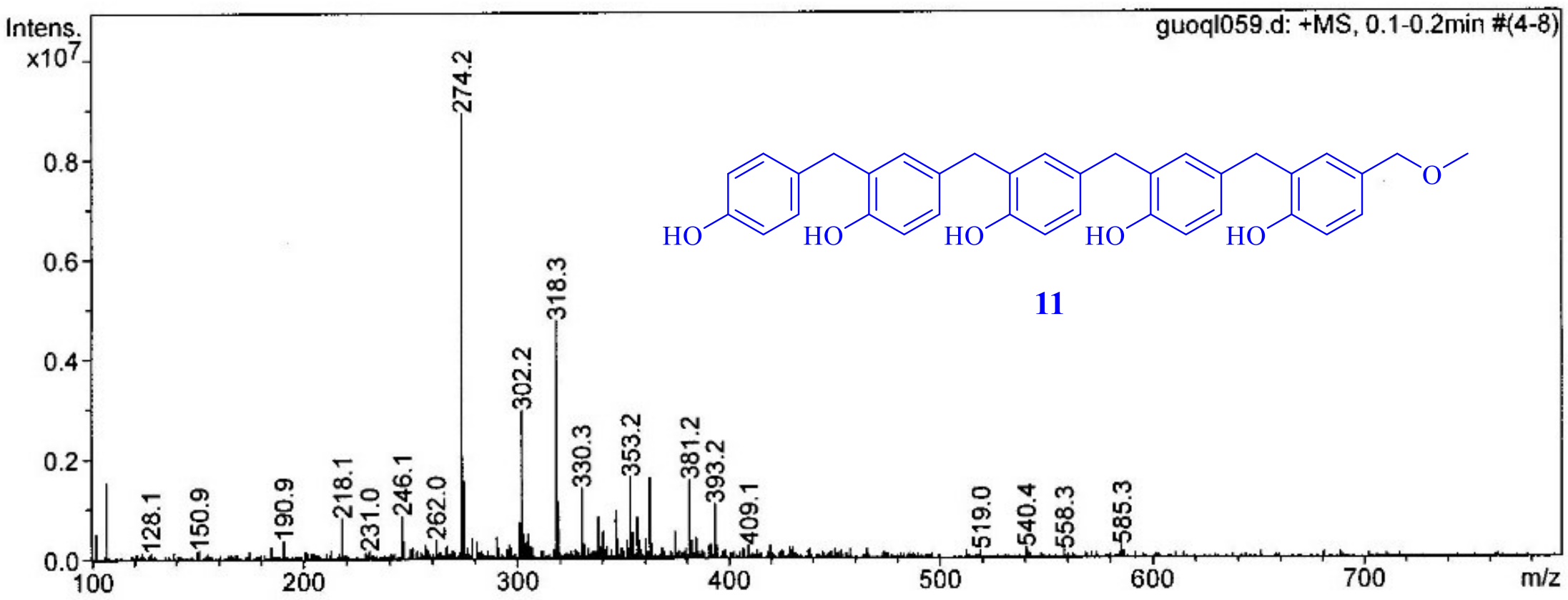

Component	Molecular Mass	Molecule	Absolute Abundance	Relative Abundance

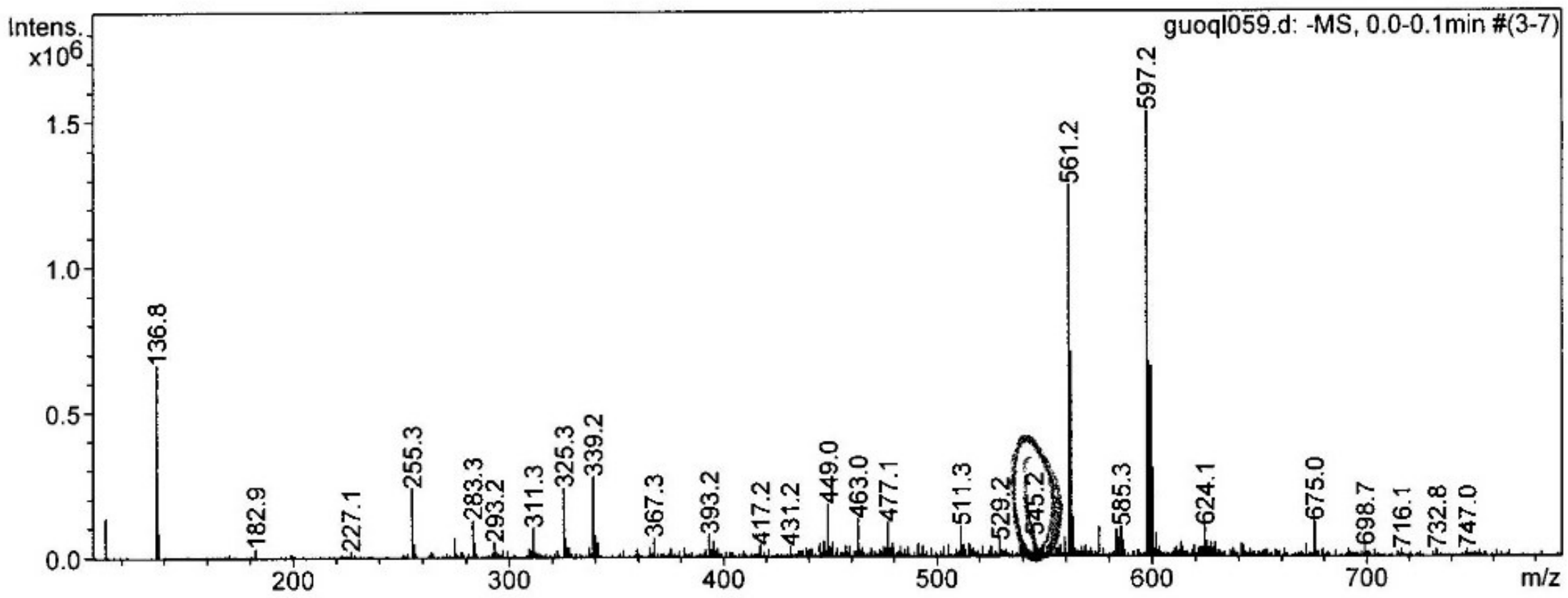

Component	Molecular Mass	Molecule	Absolute Abundance	Relative Abundance

MSD Trap Report v 4 (A4-Opt2) Page 1 of 1 Agilent Technologies

Figure S139. The ESIMS of compound **11**

Qualitative Analysis Report

**Data Filename**	2014022605.d	**Sample Name**	TMG-46
**Sample Type**	Sample	**Position**	P1-C5
**Instrument Name**	Instrument 1	**User Name**	
**Acq Method**		**IRM Calibration Status**	Success
**DA Method**	TEST LCMS.m	**Comment**	

User Chromatograms

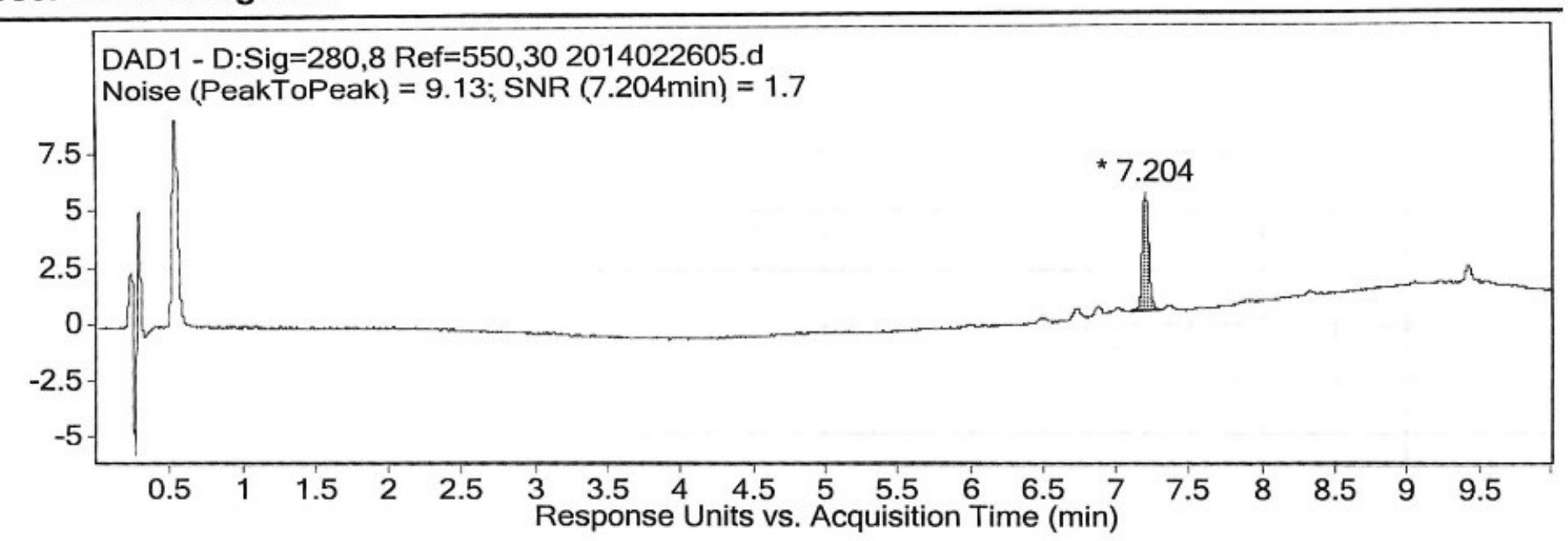

Integration Peak List

Peak	Start	RT	End	Height	Area	Area %	Signal To Noise
1	7.099	7.204	7.316	5.19	15.48	100	1.7

Noise Measurements

Noise Type	Signal Definition	Noise Multiplier	Noise Value
Peak-to-Peak	Area	1	9.134292603

Noise Regions

Start	End
0.5	1
5	5.3
9.99	11

Fragmentor Voltage 135 **Collision Energy** 0 **Ionization Mode** ESI

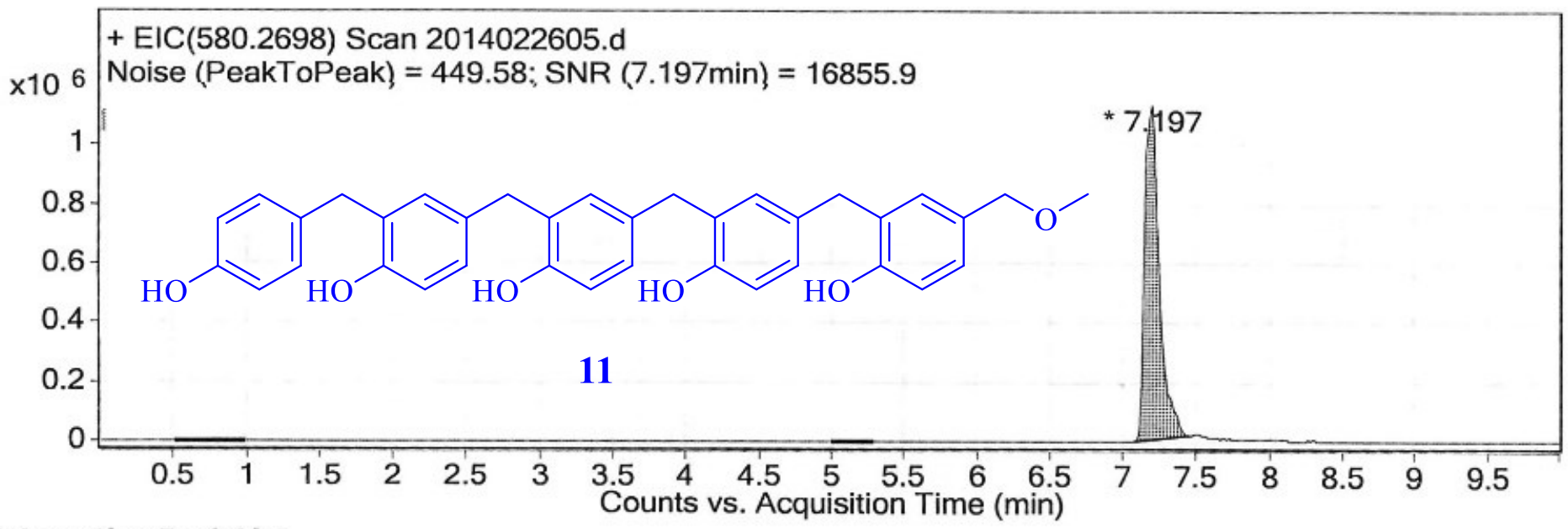

Integration Peak List

Peak	Start	RT	End	Height	Area	Area %	Signal To Noise
1	7.068	7.197	7.455	1125859	7578147	100	16855.9

Noise Measurements

Noise Type	Signal Definition	Noise Multiplier	Noise Value
Peak-to-Peak	Area	1	449.5845947

Noise Regions

Start	End
0.5	1
5	5.3
9.99	11

User Spectra

Figure S140. The HR-ESIMS report of compound **11** (page 1)

Qualitative Analysis Report

Fragmentor Voltage	Collision Energy	Ionization Mode
135	0	Esi

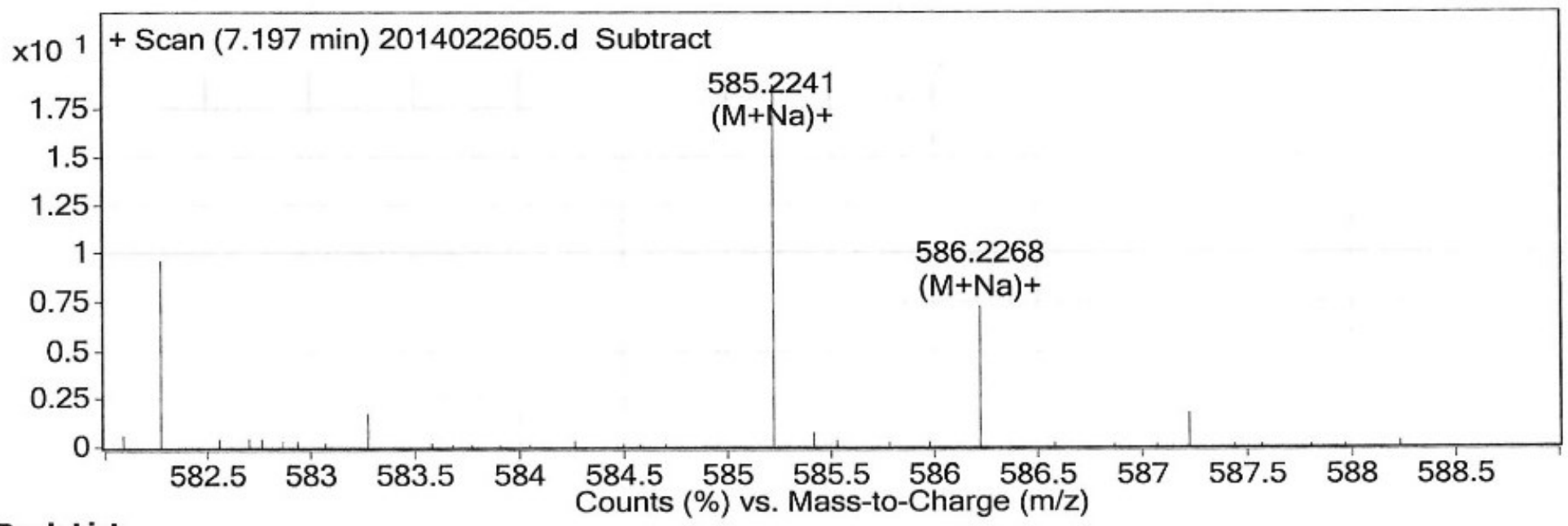

Peak List

m/z	z	Abund	Formula	Ion
240.1947		119719		
580.2693	1	1132983		
581.2727	1	486759		
582.2749	1	108578		
585.2241	1	212237	C36 H34 Na O6	(M+Na)+
586.2268	1	82169	C36 H34 Na O6	(M+Na)+
594.2845	1	221004		
595.2879	1	87263		
601.1973		139968		
922.0096		81045		

Formula Calculator Element Limits

Element	Min	Max
C	3	100
H	0	500
O	0	90
N	0	5
S	0	5
Cl	0	2
Br	0	0
Si	0	0
F	0	0
P	0	0

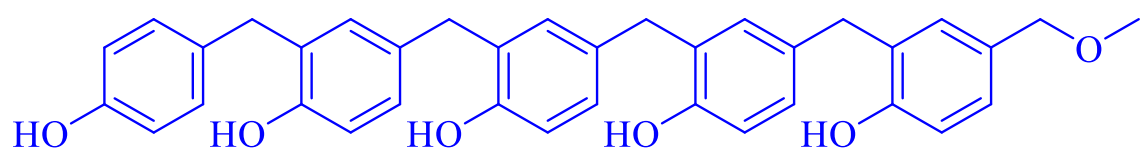

11

Formula Calculator Results

Formula	Best	Mass	Tgt Mass	Diff (ppm)	Ion Species	Score
C36 H34 O6	TRUE	562.2348	562.2355	1.31	C36 H34 Na O6	99.88
C37 H30 N4 O2		562.2348	562.2369	3.66	C37 H30 N4 Na O2	99.53
C31 H34 N2 O8		562.2348	562.2315	-5.86	C31 H34 N2 Na O8	99.18
C40 H34 O S		562.2348	562.233	-3.16	C40 H34 Na O S	99.14
C28 H38 N2 O8 S		562.2348	562.2349	0.12	C28 H38 N2 Na O8 S	98.86
C37 H38 O S2		562.2348	562.2364	2.83	C37 H38 Na O S2	98.14
C32 H38 N2 O3 S2		562.2348	562.2324	-4.34	C32 H38 N2 Na O3 S2	97.91
C24 H38 N2 O13		562.2348	562.2374	4.58	C24 H38 N2 Na O13	97.47

--- End Of Report ---

Figure S141. The HR-ESIMS report of compound **11** (page 2)

MS Formula Results: + Scan (7.197 min) Sub (2014022605.d)

m/z	Ion	Formula	Abundance
585.2241	(M+Na)+	C36 H34 Na O6	212236.7

Best	Formula (M)	Ion Formula	Calc m/z	Score	Cross S	Mass	Calc Mass	Diff (ppm)	Abs Diff (ppm)	Abund Match	Spacing Mat	Mass Match	m/z	DBE
☑	C36 H34 O6	C36 H34 Na O6	585.2248	99.88		562.2348	562.2355	1.31	1.31	99.89	99.73	99.94	585.2241	20
☐	C37 H30 N4 O2	C37 H30 N4 Na O2	585.2261	99.53		562.2348	562.2369	3.66	3.66	99.26	99.79	99.57	585.2241	25
☐	C31 H34 N2 O8	C31 H34 N2 Na O8	585.2207	99.18		562.2348	562.2315	-5.86	5.86	99.14	99.81	98.89	585.2241	16
☐	C40 H34 O S	C40 H34 Na O S	585.2223	99.14		562.2348	562.233	-3.16	3.16	97.56	99.96	99.68	585.2241	24
☐	C28 H38 N2 O8 S	C28 H38 N2 Na O8 S	585.2241	98.86		562.2348	562.2349	0.12	0.12	96.12	99.86	100	585.2241	11
☐	C37 H38 O S2	C37 H38 Na O S2	585.2256	98.14		562.2348	562.2364	2.83	2.83	94.15	99.74	99.74	585.2241	19
☐	C32 H38 N2 O3 S2	C32 H38 N2 Na O3 S2	585.2216	97.91		562.2348	562.2324	-4.34	4.34	94.02	99.63	99.39	585.2241	15
☐	C24 H38 N2 O13	C24 H38 N2 Na O13	585.2266	97.47		562.2348	562.2374	4.58	4.58	92.38	99.87	99.32	585.2241	7

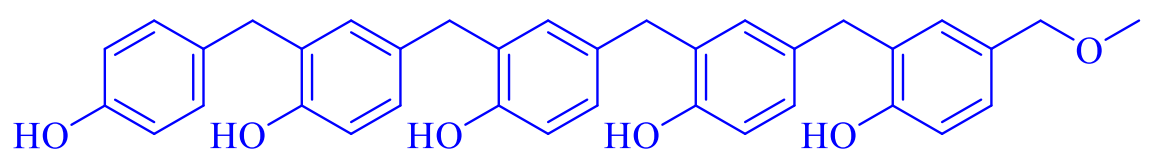

11

page 1

Figure S142. The HR-ESIMS report of compound **11** (page 3)

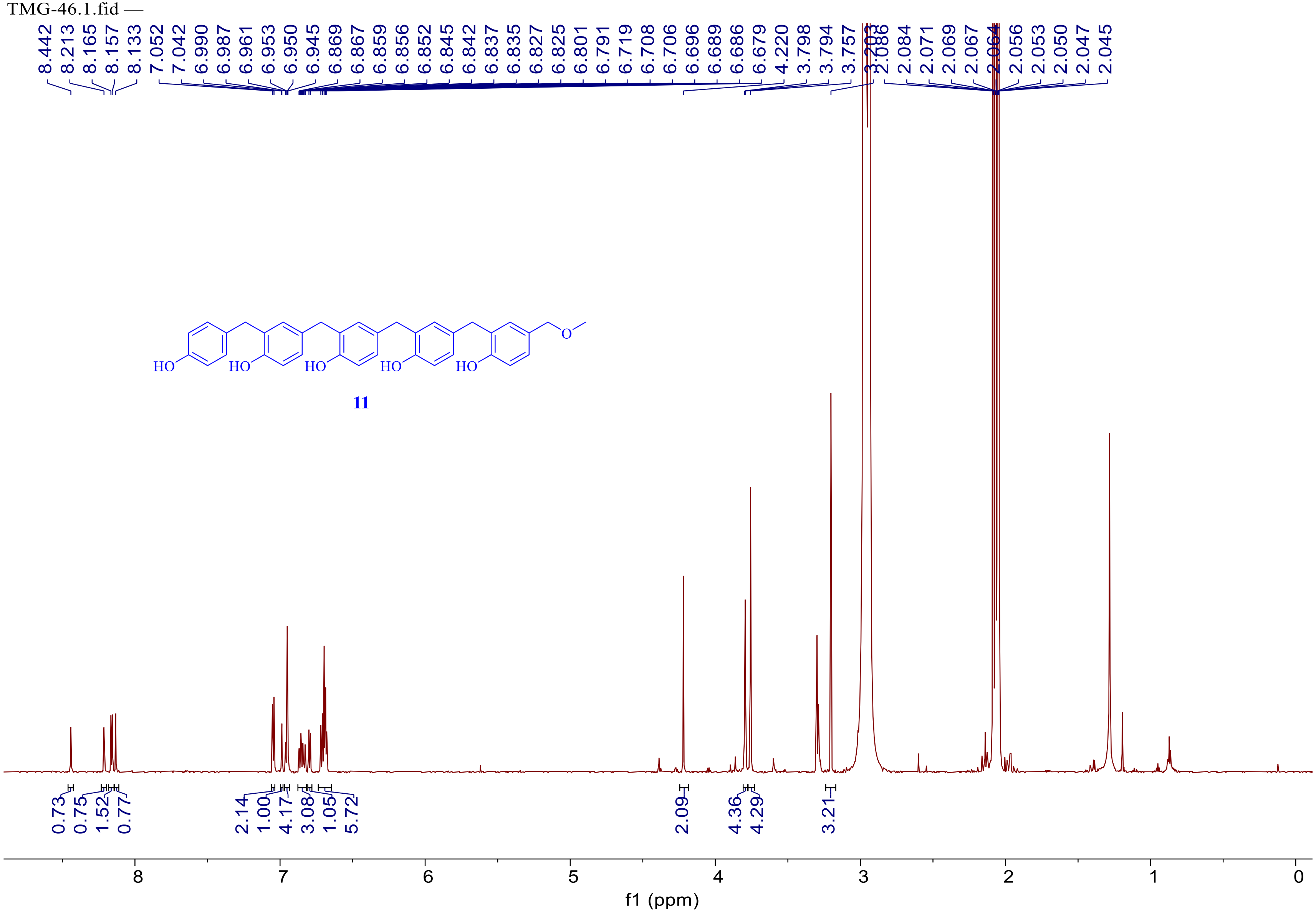

Figure S143. The ^{1}H NMR spectrum of compound **11** in acetone-d_6 (800 MHz)

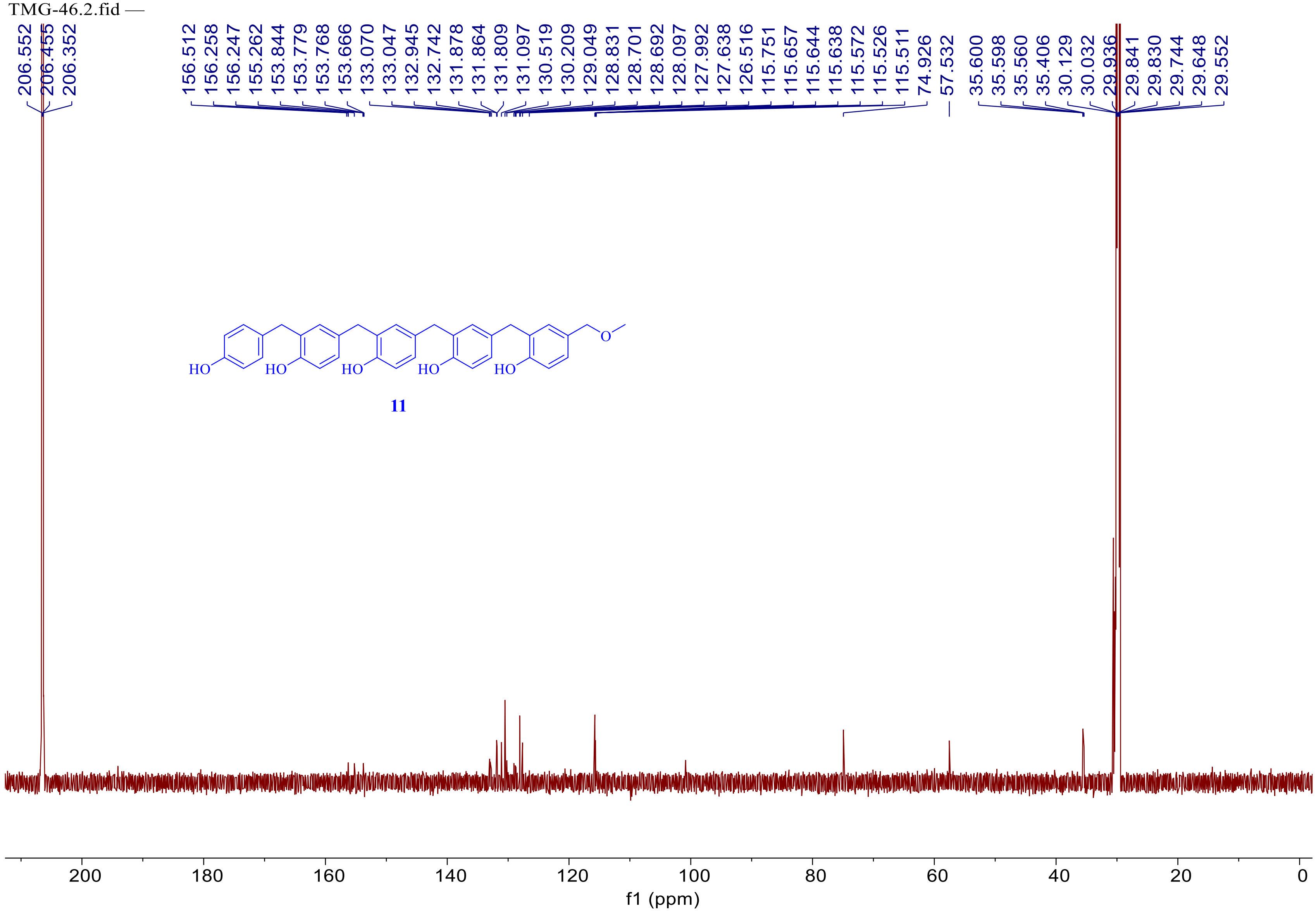

Figure S144. The ^{13}C NMR spectrum of compound **11** in acetone-d_6 (200 MHz)

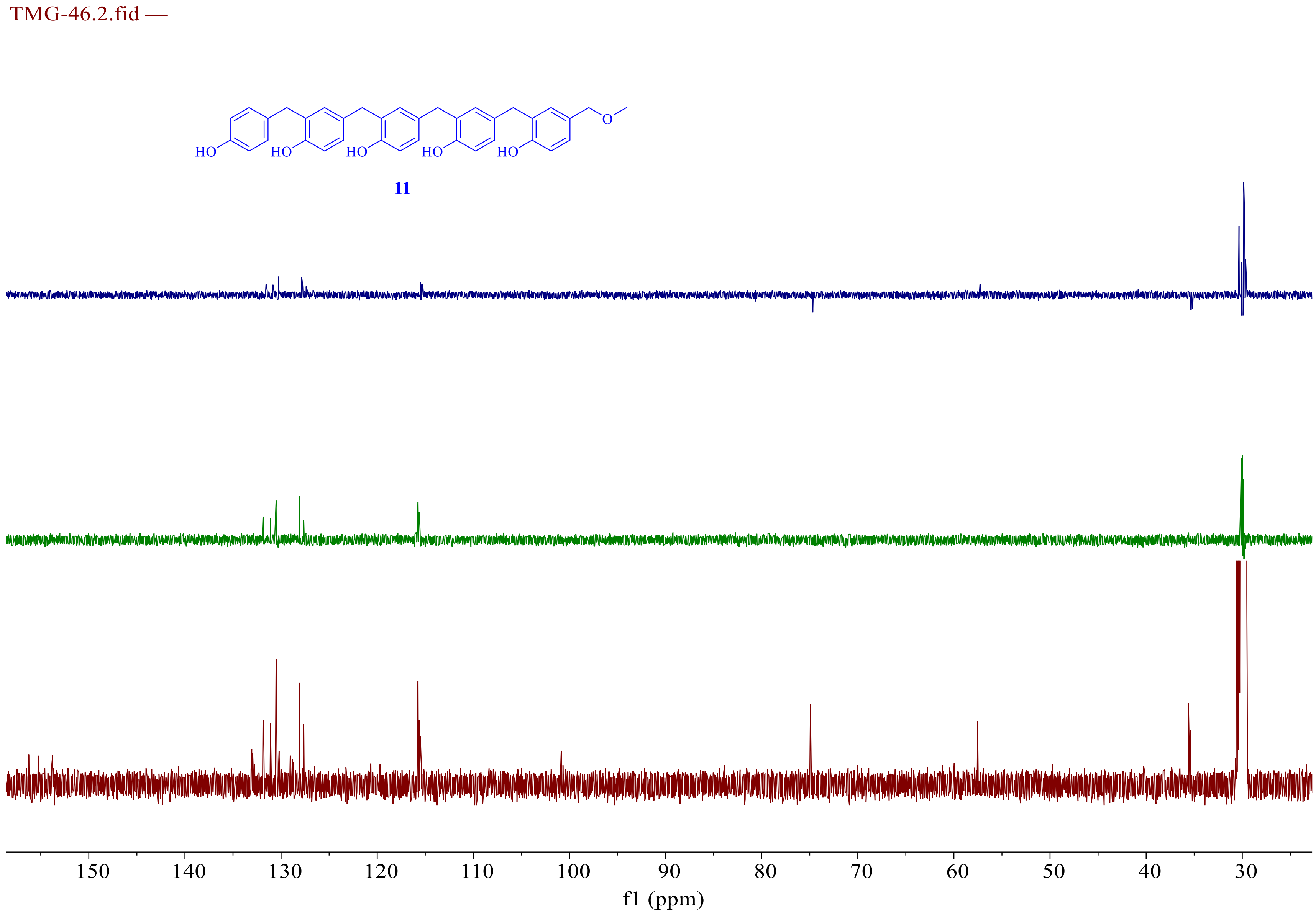

Figure S145. The DEPT spectrum of compound **11** in acetone-d_6 (200 MHz)

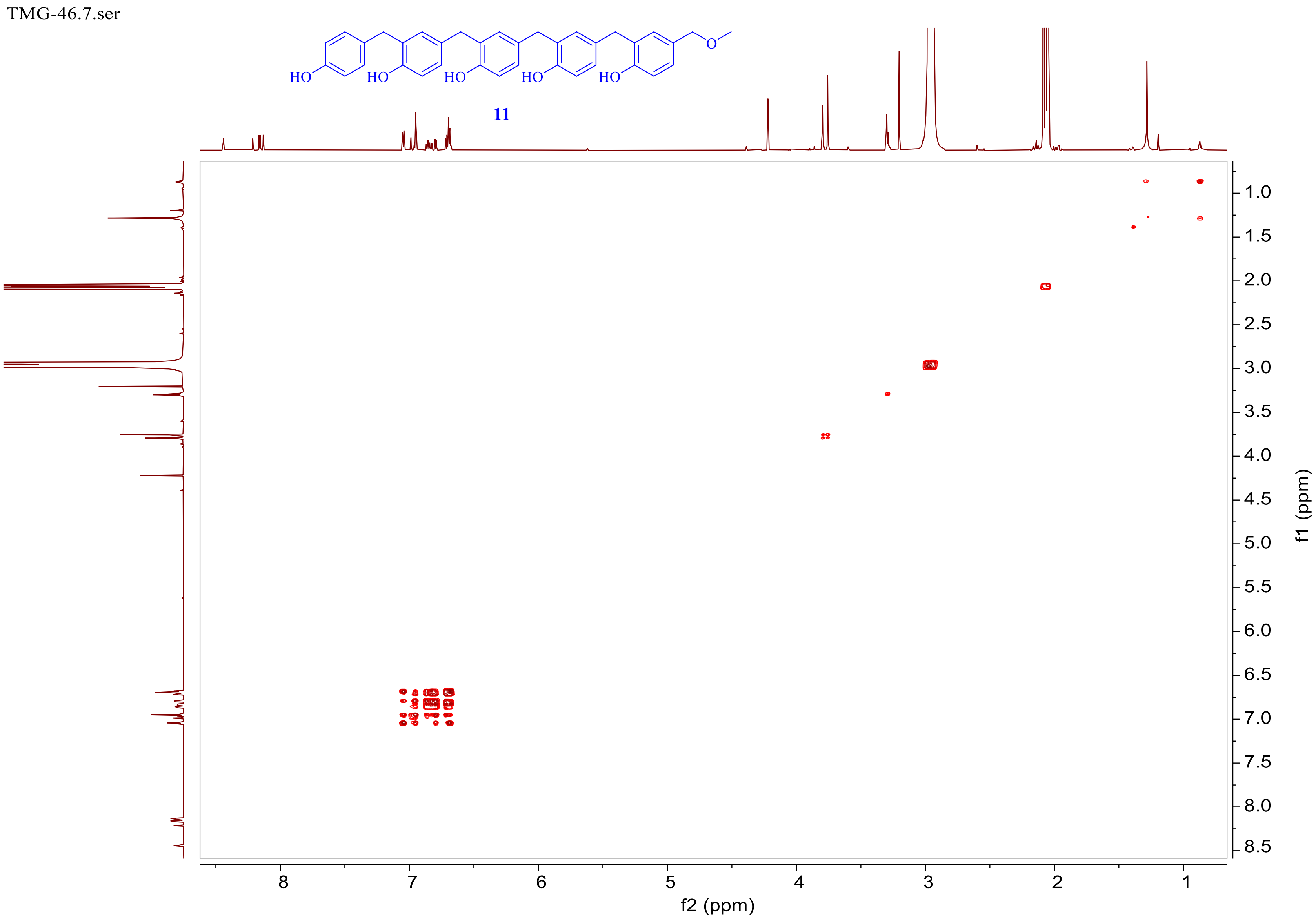

Figure S146. The ^{1}H-^{1}H COSY spectrum of compound **11** in acetone-d_6 (800 MHz)

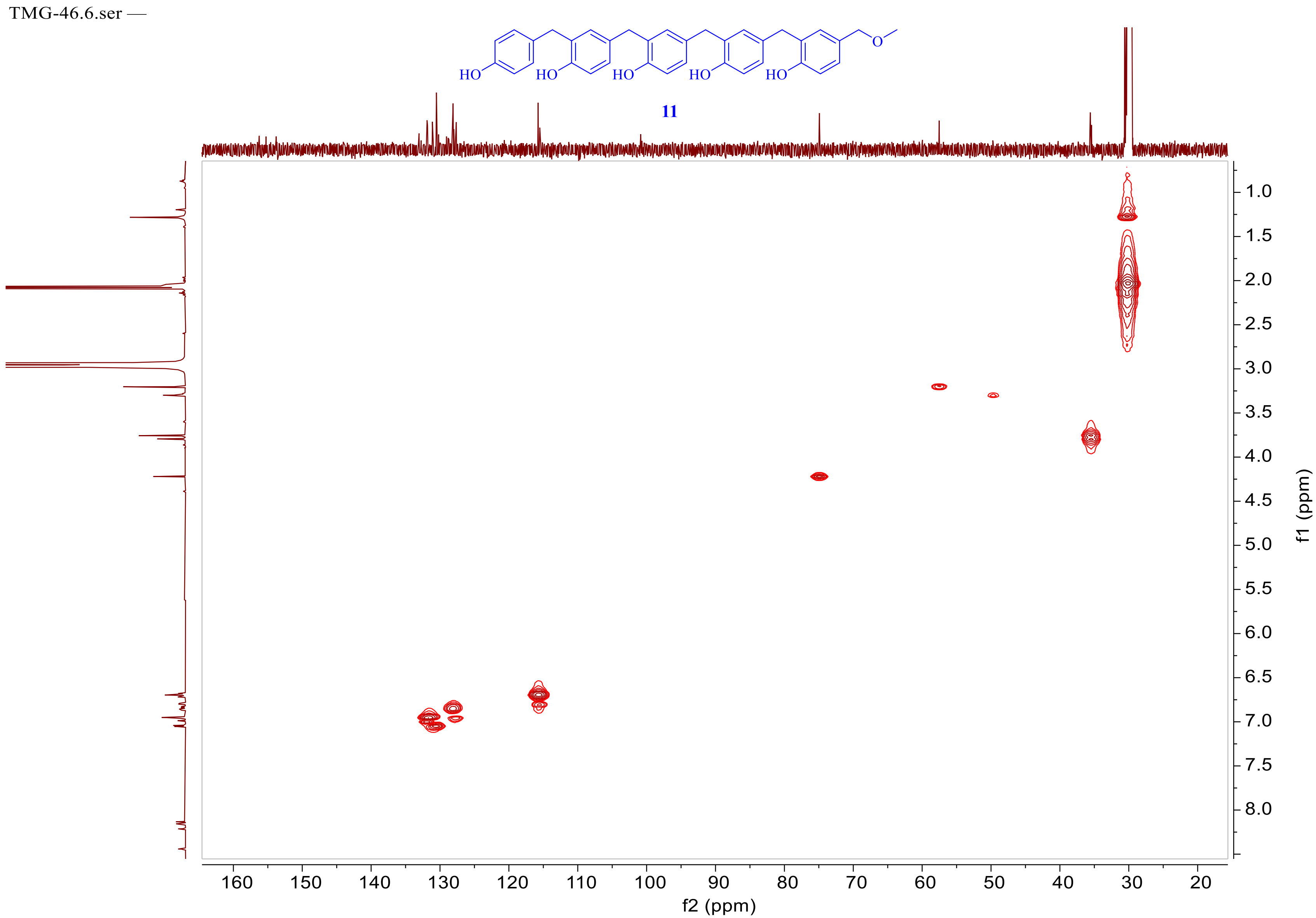

Figure S147. The HSQC spectrum of compound **11** in acetone-d_6 (800 MHz for ^{1}H)

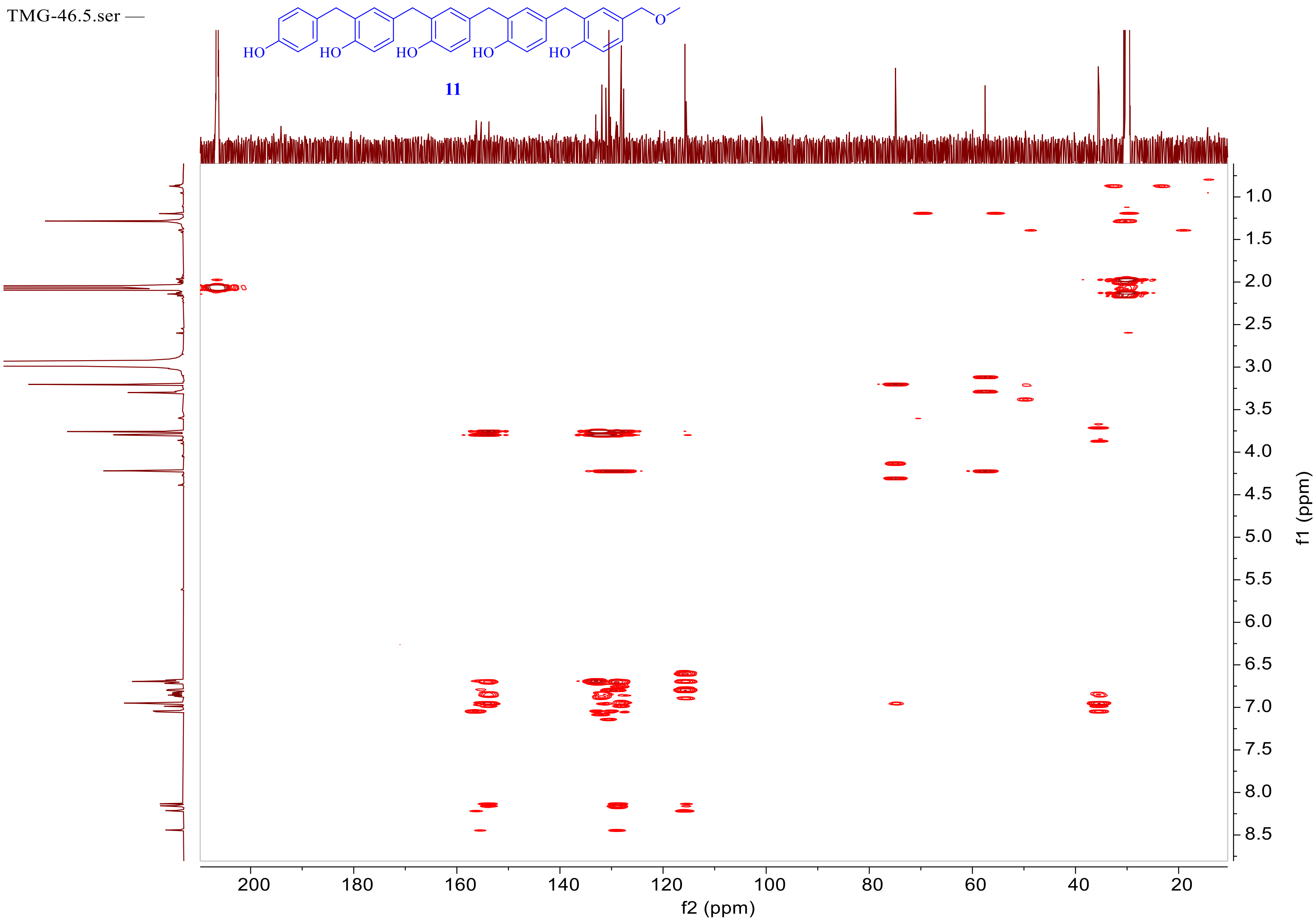

Figure S148. The HMBC spectrum of compound **11** in acetone-d_6 (800 MHz for ^{1}H)

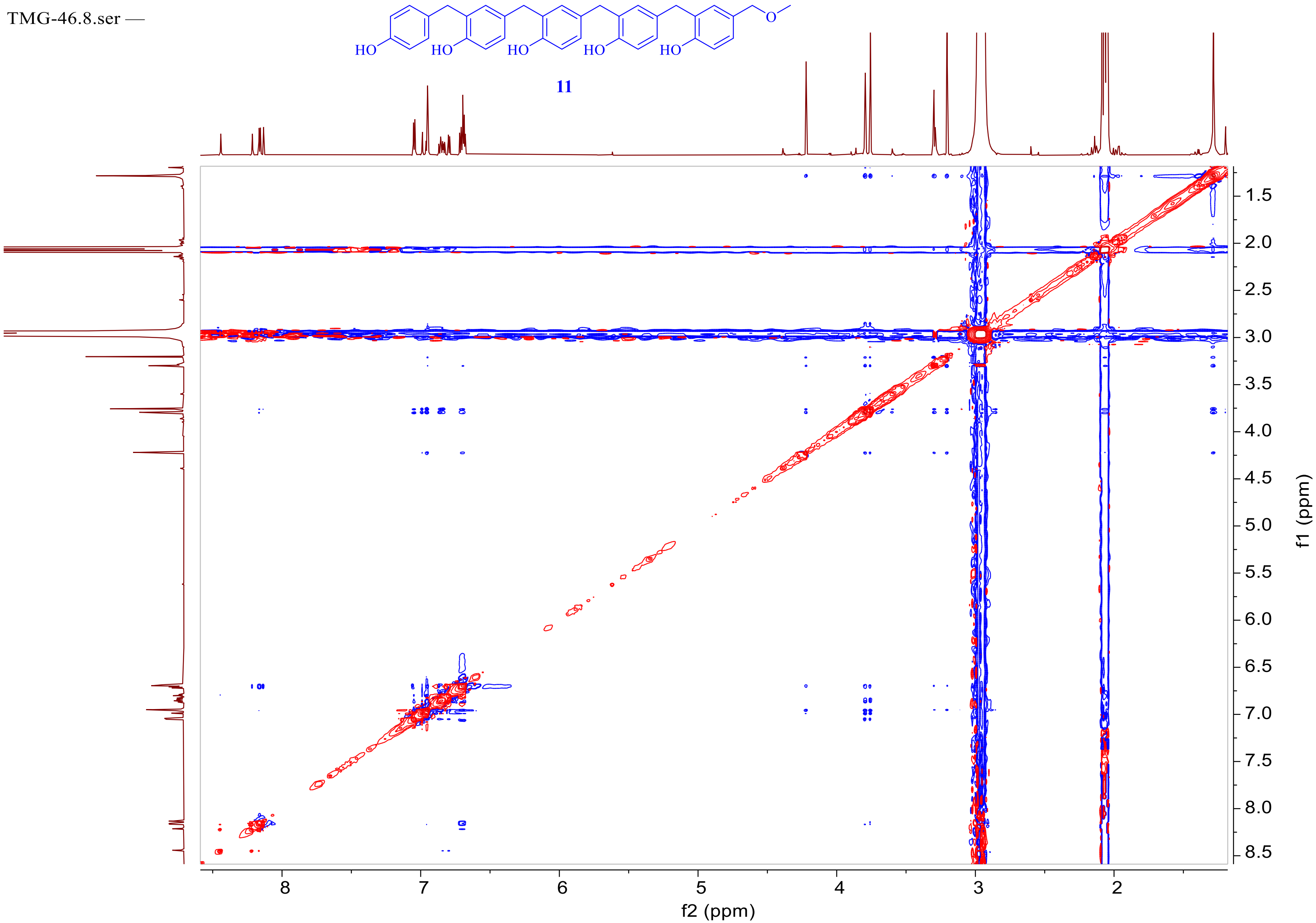

Figure S149. The NOESY spectrum of compound **11** in acetone-d_6 (800 MHz)

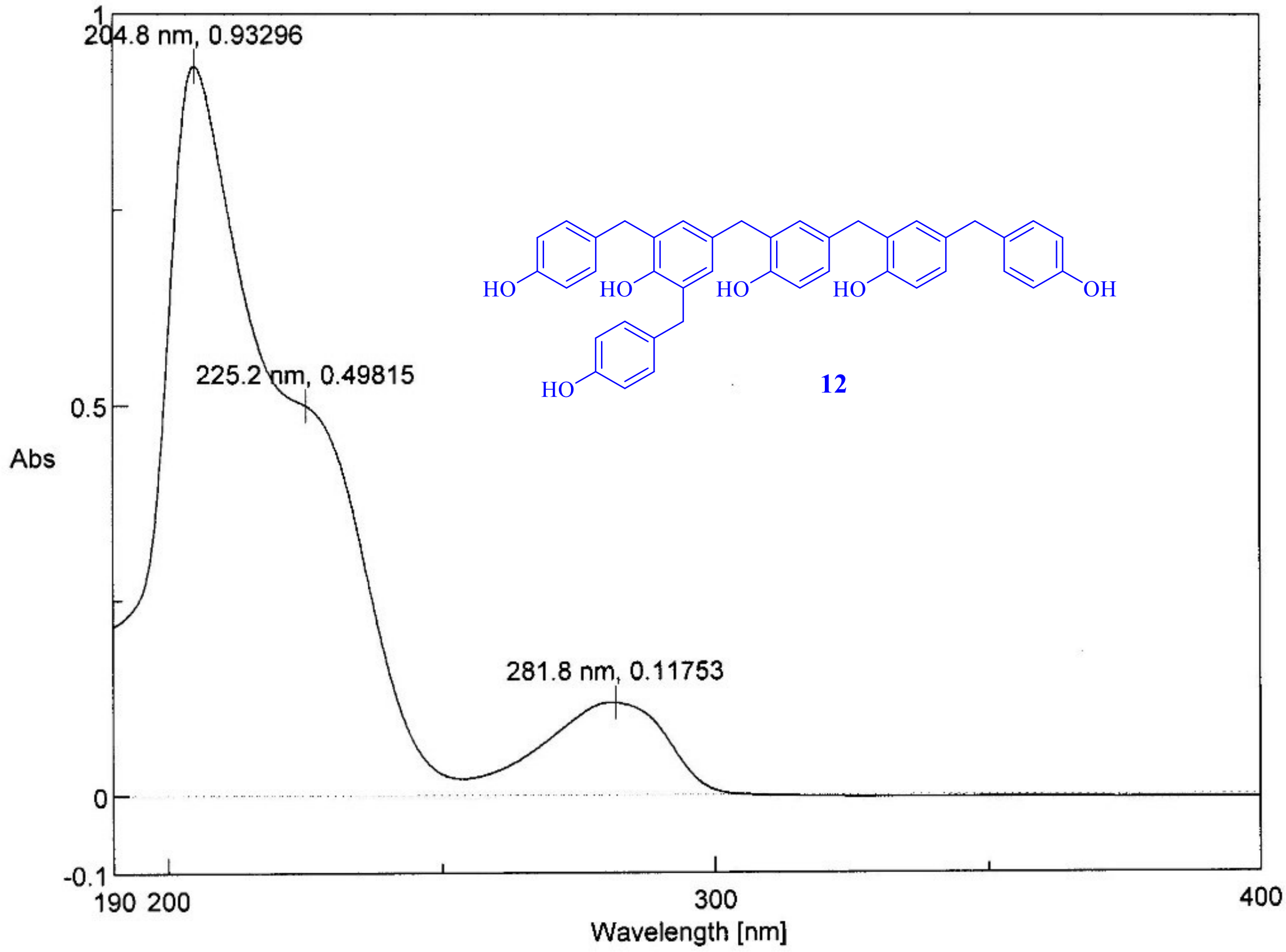

Memory-23

[Comment]
Sample Name TMG-65
Comment 0.02
User
Division UV
Company 324

[Measurement Information]
Instrument Name V-650
Model Name V-650
Serial No. A034461150

Accessory PSC-718
Accessory S/N A001761114
Position 1
Cell Length 10 mm
Temperature 19.97 C
Control Sonsor Holder
Monitor Sensor Holder
Start Mode Start immediately

Photometric Mode Abs
Measurement range 400 - 190 nm
Data pitch 0.2 nm
Band width(UV/Vis) 2.0 nm
Response Medium
Scanning speed 200 nm/min
Source Change 340 nm
Light Source D2/WI
Filter Exchange Step
Correction Baseline

[Data Information]
Creation Date 2014-2-26 9:55

Data array type Linear data array
Horizontal Wavelength [nm]
Vertical Abs
Start 400 nm
End 190 nm
Data pitch 0.2 nm
Data points 1051

Figure S150. The UV spectrum of compound **12** in MeOH

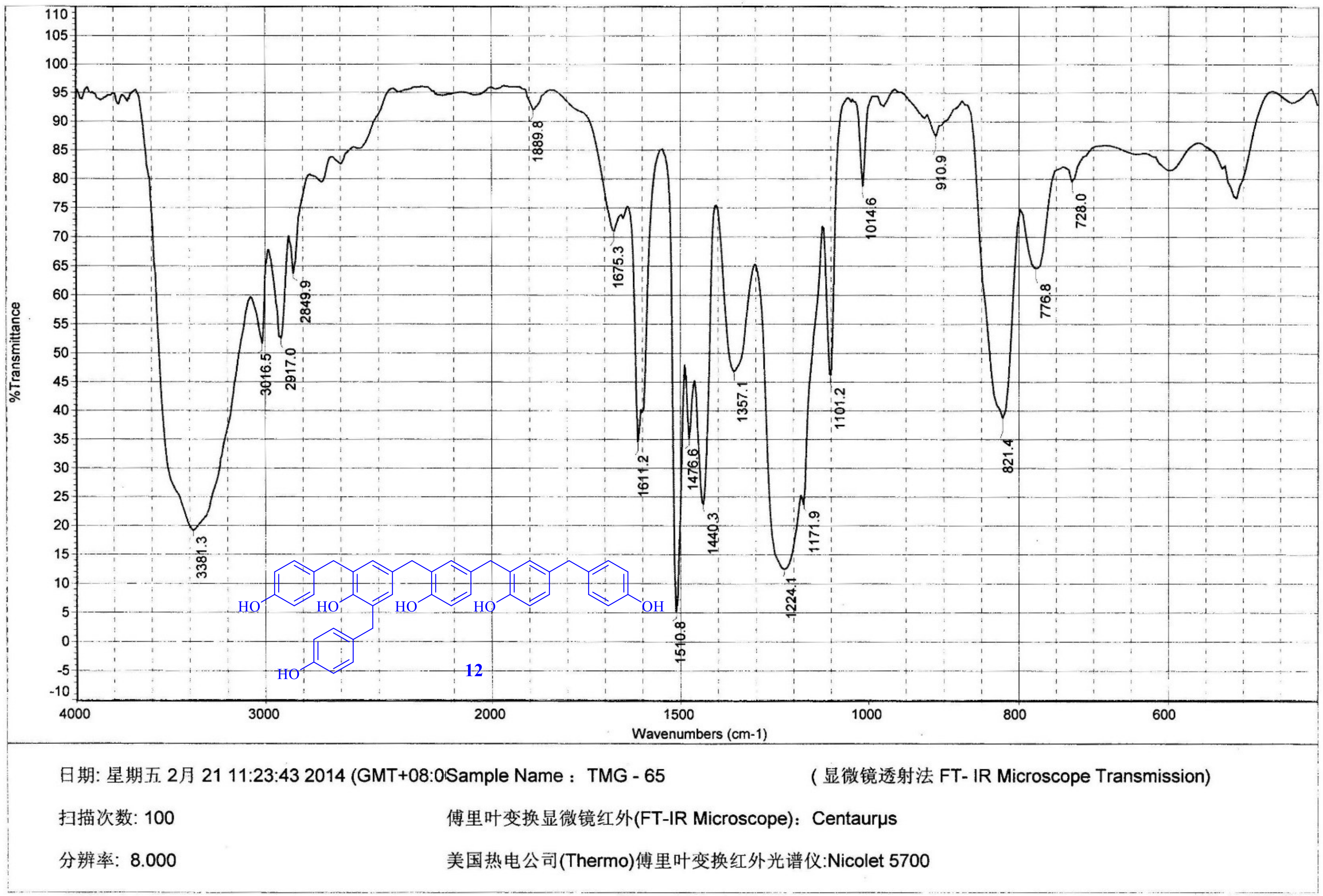

Figure S151. The IR spectrum of compound **12**

Single Mass Spectrum Deconvolution Report

Analysis Name: guoql096.d **Instrument:** LC-MSD-Trap-SL **Print Date:** 12/12/2012 10:52:32 AM
Method: TEST.MS **Operator:** Operator **Acq. Date:** 12/12/2012 10:33:41 AM
Sample Name: TMG-65
Analysis Info:

Acquisition Parameter:

Mass Range Mode	Std/Normal	Trap Drive	53.0	Scan Begin	100 m/z
Ion Polarity	Positive	Octopole RF Amplitude	171.0 Vpp	Scan End	800 m/z
Ion Source Type	ESI	Capillary Exit	-106.0 Volt	Averages	5 Spectra
Dry Temp (Set)	330 °C	Skimmer	-40.0 Volt	Max. Accu Time	200000 µs
Nebulizer (Set)	15.00 psi	Oct 1 DC	-12.00 Volt	ICC Target	-1
Dry Gas (Set)	6.00 l/min	Oct 2 DC	-1.70 Volt	Charge Control	on

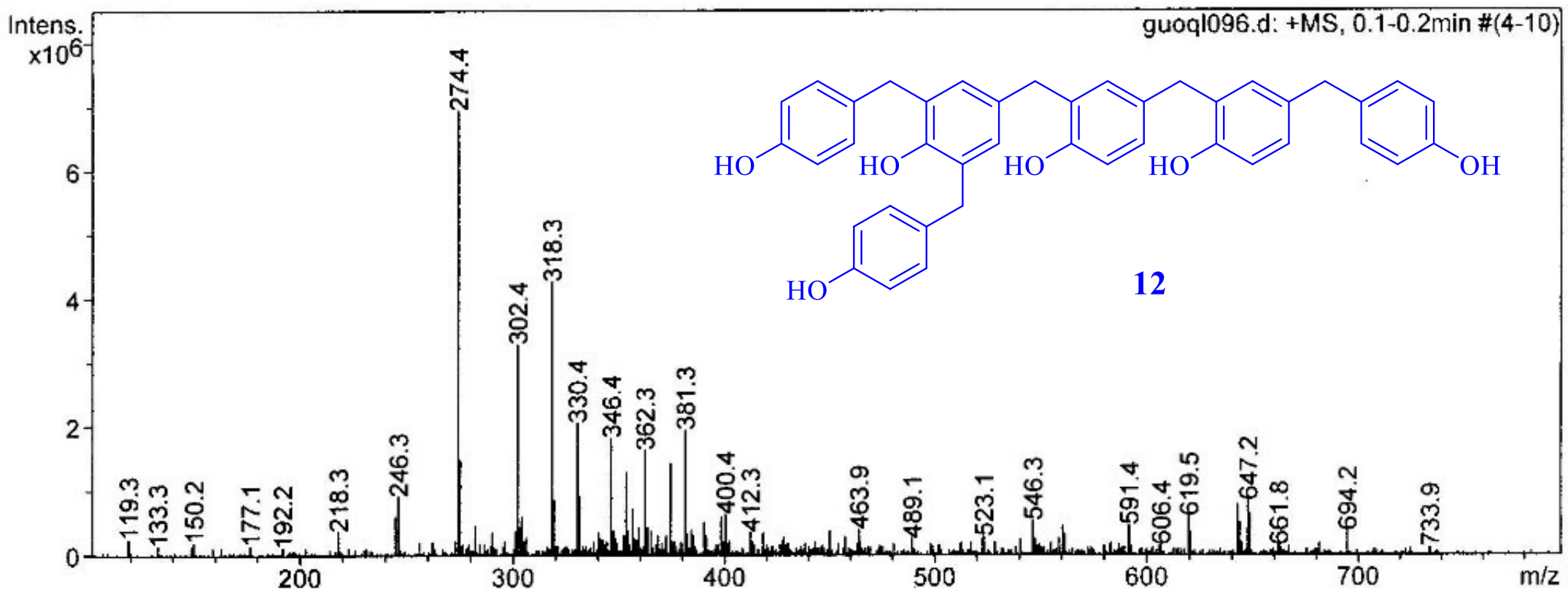

Component Molecular Mass Molecule Absolute Abundance Relative Abundance

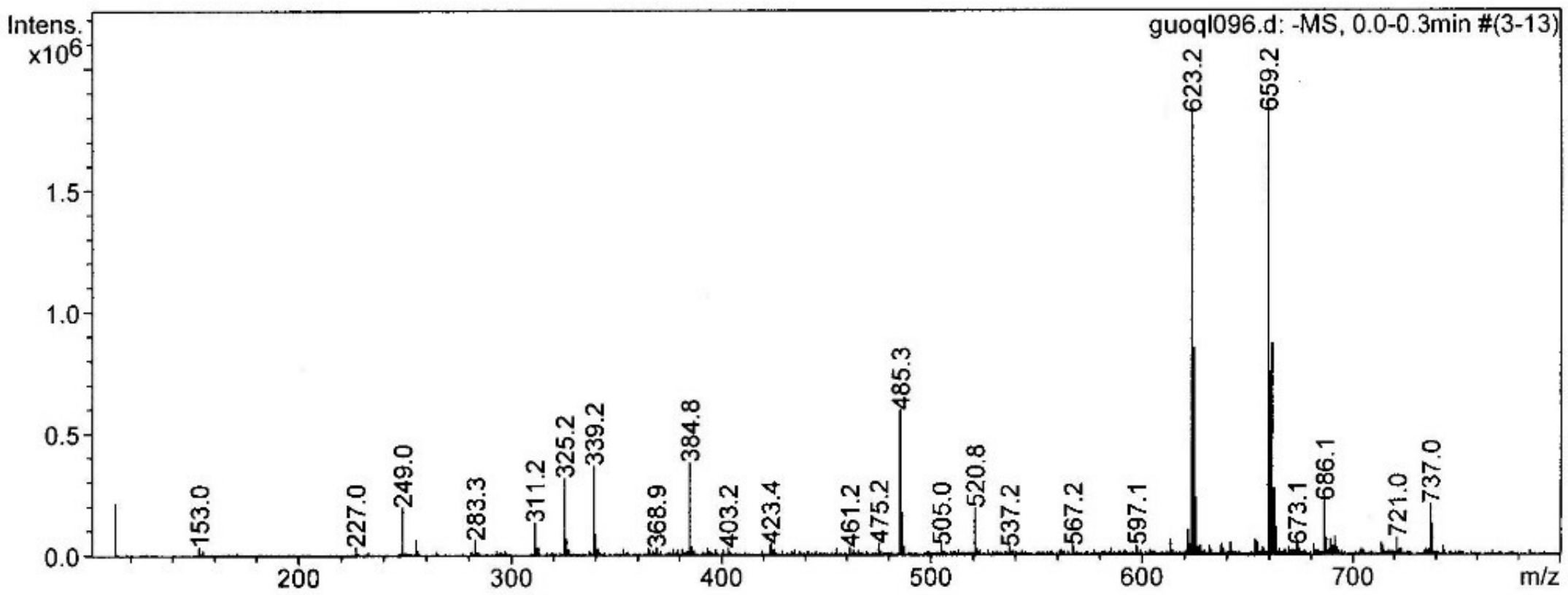

Component Molecular Mass Molecule Absolute Abundance Relative Abundance

MSD Trap Report v 4 (A4-Opt2) Page 1 of 1 Agilent Technologies

Figure S152. The ESIMS of compound **12**

Qualitative Analysis Report

**Data Filename**	2014022610.d	**Sample Name**	TMG-65
**Sample Type**	Sample	**Position**	P1-C1
**Instrument Name**	Instrument 1	**User Name**	
**Acq Method**		**IRM Calibration Status**	Success
**DA Method**	TEST LCMS.m	**Comment**	

User Chromatograms

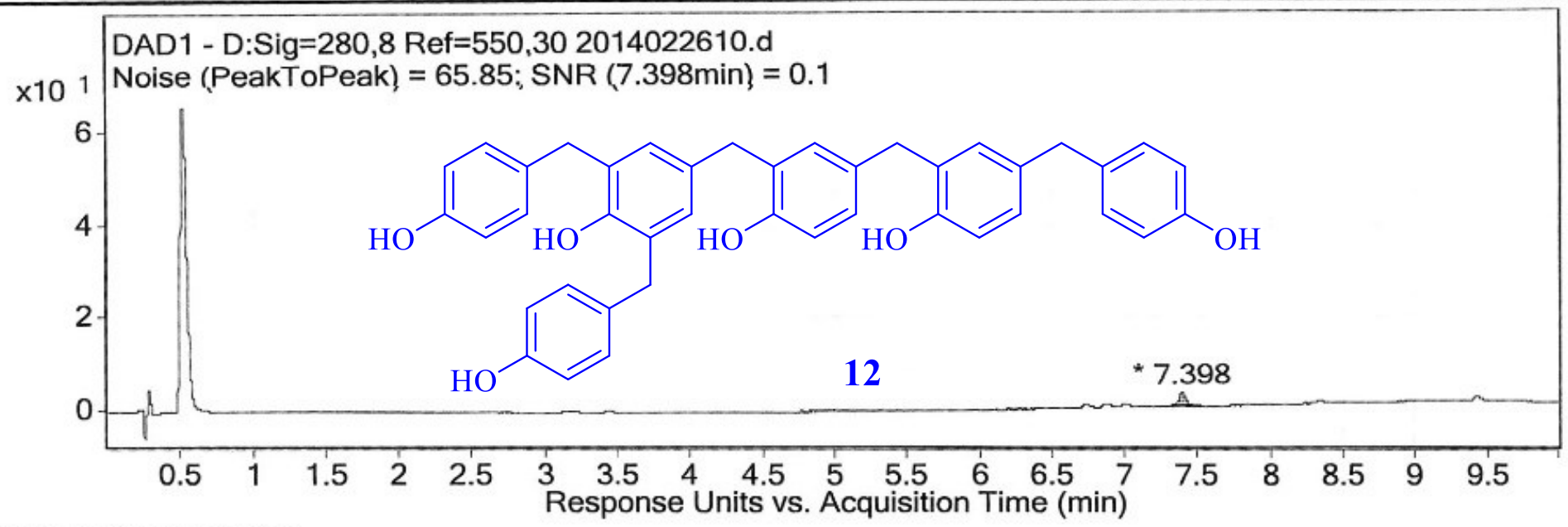

Integration Peak List

Peak	Start	RT	End	Height	Area	Area %	Signal To Noise
1	7.321	7.398	7.523	2.86	8.82	100	0.1

Noise Measurements

Noise Type	Signal Definition	Noise Multiplier	Noise Value
Peak-to-Peak	Area	1	65.85263824

Noise Regions

Start	End
0.5	1
5	5.3
9.99	11

Fragmentor Voltage 135 **Collision Energy** 0 **Ionization Mode** ESI

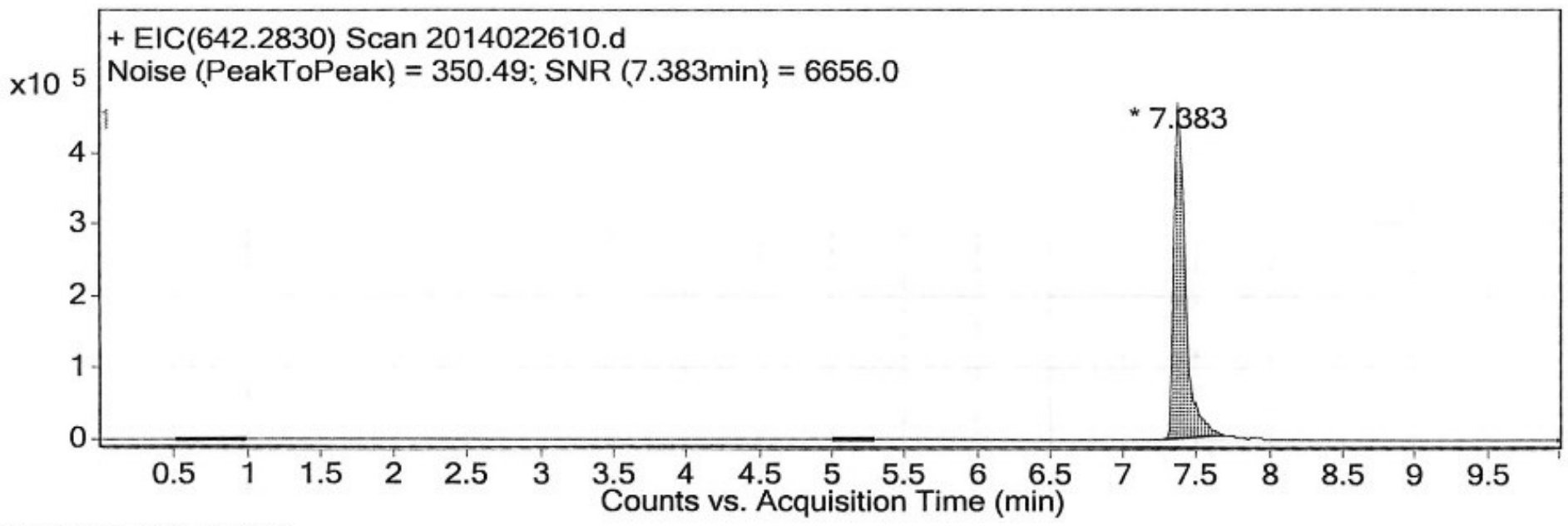

Integration Peak List

Peak	Start	RT	End	Height	Area	Area %	Signal To Noise
1	7.271	7.383	7.689	468866	2332844	100	6656

Noise Measurements

Noise Type	Signal Definition	Noise Multiplier	Noise Value
Peak-to-Peak	Area	1	350.4895935

Noise Regions

Start	End
0.5	1
5	5.3
9.99	11

User Spectra

Figure S153. The HR-ESIMS report of compound **12** (page 1)

Qualitative Analysis Report

Fragmentor Voltage	Collision Energy	Ionization Mode
135	0	Esi

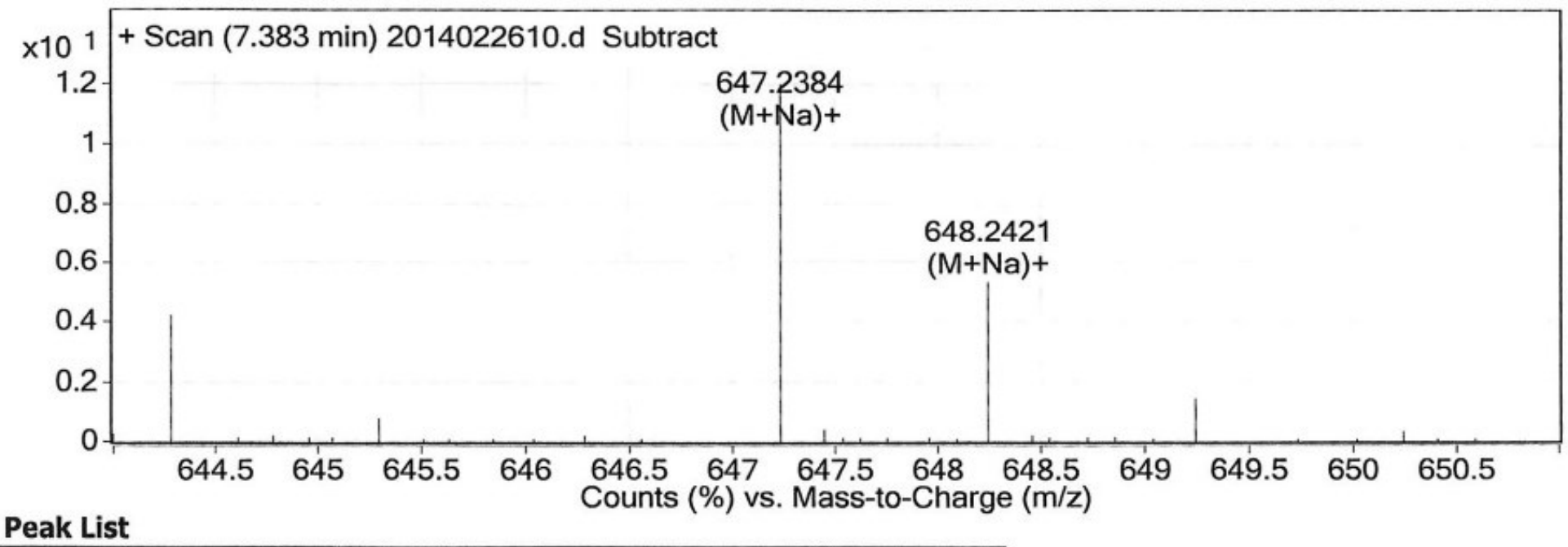

Peak List

m/z	z	Abund	Formula	Ion
227.1639	1	873375		
228.1672	1	129354		
244.1902	1	641564		
249.1455		242738		
274.2735	1	1240147		
275.2766	1	235635		
642.2842	1	471086		
643.2873	1	215317		
647.2384	1	148442	C41 H36 Na O6	(M+Na)+
656.2998	1	143940		

Formula Calculator Element Limits

Element	Min	Max
C	3	100
H	0	500
O	0	90
N	0	5
S	0	0
Cl	0	0
Br	0	0
Si	0	0
F	0	0
P	0	0

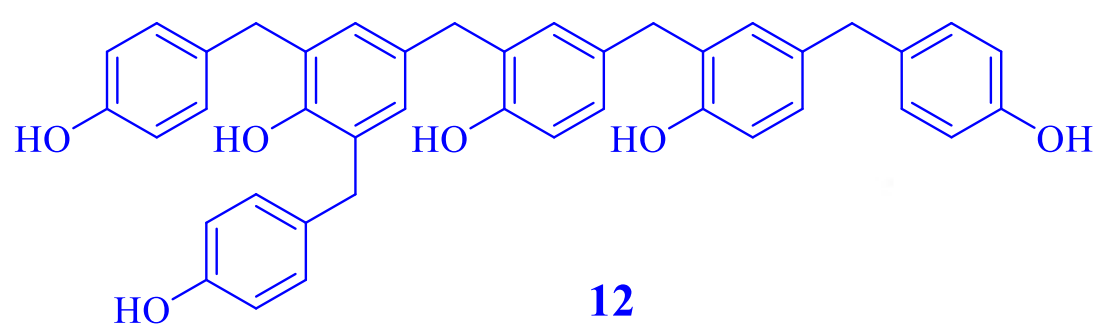

12

Formula Calculator Results

Formula	Best	Mass	Tgt Mass	Diff (ppm)	Ion Species	Score
C39 H34 N3 O5		624.2493	624.2498	0.94	C39 H34 N3 Na O5	99.93
C41 H36 O6	TRUE	624.2492	624.2512	3.11	C41 H36 Na O6	99.82
C36 H36 N2 O8		624.2493	624.2472	-3.35	C36 H36 N2 Na O8	99.56
C42 H32 N4 O2		624.2493	624.2525	5.23	C42 H32 N4 Na O2	99.41
C34 H34 N5 O7		624.2493	624.2458	-5.52	C34 H34 N5 Na O7	99.09
C27 H38 N5 O12		624.2493	624.2517	3.88	C27 H38 N5 Na O12	97.41

--- End Of Report ---

Figure S154. The HR-ESIMS report of compound **12** (page 2)

MS Formula Results: + Scan (7.383 min) Sub (2014022610.d)

m/z	Ion	Formula	Abundance
647.2384	(M+Na)+	C41 H36 Na O6	148442

Best	Formula (M)	Ion Formula	Calc m/z	Score	Cross S	Mass	Calc Mass	Diff (ppm)	Abs Diff (ppm)	Abund Match	Spacing Mat	Mass Match	m/z	DBE
☐	C39 H34 N3 O5	C39 H34 N3 Na O5	647.2391	99.93		624.2493	624.2498	0.94	0.94	99.81	99.99	99.97	647.2384	24.5
☑	C41 H36 O6	C41 H36 Na O6	647.2404	99.82		624.2492	624.2512	3.11	3.11	99.9	100	99.68	647.2384	24
☐	C36 H36 N2 O8	C36 H36 N2 Na O8	647.2364	99.56		624.2493	624.2472	-3.35	3.35	99.1	99.99	99.63	647.2384	20
☐	C42 H32 N4 O2	C42 H32 N4 Na O2	647.2417	99.41		624.2493	624.2525	5.23	5.23	99.45	99.99	99.1	647.2384	29
☐	C34 H34 N5 O7	C34 H34 N5 Na O7	647.235	99.09		624.2493	624.2458	-5.52	5.52	98.52	99.97	99	647.2384	20.5
☐	C27 H38 N5 O12	C27 H38 N5 Na O12	647.2409	97.41		624.2493	624.2517	3.88	3.88	91.82	99.95	99.51	647.2384	11.5

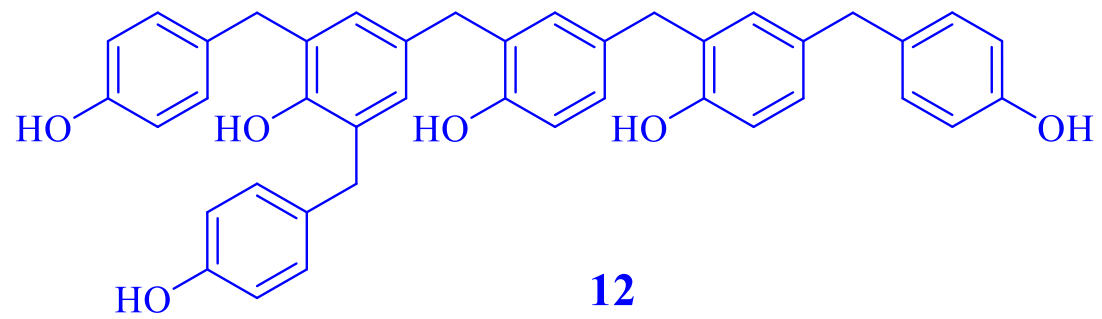

12

page 1

Figure S155. The HR-ESIMS report of compound **12** (page 3)

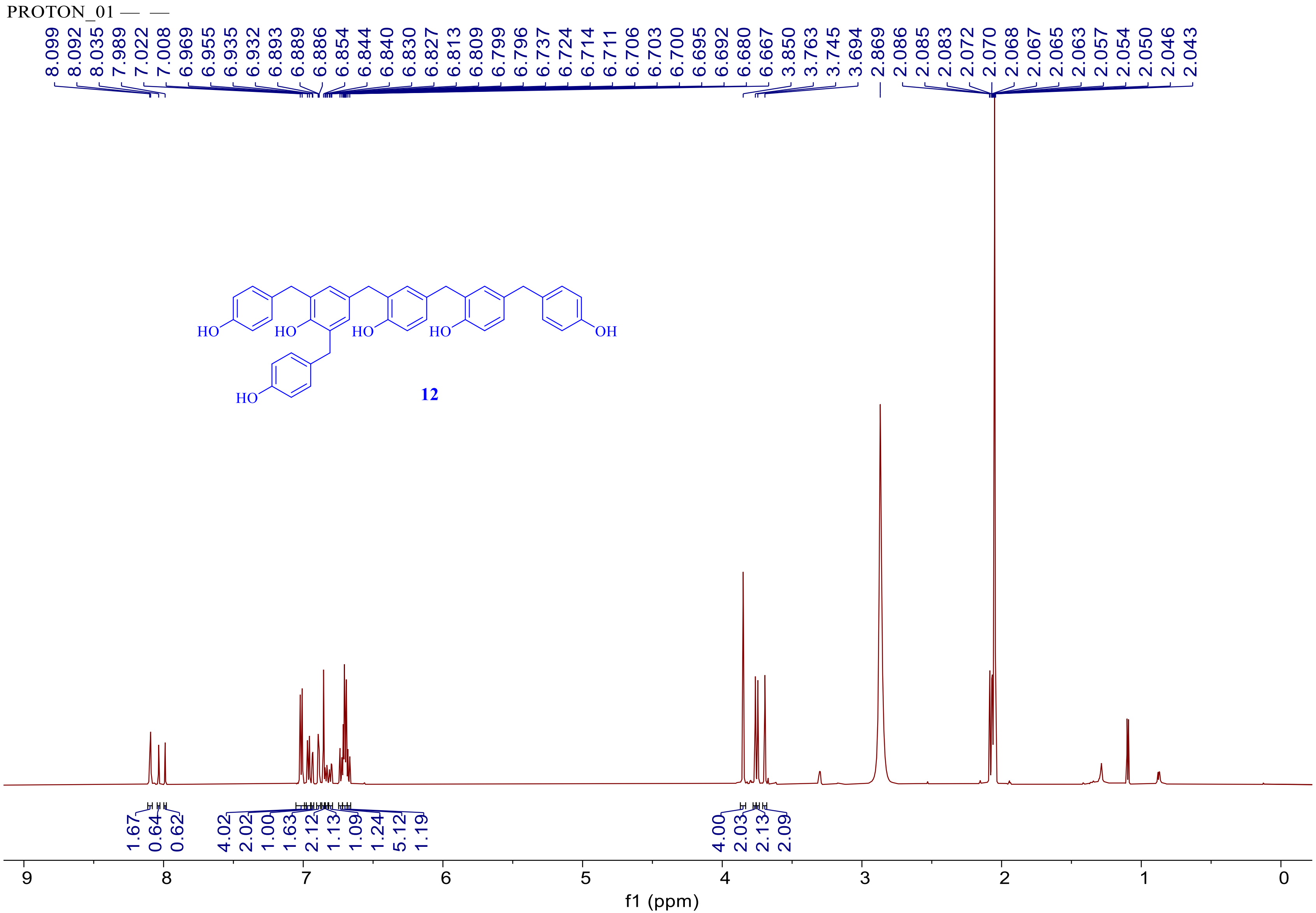

Figure S156. The ^{1}H NMR spectrum of compound **12** in acetone-d_6 (600 MHz)

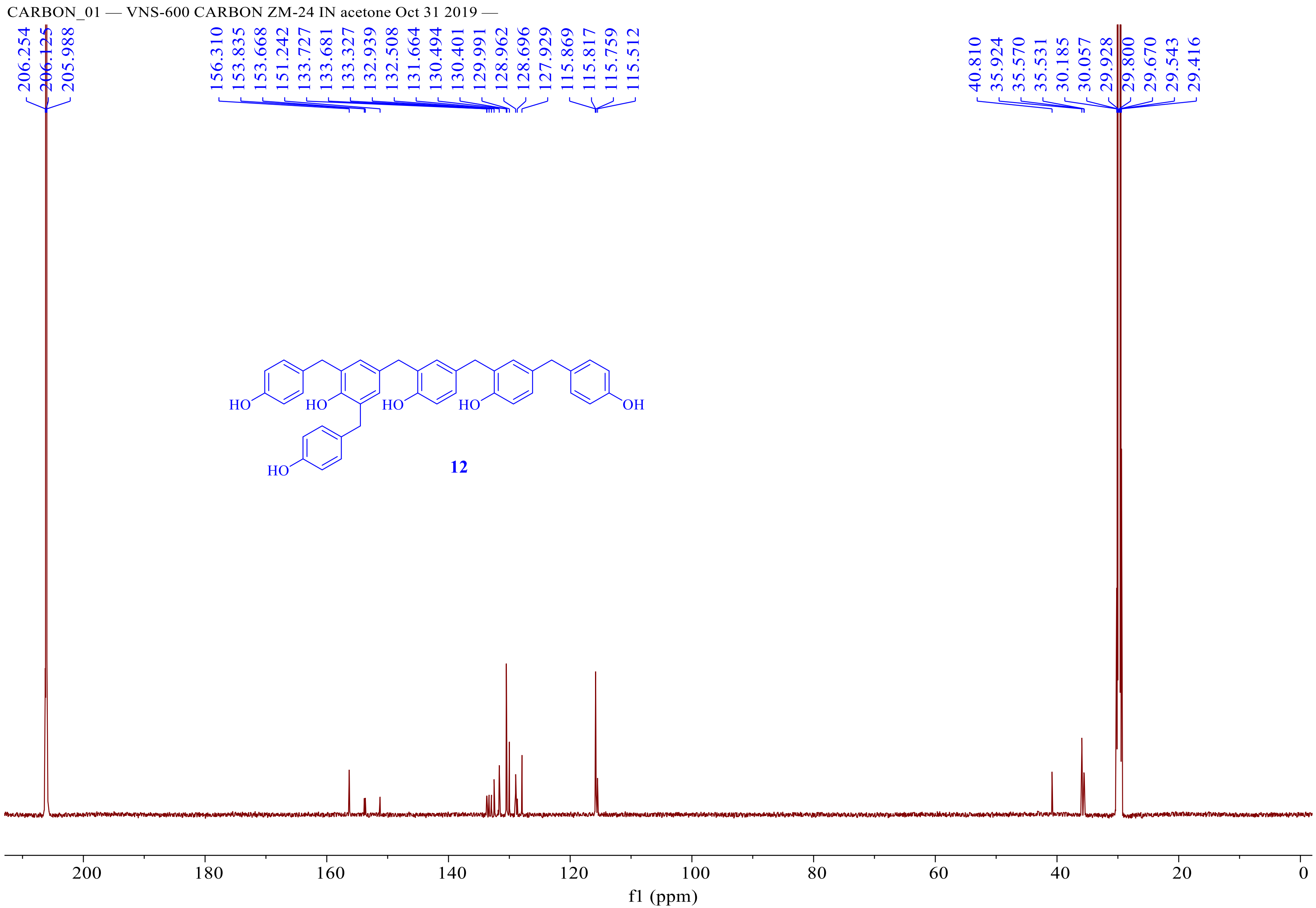

Figure S157. The ^{13}C NMR spectrum of compound **12** in acetone-d_6 (150 MHz)

DEPT_01 — VNS-600 DEPT ZM-24 IN acetone Oct 31 2019 —

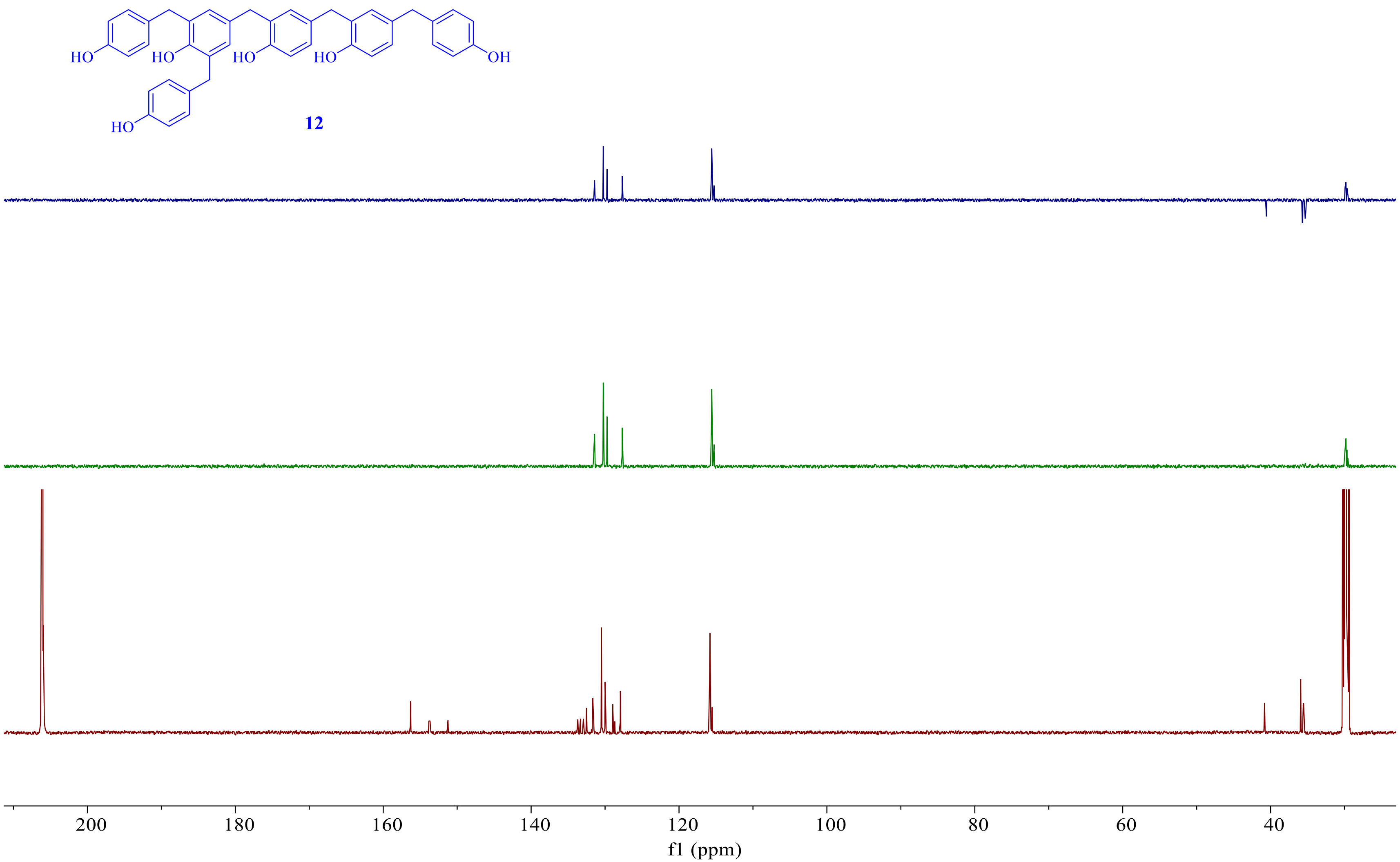

Figure S158. The DEPT spectrum of compound **12** in acetone-d_6 (150 MHz)

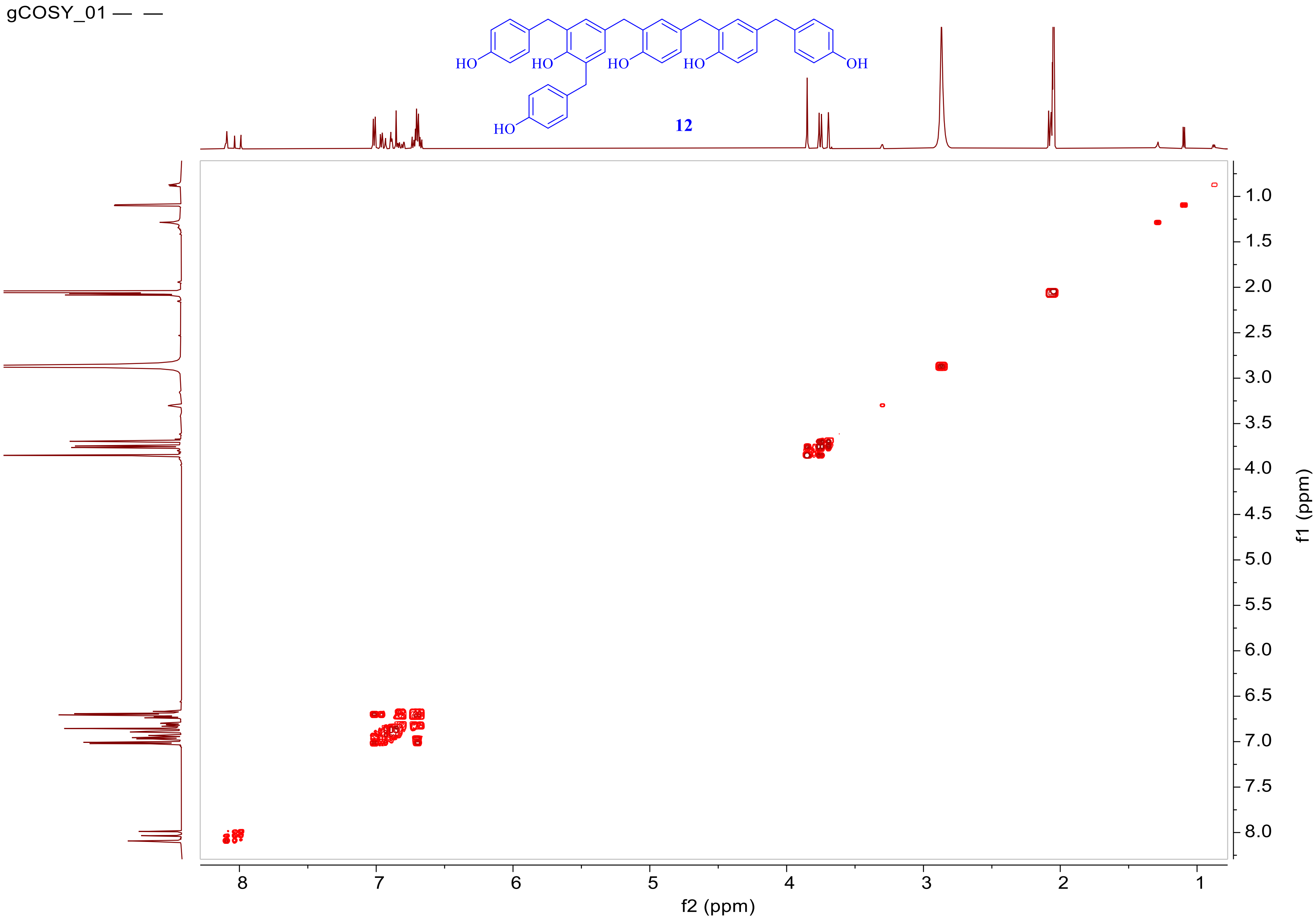

Figure S159. The ^{1}H-^{1}H COSY spectrum of compound **12** in acetone-d_6 (600 MHz)

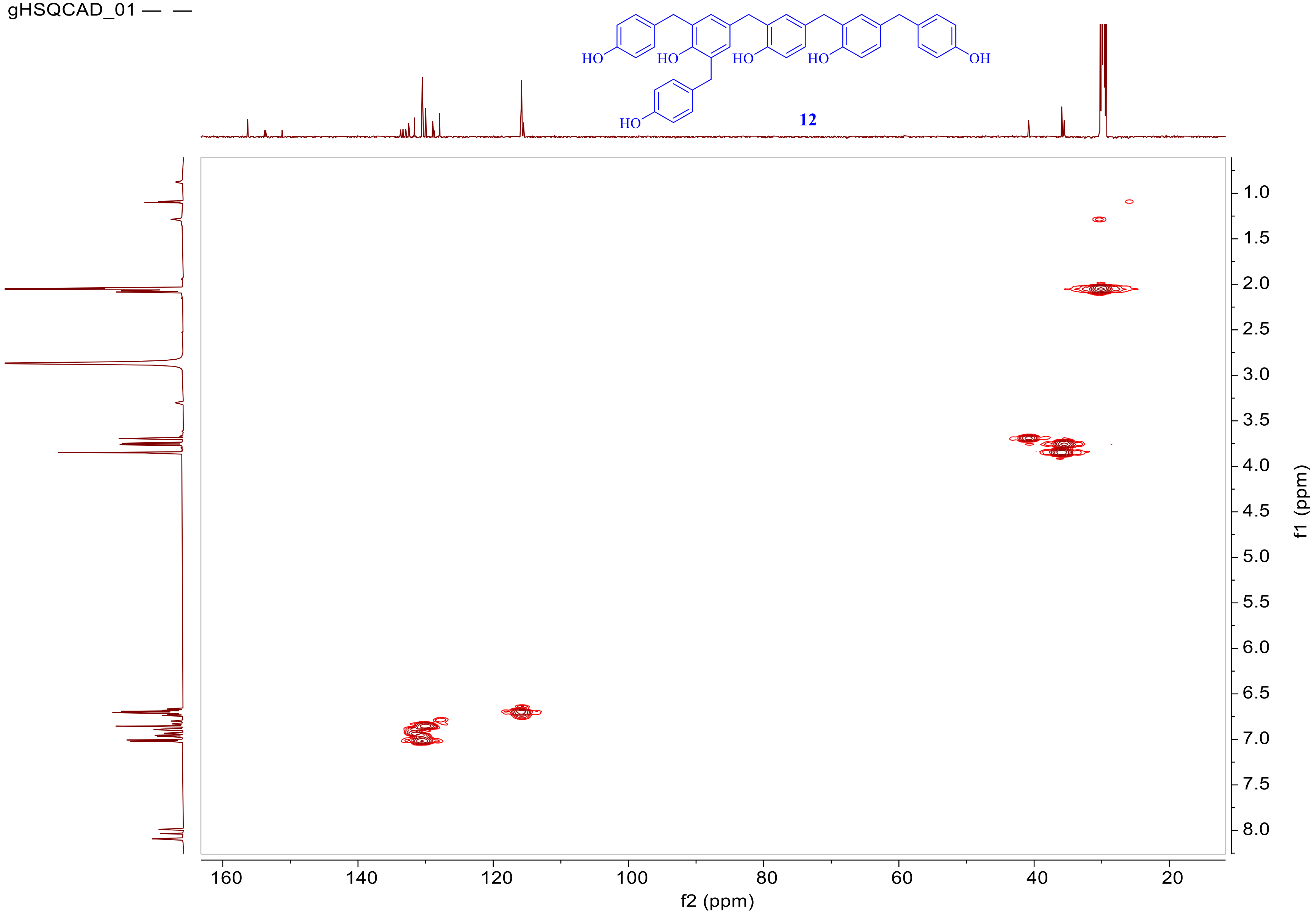

Figure S160. The HSQC spectrum of compound **12** in acetone-d_6 (600 MHz for ^{1}H)

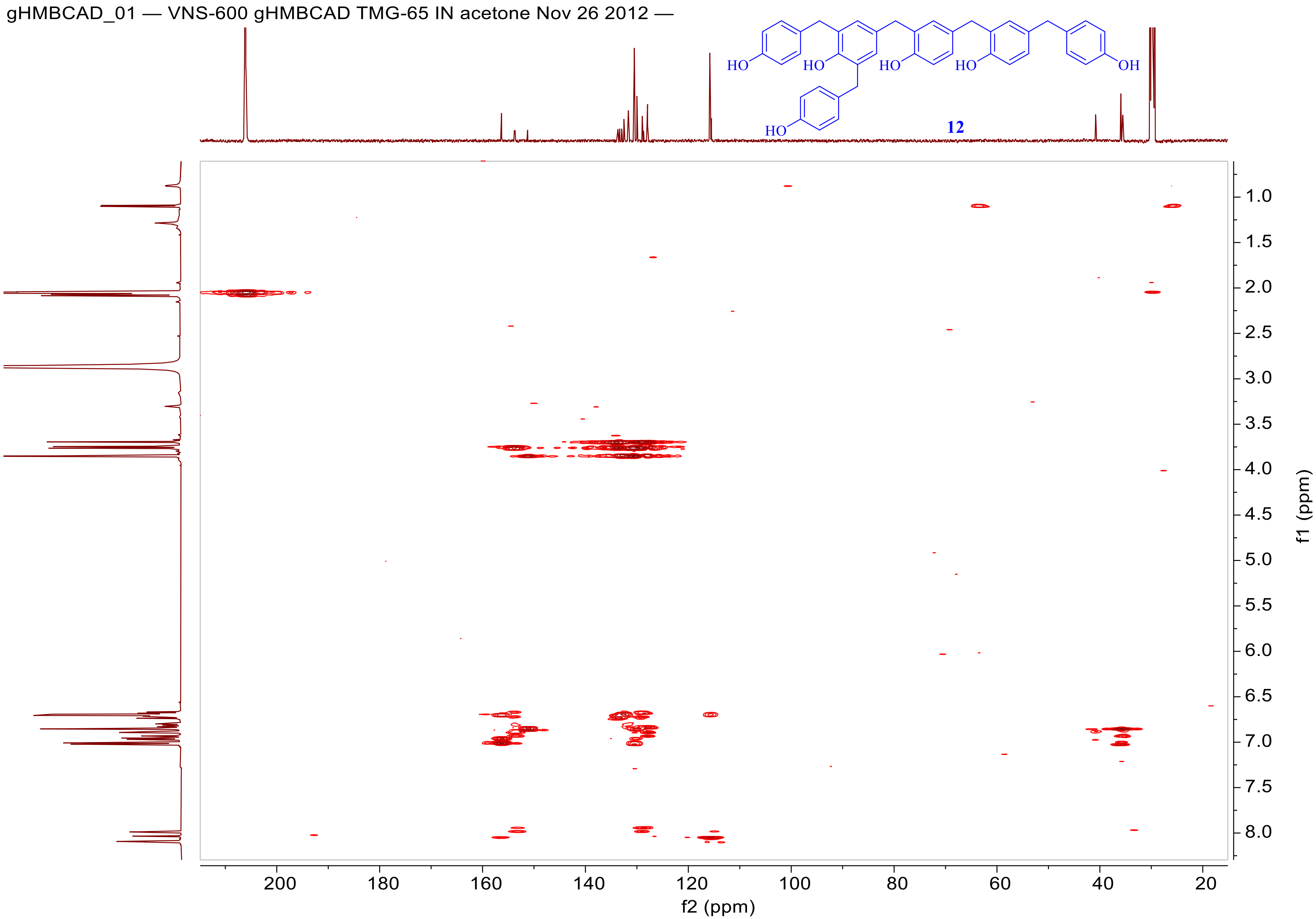

Figure S161. The HMBC spectrum of compound **12** in acetone-d_6 (600 MHz for ^{1}H)

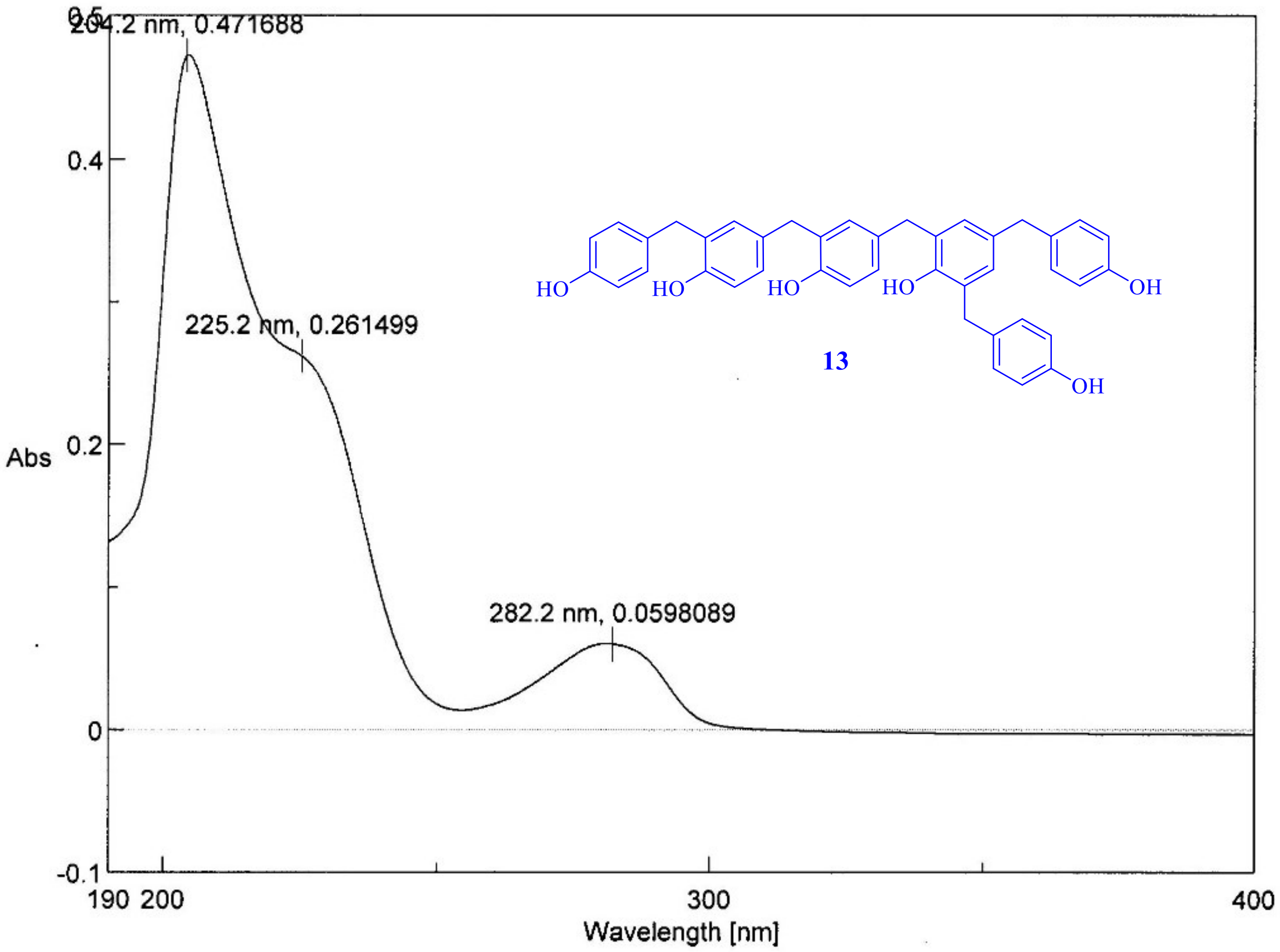

[Comment]
Sample Name TMG-44B
Comment 0.02
User
Division UV
Company 324
[Measurement Information]
Instrument Name V-650
Model Name V-650
Serial No. A034461150

Accessory PSC-718
Accessory S/N A001761114
Position 1
Cell Length 10 mm
Temperature 20.01 C
Control Sonsor Holder
Monitor Sensor Holder
Start Mode Start immediately

Photometric Mode Abs
Measurement range 400 - 190 nm
Data pitch 0.2 nm
Band width(UV/Vis) 2.0 nm
Response Medium
Scanning speed 200 nm/min
Source Change 340 nm
Light Source D2/WI
Filter Exchange Step
Correction Baseline

——— Memory-25

[Data Information]
Creation Date 2014-2-26 9:58

Data array type Linear data array
Horizontal Wavelength [nm]
Vertical Abs
Start 400 nm
End 190 nm
Data pitch 0.2 nm
Data points 1051

Figure S162. The UV spectrum of compound **13** in MeOH

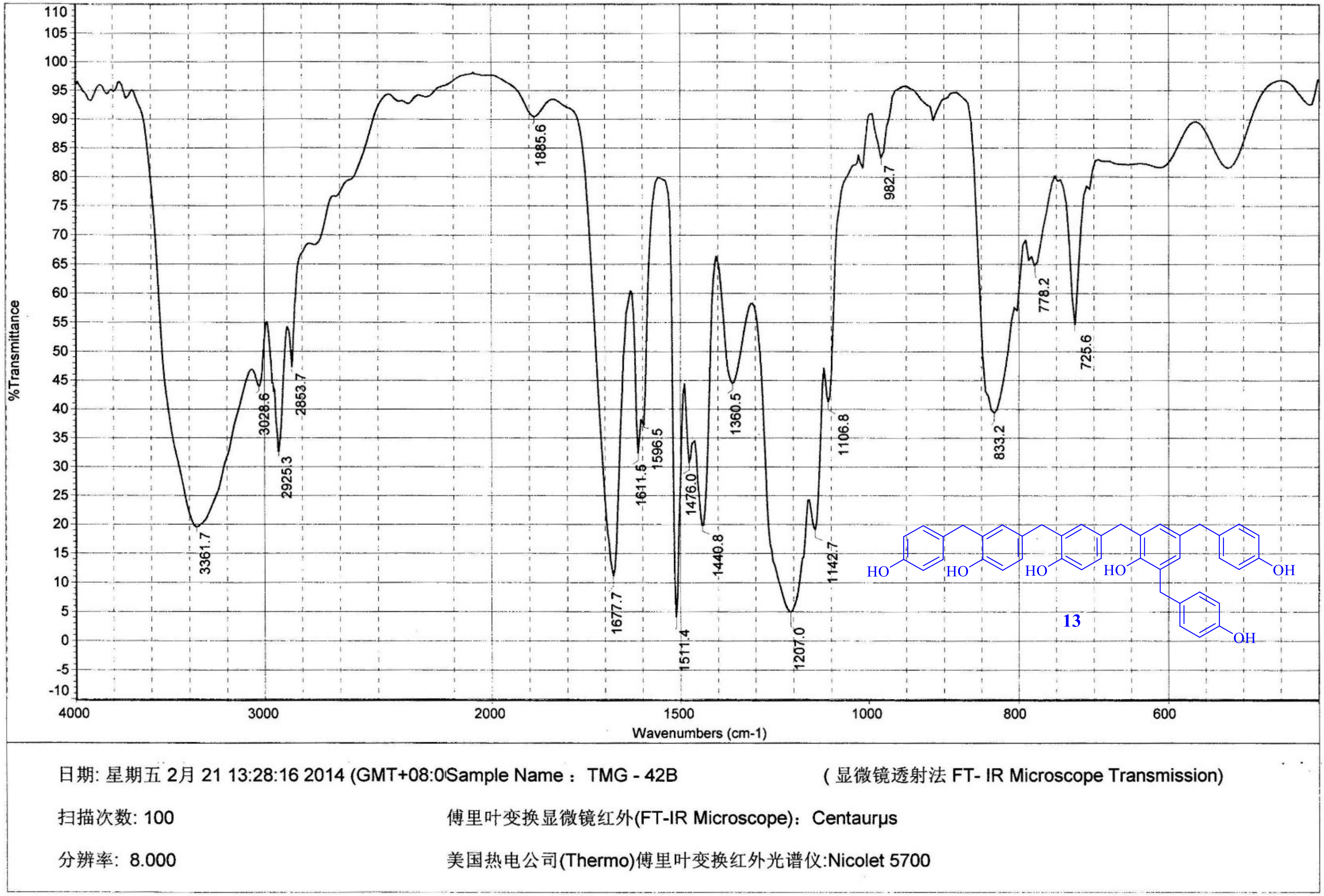

Figure S163. The IR spectrum of compound **13**

Single Mass Spectrum Deconvolution Report

Analysis Name: guoql121.d **Instrument:** LC-MSD-Trap-SL **Print Date:** 8/28/2012 1:20:56 PM
Method: standby.m **Operator:** Operator **Acq. Date:** 8/28/2012 1:11:39 PM
Sample Name: TMG-44B
Analysis Info:

Acquisition Parameter:

Mass Range Mode	Std/Normal	Trap Drive	45.5	Scan Begin	100 m/z
Ion Polarity	Positive	Octopole RF Amplitude	152.8 Vpp	Scan End	800 m/z
Ion Source Type	ESI	Capillary Exit	-102.3 Volt	Averages	5 Spectra
Dry Temp (Set)	325 °C	Skimmer	-40.0 Volt	Max. Accu Time	200000 µs
Nebulizer (Set)	15.00 psi	Oct 1 DC	-12.00 Volt	ICC Target	50000
Dry Gas (Set)	5.00 l/min	Oct 2 DC	-1.70 Volt	Charge Control	on

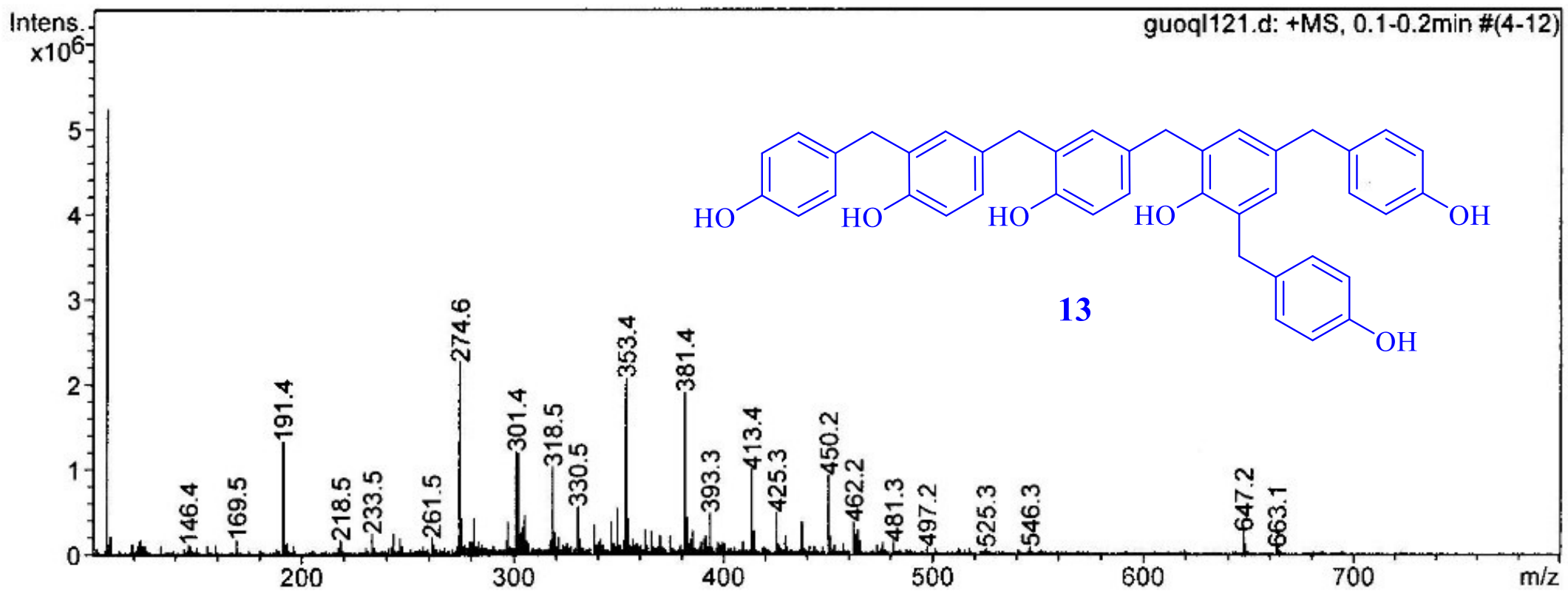

Component	Molecular Mass	Molecule	Absolute Abundance	Relative Abundance

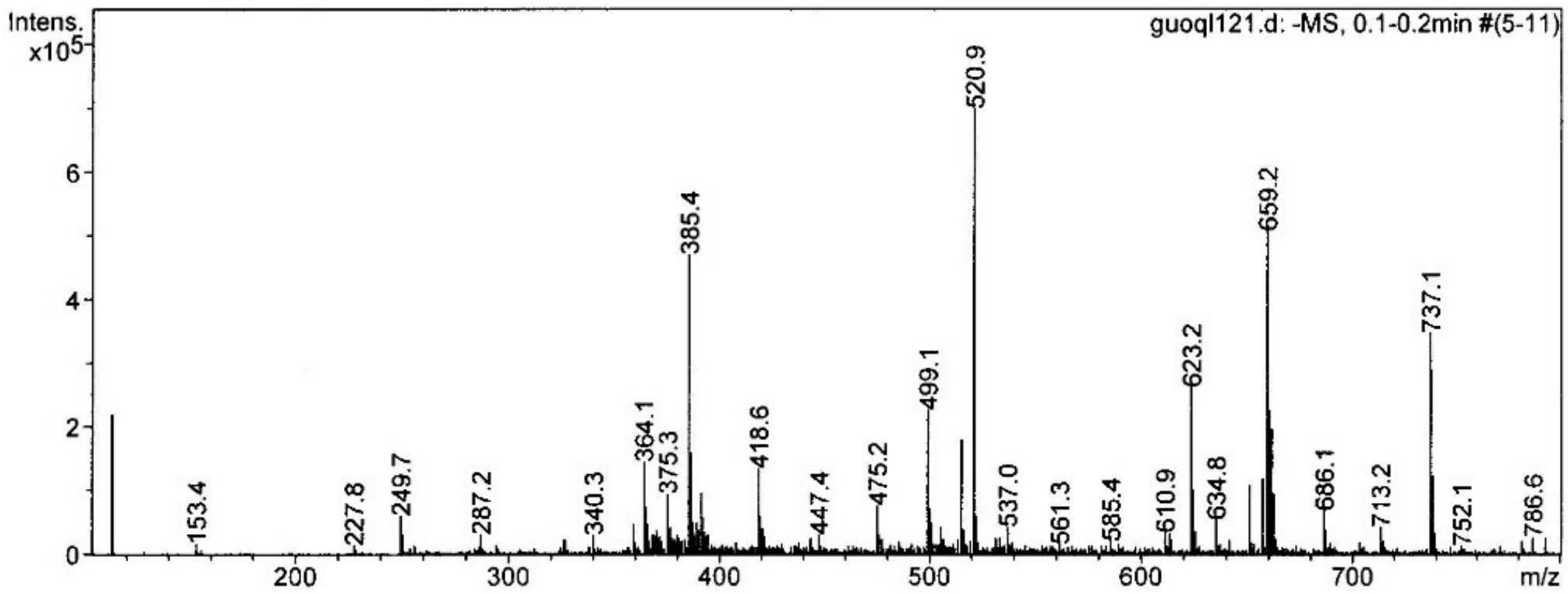

Component	Molecular Mass	Molecule	Absolute Abundance	Relative Abundance

Figure S164. The ESIMS of compound **13**

Qualitative Analysis Report

**Data Filename**	2013030101.d	**Sample Name**	TMG-44B
**Sample Type**	Sample	**Position**	P1-D1
**Instrument Name**	Instrument 1	**User Name**	
**Acq Method**		**IRM Calibration Status**	Success
**DA Method**	TEST LCMS.m	**Comment**	

User Chromatograms

Fragmentor Voltage 135 **Collision Energy** 0 **Ionization Mode** ESI

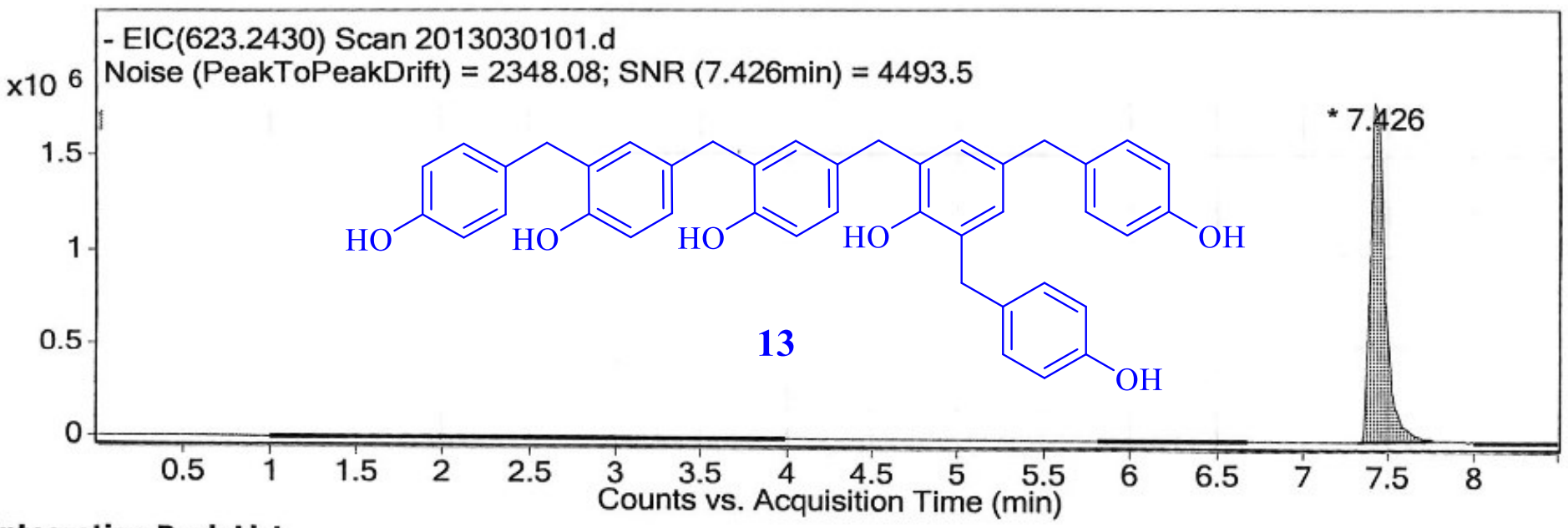

Integration Peak List

Peak	Start	RT	End	Height	Area	Area %	Signal To Noise
1	7.313	7.426	7.764	1765091	10551037	100	4493.5

Noise Measurements

Noise Type	Signal Definition	Noise Multiplier	Noise Value
Peak-to-Peak from Drift	Area	1	2348.079834

Noise Regions

Start	End
1	4
5.8	6.7
8	9

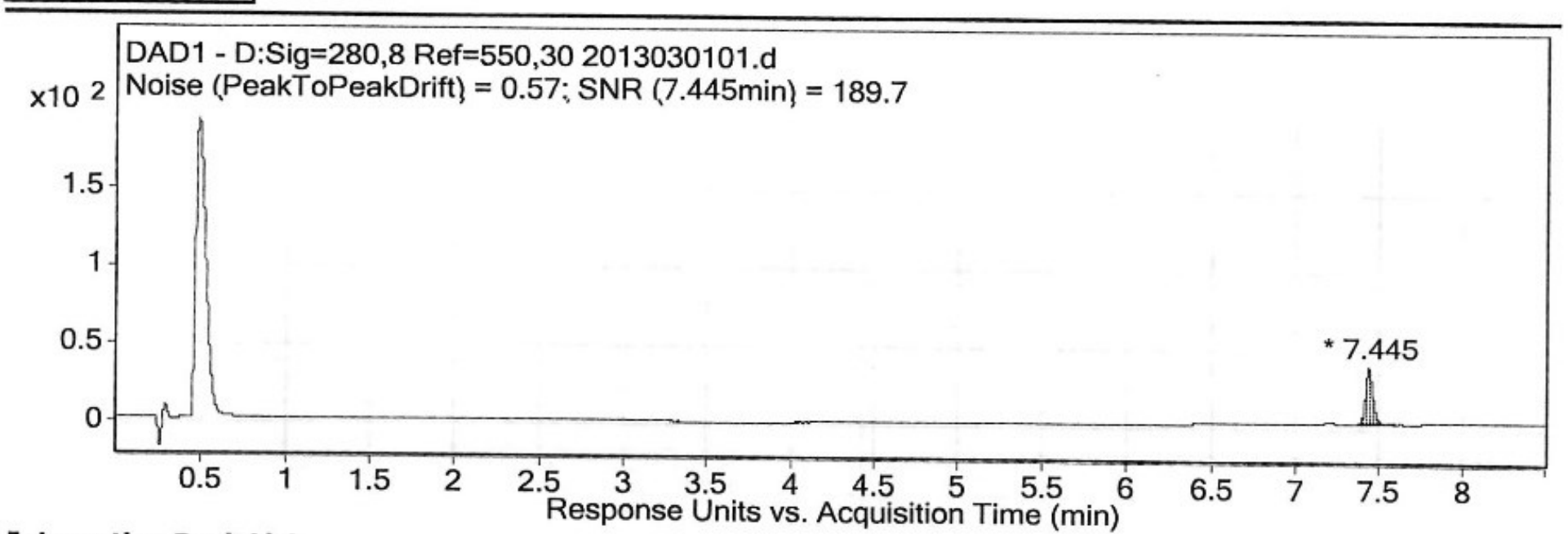

Integration Peak List

Peak	Start	RT	End	Height	Area	Area %	Signal To Noise
1	7.367	7.445	7.598	35.61	108.22	100	189.7

Noise Measurements

Noise Type	Signal Definition	Noise Multiplier	Noise Value
Peak-to-Peak from Drift	Area	1	0.570535064

Noise Regions

Start	End
1	4
5.8	6.7
8	9

User Spectra

Figure S165. The HR-ESIMS report of compound **13** (page 1)

Qualitative Analysis Report

Fragmentor Voltage	Collision Energy	Ionization Mode
135	0	Esi

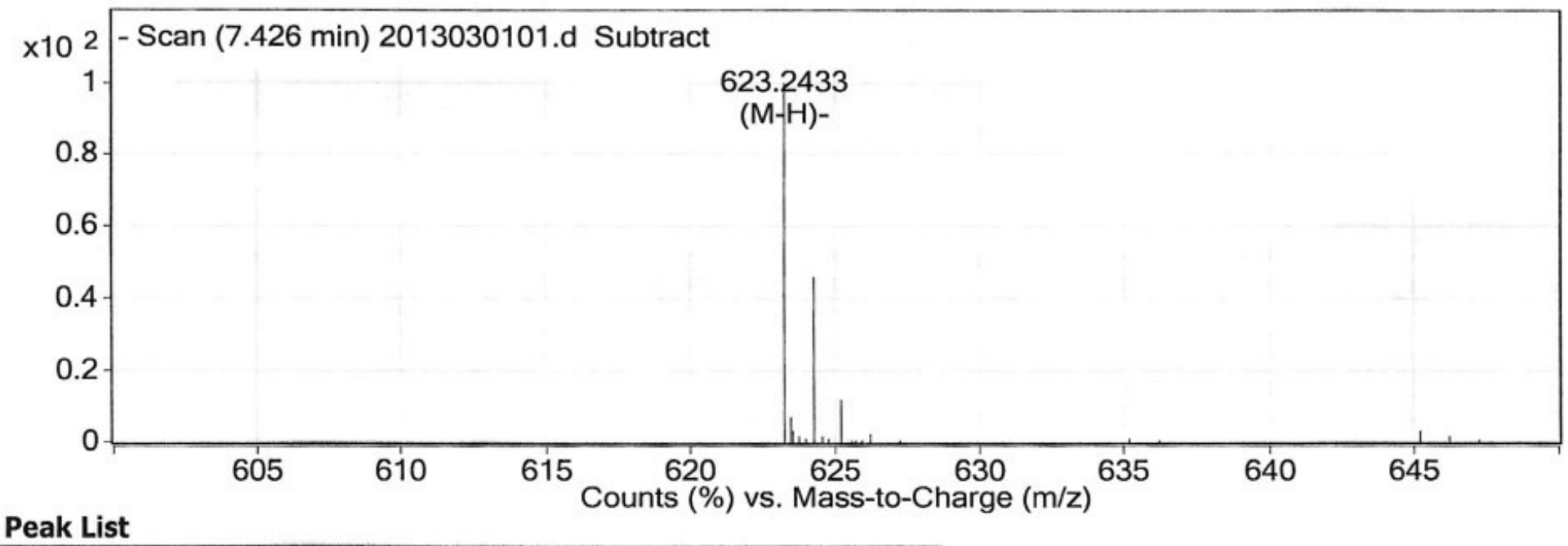

Peak List

m/z	z	Abund	Formula	Ion
248.9597		120396		
623.2433	1	1767662	C41 H35 O6	(M-H)-
623.452		124242		
624.2479	1	816195	C41 H35 O6	(M-H)-
625.2501	1	212192	C41 H35 O6	(M-H)-
691.2299		161018		
737.2363	1	251644		
1247.4959	1	311355		
1248.4995	1	286493		
1249.5017	1	132129		

Formula Calculator Element Limits

Element	Min	Max
C	3	100
H	0	500
O	0	90
N	0	3
S	0	2
Cl	0	0
Br	0	0
Si	0	0
F	0	0
P	0	0

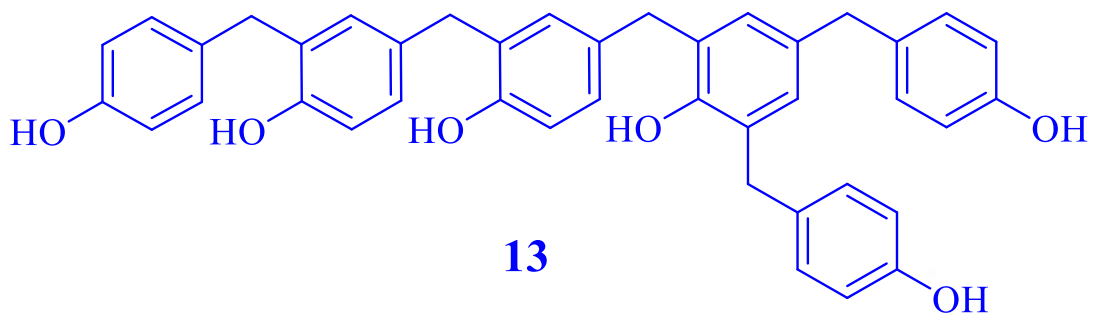

13

Formula Calculator Results

Formula	Best	Mass	Tgt Mass	Diff (ppm)	Ion Species	Score
C41 H36 O6	TRUE	624.2507	624.2512	0.81	C41 H35 O6	99.94
C45 H36 O S		624.2507	624.2487	-3.22	C45 H35 O S	99.23
C36 H36 N2 O8		624.2507	624.2472	-5.65	C36 H35 N2 O8	98.99
C38 H40 O6 S		624.2507	624.2546	6.19	C38 H39 O6 S	98.58
C33 H40 N2 O8 S		624.2507	624.2505	-0.28	C33 H39 N2 O8 S	98.45
C48 H32 O		624.2507	624.2453	-8.6	C48 H31 O	98.35
C47 H32 N2		624.2507	624.2565	9.38	C47 H31 N2	98.14
C42 H40 O S2		624.2507	624.2521	2.17	C42 H39 O S2	98.03

--- End Of Report ---

Figure S166. The HR-ESIMS report of compound **13** (page 2)

MS Formula Results: - Scan (7.426 min) Sub (2013030101.d)

m/z	Ion	Formula	Abundance
623.2433	(M-H)-	C41 H35 O6	1767661.5

Best	Formula (M)	Ion Formula	Calc m/z	Score	Cross S	Mass	Calc Mass	Diff (ppm)	Abs Diff (ppm)	Abund Match	Spacing Mat	Mass Match	m/z	DBE
✓	C41 H36 O6	C41 H35 O6	623.2439	99.94		624.2507	624.2512	0.81	0.81	99.91	99.88	99.98	623.2433	24
	C45 H36 O S	C45 H35 O S	623.2414	99.23		624.2507	624.2487	-3.22	3.22	98.27	99.56	99.63	623.2433	28
	C36 H36 N2 O8	C36 H35 N2 O8	623.2399	98.99		624.2507	624.2472	-5.65	5.65	98.48	99.85	98.87	623.2433	20
	C38 H40 O6 S	C38 H39 O6 S	623.2473	98.58		624.2507	624.2546	6.19	6.19	97.77	99.42	98.65	623.2433	19
	C33 H40 N2 O8 S	C33 H39 N2 O8 S	623.2433	98.45		624.2507	624.2505	-0.28	0.28	95.22	99.24	100	623.2433	15
	C48 H32 O	C48 H31 O	623.238	98.35		624.2507	624.2453	-8.6	8.6	98.63	99.88	97.41	623.2433	33
	C47 H32 N2	C47 H31 N2	623.2493	98.14		624.2507	624.2565	9.38	9.38	98.73	99.87	96.93	623.2433	33
	C42 H40 O S2	C42 H39 O S2	623.2448	98.03		624.2507	624.2521	2.17	2.17	94.2	99.01	99.83	623.2433	23

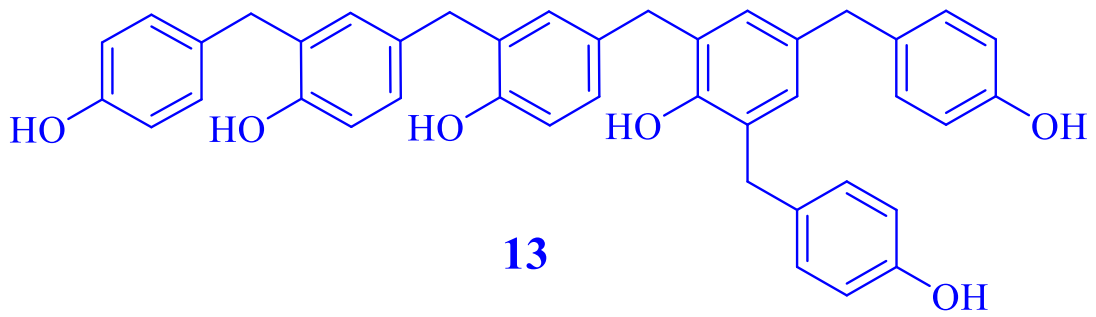

13

page 1

Figure S167. The HR-ESIMS report of compound **13** (page 3)

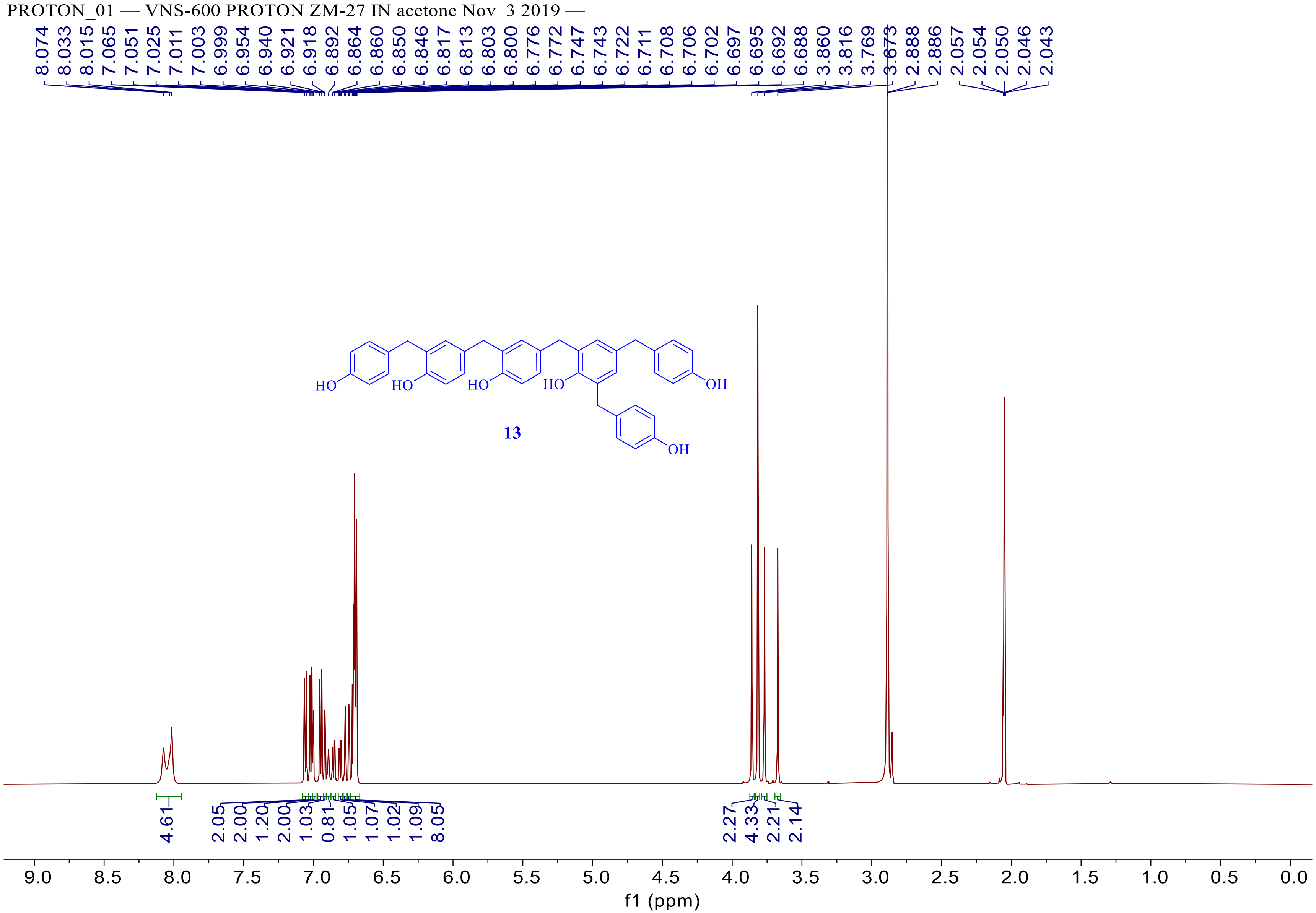

Figure S168. The ^{1}H NMR spectrum of compound **13** in acetone-d_6 (600 MHz)

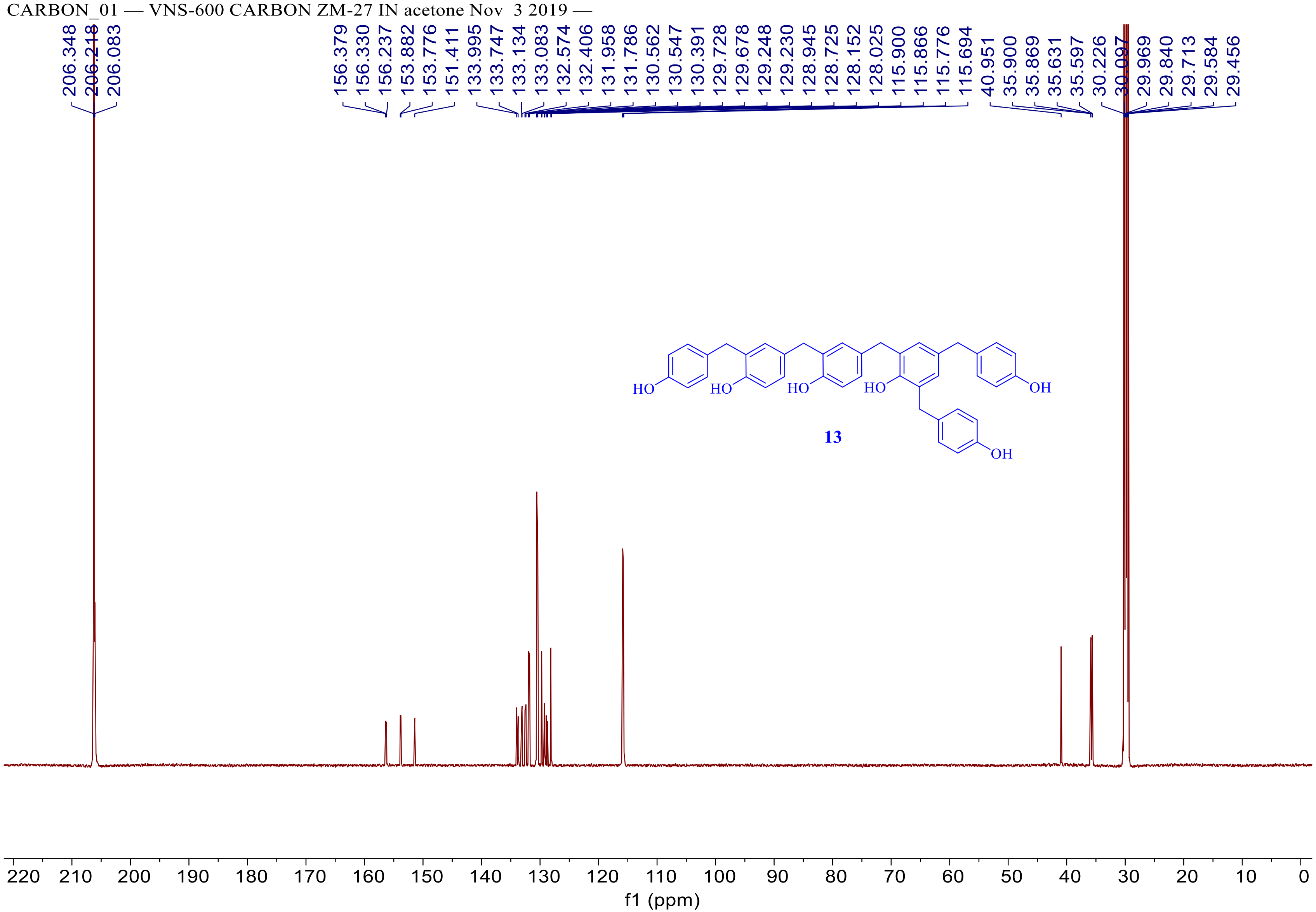

Figure S169. The ^{13}C NMR spectrum of compound **13** in acetone-d_6 (150 MHz)

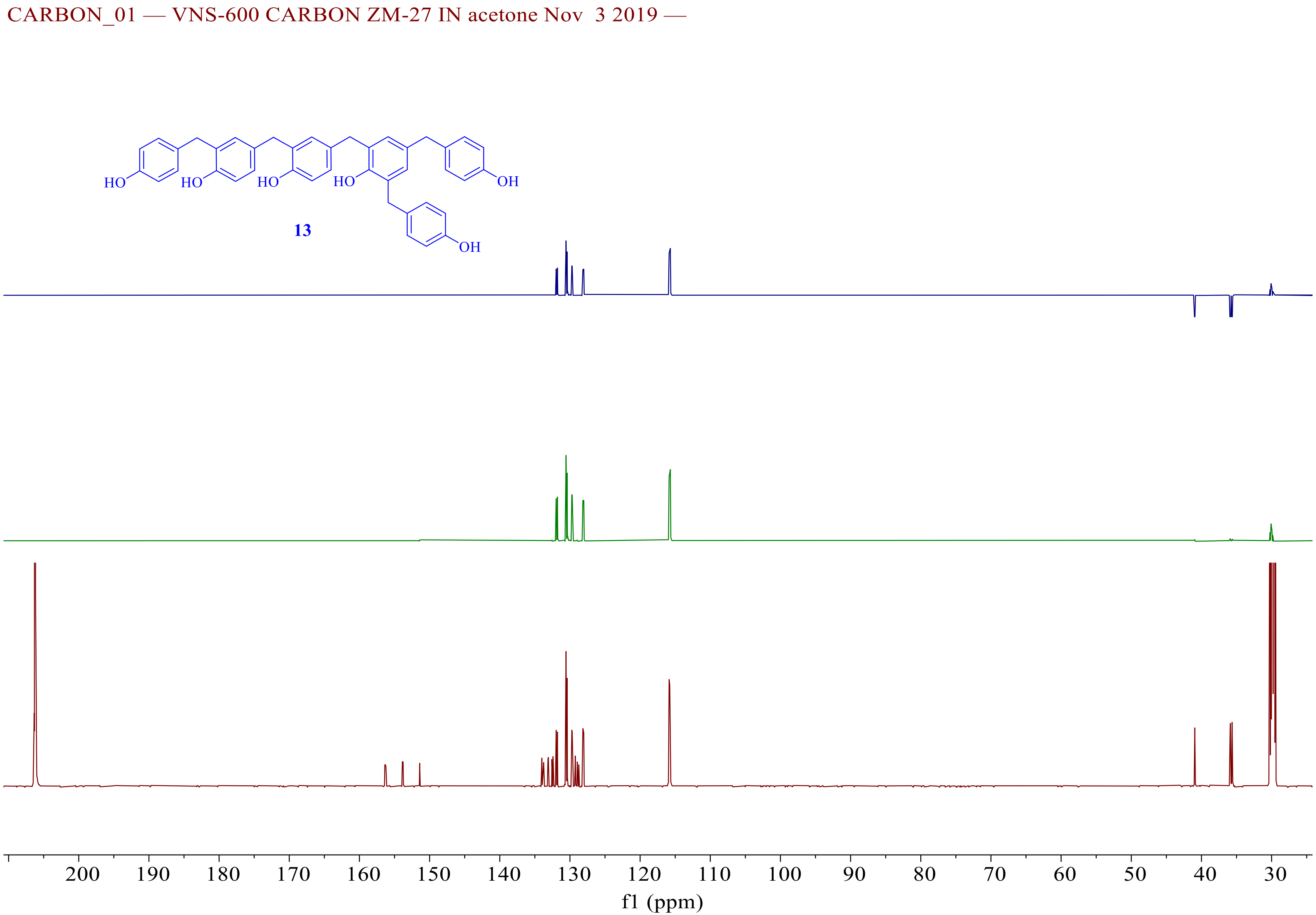

Figure S170. The DEPT spectrum of compound **13** in acetone-d_6 (150 MHz)

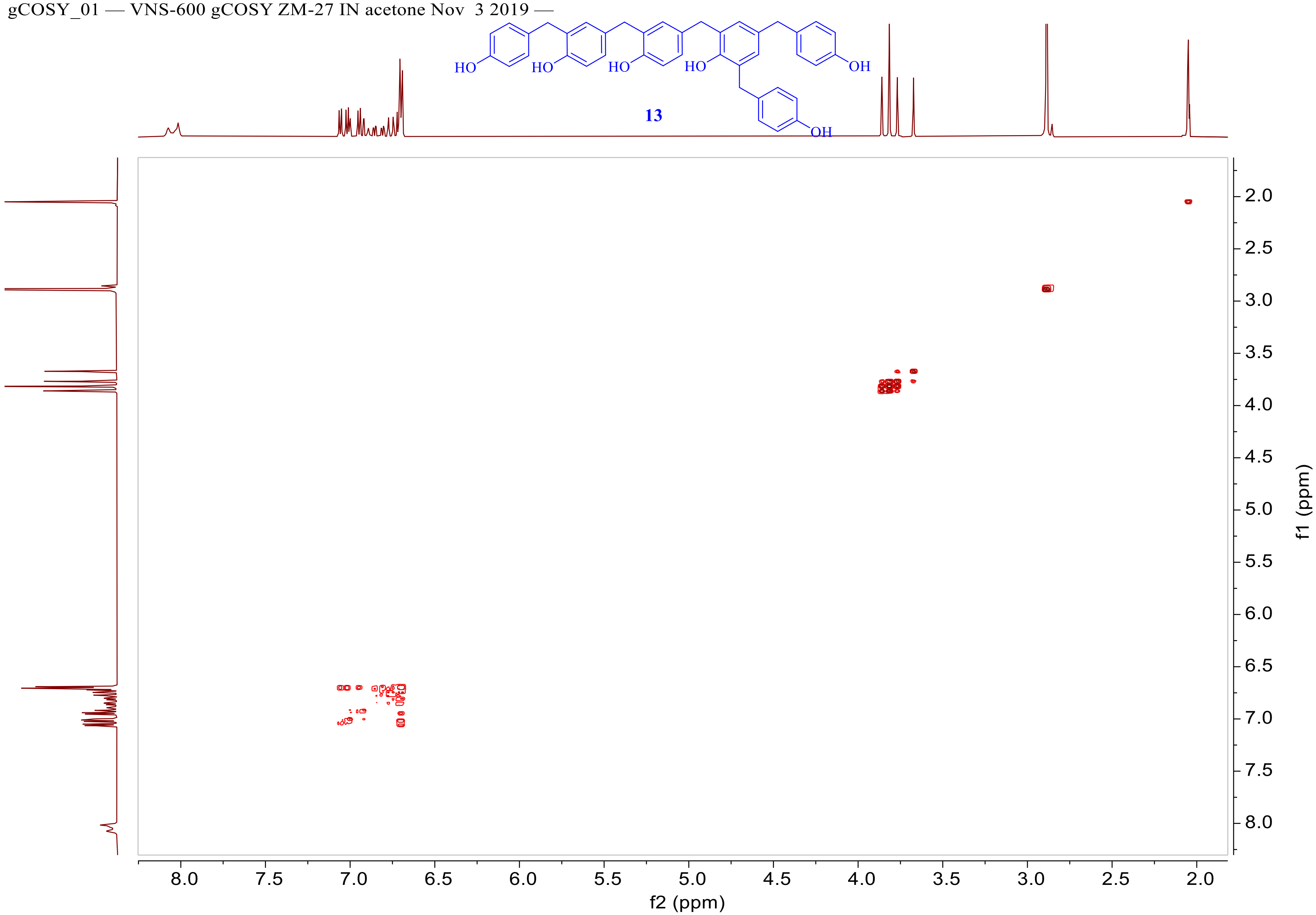

Figure S171. The ^{1}H-^{1}H COSY spectrum of compound **13** in acetone-d_6 (600 MHz)

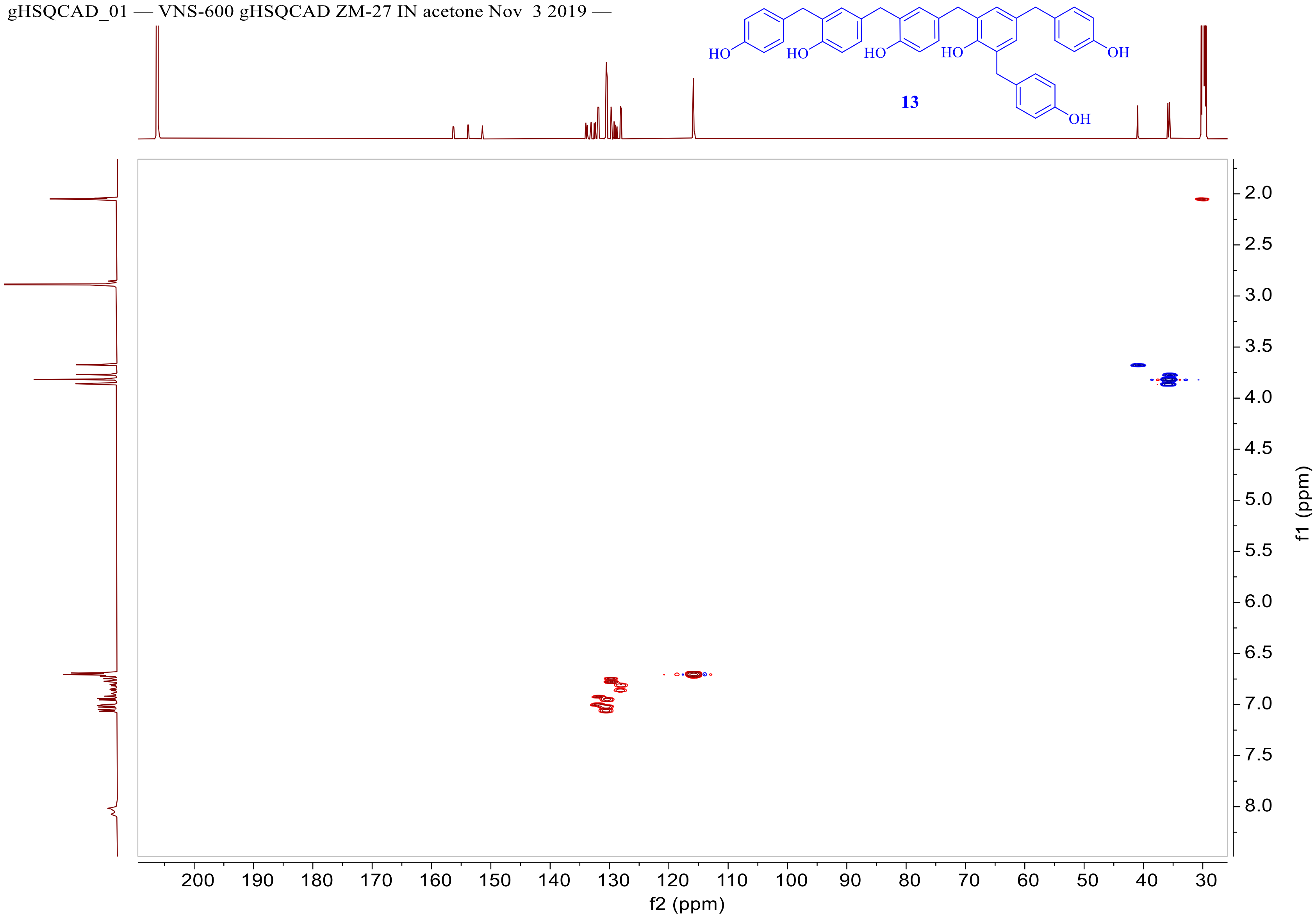

Figure S172. The HSQC spectrum of compound **13** in acetone-d_6 (600 MHz for ^{1}H)

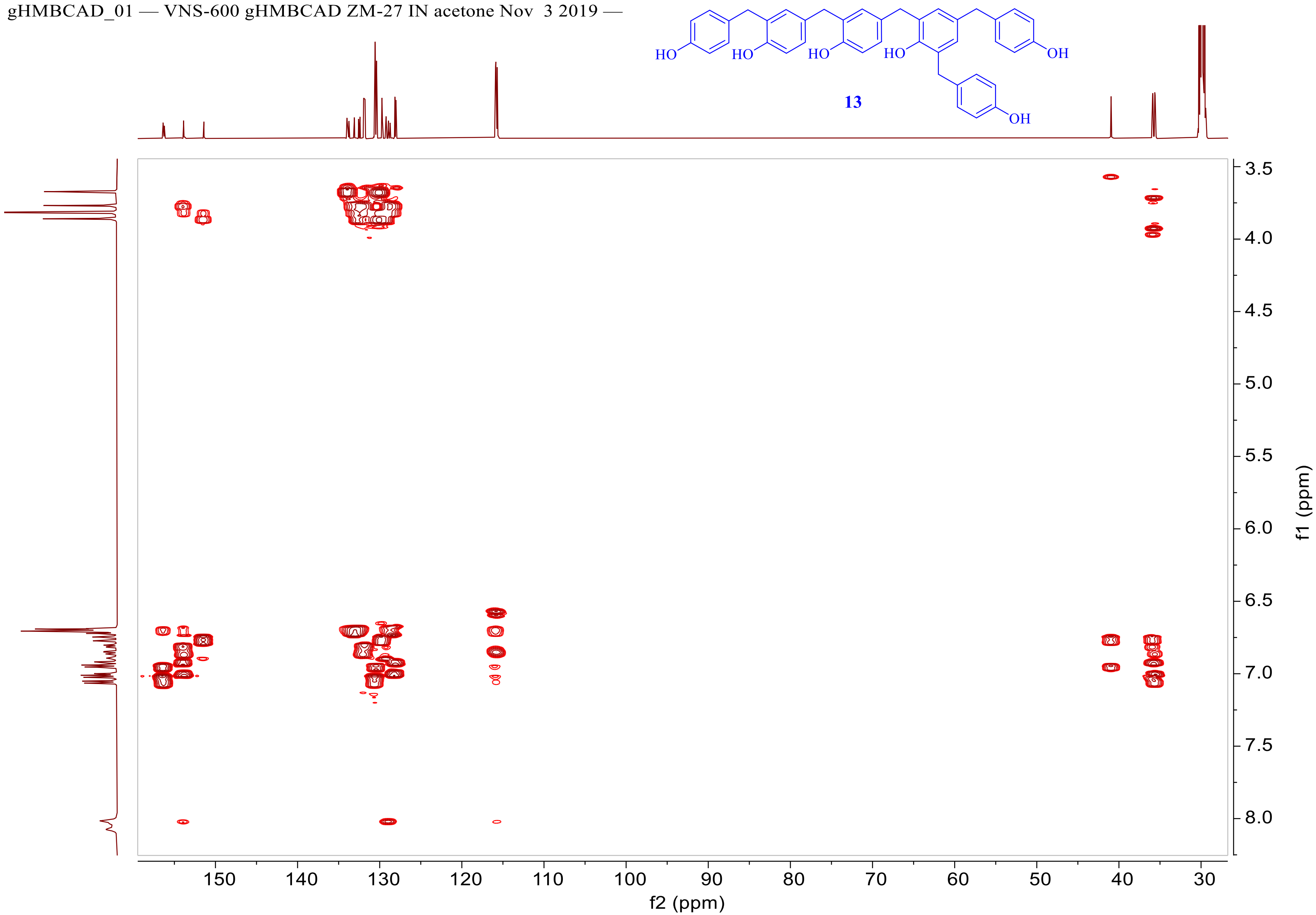

Figure S173. The HMBC spectrum of compound **13** in acetone-d_6 (600 MHz for ^1H)

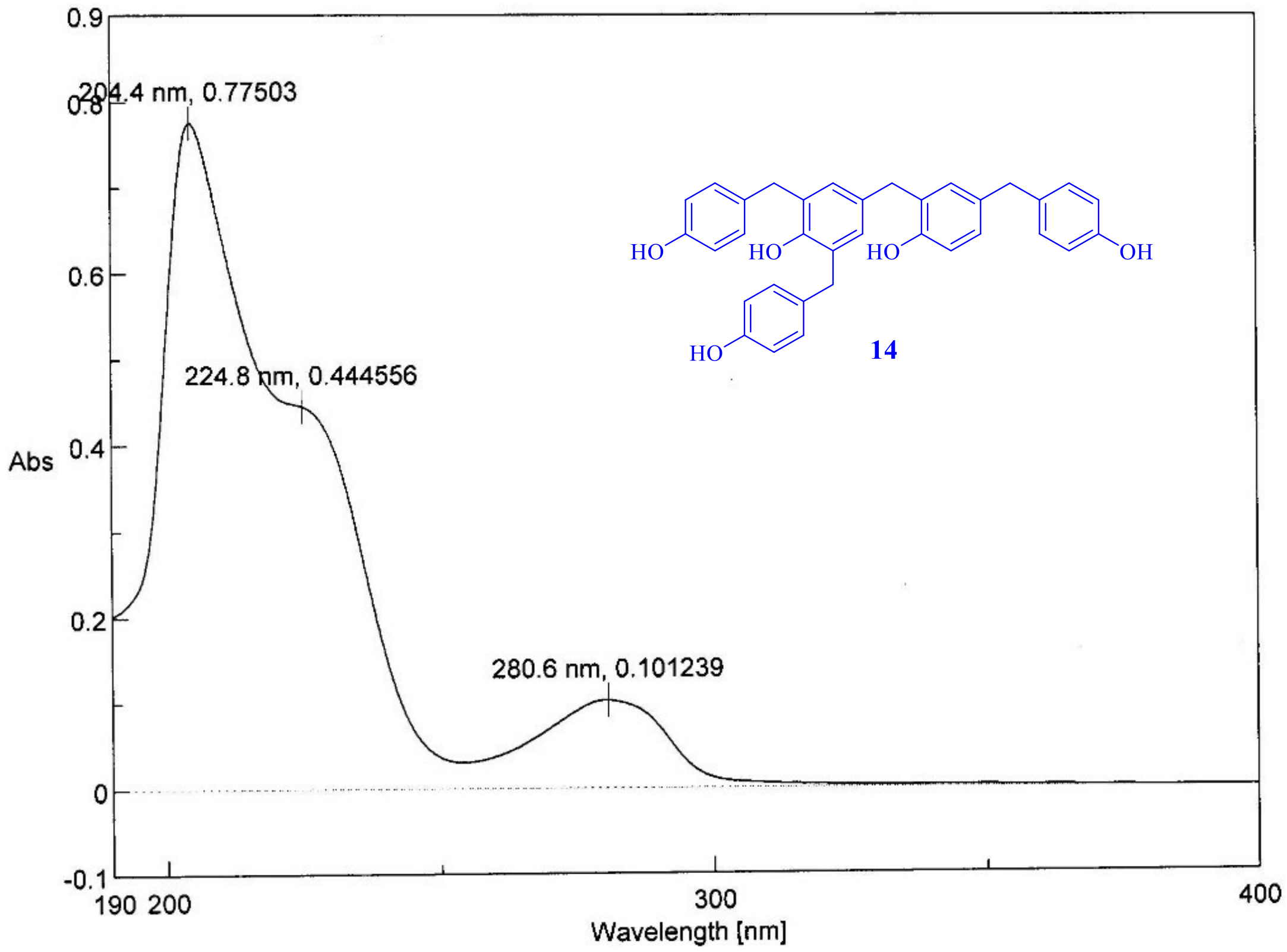

Memory-17

[Comment]	
Sample Name	TMG-39
Comment	0.02
User	
Division	UV
Company	324
[Measurement Information]	
Instrument Name	V-650
Model Name	V-650
Serial No.	A034461150
Accessory	PSC-718
Accessory S/N	A001761114
Position	1
Cell Length	10 mm
Temperature	19.98 C
Control Sonsor	Holder
Monitor Sensor	Holder
Start Mode	Start immediately
Photometric Mode	Abs
Measurement range	400 - 190 nm
Data pitch	0.2 nm
Band width(UV/Vis)	2.0 nm
Response	Medium
Scanning speed	200 nm/min
Source Change	340 nm
Light Source	D2/WI
Filter Exchange	Step
Correction	Baseline

[Data Information]	
Creation Date	2014-2-26 9:42
Data array type	Linear data array
Horizontal	Wavelength [nm]
Vertical	Abs
Start	400 nm
End	190 nm
Data pitch	0.2 nm
Data points	1051

Figure S174. The UV spectrum of compound **14** in MeOH

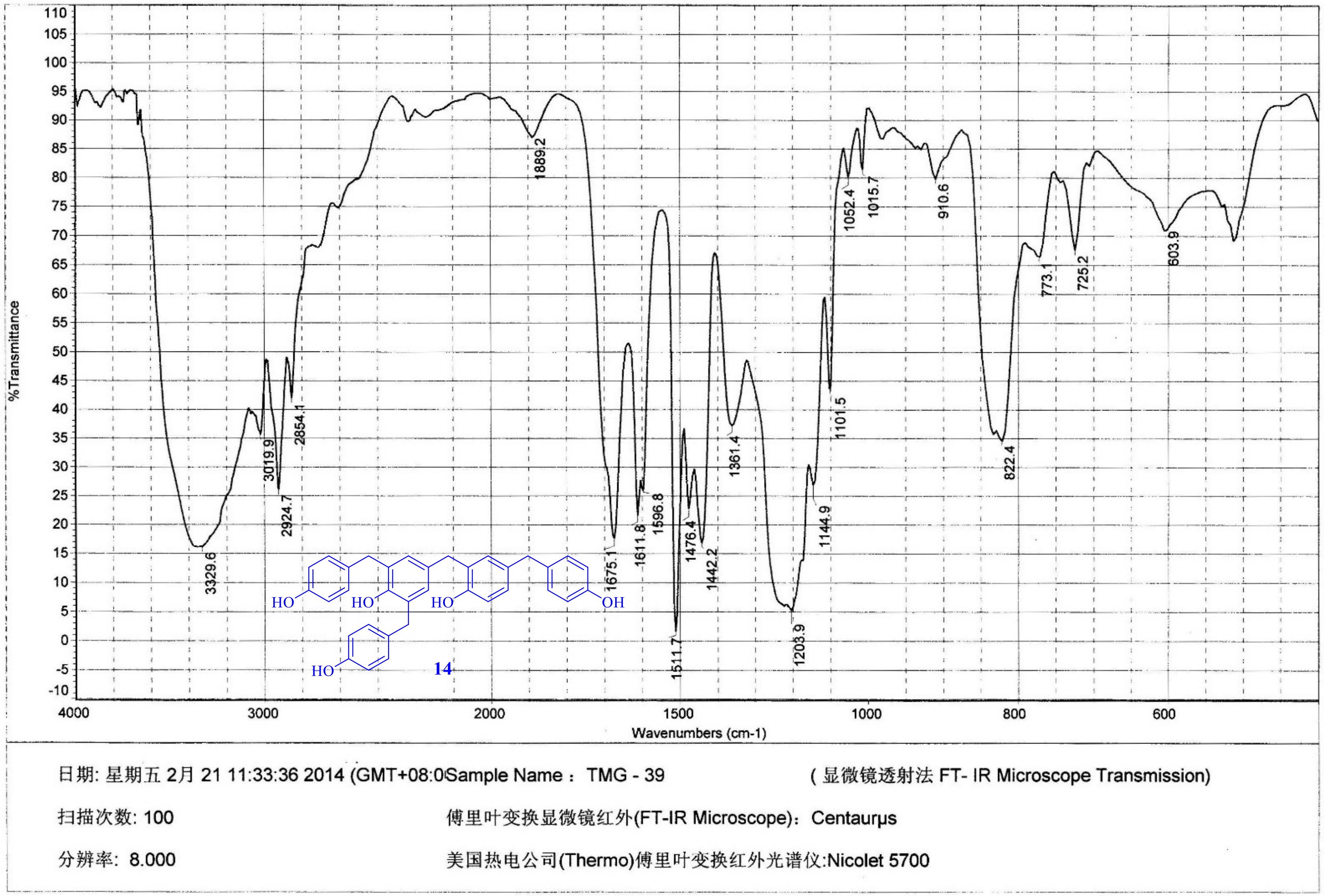

Figure S175. The IR spectrum of compound **14**

Single Mass Spectrum Deconvolution Report

**Analysis Name:**	guoql052.d	**Instrument:**	LC-MSD-Trap-SL	**Print Date:**	7/3/2012 11:16:58 AM
**Method:**	TEST.MS	**Operator:**	Operator	**Acq. Date:**	7/3/2012 11:11:04 AM
**Sample Name:**	TMG-39				
**Analysis Info:**					

Acquisition Parameter:

Mass Range Mode	Std/Normal	Trap Drive	45.5	Scan Begin	100 m/z
Ion Polarity	Positive	Octopole RF Amplitude	152.8 Vpp	Scan End	800 m/z
Ion Source Type	ESI	Capillary Exit	-102.3 Volt	Averages	5 Spectra
Dry Temp (Set)	330 °C	Skimmer	-40.0 Volt	Max. Accu Time	200000 µs
Nebulizer (Set)	15.00 psi	Oct 1 DC	-12.00 Volt	ICC Target	20000
Dry Gas (Set)	5.00 l/min	Oct 2 DC	-1.70 Volt	Charge Control	on

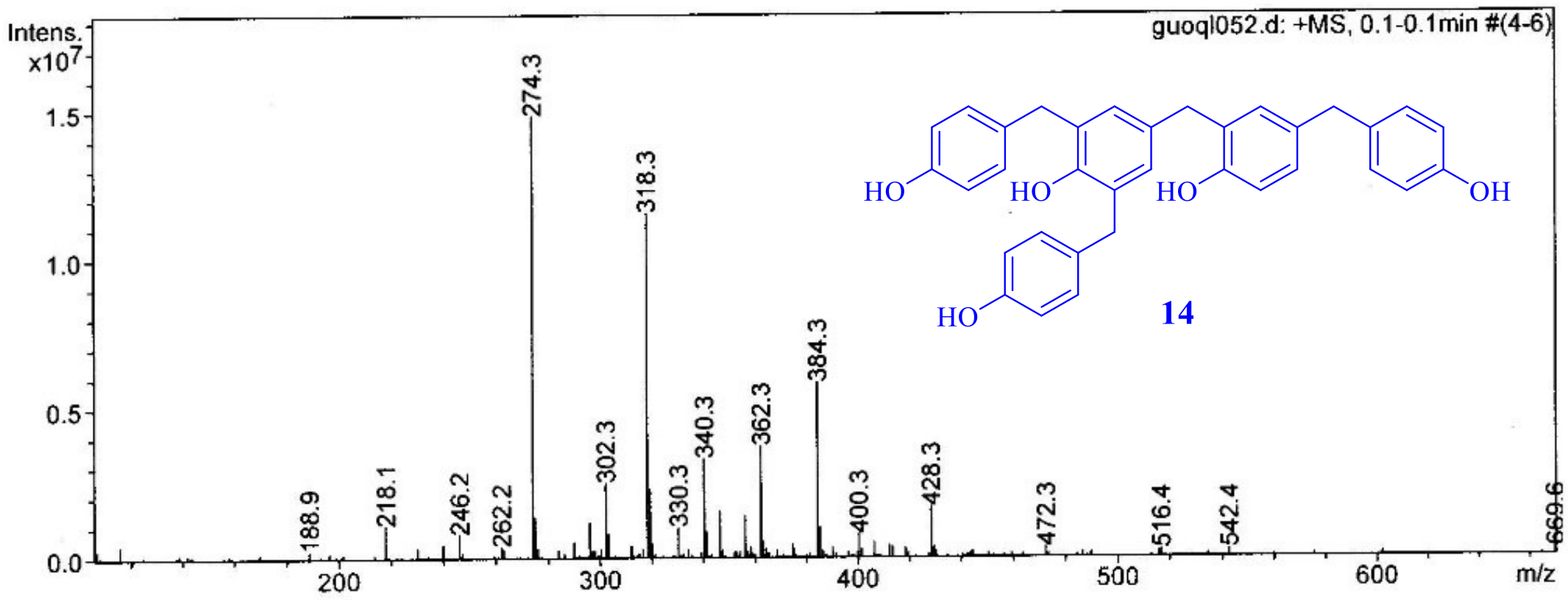

Component	Molecular Mass	Molecule	Absolute Abundance	Relative Abundance

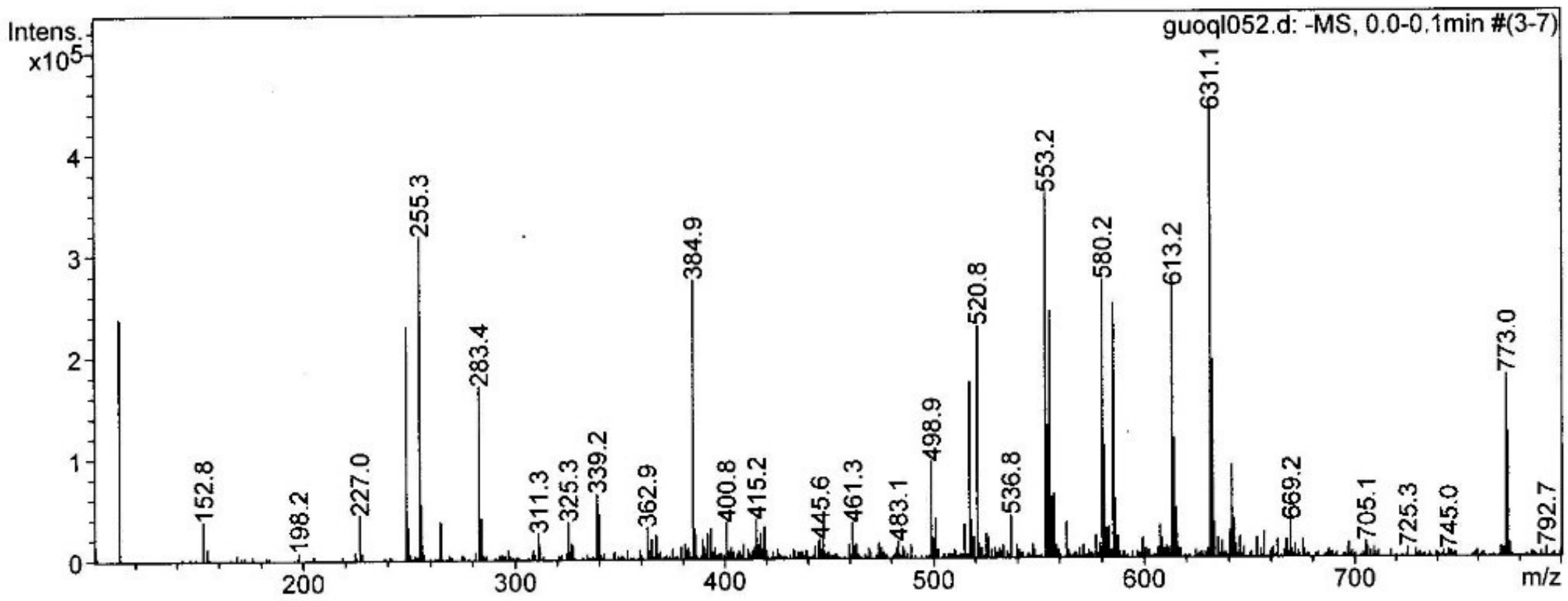

Component	Molecular Mass	Molecule	Absolute Abundance	Relative Abundance

Figure S176. The ESIMS of compound **14**

Qualitative Analysis Report

**Data Filename**	2012061201.d	**Sample Name**	TMG-39
**Sample Type**	Sample	**Position**	P1-D4
**Instrument Name**	Instrument 1	**User Name**	
**Acq Method**		**IRM Calibration Status**	Success
**DA Method**	TEST LCMS.m	**Comment**	

User Chromatograms

Fragmentor Voltage 135 **Collision Energy** 0 **Ionization Mode** ESI

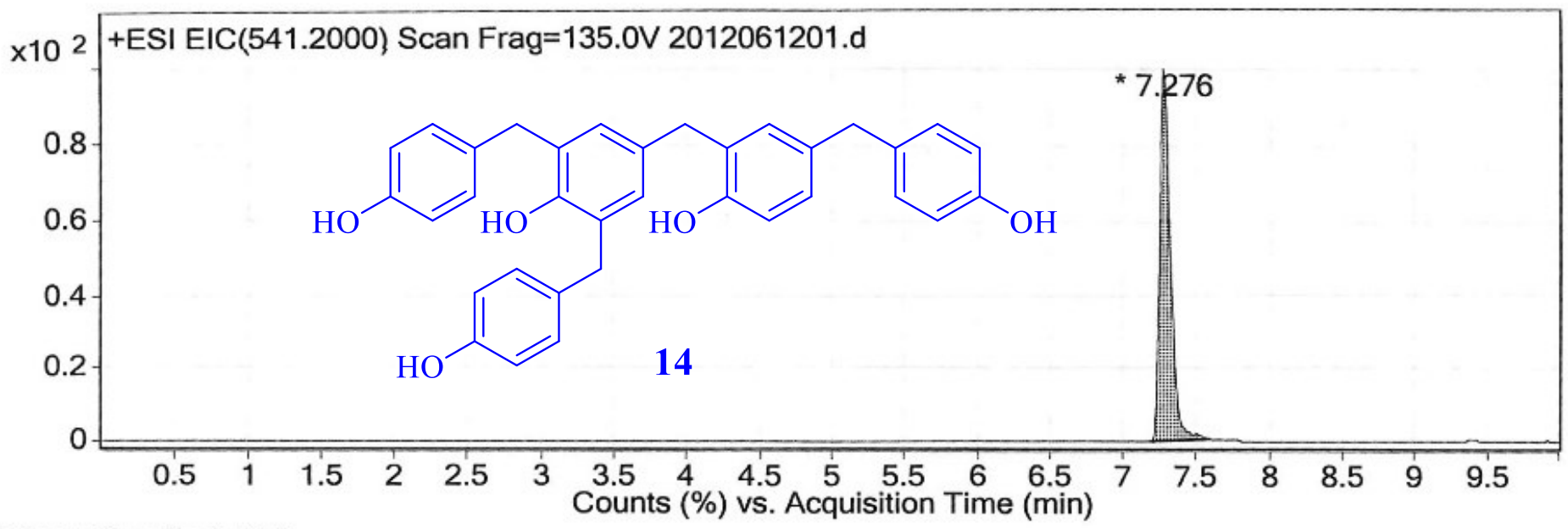

Integration Peak List

Peak	Start	RT	End	Height	Area	Area %
1	7.179	7.276	7.598	397745	2096576	100

User Spectra

Fragmentor Voltage	Collision Energy	Ionization Mode
135	0	Esi

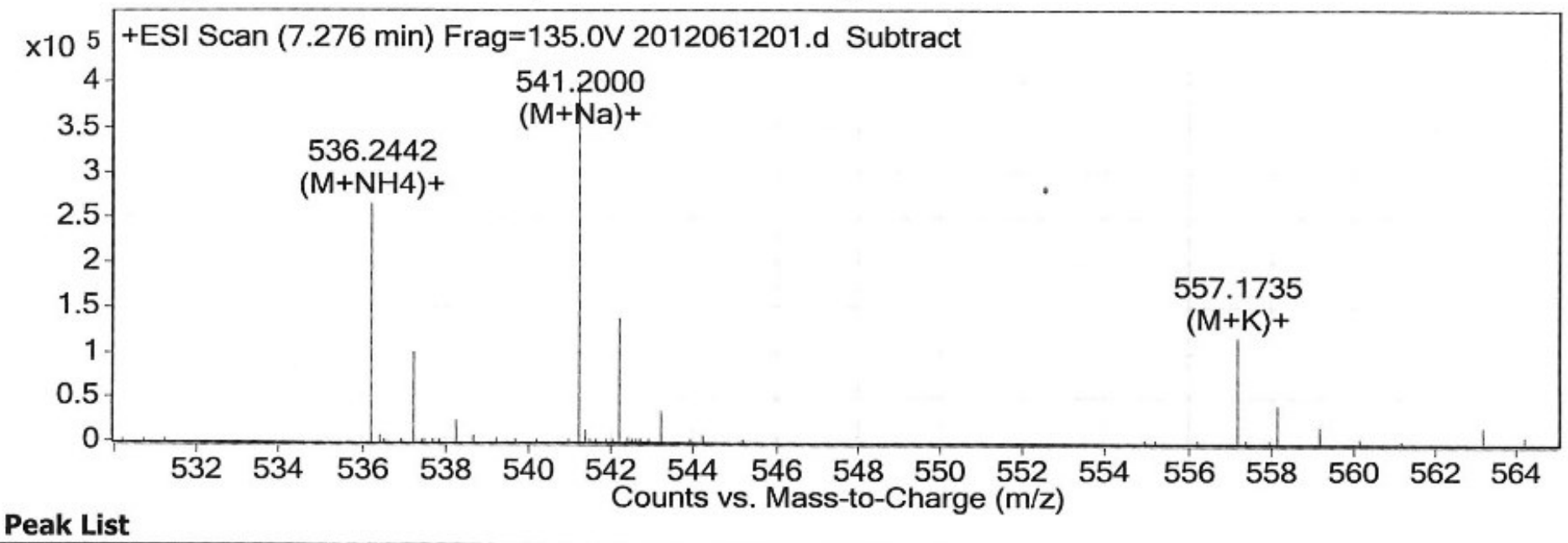

Peak List

m/z	z	Abund	Formula	Ion
104.993		109302		
186.9956		54411		
263.126		46393		
425.1757	1	176293		
426.179	1	52565		
536.2442	1	264700	C34 H34 N O5	(M+NH4)+
537.2471	1	98612	C34 H34 N O5	(M+NH4)+
541.2	1	398406	C34 H30 Na O5	(M+Na)+
542.2032	1	138888	C34 H30 Na O5	(M+Na)+
557.1735	1	116022	C34 H30 K O5	(M+K)+

Formula Calculator Element Limits

Element	Min	Max
C	3	100
H	0	500
O	0	90

Figure S177. The HR-ESIMS report of compound **14** (page 1)

Qualitative Analysis Report

Element	Min	Max
N	0	3
S	0	2
Cl	0	0

Formula Calculator Results

Formula	Best	Mass	Tgt Mass	Diff (ppm)	Ion Species	Score
C34 H30 O5	TRUE	518.2108	518.2093	-2.75	C34 H30 Na O5	99.68
C31 H34 O5 S		518.2108	518.2127	3.74	C31 H34 Na O5 S	99.3
C26 H34 N2 O7 S		518.2108	518.2087	-4.04	C26 H34 N2 Na O7 S	98.79
C27 H34 O10		518.2108	518.2152	8.58	C27 H34 Na O10	98.43
C22 H34 N2 O12		518.2108	518.2112	0.81	C22 H34 N2 Na O12	98.21
C35 H34 S2		518.2108	518.2102	-1.1	C35 H34 Na S2	98.07
C38 H30 S		518.2108	518.2068	-7.59	C38 H30 Na S	98.05
C34 H30 O5	TRUE	518.2103	518.2093	-1.98	C34 H34 N O5	99.22
C31 H34 O5 S		518.2104	518.2127	4.51	C31 H38 N O5 S	99.14
C26 H34 N2 O7 S		518.2104	518.2087	-3.27	C26 H38 N3 O7 S	98.6
C38 H30 S		518.2104	518.2068	-6.82	C38 H34 N S	98.5
C35 H34 S2		518.2104	518.2102	-0.32	C35 H38 N S2	98.41
C34 H30 O5	TRUE	518.2104	518.2093	-1.99	C34 H30 K O5	99.88
C31 H34 O5 S		518.2104	518.2127	4.49	C31 H34 K O5 S	99.04
C26 H34 N2 O7 S		518.2104	518.2087	-3.28	C26 H34 K N2 O7 S	98.56
C38 H30 S		518.2104	518.2068	-6.84	C38 H30 K S	98.46
C29 H30 N2 O7		518.2104	518.2053	-9.77	C29 H30 K N2 O7	98.46
C35 H34 S2		518.2104	518.2102	-0.35	C35 H34 K S2	98.17
C27 H34 O10		518.2104	518.2152	9.34	C27 H34 K O10	98.14

--- End Of Report ---

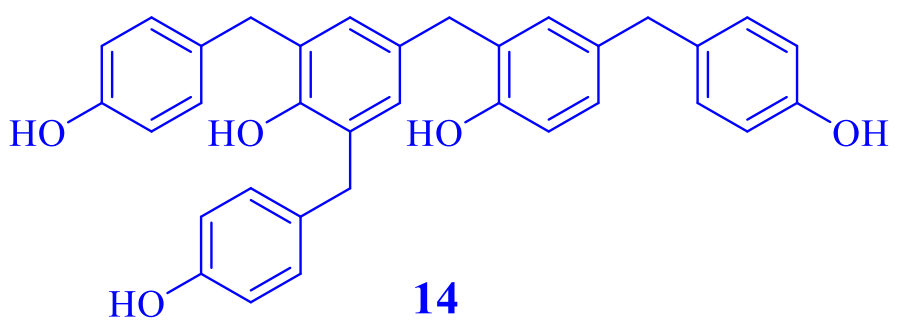

14

Figure S178. The HR-ESIMS report of compound **14** (page 2)

MS Formula Results: + Scan (7.276 min) Sub (2012061201.d)

m/z	Ion	Formula	Abundance
536.2442	(M+NH4)+	C34 H34 N O5	264700.3

Best	Formula (M)	Ion Formula	Calc m/z	Score	Cross S	Mass	Calc Mass	Diff (ppm)	Abs Diff (ppm)	Abund Match	Spacing Mat	Mass Match	m/z	DBE
[x]	C34 H30 O5	C34 H34 N O5	536.2431	99.22		518.2103	518.2093	-1.98	1.98	99.29	97.82	99.87	536.2442	20
[ ]	C31 H34 O5 S	C31 H38 N O5 S	536.2465	99.14		518.2104	518.2127	4.51	4.51	98.53	99.48	99.34	536.2442	15
[ ]	C26 H34 N2 O7 S	C26 H38 N3 O7 S	536.2425	98.6		518.2104	518.2087	-3.27	3.27	95.98	99.65	99.66	536.2442	11
[ ]	C38 H30 S	C38 H34 N S	536.2406	98.5		518.2104	518.2068	-6.82	6.82	97.86	99.26	98.51	536.2442	24
[ ]	C35 H34 S2	C35 H38 N S2	536.244	98.41		518.2104	518.2102	-0.32	0.32	94.64	99.78	100	536.2442	19

m/z	Ion	Formula	Abundance
541.2	(M+Na)+	C34 H30 Na O5	398406.4

Best	Formula (M)	Ion Formula	Calc m/z	Score	Cross S	Mass	Calc Mass	Diff (ppm)	Abs Diff (ppm)	Abund Match	Spacing Mat	Mass Match	m/z	DBE
[x]	C34 H30 O5	C34 H30 Na O5	541.1985	99.68		518.2108	518.2093	-2.75	2.75	99.3	99.96	99.76	541.2	20
[ ]	C31 H34 O5 S	C31 H34 Na O5 S	541.2019	99.3		518.2108	518.2127	3.74	3.74	99.03	99.12	99.56	541.2	15
[ ]	C26 H34 N2 O7 S	C26 H34 N2 Na O7 S	541.1979	98.79		518.2108	518.2087	-4.04	4.04	97.54	98.89	99.48	541.2	11
[ ]	C27 H34 O10	C27 H34 Na O10	541.2044	98.43		518.2108	518.2152	8.58	8.58	98.42	99.92	97.69	541.2	11
[ ]	C22 H34 N2 O12	C22 H34 N2 Na O12	541.2004	98.21		518.2108	518.2112	0.81	0.81	93.86	99.87	99.98	541.2	7
[ ]	C35 H34 S2	C35 H34 Na S2	541.1994	98.07		518.2108	518.2102	-1.1	1.1	94.48	98.61	99.96	541.2	19
[ ]	C38 H30 S	C38 H30 Na S	541.196	98.05		518.2108	518.2068	-7.59	7.59	96.73	99.36	98.18	541.2	24

m/z	Ion	Formula	Abundance
557.1735	(M+K)+	C34 H30 K O5	116021.6

Best	Formula (M)	Ion Formula	Calc m/z	Score	Cross S	Mass	Calc Mass	Diff (ppm)	Abs Diff (ppm)	Abund Match	Spacing Mat	Mass Match	m/z	DBE
[x]	C34 H30 O5	C34 H30 K O5	557.1725	99.88		518.2104	518.2093	-1.99	1.99	99.84	99.92	99.88	557.1735	20
[ ]	C31 H34 O5 S	C31 H34 K O5 S	557.1759	99.04		518.2104	518.2127	4.49	4.49	97.92	99.69	99.39	557.1735	15
[ ]	C26 H34 N2 O7 S	C26 H34 K N2 O7 S	557.1718	98.56		518.2104	518.2087	-3.28	3.28	95.85	99.6	99.68	557.1735	11
[ ]	C38 H30 S	C38 H30 K S	557.17	98.46		518.2104	518.2068	-6.84	6.84	97.09	99.81	98.6	557.1735	24
[ ]	C29 H30 N2 O7	C29 H30 K N2 O7	557.1685	98.46		518.2104	518.2053	-9.77	9.77	99.41	99.9	97.17	557.1735	16
[ ]	C35 H34 S2	C35 H34 K S2	557.1734	98.17		518.2104	518.2102	-0.35	0.35	93.96	99.55	100	557.1735	19
[ ]	C27 H34 O10	C27 H34 K O10	557.1784	98.14		518.2104	518.2152	9.34	9.34	97.92	99.87	97.41	557.1735	11

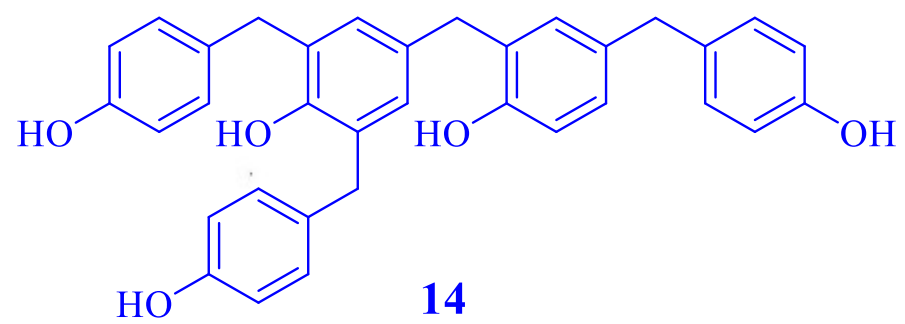

14

page 1

Figure S179. The HR-ESIMS report of compound **14** (page 3)

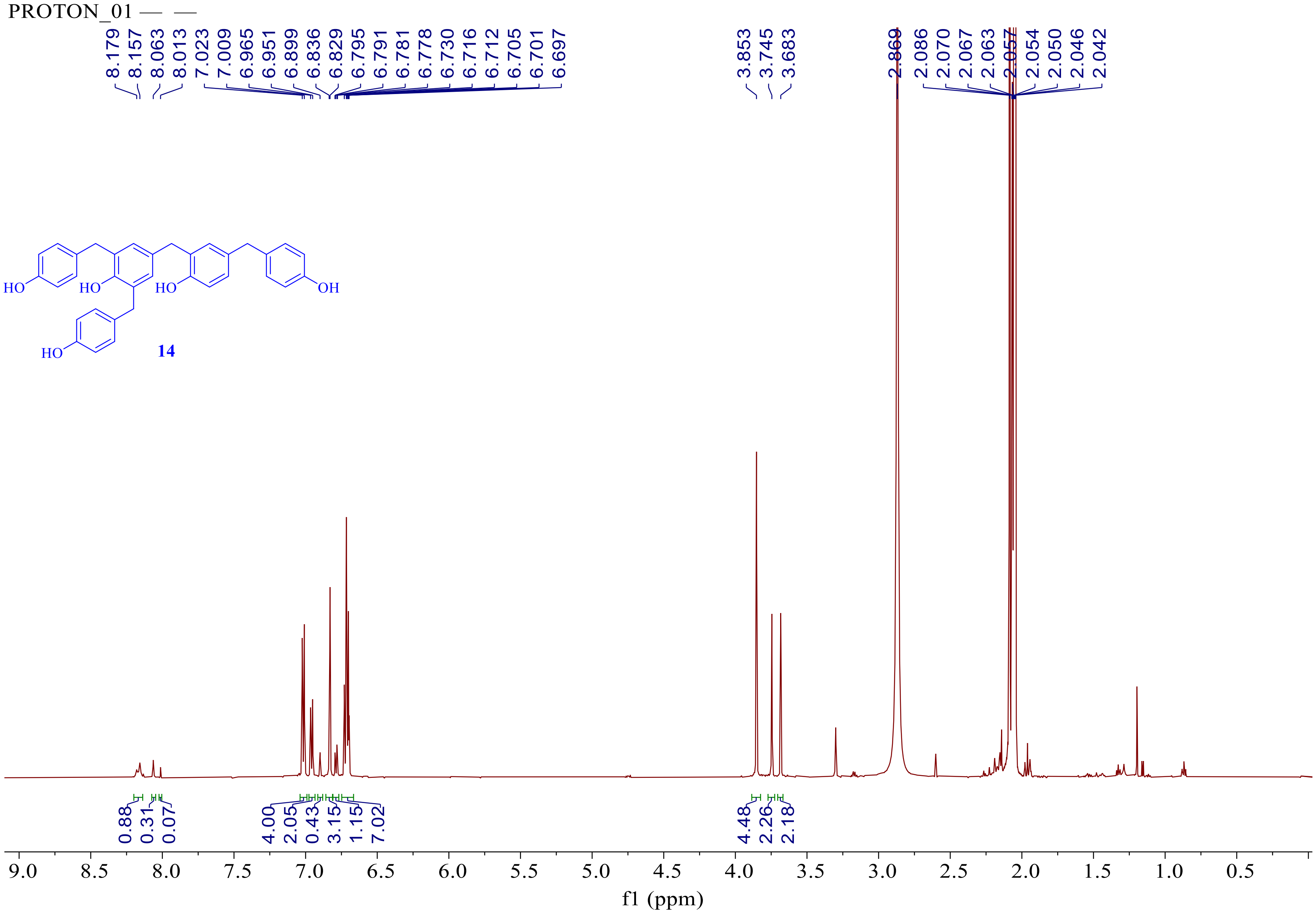

Figure S180. The ^{1}H NMR spectrum of compound **14** in acetone-d_6 (600 MHz)

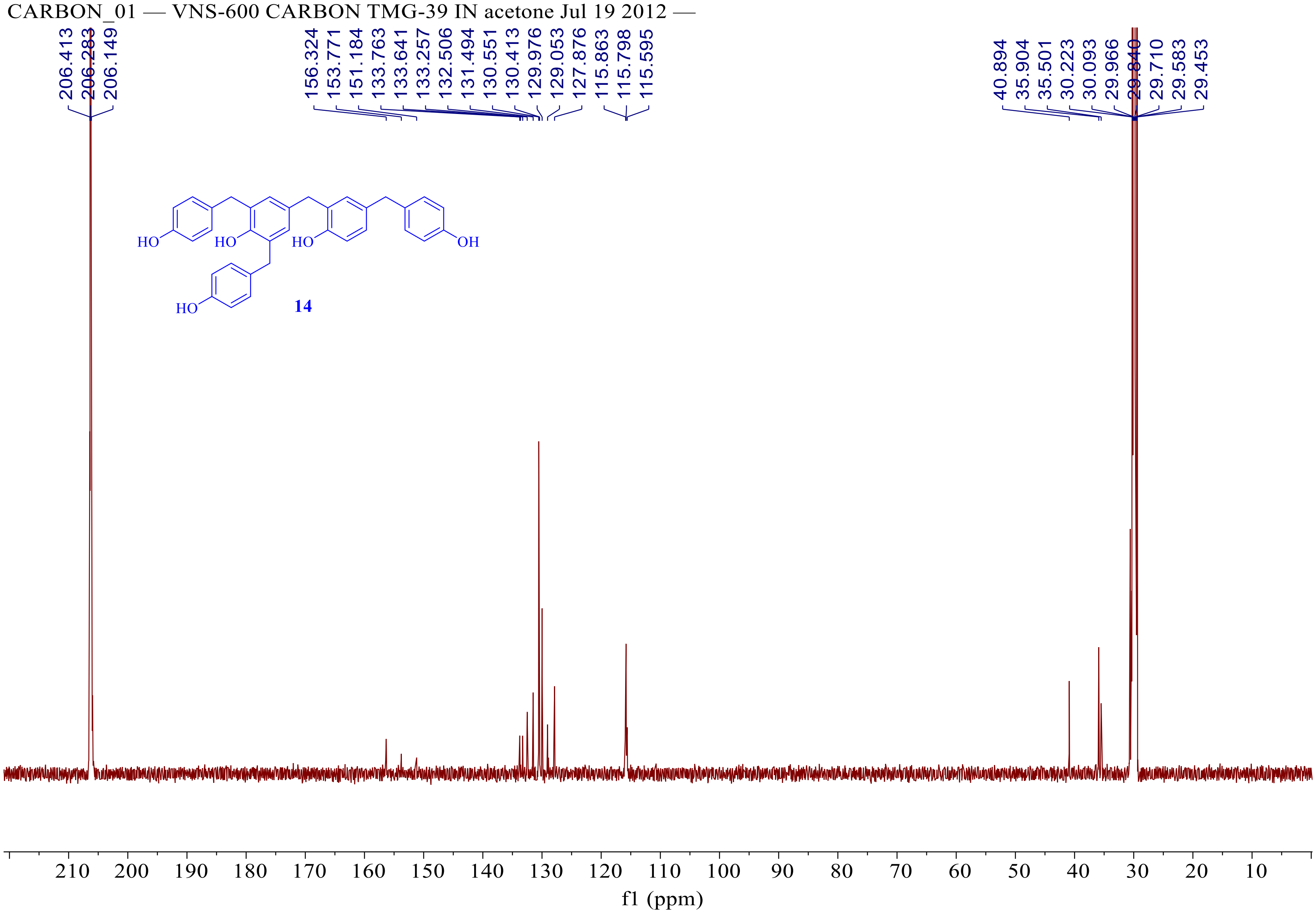

Figure S181. The ^{13}C NMR spectrum of compound **14** in acetone-d_6 (150 MHz)

CARBON_01 — VNS-600 CARBON TMG-39 IN acetone Jul 19 2012 —

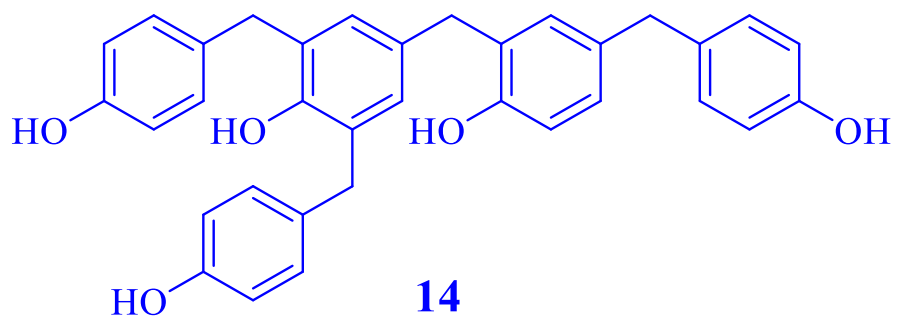

14

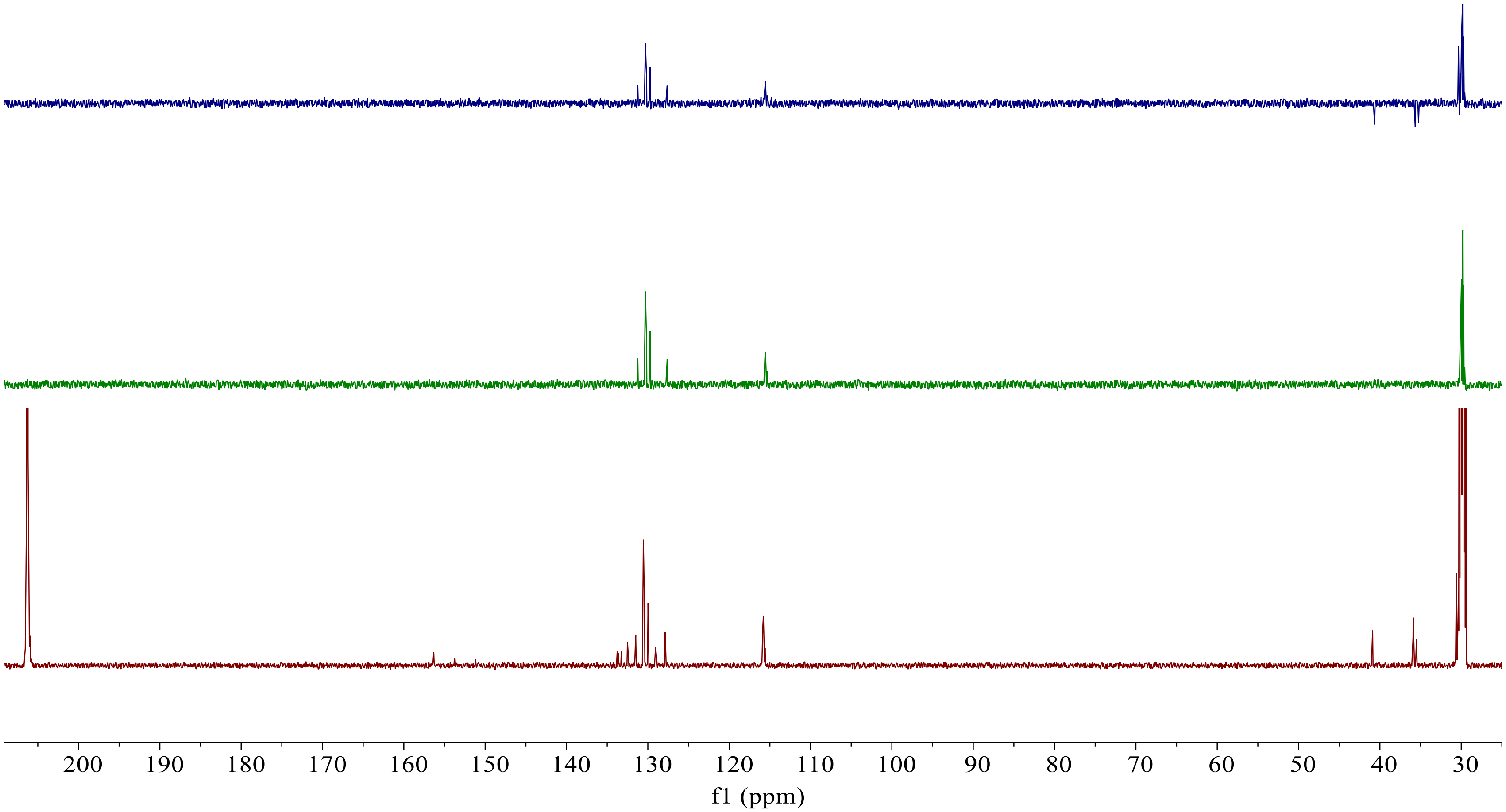

Figure S182. The DEPT spectrum of compound **14** in acetone-d_6 (150 MHz)

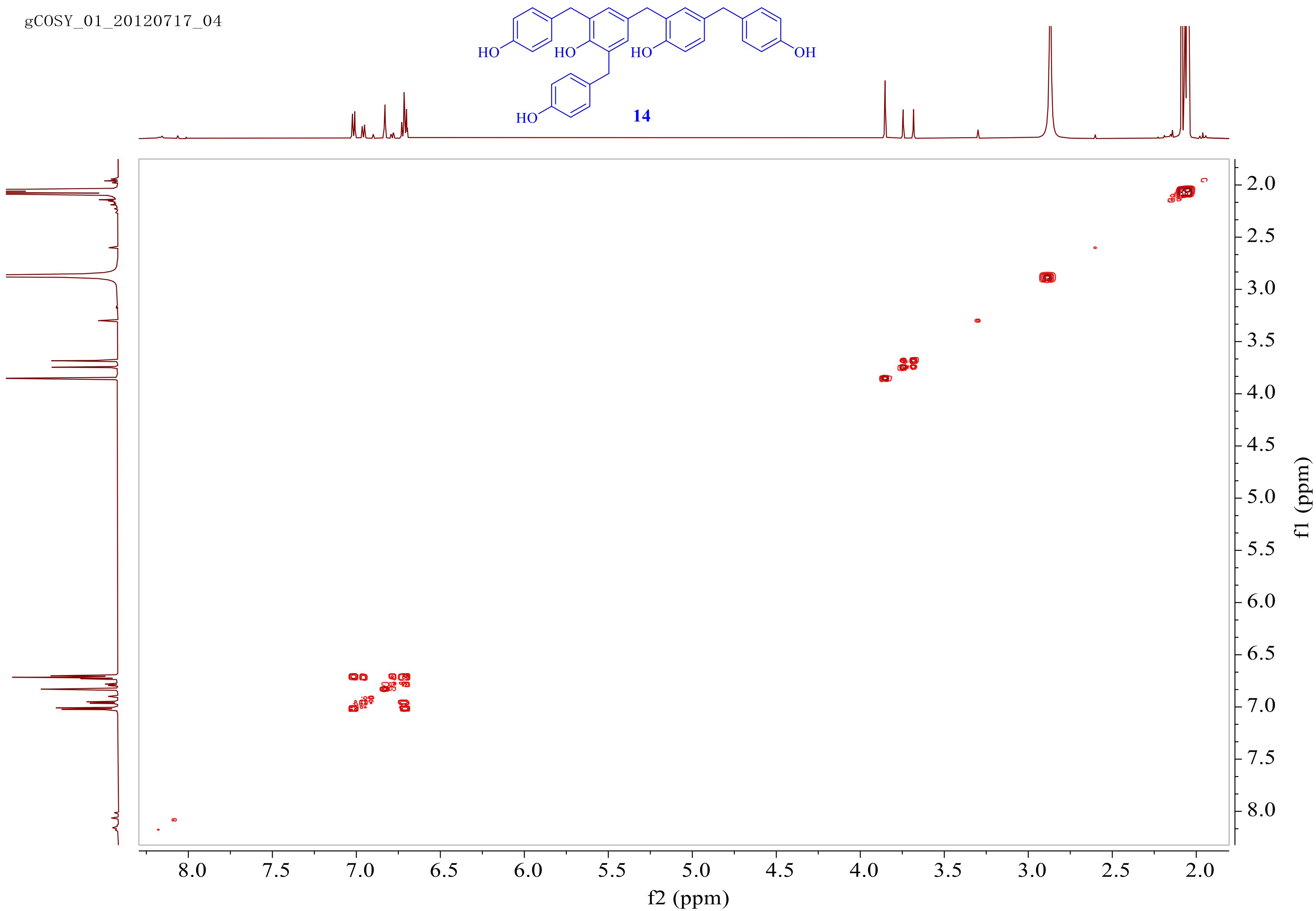

Figure S183. The ^{1}H-^{1}H COSY spectrum of compound **14** in acetone-d_6 (600 MHz)

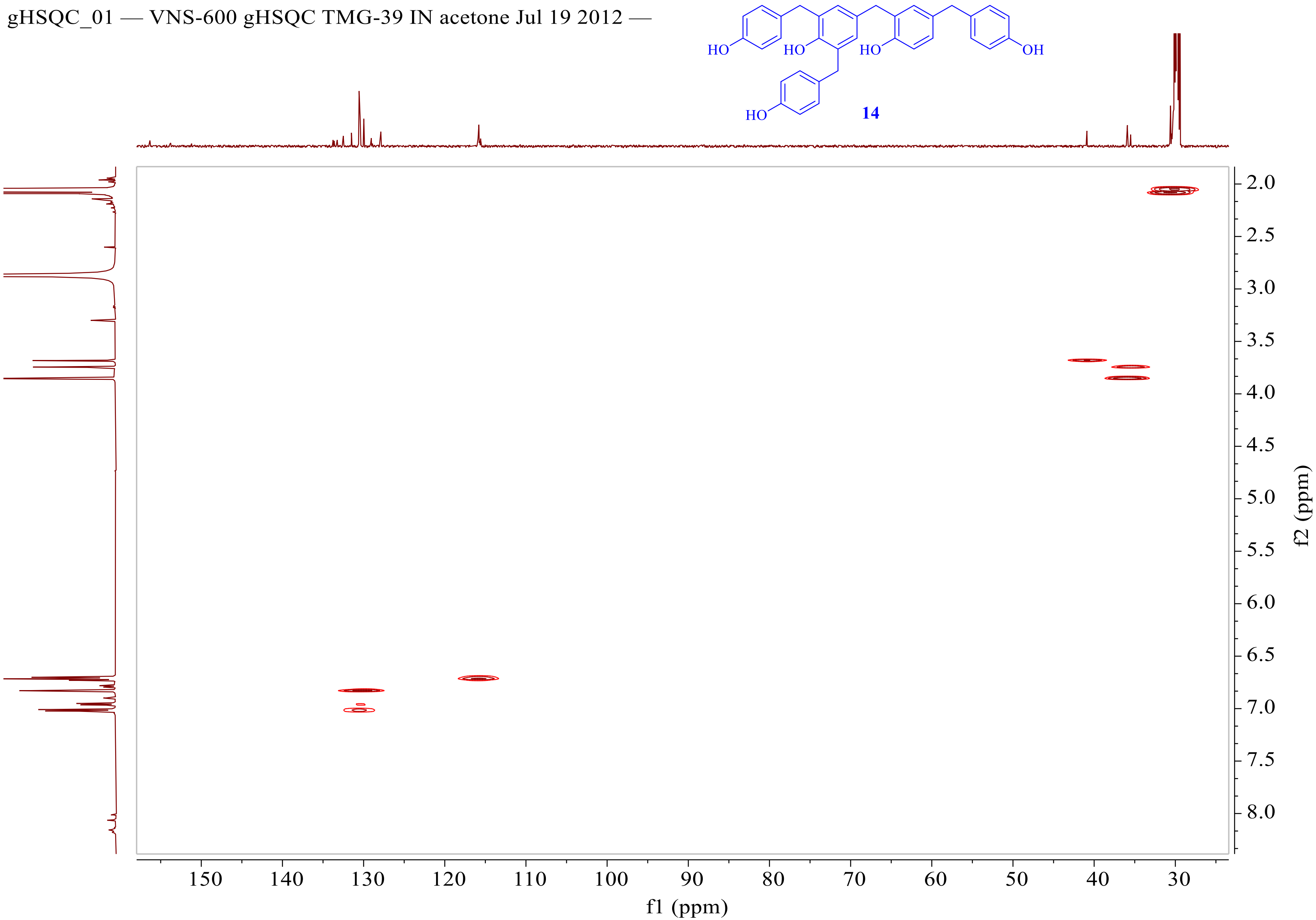

Figure S184. The HSQC spectrum of compound **14** in acetone-d_6 (600 MHz for ^{1}H)

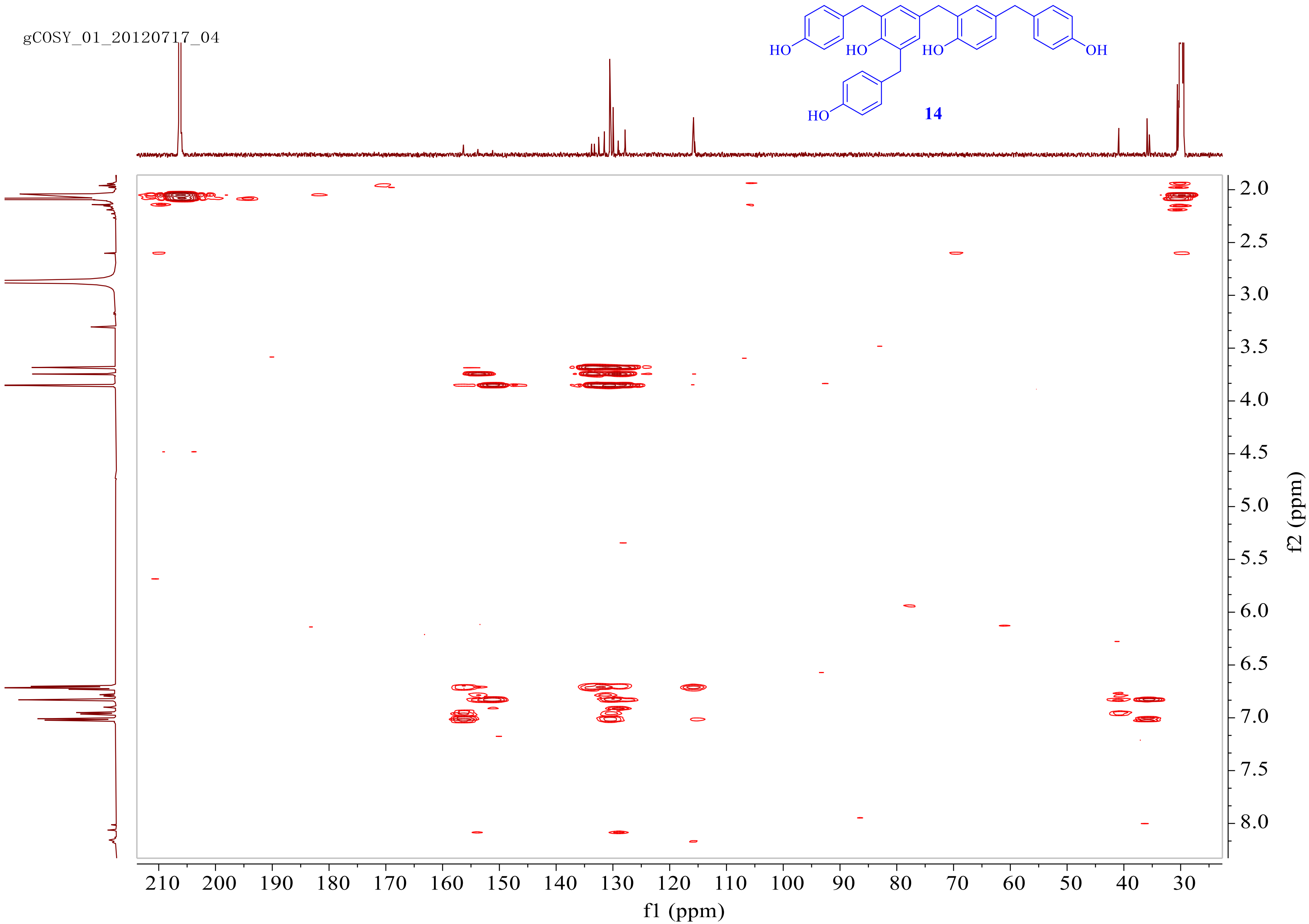

Figure S185. The HMBC spectrum of compound **14** in acetone-d_6 (600 MHz for ^{1}H)

DD2-500 NOESY1D TMG-39 IN acetone coldprobe-Probe

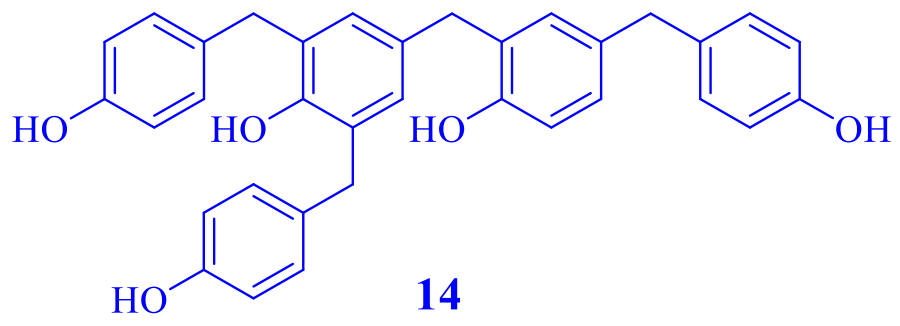

14

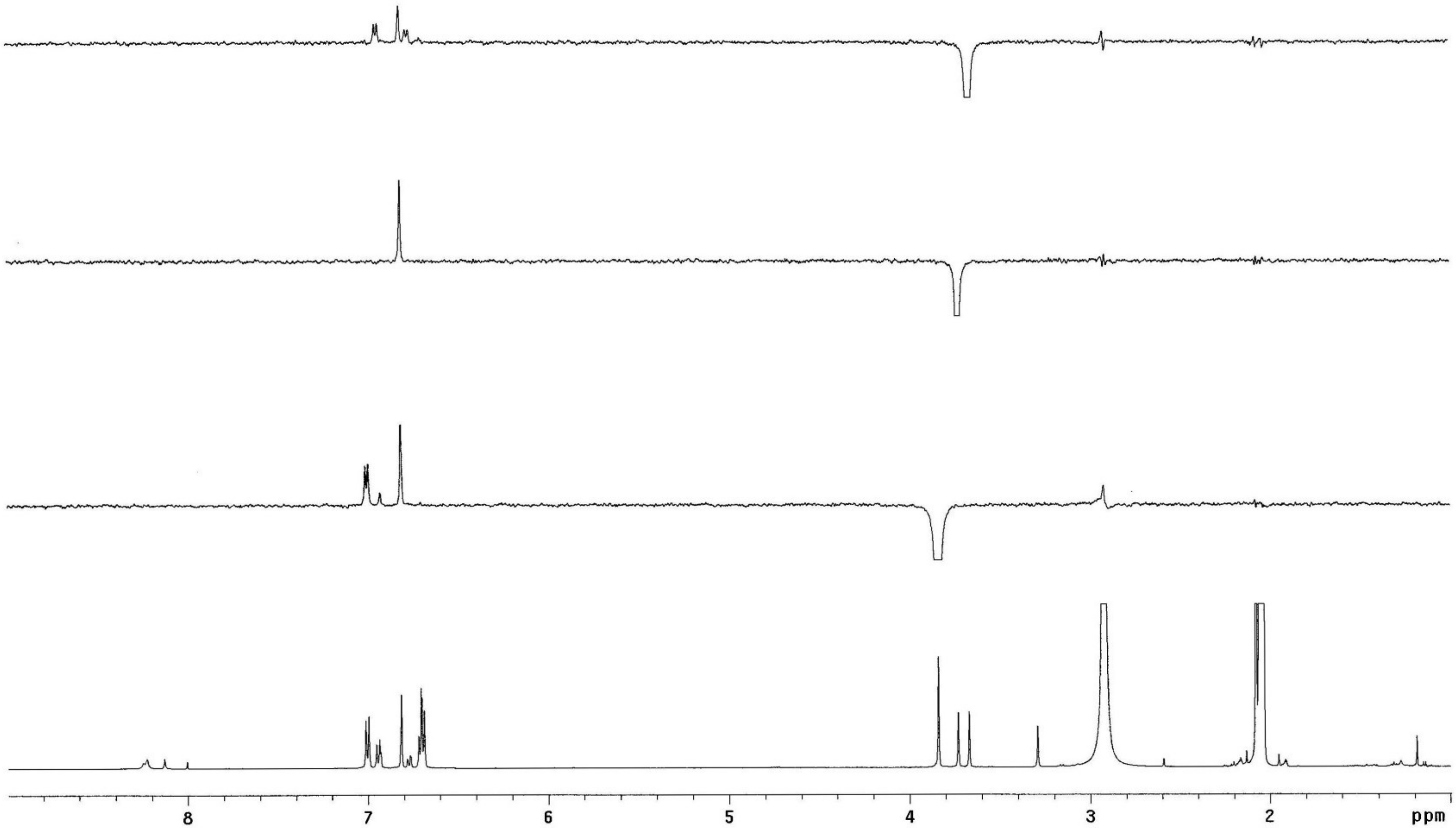

Figure S186. The NOESY1D spectrum of compound **14** in acetone-d_6 (600 MHz)

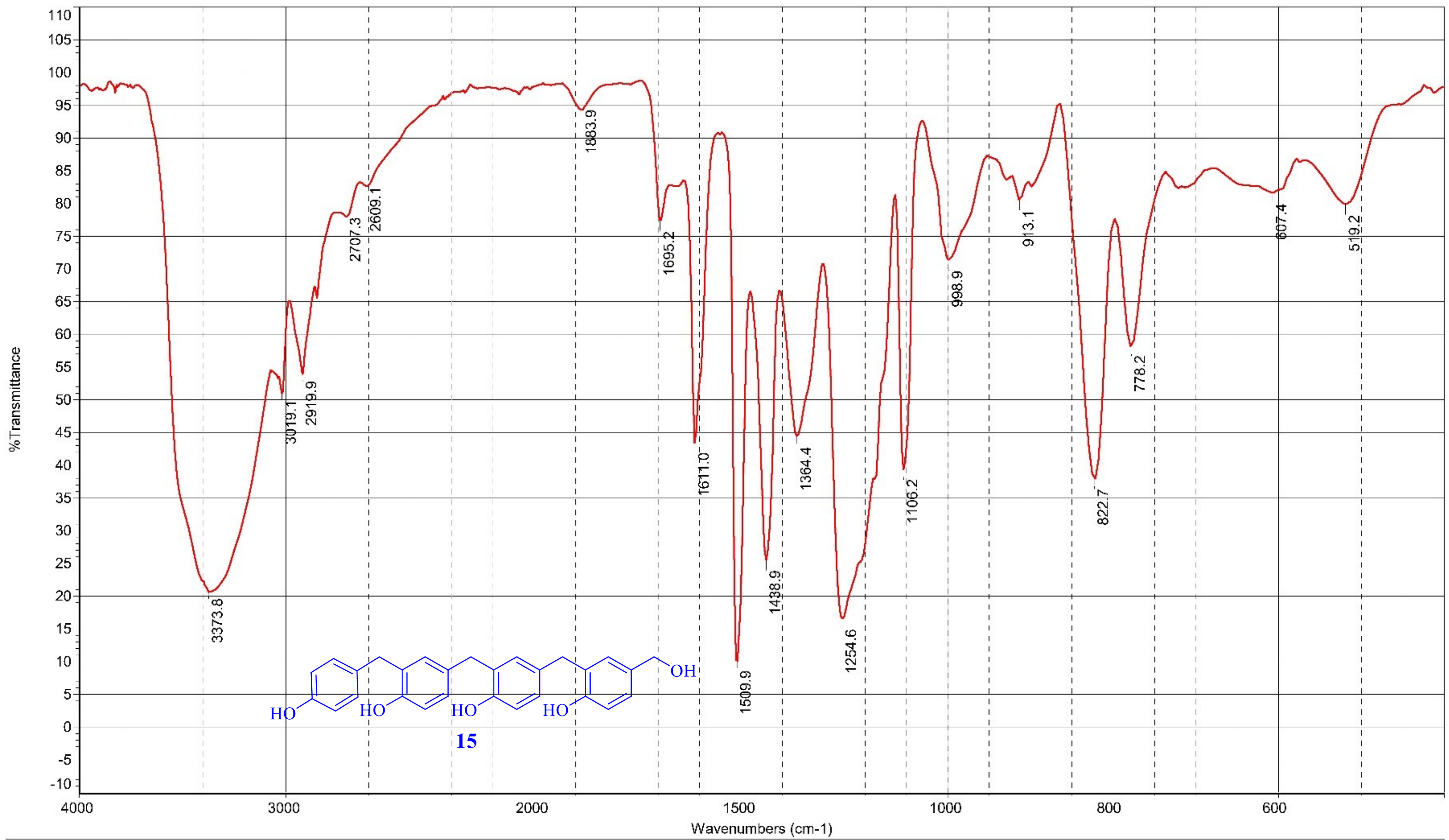

日期: 星期二 12月 15 16:35:01 2020 (GMT+08:00Sample Name： ZM-1 (显微镜透射法FT- IR Microscope Transmission)

扫描次数: 100

分辨率: 8.000

Figure S187. The IR spectrum of compound **15**

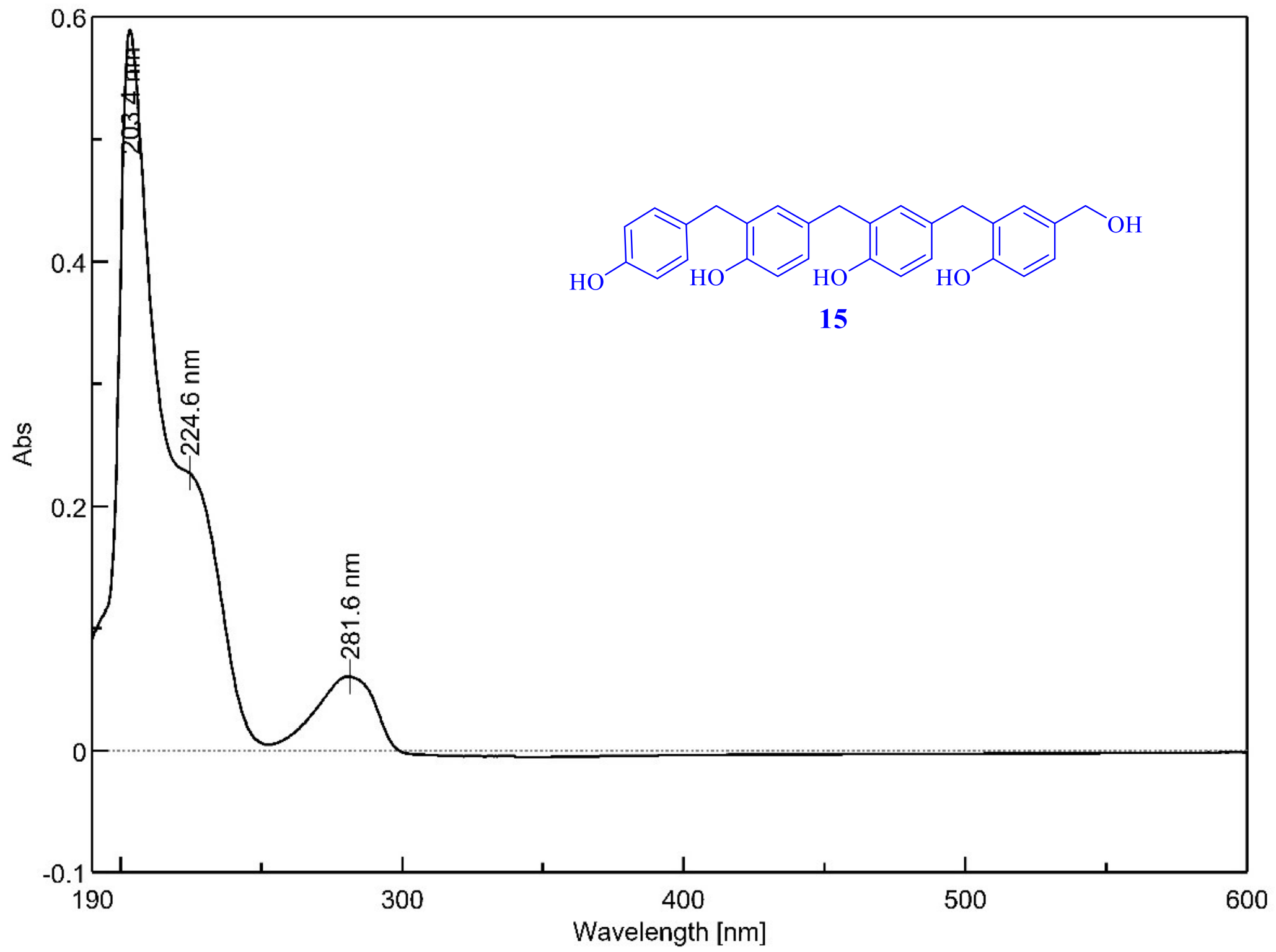

ZM-1

[Comment]
Sample Name DBT
Comment 0.02
User
Division UV
Company 324
[Measurement Information]
Instrument Name V-650
Model Name V-650
Serial No. A034461150

Accessory PSC-718
Accessory S/N A001761114
Position 1
Cell Length 10 mm
Temperature 20.00 C
Control Sonsor Holder
Monitor Sensor Holder
Start Mode Start immediately

Photometric Mode Abs
Measurement range 600 - 190 nm
Data pitch 0.2 nm
Band width(UV/Vis) 2.0 nm
Response Medium
Scanning speed 200 nm/min
Source Change 340 nm
Light Source D2/WI
Filter Exchange Step
Correction Baseline

[Data Information]
Creation Date 2020-11-11 15:09

Data array type Linear data array
Horizontal Wavelength [nm]
Vertical Abs
Start 600 nm
End 190 nm
Data pitch 0.2 nm
Data points 2051

Figure S188. The UV spectrum of compound **15** in MeOH

Thermo Qexactive Focus Report

compound NO. : ZM-1
Method : LCMS(compound)-low

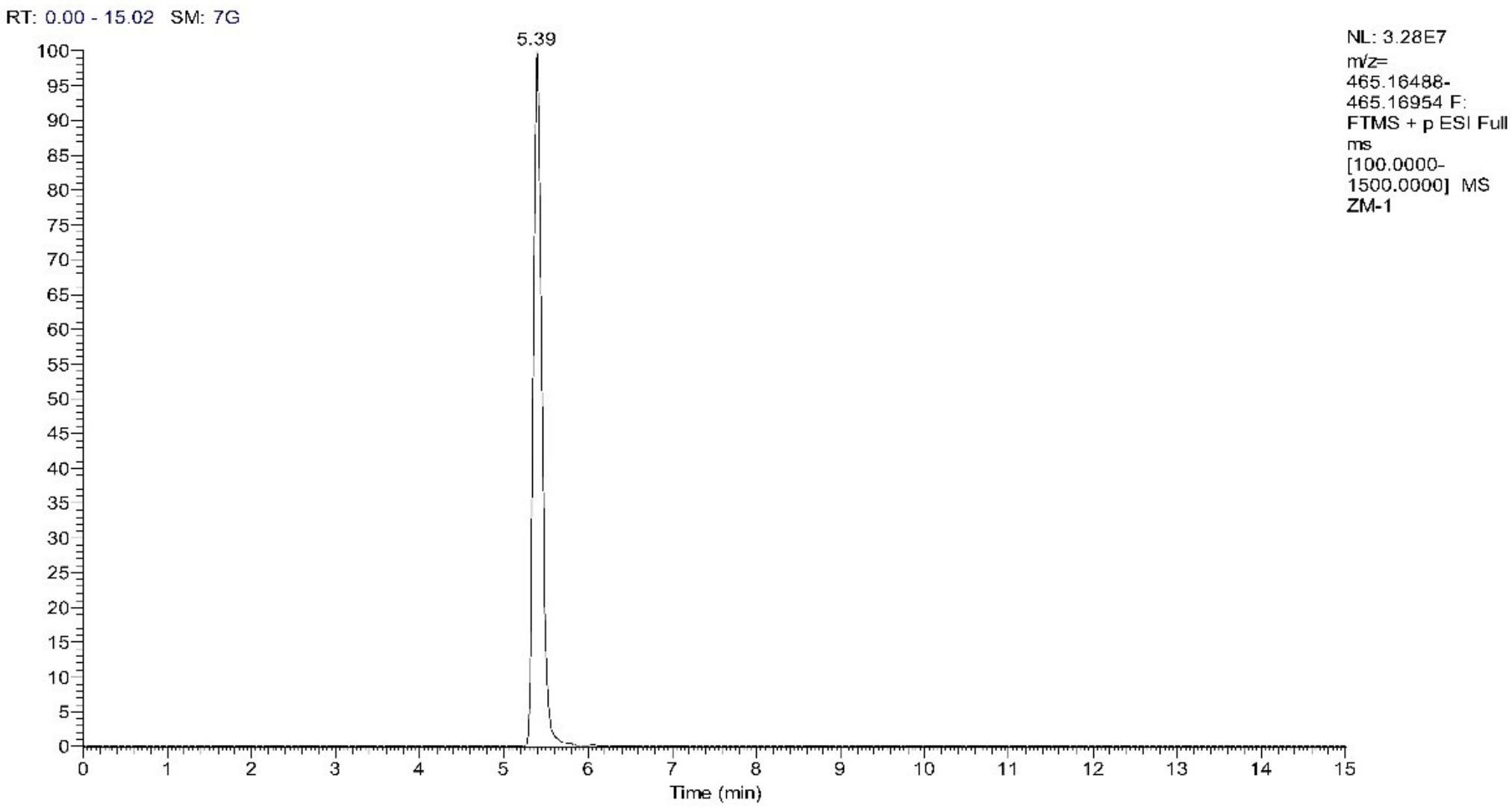

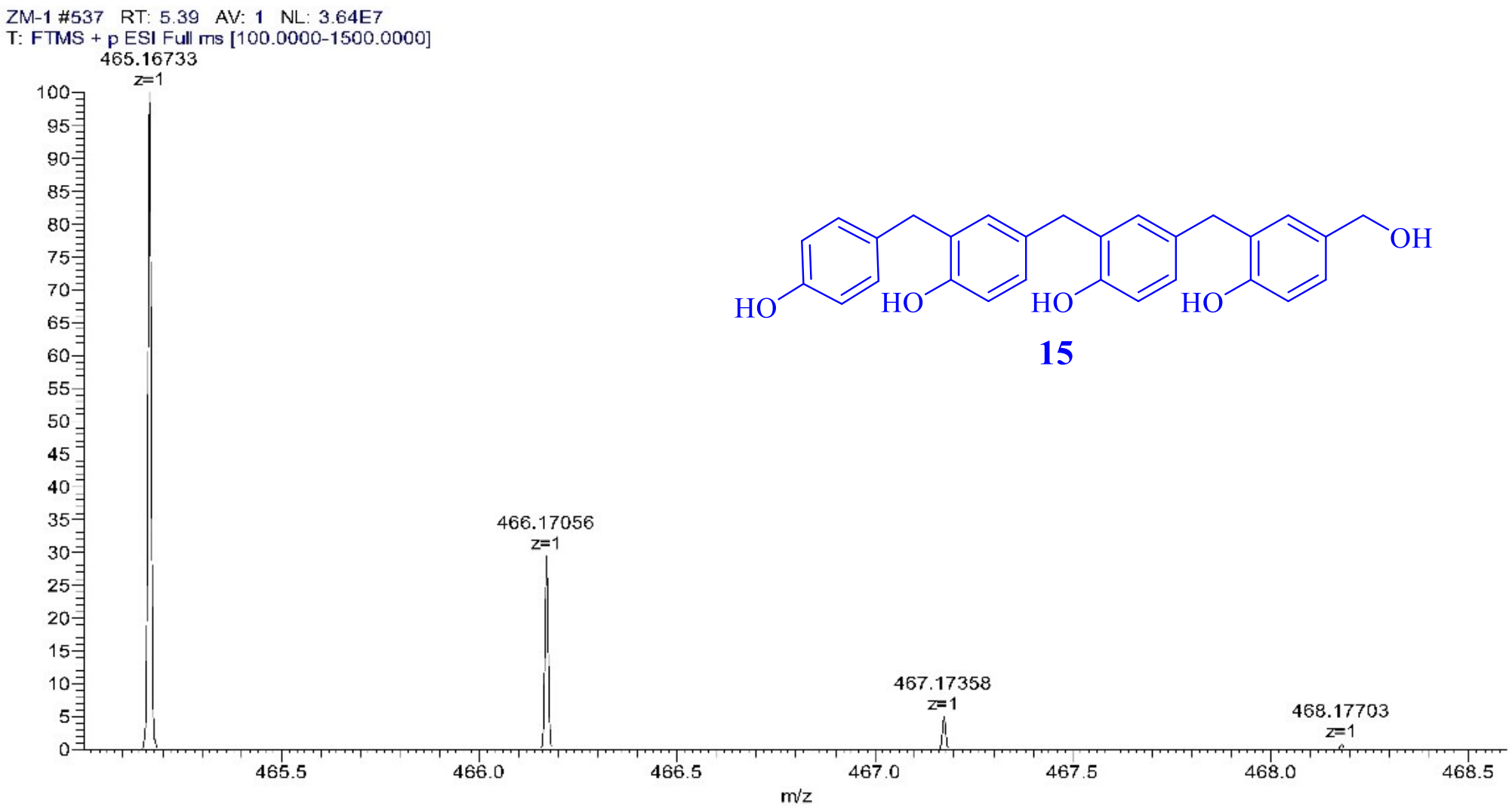

m/z	Theo. Mass	Delta (ppm)	RDB equiv.	Composition	
465.16733	465.16725	0.18	15.5	C28 H26 O5 Na	M+Na

Figure S189. The HR-ESIMS report of compound **15**

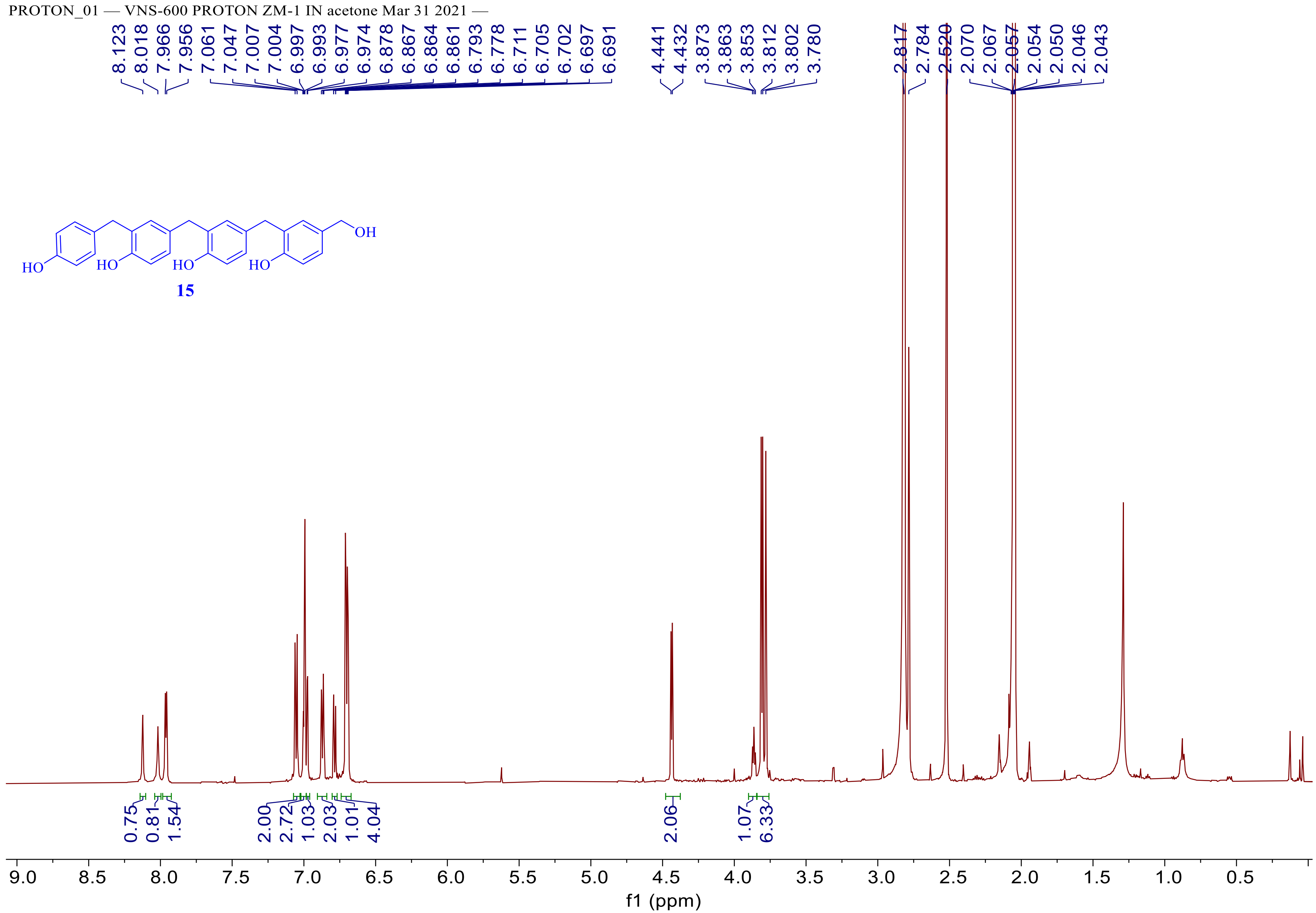

Figure S190. The ^{1}H NMR spectrum of compound **15** in acetone-d_6 (600 MHz)

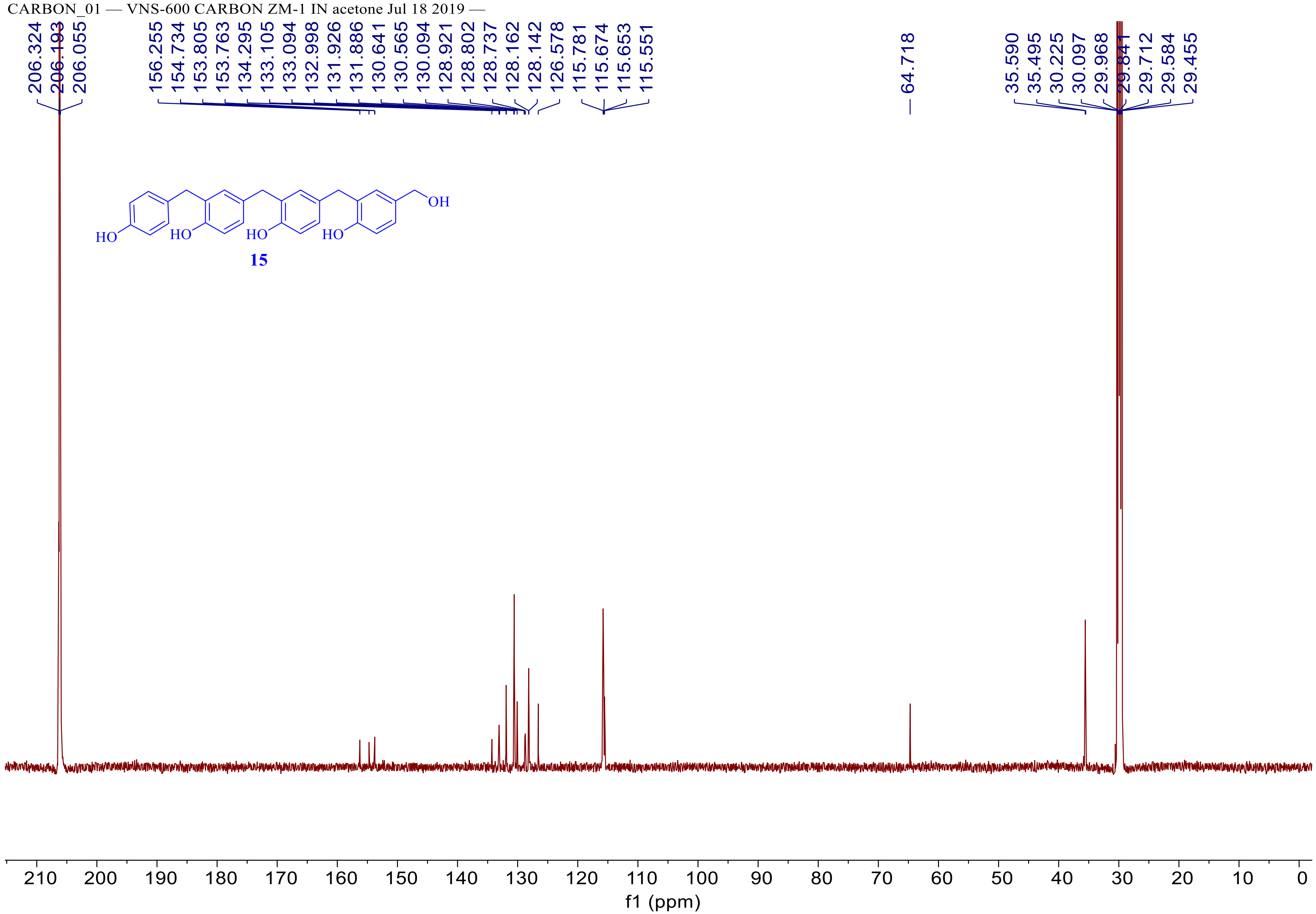

Figure S191. The ^{13}C NMR spectrum of compound **15** in acetone-d_6 (150 MHz)

DEPT_01 — VNS-600 DEPT ZM-1 IN acetone Jul 18 2019 —

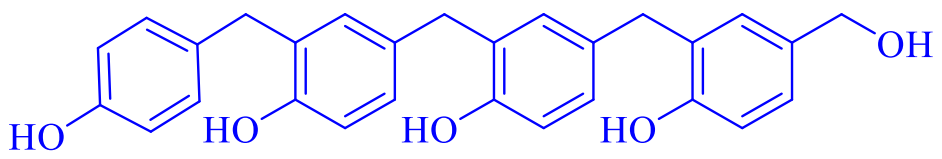

15

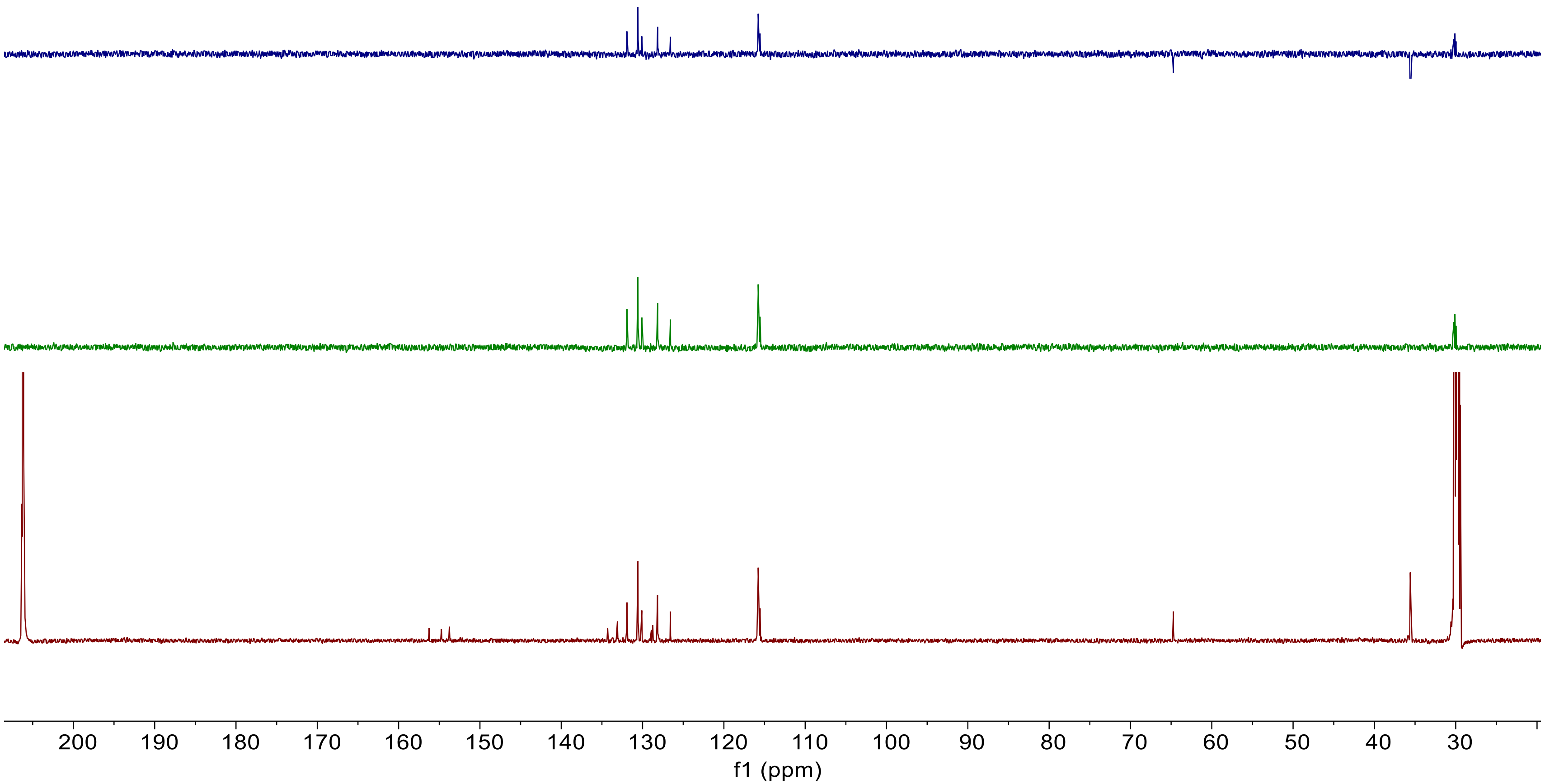

Figure S192. The DEPT spectrum of compound **15** in acetone-d_6 (150 MHz)

gCOSY_01 — VNS-600 gCOSY ZM-1 IN acetone Sep 16 2019 —

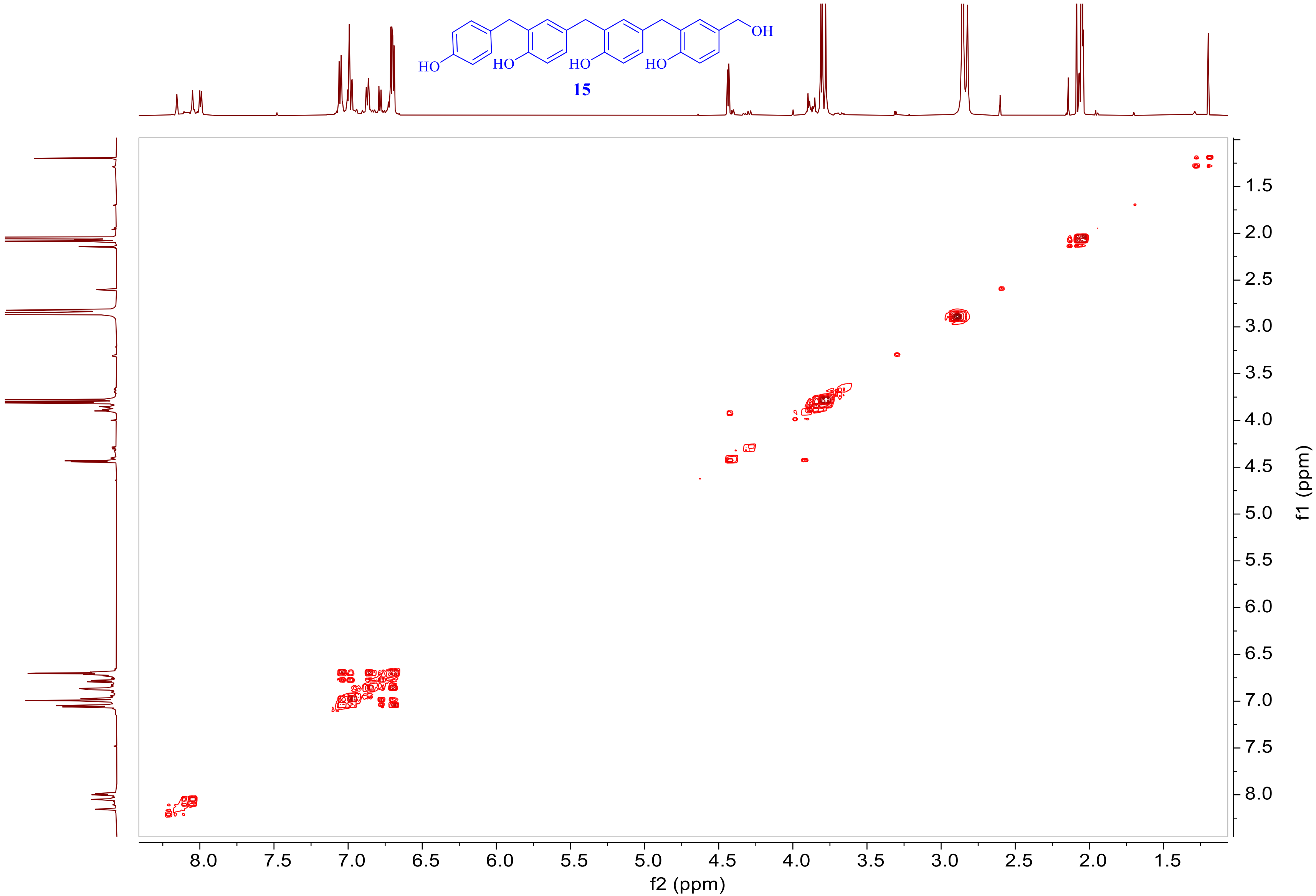

Figure S193. The ¹H-¹H COSY spectrum of compound **15** in acetone-d_6 (600 MHz)

gHSQCAD_01 — VNS-600 gHSQCAD ZM-1 IN acetone Mar 31 2021 —

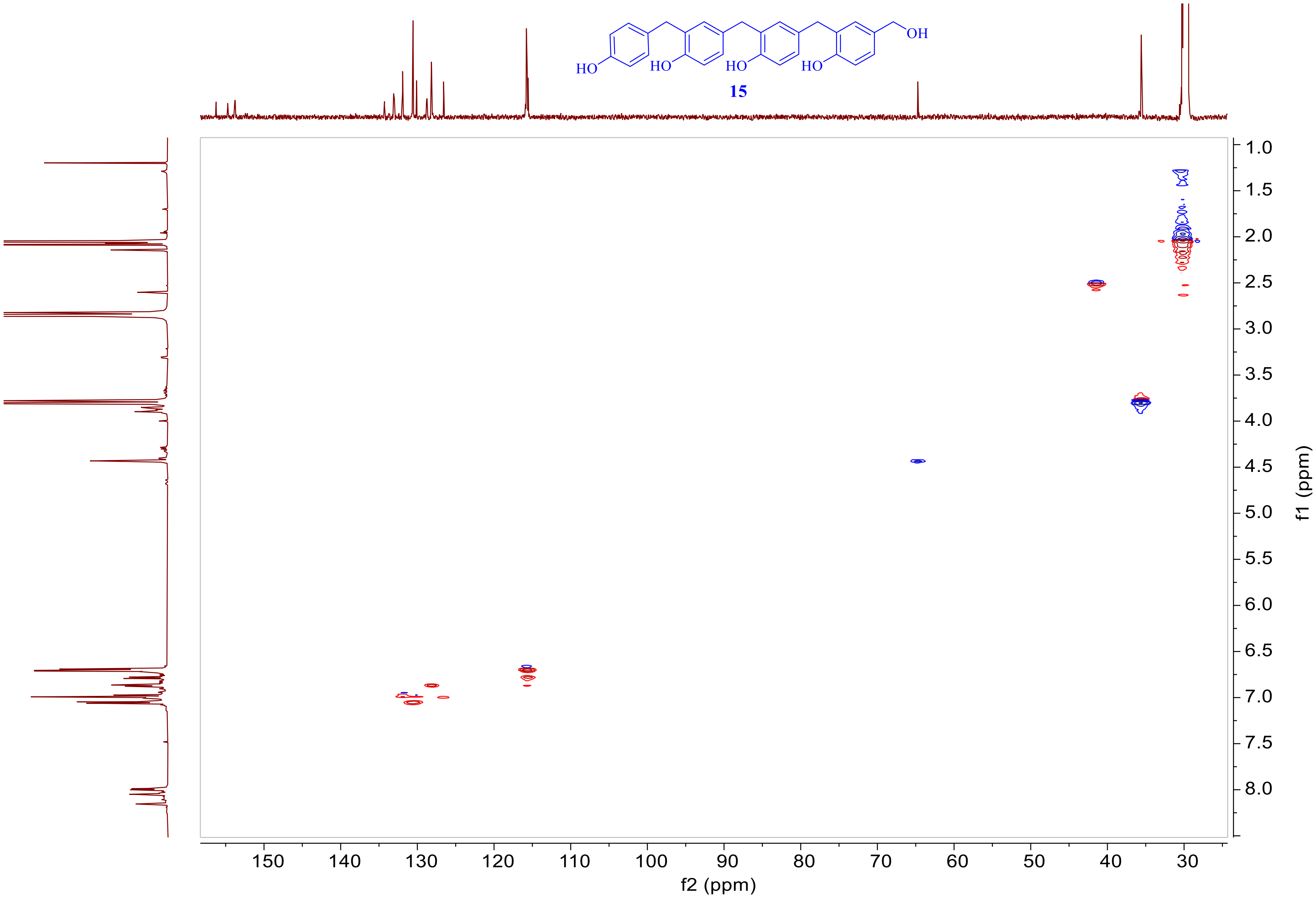

Figure S194. The HSQC spectrum of compound **15** in acetone-d_6 (600 MHz for ^{1}H)

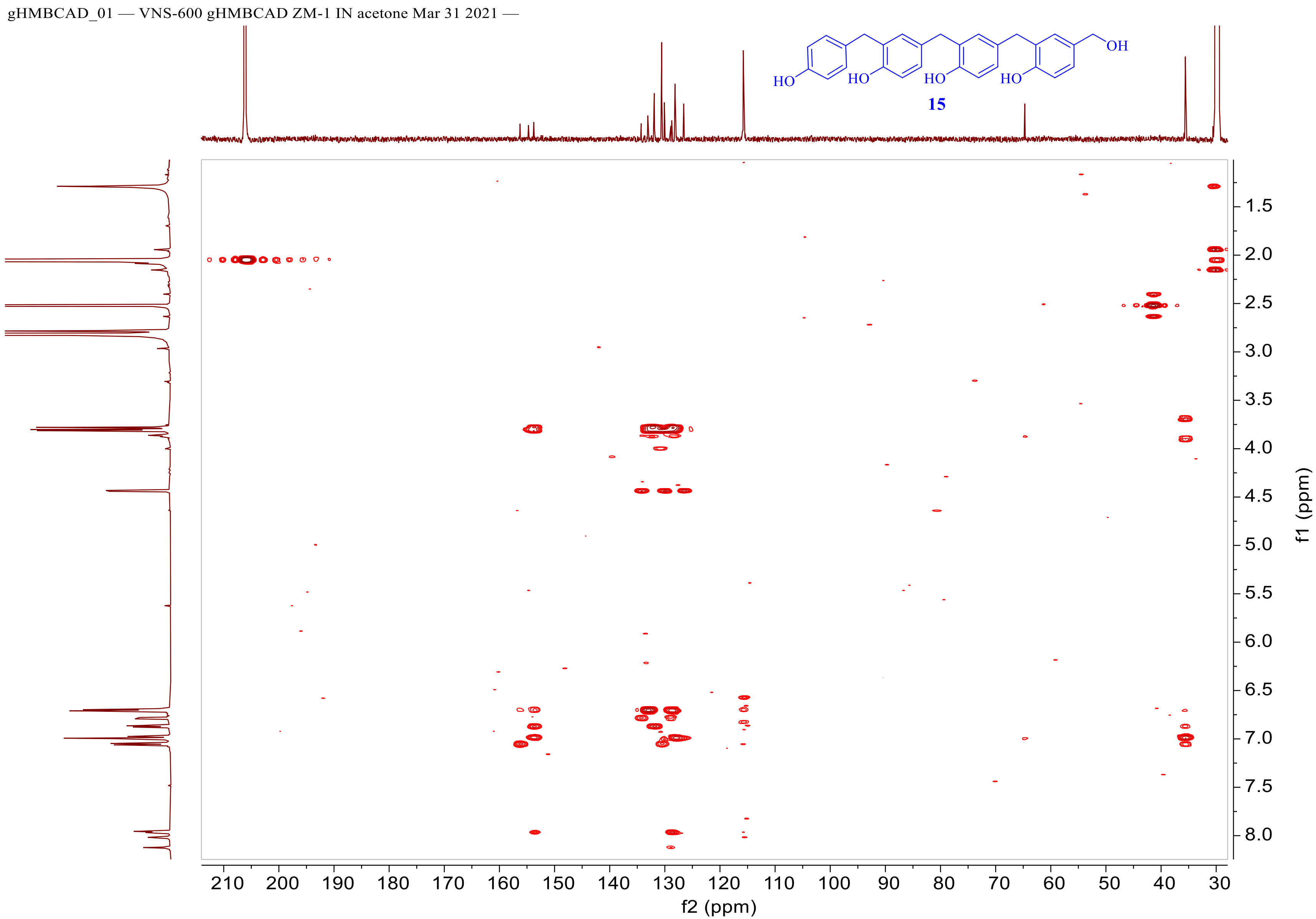

Figure S195. The HMBC spectrum of compound **15** in acetone-d_6 (600 MHz for ^{1}H)

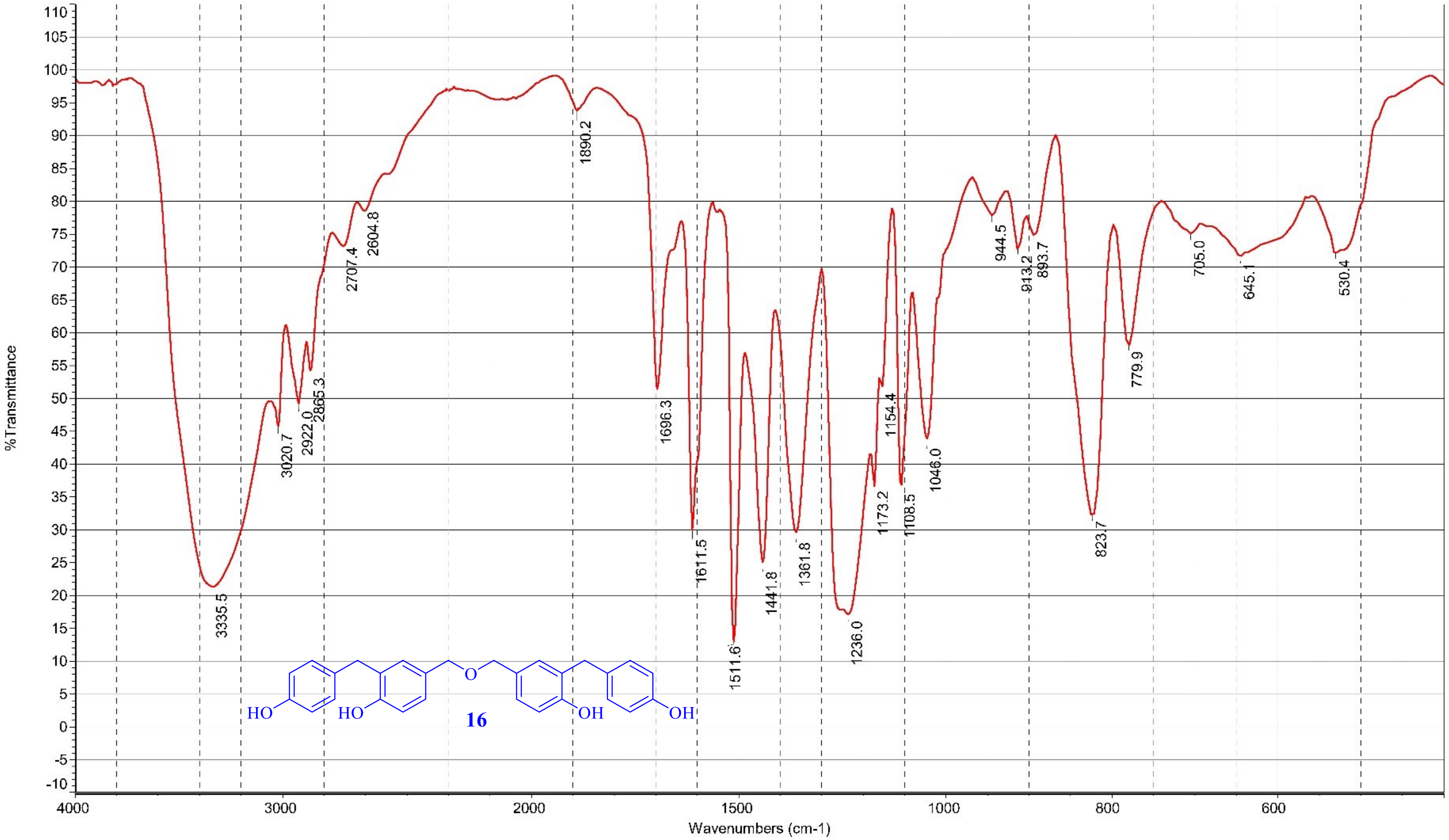

日期: 星期二 12月 15 15:46:08 2020 (GMT+08:00Sample Name： ZM-d4b2 (显微镜透射法FT- IR Microscope Transmission)

扫描次数: 100

分辨率: 8.000

Figure S196. The IR spectrum of compound **16**

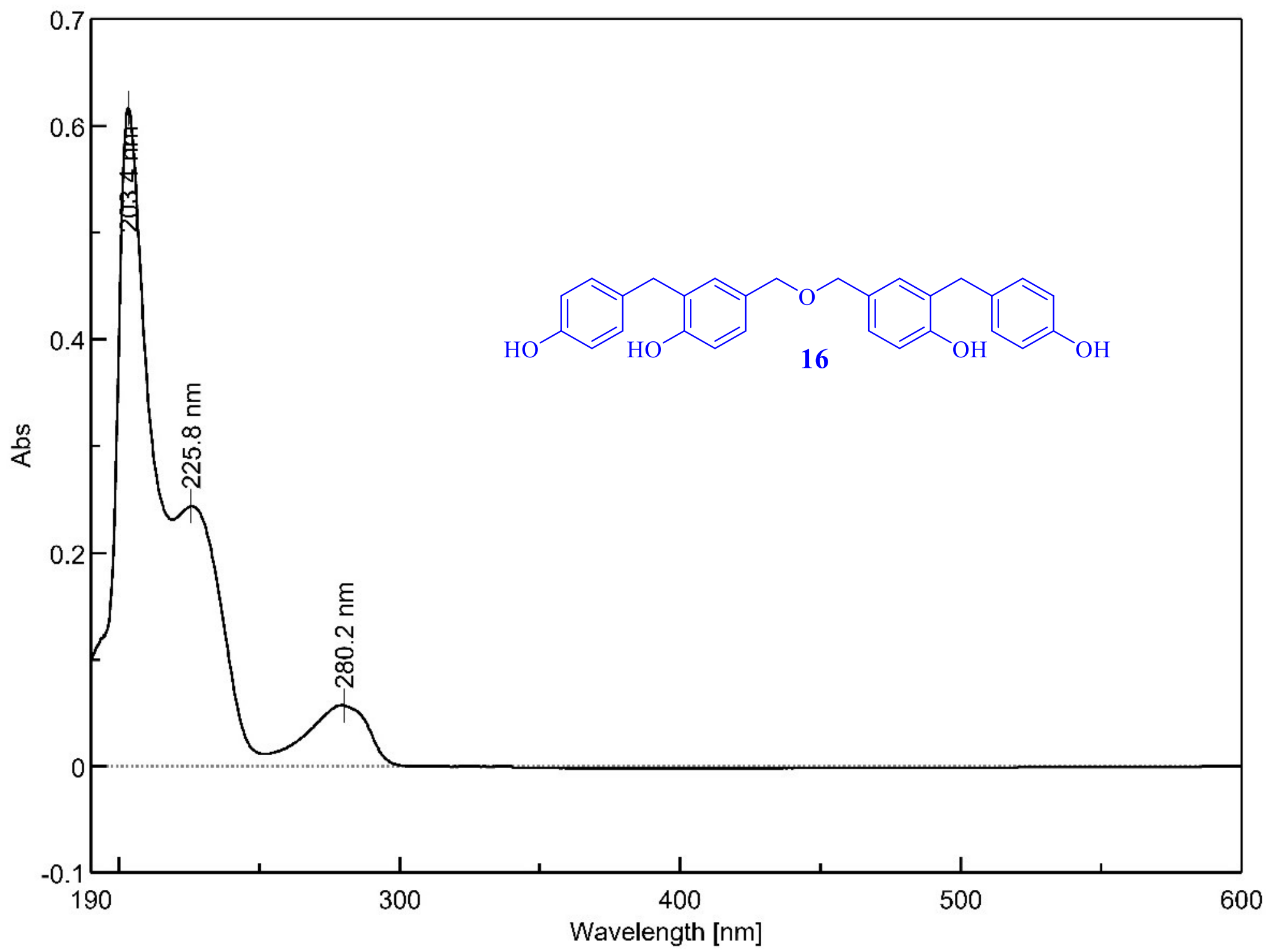

[Comment]
Sample Name DBT
Comment 0.02
User
Division UV
Company 324

d4b2

[Measurement Information]
Instrument Name V-650
Model Name V-650
Serial No. A034461150

Accessory PSC-718
Accessory S/N A001761114
Position 1
Cell Length 10 mm
Temperature 19.99 C
Control Sensor Holder
Monitor Sensor Holder
Start Mode Start immediately

Photometric Mode Abs
Measurement range 600 - 190 nm
Data pitch 0.2 nm
Band width(UV/Vis) 2.0 nm
Response Medium
Scanning speed 200 nm/min
Source Change 340 nm
Light Source D2/WI
Filter Exchange Step
Correction Baseline

[Data Information]
Creation Date 2020-11-11 15:01

Data array type Linear data array
Horizontal Wavelength [nm]
Vertical Abs
Start 600 nm
End 190 nm
Data pitch 0.2 nm
Data points 2051

Figure S197. The UV spectrum of compound **16** in MeOH

Thermo Qexactive Focus Report

compound NO. : d4b2
Method : LCMS(compound)-low

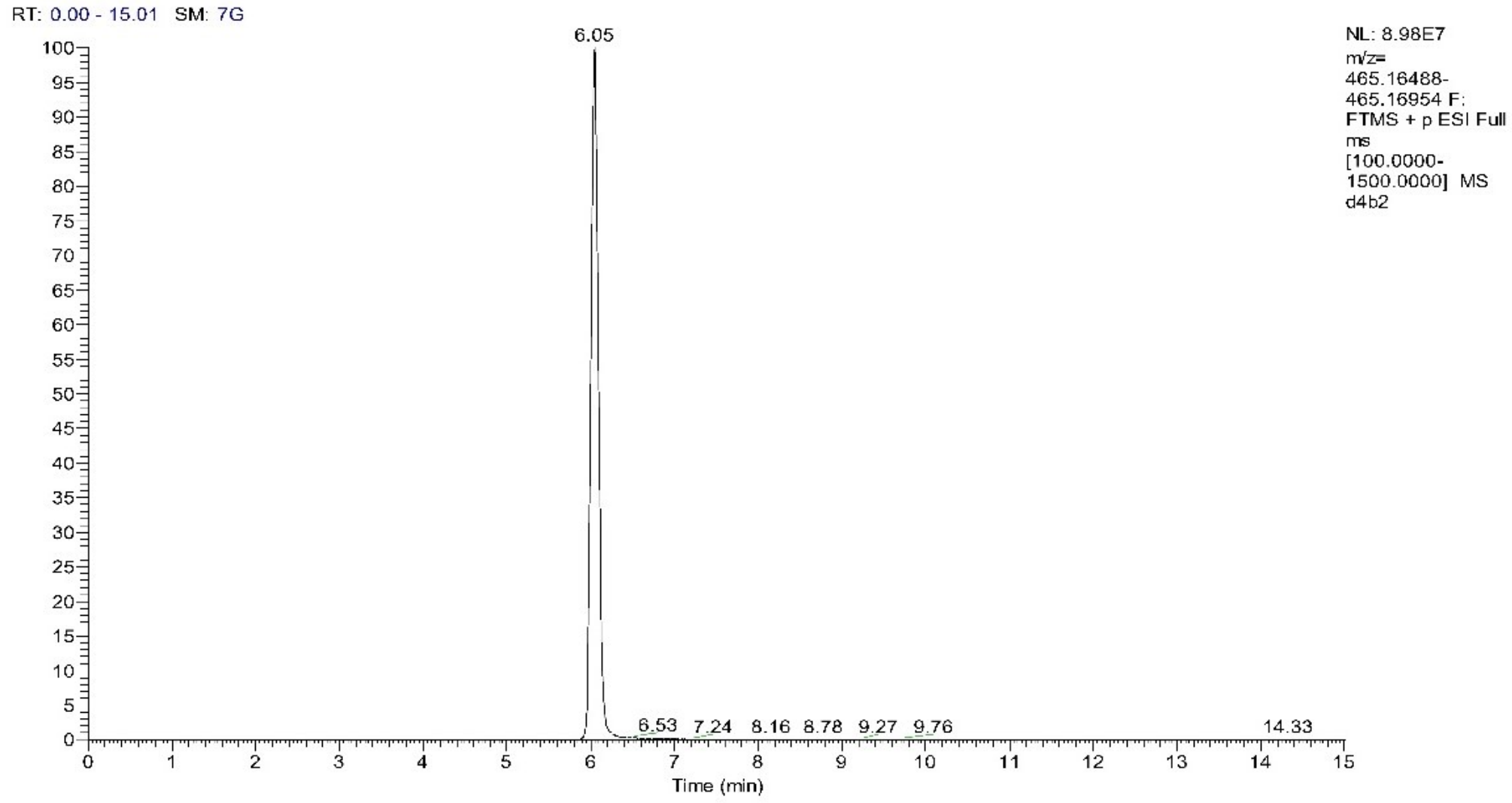

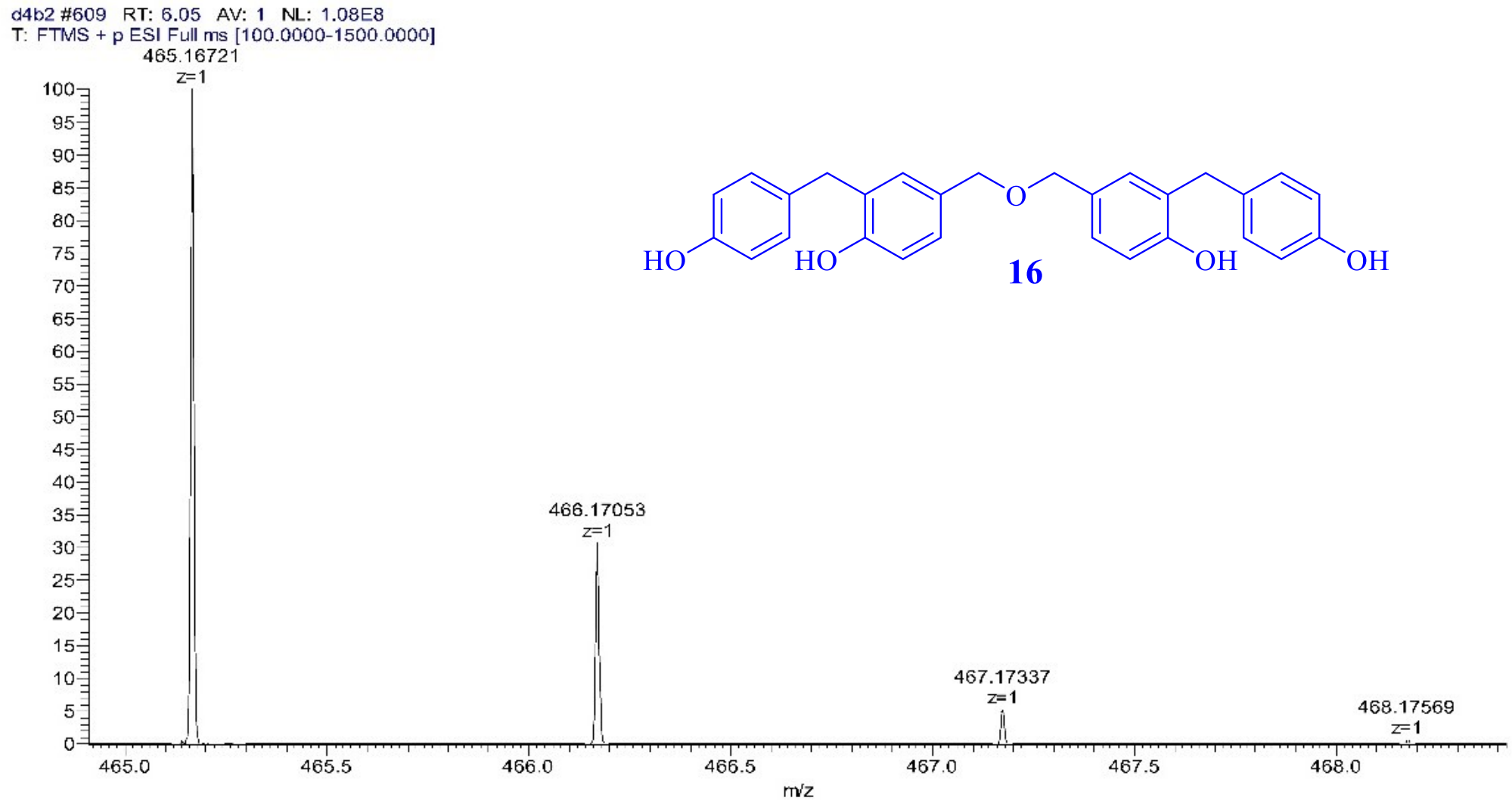

m/z	Theo. Mass	Delta (ppm)	RDB equiv.	Composition	
465.16721	465.16725	-0.08	15.5	C28 H26 O5 Na	M+Na

Figure S198. The HR-ESIMS report of compound **16**

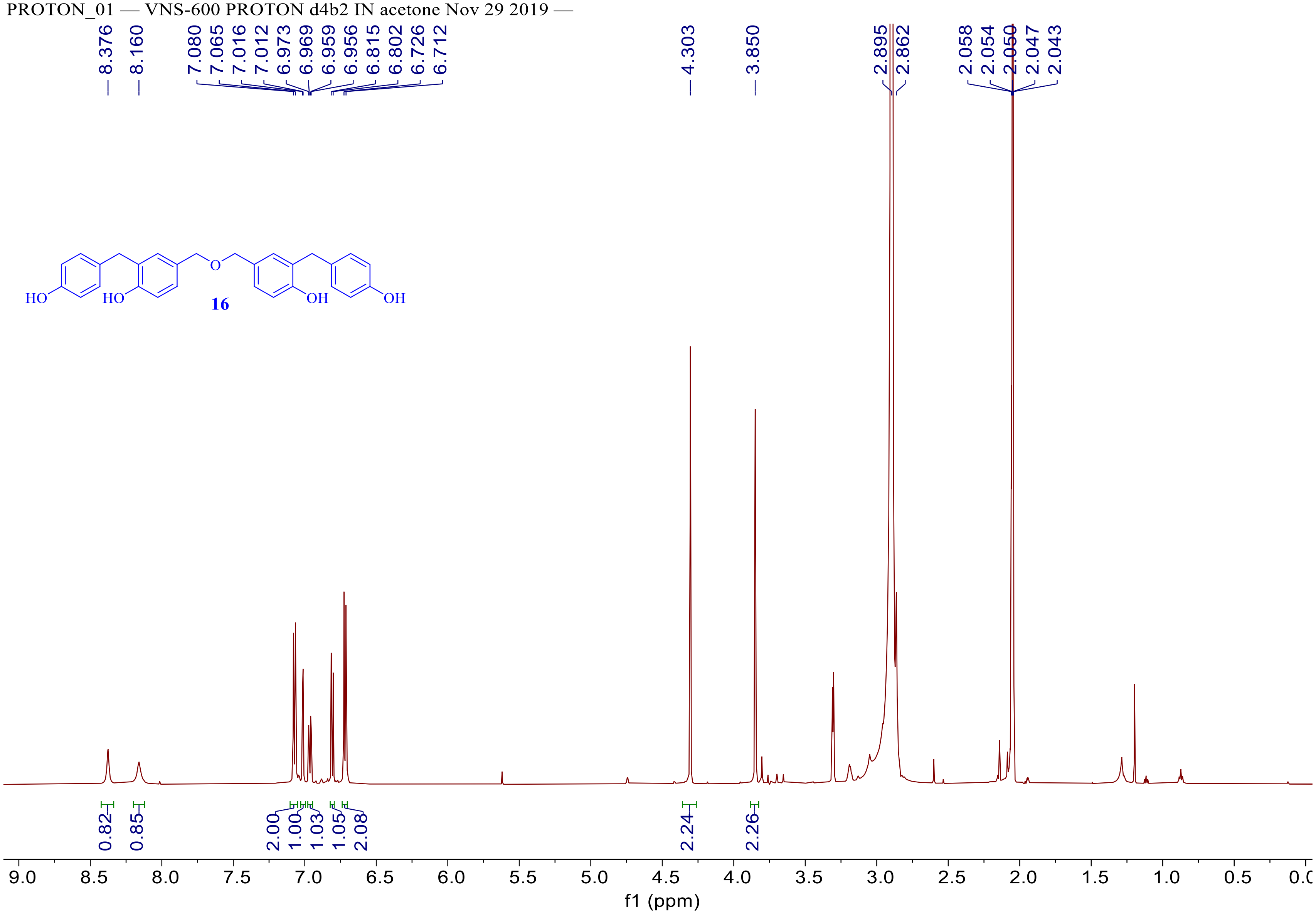

Figure S199. The ^{1}H NMR spectrum of compound **16** in acetone-d_6 (600 MHz)

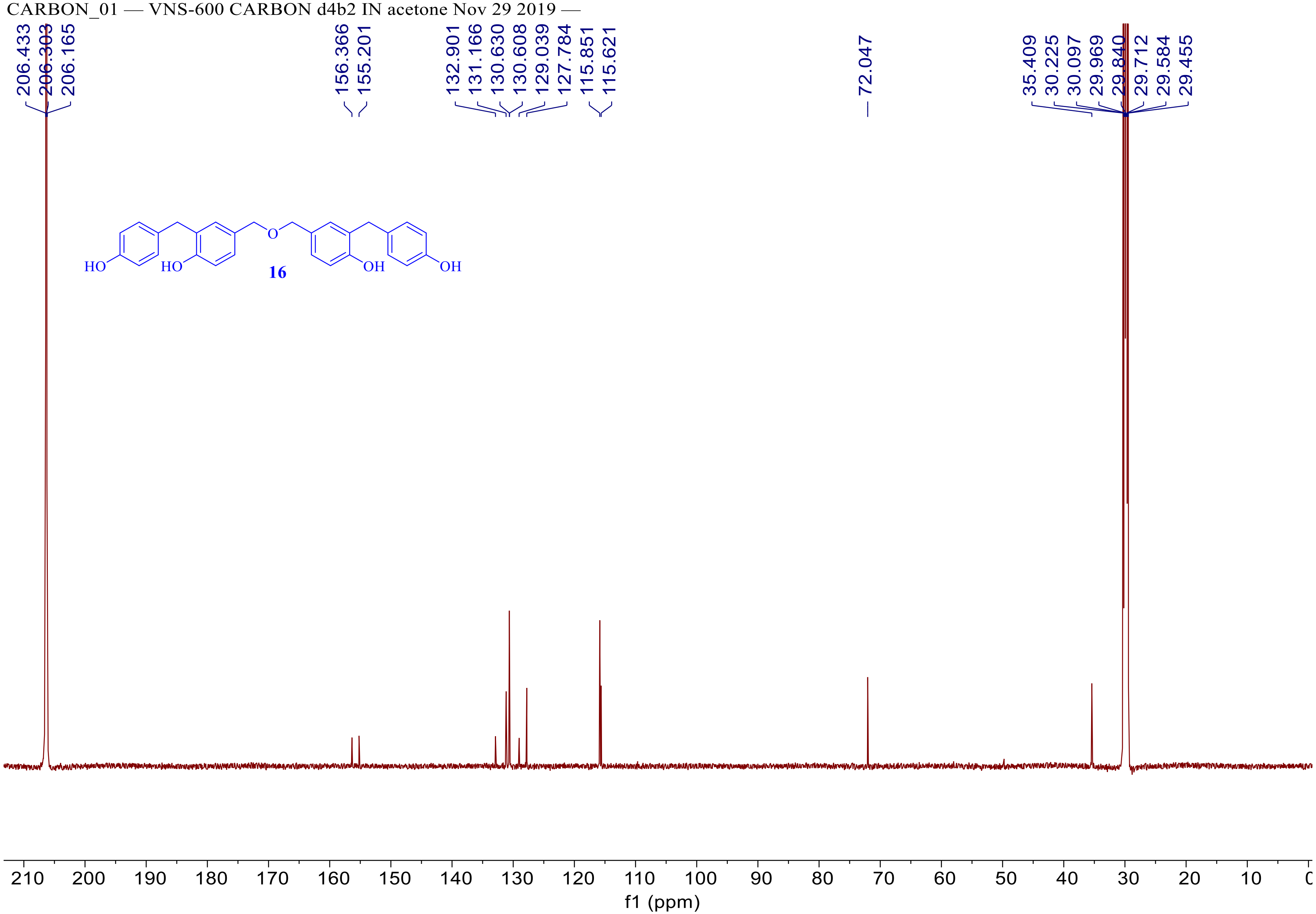

Figure S200. The ^{13}C NMR spectrum of compound **16** in acetone-d_6 (150 MHz)

DEPT_01 — VNS-600 DEPT d4b2 IN acetone Nov 29 2019 —

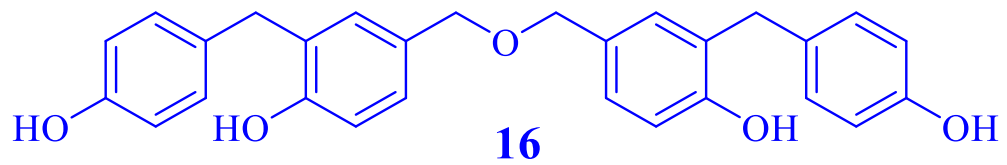

16

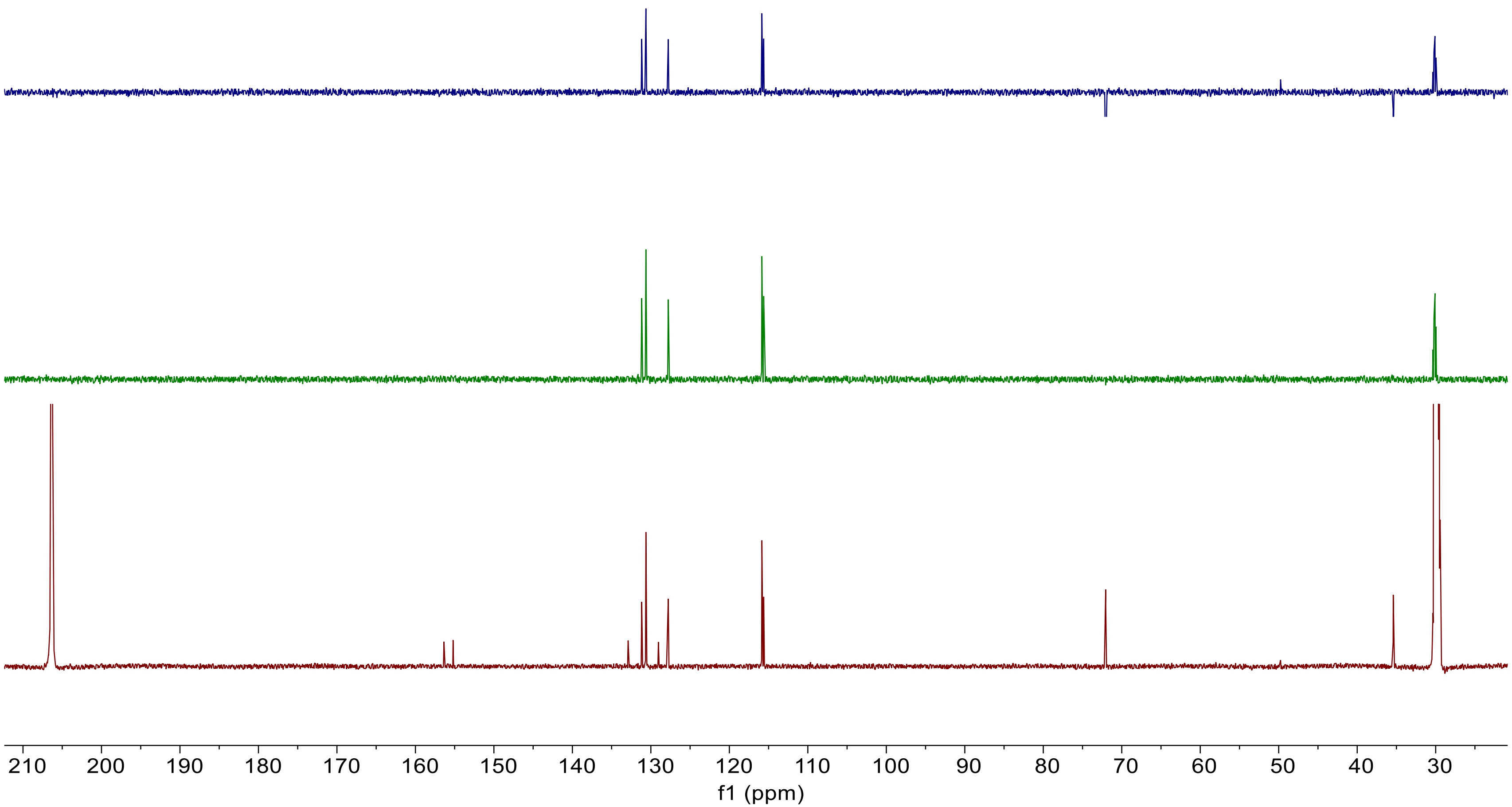

Figure S201. The DEPT spectrum of compound **16** in acetone-d_6 (150 MHz)

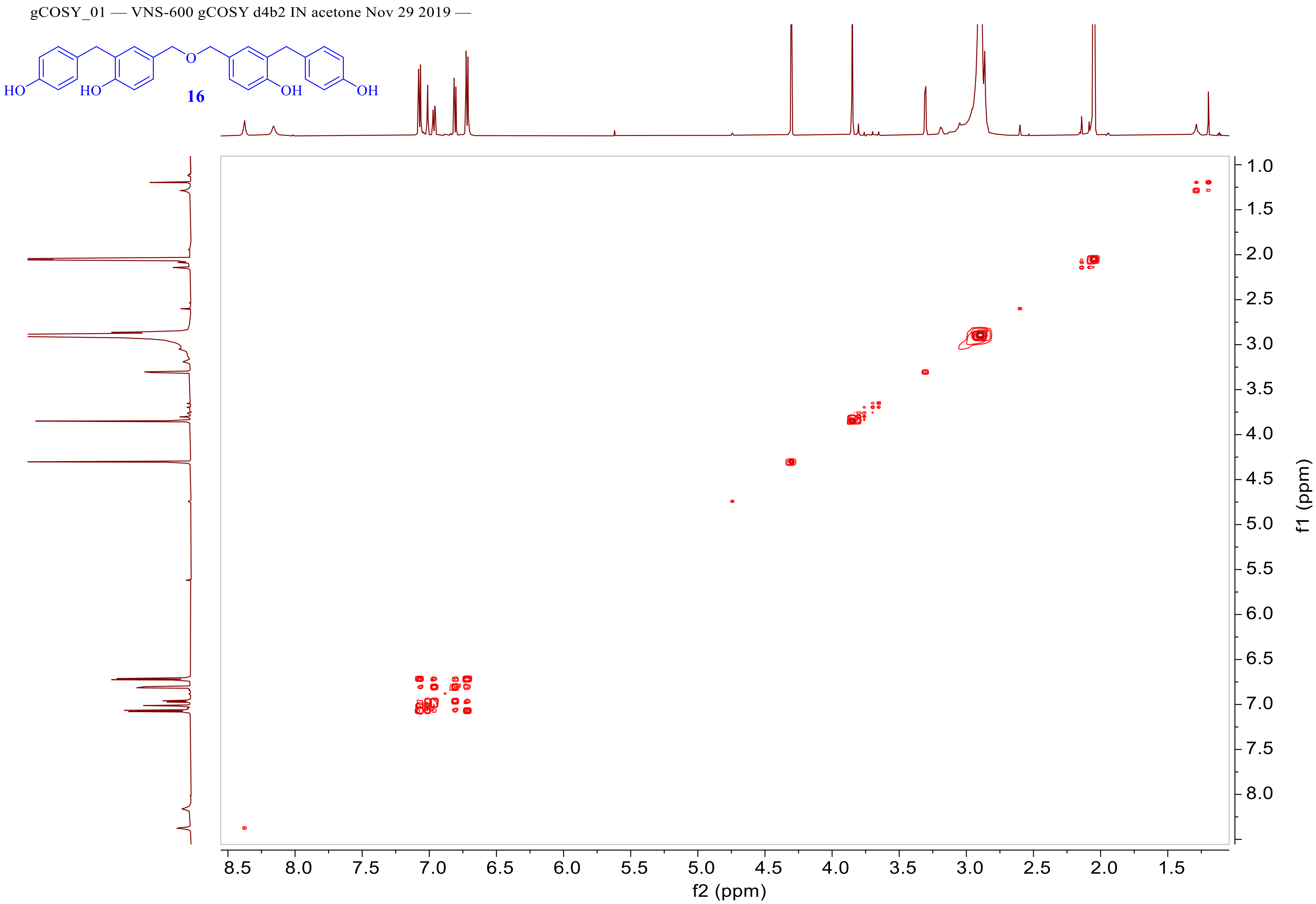

Figure S202. The ^{1}H-^{1}H COSY spectrum of compound **16** in acetone-d_6 (600 MHz)

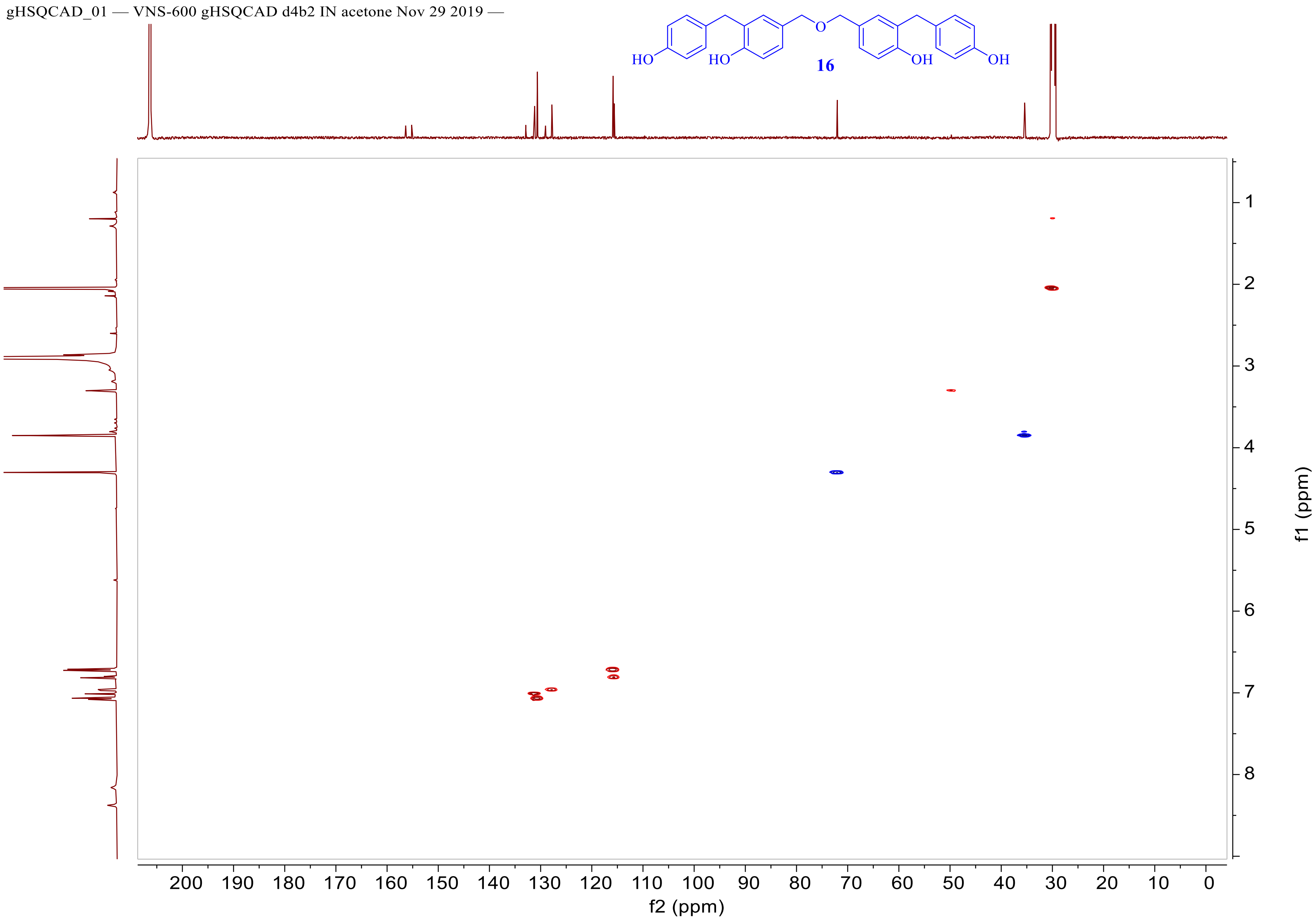

Figure S203. The HSQC spectrum of compound **16** in acetone-d_6 (600 MHz for ^{1}H)

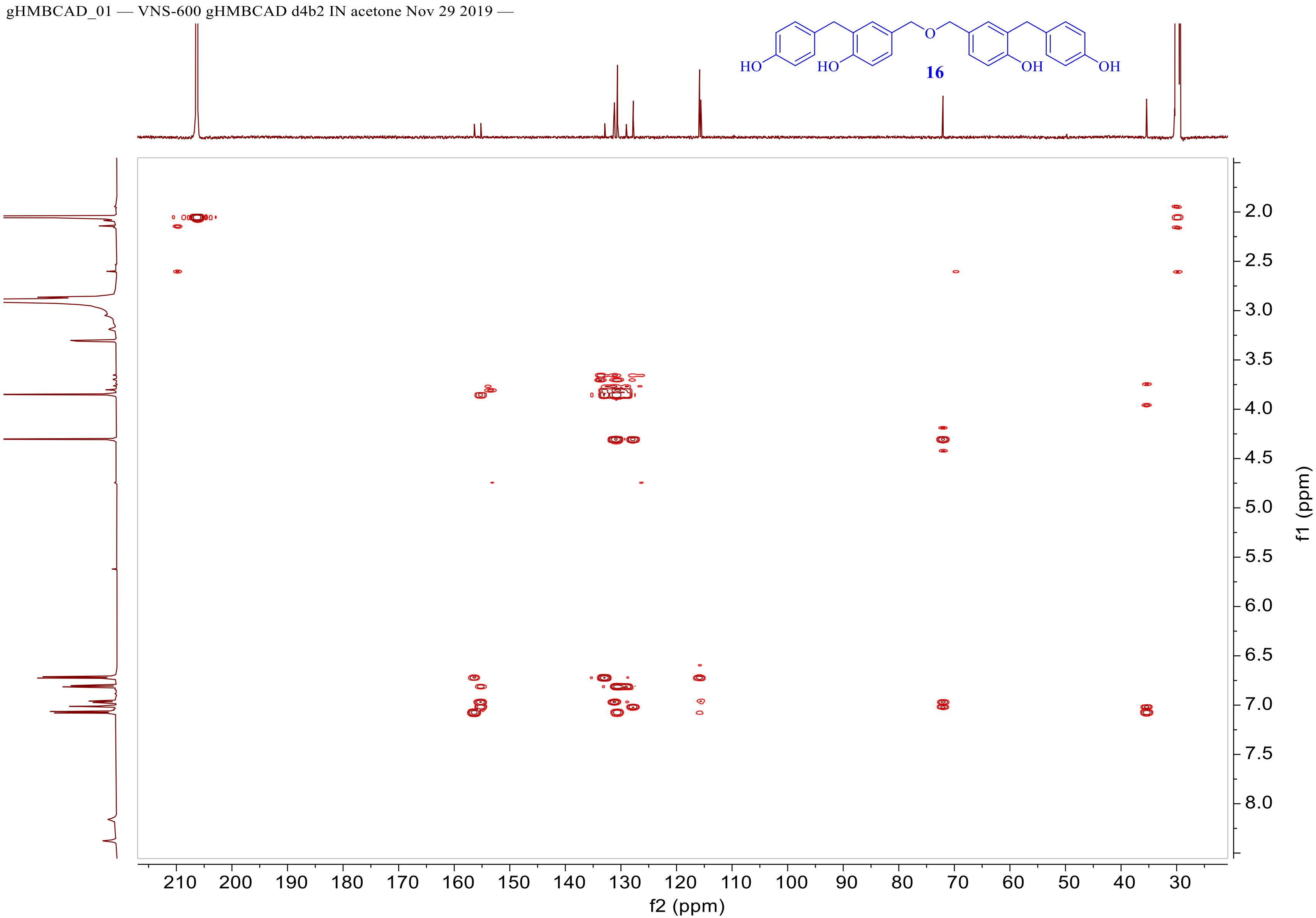

Figure S204. The HMBC spectrum of compound **16** in acetone-d_6 (600 MHz for ^{1}H)

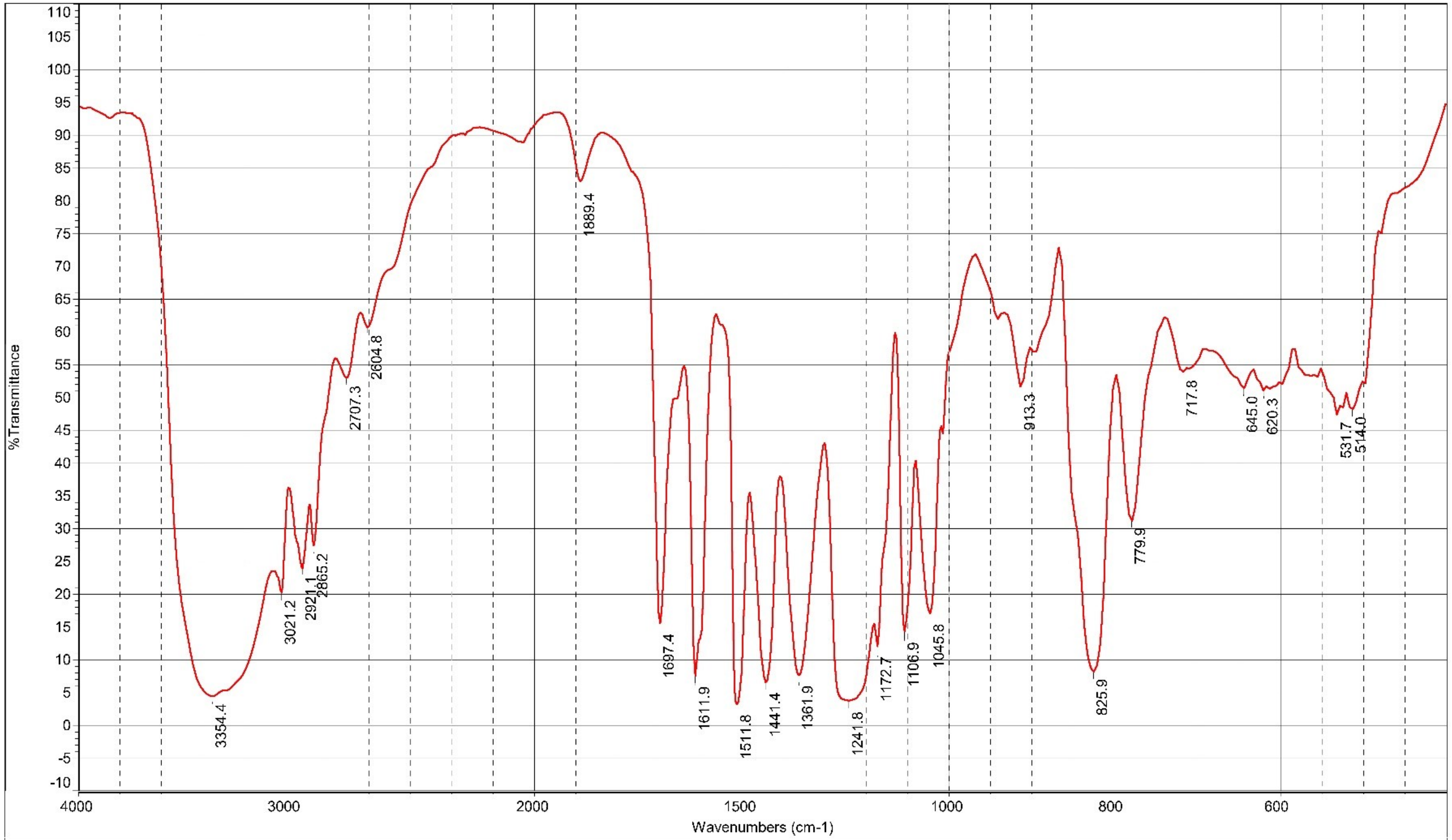

日期: 星期一 12月 21 16:53:58 2020 (GMT+08:00Sample Name：ZM-4 (显微镜透射法FT- IR Microscope Transmission)

扫描次数: 100

分辨率: 8.000

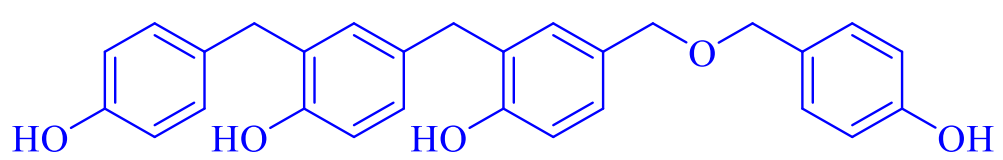

17

Figure S205. The IR spectrum of compound **17**

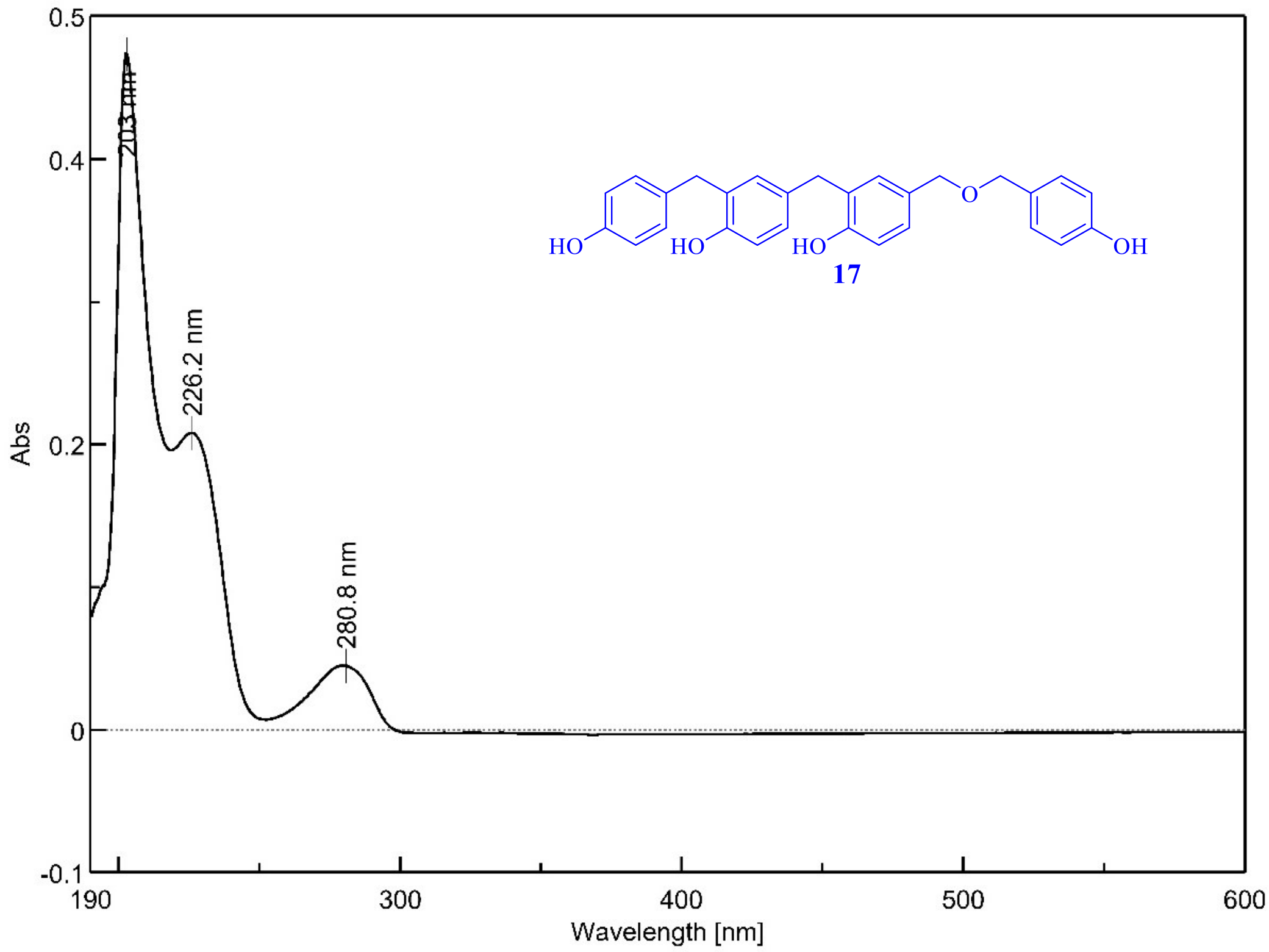

ZM-4

[Comment]
Sample Name DBT
Comment 0.02
User
Division UV
Company 324
[Measurement Information]
Instrument Name V-650
Model Name V-650
Serial No. A034461150

Accessory PSC-718
Accessory S/N A001761114
Position 1
Cell Length 10 mm
Temperature 19.97 C
Control Sonsor Holder
Monitor Sensor Holder
Start Mode Start immediately

Photometric Mode Abs
Measurement range 600 - 190 nm
Data pitch 0.2 nm
Band width(UV/Vis) 2.0 nm
Response Medium
Scanning speed 200 nm/min
Source Change 340 nm
Light Source D2/WI
Filter Exchange Step
Correction Baseline

[Data Information]
Creation Date 2020-11-11 15:16

Data array type Linear data array
Horizontal Wavelength [nm]
Vertical Abs
Start 600 nm
End 190 nm
Data pitch 0.2 nm
Data points 2051

Figure S206. The UV spectrum of compound **17** in MeOH

Thermo Qexactive Focus Report

compound NO. : ZM-4
Method : LCMS(compound)-low

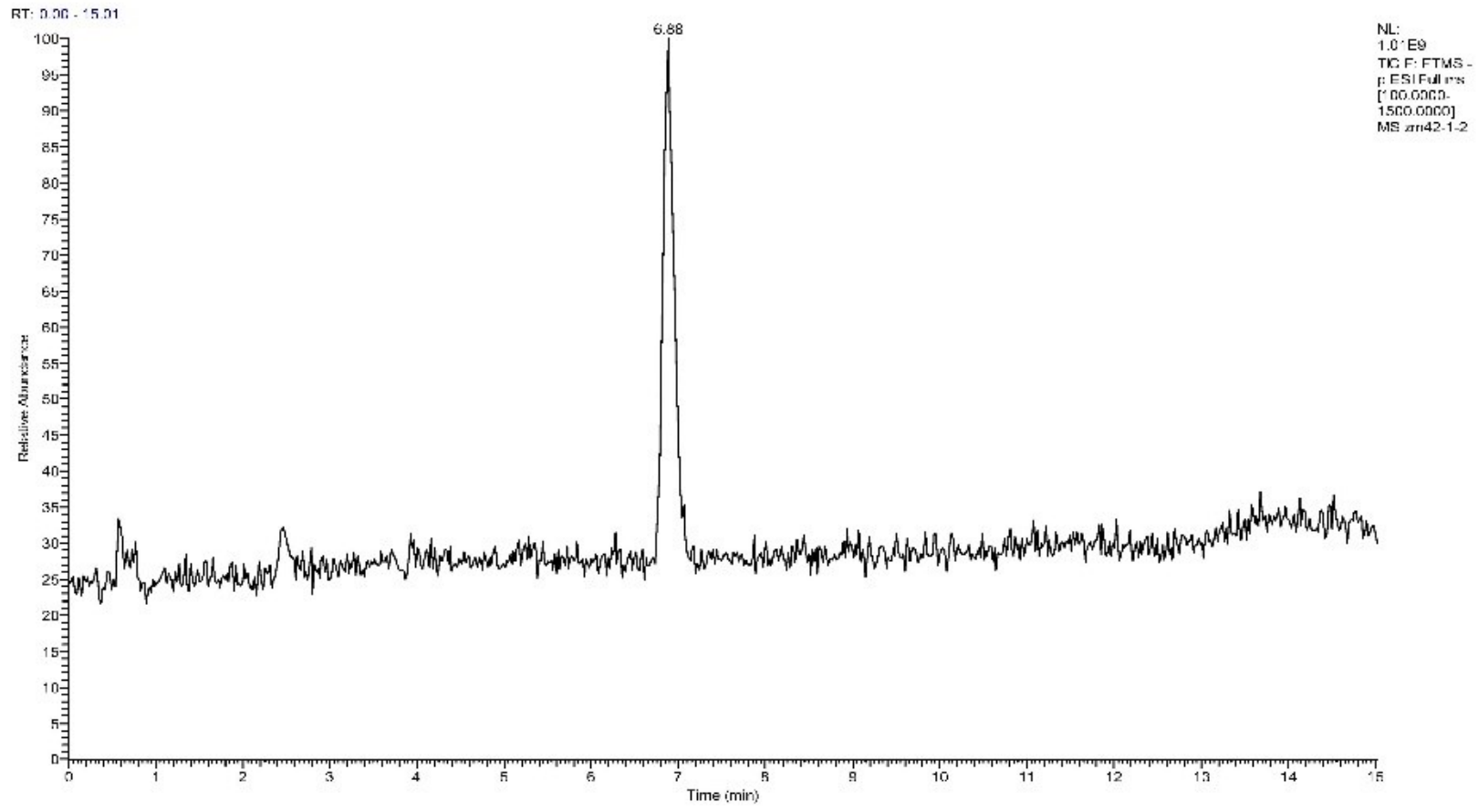

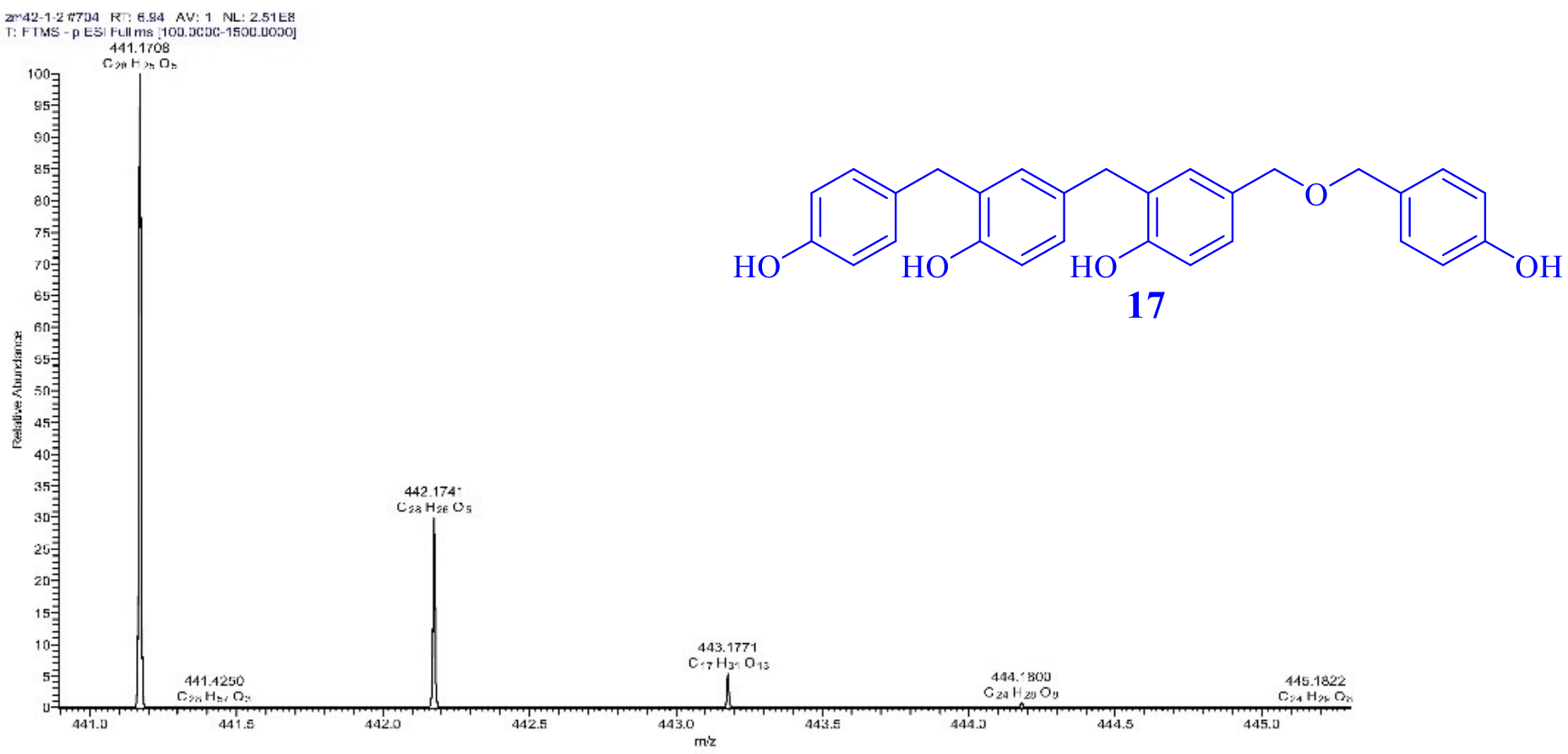

17

m/z	Theo. Mass	Delta (mmu)	RDB equiv.	Composition
441.1708	441.1697	1.16	16.5	C28 H25 O5

Figure S207. The HR-ESIMS report of compound **17**

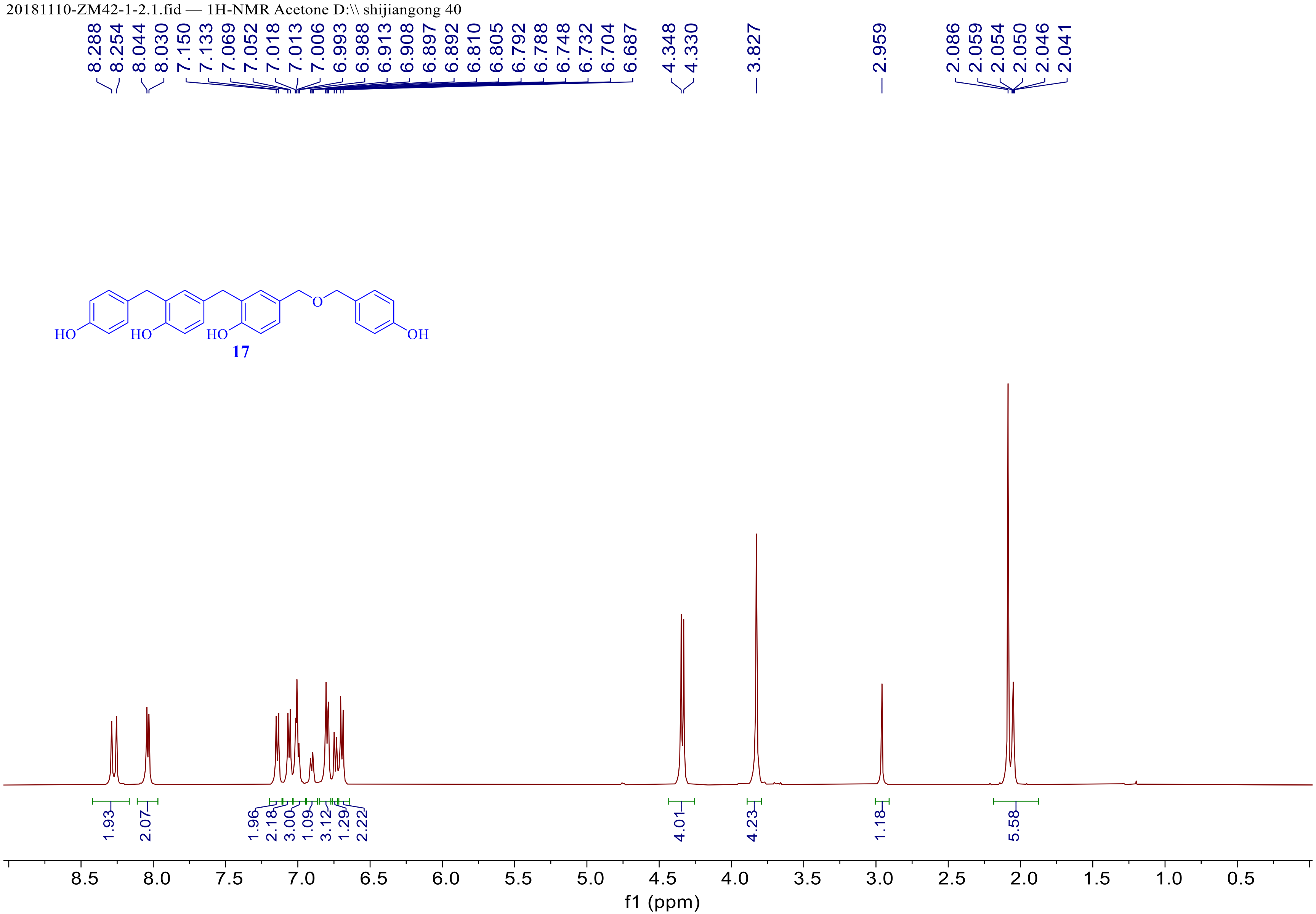

Figure S208. The ^{1}H NMR spectrum of compound **17** in acetone-d_6 (600 MHz)

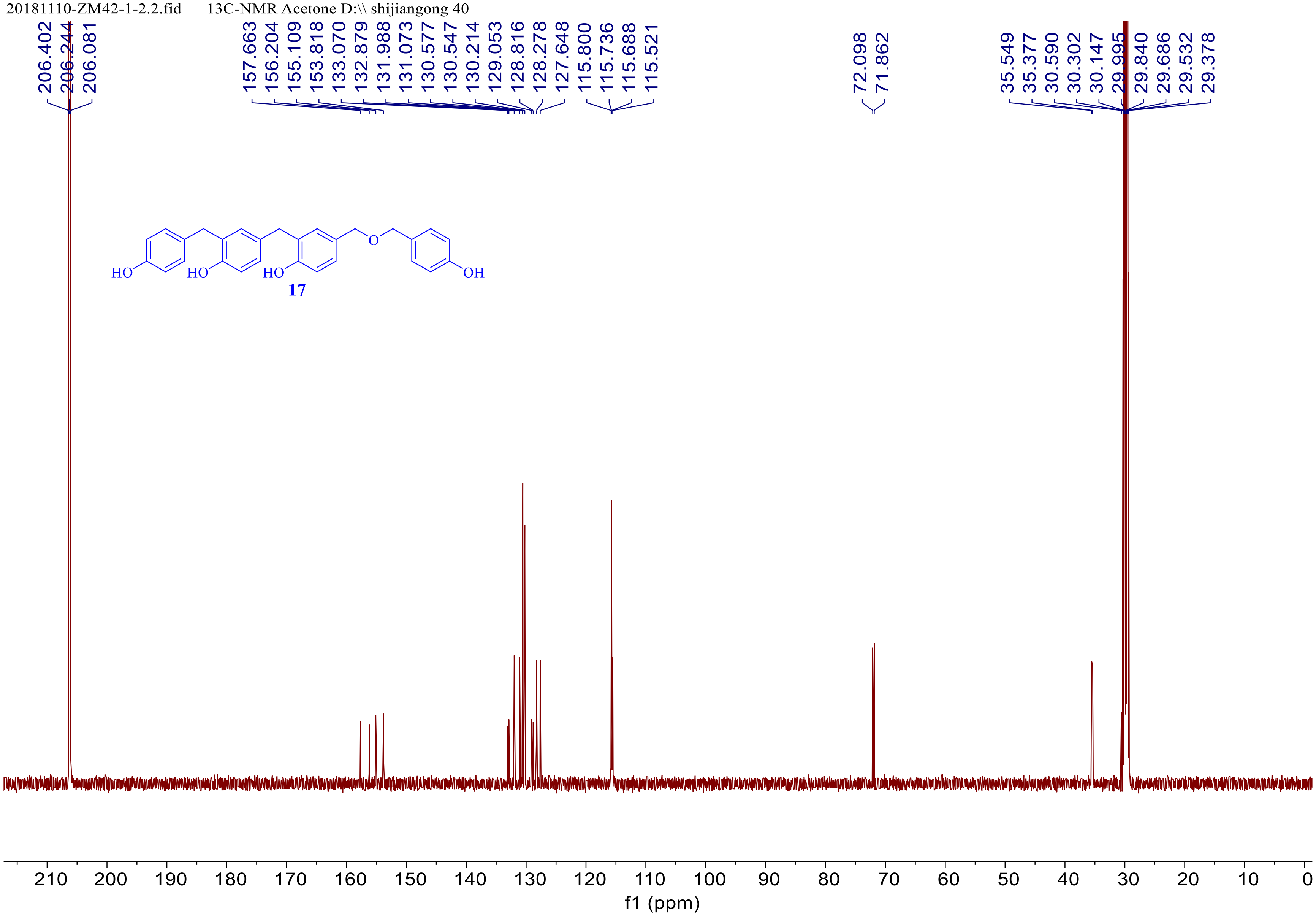

Figure S209. The ^{13}C NMR spectrum of compound **17** in acetone-d_6 (150 MHz)

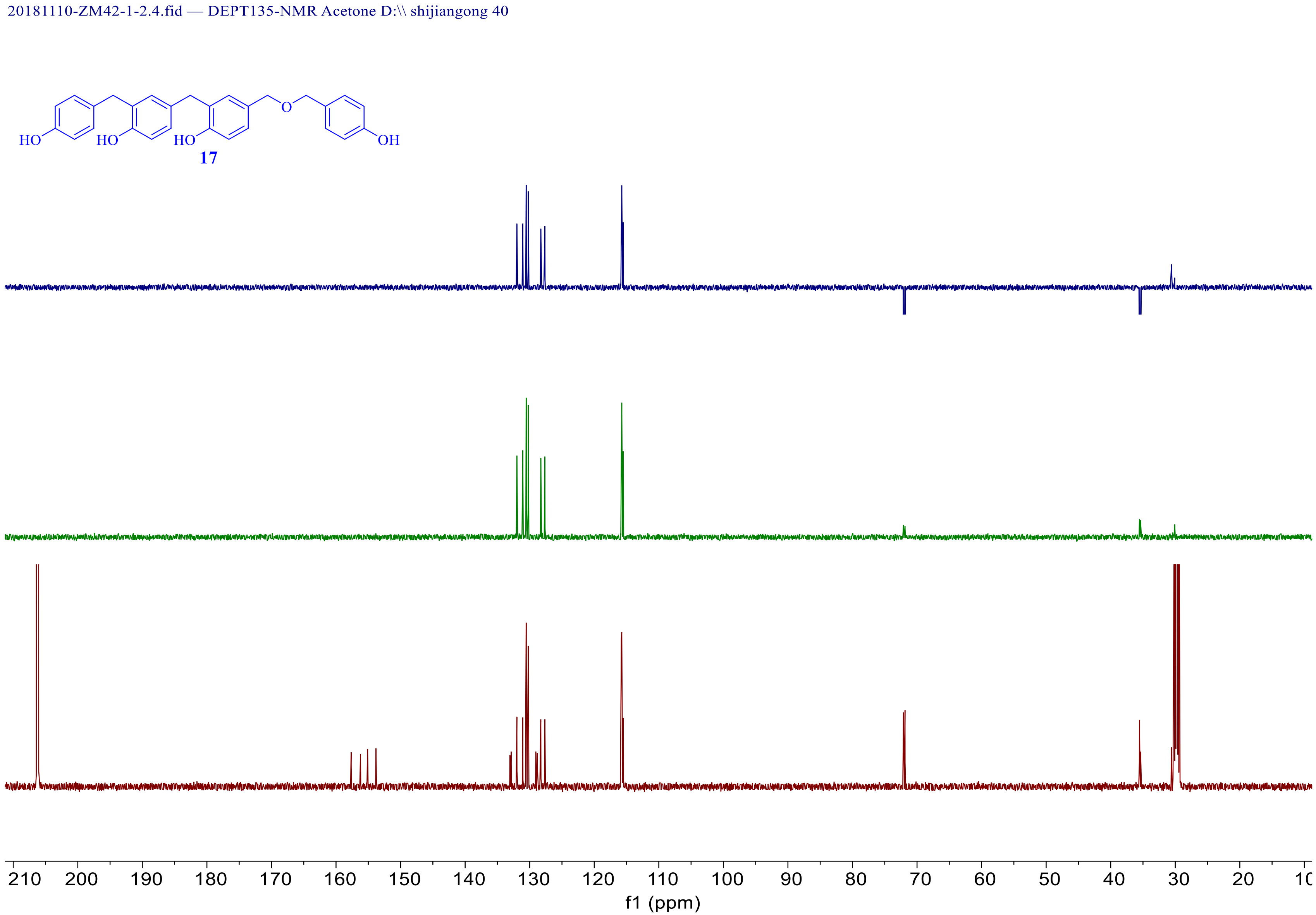

Figure S210. The DEPT spectrum of compound **17** in acetone-d_6 (150 MHz)

20181110-ZM42-1-2.6.ser — COSYGPMFSW Acetone D:\\ shijiangong 40

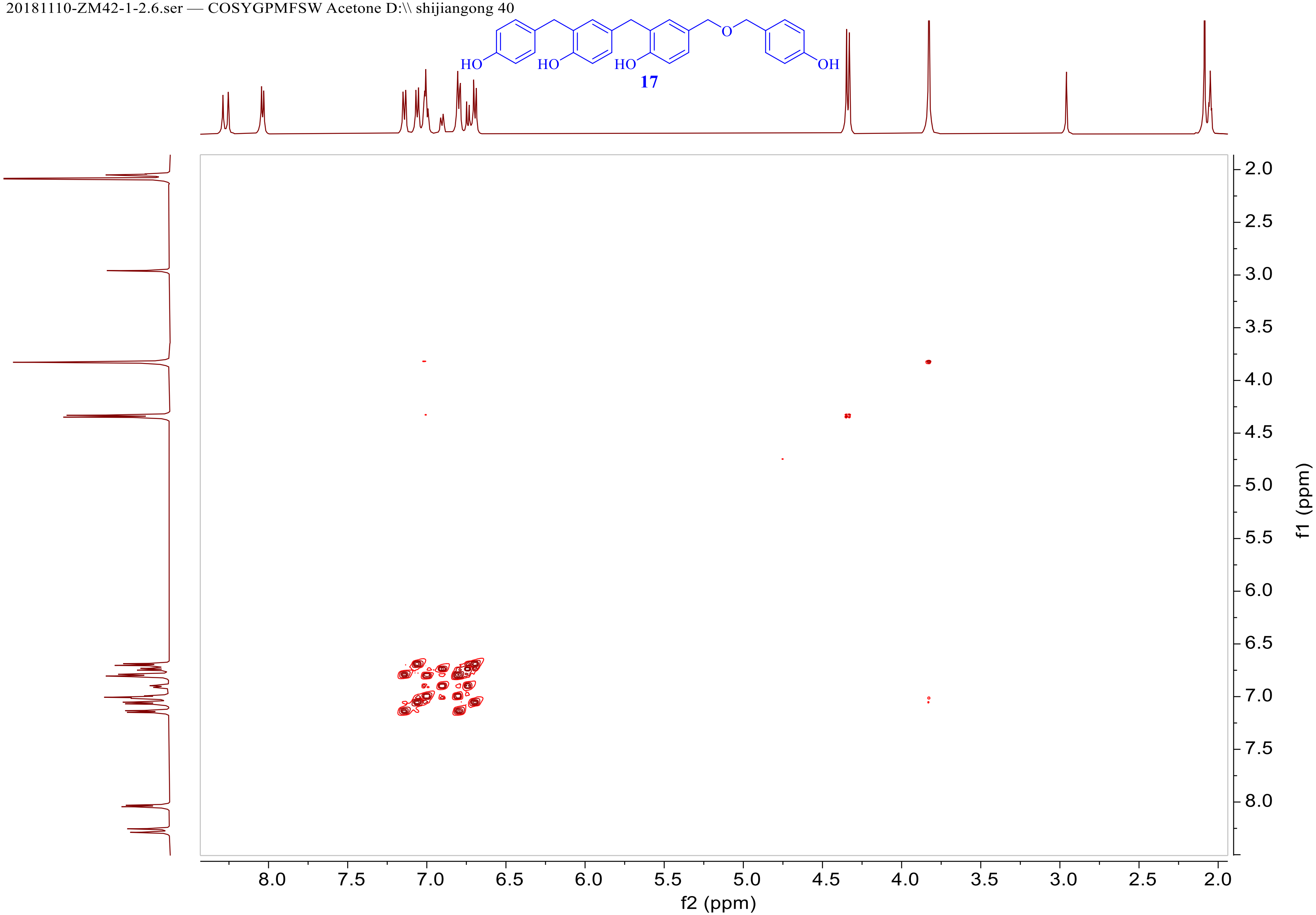

Figure S211. The ^{1}H-^{1}H COSY spectrum of compound **17** in acetone-d_6 (600 MHz)

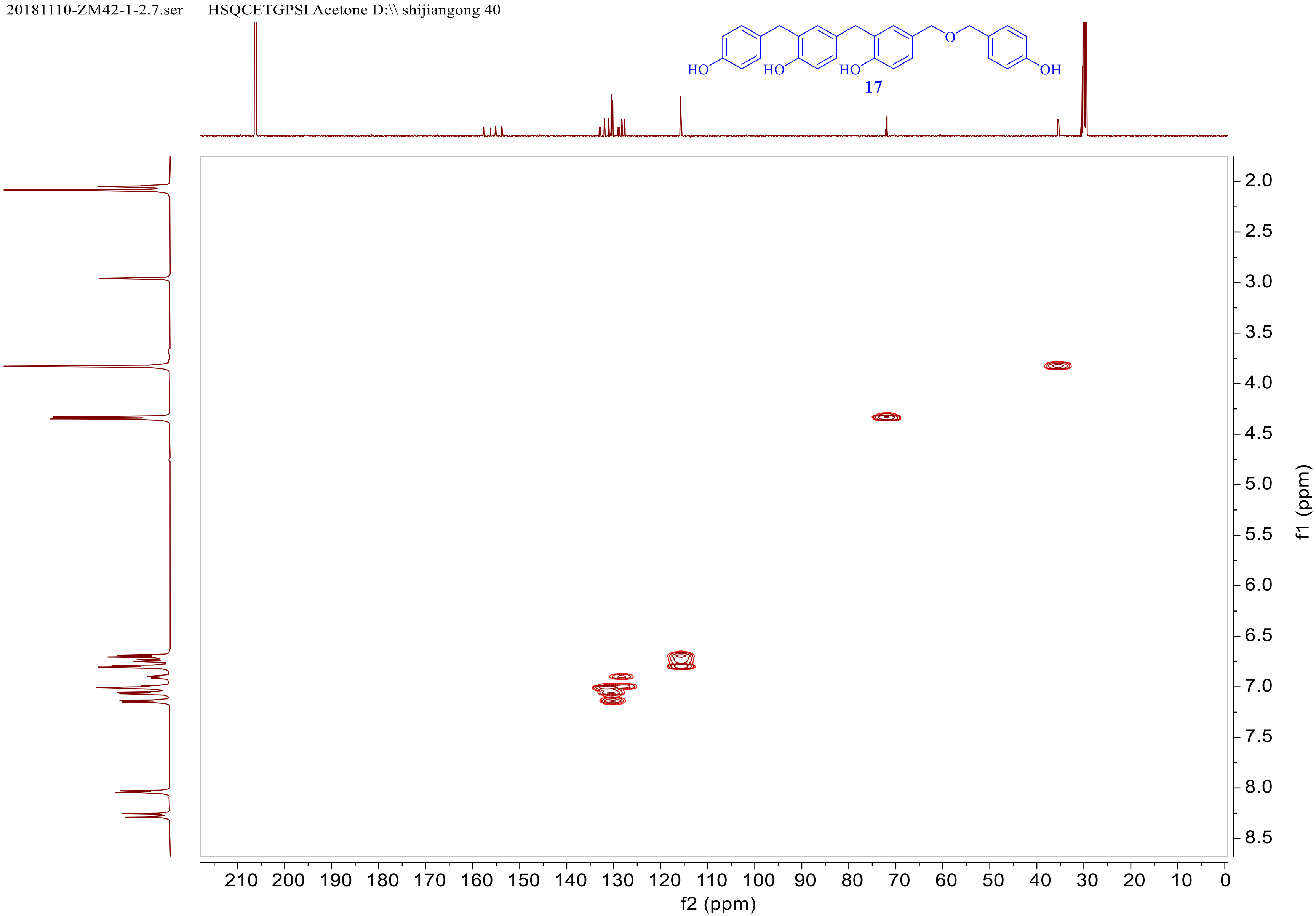

Figure S212. The HSQC spectrum of compound **17** in acetone-d_6 (600 MHz for ^{1}H)

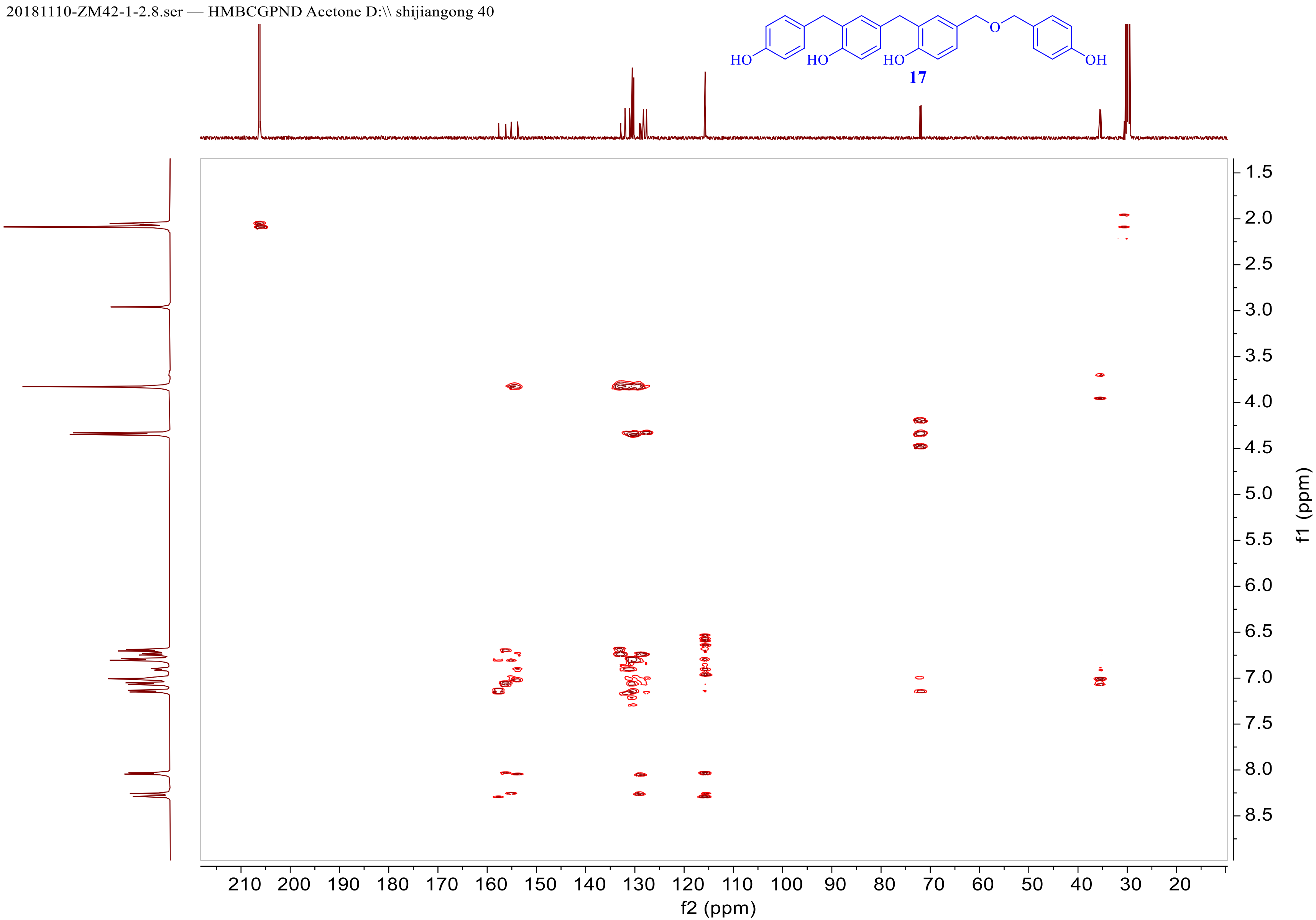

Figure S213. The HMBC spectrum of compound **17** in acetone-d_6 (600 MHz for ^{1}H)

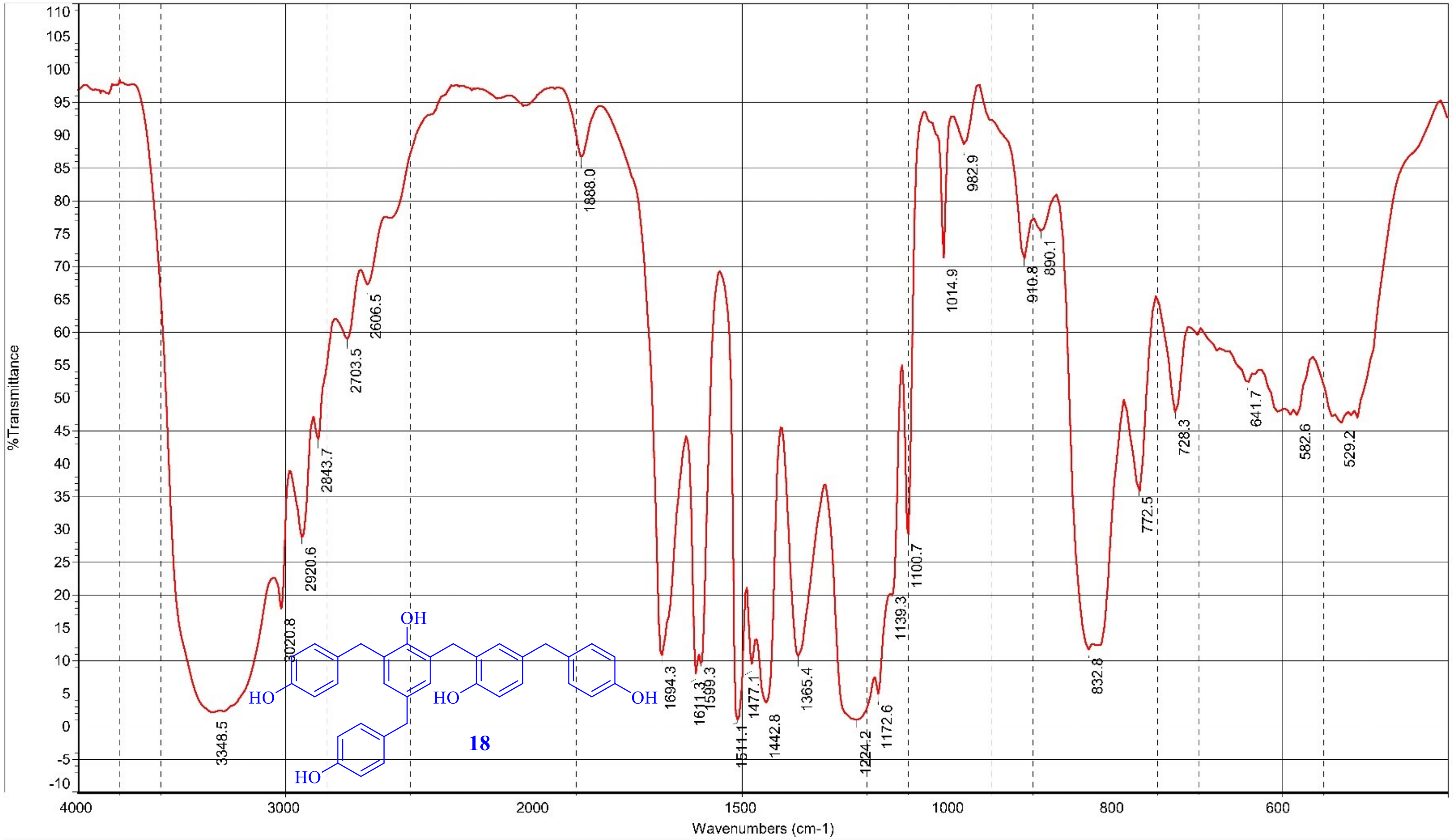

日期: 星期 : 12月 15 16:03:14 2020 (GMT+08:00Sample Name： ZM-33 (显微镜透射法FT- IR Microscope Transmission)

扫描次数: 100

分辨率: 8.000

Figure S214. The IR spectrum of compound **18**

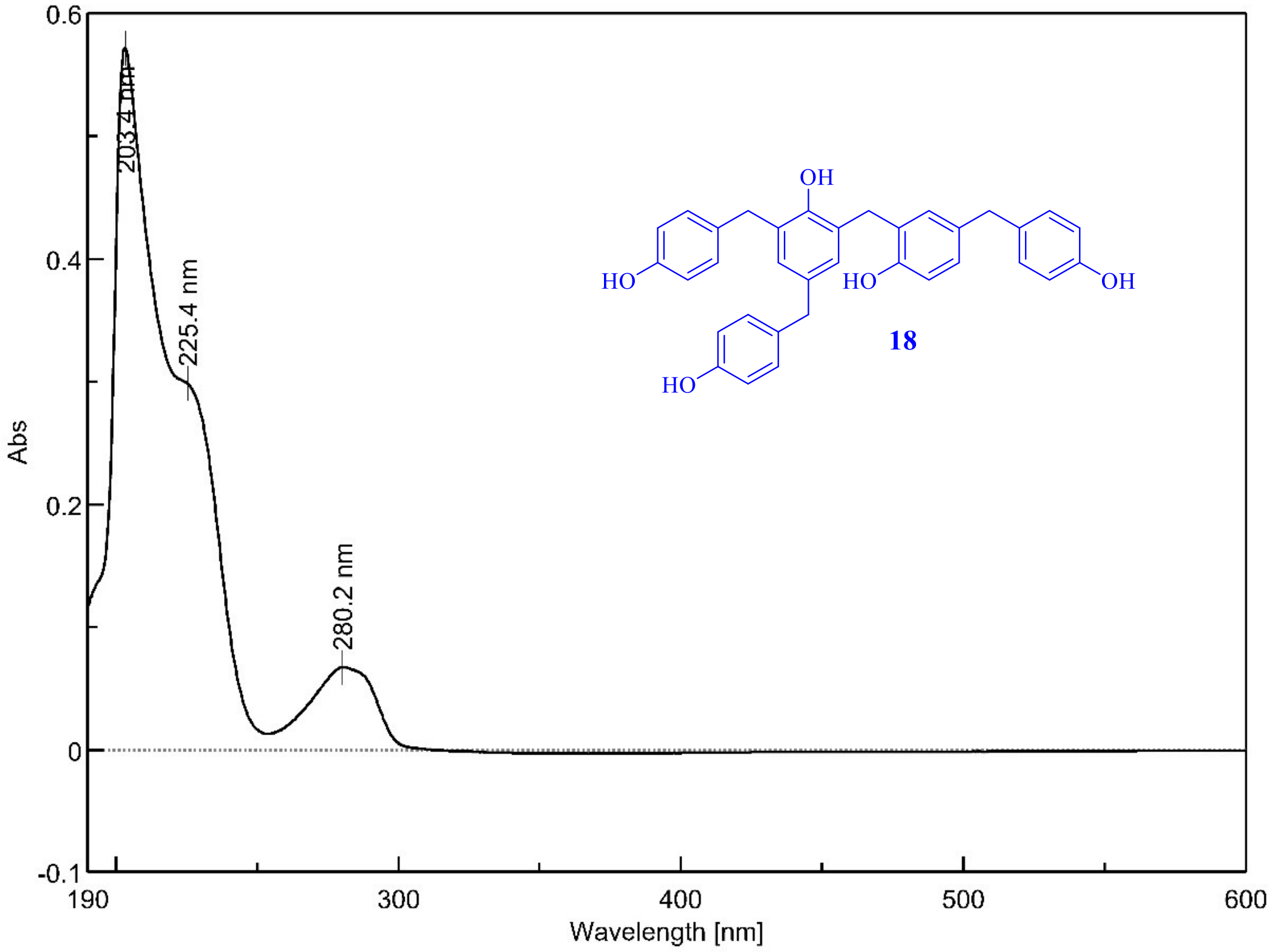

[Comment]
Sample Name DBT
Comment 0.02
User
Division UV
Company 324

ZM-33

[Measurement Information]
Instrument Name V-650
Model Name V-650
Serial No. A034461150

Accessory PSC-718
Accessory S/N A001761114
Position 1
Cell Length 10 mm
Temperature 20.00 C
Control Sonsor Holder
Monitor Sensor Holder
Start Mode Start immediately

Photometric Mode Abs
Measurement range 600 - 190 nm
Data pitch 0.2 nm
Band width(UV/Vis) 2.0 nm
Response Medium
Scanning speed 200 nm/min
Source Change 340 nm
Light Source D2/WI
Filter Exchange Step
Correction Baseline

[Data Information]
Creation Date 2020-11-11 16:08

Data array type Linear data array
Horizontal Wavelength [nm]
Vertical Abs
Start 600 nm
End 190 nm
Data pitch 0.2 nm
Data points 2051

Figure S215. The UV spectrum of compound **18** in MeOH

Thermo Qexactive Focus Report

compound NO. : ZM-33
Method : LCMS(compound)-low

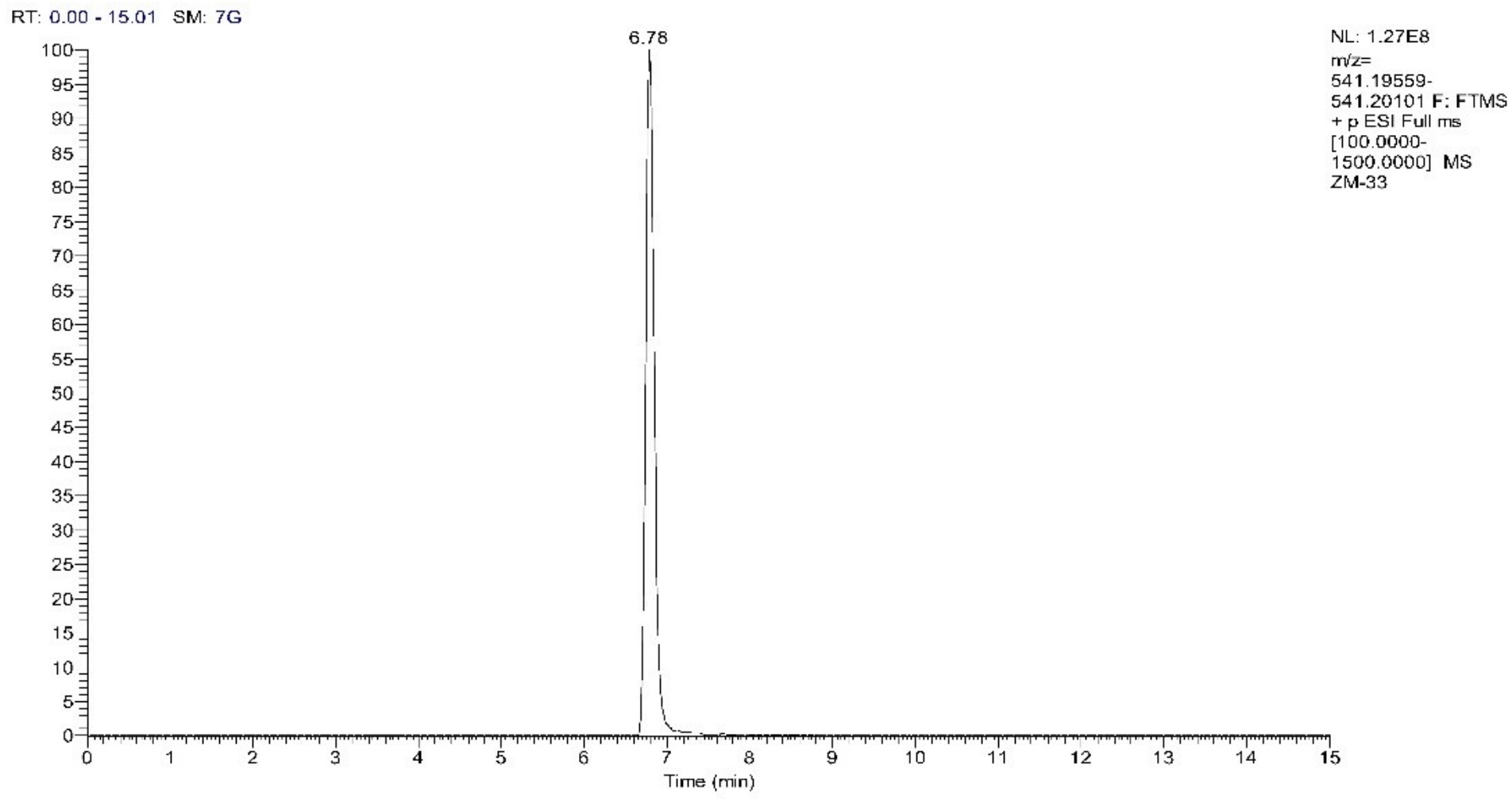

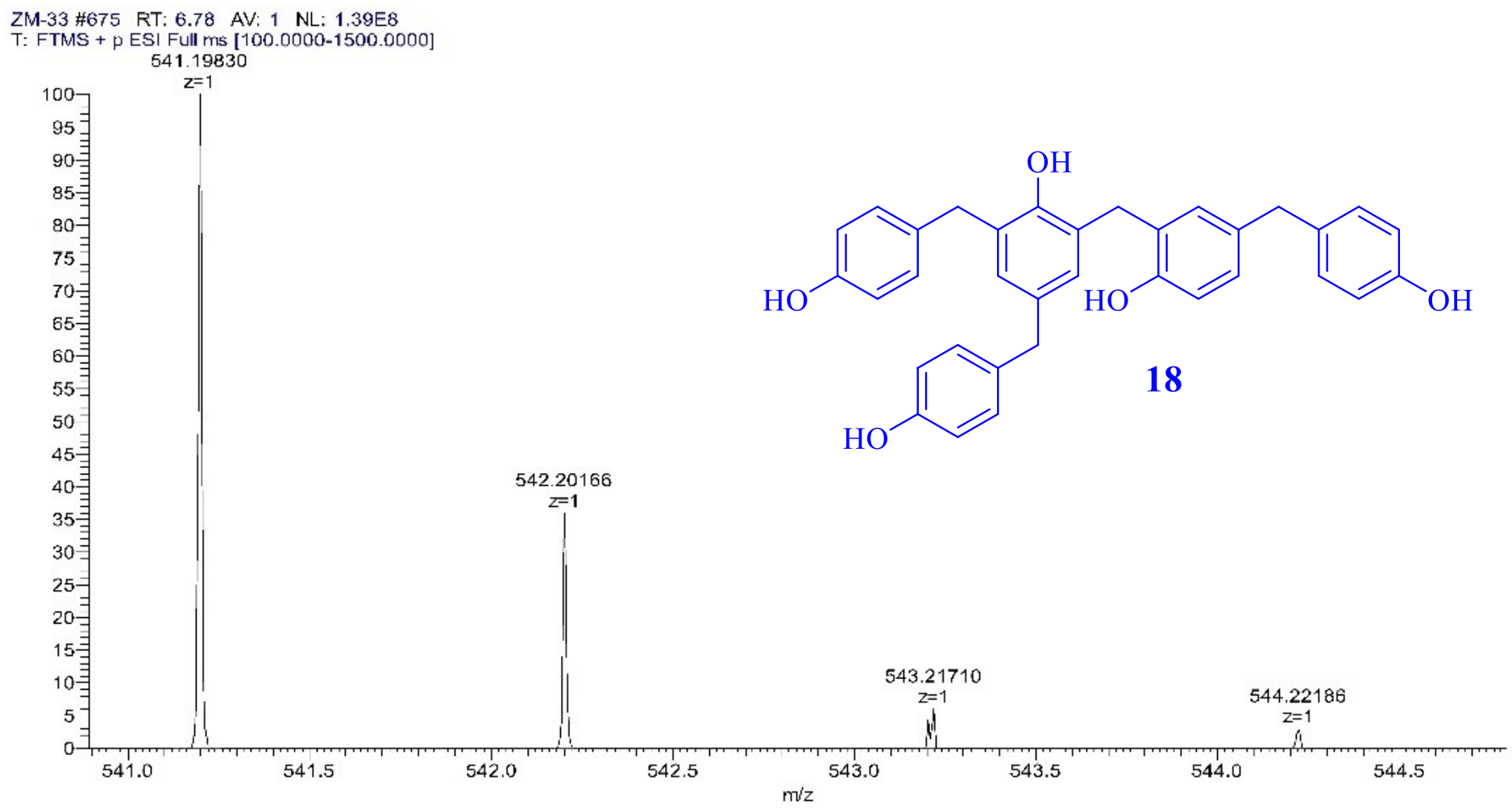

m/z	Theo. Mass	Delta (ppm)	RDB equiv.	Composition	
541.19830	541.19855	-0.45	19.5	C34 H30 O5 Na	M+Na

Figure S216. The HR-ESIMS report of compound **18**

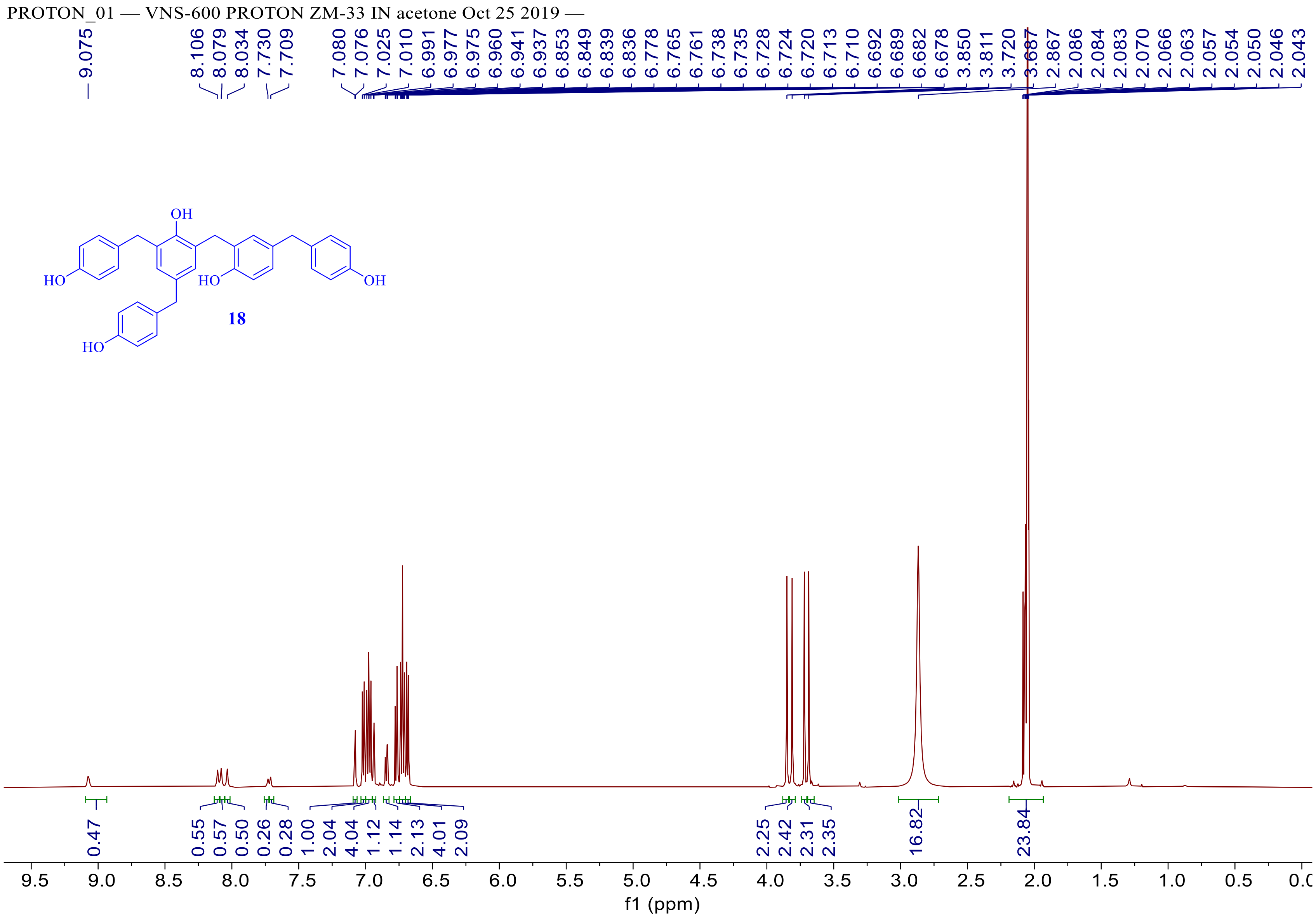

Figure S217. The ^1H NMR spectrum of compound **18** in acetone-d_6 (600 MHz)

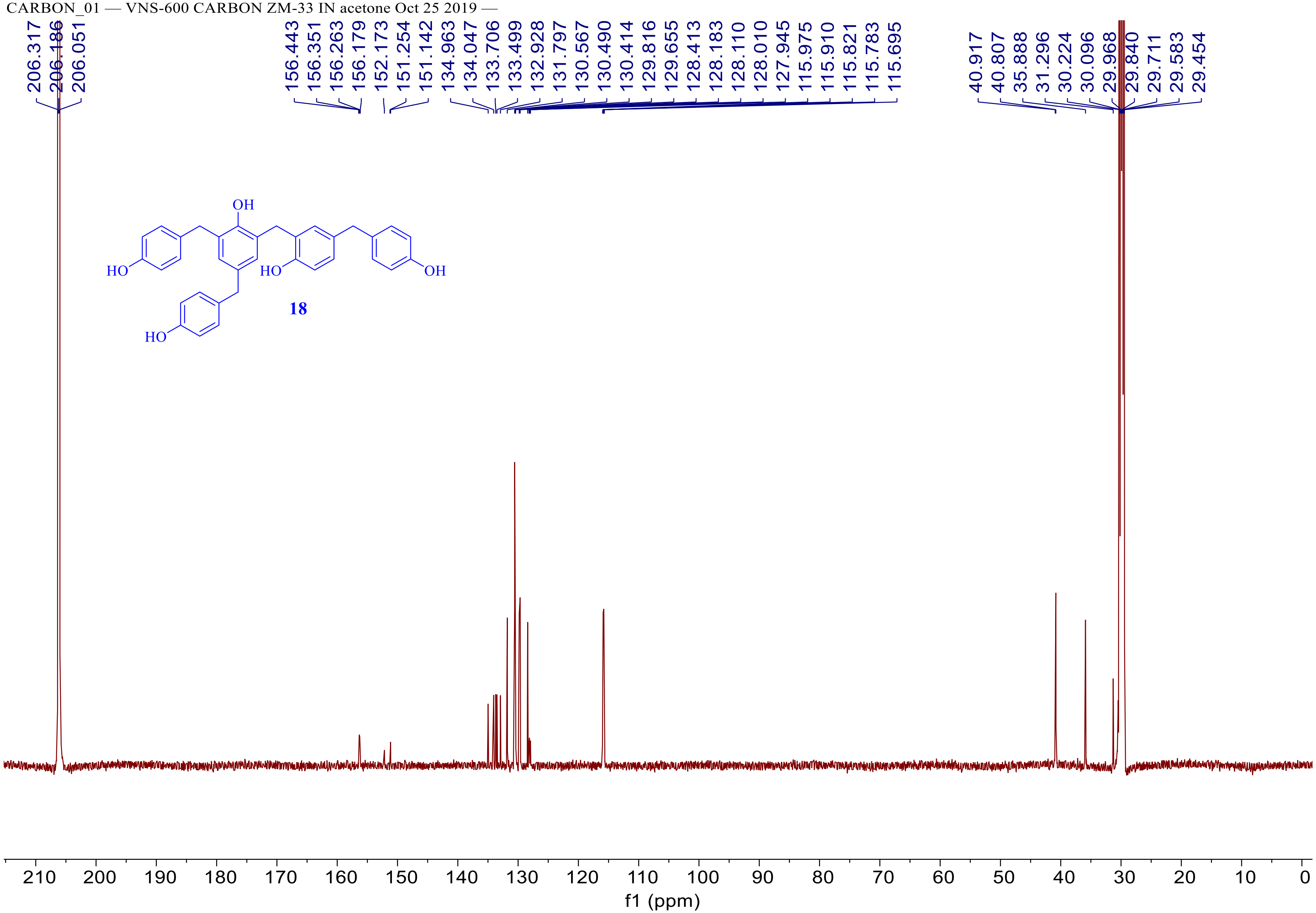

Figure S218. The ^{13}C NMR spectrum of compound **18** in acetone-d_6 (150 MHz)

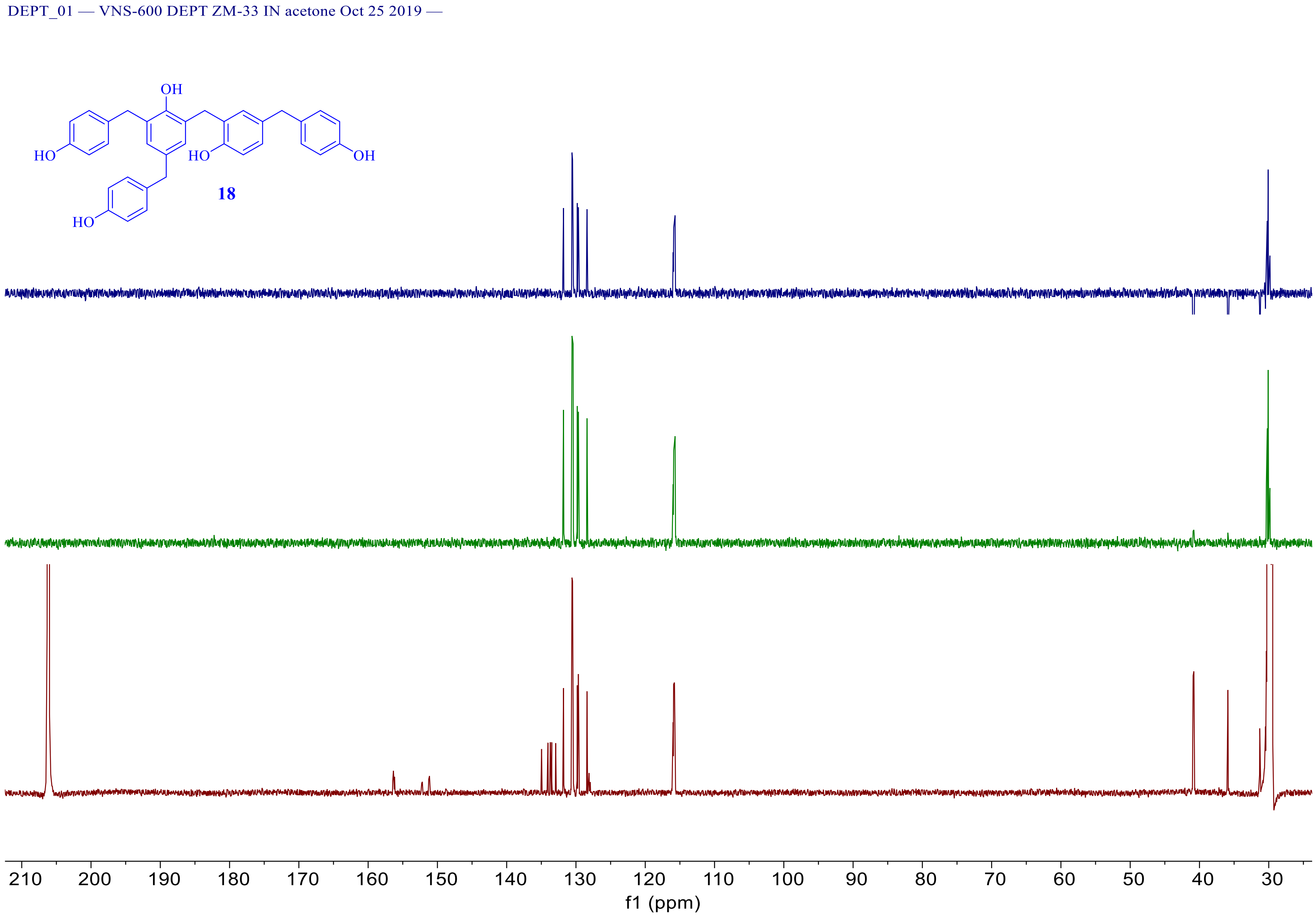

Figure S219. The DEPT spectrum of compound **18** in acetone-d_6 (150 MHz)

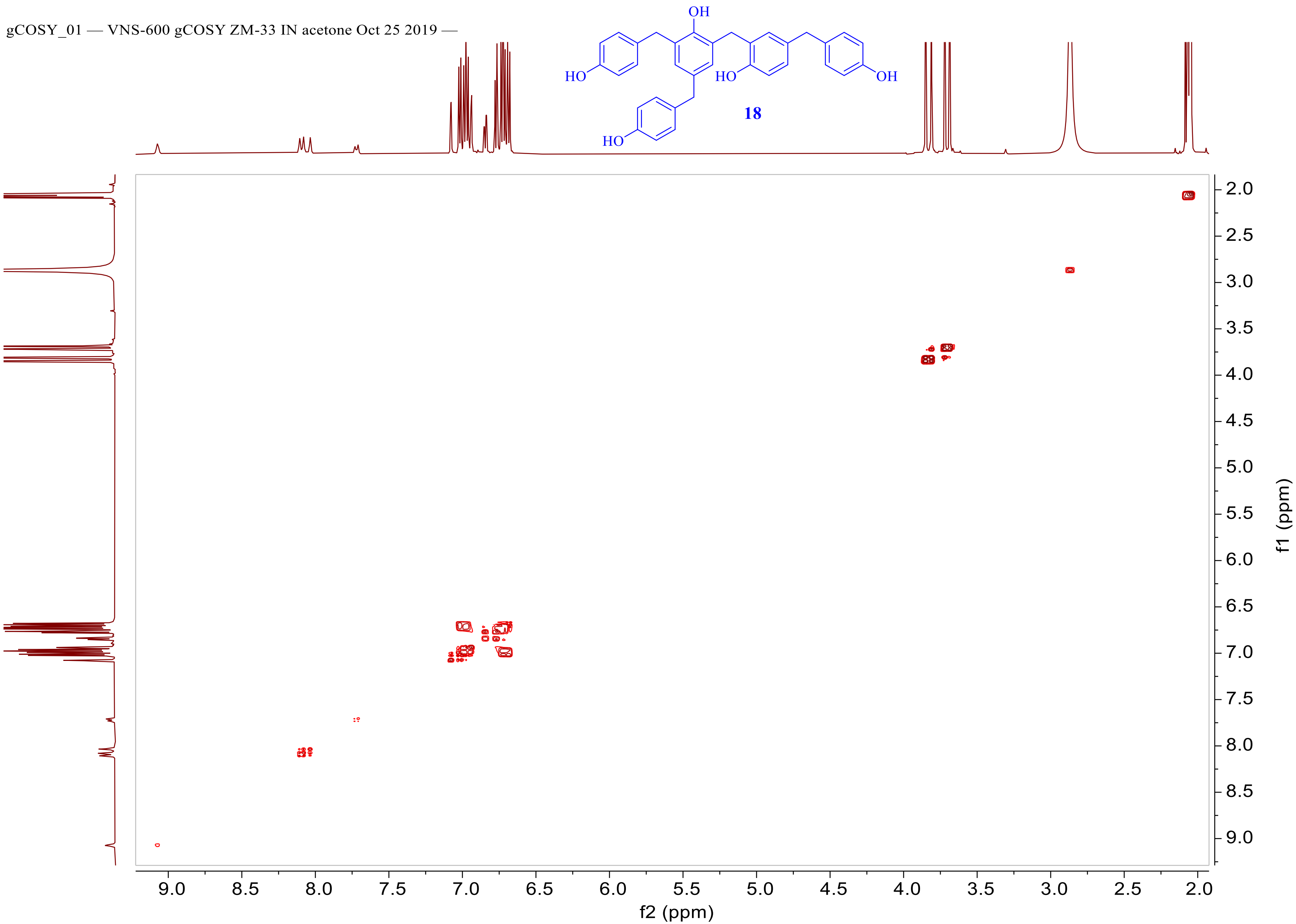

Figure S220. The ^{1}H-^{1}H COSY spectrum of compound **18** in acetone-d_6 (600 MHz)

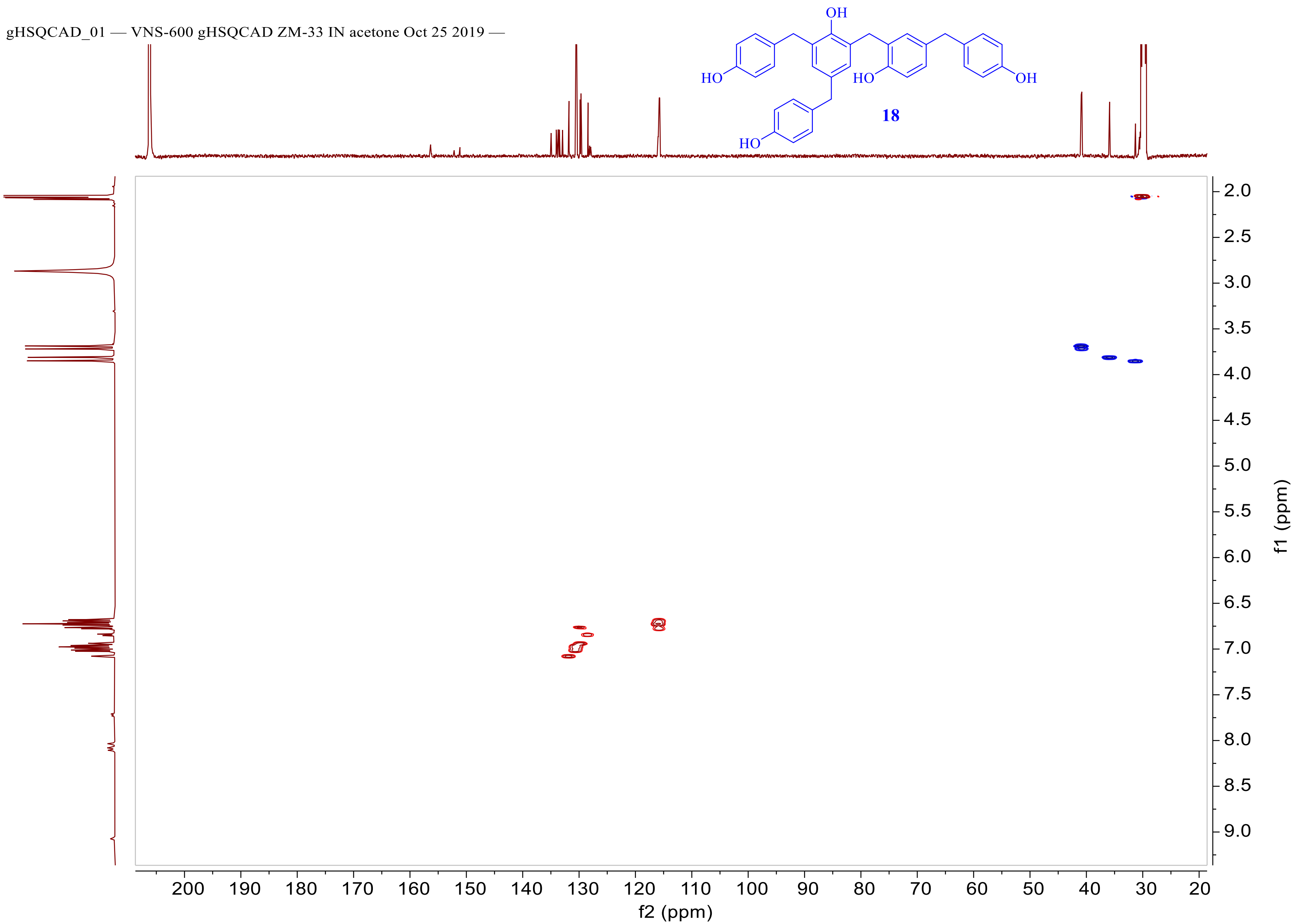

Figure S221. The HSQC spectrum of compound **18** in acetone-d_6 (600 MHz for ^{1}H)

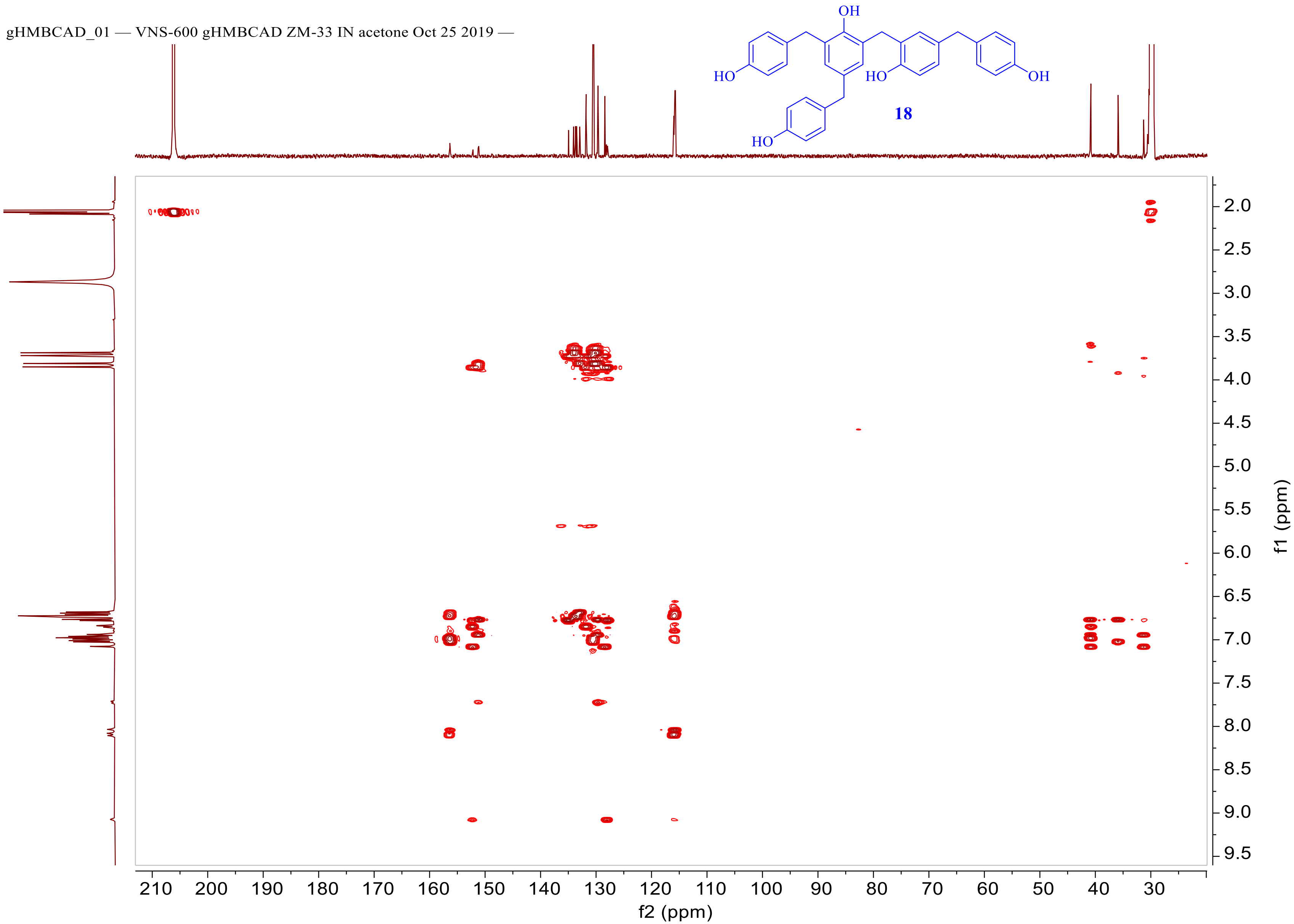

Figure S222. The HMBC spectrum of compound **18** in acetone-d_6 (600 MHz for ^{1}H)

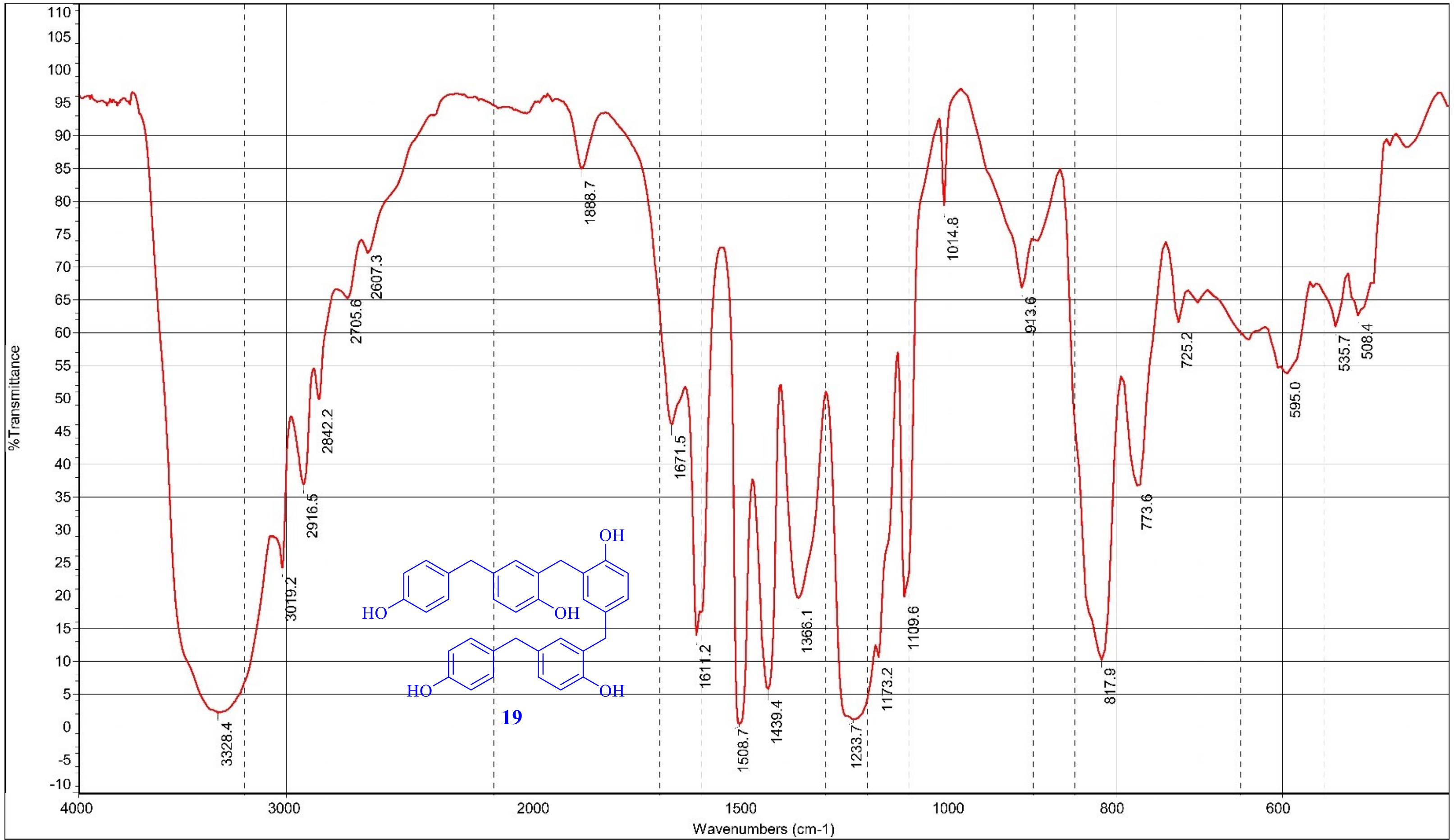

日期: 星期三 12月 02 15:10:56 2020 (GMT+08:00Sample Name ： ZM-30 (显微镜透射法FT- IR Microscope Transmission)

扫描次数: 100

分辨率: 8.000

Figure S223. The IR spectrum of compound **19**

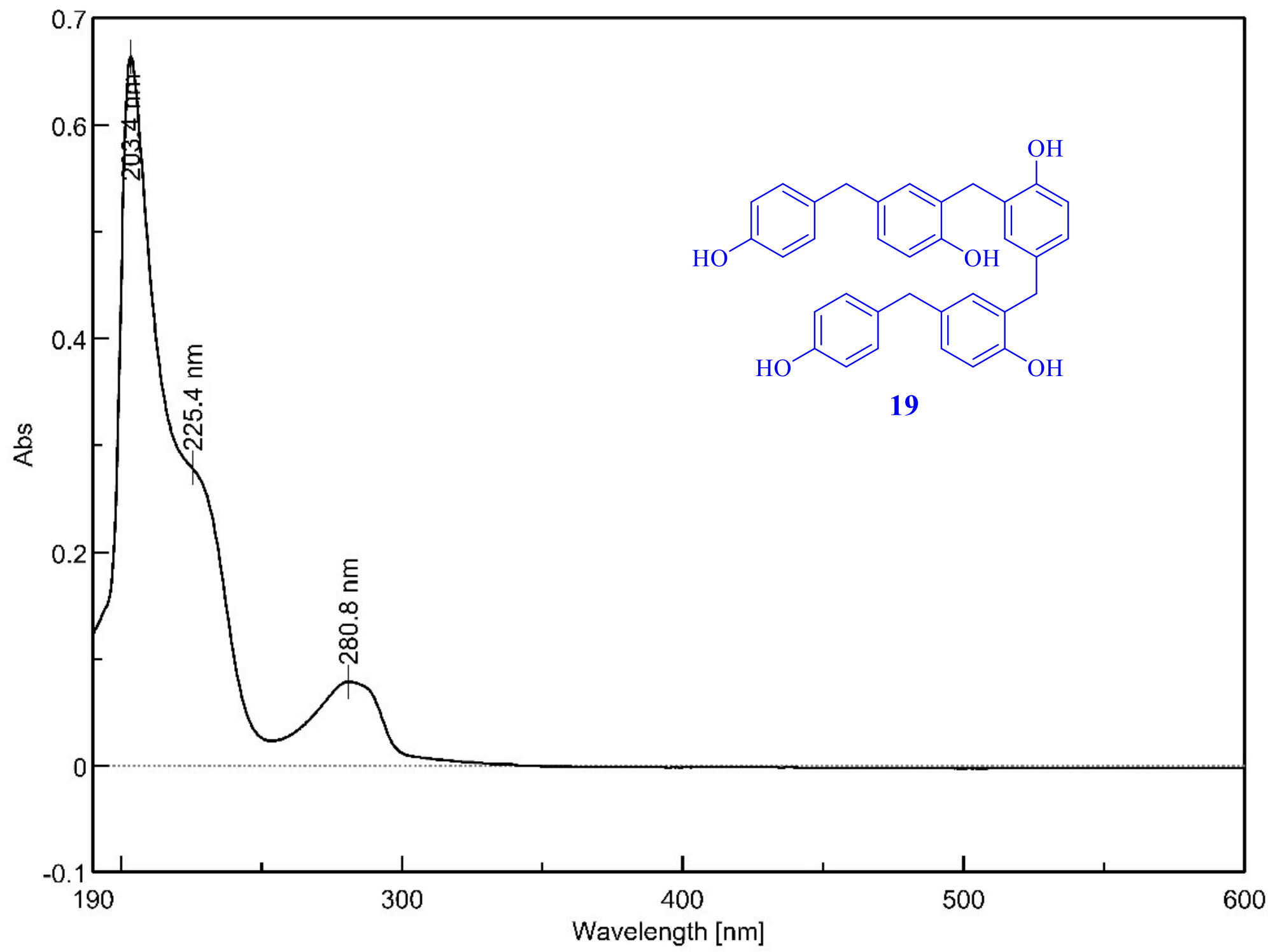

[Comment]
Sample Name DBT
Comment 0.02
User
Division UV
Company 324

ZM-30

[Measurement Information]
Instrument Name V-650
Model Name V-650
Serial No. A034461150

Accessory PSC-718
Accessory S/N A001761114
Position 1
Cell Length 10 mm
Temperature 19.99 C
Control Sonsor Holder
Monitor Sensor Holder
Start Mode Start immediately

Photometric Mode Abs
Measurement range 600 - 190 nm
Data pitch 0.2 nm
Band width(UV/Vis) 2.0 nm
Response Medium
Scanning speed 200 nm/min
Source Change 340 nm
Light Source D2/WI
Filter Exchange Step
Correction Baseline

[Data Information]
Creation Date 2020-11-11 15:56

Data array type Linear data array
Horizontal Wavelength [nm]
Vertical Abs
Start 600 nm
End 190 nm
Data pitch 0.2 nm
Data points 2051

Figure S224. The UV spectrum of compound **19** in MeOH

Thermo Qexactive Focus Report

compound NO. : ZM-35
Method : LCMS(compound)-low

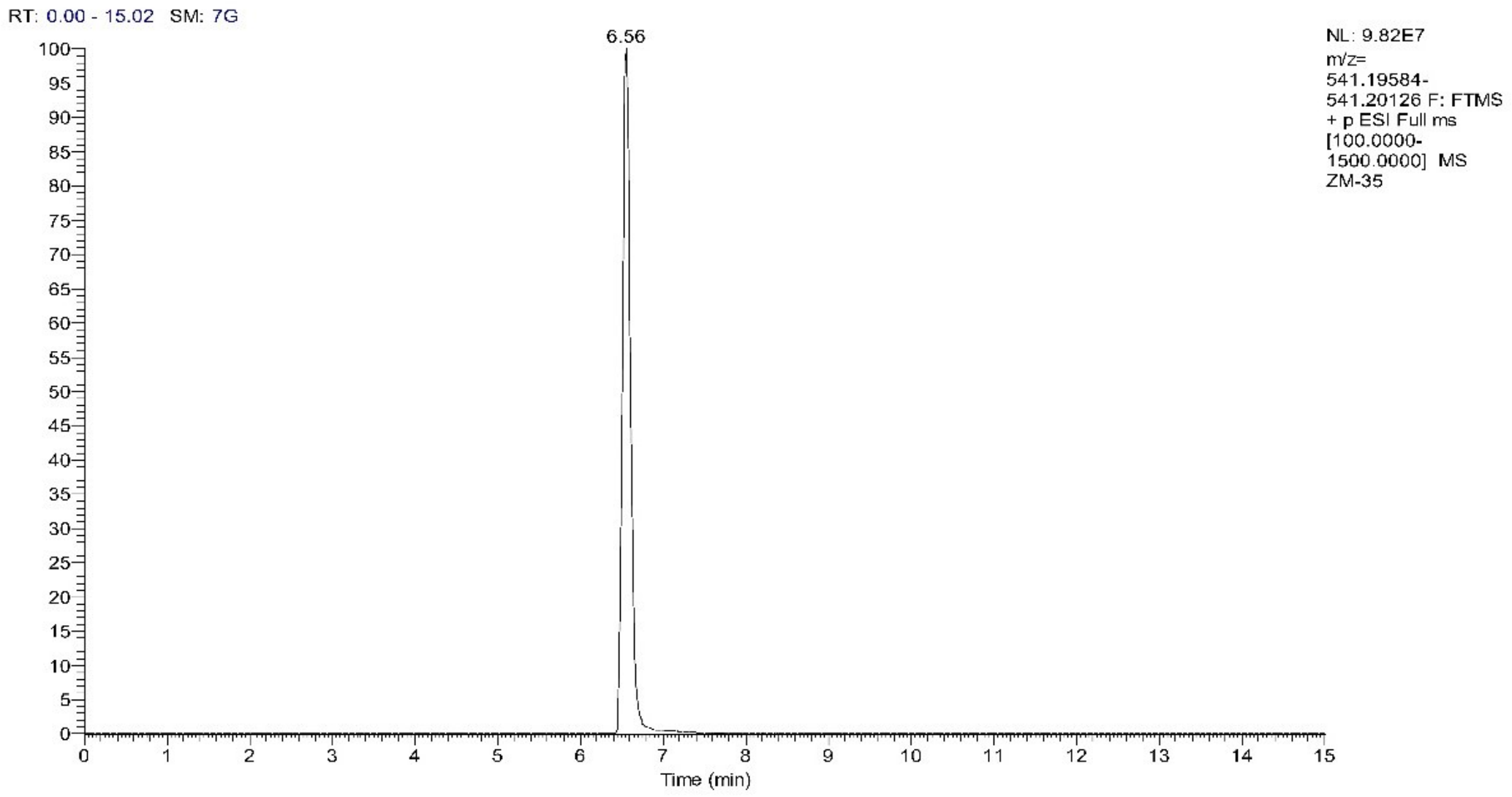

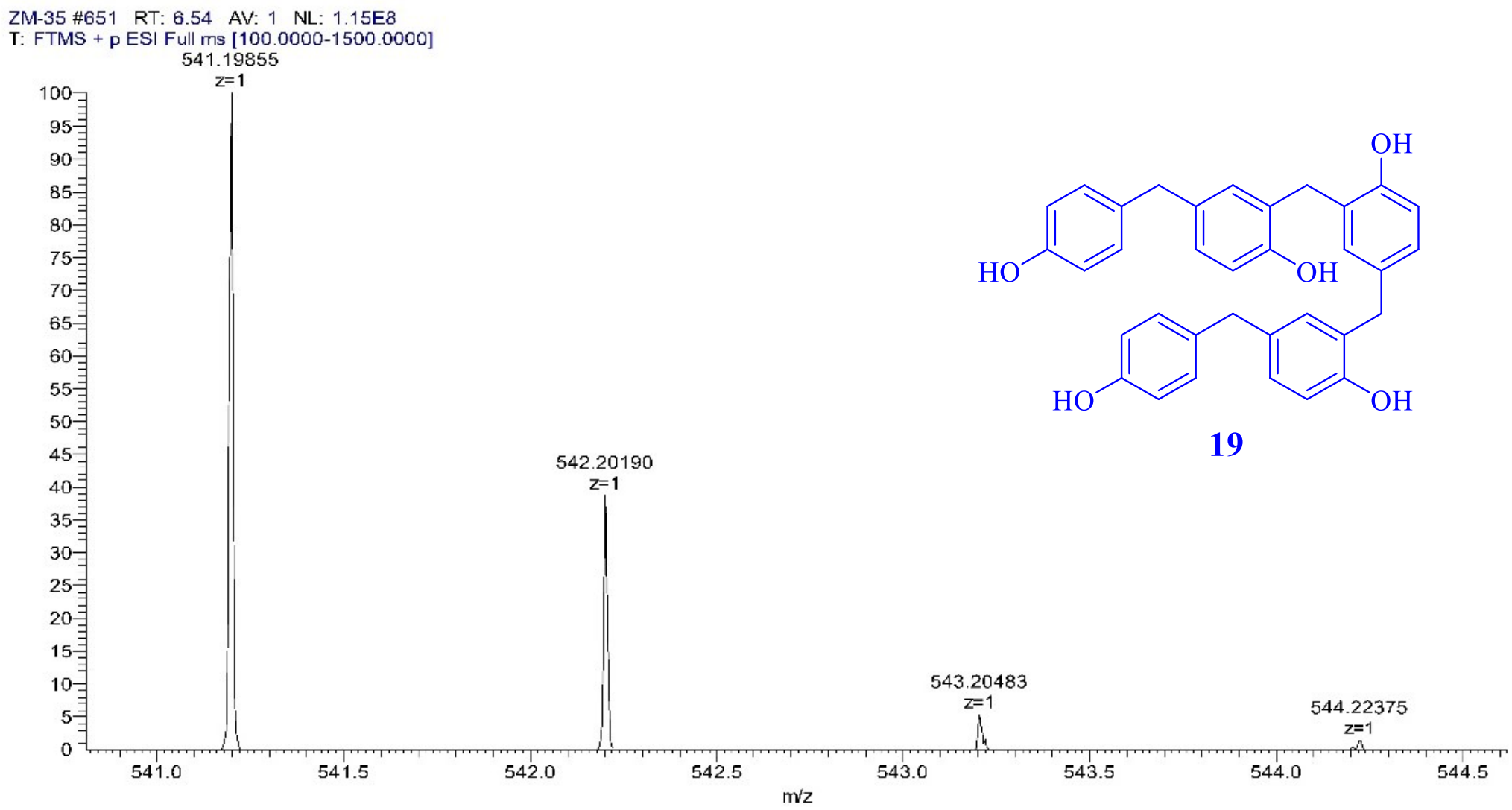

m/z	Theo. Mass	Delta (ppm)	RDB equiv.	Composition	
541.19855	541.19855	0.01	19.5	C34 H30 O5 Na	M+Na

Figure S225. The HR-ESIMS report of compound **19**

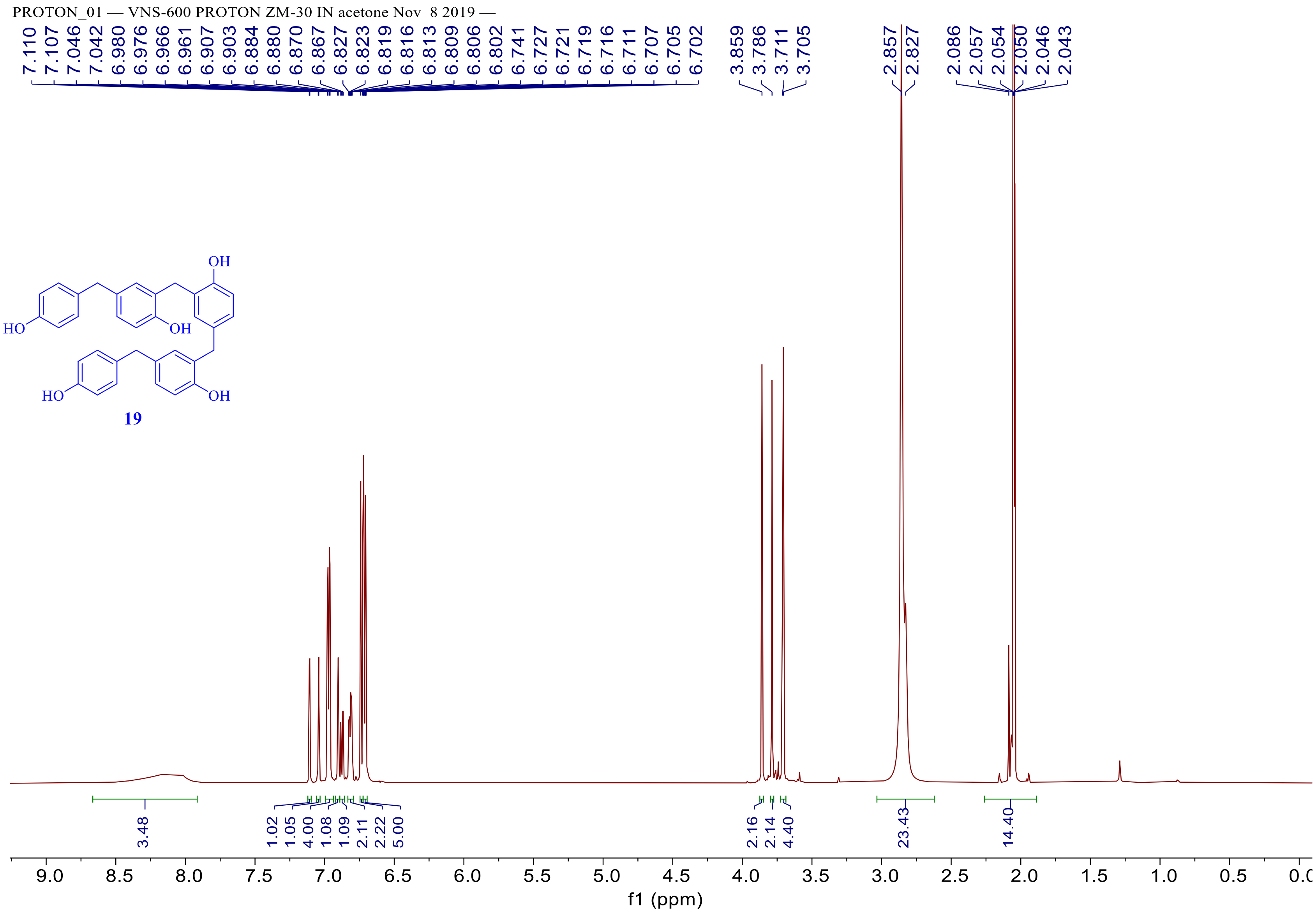

Figure S226. The ^{1}H NMR spectrum of compound **19** in acetone-d_6 (600 MHz)

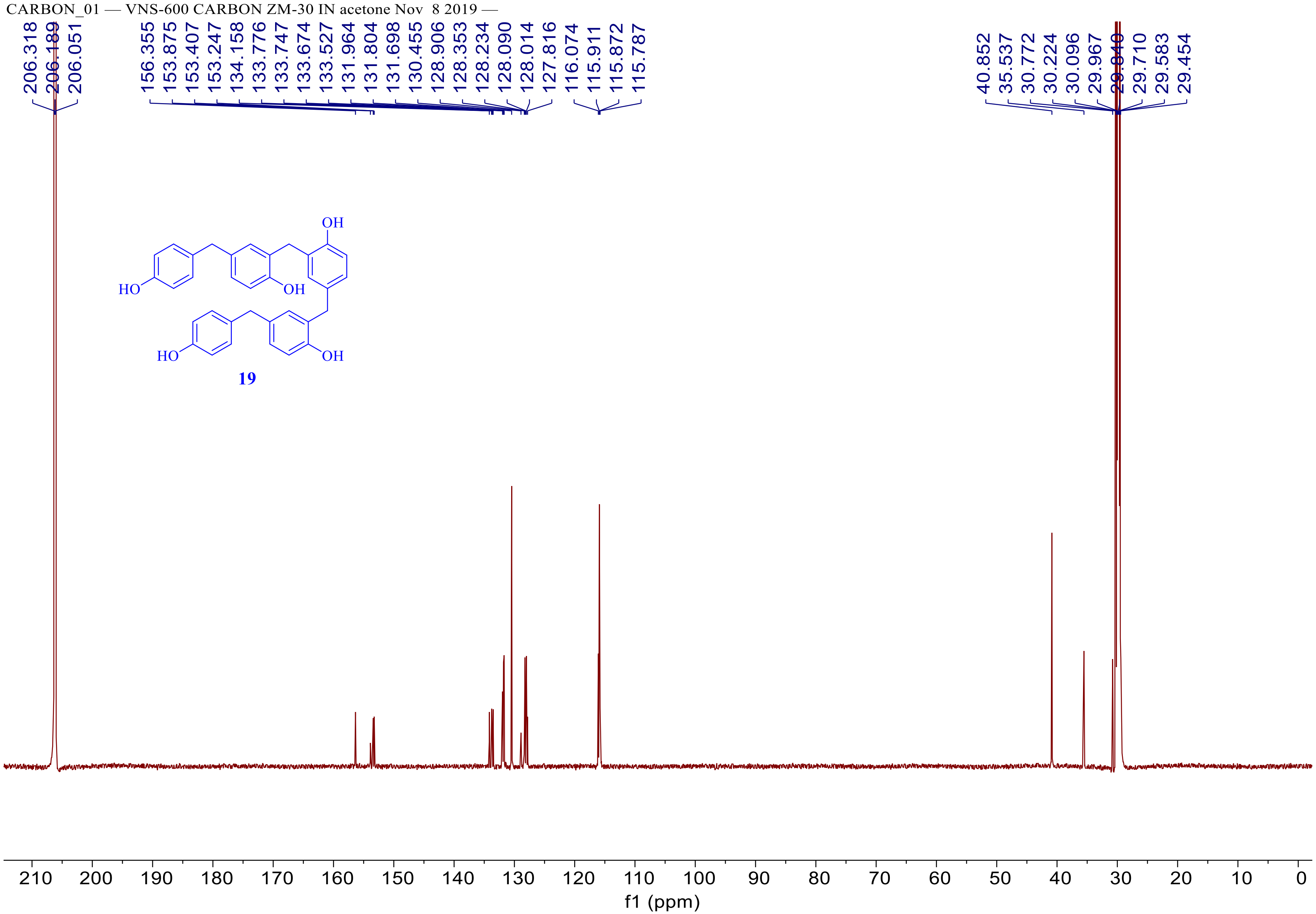

Figure S227. The ^{13}C NMR spectrum of compound **19** in acetone-d_6 (150 MHz)

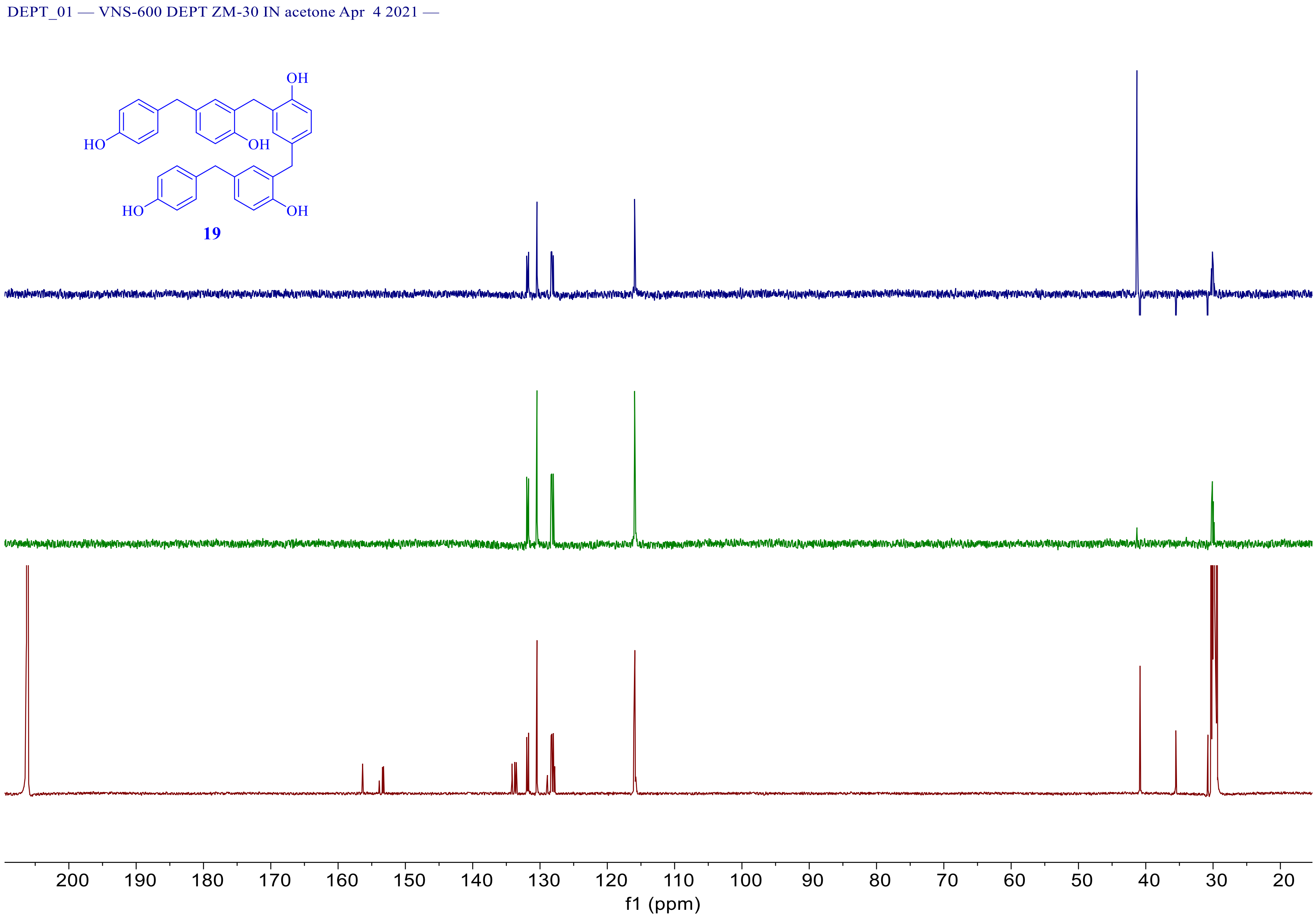

Figure S228. The DEPT spectrum of compound **19** in acetone-d_6 (150 MHz)

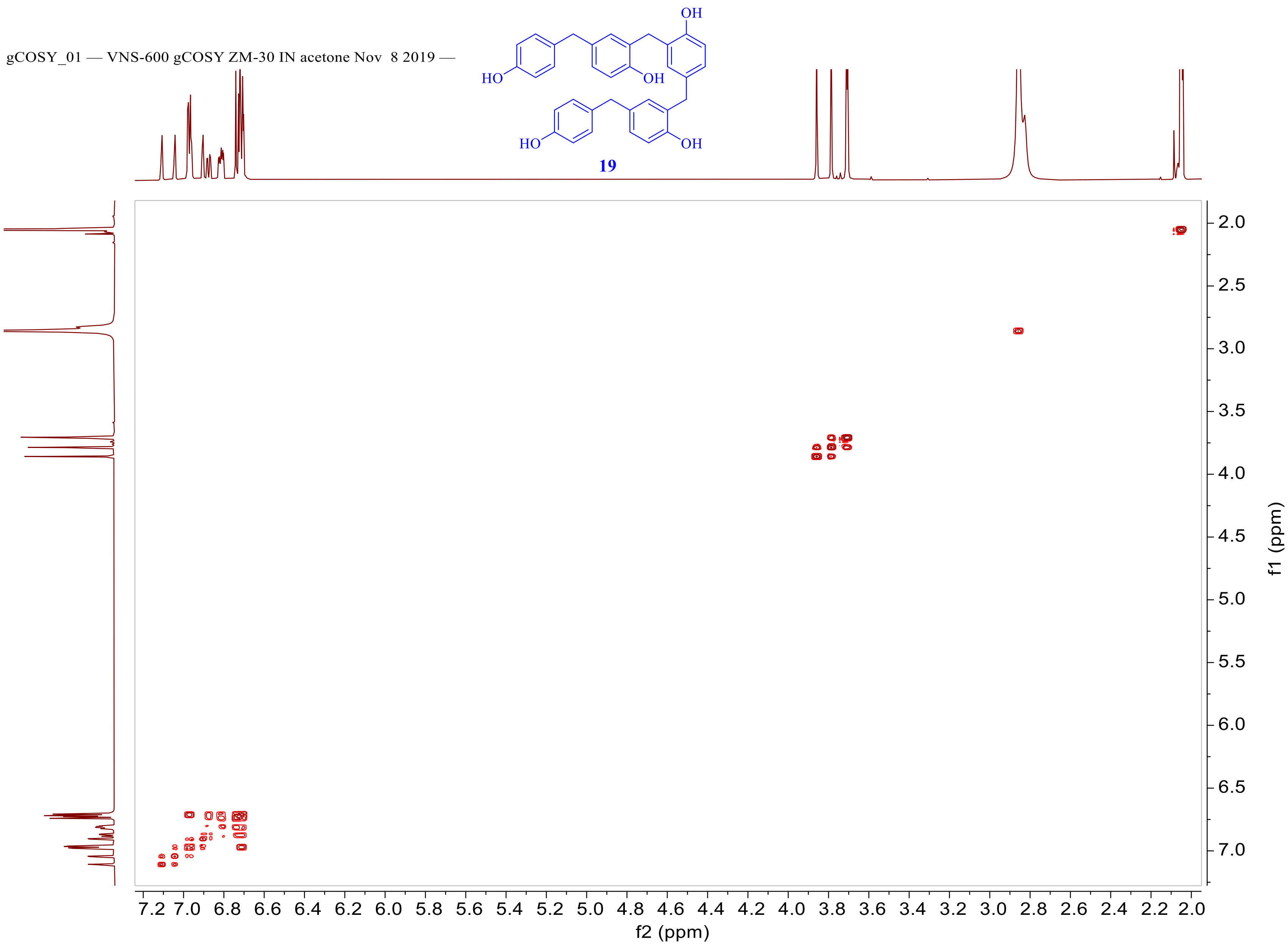

Figure S229. The ^{1}H-^{1}H COSY spectrum of compound **19** in acetone-d_6 (600 MHz)

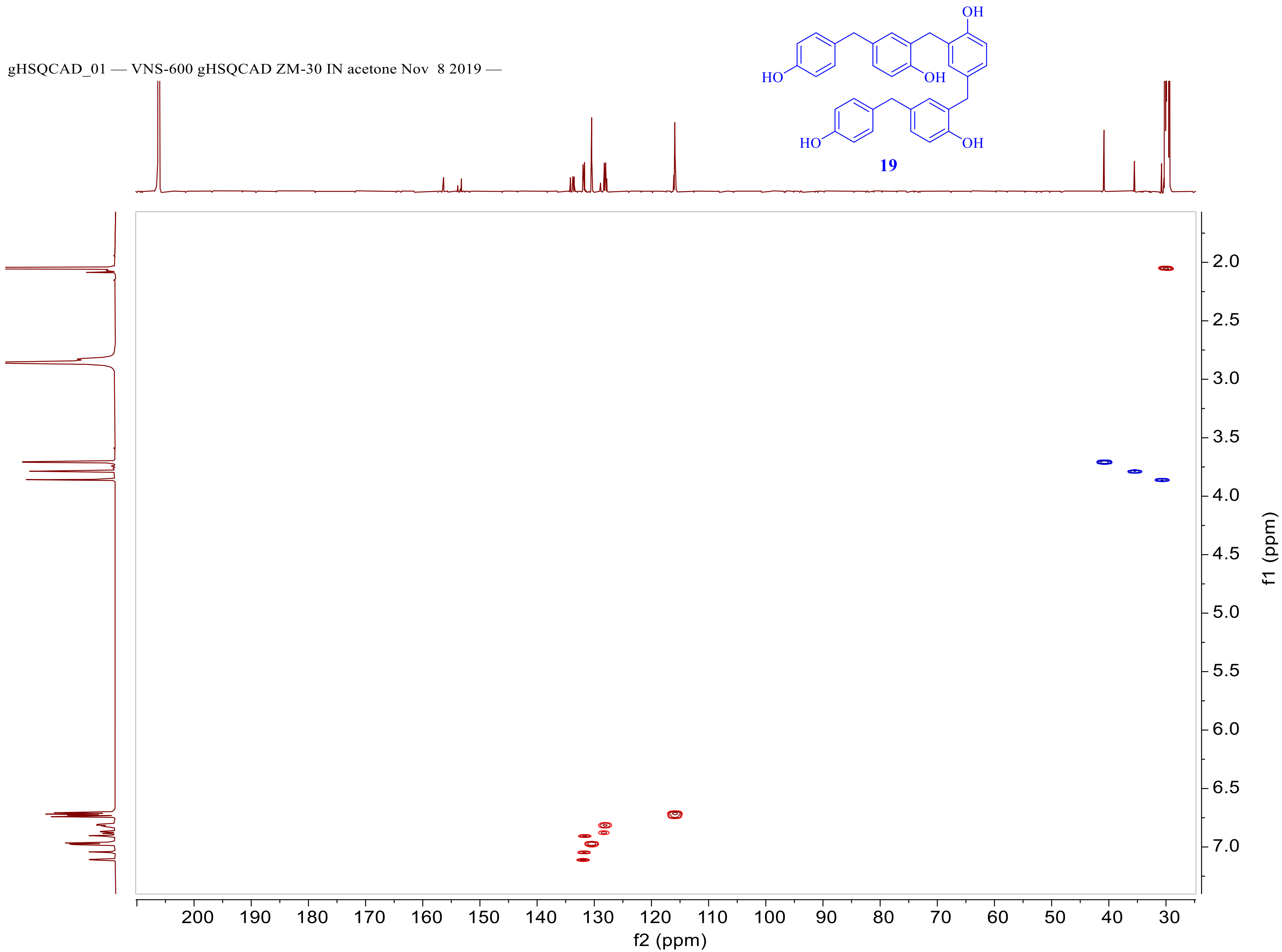

Figure S230. The HSQC spectrum of compound **19** in acetone-d_6 (600 MHz for ^{1}H)

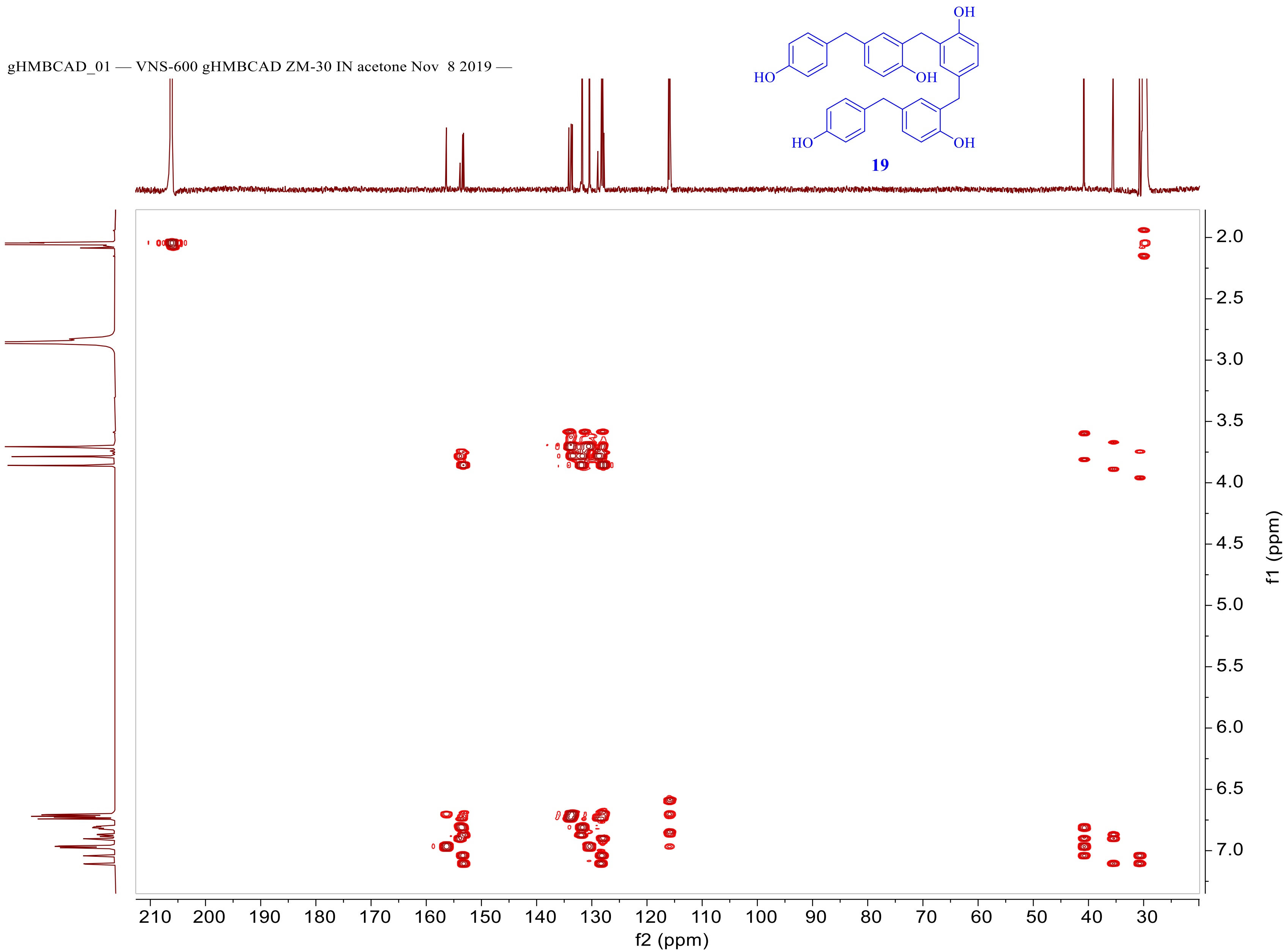

Figure S231. The HMBC spectrum of compound **19** in acetone-d_6 (600 MHz for ^{1}H)

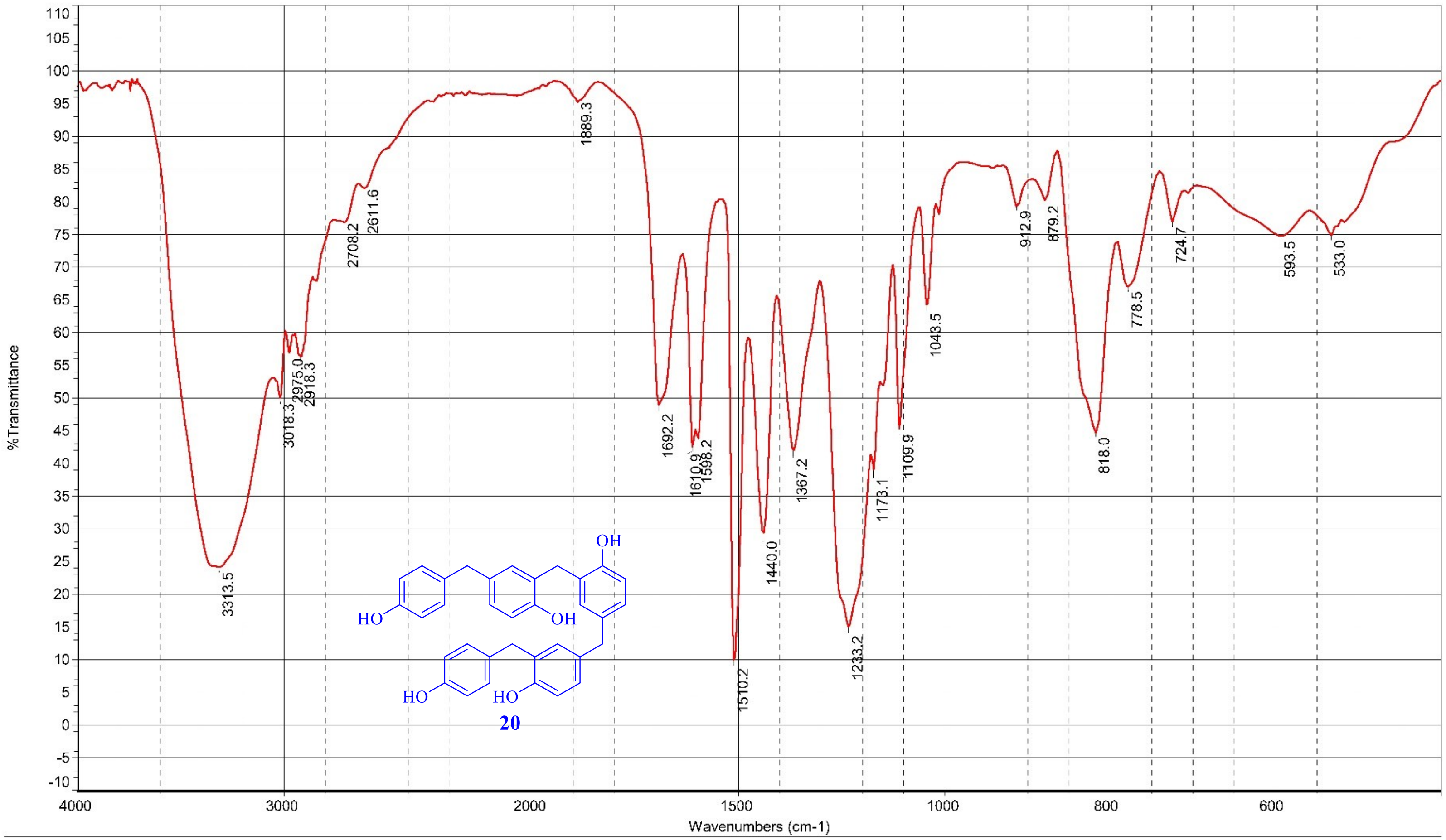

日期: 星期一 12月 21 17:00:25 2020 (GMT+08:00Sample Name： ZM-34 (显微镜透射法FT- IR Microscope Transmission)

扫描次数: 100

分辨率: 8.000

Figure S232. The IR spectrum of compound **20**

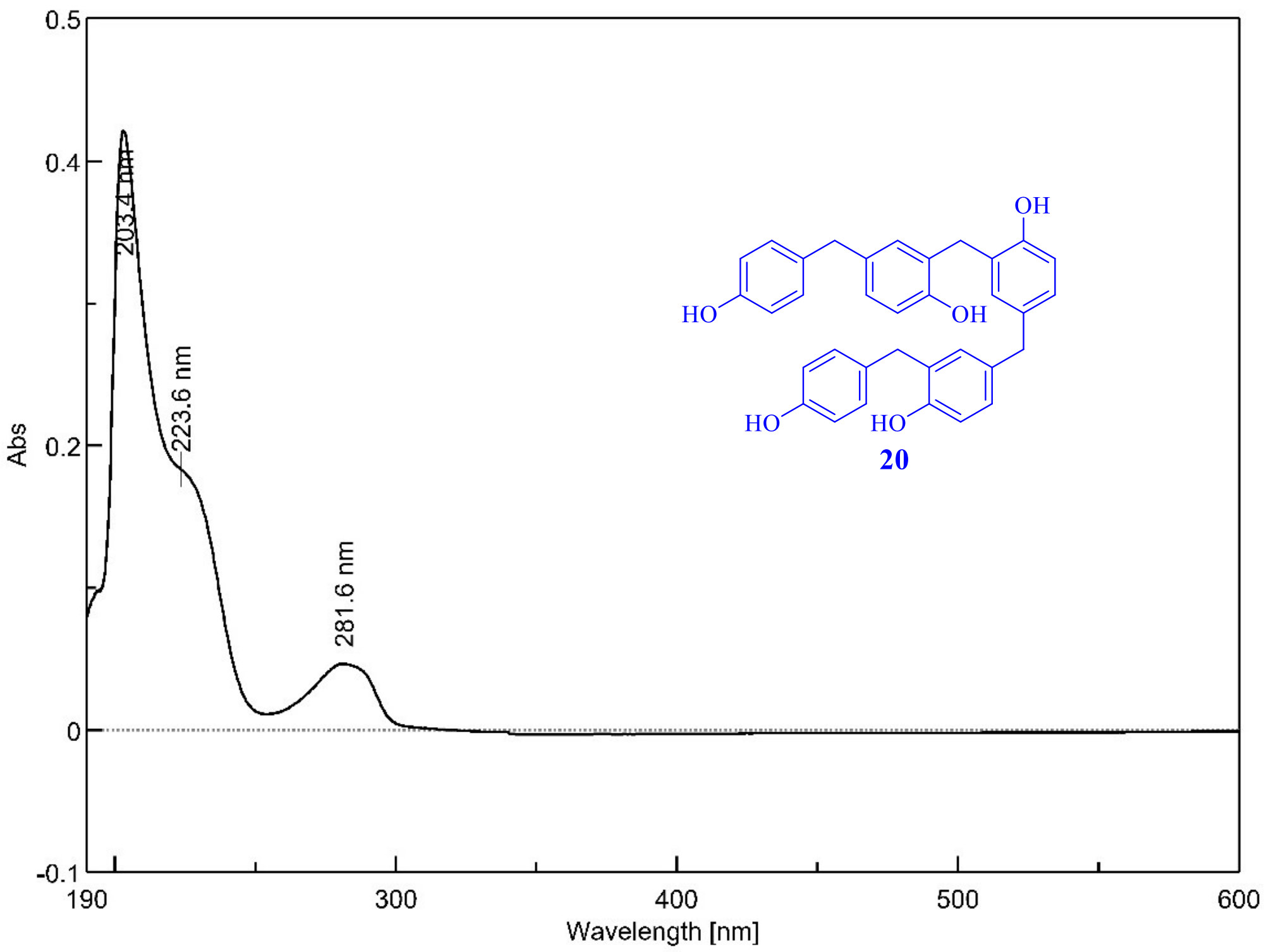

[Comment]		ZM-34
Sample Name	DBT	
Comment	0.02	
User		
Division	UV	
Company	324	

[Measurement Information]

Instrument Name	V-650
Model Name	V-650
Serial No.	A034461150
Accessory	PSC-718
Accessory S/N	A001761114
Position	1
Cell Length	10 mm
Temperature	19.98 C
Control Sonsor	Holder
Monitor Sensor	Holder
Start Mode	Start immediately
Photometric Mode	Abs
Measurement range	600 - 190 nm
Data pitch	0.2 nm
Band width(UV/Vis)	2.0 nm
Response	Medium
Scanning speed	200 nm/min
Source Change	340 nm
Light Source	D2/WI
Filter Exchange	Step
Correction	Baseline

[Data Information]

Creation Date	2020-11-11 16:12
Data array type	Linear data array
Horizontal	Wavelength [nm]
Vertical	Abs
Start	600 nm
End	190 nm
Data pitch	0.2 nm
Data points	2051

Figure S233. The UV spectrum of compound **20** in MeOH

Thermo Qexactive Focus Report

compound NO. : ZM-34

Method : LCMS(compound)-low

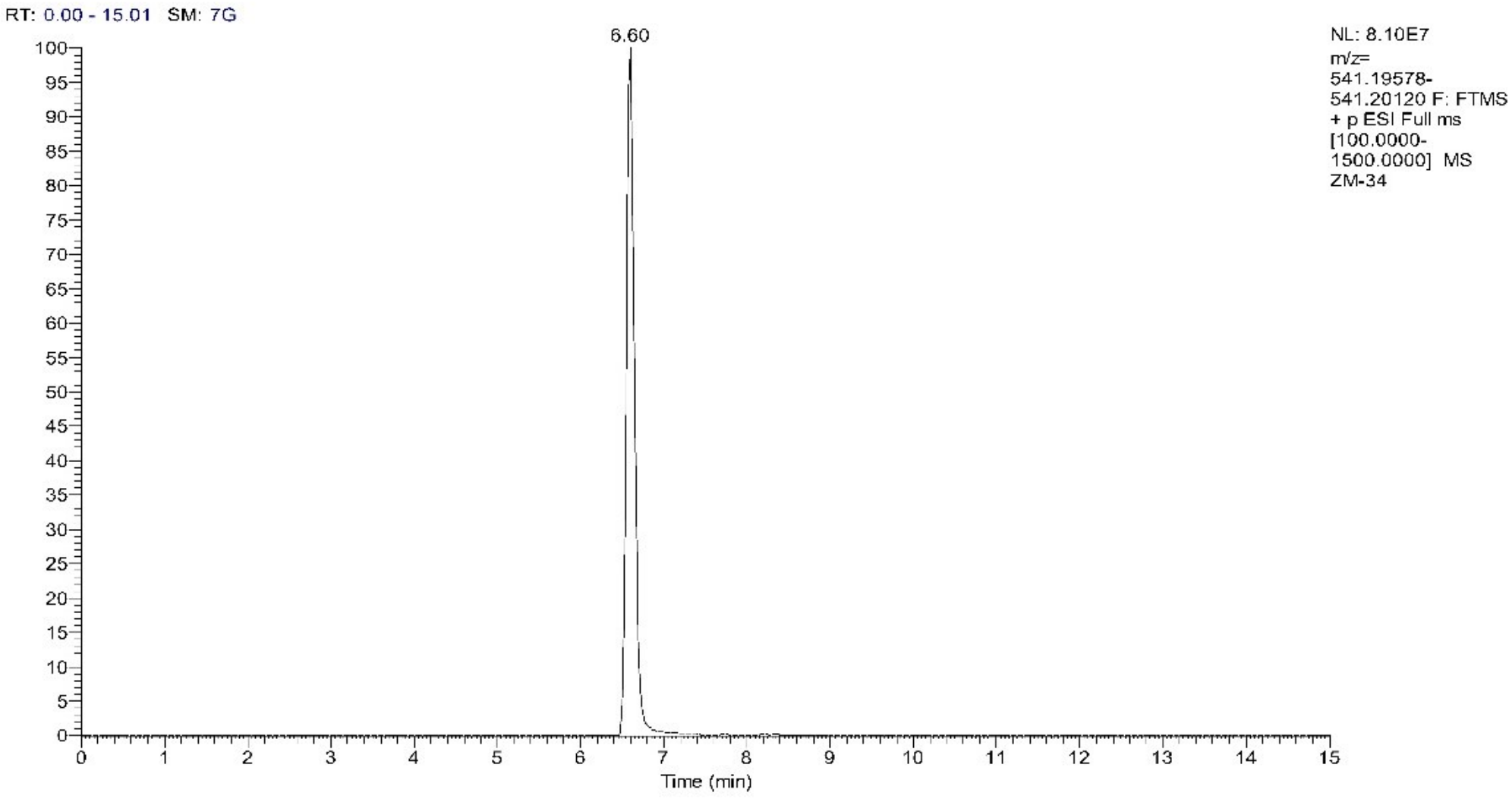

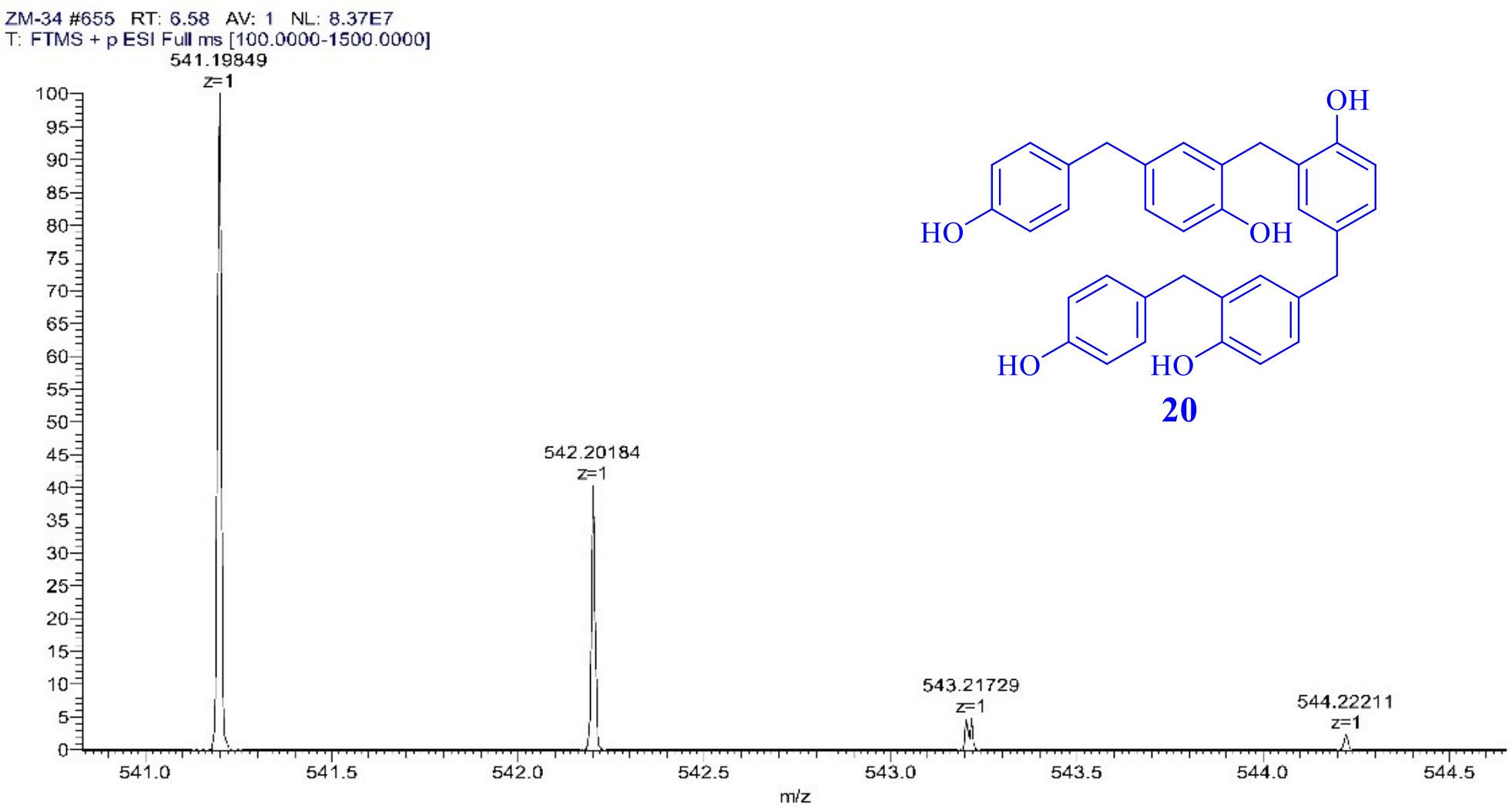

m/z	Theo. Mass	Delta (ppm)	RDB equiv.	Composition	
541.19849	541.19855	-0.1	19.5	C34 H30 O5 Na	M+Na

Figure S234. The HR-ESIMS report of compound **20**

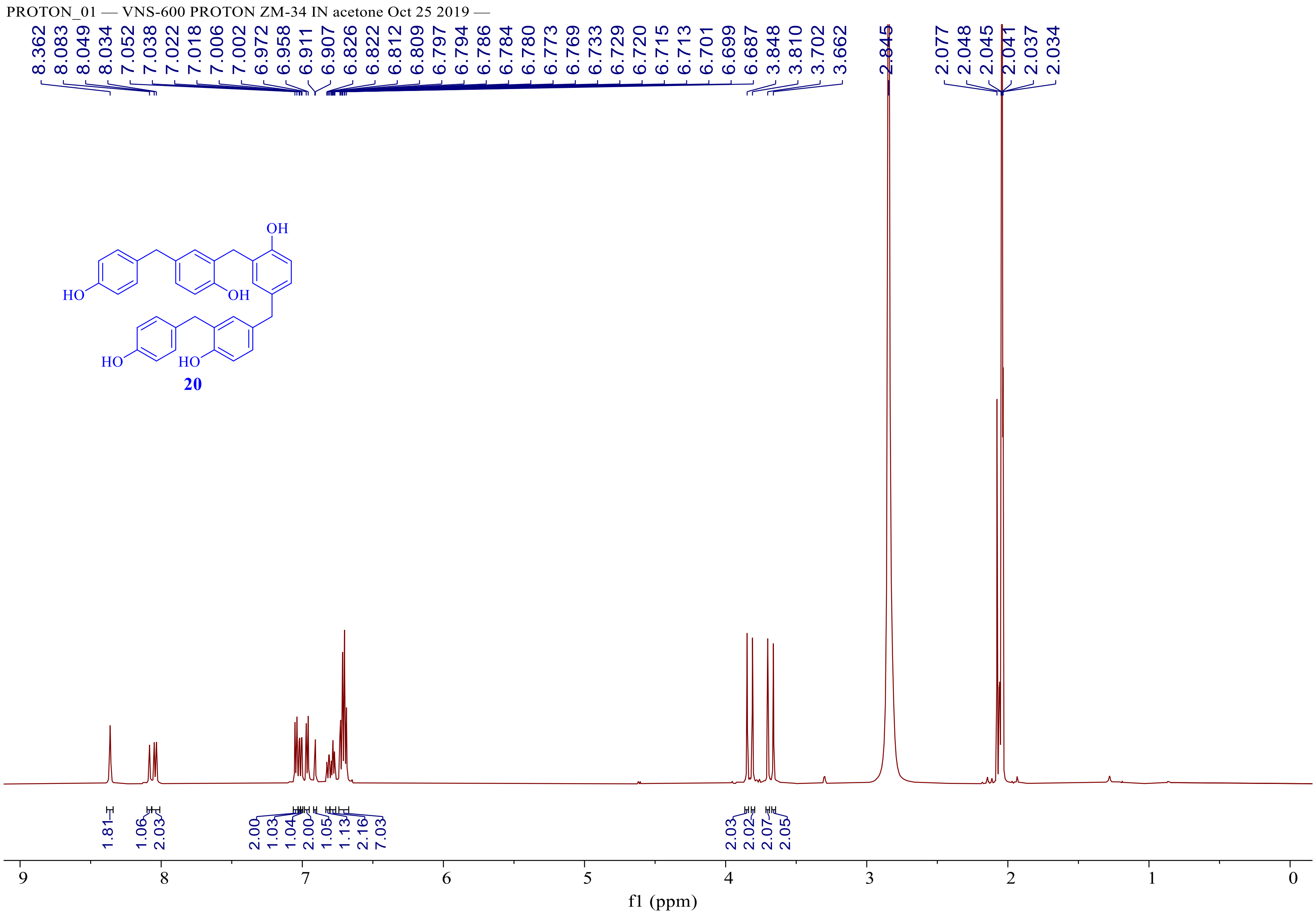

Figure S235. The ^{1}H NMR spectrum of compound **20** in acetone-d_6 (600 MHz)

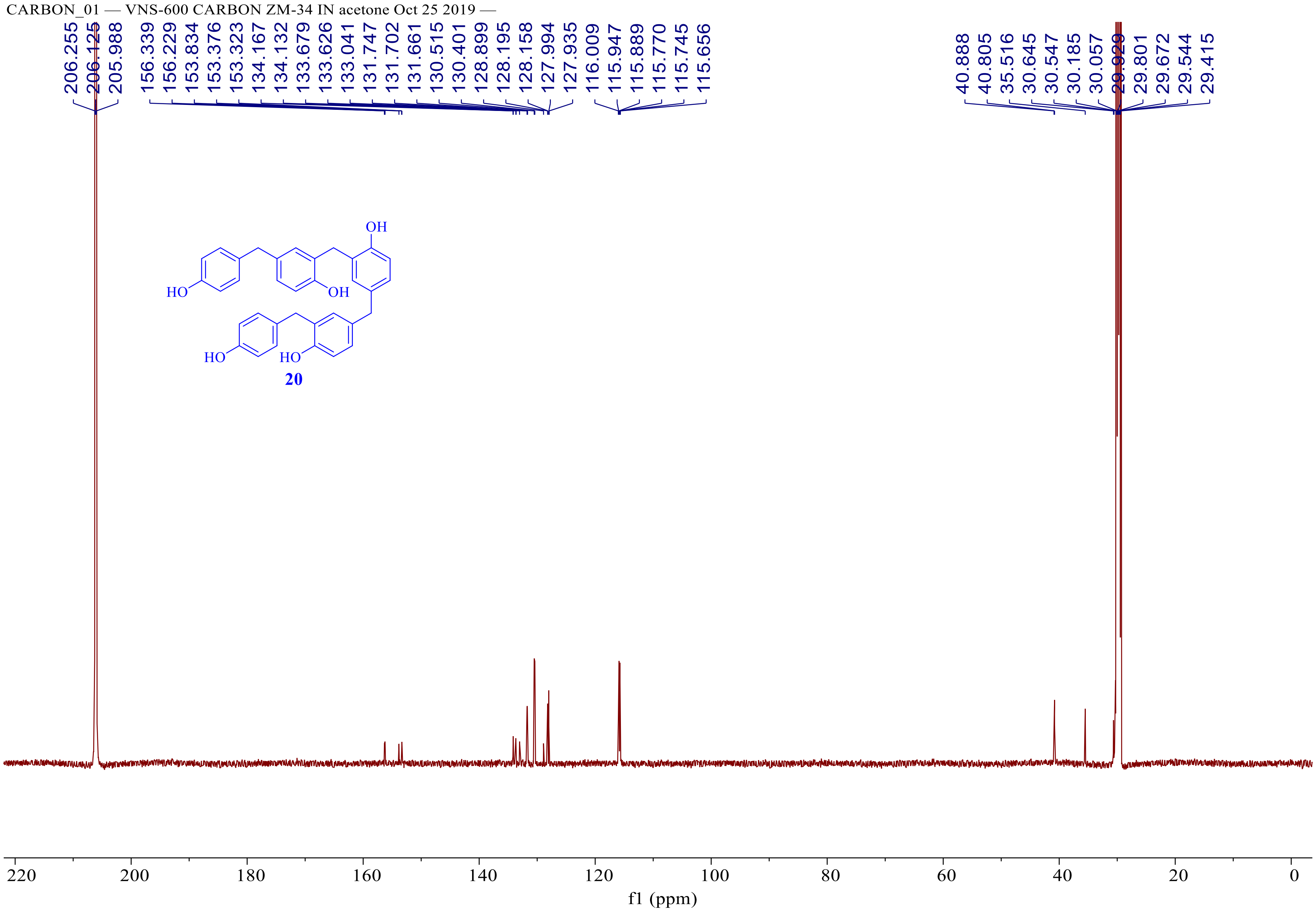

Figure S236. The ^{13}C NMR spectrum of compound **20** in acetone-d_6 (150 MHz)

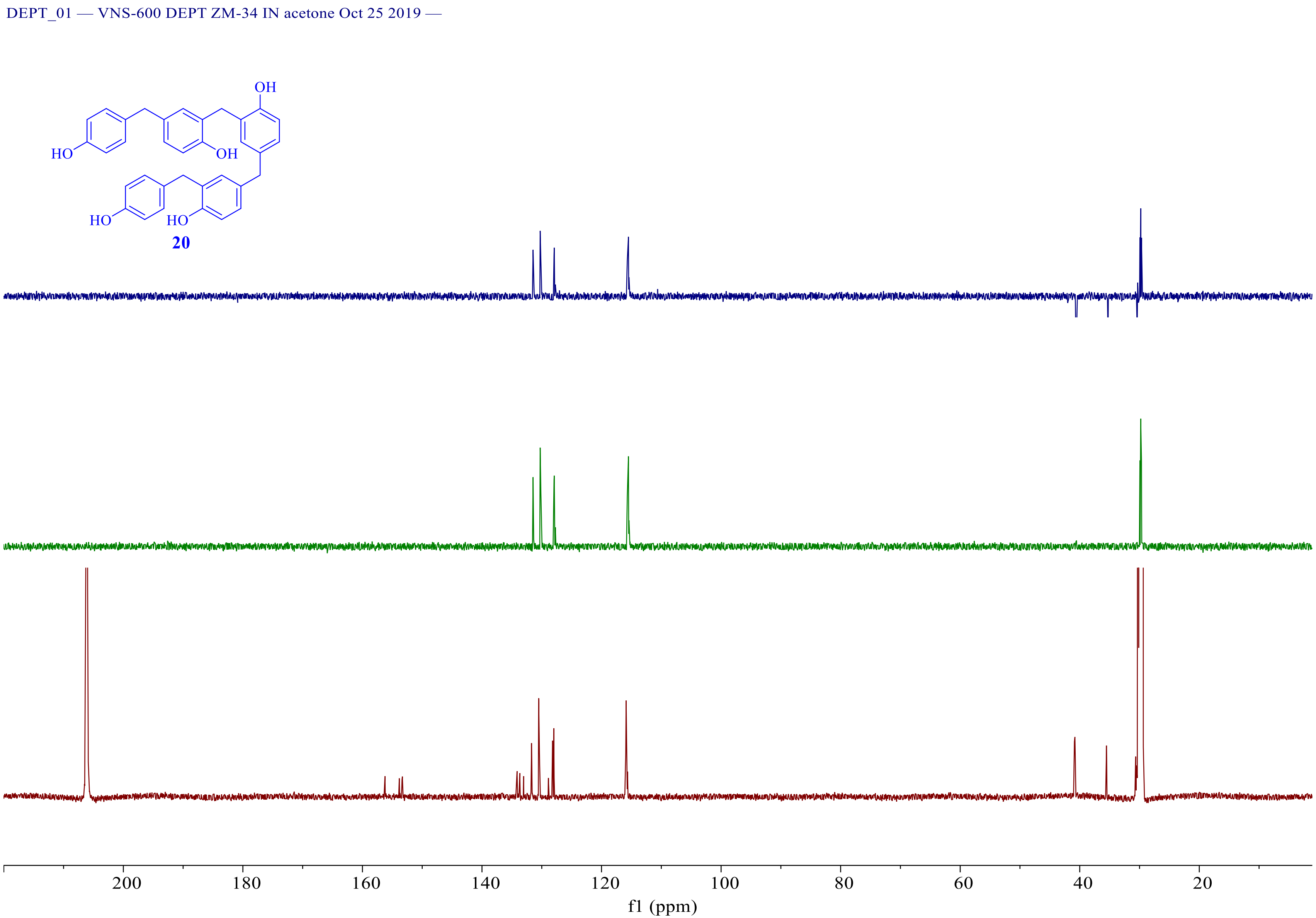

Figure S237. The DEPT spectrum of compound **20** in acetone-d_6 (150 MHz)

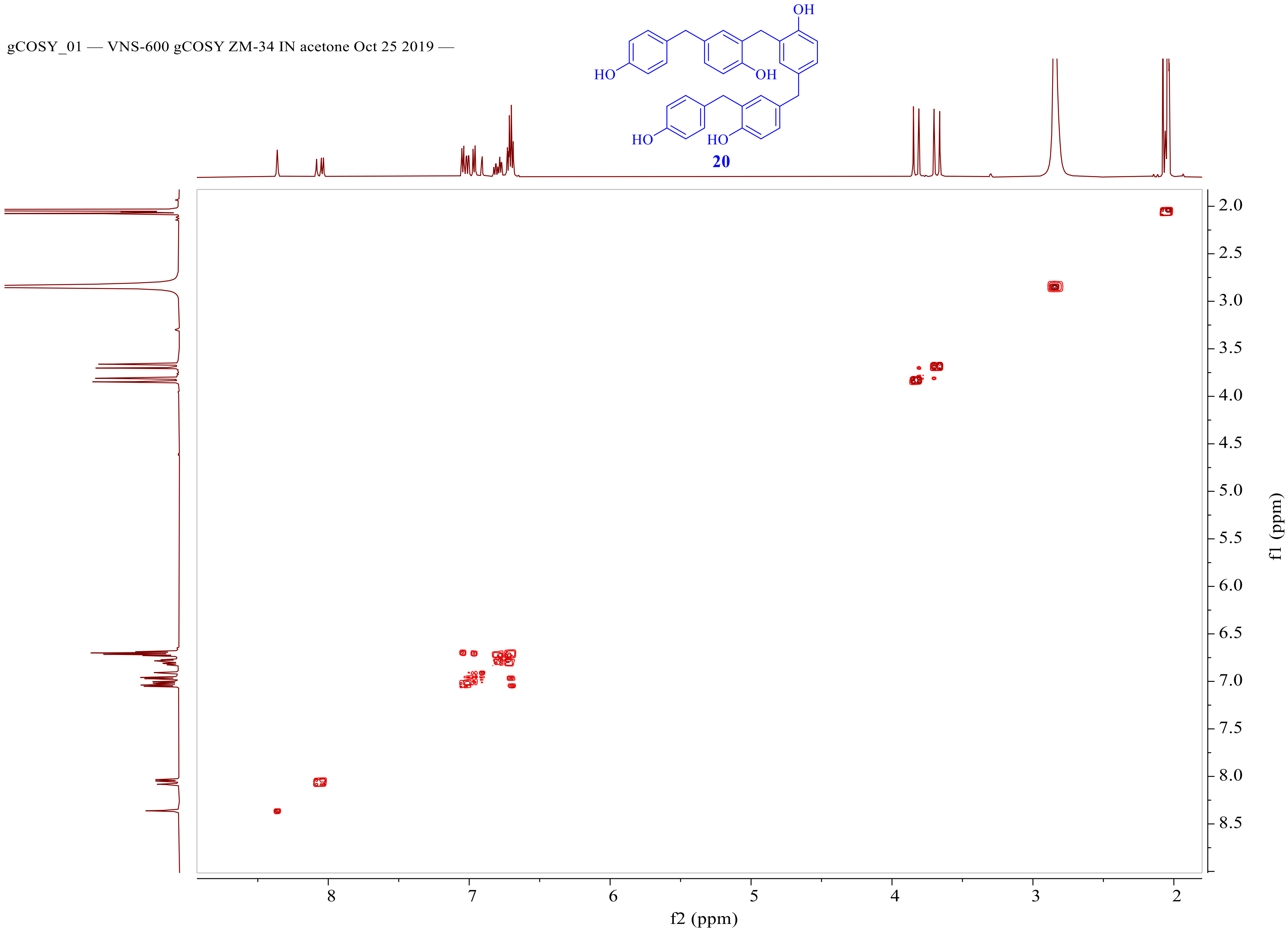

Figure S238. The ^{1}H-^{1}H COSY spectrum of compound **20** in acetone-d_6 (600 MHz)

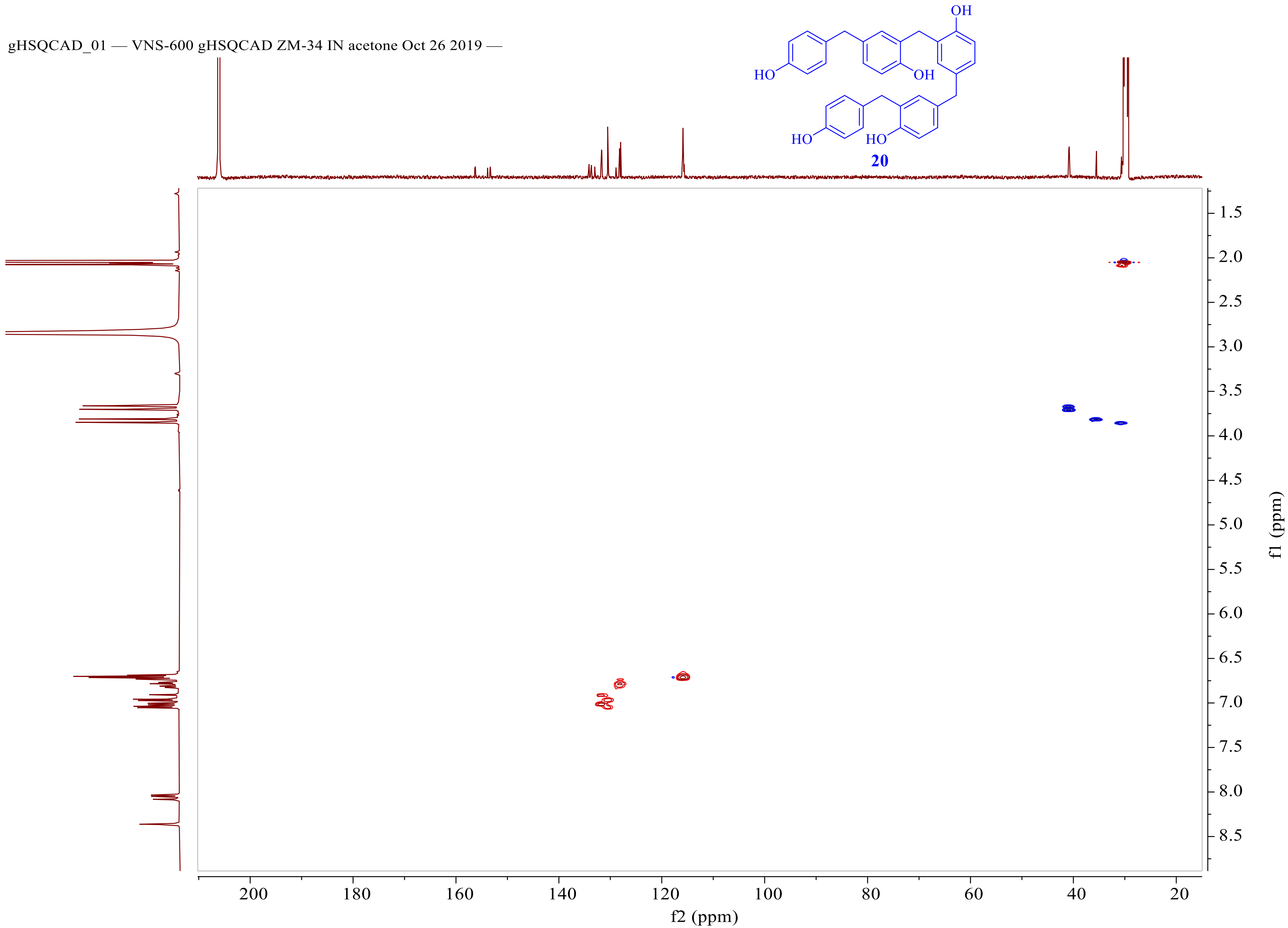

Figure S239. The HSQC spectrum of compound **20** in acetone-d_6 (600 MHz for ^{1}H)

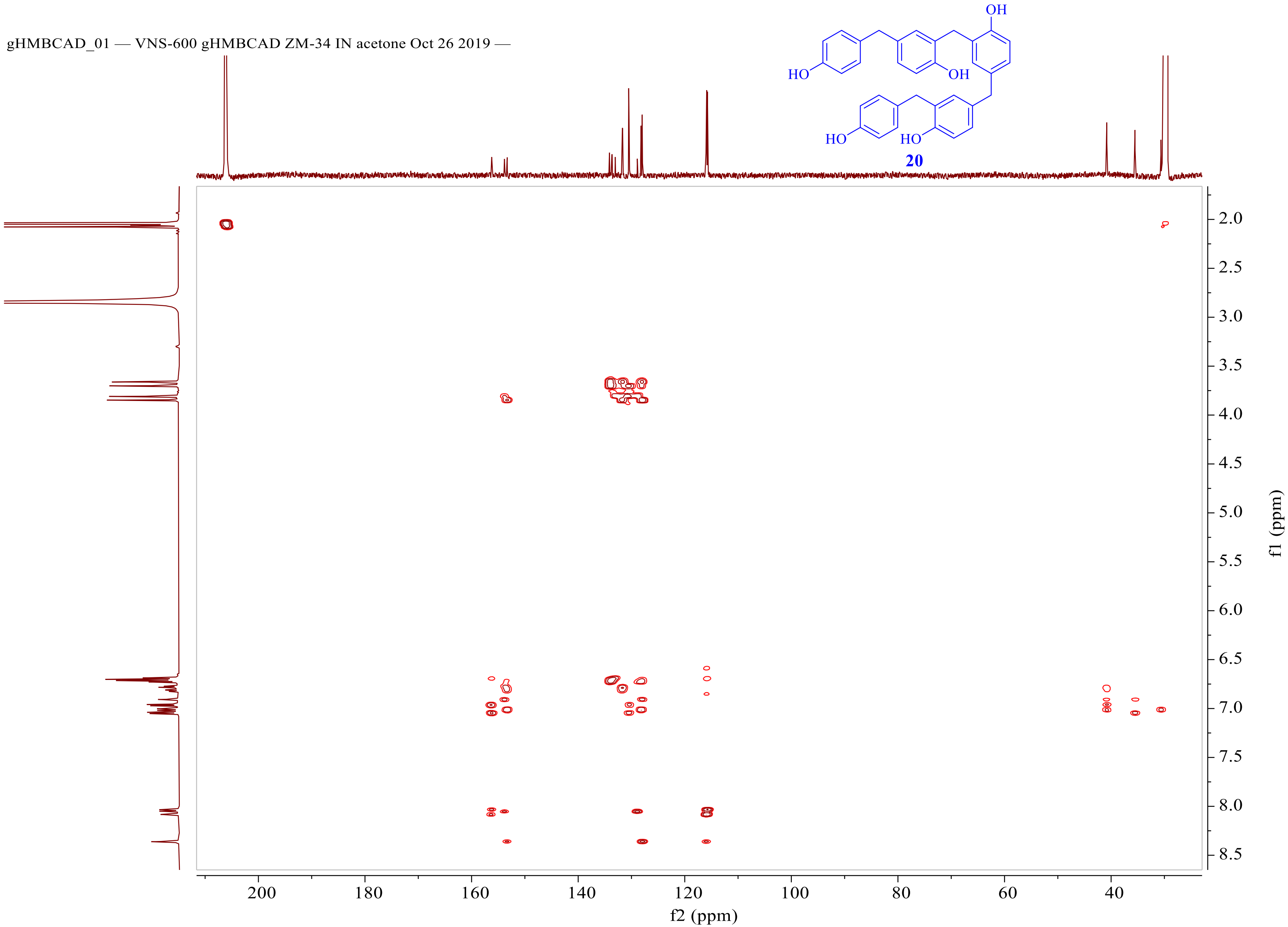

Figure S240. The HMBC spectrum of compound **20** in acetone-d_6 (600 MHz for ^{1}H)

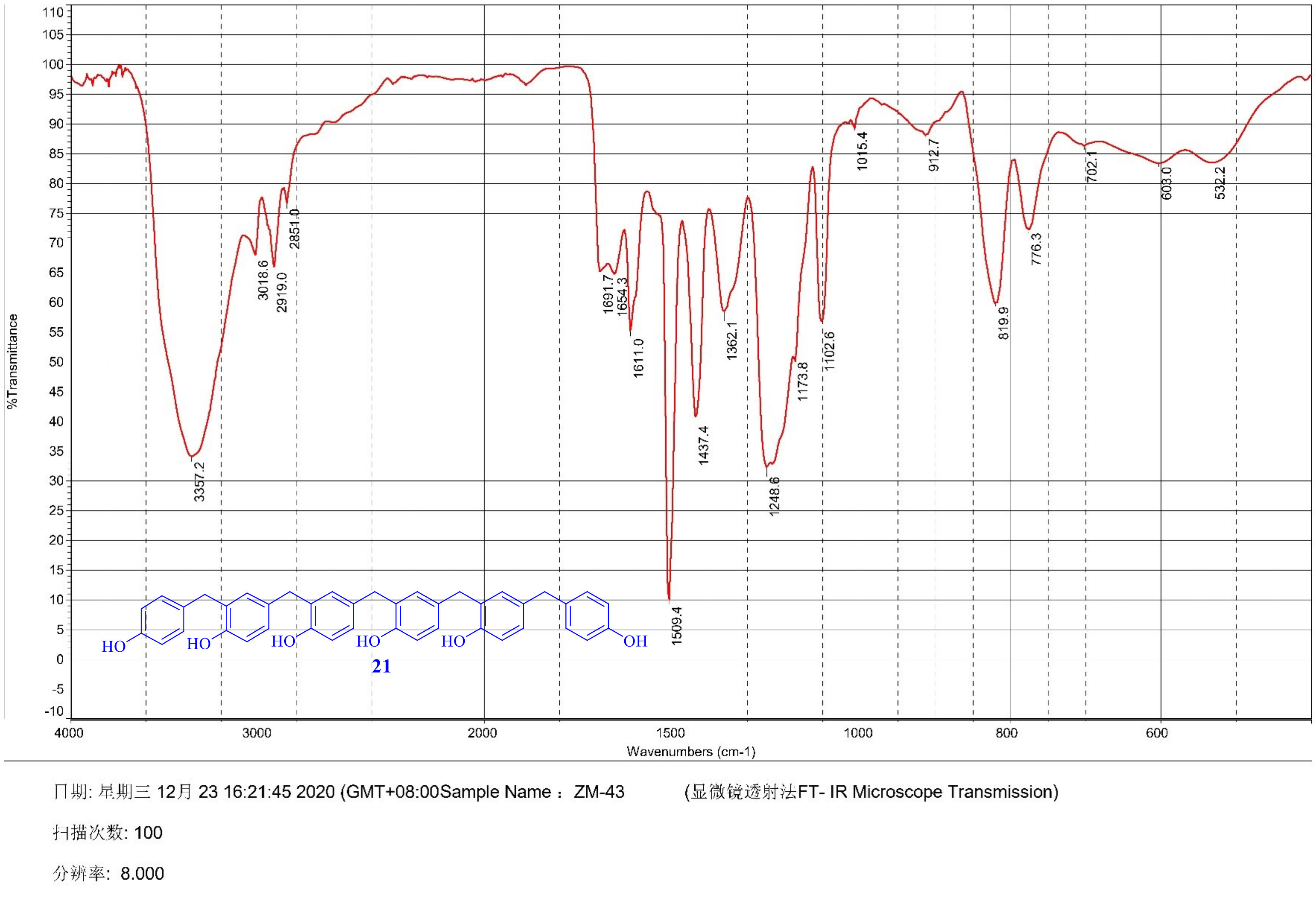

Figure S241. The IR spectrum of compound **21**

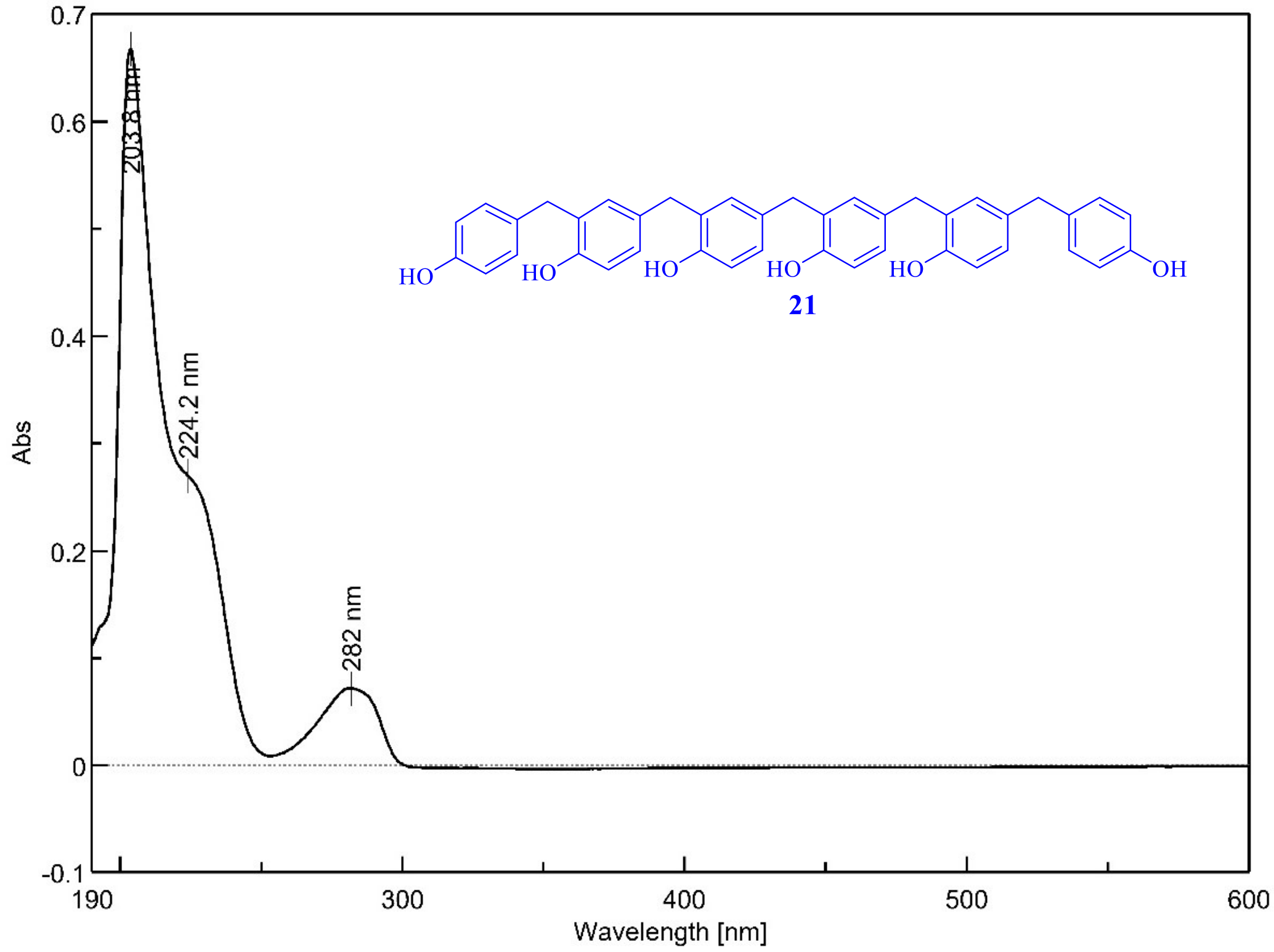

ZM-43

[Comment]
Sample Name DBT
Comment 0.02
User
Division UV
Company 324

[Measurement Information]
Instrument Name V-650
Model Name V-650
Serial No. A034461150

Accessory PSC-718
Accessory S/N A001761114
Position 1
Cell Length 10 mm
Temperature 19.97 C
Control Sonsor Holder
Monitor Sensor Holder
Start Mode Start immediately

Photometric Mode Abs
Measurement range 600 - 190 nm
Data pitch 0.2 nm
Band width(UV/Vis) 2.0 nm
Response Medium
Scanning speed 200 nm/min
Source Change 340 nm
Light Source D2/WI
Filter Exchange Step
Correction Baseline

[Data Information]
Creation Date 2020-11-11 16:59

Data array type Linear data array
Horizontal Wavelength [nm]
Vertical Abs
Start 600 nm
End 190 nm
Data pitch 0.2 nm
Data points 2051

Figure S242. The UV spectrum of compound **21** in MeOH

Thermo Qexactive Focus Report

compound NO. : ZM-43
Method : LCMS(compound)-low

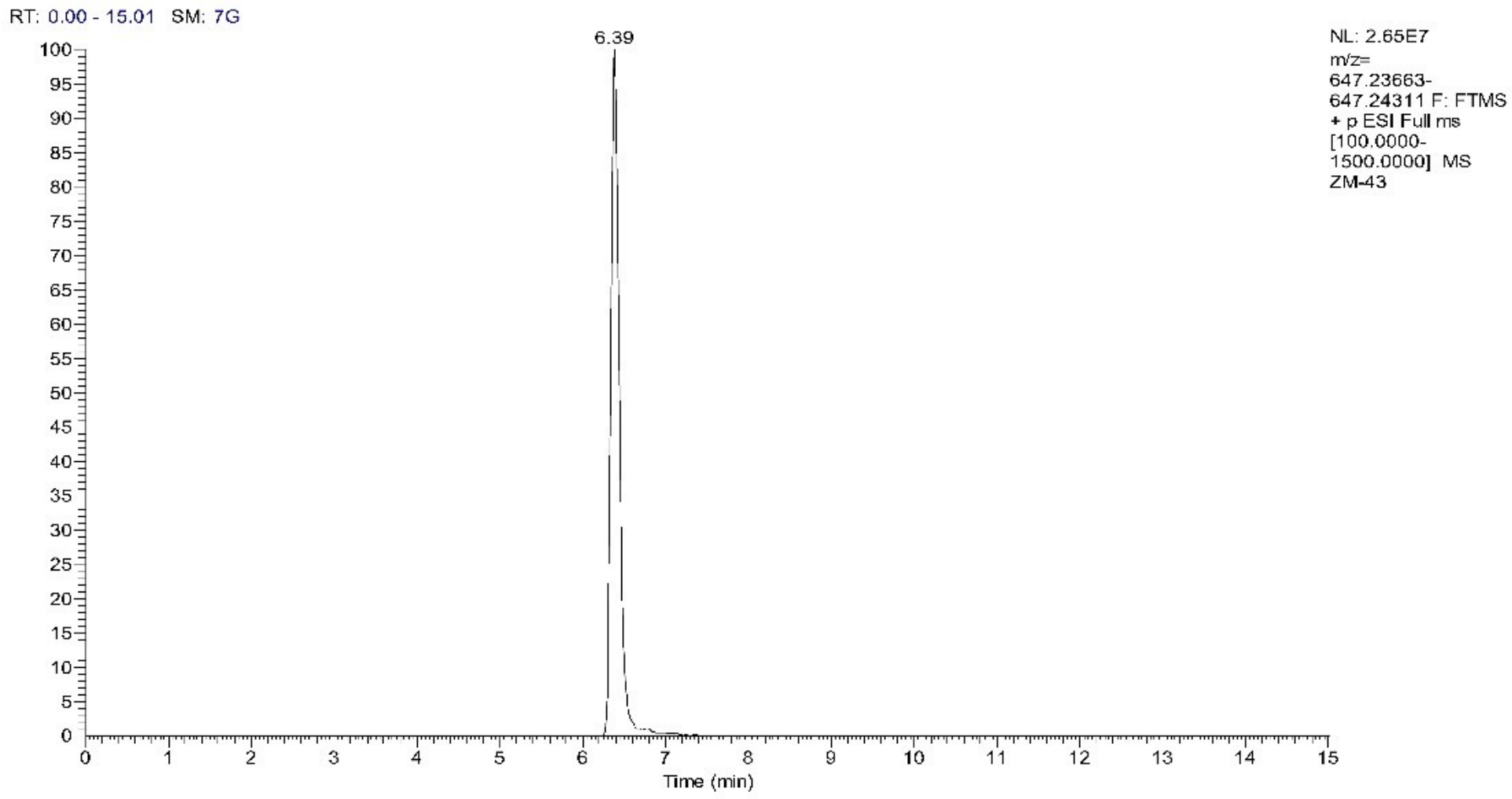

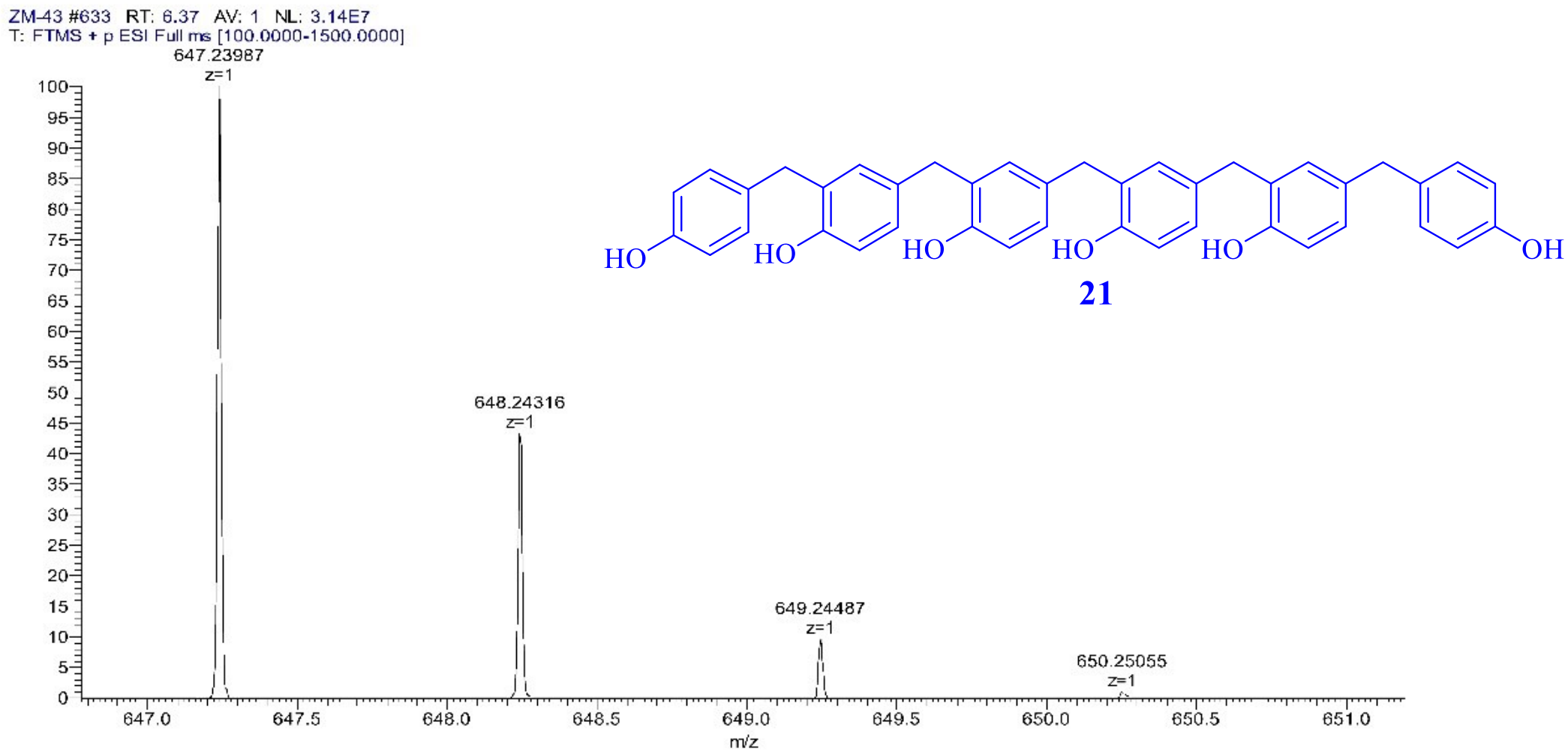

21

m/z	Theo. Mass	Delta (ppm)	RDB equiv.	Composition	
647.23987	647.24041	-0.83	23.5	C41 H36 O6 Na	M+Na

Figure S243. The HR-ESIMS report of compound **21**

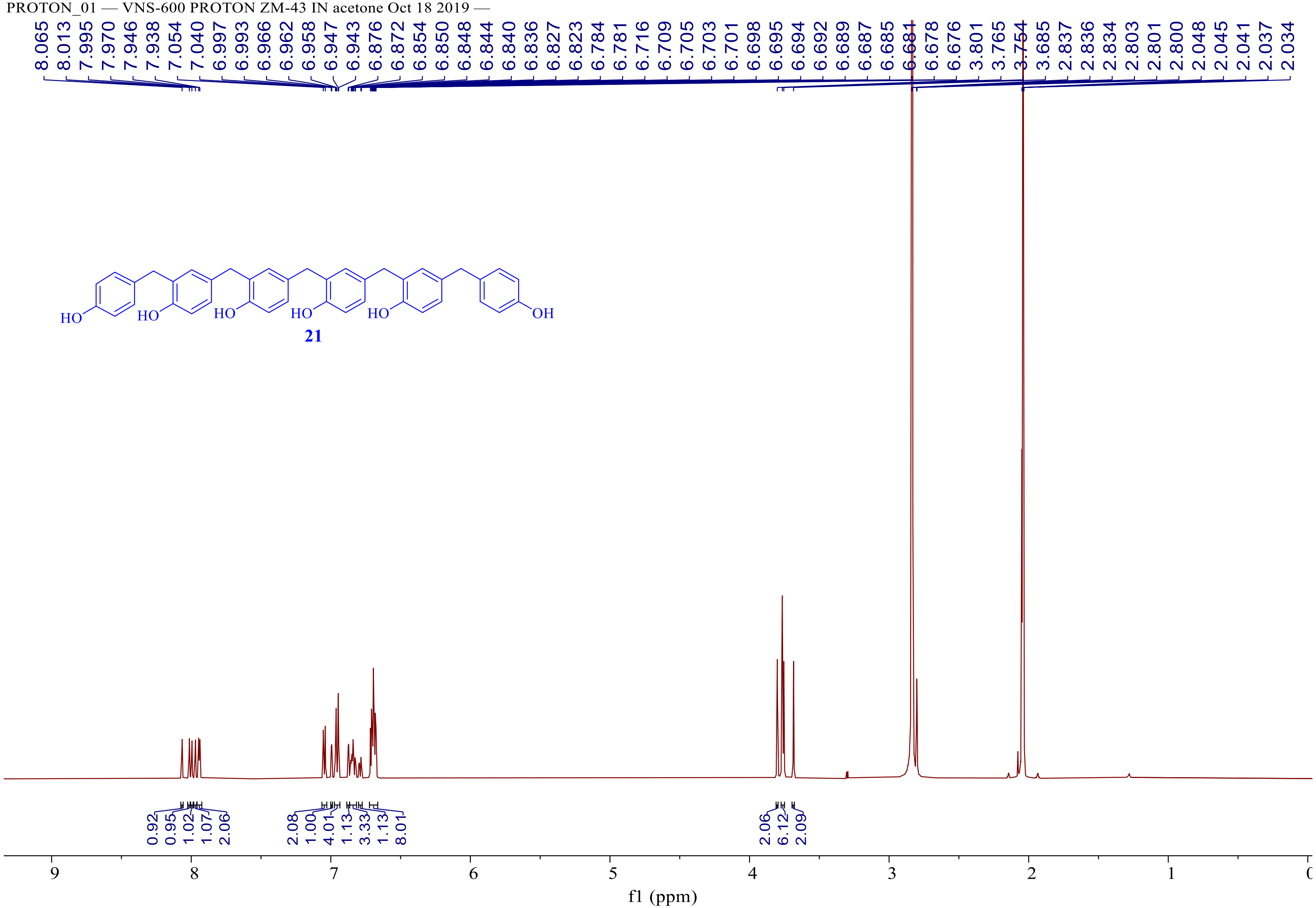

Figure S244. The ^{1}H NMR spectrum of compound **21** in acetone-d_6 (600 MHz)

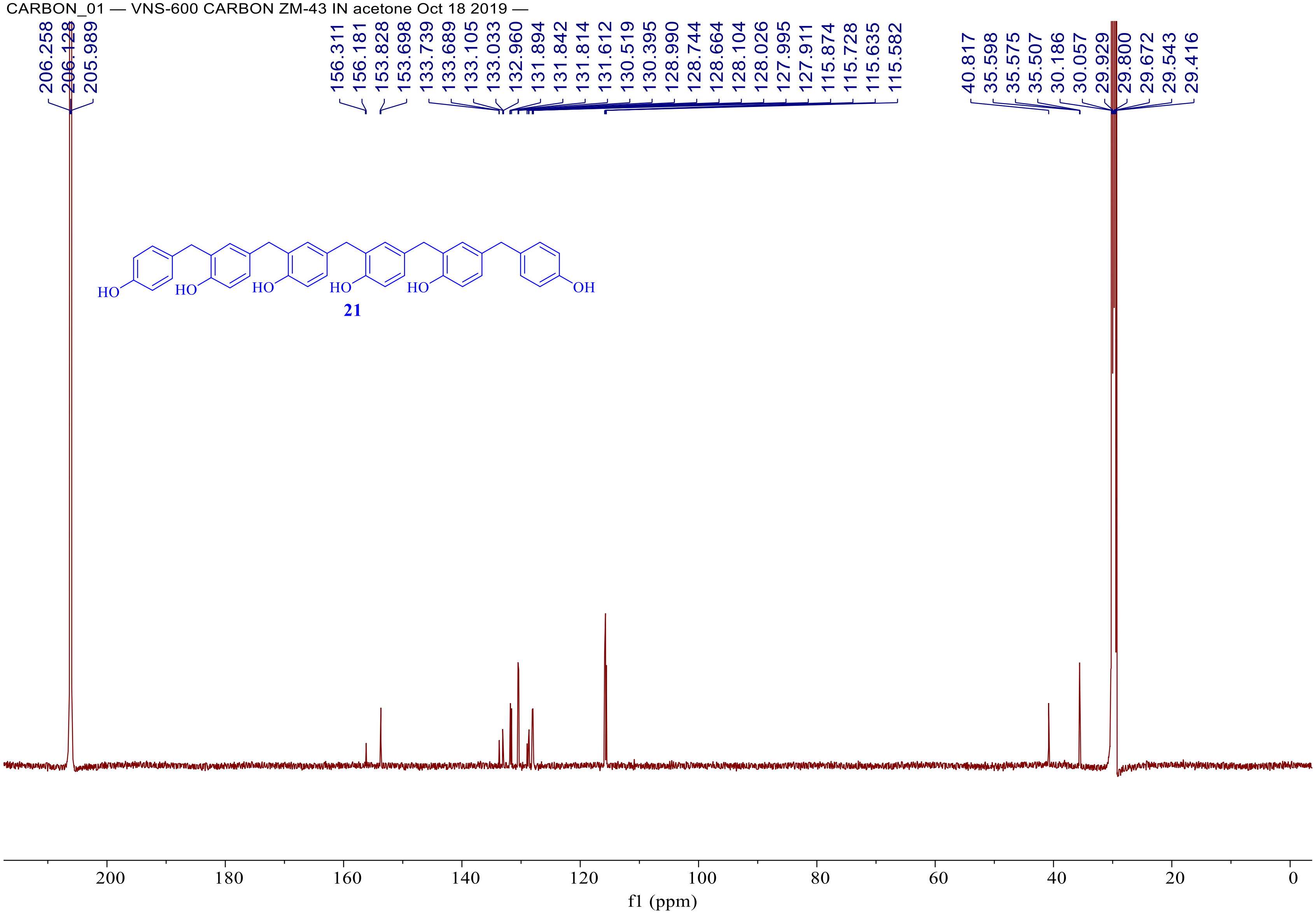

Figure S245. The ^{13}C NMR spectrum of compound **21** in acetone-d_6 (150 MHz)

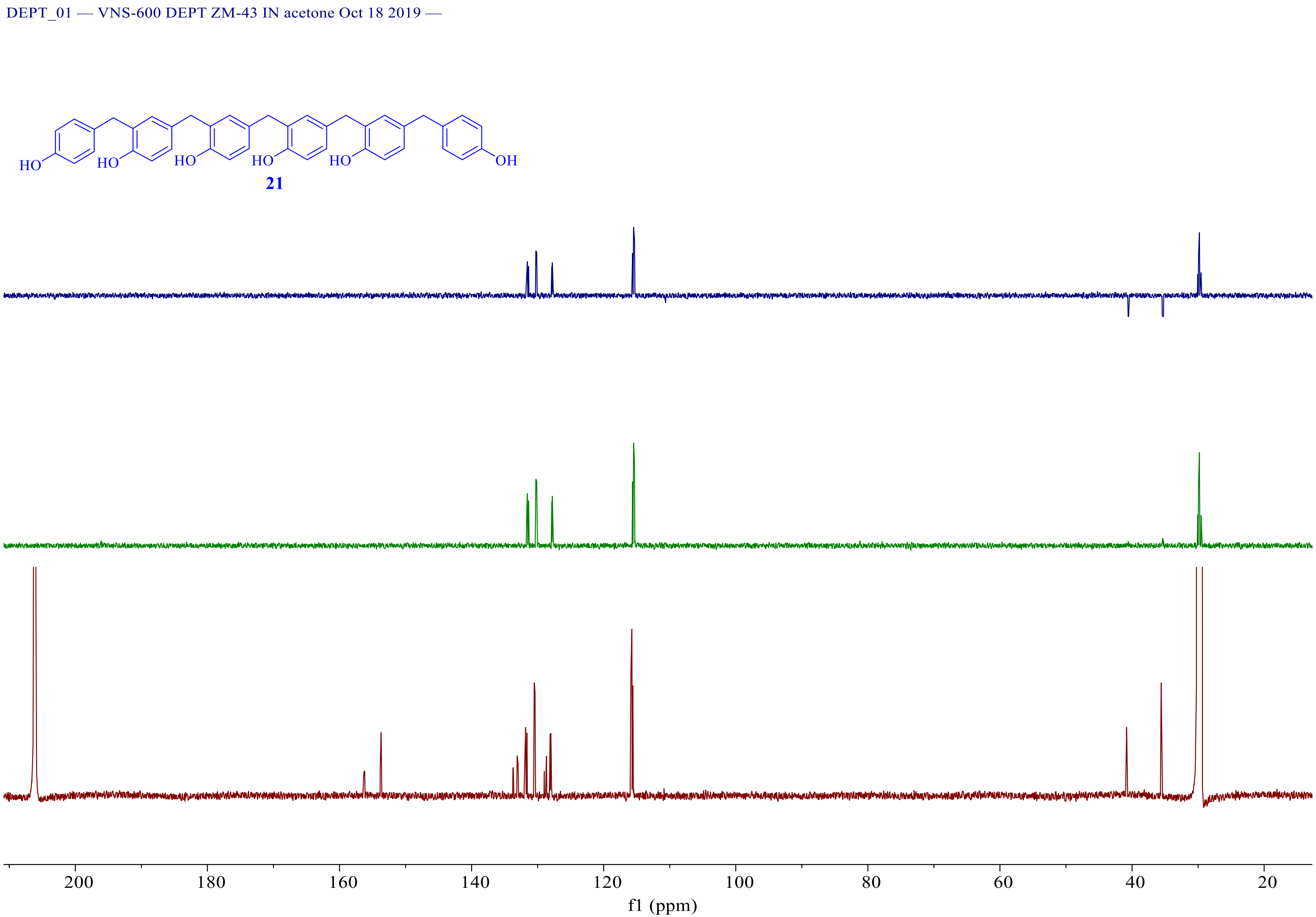

Figure S246. The DEPT spectrum of compound **21** in acetone-d_6 (150 MHz)

gCOSY_01 — VNS-600 gCOSY ZM-43 IN acetone Oct 18 2019 —

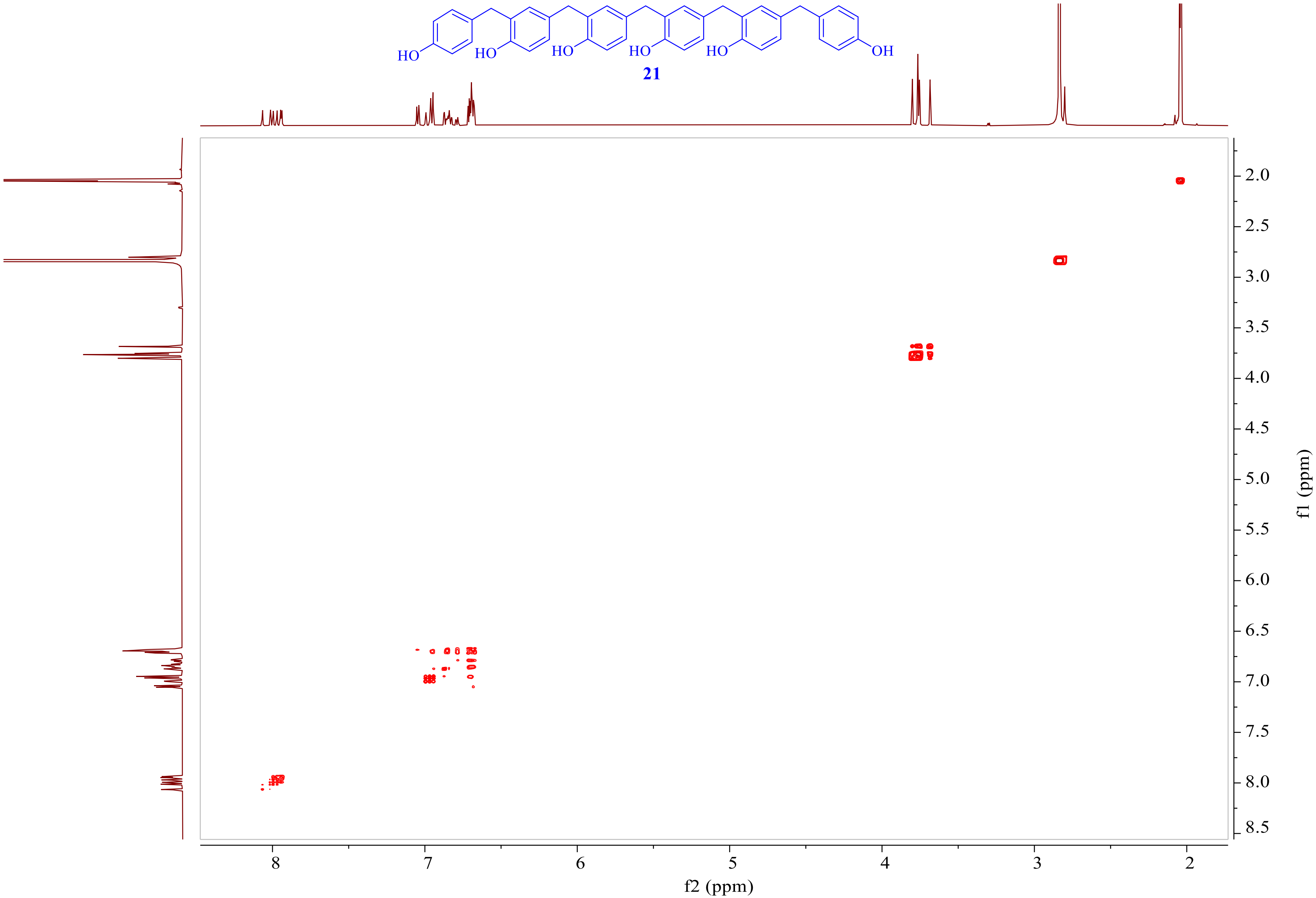

Figure S247. The ^{1}H-^{1}H COSY spectrum of compound **21** in acetone-d_6 (600 MHz)

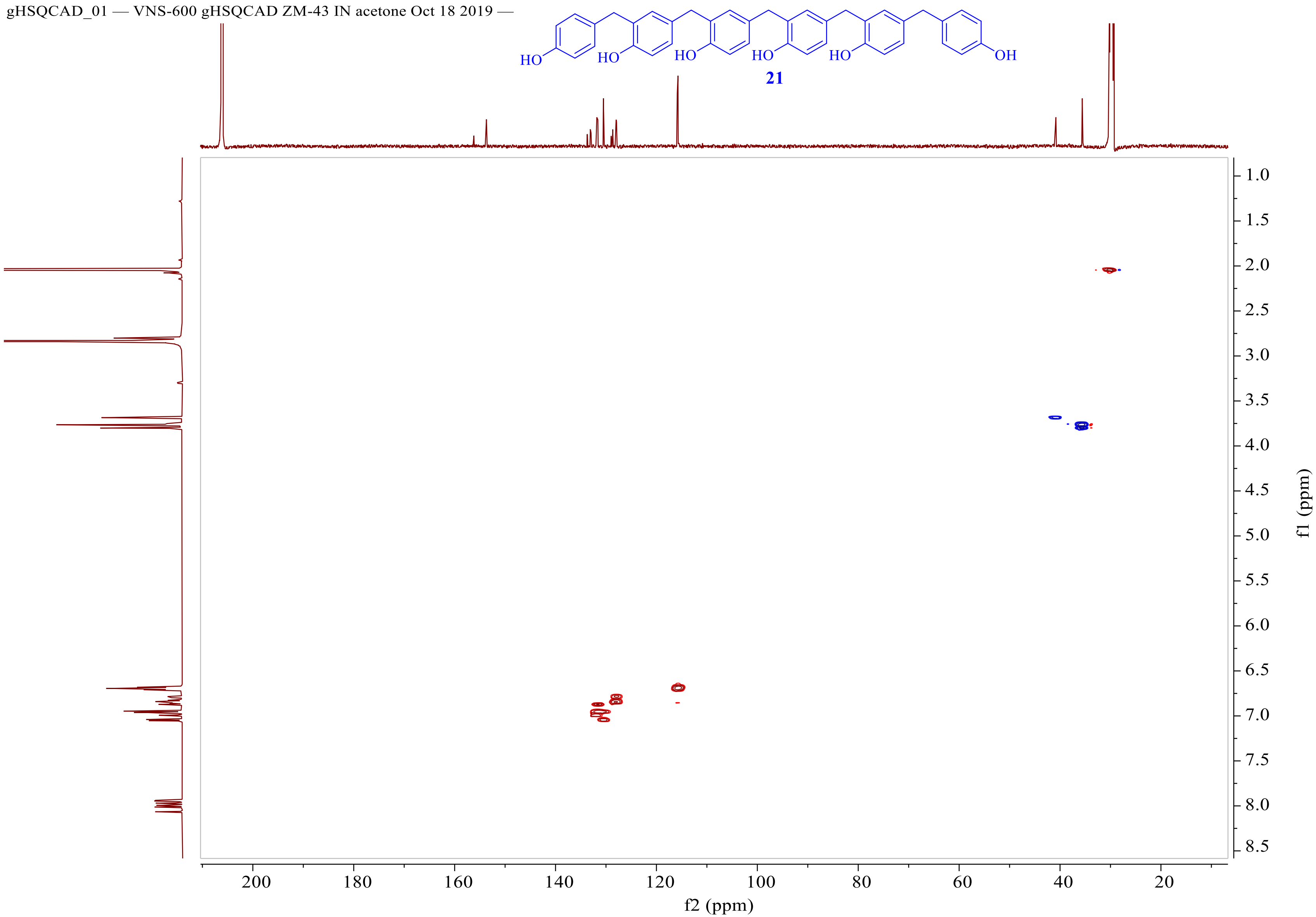

Figure S248. The HSQC spectrum of compound **21** in acetone-d_6 (600 MHz for ^{1}H)

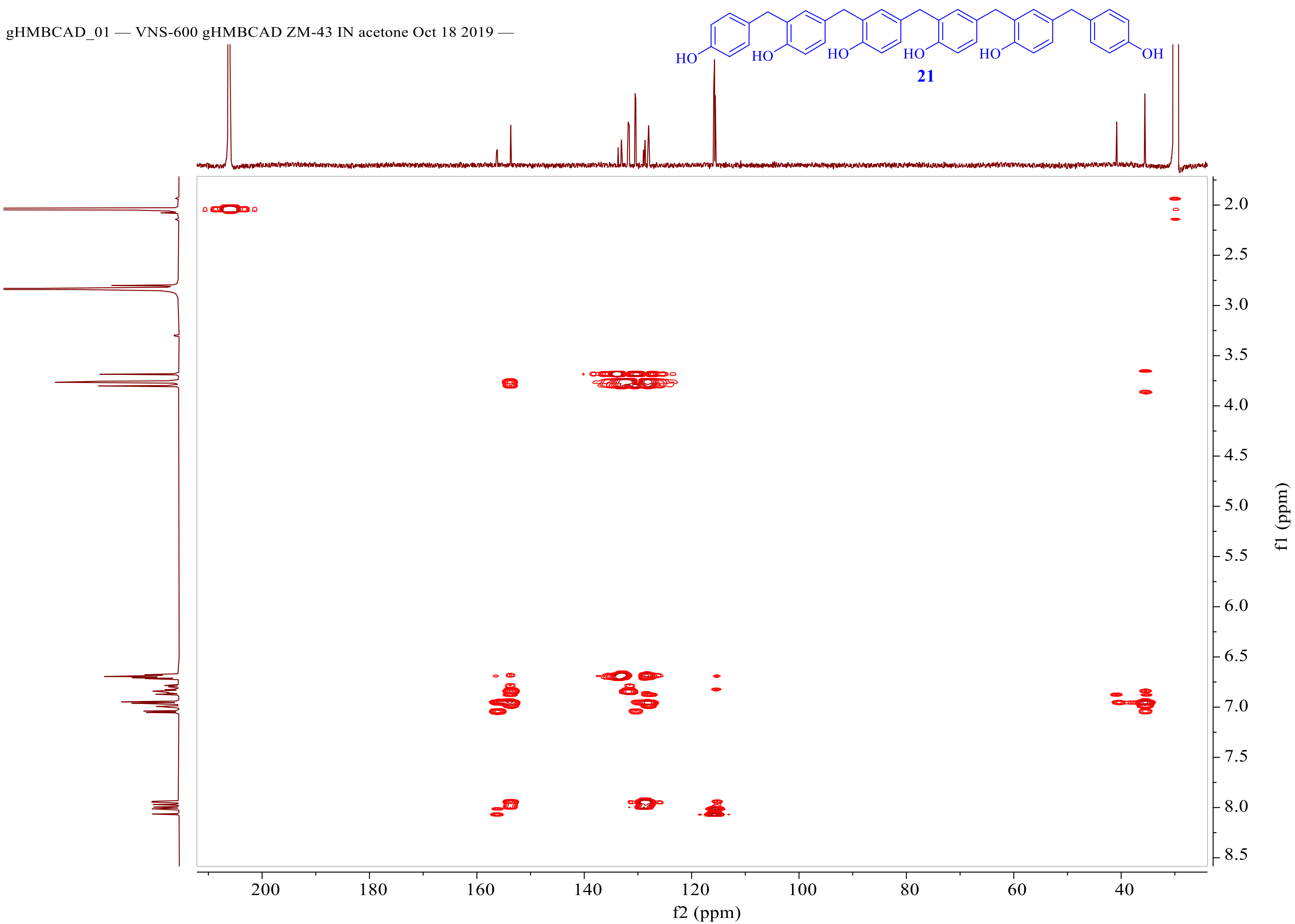

Figure S249. The HMBC spectrum of compound **21** in acetone-d_6 (600 MHz for ^{1}H)

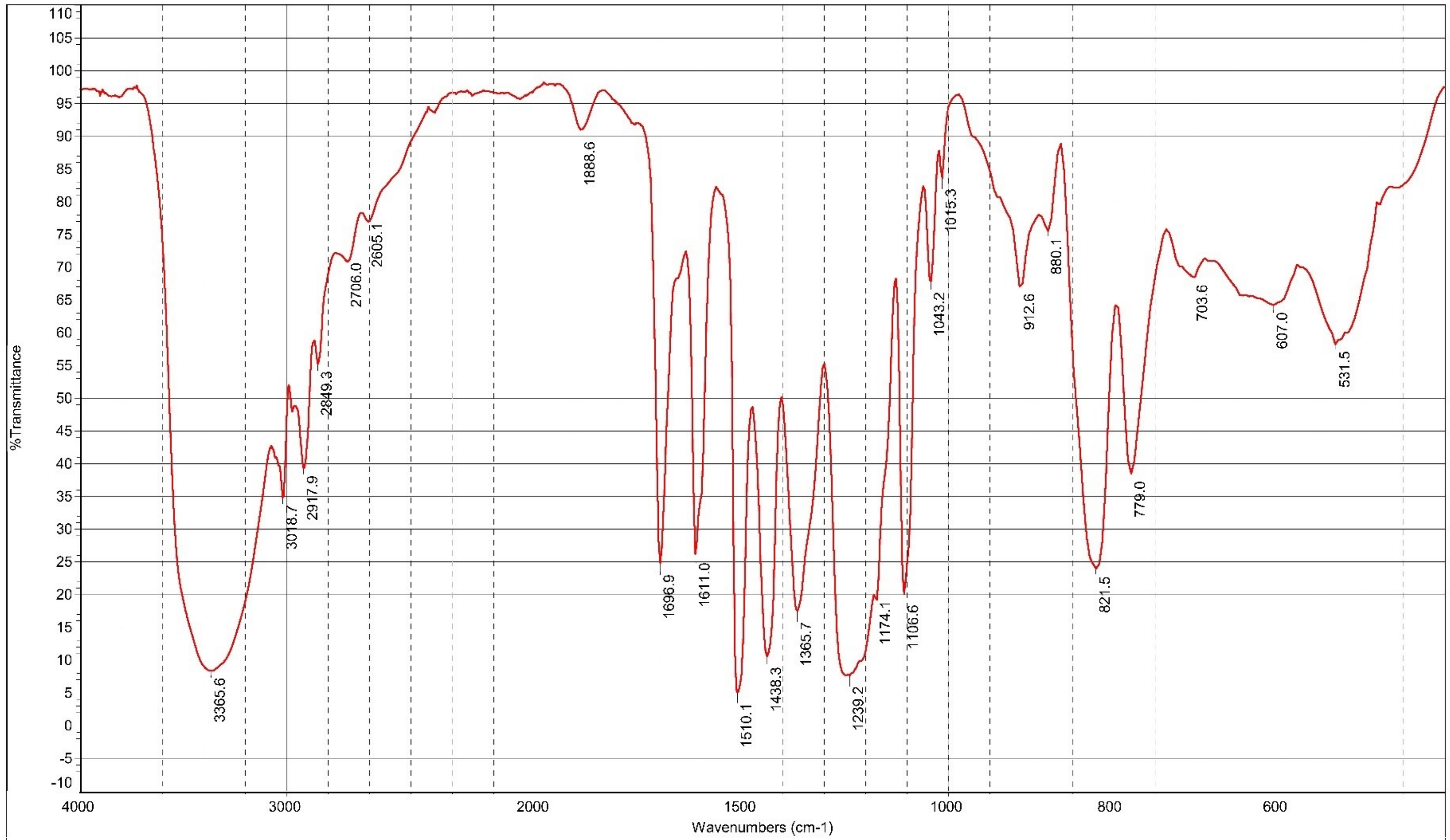

日期: 星期二 12月 15 15:56:23 2020 (GMT+08:00Sample Name： ZM-44 (显微镜透射法FT- IR Microscope Transmission)

扫描次数: 100

分辨率: 8.000

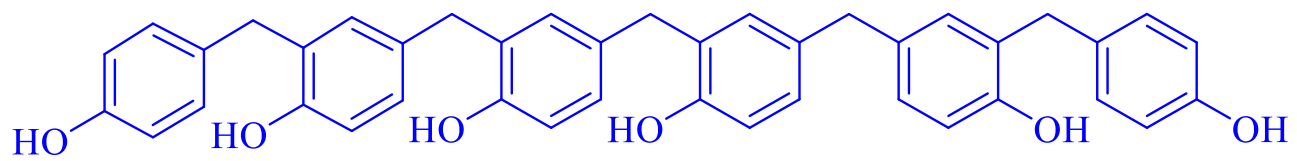

22

Figure S250. The IR spectrum of compound **22**

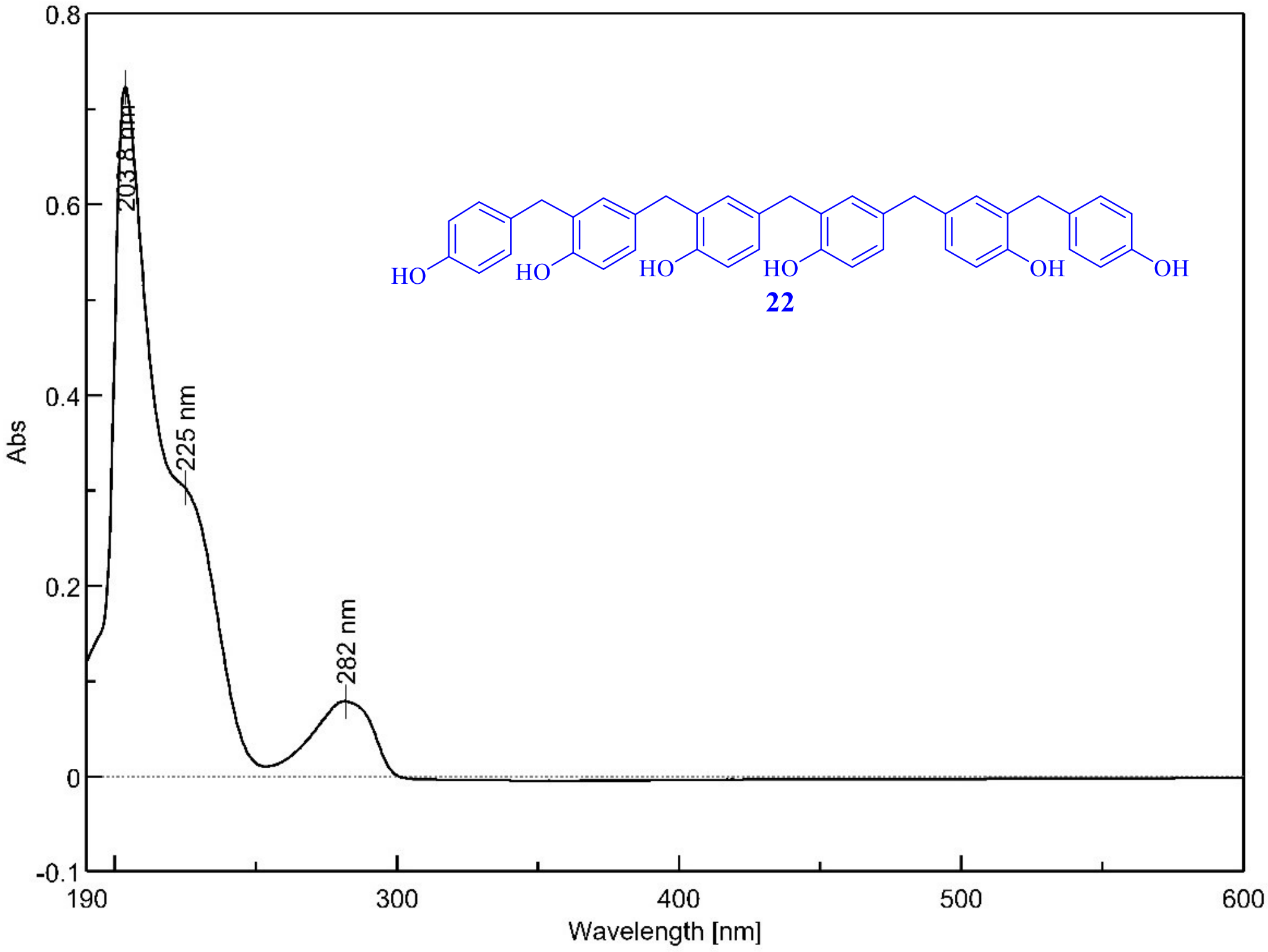

[Comment]
Sample Name DBT
Comment 0.02
User
Division UV
Company 324

ZM-44

[Measurement Information]
Instrument Name V-650
Model Name V-650
Serial No. A034461150

Accessory PSC-718
Accessory S/N A001761114
Position 1
Cell Length 10 mm
Temperature 19.96 C
Control Sonsor Holder
Monitor Sensor Holder
Start Mode Start immediately

Photometric Mode Abs
Measurement range 600 - 190 nm
Data pitch 0.2 nm
Band width(UV/Vis) 2.0 nm
Response Medium
Scanning speed 200 nm/min
Source Change 340 nm
Light Source D2/WI
Filter Exchange Step
Correction Baseline

[Data Information]
Creation Date 2020-11-11 17:02

Data array type Linear data array
Horizontal Wavelength [nm]
Vertical Abs
Start 600 nm
End 190 nm
Data pitch 0.2 nm
Data points 2051

Figure S251. The UV spectrum of compound **22** in MeOH

Thermo Qexactive Focus Report

compound NO. : ZM-44
Method : LCMS(compound)-low

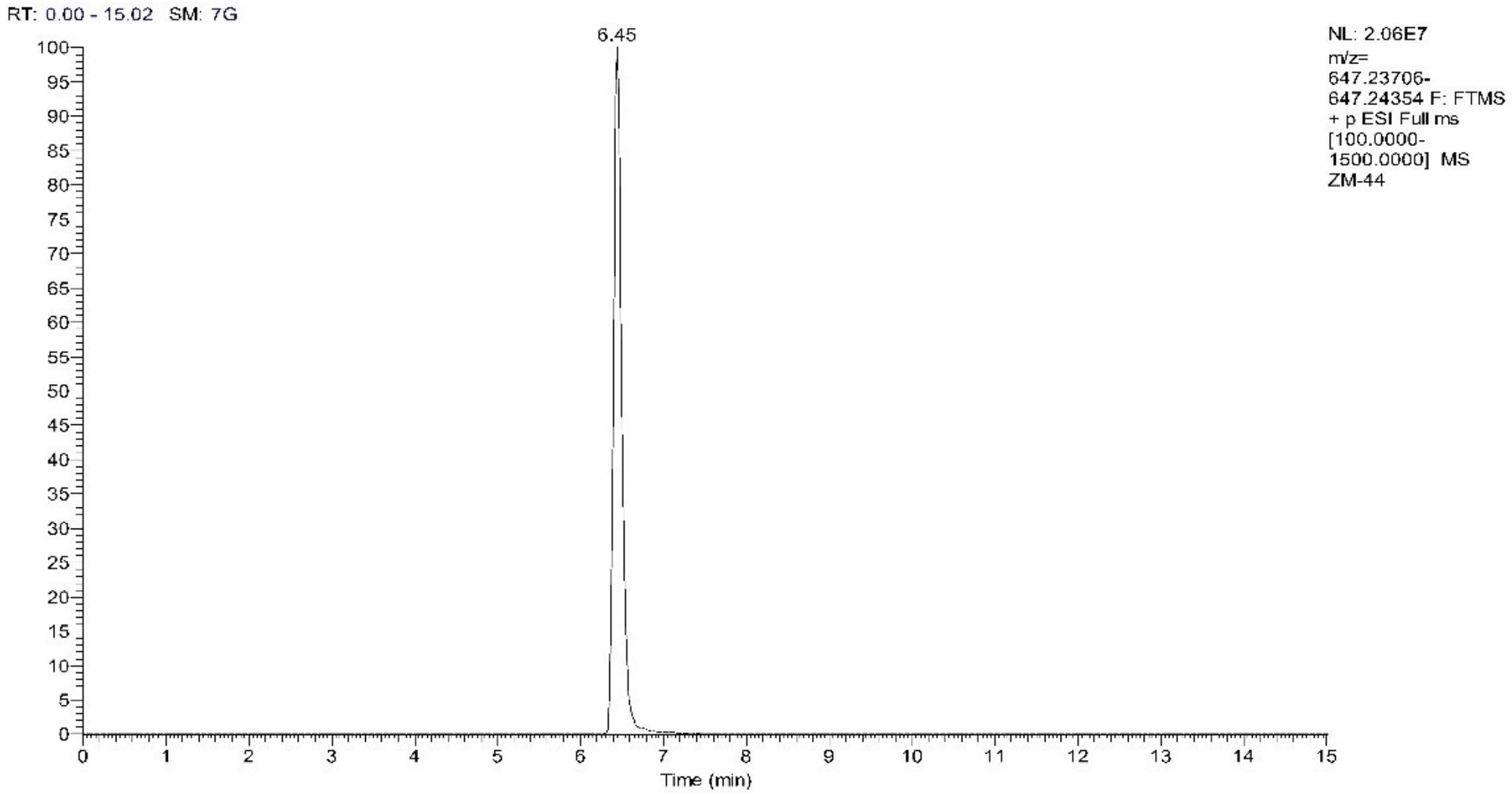

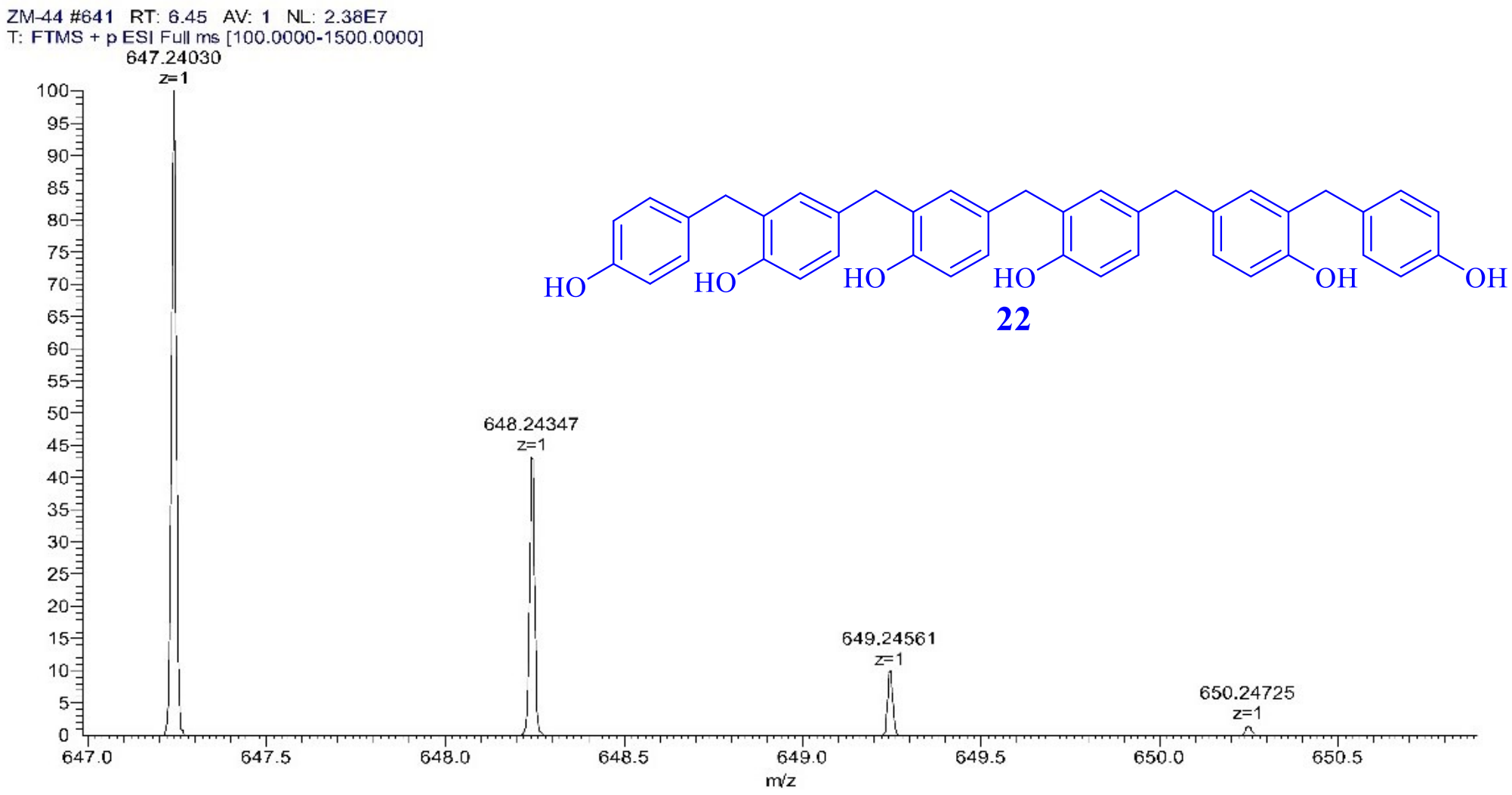

m/z	Theo. Mass	Delta (ppm)	RDB equiv.	Composition	
647.24030	647.24041	-0.17	23.5	C41 H36 O6 Na	M+Na

Figure S252. The HR-ESIMS report of compound **22**

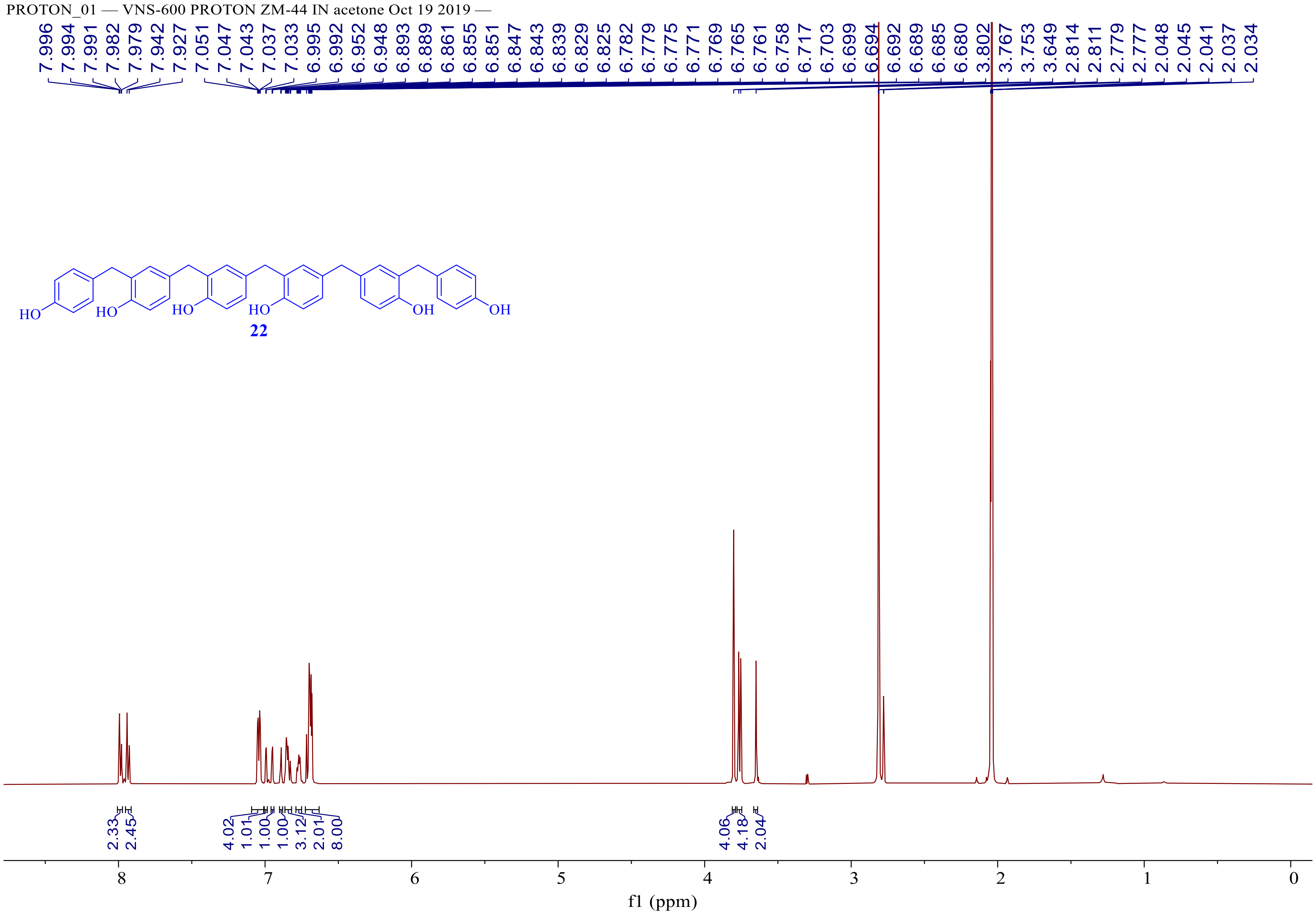

Figure S253. The ^{1}H NMR spectrum of compound **22** in acetone-d_6 (600 MHz)

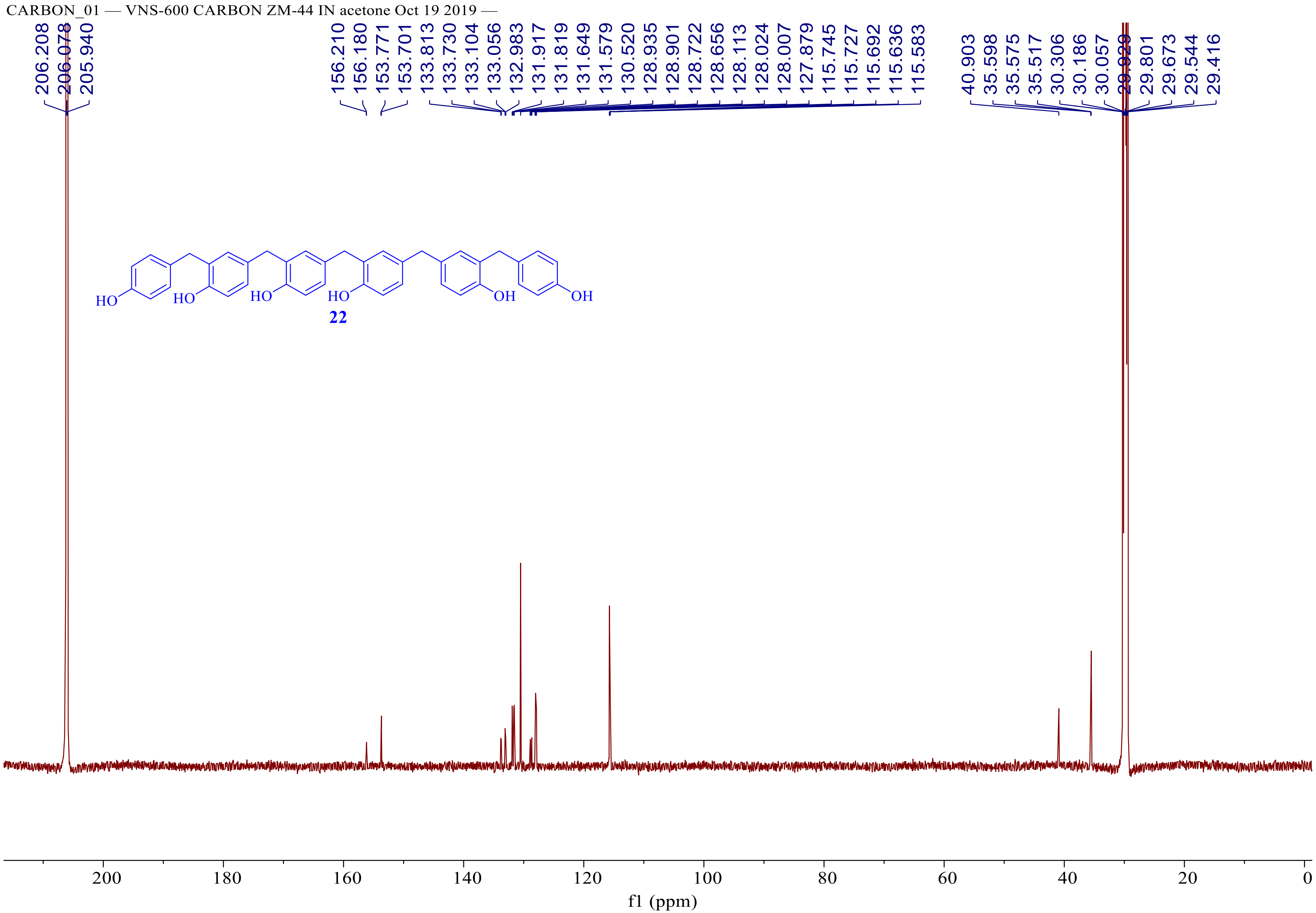

Figure S254. The ^{13}C NMR spectrum of compound **22** in acetone-d_6 (150 MHz)

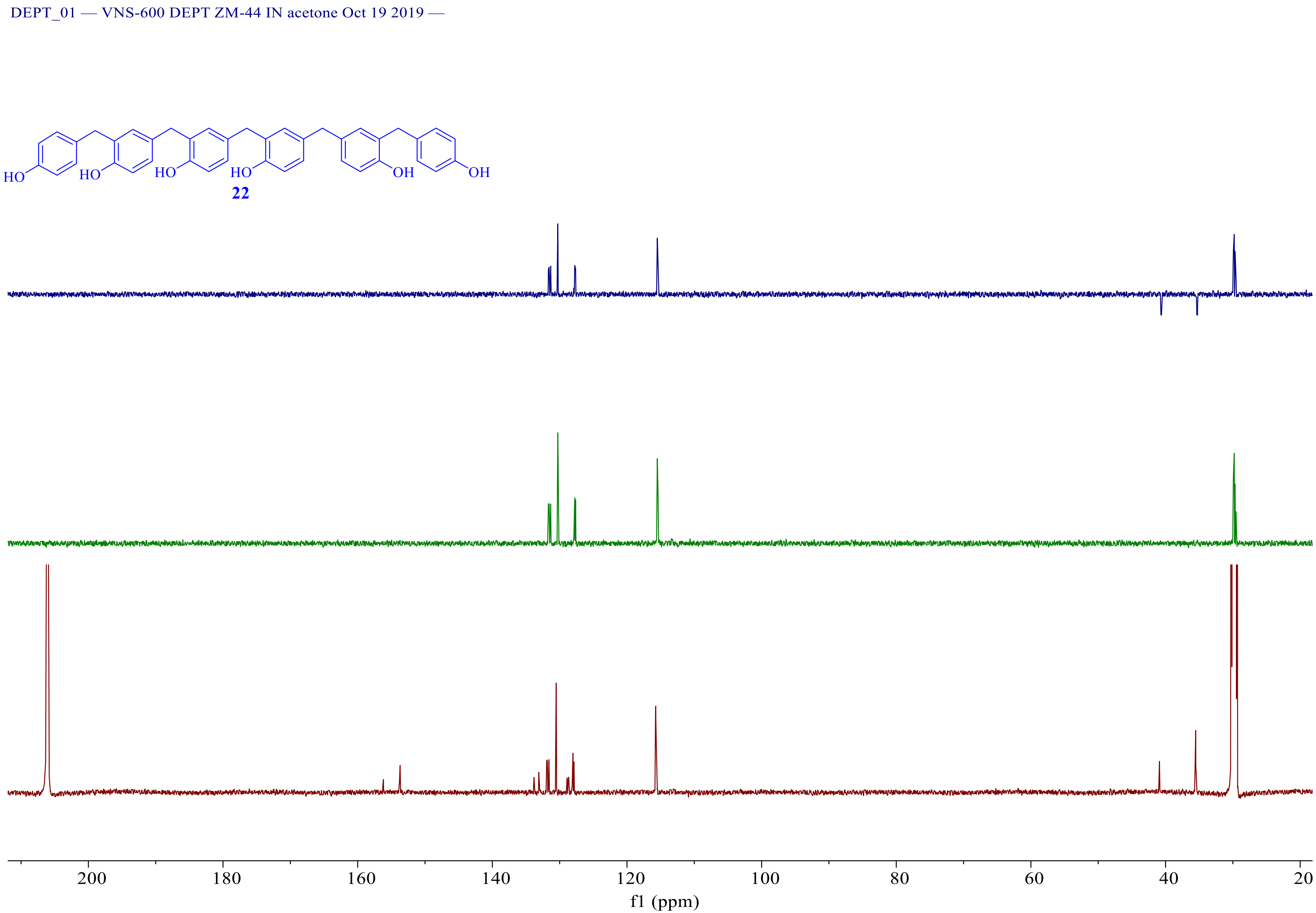

Figure S255. The DEPT spectrum of compound **22** in acetone-d_6 (150 MHz)

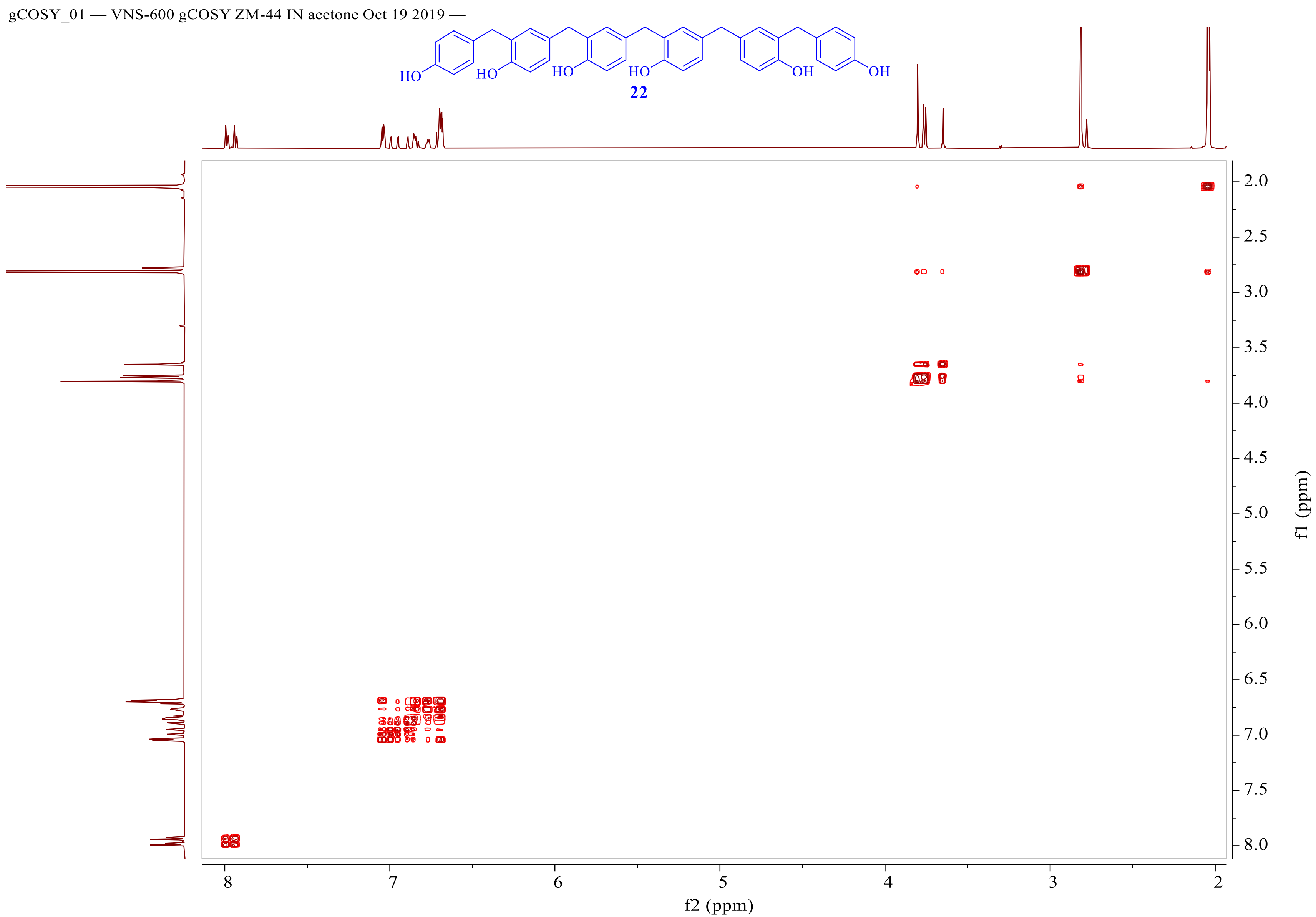

Figure S256. The ^{1}H-^{1}H COSY spectrum of compound **22** in acetone-d_6 (600 MHz)

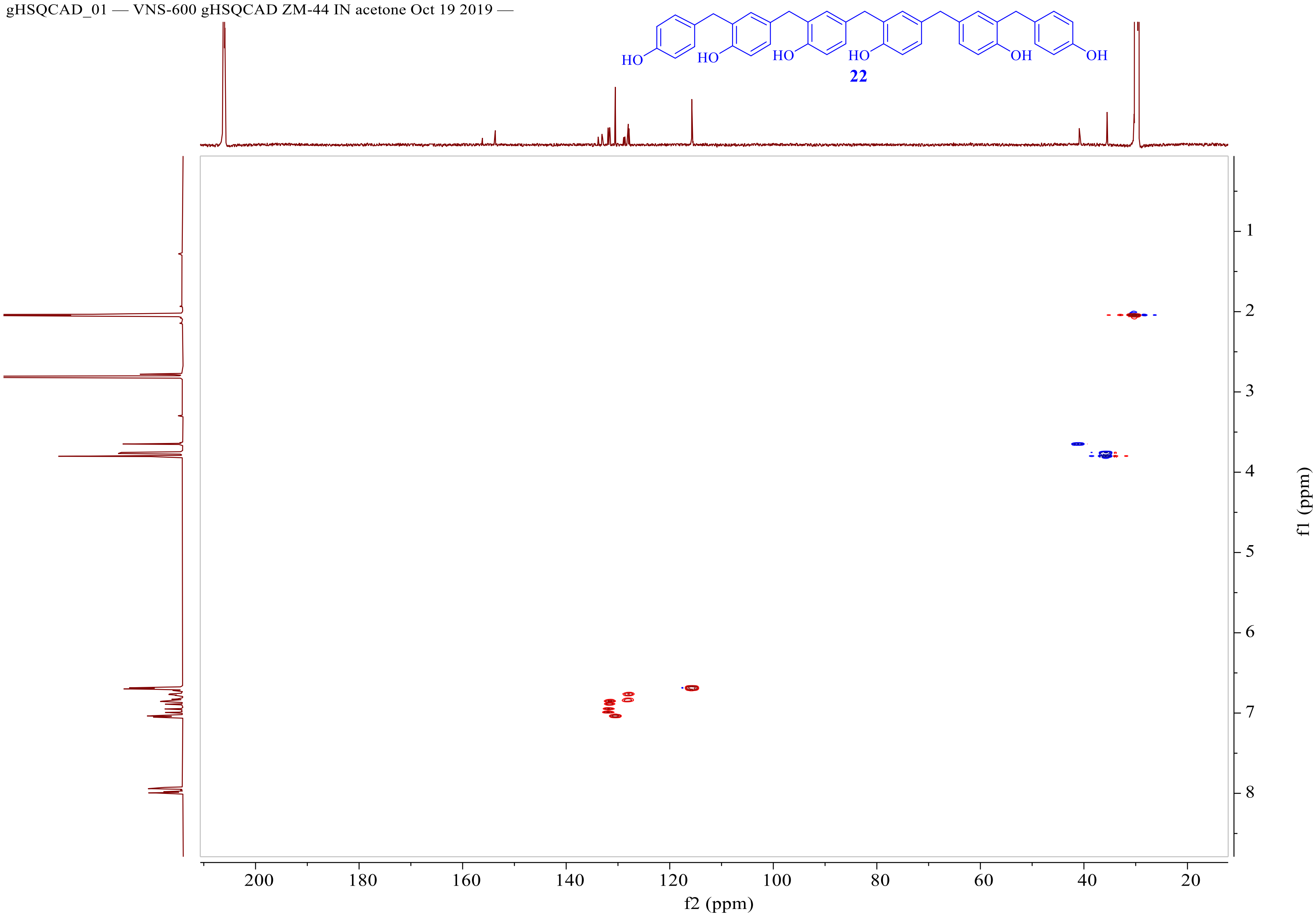

Figure S257. The HSQC spectrum of compound **22** in acetone-d_6 (600 MHz for ^{1}H)

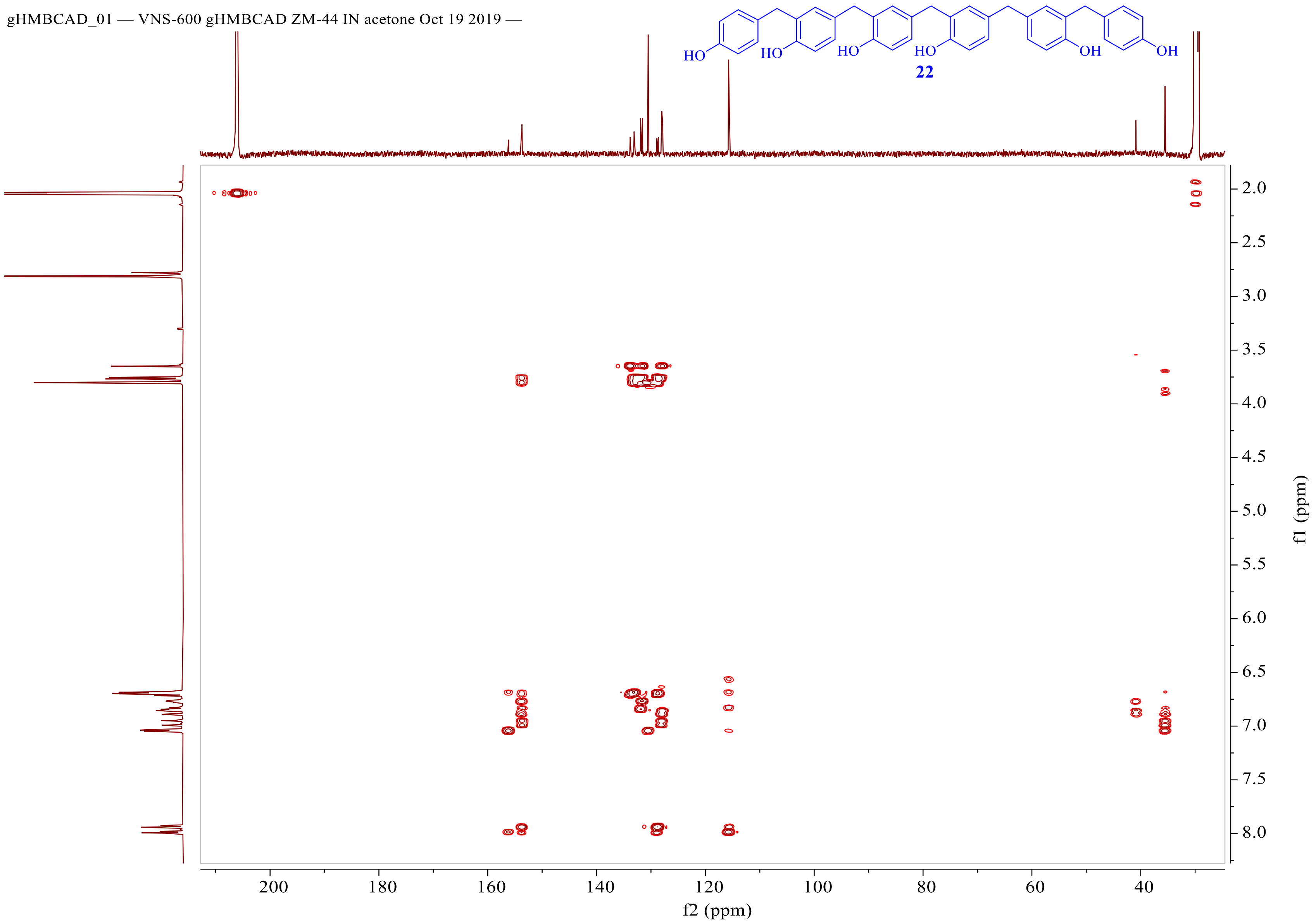

Figure S258. The HMBC spectrum of compound **22** in acetone-d_6 (600 MHz for ^{1}H)

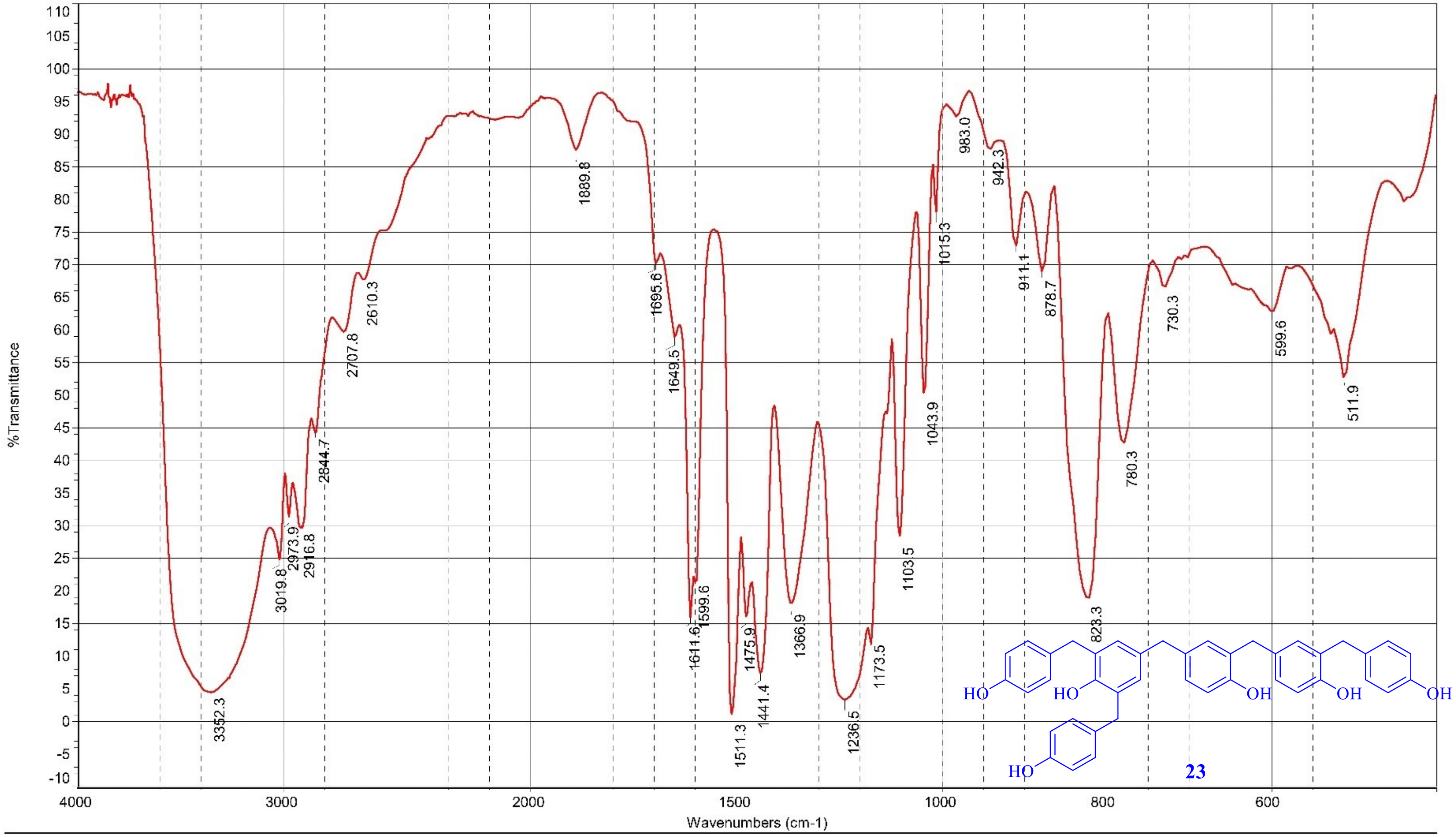

日期: 星期三 12月 02 16:38:31 2020 (GMT+08:00Sample Name：ZM-28 (显微镜透射法FT- IR Microscope Transmission)

扫描次数: 100

分辨率: 8.000

Figure S259. The IR spectrum of compound **23**

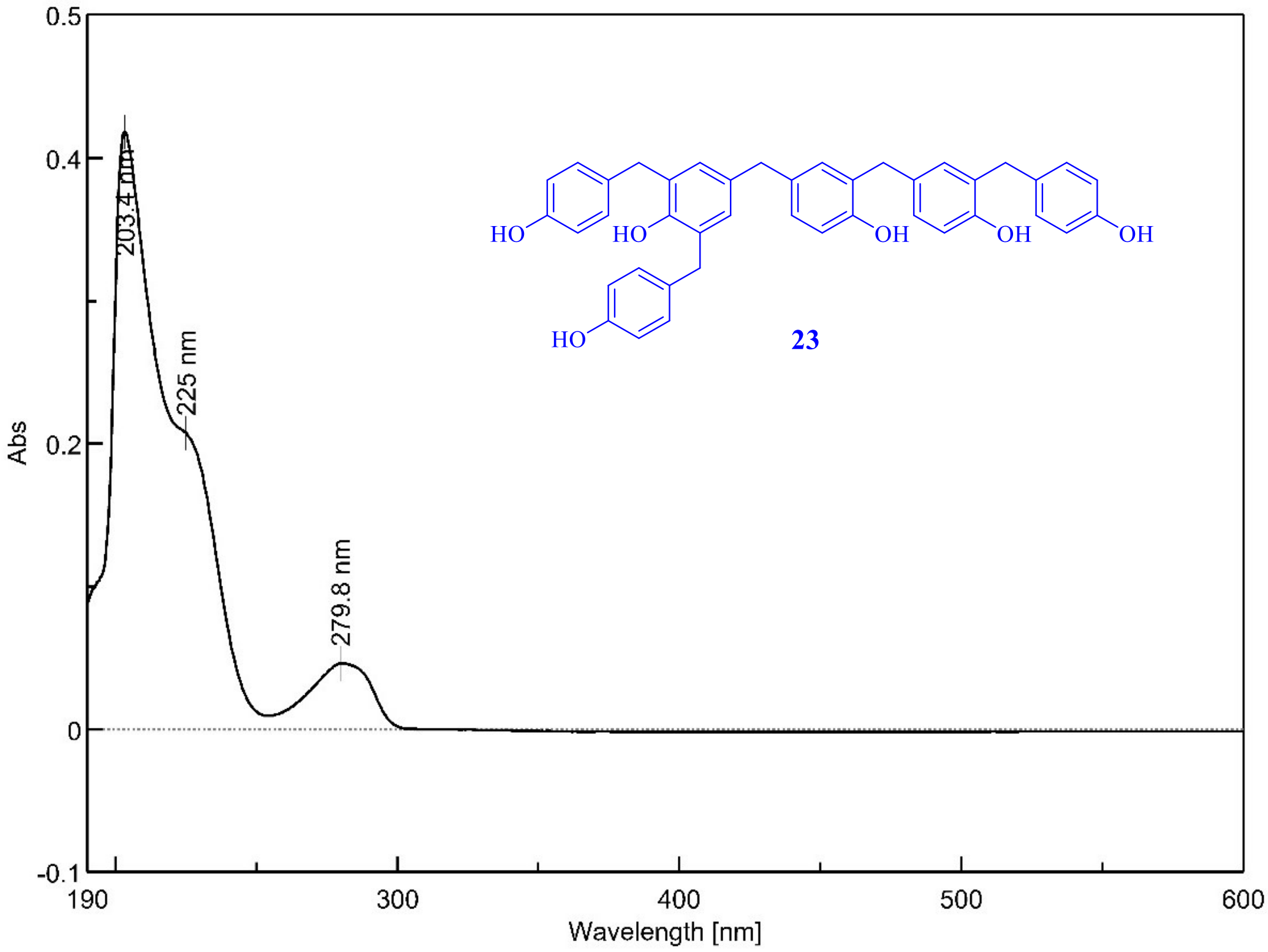

[Comment]
Sample Name DBT
Comment 0.02
User
Division UV
Company 324

ZM-28

[Measurement Information]
Instrument Name V-650
Model Name V-650
Serial No. A034461150

Accessory PSC-718
Accessory S/N A001761114
Position 1
Cell Length 10 mm
Temperature 20.01 C
Control Sonsor Holder
Monitor Sensor Holder
Start Mode Start immediately

Photometric Mode Abs
Measurement range 600 - 190 nm
Data pitch 0.2 nm
Band width(UV/Vis) 2.0 nm
Response Medium
Scanning speed 200 nm/min
Source Change 340 nm
Light Source D2/WI
Filter Exchange Step
Correction Baseline

[Data Information]
Creation Date 2020-11-11 16:45

Data array type Linear data array
Horizontal Wavelength [nm]
Vertical Abs
Start 600 nm
End 190 nm
Data pitch 0.2 nm
Data points 2051

Figure S260. The UV spectrum of compound **23** in MeOH

Thermo Qexactive Focus Report

compound NO. : ZM-28
Method : LCMS(compound)-low

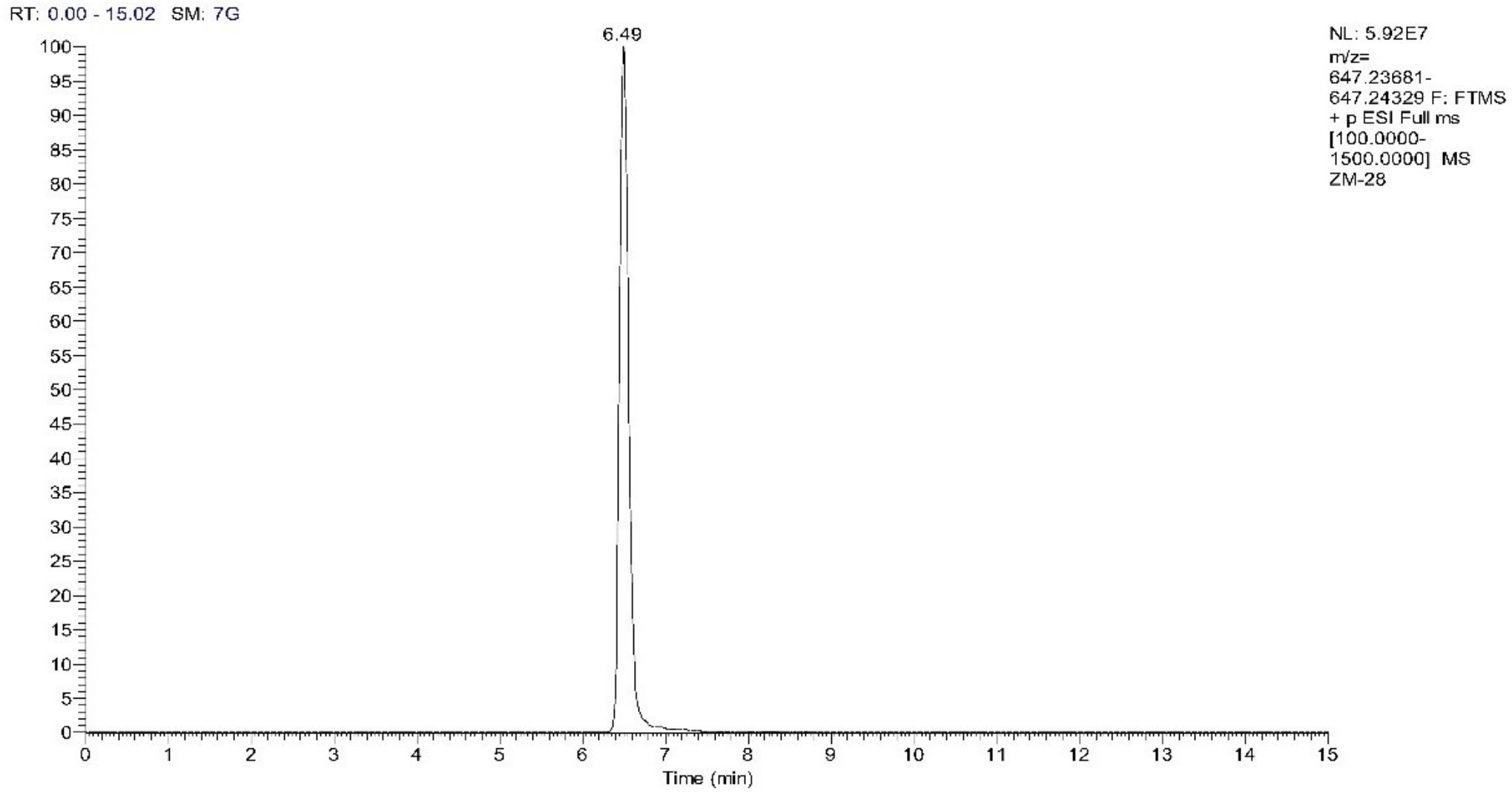

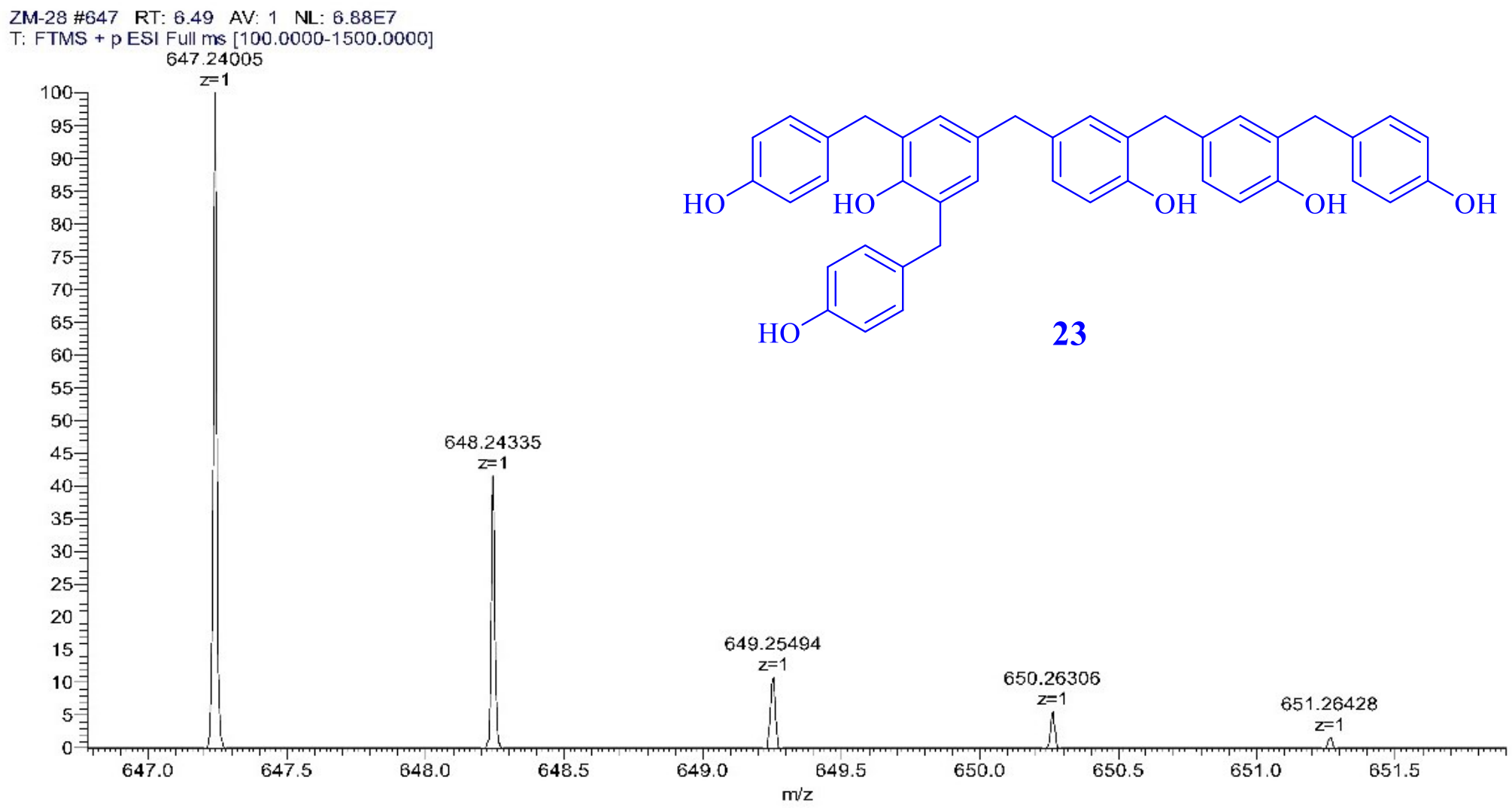

m/z	Theo. Mass	Delta (ppm)	RDB equiv.	Composition	
647.24005	647.24041	-0.56	23.5	C41 H36 O6 Na	M+Na

Figure S261. The HR-ESIMS report of compound **23**

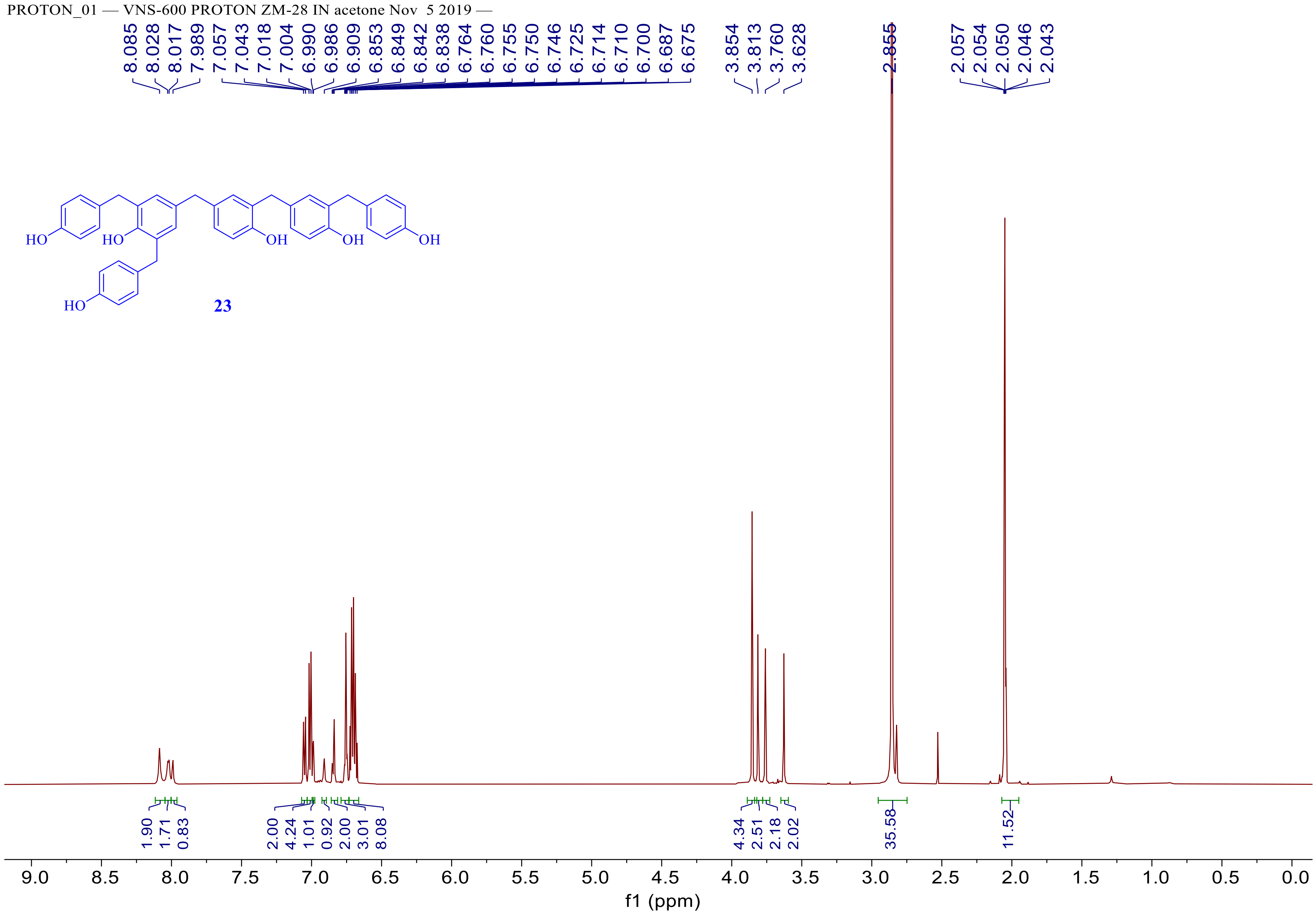

Figure S262. The ^{1}H NMR spectrum of compound **23** in acetone-d_6 (600 MHz)

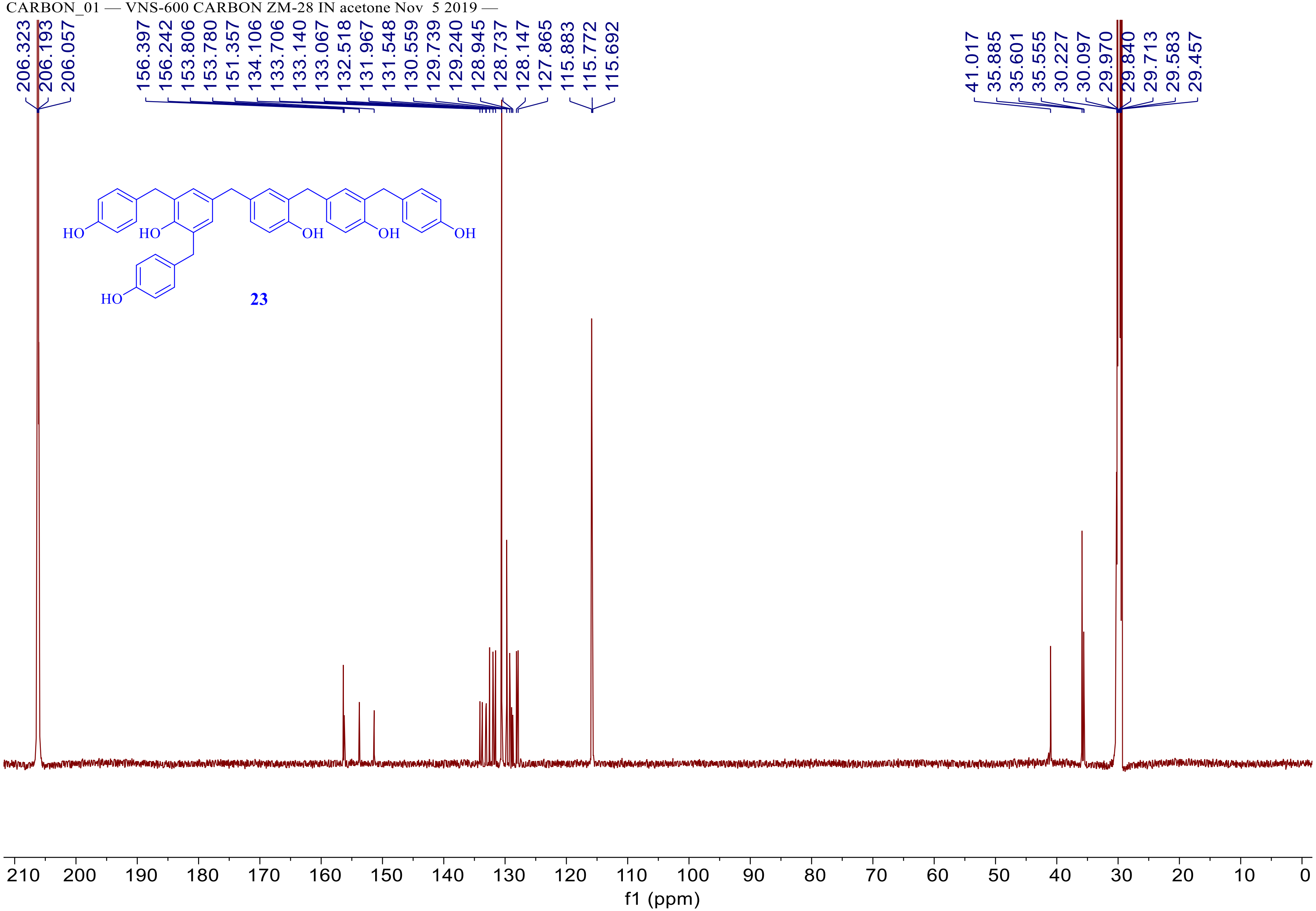

Figure S263. The ^{13}C NMR spectrum of compound **23** in acetone-d_6 (150 MHz)

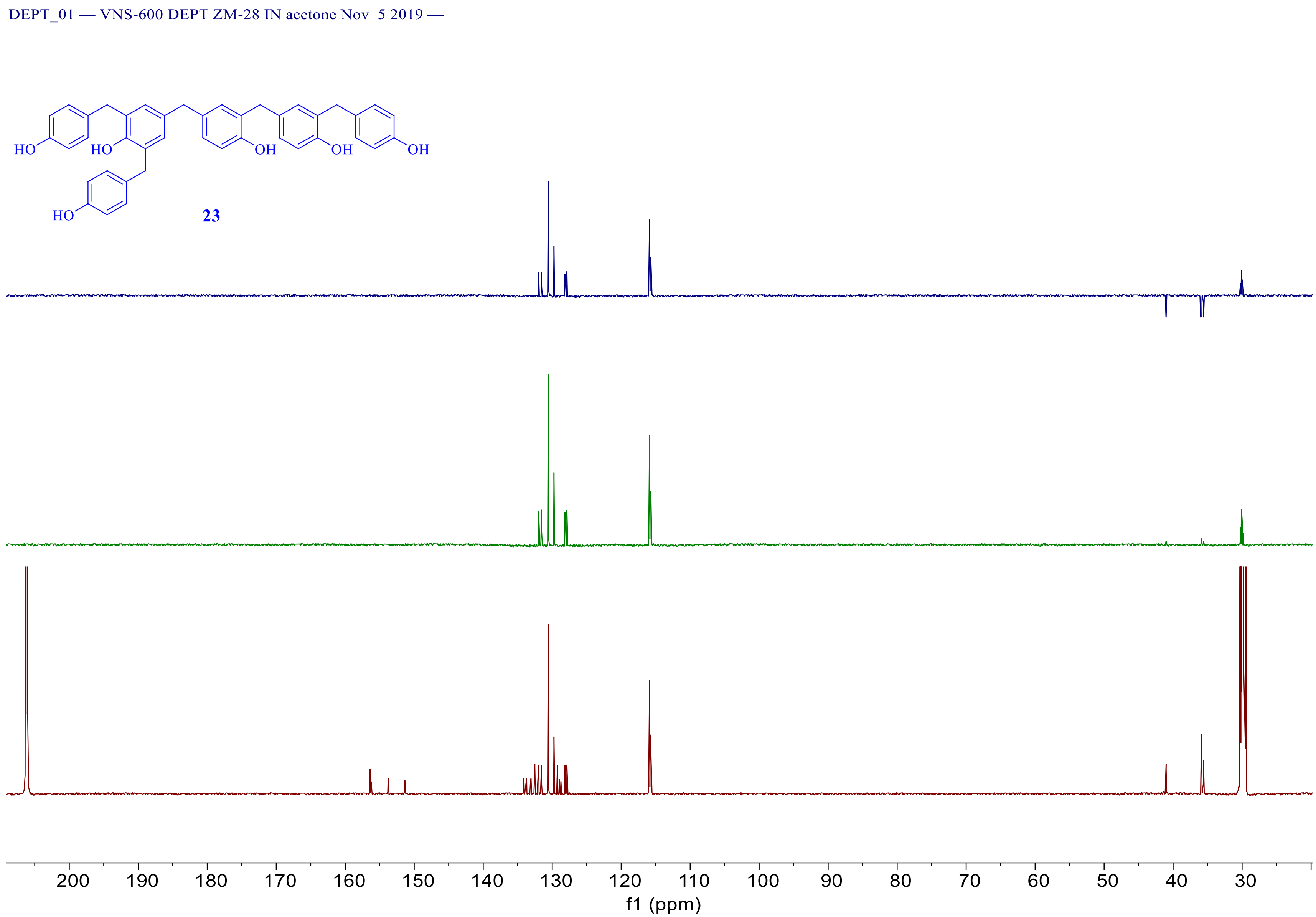

Figure S264. The DEPT spectrum of compound **23** in acetone-d_6 (150 MHz)

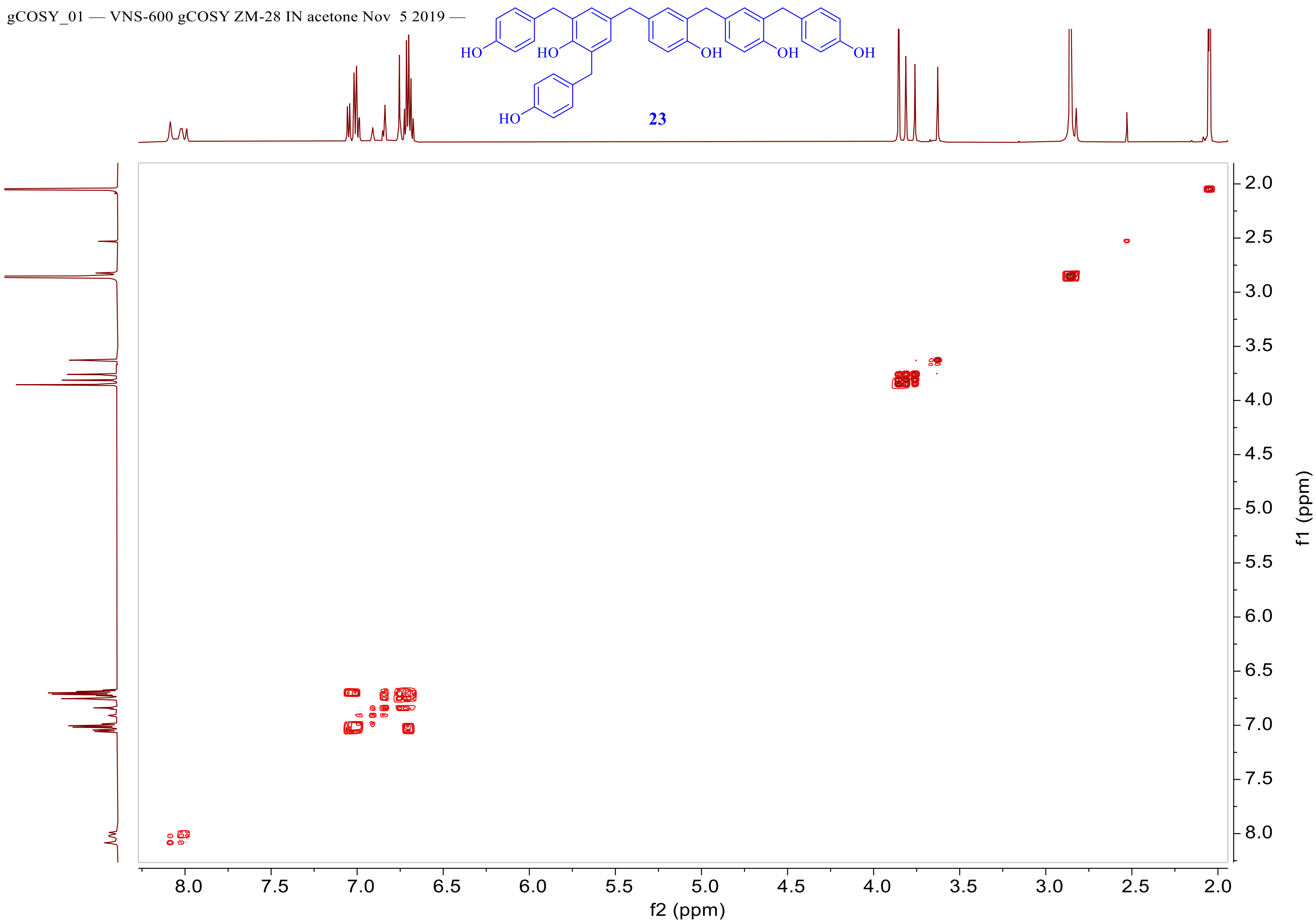

Figure S265. The ^{1}H-^{1}H COSY spectrum of compound **23** in acetone-d_6 (600 MHz)

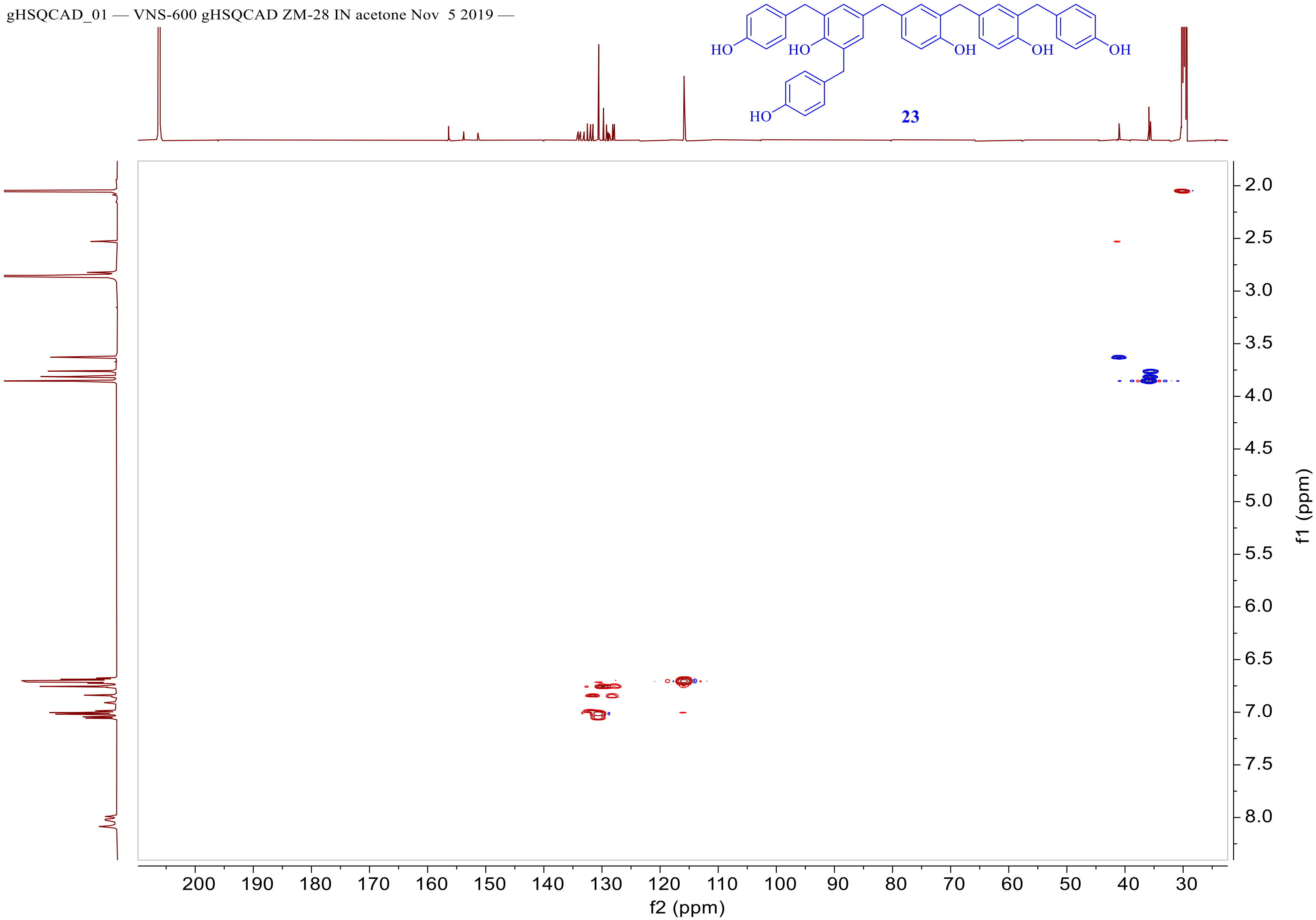

Figure S266. The HSQC spectrum of compound **23** in acetone-d_6 (600 MHz for ^{1}H)

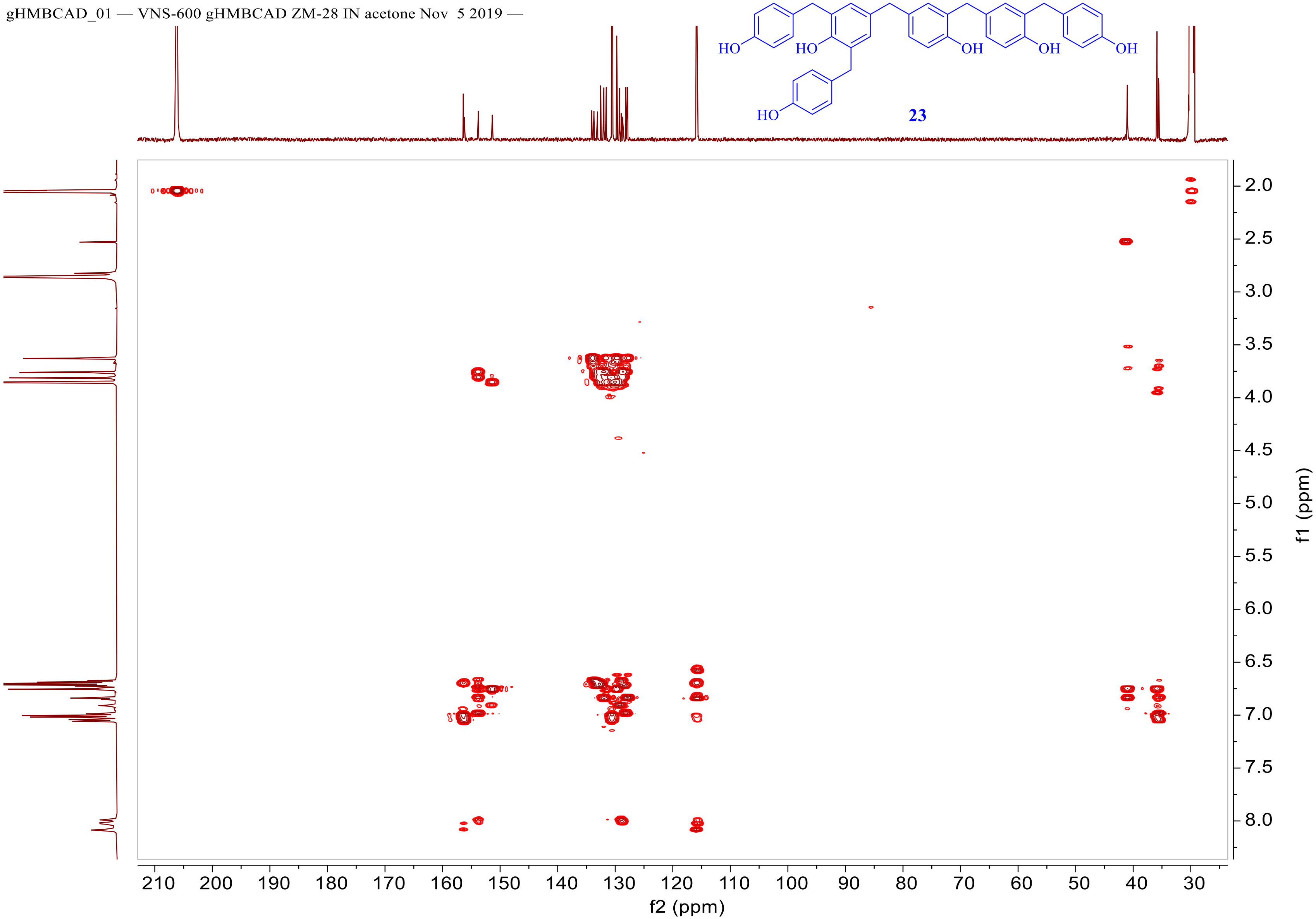

Figure S267. The HMBC spectrum of compound **23** in acetone-d_6 (600 MHz for ^1H)

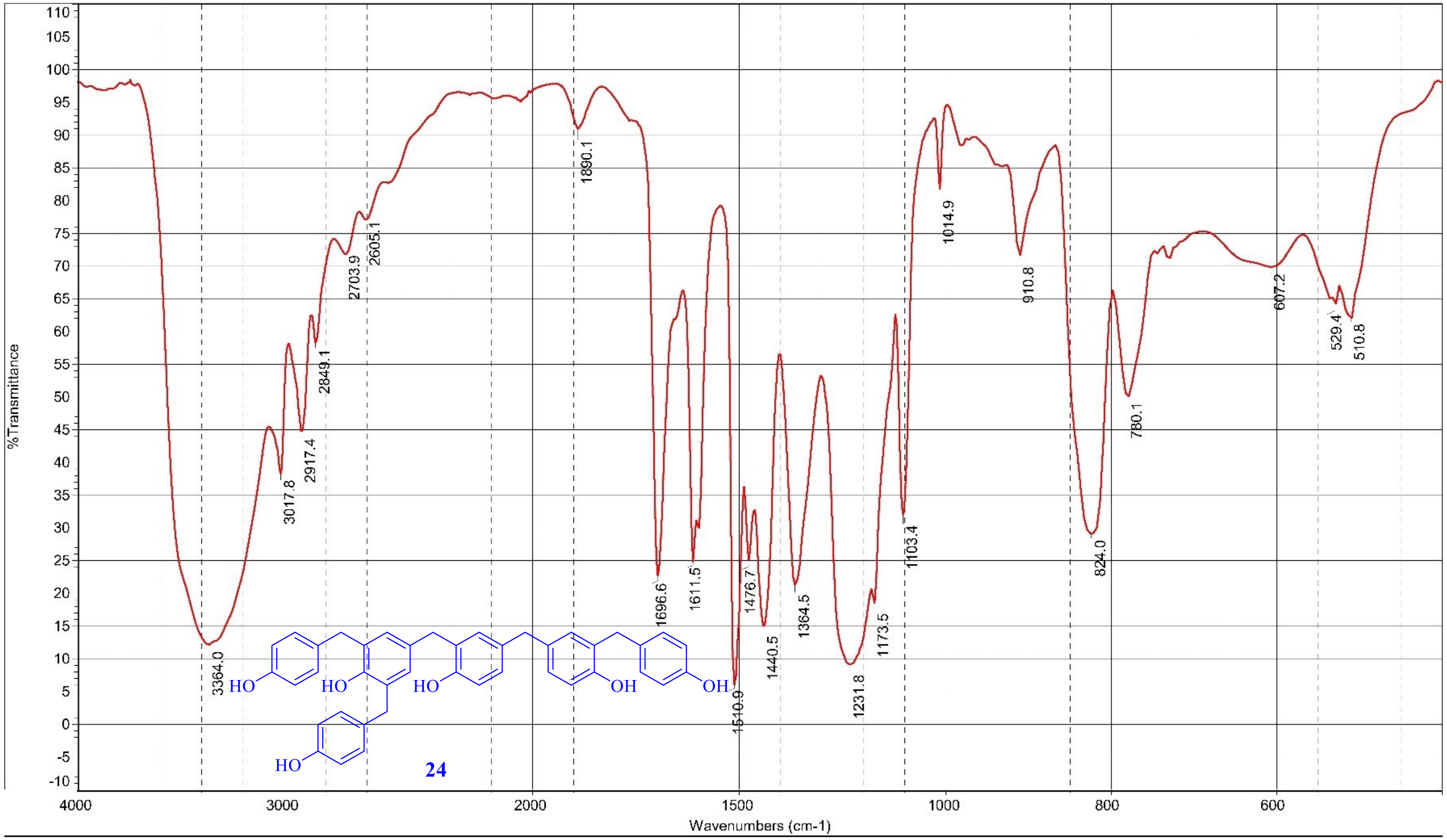

日期: 星期二 12月 22 10:52:23 2020 (GMT+08:00Sample Name：ZM-26 (显微镜透射法FT- IR Microscope Transmission)

扫描次数: 100

分辨率: 8.000

Figure S268. The IR spectrum of compound **24**

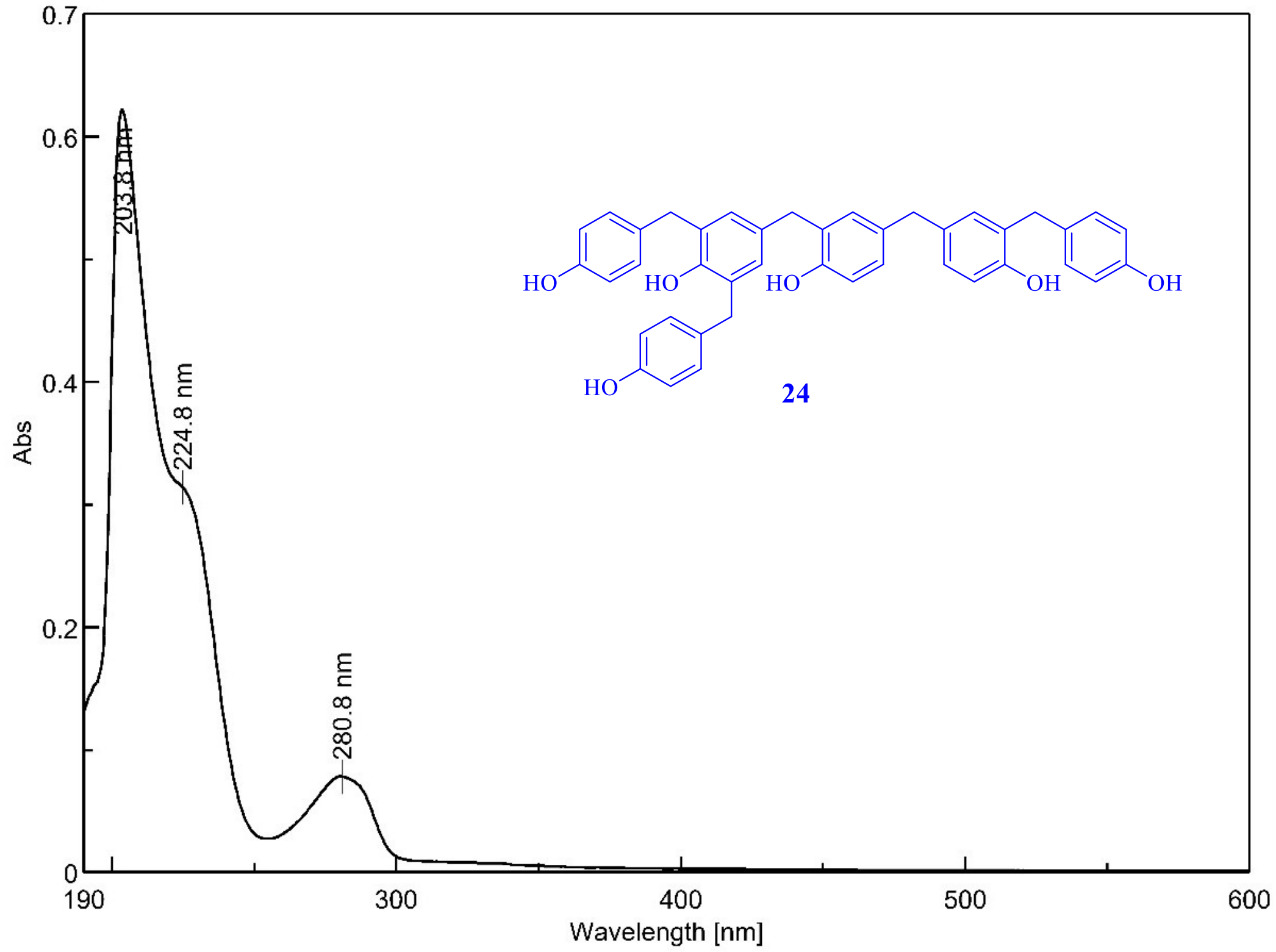

[Comment]
Sample Name DBT
Comment 0.02
User
Division UV
Company 324

ZM-26

[Measurement Information]
Instrument Name V-650
Model Name V-650
Serial No. A034461150

Accessory PSC-718
Accessory S/N A001761114
Position 1
Cell Length 10 mm
Temperature 20.01 C
Control Sonsor Holder
Monitor Sensor Holder
Start Mode Start immediately

Photometric Mode Abs
Measurement range 600 - 190 nm
Data pitch 0.2 nm
Band width(UV/Vis) 2.0 nm
Response Medium
Scanning speed 200 nm/min
Source Change 340 nm
Light Source D2/WI
Filter Exchange Step
Correction Baseline

[Data Information]
Creation Date 2020-11-11 16:37

Data array type Linear data array
Horizontal Wavelength [nm]
Vertical Abs
Start 600 nm
End 190 nm
Data pitch 0.2 nm
Data points 2051

Figure S269. The UV spectrum of compound **24** in MeOH

Thermo Qexactive Focus Report

compound NO. : ZM-26
Method : LCMS(compound)-low

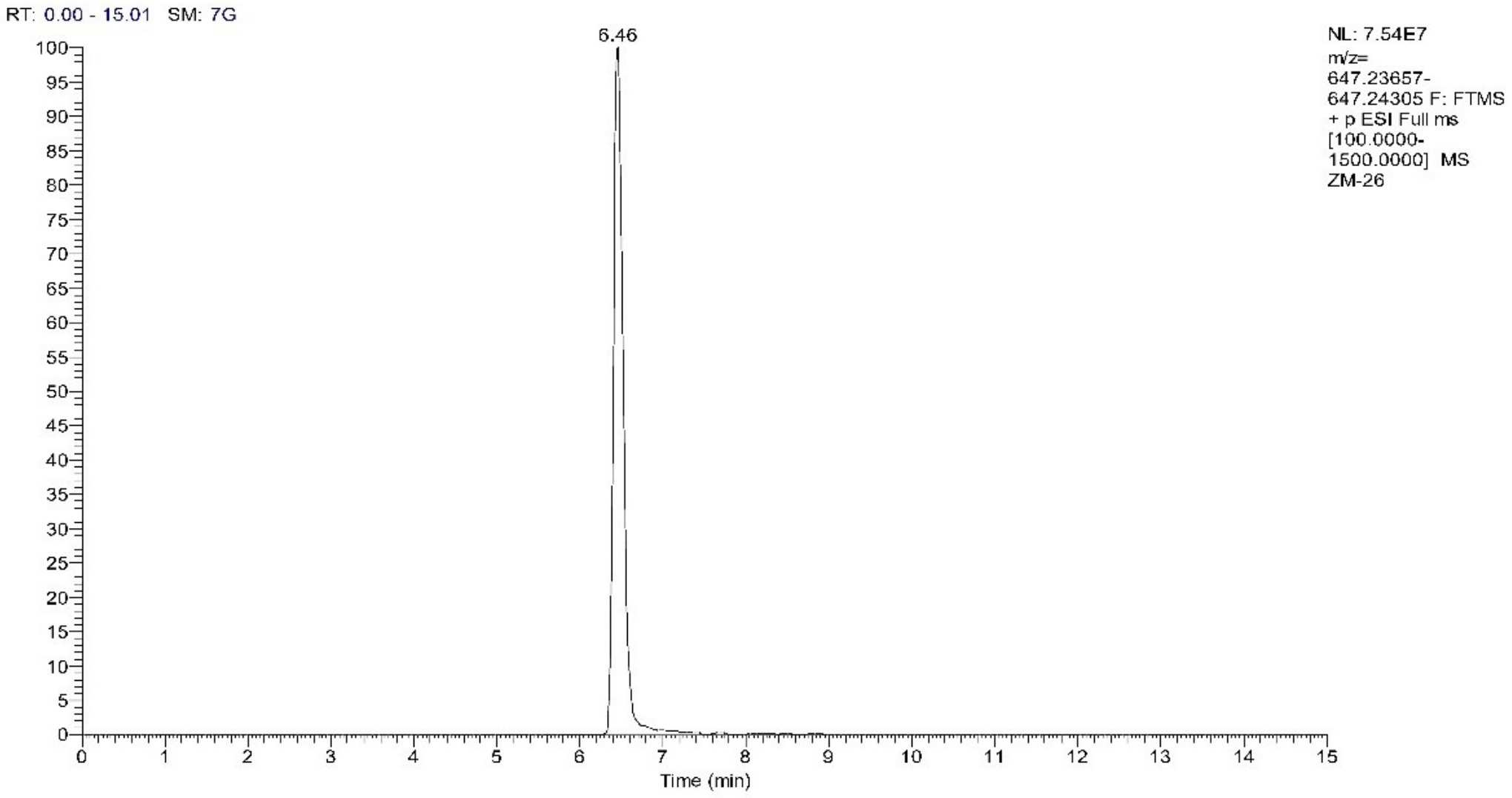

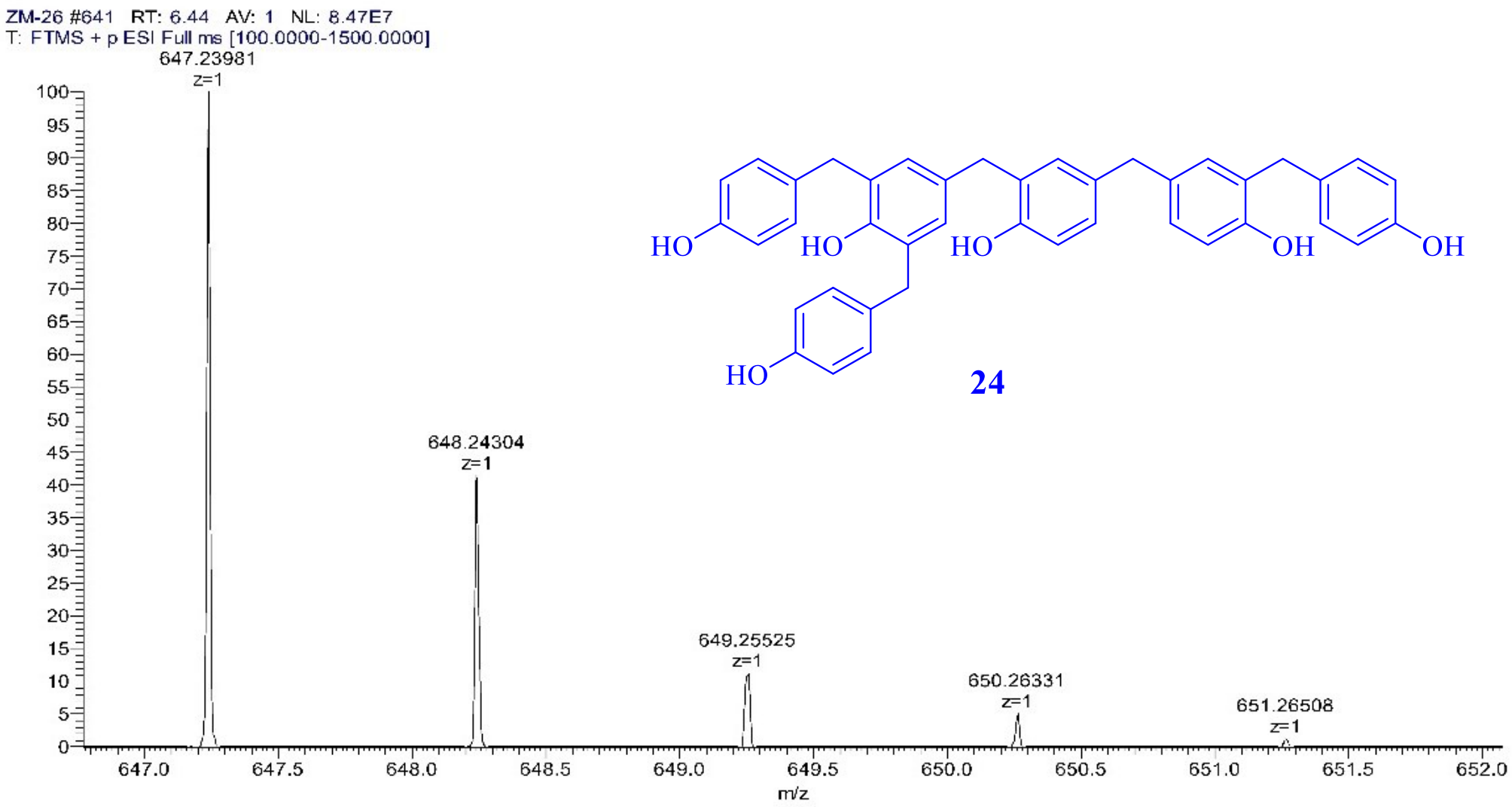

m/z	Theo. Mass	Delta (ppm)	RDB equiv.	Composition	
647.23981	647.24041	-0.93	23.5	C41 H36 O6 Na	M+Na

Figure S270. The HR-ESIMS report of compound **24**

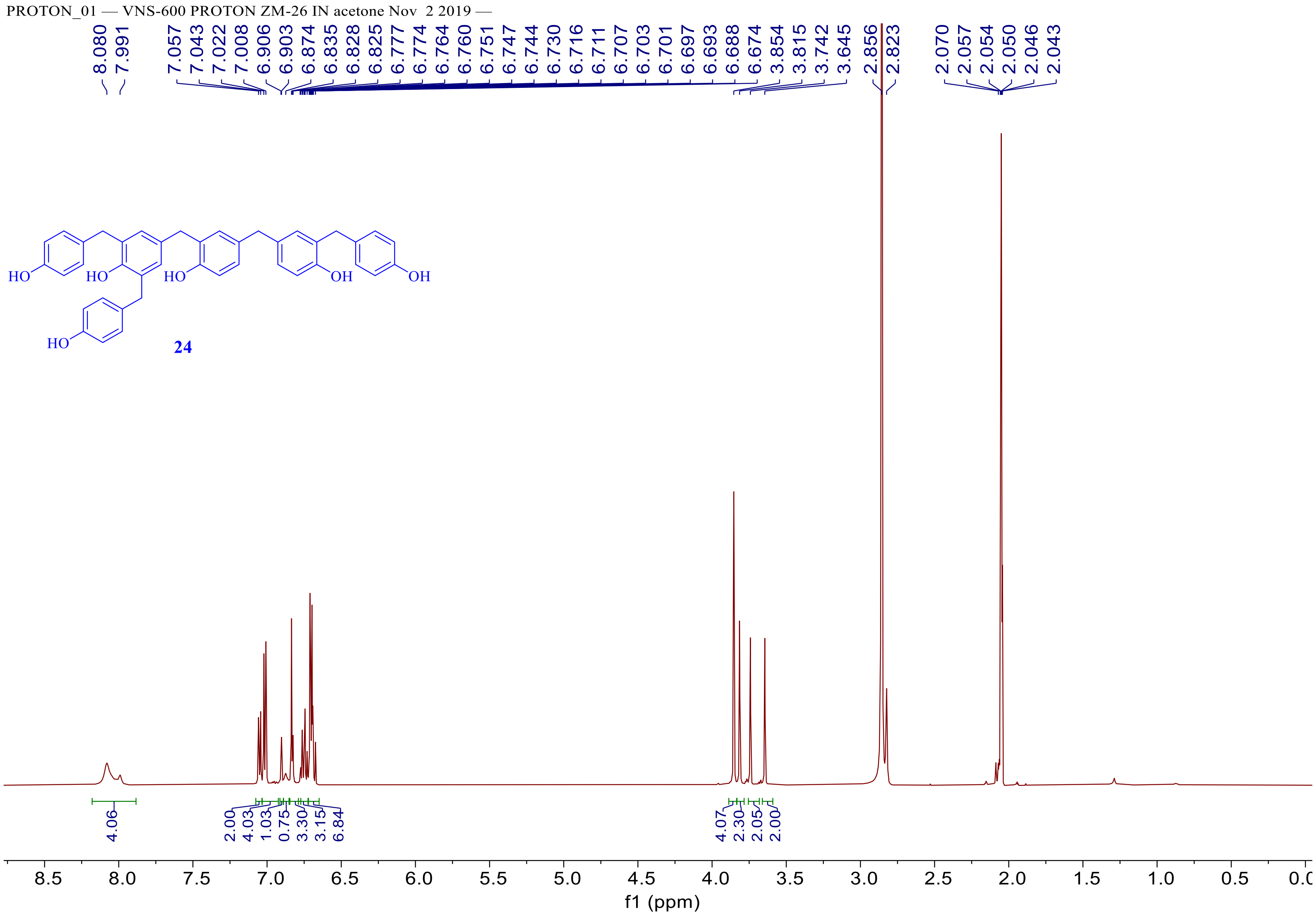

Figure S271. The ^{1}H NMR spectrum of compound **24** in acetone-d_6 (600 MHz)

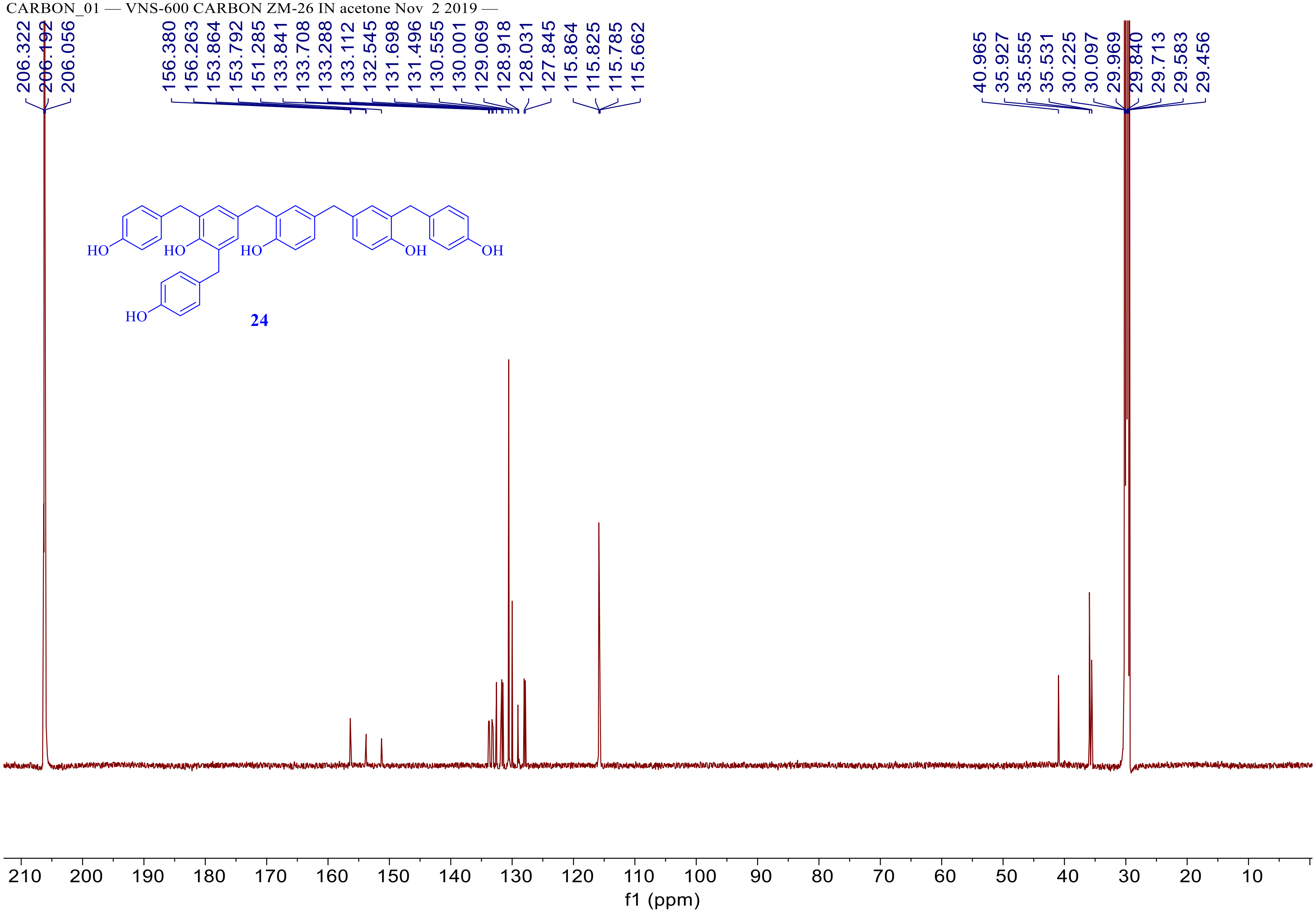

Figure S272. The ^{13}C NMR spectrum of compound **24** in acetone-d_6 (150 MHz)

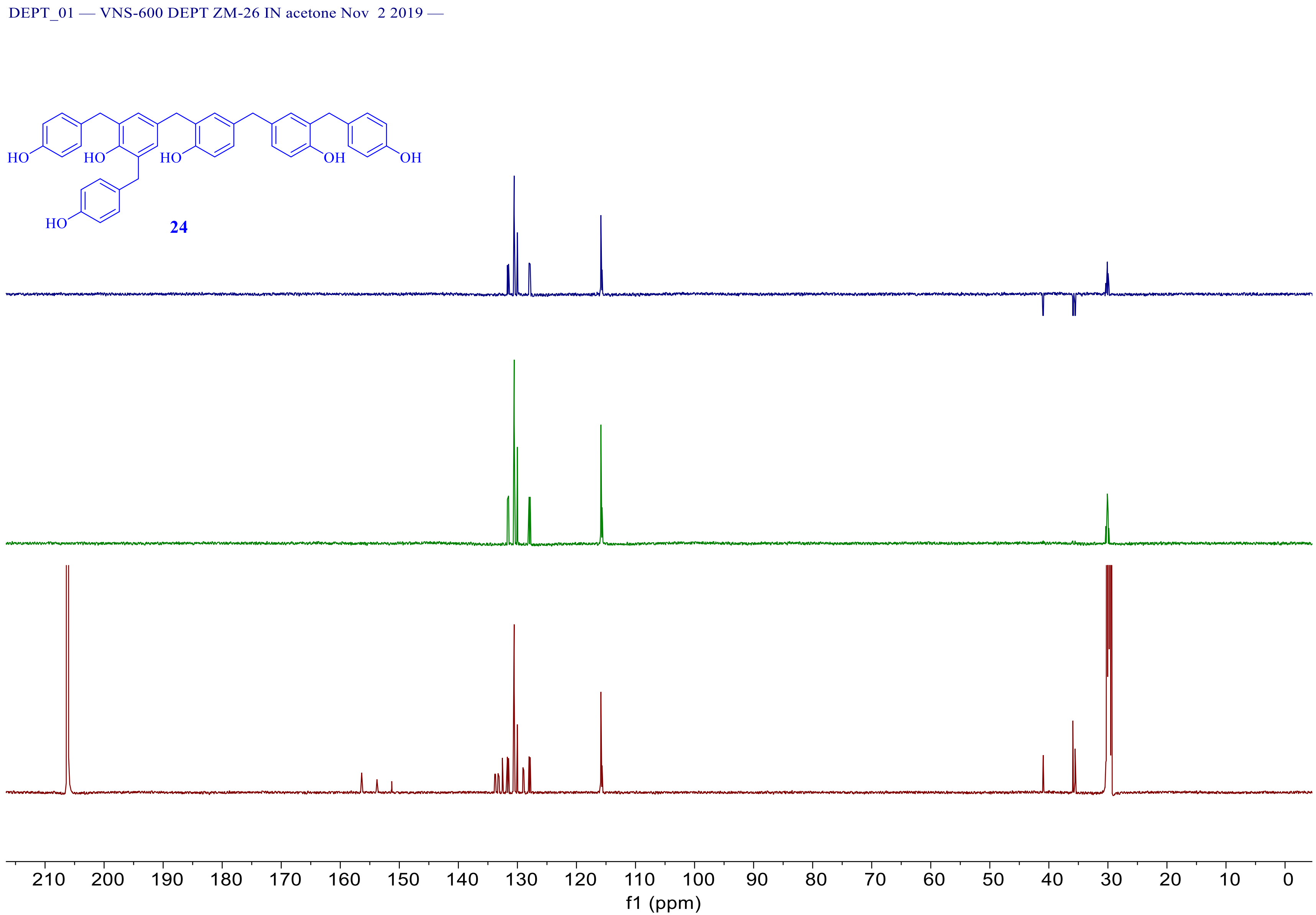

Figure S273. The DEPT spectrum of compound **24** in acetone-d_6 (150 MHz)

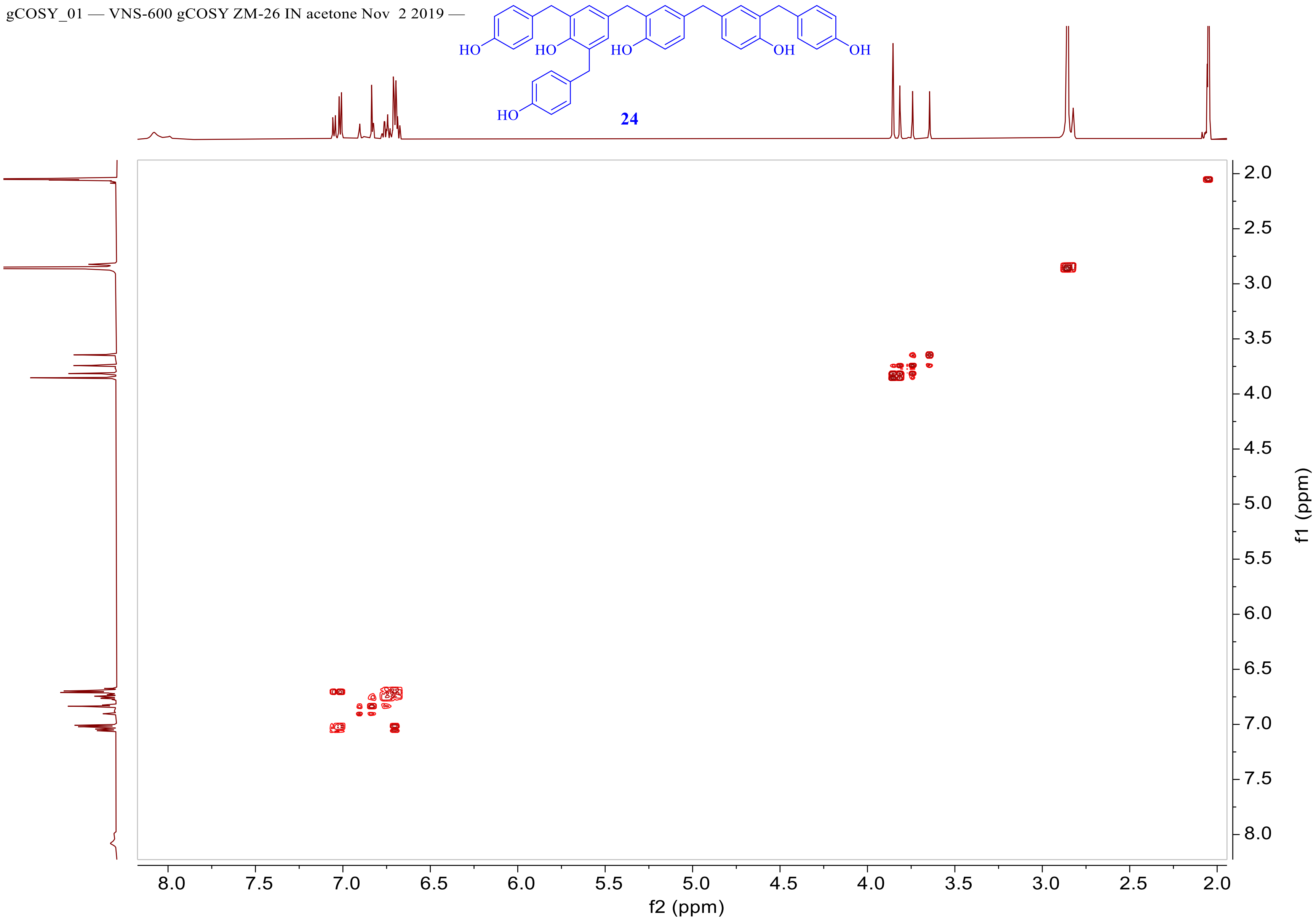

Figure S274. The ^{1}H-^{1}H COSY spectrum of compound **24** in acetone-d_6 (600 MHz)

gHSQCAD_01 — VNS-600 gHSQCAD ZM-26 IN acetone Nov 2 2019 —

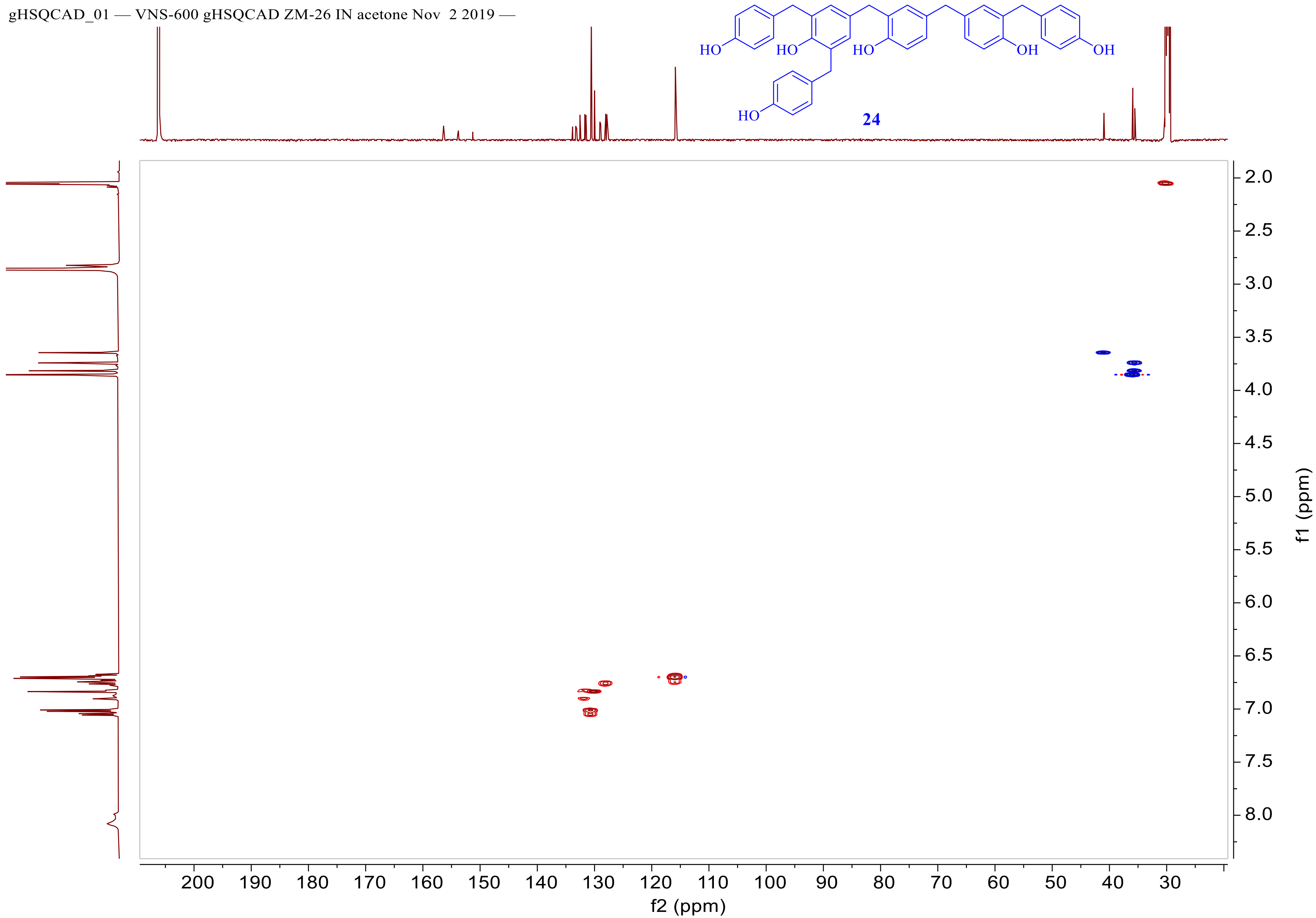

Figure S275. The HSQC spectrum of compound **24** in acetone-d_6 (600 MHz for ^{1}H)

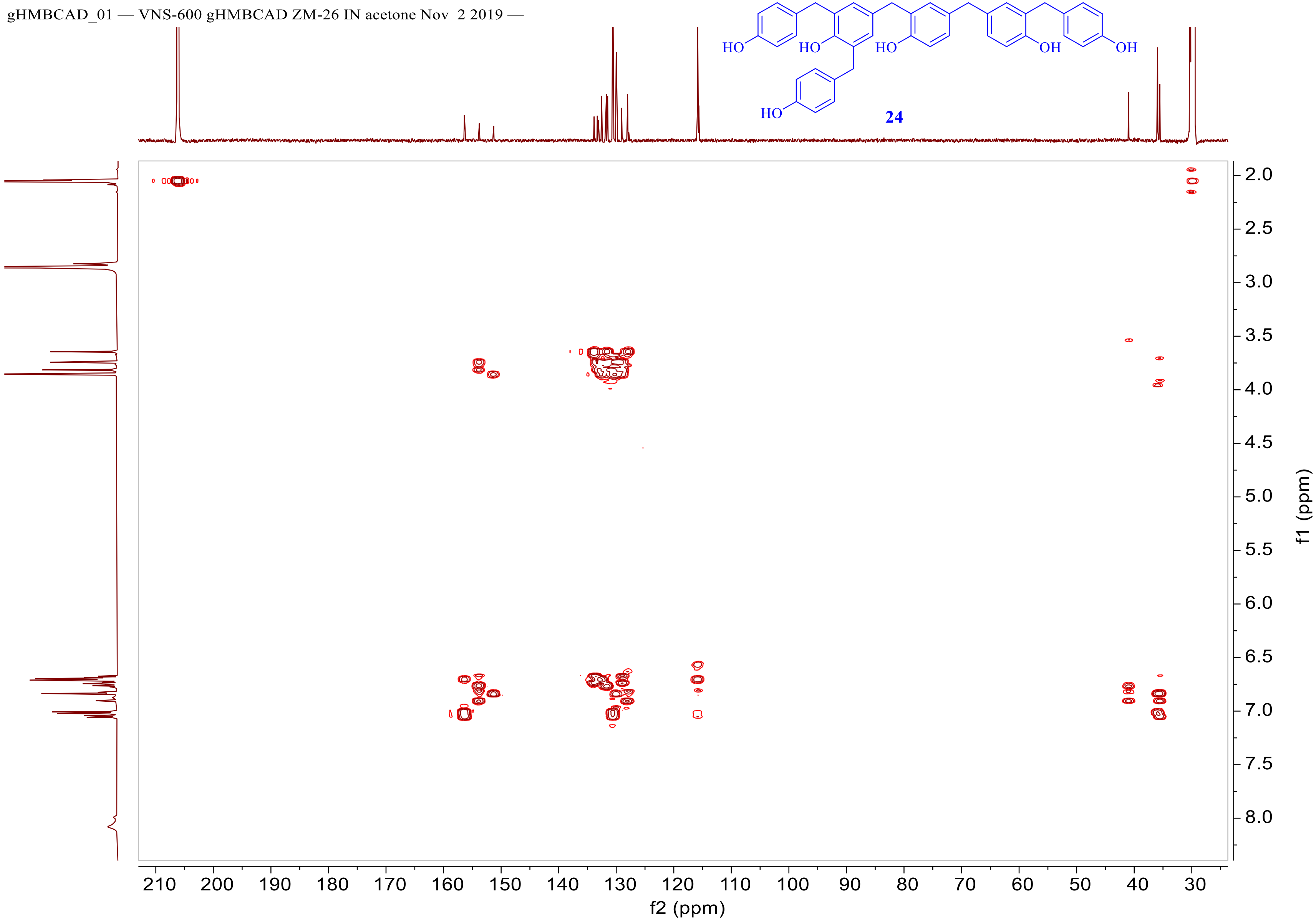

Figure S276. The HMBC spectrum of compound **24** in acetone-d_6 (600 MHz for ^{1}H)

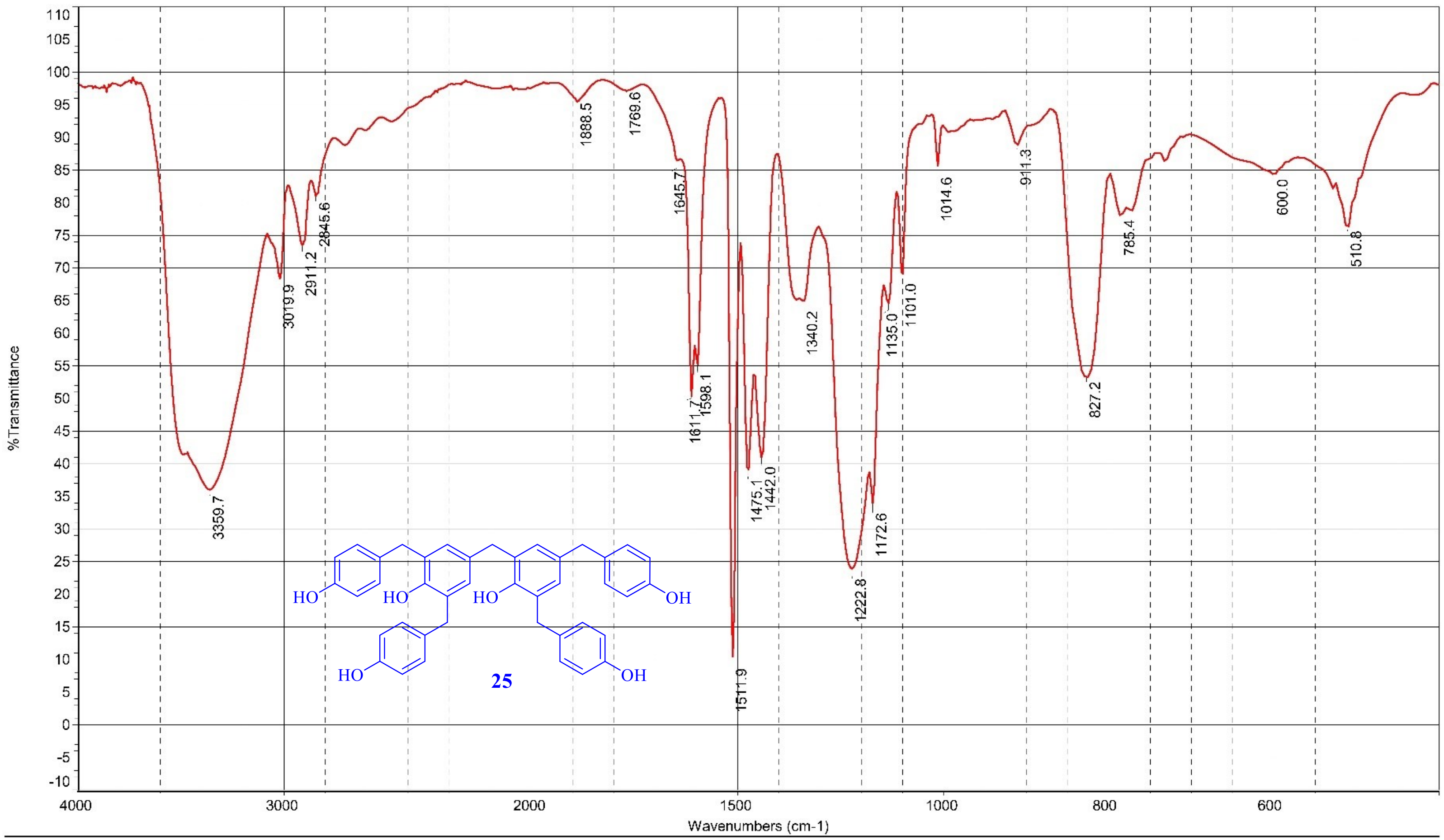

日期: 星期 ·12月 21 16:29:21 2020 (GMT+08:00Sample Name： ZM-29 (显微镜透射法FT- IR Microscope Transmission)

扌描次数: 100

分辨率: 8.000

Figure S277. The IR spectrum of compound **25**

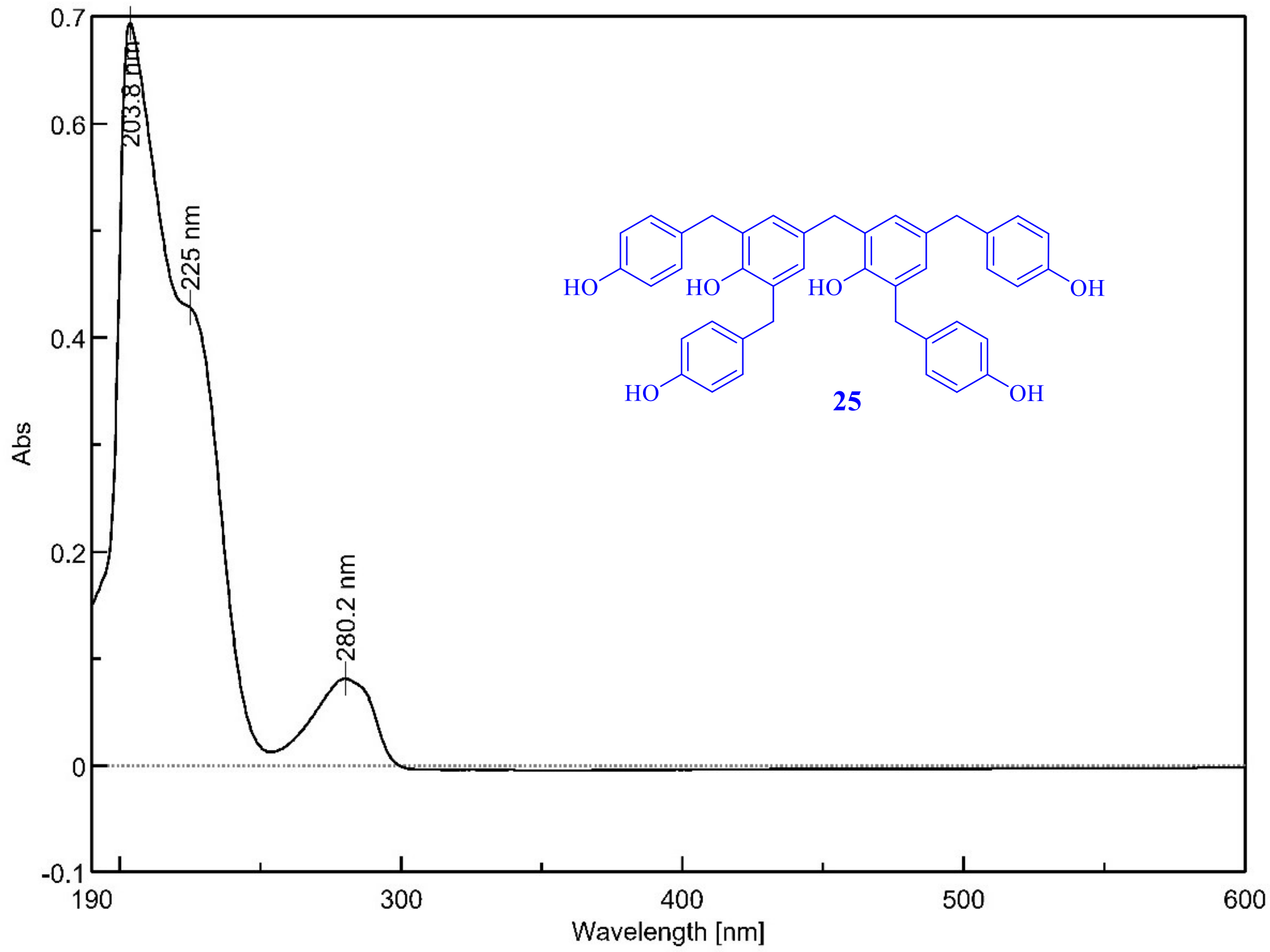

[Comment]			ZM-29
Sample Name	DBT		
Comment	0.02		
User			
Division	UV		
Company	324		
[Measurement Information]			
Instrument Name	V-650		
Model Name	V-650		
Serial No.	A034461150		
		[Data Information]	
Accessory	PSC-718	Creation Date	2020-11-11 16:49
Accessory S/N	A001761114		
Position	1	Data array type	Linear data array
Cell Length	10 mm	Horizontal	Wavelength [nm]
Temperature	20.00 C	Vertical	Abs
Control Sonsor	Holder	Start	600 nm
Monitor Sensor	Holder	End	190 nm
Start Mode	Start immediately	Data pitch	0.2 nm
		Data points	2051
Photometric Mode	Abs		
Measurement range	600 - 190 nm		
Data pitch	0.2 nm		
Band width(UV/Vis)	2.0 nm		
Response	Medium		
Scanning speed	200 nm/min		
Source Change	340 nm		
Light Source	D2/WI		
Filter Exchange	Step		
Correction	Baseline		

Figure S278. The UV spectrum of compound **25** in MeOH

Thermo Qexactive Focus Report

compound NO. : ZM-29
Method : LCMS(compound)-low

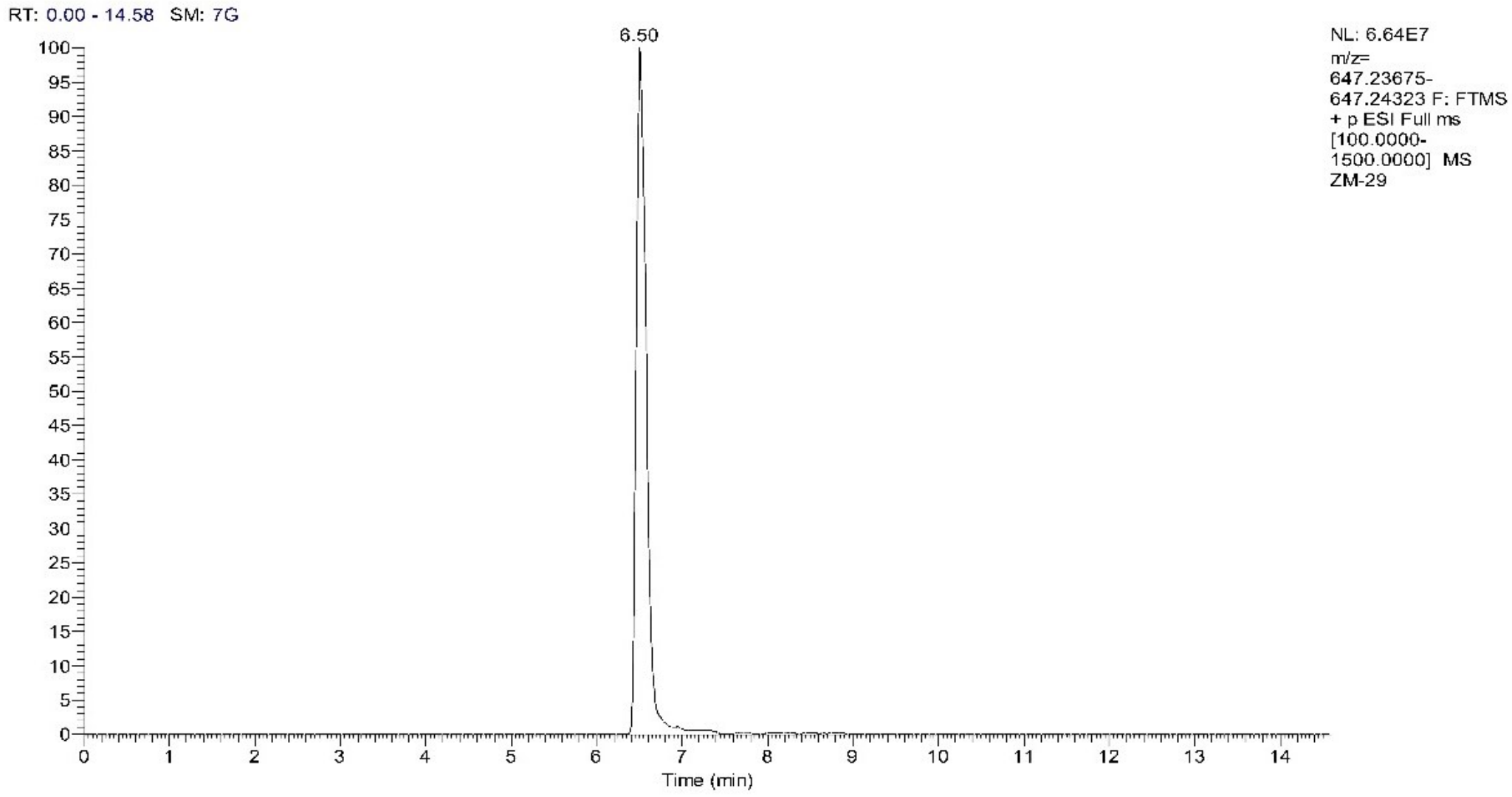

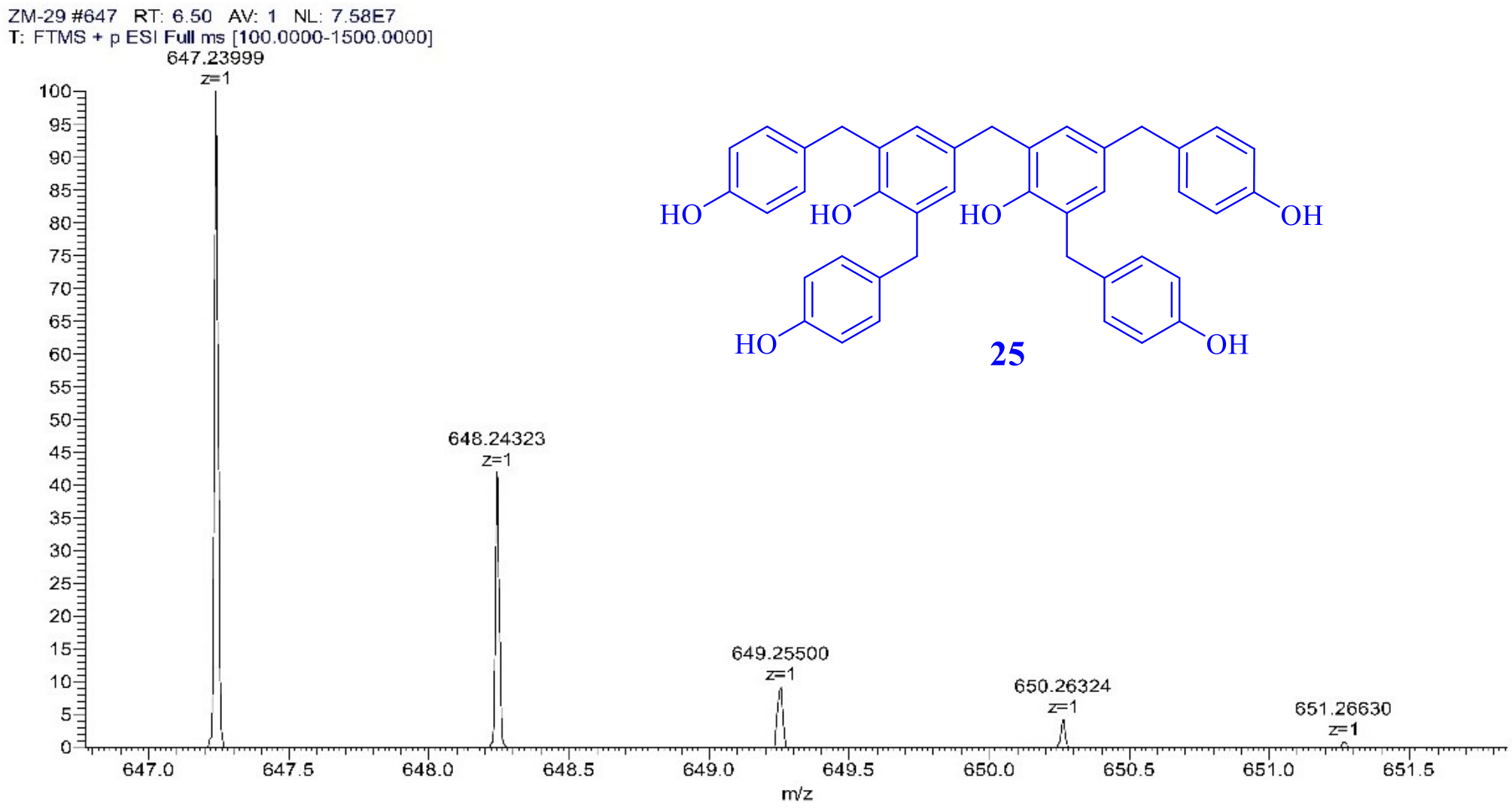

m/z	Theo. Mass	Delta (ppm)	RDB equiv.	Composition	
647.23999	647.24041	-0.65	23.5	C41 H36 O6 Na	M+Na

Figure S279. The HR-ESIMS report of compound **25**

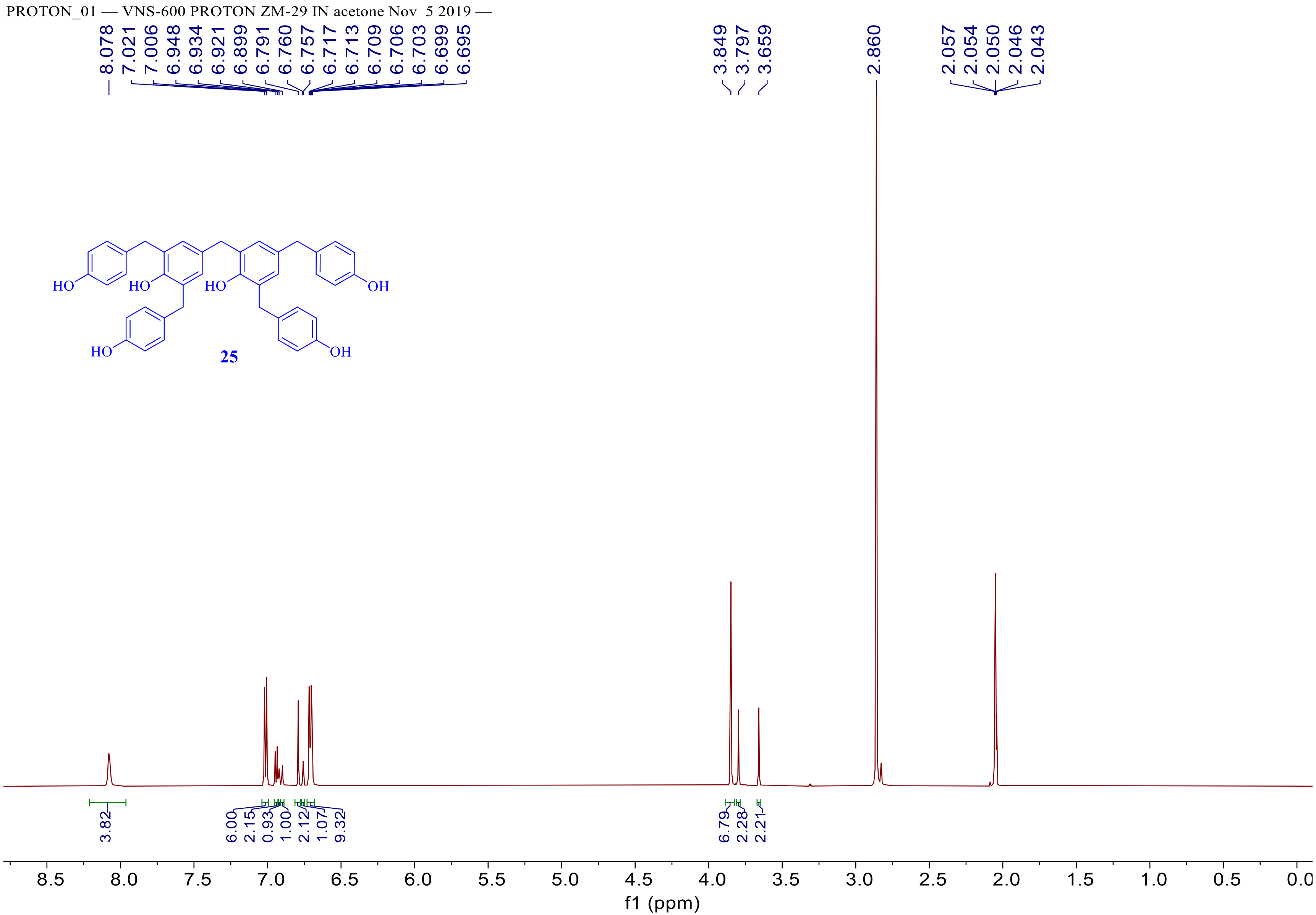

Figure S280. The ^{1}H NMR spectrum of compound **25** in acetone-d_6 (600 MHz)

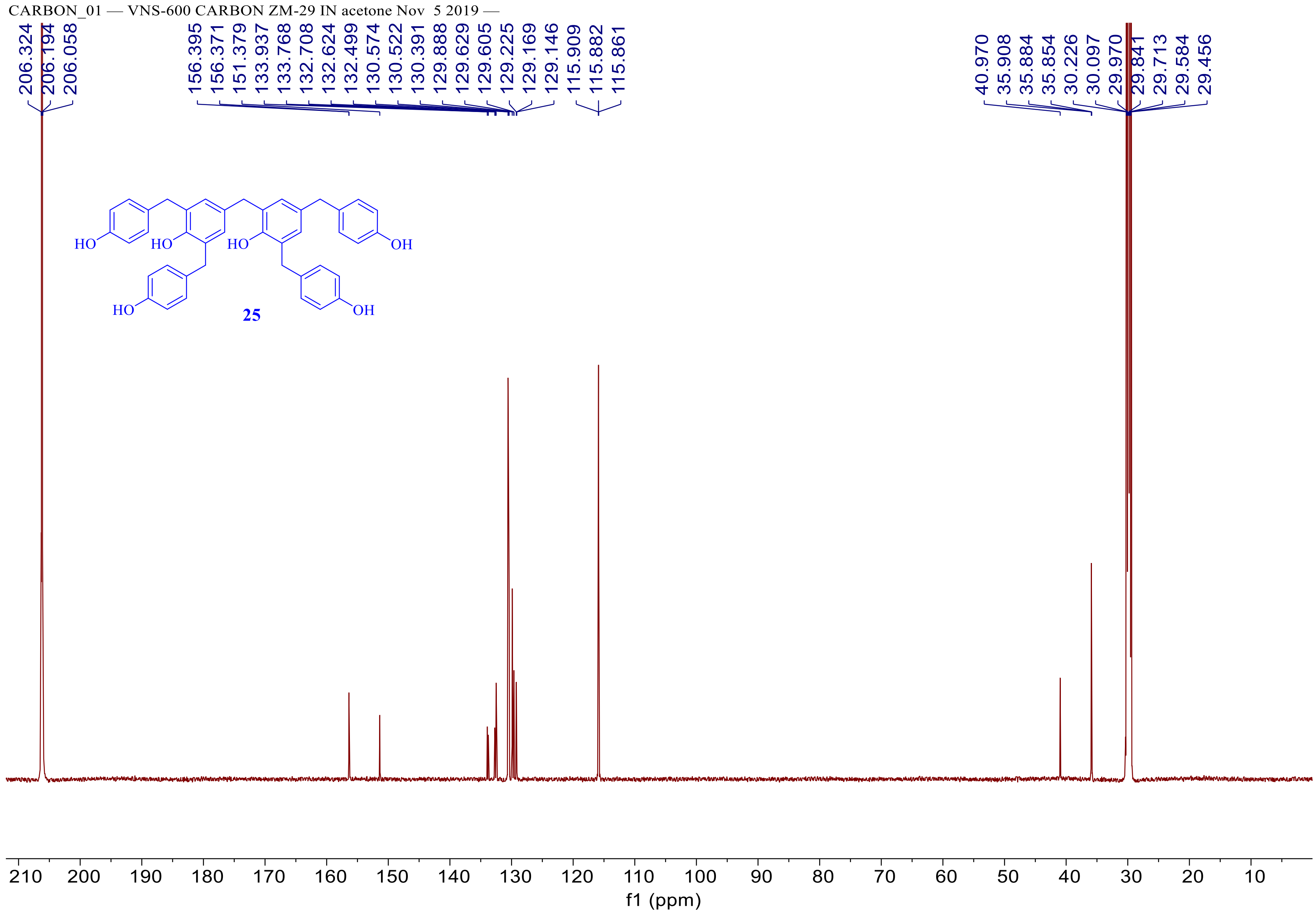

Figure S281. The ^{13}C NMR spectrum of compound **25** in acetone-d_6 (150 MHz)

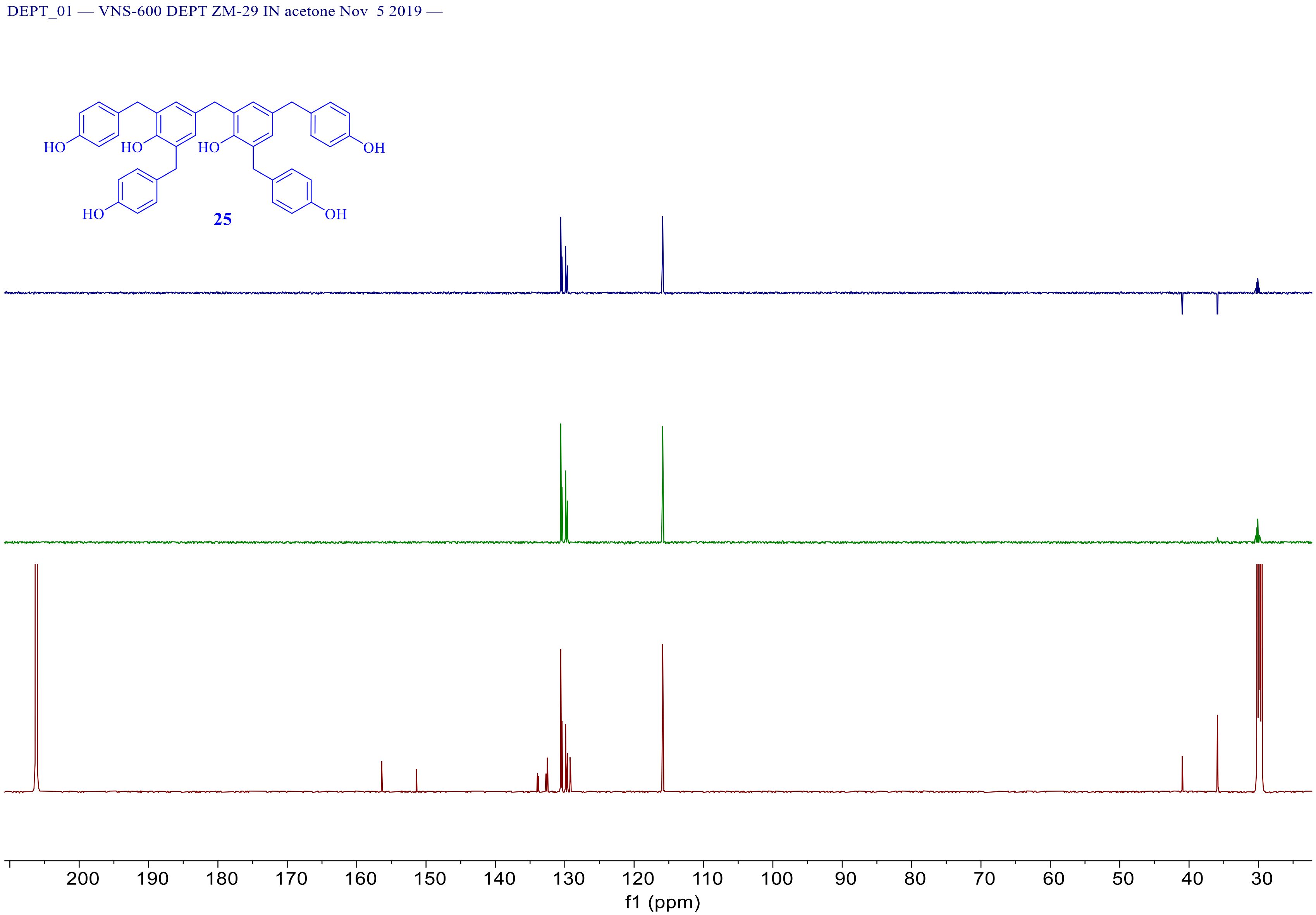

Figure S282. The DEPT spectrum of compound **25** in acetone-d_6 (150 MHz)

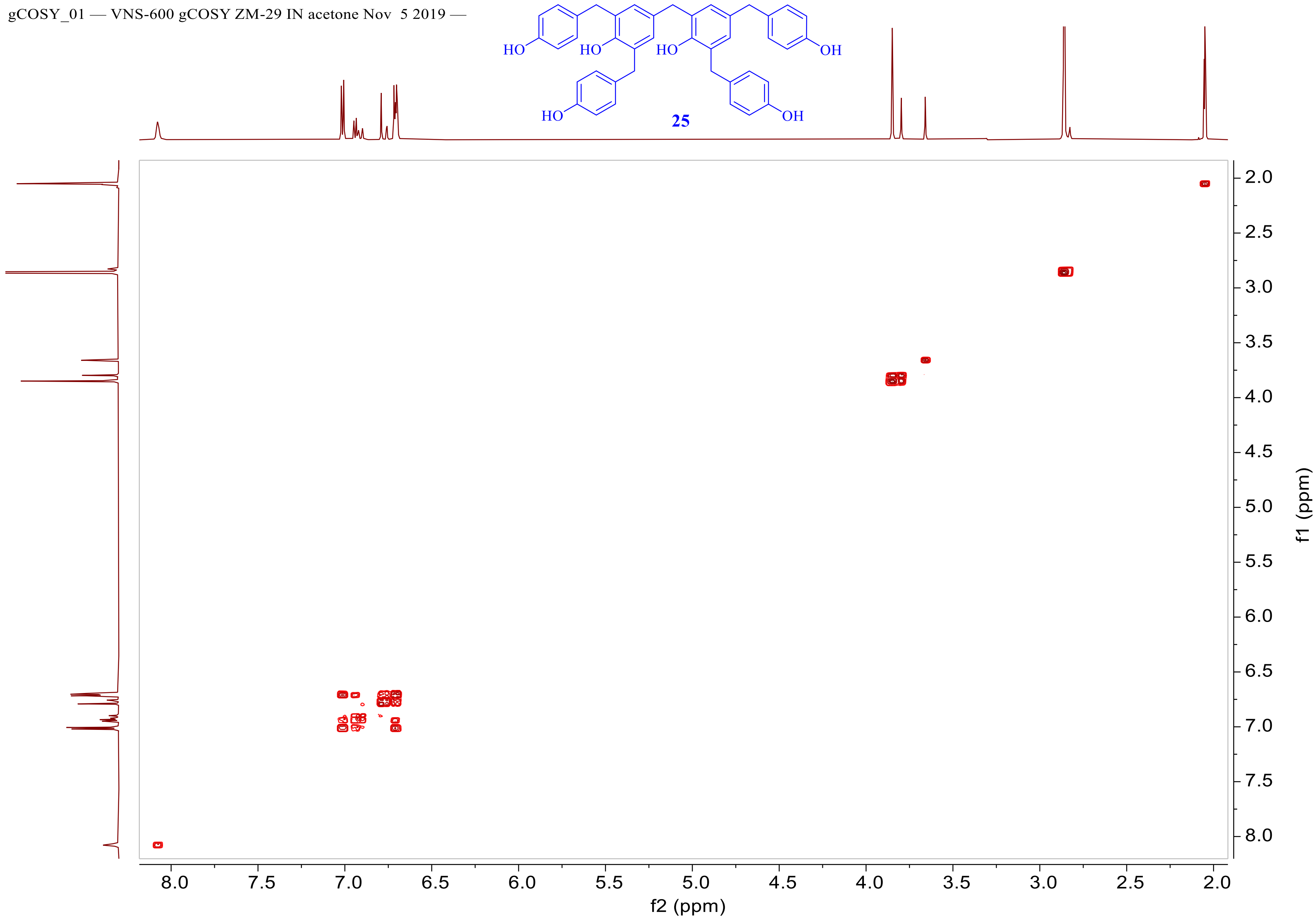

Figure S283. The ^{1}H-^{1}H COSY spectrum of compound **25** in acetone-d_6 (600 MHz)

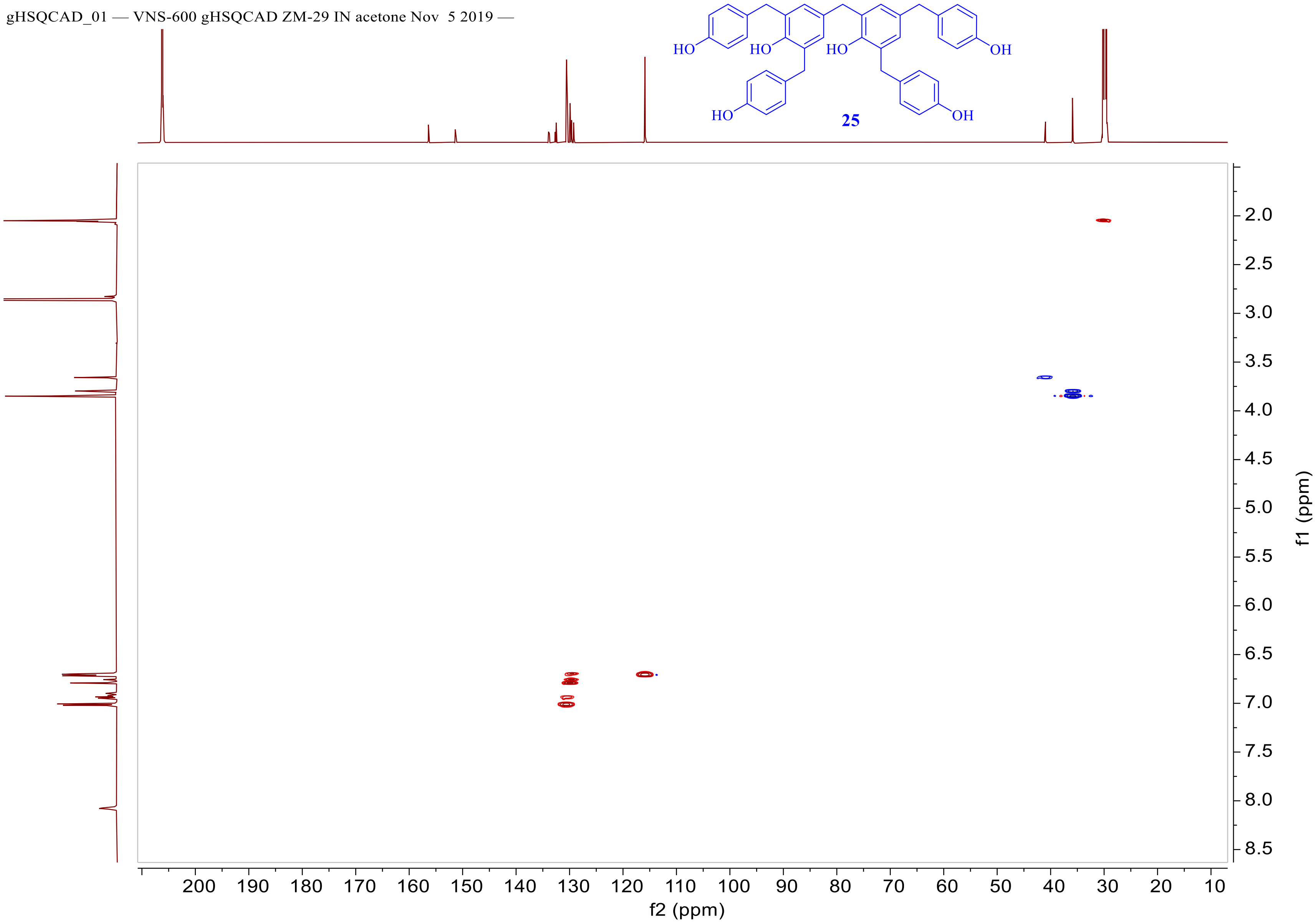

Figure S284. The HSQC spectrum of compound **25** in acetone-d_6 (600 MHz for ^{1}H)

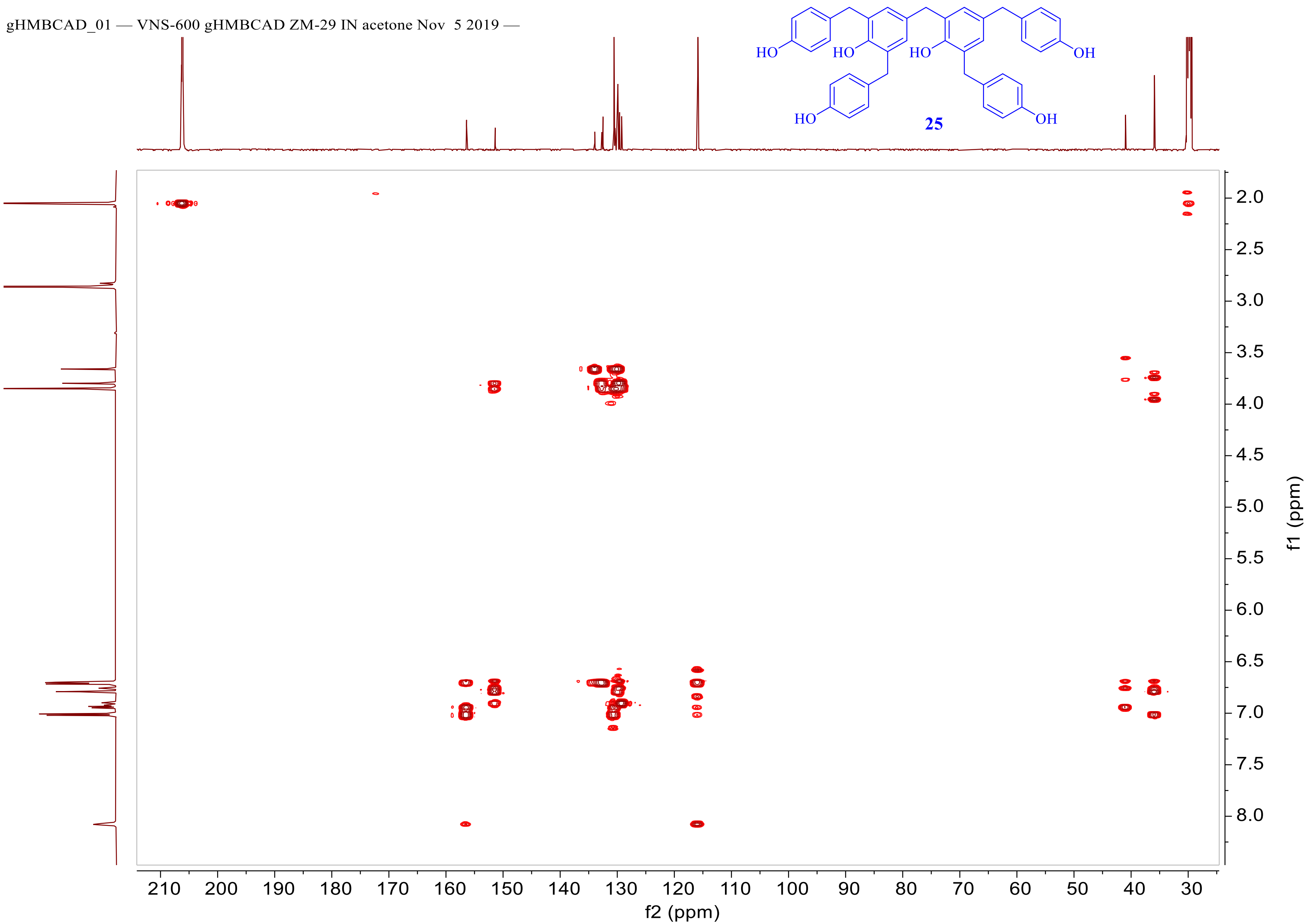

Figure S285. The HMBC spectrum of compound **25** in acetone-d_6 (600 MHz for ^{1}H)

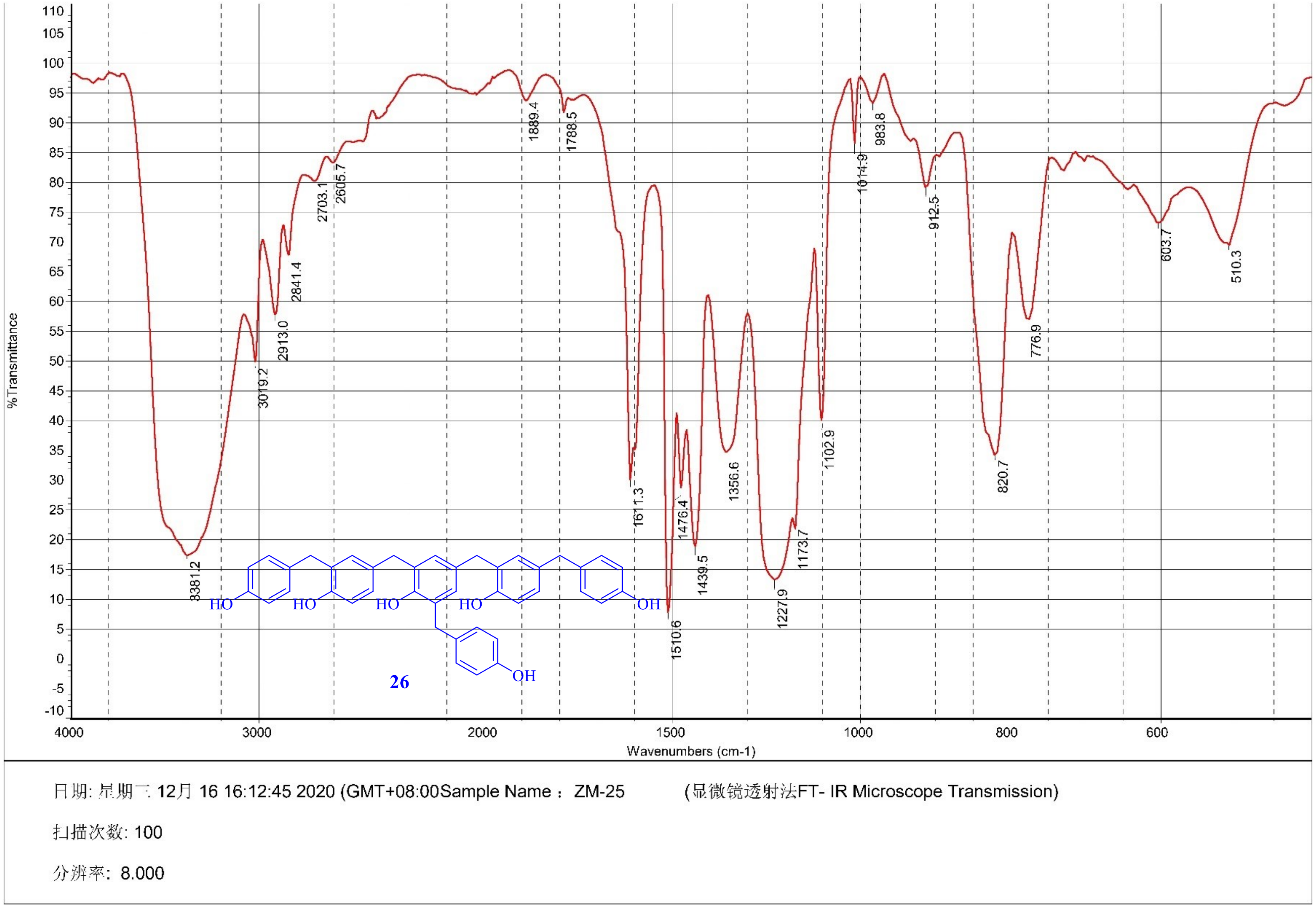

Figure S286. The IR spectrum of compound **26**

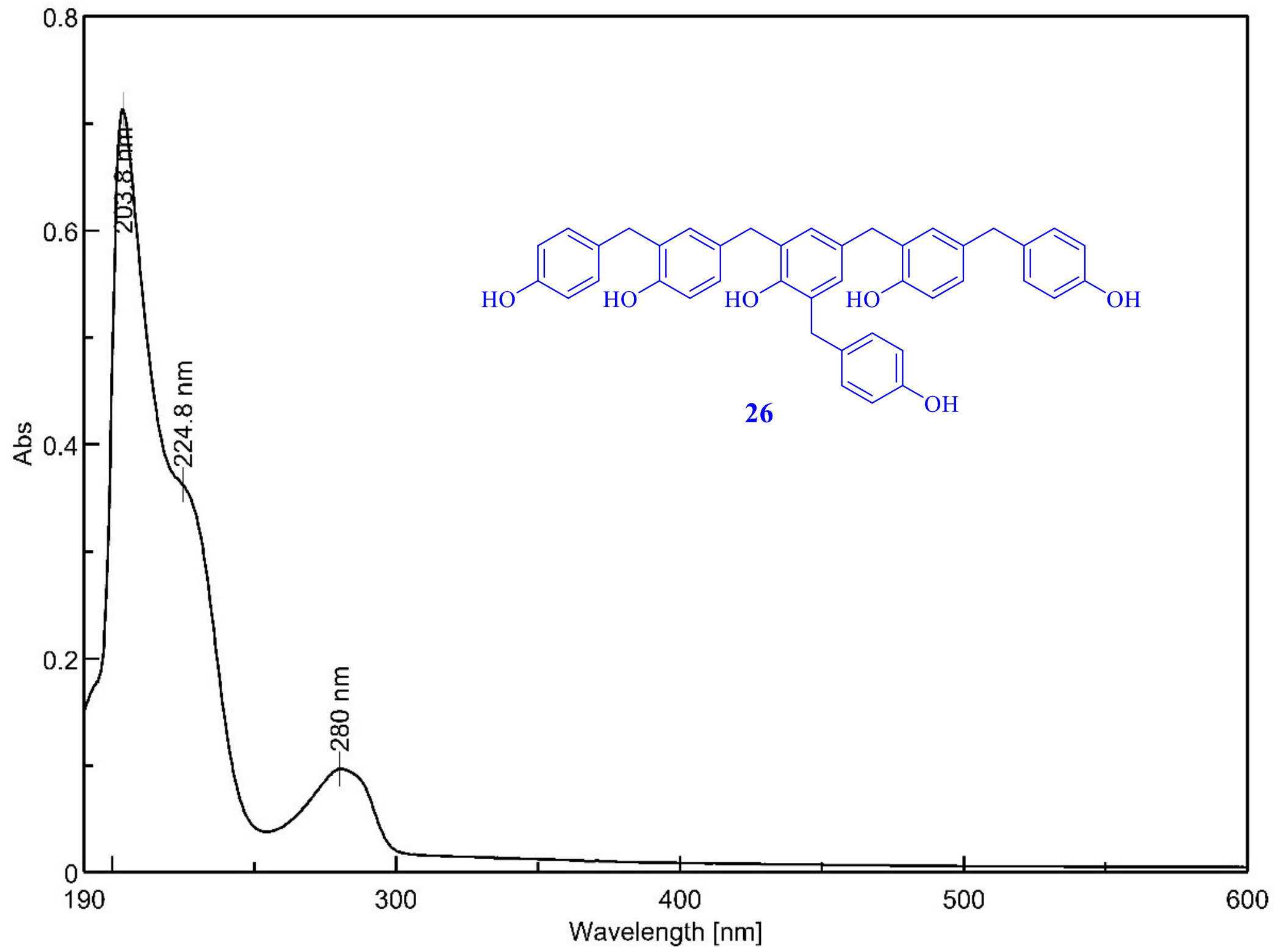

zm-25

[Comment]
Sample Name DBT
Comment 0.02
User
Division UV
Company 324

[Measurement Information]
Instrument Name V-650
Model Name V-650
Serial No. A034461150

Accessory PSC-718
Accessory S/N A001761114
Position 1
Cell Length 10 mm
Temperature 19.96 C
Control Sonsor Holder
Monitor Sensor Holder
Start Mode Start immediately

Photometric Mode Abs
Measurement range 600 - 190 nm
Data pitch 0.2 nm
Band width(UV/Vis) 2.0 nm
Response Medium
Scanning speed 200 nm/min
Source Change 340 nm
Light Source D2/WI
Filter Exchange Step
Correction Baseline

[Data Information]
Creation Date 2020-11-11 16:33

Data array type Linear data array
Horizontal Wavelength [nm]
Vertical Abs
Start 600 nm
End 190 nm
Data pitch 0.2 nm
Data points 2051

Figure S287. The UV spectrum of compound **26** in MeOH

Thermo Qexactive Focus Report

compound NO. : ZM-25
Method : LCMS(compound)-low

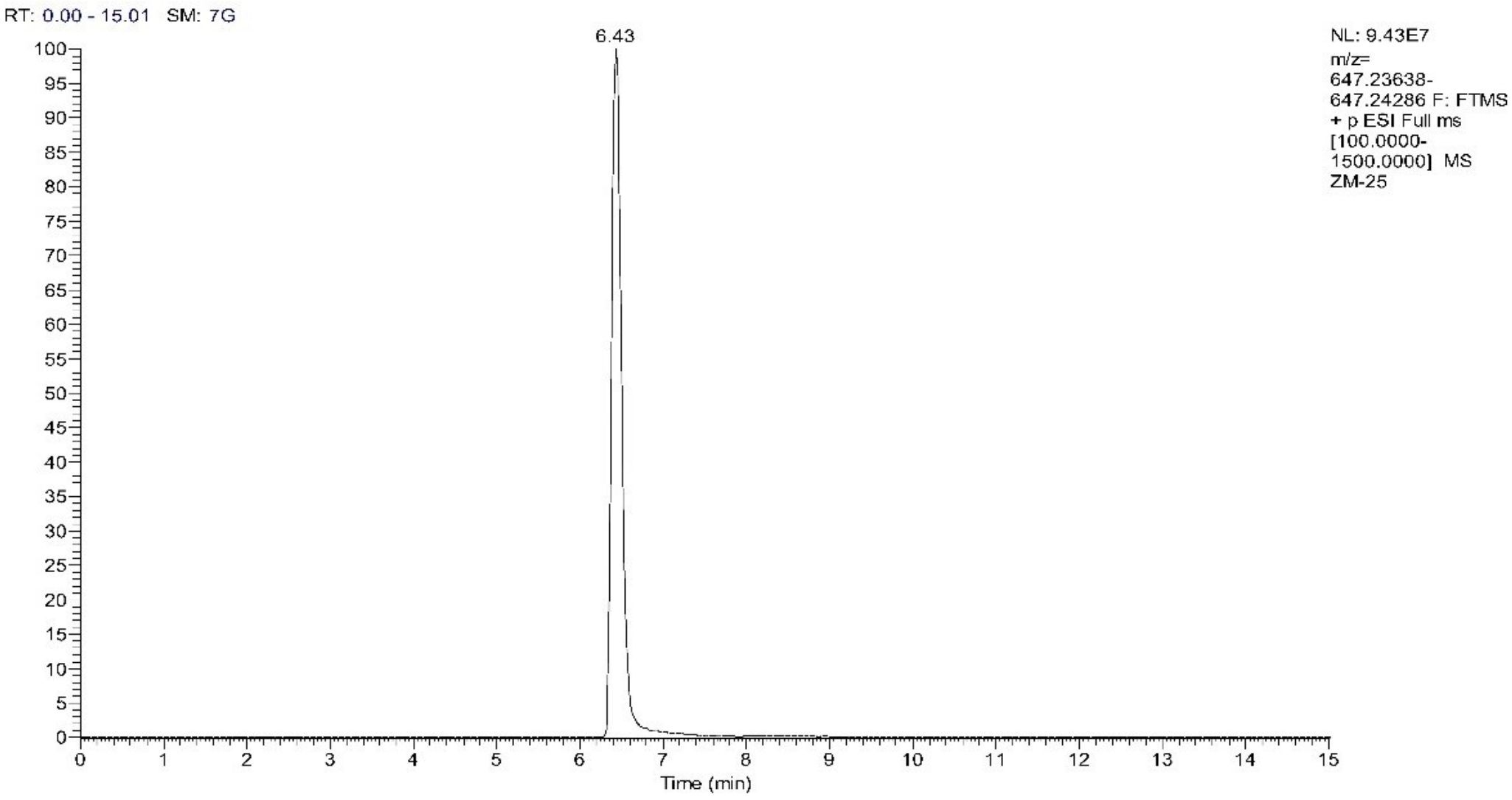

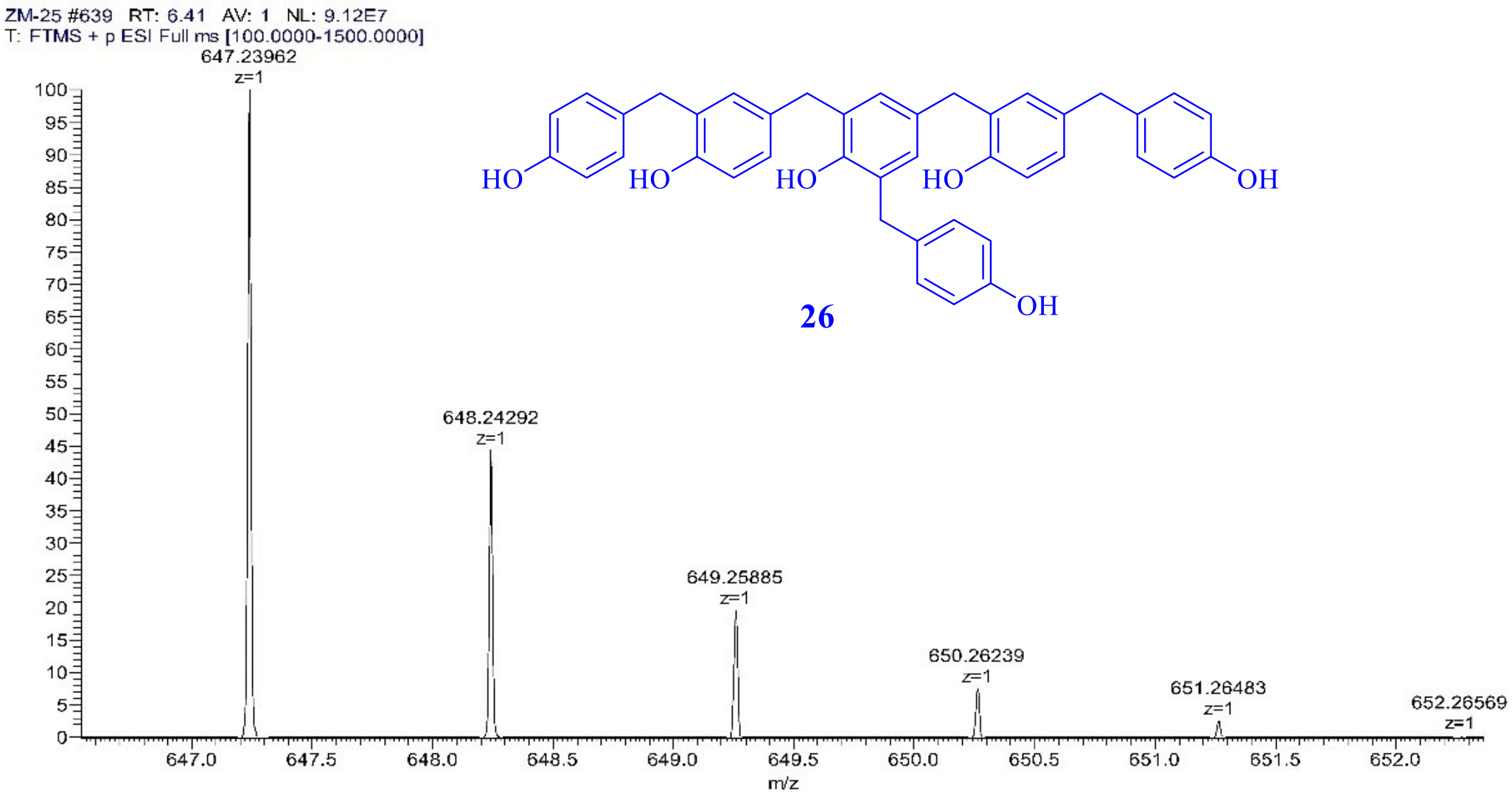

m/z	Theo. Mass	Delta (ppm)	RDB equiv.	Composition	
647.23962	647.24041	-1.22	23.5	C41 H36 O6 Na	M+Na

Figure S288. The HR-ESIMS report of compound **26**

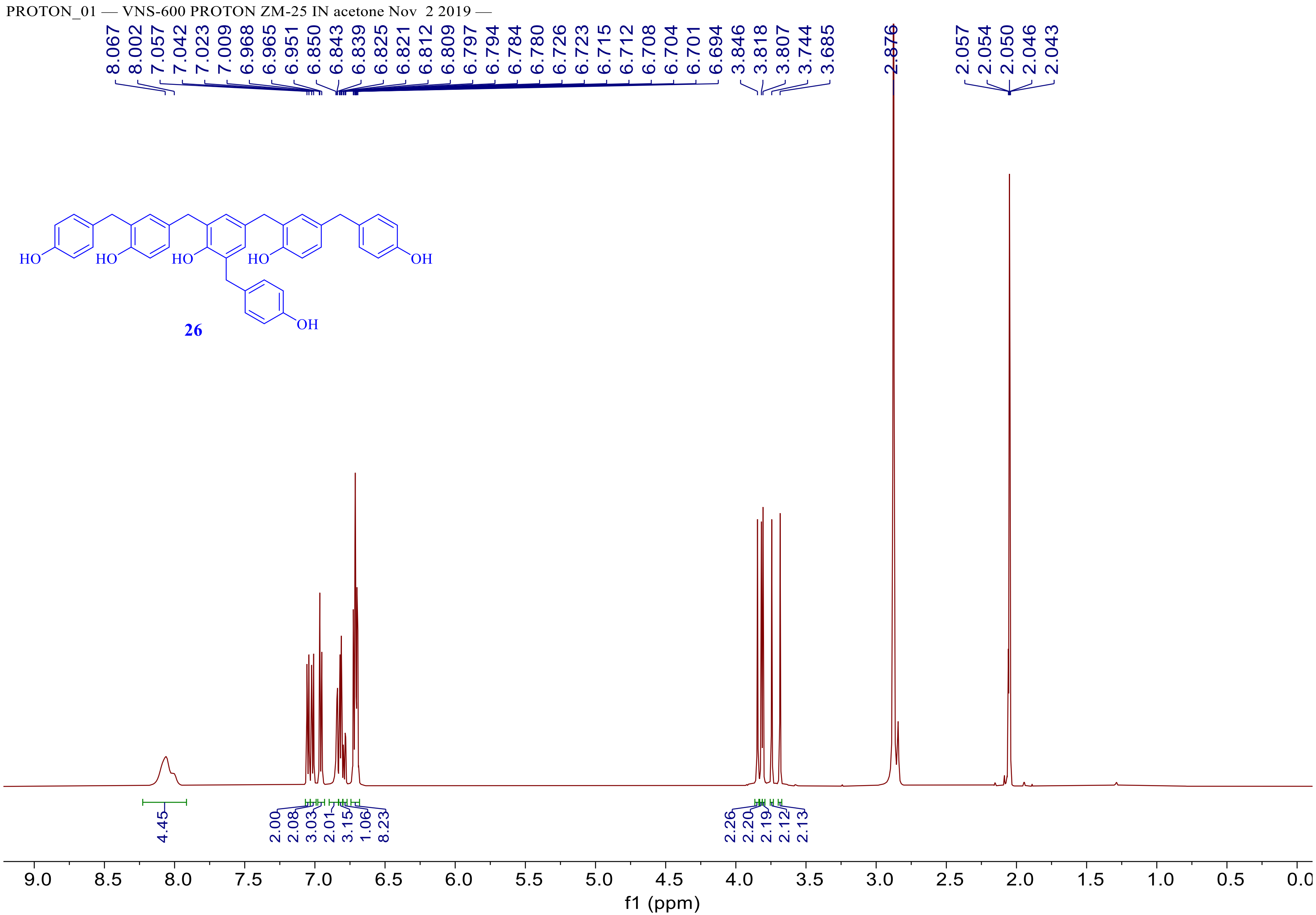

Figure S289. The ^{1}H NMR spectrum of compound **26** in acetone-d_6 (600 MHz)

CARBON_01 — VNS-600 CARBON ZM-25 IN acetone Nov 2 2019 —

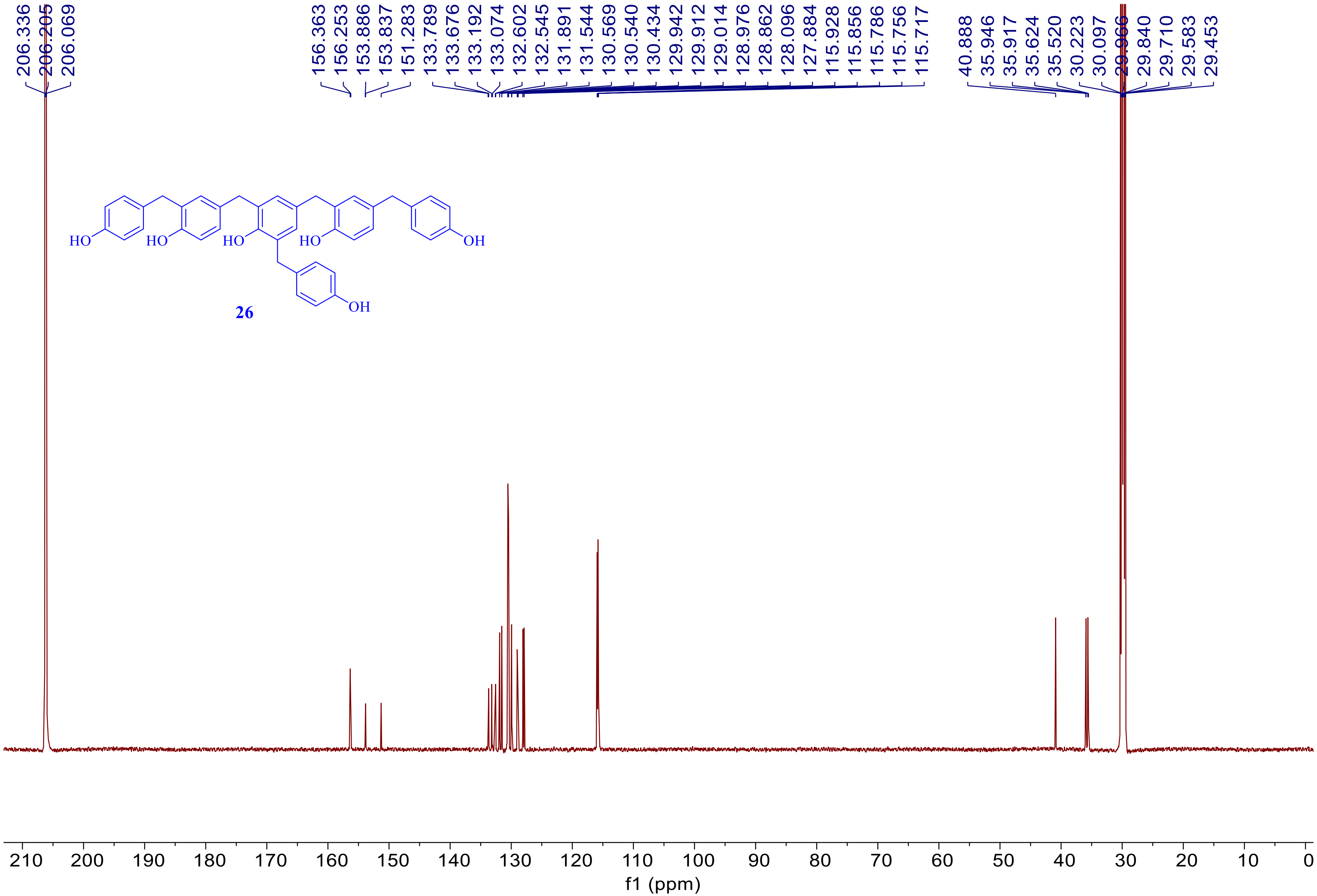

Figure S290. The ^{13}C NMR spectrum of compound **26** in acetone-d_6 (150 MHz)

DEPT_01 — VNS-600 DEPT ZM-25 IN acetone Nov 2 2019 —

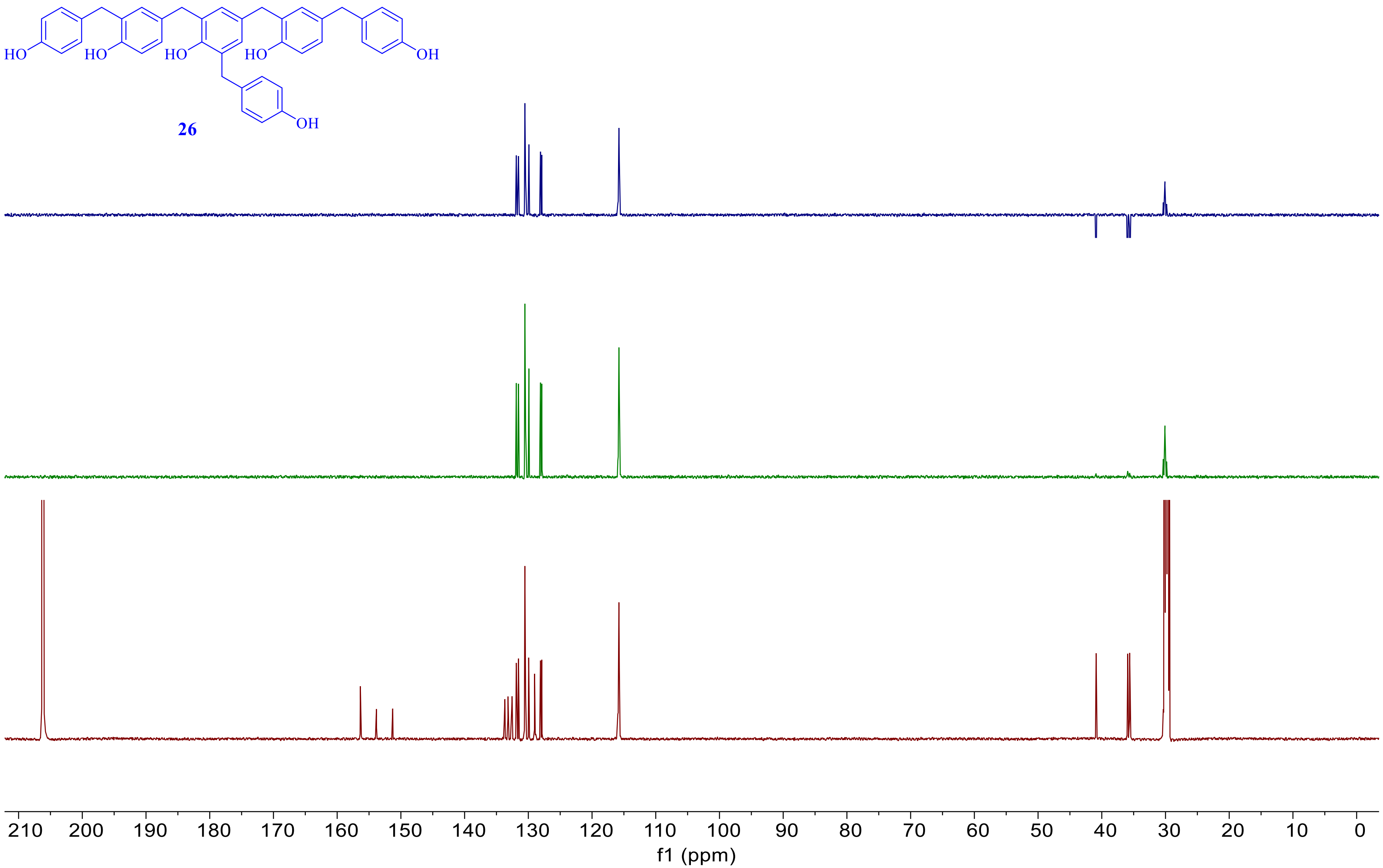

Figure S291. The DEPT spectrum of compound **26** in acetone-d_6 (150 MHz)

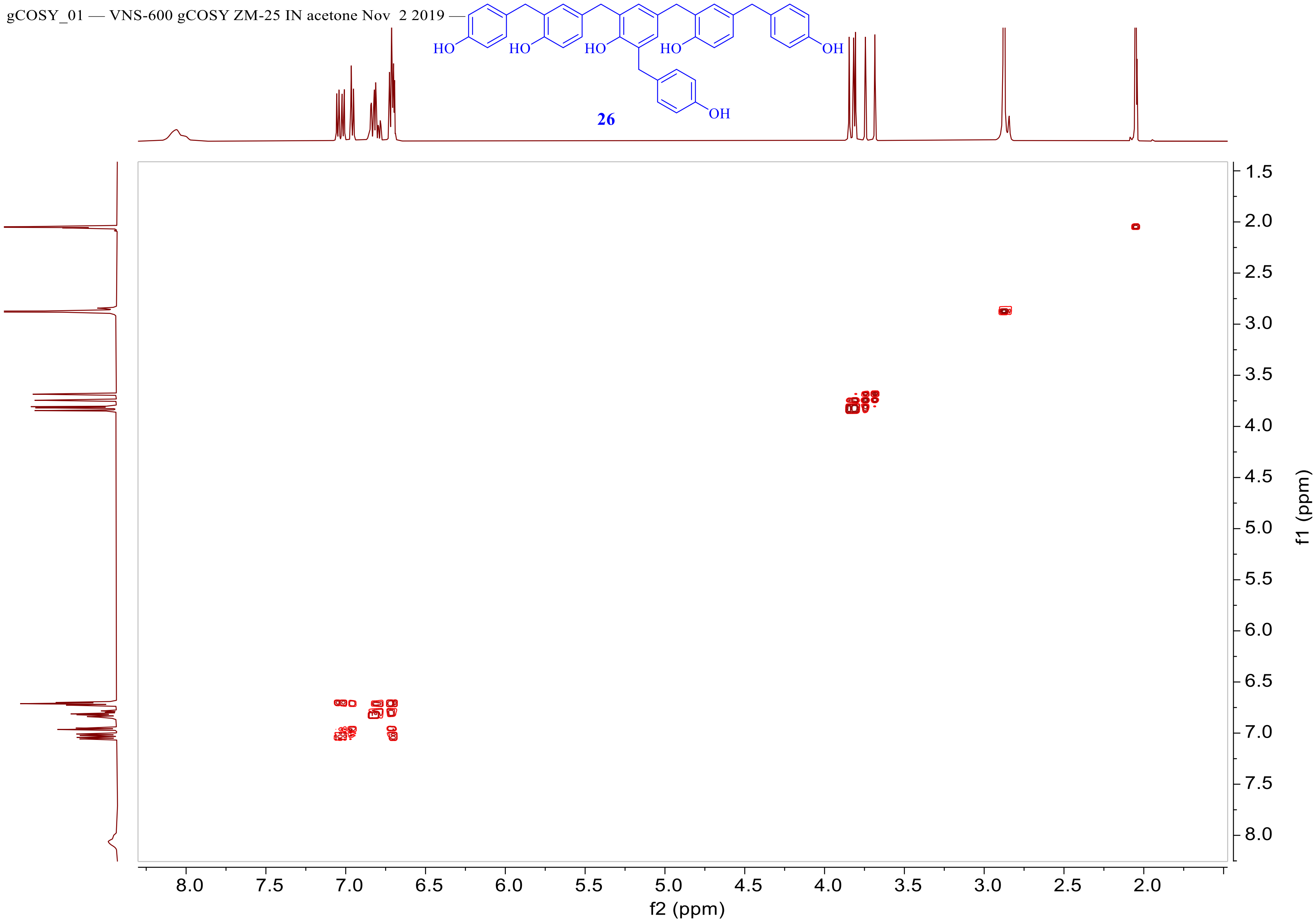

Figure S292. The ^{1}H-^{1}H COSY spectrum of compound **26** in acetone-d_6 (600 MHz)

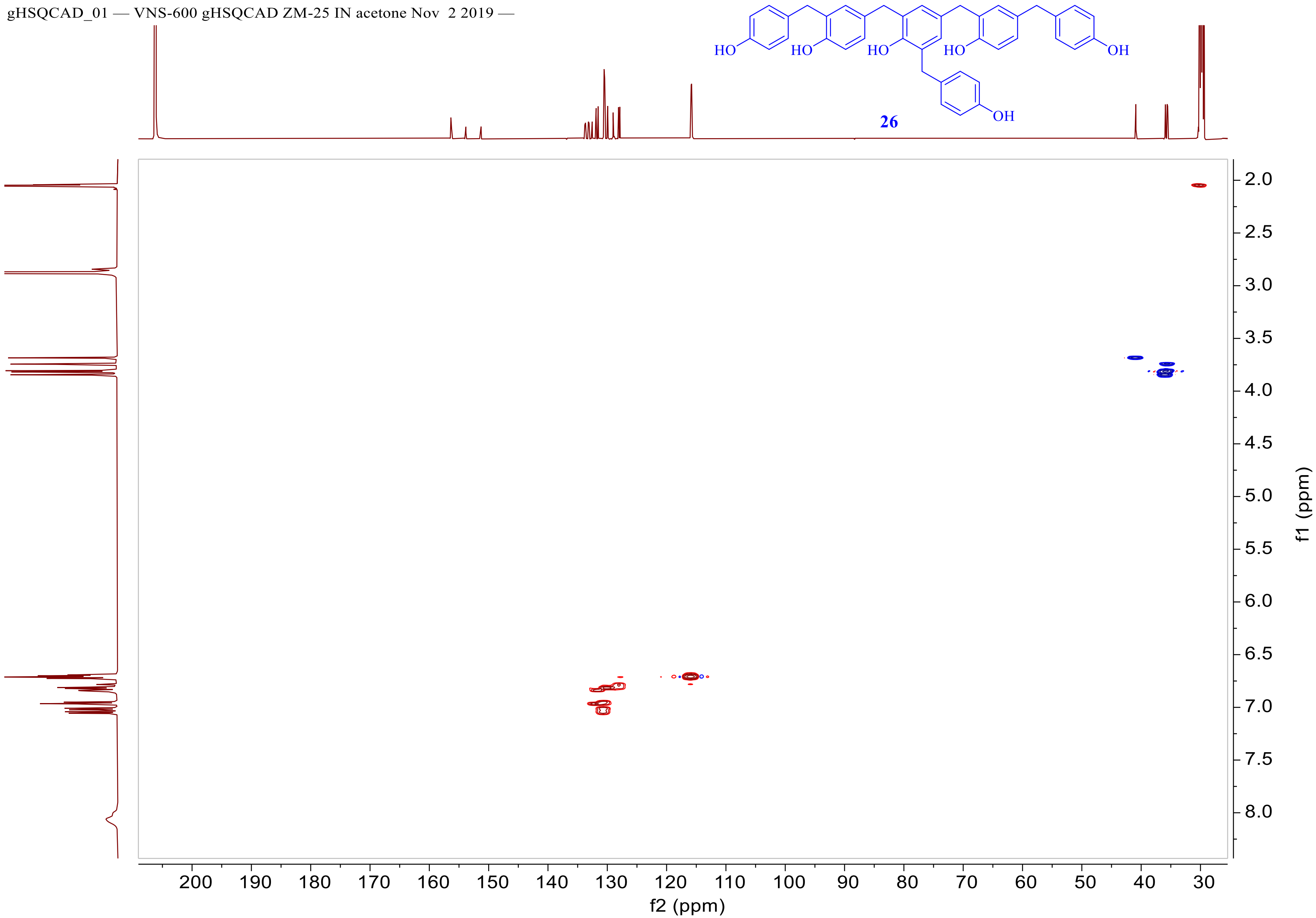

Figure S293. The HSQC spectrum of compound **26** in acetone-d_6 (600 MHz for ^{1}H)

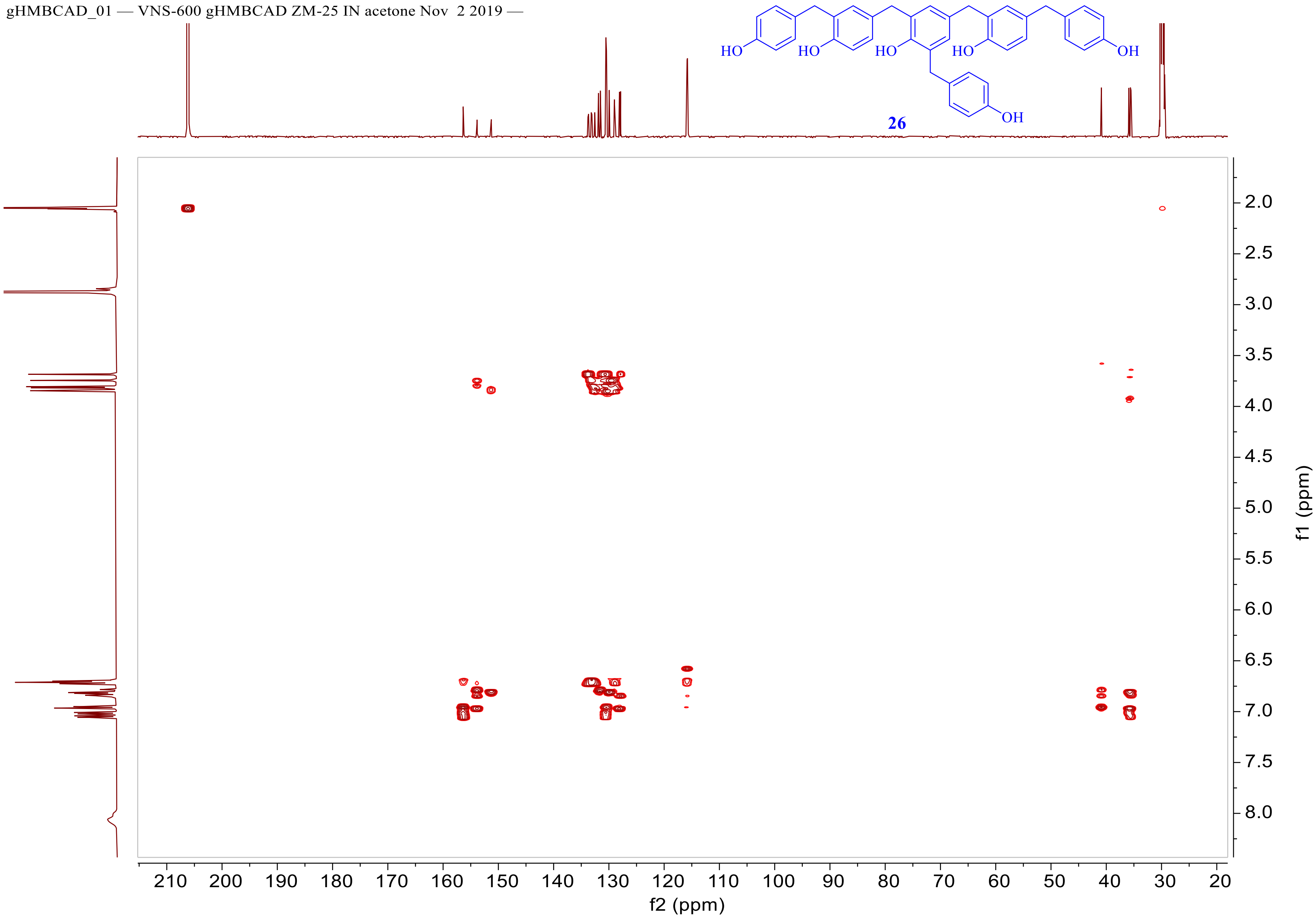

Figure S294. The HMBC spectrum of compound **26** in acetone-d_6 (600 MHz for ^{1}H)

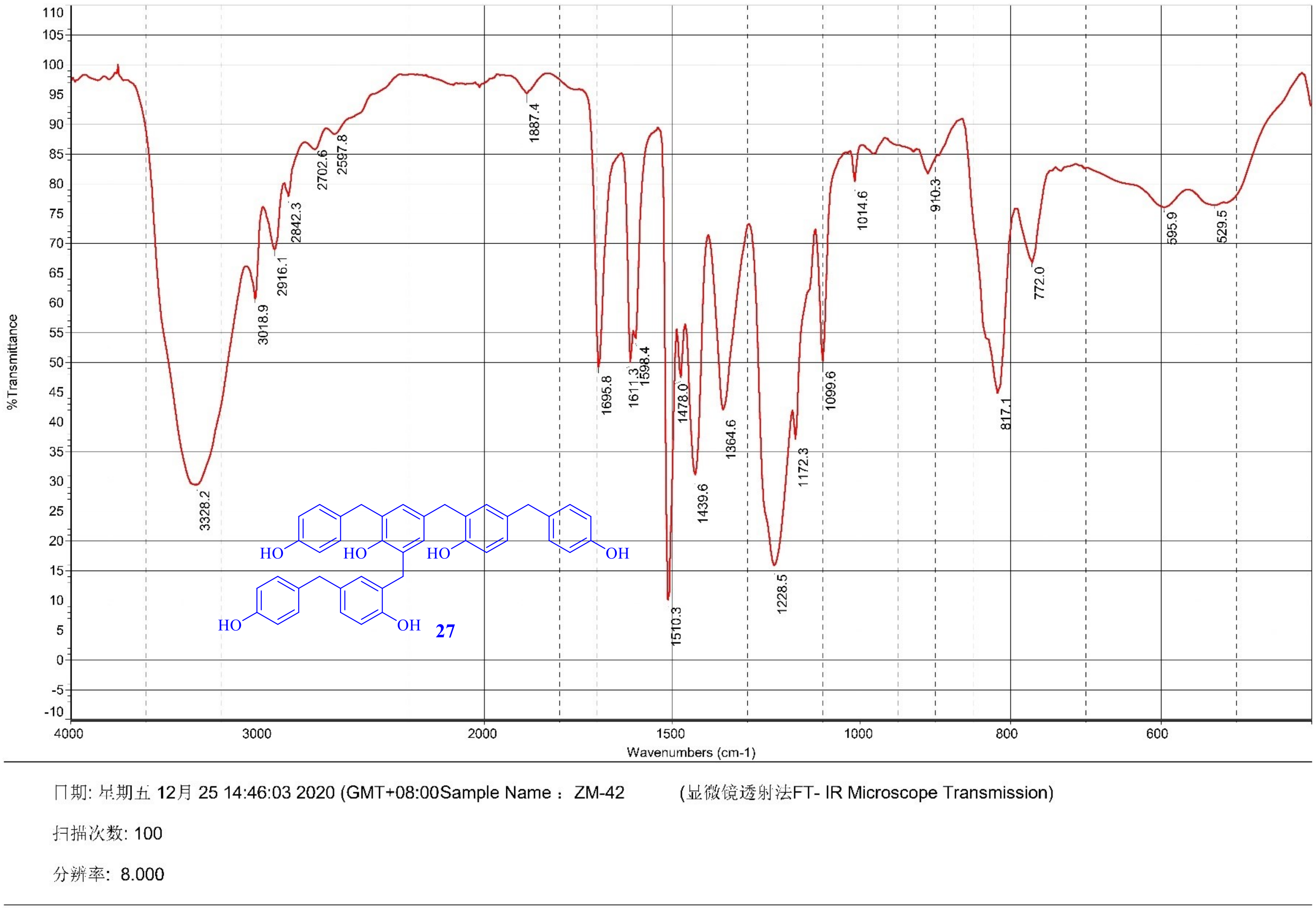

Figure S295. The IR spectrum of compound **27**

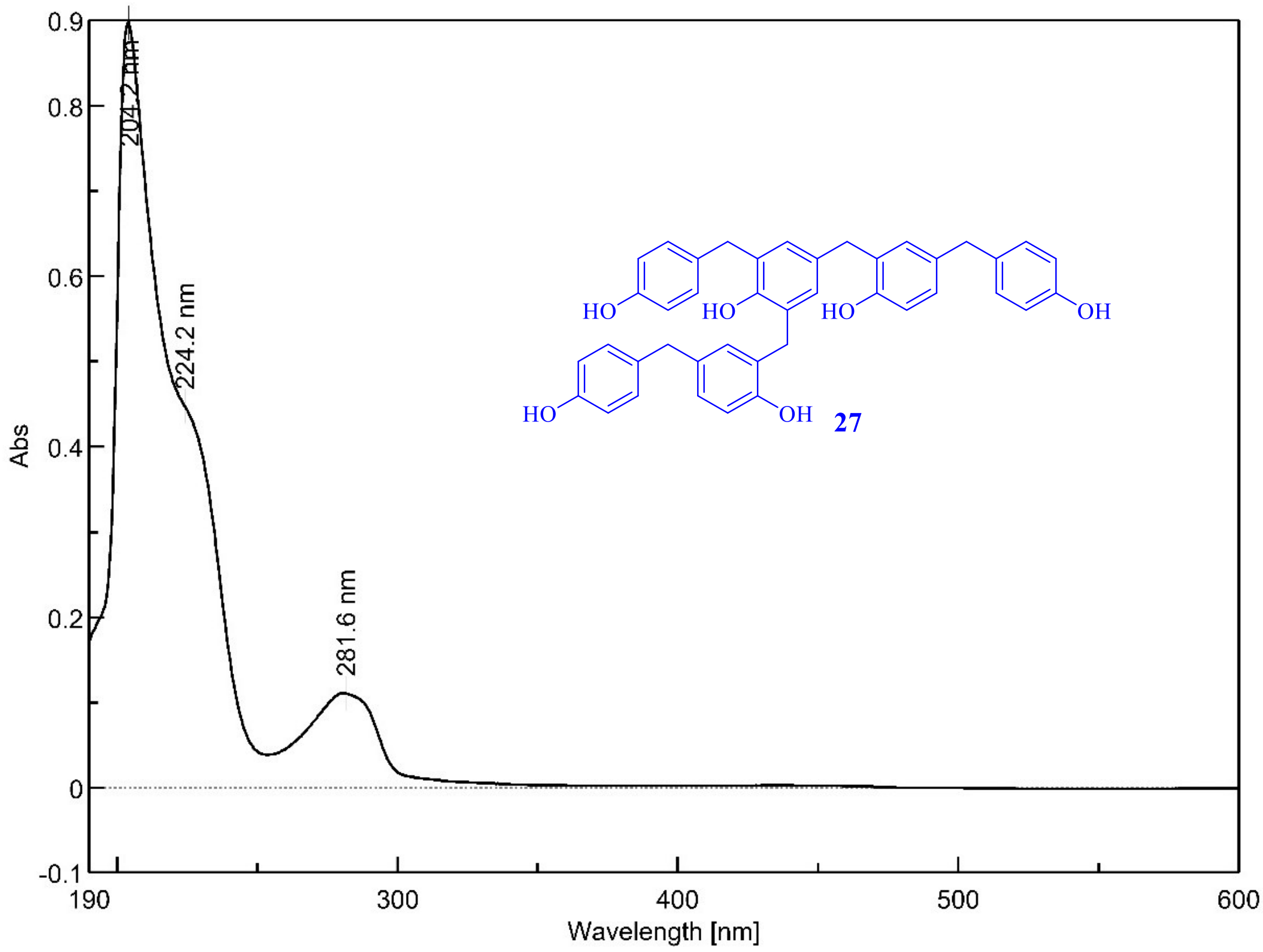

ZM-42

[Comment]
Sample Name DBT
Comment 0.02
User
Division UV
Company 324

[Measurement Information]
Instrument Name V-650
Model Name V-650
Serial No. A034461150

Accessory PSC-718
Accessory S/N A001761114
Position 1
Cell Length 10 mm
Temperature 20.05 C
Control Sonsor Holder
Monitor Sensor Holder
Start Mode Start immediately

Photometric Mode Abs
Measurement range 600 - 190 nm
Data pitch 0.2 nm
Band width(UV/Vis) 2.0 nm
Response Medium
Scanning speed 200 nm/min
Source Change 340 nm
Light Source D2/WI
Filter Exchange Step
Correction Baseline

[Data Information]
Creation Date 2020-11-11 16:52

Data array type Linear data array
Horizontal Wavelength [nm]
Vertical Abs
Start 600 nm
End 190 nm
Data pitch 0.2 nm
Data points 2051

Figure S296. The UV spectrum of compound **27** in MeOH

Thermo Qexactive Focus Report

compound NO. : ZM-42
Method : LCMS(compound)-low

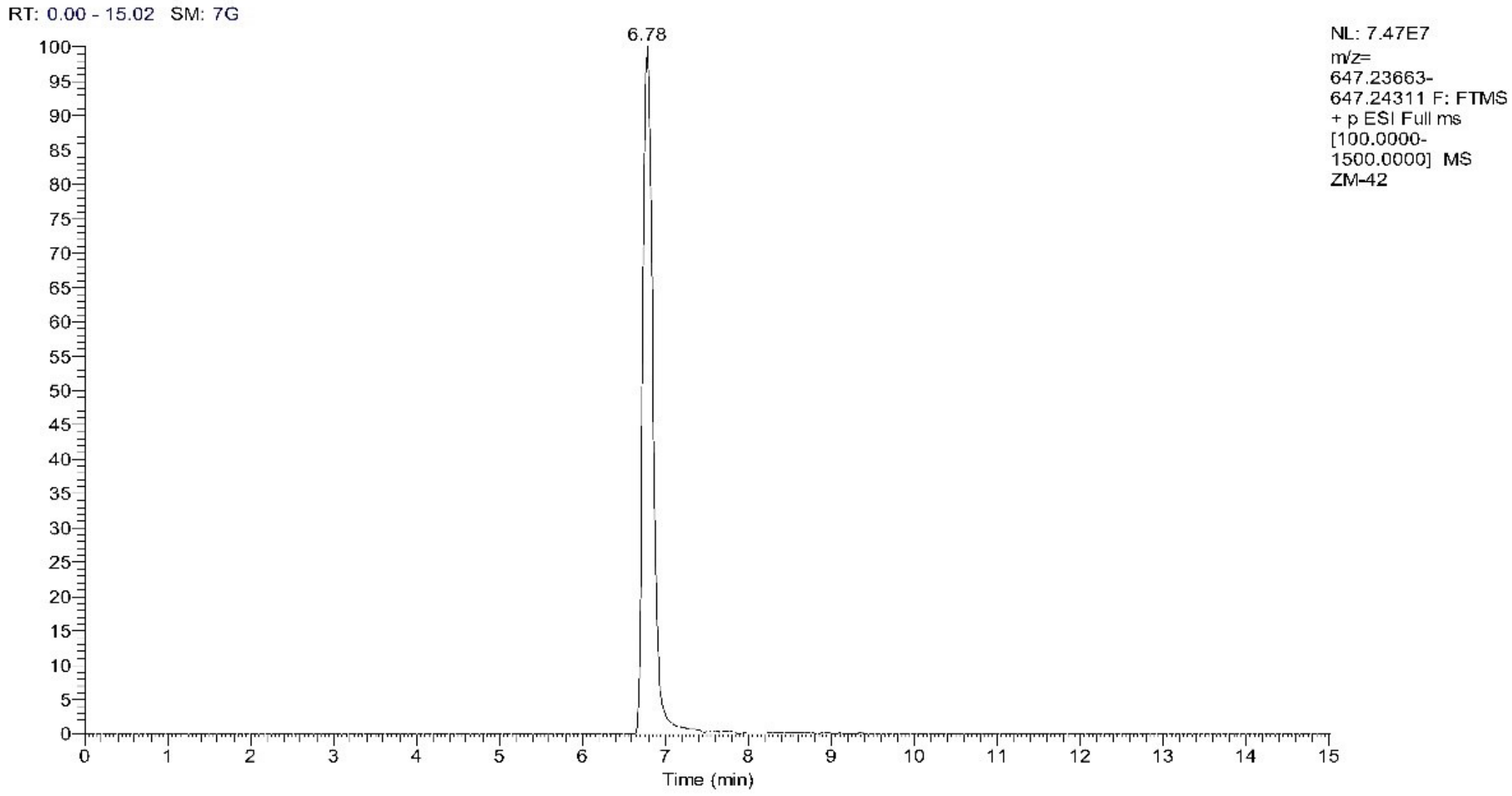

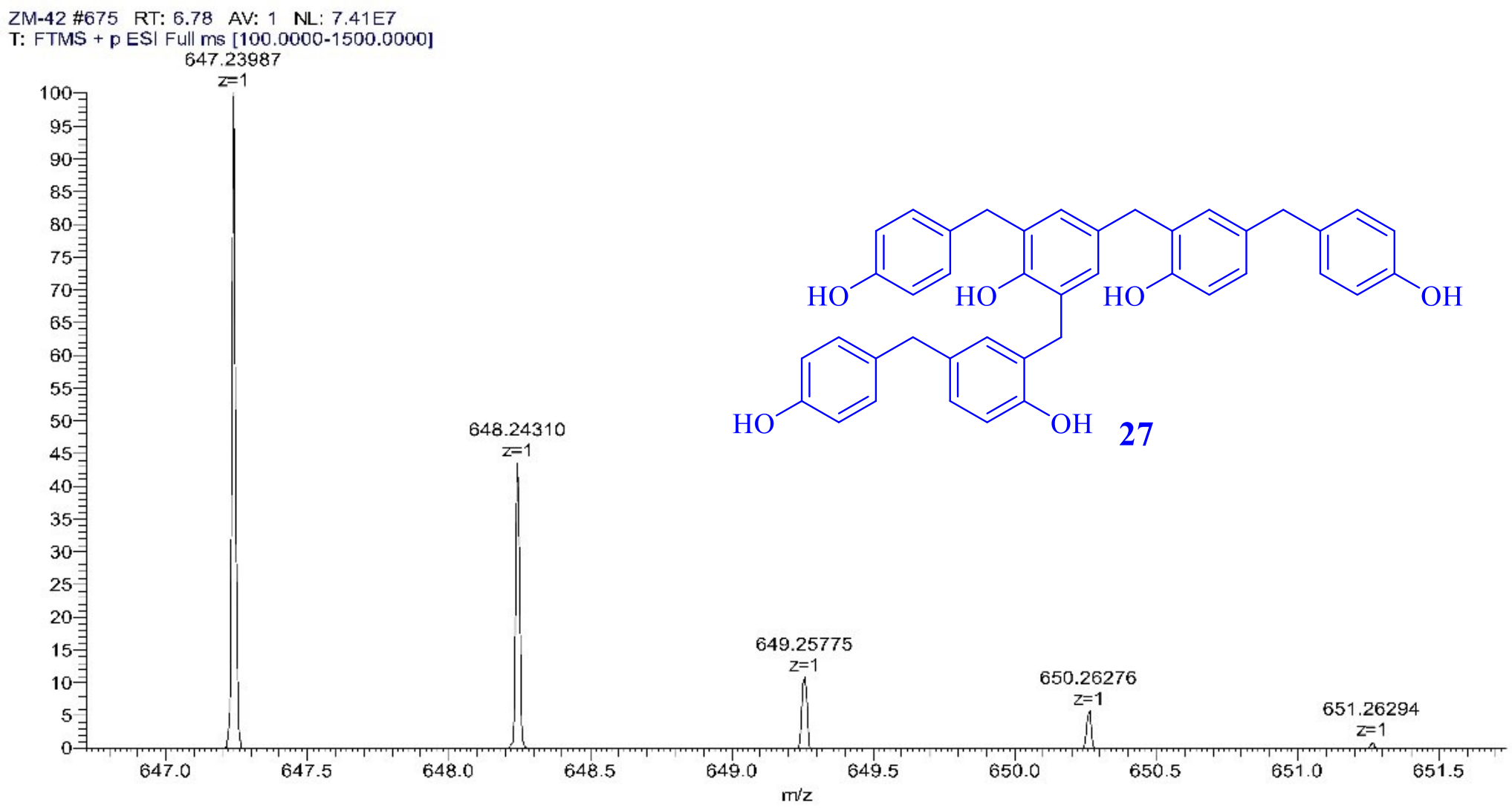

m/z	Theo. Mass	Delta (ppm)	RDB equiv.	Composition	
647.23987	647.24041	-0.83	23.5	C41 H36 O6 Na	M+Na

Figure S297. The HR-ESIMS report of compound **27**

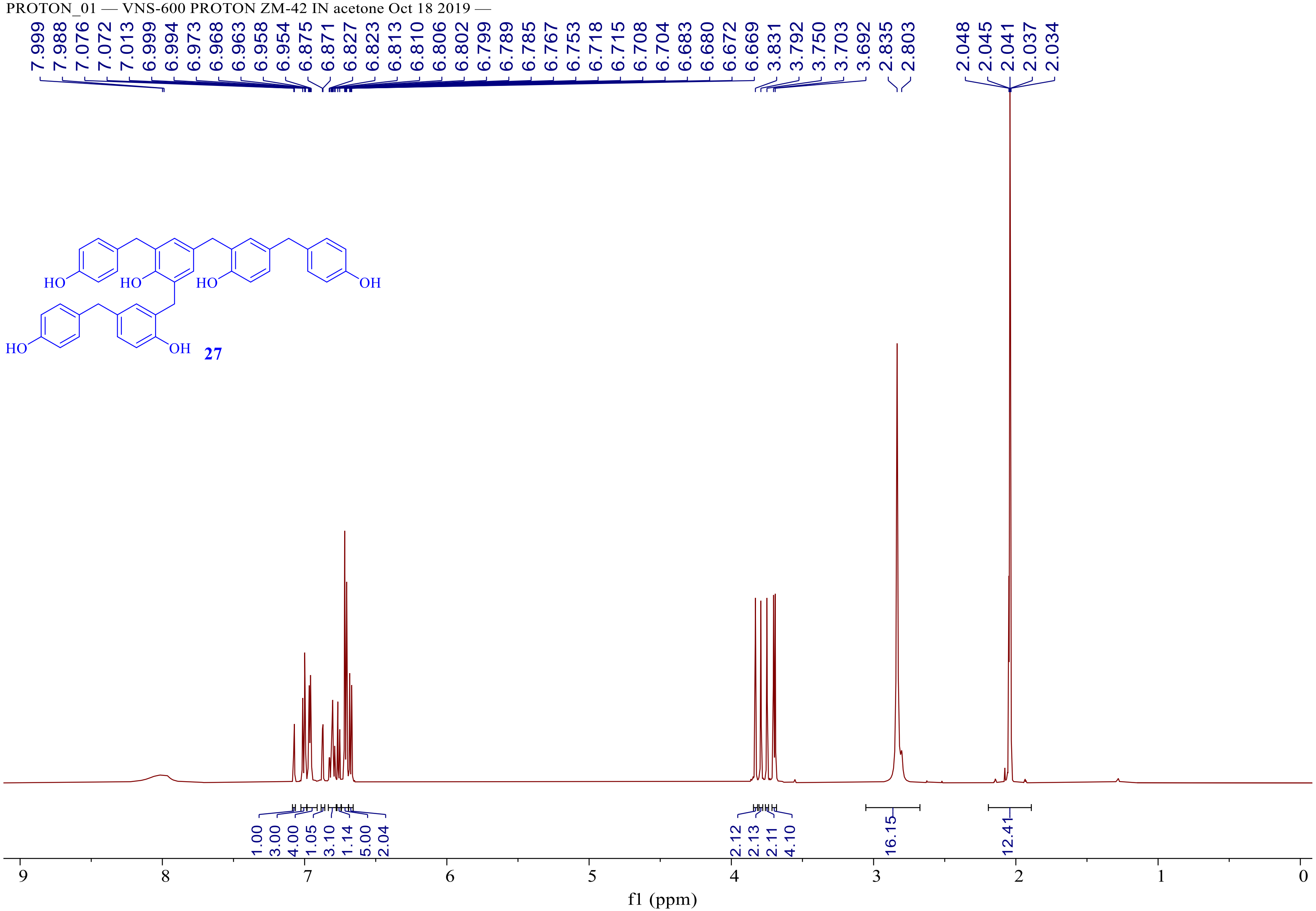

Figure S298. The ^{1}H NMR spectrum of compound **27** in acetone-d_6 (600 MHz)

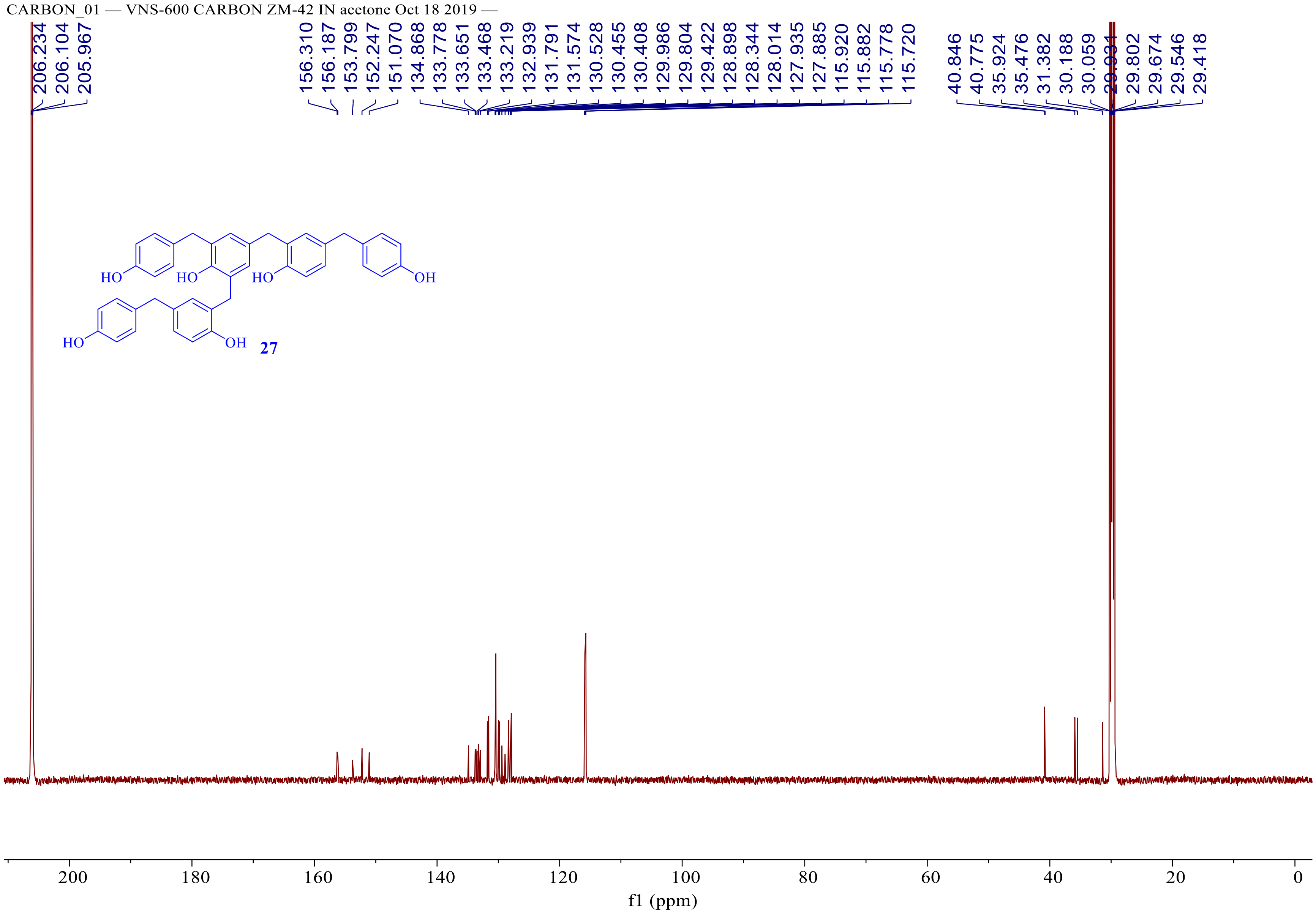

Figure S299. The ^{13}C NMR spectrum of compound **27** in acetone-d_6 (150 MHz)

DEPT_01 — VNS-600 DEPT ZM-42 IN acetone Oct 18 2019 —

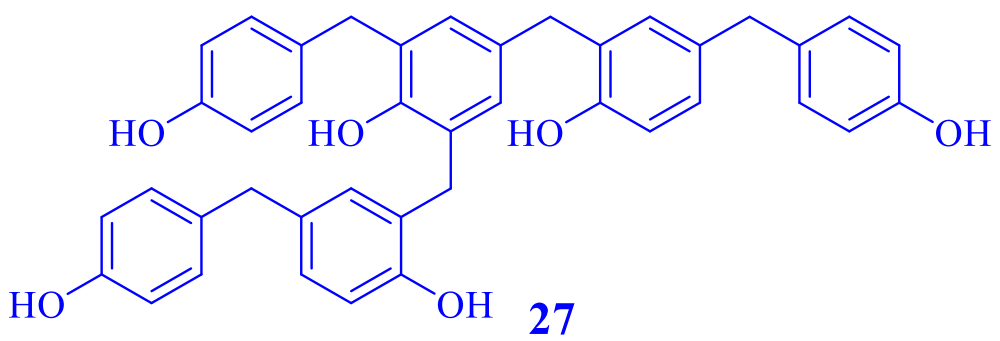

27

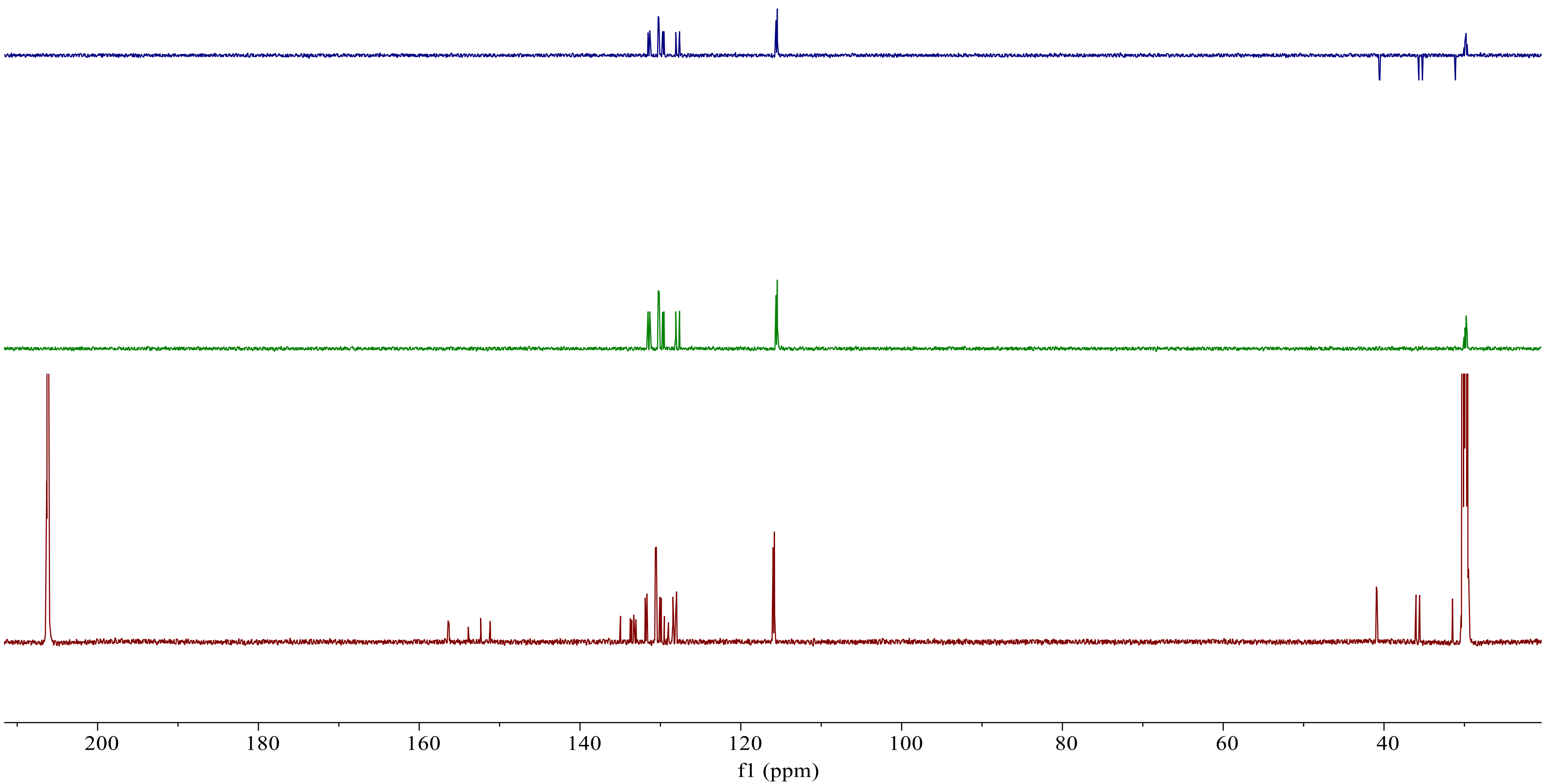

Figure S300. The DEPT spectrum of compound **27** in acetone-d_6 (150 MHz)

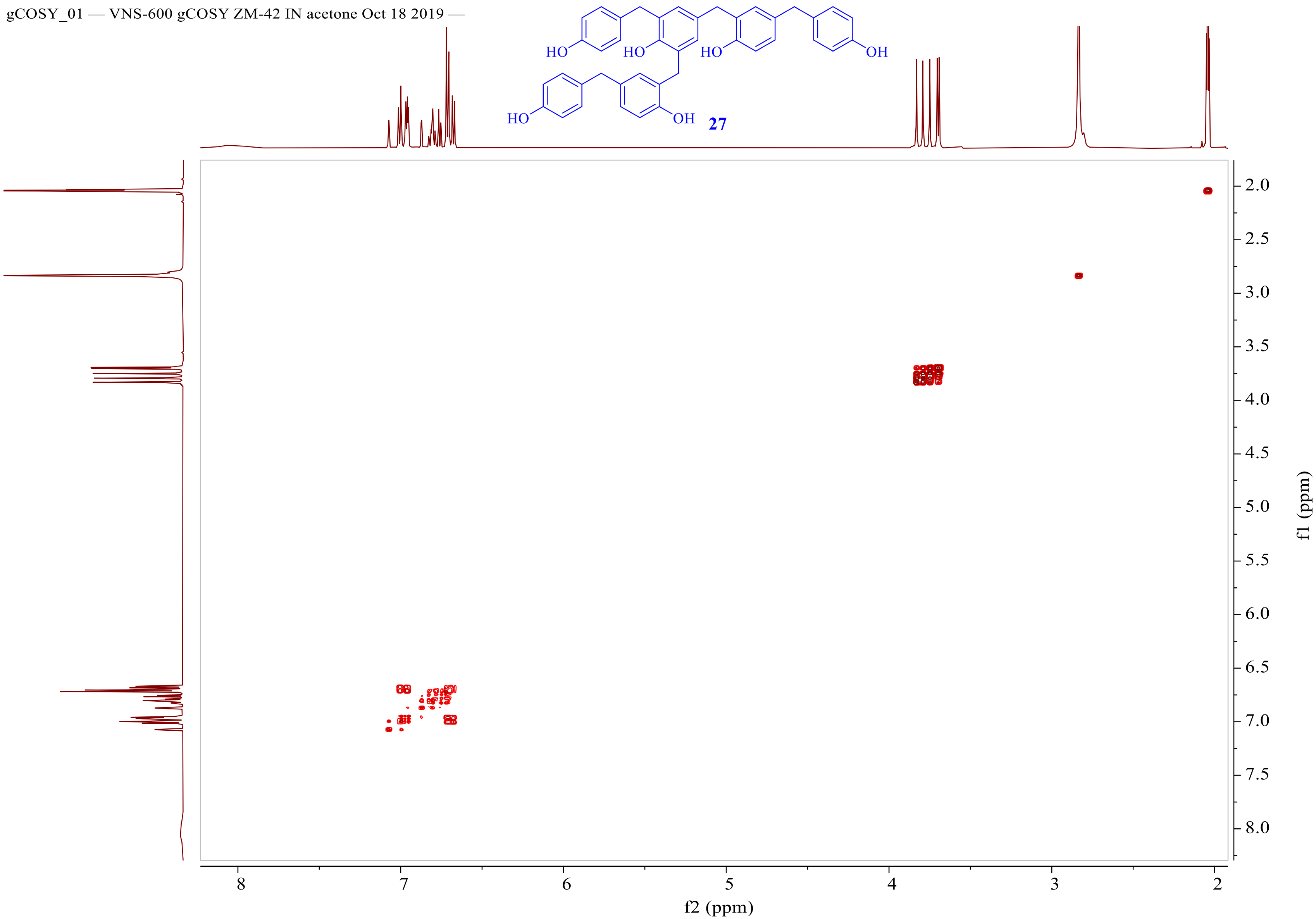

Figure S301. The ^{1}H-^{1}H COSY spectrum of compound **27** in acetone-d_6 (600 MHz)

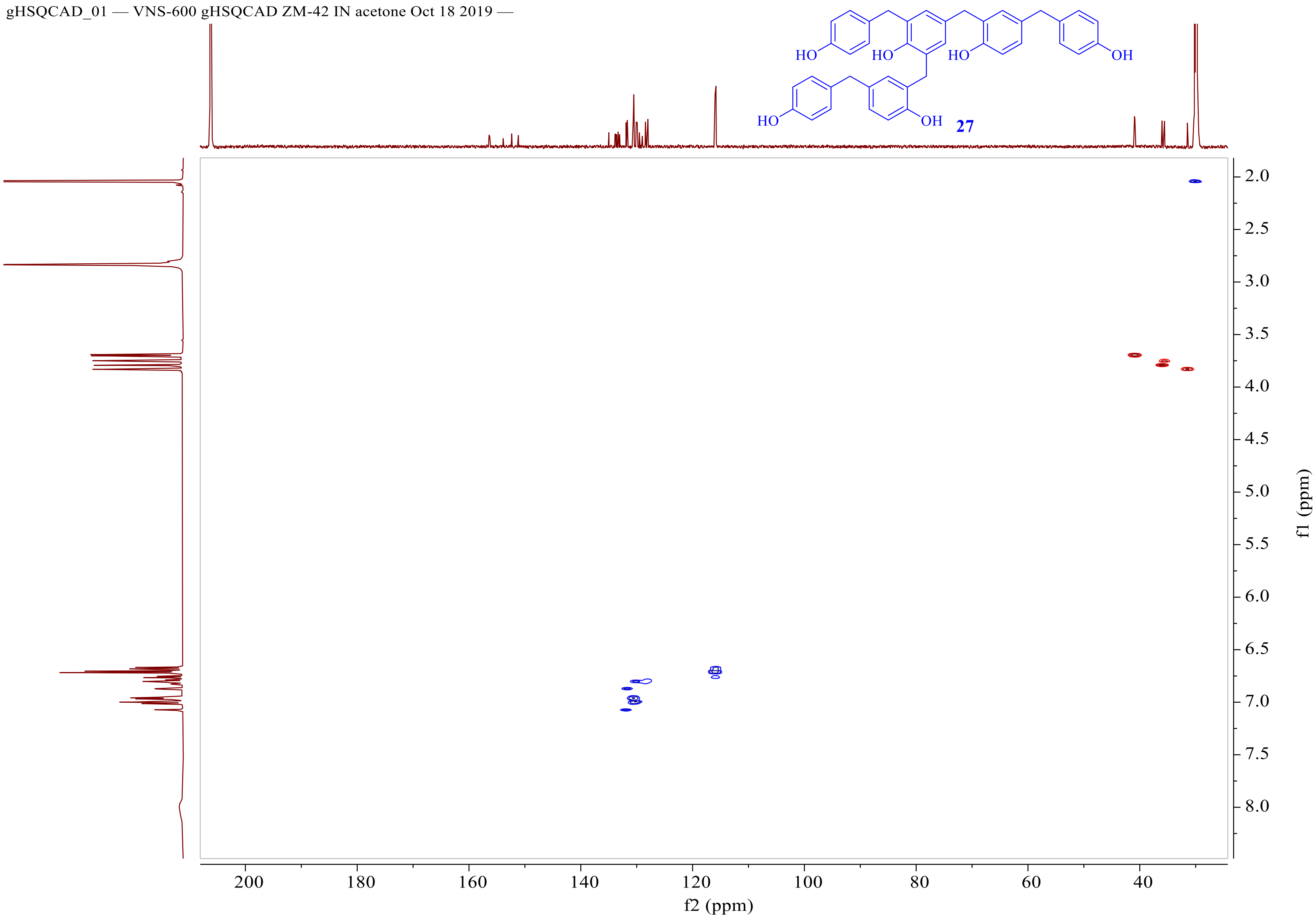

Figure S302. The HSQC spectrum of compound **27** in acetone-d_6 (600 MHz for ^{1}H)

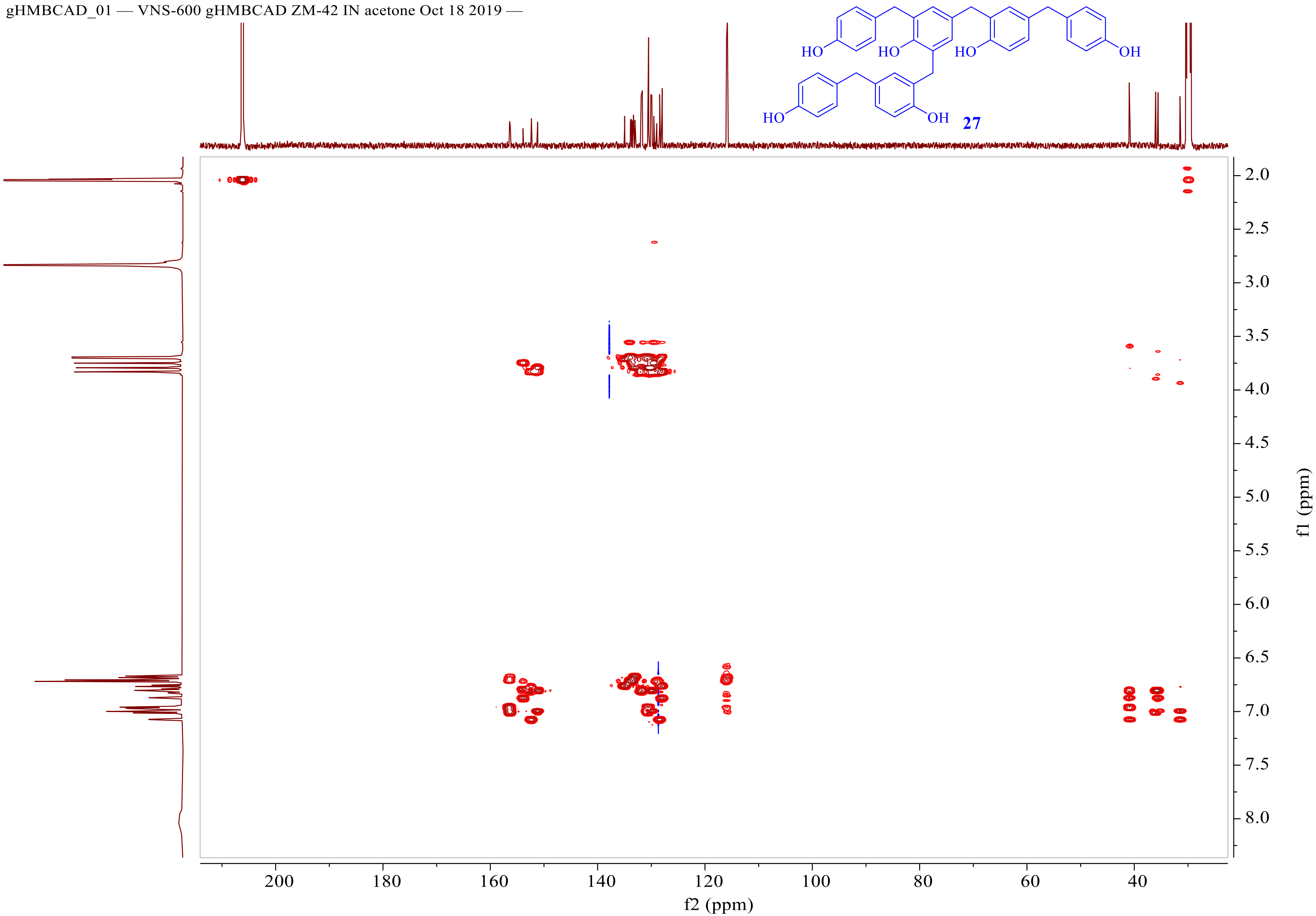

Figure S303. The HMBC spectrum of compound **27** in acetone-d_6 (600 MHz for ^{1}H)

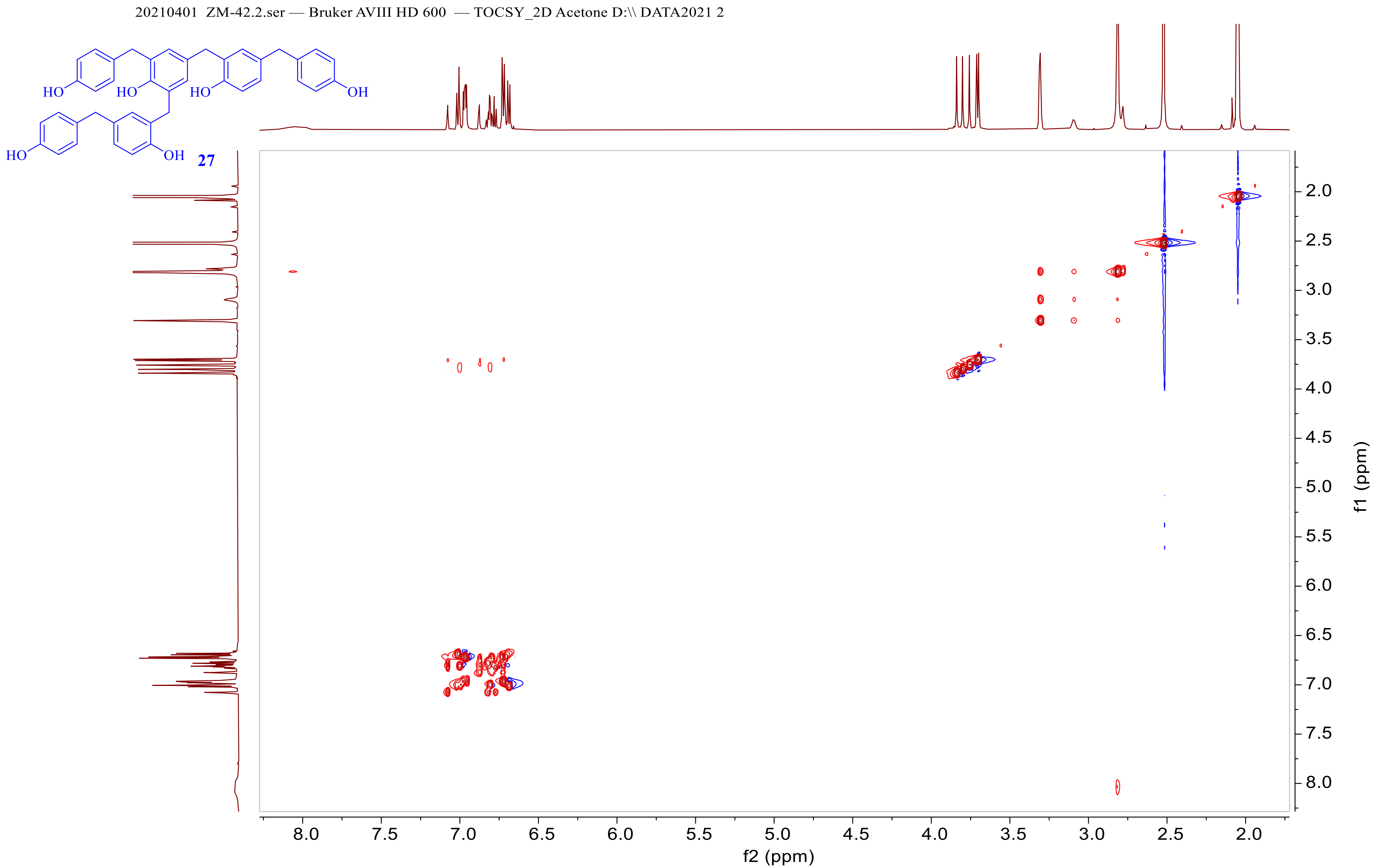

Figure S304. The 2D TOCSY spectrum of compound **27** in acetone-d_6 (600 MHz)

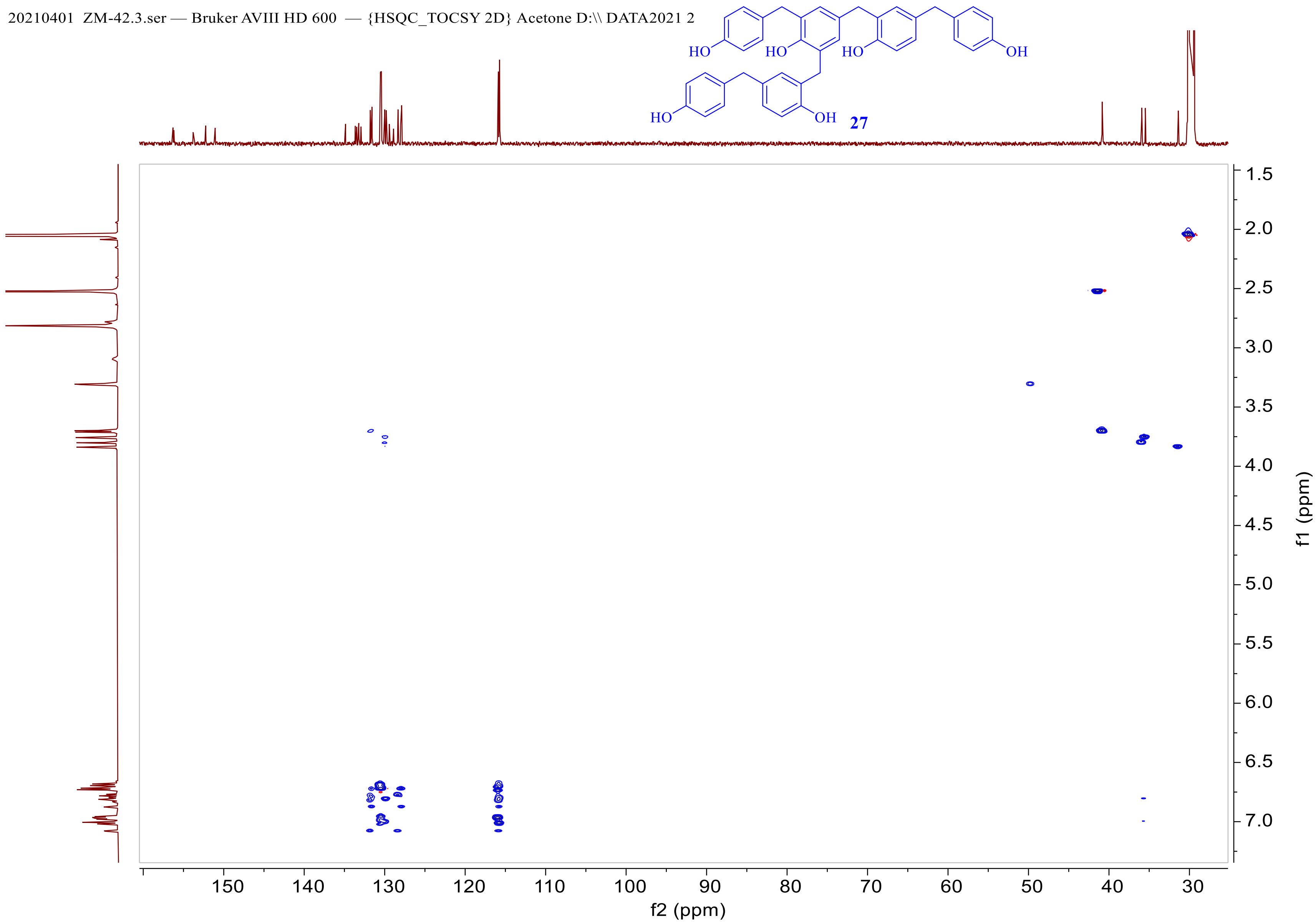

Figure S305. The HSQC-TOCSY spectrum of compound **27** in acetone-d_6 (600 MHz for ^{1}H)

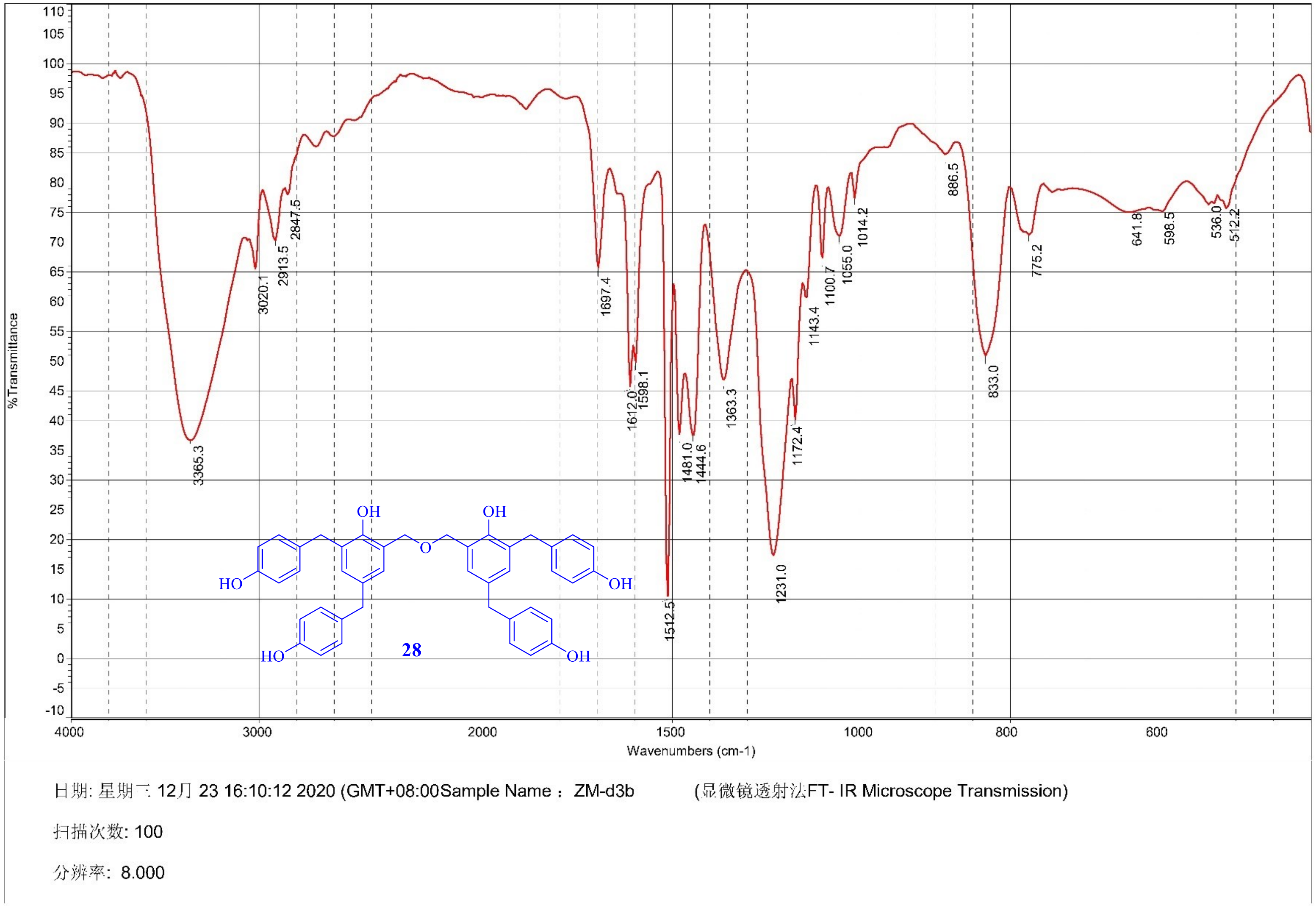

Figure S306. The IR spectrum of compound **28**

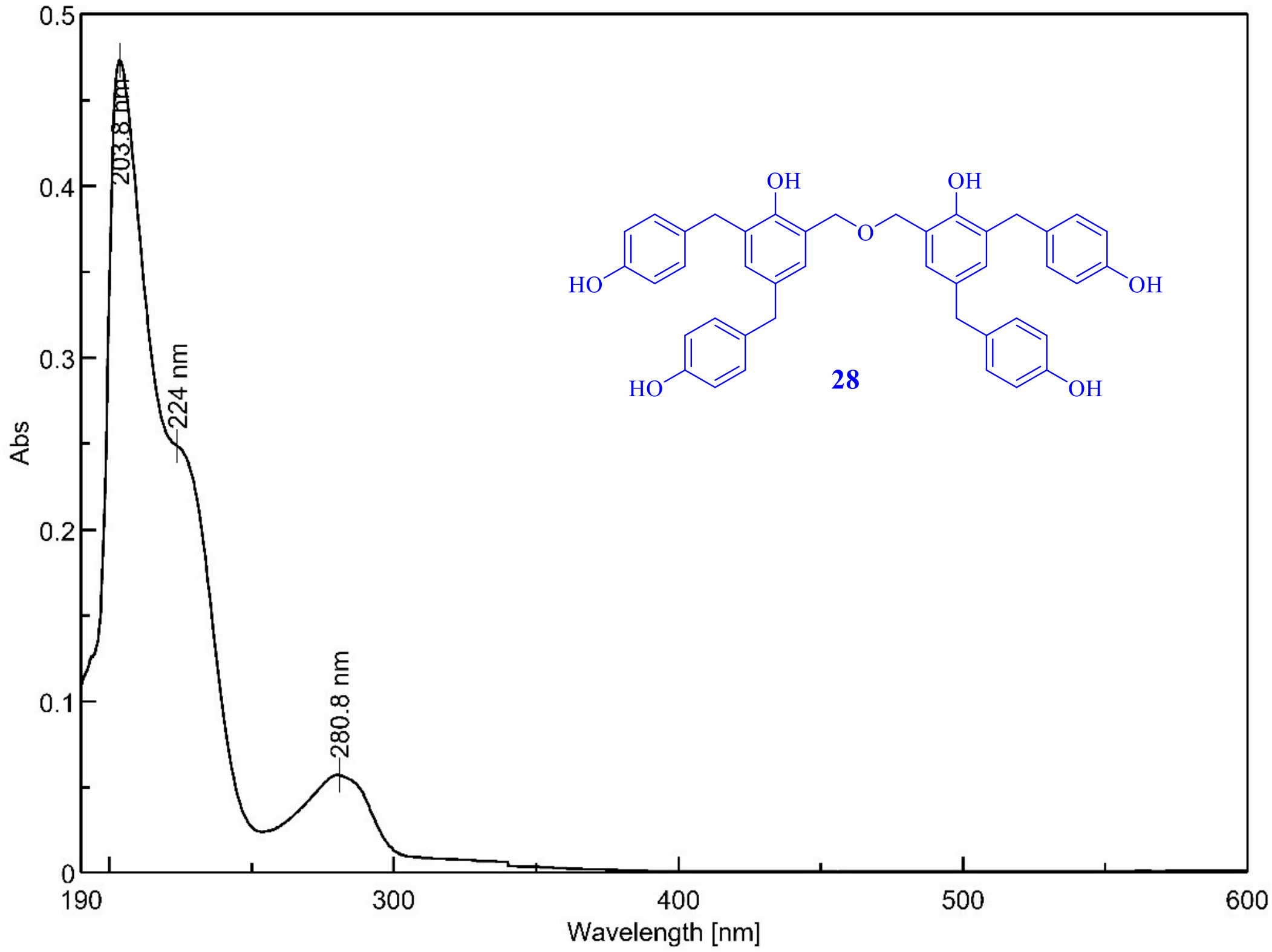

d3b

[Comment]
Sample Name DBT
Comment 0.02
User
Division UV
Company 324
[Measurement Information]
Instrument Name V-650
Model Name V-650
Serial No. A034461150

Accessory PSC-718
Accessory S/N A001761114
Position 1
Cell Length 10 mm
Temperature 19.97 C
Control Sonsor Holder
Monitor Sensor Holder
Start Mode Start immediately

Photometric Mode Abs
Measurement range 600 - 190 nm
Data pitch 0.2 nm
Band width(UV/Vis) 2.0 nm
Response Medium
Scanning speed 200 nm/min
Source Change 340 nm
Light Source D2/WI
Filter Exchange Step
Correction Baseline

[Data Information]
Creation Date 2020-11-11 16:16

Data array type Linear data array
Horizontal Wavelength [nm]
Vertical Abs
Start 600 nm
End 190 nm
Data pitch 0.2 nm
Data points 2051

Figure S307. The UV spectrum of compound **28** in MeOH

Thermo Qexactive Focus Report

compound NO. : d3b

Method : LCMS(compound)-low

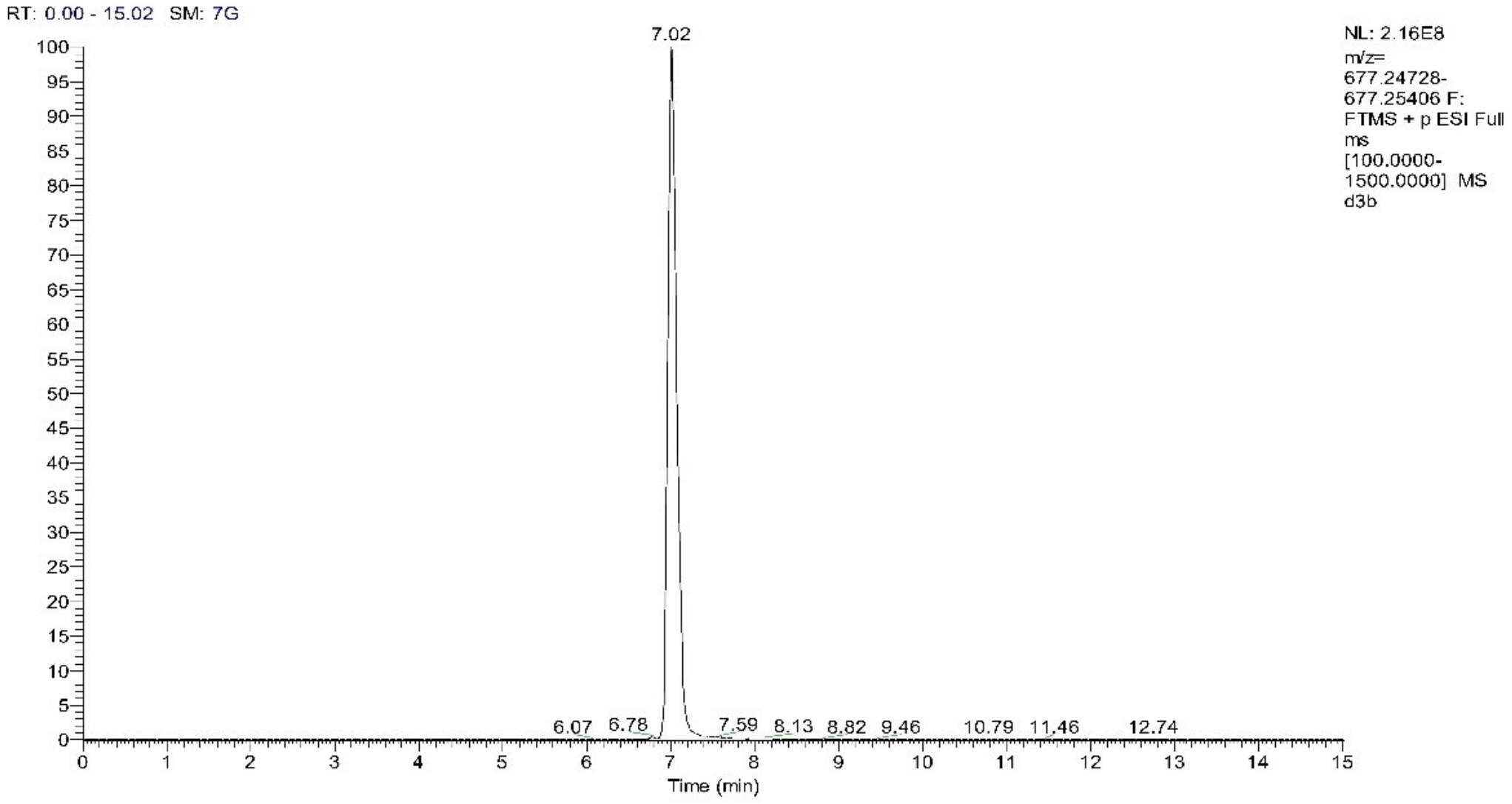

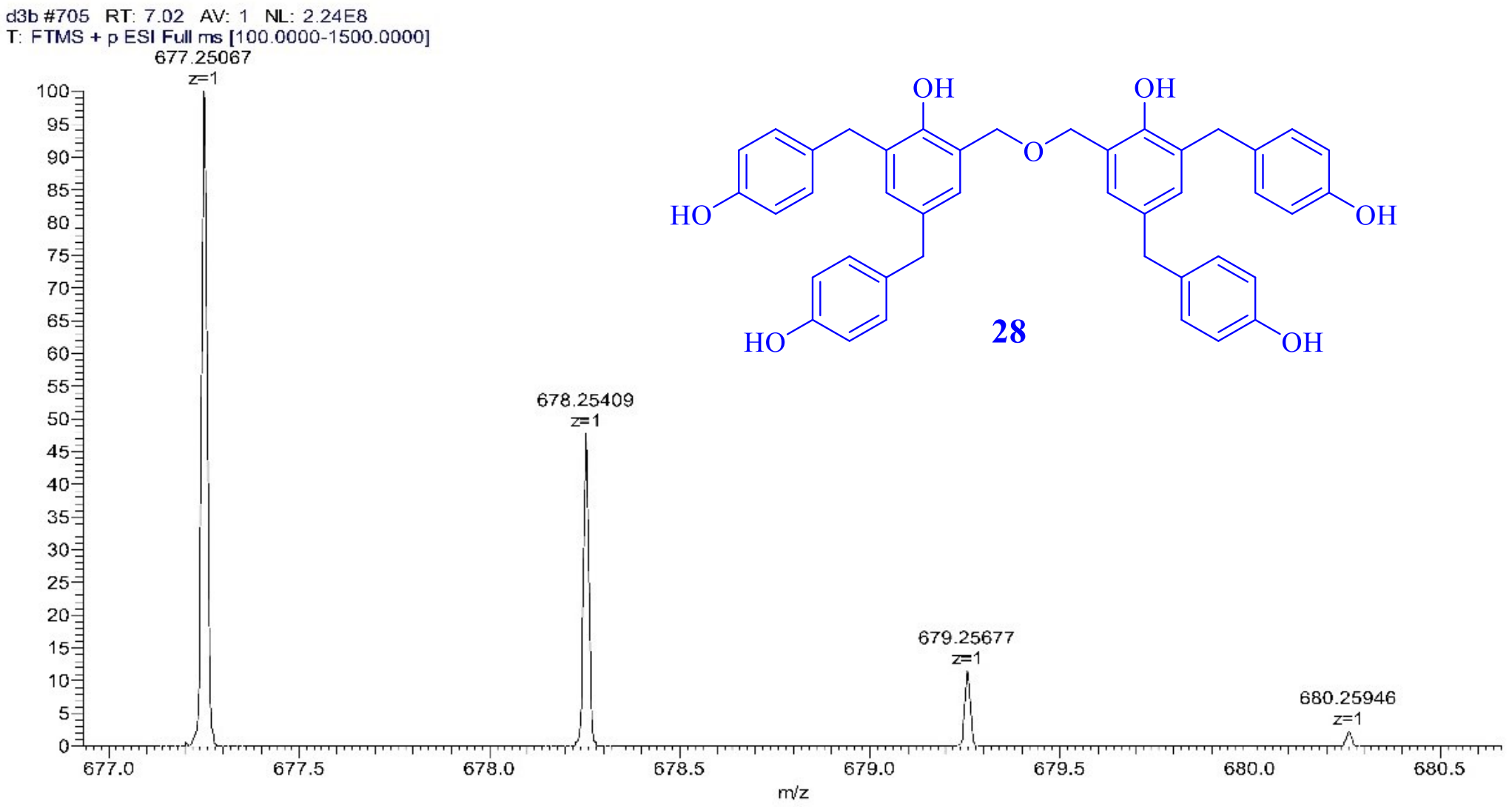

m/z	Theo. Mass	Delta (ppm)	RDB equiv.	Composition	
677.25067	677.25097	-0.45	23.5	C42 H38 O7 Na	M+Na

Figure S308. The HR-ESIMS report of compound **28**

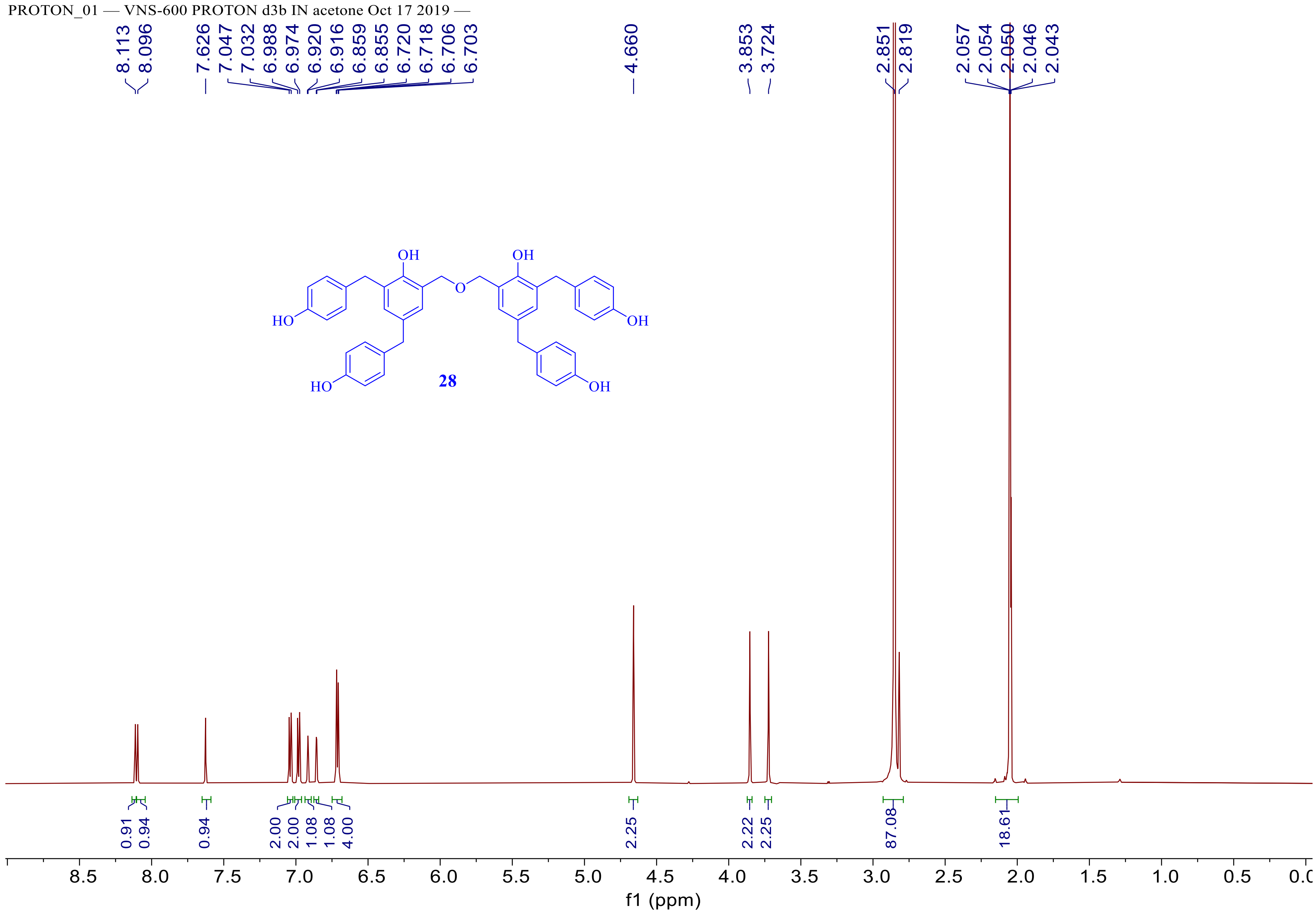

Figure S309. The ^{1}H NMR spectrum of compound **28** in acetone-d_6 (600 MHz)

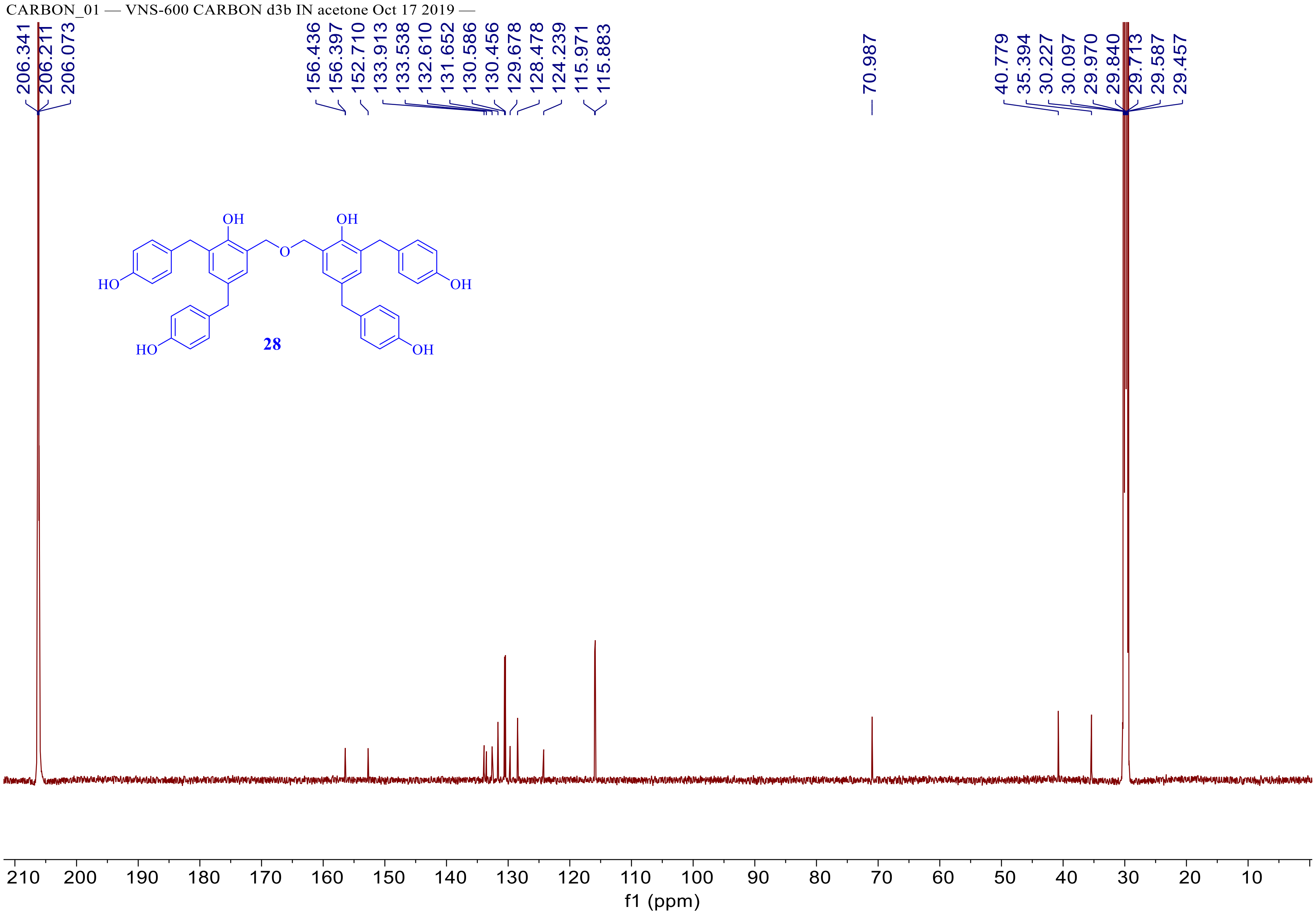

Figure S310. The ^{13}C NMR spectrum of compound **28** in acetone-d_6 (150 MHz)

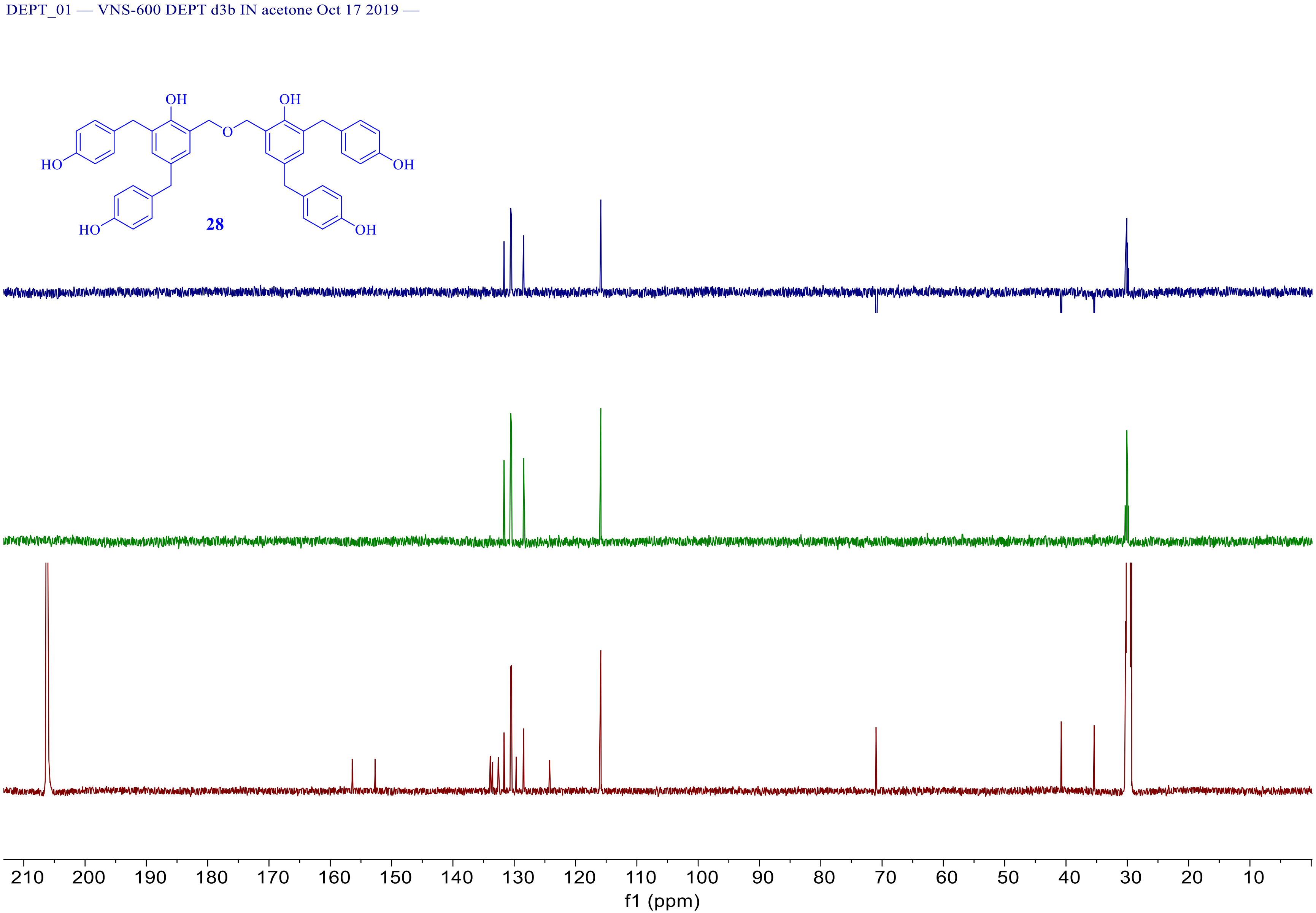

Figure S311. The DEPT spectrum of compound **28** in acetone-d_6 (150 MHz)

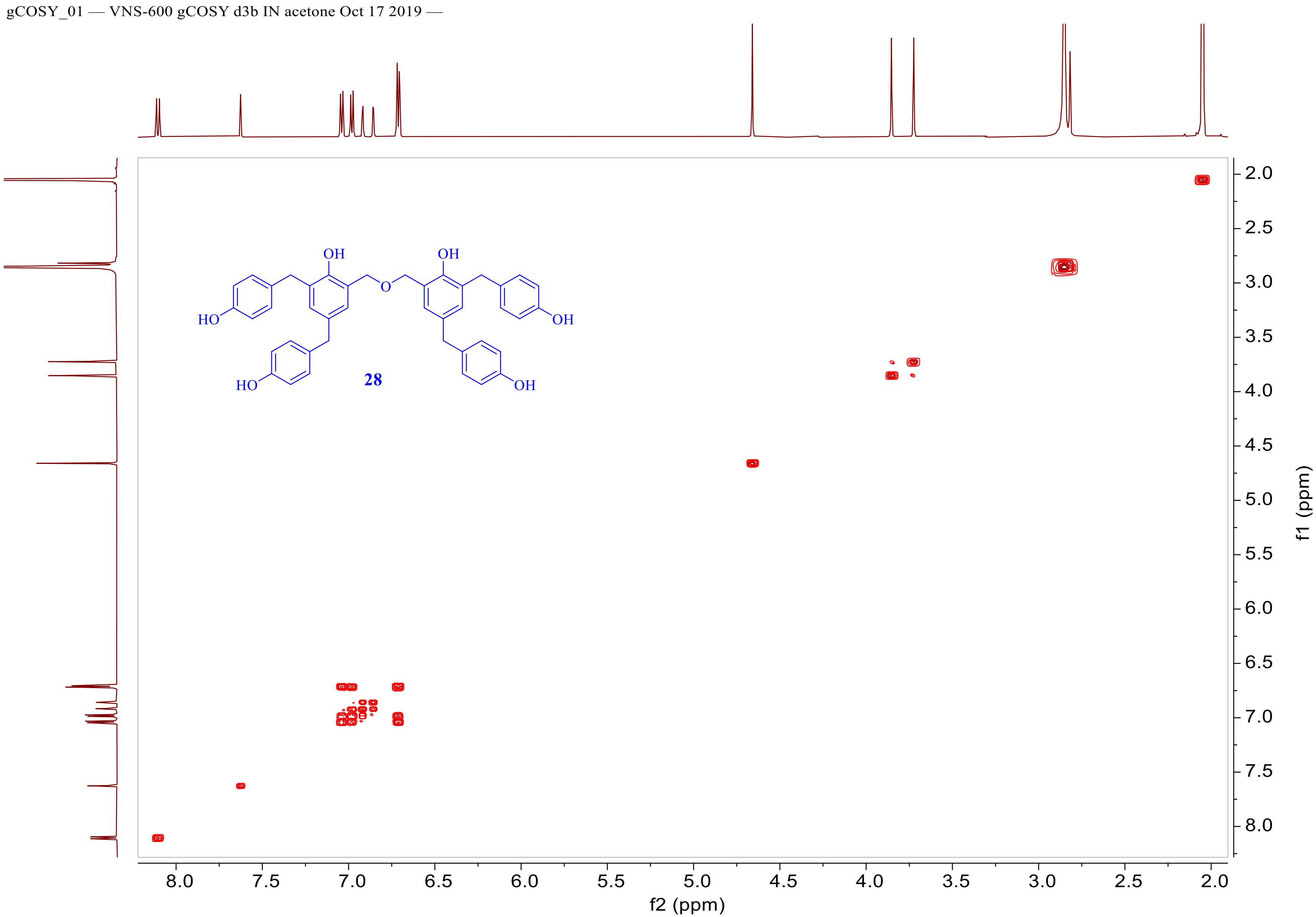

Figure S312. The ^{1}H-^{1}H COSY spectrum of compound **28** in acetone-d_6 (600 MHz)

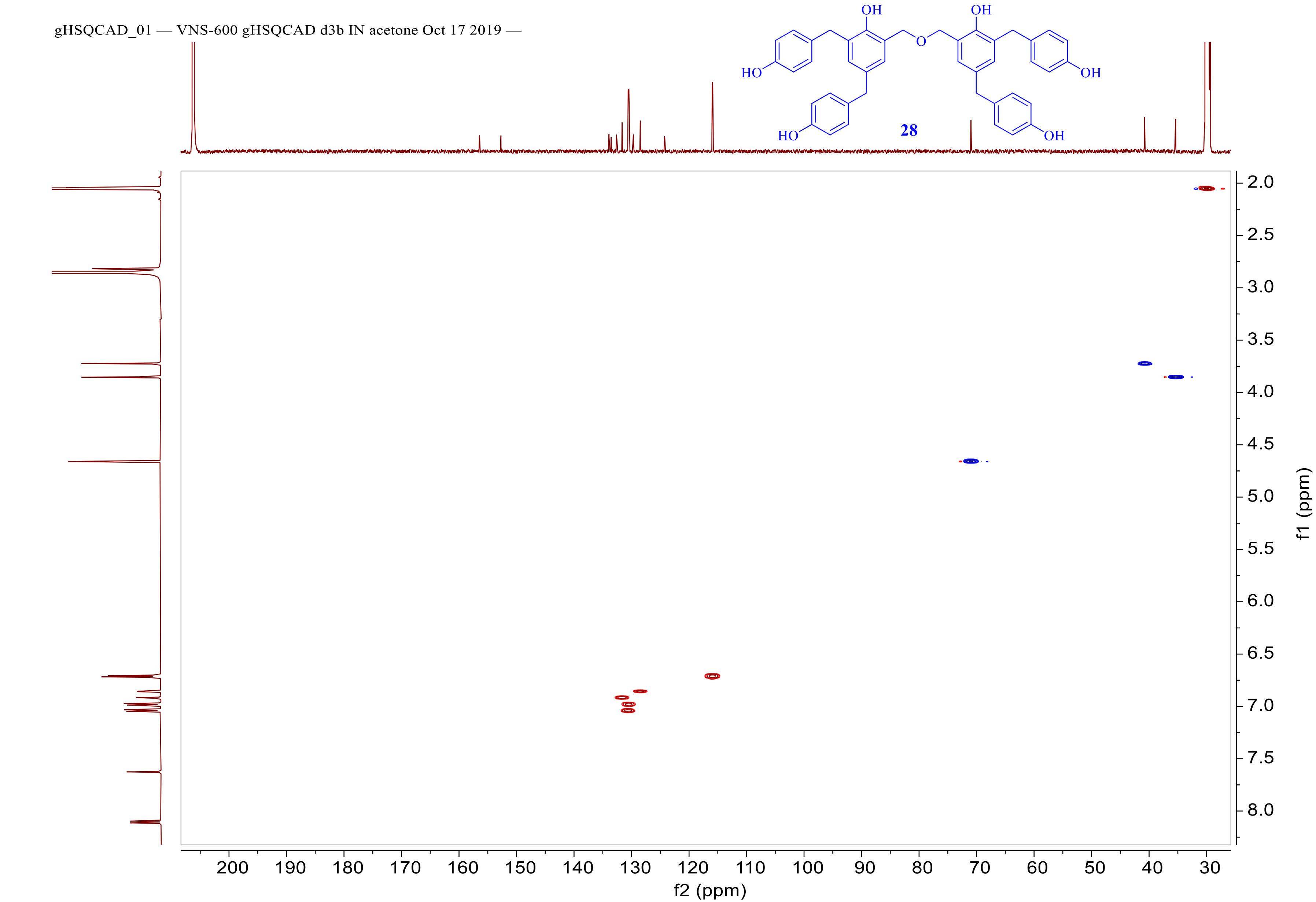

Figure S313. The HSQC spectrum of compound **28** in acetone-d_6 (600 MHz for ^1H)

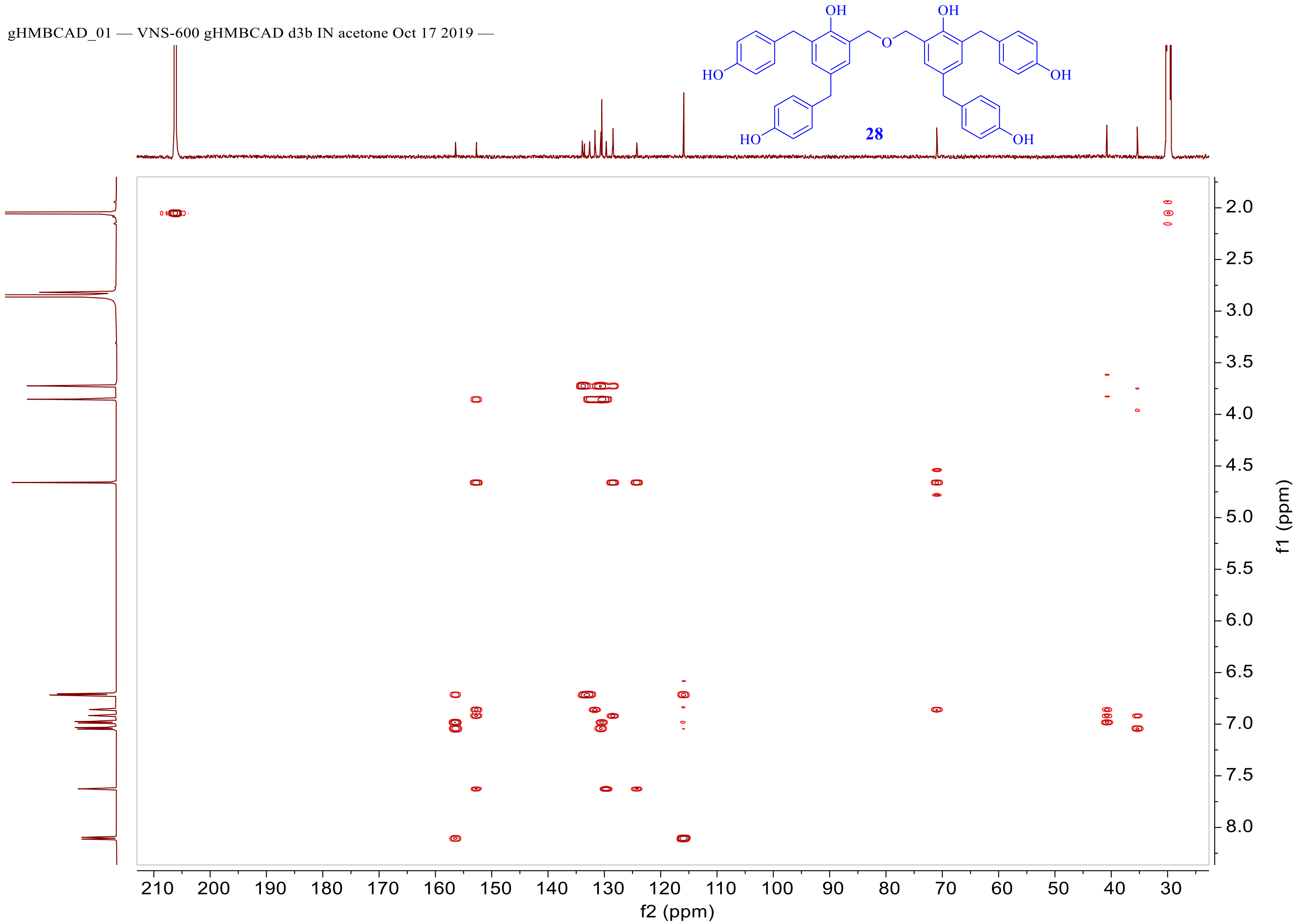

Figure S314. The HMBC spectrum of compound **28** in acetone-d_6 (600 MHz for ^{1}H)

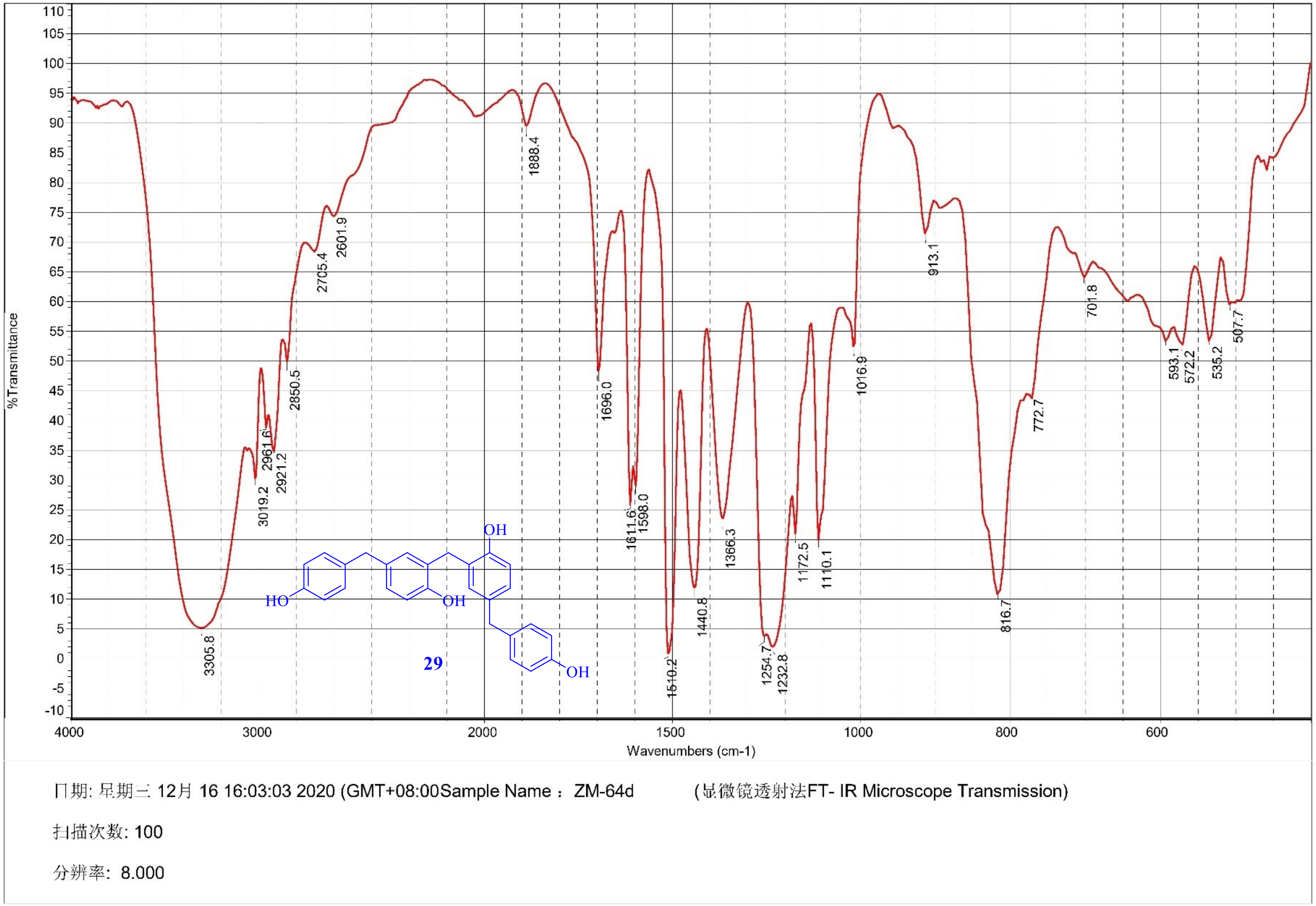

Figure S315. The IR spectrum of compound **29**

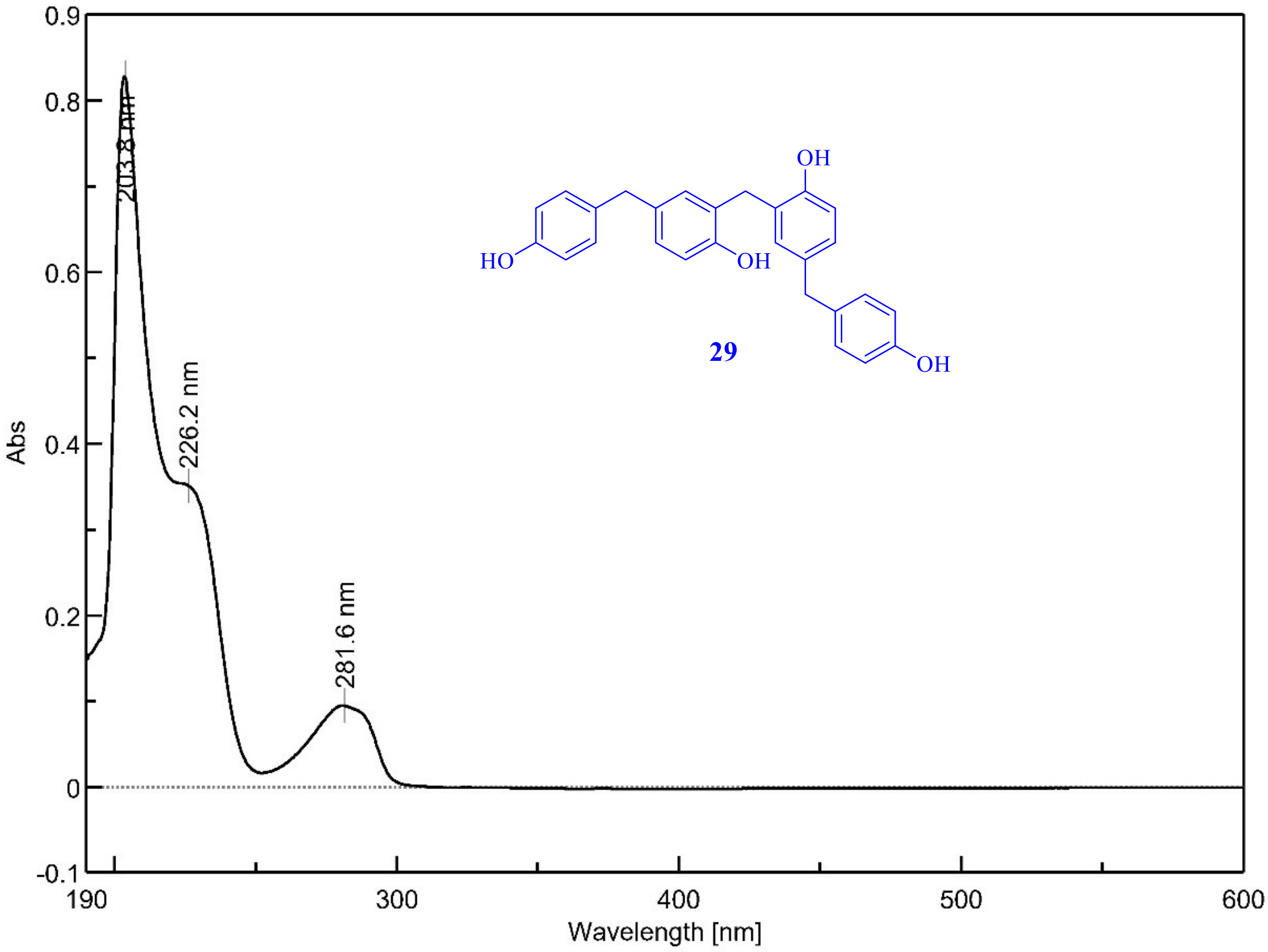

[Comment]	
Sample Name	DBT
Comment	0.02
User	
Division	UV
Company	324

——— b4d...2

[Measurement Information]	
Instrument Name	V-650
Model Name	V-650
Serial No.	A034461150
Accessory	PSC-718
Accessory S/N	A001761114
Position	1
Cell Length	10 mm
Temperature	20.00 C
Control Sonsor	Holder
Monitor Sensor	Holder
Start Mode	Start immediately
Photometric Mode	Abs
Measurement range	600 - 190 nm
Data pitch	0.2 nm
Band width(UV/Vis)	2.0 nm
Response	Medium
Scanning speed	200 nm/min
Source Change	340 nm
Light Source	D2/WI
Filter Exchange	Step
Correction	Baseline

[Data Information]	
Creation Date	2020-11-11 15:24
Data array type	Linear data array
Horizontal	Wavelength [nm]
Vertical	Abs
Start	600 nm
End	190 nm
Data pitch	0.2 nm
Data points	2051

Figure S316. The UV spectrum of compound **29** in MeOH

Thermo Qexactive Focus Report

compound NO. : b5d
Method : LCMS(compound)-low

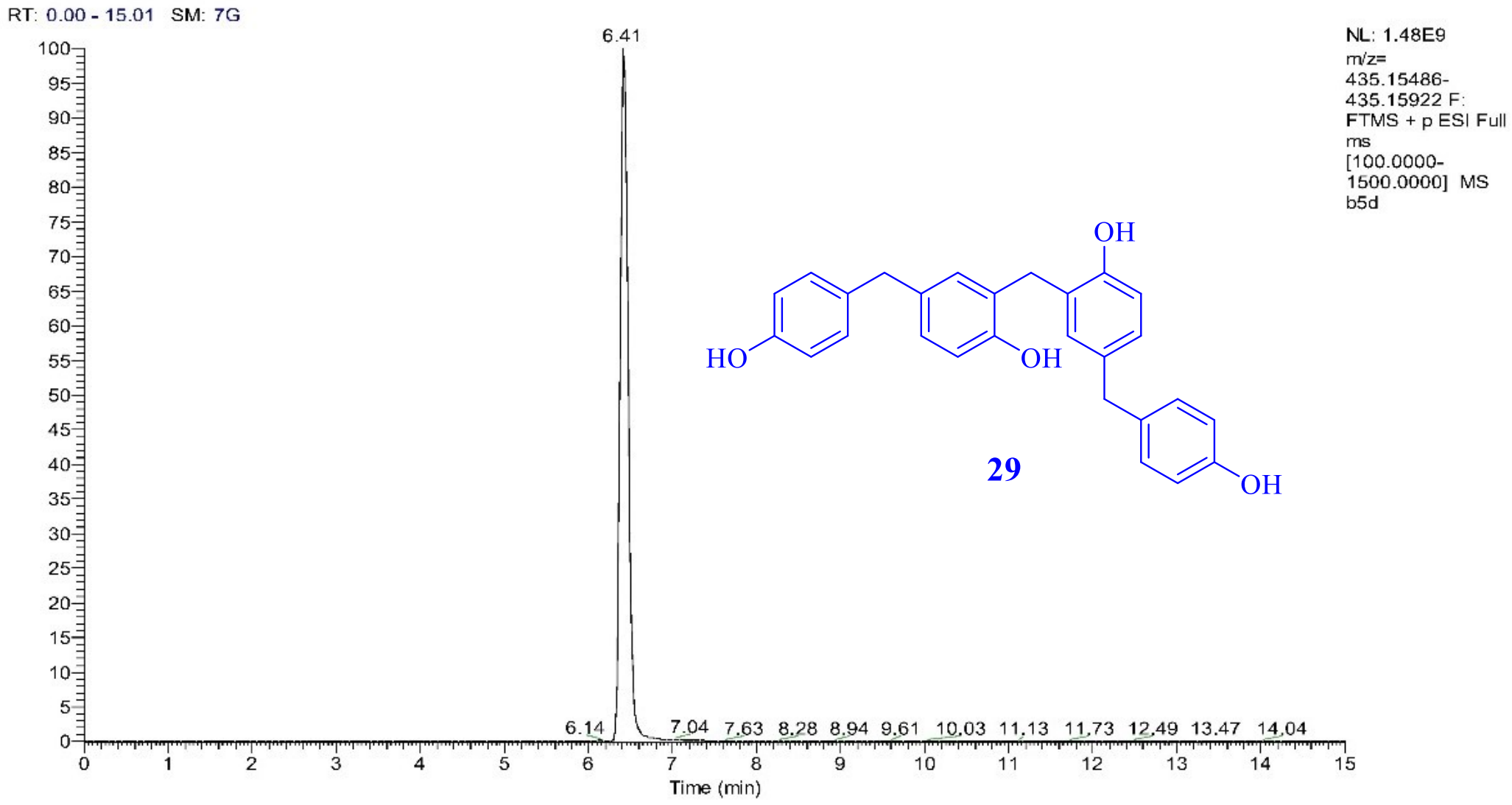

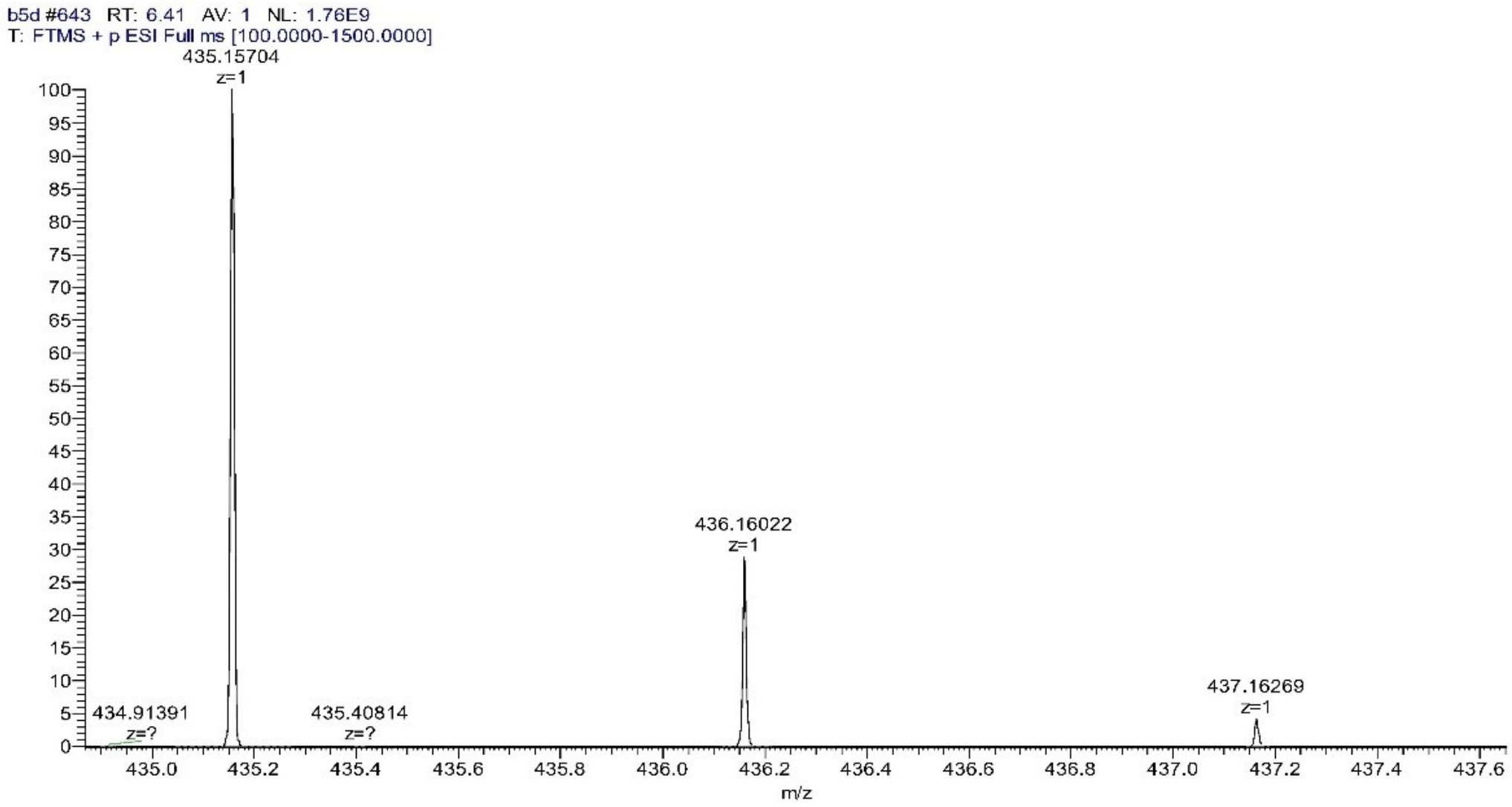

m/z	Theo. Mass	Delta (ppm)	RDB equiv.	Composition	
435.15704	435.15668	0.83	15.5	C27 H24 O4 Na	M+Na

Figure S317. The HR-ESIMS report of compound **29**

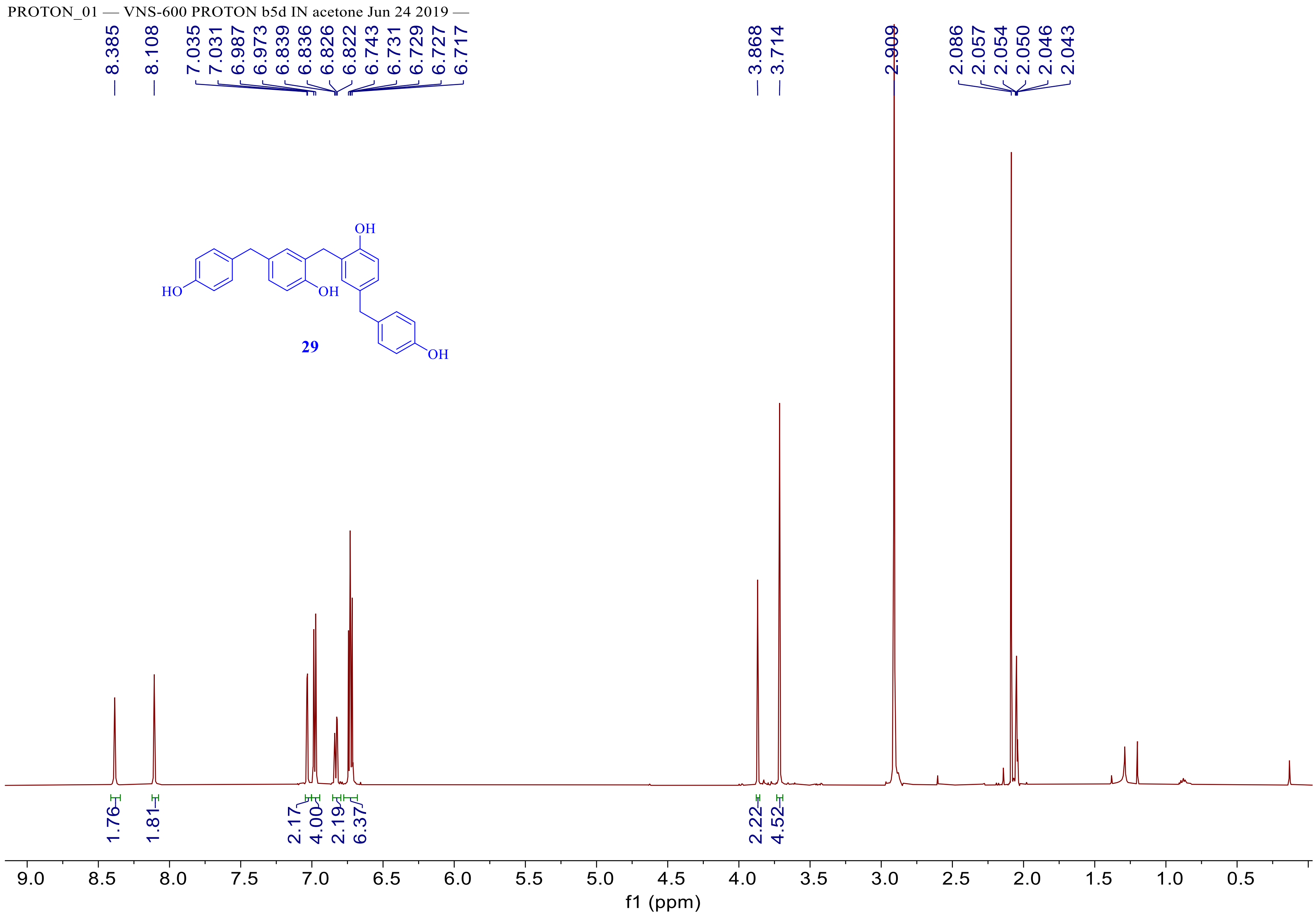

Figure S318. The ^{1}H NMR spectrum of compound **29** in acetone-d_6 (600 MHz)

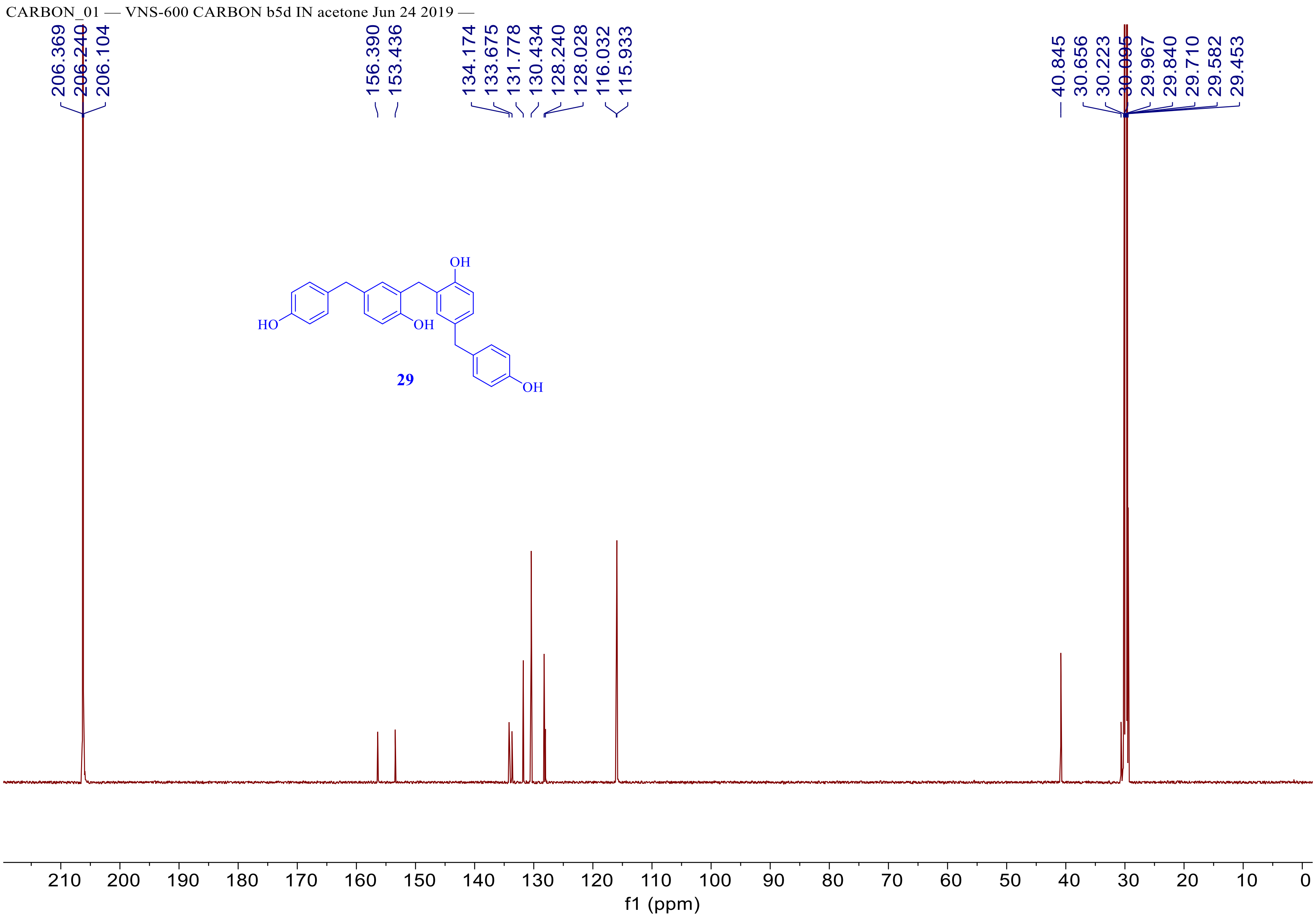

Figure S319. The ^{13}C NMR spectrum of compound **29** in acetone-d_6 (150 MHz)

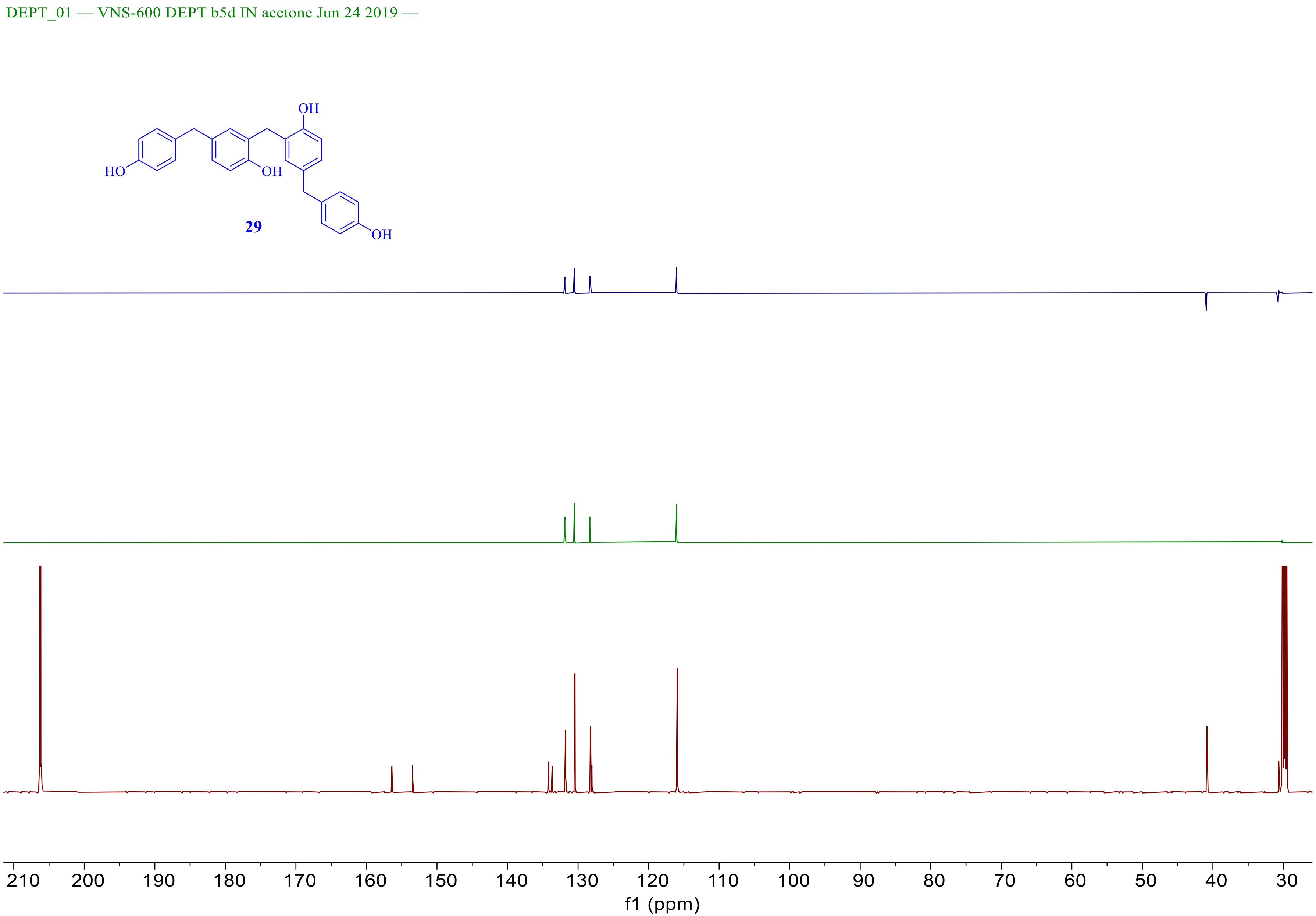

Figure S320. The DEPT spectrum of compound **29** in acetone-d_6 (150 MHz)

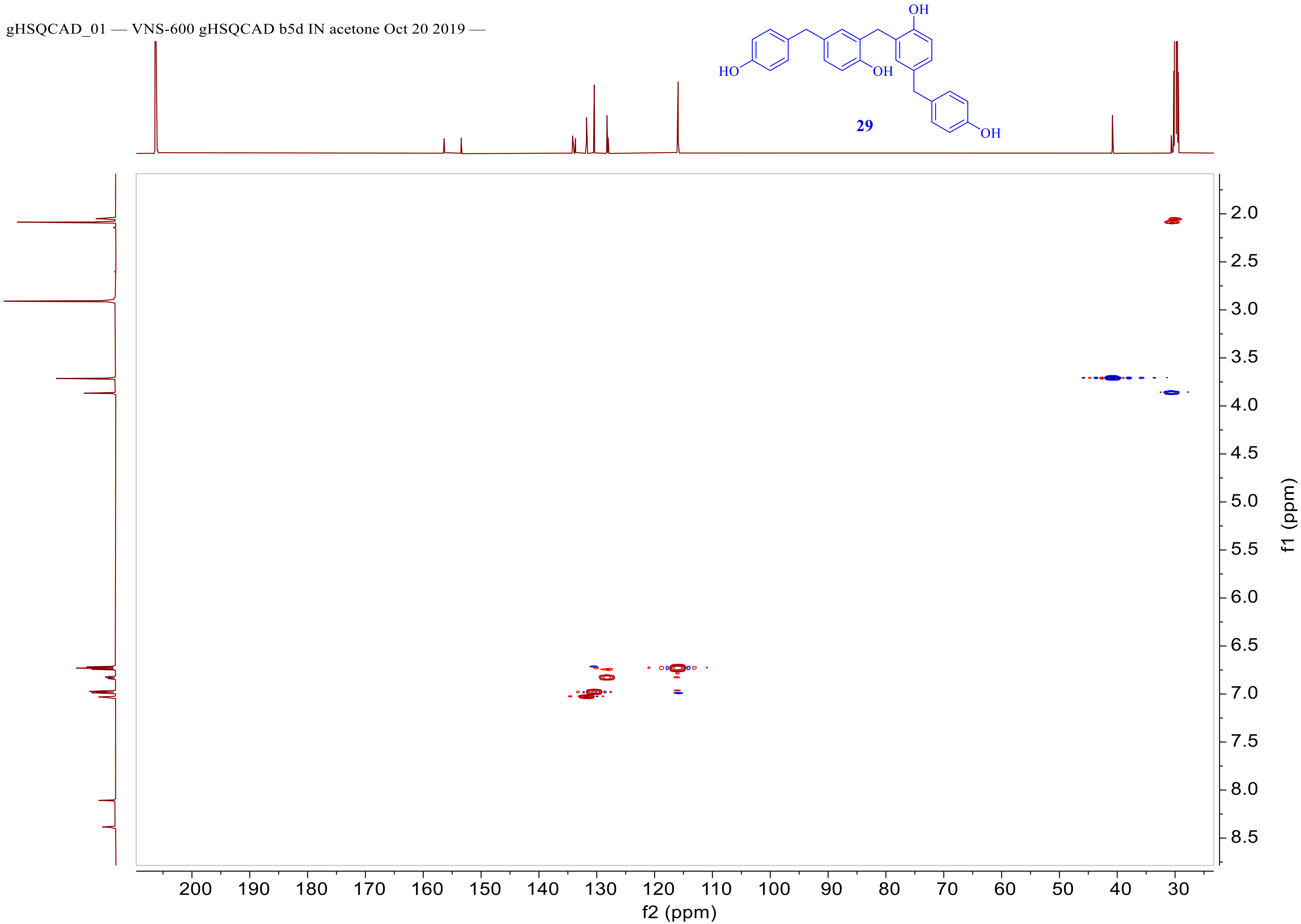

Figure S321. The HSQC spectrum of compound **29** in acetone-d_6 (600 MHz for ^{1}H)

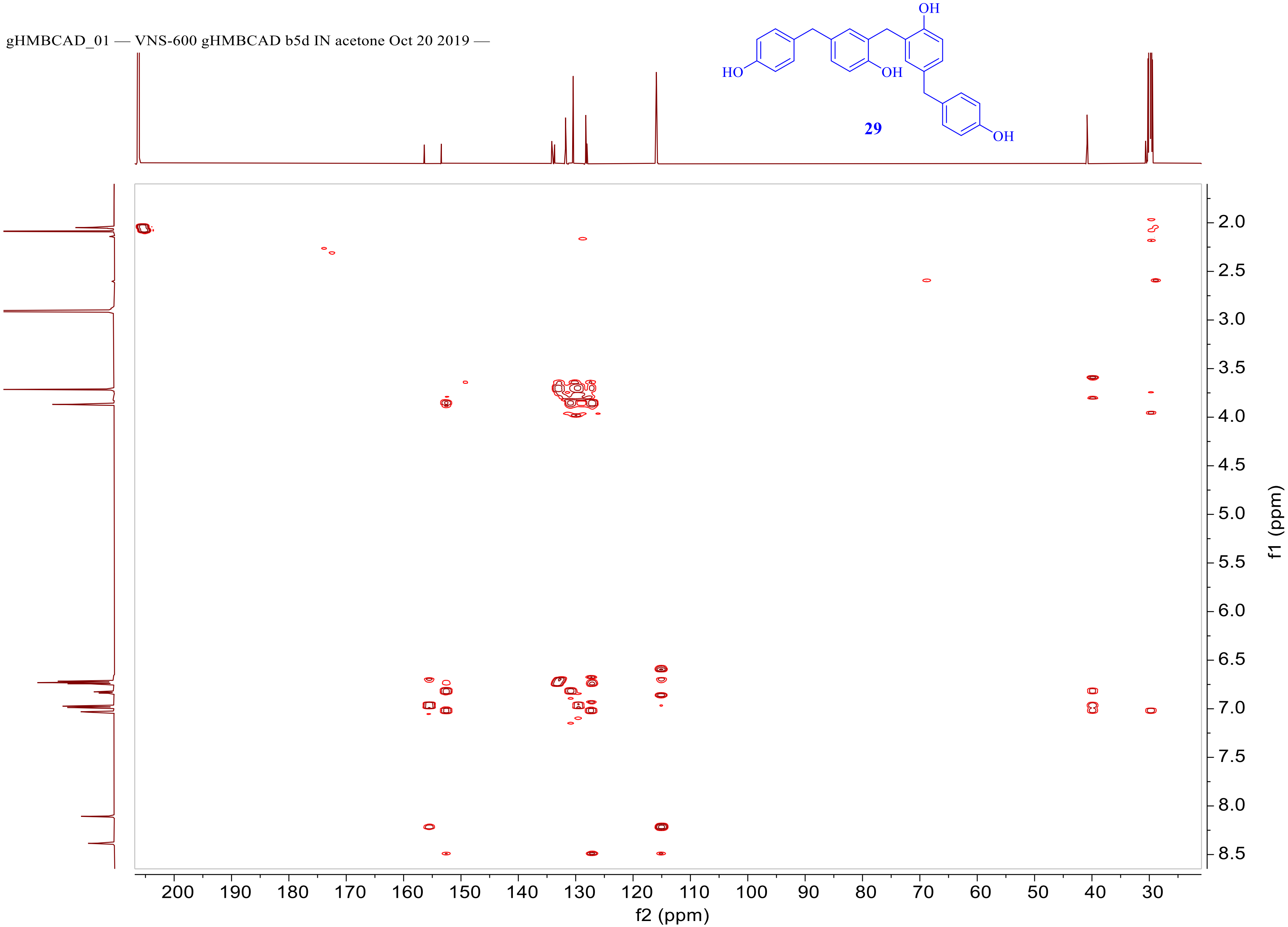

Figure S322. The HMBC spectrum of compound **29** in acetone-d_6 (600 MHz for ^{1}H)

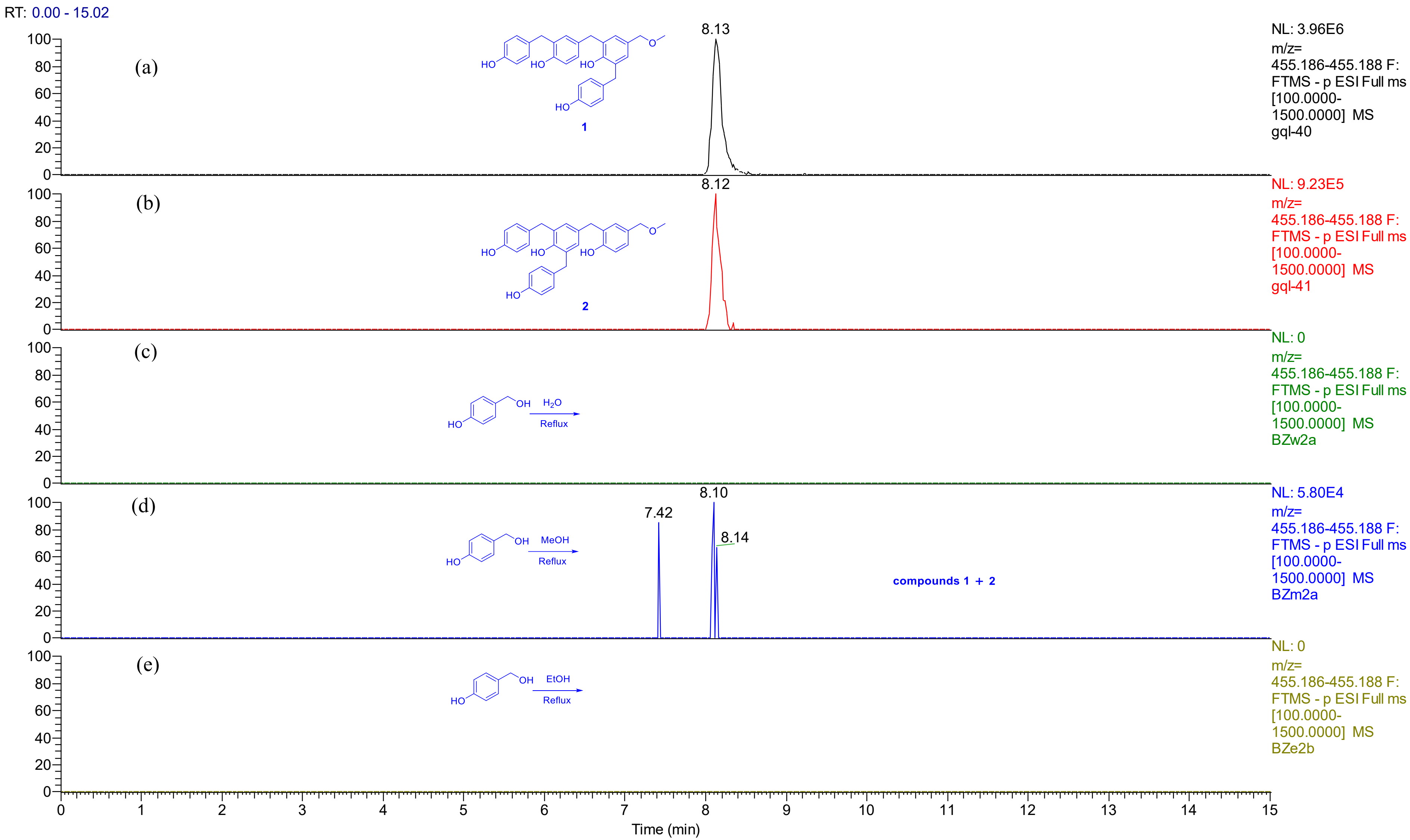

Figure S323. Overlaid chromatograms of the extracted negative ion at *m/z* 455.187 $[M–H]^-$: (a) and (b) compounds **1** and **2** in CH_3CN, respectively; (c)–(e) H_2O, MeOH, and EtOH solutions of *p*-hydroxybenzyl alcohol were sonicated for 0.5 h then refluxed for 1.0 h, respectively.

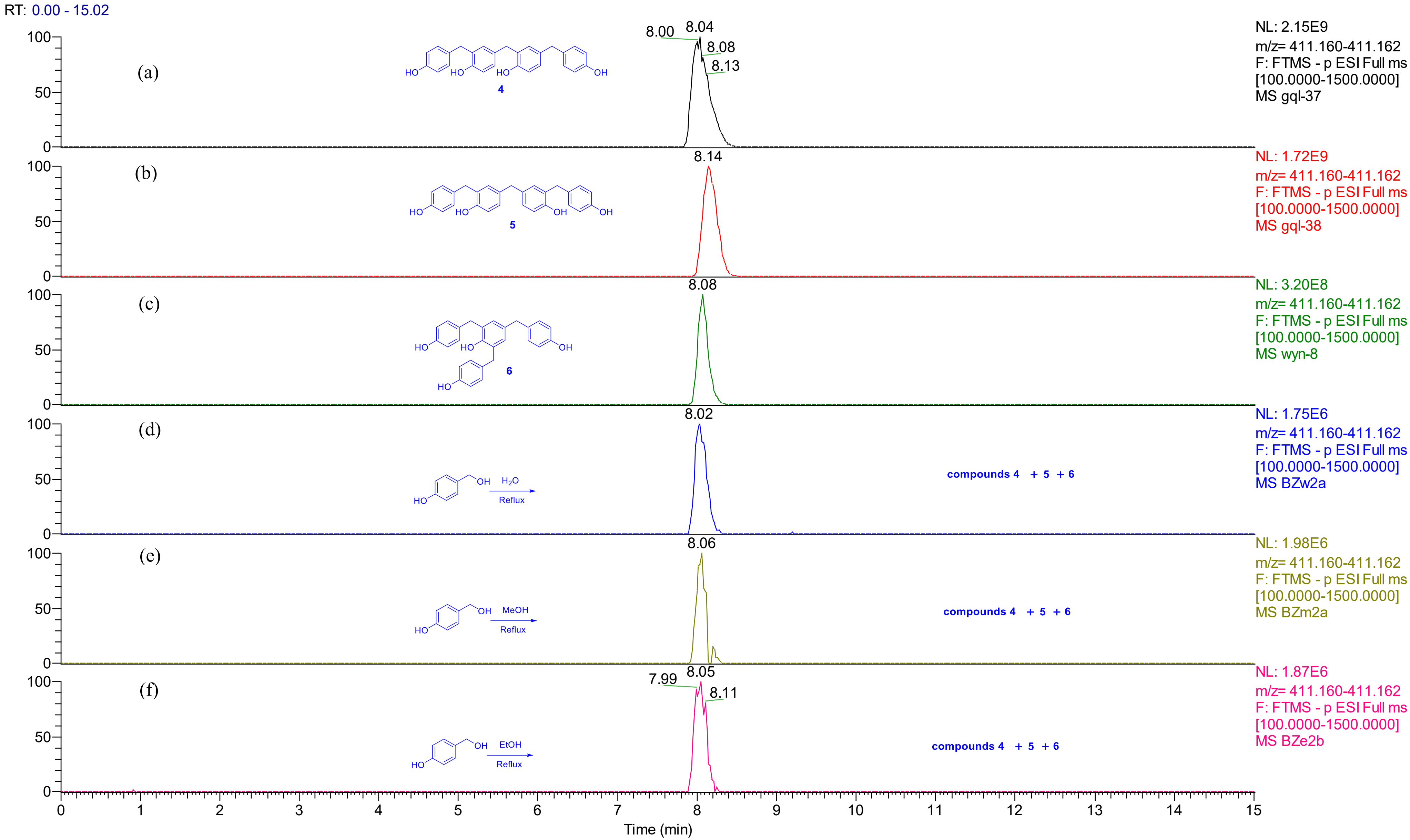

Figure S324. Overlaid chromatograms of the extracted negative ion at *m/z* 411.161 [M–H]$^-$: (a)–(c) compounds **4**–**6** in CH_3CN, respectively; (d)–(f) H_2O, MeOH, and EtOH solutions of *p*-hydroxybenzyl alcohol were sonicated for 0.5 h then refluxed for 1.0 h, respectively.

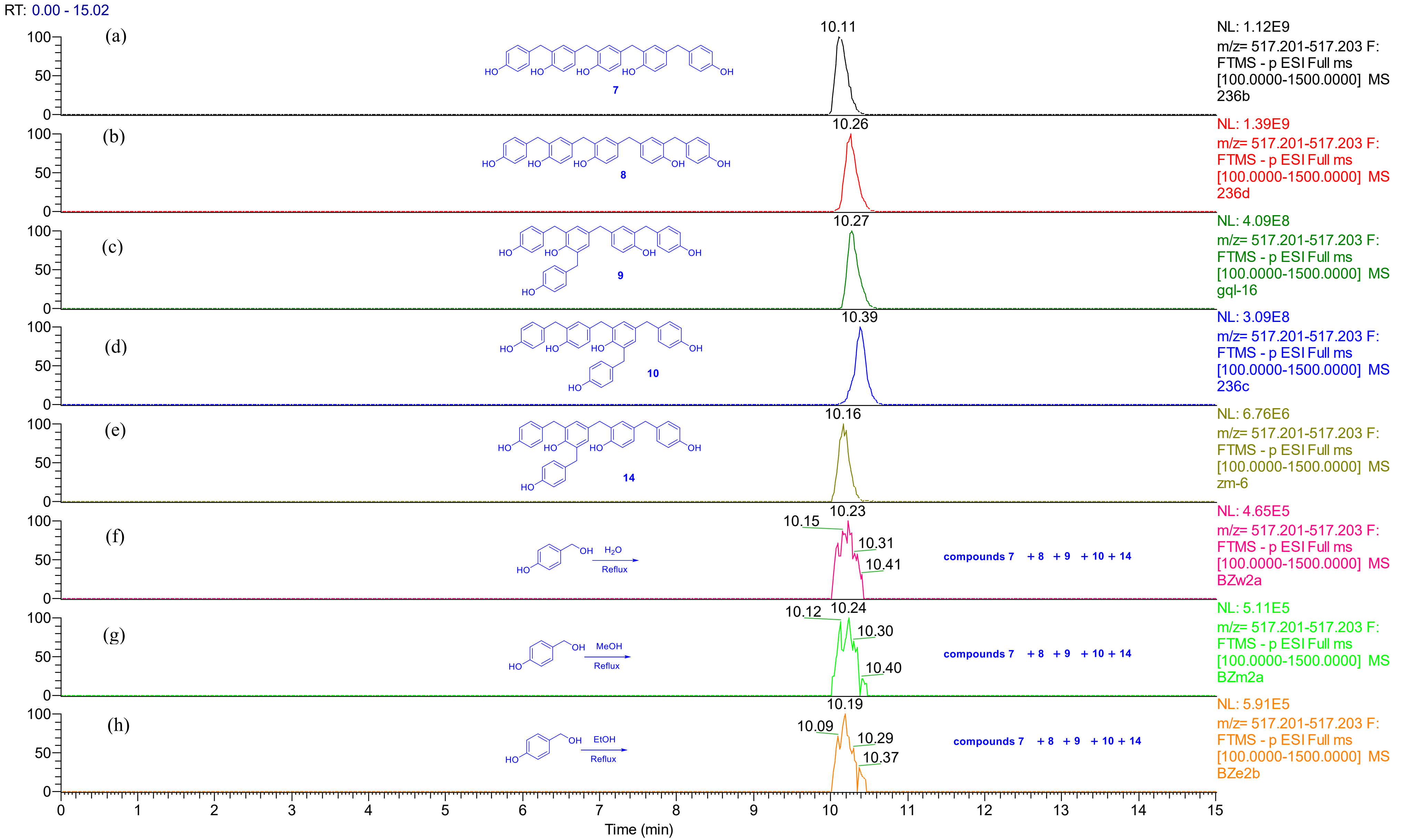

Figure S325. Overlaid chromatograms of the extracted negative ion at *m/z* 517.202 [M–H]⁻: (a)–(e) compounds **7**–**10** and **14** in CH_3CN, respectively; (f)–(h) H_2O, MeOH, and EtOH solutions of *p*-hydroxybenzyl alcohol were sonicated for 0.5 h then refluxed for 1.0 h, respectively.

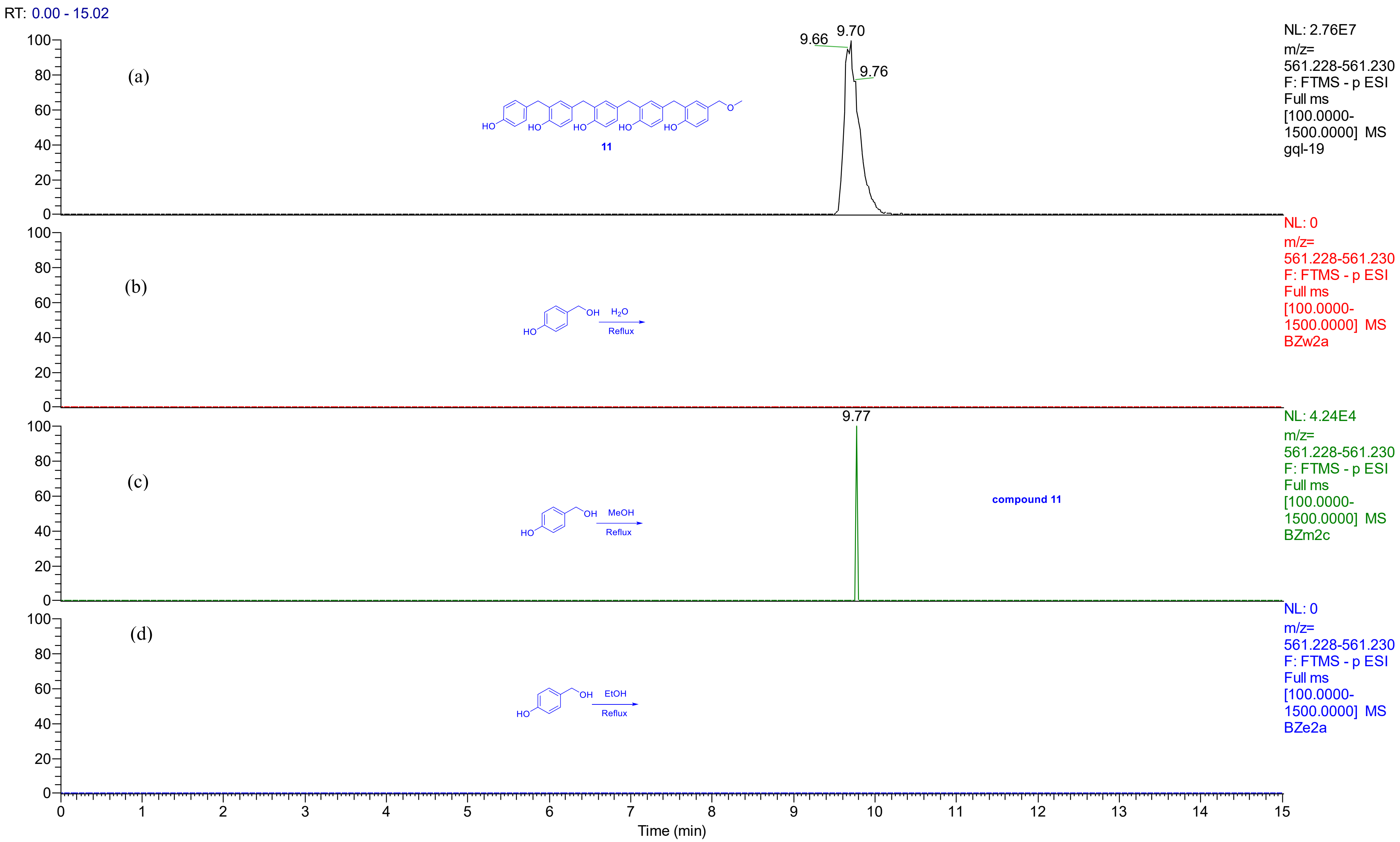

Figure S326. Overlaid chromatograms of the extracted negative ion at *m/z* 561.229 $[M–H]^-$: (a) compound **11** in CH_3CN; (b)–(d) H_2O, MeOH, and EtOH solutions of *p*-hydroxybenzyl alcohol were sonicated for 0.5 h then refluxed for 1.0 h, respectively.

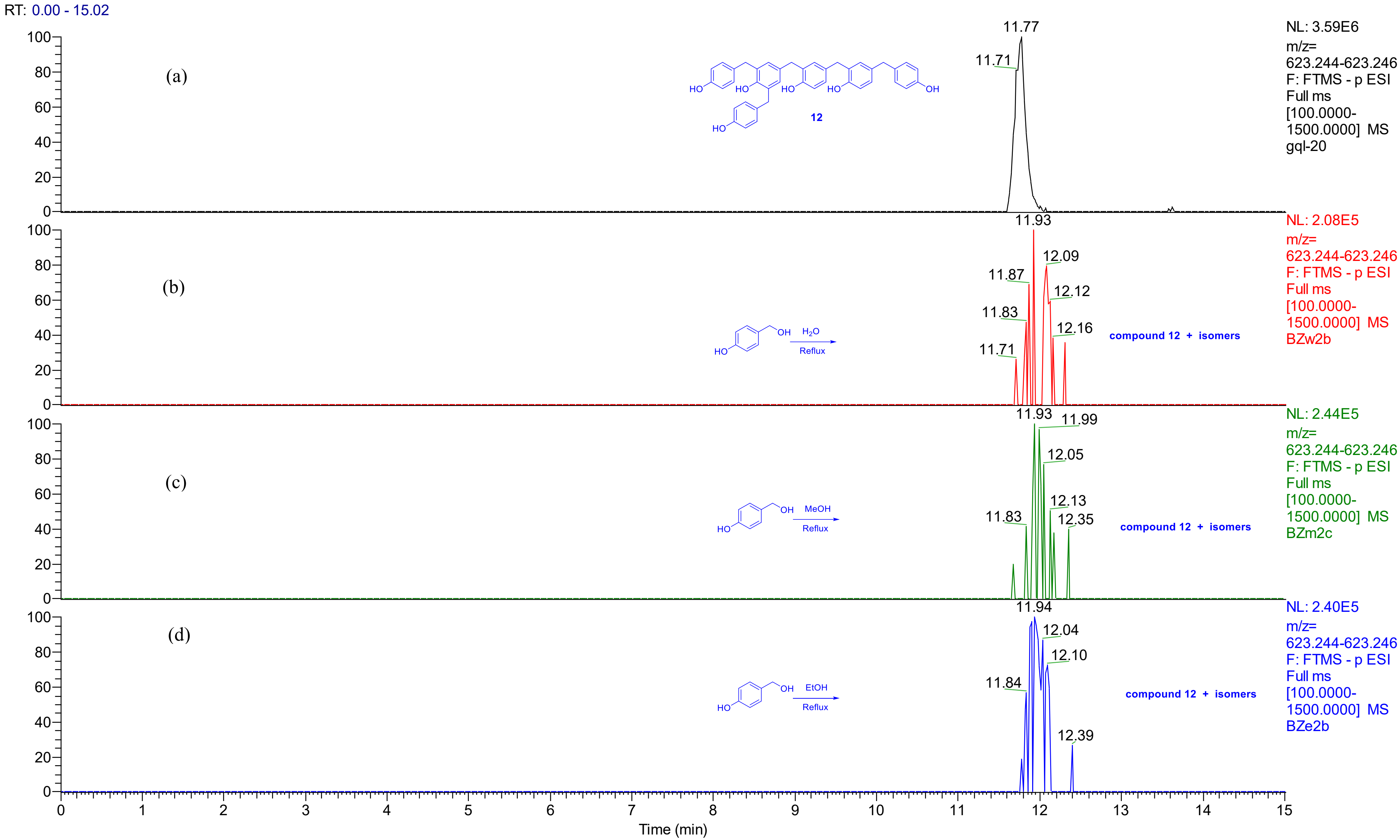

Figure S327. Overlaid chromatograms of the extracted negative ion at *m/z* 623.245 $[M-H]^-$: (a) and (b) compound **12** in CH_3CN, respectively; (c)–(e) H_2O, MeOH, and EtOH solutions of *p*-hydroxybenzyl alcohol were sonicated for 0.5 h then refluxed for 1.0 h, respectively.

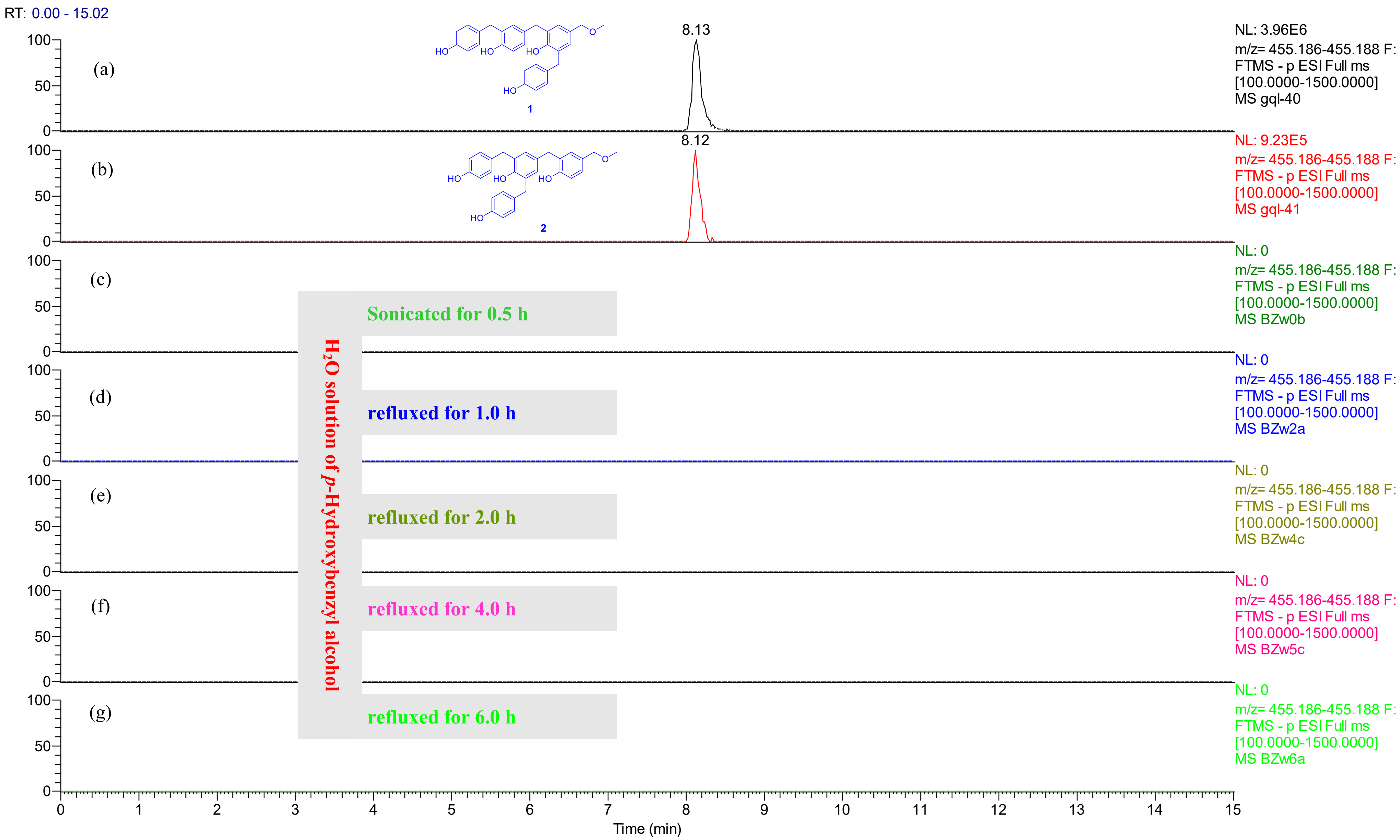

Figure S328. Overlaid chromatograms of the extracted negative ion at *m/z* 455.187 $[M–H]^-$: (a) and (b) compounds **1** and **2** in CH_3CN, respectively; (c)–(g) H_2O solution of *p*-hydroxybenzyl alcohol was sonicated for 0.5 h then refluxed for 1.0 h, 2.0 h, 4.0 h, and 6.0 h, respectively.

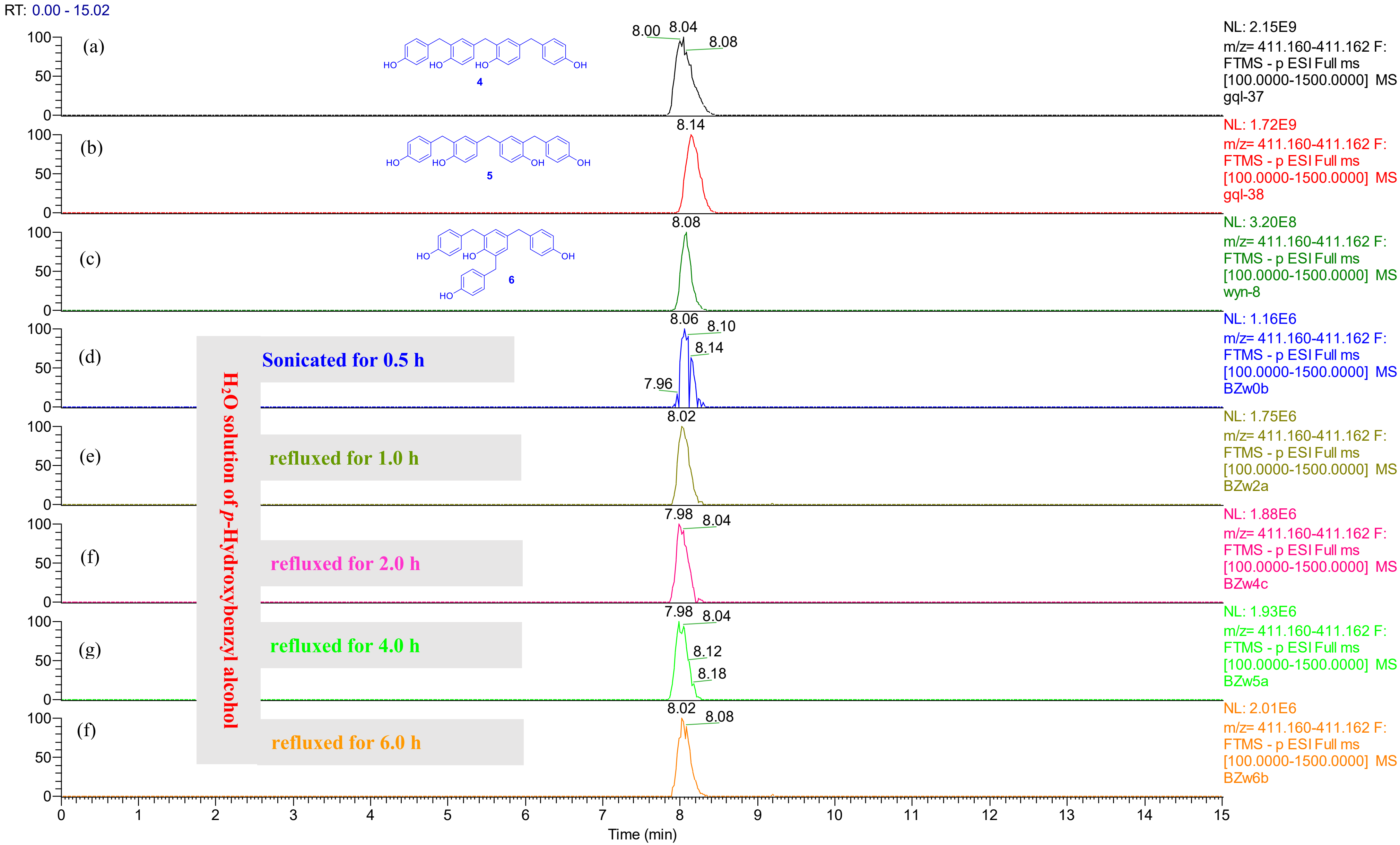

Figure S329. Overlaid chromatograms of the extracted negative ion at *m/z* 411.161 $[M{-}H]^-$: (a)–(c) compounds **4–6** in CH_3CN, respectively; (d)–(f) H_2O solution of *p*-hydroxybenzyl alcohol was sonicated for 0.5 h then refluxed for 1.0 h, 2.0 h, 4.0 h, and 6.0 h, respectively.

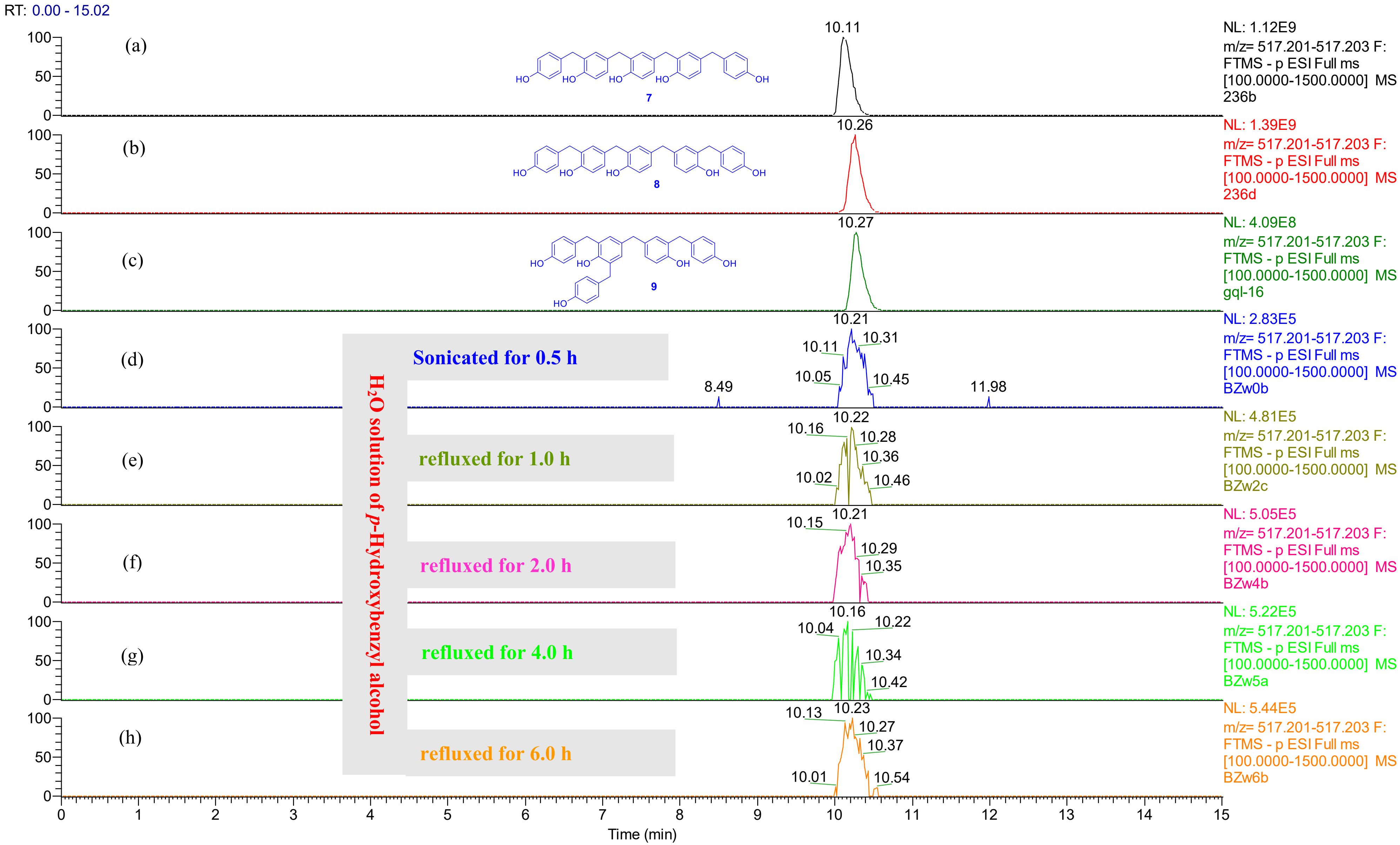

Figure S330. Overlaid chromatograms of the extracted negative ion at *m/z* 517.202 [M–H]$^-$: (a)–(c) compounds **7–9** in CH_3CN, respectively; (d)–(h) H_2O solution of *p*-hydroxybenzyl alcohol was sonicated for 0.5 h then refluxed for 1.0 h, 2.0 h, 4.0 h, and 6.0 h, respectively.

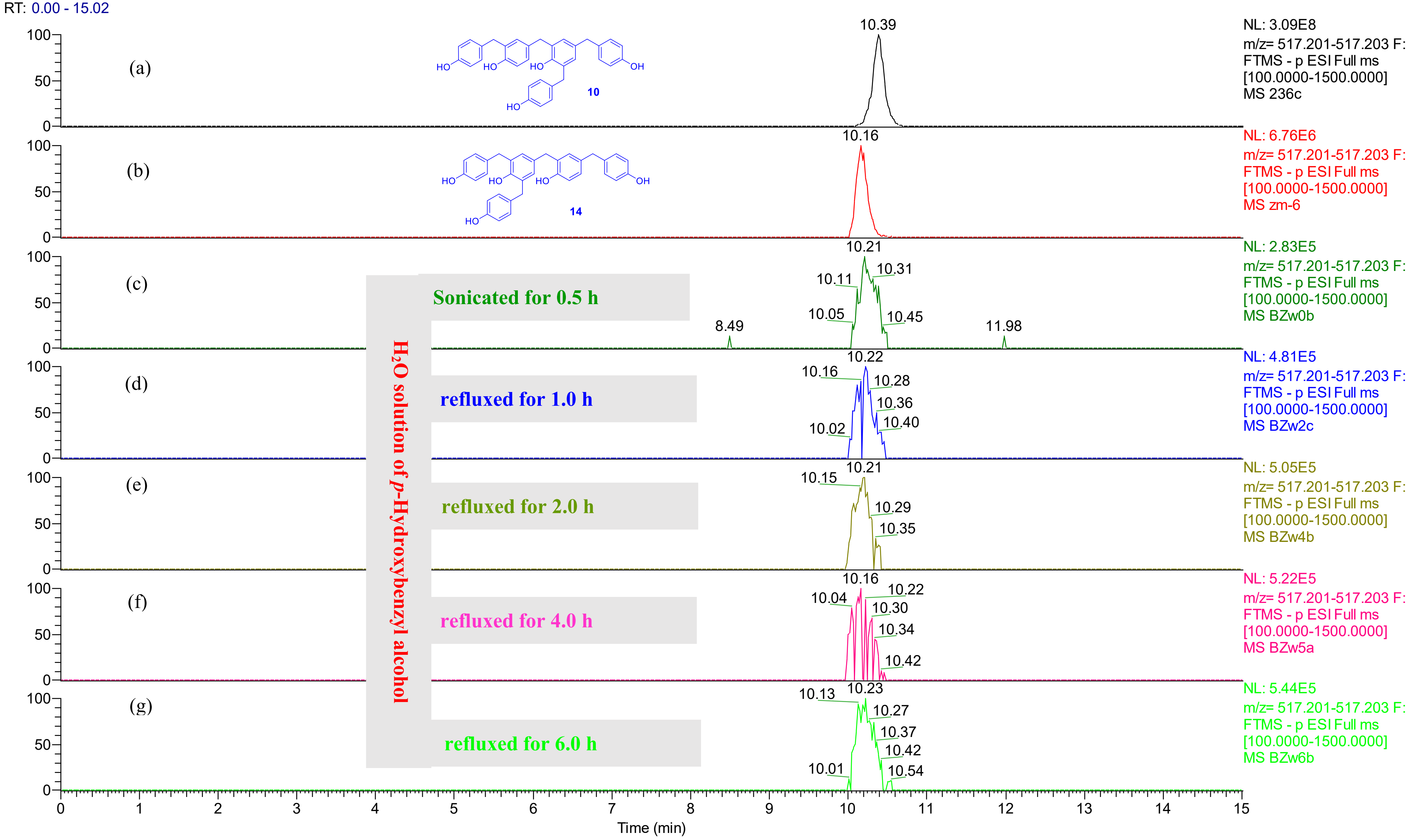

Figure S331. Overlaid chromatograms of the extracted negative ion at *m/z* 517.202 [M–H]⁻: (a) and (b) compounds **10** and **14** in CH_3CN, respectively; (c)–(g) H_2O solution of *p*-hydroxybenzyl alcohol was sonicated for 0.5 h then refluxed for 1.0 h, 2.0 h, 4.0 h, and 6.0 h, respectively.

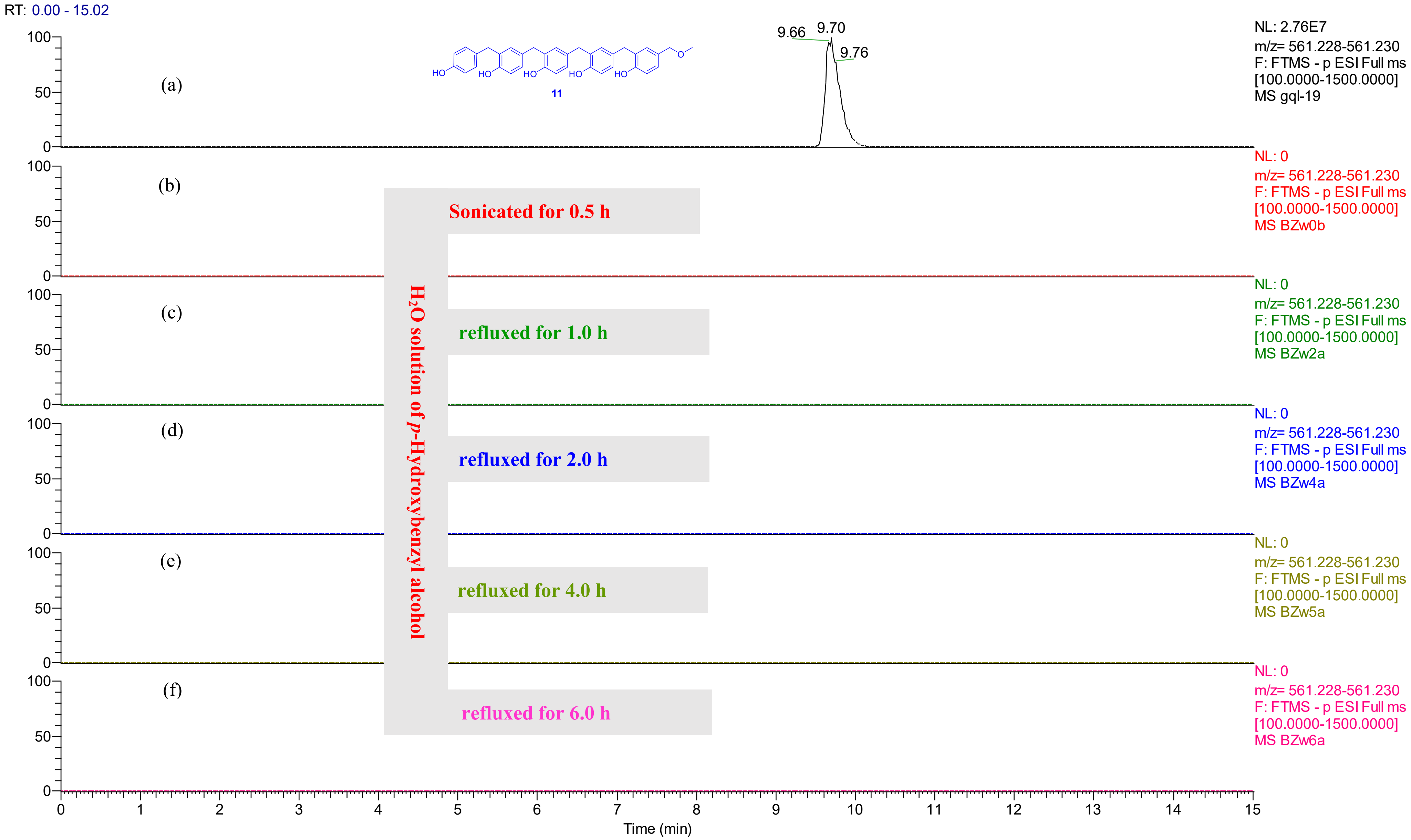

Figure S332. Overlaid chromatograms of the extracted negative ion at *m/z* 561.229 [M–H]⁻: (a) compound **11** in CH_3CN; (b)–(f) H_2O solution of *p*-hydroxybenzyl alcohol was sonicated for 0.5 h then refluxed for 1.0 h, 2.0 h, 4.0 h, and 6.0 h, respectively.

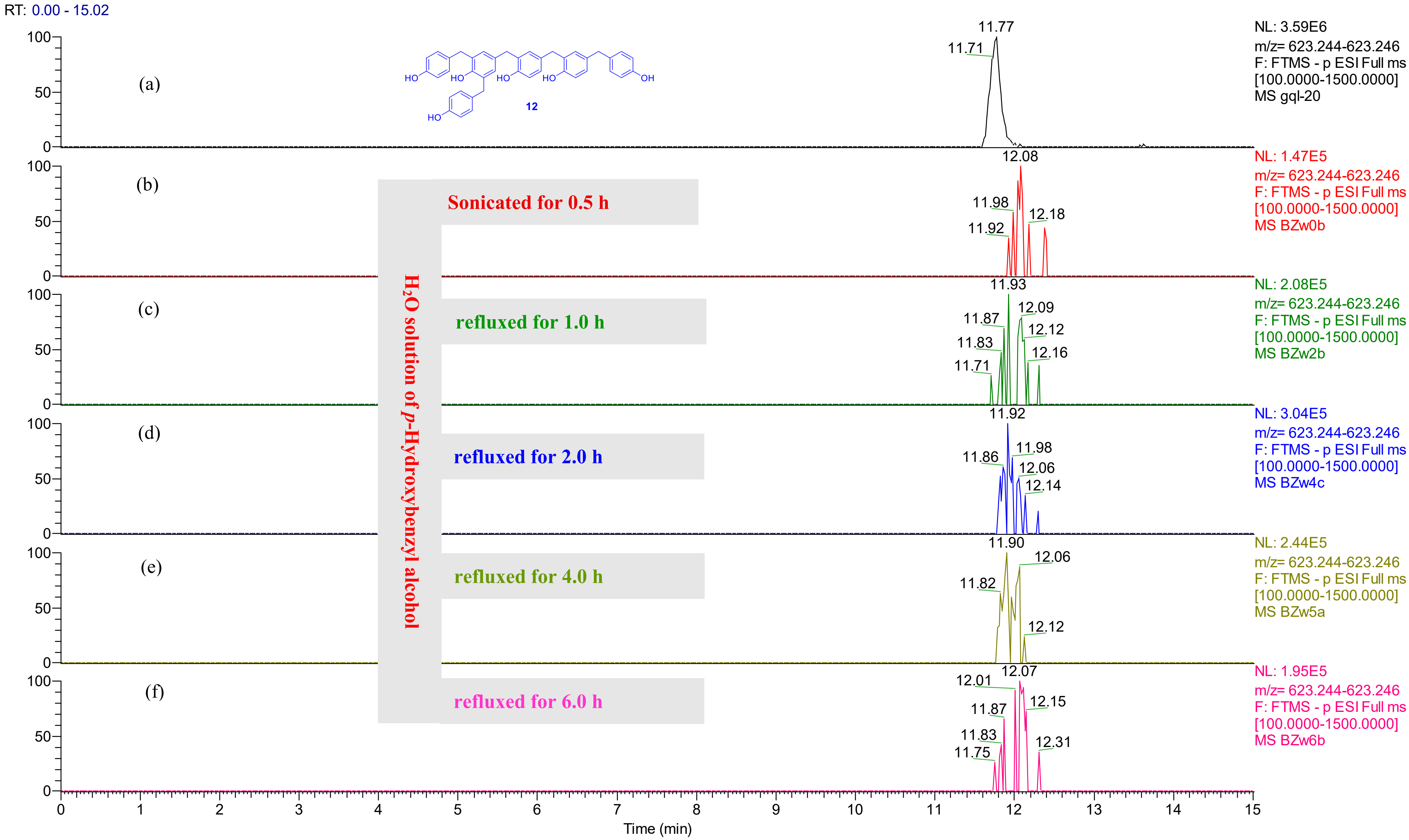

Figure S333. Overlaid chromatograms of the extracted negative ion at *m/z* 623.245 [M–H]⁻: (a) compound **12** in CH_3CN; (b)–(f) H_2O solution of *p*-hydroxybenzyl alcohol was sonicated for 0.5 h then refluxed for 1.0 h, 2.0 h, 4.0 h, and 6.0 h, respectively.

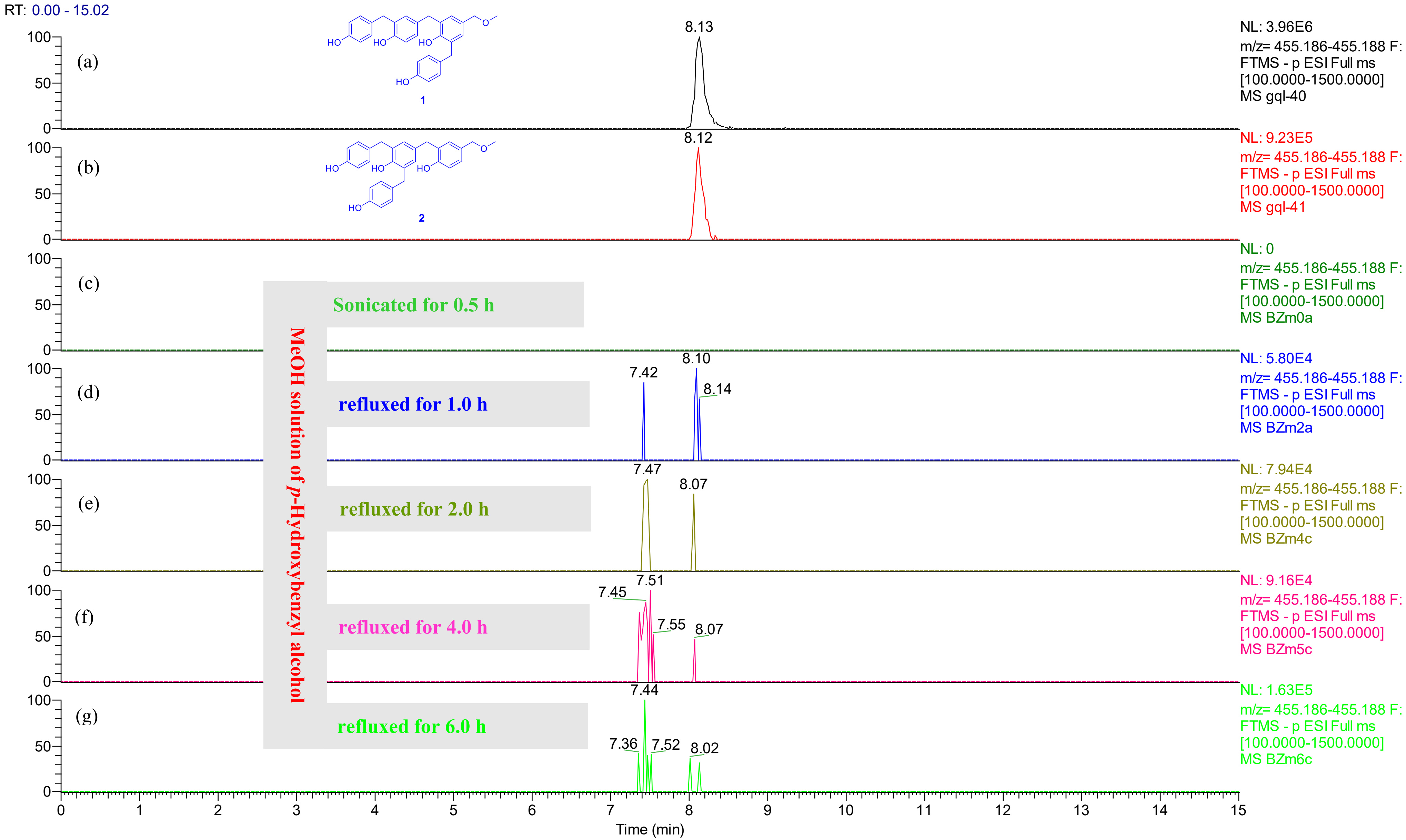

Figure S334. Overlaid chromatograms of the extracted negative ion at *m/z* 455.187 [M–H]$^-$: (a) and (b) compounds **1** and **2** in CH_3CN, respectively; (c)–(g) MeOH solution of *p*-hydroxybenzyl alcohol was sonicated for 0.5 h then refluxed for 1.0 h, 2.0 h, 4.0 h, and 6.0 h, respectively.

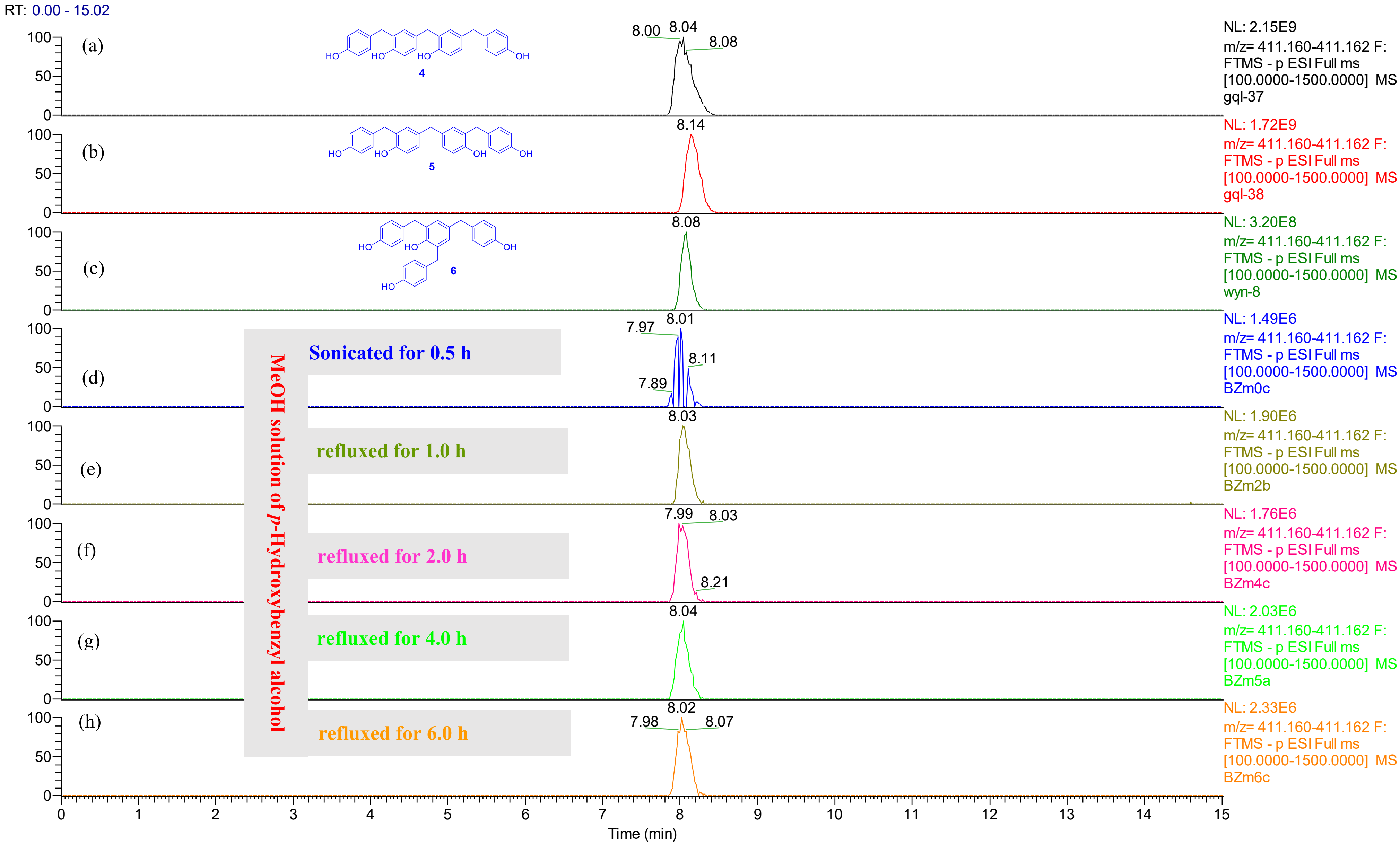

Figure S335. Overlaid chromatograms of the extracted negative ion at *m/z* 411.161 $[M–H]^-$: (a)–(c) compounds **4**–**6** in CH_3CN, respectively; (d)–(h) MeOH solution of *p*-hydroxybenzyl alcohol was sonicated for 0.5 h then refluxed for 1.0 h, 2.0 h, 4.0 h, and 6.0 h, respectively.

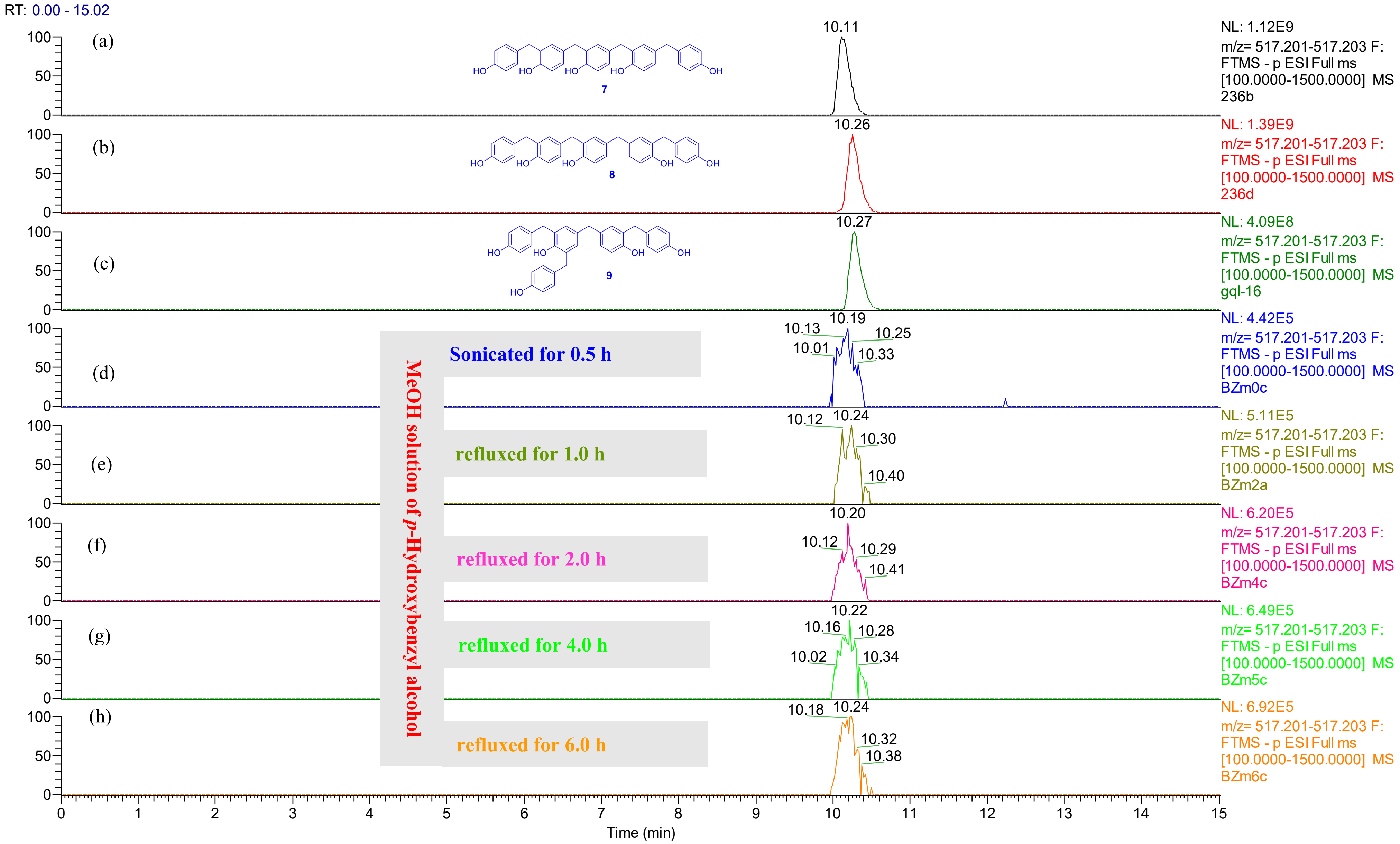

Figure S336. Overlaid chromatograms of the extracted negative ion at *m/z* 517.202 [M–H]⁻: (a)–(c) compounds **7**–**9** in CH_3CN, respectively; (d)–(h) MeOH solution of *p*-hydroxybenzyl alcohol was sonicated for 0.5 h then refluxed for 1.0 h, 2.0 h, 4.0 h, and 6.0 h, respectively.

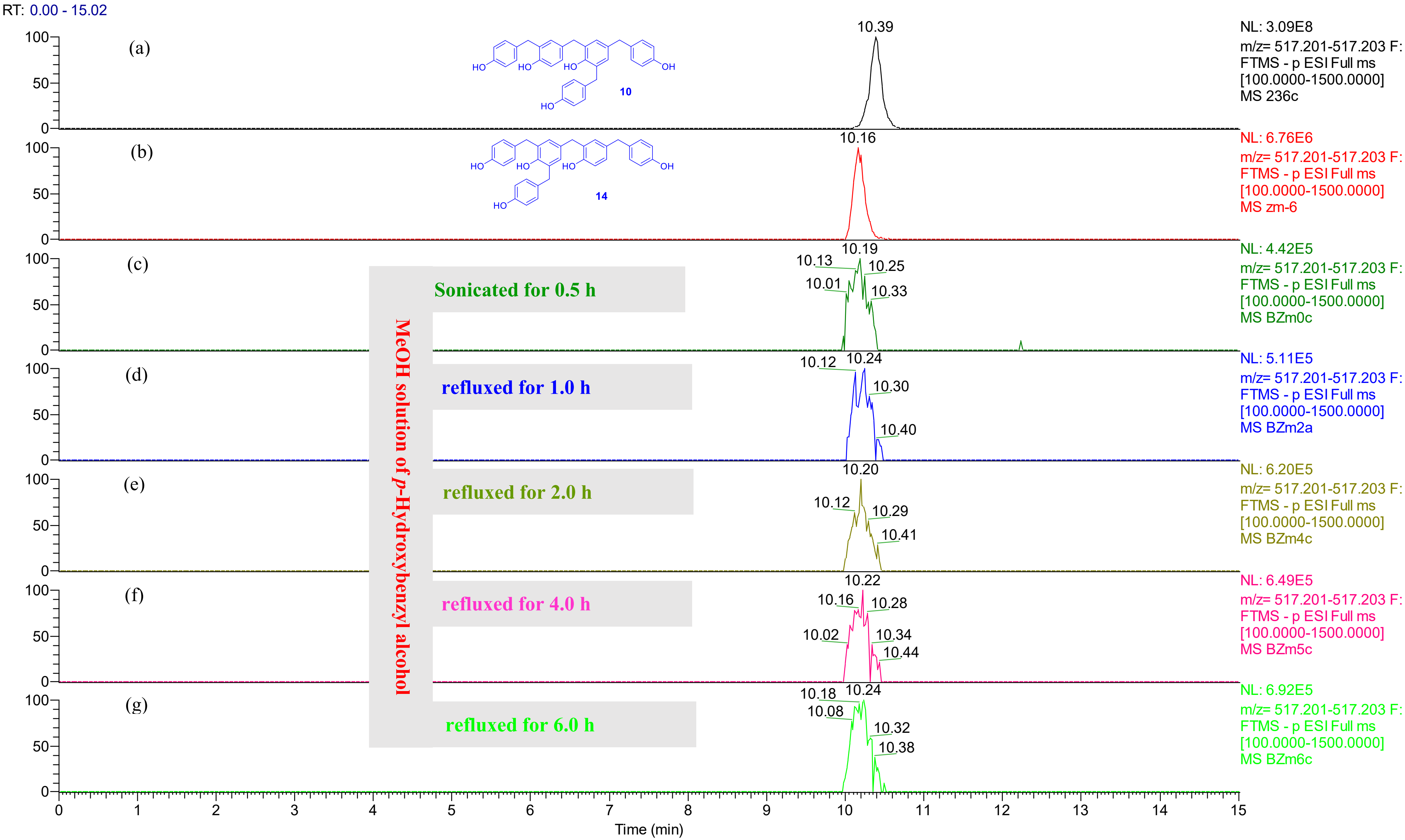

Figure S337. Overlaid chromatograms of the extracted negative ion at *m/z* 517.202 [M–H]⁻: (a) and (b) compounds **10** and **14** in CH_3CN, respectively; (c)–(g) MeOH solution of *p*-hydroxybenzyl alcohol was sonicated for 0.5 h then refluxed for 1.0 h, 2.0 h, 4.0 h, and 6.0 h, respectively.

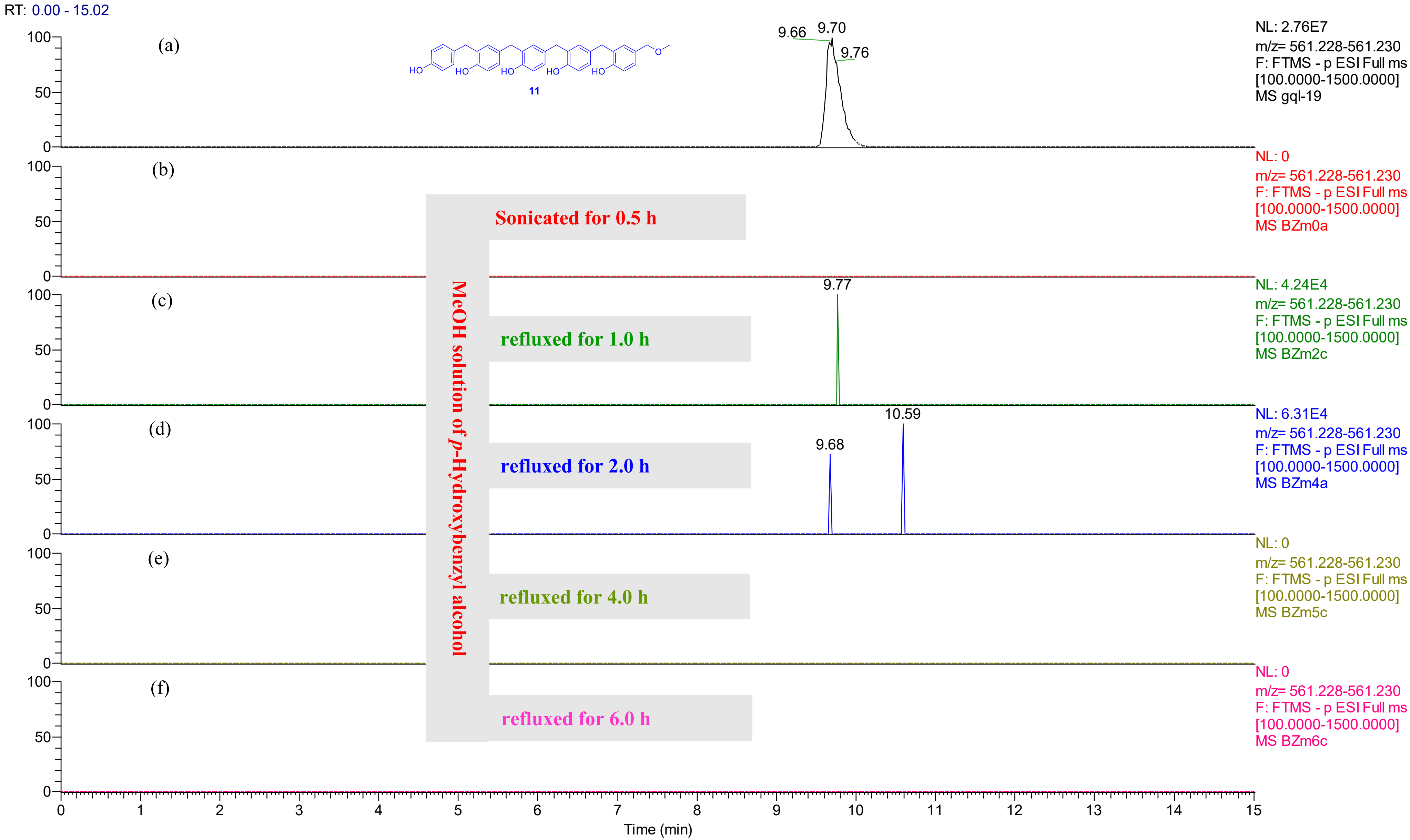

Figure S338. Overlaid chromatograms of the extracted negative ion at *m/z* 561.229 [M–H]⁻: (a) compound **11** in CH_3CN; (b)–(f) MeOH solution of *p*-hydroxybenzyl alcohol was sonicated for 0.5 h then refluxed for 1.0 h, 2.0 h, 4.0 h, and 6.0 h, respectively.

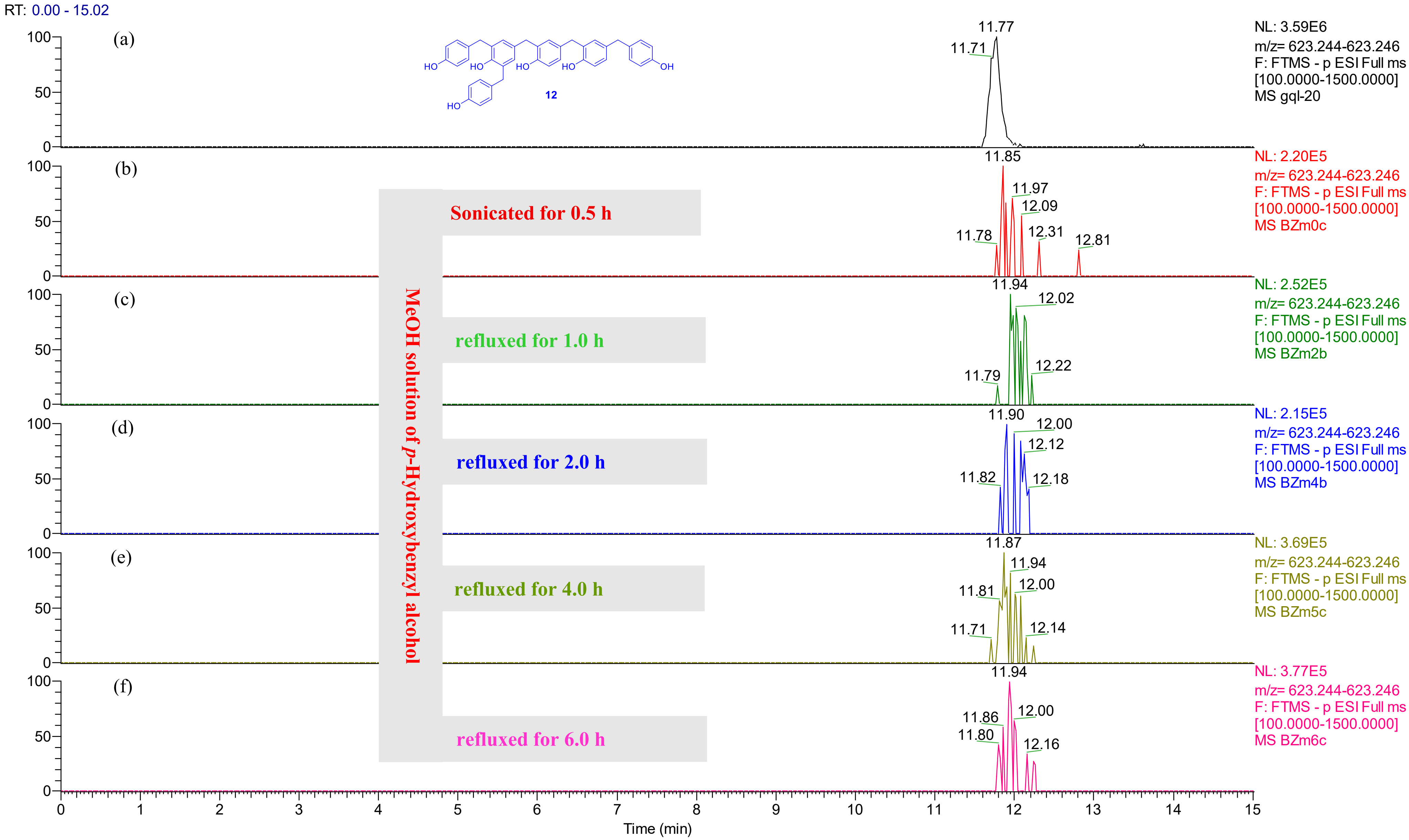

Figure S339. Overlaid chromatograms of the extracted negative ion at *m/z* 623.245 [M–H]⁻: (a) compound **12** in CH_3CN; (b)–(f) MeOH solution of *p*-hydroxybenzyl alcohol was sonicated for 0.5 h then refluxed for 1.0 h, 2.0 h, 4.0 h, and 6.0 h, respectively.

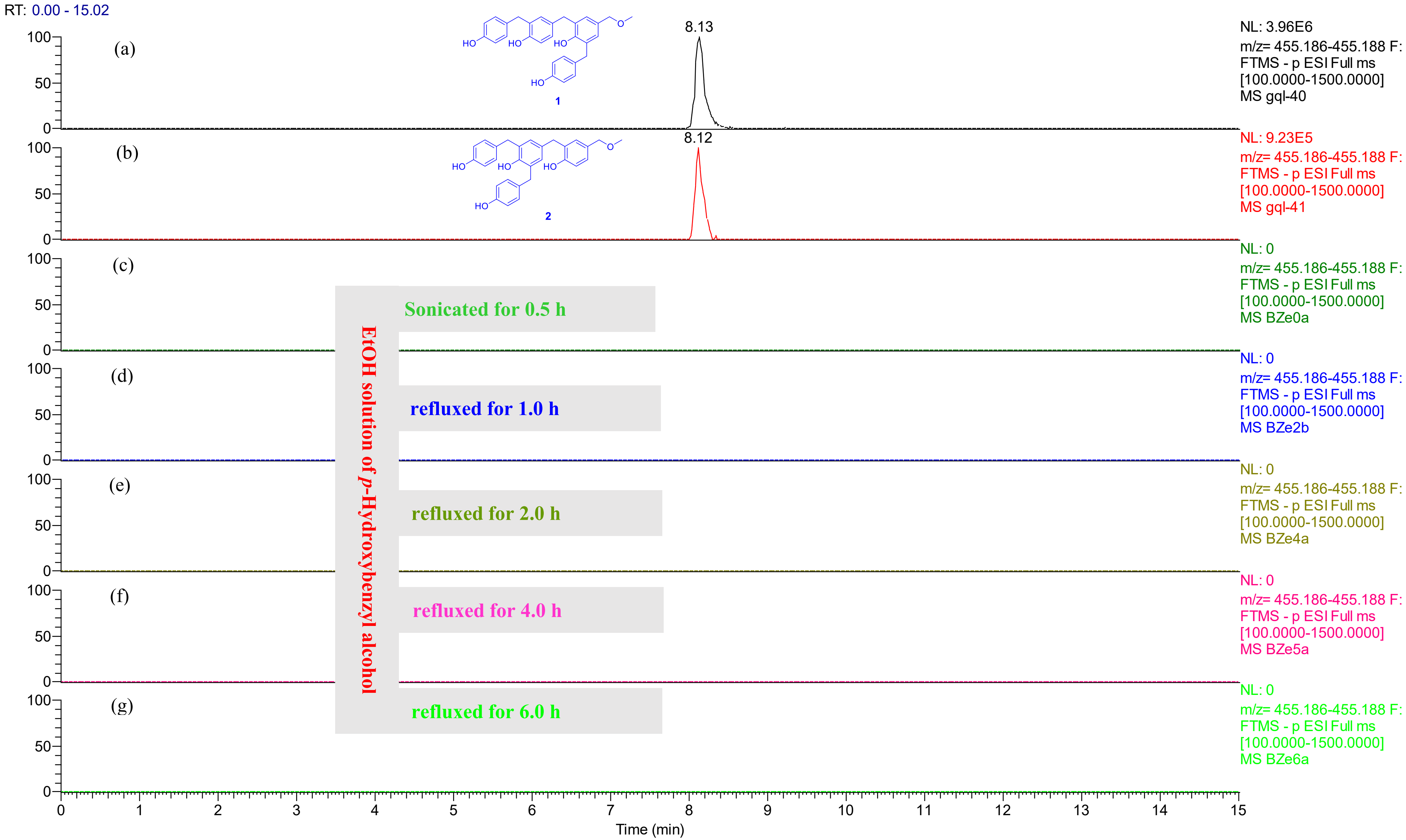

Figure S340. Overlaid chromatograms of the extracted negative ion at *m/z* 455.187 [M–H]⁻: (a) and (b) compounds **1** and **2** in CH_3CN, respectively; (c)–(g) EtOH solution of *p*-hydroxybenzyl alcohol was sonicated for 0.5 h then refluxed for 1.0 h, 2.0 h, 4.0 h, and 6.0 h, respectively.

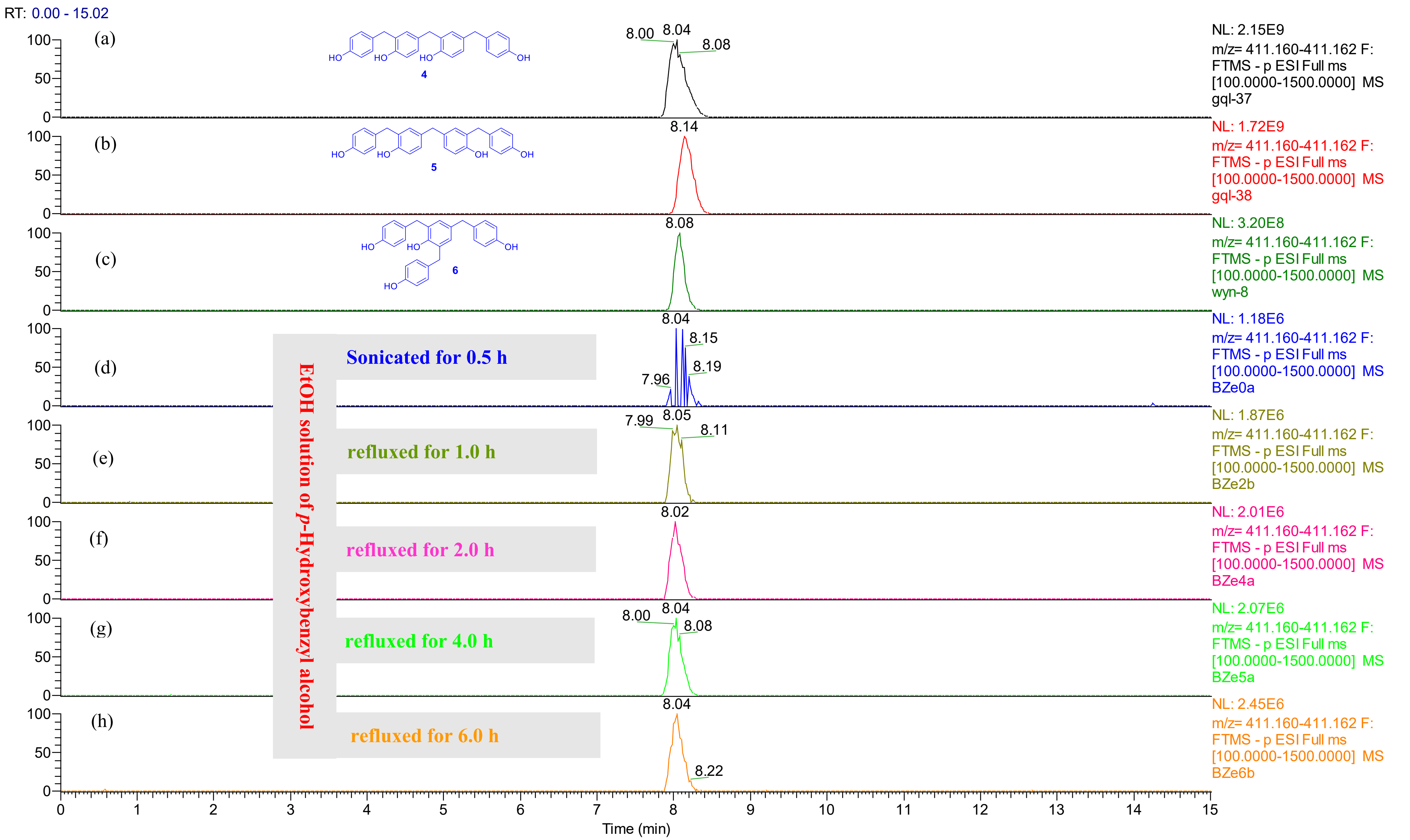

Figure S341. Overlaid chromatograms of the extracted negative ion at *m/z* 411.161 [M–H]$^-$: (a)–(c) compounds **4**–**6** in CH_3CN, respectively; (d)–(h) EtOH solution of *p*-hydroxybenzyl alcohol was sonicated for 0.5 h then refluxed for 1.0 h, 2.0 h, 4.0 h, and 6.0 h, respectively.

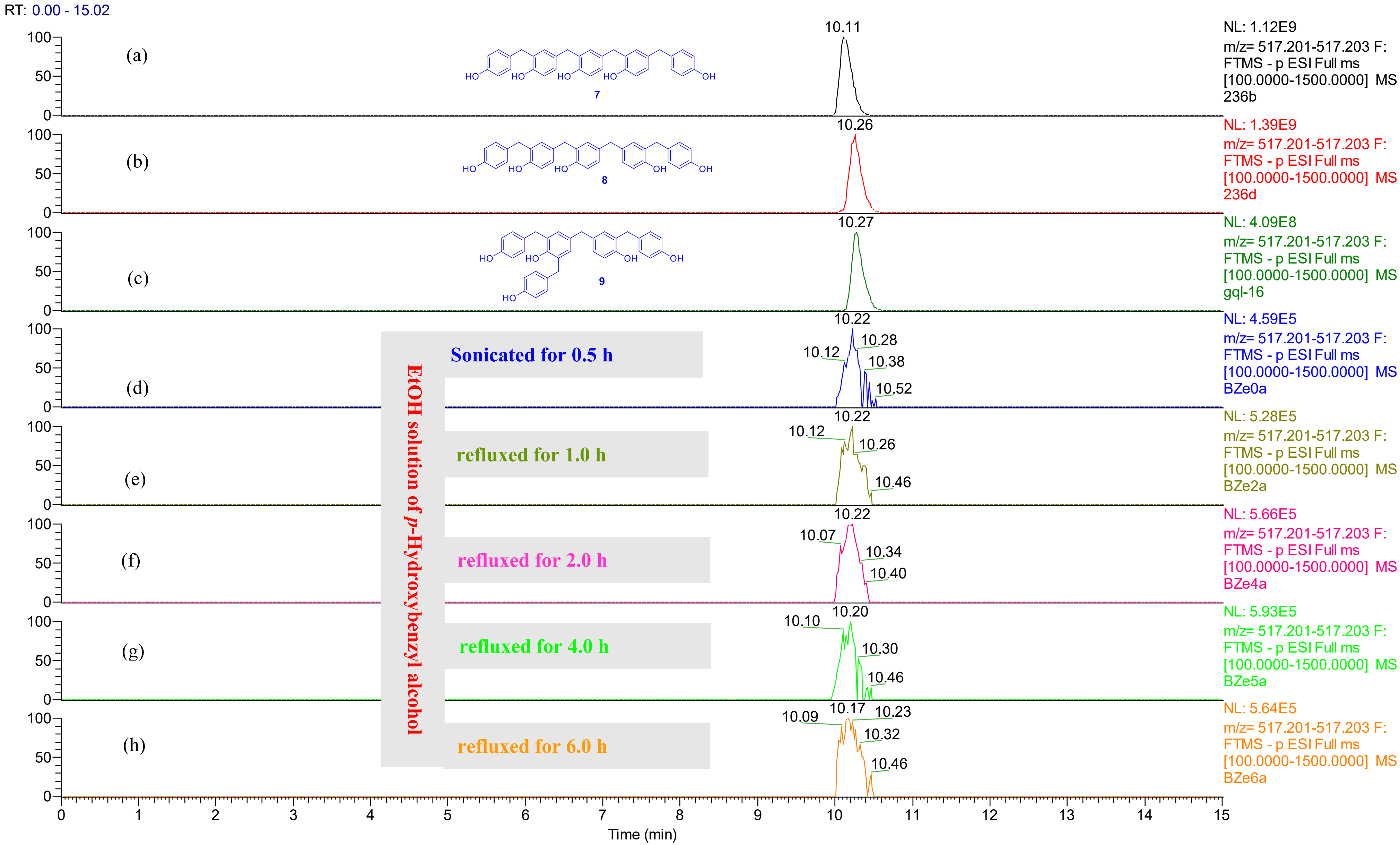

Figure S342. Overlaid chromatograms of the extracted negative ion at *m/z* 517.202 [M–H]$^-$: (a)–(c) compounds **7**–**9** in CH_3CN, respectively; (d)–(h) EtOH solution of *p*-hydroxybenzyl alcohol was sonicated for 0.5 h then refluxed for 1.0 h, 2.0 h, 4.0 h, and 6.0 h, respectively.

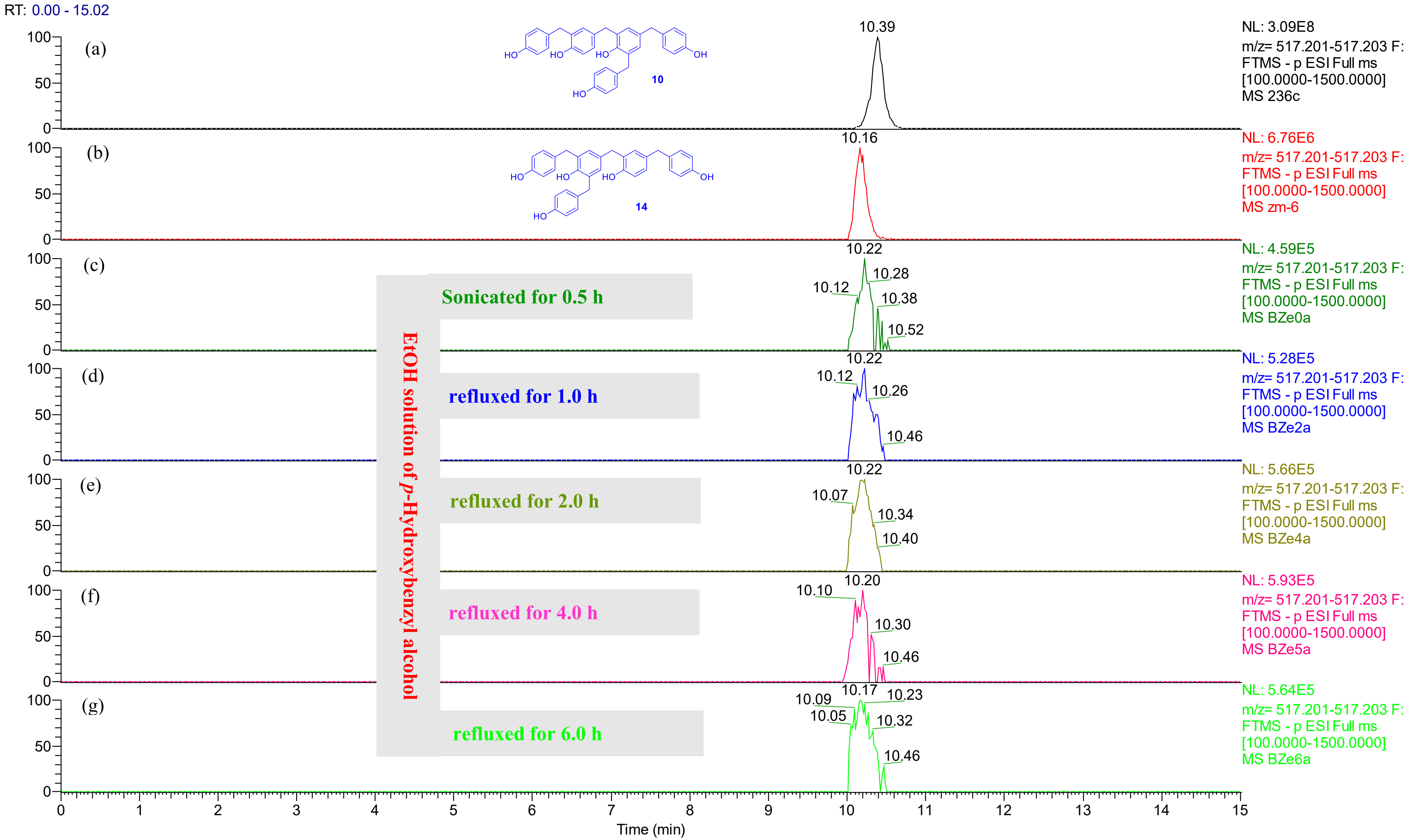

Figure S343. Overlaid chromatograms of the extracted negative ion at *m/z* 517.202 [M–H]⁻: (a) and (b) compounds **10** and **14** in CH_3CN, respectively; (c)–(g) EtOH solution of *p*-hydroxybenzyl alcohol was sonicated for 0.5 h then refluxed for 1.0 h, 2.0 h, 4.0 h, and 6.0 h, respectively.

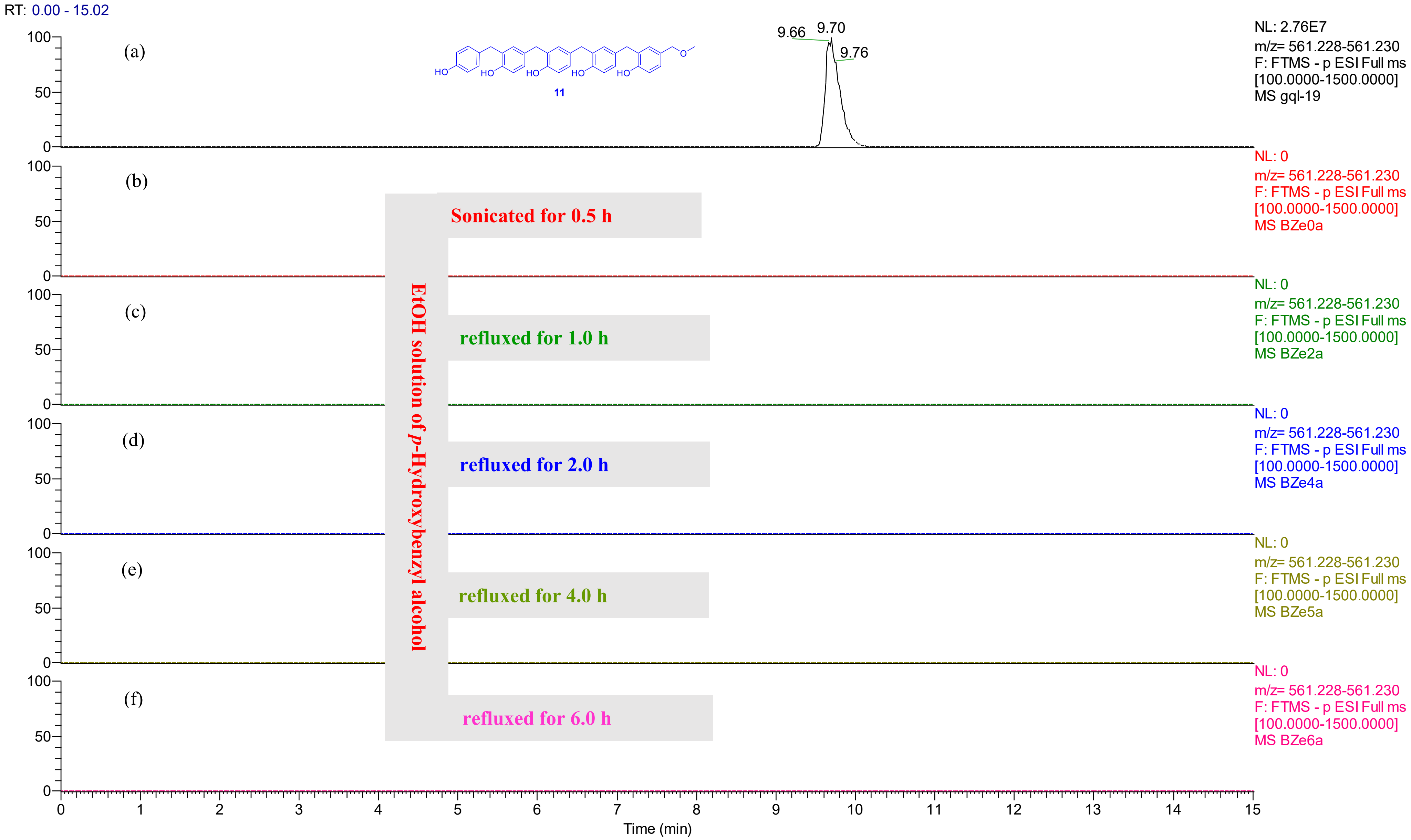

Figure S344. Overlaid chromatograms of the extracted negative ion at *m/z* 561.229 $[M–H]^-$: (a) compound **11** in CH_3CN; (b)–(f) EtOH solution of *p*-hydroxybenzyl alcohol was sonicated for 0.5 h then refluxed for 1.0 h, 2.0 h, 4.0 h, and 6.0 h, respectively.

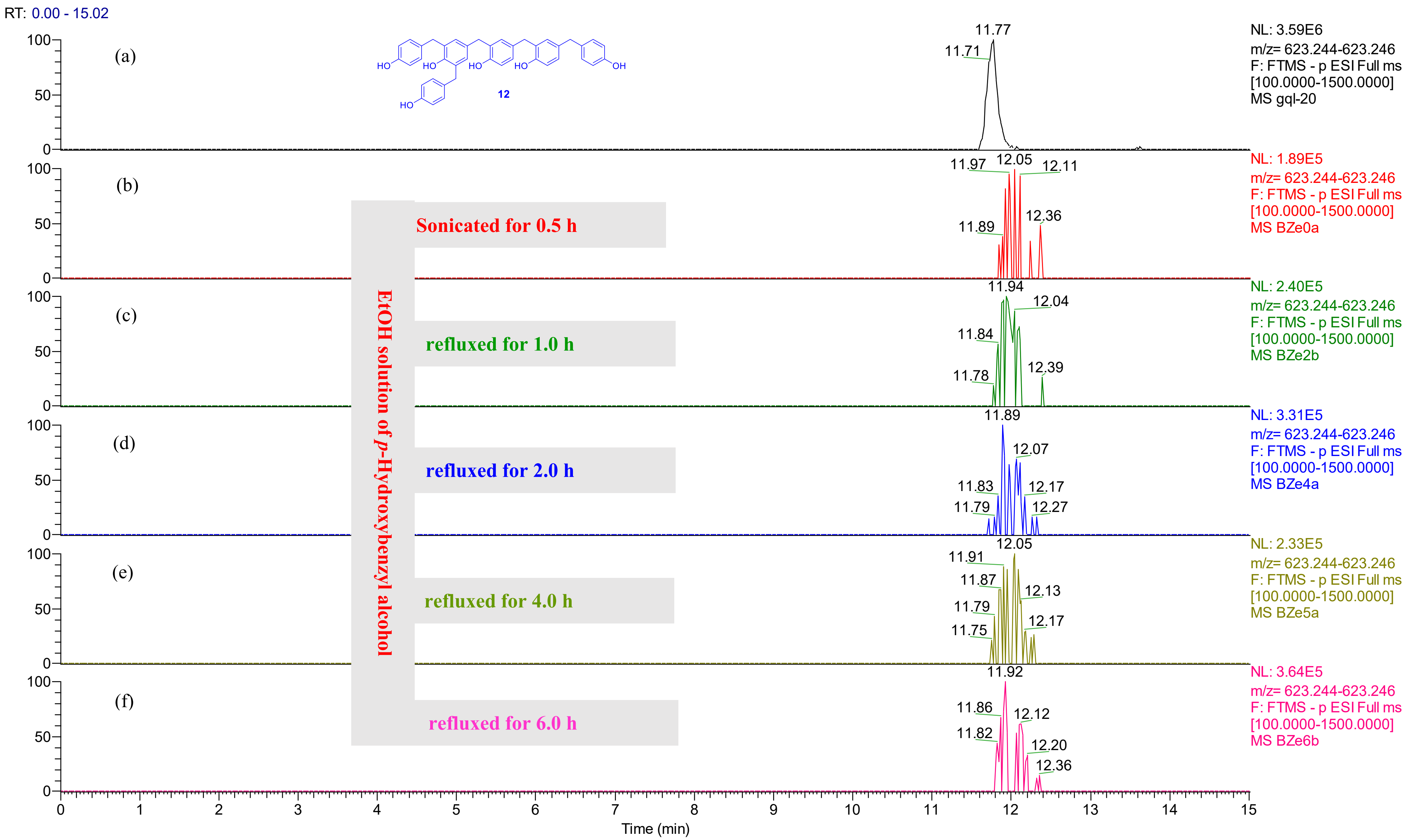

Figure S345. Overlaid chromatograms of the extracted negative ion at *m/z* 623.245 [M–H]⁻: (a) compound **12** in CH_3CN; (b)–(f) EtOH solution of *p*-hydroxybenzyl alcohol was sonicated for 0.5 h then refluxed for 1.0 h, 2.0 h, 4.0 h, and 6.0 h, respectively.

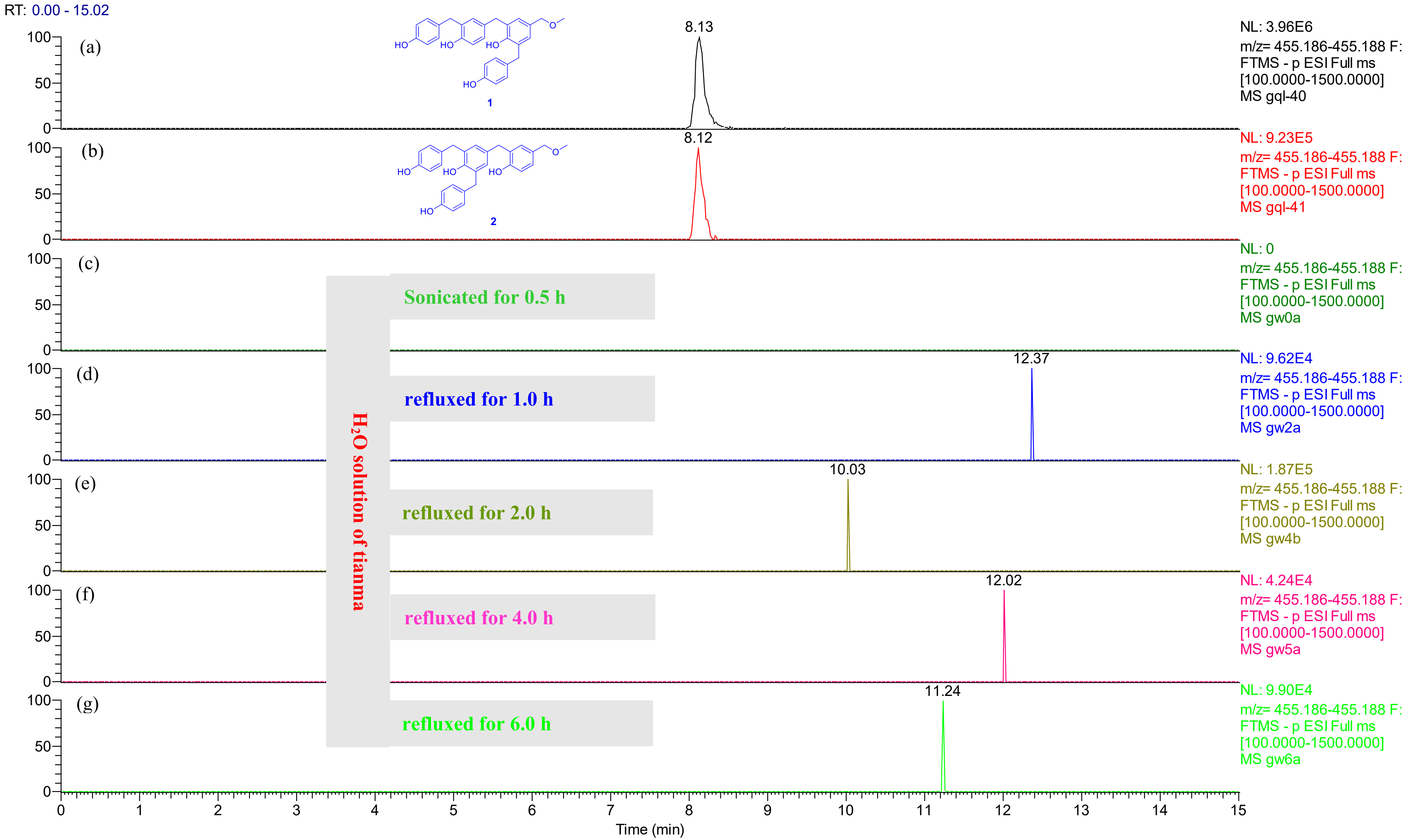

Figure S346. Overlaid chromatograms of the extracted negative ion at *m/z* 455.187 $[M–H]^-$: (a) and (b) compounds **1** and **2** in CH_3CN, respectively; (c)–(g) H_2O solution of “tianma” was sonicated for 0.5 h then refluxed for 1.0 h, 2.0 h, 4.0 h, and 6.0 h, respectively.

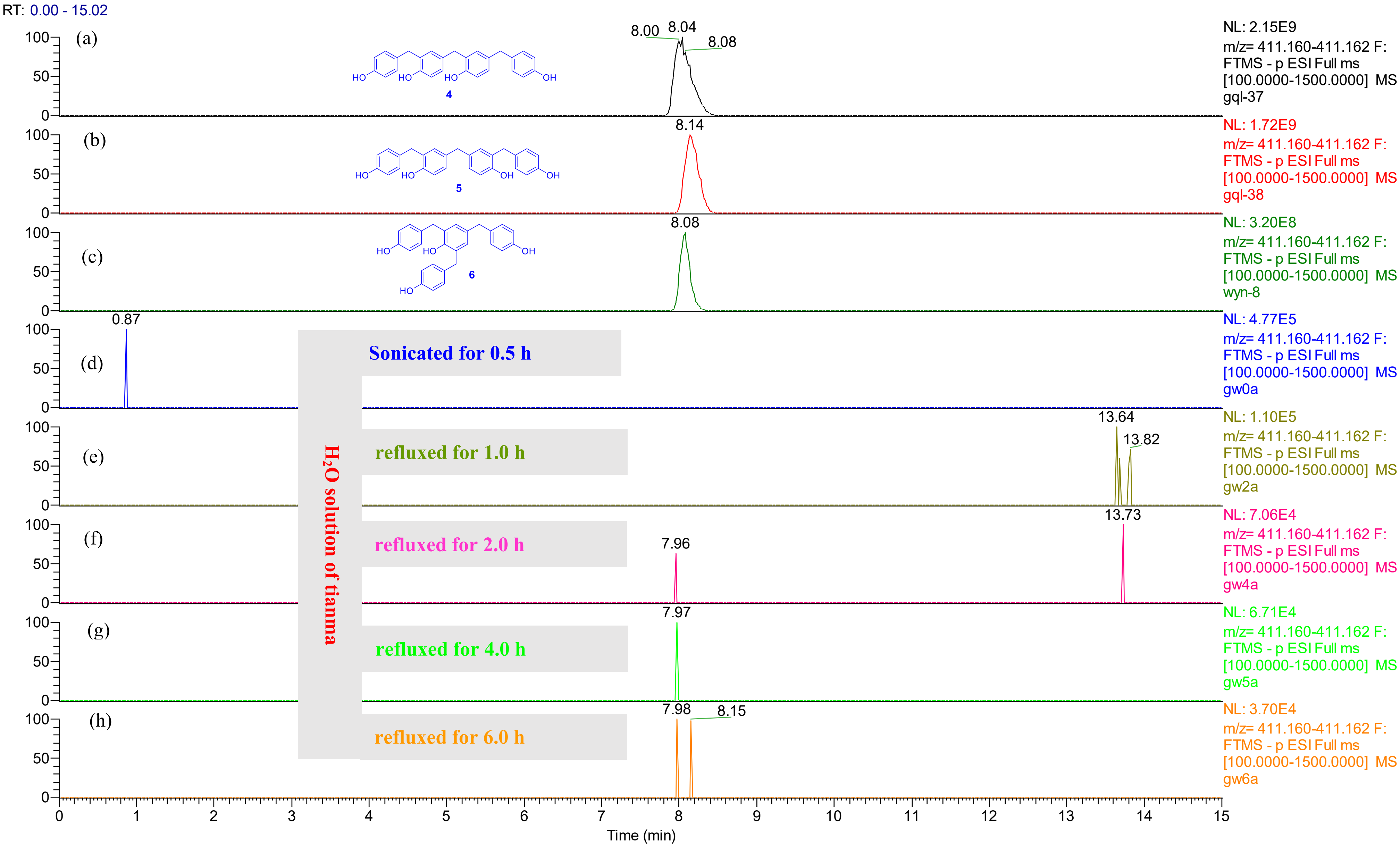

Figure S347. Overlaid chromatograms of the extracted negative ion at *m/z* 411.161 $[M–H]^-$: (a)–(c) compounds **4–6** in CH_3CN, respectively; (d)–(h) H_2O solution of "tianma" was sonicated for 0.5 h then refluxed for 1.0 h, 2.0 h, 4.0 h, and 6.0 h, respectively.

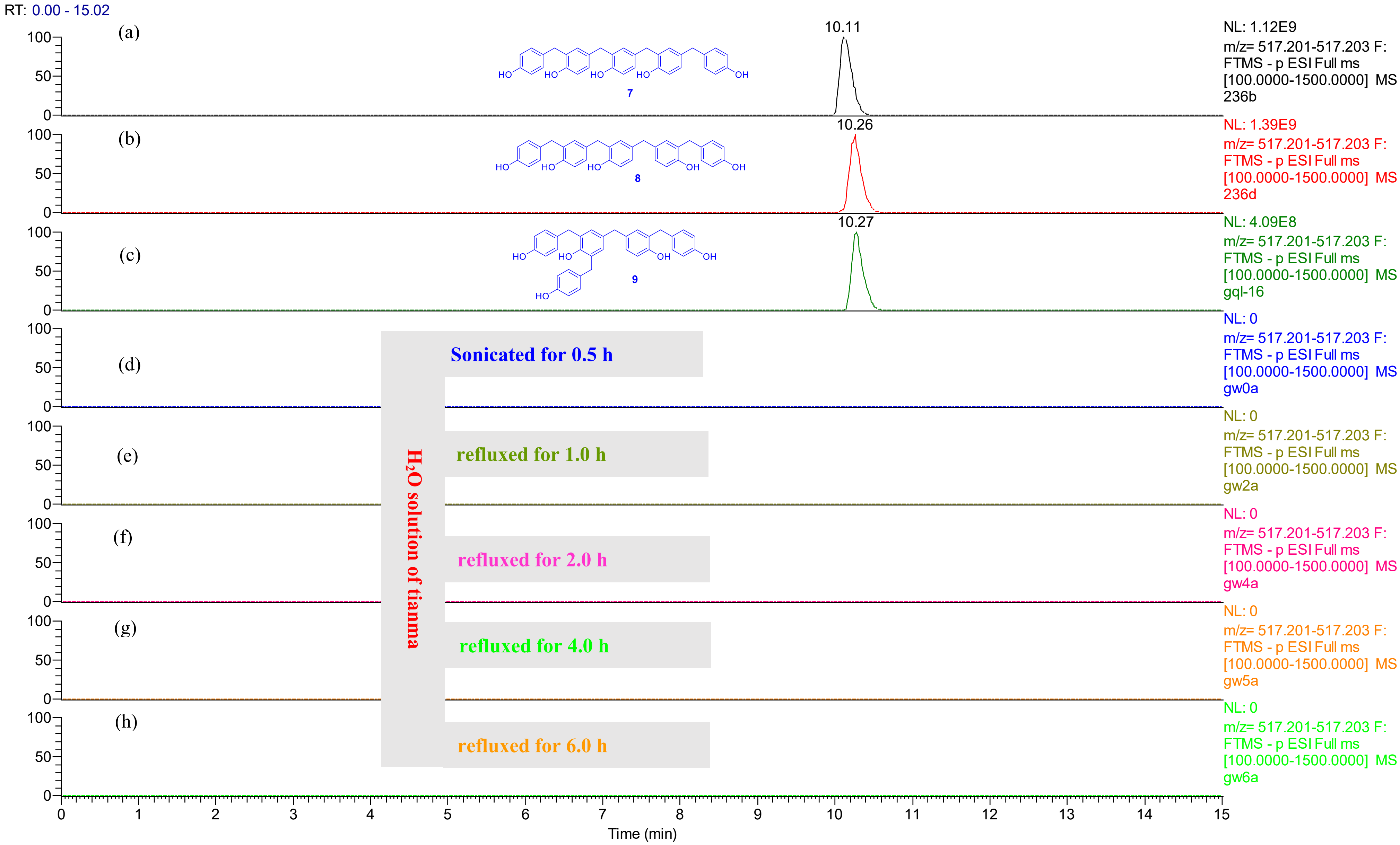

Figure S348. Overlaid chromatograms of the extracted negative ion at *m/z* 517.202 $[M-H]^-$: (a)–(c) compounds **7–9** in CH_3CN, respectively; (d)–(h) H_2O solution of "tianma" was sonicated for 0.5 h then refluxed for 1.0 h, 2.0 h, 4.0 h, and 6.0 h, respectively.

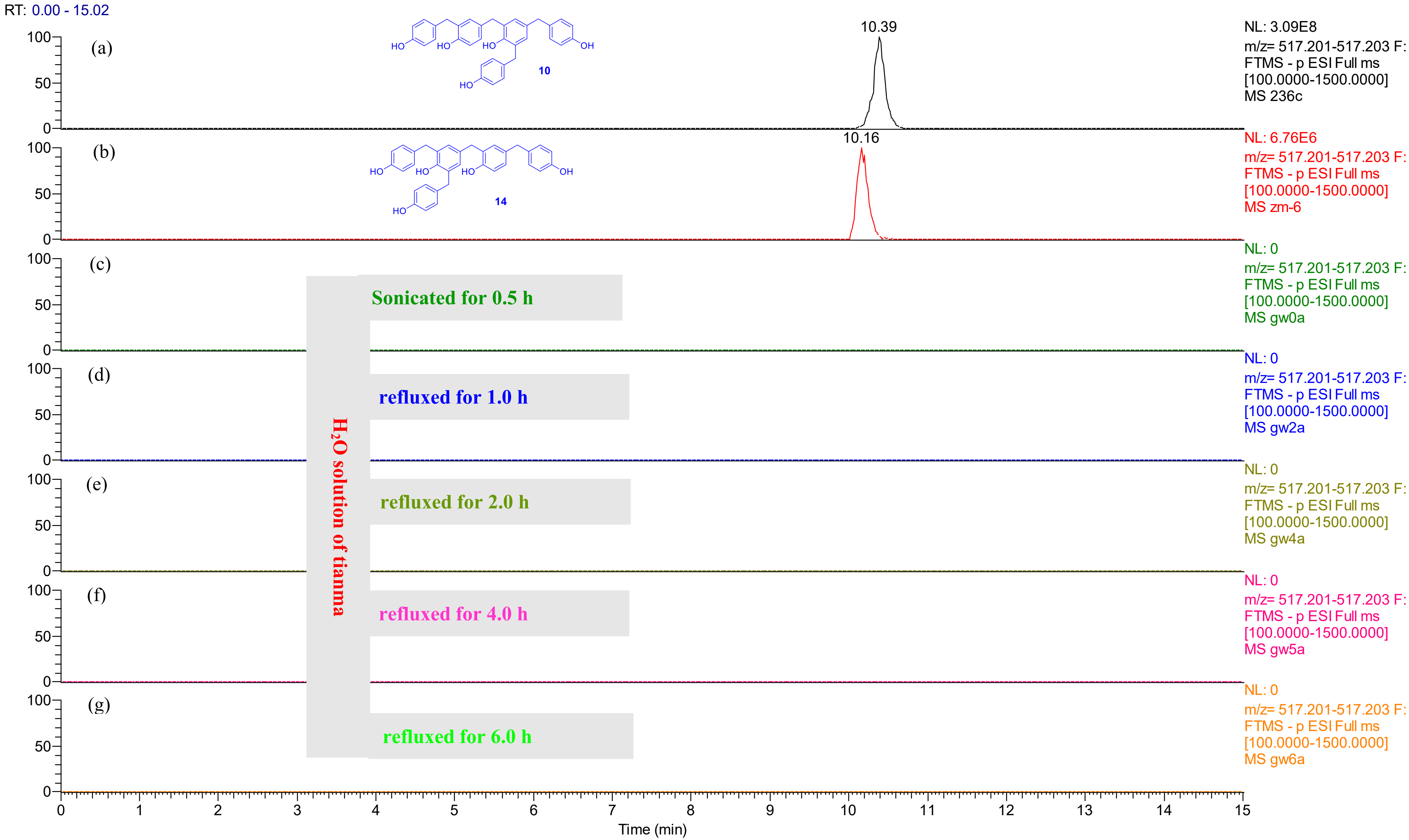

Figure S349. Overlaid chromatograms of the extracted negative ion at *m/z* 517.202 $[M-H]^-$: (a) and (b) compounds **10** and **14** in CH_3CN, respectively; (c)–(g) H_2O solution of "tianma" was sonicated for 0.5 h then refluxed for 1.0 h, 2.0 h, 4.0 h, and 6.0 h, respectively.

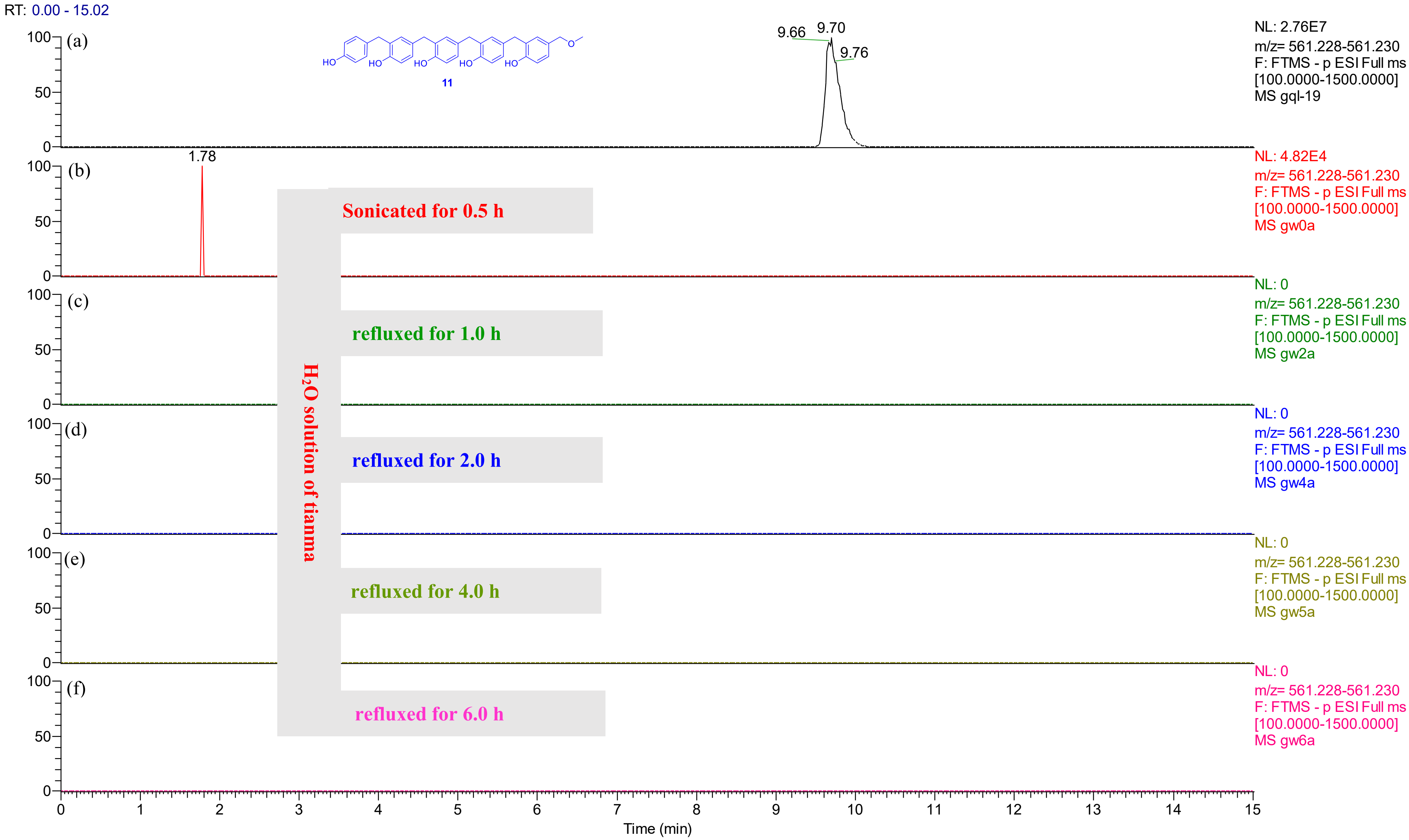

Figure S350. Overlaid chromatograms of the extracted negative ion at *m/z* 561.229 $[M–H]^-$: (a) compound **11** in CH_3CN; (b)–(f) H_2O solution of "tianma" was sonicated for 0.5 h then refluxed for 1.0 h, 2.0 h, 4.0 h, and 6.0 h, respectively.

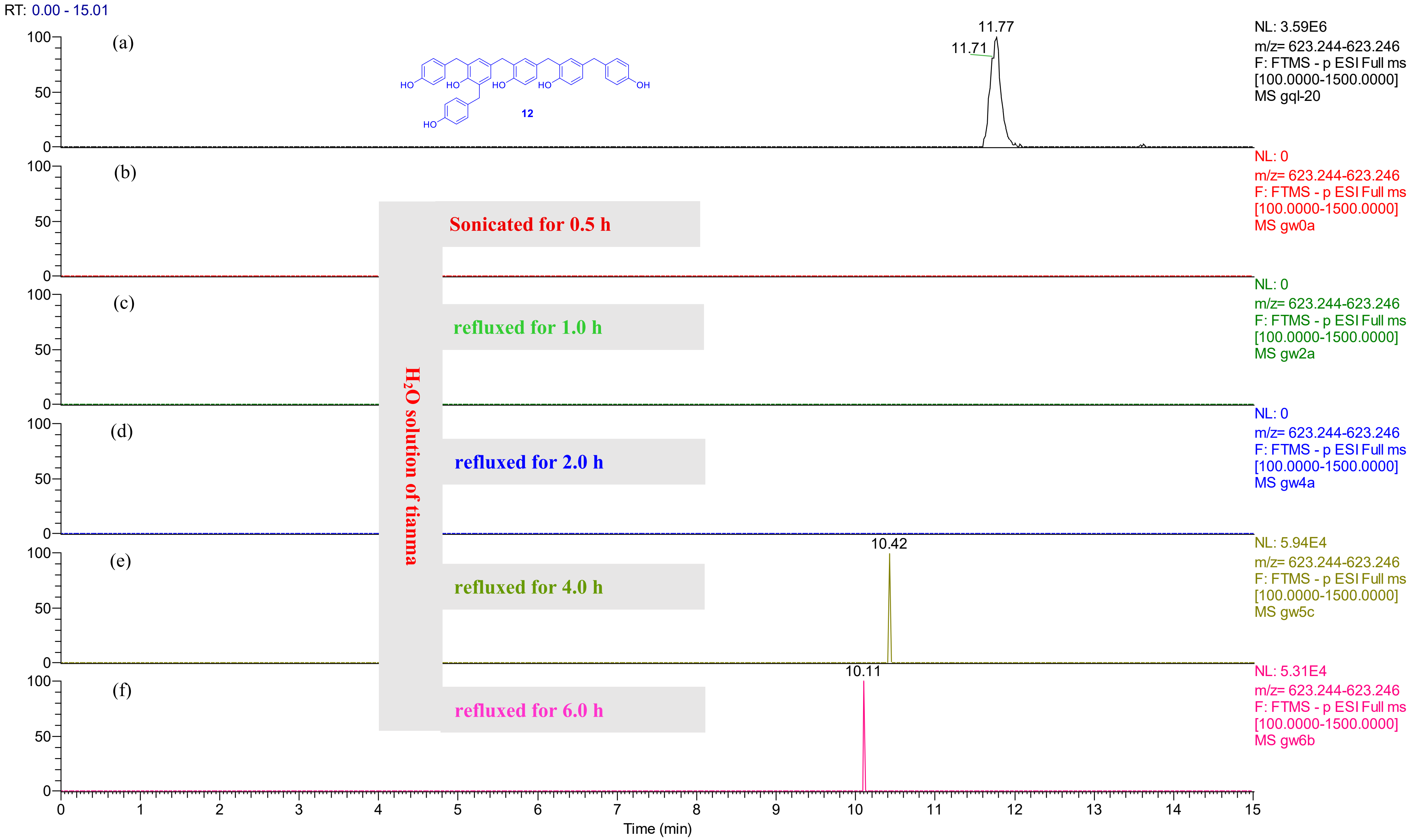

Figure S351. Overlaid chromatograms of the extracted negative ion at *m/z* 623.245 $[M-H]^-$: (a) compound **12** in CH_3CN; (b)–(f) H_2O solution of "tianma" was sonicated for 0.5 h then refluxed for 1.0 h, 2.0 h, 4.0 h, and 6.0 h, respectively.

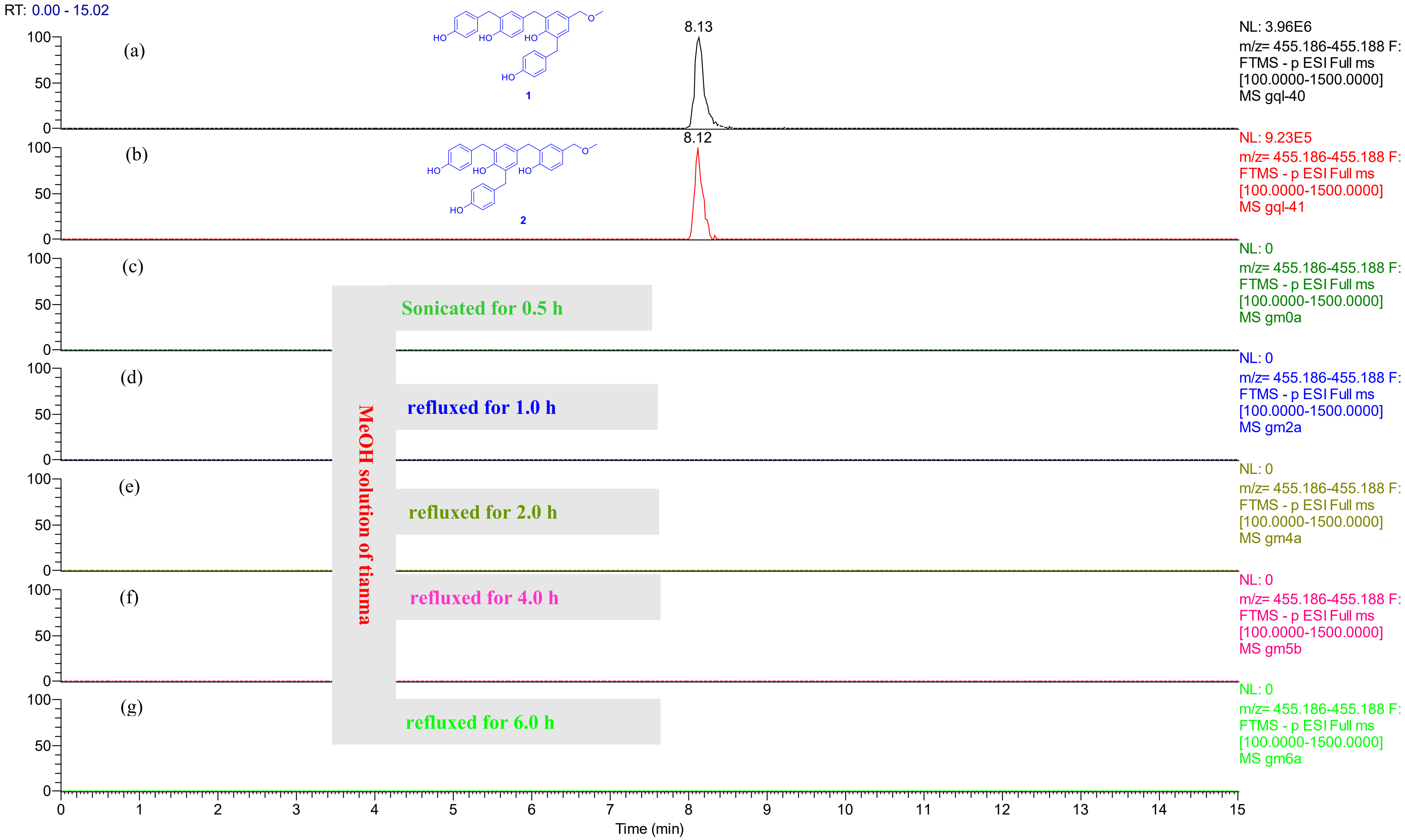

Figure S352. Overlaid chromatograms of the extracted negative ion at *m/z* 455.187 $[M–H]^-$: (a) and (b) compounds **1** and **2** in CH_3CN, respectively; (c)–(g) MeOH solution of "tianma" was sonicated for 0.5 h then refluxed for 1.0 h, 2.0 h, 4.0 h, and 6.0 h, respectively.

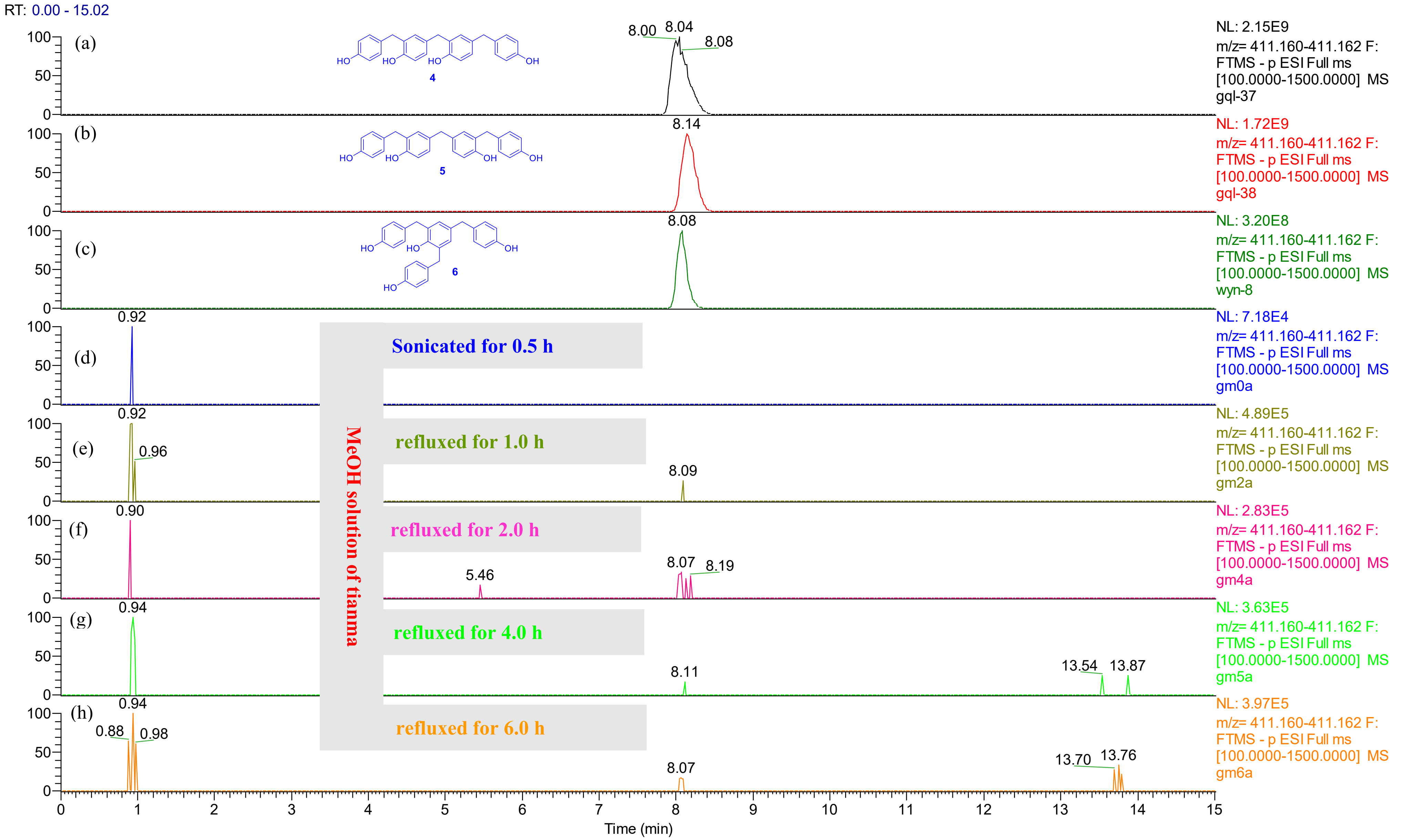

Figure S353. Overlaid chromatograms of the extracted negative ion at *m/z* 411.161 $[M-H]^-$: (a)−(c) compounds **4−6** in CH_3CN, respectively; (d)–(h) MeOH solution of “tianma” was sonicated for 0.5 h then refluxed for 1.0 h, 2.0 h, 4.0 h, and 6.0 h, respectively.

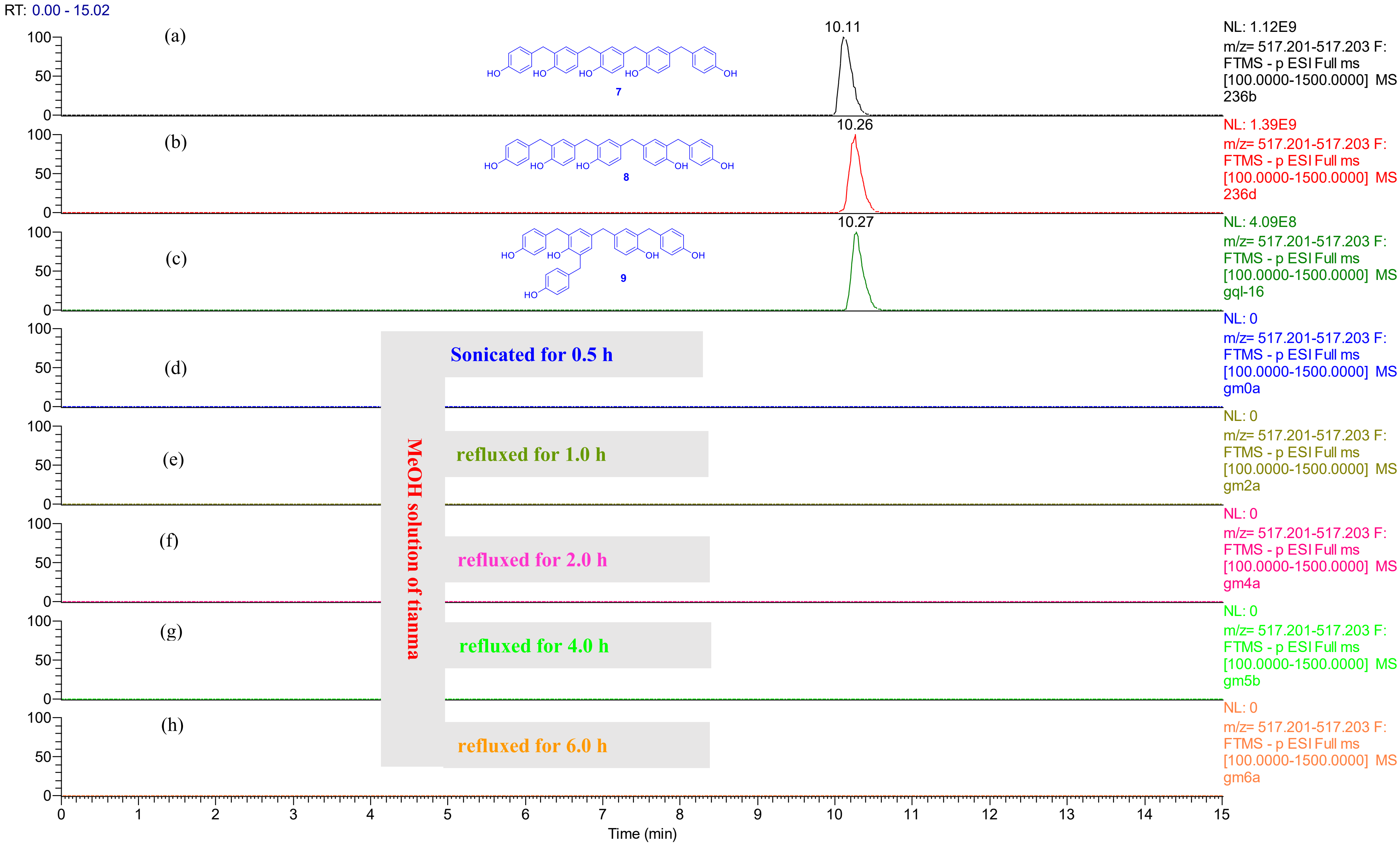

Figure S354. Overlaid chromatograms of the extracted negative ion at *m/z* 517.202 $[M-H]^-$: (a)−(c) compounds **7−9** in CH_3CN, respectively; (d)–(h) MeOH solution of "tianma" was sonicated for 0.5 h then refluxed for 1.0 h, 2.0 h, 4.0 h, and 6.0 h, respectively.

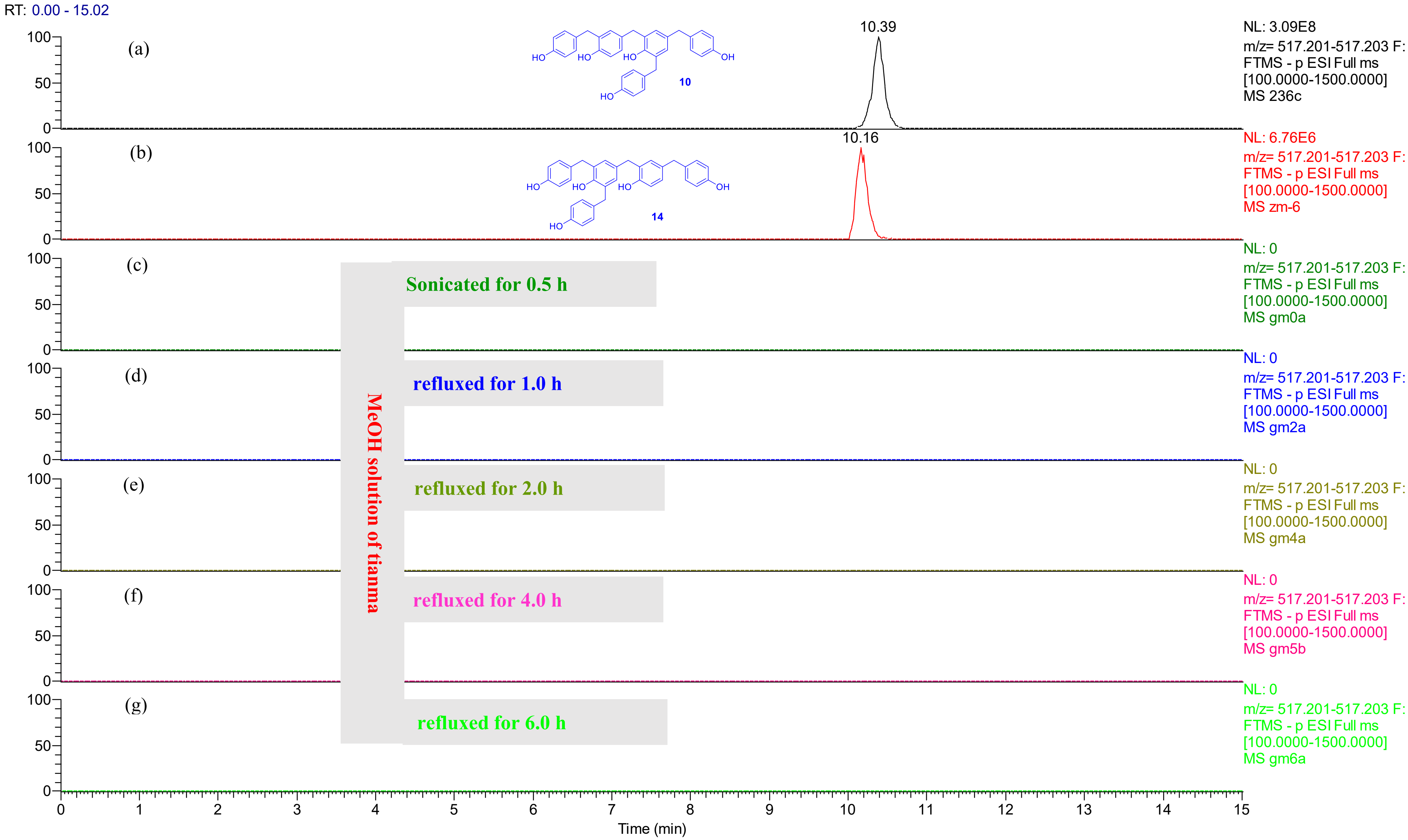

Figure S355. Overlaid chromatograms of the extracted negative ion at *m/z* 517.202 [M–H]⁻: (a) and (b) compounds **10** and **14** in CH_3CN, respectively; (c)–(g) MeOH solution of "tianma" was sonicated for 0.5 h then refluxed for 1.0 h, 2.0 h, 4.0 h, and 6.0 h, respectively.

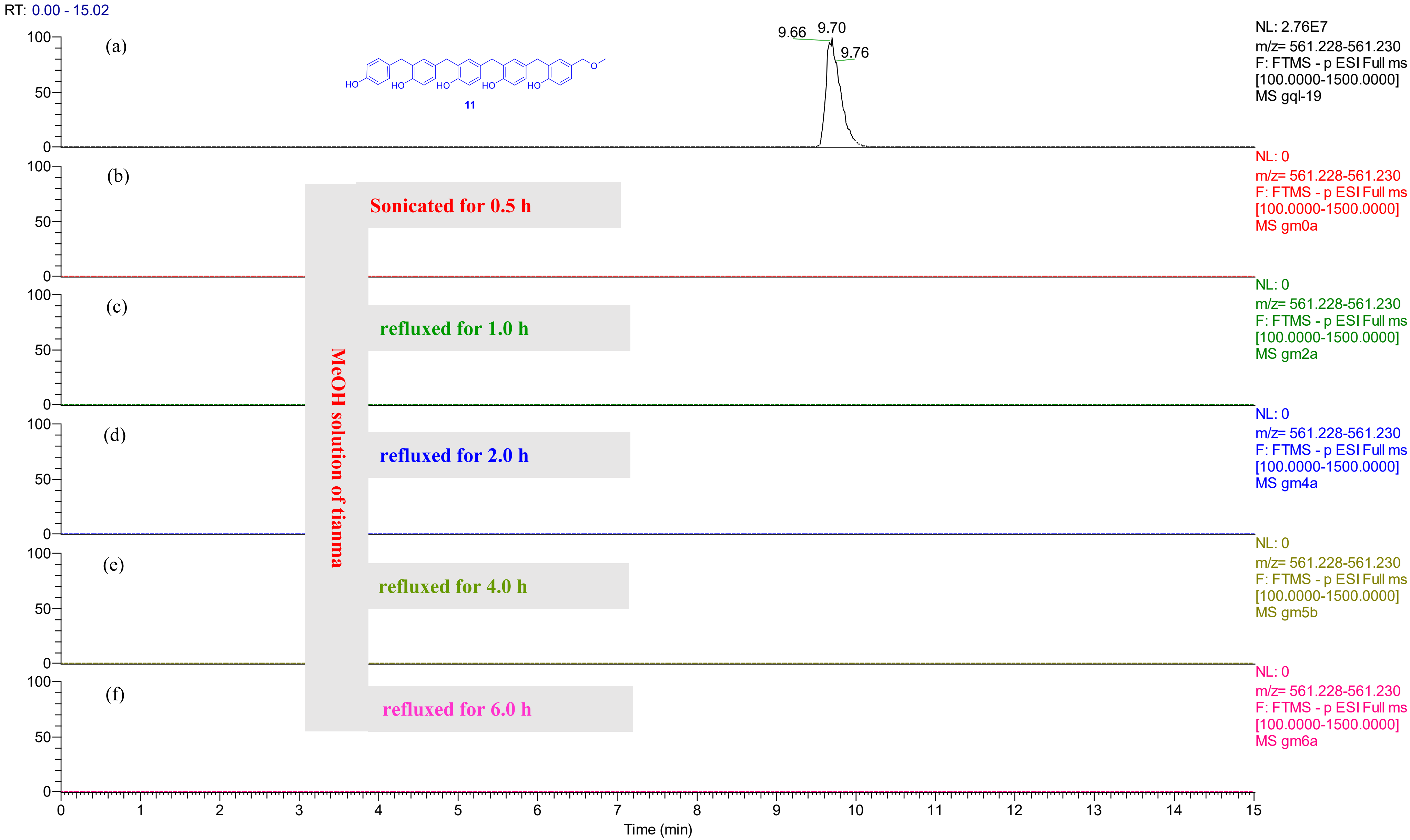

Figure S356. Overlaid chromatograms of the extracted negative ion at *m/z* 561.229 $[M-H]^-$: (a) compound **11** in CH_3CN; (b)–(f) MeOH solution of "tianma" was sonicated for 0.5 h then refluxed for 1.0 h, 2.0 h, 4.0 h, and 6.0 h, respectively.

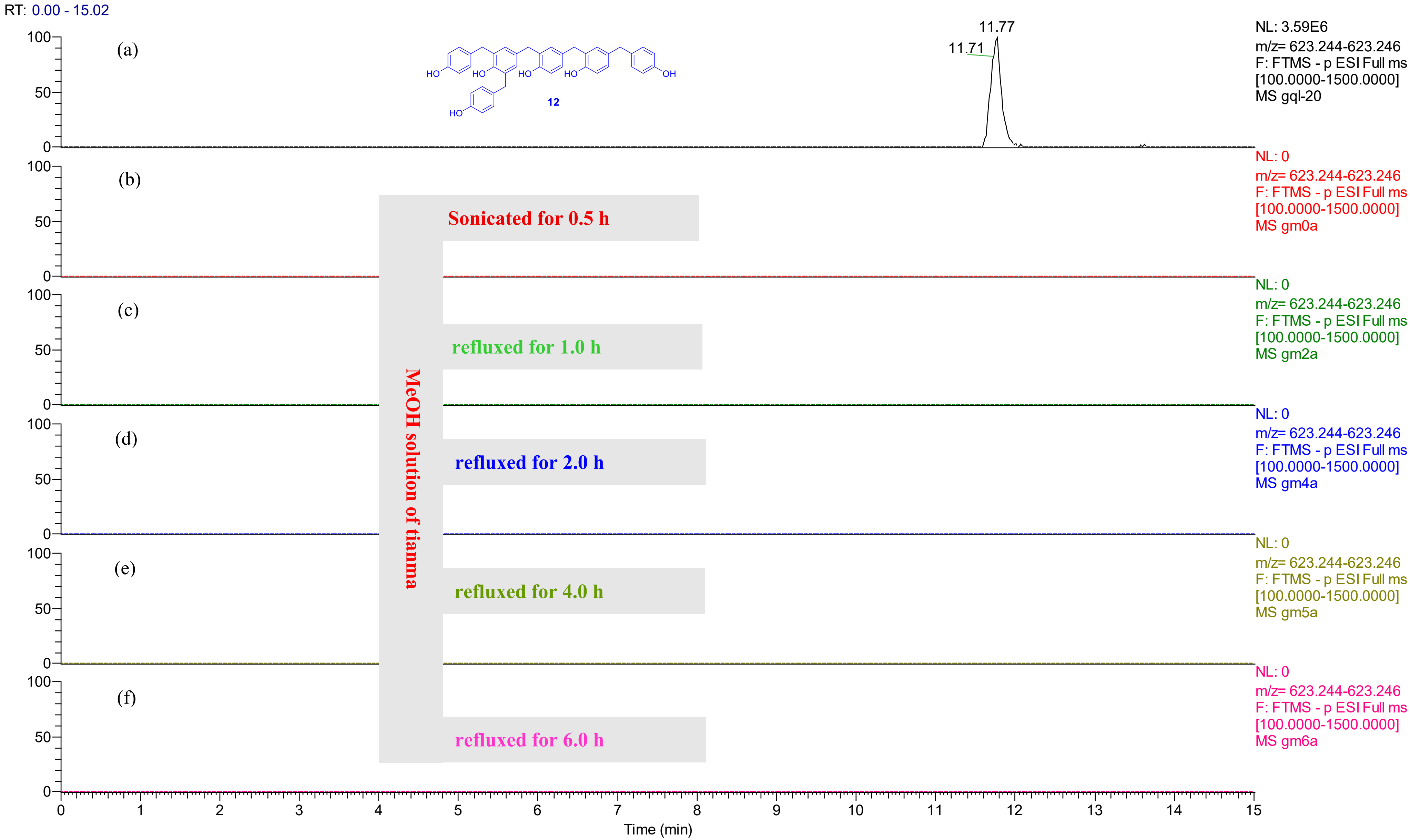

Figure S357. Overlaid chromatograms of the extracted negative ion at *m/z* 623.245 $[M–H]^-$: (a) compound **12** in CH_3CN; (b)–(f) MeOH solution of "tianma" was sonicated for 0.5 h then refluxed for 1.0 h, 2.0 h, 4.0 h, and 6.0 h, respectively.

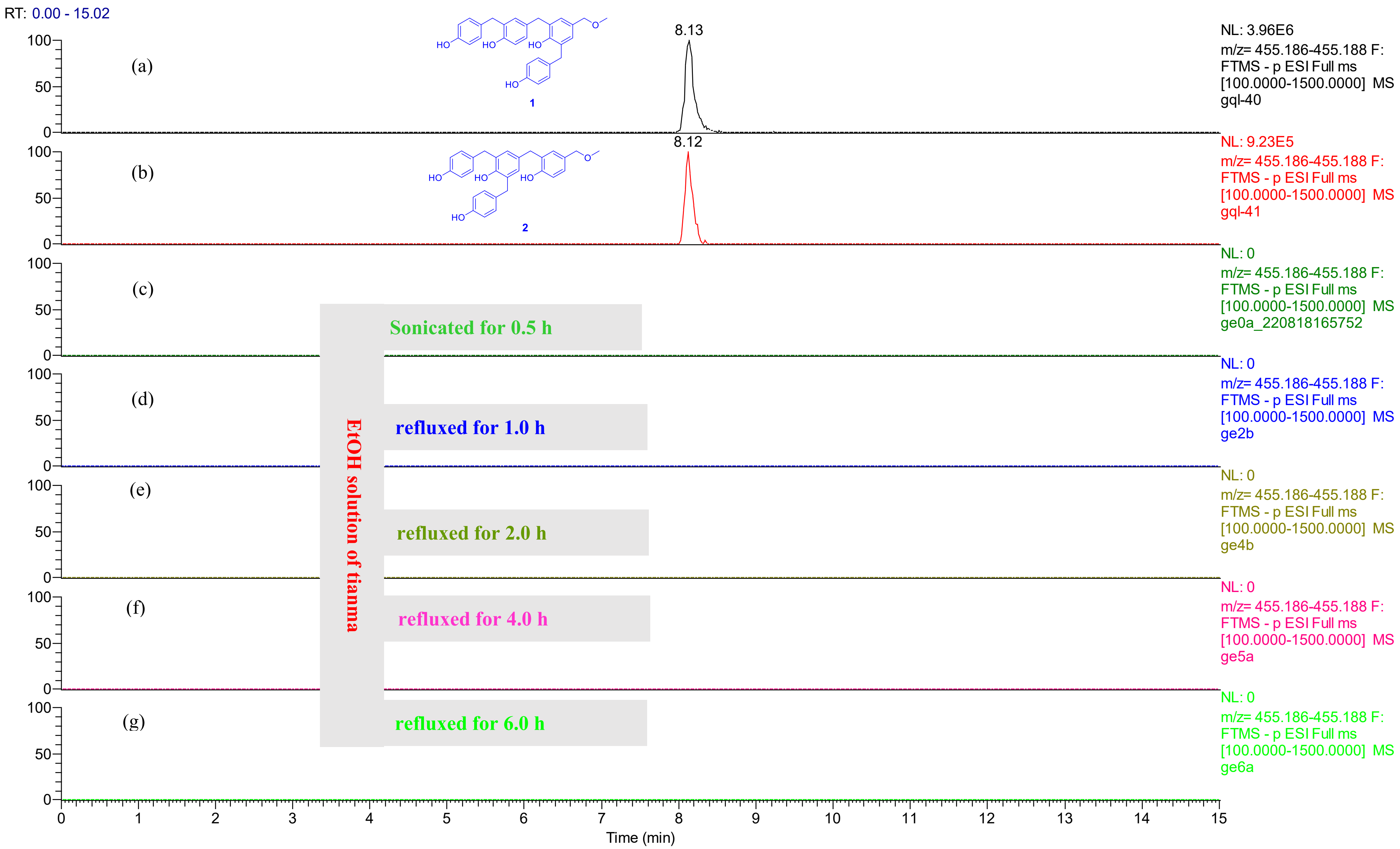

Figure S358. Overlaid chromatograms of the extracted negative ion at *m/z* 455.187 $[M–H]^-$: (a) and (b) compounds **1** and **2** in CH_3CN, respectively; (c)–(g) EtOH solution of "tianma" was sonicated for 0.5 h then refluxed for 1.0 h, 2.0 h, 4.0 h, and 6.0 h, respectively.

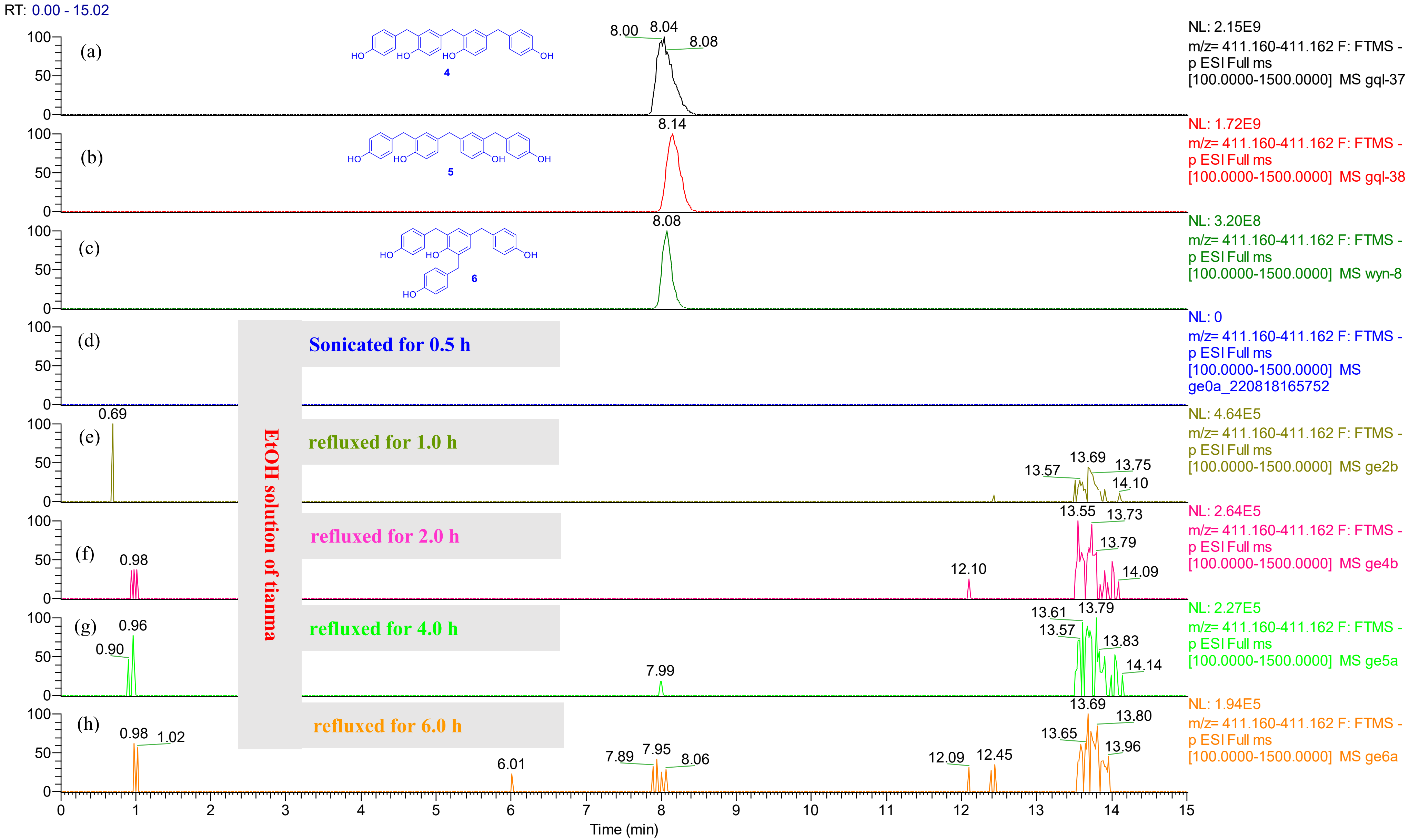

Figure S359. Overlaid chromatograms of the extracted negative ion at *m/z* 411.161 [M–H]$^-$: (a)–(c) compounds **4–6** in CH_3CN, respectively; (d)–(h) EtOH solution of "tianma" was sonicated for 0.5 h then refluxed for 1.0 h, 2.0 h, 4.0 h, and 6.0 h, respectively.

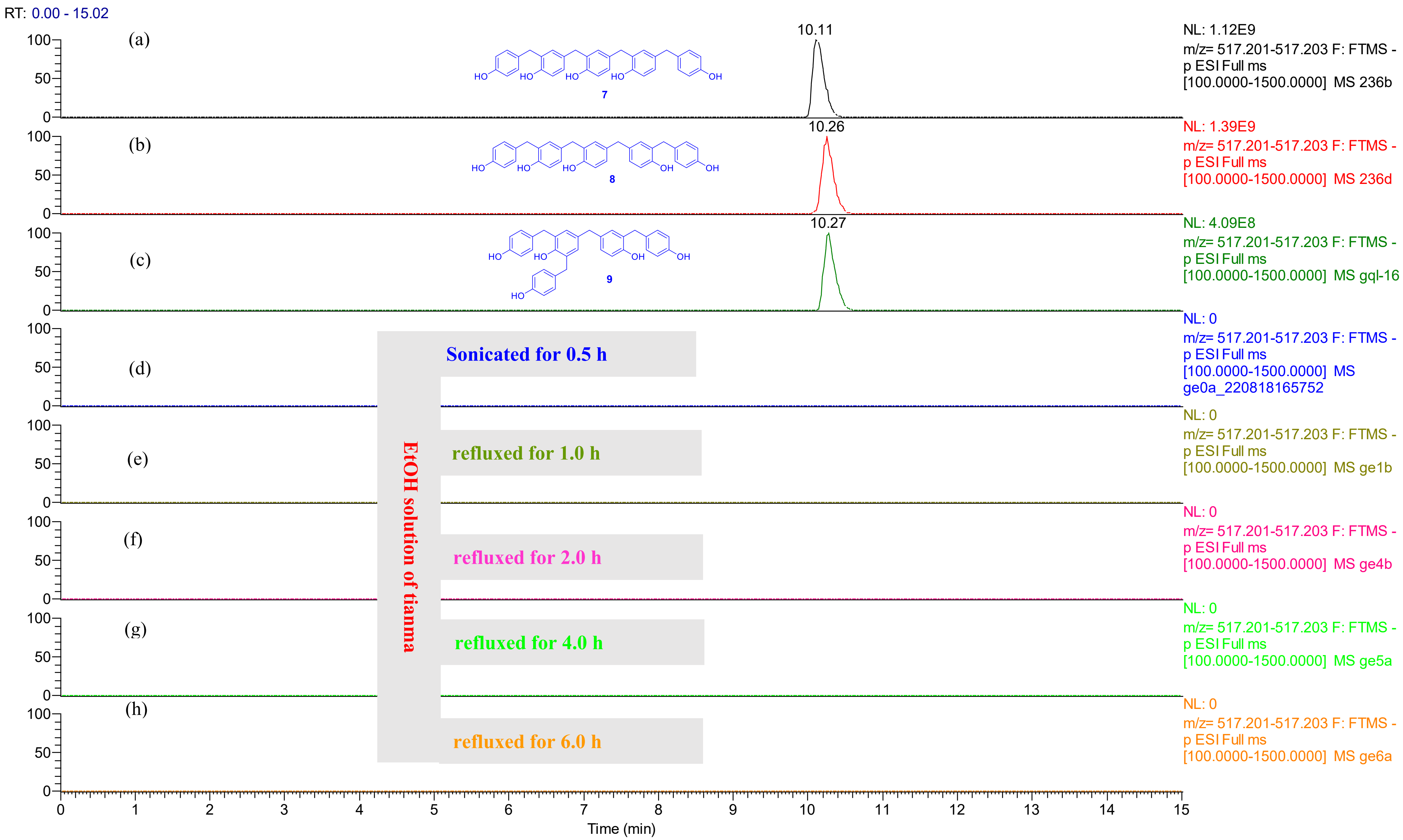

Figure S360. Overlaid chromatograms of the extracted negative ion at *m/z* 517.202 [M–H]⁻: (a)–(c) compounds **7**–**9** in CH_3CN, respectively; (d)–(h) EtOH solution of "tianma" was sonicated for 0.5 h then refluxed for 1.0 h, 2.0 h, 4.0 h, and 6.0 h, respectively.

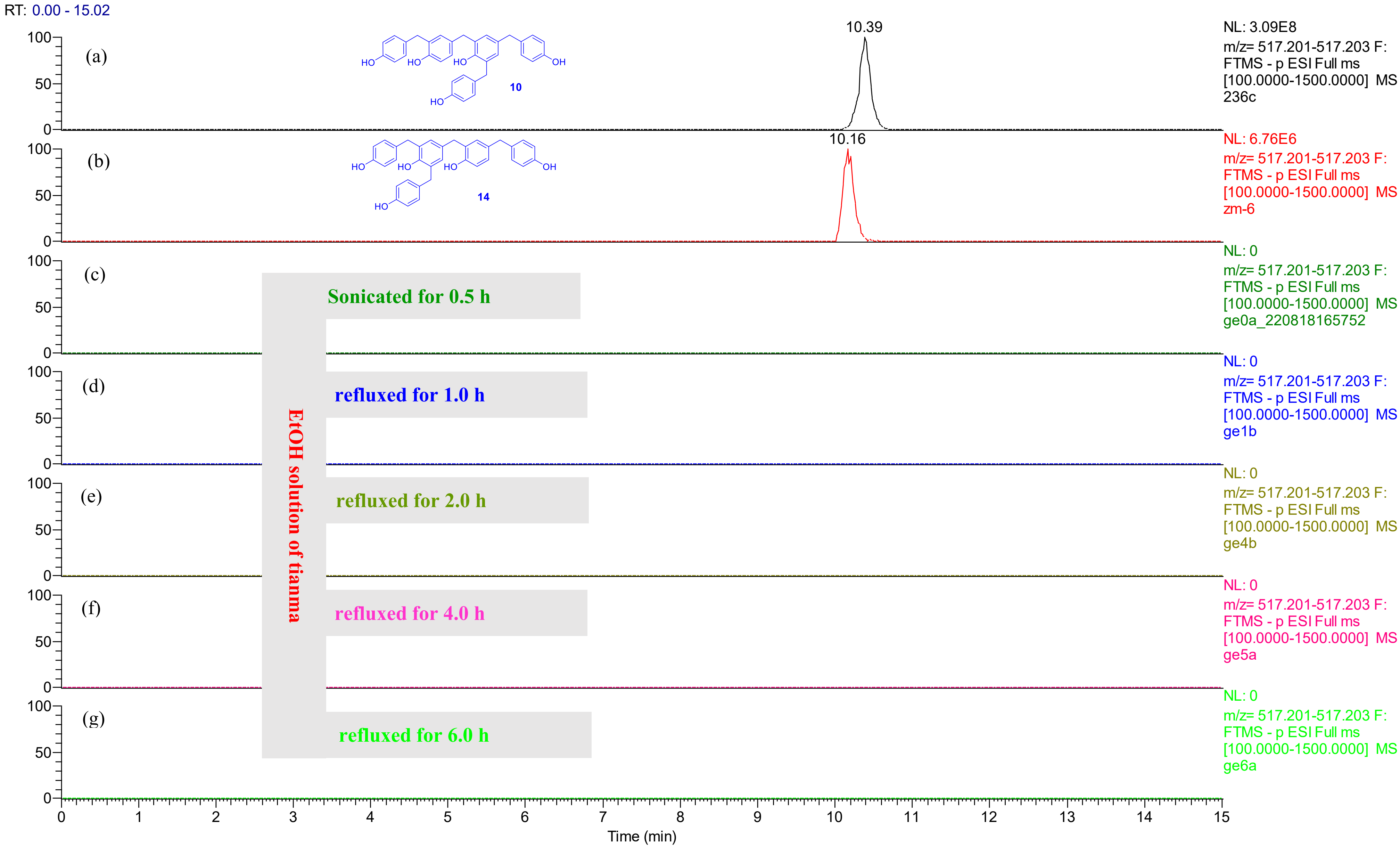

Figure S361. Overlaid chromatograms of the extracted negative ion at *m/z* 517.202 [M–H]$^-$: (a) and (b) compounds **10** and **14** in CH_3CN, respectively; (c)–(g) EtOH solution of "tianma" was sonicated for 0.5 h then refluxed for 1.0 h, 2.0 h, 4.0 h, and 6.0 h, respectively.

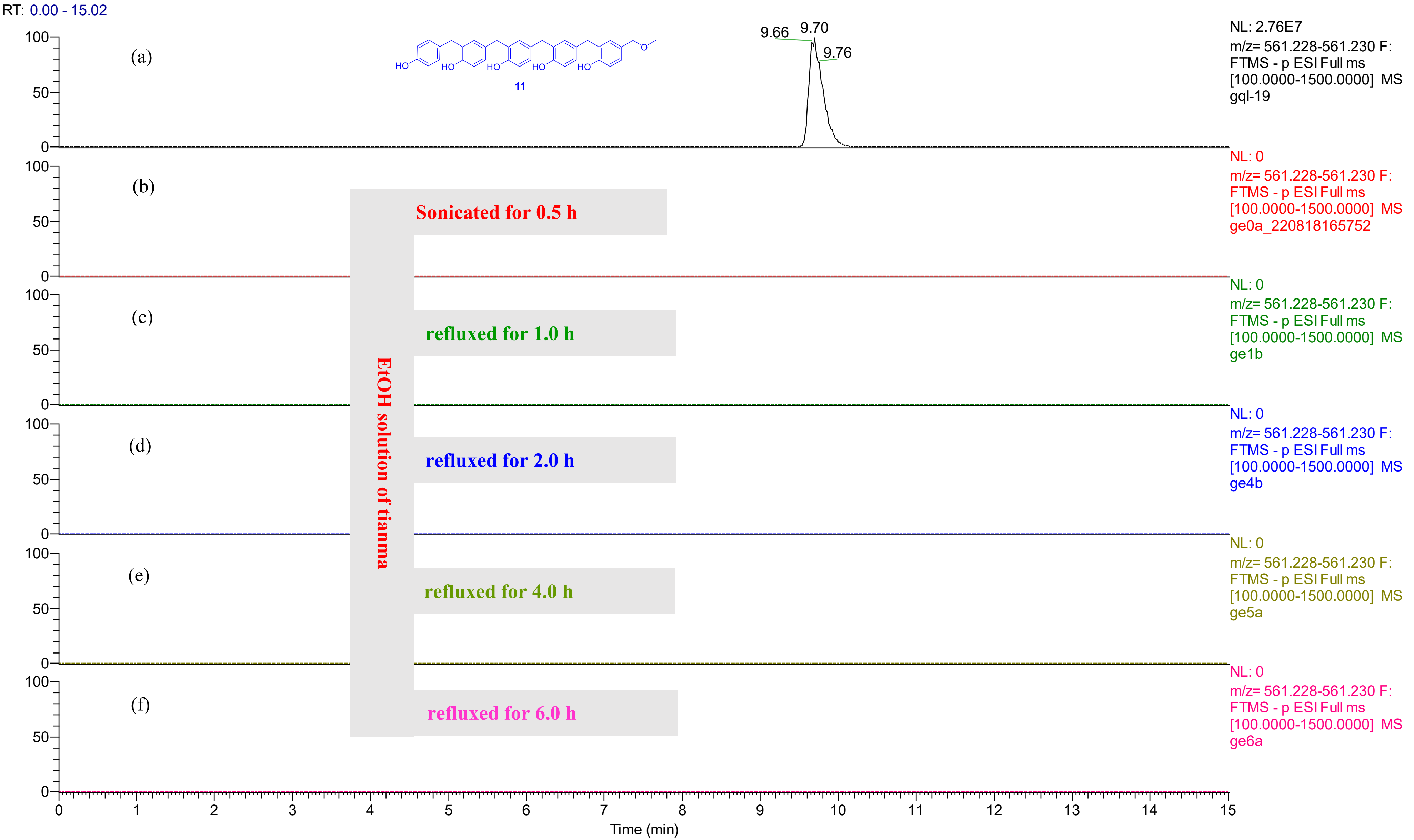

Figure S362. Overlaid chromatograms of the extracted negative ion at *m/z* 561.229 $[M–H]^-$: (a) compound **11** in CH_3CN; (b)–(f) EtOH solution of "tianma" was sonicated for 0.5 h then refluxed for 1.0 h, 2.0 h, 4.0 h, and 6.0 h, respectively.

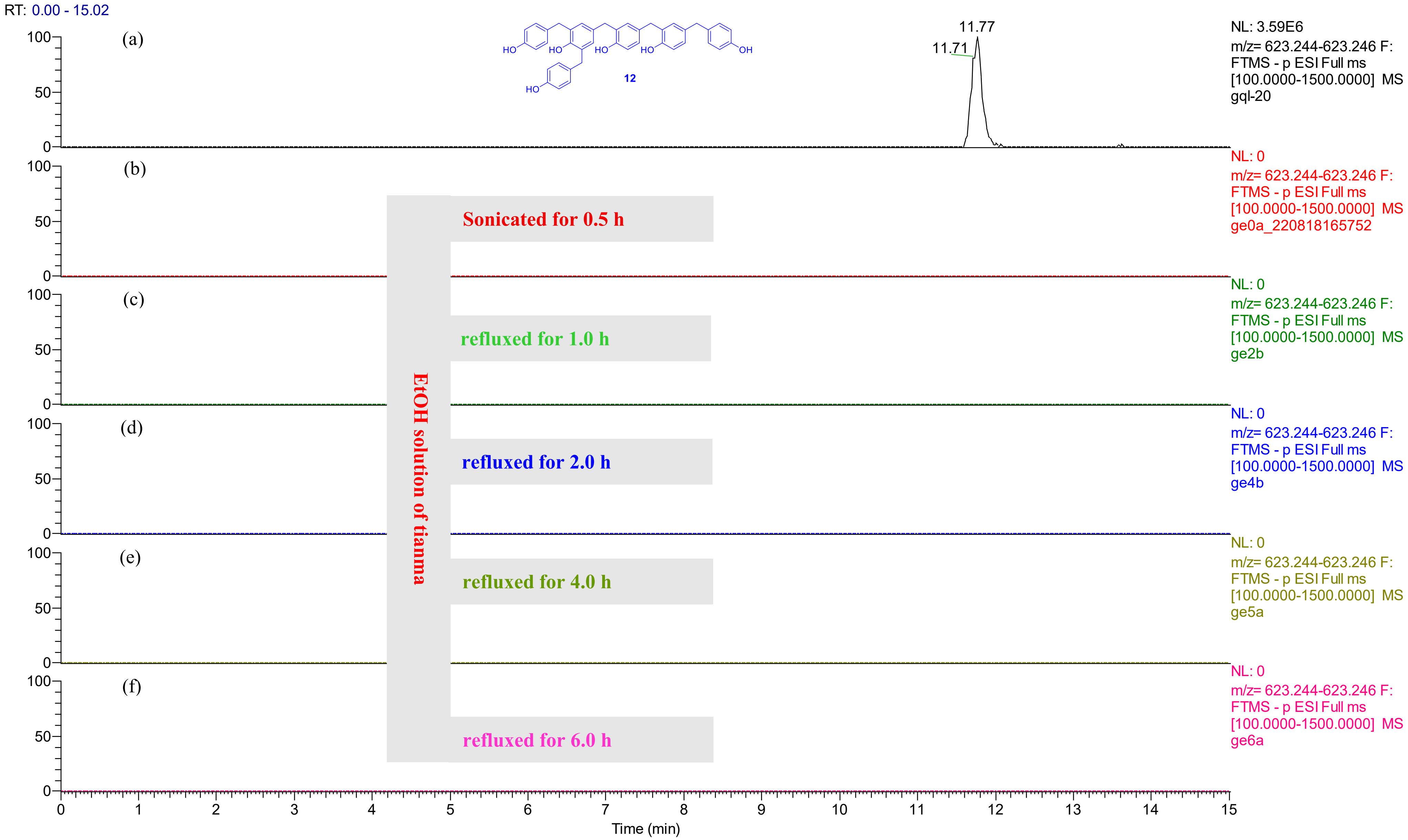

Figure S363. Overlaid chromatograms of the extracted negative ion at *m/z* 623.245 [M–H]$^-$: (a) compound **12** in CH_3CN; (b)–(f) EtOH solution of “tianma” was sonicated for 0.5 h then refluxed for 1.0 h, 2.0 h, 4.0 h, and 6.0 h, respectively.

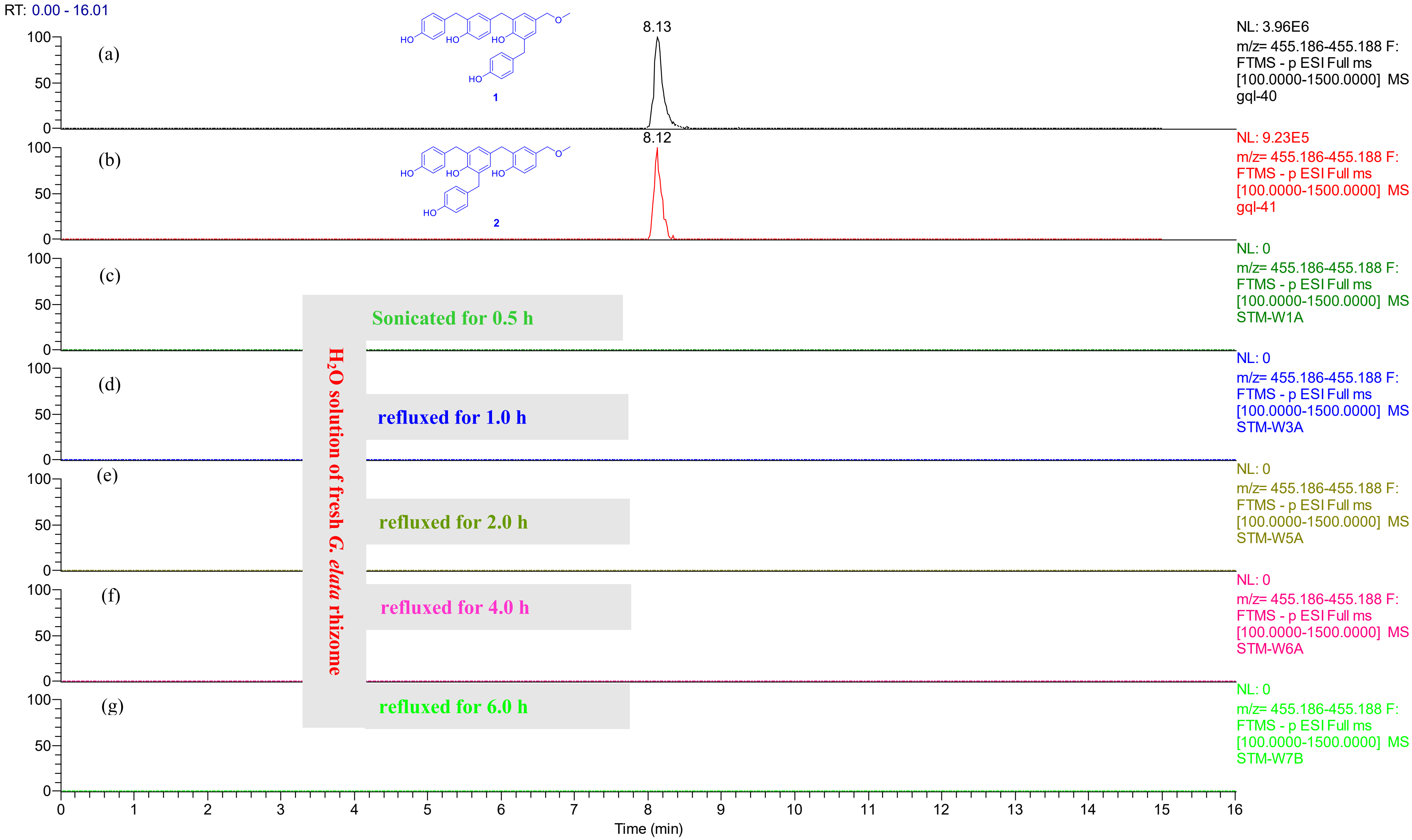

Figure S364. Overlaid chromatograms of the extracted negative ion at *m/z* 455.187 [M–H]⁻: (a) and (b) compounds **1** and **2** in CH_3CN, respectively; (c)–(g) H_2O solution of fresh *G. elata* rhizome was sonicated for 0.5 h then refluxed for 1.0 h, 2.0 h, 4.0 h, and 6.0 h, respectively.

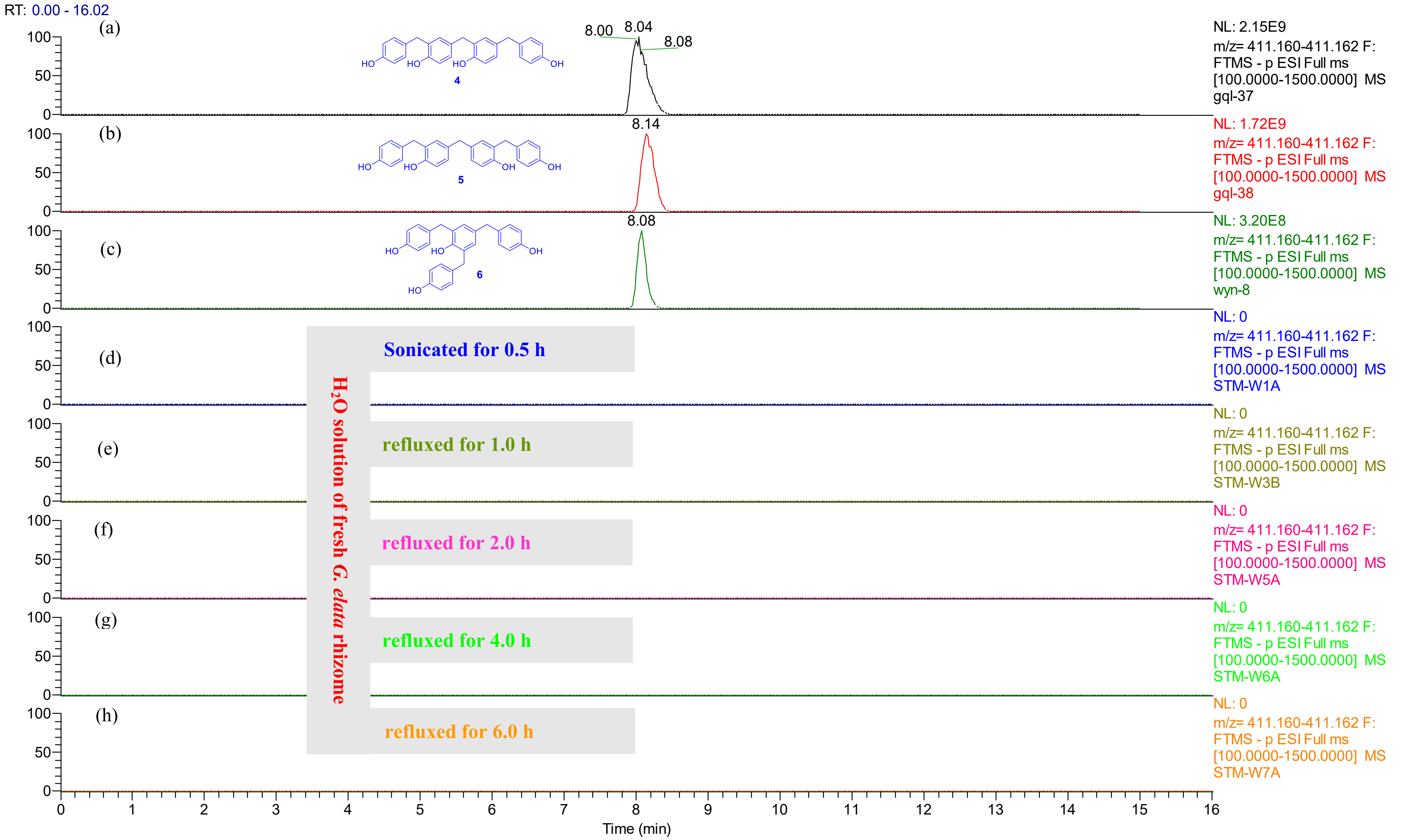

Figure S365. Overlaid chromatograms of the extracted negative ion at *m/z* 411.161 $[M–H]^-$: (a)–(c) compounds **4**–**6** in CH_3CN, respectively; (d)–(h) H_2O solution of fresh *G. elata* rhizome was sonicated for 0.5 h then refluxed for 1.0 h, 2.0 h, 4.0 h, and 6.0 h, respectively.

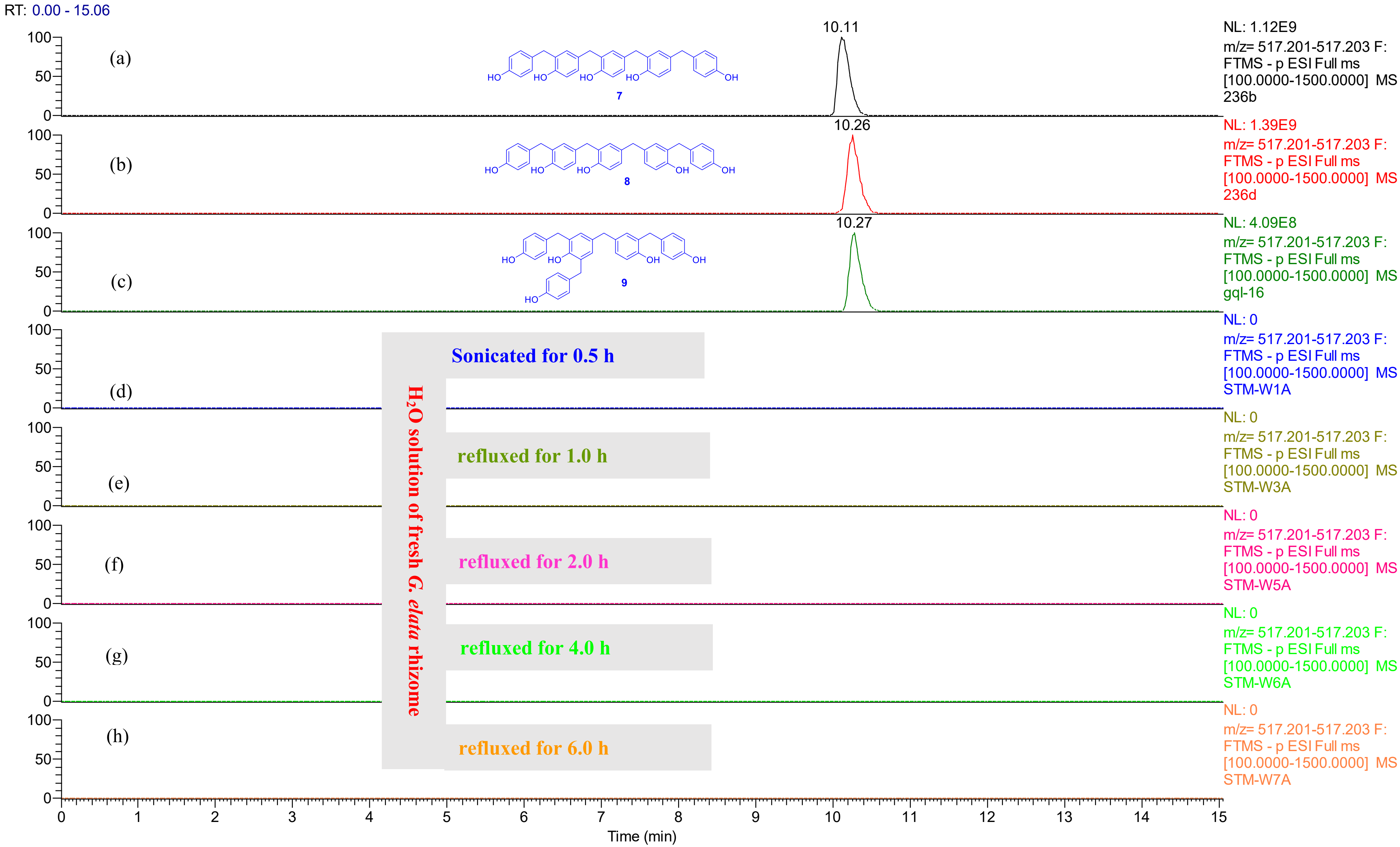

Figure S366. Overlaid chromatograms of the extracted negative ion at *m/z* 517.202 [M–H]$^-$: (a)–(c) compounds **7–9** in CH_3CN, respectively; (d)–(h) H_2O solution of fresh *G. elata* rhizome was sonicated for 0.5 h then refluxed for 1.0 h, 2.0 h, 4.0 h, and 6.0 h, respectively.

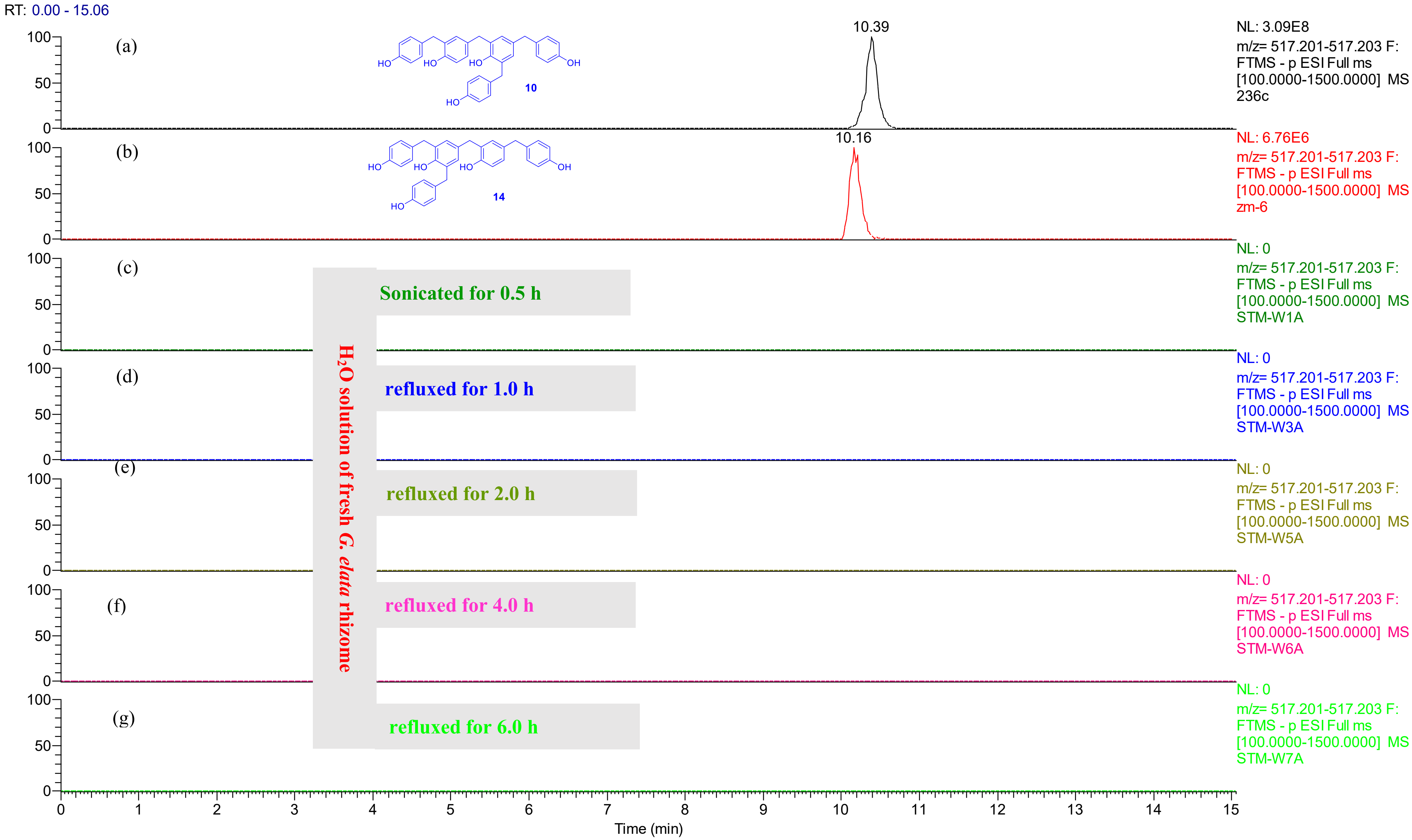

Figure S367. Overlaid chromatograms of the extracted negative ion at *m/z* 517.202 [M–H]⁻: (a) and (b) compounds **10** and **14** in CH_3CN, respectively; (c)–(g) H_2O solution of fresh *G. elata* rhizome was sonicated for 0.5 h then refluxed for 1.0 h, 2.0 h, 4.0 h, and 6.0 h, respectively.

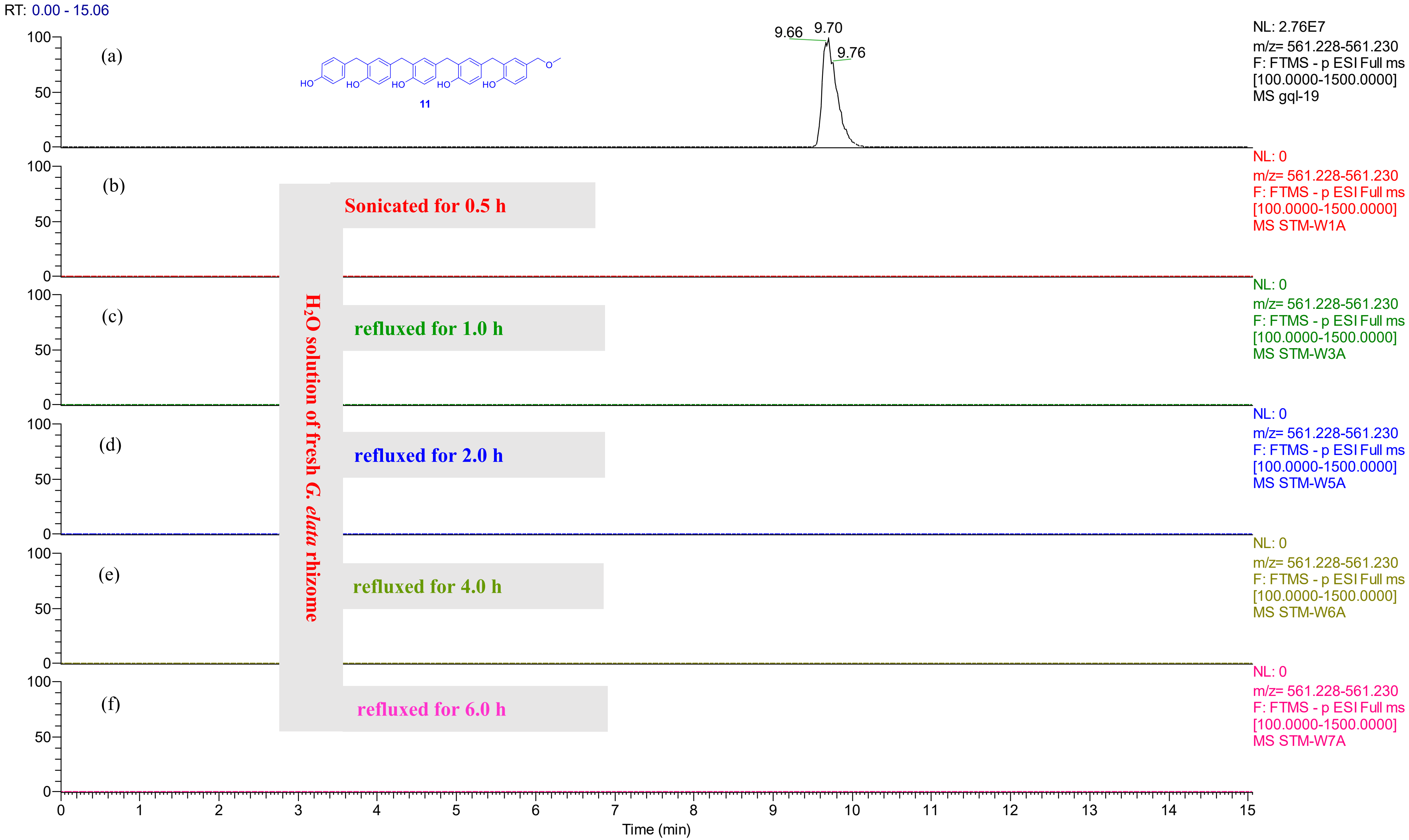

Figure S368. Overlaid chromatograms of the extracted negative ion at *m/z* 561.229 $[M–H]^-$: (a) compound **11** in CH_3CN; (b)–(f) H_2O solution of fresh *G. elata* rhizome was sonicated for 0.5 h then refluxed for 1.0 h, 2.0 h, 4.0 h, and 6.0 h, respectively.

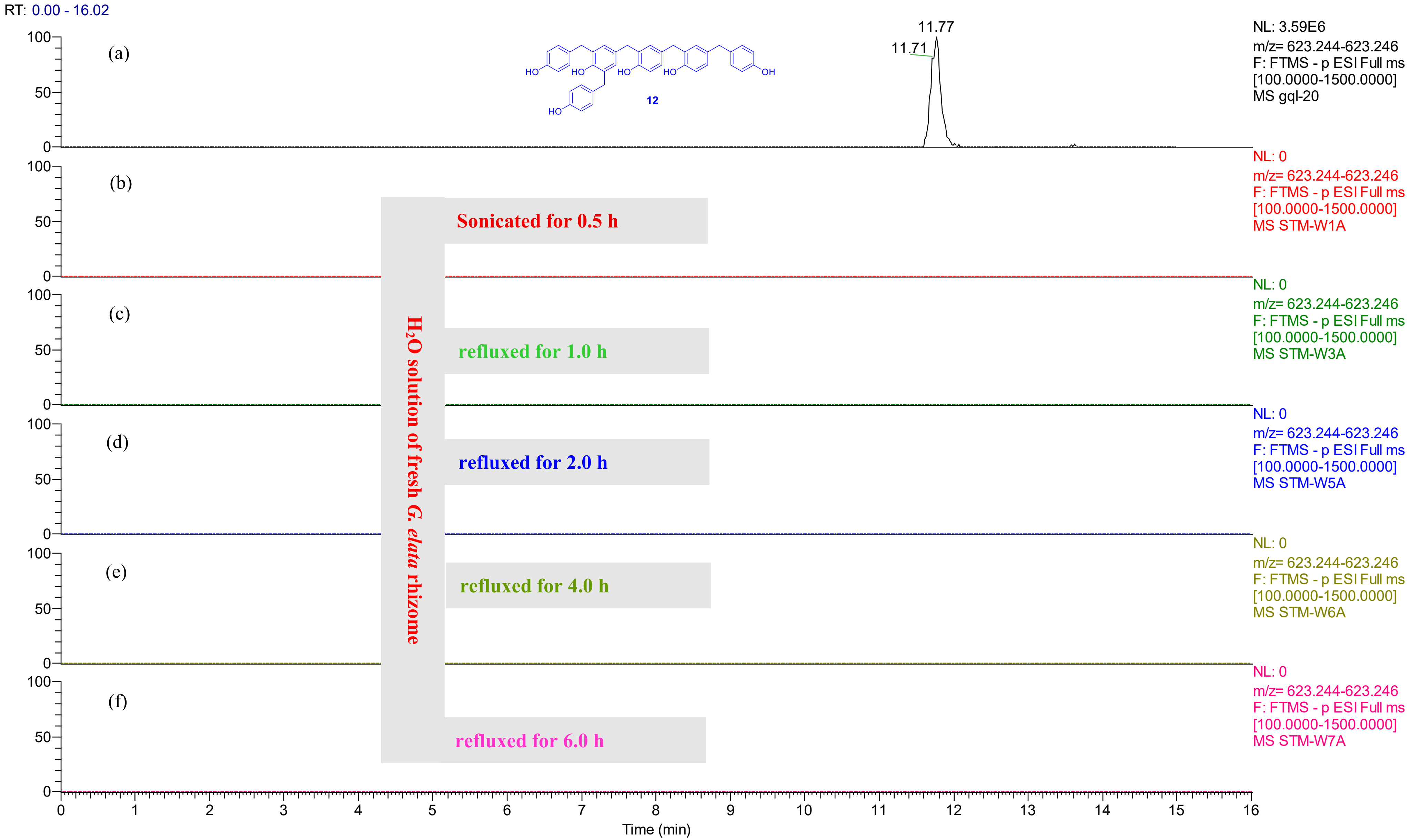

Figure S369. Overlaid chromatograms of the extracted negative ion at *m/z* 623.245 [M–H]$^-$: (a) compound **12** in CH_3CN; (b)–(f) H_2O solution of fresh *G. elata* rhizome was sonicated for 0.5 h then refluxed for 1.0 h, 2.0 h, 4.0 h, and 6.0 h, respectively.

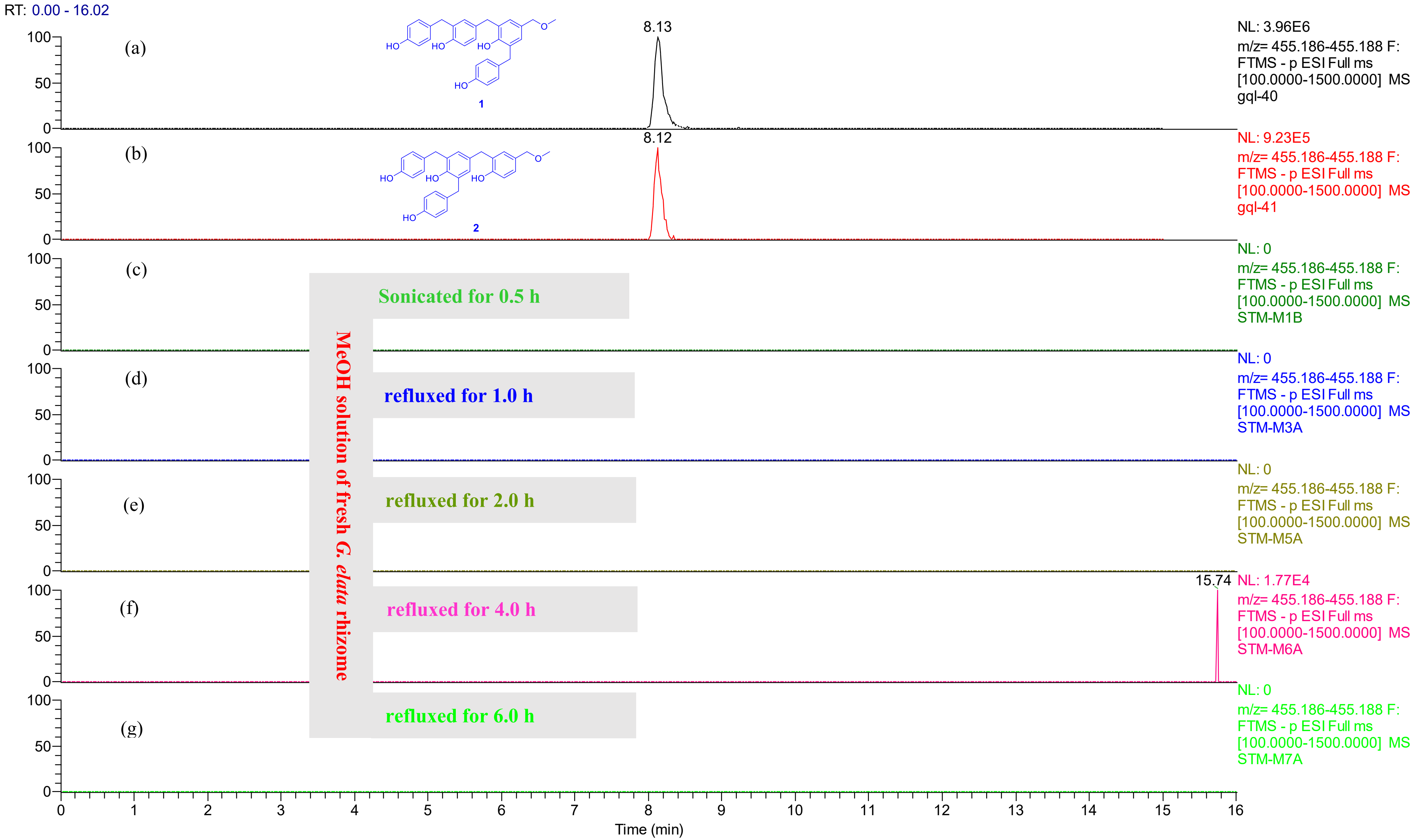

Figure S370. Overlaid chromatograms of the extracted negative ion at *m/z* 455.187 [M–H]⁻: (a) and (b) compounds **1** and **2** in CH_3CN, respectively; (c)–(g) MeOH solution of fresh *G. elata* rhizome was sonicated for 0.5 h then refluxed for 1.0 h, 2.0 h, 4.0 h, and 6.0 h, respectively.

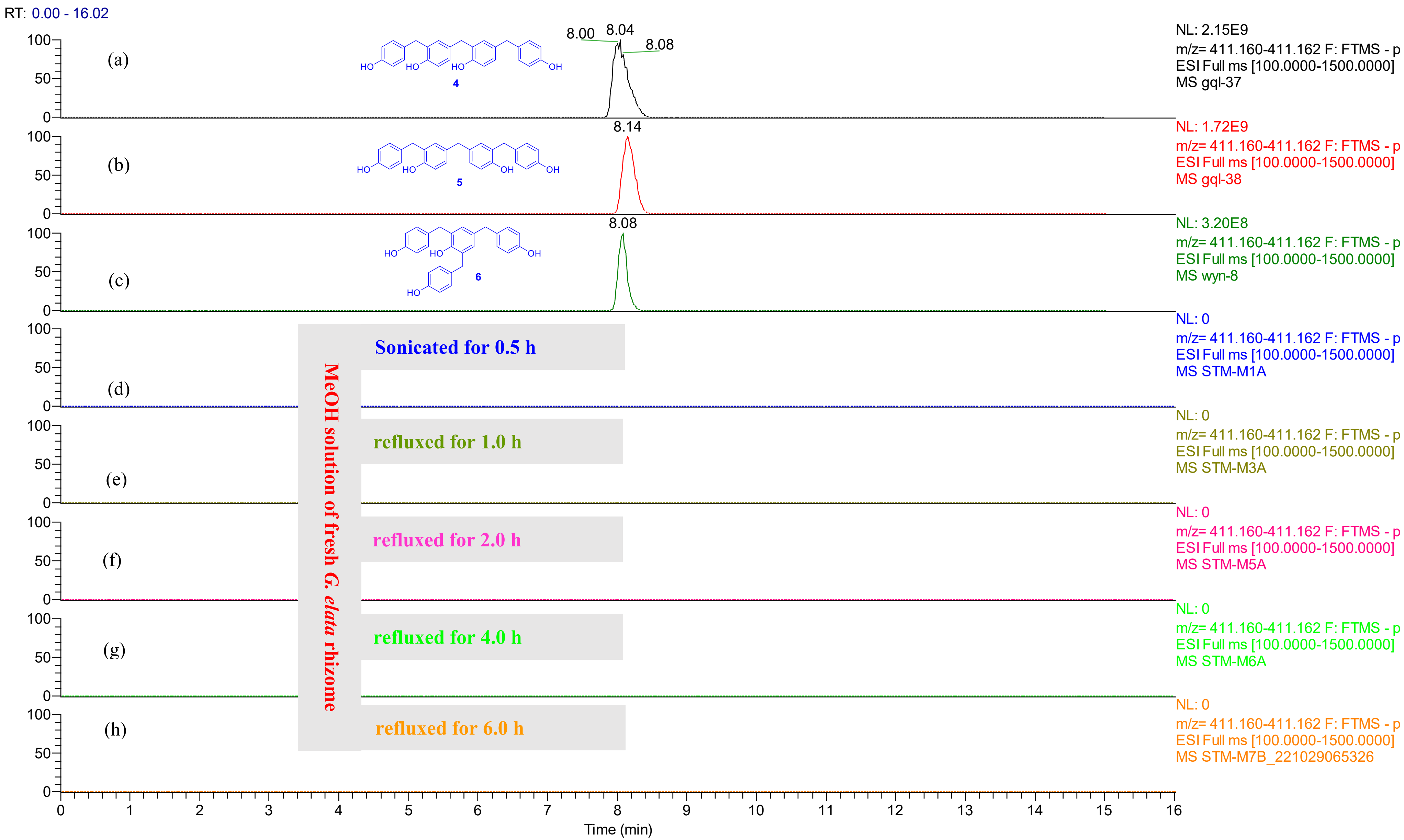

Figure S371. Overlaid chromatograms of the extracted negative ion at *m/z* 411.161 [M–H]⁻: (a)–(c) compounds **4**–**6** in CH_3CN, respectively; (d)–(h) MeOH solution of fresh *G. elata* rhizome was sonicated for 0.5 h then refluxed for 1.0 h, 2.0 h, 4.0 h, and 6.0 h, respectively.

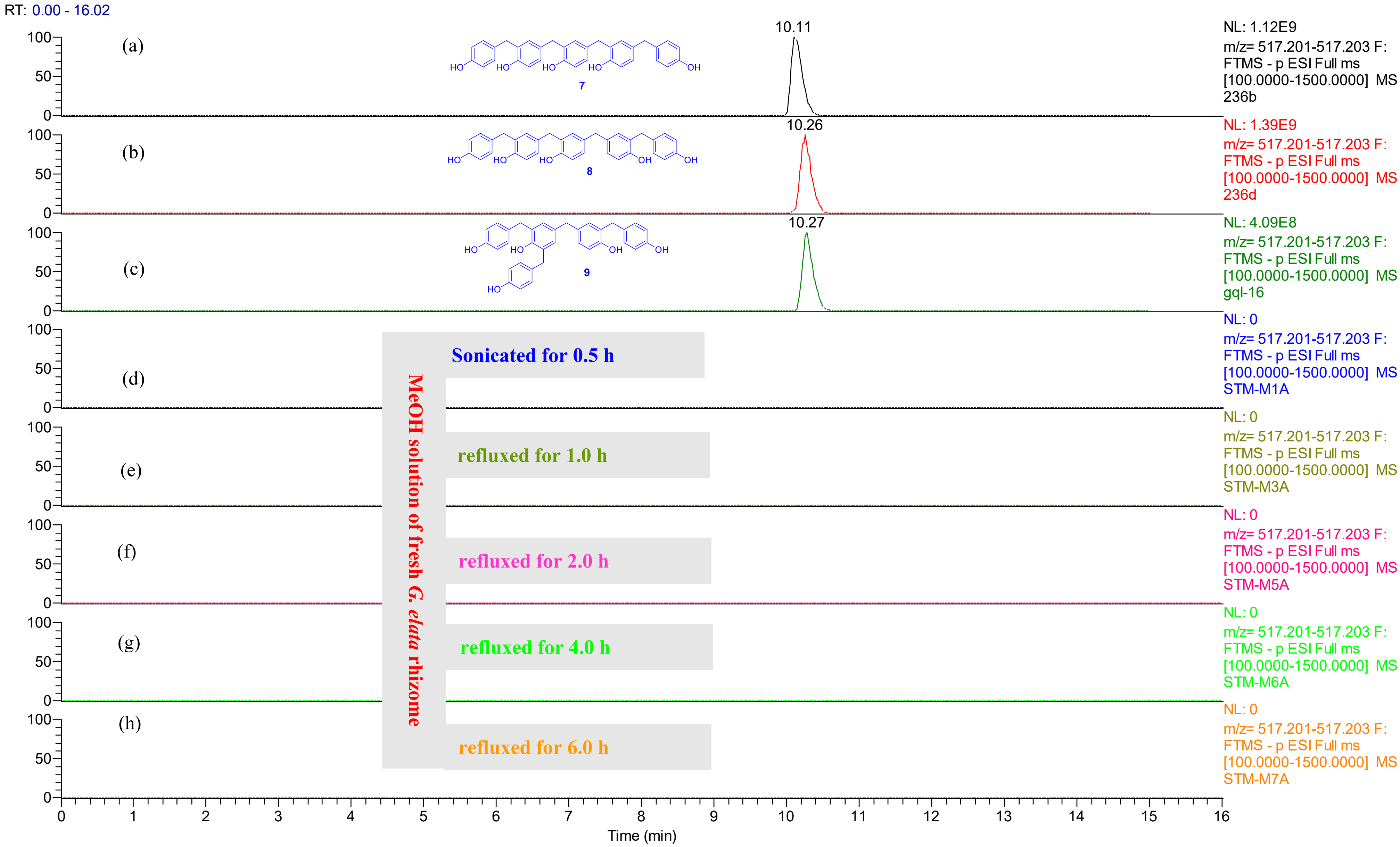

Figure S372. Overlaid chromatograms of the extracted negative ion at *m/z* 517.202 $[M–H]^-$: (a)–(c) compounds **7**–**9** in CH_3CN, respectively; (d)–(h) MeOH solution of fresh *G. elata* rhizome was sonicated for 0.5 h then refluxed for 1.0 h, 2.0 h, 4.0 h, and 6.0 h, respectively.

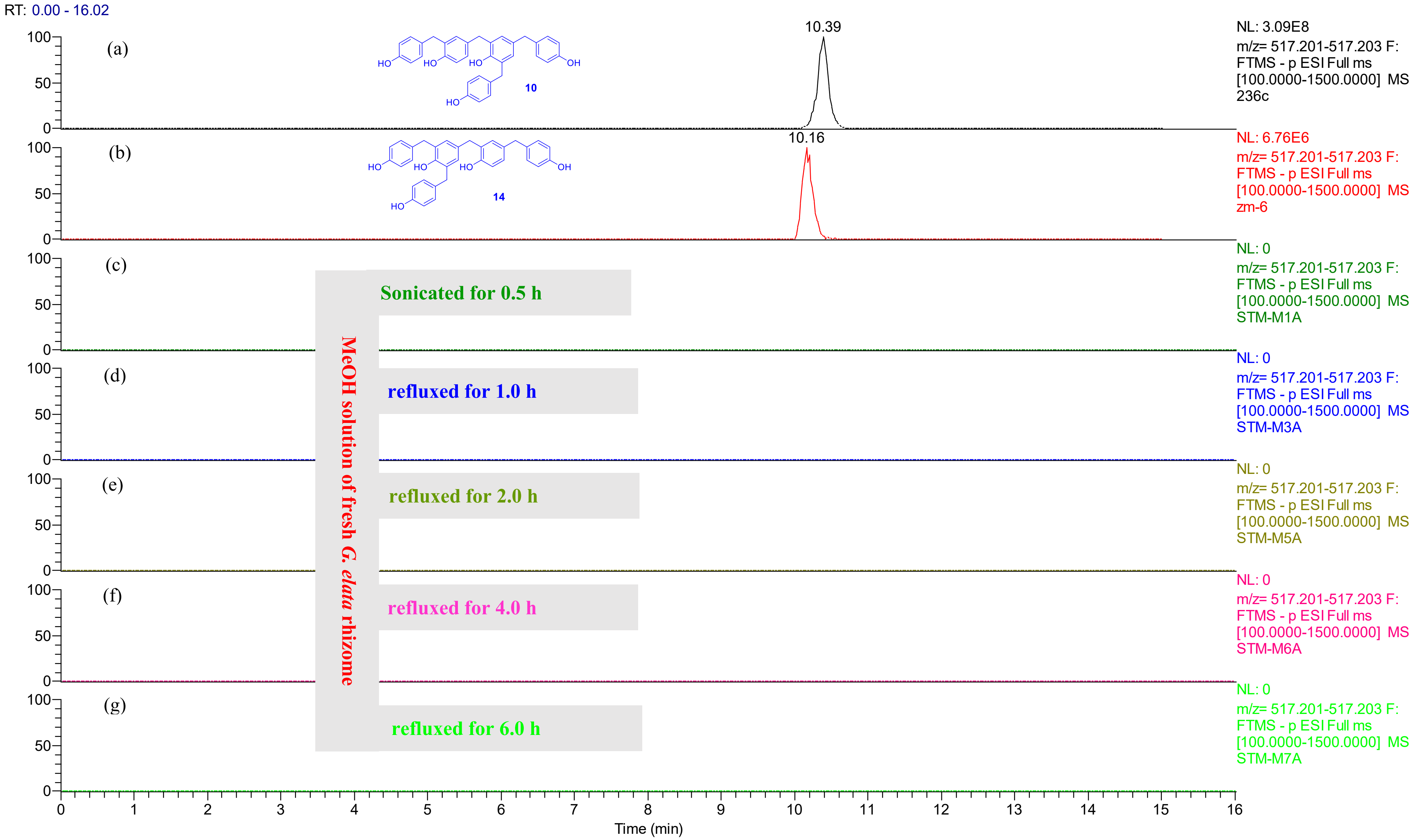

Figure S373. Overlaid chromatograms of the extracted negative ion at *m/z* 517.202 $[M–H]^-$: (a) and (b) compounds **10** and **14** in CH_3CN, respectively; (c)–(g) MeOH solution of fresh *G. elata* rhizome was sonicated for 0.5 h then refluxed for 1.0 h, 2.0 h, 4.0 h, and 6.0 h, respectively.

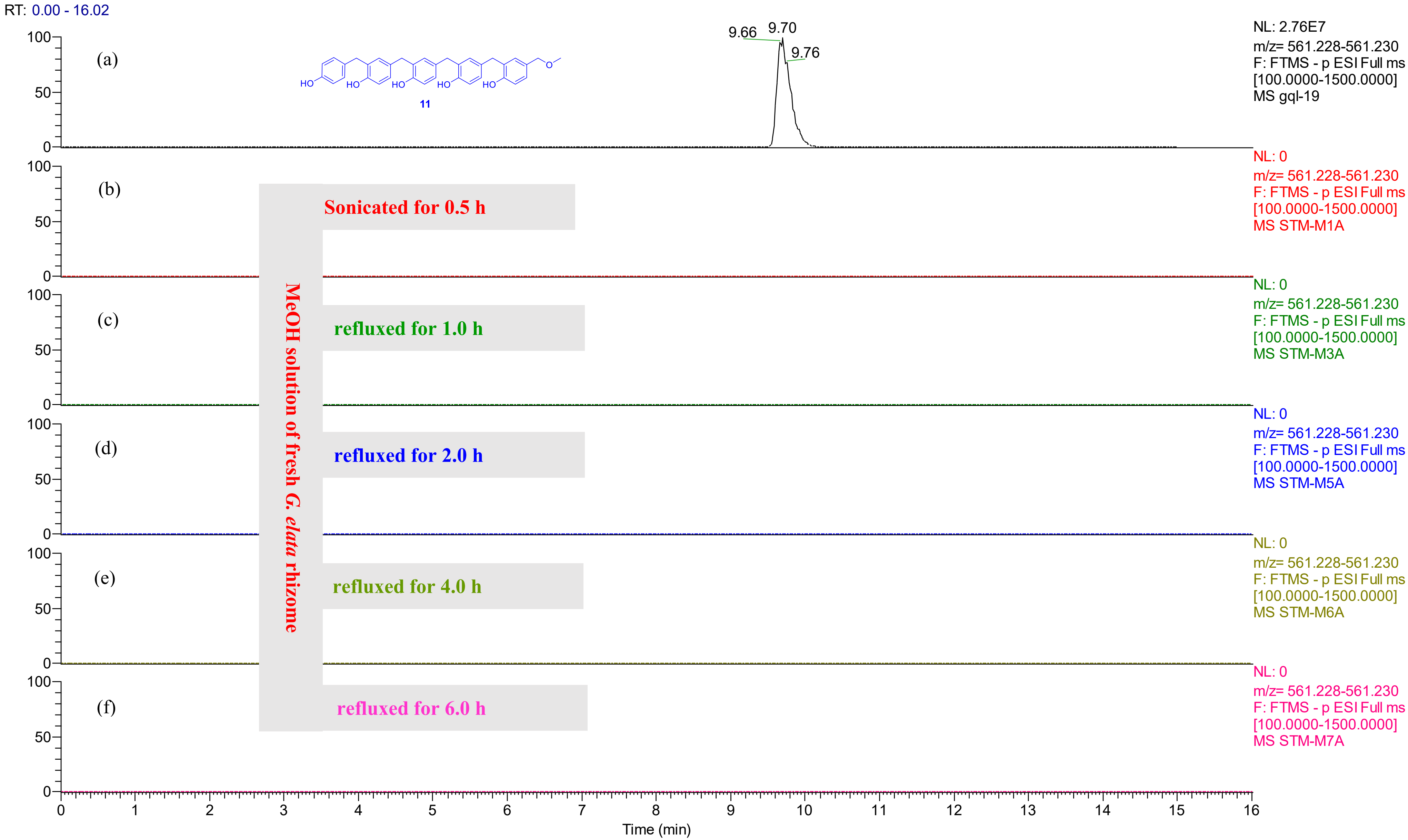

Figure S374. Overlaid chromatograms of the extracted negative ion at *m/z* 561.229 [M–H]⁻: (a) compound **11** in CH_3CN; (b)–(f) MeOH solution of fresh *G. elata* rhizome was sonicated for 0.5 h then refluxed for 1.0 h, 2.0 h, 4.0 h, and 6.0 h, respectively.

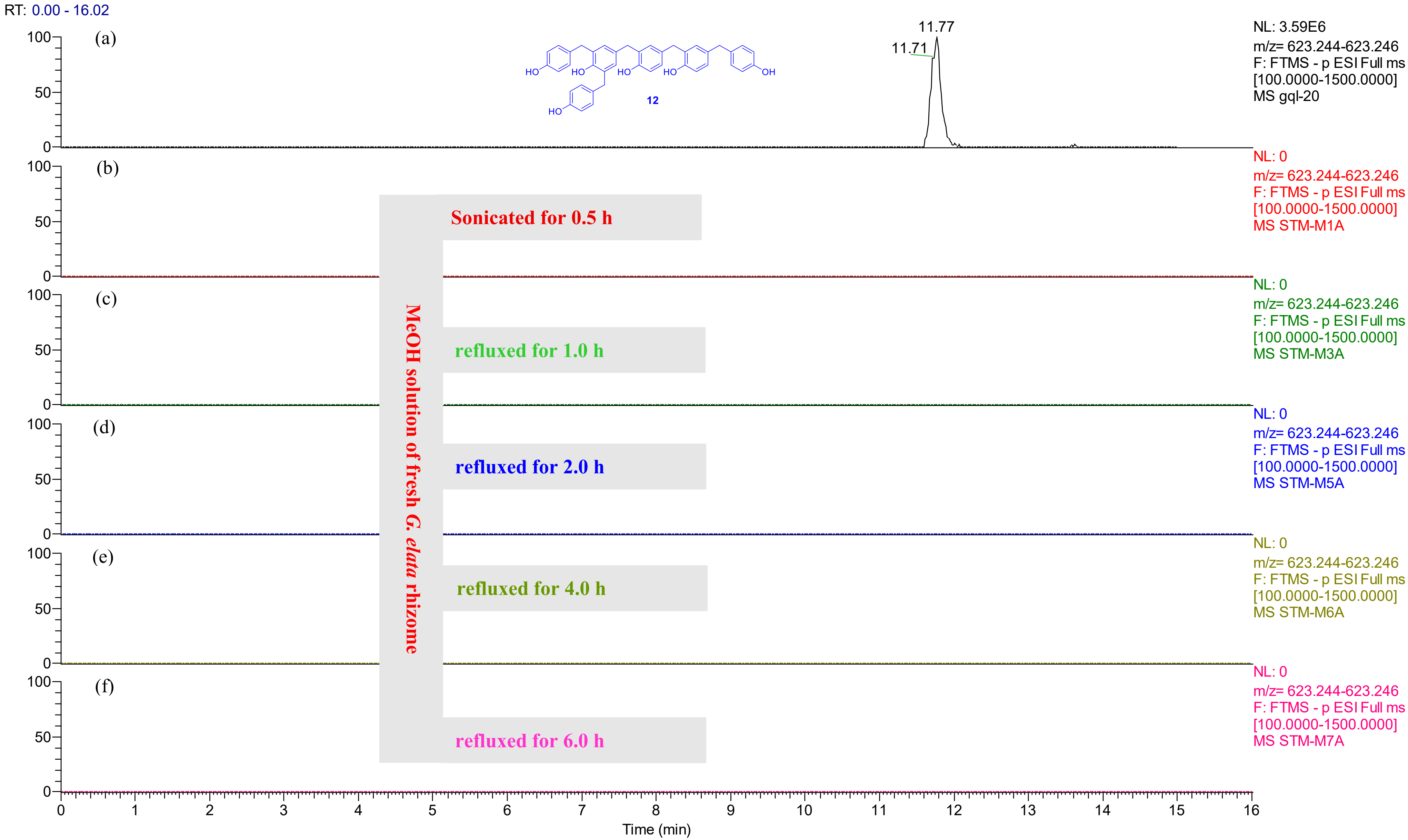

Figure S375. Overlaid chromatograms of the extracted negative ion at *m/z* 623.245 [M–H]$^-$: (a) compound **12** in CH_3CN; (b)–(f) MeOH solution of fresh *G. elata* rhizome was sonicated for 0.5 h then refluxed for 1.0 h, 2.0 h, 4.0 h, and 6.0 h, respectively.

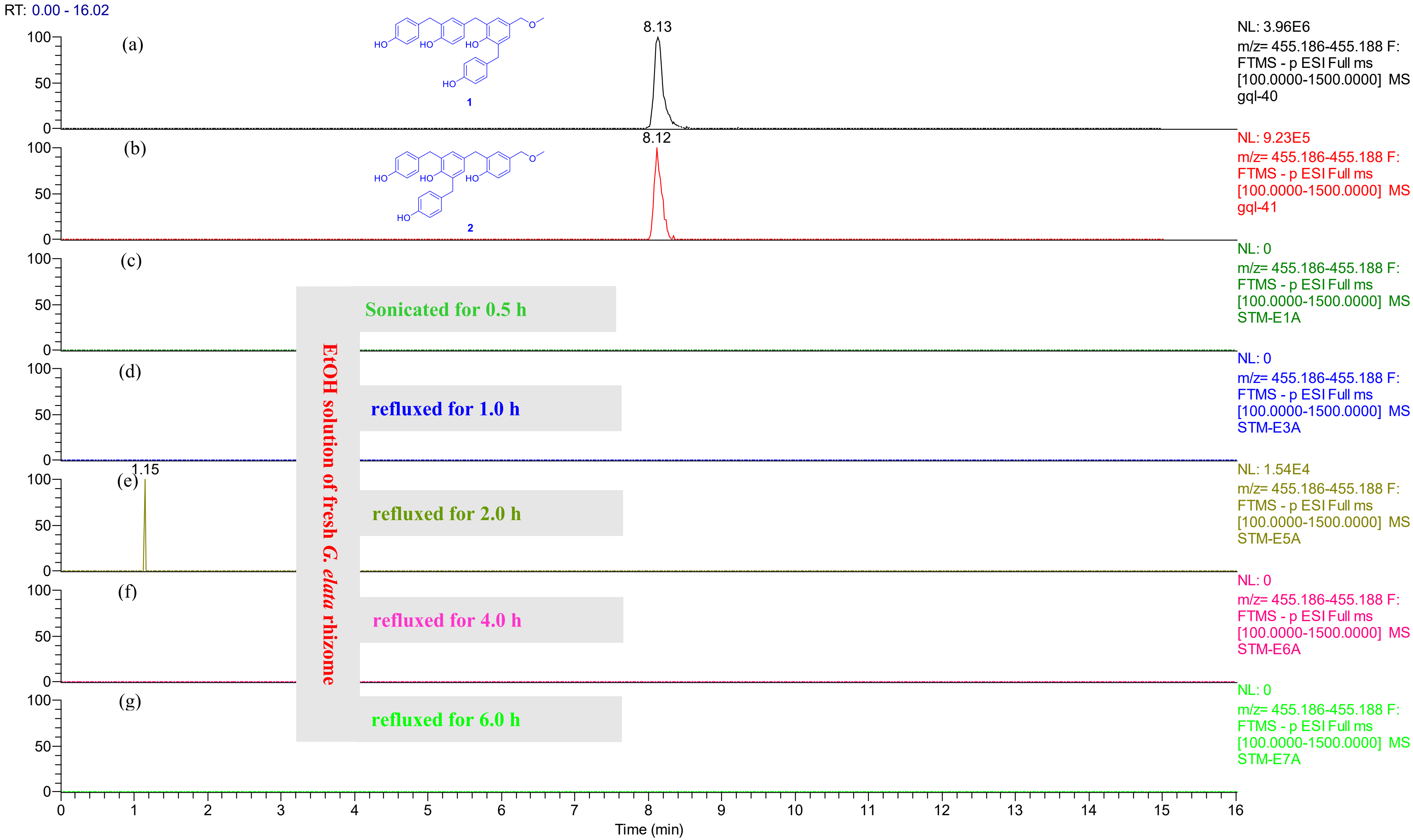

Figure S376. Overlaid chromatograms of the extracted negative ion at *m/z* 455.187 $[M–H]^-$: (a) and (b) compounds **1** and **2** in CH_3CN, respectively; (c)–(g) EtOH solution of fresh *G. elata* rhizome was sonicated for 0.5 h then refluxed for 1.0 h, 2.0 h, 4.0 h, and 6.0 h, respectively.

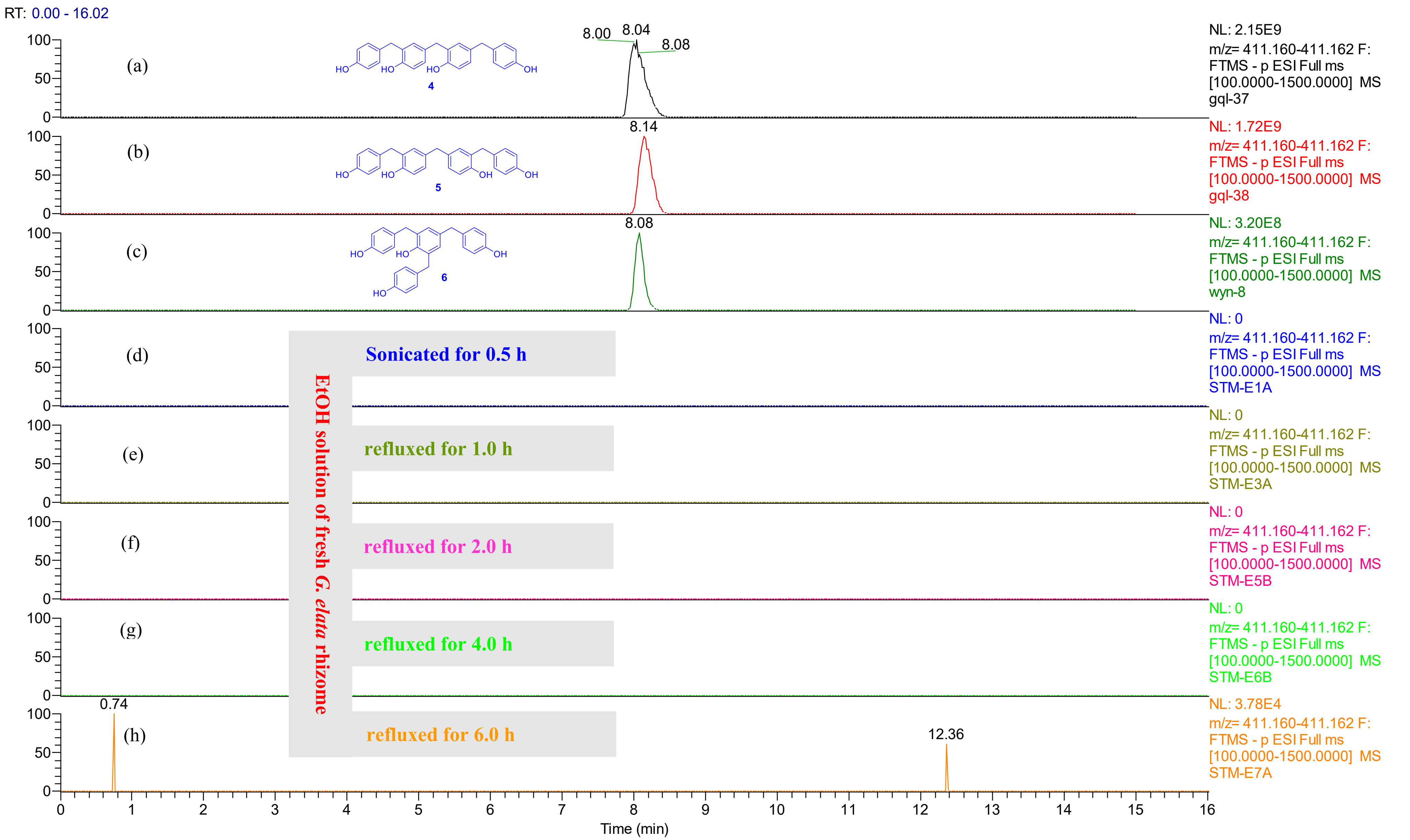

Figure S377. Overlaid chromatograms of the extracted negative ion at *m/z* 411.161 [M–H]⁻: (a)–(c) compounds **4**–**6** in CH_3CN, respectively; (d)–(h) EtOH solution of fresh *G. elata* rhizome was sonicated for 0.5 h then refluxed for 1.0 h, 2.0 h, 4.0 h, and 6.0 h, respectively.

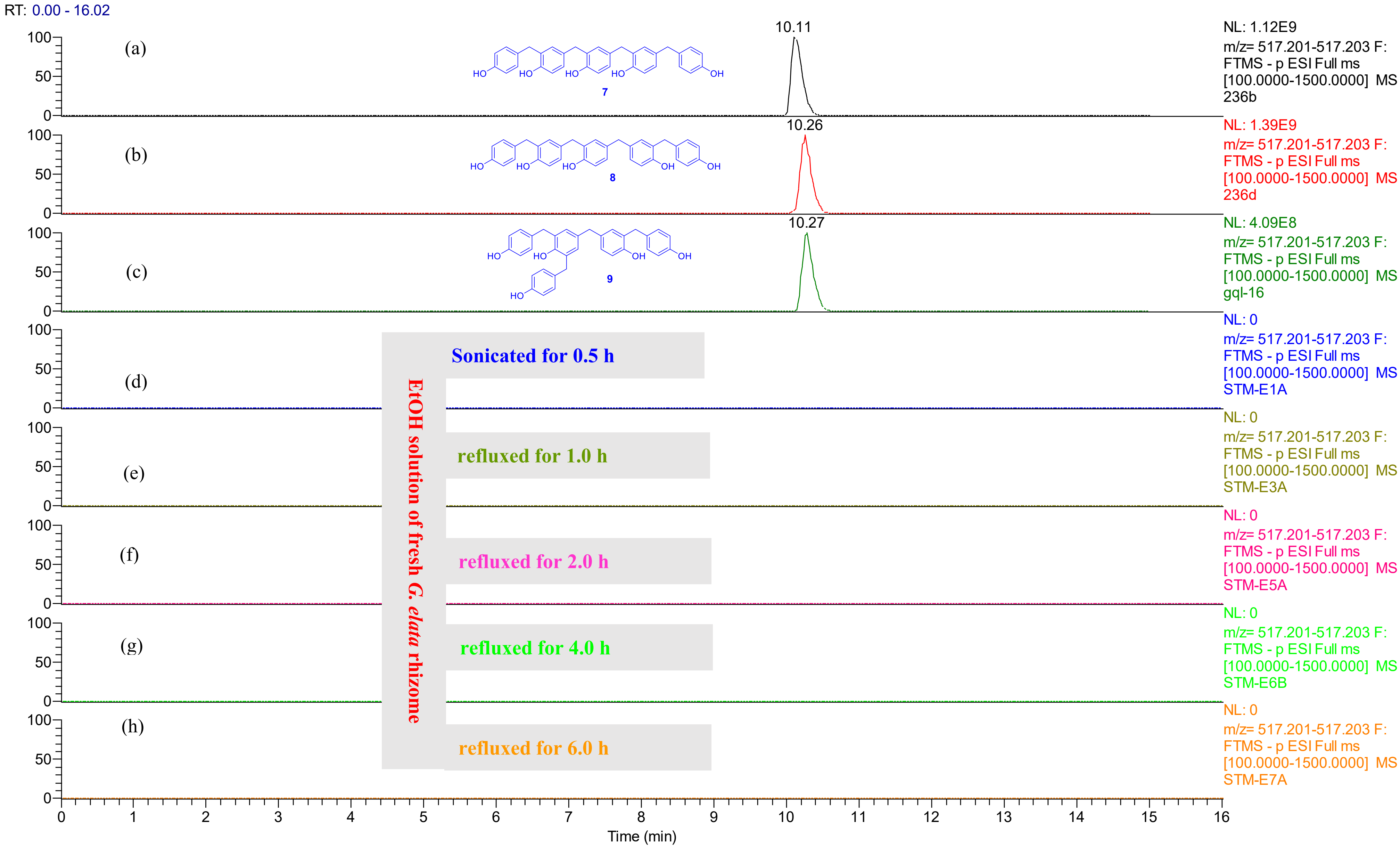

Figure S378. Overlaid chromatograms of the extracted negative ion at *m/z* 517.202 [M–H]$^-$: (a)–(c) compounds **7**–**9** in CH_3CN, respectively; (d)–(h) EtOH solution of fresh *G. elata* rhizome was sonicated for 0.5 h then refluxed for 1.0 h, 2.0 h, 4.0 h, and 6.0 h, respectively.

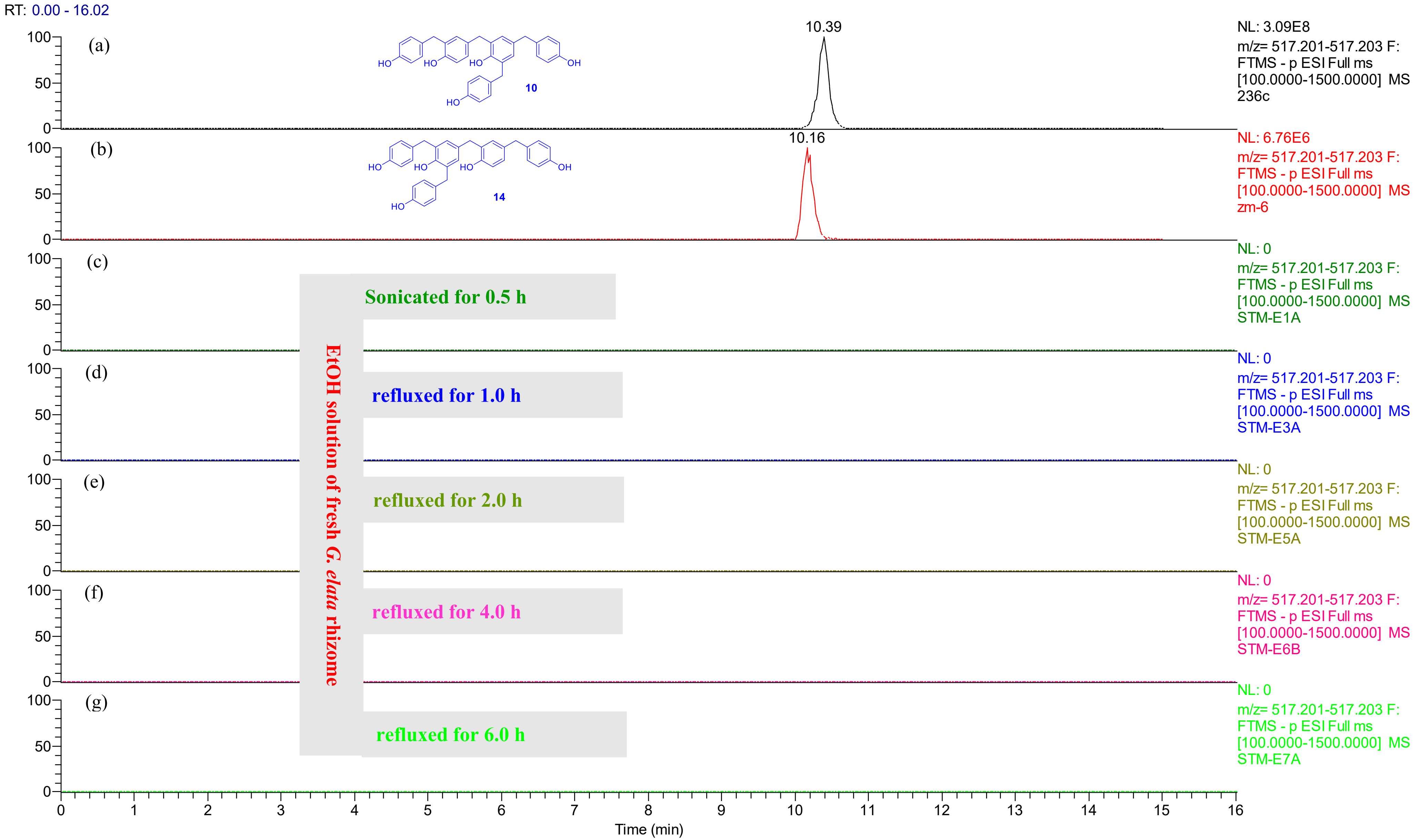

Figure S379. Overlaid chromatograms of the extracted negative ion at *m/z* 517.202 [M–H]⁻: (a) and (b) compounds **10** and **14** in CH_3CN, respectively; (c)–(g) EtOH solution of fresh *G. elata* rhizome was sonicated for 0.5 h then refluxed for 1.0 h, 2.0 h, 4.0 h, and 6.0 h, respectively.

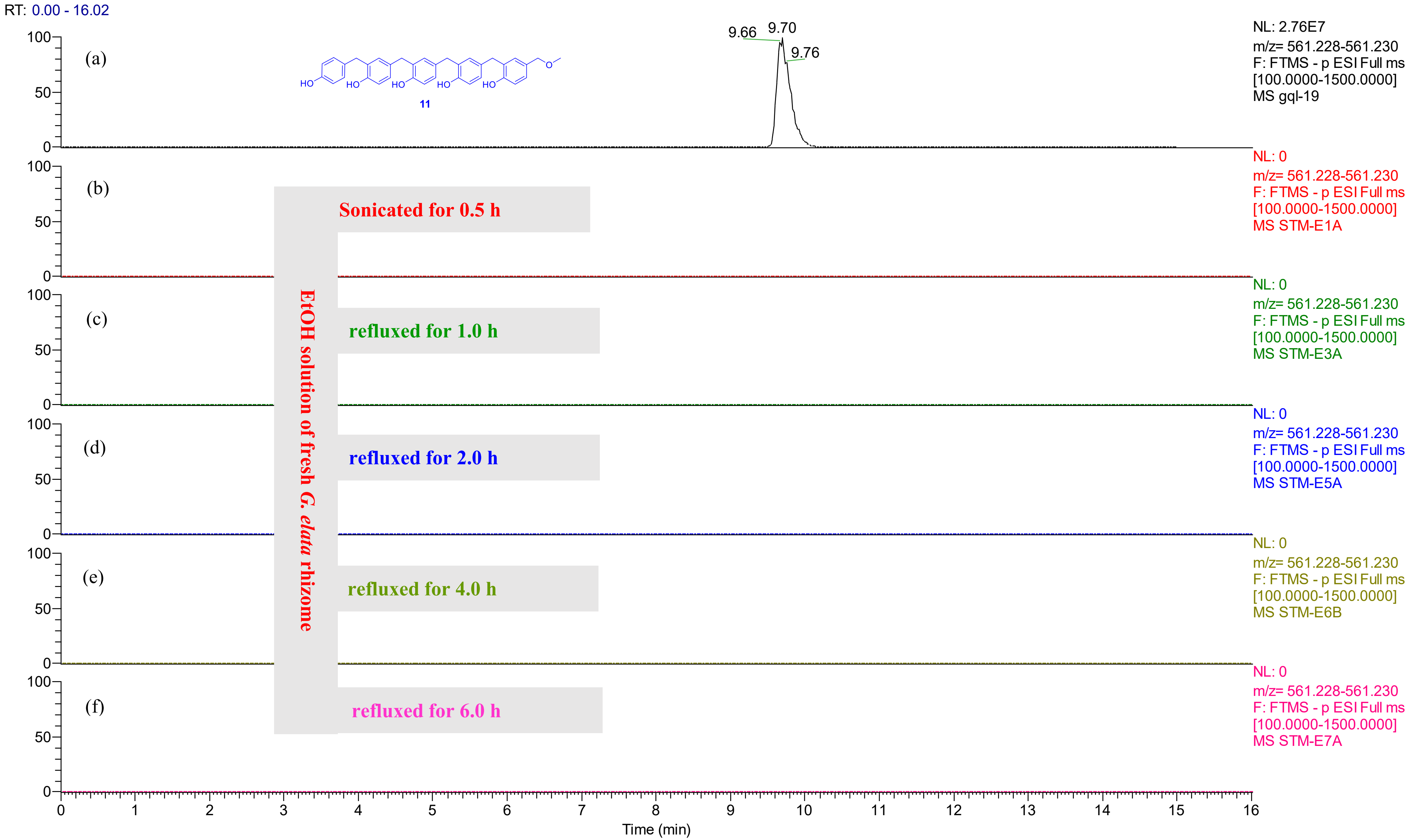

Figure S380. Overlaid chromatograms of the extracted negative ion at *m/z* 561.229 [M–H]$^-$: (a) compound **11** in CH_3CN; (b)–(f) EtOH solution of fresh *G. elata* rhizome was sonicated for 0.5 h then refluxed for 1.0 h, 2.0 h, 4.0 h, and 6.0 h, respectively.

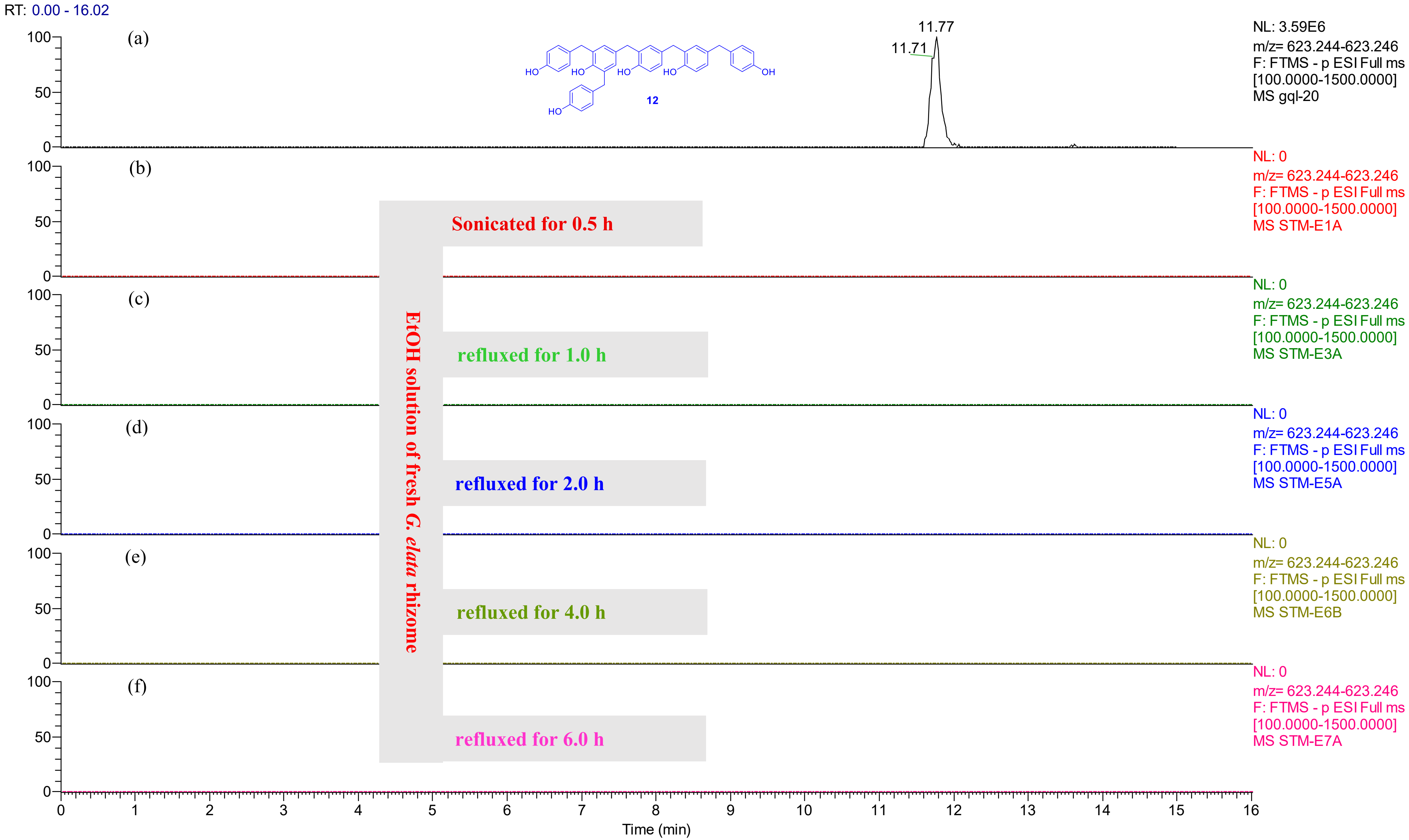

Figure S381. Overlaid chromatograms of the extracted negative ion at *m/z* 623.245 $[M–H]^-$: (a) compound **12** in CH_3CN; (b)–(f) EtOH solution of fresh *G. elata* rhizome was sonicated for 0.5 h then refluxed for 1.0 h, 2.0 h, 4.0 h, and 6.0 h, respectively.
